# Genome-wide DNA methylation profiles of low- and high-grade adenoma reveals potential biomarkers for early detection of colorectal carcinoma

**DOI:** 10.1186/s13148-020-00851-3

**Published:** 2020-04-21

**Authors:** Jian Fan, Jun Li, Shicheng Guo, Chengcheng Tao, Haikun Zhang, Wenjing Wang, Ying Zhang, Dake Zhang, Shigang Ding, Changqing Zeng

**Affiliations:** 1 Key Laboratory of Genomic and Precision Medicine, Beijing Institute of Genomics, Chinese Academy of Sciences, Beijing, 100101 China; 2 Department of Gastroenterology, Peking University Third Hospital, Beijing, 100191 China; 3 Department of Medical Genetics, School of Medicine and Public Health, University of Wisconsin-Madison, Madison, WI 53726 USA; 4 Center for Precision Medicine Research, Marshfield Clinic Research Institute, Marshfield, WI 54449 USA; 5 Beijing Advanced Innovation Center for Biomedical Engineering, School of Biological Science and Medical Engineering, Beihang University, Beijing, 100191 China; 6 University of Chinese Academy of Sciences, Beijing, 100049 China

**Keywords:** DNA methylation, Low-grade adenoma, High-grade adenoma, Colorectal cancer, Biomarker

## Abstract

**Background:**

Abnormal DNA methylation is a hallmark of human cancers and may be a promising biomarker for early diagnosis of human cancers. However, the majority of DNA methylation biomarkers that have been identified are based on the hypothesis that early differential methylation regions (DMRs) are maintained throughout carcinogenesis and could be detected at all stages of cancer.

**Methods:**

In this study, we identified potential early biomarkers of colorectal cancer (CRC) development by genome-wide DNA methylation assay (Illumina infinium450, 450 K) of normal (*N* = 20) and pre-colorectal cancer samples including 18 low-grade adenoma (LGA) and 22 high-grade adenoma (HGA), integrated with GEO and ArrayExpress datasets (*N* = 833).

**Results:**

We identified 209 and 8692 CpG sites that were significantly hyper-methylated in LGA and HGA, respectively. Pathway analysis identified nervous system-related methylation changes that are significantly associated with early adenoma development. Integration analysis revealed that DNA methylation in the promoter region of *ADHFE1* has the most potential for being an early diagnostic biomarker for colorectal adenoma and cancer (sensitivity = 0.96, specificity = 0.95, area under the curve = 0.97).

**Conclusions:**

Overall, we demonstrated that DNA methylation have been shown significant changes in the stage of LGA and HGA in the development of colon cancer. Genome-wide DNA methylation to LGA and HGA provided an important proxy to identify promising early diagnosis biomarkers for colorectal cancer.

## Background

Colorectal cancer (CRC) is the third leading cause of cancer-related deaths worldwide [1, 2]. Current evidence indicates that genetic mutations and epigenetic alterations progressively accumulate in the tumor genome during carcinogenesis, and these alterations may serve as primary biomarkers for early detection and treatment of cancer [3]. Abnormal alterations in methylation status specifically hyper-methylation or hypo-methylation have also been associated with abnormal tissue differentiation. Altered methylation has been observed in the promoter regions of tumor suppressor genes and miRNA have been observed in almost all cancer types [4, 5]. Over the past decades, detection of altered DNA methylation has been widely studied to develop cancer biomarkers [6] and the majority that have been identified are based on the hypothesis that early differential methylation regions (DMRs) are maintained throughout carcinogenesis and could be detected at all stages of cancer. For example, altered methylation patterns have been detected with hepatic disease progression in the context of hepatitis, cirrhosis, and hepatocellular carcinoma (HCC) [7]. Moreover, recent evidence demonstrated that cell-free DNA (cfDNA) methylation can be used for early cancer diagnosis and tissue-of-origin mapping for metastatic cancer [4].

Abnormal alterations of DNA methylation have been recognized as an important event in cancer development [8]. Global hypo-methylation arises early in carcinogenesis and has been linked to chromosomal instability and loss of imprinting [9, 10]. Generally, during cancer development, hundreds of genes are silenced or activated [11–13]. Although silencing of some genes in cancers occurs by mutation, a large proportion of carcinogenic gene silencing is a result of altered DNA methylation [14]. DNA methylation-based silencing in cancer typically occurs at multiple CpG sites in the CpG islands present in the promoters of protein-coding genes [15]. Although extensive epigenetic alterations have been defined over the past years, CRC is still not well understood at the molecular level [3]. Against a background of whole-genome hypo-methylation, gene-specific promoter hyper-methylation has been found to promote CRC by downregulating the expression of key tumor suppressor genes such as *CDKN2A*, *MLH1*, and *CDH1* [16–18]. CRC is a heterogeneous disease that typically originates from a pre-cancerous lesion, often in the form of an adenoma, eventually progressing to a malignant cancer within a temporal window that may exceed 10 years [19]. Because CRC exceeds many other cancers in both incidence and mortality, capacity to detect and monitor molecular changes during the colorectal adenoma (AD) stage provides an excellent opportunity to prevent cancer progression and improve survival outcomes [20]. While a large number of studies have focused on CRC, a subset of them has focused specifically on the adenoma as an intermediate stage which required more specific molecular definition. For instance, a ten-gene methylation signature in adenoma was found to change with pathologic progress [21]. Notably, colorectal adenoma has two pathologic stages: low-grade adenoma (LGA) and high-grade adenoma (HGA) [22]. Our research compared and defined differences of genome-wide profiling of DNA methylation, especially changes across these two pre-cancerous stages that had not been reported [23]. We hypothesized that these alterations in LGA methylation represented candidates as potential early diagnostic biomarkers. We further posit that comprehensive understanding of the genome-wide DNA methylation profile for early stage pre-cancerous lesions (LGA and HGA) will provide important resources, early diagnosis, and candidate biomarkers for potential oncogenic progression.

In this study, we conducted a series of genome-wide DNA methylation array of 18 LGA and 22 HGA and compared the frequency, location, and pattern of methylation status of 20 normal tissue samples. Dynamic DNA methylation changes were identified for LGA and HGA, and we found that methylation changes that appeared in LGA were increased or maintained in HGA and cancer. Enrichment analyses to DMRs were performed to further investigate the potential influence of DNA methylation on functional difference in adenoma initiation and development. Moreover, we separated different methylation sites (DMSs) between LGA and normal into hyper-DMS and hypo-DMS and evaluated their respective performance for CA and CRC prediction. To validate our findings, we compared them to genome-wide DNA methylation profiles of 833 samples from public database. Finally, we describe the identification and analysis of one functional methylation signature at the promotor region of *ADHFE1* as a potential biomarker for early CRC development.

## Results

### Landscape of DNA methylation of pre-cancerous lesions

**Fig. 1:**
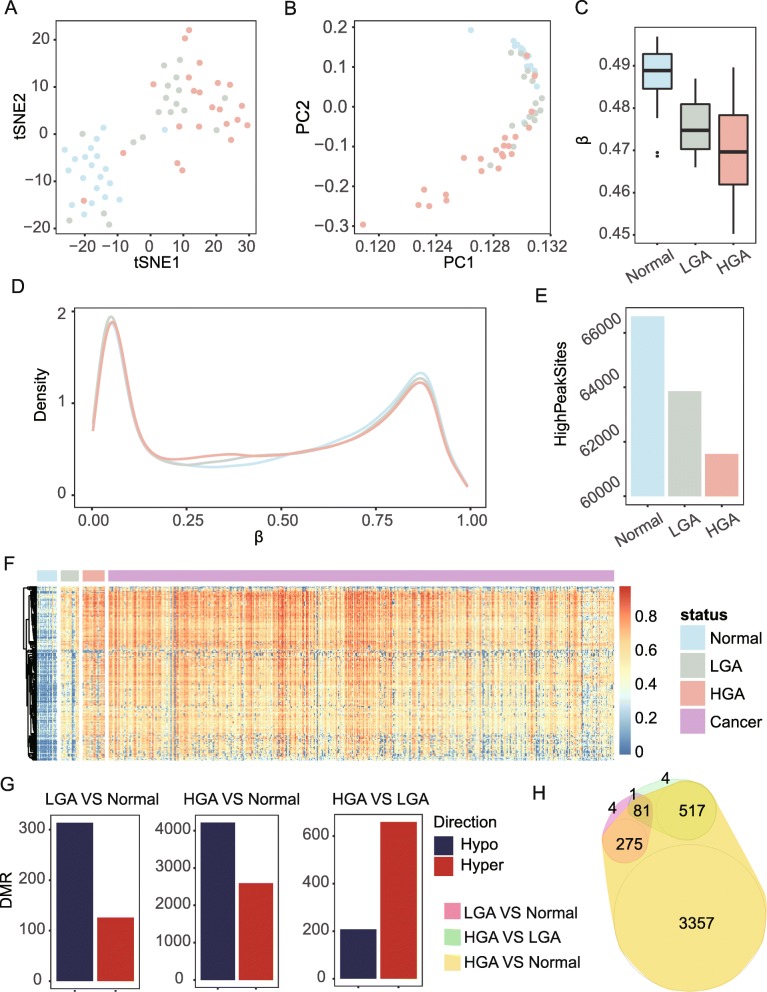
Genome-wide DNA methylation of low-grade adenoma (LGA), high-grade colorectal adenoma (HGA), and normal colorectal tissue. **a** t-SNE analysis highlights the data structure and sample relationship among the sample groups. **b** PCA analysis confirms the data structure and sample relationship of the t-SNE analysis. **c** Average methylation levels of normal (N), LGA, and HGA samples. **d** Density plot reveals the distribution of the whole array probes for N, LGA, and HGA samples. **e** Number of sites in *β* ranging from 0.7 to 0.9. **f** Heatmap of the 209 hyper-methylated DMSs of in-house datasets and samples from 504 public cancer datasets. **g** DMR between LGA and normal tissues, HGA and normal tissue, and HGA and LGA. **h** Venn graph highlights the relationships among all DMRs

We profiled DNA methylation at the single-base level for 18 LGA, 22 HGA, and 20 normal tissues. We found significant genome-wide DNA methylation differences among normal-, low-, and high-grade adenoma (Fig. 1a, b). Compared to normal tissue, LGA had genome-wide hypo-methylation (*P* = 5.2 × 10^−5^, rank sum test) which was even lower in HGA (*P* = 3.7 × 10^−6^, compared with normal, rank sum test, Fig. 1c). Methylation levels of all target sites in the array demonstrated the known bimodal distribution in normal, LGA, and HGA (Fig. 1d), and the amount of fully methylated sites of lesions decreased with increasing degree of malignancy (right peak, Fig. 1d, e). Almost all DMSs in LGA compared to normal tissues kept at least an equivalent methylation level if not higher than in HGA and cancer (Additional file 1: Fig. S1). The 209 significantly hyper-methylated sites in LGA were further hyper-methylated in 22 HGA and 504 cancer samples collected from public databases (Fig. 1f and Additional file 1: Fig. S2, Table S1), and hypo-DMSs had a diametric tendency (Additional file 1: Fig. S3) suggesting that DNA demethylation may occur very early in pre-cancerous lesions. Over 60% of DMRs that were observed in both LGA (71.4%, 314/440) and HGA (61.9%, 4,213/6,805) were hypo-methylated compared to normal tissues (Fig. 1g, Additional file 1: Table S2 and S3). However, with LGA as the reference, most DMRs observed in HGA were hyper-methylated (76.0%, 660/868) (Fig. 1g, Additional file 1: Table S4). In addition, there were limited overlaps between genes with DMRs in LGA compared to normal tissues and those compared to HGA, suggesting different epigenetic process (Fig. 1h) [24].

### Nervous system processes were associated with adenoma development

**Fig. 2:**
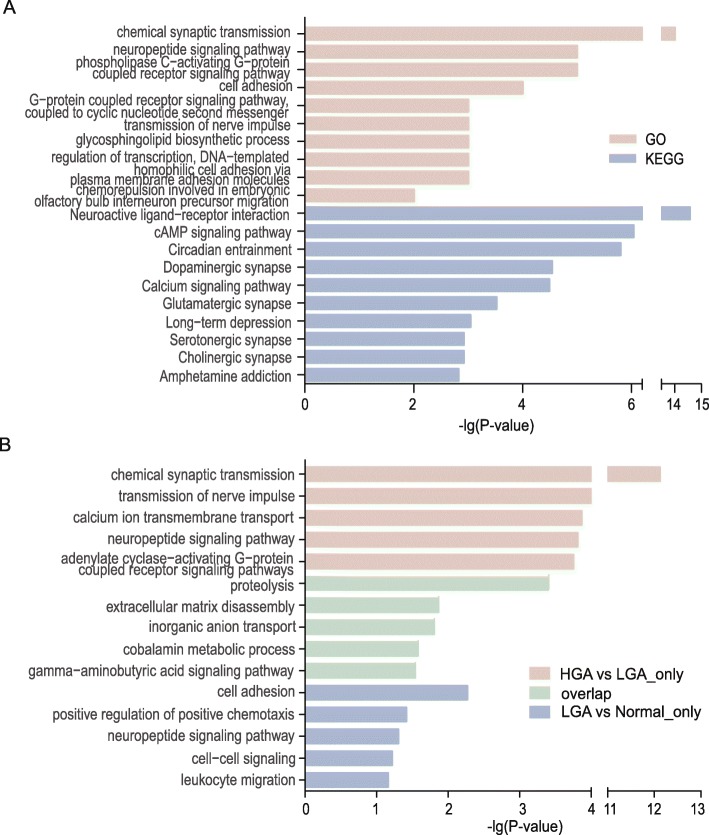
Enrichment analysis shows the top 5–10 terms associated with methylation differences between LGA and HGA. **a** GO and KEGG analysis of the genes with DMRs associated with LGA and HGA. **b** GO analysis of the genes with alterations in DMRs including differences in DMRs only in HGA vs LGA, only in LGA vs normal, and areas where HGA vs LGA and LGA vs normal overlapped

Enrichment analysis of 603 DMRs which were located between HGA and LGA, and most highly enriched functional terms, included the nervous system and those associated with signal transduction (Fig. 2a), specifically dopaminergic synapse and serotonergic synapse pathways, which play a role in the gut–brain axis model of signaling cross-talk between organ systems [25]. These results correspond to gene methylation findings in Fig. 1g where HGA vs normal includes almost all genes that are listed in LGA vs normal and HGA vs LGA DMRs. To figure out the potential function changes from LGA to HGA, Gene Ontology (GO) enrichment was performed for 275 genes that were significantly different in methylation status between LGA vs normal and HGA vs normal without considering the differences in methylation status between HGA vs LGA. Five hundred seventy-one significantly different methylated genes were highlighted in HGA vs LGA and HGA vs normal without LGA vs normal (Fig. 2b). For the 275 genes with significantly different methylation patterns in only the LGA vs normal and HGA vs normal comparisons, GO analysis selected the top enriched terms of proteolysis as well as extracellular matrix disassembly, inorganic anion transport, and cobalamin metabolic processes. Cell adhesion, positive regulation of positive chemotaxis, and neuropeptide signaling pathway were term hits on the overlapping part between LGA vs normal and HGA vs LGA. Genes that were significantly different in methylation status between LGA and HGA were enriched for chemical synaptic transmission, transmission of nerve impulse, calcium ion transmembrane transport, and similar neural processing terms. Like the DMR enrichment analysis, terms related to the nervous system were selected yet exhibited different term patterns between HGA vs LGA compared to LGA vs normal.

### Hyper-methylated CpG sites exhibited better discrimination between normal, pre-cancerous, and cancerous tissues than the hypo-methylated pattern for CRC

**Fig. 3:**
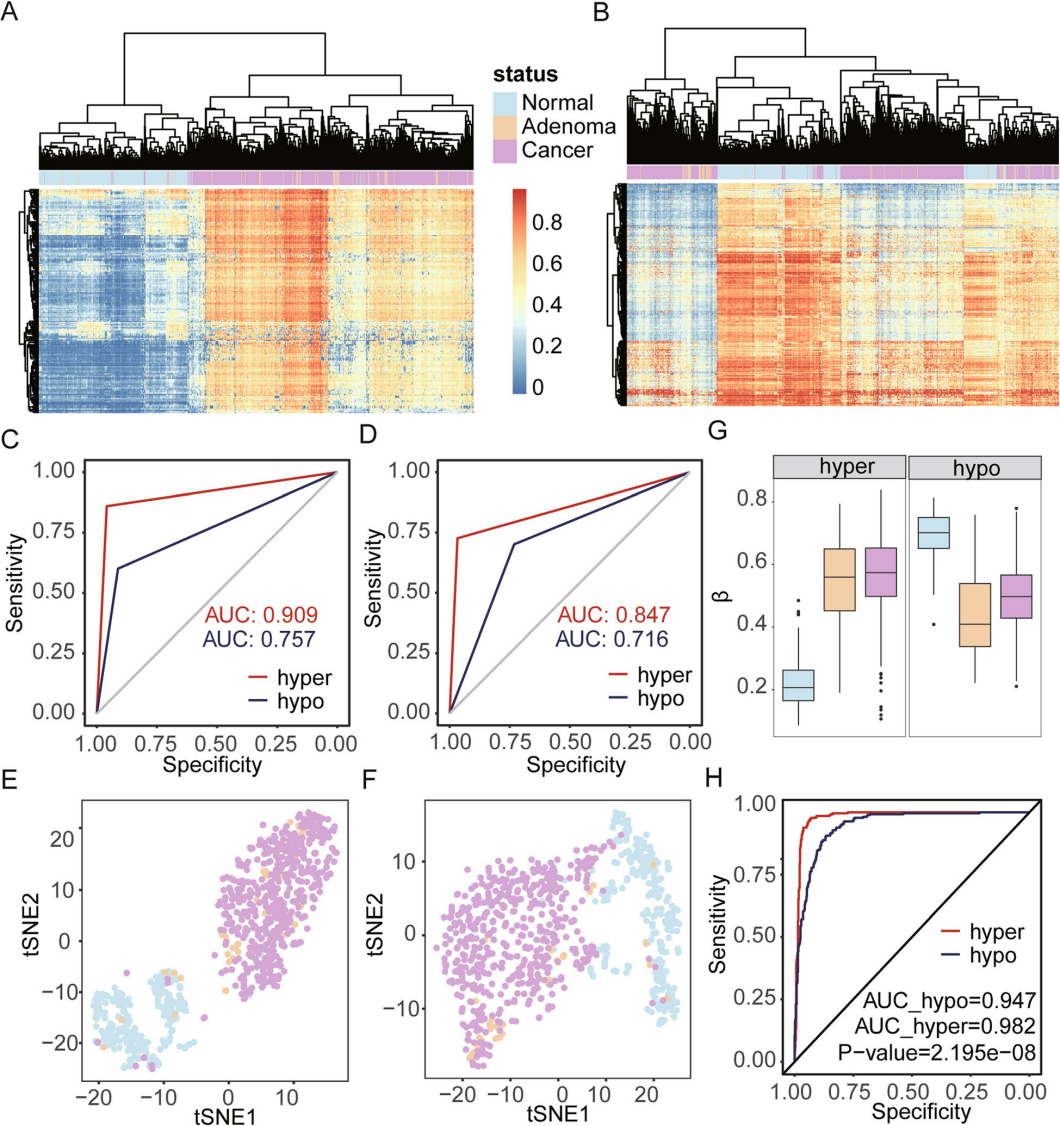
Hyper-methylated CpG sites showed better diagnostic performance than the hypo-methylated pattern. **a** Cluster analysis based on hyper-DMSs among normal, adenoma, and cancer samples. **b** Cluster analysis based on hypo-DMSs among normal, adenoma, and cancer samples. **c** Random forest prediction performance based on hyper- and hypo-DMSs. **d** Neural network prediction performance based on hyper- and hypo-DMSs. **e** t-SNE analysis highlights the data structure and sample relationship based on hyper-DMSs. **f** t-SNE analysis highlights the data structure and sample relationship based on hypo-DMSs. **g** Average methylation level of hyper- and hypo-DMSs. **h** ROC curve of hyper-mBV and hypo-mBV

To distinguish the discriminatory ability of DNA methylation patterns for normal tissue, CA, and CRC, we collected 833 genome-wide DNA methylation datasets from GEO and ArrayExpress, public datasets which included 278 normal tissue samples, 51 adenoma samples, and 504 cancer samples. We separated DMSs of LGA vs normal into two groups including hyper-DMSs (209 sites) and hypo-DMSs (441 sites). We found both hyper-DMSs and hypo-DMSs could effectively distinguish methylation pattern differences between disease (adenoma and cancer) and normal samples (Fig. 3a, b). Meanwhile, we also conducted two machine learning-based predictions with the DMSs identified in our dataset and observed that hyper-methylated sites can better distinguish between normal samples and disease samples via random forest and neural network methods (Table 1). For hyper-methylated sites, the area under the curves (AUCs) of receiver operating characteristic (ROC) curves were 0.91 and 0.85, respectively. For hypo-methylated sites, AUCs of ROC curves were lower at 0.72 and 0.76, respectively (Fig. 3c, d). Unsupervised t-SNE cluster analysis produced the same result (Fig. 3e, f). To avoid inconsistent results caused by unstable methylation based on single CpG sites, we compared the mean beta value (mBV) of these sites. We found that hyper-methylated mBVs were significantly different between normal tissue and CRC (*P* < 2.2 × 10^−16^); however, there was no significant difference between the adenoma and cancer (*P* = 0.29, Fig. 3g) in which the average mBV of the normal tissue, adenoma, and cancer are 0.22, 0.54, and 0.57, respectively. We observed similar results for hypo-methylation sites in which the average mBV of the normal tissue, adenoma, and cancer were 0.70, 0.44, and 0.50, respectively (Fig. 3g). Finally, we found the AUCs of ROC curves with hyper-mBV and hypo-mBV were 0.98 and 0.95, respectively. Permutation analysis based on a bootstrap strategy indicated that the model based on hyper-methylated sites had better discriminatory power than the model of hypo-methylated sites (*P* < 2.2 × 10^−8^, Fig. 3h).

**Table 1:** Prediction performance based on hyper-DMS and hypo-DMS to distinguish between disease and normal colorectal tissues

Model	Methylation	Observation	Prediction		Sensitivity	Specificity
			Disease	Normal		
Random forest	Hyper	Disease	532	23	0.959	0.860
		Normal	39	239		
	Hypo	Disease	507	48	0.914	0.601
		Normal	111	167		
Neural network	Hyper	Disease	537	18	0.968	0.727
		Normal	76	202		
	Hypo	Disease	406	149	0.732	0.701
		Normal	83	195		

### The promoter of ADHFE1 may be a potential biomarker for colorectal adenoma and cancer

**Fig. 4:**
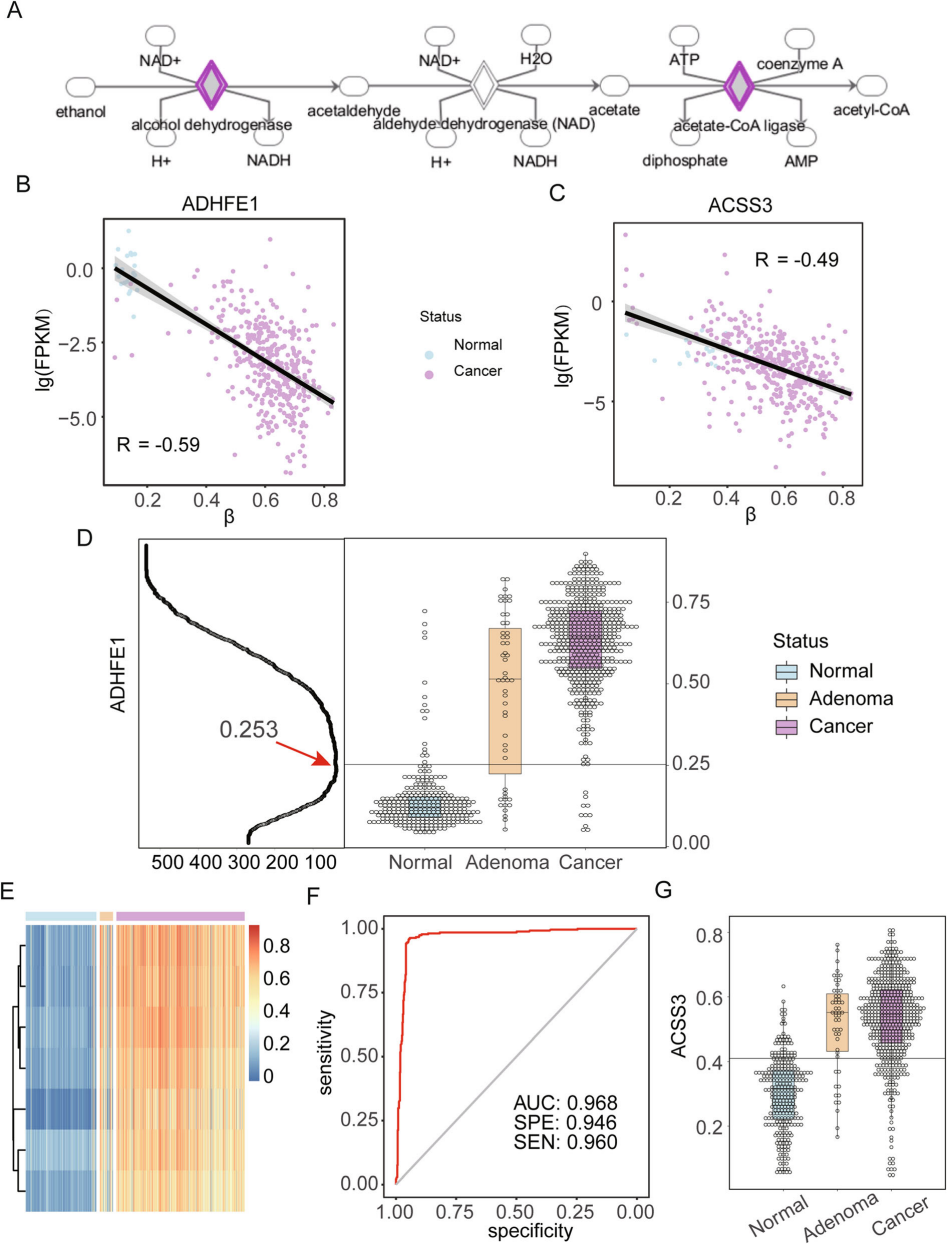
DNA methylation *ADHFE1* and *ACSS3* in normal, adenoma, and cancer. **a** Pathway of ethanol degradation II [26]. **b** Relationship between DNA methylation and gene expression of *ADHFE1*. **c** Relationship between DNA methylation and gene expression of *ACSS3*. **d** Left panel is identification of cutoff where the *X* axis is sample number of classification error; right panel is DNA methylation of *ADHFE1* in normal, adenoma, and cancer samples. **e** Heatmap of sites within *ADHFE1* promoter in normal, adenoma, and cancer samples. **f** ROC of the prediction of *ADHFE1* for colorectal adenoma and cancer. **g** DNA methylation of *ACSS3* in normal, adenoma, and cancer samples

**Fig. 5:**
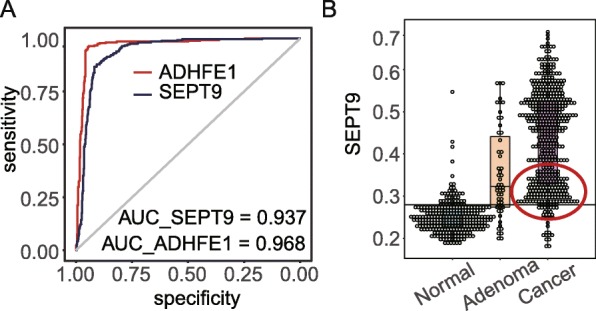
Comparison of *ADHFE1* with *SEPT9*. **a** ROC comparison of *ADHFE1* and *SEPT9*. **b** DNA methylation of *SEPT9* in normal, adenoma, and cancer samples

Next, we grouped the DMRs of normal tissue and LGA into hyper- and hypo-DMRs and performed enrichment analysis by Ingenuity Pathway Analysis (IPA). The top enriched functional term for hyper-DMRs was ethanol degradation II (*P* = 5.4 × 10^−3^) which was mostly contributed to methylation sites on two genes, *ADHFE1* and ACSS3, which can facilitate the conversion from ethanol to acetaldehyde and from acetic acid to acetyl-CoA, respectively (Fig. 4a). The expression of both genes were downregulated in colonic and rectal cancer tissue compared with normal tissue (*P* < 0.01), a result consistent with the DNA methylation changes between LGA and HGA (R2 = − 0.49 and − 0.59, Fig. 4b, c). We found that the average methylation level of CpG sites located in CpG islands within the promoter regions of *ADHFE1* and ACSS3 were significantly increased in cancer samples compared to normal samples (*∆*mBVs = 0.2 and 0.18, respectively). We further analyzed the promoter region within the CpG island of the two genes to distinguish between normal and disease tissues. When setting the cutoff at 0.25 for the *ADHFE1* promoter, the minimal error rate was only 4.68% (39/833, Fig. 4d); the heatmap of sites within the region reflected the same result (Fig. 4e). ROC curve analysis of mBV of the *ADHFE1* promoter for all 833 samples produced an AUC of 0.97 with specificity and sensitivity at 0.95 and 0.96 (Fig. 4f). For cancer samples, an AUC as high as 0.98 was determined (Additional file 1: Fig. S4). For ACSS3, the minimal error rate of its promoter was 16.68% (139/833) with a cutoff set at 0.42 (Fig. 4g) which performed inferiorly to *ADHFE1* in terms of discrimination power. Meanwhile, we also compared *ADHFE1* with *SEPT9*, an FDA-approved methylation-based biomarker for CRC screening. The correlation of the two genes was 0.77, and we determined that *ADHFE1* had a better prediction power than *SEPT9* (Fig. 5a and Additional file 1: Fig. S5) [27]. Furthermore, we observed *ADHFE1* to have a much better separation boundary compared to *SEPT9* (Fig. 5b). In view of most detected cfDNA being actually the fragments from white blood cells, we checked DNA methylation status of *ADHFE1* promoter in 656 whole blood cases from public data. As expected, all sites in the promoter were consistently at low methylation level (Additional file 1: Fig. S6).

## Discussion

Whole-genome DNA hypo-methylation and hyper-methylation analysis of the promoter regions of cancer-related genes is regarded as a common method of characterizing diverse cancers [28]. In our study, we found that whole-genome DNA hypo-methylation may start at the LGA stage and lead to further hypo-methylation at HGA and CRC (Fig. 1c). As many previous studies have reported, a bimodal distribution can characterize DNA methylation pattern, and we noted that the hyper-methylated peak can clearly reflect progressive hypo-methylation (Fig. 1d, e) [29]. We identified 440 and 6805 DMRs in low- and hyper-grade adenoma, respectively, and of these DMRs, 314 (71.4%) in LGA and 4213 (61.9%) in HGA were hypo-methylated compared to normal tissues. On the contrary, most DMR (660/868, 76.0%) differences between HGA and LGA were hyper-methylated. Aside from a little overlap between HGA genes, significantly distinct DMRs were located between LGA vs normal and HGA vs LGA which indicates that LGA vs normal and HGA vs LGA are possibly not the same process with a degree difference but two different epigenetic processes. These genome-wide demethylation patterns may indicate that though hypo-methylation dominates the carcinogenesis of CRC, hyper-methylation sites may contribute more to the distinct malignancy of these lesions.

To find functional differences between differing methylation patterns in normal, pre-cancerous, and cancerous tissues, enrichment analysis was applied to 603 genes with DMRs between HGA and LGA which determined that most enriched terms were related to nervous system and signal transduction (Fig. 2a). The term gut–brain-axis describes an integrative physiology concept that incorporates all, including afferent and efferent neural, endocrine, nutrient, and immunological signals, cross-talk between the central nervous system, and the gastrointestinal system that may be dysregulated during carcinogenesis [25]. Our Kyoto Encyclopedia of Genes and Genomes (KEGG) enrichment analysis further highlighted the significance of dopaminergic synapse and serotonergic synapse to CRC development. Serotonin (5-hydroxytryptamine; 5-HT) is popularized as a contributor to feelings of well-being and happiness though its actual biological function is complex and multifaceted with roles in modulating cognition, reward, learning, memory, and numerous physiological processes [30]. Brain 5-HT gets much more respect, and certainly more press and research, than the vastly larger store of 5-HT in the gut though both are important for physiological functions [31]. Dopamine (3,4-dihydroxyphenethylamine; DA) is an organic chemical of the catecholamine and phenethylamine families that functions as both a hormone and a neurotransmitter and plays several important roles in the brain and body [32]. In the brain, dopamine functions as a neurotransmitter to send signals to other nerve cells [32]. Outside the central nervous system, dopamine functions primarily as a local paracrine messenger to reduce gastrointestinal motility and protect the intestinal mucosa [32]. The interaction of tumor and the nervous system has also been found in gastric cancer and liver cancer [33, 34]. Our study suggests that the gut–brain axis and related molecules may be important contributors to the development and progression of CRC even at the adenoma stage.

DNA methylation has always been considered as a potential biomarker for many diseases due to its tissue specificity and stability [35]. Here, we analyzed DNA methylation patterns as a mechanism to distinguish disease samples (including adenoma and cancer) from normal samples during CRC development. We identified 209 hyper-methylated sites and 441 hypo-methylated sites between LGA and normal samples and noted that both hyper- and hypo-methylated sites could effectively distinguish between normal and CRC tissues. Further validation with random forest and neural network analyses confirmed our observations. Specifically, AUCs of ROC curves for our prediction model using hyper-methylated sites were larger than those using hypo-methylated sites, despite the observation that hypo-methylated sites were more than twice the number of hyper-methylated ones. Since tumors are known to have whole-genome hypo-methylation, we speculate that gene hyper-methylation at several key sites and/or global hypo-methylation during early CA may be the driver events for CRC. To reduce bias caused by unstable methylation on single CpG sites, we compared mBV of these sites among tissue groups. We found that hyper-methylated mBVs were significantly different between normal tissue and cancers (*P* < 2.2 × 10^−16^), while no significance was found between the adenoma and CRC (*P* = 0.288, Fig. 3g). Permutation analysis based on bootstrap strategy suggest that the model based on hyper-methylated sites has better discrimination power than the model of hypo-methylated sites (*P* < 2.2 × 10^−8^, Fig. 3h) which may lend support to the theory that hyper-methylation at several key sites may trigger widespread hypo-methylation throughout the genome during cancer development.

Colorectal adenoma is considered the middle stage between normal status and cancer; therefore, our study focused on identifying and comparing the differences in DNA methylation patterns among normal, pre-cancerous, and cancerous colorectal tissues. IPA enrichment analysis of hyper-DMRs identified in very early stage cancers selected Ethanol degradation II as the top term for functional impact, in which *ADHFE1* and *ACSS3* were hit. Intense early changes in DNA methylation patterns at the promotor region of these genes support their potential use as adenoma biomarker. It is known that *ADHFE1* encodes for hydroxyacid-oxoacid transhydrogenase which is responsible for the oxidation of 4-hydroxybutyrate in mammalian tissues [36]. Some studies have also reported that the gene is associated with cell proliferation and differentiation [36–38]. In CRC tissue, *ADHFE1* is hyper-methylated in the promoter region corresponding to downregulation of expression that may facilitate tumor growth [38]. Our results suggest that the DNA methylation of the *ADHFE1* promoter is a potential biomarker for distinguishing colorectal adenoma and cancer from normal tissue.

As the only FDA-approved liquid biopsy marker for DNA methylation, *SEPT9* has been applied for colon cancers screening [39]. Actually the detection signal of *SEPT9* has been shown to be more distinguishable in tissues than at cfDNA samples [40]. The better performance of *ADHFE1* than *SEPT9* at tissue level made it a promising liquid biopsy biomarker for CRC. Further efforts with a larger, more diverse sample population are needed to validate the predictive efficacy of this biomarker at cfDNA.

In addition, a recent study found a promising biomarker cg10673833 which distinguished tumor patients from healthy people by cfDNA [41]. However, the methylation level of this marker showed only a slight upward trend from normal tissues to adenoma and cancer, in our samples as well as in public data. In view of the very low methylation of cg10673833 in the blood, most likely its detection of cancer was mainly due to largely increased metabolism of the tumor tissue that caused increased shedding of ctDNA. Comparing with cg10673833, the better discrimination of normal to adenoma and cancer by *ADHFE1* raises a great potential for this candidate as a methylation marker to indicate pathological changes.

Besides *ADHFE1*, we obtained a group of 209 hyper-methylated DMSs in our LGA samples. For their potential being candidates of methylation markers, we examined these sites in 656 cases of whole blood from GEO. As shown in the heatmap of Additional file 1: Fig. S7, 207 out of 209 sites showed their low methylation level as < 0.3 in average, implying the potential of these sites deserving further investigation for early diagnosis.

## Conclusions

Adenoma samples are perfect proxy for colorectal carcinoma early biomarker identification. Our study focused on adenoma, in order to get the earliest clue to detect colorectal disease. DNA methylation is a promising biomarker for cancer diagnosis and surveillance for its tissue specificity and robustness. We established the DNA methylation landscape of LGA and HGA and noted the hyper-methylated peak has a regular decrease companied with disease procession. Furthermore, we found the development of adenoma is associated with functions of nervous system, while the initiation of the adenoma is more associated with cell biological functions. Another relatively independent work was based on the precious finding in LGA, in which we found *ADHFE1* is a potential early diagnosis biomarker of colorectal carcinoma and adenoma. Eight hundred thirty-three samples from the public database strongly support the gene is a promising biomarker.

## Methods

### Sample collection and pathological confirmation

In the Department of Gastroenterology of Peking University Third hospital from March 2015 to June 2016, we collected 18 LGA and 22 HGA specimens from patients who underwent endoscopic treatment for CA removal and obtained adjacent normal tissue specimens from 20 patients with adenoma during the treatment. Tissue specimens were embedded in paraffin, sectioned and stained with hematoxylin and eosin, and confirmed by pathologist by light microscopy. All the patients were treatment naive before their surgeries. Clinical information of patients, and sample position in corresponding microarray are provided in Additional file 1: Table S5 and S6.

### DNA isolation and bisulfite conversion

DNA was isolated using QIAmp DNA Mini Kit (Qiagen, Hilden, Germany) according to the manufacturer’s protocol. Bisulfite conversion was performed using the EZ DNA Methylation-Gold Kit according to the instruction manual (Zymo Research, Irvine, CA, USA).

### Methylation data processing

Epigenome-wide DNA methylation assessment for this study was performed using the Illumina Infinium Human Methylation 450 BeadChip (Illumina, San Diego, CA, USA), which simultaneously profiles the methylation status for > 485,000 CpG sites at single-nucleotide resolution and covers 96% of CpG islands with additional coverage of island shores (< 2 Kb from CpG Islands), island shelves (2–4 Kb from CpG islands), and regions flanking them. The raw data from the array was processed using the GenomeStudio Methylation (version 1.8, Illumina) module which calculated methylation levels. The GenomeStudio is the software for array data processing of Illumina, which integrates data normalization, background adjustment, and methylation calculation. Normalization was performed by comparing control probes when the option was set as controls, and background adjustment was performed automatically by the software selecting Subtract Background. The distribution of beta values before and after normalization across all was analyzed (Additional file 1: Fig. S8), and multi-dimensional scaling (MDS) according to 10,000 most variable positions showed the homogeneity of samples and their clustering according to pathological groups. Beta MDS were also analyzed according to 1000 and 20,000 most variable positions for all samples before and after normalization (Additional file 1: Fig. S9). The methylation status for each CpG site was calculated as the ratio of fluorescent signals (β = Max(M,0)/[Max(M,0) + Max(U,0) + 100]), ranging from 0 to 1 using the average probe intensity for the methylated (M) and unmethylated (U) alleles. *β* = 1 indicates complete methylation; *β* = 0 represents no methylation. Probes located on sex chromosomes or failed detection *P* value testing of at least one sample or SNP (single-nucleotide polymorphism) were removed from analysis using R package IMA (vision 3.1.2) [42]. DMRs were defined as rank sum test following false discovery rate (FDR) adjusted *P* value < 0.05 and |*∆*β| > 0.15, and DMSs were defined as rank sum test following FDR adjusted *P* value < 0.05 and |*∆*β| > 0.20. Promoter regions were defined as 5′UTR, TSS200, TSS1500, and first exons.

### Public datasets and processing

To ensure consistency of data processing, we only compared our samples with publically accessible samples with raw *idat* files. GSE68060, GSE68838, GSE77954, GSE77965, GSE81211, GSE101764, GSE107352, and GSE75546 were collected from GEO while E-MTAB-6450 was collected from ArrayExpress [43–48] (Additional file 1: Table S6). Some cell line samples and metastatic cancer samples were removed upon further study. In total, we collected 278 normal samples, 51 adenoma samples, and 504 cancer samples. All datasets using raw *idat* files were preprocessed using R package minfi (vision 1.28.4) [49]. The sites which failed detection at *P* = 0.01 were rewritten to the nearest neighbor average to ensure an adequate number of sites for analysis. Six hundred fifty-six cases of whole blood data were collected from GEO (accession number GSE40279).

### Comparison of the ability of discrimination between normal, LGA, HGA, and CRC tissue

For random forest prediction, we used R package randomForest (vision 4.6.14) with the number of trees set at 5000 [50]. For neural network prediction, we used R package nnet (vision 7.3.12) with number of units in the hidden layer as 2, weight decay as 10^−4^, and with a maximum number of iterations at 400 [51]. The R package pROC (vision 1.14.0) was used for ROC analysis to compare the abilities of various models to distinguish between hyper- and hypo-methylated sites by the area under the curve (AUC) analysis [52].

### t-SNE analysis, PCA analysis, and gene enrichment analysis

t-Distributed stochastic neighbor embedding (t-SNE) analysis was performed by R package t-sne (vision 0.1-3) [53]. PCA was performed by R function princomp and visualized by first two principal components. KEGG and GO enrichment were analyzed online by DAVID 6.8 (https://david.ncifcrf.gov) [54, 55]. Ingenuity Pathway Analysis (IPA) was also used for enrichment analysis for more elaborate results with the *P* value cutoff set at 0.05 [26].

## Supplementary information

**Additional file 1.** Supplementary figures and tables

## Data Availability

All methylation array data are available at GEO under accession number GSE139404. Other public data involved in this study included GSE68060, GSE68838, GSE77954, GSE77965, GSE81211, GSE101764, GSE107352, GSE75546, GSE40279, and E-MTAB-6450.

## Abbreviations

LGA: Low-grade adenoma HGA High-grade adenoma LGA vs Normal Comparison of low-grade adenoma with normal tissue HGA vs Normal Comparison of high-grade adenoma with normal tissue HGA vs LGA Comparison of high-grade adenoma with low-grade adenoma DMR Different methylation region DMS Different methylation site ROC Receiver operating characteristic AUC Area under the curve IPA Ingenuity Pathway Analysis KEGG Kyoto Encyclopedia of Genes and Genomes GO Gene Ontology t-SNE t-distributed stochastic neighbor embedding PCA Principal components analysis mBV Mean beta values FDR False discovery rate SNP Single-nucleotide polymorphism 5′UTR5 5′ Untranslated region CHR Chromosome

## References

[1] Siegel RL, Miller KD, Jemal A (2018). Cancer statistics, 2018. CA Cancer J Clin.

[2] Chen W, Zheng R, Baade PD, Zhang S, Zeng H, Bray F, Jemal A, Yu XQ, He J (2016). Cancer statistics in China, 2015. CA Cancer J Clin.

[3] Kuipers EJ, Grady WM, Lieberman D, Seufferlein T, Sung JJ, Boelens PG, van de Velde CJ, Watanabe T (2015). Colorectal cancer. Nat Rev Dis Primers.

[4] Guo S, Diep D, Plongthongkum N, Fung HL, Zhang K, Zhang K (2017). Identification of methylation haplotype blocks aids in deconvolution of heterogeneous tissue samples and tumor tissue-of-origin mapping from plasma DNA. Nat Genet.

[5] Wang X, Wang L, Guo S, Bao Y, Ma Y, Yan F, Xu K, Xu Z, Jin L, Lu D (2013). Hypermethylation reduces expression of tumor-suppressor PLZF and regulates proliferation and apoptosis in non-small-cell lung cancers. FASEB journal : official publication of the Federation of American Societies for Experimental Biology.

[6] Guo S, Yan F, Xu J, Bao Y, Zhu J, Wang X, Wu J, Li Y, Pu W, Liu Y (2015). Identification and validation of the methylation biomarkers of non-small cell lung cancer (NSCLC). Clin Epigenetics.

[7] Zhao Y, Xue F, Sun J, Guo S, Zhang H, Qiu B, Geng J, Gu J, Zhou X, Wang W (2014). Genome-wide methylation profiling of the different stages of hepatitis B virus-related hepatocellular carcinoma development in plasma cell-free DNA reveals potential biomarkers for early detection and high-risk monitoring of hepatocellular carcinoma. Clin Epigenetics.

[8] Patai AV, Molnár B, Kalmár A, Schöller A, Tóth K, Tulassay Z (2012). Role of DNA methylation in colorectal carcinogenesis. Dig Dis.

[9] Grady WM, Carethers JM (2008). Genomic and epigenetic instability in colorectal cancer pathogenesis. Gastroenterology.

[10] Hidaka H, Higashimoto K, Aoki S, Mishima H, Hayashida C, Maeda T, Koga Y, Yatsuki H, Joh K, Noshiro H (2018). Comprehensive methylation analysis of imprinting-associated differentially methylated regions in colorectal cancer. Clin Epigenetics.

[11] Shi YX, Wang Y, Li X, Zhang W, Zhou HH, Yin JY, Liu ZQ (2017). Genome-wide DNA methylation profiling reveals novel epigenetic signatures in squamous cell lung cancer. BMC Genomics.

[12] Lindqvist BM, Wingren S, Motlagh PB, Nilsson TK (2014). Whole genome DNA methylation signature of HER2-positive breast cancer. Epigenetics.

[13] Raggi C, Invernizzi P (2013). Methylation and liver cancer. Clin Res Hepatol Gastroenterol.

[14] Jones PA (2012). Functions of DNA methylation: islands, start sites, gene bodies and beyond. Nat Rev Genet.

[15] Morris MR, Latif F (2017). The epigenetic landscape of renal cancer. Nat Rev Nephrol.

[16] Herman JG, Merlo A, Mao L, Lapidus RG, Issa J-PJ, Davidson NE, Sidransky D, Baylin SB (1995). Inactivation of the CDKN2/p16/MTS1 gene is frequently associated with aberrant DNA methylation in all common human cancers. Cancer Res.

[17] Kane MF, Loda M, Gaida GM, Lipman J, Mishra R, Goldman H, Jessup JM, Kolodner R (1997). Methylation of the hMLH1 promoter correlates with lack of expression of hMLH1 in sporadic colon tumors and mismatch repair-defective human tumor cell lines. Cancer Res.

[18] Yoshiura K, Kanai Y, Ochiai A, Shimoyama Y, Sugimura T, Hirohashi S (1995). Silencing of the E-cadherin invasion-suppressor gene by CpG methylation in human carcinomas. Proc Natl Acad Sci.

[19] Witold K, Anna K, Maciej T, Jakub J (2018). Adenomas - genetic factors in colorectal cancer prevention. Rep Pract Oncol Radiother.

[20] Zauber AG, Winawer SJ, O'Brien MJ, Lansdorp-Vogelaar I, van Ballegooijen M, Hankey BF, Shi W, Bond JH, Schapiro M, Panish JF (2012). Colonoscopic polypectomy and long-term prevention of colorectal-cancer deaths. N Engl J Med.

[21] Patai Á, Valcz G, Hollósi P, Kalmár A, Péterfia B, Patai Á, Wichmann B, Spisák S, Barták BK, Leiszter K (2015). Comprehensive DNA methylation analysis reveals a common ten-gene methylation signature in colorectal adenomas and carcinomas. PLoS One.

[22] Schlemper RJ, Riddell RH, Kato Y, Borchard F, Cooper HS, Dawsey SM, Dixon MF, Fenoglio-Preiser CM, Flejou JF, Geboes K (2000). The Vienna classification of gastrointestinal epithelial neoplasia. Gut.

[23] Rex DK, Johnson DA, Anderson JC, Schoenfeld PS, Burke CA, Inadomi JM (2009). American College of G: American College of Gastroenterology guidelines for colorectal cancer screening 2009 [corrected]. Am J Gastroenterol.

[24] Perez-Silva JG, Araujo-Voces M, Quesada V (2018). nVenn: generalized, quasi-proportional Venn and Euler diagrams. Bioinformatics.

[25] Clemmensen C, Muller TD, Woods SC, Berthoud HR, Seeley RJ (2017). Tschop MH: gut-brain cross-talk in metabolic control. Cell.

[26] Kramer A, Green J, Pollard J, Tugendreich S (2014). Causal analysis approaches in Ingenuity Pathway Analysis. Bioinformatics.

[27] Church TR, Wandell M, Lofton-Day C, Mongin SJ, Burger M, Payne SR, Castanos-Velez E, Blumenstein BA, Rosch T, Osborn N (2014). Prospective evaluation of methylated SEPT9 in plasma for detection of asymptomatic colorectal cancer. Gut.

[28] Kulis M, Esteller M (2010). DNA methylation and cancer. Adv Genet.

[29] Straussman R, Nejman D, Roberts D, Steinfeld I, Blum B, Benvenisty N, Simon I, Yakhini Z, Cedar H (2009). Developmental programming of CpG island methylation profiles in the human genome. Nat Struct Mol Biol.

[30] Swami T, Weber HC (2018). Updates on the biology of serotonin and tryptophan hydroxylase. Curr Opin Endocrinol Diabetes Obes.

[31] Xiaolong G, Junhai P, Yichang L, Hongkan W, Wei Z, Xianfa W (2018). Intestinal crosstalk between microbiota and serotonin and its impact on gut motility. Curr Pharm Biotechnol.

[32] Berke JD (2018). What does dopamine mean?. Nat Neurosci.

[33] Jeong S, Zheng B, Wang H, Xia Q, Chen L (2018). Nervous system and primary liver cancer. Biochim Biophys Acta Rev Cancer.

[34] Wang K, Zhao XH, Liu J, Zhang R, Li JP (1873). Nervous system and gastric cancer. Biochim Biophys Acta Rev Cancer.

[35] Pan Y, Liu G, Zhou F, Su B, Li Y (2018). DNA methylation profiles in cancer diagnosis and therapeutics. Clin Exp Med.

[36] Deng Y, Wang Z, Gu S, Ji C, Ying K, Xie Y, Mao Y (2002). Cloning and characterization of a novel human alcohol dehydrogenase gene (ADHFe1). DNA Seq.

[37] Moon JW, Lee SK, Lee YW, Lee JO, Kim N, Lee HJ, Seo JS, Kim J, Kim HS, Park SH (2014). Alcohol induces cell proliferation via hypermethylation of ADHFE1 in colorectal cancer cells. BMC Cancer.

[38] Tae CH, Ryu KJ, Kim SH, Kim HC, Chun HK, Min BH, Chang DK, Rhee PL, Kim JJ, Rhee JC (2013). Alcohol dehydrogenase, iron containing, 1 promoter hypermethylation associated with colorectal cancer differentiation. BMC Cancer.

[39] Tóth K, Sipos F, Kalmár A, Patai AV, Wichmann B, Stoehr R, Golcher H, Schellerer V, Tulassay Z, Molnár B (2012). Detection of methylated SEPT9 in plasma is a reliable screening method for both left- and right-sided colon cancers. PLoS One.

[40] Tóth K, Wasserkort R, Sipos F, Kalmár A, Wichmann B, Leiszter K, Valcz G, Juhász M, Miheller P, Patai Á (2014). Detection of methylated septin 9 in tissue and plasma of colorectal patients with neoplasia and the relationship to the amount of circulating cell-free DNA. PLoS One.

[41] Luo H, Zhao Q, Wei W, Zheng L, Yi S, Li G, Wang W, Sheng H, Pu H, Mo H (2020). Circulating tumor DNA methylation profiles enable early diagnosis, prognosis prediction, and screening for colorectal cancer. Sci Transl Med.

[42] Wang D, Yan L, Hu Q, Sucheston LE, Higgins MJ, Ambrosone CB, Johnson CS, Smiraglia DJ, Liu S (2012). IMA: an R package for high-throughput analysis of Illumina's 450 K Infinium methylation data. Bioinformatics.

[43] Qu X, Sandmann T, Frierson H, Fu L, Fuentes E, Walter K, Okrah K, Rumpel C, Moskaluk C, Lu S (2016). Integrated genomic analysis of colorectal cancer progression reveals activation of EGFR through demethylation of the EREG promoter. Oncogene.

[44] consortium B (2016). Quantitative comparison of DNA methylation assays for biomarker development and clinical applications. Nat Biotechnol.

[45] Kang K, Bae JH, Han K, Kim ES, Kim TO, Yi JM (2016). A genome-wide methylation approach identifies a new hypermethylated gene panel in ulcerative colitis. Int J Mol Sci.

[46] Barrow TM, Klett H, Toth R, Bohm J, Gigic B, Habermann N, Scherer D, Schrotz-King P, Skender S, Abbenhardt-Martin C (2017). Smoking is associated with hypermethylation of the APC 1A promoter in colorectal cancer: the ColoCare Study. J Pathol.

[47] Damaso E, Castillejo A, Arias MDM, Canet-Hermida J, Navarro M, Del Valle J, Campos O, Fernandez A, Marin F, Turchetti D (2018). Primary constitutional MLH1 epimutations: a focal epigenetic event. Br J Cancer.

[48] Bormann F, Rodriguez-Paredes M, Lasitschka F, Edelmann D, Musch T, Benner A, Bergman Y, Dieter SM, Ball CR, Glimm H (2018). Cell-of-origin DNA methylation signatures are maintained during colorectal carcinogenesis. Cell Rep.

[49] Aryee MJ, Jaffe AE, Corrada-Bravo H, Ladd-Acosta C, Feinberg AP, Hansen KD, Irizarry RA (2014). Minfi: a flexible and comprehensive Bioconductor package for the analysis of Infinium DNA methylation microarrays. Bioinformatics.

[50] Wiener ALaM (2002). Classification and regression by randomForest. R News.

[51] Ripley WNVaBD (2002). Modern applied statistics with S, Fourth edn.

[52] Robin X, Turck N, Hainard A, Tiberti N, Lisacek F, Sanchez JC, Muller M (2011). pROC: an open-source package for R and S+ to analyze and compare ROC curves. BMC Bioinformatics.

[53] Hinton GE (2008). Visualizing high-dimensional data using t-SNE. J Mach Learn Res.

[54] da Huang W, Sherman BT, Lempicki RA (2009). Systematic and integrative analysis of large gene lists using DAVID bioinformatics resources. Nat Protoc.

[55] Huang DW, Sherman BT, Lempicki RA (2009). Bioinformatics enrichment tools: paths toward the comprehensive functional analysis of large gene lists. Nucleic Acids Res.

