# Supplementary material for: Genome-wide DNA methylation profiles of low- and high-grade adenoma reveals potential biomarkers for early detection of colorectal carcinoma
Source: Clin Epigenetics. 2020 Apr 21;12:56. doi: 10.1186/s13148-020-00851-3 (PMC7175491; doi:10.1186/s13148-020-00851-3)
Supplement: Supplementary file 1 — Additional file 1. Supplementary figures and tables [file 13148_2020_851_MOESM1_ESM.pdf]

Supplementary

Figure S1-S and Table S1-S7

Figure S1. Hyper DMSs (Left) and hypo DMSs (Right) shared between HGA (High grade adenoma), LGA (Low grade adenoma) and Normal groups or specific to each group.

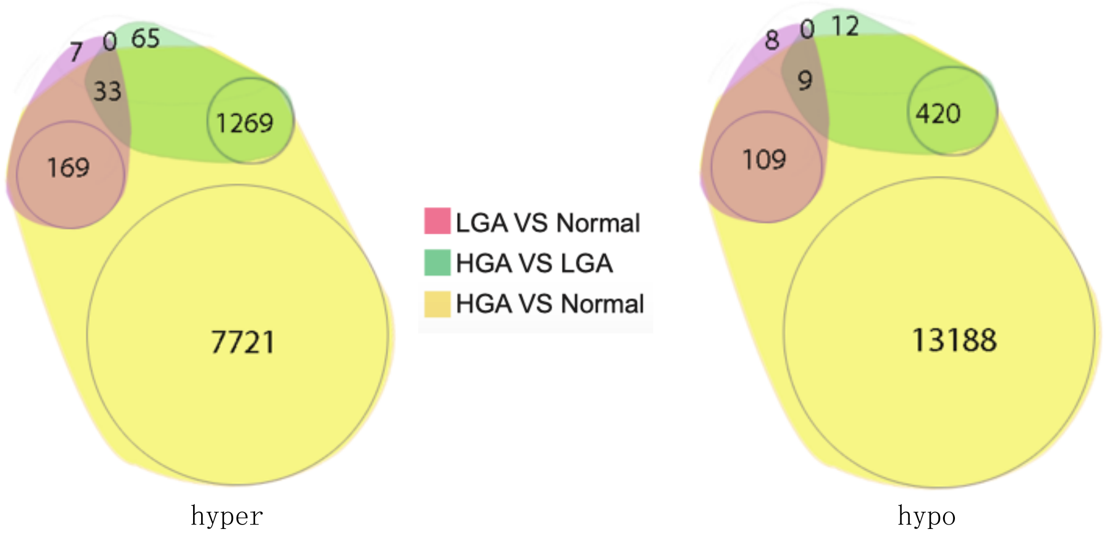

Figure S2. Methylation levels of 209 hyper-methylated DMSs in our datasets (Normal, LGA, HGA), 51 public adenoma datasets (AD) and 504 public datasets cancer (Cancer).

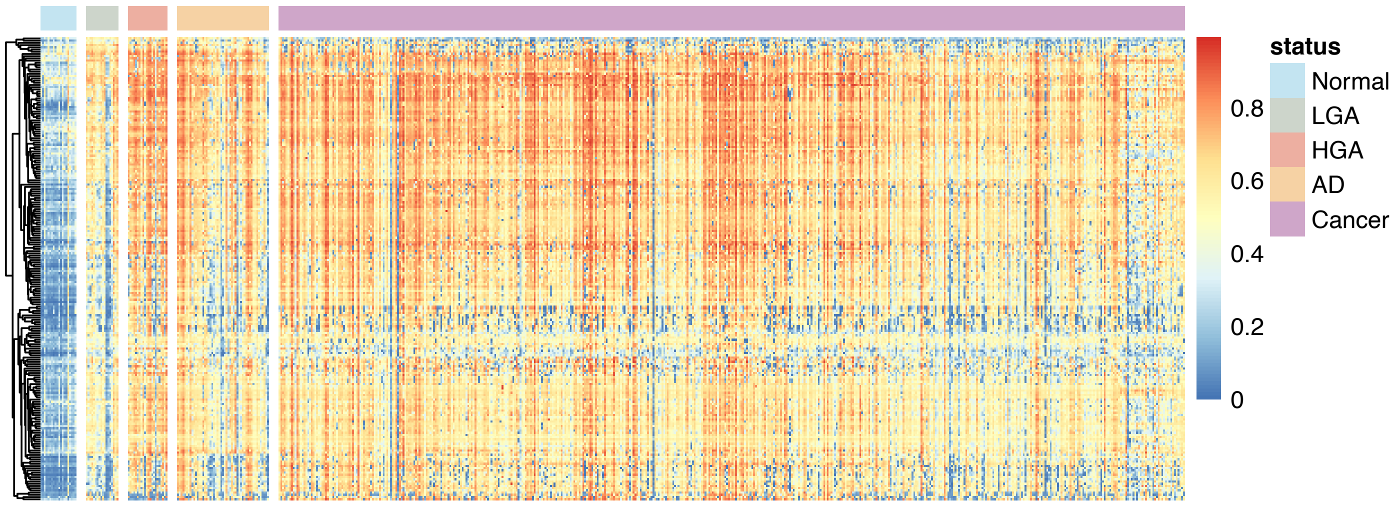

AD, Adenoma; HGA, High grade adenoma; LGA, Low grade adenoma.

Figure S3. Methylation levels of the 441 hypo-methylated DMSs in our datasets (Normal, LGA, HGA), and 504 public cancer datasets (Cancer).

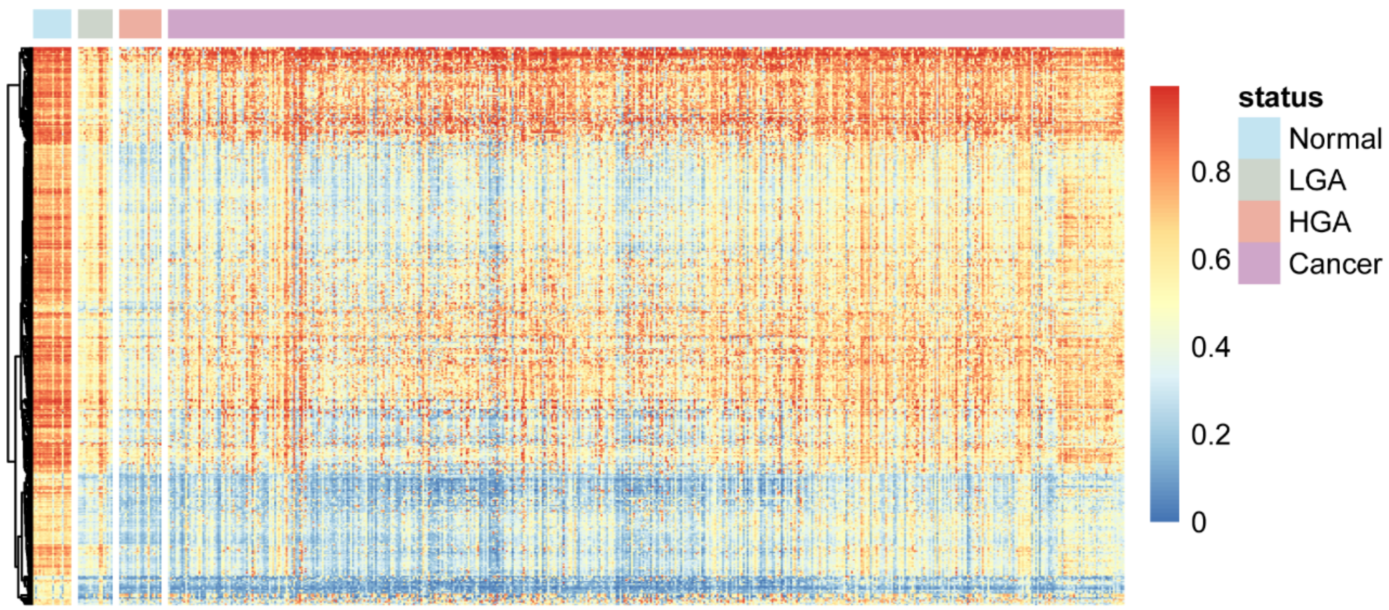

HGA, High grade adenoma; LGA, Low grade adenoma.

Figure S4. Receiver operating characteristic curve for the performance of *ACSS3* in the prediction of colorectal adenoma and cancer.

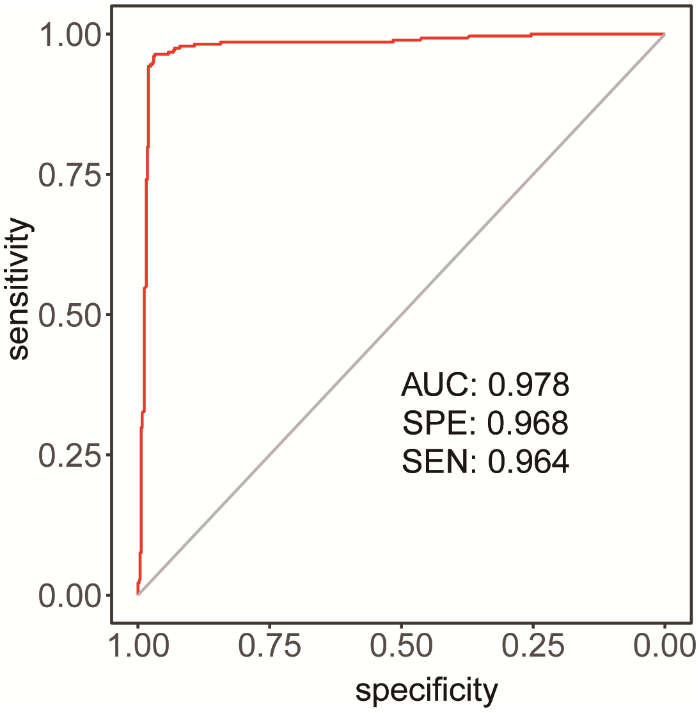

AUC, Area under the ROC Curve; SPE, specificity; SEN, sensitivity.

Figure S5. The correlation of methylation levels (beta value) between *ADHFE1* and *SEPT9*(Pearson correlation coefficient=0.77, p-value <2.2e-16).

.

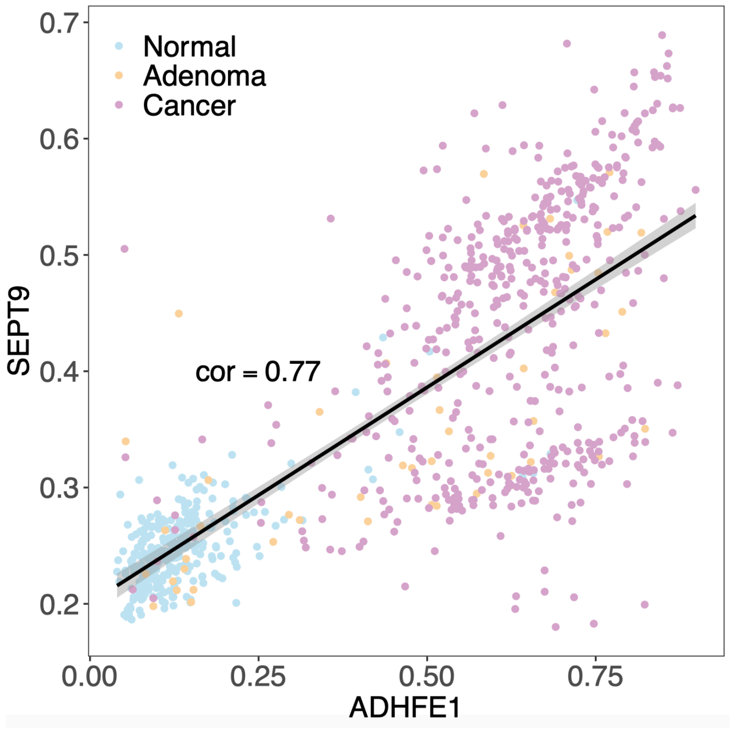

Figure S6. The DNA methylation level of *ADHFE1* promoter in the public datasets obtained from whole blood (WB), normal colorectal tissue (Normal), adenoma (AD) and cancer (Cancer).

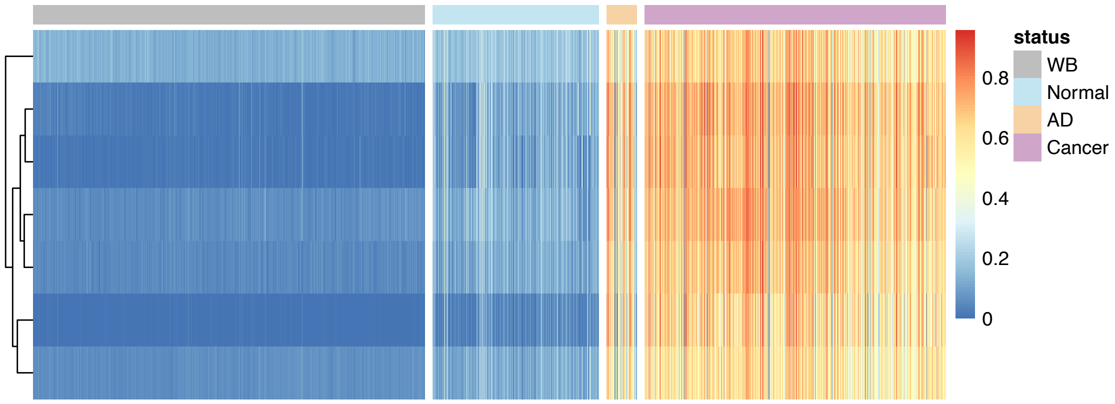

Figure S7. Methylation levels in our normal samples (Normal) and public datasets (WB, AD, and Cancer) for 209 hyper-methylated DMSs identified in LGA. (A) Public datasets include 656 whole blood (WB) samples and 51 adenoma (AD) and 504 cancer samples. (B) Density plot of DNA methylation values (beta values) of 209 sites in all samples.

A

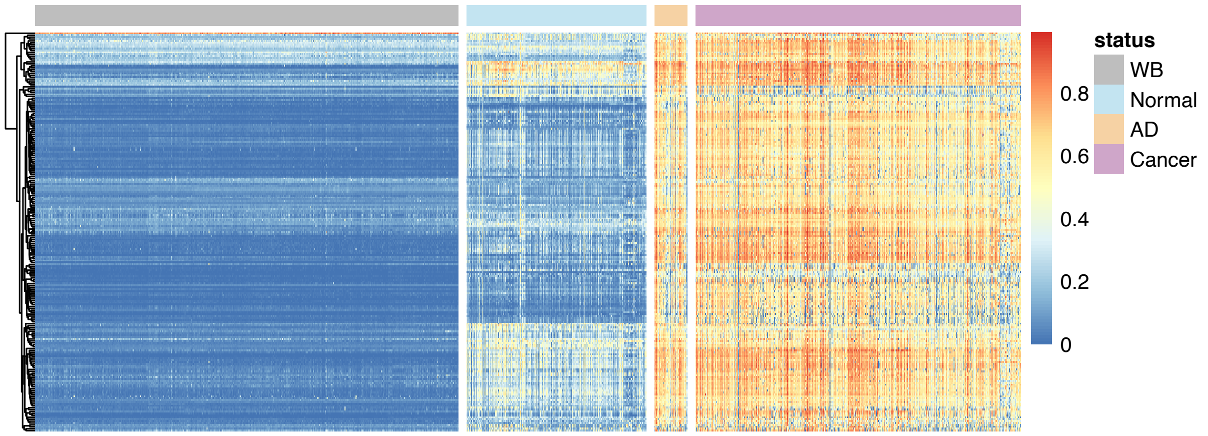

B

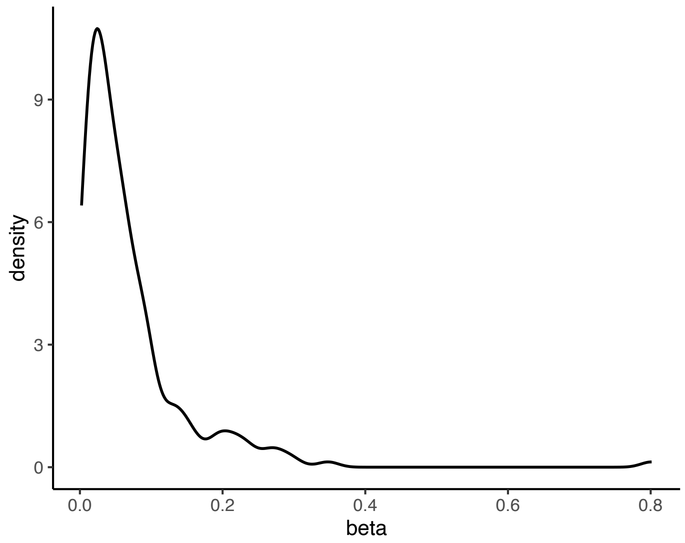

Figure S8. Density of all beta values (top panel) and multi-dimensional scaling (MDS) (bottom panel) according to 10000 most variable positions for samples in high grade adenoma (HGA), low grade adenoma (LGA) and normal tissue (CTR) before (A) and after (B) normalization. The density plots demonstrate relatively consistent distribution of beta values across all sample even before normalization. According to the 10000 most variable positions, the MDS plots show the samples clustering according to their pathological groups. Particularly, normal tissues (green dots) have high homogeneity, forming a dense cluster in the bottom left corner of the plot; and heterogeneity of samples increase from normal tissues, low-grade lesions (orange dots) to high-grade ones (purple dots), as sample dots having increased spacing from bottom left to top right.

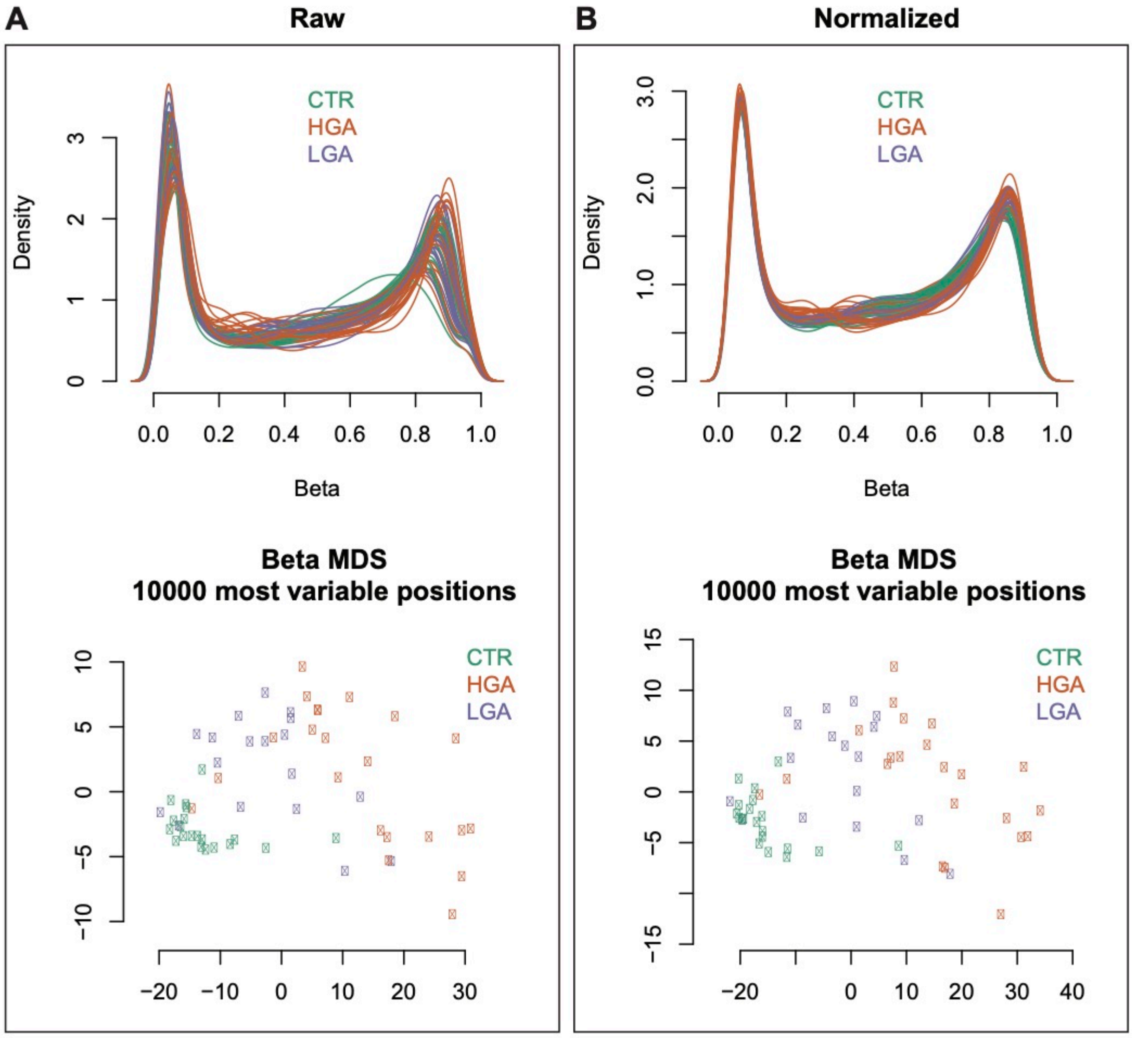

Figure S9. Multi-dimensional scaling (MDS) according to 1000 (Top panel) and 20000 (Bottom panel) most variable positions for samples in high grade adenoma (HGA), low grade adenoma (LGA) and normal tissue (CTR) before (**A**) and after (**B**) normalization.

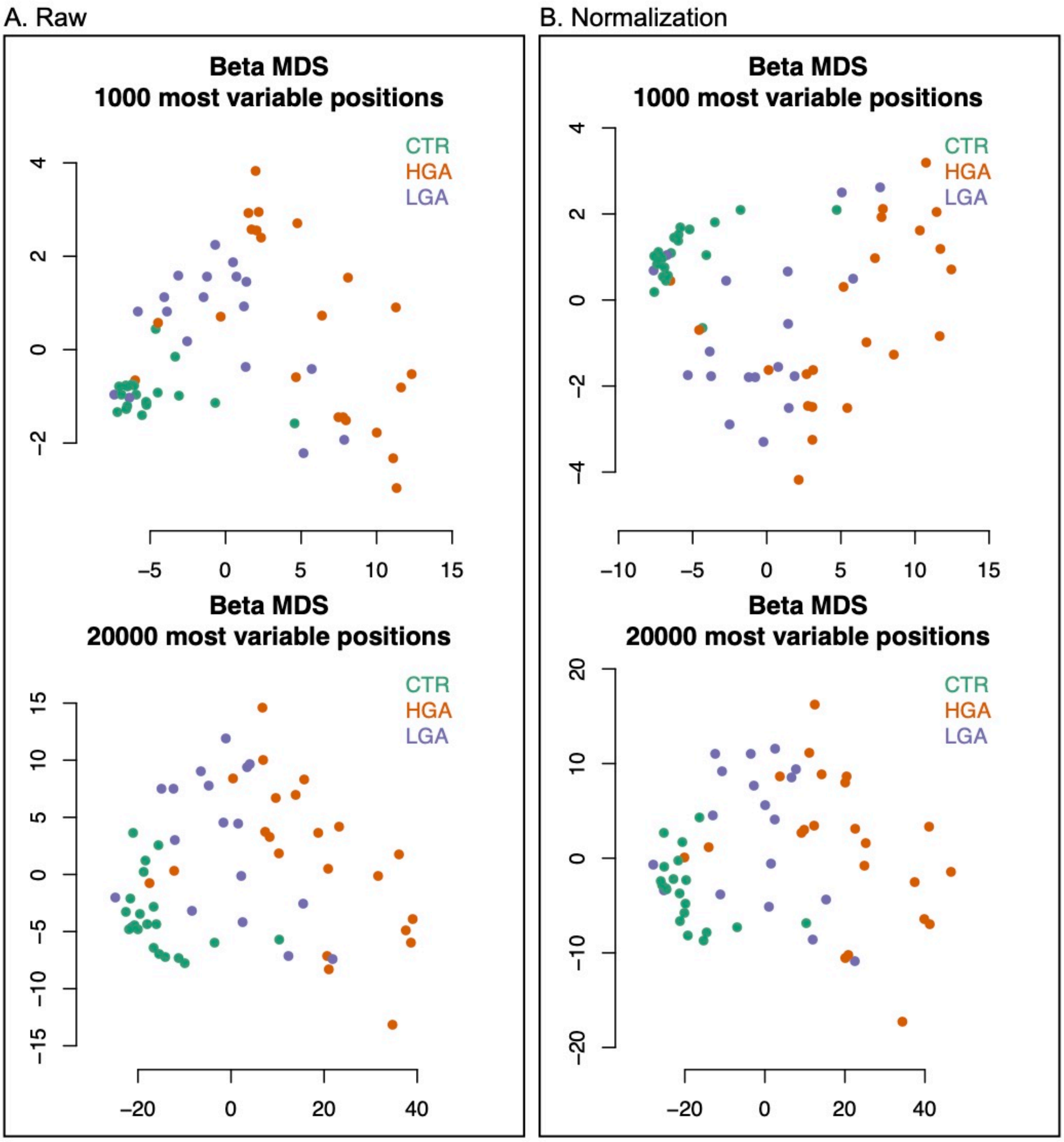

Table S1. 209 hypermethylated sites identified in low grade adenoma (LGA)

Site	Genome Build	CHR	MAPINFO	Normal	LGA	HGA	adjust_Pvalue NvsLGA	adjust_Pvalue LGAvsHGA	adjust_Pvalue NvsHGA
cg00020052	37	2	68546899	0.23136902	0.466805272	0.635286005	0.000898052	0.03969754	8.01E-09
cg00040312	37	12	54424679	0.24658703	0.533574522	0.53622255	0.000197276	0.984520615	6.46E-05
cg00282249	37	13	37005566	0.36844691	0.583477789	0.791529464	0.006565803	0.014133303	3.84E-09
cg00355909	37	16	66638320	0.13975618	0.401161351	0.561495149	0.000840753	0.064344031	1.63E-08
cg00733780	37	10	26505127	0.16012539	0.426726882	0.665615991	0.003593066	0.030625483	1.56E-08
cg00813378	37	1	1475209	0.30676306	0.544580617	0.714805482	0.00016874	0.013619349	8.14E-12
cg00852573	37	6	73331405	0.236188	0.456947117	0.638643491	0.001665943	0.021093417	3.75E-10
cg00875960	37	19	12267308	0.10272328	0.327694726	0.425758516	0.001322148	0.375074123	9.34E-05
cg01030534	37	7	143579698	0.32621562	0.545651991	0.755693005	0.004370561	0.019170311	3.23E-08
cg01218180	37	6	168079427	0.38557129	0.586262172	0.609416336	0.000925468	0.844305442	0.001945823
cg01423964	37	1	111217575	0.17318003	0.378962396	0.5024522	0.000791049	0.048748879	4.68E-10
cg01434608	37	16	66637927	0.14964989	0.483057287	0.6921871	8.95E-05	0.027798156	3.61E-10
cg01645753	37	15	83316734	0.12867791	0.342694529	0.487296186	0.000851587	0.061416152	7.19E-09
cg01733176	37	4	111561070	0.14940011	0.352511639	0.455303493	0.005952565	0.31219778	3.14E-06
cg02183671	37	4	15779999	0.21345019	0.439392783	0.552727605	0.000174878	0.135764333	8.97E-08
cg02379560	37	4	40859280	0.40072311	0.604289656	0.7104728	0.001468454	0.139886289	3.27E-08
cg02721000	37	12	54441213	0.24752654	0.460540631	0.481018005	0.00215586	0.864691564	0.000394073
cg02764245	37	2	66803033	0.27810112	0.492759667	0.675875877	0.004900115	0.045948804	1.39E-08
cg02864844	37	7	149917263	0.2320133	0.510892178	0.728712064	0.000863947	0.02435264	1.16E-09
cg02866454	37	10	7453455	0.1643021	0.427882621	0.590275759	0.001679439	0.1615268	2.03E-06
cg02899206	37	10	1779835	0.09471257	0.305062148	0.495525459	0.003073409	0.0806002	6.28E-06
cg03078363	37	12	54408664	0.23129798	0.441945717	0.519815255	0.002368046	0.439506168	9.61E-06
cg03117976	37	18	11149435	0.3027036	0.522335483	0.666185114	0.002263847	0.103753323	8.10E-08
cg03225210	37	7	143579951	0.19720376	0.430098596	0.677633299	0.001239685	0.01023122	3.04E-09
cg03257172	37	1	200010283	0.21446685	0.554361689	0.720850982	2.22E-05	0.027849179	1.31E-11
cg03279535	37	2	31360817	0.09539126	0.30993486	0.483015598	0.00032385	0.04246646	2.76E-07
cg03377355	37	22	39853885	0.13826644	0.349524212	0.496863701	0.001864993	0.110921965	1.81E-06

cg03596016	37	1	246952362	0.39488229	0.648997217	0.695571504	5.67E-05	0.62895814	3.38E-05
cg03701427	37	10	82117089	0.26275661	0.526916167	0.688774482	0.000577396	0.097578083	3.12E-07
cg03807298	37	5	174159039	0.31149649	0.594163906	0.732847723	6.15E-05	0.040692162	1.05E-10
cg03940848	37	1	229543112	0.16761911	0.380126936	0.578137168	0.003376184	0.040990526	1.50E-07
cg03943773	37	4	111552308	0.15552379	0.35999463	0.323877935	0.018927843	0.793411377	0.015851446
cg03958798	37	10	106400686	0.24316737	0.444487456	0.647952041	0.003990279	0.030678859	2.21E-07
cg04021697	37	1	3567303	0.26262794	0.487904978	0.717875327	0.003273044	0.012568362	1.25E-10
cg04249706	37	10	11206772	0.46460104	0.697093106	0.764317677	0.000105792	0.068093127	3.71E-07
cg04286194	37	5	54519023	0.42022171	0.641657806	0.748797173	0.000213123	0.09562058	3.28E-08
cg04308769	37	1	200007623	0.34395261	0.560091261	0.695229973	3.70E-05	0.011297606	3.65E-11
cg04366687	37	8	145107199	0.23648703	0.475554278	0.587057318	0.000208896	0.124390075	2.16E-08
cg04897742	37	1	16085148	0.07020286	0.292923521	0.476116717	8.90E-05	0.019075262	2.29E-08
cg04996873	37	1	14925992	0.32198241	0.560278044	0.680339914	0.00026173	0.118268026	2.60E-08
cg05089090	37	13	37005570	0.33815074	0.568008194	0.784545614	0.002766968	0.010218487	7.78E-10
cg05143887	37	12	81471938	0.30218817	0.503878411	0.559543672	0.000236301	0.425037083	2.04E-06
cg05297854	37	22	50623687	0.37414293	0.576275728	0.708728995	0.004354577	0.091493792	6.98E-07
cg05362548	37	8	11205166	0.17393834	0.379020909	0.539407434	0.011859945	0.179406536	2.67E-05
cg05447008	37	6	73331114	0.15404172	0.367787363	0.60889319	0.003320673	0.010990564	5.72E-09
cg05494604	37	13	23733862	0.29113246	0.493931233	0.682258318	0.003627745	0.020980083	1.12E-09
cg05590196	37	1	33220274	0.34146534	0.565907633	0.660017491	0.000431062	0.191687144	2.16E-08
cg05827631	37	1	121261404	0.29990762	0.509273911	0.628046886	0.000132054	0.019841876	1.85E-09
cg05901579	37	15	88800567	0.268582	0.490748681	0.704944941	0.008616924	0.024812339	2.29E-09
cg05915293	37	16	2041512	0.18901523	0.413525065	0.597378009	0.001198049	0.062850114	7.60E-07
cg05966978	37	10	11207437	0.33117169	0.563978028	0.647202436	3.59E-05	0.038327671	2.32E-08
cg06223767	37	6	393616	0.19745887	0.42543624	0.568273864	0.004387658	0.197026637	4.29E-06
cg06319475	37	8	145105829	0.20025257	0.459004558	0.607950418	0.00119891	0.12105156	1.22E-07
cg06392169	37	6	391936	0.21707642	0.4782926	0.623024132	0.000727694	0.11420806	1.44E-07
cg06611358	37	19	37464417	0.23743883	0.439464383	0.613484223	0.001076645	0.024453293	8.37E-09
cg06750832	37	1	111217691	0.22437946	0.432035202	0.600178445	0.001162531	0.017384841	7.80E-11
cg06970370	37	1	121261077	0.41841467	0.634441194	0.736875159	0.000257709	0.050626099	8.34E-09

cg07080358	37	2	68546507	0.17231872	0.447836992	0.587517359	0.000282088	0.111095224	5.36E-09
cg07208853	37	1	200005219	0.26654876	0.522160978	0.68638435	0.000162658	0.012673314	1.70E-10
cg07594636	37	2	68547088	0.19553145	0.438786299	0.540901895	0.001289591	0.298849976	3.28E-06
cg07642043	37	16	10276674	0.24798141	0.4605715	0.65565925	0.001397695	0.010842146	8.63E-10
cg07655636	37	19	22034418	0.10441059	0.317618068	0.533180755	0.001208299	0.013191896	2.32E-09
cg07794500	37	1	121261369	0.10319584	0.338501566	0.524292668	0.000246717	0.012612491	7.75E-11
cg07808555	37	1	111217712	0.20697252	0.446212363	0.668583259	0.001237626	0.011894518	1.42E-10
cg07900968	37	19	36909326	0.11971934	0.333334221	0.534461161	0.010959468	0.066482339	1.16E-06
cg07926895	37	1	200005833	0.22162016	0.449776522	0.575958259	0.000104459	0.037871047	1.97E-11
cg07981013	37	7	156795767	0.40524431	0.619073344	0.645985473	1.53E-05	0.675611608	1.11E-05
cg08002883	37	3	192127457	0.22483468	0.441932864	0.648836123	0.005512088	0.015235834	1.33E-09
cg08430489	37	7	142494953	0.11813035	0.338193283	0.48858393	0.004169911	0.176474035	1.20E-05
cg08516516	37	5	115152492	0.23909601	0.446184928	0.573900282	0.001018973	0.078599784	4.97E-08
cg08567279	37	13	53313529	0.16682558	0.397564093	0.53051646	0.000769091	0.214394127	2.94E-05
cg08682544	37	16	66638438	0.10423291	0.316370249	0.492182223	0.004263074	0.088408107	2.43E-06
cg08684639	37	4	176987313	0.07221805	0.295100084	0.518040873	0.003888031	0.03238548	1.20E-07
cg08738570	37	1	1475265	0.41814081	0.650283056	0.793225786	9.02E-05	0.018956077	5.53E-11
cg08864645	37	6	33943929	0.33360094	0.552362239	0.602552964	0.000149779	0.539660217	6.96E-06
cg09087503	37	7	127672473	0.14065394	0.458066792	0.563698418	1.12E-05	0.039550764	1.06E-10
cg09179211	37	21	42689051	0.42713669	0.670851611	0.701057123	3.71E-06	0.565822304	6.83E-09
cg09241022	37	14	29254530	0.23672459	0.457097265	0.706799095	0.006909574	0.013037309	9.26E-10
cg09257332	37	19	36909913	0.15465059	0.358610443	0.523989776	0.005840896	0.107216149	2.67E-06
cg09296001	37	7	127672564	0.11960398	0.486328606	0.676162732	1.73E-05	0.01132836	9.16E-12
cg09493063	37	7	142494985	0.17287638	0.433485374	0.5905749	0.000403188	0.089282317	7.56E-08
cg09731694	37	9	132383003	0.31731967	0.549861672	0.697157327	6.31E-05	0.013329171	6.40E-12
cg10580144	37	2	61372316	0.26211866	0.462415078	0.621634555	0.0006913	0.033319568	1.20E-08
cg10640119	37	9	132383095	0.18027348	0.386498772	0.440187277	3.14E-05	0.335376832	6.09E-09
cg10967023	37	12	115134886	0.26662428	0.477118411	0.662906236	0.000455678	0.010688304	1.75E-10
cg10972873	37	1	200007592	0.1915096	0.482346189	0.613956827	2.18E-05	0.037605026	9.16E-12
cg11017065	37	11	128564874	0.13089261	0.40331417	0.502189123	0.008609173	0.539373465	0.000455365

cg11167100	37	15	83776269	0.15124129	0.376860035	0.565246468	0.000729525	0.014732419	2.84E-11
cg11213520	37	12	113901529	0.29873246	0.504726061	0.697847714	0.011895421	0.042019711	4.18E-09
cg11267630	37	17	46811527	0.168021	0.372076871	0.245838204	0.014284331	0.325324399	0.374715445
cg11328303	37	10	26505440	0.34449526	0.562013717	0.717911468	0.00162688	0.028249755	1.05E-09
cg11573679	37	2	68546467	0.19505713	0.464358673	0.608779686	0.000602901	0.141005497	8.89E-08
cg11585893	37	12	54447430	0.32405063	0.532029239	0.480332877	0.001844368	0.584534459	0.002128927
cg11595545	37	1	111217497	0.2776204	0.515101217	0.655121436	0.000747477	0.035863616	3.41E-09
cg11646192	37	4	75230615	0.34960866	0.557918194	0.592499577	1.52E-05	0.440035744	3.11E-07
cg11739675	37	12	104852151	0.09531603	0.312625525	0.453399855	0.001501001	0.167645331	9.90E-06
cg12374721	37	17	46799640	0.24173976	0.450211318	0.256488038	0.022525306	0.134678458	0.871702093
cg12628196	37	7	127672458	0.15193655	0.501270849	0.651546045	1.34E-05	0.018479175	9.16E-12
cg12652174	37	7	142494773	0.15590702	0.360770199	0.51864221	0.00954251	0.144834836	9.24E-06
cg12863545	37	17	8869136	0.13106992	0.343828784	0.448385975	0.000704801	0.230257035	3.20E-06
cg12973591	37	7	93519473	0.13710771	0.342440626	0.555263036	0.001171484	0.014445413	1.07E-08
cg13273396	37	12	81471867	0.40574718	0.609897189	0.6926126	0.000424142	0.254338652	1.14E-06
cg13405887	37	9	132382812	0.23655422	0.518834067	0.636290527	1.92E-05	0.02609117	3.31E-11
cg13484546	37	1	16084939	0.27112703	0.546401128	0.696715418	6.61E-05	0.054760235	2.22E-09
cg13702005	37	12	103889789	0.22617616	0.430759778	0.577339564	0.000421791	0.037413136	6.80E-09
cg13895235	37	7	752292	0.28076957	0.522974644	0.7541878	0.00384676	0.016638384	1.38E-10
cg14015706	37	9	132382433	0.14041415	0.496310872	0.669601223	1.46E-05	0.015271792	4.72E-12
cg14443519	37	6	29760410	0.12135186	0.346379128	0.5130659	0.000814657	0.039915164	1.22E-08
cg14470895	37	5	115152431	0.23202586	0.438701777	0.587899191	0.001797933	0.064930952	1.60E-07
cg14515424	37	2	97193524	0.26217152	0.48441905	0.617911059	0.000389318	0.061858791	2.41E-09
cg14531560	37	1	16085371	0.17749578	0.384927916	0.499278786	0.000180783	0.070232982	3.75E-09
cg14676825	37	17	27038886	0.21555884	0.416171528	0.529094294	0.002189807	0.155393155	7.95E-07
cg15313459	37	2	68547052	0.27302266	0.506919083	0.624938191	0.00062193	0.12371293	1.58E-08
cg15760257	37	17	26699169	0.09469316	0.300719943	0.428823386	0.002920958	0.21410497	9.56E-06
cg15929698	37	7	24323792	0.28823611	0.506507389	0.660767768	0.000909298	0.038755244	6.87E-10
cg16283362	37	7	156795914	0.39542097	0.681558561	0.70313515	2.62E-05	0.830386619	8.32E-05
cg16306898	37	1	1475675	0.08378309	0.287352092	0.548531944	0.004801796	0.008718217	1.37E-09

cg16328106	37	2	61372138	0.42593166	0.650445444	0.766956477	0.000436801	0.084647575	1.30E-08
cg16335762	37	16	66637919	0.25767907	0.551577161	0.687751986	9.73E-05	0.072445234	9.08E-10
cg16376000	37	3	192127330	0.1977144	0.433706533	0.637475205	0.001238895	0.012487491	4.91E-11
cg16519300	37	1	16084830	0.34615247	0.557758456	0.679838223	0.00022071	0.083859179	6.57E-08
cg16818993	37	10	7453471	0.12319822	0.357807609	0.496737362	0.002087463	0.185186315	3.15E-06
cg16848116	37	19	57862627	0.10915421	0.320346518	0.505729096	0.001839052	0.054014676	6.57E-07
cg16918989	37	17	8869213	0.14330585	0.371879237	0.509159732	0.002699492	0.223198376	2.62E-05
cg16933229	37	4	111561352	0.29900199	0.503869439	0.6433803	0.002265024	0.074204209	1.80E-09
cg16940012	37	5	174159564	0.34380108	0.563916339	0.679196955	0.000104649	0.061462955	2.93E-10
cg16964348	37	7	24323799	0.22843381	0.532019886	0.697218914	0.000314299	0.069454823	7.19E-10
cg16993043	37	1	200008026	0.18576853	0.404809789	0.541815682	0.00029003	0.062515195	1.69E-08
cg17099397	37	1	108508185	0.2157719	0.421123528	0.541775714	0.00138503	0.209575782	1.20E-05
cg17128947	37	4	779480	0.30317507	0.598843256	0.738379686	6.42E-05	0.083601599	3.54E-09
cg17228900	37	6	391764	0.10086644	0.314322966	0.585962585	0.00810266	0.017659596	1.81E-07
cg17301223	37	8	145106438	0.21589771	0.558372257	0.717536483	0.000529411	0.145729355	4.52E-08
cg17338208	37	7	93520024	0.11281649	0.317955754	0.561411876	0.003433642	0.01123065	2.95E-09
cg17394649	37	6	29760164	0.30998724	0.514208433	0.650893045	0.000163494	0.041285642	2.42E-08
cg17418463	37	1	35395541	0.09753415	0.311559294	0.478673967	0.002631177	0.08478803	1.03E-06
cg17651959	37	20	21498100	0.27121404	0.5118559	0.699027482	0.000768961	0.038850635	2.04E-08
cg17698295	37	8	145106299	0.17974763	0.499737868	0.677240764	0.000643804	0.083023014	1.53E-08
cg17853216	37	10	81002480	0.29387326	0.526592414	0.691552	0.001330294	0.021859693	1.03E-08
cg17892556	37	19	12267464	0.18447788	0.425167174	0.539363618	0.00129357	0.285368842	1.20E-05
cg17987112	37	5	174158654	0.26887041	0.567894911	0.712589064	2.40E-05	0.029731176	6.92E-12
cg18110165	37	5	49736988	0.23066055	0.447511756	0.520510282	4.90E-05	0.142908484	2.33E-10
cg18126097	37	1	200014673	0.35108931	0.583348967	0.752481505	0.000205295	0.011870629	2.30E-11
cg18412834	37	20	61885291	0.13028249	0.342987363	0.584163317	0.00687043	0.017752436	5.54E-09
cg18601167	37	7	752286	0.20045765	0.436683035	0.715815723	0.009388186	0.0122307	4.03E-10
cg18638914	37	4	779568	0.13458443	0.362573881	0.563422922	0.001455331	0.037502727	1.25E-07
cg18794404	37	10	22542024	0.19588244	0.400244149	0.60372107	0.014737974	0.043300788	3.73E-07
cg18845377	37	7	156795892	0.16616357	0.4313026	0.498587564	9.41E-06	0.480093051	2.66E-05

cg18856388	37	5	1875886	0.28775304	0.517882422	0.707723314	0.001377434	0.020539188	8.72E-10
cg19211077	37	19	21658001	0.26794163	0.476968567	0.686166395	0.00222849	0.010530037	9.79E-11
cg19256731	37	20	1874527	0.4890894	0.709429189	0.766691545	2.61E-05	0.187733159	4.02E-08
cg19602081	37	4	111553161	0.12267959	0.324632192	0.213142062	0.024239798	0.371977737	0.17908688
cg20072171	37	3	62356962	0.32313188	0.532037894	0.690760891	0.003903988	0.081093118	2.82E-07
cg20230721	37	7	93519855	0.19841991	0.406294992	0.673774695	0.003482302	0.00632457	1.04E-10
cg20275528	37	17	75369484	0.22486749	0.469561817	0.645812177	0.001108948	0.018068934	1.79E-09
cg20506184	37	10	7453329	0.15607953	0.405638556	0.595730313	0.000733362	0.056525135	3.02E-07
cg21097881	37	7	24323939	0.27814463	0.48003987	0.591711932	0.001808281	0.106376689	1.59E-08
cg21101720	37	17	27940509	0.12959639	0.346317797	0.52536108	0.002713125	0.057644253	1.09E-07
cg21109167	37	3	37034084	0.30884542	0.516913494	0.499128399	2.48E-05	0.846225033	0.001202377
cg21229570	37	12	115124768	0.36789011	0.597167339	0.698927241	0.000108591	0.16817136	1.06E-07
cg21277995	37	6	393239	0.16510713	0.442939307	0.590890232	0.001392841	0.17833566	5.90E-07
cg21583226	37	2	31360693	0.03941112	0.291918393	0.44265212	0.000812392	0.16921246	3.16E-06
cg21621906	37	7	142494914	0.08387588	0.297790843	0.43538198	0.006621702	0.274107072	0.000149788
cg21785034	37	7	142494439	0.27744545	0.480007233	0.652978895	0.005805822	0.059830601	3.54E-07
cg22488797	37	5	134363324	0.31080755	0.529096983	0.685790845	0.000148575	0.011644187	1.64E-11
cg22747076	37	12	54447873	0.16273474	0.386115763	0.63331148	0.009333099	0.033784685	2.47E-07
cg22882523	37	8	145107012	0.28860428	0.530474328	0.604519914	0.000174336	0.196429152	4.29E-07
cg23002761	37	1	16085642	0.23213171	0.433610653	0.555579925	0.000243236	0.058782133	5.23E-09
cg23141355	37	3	44063593	0.1858334	0.399355892	0.590379123	0.007002546	0.032048243	1.50E-09
cg23180938	37	5	115152485	0.30669453	0.520394339	0.652532795	0.000856829	0.048876802	4.47E-09
cg23241781	37	14	70653964	0.28192342	0.50393235	0.650799795	0.00023633	0.030252572	3.19E-09
cg23297477	37	16	66638293	0.14618709	0.445816128	0.633244426	0.000721243	0.054637713	4.27E-09
cg23614791	37	12	81471754	0.22725169	0.504489091	0.594297345	9.24E-05	0.264964468	4.72E-08
cg23663774	37	4	111555440	0.1940965	0.417480166	0.497803497	0.013101679	0.553387459	0.00016661
cg23685155	37	12	54396440	0.2267097	0.460537056	0.413707702	0.000188812	0.608666131	0.00070961
cg23927970	37	11	504933	0.3785112	0.581507511	0.654239836	0.000206189	0.351341961	5.28E-06
cg23965931	37	4	184828386	0.20583214	0.446963956	0.450285145	0.000119059	0.976611774	7.92E-05
cg23977631	37	2	100938799	0.0500622	0.264478068	0.427493988	0.00134448	0.063121914	9.34E-08

cg24171907	37	2	68546579	0.14914202	0.427902529	0.58113185	0.000329976	0.074527655	5.70E-09
cg24242823	37	7	24323675	0.15220519	0.419191342	0.580060555	0.000227216	0.054904154	3.33E-09
cg24334591	37	13	37005562	0.33770585	0.551817906	0.768858068	0.005763339	0.011581796	3.41E-09
cg24724633	37	19	36909413	0.16374906	0.409758896	0.598540674	0.005468546	0.085364716	1.93E-07
cg24799921	37	10	77168180	0.25247683	0.460714489	0.679979359	0.00807251	0.017135973	2.91E-08
cg24857620	37	8	53851707	0.18948399	0.423688327	0.643781686	0.003089661	0.030693307	2.19E-08
cg24885417	37	7	24323764	0.20644837	0.423838912	0.549854986	0.000584477	0.072987204	7.71E-09
cg25200152	37	1	147790458	0.31490991	0.516403539	0.666488345	0.00463963	0.087053495	2.54E-07
cg25209842	37	10	103536342	0.31100206	0.521462467	0.649740736	0.000282517	0.047555107	2.01E-09
cg25223771	37	8	145105503	0.26538792	0.482063944	0.608795473	0.002199743	0.166703939	8.83E-07
cg25358039	37	12	81471757	0.21840726	0.466764444	0.535818175	7.02E-05	0.361714952	1.42E-07
cg25652029	37	2	182321786	0.26622392	0.488845633	0.678604982	0.000744622	0.011495101	3.44E-10
cg25737323	37	2	29338100	0.3435614	0.54400485	0.692333405	0.002481309	0.035383537	1.63E-08
cg25761326	37	10	11206792	0.36939478	0.586160006	0.674891786	4.21E-05	0.040485268	1.46E-08
cg25832771	37	8	72756058	0.14874316	0.350615372	0.51421722	0.002341418	0.035667599	1.68E-08
cg25840208	37	2	31457043	0.06924076	0.284220198	0.368918062	0.007393468	0.504799642	0.000318523
cg25971727	37	12	81471533	0.11960814	0.331226627	0.4357716	0.000139544	0.168899219	5.24E-07
cg26036626	37	1	16085597	0.26460909	0.568858889	0.71711015	6.69E-05	0.060557988	7.37E-10
cg26107890	37	3	124860686	0.47352978	0.680593417	0.785735245	4.92E-05	0.010598622	3.00E-09
cg26171871	37	7	156796011	0.30307987	0.570377317	0.543243115	9.12E-05	0.753393962	0.0008557
cg26199906	37	8	145106246	0.22989506	0.461937456	0.611742764	0.000586303	0.051665177	2.13E-08
cg26246807	37	19	58095659	0.37561466	0.597706644	0.758164927	0.001412268	0.018672059	4.89E-09
cg26251325	37	5	54518745	0.19233364	0.427734239	0.600361759	0.000245109	0.032650371	1.15E-08
cg26256223	37	8	145106582	0.19397929	0.491213063	0.629234711	0.000382574	0.145586962	4.91E-08
cg26272220	37	12	81471884	0.30593378	0.534207867	0.622310707	0.000348025	0.247982834	2.59E-07
cg26530758	37	8	41754060	0.36181951	0.561986728	0.726832173	0.005275407	0.062471324	6.16E-08
cg26560414	37	16	66638433	0.10853241	0.326809853	0.533422231	0.004001557	0.046372404	7.78E-07
cg26654807	37	10	81002218	0.36274442	0.586278106	0.703355064	0.001518356	0.053832238	3.28E-07
cg26878816	37	10	7452598	0.10888183	0.376759237	0.533090574	0.000431883	0.147645742	2.49E-06
cg26978668	37	10	7453452	0.13735535	0.358732524	0.491510505	0.000922558	0.117953075	2.53E-07

cg26998044	37	17	8869155	0.14502449	0.388268417	0.52828789	0.000774818	0.154987662	1.89E-06
cg27659049	37	12	115173713	0.47022311	0.684768456	0.703127759	0.000230007	0.863564579	0.000376123

Table S2 Differentially methylated regions (DMRs) between low grade adenoma (LGA) and Normal samples

Location	Gene	CHR	MAPINFO	Number of sites	Designed Probes	Normal	LGA	HGA	Different β	P value	Adjusted P value
TSS1500	A4GNT	3	137851874/137852643/137851776	3	cg18931888/cg19734045/cg23669440	0.58586656	0.41358386	0.304446464	-0.1722827	9.43E-07	3.76E-05
TSS1500	ACSS3	12	81471419/81471311/81471533	3	cg00354572/cg08314603/cg25971727	0.21885051	0.4004886	0.471809096	0.18163809	2.00E-06	5.51E-05
TSS1500	AGR2	7	16845331	1	cg12565635	0.55886628	0.40800231	0.323608105	-0.150864	2.62E-08	9.16E-06
TSS1500	APOBEC1	12	7819180	1	cg16879115	0.65364044	0.46522647	0.339518605	-0.188414	1.00E-07	1.34E-05
TSS1500	ARHGAP28	18	6833417	1	cg03026004	0.53171601	0.34391694	0.202823204	-0.1877991	1.25E-05	1.67E-04
TSS1500	AVIL	12	58211193/58210661/58210878/58210716	4	cg01424889/cg04046364/cg06317209/cg26620147	0.54993057	0.39294288	0.316282894	-0.1569877	8.42E-09	5.71E-06
TSS1500	BRF2	8	37707692/37708609/37708905	3	cg19904653/cg20466478/cg22871204	0.46839186	0.31684988	0.205657763	-0.151542	1.10E-06	4.11E-05
TSS1500	BSG	19	571107/571204/571744/571321/571416/571802/572309/571323/570458	9	cg01532103/cg05622915/cg07513435/cg13578393/cg17522907/cg18341969/cg22431486/cg25660646/cg26529300	0.78931954	0.60699698	0.594050391	-0.1823226	8.82E-07	3.70E-05
TSS1500	C16orf89	16	5117565/5116674	2	cg02878222/cg27664407	0.54217156	0.38745737	0.294618095	-0.1547142	3.10E-06	7.17E-05
TSS1500	C20orf79	20	18794097/18793817	2	cg08567760/cg08716736	0.69846479	0.54324435	0.404466973	-0.1552204	3.75E-05	3.44E-04
TSS1500	C7orf16	7	31726494/31725973/31725748	3	cg01001101/cg04866380/cg23348723	0.53779831	0.38658703	0.287442248	-0.1512113	4.10E-07	2.44E-05
TSS1500	CCDC50	3	191046547/191046380	2	cg19066520/cg25342125	0.60527054	0.41069312	0.355004173	-0.1945774	1.08E-07	1.39E-05
TSS1500	CCL8	17	32645753/32645806/32645748	3	cg00038857/cg04656009/cg27000831	0.60098327	0.43562701	0.316505959	-0.1653563	6.09E-06	1.04E-04
TSS1500	CD207	2	71063282/71064086	2	cg08989942/cg26946836	0.62314848	0.46427469	0.335575727	-0.1588738	1.06E-05	1.48E-04
TSS1500	CDX2	13	28544814/28543520/28544592/28544760	4	cg01499197/cg02055963/cg07984133/cg16201038	0.44334652	0.28001494	0.431752602	-0.1633316	7.46E-04	3.25E-03
TSS1500	CHST15	10	125806958/125807504/125807047	3	cg06034255/cg09595290/cg16449489	0.71921853	0.53196769	0.380170368	-0.1872508	6.30E-07	3.11E-05
TSS1500	CMTM3	16	66638320/66637927/66637919/66638293/66638210	5	cg00355909/cg01434608/cg16335762/cg23297477/cg26658728	0.17331806	0.47040298	0.643669665	0.29708492	9.33E-06	1.36E-04

TSS1500	CNRIP1	2	68548060/68547655	2	cg08174244/cg21216118	0.70666885	0.54521699	0.394946445	-0.1614519	3.51E-06	7.68E-05
TSS1500	COQ3	6	99842368/99842351/99842353	3	cg03514381/cg18751133/cg24831427	0.59824407	0.44292576	0.349923117	-0.1553183	3.28E-06	7.37E-05
TSS1500	CP110	16	19533815/19534852	2	cg06784594/cg27259633	0.70235553	0.55044527	0.462975068	-0.1519103	1.28E-06	4.31E-05
TSS1500	DKFZP686I15217	6	2986848/2987708/2986843	3	cg00184279/cg01454349/cg12335476	0.66529225	0.50722289	0.410613164	-0.1580694	2.03E-08	8.01E-06
TSS1500	DTX1	12	113495386/113494422/113494429/113495037	4	cg04562491/cg10670310/cg11939071/cg22685245	0.28981724	0.44424425	0.548626283	0.15442701	3.81E-04	1.90E-03
TSS1500	DYDC1	10	82117089/82117119/82117257	3	cg03701427/cg06411551/cg17703212	0.34139758	0.54391812	0.676124495	0.20252054	3.45E-05	3.26E-04
TSS1500	EID3	12	104697193/104697220/104697224	3	cg01857475/cg09884423/cg10572274	0.24794737	0.42128987	0.607087922	0.1733425	9.39E-04	3.90E-03
TSS1500	EVI2B	17	29641333	1	cg05109049	0.36786521	0.14928822	0.121072471	-0.218577	1.33E-05	0.00017417
TSS1500	FAM38B	18	11149841/11149435/11149470/11149763/11149068/11150043	6	cg02664349/cg03117976/cg03686593/cg06510438/cg10567810/cg12673429	0.34106711	0.4923372	0.61288286	0.15127009	6.35E-04	2.84E-03
TSS1500	FAM55B	11	114548759/114548872	2	cg07340922/cg19113081	0.64500476	0.48287508	0.366004345	-0.1621297	5.60E-07	2.90E-05
TSS1500	FAM75A6	9	43631161	1	cg10034990	0.53034953	0.37100266	0.276844323	-0.1593469	1.17E-06	4.19E-05
TSS1500	FKBPL	6	32099450/32099564/32098344/32098346	4	cg03514937/cg07790778/cg08577517/cg16437896	0.69236295	0.52954162	0.446631627	-0.1628213	3.98E-06	8.24E-05
TSS1500	FLG2	1	152333922/152333423/152333596	3	cg03439811/cg03957898/cg23812489	0.48509233	0.33422547	0.227919445	-0.1508669	3.06E-04	1.60E-03
TSS1500	GABRR3	3	97754720	1	cg11726229	0.68051329	0.52752134	0.427380682	-0.1529919	3.30E-05	3.17E-04
TSS1500	GDE1	16	19533815/19534852	2	cg06784594/cg27259633	0.70235553	0.55044527	0.462975068	-0.1519103	1.28E-06	4.31E-05
TSS1500	GHITM	10	85898700/85898649	2	cg12706330/cg25783892	0.50817092	0.3533819	0.258773808	-0.154789	8.16E-06	1.25E-04
TSS1500	GIP	17	47046292	1	cg20551517	0.83768907	0.65942629	0.564808618	-0.1782628	1.01E-06	3.92E-05
TSS1500	HDGFL1	6	22568625/22568278	2	cg20209681/cg21898884	0.59318242	0.4328928	0.344404064	-0.1602896	7.16E-05	5.41E-04
TSS1500	HS3ST5	6	114385144/114384871/114384629	3	cg06694119/cg18192491/cg23968650	0.55737003	0.39628769	0.334053877	-0.1610823	1.42E-05	1.82E-04
TSS1500	IL17A	6	52050133/52050597	2	cg09582880/cg11924517	0.67080109	0.52038039	0.348856723	-0.1504207	4.22E-04	2.06E-03
TSS1500	KHDC1L	6	73935584/73935676	2	cg00732807/cg10190334	0.71055764	0.55352034	0.403592364	-0.1570373	7.65E-05	5.66E-04

TSS1500	KRTAP19-8	21	32412191	1	cg06790305	0.64860721	0.48350282	0.346723436	-0.1651044	4.91E-06	9.23E-05
TSS1500	KRTAP6-2	21	31972533/31972672	2	cg05572621/cg07945323	0.62244242	0.47091749	0.362859402	-0.1515249	2.54E-05	2.68E-04
TSS1500	LOC100133893	12	27925367	1	cg04279139	0.45117092	0.29900699	0.241501863	-0.1521639	2.80E-08	9.16E-06
TSS1500	LOC149134	1	246952508/246952362/246952118/246952495/246952215/246952048	6	cg03340119/cg03596016/cg05314024/cg08535928/cg15176829/cg26898024	0.44164432	0.59378095	0.640010458	0.15213663	9.17E-07	3.73E-05
TSS1500	LOC285627	5	158894390/158894440/158893488	3	cg07829289/cg16338178/cg19017803	0.75000719	0.52088019	0.426324782	-0.229127	3.04E-09	5.21E-06
TSS1500	LONP2	16	48277879/48277559	2	cg03354717/cg09539739	0.59426907	0.44363838	0.358753823	-0.1506307	8.20E-06	1.25E-04
TSS1500	LRRC4	7	127672235/127672473/127672458/127672169/127672152	5	cg04153784/cg09087503/cg12628196/cg15087147/cg27573591	0.08853242	0.26445421	0.408407066	0.17592179	6.50E-07	3.16E-05
TSS1500	LRRC55	11	56948018/56947754/56948015	3	cg03717588/cg11751645/cg24458609	0.23524251	0.40174317	0.461201923	0.16650066	8.17E-04	3.50E-03
TSS1500	MIR135B	1	205418278/205418410	2	cg02520707/cg13061767	0.72598226	0.55068704	0.453863557	-0.1752952	2.41E-07	1.96E-05
TSS1500	MRGPRD	11	68748907/68749124	2	cg11903239/cg20791323	0.62277544	0.44366822	0.358181995	-0.1791072	4.89E-07	2.72E-05
TSS1500	MUC13	3	124654885	1	cg14497879	0.62254475	0.46971605	0.371612186	-0.1528287	3.25E-07	2.24E-05
TSS1500	MYCT1	6	153018721	1	cg02830467	0.61936345	0.46878502	0.323156964	-0.1505784	1.26E-04	8.16E-04
TSS1500	NCRNA00157	21	19258310	1	cg26060835	0.50022788	0.34604623	0.238001077	-0.1541816	8.55E-06	1.28E-04
TSS1500	NIPSNAP1	22	29977949/29977677/29978158/29977742	4	cg00266865/cg00939432/cg08492625/cg13797031	0.69214889	0.53306889	0.442208673	-0.15908	4.32E-06	8.63E-05
TSS1500	NPBWR1	8	53852101/53851613/53852184/53852030/53851707/53851156	6	cg02115041/cg07629017/cg15531403/cg16330450/cg24857620/cg26205771	0.25370952	0.4133378	0.606035915	0.15962828	9.74E-04	4.02E-03
TSS1500	NPY	7	24323128/24323559/24323261	3	cg01050885/cg05158615/cg18106668	0.34957567	0.50974646	0.621323027	0.16017079	2.36E-05	2.55E-04
TSS1500	OC90	8	133072665/133072043/133072873/133072269	4	cg02759725/cg21594839/cg23399286/cg25626375	0.49354095	0.33971764	0.227361109	-0.1538233	2.04E-07	1.87E-05
TSS1500	OR56A1	11	6049729	1	cg18512262	0.57736578	0.41692918	0.255862718	-0.1604366	3.09E-05	3.03E-04
TSS1500	OR56A3	11	5967520	1	cg25451456	0.61033697	0.45474864	0.318152514	-0.1555883	2.21E-06	5.89E-05
TSS1500	OR5H15	3	97886467/97886603	2	cg01495831/cg03507018	0.5079947	0.33962278	0.22202145	-0.1683719	1.72E-06	5.14E-05
TSS1500	OR5T3	11	56018532	1	cg14284630	0.55136269	0.35941318	0.227832333	-0.1919495	3.20E-06	7.27E-05

TSS1500	OR8H1	11	56059643	1	cg22629065	0.40095448	0.23084966	0.166024259	-0.1701048	1.49E-06	4.76E-05
TSS1500	PI3	20	43802992/43803224	2	cg02733351/cg19931348	0.54707724	0.39662087	0.291040902	-0.1504564	6.53E-08	1.12E-05
TSS1500	PIGB	15	55610624	1	cg19991916	0.88449073	0.62599462	0.479371295	-0.2584961	1.45E-06	4.71E-05
TSS1500	PLIN1	15	90223301/90224098/90223712	3	cg01348757/cg08749443/cg11526413	0.67200997	0.51477628	0.43185497	-0.1572337	7.13E-09	5.71E-06
TSS1500	POLD3	11	74302893/74303294/74303012	3	cg01173485/cg08923614/cg26885816	0.67833687	0.51232671	0.399130685	-0.1660102	5.47E-07	2.88E-05
TSS1500	POM121L12	7	53102694/53102721	2	cg12333737/cg20170989	0.42947934	0.27930117	0.187995841	-0.1501782	9.58E-06	1.38E-04
TSS1500	PPP4R2	3	73045556/73045440/73045686	3	cg03847932/cg06051312/cg20540428	0.79815772	0.58513543	0.506348109	-0.2130223	1.14E-03	4.53E-03
TSS1500	PRB2	12	11548867/11549192/11549555	3	cg20480899/cg23372297/cg24891312	0.51697371	0.33488602	0.339073968	-0.1820877	1.20E-05	1.62E-04
TSS1500	PRSS1	7	142456124	1	cg12878228	0.61023168	0.44369722	0.330479082	-0.1665345	3.87E-06	8.08E-05
TSS1500	PUM2	2	20527635/20527677/20527833	3	cg01888166/cg08659707/cg22528172	0.82310104	0.66700006	0.617046847	-0.156101	5.60E-07	2.90E-05
TSS1500	RASAL1	12	113574276/113574368/113575128	3	cg08865522/cg16890739/cg24109790	0.55663769	0.3944711	0.306957808	-0.1621666	1.13E-08	6.92E-06
TSS1500	RFTN2	2	198541655/198541772	2	cg00826638/cg22387019	0.72952394	0.55098158	0.404589005	-0.1785424	2.14E-05	2.39E-04
TSS1500	SIRPG	20	1639325/1639816/1639250	3	cg11061975/cg13408605/cg23295754	0.70264101	0.50884555	0.339862545	-0.1937955	3.03E-06	7.09E-05
TSS1500	SLC17A2	6	25931205/25931659/25931557	3	cg01936555/cg20765441/cg24163360	0.6328762	0.46542842	0.373585605	-0.1674478	1.53E-07	1.55E-05
TSS1500	SLC22A24	11	62912823	1	cg04426607	0.67878832	0.51637331	0.407864932	-0.162415	7.58E-05	5.63E-04
TSS1500	SLC35B4	7	134003124/134002145/134002209	3	cg02659138/cg02820759/cg27581047	0.71033233	0.54311664	0.410342268	-0.1672157	4.93E-07	2.72E-05
TSS1500	SNORA80	21	33750733/33749986	2	cg09199848/cg09516696	0.79011717	0.63831289	0.608907745	-0.1518043	1.31E-06	4.35E-05
TSS1500	SNORD87	8	67835853/67835351	2	cg00640314/cg20217649	0.65718307	0.50143899	0.424463568	-0.1557441	4.93E-05	4.14E-04
TSS1500	SPAG11B	8	7311130	1	cg14600420	0.61582796	0.44269327	0.288175909	-0.1731347	9.15E-06	1.34E-04
TSS1500	STK38	6	36515676/36515856	2	cg02964385/cg23670203	0.5986102	0.44351593	0.373382268	-0.1550943	1.19E-07	1.42E-05
TSS1500	TCN1	11	59634276	1	cg03928812	0.50406189	0.33864056	0.238165301	-0.1654213	1.09E-04	7.35E-04
TSS1500	TMEM200A	6	130757762	1	cg26931050	0.74042799	0.58357244	0.493609582	-0.1568556	2.27E-05	2.50E-04
TSS1500	TSPAN12	7	120498729/120499546	2	cg22032706/cg23627038	0.61816603	0.46006451	0.394421307	-0.1581015	1.15E-06	4.19E-05

TSS1500	UNC5CL	6	41007485/41007692/41007450/41007522	4	cg15849648/cg17826424/cg19175193/cg22346765	0.72997075	0.56641271	0.438502102	-0.163558	7.48E-07	3.35E-05
TSS1500	YIPF7	4	44654206	1	cg03815116	0.65073397	0.49411667	0.3368489	-0.1566173	8.52E-05	6.11E-04
TSS1500	ZNF484	9	95640659	1	cg14646548	0.67635967	0.51935911	0.425553664	-0.1570006	2.67E-07	2.07E-05
TSS1500	ZNF543	19	57831632/57831544/57831549/57831662	4	cg00846300/cg14383655/cg14603375/cg19533530	0.09692006	0.26803743	0.374851989	0.17111737	1.73E-02	4.37E-02
TSS1500	ZNF667	19	56989102/56989543/56989812/56989740	4	cg03289872/cg08063125/cg21287936/cg24411929	0.41428735	0.56680469	0.724532957	0.15251734	8.89E-04	3.74E-03
TSS1500	ZP4	1	238055429/238054569	2	cg03673470/cg26810908	0.66332982	0.50223756	0.392643039	-0.1610923	1.03E-05	1.45E-04
TSS1500	ZPBP	7	50133212/50133131/50133100	3	cg00303745/cg09921682/cg10349880	0.84136324	0.669956	0.48186017	-0.1714072	9.92E-06	1.41E-04
TSS200	ACAN	15	89346629	1	cg16968596	0.20700132	0.40183949	0.631561205	0.19483817	6.70E-03	3.68E-02
TSS200	ACSS3	12	81471678/81471649/81471754/81471757	4	cg11659501/cg21754400/cg23614791/cg25358039	0.28644777	0.49718931	0.572053364	0.21074154	3.31E-06	1.54E-04
TSS200	APBB1IP	10	26727173/26727261/26727069/26727246/26727195	5	cg06157318/cg06535993/cg14022711/cg26241806/cg27303981	0.23712799	0.39955754	0.505817762	0.16242955	1.56E-05	3.85E-04
TSS200	BPIL3	20	31619420	1	cg25345102	0.68629173	0.52654817	0.469432586	-0.1597436	4.48E-06	1.83E-04
TSS200	C10orf90	10	128210076/128210036	2	cg09621603/cg10005270	0.60503916	0.43117819	0.292599823	-0.173861	8.73E-08	3.44E-05
TSS200	C12orf42	12	103889798/103889903/103889784/103889789/103889928/103889772	6	cg03882242/cg06664258/cg11342468/cg13702005/cg14380586/cg26381364	0.23822222	0.4112333	0.54528253	0.17301108	8.63E-05	1.28E-03
TSS200	C14orf48	14	94463488	1	cg16506185	0.69540914	0.52983259	0.4340211	-0.1655765	7.44E-06	2.41E-04
TSS200	C17orf73	17	48845064/48845052/48845009	3	cg07443932/cg12235260/cg25518968	0.72978819	0.56321029	0.471729965	-0.1665779	1.30E-05	3.46E-04
TSS200	C1orf14	1	182922705/182922593/182922582	3	cg02721693/cg02925590/cg22237670	0.43660276	0.28408398	0.213868618	-0.1525188	6.35E-07	6.75E-05
TSS200	C1orf180	1	85100819	1	cg00062020	0.72743088	0.51403294	0.472090786	-0.2133979	2.66E-07	5.19E-05
TSS200	C2orf74	2	61372066/61372117/61372138/61372165/61372058/61372226	6	cg01648237/cg05037927/cg16328106/cg19790321/cg23405039/cg24757310	0.38050331	0.56006012	0.670541623	0.17955681	1.18E-05	3.21E-04

TSS200	C9orf50	9	132383095	1	cg10640119	0.18027348	0.38649877	0.440187277	0.2062253	2.50E-07	5.15E-05
TSS200	CCDC42	17	8648180/8648193/8648175	3	cg15394558/cg19093680/cg27513572	0.70604267	0.55461281	0.448698632	-0.1514299	1.85E-06	1.14E-04
TSS200	CCDC70	13	52436047	1	cg11631011	0.71729355	0.56023082	0.398430955	-0.1570627	3.54E-05	0.000666547
TSS200	CDH19	18	64271341	1	cg16489926	0.75453834	0.57983383	0.40824685	-0.1747045	1.14E-06	8.76E-05
TSS200	CDO1	5	115152420/115152492/115152494/115152431/115152413/115152485	6	cg02792792/cg08516516/cg11036833/cg14470895/cg16707405/cg23180938	0.27291541	0.46358363	0.59145335	0.19066822	1.01E-04	1.43E-03
TSS200	CLIP4	2	29338258/29338109/29338121/29338113	4	cg03135351/cg13257636/cg23255835/cg23428985	0.35432474	0.52057464	0.639799002	0.1662499	2.69E-04	2.93E-03
TSS200	CMTM3	16	66638407/66638396/66638438/66638412/66638433/66638210	6	cg06445928/cg08601917/cg08682544/cg09233013/cg26560414/cg26658728	0.10943804	0.27126299	0.43647724	0.16182494	1.39E-03	1.06E-02
TSS200	DEFA6	8	6783670	1	cg08312330	0.76306387	0.60294842	0.492708895	-0.1601154	2.87E-06	1.42E-04
TSS200	DSPP	4	88529516/88529584	2	cg04503912/cg05292310	0.61235391	0.44671017	0.312553486	-0.1656437	1.10E-06	8.56E-05
TSS200	EDNRB	13	78493100/78493012/78493066/78493126/78493128	5	cg04390523/cg06179060/cg06971129/cg13818654/cg15836660	0.24517754	0.3956077	0.575186885	0.15043016	4.76E-04	0.004563924
TSS200	EID3	12	104697389/104697545/104697387/104697419/104697526/104697532/104697514	7	cg03817911/cg09477407/cg18633684/cg20923245/cg21234561/cg26614816/cg27205904	0.24590105	0.40008409	0.594826503	0.15418304	1.39E-03	1.06E-02
TSS200	FBLIM1	1	16085148	1	cg04897742	0.07020286	0.29292352	0.476116717	0.22272066	2.03E-06	1.20E-04
TSS200	FREM3	4	144621938/144621952/144621898/144621971/144621945	5	cg08334984/cg09556043/cg10056356/cg15042811/cg17495719	0.27677518	0.44499661	0.597750659	0.16822143	6.55E-05	0.001049985
TSS200	GABRA6	5	161112530	1	cg02703002	0.649197	0.48022479	0.373673241	-0.1689722	2.05E-07	4.65E-05
TSS200	GAD2	10	26505127/26505069/26505156	3	cg00733780/cg04838147/cg23050442	0.23479006	0.39387735	0.584842092	0.15908729	2.50E-03	1.70E-02
TSS200	GDEP	4	80748552	1	cg07124719	0.6897729	0.53145599	0.462399959	-0.1583169	7.86E-06	2.50E-04
TSS200	GPSM1	9	139247374	1	cg14040131	0.64215983	0.38156756	0.278549441	-0.2605923	2.34E-06	1.27E-04
TSS200	HSPB3	5	53751272/53751411	2	cg17839166/cg20169062	0.61586898	0.42549987	0.292604077	-0.1903691	3.97E-07	5.95E-05

TSS200	IL20RA	6	137366432/137366346/137366322/137366336/137366369/137366492	6	cg13714540/cg15992935/cg22487322/cg24405068/cg26864905/cg27656754	0.60633893	0.43779739	0.326750488	-0.1685415	1.36E-06	9.64E-05
TSS200	INHBA	7	41742795	1	cg18413237	0.77036114	0.61803652	0.489446836	-0.1523246	1.25E-05	3.35E-04
TSS200	KCNA3	1	111217691/111217712/111217715/111217785	4	cg06750832/cg07808555/cg09410234/cg26071016	0.25023847	0.4377103	0.609975493	0.18747183	9.26E-05	1.34E-03
TSS200	KCNQ5	6	73331405	1	cg00852573	0.236188	0.45694712	0.638643491	0.22075912	0.00018249	0.002173448
TSS200	KHDC1L	6	73935200/73935270	2	cg19565171/cg24543552	0.6973452	0.53602931	0.394430645	-0.1613159	5.27E-06	0.000198442
TSS200	KRT20	17	39041602	1	cg00091693	0.6548402	0.47848071	0.349085609	-0.1763595	3.80E-08	2.85E-05
TSS200	KRT6B	12	52845930/52846073/52845938/52846088	4	cg02068361/cg11601375/cg12795208/cg13221107	0.60088502	0.39943051	0.348907464	-0.2014545	2.87E-07	5.32E-05
TSS200	KRT71	12	52947004	1	cg05358758	0.60128914	0.41367666	0.312331736	-0.1876125	3.67E-06	1.59E-04
TSS200	KRTAP25-1	21	31661998	1	cg24145481	0.55644044	0.39766739	0.302504641	-0.158773	7.55E-07	7.07E-05
TSS200	KRTAP4-8	17	39254427/39254439	2	cg12981595/cg20663846	0.55585559	0.40332063	0.297085225	-0.152535	2.71E-06	1.39E-04
TSS200	L1TD1	1	62660421/62660281	2	cg14254748/cg27300647	0.60533681	0.44314349	0.360132848	-0.1621933	1.48E-06	9.96E-05
TSS200	LOC284688	1	170253405	1	cg25100722	0.75726553	0.58327737	0.524935673	-0.1739882	1.58E-06	0.00010235
TSS200	LOC285629	5	160365753	1	cg04390328	0.54416927	0.39034162	0.310438068	-0.1538276	9.26E-07	7.75E-05
TSS200	LONRF2	2	100939284	1	cg08017756	0.25777184	0.41601109	0.529280941	0.15823925	2.41E-05	5.23E-04
TSS200	MIR1204	8	128808063/128808017	2	cg11201447/cg25247520	0.43689859	0.2627119	0.179305958	-0.1741867	1.90E-06	0.000116526
TSS200	MIR1910	16	85775359	1	cg25575961	0.65931495	0.50370954	0.3686335	-0.1556054	6.02E-06	2.12E-04
TSS200	MIR338	17	79099785/79099882/79099777/79099819/79099942	5	cg06807993/cg06869212/cg18637486/cg21473782/cg23295826	0.77246894	0.59259095	0.577218555	-0.179878	1.65E-09	2.43E-05
TSS200	MIR563	3	15915216	1	cg23023604	0.66340685	0.50863573	0.382680332	-0.1547711	4.16E-07	5.95E-05
TSS200	MMP13	11	102826570/102826565/102826469	3	cg14995062/cg19620758/cg22658979	0.75263497	0.55762536	0.396195655	-0.1950096	7.00E-07	6.89E-05
TSS200	MMP26	11	5009311	1	cg12493906	0.7180576	0.54207433	0.422492664	-0.1759833	1.24E-06	9.26E-05
TSS200	NETO1	18	70533078/70535005	2	cg13861749/cg18651870	0.6870667	0.5101433	0.36370005	-0.1769234	1.54E-06	1.01E-04
TSS200	NPY	7	24323767/24323792/24323799/24323675/24323764	5	cg00355281/cg15929698/cg16964348/cg24242823/cg24885417	0.22485096	0.45930533	0.603738122	0.23445437	3.18E-05	6.22E-04

TSS200	NTRK3	15	88799820/88799707	2	cg13773705/cg21956337	0.24426909	0.41277991	0.575837173	0.16851082	1.62E-03	1.20E-02
TSS200	NUAK1	12	106533903/106533840/106533867/106533898/106533863	5	cg02518216/cg17393917/cg18525352/cg20060685/cg23555120	0.49637478	0.6539193	0.726852429	0.15754452	2.65E-07	5.19E-05
TSS200	OR2M3	1	248366332	1	cg06393354	0.65267757	0.49860187	0.411762314	-0.1540757	9.89E-06	2.88E-04
TSS200	OR5A1	11	59210634	1	cg10919344	0.51877846	0.36691158	0.2463723	-0.1518669	6.66E-06	0.000227821
TSS200	OR8J3	11	55905246	1	cg11323650	0.5785646	0.40816134	0.2954314	-0.1704033	1.23E-06	9.26E-05
TSS200	PCDHGA11	5	140800495/140800474/140800424/140800398	4	cg03892308/cg17857974/cg18118262/cg26647197	0.42006242	0.58893817	0.720942086	0.16887575	8.41E-05	1.26E-03
TSS200	PCDHGB6	5	140787623	1	cg09465698	0.5019589	0.65708599	0.708289209	0.15512709	1.81E-05	4.30E-04
TSS200	PCDHGC4	5	140864578/140864593/140864701/140864549/140864733	5	cg02022733/cg03640756/cg15361590/cg15949044/cg23445461	0.29612909	0.45979874	0.667165305	0.16366966	0.00026812	0.002923945
TSS200	PRND	20	4702381/4702538/4702464/4702516	4	cg04636876/cg05837890/cg12587930/cg24427947	0.74509379	0.58162754	0.455482394	-0.1634662	6.76E-06	2.30E-04
TSS200	PRSS1	7	142457299/142457284	2	cg02329916/cg14795939	0.65231582	0.46982979	0.349246105	-0.182486	1.42E-06	9.80E-05
TSS200	PTPLAD2	9	21031822	1	cg14498666	0.38264566	0.53543813	0.649741386	0.15279248	8.34E-05	1.25E-03
TSS200	RLBP1	15	89765061/89765045/89764999	3	cg00376910/cg08746496/cg20989926	0.62221355	0.45316784	0.37248542	-0.1690457	1.08E-08	2.43E-05
TSS200	S100P	4	6695475/6695380/6695377/6695464	4	cg07210669/cg14140379/cg14900031/cg25083732	0.66811744	0.4932814	0.366300711	-0.174836	1.09E-05	3.03E-04
TSS200	SERPINB12	18	61223377	1	cg00005215	0.68915803	0.49351439	0.371855277	-0.1956436	7.04E-08	3.25E-05
TSS200	SERPINB5	18	61143961/61143945/61143993	3	cg11862144/cg19022006/cg26753302	0.58269762	0.42124933	0.317292064	-0.1614483	3.14E-05	6.19E-04
TSS200	SIRPD	20	1538366/1538353	2	cg01247127/cg11295724	0.67158648	0.49256962	0.370846718	-0.1790169	1.56E-05	3.85E-04
TSS200	SLC16A4	1	110933767	1	cg00961640	0.6624791	0.48953676	0.375002668	-0.1729423	1.03E-06	8.31E-05
TSS200	SNORA80B	2	10587052	1	cg15075241	0.66628874	0.49080281	0.352196073	-0.1754859	9.88E-06	0.000288389
TSS200	SORCS3	10	106400686/106400824/106400702/106400667	4	cg03958798/cg08495770/cg10601616/cg20482521	0.3097744	0.48295773	0.657551995	0.17318333	5.11E-04	4.82E-03
TSS200	SPERT	13	46276342	1	cg05014103	0.54399399	0.39381889	0.3123524	-0.1501751	7.01E-06	2.35E-04

TSS200	SUSD5	3	33260743/33260732/33260752	3	cg02239862/cg04545136/cg13601427	0.23278465	0.38715645	0.541407395	0.1543718	4.97E-04	4.72E-03
TSS200	TCN1	11	59634218/59634191	2	cg00187686/cg23741006	0.47672392	0.32445191	0.230647861	-0.152272	2.41E-05	5.23E-04
TSS200	TM6SF1	15	83776271/83776269	2	cg08452658/cg11167100	0.15124129	0.37686004	0.565246468	0.22561874	5.83E-05	9.60E-04
TSS200	TMEM155	4	122686432/122686453/122686493/122686456	4	cg03227184/cg04638468/cg07978472/cg08553437	0.29977716	0.45178774	0.640223931	0.15201058	1.54E-03	1.15E-02
TSS200	TMPRSS11B	4	69111580	1	cg19510180	0.71475154	0.54210318	0.4340898	-0.1726484	1.57E-06	1.02E-04
TSS200	TMPRSS5	11	113577071/113577118/113577120	3	cg15844374/cg18165914/cg25460340	0.78128987	0.61498127	0.563340545	-0.1663086	1.79E-07	4.47E-05
TSS200	TTYH1	19	54926514/54926617/54926437	3	cg15241920/cg15723536/cg21883754	0.1925932	0.35211354	0.553464397	0.15952034	7.57E-03	4.08E-02
TSS200	VPREB1	22	22599128/22599189	2	cg03763796/cg25330366	0.37156307	0.21550552	0.142456698	-0.1560575	2.33E-05	5.12E-04
TSS200	ZIK1	19	58095518/58095588/58095468/58095581/58095445/58095595	6	cg00800512/cg01046104/cg04342092/cg12060744/cg18435449/cg18579862	0.37098189	0.54218254	0.688697526	0.17120064	1.01E-04	1.43E-03
TSS200	ZNF217	20	52199778/52199748/52199729	3	cg09228833/cg20979153/cg22164891	0.2894478	0.13280495	0.167094655	-0.1566429	5.46E-07	6.53E-05
TSS200	ZNF304	19	57862484/57862612/57862554/57862627/57862480/57862638	6	cg00397851/cg13788592/cg14015503/cg16848116/cg17849956/cg21627760	0.09296582	0.24668245	0.401504245	0.15371663	5.19E-04	4.87E-03
TSS200	ZNF334	20	45142244/45142258/45142214/45142206/45142336	5	cg07139762/cg10140114/cg25828181/cg26238800/cg27385032	0.22210235	0.38413437	0.487326399	0.16203201	2.28E-04	2.58E-03
TSS200	ZNF549	19	58038689/58038573/58038585/58038633/58038588/58038621	6	cg01601746/cg06458239/cg10729426/cg16697731/cg19060970/cg20607331	0.28512836	0.45651963	0.650572955	0.17139127	0.00167747	0.012303265
TSS200	ZNF625	19	12267676/12267685/12267592	3	cg02914087/cg06429617/cg17287725	0.10140589	0.26155096	0.374411985	0.16014507	6.65E-04	5.89E-03
TSS200	ZNF626	19	20844422	1	cg01808171	0.27876178	0.44659247	0.591171732	0.16783069	1.24E-04	1.68E-03
UTR5	ACSS3	12	81471882/81471867/81471884	3	cg10789281/cg13273396/cg26272220	0.33108129	0.53160677	0.618354419	0.20052548	2.02E-05	0.000357762
UTR5	ADAM28	8	24151625	1	cg22915945	0.31534811	0.1558054	0.11973464	-0.1595427	1.49E-05	0.000291675
UTR5	ADHFE1	8	67344720	1	cg20912169	0.25645994	0.41119362	0.660422359	0.15473369	0.00442623	0.020682407

UTR5	AKNA	9	117150495	1	cg14080475	0.2317446	0.39307568	0.524686191	0.16133108	4.50E-06	0.000132524
UTR5	ALG1L	3	125655381/125655407	2	cg05084668/cg07473553	0.68058229	0.46332182	0.311121282	-0.2172605	2.23E-06	8.53E-05
UTR5	BEND5	1	49242513/49242519	2	cg11666087/cg16573178	0.06582538	0.24613148	0.318706693	0.1803061	0.00489386	0.022395124
UTR5	BHLHE23	20	61638149	1	cg21161253	0.10811316	0.26170771	0.349949555	0.15359455	0.00160085	0.009303669
UTR5	C10orf90	10	128209980	1	cg24143287	0.67340542	0.46553872	0.322228568	-0.2078667	4.43E-06	0.000131605
UTR5	C1orf70	1	1475737	1	cg16601494	0.18564747	0.36169337	0.665361532	0.1760459	0.00913281	0.03718464
UTR5	C9orf50	9	132383003	1	cg09731694	0.31731967	0.54986167	0.697157327	0.232542	1.07E-06	5.63E-05
UTR5	CAPN14	2	31440349/31439687	2	cg04132353/cg15941014	0.50051138	0.31460821	0.241193082	-0.1859032	5.60E-06	0.000149667
UTR5	CD38	4	15779999/15780011	2	cg02183671/cg24819835	0.21345019	0.43939278	0.552727605	0.22594259	6.61E-06	0.000167928
UTR5	CDO1	5	115152386/115152326	2	cg12880658/cg16265906	0.25933619	0.42570901	0.578118532	0.16637282	0.00021965	0.001979774
UTR5	CLLU1	12	92818240	1	cg13003571	0.85730143	0.69463078	0.60484675	-0.1626706	6.33E-05	0.000770386
UTR5	CMTM3	16	66638320/66638599/66638702/66638407/66638396/66638438/66638412/66638293/66638433/66638210	10	cg00355909/cg03860859/cg06314872/cg06445928/cg08601917/cg08682544/cg09233013/cg23297477/cg26560414/cg26658728	0.11073596	0.28202328	0.429371915	0.17128733	0.00033152	0.002747377
UTR5	CNRIP1	2	68546899/68546688/68547088/68547141/68547052/68546579	6	cg00020052/cg04020079/cg07594636/cg08157672/cg15313459/cg24171907	0.22884967	0.44246169	0.576738373	0.21361202	4.86E-05	0.000648239
UTR5	CPSF4L	17	71257980	1	cg18772573	0.8359671	0.67650444	0.703358664	-0.1594627	3.68E-05	0.000534648
UTR5	CYP2C18	10	96443383	1	cg14196507	0.65630379	0.49214716	0.376205891	-0.1641566	1.99E-07	2.07E-05
UTR5	DKK2	4	107957265/107957362/107956955/107957430/107957072	5	cg01471384/cg01962428/cg08341316/cg13139972/cg26955835	0.18229562	0.34234373	0.467727194	0.16004812	4.22E-05	0.000584476
UTR5	DSPP	4	88530371	1	cg09590733	0.63317119	0.47564467	0.391535282	-0.1575265	9.50E-08	1.50E-05
UTR5	EHD3	2	31457287/31457061/31456966/31457058/31456964/31457043	6	cg01163837/cg13795465/cg15355118/cg24743639/cg25428398/cg25840208	0.11376018	0.29621726	0.397088917	0.18245708	0.0005367	0.003924496
UTR5	EMB	5	49737178/49737182/49736988	3	cg03179043/cg08722065/cg18110165	0.23066055	0.44751176	0.520510282	0.21685121	6.39E-07	4.20E-05
UTR5	FOLH1B	11	89392829	1	cg02915544	0.59441058	0.41269511	0.265445155	-0.1817155	6.85E-06	0.000173394

UTR5	GAD2	10	26505442/26505503/26505440/26505245	4	cg04599297/cg09056181/cg11328303/cg15850954	0.27707442	0.44180043	0.613517622	0.16472601	0.00081042	0.005440494
UTR5	GIF	11	59612937	1	cg01282204	0.75930133	0.53320967	0.396963041	-0.2260917	1.62E-08	9.11E-06
UTR5	GYPC	2	127413831	1	cg15975865	0.30371499	0.48904628	0.648228845	0.18533129	0.0025577	0.013399792
UTR5	HSPB3	5	53751493	1	cg01246617	0.69732705	0.49313077	0.357341864	-0.2041963	5.98E-08	1.20E-05
UTR5	IRF4	6	391936/392555/393110/392131/391764	5	cg06392169/cg12612118/cg12684209/cg12741420/cg17228900	0.23761797	0.4350776	0.610867689	0.19745963	0.00013128	0.001341603
UTR5	ITGA4	2	182322268/182322058/182321855/182322279/182321786	5	cg06952671/cg10526659/cg20415809/cg21995919/cg25652029	0.26461093	0.46416069	0.63316878	0.19954977	0.00029217	0.002480946
UTR5	KCNA3	1	111217575/111217497	2	cg01423964/cg11595545	0.22540021	0.44703181	0.578786818	0.2216316	5.41E-05	0.000690954
UTR5	KIAA0802	18	8717926/8717905	2	cg07955887/cg19522644	0.68367303	0.47154349	0.395284905	-0.2121295	6.76E-10	2.85E-06
UTR5	KRT7	12	52627047	1	cg09670128	0.37070131	0.53105941	0.734100745	0.1603581	0.00504659	0.023017625
UTR5	KRTAP24-1	21	31655261	1	cg07172334	0.56064531	0.33288377	0.239309864	-0.2277615	7.66E-08	1.38E-05
UTR5	LCE3D	1	152552494	1	cg20676475	0.71794003	0.55167926	0.380826986	-0.1662608	1.31E-05	0.000268016
UTR5	LFNG	7	2557715	1	cg20572537	0.53902541	0.37118509	0.294986845	-0.1678403	4.69E-07	3.43E-05
UTR5	LONRF2	2	100939045/100938903/100938813/100938799	4	cg07304692/cg14675211/cg14997226/cg23977631	0.08408882	0.25808533	0.391470726	0.17399651	4.67E-05	0.000627757
UTR5	MAL	2	95691500	1	cg22403344	0.13239692	0.29368619	0.438046159	0.16128928	0.0008229	0.005503434
UTR5	MMP12	11	102745682	1	cg20487452	0.61555095	0.45573393	0.3300052	-0.159817	8.32E-06	0.000195493
UTR5	NPY	7	24324570/24324435/24323939/24323840	4	cg11475550/cg12614105/cg21097881/cg25884711	0.28880786	0.44413203	0.564067368	0.15532416	0.00019345	0.001796017
UTR5	PCDHGA11	5	140800586/140800629/140800761	3	cg05870739/cg10679597/cg24954895	0.41512593	0.57235891	0.715389425	0.15723298	7.43E-05	0.000861528
UTR5	RIC3	11	8190565/8190572	2	cg08383315/cg25778535	0.30006801	0.45225332	0.576551684	0.15218531	0.0001837	0.00173835
UTR5	RLBP1	15	89764836/89764895	2	cg10173075/cg15808008	0.64004254	0.45511398	0.3678254	-0.1849286	1.55E-09	3.18E-06
UTR5	SCGB2A2	11	62037659	1	cg22862656	0.81272038	0.60955429	0.447701014	-0.2031661	1.69E-06	7.18E-05
UTR5	SLC16A4	1	110933248/110933345	2	cg09494546/cg25320780	0.69638202	0.54066308	0.445331232	-0.1557189	2.07E-07	2.07E-05
UTR5	SLC36A2	5	150727045	1	cg19562321	0.47641976	0.32174868	0.196324459	-0.1546711	8.87E-06	0.000204848
UTR5	SORCS3	10	106400880	1	cg16787600	0.34106859	0.5001111	0.680446518	0.15904252	0.00149142	0.008798153

UTR5	SPAG4L	20	31592147	1	cg02510802	0.74023952	0.57363131	0.371162491	-0.1666082	3.14E-05	0.000483694
UTR5	TFPI2	7	93520036/93520024/93520012	3	cg14775114/cg17338208/cg24531255	0.13930434	0.32959887	0.545375324	0.19029454	0.00051541	0.003801517
UTR5	TM6SF1	15	83776422/83776420	2	cg03063639/cg26460092	0.28852964	0.47710419	0.674643993	0.18857455	0.00055142	0.004003174
UTR5	TMEM71	8	133772657/133772889/133772742	3	cg03141298/cg20955688/cg27159719	0.3211927	0.1693228	0.169164931	-0.1518699	2.05E-07	2.07E-05
UTR5	TTN	2	179672121/179672126	2	cg05185019/cg10859358	0.77635657	0.58309665	0.495723477	-0.1932599	2.49E-06	9.24E-05
UTR5	UMOD	16	20363009/20363711	2	cg04231019/cg07456201	0.65645896	0.48850246	0.322445711	-0.1679565	1.90E-05	0.000345858
UTR5	ZFP82	19	36909326/36906819/36909413/36909418	4	cg07900968/cg19750174/cg24724633/cg25886284	0.34075043	0.49869339	0.634785768	0.15794296	0.00130542	0.007907773
UTR5	ZIK1	19	58095659	1	cg26246807	0.37561466	0.59770664	0.758164927	0.22209199	0.00014617	0.001454171
UTR5	ZNF304	19	57862713	1	cg07494047	0.08492214	0.23621898	0.429114705	0.15129684	0.00021295	0.001935385
UTR5	ZNF625	19	12267308/12267464/12266489	3	cg00875960/cg17892556/cg22606224	0.28509169	0.48990408	0.591177046	0.20481239	1.18E-05	0.000246009
EXON1	ABCC11	16	48265760	1	cg09228051	0.52717301	0.33331525	0.231701973	-0.1938578	1.49E-06	1.46E-04
EXON1	ACSS3	12	81472177/81471938/81471882/81471867/81472118/81471884	6	cg01283289/cg05143887/cg10789281/cg13273396/cg24711649/cg26272220	0.33318431	0.50958241	0.580906063	0.1763981	1.12E-05	4.41E-04
EXON1	ADAM28	8	24151625	1	cg22915945	0.31534811	0.1558054	0.11973464	-0.1595427	1.49E-05	5.19E-04
EXON1	ADHFE1	8	67344720	1	cg20912169	0.25645994	0.41119362	0.660422359	0.15473369	4.43E-03	2.88E-02
EXON1	ANKS4B	16	21245151	1	cg26937434	0.56735228	0.40597067	0.279589323	-0.1613816	2.62E-05	7.45E-04
EXON1	APBB1IP	10	26727318	1	cg05475934	0.3810481	0.5482303	0.644688227	0.16718221	7.34E-06	0.000346893
EXON1	BEND5	1	49242359/49242513/49242519	3	cg06744574/cg11666087/cg16573178	0.07530915	0.25973101	0.33691097	0.18442186	3.05E-03	2.18E-02
EXON1	BEST2	19	12863557	1	cg19743791	0.65604893	0.50185895	0.384092309	-0.15419	7.37E-06	3.47E-04
EXON1	C10orf90	10	128209980	1	cg24143287	0.67340542	0.46553872	0.322228568	-0.2078667	4.43E-06	2.62E-04
EXON1	C1orf70	1	1475675/1475737	2	cg16306898/cg16601494	0.13471528	0.32452273	0.606946738	0.18980745	2.76E-03	2.02E-02
EXON1	C2orf74	2	61372316/61372256	2	cg10580144/cg18158151	0.31029721	0.50879332	0.667898914	0.1984961	1.70E-05	5.68E-04
EXON1	C9orf50	9	132383003/132382812/132382433	3	cg09731694/cg13405887/cg14015706	0.23142934	0.52166887	0.667683026	0.29023953	8.88E-08	4.86E-05
EXON1	CD38	4	15779999/15780011/15780238	3	cg02183671/cg24819835/cg26043257	0.21345019	0.43939278	0.552727605	0.22594259	6.61E-06	3.34E-04

EXON1	CDO1	5	115152019/115152386/115152326	3	cg07405021/cg12880658/cg16265906	0.24518058	0.39715734	0.52278282	0.15197677	0.00031505	0.004066793
EXON1	CLIP4	2	29338432	1	cg08808128	0.15299871	0.32100477	0.562527791	0.16800606	0.00223993	0.017277059
EXON1	CLLU1	12	92818240	1	cg13003571	0.85730143	0.69463078	0.60484675	-0.1626706	6.33E-05	1.33E-03
EXON1	CMTM3	16	66638320/66638599/66638702/66638407/66638396/66638438/66638412/66638293/66638433/66638210	10	cg00355909/cg03860859/cg06314872/cg06445928/cg08601917/cg08682544/cg09233013/cg23297477/cg26560414/cg26658728	0.11073596	0.28202328	0.429371915	0.17128733	3.32E-04	4.22E-03
EXON1	CNRIP1	2	68546899/68546688/68546507/68547088/68547141/68546467/68547052/68546579	8	cg00020052/cg04020079/cg07080358/cg07594636/cg08157672/cg11573679/cg15313459/cg24171907	0.21755924	0.44587073	0.58209091	0.22831149	3.46E-05	8.84E-04
EXON1	CPSF4L	17	71257980	1	cg18772573	0.8359671	0.67650444	0.703358664	-0.1594627	3.68E-05	9.34E-04
EXON1	CYP2C18	10	96443383	1	cg14196507	0.65630379	0.49214716	0.376205891	-0.1641566	1.99E-07	5.79E-05
EXON1	DEFB135	8	11839844	1	cg22743884	0.6567988	0.48981156	0.313000655	-0.1669872	6.59E-05	1.36E-03
EXON1	DKK2	4	107956556/107957265/107957362/107956955/107957430/107957072	6	cg00594011/cg01471384/cg01962428/cg08341316/cg13139972/cg26955835	0.20238016	0.36249417	0.489249718	0.16011401	4.50E-05	1.07E-03
EXON1	DMRT1	9	841850	1	cg03168582	0.09390504	0.2643655	0.371218771	0.17046046	7.11E-03	4.15E-02
EXON1	EFCAB3	17	60447682	1	cg17876595	0.77645862	0.60523542	0.423534982	-0.1712232	2.15E-05	6.54E-04
EXON1	EMB	5	49737178/49737182/49736988	3	cg03179043/cg08722065/cg18110165	0.23066055	0.44751176	0.520510282	0.21685121	6.39E-07	9.98E-05
EXON1	FHL5	6	97010439	1	cg23978322	0.78676366	0.62782916	0.527864168	-0.1589345	3.64E-06	2.38E-04
EXON1	FOLH1B	11	89392829	1	cg02915544	0.59441058	0.41269511	0.265445155	-0.1817155	6.85E-06	3.39E-04
EXON1	GAD2	10	26505442/26505503/26505440/26505245	4	cg04599297/cg09056181/cg11328303/cg15850954	0.27707442	0.44180043	0.613517622	0.16472601	8.10E-04	7.97E-03
EXON1	GATA4	8	11561724/11561728	2	cg14666113/cg18283386	0.20684506	0.37116059	0.619421941	0.16431554	0.00284085	0.020584483
EXON1	GIF	11	59612937	1	cg01282204	0.75930133	0.53320967	0.396963041	-0.2260917	1.62E-08	2.58E-05
EXON1	GPR128	3	100328745/100328713	2	cg04071964/cg23855093	0.52189019	0.36477268	0.299061832	-0.1571175	3.37E-08	4.05E-05
EXON1	GPT2	16	46919112/46919194	2	cg03533472/cg23684449	0.68016188	0.50773397	0.4307959	-0.1724279	4.64E-06	2.68E-04
EXON1	GYPC	2	127413831	1	cg15975865	0.30371499	0.48904628	0.648228845	0.18533129	2.56E-03	1.93E-02

EXON1	IRF4	6	391764	1	cg17228900	0.10086644	0.31432297	0.585962585	0.21345653	1.45E-03	1.24E-02
EXON1	ITGA4	2	182322268/182322058/182321855/182322279/182322501/182321786	6	cg06952671/cg10526659/cg20415809/cg21995919/cg25024074/cg25652029	0.26076316	0.45898675	0.611804352	0.19822359	1.34E-04	2.22E-03
EXON1	ITIH2	10	7745454	1	cg06224510	0.52290188	0.36192469	0.230658309	-0.1609772	4.35E-06	2.61E-04
EXON1	KPNA7	7	98805084	1	cg22498453	0.76428644	0.59490261	0.590208441	-0.1693838	8.77E-09	2.32E-05
EXON1	KRT20	17	39041266/39041199/39041119/39041110	4	cg19114576/cg19449377/cg23901700/cg25124433	0.78386269	0.5532605	0.399192683	-0.2306022	2.52E-07	6.67E-05
EXON1	KRTAP15-1	21	31813075	1	cg16812893	0.59362799	0.44057094	0.334187759	-0.153057	3.64E-06	2.38E-04
EXON1	KRTAP7-1	21	32202010	1	cg05533037	0.61733834	0.43981978	0.331629845	-0.1775186	5.50E-05	1.22E-03
EXON1	LONRF2	2	100937944/100939045/100938444/100938903/100938813/100938799	6	cg03559235/cg07304692/cg14348439/cg14675211/cg14997226/cg23977631	0.08408882	0.25808533	0.391470726	0.17399651	4.67E-05	0.001094305
EXON1	LRRIQ4	3	169539775/169540297/169540504/169540079	4	cg04748593/cg13793580/cg18155853/cg23081534	0.81034238	0.57668054	0.389583758	-0.2336618	3.58E-07	7.77E-05
EXON1	MAL	2	95691500	1	cg22403344	0.13239692	0.29368619	0.438046159	0.16128928	0.0008229	0.008069767
EXON1	MMP12	11	102745682	1	cg20487452	0.61555095	0.45573393	0.3300052	-0.159817	8.32E-06	3.71E-04
EXON1	MSC	8	72756155/72756341/72756656/72756058	4	cg09734791/cg14409559/cg23710218/cg25832771	0.20825075	0.38081241	0.516248264	0.17256166	0.00117507	0.010567666
EXON1	MST1R	3	49941151/49940853/49940126/49940919/49940245/49940353	6	cg03332271/cg08687163/cg11001085/cg11839681/cg18706476/cg23437479	0.73540785	0.54741605	0.432983918	-0.1879918	3.19E-07	7.65E-05
EXON1	OLFM4	13	53603157	1	cg24932628	0.6151768	0.43447863	0.355098023	-0.1806982	7.79E-07	1.03E-04
EXON1	OR10T2	1	158369112/158368889	2	cg15593510/cg15852352	0.67808873	0.50158904	0.350021123	-0.1764997	5.51E-07	9.47E-05
EXON1	OR2AK2	1	248129192	1	cg11977100	0.59376515	0.44018931	0.286650286	-0.1535758	8.58E-06	0.000374771
EXON1	OR9Q1	11	57791564	1	cg03034198	0.75049994	0.57641685	0.519473877	-0.1740831	4.01E-06	2.48E-04
EXON1	PREX1	20	47444241	1	cg03361585	0.10036197	0.26961979	0.393830719	0.16925783	4.06E-04	4.82E-03
EXON1	PRND	20	4702581	1	cg09906458	0.66278959	0.50445671	0.349004655	-0.1583329	7.34E-06	3.47E-04
EXON1	PRR4	12	11324142/11002071/11324180	3	cg21216543/cg23272369/cg26983578	0.81355756	0.65203154	0.578963895	-0.161526	5.92E-06	0.000308392
EXON1	PYGO1	15	55881044	1	cg25902187	0.17366485	0.35751739	0.522236799	0.18385254	4.13E-03	2.73E-02
EXON1	RAMP3	7	45197441	1	cg14999001	0.24034283	0.41848183	0.540330959	0.178139	5.57E-04	6.02E-03

EXON1	RLBP1	15	89764836/89764895	2	cg10173075/cg15808008	0.64004254	0.45511398	0.3678254	-0.1849286	1.55E-09	7.41E-06
EXON1	SARM1	17	26699187/26699169/26699485	3	cg05854644/cg15760257/cg20686234	0.13072045	0.29554842	0.409477434	0.16482797	5.10E-04	5.68E-03
EXON1	SCGB2A2	11	62037659	1	cg22862656	0.81272038	0.60955429	0.447701014	-0.2031661	1.69E-06	1.50E-04
EXON1	SERPINA1	14	94855034/94856984/94855088/94855099	4	cg02126235/cg02181506/cg05346611/cg22828990	0.72827076	0.52588463	0.3817073	-0.2023861	7.48E-07	1.03E-04
EXON1	SFMBT2	10	7453329	1	cg20506184	0.15607953	0.40563856	0.595730313	0.24955903	5.88E-05	1.25E-03
EXON1	SLC36A2	5	150727045	1	cg19562321	0.47641976	0.32174868	0.196324459	-0.1546711	8.87E-06	3.82E-04
EXON1	SLC3A1	2	44502740/44502977	2	cg02192965/cg03217954	0.59685885	0.42119046	0.334986914	-0.1756684	1.42E-06	1.43E-04
EXON1	SLC9A9	3	143567031	1	cg23538718	0.16155301	0.33876684	0.377645814	0.17721383	1.76E-04	2.68E-03
EXON1	SPAG4L	20	31592147	1	cg02510802	0.74023952	0.57363131	0.371162491	-0.1666082	3.14E-05	8.32E-04
EXON1	TAS2R9	12	10962437	1	cg20090497	0.68535001	0.52584179	0.424362132	-0.1595082	1.61E-05	5.47E-04
EXON1	TFPI2	7	93520036/93520024/93519924/93520012	4	cg14775114/cg17338208/cg23686014/cg24531255	0.14245076	0.32175358	0.519702184	0.17930281	4.35E-04	5.07E-03
EXON1	TM6SF1	15	83776422/83776446/83776420	3	cg03063639/cg14696396/cg26460092	0.28852964	0.47710419	0.674643993	0.18857455	5.51E-04	0.005979301
EXON1	TMEM71	8	133772889/133772742	2	cg20955688/cg27159719	0.3211927	0.1693228	0.169164931	-0.1518699	2.05E-07	5.79E-05
EXON1	TP63	3	189349278/189349323	2	cg04483101/cg21723486	0.73135097	0.58075488	0.449463195	-0.1505961	3.98E-05	9.85E-04
EXON1	TTN	2	179672121/179672126	2	cg05185019/cg10859358	0.77635657	0.58309665	0.495723477	-0.1932599	2.49E-06	1.96E-04
EXON1	USP54	10	75335352	1	cg12303623	0.56002716	0.40020419	0.346340677	-0.159823	9.31E-08	4.86E-05
EXON1	ZFP82	19	36909413/36909418	2	cg24724633/cg25886284	0.1621262	0.37458051	0.543410133	0.21245431	0.00078024	0.007743324
EXON1	ZIK1	19	58095659	1	cg26246807	0.37561466	0.59770664	0.758164927	0.22209199	1.46E-04	2.36E-03
EXON1	ZNF304	19	57863057/57862713	2	cg04685743/cg07494047	0.08492214	0.23621898	0.429114705	0.15129684	2.13E-04	3.03E-03
EXON1	ZNF625	19	12267464	1	cg17892556	0.18447788	0.42516717	0.539363618	0.24068929	1.30E-04	2.17E-03
EXON1	ZNF793	19	37997867	1	cg15139588	0.09884138	0.25572428	0.475644413	0.15688291	2.23E-03	1.73E-02
GENEBODY	ABCD2	12	39981136	1	cg13406085	0.71778078	0.56527744	0.388307936	-0.1525033	2.27E-05	2.25E-04
GENEBODY	APOL5	22	36123779	1	cg25036527	0.61314906	0.45581511	0.348639441	-0.157334	1.32E-05	1.55E-04
GENEBODY	ASCC2	22	30195299	1	cg14742445	0.62358413	0.42714246	0.297950882	-0.1964417	4.89E-06	8.40E-05
GENEBODY	ASCL3	11	8959438	1	cg14711859	0.80166068	0.6463918	0.528616877	-0.1552689	5.33E-06	8.84E-05
GENEBODY	ATP6V1G3	1	198509669	1	cg12958813	0.8201387	0.62559844	0.495645964	-0.1945403	6.53E-05	4.78E-04
GENEBODY	BPIL3	20	31619647	1	cg18223379	0.69019139	0.52916155	0.452184077	-0.1610298	3.25E-05	2.87E-04
GENEBODY	C18orf26	18	52262555	1	cg01174441	0.704882	0.53391837	0.387608936	-0.1709636	1.26E-06	3.83E-05

GENEBODY	C1orf180	1	85100313/85098598	2	cg11387751/cg25388882	0.62865284	0.47617227	0.39536335	-0.1524806	5.78E-07	2.49E-05
GENEBODY	C1QTNF4	11	47612070/47611780	2	cg05537653/cg18356785	0.25802581	0.41192562	0.491033436	0.15389981	3.94E-05	3.30E-04
GENEBODY	C6	5	41160373/41152767	2	cg02598319/cg16582255	0.66406226	0.50852991	0.424378073	-0.1555323	4.34E-08	7.64E-06
GENEBODY	C9orf70	9	3899055	1	cg14269813	0.51581376	0.29938383	0.253730073	-0.2164299	9.84E-07	3.29E-05
GENEBODY	CD1B	1	158301038/158300733	2	cg04574507/cg15952487	0.51078378	0.35851476	0.2767323	-0.152269	8.20E-08	9.87E-06
GENEBODY	CLDN12	7	90042240	1	cg25244356	0.78811969	0.56706839	0.470522159	-0.2210513	2.66E-07	1.72E-05
GENEBODY	CTSB	8	11710911	1	cg03654169	0.64798756	0.46403137	0.3500838	-0.1839562	1.45E-07	1.30E-05
GENEBODY	CTSS	1	150722805	1	cg10954654	0.80894692	0.59973143	0.489601082	-0.2092155	2.08E-07	1.52E-05
GENEBODY	DCD	12	55041948/55041379	2	cg00868860/cg13396068	0.57983145	0.41798674	0.28471787	-0.1618447	1.71E-05	1.85E-04
GENEBODY	EIF3IP1	7	109599644	1	cg14146669	0.7158862	0.55539858	0.426187	-0.1604876	2.73E-05	2.55E-04
GENEBODY	FAM49A	2	16767476	1	cg10090418	0.77069938	0.59044391	0.396925518	-0.1802555	2.19E-04	1.19E-03
GENEBODY	FCGR3B	1	161600769	1	cg04567009	0.67330976	0.51061459	0.379355841	-0.1626952	2.18E-05	2.18E-04
GENEBODY	GPR171	3	150916446	1	cg15715853	0.76345609	0.56104524	0.4579293	-0.2024108	1.33E-07	1.21E-05
GENEBODY	IL7R	5	35860337	1	cg01027405	0.47155128	0.32000952	0.238115591	-0.1515418	1.99E-06	4.94E-05
GENEBODY	ITLN1	1	160850377	1	cg07177437	0.67076678	0.50537068	0.445825432	-0.1653961	1.80E-06	4.63E-05
GENEBODY	KIAA0748	12	55362424/55368244	2	cg02710015/cg06352538	0.70253258	0.51994114	0.423248273	-0.1825914	7.25E-07	2.75E-05
GENEBODY	KRT14	17	39739598/39739541/39739912/39739860/39739524	5	cg03508348/cg05376477/cg06339505/cg12362508/cg15748407	0.82813819	0.67396028	0.631046352	-0.1541779	2.33E-06	5.36E-05
GENEBODY	LOC100128288	17	8263420	1	cg08906309	0.63322548	0.475523	0.337774082	-0.1577025	6.44E-05	4.74E-04
GENEBODY	LOC339568	20	37850248	1	cg04742334	0.57372006	0.39118003	0.263340682	-0.18254	4.10E-06	7.59E-05
GENEBODY	LOC643719	19	35068555/35068221	2	cg23200020/cg25418852	0.43822757	0.58900259	0.745249427	0.15077502	6.59E-04	2.86E-03
GENEBODY	LOC647121	1	121261146/121261404/121261077/121261369/121261602/121305260	6	cg04374231/cg05827631/cg06970370/cg07794500/cg19165105/cg20769683	0.32391262	0.48947165	0.5845832	0.16555903	3.42E-06	6.79E-05
GENEBODY	LYG2	2	99867344	1	cg12213414	0.73208942	0.52563324	0.400153191	-0.2064562	2.19E-06	5.19E-05
GENEBODY	MCHR2	6	100380550	1	cg07108581	0.64954945	0.48530788	0.381360482	-0.1642416	3.49E-06	6.86E-05
GENEBODY	MIR338	17	79099711	1	cg26766064	0.5117175	0.34908266	0.3546869	-0.1626348	1.78E-08	6.06E-06
GENEBODY	MIR376C	14	101506087	1	cg13995230	0.46416821	0.28744265	0.192639905	-0.1767256	7.91E-06	1.14E-04
GENEBODY	MIR495	14	101500125	1	cg14910227	0.69446684	0.53624244	0.459293677	-0.1582244	5.33E-06	8.84E-05
GENEBODY	MST1R	3	49938721/49938004/49939703	3	cg01917209/cg15211623/cg18292904	0.72026333	0.5503557	0.421140836	-0.1699076	2.66E-06	5.78E-05

GENEBODY	NDST3	4	119093021/118975504/119095240	3	cg06837981/cg12311175/cg26126295	0.57959507	0.42620212	0.344429245	-0.1533929	1.75E-04	1.00E-03
GENEBODY	ORMDL2	12	56213916	1	cg27554856	0.88013005	0.72482816	0.691191491	-0.1553019	7.11E-05	5.07E-04
GENEBODY	PATE3	11	125660444	1	cg24746315	0.74707571	0.58069167	0.517078341	-0.166384	7.64E-04	3.23E-03
GENEBODY	PRAC	17	46799640/46799479	2	cg12374721/cg14326989	0.24173976	0.45021132	0.256488038	0.20847155	5.30E-03	1.56E-02
GENEBODY	PSMG3	7	1607787/1607868/1607601/1607408/1607663	5	cg00375608/cg13042250/cg18958693/cg20151476/cg23903588	0.66348395	0.49326142	0.37804747	-0.1702225	5.79E-07	2.49E-05
GENEBODY	RSPH3	6	159420005/159416530	2	cg02895126/cg24249648	0.70916378	0.54519447	0.439121773	-0.1639693	4.86E-06	8.38E-05
GENEBODY	SBSN	19	36017061	1	cg06924602	0.56526531	0.38481138	0.283984809	-0.1804539	2.11E-07	1.53E-05
GENEBODY	SDR16C5	8	57228615	1	cg02141358	0.77667526	0.61986331	0.553771795	-0.1568119	2.59E-06	5.73E-05
GENEBODY	SELV	19	40007922	1	cg23201817	0.60075549	0.44122896	0.375011618	-0.1595265	1.18E-06	3.68E-05
GENEBODY	SLC22A25	11	62947565	1	cg21961694	0.58823213	0.39719341	0.254938509	-0.1910387	1.07E-05	1.35E-04
GENEBODY	ST3GAL1	8	134474257/134475410	2	cg12292213/cg14640789	0.76999565	0.53557833	0.411137514	-0.2344173	1.76E-06	4.58E-05
GENEBODY	SUSD5	3	33259920/33245094/33259889	3	cg10906602/cg15469655/cg26298814	0.25051059	0.41002647	0.54748555	0.15951589	1.67E-03	6.09E-03
GENEBODY	TEX15	8	30691170	1	cg26631437	0.62286601	0.45349208	0.33232755	-0.1693739	6.96E-06	1.04E-04
GENEBODY	TST	22	37414044/37412173/37414747/37414442	4	cg01419582/cg01553653/cg08727202/cg17575915	0.74000971	0.56872129	0.472251452	-0.1712884	6.97E-07	2.71E-05
GENEBODY	WDR64	1	241912764/241912510/241912768	3	cg05940691/cg06082598/cg07233230	0.88346178	0.72821386	0.780308389	-0.1552479	7.50E-04	3.18E-03
GENEBODY	ZNF563	19	12440923/12444273	2	cg13402083/cg25682920	0.75270955	0.57215202	0.47094505	-0.1805575	4.23E-10	2.44E-06
UTR3	APCDD1	18	10488229	1	cg18208602	0.75776442	0.59104243	0.443936245	-0.166722	5.98E-06	1.49E-04
UTR3	ARRDC5	19	4890970	1	cg10130497	0.64906054	0.49272441	0.345601477	-0.1563361	7.25E-06	1.65E-04
UTR3	ASB15	7	123277601	1	cg02621087	0.69356255	0.53329156	0.408837964	-0.160271	3.33E-06	0.000110987
UTR3	C10orf128	10	50363896	1	cg25921502	0.56676901	0.40965711	0.286231195	-0.1571119	2.03E-06	8.54E-05
UTR3	C1S	12	7178171	1	cg24212392	0.72310005	0.56616341	0.452192391	-0.1569366	3.12E-07	3.20E-05
UTR3	C3orf32	3	8661519	1	cg09248934	0.56862798	0.36792397	0.268243714	-0.200704	2.77E-08	1.29E-05
UTR3	CCDC46	17	63632069	1	cg11989257	0.86697215	0.70537346	0.653869918	-0.1615987	1.71E-06	7.88E-05
UTR3	CCDC74B	2	130897120	1	cg15638768	0.67402883	0.51184596	0.451492682	-0.1621829	7.08E-09	6.57E-06
UTR3	CD1D	1	158155757	1	cg18234111	0.57826903	0.39269838	0.269737286	-0.1855707	5.39E-06	1.39E-04
UTR3	CD300LF	17	72690539	1	cg08808817	0.71191849	0.54756676	0.448119541	-0.1643517	4.40E-06	1.23E-04
UTR3	CDC7	1	91990662	1	cg22013790	0.81067116	0.65403663	0.662419995	-0.1566345	5.89E-08	1.55E-05

UTR3	CDH13	16	83829911	1	cg05847519	0.67286552	0.47969204	0.321072532	-0.1931735	2.81E-05	3.89E-04
UTR3	CENPQ	6	49460031	1	cg14479014	0.80903437	0.61965753	0.547317405	-0.1893768	3.34E-10	1.70E-06
UTR3	CHST10	2	101008576	1	cg01660407	0.87220465	0.67662627	0.578576291	-0.1955784	1.08E-06	6.10E-05
UTR3	CIB3	19	16272242	1	cg01541846	0.66506362	0.51021043	0.394911218	-0.1548532	1.89E-07	2.63E-05
UTR3	CLDN18	3	137750103	1	cg09236311	0.64847136	0.48208906	0.334410223	-0.1663823	4.61E-05	0.000545028
UTR3	CLEC4D	12	8674684	1	cg05582397	0.66002753	0.50319973	0.378058127	-0.1568278	2.10E-05	3.24E-04
UTR3	CMTM1	16	66612955	1	cg09849405	0.2045236	0.37778662	0.654003182	0.17326303	7.24E-03	2.64E-02
UTR3	CNDP1	18	72252164	1	cg21700214	0.68480289	0.50947208	0.431826264	-0.1753308	1.21E-05	0.000226317
UTR3	CPLX1	4	779173/778827/778924/779230/779691/779480/779880/779568	8	cg05448404/cg06672737/cg06673130/cg06821199/cg07434284/cg17128947/cg17397631/cg18638914	0.37324724	0.5248243	0.645681274	0.15157706	1.45E-05	2.52E-04
UTR3	CRP	1	159682497	1	cg04861230	0.58299582	0.42996221	0.335184786	-0.1530336	5.74E-08	1.55E-05
UTR3	CXCL2	4	74963112	1	cg01470535	0.58833002	0.43652258	0.34538545	-0.1518074	5.59E-07	4.26E-05
UTR3	DAB1	1	57470853	1	cg01421140	0.50178006	0.35161283	0.254225914	-0.1501672	4.40E-06	1.23E-04
UTR3	DOCK2	5	169510081	1	cg09175734	0.63747036	0.46170475	0.335555305	-0.1757656	3.56E-06	1.13E-04
UTR3	EDNRA	4	148465429	1	cg03573792	0.66495647	0.51416448	0.458468332	-0.150792	4.54E-06	1.24E-04
UTR3	ELK4	1	205585639	1	cg04105597	0.68138763	0.51863703	0.415228641	-0.1627506	2.15E-07	2.68E-05
UTR3	FAM126A	7	22982162	1	cg27229764	0.83985201	0.64416748	0.495510705	-0.1956845	2.33E-06	9.23E-05
UTR3	FAM153B	5	175541958	1	cg08252384	0.66048473	0.50497632	0.3864653	-0.1555084	7.49E-06	1.68E-04
UTR3	FAM181A	14	94395516	1	cg18857951	0.73147512	0.55981623	0.464809332	-0.1716589	1.76E-06	7.96E-05
UTR3	FAM26D	6	116879492	1	cg25351588	0.72995594	0.51286077	0.382538236	-0.2170952	1.77E-07	2.57E-05
UTR3	GALNT14	2	31133547	1	cg10582371	0.56709419	0.40729544	0.319374855	-0.1597987	1.61E-06	7.66E-05
UTR3	GHR	5	42720521	1	cg18304305	0.62983734	0.44958044	0.387788564	-0.1802569	9.85E-06	1.96E-04
UTR3	GP6	19	55526208	1	cg27154418	0.78008026	0.60738721	0.41956515	-0.172693	1.44E-03	7.34E-03
UTR3	GRM2	3	51752143	1	cg11727252	0.77556587	0.61148758	0.5510914	-0.1640783	7.77E-06	1.72E-04
UTR3	GYPC	2	127453782	1	cg11611448	0.79602001	0.59034018	0.401720773	-0.2056798	3.01E-05	4.09E-04
UTR3	GZMA	5	54406016	1	cg05648002	0.67577133	0.51063234	0.356100214	-0.165139	2.52E-07	2.82E-05
UTR3	HOXC9	12	54396990	1	cg22679316	0.51111691	0.67966348	0.681937423	0.16854658	5.36E-06	0.000138764
UTR3	HOXD10	2	176984634	1	cg06005169	0.51182921	0.34905639	0.349526773	-0.1627728	1.72E-04	1.42E-03
UTR3	IFLTD1	12	25649090/25656194/25644389	3	cg06680863/cg11130955/cg21254753	0.52038823	0.35288952	0.271136409	-0.1674987	3.45E-06	1.13E-04
UTR3	IRF4	6	408730	1	cg16478536	0.67309126	0.51059167	0.359100409	-0.1624996	2.28E-04	1.75E-03
UTR3	KCNMB1	5	169805583	1	cg16425489	0.65433255	0.45722991	0.333462355	-0.1971026	1.70E-06	7.87E-05
UTR3	KCTD7	7	66105984	1	cg07522403	0.58708411	0.43470409	0.3336382	-0.15238	4.16E-06	1.18E-04

UTR3	KIAA1486	2	226518461	1	cg03731588	0.7869686	0.56651525	0.408501177	-0.2204533	1.81E-07	2.57E-05
UTR3	KRTAP15-1	21	31813075	1	cg16812893	0.59362799	0.44057094	0.334187759	-0.153057	3.64E-06	1.13E-04
UTR3	LASS3	15	100941674	1	cg27358936	0.64713756	0.48955094	0.428181414	-0.1575866	2.69E-06	9.84E-05
UTR3	LCE1C	1	152777382	1	cg16011217	0.63745746	0.48433732	0.364683118	-0.1531201	6.64E-06	1.58E-04
UTR3	LCE2A	1	152671707	1	cg16354520	0.54498256	0.37940957	0.252350123	-0.165573	2.59E-06	9.69E-05
UTR3	MFAP5	12	8799841	1	cg19543296	0.88506451	0.71806728	0.608226045	-0.1669972	9.45E-07	5.71E-05
UTR3	MMP16	8	89053427	1	cg02526439	0.60355949	0.43655326	0.351015227	-0.1670062	1.44E-06	7.12E-05
UTR3	NCKAP1L	12	54936840	1	cg26787915	0.87019336	0.71703318	0.51087105	-0.1531602	1.54E-05	2.63E-04
UTR3	NDRG4	16	58545502/58545770	2	cg04194840/cg26514728	0.73900369	0.57622246	0.491452386	-0.1627812	6.31E-05	6.89E-04
UTR3	NEU4	2	242758725	1	cg05195017	0.44223969	0.2641673	0.193258385	-0.1780724	2.17E-04	1.70E-03
UTR3	NLRP14	11	7092719	1	cg07103673	0.60690567	0.39699801	0.296656455	-0.2099077	5.53E-08	1.55E-05
UTR3	NUP43	6	150045504	1	cg00424166	0.76030968	0.60171094	0.534083577	-0.1585987	8.66E-09	6.96E-06
UTR3	PABPC4L	4	135121050	1	cg15867829	0.66543919	0.51234776	0.362865264	-0.1530914	4.51E-06	1.24E-04
UTR3	PANX3	11	124490178	1	cg10118227	0.79644059	0.60071637	0.471743309	-0.1957242	2.55E-07	2.82E-05
UTR3	PGK2	6	49753577	1	cg02482438	0.54314964	0.39180955	0.281179145	-0.1513401	1.59E-05	2.69E-04
UTR3	PLCL1	2	199013270	1	cg09349723	0.66798426	0.4970543	0.349158882	-0.17093	9.47E-06	1.91E-04
UTR3	POLR1B	2	113333716	1	cg02614999	0.60449706	0.45309597	0.372716527	-0.1514011	4.36E-07	3.77E-05
UTR3	POU4F2	4	147562073	1	cg21200539	0.37320488	0.54191748	0.697049414	0.1687126	0.00052295	0.003327761
UTR3	PROZ	13	113826554	1	cg02898500	0.80941878	0.63143543	0.659898791	-0.1779833	1.55E-04	1.31E-03
UTR3	PXDN	2	1637068	1	cg10888111	0.8753676	0.59430805	0.438202605	-0.2810596	2.80E-07	3.01E-05
UTR3	RBMXL2	11	7112356	1	cg06176750	0.65238308	0.45420675	0.274610925	-0.1981763	3.24E-05	4.26E-04
UTR3	RHOJ	14	63757917	1	cg24186774	0.68639383	0.51543068	0.367702455	-0.1709631	1.23E-05	2.28E-04
UTR3	RIMS2	8	105264286	1	cg07664999	0.5521154	0.37394201	0.249843391	-0.1781734	3.31E-07	3.31E-05
UTR3	SELE	1	169693534	1	cg18229820	0.66968789	0.51538248	0.47013515	-0.1543054	4.04E-08	1.39E-05
UTR3	SELV	19	40009835	1	cg07052524	0.61414098	0.45252968	0.299807617	-0.1616113	6.77E-05	7.22E-04
UTR3	SIRPD	20	1514929	1	cg00472837	0.70686384	0.54144523	0.422869095	-0.1654186	1.36E-05	2.41E-04
UTR3	SIX3	2	45172201/45172169/45172265/45172972	4	cg03717442/cg08696165/cg16509777/cg23245620	0.3196168	0.47360698	0.553462845	0.15399019	2.57E-03	1.15E-02
UTR3	SLC26A4	7	107357932	1	cg06535968	0.72728662	0.55776256	0.438293591	-0.1695241	1.88E-06	8.15E-05
UTR3	TCF15	20	584773	1	cg02939767	0.5923177	0.34688905	0.227455723	-0.2454287	5.51E-07	4.26E-05
UTR3	TMEM134	11	67231878	1	cg07303548	0.61020254	0.45479691	0.32130235	-0.1554056	4.87E-06	1.31E-04
UTR3	TMEM30A	6	75965559	1	cg11887083	0.74361467	0.57378759	0.550888273	-0.1698271	1.03E-08	7.48E-06
UTR3	TMPRSS11A	4	68776652	1	cg26673722	0.59197598	0.42410026	0.300292691	-0.1678757	3.41E-06	1.12E-04
UTR3	TRPS1	8	116422845	1	cg18430465	0.63577677	0.47986874	0.343752909	-0.155908	1.90E-05	3.01E-04

UTR3	TSC22D1	13	45008447/45008526	2	cg03097389/cg17600231	0.80057919	0.62008318	0.527408509	-0.180496	7.18E-07	4.76E-05
UTR3	UGT3A1	5	35954233	1	cg17210837	0.50550368	0.34167663	0.250504545	-0.1638271	3.83E-08	1.39E-05
UTR3	UNC5C	4	96090330	1	cg03024478	0.62998052	0.46755009	0.362151282	-0.1624304	1.81E-05	2.93E-04
UTR3	USH2A	1	216348212/215796556	2	cg00972761/cg14964274	0.67150538	0.49637768	0.362080127	-0.1751277	2.36E-05	3.49E-04
UTR3	VCAM1	1	101204337	1	cg22454770	0.65708404	0.50568061	0.373783205	-0.1514034	2.40E-06	9.41E-05
UTR3	ZAR1	4	48496404	1	cg22289785	0.57601956	0.42430389	0.342240323	-0.1517157	1.43E-08	9.14E-06
UTR3	ZNF470	19	57091383	1	cg09287295	0.71308506	0.53275462	0.3893565	-0.1803304	1.17E-05	2.20E-04
UTR3	ZSCAN5A	19	56732932	1	cg17767567	0.73060592	0.56120494	0.473755191	-0.169401	4.28E-07	3.77E-05

Table S3.Differentially methylated regions (DMRs) between high grade adenoma (HGA) and Normal samples

Location	Gene	CHR	MAPINFO	Number of sites	Designed Probes	Normal	LGA	HGA	Different β	P value	Adjusted P value
TSS1500	MARCH1	4	165304842/165305815/165304855/165305050/165305334/165305022/165305238/165305314	8	cg02990869/cg06166885/cg07160277/cg10453719/cg17310600/cg18685561/cg19037454/cg25291004	0.486301355	0.548860102	0.642036755	0.1557354	3.86E-08	2.77E-07
TSS1500	MARCH11	5	16180315/16180393/16180419/16180259/16181039/16180266	6	cg11452391/cg12456714/cg16182986/cg18325622/cg21582253/cg23065934	0.410983289	0.468445128	0.595432839	0.18444955	2.55E-07	1.45E-06
TSS1500	A1CF	10	52645847	1	cg16531903	0.498551665	0.390118706	0.30903515	-0.189516515	1.91E-10	3.89E-09
TSS1500	A2M	12	9269178/9269671	2	cg00134295/cg00146928	0.40638476	0.299459397	0.221163832	-0.185220928	1.34E-13	5.30E-11
TSS1500	A2ML1	12	8973966/8974844/8974786	3	cg12168926/cg15769388/cg23546356	0.593964413	0.502333325	0.41010355	-0.183860863	1.62E-10	3.43E-09
TSS1500	A4GNT	3	137851874/137852643/137851776	3	cg18931888/cg19734045/cg23669440	0.585866555	0.413583856	0.304446464	-0.281420091	6.45E-12	4.20E-10
TSS1500	ABCC11	16	48269349/48269424/48269819	3	cg04388863/cg08404739/cg09147400	0.5984097	0.486570378	0.408502702	-0.189906998	4.18E-12	3.36E-10
TSS1500	ABCC13	21	15645787/15645810/15645649	3	cg18631692/cg19211382/cg23322112	0.707675485	0.621322794	0.530965095	-0.17671039	1.75E-08	1.42E-07
TSS1500	ABHD8	19	17414977/17414770/17415626/17415155	4	cg01445659/cg04614656/cg08702612/cg17328880	0.544542253	0.449392267	0.385518386	-0.159023866	8.16E-12	4.90E-10
TSS1500	ABRA	8	107783270/107782734	2	cg07067773/cg17049320	0.64380502	0.579914133	0.461314023	-0.182490997	2.11E-08	1.66E-07
TSS1500	ACAN	15	89346205/89345688/89346269	3	cg06675190/cg14914908/cg27621340	0.42568255	0.500547817	0.614764559	0.189082009	8.81E-09	7.95E-08
TSS1500	ACCSL	11	44068709/44069008/44068491	3	cg08403064/cg14686207/cg15035528	0.48815877	0.414019125	0.31807552	-0.17008325	6.77E-08	4.52E-07
TSS1500	ACIN1	14	23565052/23565044/23541523/23565029	4	cg00855938/cg00884794/cg09367198/cg11819429	0.470758145	0.4121575	0.316091795	-0.15466635	4.56E-09	4.68E-08
TSS1500	ACMSD	2	135594780/135595323/135595627	3	cg02499308/cg02812142/cg18766847	0.556894303	0.470244541	0.360534591	-0.196359712	3.12E-09	3.42E-08
TSS1500	ACOT2	14	74034619/74035398/74034893	3	cg07179457/cg20067142/cg25598083	0.49342484	0.359211117	0.279924964	-0.213499876	2.39E-10	4.61E-09
TSS1500	ACR	22	51176407/51175515/51175364	3	cg05615487/cg25232725/cg26034629	0.644708405	0.553545344	0.4231613	-0.221547105	4.55E-09	4.68E-08

TSS1500	ACSBG2	19	6134933/6135279	2	cg01452549/cg27274989	0.697282073	0.5711237	0.491302093	-0.205979979	9.97E-10	1.37E-08
TSS1500	ACSL3	2	223725497/223725459/223724754	3	cg03954616/cg21498125/cg23325384	0.47301181	0.388806661	0.297538968	-0.175472842	3.88E-10	6.61E-09
TSS1500	ACSM5	16	20420652/20420222	2	cg04428453/cg06686156	0.758055383	0.658663292	0.5910735	-0.166981883	5.93E-09	5.77E-08
TSS1500	ACSS3	12	81471419/81471311/81471533	3	cg00354572/cg08314603/cg25971727	0.21885051	0.4004886	0.471809096	0.252958586	1.12E-07	7.01E-07
TSS1500	ACTA1	1	229570057/229570665/229570188/229570052/229570288/229570230	6	cg00064261/cg04900080/cg07777790/cg11689813/cg12908399/cg20025656	0.283263839	0.304019213	0.43376713	0.150503291	6.92E-06	2.64E-05
TSS1500	ACTL9	19	8810253/8810115/8810154	3	cg02339243/cg12148774/cg19611002	0.668944728	0.576734089	0.482399359	-0.186545369	1.17E-10	2.74E-09
TSS1500	ACTR3C	7	150021553/150001179/150021488/150021518	4	cg01234517/cg18222543/cg21577356/cg24474409	0.561481453	0.449447733	0.369773582	-0.191707871	4.04E-10	6.77E-09
TSS1500	ADAM18	8	39441473	1	cg27485269	0.598169635	0.507976156	0.395113705	-0.20305593	7.57E-09	7.00E-08
TSS1500	ADAM21	14	70923456	1	cg03512208	0.75156432	0.675034067	0.576029118	-0.175535202	0.00026014	0.000700924
TSS1500	ADAM21P1	14	70715926/70715730	2	cg19992629/cg20576094	0.798079973	0.721657458	0.63929822	-0.158781752	5.80E-10	8.92E-09
TSS1500	ADAM7	8	24297818/24297103	2	cg08383526/cg17897391	0.665746573	0.578878569	0.496065175	-0.169681398	1.99E-06	8.75E-06
TSS1500	ADAMTS16	5	5140029/5139626/5140003/5139878/5139610/5139510/5139069/5139866/5139855/5139334/5139874/5139797/5139512/5139508/5139643/5140001/5139458/5139853	18	cg00127167/cg01085224/cg03209854/cg04136610/cg07834743/cg10881764/cg13603589/cg15048991/cg15409013/cg16068490/cg16508480/cg16670809/cg18503643/cg19452011/cg19813025/cg22954449/cg25536014/cg25973534	0.41784288	0.516642298	0.651251281	0.233408402	4.85E-11	1.51E-09
TSS1500	ADAMTS20	12	43946880/43945998/43946341/43946518/43946284/43946493	6	cg00938429/cg01345315/cg17459298/cg21911301/cg24116870/cg24606791	0.554121641	0.629093916	0.705131964	0.151010323	2.89E-10	5.32E-09

TSS1500	ADAMTS5	21	28340304/28340139/28339711/28339705/28339907	5	cg02462195/cg02800810/cg07942598/cg18895813/cg21646598	0.364109825	0.427270481	0.557634634	0.193524809	7.27E-08	4.80E-07
TSS1500	ADARB2	10	1779930/1780184/1780263/1779972/1780003	5	cg02270183/cg02609848/cg17000076/cg18727303/cg23684973	0.12260361	0.2097999	0.334762288	0.212158678	3.25E-06	1.35E-05
TSS1500	ADCY1	7	45613825/45613752/45613725/45613410/45613676	5	cg04028634/cg13070193/cg13523557/cg24676071/cg26459372	0.212489829	0.274238133	0.41812544	0.205635611	6.76E-08	4.52E-07
TSS1500	ADCY8	8	132053823/132053262/132053773/132053780	4	cg01245966/cg02842496/cg07312654/cg26351229	0.356303169	0.448107531	0.586348588	0.230045419	4.96E-10	7.93E-09
TSS1500	ADCYAP1	18	904963/904899/904851/904920/904523/904885/904878/903764	8	cg11850773/cg15458338/cg16457786/cg18319029/cg19962990/cg21438101/cg24287438/cg25911901	0.266016248	0.37385272	0.514081076	0.248064829	6.33E-07	3.20E-06
TSS1500	ADCYAP1R1	7	31091245/31091825/31091813/31091718	4	cg00256593/cg17617930/cg20165074/cg27076139	0.121036297	0.162474221	0.386411279	0.265374982	6.44E-07	3.25E-06
TSS1500	ADH1A	4	100212536/100212502	2	cg03806087/cg23949936	0.63870982	0.558197378	0.429118041	-0.209591779	2.03E-10	4.09E-09
TSS1500	ADH6	4	100140905	1	cg07219303	0.36205541	0.274296989	0.207151132	-0.154904278	7.15E-09	6.67E-08
TSS1500	ADORA2A	22	24823141/24823110/24823050/24822802	4	cg04250930/cg15499799/cg26001125/cg26354221	0.765259763	0.679375379	0.55487363	-0.210386133	2.77E-11	1.03E-09
TSS1500	ADRA1A	8	26723946/26724234/26723584/26723365/26724001	5	cg00460235/cg03651608/cg22295787/cg22461835/cg26730369	0.273442668	0.324968076	0.469693704	0.196251036	8.14E-09	7.45E-08
TSS1500	ADSSL1	14	105190158/105195816/105189737/105189936/105195480/105194980	6	cg02043070/cg02263377/cg04578317/cg13636887/cg20546518/cg25954705	0.496639282	0.409192559	0.312430763	-0.184208519	2.69E-10	5.03E-09
TSS1500	AEBP1	7	44142973/44143290/44143438	3	cg06852744/cg08495088/cg12955216	0.72110375	0.626615009	0.546929333	-0.174174417	3.20E-10	5.72E-09
TSS1500	AFAP1	4	7941894/7942031/7942020	3	cg10028549/cg18688746/cg19702703	0.61293722	0.502182494	0.433697123	-0.179240097	4.60E-08	3.24E-07

TSS1500	AFAP1L2	10	116165190/116164955/116164849	3	cg11453400/cg15657704/cg20196291	0.44985006	0.358432519	0.29841968	-0.15143038	5.90E-10	9.04E-09
TSS1500	AGR2	7	16845331	1	cg12565635	0.558866275	0.408002306	0.323608105	-0.23525817	3.24E-11	1.15E-09
TSS1500	AICDA	12	8766467/8766941	2	cg10465202/cg27562311	0.756507785	0.647370642	0.488450232	-0.268057553	1.21E-09	1.60E-08
TSS1500	AJAP1	1	4714672/4714033/4714171/4714574/4714008	5	cg12475759/cg13495205/cg15484532/cg17741689/cg20959866	0.358840883	0.457001437	0.596548438	0.237707555	6.61E-12	4.28E-10
TSS1500	AKR1B1	7	134144288/134144106/134144118	3	cg04663564/cg08167706/cg23918923	0.33867475	0.432639777	0.511341922	0.172667172	0.001061435	0.002519034
TSS1500	AKR1CL1	10	5228106/5227757	2	cg13585175/cg14011422	0.56437276	0.47936385	0.391363427	-0.173009333	2.33E-11	8.97E-10
TSS1500	ALX1	12	85673200/85673221/85673270/85672623/85673347	5	cg03781931/cg13916459/cg14996220/cg16413687/cg25800765	0.393886586	0.488537192	0.58970695	0.195820365	1.40E-08	1.17E-07

TSS1500	ALX4	11	44333030/44332653/44332454/44332138/44332216/44333072/44332385/44332790/44332636/44332664/44332369/44332938/44332340/44332940/44333067/44332195/44332913/44333070/44332540/44332224/44332134/44331978/44333131/44332958/44332719/44332972/44332838/44332624/44332891/44332332/44332558/44331934/44332756/44332685/44332128/44333192/44332507/44332754/44332849	39	cg00221654/cg00268840/cg00374839/cg01483681/cg01564033/cg02184008/cg03442378/cg04367252/cg04367345/cg04578774/cg04858155/cg06245037/cg07462292/cg07704934/cg08149193/cg09649245/cg10749822/cg11391201/cg13807496/cg14252492/cg14410064/cg14868747/cg15786900/cg16065899/cg16125371/cg16321280/cg17471467/cg18321548/cg18486698/cg19020830/cg21704109/cg21937554/cg21978924/cg22974228/cg23385200/cg24008884/cg24362098/cg26717041/cg27318318	0.278009027	0.337785395	0.472054544	0.194045516	5.51E-08	3.78E-07
TSS1500	AMTN	4	71382958/71383999	2	cg15964593/cg17536071	0.50848989	0.419287194	0.338948332	-0.169541558	1.87E-06	8.24E-06
TSS1500	ANGEL2	1	213189641/213189823/213190571/213189773	4	cg14610958/cg19863073/cg21951459/cg24410391	0.805590036	0.70427454	0.59785071	-0.207739326	1.74E-09	2.14E-08
TSS1500	ANKRD22	10	90612232/90612228/90613015	3	cg00103806/cg03818395/cg24065504	0.498195385	0.400352393	0.339889011	-0.158306374	4.52E-10	7.37E-09
TSS1500	ANO2	12	6056169/6056330/6056642	3	cg12666919/cg16451358/cg23363039	0.67893045	0.618869761	0.515809315	-0.163121135	7.26E-08	4.80E-07
TSS1500	APCS	1	159556997/159557257	2	cg05919685/cg13968061	0.367865315	0.254767489	0.176404172	-0.191461143	6.59E-11	1.81E-09
TSS1500	APOA2	1	161193991/161193965/161193933/161194369	4	cg01053621/cg08922317/cg18281418/cg27433062	0.719119685	0.622324496	0.535912568	-0.183207117	8.82E-10	1.24E-08
TSS1500	APOBEC1	12	7819180	1	cg16879115	0.65364044	0.465226467	0.339518605	-0.314121835	3.08E-13	7.87E-11

TSS1500	APOE	19	45408121/45407945/45407868	3	cg01032398/cg04406254/cg14123992	0.516337348	0.427040581	0.35489253	-0.161444818	3.06E-13	7.87E-11
TSS1500	AQP5	12	50354962/50354998/50353951/50354840	4	cg03020208/cg08266366/cg19220825/cg26328335	0.300126293	0.318647856	0.47726052	0.177134227	3.12E-08	2.33E-07
TSS1500	ARFGAP3	22	43254081/43254168/43254043	3	cg00539564/cg09367092/cg23778094	0.791412908	0.706126272	0.626928179	-0.16448473	4.26E-07	2.26E-06
TSS1500	ARHGAP20	11	110584063/110584082/110583662/110583882/110584327/110583945	6	cg03110368/cg03927037/cg04282419/cg04417028/cg06106428/cg08012199	0.273987107	0.343640627	0.445062776	0.171075669	0.000153952	0.000434981
TSS1500	ARHGAP28	18	6833417	1	cg03026004	0.531716005	0.343916939	0.202823204	-0.328892801	7.12E-15	1.50E-11
TSS1500	ARID5A	2	97202260/97201196	2	cg02085953/cg06496010	0.648115905	0.531712394	0.495789245	-0.15232666	1.28E-07	7.92E-07
TSS1500	ARMC3	10	23216727/23216695/23216637	3	cg11673092/cg19542987/cg22631555	0.177264821	0.293968522	0.417631586	0.240366765	1.93E-05	6.67E-05
TSS1500	ARRDC5	19	4903909/4903179/4903952	3	cg02701435/cg12255509/cg22898924	0.775775478	0.698670139	0.619307659	-0.156467818	1.42E-08	1.19E-07
TSS1500	ASAP1	8	131414474/131414614/131415376	3	cg00077877/cg00659129/cg18582010	0.68526912	0.545931603	0.449298914	-0.235970206	1.17E-09	1.56E-08
TSS1500	ASB10	7	150885654/150884979/150885084/150885361/150885458/150885027/150885103/150884848/150884914	9	cg01954686/cg04671739/cg05181846/cg07609372/cg09539720/cg10980293/cg13645954/cg16537044/cg26159905	0.511255849	0.413287321	0.321359829	-0.189896019	5.26E-13	9.59E-11
TSS1500	ASB4	7	95114680/95115026	2	cg02395581/cg09375488	0.579556483	0.482327283	0.377546509	-0.202009973	7.55E-11	1.98E-09
TSS1500	ASB5	4	177191536	1	cg26417152	0.751656895	0.678414217	0.537736323	-0.213920572	2.45E-05	8.28E-05
TSS1500	ASCL4	12	108167730/108167502/108167448	3	cg16207673/cg20173428/cg20318358	0.620546271	0.489181969	0.408143162	-0.212403108	1.29E-10	2.92E-09
TSS1500	ASGR1	17	7083519/7083413/7083257	3	cg09245073/cg13335341/cg13808223	0.622772678	0.54892322	0.468054705	-0.154717974	3.76E-09	3.99E-08
TSS1500	ATAD3C	1	1384091/1384038	2	cg11405695/cg21001441	0.44518399	0.346447419	0.285277195	-0.159906795	1.23E-10	2.82E-09
TSS1500	ATG5	6	106774064/106773949/106774172	3	cg09354267/cg14951955/cg21148531	0.522668297	0.469559404	0.354837779	-0.167830518	1.75E-09	2.14E-08
TSS1500	ATP13A5	3	193097361/193097879/193096836/193097750	4	cg01748263/cg05503824/cg09449853/cg24935655	0.809145518	0.711243642	0.607231131	-0.201914387	2.98E-09	3.31E-08

TSS1500	ATP6V1G1	9	117349544/117349621/117349316	3	cg14391907/cg14535884/cg21249829	0.497025258	0.413479222	0.33261612	-0.164409137	4.22E-08	3.01E-07
TSS1500	ATP8A2	13	25945811/25945255/25945656/25945413	4	cg00044871/cg07973479/cg17713780/cg26153234	0.677211385	0.581965215	0.492757221	-0.184454164	2.27E-11	8.87E-10
TSS1500	ATP9B	18	76828521/76828655/76829143/76829138	4	cg06556497/cg16665444/cg16961218/cg22093035	0.53972239	0.618516867	0.730038395	0.190316005	7.90E-09	7.27E-08
TSS1500	AVIL	12	58211193/58210661/58210878/58210716	4	cg01424889/cg04046364/cg06317209/cg26620147	0.549930572	0.392942883	0.316282894	-0.233647678	1.10E-12	1.54E-10
TSS1500	AZGP1	7	99574466/99574383/99574420	3	cg10372302/cg12019109/cg26680675	0.69409815	0.554441141	0.425508592	-0.268589558	6.23E-12	4.16E-10
TSS1500	B3GALT1	2	168674769/168673805/168674479	3	cg00282195/cg10224537/cg18015625	0.655533025	0.586083128	0.457510014	-0.198023011	4.98E-06	1.97E-05
TSS1500	B3GNT5	3	182970719/182970675/182970728	3	cg21450829/cg26436136/cg27324128	0.519082595	0.410640535	0.325359411	-0.193723184	1.03E-10	2.49E-09
TSS1500	B3GNT6	11	76745087/76744970/76744076	3	cg00721016/cg09933401/cg10101468	0.614882115	0.504360372	0.4274087	-0.187473415	2.20E-08	1.73E-07
TSS1500	BACH2	6	91007365/91007625/91007563	3	cg09790829/cg10913050/cg15380603	0.50720581	0.436758428	0.3537978	-0.15340801	8.59E-09	7.80E-08
TSS1500	BAIAP2L2	22	38507451/38507857/38508132	3	cg11461302/cg15944459/cg21007971	0.621353555	0.527046708	0.438863032	-0.182490523	1.76E-09	2.14E-08
TSS1500	BASE	20	31780882/31781151/31781060	3	cg01775265/cg10637995/cg12161192	0.553312482	0.464440576	0.371211789	-0.182100692	1.75E-10	3.65E-09
TSS1500	BAT2L2	1	171454351/171453943	2	cg00526163/cg10143731	0.536697805	0.424156072	0.264537845	-0.27215996	2.00E-12	2.10E-10
TSS1500	BBOX1	11	27061533/27061899/27061756	3	cg11832339/cg12805865/cg19320730	0.527654537	0.429284387	0.343140339	-0.184514197	1.65E-09	2.05E-08
TSS1500	BCL2A1	15	80264706/80264696/80264770	3	cg23708261/cg24924631/cg27177709	0.79074286	0.649559333	0.513380041	-0.277362819	3.34E-09	3.62E-08
TSS1500	BCL2L15	1	114430903/114431096/114431234	3	cg08266106/cg13063617/cg18941151	0.86143578	0.778888897	0.663659423	-0.197776357	2.57E-07	1.46E-06
TSS1500	BET3L	6	116867006/116867864	2	cg00953399/cg07258838	0.619891508	0.509587497	0.409660936	-0.210230571	3.74E-10	6.45E-09
TSS1500	BEYLA	8	47751953/47751494/47751623	3	cg00658449/cg07531663/cg21767657	0.350532735	0.258877394	0.184446652	-0.166086083	9.98E-12	5.31E-10

TSS1500	BFSP2	3	133117569/133117420/133118114	3	cg15081844/cg20383064/cg22465341	0.757362667	0.653993333	0.532930911	-0.224431756	1.02E-08	8.97E-08
TSS1500	BLID	11	121987180/121987155/121987203	3	cg13125488/cg15056794/cg15598802	0.748611845	0.656468039	0.543466927	-0.205144918	0.000118404	0.000343568
TSS1500	BMP10	2	69100119/69099285/69099584	3	cg10881615/cg12711530/cg16980530	0.791024762	0.66490742	0.552936695	-0.238088066	8.09E-10	1.16E-08
TSS1500	BMP2	20	6747409	1	cg26665274	0.55499735	0.439309483	0.357869023	-0.197128327	1.05E-10	2.52E-09
TSS1500	BNC1	15	83954392/83953998/83953883/83953929/83953780/83954246/83953765/83954849/83954395/83954150/83953880/83953709/83953744/83953819/83954231/83953775/83953864/83954229/83953690	19	cg00768409/cg02713760/cg06523224/cg07016258/cg09180848/cg10275315/cg14385245/cg14786290/cg16049391/cg16254374/cg17124224/cg18560204/cg18952647/cg20941110/cg23741520/cg26429925/cg26654798/cg27169020/cg27304406	0.418563902	0.507321721	0.597384941	0.178821039	1.10E-09	1.48E-08
TSS1500	BNIP3	10	133796348/133796494/133796164/133795885/133795796/133796523/133796476/133796559	8	cg02388416/cg02835981/cg02886408/cg08079580/cg11197015/cg13599131/cg21762589/cg27353308	0.473879868	0.544565964	0.639898829	0.166018961	4.70E-09	4.80E-08
TSS1500	BOK	2	242497595/242497960	2	cg03558769/cg11374368	0.530508728	0.428008608	0.368517734	-0.161990993	4.07E-11	1.34E-09
TSS1500	BOLL	2	198651498/198650880/198650985/198651076/198650603/198650752/198650987/198651576/198651303/198651347	10	cg00926400/cg03774803/cg05783139/cg07495363/cg07734961/cg10124201/cg13356896/cg16002818/cg17560332/cg24589459	0.410338441	0.486456147	0.65089284	0.240554398	1.50E-08	1.25E-07
TSS1500	BPIL3	20	31618468/31618209/31618931	3	cg00471000/cg00582765/cg02309431	0.699153855	0.607729461	0.532883032	-0.166270823	3.44E-08	2.53E-07
TSS1500	BRD7P3	6	118822131/118821449	2	cg01019344/cg24520968	0.785562243	0.678681686	0.58263605	-0.202926193	4.80E-10	7.73E-09
TSS1500	BRF2	8	37707692/37708609/37708905	3	cg19904653/cg20466478/cg22871204	0.46839186	0.316849877	0.205657763	-0.262734097	3.23E-14	2.45E-11

TSS1500	BRUNOL4	18	35146726/35146393/35146770/35146766/35146474/35146589/35146478/35147090/35147162/35146227/35147079/35147056/35146591/35147276/35146668/35147165/35146454/35147246/35146670/35146450/35147465	21	cg00590960/cg01273679/cg03151187/cg06103031/cg07600871/cg12466737/cg13138137/cg13382322/cg14350197/cg14839351/cg15179725/cg17319486/cg17973164/cg18409730/cg19853833/cg20346912/cg23243463/cg24107979/cg24506862/cg25023275/cg25216403	0.203256005	0.271151456	0.413594709	0.210338704	1.07E-05	3.91E-05
TSS1500	BSG	19	571107/571204/571744/571321/571416/571802/572309/571323/570458	9	cg01532103/cg05622915/cg07513435/cg13578393/cg17522907/cg18341969/cg22431486/cg25660646/cg26529300	0.789319535	0.606996978	0.594050391	-0.195269144	1.80E-05	6.27E-05
TSS1500	BTG4	11	111383668/111384124/111384048/111384223/111383603/111383288/111383576/111383515/111383624/111383889/111383653	11	cg01192900/cg05281603/cg08827001/cg09148270/cg13767940/cg18515591/cg21881253/cg22879515/cg23211240/cg24041078/cg26561785	0.197607313	0.243806879	0.403806738	0.206199425	1.13E-05	4.12E-05
TSS1500	BTNL2	6	32375231/32375317/32376095/32375447/32376066/32375672	6	cg00978102/cg02158755/cg06871764/cg14853443/cg18060330/cg18067840	0.447310635	0.361828749	0.297159351	-0.150151284	6.55E-10	9.80E-09
TSS1500	C10orf114	10	21787596/21787079/21786419	3	cg10973934/cg18224988/cg24823751	0.558163741	0.478522211	0.384827344	-0.173336397	1.00E-07	6.35E-07
TSS1500	C10orf67	10	23635200	1	cg25844471	0.82135045	0.789701489	0.669210132	-0.152140318	1.28E-05	4.61E-05
TSS1500	C10orf81	10	115510107/115510718/115510928	3	cg07941628/cg16526673/cg22618337	0.617620295	0.485419153	0.40333993	-0.214280365	1.37E-10	3.05E-09
TSS1500	C10orf93	10	134756503/134756306/134756610/134756994/134756707	5	cg03354772/cg07421032/cg08325191/cg17000480/cg20100445	0.357539535	0.466157022	0.583555727	0.226016192	2.59E-09	2.95E-08

TSS1500	C10orf96	10	118083701/118082519	2	cg04827379/cg06640662	0.779178033	0.741080519	0.618550866	-0.160627167	3.42E-06	1.41E-05
TSS1500	C11orf49	11	46957888/46957977/46957758	3	cg08941576/cg18100564/cg26198807	0.490478218	0.407150744	0.325264809	-0.165213408	1.85E-09	2.24E-08
TSS1500	C12orf39	12	21678044/21678386	2	cg03833948/cg24957964	0.661306323	0.520749944	0.394534359	-0.266771963	1.62E-11	7.24E-10
TSS1500	C12orf56	12	64784663/64784686/64784626	3	cg01031441/cg20116199/cg24990968	0.36124463	0.442400617	0.587324802	0.226080172	1.63E-08	1.34E-07
TSS1500	C13orf28	13	113030011/113029350/113030235	3	cg03625684/cg04107005/cg14591352	0.61547812	0.52829282	0.437087852	-0.178390268	4.08E-11	1.34E-09
TSS1500	C13orf29	13	111522975/111522932/111522865/111522985	4	cg13531120/cg15703035/cg22131172/cg22790835	0.494284705	0.414499193	0.340627084	-0.153657621	4.30E-07	2.28E-06
TSS1500	C13orf30	13	43354674/43354421/43354767	3	cg07409200/cg18860329/cg26529044	0.550397928	0.484633502	0.38539901	-0.164998918	2.20E-06	9.58E-06
TSS1500	C14orf118	14	76617755/76617402/76617983	3	cg16333262/cg18373317/cg26082202	0.78633787	0.670777908	0.590537384	-0.195800486	9.40E-10	1.31E-08
TSS1500	C14orf184	14	92042045/92041829/92042040/92042333	4	cg06181761/cg12156272/cg16120275/cg16555466	0.581622065	0.485858207	0.421224535	-0.16039753	2.26E-07	1.30E-06
TSS1500	C14orf50	14	65015998/65016258	2	cg08152588/cg13540480	0.71695364	0.594683217	0.494283345	-0.222670295	4.08E-10	6.82E-09
TSS1500	C15orf26	15	81426360/81426347/81426434	3	cg14380111/cg25029264/cg26546864	0.231070845	0.277867735	0.402607574	0.171536729	0.000183828	0.000511804
TSS1500	C15orf38	15	90456541/90456924/90456686/90456619	4	cg01256846/cg06184785/cg07961715/cg26702670	0.496708204	0.394856324	0.312790943	-0.183917261	8.14E-11	2.11E-09
TSS1500	C15orf5	15	77518882/77519170	2	cg06817669/cg08130265	0.5018212	0.370531797	0.288939598	-0.212881602	1.31E-11	6.27E-10
TSS1500	C15orf63	15	44092166/44092345/44092314/44092398	4	cg02556940/cg08278937/cg13793048/cg26552774	0.796005046	0.731434783	0.614546082	-0.181458964	1.14E-06	5.36E-06
TSS1500	C16orf73	16	1922425/1922370/1922400/1922577	4	cg01422476/cg03467725/cg08734647/cg27105890	0.315759298	0.350905211	0.511049441	0.195290143	0.000521927	0.001322874
TSS1500	C16orf78	16	49407465/49407186	2	cg03478444/cg07804582	0.779754363	0.698456039	0.581780732	-0.197973631	4.83E-10	7.78E-09
TSS1500	C16orf89	16	5117565/5116674	2	cg02878222/cg27664407	0.54217156	0.387457372	0.294618095	-0.247553465	4.82E-12	3.60E-10

TSS1500	C17orf102	17	32907132/32907639/32906622/32907388/32907002/32907705/32907636/32906991/32907731	9	cg00196787/cg03442425/cg04790084/cg06459443/cg06557358/cg10451253/cg21745612/cg23791505/cg27213352	0.239433865	0.29955866	0.451134977	0.211701112	1.15E-06	5.37E-06
TSS1500	C17orf74	17	7328585/7328520/7328532	3	cg04374959/cg08568447/cg08903422	0.757359278	0.689864628	0.591138732	-0.166220546	3.63E-07	1.97E-06
TSS1500	C17orf77	17	72580515/72580458	2	cg06794038/cg21828432	0.486370203	0.383258878	0.301296625	-0.185073578	2.71E-09	3.08E-08
TSS1500	C17orf82	17	59488635/59488443/59488240/59488116	4	cg07482936/cg12658720/cg13918518/cg15885672	0.222752795	0.27811998	0.3869746	0.164221805	1.13E-08	9.84E-08
TSS1500	C17orf88	17	41994096/41993127/41994119/41994181	4	cg12496455/cg16631417/cg18464244/cg24631052	0.727791195	0.680008733	0.558075757	-0.169715438	4.63E-08	3.26E-07
TSS1500	C18orf34	18	31020754/31021003/31020511/31020418/31020806/31021065/31020973/31020627	8	cg00625334/cg03478497/cg05245861/cg05332960/cg05994094/cg18382305/cg20615848/cg20820063	0.295185116	0.358932728	0.498209496	0.203024381	1.58E-07	9.56E-07
TSS1500	C19orf41	19	50667404/50667086/50667801	3	cg01867155/cg03095288/cg06708133	0.441437175	0.317565419	0.254091046	-0.187346129	1.86E-09	2.25E-08
TSS1500	C1orf110	1	162838907/162838809	2	cg01403607/cg12610079	0.651612248	0.521472908	0.41654955	-0.235062698	4.58E-12	3.51E-10
TSS1500	C1orf116	1	207206447	1	cg15485859	0.586296185	0.479907433	0.367807273	-0.218488912	1.06E-10	2.54E-09
TSS1500	C1orf14	1	182923115/182923750/182923215	3	cg00379090/cg05969496/cg26419869	0.786517318	0.748356169	0.628802375	-0.157714943	8.06E-09	7.38E-08
TSS1500	C1orf144	1	16692150/16693079	2	cg01467503/cg10308193	0.503347975	0.392485006	0.3221469	-0.181201075	1.92E-12	2.06E-10
TSS1500	C1orf161	1	116653399/116653561/116653300/116653093	4	cg03847796/cg07577997/cg15469709/cg26678765	0.437585741	0.341161032	0.272080109	-0.165505632	4.18E-11	1.37E-09
TSS1500	C1orf56	1	151019134/151019560/151019727/151019274	4	cg04244851/cg11852073/cg13072446/cg23822289	0.853799177	0.731346567	0.640995742	-0.212803434	2.62E-07	1.48E-06
TSS1500	C1orf68	1	152691125/152691758	2	cg13927088/cg26420391	0.758410478	0.653446333	0.523588995	-0.234821482	1.49E-07	9.08E-07
TSS1500	C1QL2	2	119916686/119917385	2	cg12891160/cg20883684	0.38175445	0.493679667	0.623964523	0.242210073	1.53E-07	9.32E-07
TSS1500	C1QTNF5	11	119218027/119217884/119218238	3	cg10904867/cg16996281/cg19560971	0.499881308	0.387882361	0.274490673	-0.225390635	6.58E-13	1.11E-10

TSS1500	C1QTNF7	4	15428768/15374950/15375866/15428750/15375625	5	cg05003411/cg06742778/cg11290265/cg14547240/cg22686240	0.622566925	0.509714476	0.38269783	-0.239869095	8.43E-12	4.92E-10
TSS1500	C1QTNF8	16	1146445/1147214/1146545	3	cg01006126/cg01767885/cg02726898	0.659105105	0.576308583	0.497361834	-0.161743271	1.18E-07	7.34E-07
TSS1500	C1QTNF9B	13	24471857/24471371/24471776	3	cg02406148/cg03587877/cg22523074	0.681063265	0.604757046	0.527251362	-0.153811903	6.23E-08	4.20E-07
TSS1500	C20orf151	20	61003303/61002866/61002857	3	cg00334976/cg03359095/cg17976205	0.451815188	0.357156628	0.2750867	-0.176728488	2.81E-13	7.87E-11
TSS1500	C20orf160	20	30597590/30597694/30597664	3	cg09685763/cg11801374/cg12084436	0.635684392	0.520321391	0.442416745	-0.193267646	6.85E-10	1.02E-08
TSS1500	C20orf185	20	31642282/31641808/31642120	3	cg00549758/cg20926651/cg26111757	0.600620057	0.494519069	0.422745511	-0.177874546	2.91E-13	7.87E-11
TSS1500	C20orf186	20	31668141/31668448	2	cg03442592/cg05890956	0.749375975	0.709791322	0.547756936	-0.201619039	0.000103299	0.000304159
TSS1500	C20orf194	20	3388900/3389329/3389408/3389535/3388711	5	cg02893482/cg07023803/cg13516746/cg20681068/cg25506879	0.160570255	0.206632479	0.344387	0.183816744	0.000112862	0.000328844
TSS1500	C20orf195	20	62183964/62183924/62183966	3	cg15332132/cg20482390/cg26221801	0.642285345	0.537936661	0.466298805	-0.17598654	1.64E-06	7.33E-06
TSS1500	C20orf71	20	31804277/31804901/31803710	3	cg01216882/cg01671881/cg21142447	0.639549023	0.518190594	0.446516334	-0.193032688	6.69E-09	6.34E-08
TSS1500	C20orf79	20	18794097/18793817	2	cg08567760/cg08716736	0.69846479	0.54324435	0.404466973	-0.293997817	1.21E-10	2.81E-09
TSS1500	C20orf85	20	56724999/56725695/56725730	3	cg11800608/cg12813792/cg20003983	0.44296698	0.347821169	0.282244161	-0.160722819	1.38E-11	6.47E-10
TSS1500	C21orf34	21	17442467/17441953/17565263/17566381/17565450	5	cg10068793/cg10273072/cg10341719/cg10500737/cg22104103	0.61304077	0.530701658	0.456844374	-0.156196396	1.68E-12	2.01E-10
TSS1500	C21orf54	21	34543051/34542762/34543584	3	cg11056129/cg12077772/cg22109464	0.667533267	0.575913426	0.498208361	-0.169324906	3.42E-10	6.02E-09
TSS1500	C21orf96	21	36412119/36412456	2	cg05644223/cg16071713	0.336004293	0.214465603	0.16720432	-0.168799972	1.13E-12	1.54E-10
TSS1500	C2CD4A	15	62358751/62358531/62358920/62357710	4	cg00456672/cg04569583/cg19294311/cg27197276	0.59406607	0.504011655	0.439477812	-0.154588257	3.18E-10	5.71E-09

TSS1500	C2orf16	2	27798900/27798724/27798745/27798028	4	cg02621154/cg23055682/cg23058548/cg23761824	0.725657874	0.671330299	0.566360159	-0.159297715	7.04E-07	3.52E-06
TSS1500	C2orf71	2	29298362/29298474/29297717	3	cg06200607/cg20836546/cg21249563	0.642815665	0.504591417	0.402943227	-0.239872438	9.21E-13	1.38E-10
TSS1500	C3AR1	12	8220002/8219358	2	cg25757697/cg27423177	0.531033555	0.405832511	0.267374855	-0.2636587	5.15E-09	5.17E-08
TSS1500	C3orf14	3	62304554/62304779/62304629/62304165/62304470/62305003/62305147/62304264/62304515	9	cg01384143/cg01832005/cg02221302/cg07212940/cg11120913/cg15059608/cg22751696/cg25119986/cg26896572	0.420210023	0.465045378	0.57286856	0.152658537	1.23E-05	4.46E-05
TSS1500	C3orf22	3	126278020/126278943	2	cg02765820/cg07743179	0.6681791	0.603232244	0.460880923	-0.207298177	5.80E-07	2.97E-06
TSS1500	C4BPB	1	207262190	1	cg05659947	0.53585102	0.417912761	0.331053491	-0.204797529	8.63E-11	2.20E-09
TSS1500	C4orf26	4	76480681/76481053/76480905	3	cg00700728/cg03334213/cg05328761	0.44131937	0.372518822	0.289840555	-0.151478815	2.00E-08	1.59E-07
TSS1500	C4orf6	4	5525710/5526510	2	cg06887897/cg11561301	0.390493285	0.316697333	0.233324291	-0.157168994	1.40E-12	1.76E-10
TSS1500	C5AR1	19	47812519/47812780	2	cg02149446/cg23058911	0.539882465	0.458551483	0.360112323	-0.179770142	2.30E-09	2.67E-08
TSS1500	C5orf23	5	32788128/32788290/32788467	3	cg16434306/cg21494953/cg24363955	0.714513857	0.644690035	0.558314361	-0.156199496	2.06E-12	2.14E-10
TSS1500	C6orf10	6	32340354/32340955/32340208	3	cg00738945/cg25145195/cg26166145	0.845783275	0.708825333	0.630686055	-0.21509722	1.66E-07	9.97E-07
TSS1500	C6orf105	6	11779817/11779941/11779911	3	cg01129238/cg10787197/cg11210880	0.66519531	0.574712089	0.512996723	-0.152198587	4.46E-09	4.60E-08
TSS1500	C6orf122	6	170199193/170200021/170199865	3	cg08542199/cg15855251/cg19450119	0.527008092	0.436290974	0.332913965	-0.194094127	3.83E-13	8.04E-11
TSS1500	C6orf155	6	72131020/72130755/72130799/72130742/72130689	5	cg00201779/cg00920327/cg03318695/cg24772267/cg25141674	0.425418801	0.512862305	0.606534338	0.181115537	9.99E-05	0.000295242
TSS1500	C6orf173	6	126660872/126660984	2	cg20229609/cg25454804	0.531178393	0.395431	0.288893889	-0.242284504	3.73E-13	8.04E-11
TSS1500	C6orf176	6	166402699/166402638/166402416/166401921/166402081	5	cg04387240/cg06545143/cg08886973/cg12174276/cg21306006	0.227643649	0.27459121	0.385085798	0.157442149	2.57E-05	8.65E-05
TSS1500	C6orf222	6	36305071/36305155	2	cg04434994/cg18520925	0.521939075	0.389083867	0.299144136	-0.222794939	3.57E-11	1.23E-09

TSS1500	C7orf16	7	31726494/31725973/31725748	3	cg01001101/cg04866380/cg23348723	0.537798305	0.386587033	0.287442248	-0.250356057	8.22E-15	1.56E-11
TSS1500	C8orf4	8	40010631/40010443	2	cg13125627/cg17939444	0.428292015	0.355999136	0.26853297	-0.159759045	2.45E-09	2.82E-08
TSS1500	C9orf103	9	86237590/86236624	2	cg01266338/cg14219938	0.617831345	0.507959706	0.394851691	-0.222979654	1.45E-08	1.21E-07
TSS1500	C9orf125	9	104249936	1	cg05823563	0.56374433	0.45890055	0.382643477	-0.181100853	2.97E-09	3.30E-08
TSS1500	CA10	17	50236572/50237604/50237205/50237742/50237401/50237403/50236465/50237576/50236765/50237844/50237267/50236344/50237315	13	cg04881814/cg07111868/cg07398767/cg07723251/cg08605326/cg13125157/cg14056110/cg20552747/cg21328033/cg21385821/cg22855255/cg25039722/cg25592977	0.420112838	0.486412685	0.615639785	0.195526947	1.96E-09	2.34E-08
TSS1500	CA3	8	86350568/86350592/86350381/86350390/86349754/86350581/86350278/86349742	8	cg05355225/cg07560510/cg12264626/cg13721134/cg13958215/cg18674980/cg22289837/cg26461905	0.566545198	0.654038028	0.724864961	0.158319763	7.00E-11	1.88E-09
TSS1500	CACNA1A	19	13617549/13617584/13617518	3	cg03697708/cg12092090/cg27555529	0.155759768	0.231741004	0.366403472	0.210643703	1.26E-05	4.55E-05
TSS1500	CACNA1I	22	39966085/39965495/39965842/39965918	4	cg01726073/cg08800414/cg11138192/cg18070442	0.71943491	0.632944346	0.541885641	-0.177549269	1.78E-08	1.44E-07
TSS1500	CACNG3	16	24265703/24266347/24265847	3	cg04721098/cg07041488/cg16678467	0.62929972	0.522629275	0.450110909	-0.179188811	2.27E-10	4.42E-09
TSS1500	CACNG8	19	54465583/54465596	2	cg15786180/cg17866667	0.234210285	0.354253314	0.479799281	0.245588996	1.09E-05	4.00E-05
TSS1500	CAMK1G	1	209755781	1	cg04340324	0.758146105	0.663461994	0.537664268	-0.220481837	2.40E-08	1.86E-07
TSS1500	CAPN8	1	223854447/223854639/223854704/223853949/223853928	5	cg02313130/cg03543985/cg04147929/cg23251790/cg27235315	0.78770584	0.695189324	0.633208252	-0.154497588	2.60E-08	1.99E-07
TSS1500	CAPN9	1	230882959/230882481/230882809/230881857/230882603/230882387	6	cg03980366/cg07918814/cg12410939/cg17096979/cg25344142/cg27412093	0.540269695	0.426805161	0.34511362	-0.195156075	2.09E-10	4.17E-09
TSS1500	CAPZA3	12	18890579	1	cg25573386	0.473186035	0.343712533	0.266240668	-0.206945367	7.36E-10	1.08E-08
TSS1500	CASP14	19	15162623/15162515	2	cg01999333/cg02021789	0.58994144	0.492559689	0.359393386	-0.230548054	3.17E-09	3.46E-08

TSS1500	CASP5	11	104894514/104894216	2	cg08199529/cg10825847	0.661694598	0.518746817	0.463329532	-0.198365066	8.74E-12	5.02E-10
TSS1500	CATSPER1	11	65794191/65794352	2	cg01311045/cg14894216	0.724807238	0.641795083	0.537943775	-0.186863463	3.81E-08	2.75E-07
TSS1500	CBLC	19	45279765/45280184/45279770	3	cg03292149/cg14089474/cg21211730	0.57645109	0.468342774	0.393666445	-0.182784645	2.19E-10	4.32E-09
TSS1500	CCDC117	22	29168272/29168388	2	cg11501374/cg19953843	0.7903126	0.701347811	0.634316557	-0.155996043	2.94E-10	5.37E-09
TSS1500	CCDC13	3	42815903/42815945/42814948	3	cg09677287/cg20974621/cg27390245	0.815688973	0.702974219	0.63464778	-0.181041193	4.94E-10	7.90E-09
TSS1500	CCDC135	16	57727230/57728173/57727746	3	cg05859533/cg07540386/cg09418336	0.543786195	0.453630517	0.338720486	-0.205065709	3.18E-10	5.71E-09
TSS1500	CCDC140	2	223162049/223161771/223162128/223161894	4	cg00926215/cg02245378/cg08022524/cg08431536	0.347181433	0.430303367	0.575507773	0.228326339	2.85E-09	3.19E-08
TSS1500	CCDC141	2	179750458/179751100	2	cg12501949/cg26153162	0.505563165	0.380428744	0.289374618	-0.216188547	9.30E-12	5.15E-10
TSS1500	CCDC19	1	159870134/159870326/159870915/159870731	4	cg00099427/cg03685774/cg12486913/cg19805160	0.707626048	0.571555541	0.503196823	-0.204429226	3.91E-10	6.63E-09
TSS1500	CCDC50	3	191046547/191046380	2	cg19066520/cg25342125	0.60527054	0.410693117	0.355004173	-0.250266367	2.54E-10	4.82E-09
TSS1500	CCDC52	3	113235015/113234510	2	cg10517650/cg18753928	0.44128418	0.339668642	0.260373675	-0.180910505	1.66E-09	2.06E-08
TSS1500	CCDC90B	11	82997694/82997910/82997796	3	cg03000419/cg07748159/cg11933951	0.689672077	0.595661509	0.456476788	-0.233195289	5.76E-07	2.95E-06
TSS1500	CCK	3	42307866/42306974/42307761/42306737/42307799/42306852/42307193/42307519	8	cg10778780/cg11142705/cg12727529/cg17621718/cg18789547/cg23983435/cg25493589/cg25516803	0.426878942	0.497143631	0.594032686	0.167153744	5.59E-08	3.83E-07
TSS1500	CCKBR	11	6280583/6280512/6280183/6280531	4	cg05351257/cg13346411/cg26265234/cg26678978	0.202651899	0.228578226	0.3679965	0.165344601	0.000183692	0.000511501
TSS1500	CCL11	17	32611642/32612007/32611290	3	cg03297192/cg08499840/cg16764236	0.653360018	0.562948611	0.466525606	-0.186834412	2.34E-08	1.82E-07
TSS1500	CCL13	17	32683054/32682770	2	cg02706575/cg05281206	0.632535513	0.509425275	0.42220227	-0.210333242	3.63E-12	3.12E-10
TSS1500	CCL2	17	32581220/32581035/32581466/32580869	4	cg04633676/cg05440824/cg12698626/cg21109025	0.549400907	0.484713074	0.365588338	-0.183812569	3.51E-08	2.57E-07
TSS1500	CCL20	2	228678005	1	cg09425228	0.696894905	0.633821439	0.530765059	-0.166129846	8.97E-08	5.78E-07

TSS1500	CCL8	17	32645753/32645806/32645748	3	cg00038857/cg04656009/cg27000831	0.60098327	0.435627009	0.316505959	-0.284477311	1.61E-12	1.95E-10
TSS1500	CCNA1	13	37005566/37005074/37004617/37006063/37004721/37005570/37005299/37006127/37005115/37004812/37005557/37004857/37005582/37004715/37005501/37005489/37005107/37004737/37004601/37005035/37004853/37006107/37006116/37004536/37004940/37004747/37004787/37004990/37005289/37005273/37005562/37004620/37006133/37005452	34	cg00282249/cg00370179/cg01040523/cg02478448/cg03871460/cg05089090/cg05120331/cg05137358/cg06260815/cg07962128/cg08215918/cg08296176/cg08676975/cg11023392/cg11513637/cg12417071/cg13435392/cg13711394/cg14089714/cg14499563/cg17215831/cg18348647/cg18948722/cg19866195/cg21587066/cg21754388/cg23831735/cg24154053/cg24233119/cg24297049/cg24334591/cg26345046/cg26984343/cg27142924	0.336303202	0.466972428	0.62634131	0.290038107	1.06E-12	1.52E-10
TSS1500	CCR2	3	46394845/46394550	2	cg06280059/cg07743747	0.679691808	0.586713686	0.483398848	-0.19629296	2.35E-09	2.71E-08
TSS1500	CD109	6	74404526/74404879/74405048	3	cg00439709/cg23004174/cg25850808	0.719892365	0.643471176	0.552540255	-0.16735211	6.58E-10	9.83E-09
TSS1500	CD1B	1	158302740	1	cg12546793	0.35356663	0.227465033	0.163482	-0.19008463	1.88E-12	2.06E-10
TSS1500	CD1C	1	158258366/158259268/158258802	3	cg00173005/cg16993682/cg27534912	0.49024639	0.357822686	0.248668193	-0.241578197	1.02E-13	4.59E-11
TSS1500	CD2	1	117296494/117296242/117295987	3	cg10489672/cg16719404/cg21861305	0.641218083	0.510287603	0.416474898	-0.224743185	2.21E-08	1.73E-07
TSS1500	CD200R1L	3	112565651	1	cg18069403	0.846820095	0.749162411	0.652910945	-0.19390915	9.43E-06	3.49E-05
TSS1500	CD207	2	71063282/71064086	2	cg08989942/cg26946836	0.623148475	0.464274689	0.335575727	-0.287572748	9.23E-13	1.38E-10
TSS1500	CD3E	11	118174435/118174085	2	cg18085869/cg23369555	0.740834723	0.641928164	0.568956405	-0.171878318	5.86E-10	8.99E-09

TSS1500	CD40	20	44745522/44746392/44746681/44746655/44746499	5	cg01149415/cg09053081/cg17929951/cg19785066/cg19839655	0.299401668	0.394133619	0.502577614	0.203175946	1.03E-07	6.50E-07
TSS1500	CD53	1	111412880/111414364/111414984/111415232	4	cg06351337/cg07314983/cg10105218/cg10516886	0.574974268	0.498074156	0.392852312	-0.182121956	3.56E-08	2.59E-07
TSS1500	CD5L	1	157811905	1	cg23992209	0.767331115	0.641830617	0.540177964	-0.227153151	7.97E-08	5.21E-07
TSS1500	CD81	11	2397523/2397811/2397655/2397784/2397685/2398336/2397562/2397323/2397831/2398327/2397614/2397977/2398061/2397773/2398138/2397350/2397538/2397255/2397303/2397201/2397576/2397597/2397486	23	cg00319334/cg01492909/cg02078292/cg02249841/cg03848831/cg04069951/cg05002336/cg05102670/cg05490366/cg06699519/cg07111719/cg07804260/cg11705746/cg12845432/cg13894192/cg14022995/cg14398963/cg15289899/cg18409302/cg21843586/cg26053358/cg26819160/cg27055782	0.544657376	0.427742872	0.37531124	-0.169346137	3.16E-10	5.68E-09
TSS1500	CD86	3	121795768/121795811/121796071/121773564	4	cg00697440/cg06327732/cg09644952/cg11874272	0.48298169	0.412095972	0.332672009	-0.150309681	2.15E-09	2.54E-08
TSS1500	CDC14C	7	48963443/48963408	2	cg04527202/cg05563813	0.757763745	0.668332011	0.521815986	-0.235947759	1.69E-09	2.09E-08
TSS1500	CDH10	5	24645487/24645147	2	cg01058368/cg14730815	0.408848933	0.414785036	0.56782963	0.158980697	5.65E-06	2.21E-05
TSS1500	CDH12	5	22854704	1	cg15175266	0.489993885	0.386479383	0.290492182	-0.199501703	3.94E-12	3.27E-10
TSS1500	CDH19	18	64271503/64271469	2	cg13853450/cg15728909	0.747354578	0.626766872	0.47666073	-0.270693848	2.77E-12	2.69E-10
TSS1500	CDH2	18	25758159/25758430/25757710	3	cg02840109/cg12169536/cg14312538	0.172736437	0.206551427	0.351630767	0.17889433	2.78E-05	9.29E-05
TSS1500	CDH20	18	59156627	1	cg03319695	0.60413641	0.458245078	0.354302695	-0.249833715	8.07E-12	4.90E-10
TSS1500	CDH4	20	59827195/59826207/59827212	3	cg08290240/cg15534366/cg18092028	0.377546091	0.437289296	0.529764126	0.152218035	6.80E-08	4.53E-07
TSS1500	CDH8	16	62070306/62070365/62071193/62070246	4	cg01007828/cg04882273/cg05946623/cg13397057	0.449240438	0.520351579	0.620601956	0.171361518	3.02E-09	3.34E-08

TSS1500	CDK3	17	73996245/73996331/73996423/73996656	4	cg07471052/cg14040633/cg14666354/cg25711239	0.701718	0.556560665	0.481635856	-0.220082144	4.14E-09	4.32E-08
TSS1500	CDO1	5	115152938/115152785/115152835/115153223	4	cg04676799/cg07644368/cg16198692/cg23029474	0.57185475	0.693840264	0.779868445	0.208013695	1.64E-10	3.46E-09
TSS1500	CDX1	5	149545907/149545928/149546087/149546073	4	cg11524248/cg24216701/cg25132276/cg26531174	0.618906085	0.521917951	0.448638338	-0.170267748	8.82E-11	2.23E-09
TSS1500	CEACAM20	19	45033943/45034579/45034961	3	cg02904918/cg12497816/cg17384457	0.559394025	0.473612331	0.398190425	-0.1612036	3.74E-10	6.45E-09
TSS1500	CEACAM21	19	42081255/42082102/42082267	3	cg00921682/cg03745234/cg25528786	0.74048221	0.660517317	0.513324277	-0.227157933	2.20E-09	2.58E-08
TSS1500	CEACAM4	19	42134620/42134128/42134478	3	cg05891548/cg21049958/cg21529807	0.48040921	0.39834397	0.302570761	-0.177838449	1.39E-11	6.48E-10
TSS1500	CER1	9	14723496	1	cg01446692	0.585765655	0.524203822	0.388279041	-0.197486614	8.04E-07	3.96E-06
TSS1500	CFHR1	1	196787850	1	cg12687463	0.571536845	0.438112133	0.306298164	-0.265238681	6.66E-13	1.12E-10
TSS1500	CFHR2	1	196911888	1	cg09551916	0.581978375	0.451573889	0.314094859	-0.267883516	1.26E-12	1.65E-10
TSS1500	CFHR3	1	196743672	1	cg04614339	0.4525588	0.324182756	0.228356532	-0.224202268	2.05E-14	2.04E-11
TSS1500	CFHR4	1	196855851	1	cg11793499	0.78677561	0.6862508	0.510848623	-0.275926987	1.50E-09	1.90E-08
TSS1500	CFHR5	1	196946228/196945845/196945530	3	cg06952534/cg21475781/cg25840094	0.637010632	0.54514455	0.470782853	-0.166227779	2.49E-13	7.39E-11
TSS1500	CGA	6	87805033	1	cg18899818	0.68895494	0.581215422	0.465283977	-0.223670963	4.21E-11	1.37E-09
TSS1500	CGB8	19	49552606/49553743/49553761/49553788	4	cg04115602/cg16242238/cg19446264/cg27040708	0.591928043	0.499030172	0.42240017	-0.169527872	1.73E-13	6.30E-11
TSS1500	CHL1	3	238161/238392/238318/238048	4	cg00903242/cg01295518/cg04001014/cg11165881	0.319082383	0.331529224	0.563607323	0.244524939	1.10E-07	6.93E-07
TSS1500	CHMP4C	8	82644012/82643680/82644373	3	cg07583137/cg11253416/cg18584012	0.468662588	0.366355902	0.28652611	-0.182136478	1.22E-10	2.81E-09
TSS1500	CHRM2	7	136553595/136553110/136553243/136553327/136553289/136553170/136553778/136553263/136553682/136553088/136553728	11	cg00973677/cg02866106/cg04748704/cg05680531/cg09546907/cg10418044/cg10454514/cg17405012/cg22471517/cg25150440/cg25632105	0.313523998	0.387184012	0.502714533	0.189190535	1.92E-10	3.91E-09

TSS1500	CHRNA3	15	78914439/78914264/78914284/78914083	4	cg16570223/cg19993258/cg22670733/cg25527599	0.62403284	0.493439392	0.421273093	-0.202759747	5.07E-15	1.31E-11
TSS1500	CHRNA9	4	40337262/40336735/40336255	3	cg09632936/cg25190534/cg26531231	0.77104868	0.666162622	0.572421873	-0.198626807	7.58E-07	3.75E-06
TSS1500	CHST10	2	101034825/101035456	2	cg05352754/cg07618258	0.658533	0.579670811	0.486172264	-0.172360736	6.33E-11	1.75E-09
TSS1500	CHST15	10	125806958/125807504/125807047	3	cg06034255/cg09595290/cg16449489	0.719218533	0.531967692	0.380170368	-0.339048164	1.72E-12	2.01E-10
TSS1500	CHST2	3	142838082/142838320/142837252/142837472/142837529/142837995/142838003/142837564	8	cg07399369/cg10886442/cg11108893/cg17024542/cg19027710/cg19442495/cg19788429/cg21597025	0.333656144	0.374005157	0.485523315	0.151867171	4.74E-05	0.000150367
TSS1500	CHST5	16	75569527/75569852/75569459	3	cg03517083/cg03858460/cg27232078	0.60959881	0.589231594	0.396092173	-0.213506637	5.09E-08	3.54E-07
TSS1500	CIDEA	18	12254175/12254148/12254173/12253828/12254365/12254153/12253749/12254024/12254377/12254241/12253859/12254120	12	cg00851770/cg03245632/cg12072560/cg14976646/cg15063116/cg18309817/cg18649459/cg19883905/cg20950011/cg21376774/cg23754772/cg26974111	0.277911892	0.386769329	0.464986954	0.187075063	6.46E-06	2.49E-05
TSS1500	CLDN17	21	31540204	1	cg02825728	0.64226953	0.49235175	0.395951982	-0.246317548	5.76E-07	2.95E-06
TSS1500	CLDN4	7	73244836/73244073/73243736	3	cg10393875/cg14826417/cg22123387	0.464066585	0.364696317	0.276493168	-0.187573417	2.95E-12	2.76E-10
TSS1500	CLEC10A	17	6983991/6984187/6984106	3	cg01472101/cg04100395/cg21550483	0.504791538	0.433333797	0.352197964	-0.152593574	7.78E-09	7.18E-08
TSS1500	CLEC12B	12	10162301/10161757	2	cg00966659/cg26683917	0.715093493	0.635125956	0.541793627	-0.173299865	3.67E-10	6.36E-09
TSS1500	CLEC14A	14	38727014/38726189	2	cg05172197/cg22902799	0.667107875	0.551166833	0.402543582	-0.264564293	5.50E-09	5.42E-08
TSS1500	CLEC17A	19	14693303/14692759/14692863	3	cg15760345/cg25770495/cg25945273	0.788051465	0.714066522	0.636253455	-0.15179801	2.88E-05	9.58E-05
TSS1500	CLEC4E	12	8694468/8694216	2	cg14909213/cg27078893	0.675624578	0.594896872	0.519235289	-0.156389289	2.37E-11	9.12E-10
TSS1500	CLEC4G	19	7798327/7797355	2	cg05626117/cg21278129	0.545917773	0.435330403	0.357508673	-0.1884091	6.03E-12	4.11E-10

TSS1500	CLIP4	2	29337946/29338077/29337984/29337988/29338100	5	cg01777397/cg06333058/cg07285673/cg09695033/cg25737323	0.288922638	0.419389181	0.595234157	0.306311519	6.11E-09	5.89E-08
TSS1500	CLRN1	3	150691510/150663357/150691910/150662697/150692080	5	cg08153098/cg10625737/cg24365013/cg24721964/cg27322397	0.583617346	0.474174775	0.368864147	-0.2147532	2.93E-11	1.08E-09
TSS1500	CLSTN2	3	139653573/139653431/139653545/139653328	4	cg00134776/cg00355389/cg01755562/cg14498423	0.200825739	0.258721785	0.375305538	0.1744798	0.000100369	0.00029659
TSS1500	CLVS1	8	62199082	1	cg03263337	0.37187586	0.26334065	0.202037267	-0.169838593	5.49E-09	5.41E-08
TSS1500	CLVS2	6	123317124/123317184/123317212	3	cg01141812/cg01707818/cg21727532	0.28038844	0.377501308	0.470305495	0.189917055	4.80E-07	2.52E-06
TSS1500	CMA1	14	24978681/24977773	2	cg01201932/cg08020808	0.575881078	0.480491342	0.4033054	-0.172575678	3.52E-08	2.57E-07
TSS1500	CMTM2	16	66613053/66613096/66612955	3	cg01683883/cg08032924/cg09849405	0.270345705	0.400159912	0.603544868	0.333199163	7.17E-09	6.69E-08
TSS1500	CMTM3	16	66638320/66637927/66637919/66638293/66638210	5	cg00355909/cg01434608/cg16335762/cg23297477/cg26658728	0.173318059	0.470402982	0.643669665	0.470351607	2.29E-11	8.89E-10
TSS1500	CMTM8	3	32279261/32279948/32279722/32279236	4	cg01617750/cg02059034/cg08196359/cg20932822	0.544981118	0.4805895	0.386958943	-0.158022174	7.23E-10	1.07E-08
TSS1500	CNGB1	16	58005416/58006124	2	cg02259550/cg10624445	0.603636575	0.547212242	0.447105105	-0.15653147	1.05E-07	6.63E-07
TSS1500	CNRIP1	2	68548060/68547655	2	cg08174244/cg21216118	0.70666885	0.545216989	0.394946445	-0.311722405	9.27E-14	4.52E-11
TSS1500	CNTD1	17	40950290/40949954/40950545/40949811	4	cg21689613/cg22239334/cg23510443/cg24994625	0.446075058	0.351667614	0.278483134	-0.167591923	1.07E-11	5.52E-10
TSS1500	CNTNAP2	7	145812871/145813031/145813008/145813066/145812842	5	cg02024077/cg04706867/cg09571420/cg11642382/cg21322436	0.279447773	0.342891793	0.485223239	0.205775466	1.66E-05	5.85E-05
TSS1500	CNTNAP5	2	124782261/124782253/124782117/124782263/124782586	5	cg03696599/cg08523865/cg11712936/cg13358636/cg18582824	0.441849881	0.527821953	0.639774452	0.197924571	1.16E-10	2.73E-09
TSS1500	COG5	7	107205932/107205253/107205392	3	cg14157213/cg15381326/cg25298235	0.72002247	0.64611428	0.556304991	-0.163717479	9.38E-09	8.40E-08

TSS1500	COL24A1	1	86623101/86622395/86622552/86622737/86622628/86622624/86622682	7	cg07198150/cg09264282/cg13683218/cg21841239/cg21913319/cg24395801/cg24700222	0.276217379	0.309183967	0.439967317	0.163749938	0.000100751	0.000297301
TSS1500	COL28A1	7	7576090/7576351/7576792/7576116	4	cg07301377/cg19972068/cg21105443/cg22183456	0.76433848	0.629302379	0.597574568	-0.166763912	4.93E-09	4.98E-08
TSS1500	COL29A1	3	130063469/130063505	2	cg08536961/cg10592710	0.744895998	0.672279361	0.550071159	-0.194824838	1.04E-05	3.82E-05
TSS1500	COL4A1	13	110960198/110960942/110960186/110960177/110960563/110960452	6	cg15871127/cg19252199/cg22757824/cg24089600/cg25530246/cg27546237	0.269108451	0.348211675	0.491518032	0.222409581	1.39E-05	4.98E-05
TSS1500	COL4A2	13	110959356/110959428/110959104/110958418/110958977/110958892	6	cg02717061/cg06659727/cg13596497/cg15262516/cg21938148/cg26875813	0.287237584	0.378680973	0.477036666	0.189799083	9.59E-09	8.56E-08
TSS1500	COL6A6	3	130278864/130278187	2	cg14720996/cg15922045	0.783829105	0.704683022	0.598767891	-0.185061214	6.12E-07	3.11E-06
TSS1500	COMP	19	18902727/18902527/18902709	3	cg03767475/cg08177015/cg22865824	0.64956997	0.587861454	0.496676359	-0.152893611	2.95E-07	1.64E-06
TSS1500	COPS5	8	67975874/67975880/67975895	3	cg07896832/cg21320567/cg27247252	0.76009956	0.698727192	0.579640145	-0.180459415	9.69E-05	0.000287588
TSS1500	COQ3	6	99842368/99842351/99842353	3	cg03514381/cg18751133/cg24831427	0.59824407	0.442925756	0.349923117	-0.248320953	1.03E-10	2.49E-09
TSS1500	CORIN	4	47841314/47840683	2	cg02955940/cg11819342	0.76536497	0.643094467	0.611025223	-0.154339747	1.59E-07	9.62E-07
TSS1500	COX8C	14	93812936/93812051	2	cg01726287/cg20938103	0.722699625	0.592516594	0.458205541	-0.264494084	6.58E-10	9.83E-09
TSS1500	CP110	16	19533815/19534852	2	cg06784594/cg27259633	0.70235553	0.550445267	0.462975068	-0.239380462	1.39E-12	1.75E-10
TSS1500	CPA3	3	148581801/148581837	2	cg03020424/cg13424229	0.47613118	0.348653883	0.288507327	-0.187623853	3.32E-11	1.17E-09
TSS1500	CPSF4L	17	71258936/71258589/71259036	3	cg03101058/cg08813944/cg18911972	0.52308278	0.415520411	0.338294309	-0.184788471	1.35E-10	3.01E-09
TSS1500	CPZ	4	8593933/8593507/8593019/8593665	4	cg00535027/cg01105427/cg23283187/cg25706990	0.543058995	0.440476397	0.373526889	-0.169532106	3.25E-12	2.88E-10
TSS1500	CR2	1	207627372/207626214/207626386/207626535/207626345	5	cg05508558/cg07573078/cg10109747/cg14848620/cg23605027	0.696928943	0.613159381	0.531911851	-0.165017092	9.52E-09	8.51E-08
TSS1500	CRB1	1	197237119	1	cg13940125	0.59197059	0.525189533	0.402177927	-0.189792663	1.17E-07	7.29E-07

TSS1500	CRHBP	5	76247647/76248131/76247679	3	cg06495038/cg25913647/cg26196496	0.70588392	0.5585126	0.435769332	-0.270114588	2.31E-09	2.68E-08
TSS1500	CRHR2	7	30722433/30722359/30722361/30722643	4	cg02712145/cg13094036/cg14896516/cg15615793	0.286974268	0.375717207	0.492358127	0.205383859	2.55E-06	1.09E-05
TSS1500	CRIP1	14	105952171	1	cg06618474	0.5062954	0.39968705	0.337020305	-0.169275095	1.76E-11	7.71E-10
TSS1500	CRYAB	11	111783348/111783327/111783355/111783267/111783563/111783499/111783352	7	cg00514609/cg01158277/cg07476508/cg10048349/cg11694519/cg12947833/cg15204861	0.421133843	0.524254352	0.588867958	0.167734115	4.31E-09	4.46E-08
TSS1500	CRYGA	2	209029782/209029594/209029690	3	cg00139726/cg07313720/cg22386774	0.711410438	0.623585652	0.522424265	-0.188986173	1.71E-09	2.11E-08
TSS1500	CRYGD	2	208990238/208989579/208989831	3	cg09453636/cg10823322/cg11463113	0.479153925	0.405568461	0.324140325	-0.1550136	1.20E-08	1.04E-07
TSS1500	CSPP1	8	67975874/67975880/67975895	3	cg07896832/cg21320567/cg27247252	0.76009956	0.698727192	0.579640145	-0.180459415	9.69E-05	0.000287588
TSS1500	CST1	20	23732873/23732595	2	cg20206322/cg22981961	0.819557913	0.742785286	0.634607711	-0.184950201	9.27E-08	5.95E-07
TSS1500	CST2	20	23807778/23807736	2	cg21777364/cg25128583	0.500473875	0.398866633	0.305339382	-0.195134493	5.17E-11	1.57E-09
TSS1500	CST4	20	23671108/23670047/23670089	3	cg07707641/cg22238692/cg25965576	0.500733733	0.407772856	0.335448518	-0.165285215	1.86E-10	3.83E-09
TSS1500	CST6	11	65778967/65779192/65778210	3	cg02349785/cg15887846/cg19088912	0.566982552	0.47659958	0.402249852	-0.1647327	5.96E-09	5.79E-08
TSS1500	CST7	20	24929607	1	cg26450106	0.48901728	0.357791744	0.230002528	-0.259014752	3.93E-09	4.16E-08
TSS1500	CST8	20	23470461/23470274	2	cg11835442/cg11879776	0.626130803	0.492917139	0.395945152	-0.23018565	5.65E-11	1.65E-09
TSS1500	CST9L	20	23550668/23550446/23550632	3	cg01921212/cg02592239/cg11897887	0.692758855	0.636572872	0.54237625	-0.150382605	0.000184447	0.000513228
TSS1500	CTLA4	2	204731519/204731068/204731649	3	cg05092371/cg22572158/cg24077172	0.83561332	0.806892456	0.673246232	-0.162367088	0.000192291	0.000532555
TSS1500	CTNNA3	10	69456307/69456667/69425930/69426778	4	cg00132141/cg04141648/cg12768447/cg26297493	0.688468404	0.610107619	0.521445739	-0.167022665	1.09E-11	5.55E-10
TSS1500	CUBN	10	17172679/17172646	2	cg06098474/cg26276014	0.744350178	0.684790819	0.553881191	-0.190468987	4.33E-06	1.74E-05
TSS1500	CUEDC1	17	55981604/55981642/55982124	3	cg01667978/cg06723357/cg17779676	0.572390765	0.480803372	0.420474077	-0.151916688	3.61E-08	2.62E-07

TSS1500	CUX2	12	111471192/111471309/111470672/111471489/111471202/111470657	6	cg03223126/cg03352776/cg08710739/cg17190608/cg17420983/cg25752163	0.236283816	0.375212599	0.473772088	0.237488271	4.81E-06	1.91E-05
TSS1500	CXCL17	19	42947497/42948167/42948183	3	cg02831955/cg03003745/cg16512640	0.439352955	0.359269161	0.285777527	-0.153575428	2.59E-10	4.90E-09
TSS1500	CYP11A1	15	74658981/74659901/74659966/74660375/74660566/74661541	6	cg00615746/cg06285340/cg08033551/cg20330630/cg24482024/cg24849517	0.717838877	0.633770951	0.554720156	-0.163118721	3.37E-10	5.94E-09
TSS1500	CYP2E1	10	135340445/135340467/135340539	3	cg07381788/cg09208540/cg10986462	0.78078859	0.669798844	0.538530214	-0.242258376	6.23E-07	3.16E-06
TSS1500	CYP2F1	19	41619850/41618922/41619952	3	cg06210526/cg06579354/cg11267879	0.573804593	0.503680708	0.414374852	-0.15942974	4.12E-08	2.94E-07
TSS1500	CYP7A1	8	59413152/59413577	2	cg12010995/cg25634507	0.57989304	0.462345556	0.39455315	-0.18533989	1.21E-06	5.62E-06
TSS1500	CYP8B1	3	42918806/42918546/42919025/42918681	4	cg07147573/cg09430445/cg14404298/cg26124860	0.646690091	0.544080257	0.495478728	-0.151211363	8.21E-11	2.12E-09
TSS1500	CYTH3	7	6313050/6312525/6313058/6312471	4	cg08893109/cg11248491/cg20492034/cg21622555	0.470902514	0.383105812	0.313188644	-0.15771387	1.42E-10	3.12E-09
TSS1500	DAAM2	6	39759941/39760092/39760416/39759955/39759966	5	cg01499172/cg03698948/cg14135551/cg14436871/cg22174742	0.220202959	0.252259151	0.390301587	0.170098628	0.000272701	0.000731444
TSS1500	DAD1L	12	25072248/25071685	2	cg20620180/cg23656890	0.801447998	0.690498103	0.606599314	-0.194848684	2.60E-11	9.87E-10
TSS1500	DAPP1	4	100736658/100737138	2	cg07814567/cg08103568	0.533942873	0.442471456	0.362133732	-0.171809141	5.44E-10	8.51E-09
TSS1500	DBC1	9	122132103/122132178/122132052/122132379/122132054/122132261	6	cg03625109/cg13685314/cg14445814/cg14470803/cg24818566/cg25935911	0.370155049	0.44613189	0.623822819	0.25366777	9.62E-08	6.14E-07
TSS1500	DBH	9	136500855/136500234	2	cg21181594/cg25020204	0.552705915	0.460999194	0.379735759	-0.172970156	2.73E-11	1.02E-09
TSS1500	DBNL	7	44083715/44083518/44083650/44084030	4	cg01100525/cg12979234/cg22785672/cg26086607	0.542028373	0.412422661	0.32130347	-0.220724902	9.10E-11	2.29E-09

TSS1500	DBX1	11	20182644/20182695/20182122/20183267/20182324/20183057/20183008/20182575	8	cg03299487/cg06340987/cg10614045/cg15985775/cg18242712/cg19375210/cg22737282/cg24592027	0.285077934	0.347769263	0.477267793	0.192189859	2.26E-07	1.30E-06
TSS1500	DCD	12	55043559/55042383/55042893	3	cg01687040/cg15016120/cg16606256	0.58265091	0.486739959	0.383290977	-0.199359933	1.62E-11	7.24E-10
TSS1500	DCT	13	95133339/95132211	2	cg00777315/cg09189889	0.504490475	0.411465261	0.313827236	-0.190663239	1.21E-09	1.60E-08
TSS1500	DDIT4L	4	101111927/101111872/101112317	3	cg05319305/cg08663159/cg25071651	0.228163686	0.256218683	0.401769681	0.173605995	0.00032871	0.000867567
TSS1500	DEFA6	8	6783987	1	cg07974303	0.84784069	0.711118117	0.640231432	-0.207609258	4.37E-08	3.09E-07
TSS1500	DEFB104A	8	7693516/7333711/7332851/7332846	4	cg08528213/cg11604418/cg14839256/cg20223677	0.642295891	0.608098049	0.482084089	-0.160211802	6.22E-06	2.41E-05
TSS1500	DEFB104B	8	7693516/7333711/7332851/7332846	4	cg08528213/cg11604418/cg14839256/cg20223677	0.642295891	0.608098049	0.482084089	-0.160211802	6.22E-06	2.41E-05
TSS1500	DEFB118	20	29956062/29956028/29955259	3	cg03014957/cg14776195/cg19453059	0.608358398	0.499009114	0.400476609	-0.207881788	1.39E-09	1.78E-08
TSS1500	DEFB124	20	30061044/30062162/30061634	3	cg01981417/cg03529184/cg25412387	0.611096032	0.517505344	0.426677582	-0.18441845	5.29E-09	5.27E-08
TSS1500	DEFB129	20	207338/207161/207144	3	cg00769470/cg09592958/cg19931902	0.644156388	0.544957635	0.468910821	-0.175245567	2.25E-11	8.82E-10
TSS1500	DEFB132	20	237300/237002/238156	3	cg13151472/cg18011148/cg18239253	0.742723733	0.682388137	0.590867042	-0.151856691	1.75E-07	1.04E-06
TSS1500	DEFB135	8	11839138/11838532/11838843	3	cg01134551/cg08115957/cg12807628	0.289032305	0.176061816	0.129372896	-0.159659409	7.24E-13	1.19E-10
TSS1500	DEM1	1	40974057/40974132/40974110	3	cg04933954/cg04936970/cg20771287	0.083618576	0.097920608	0.238202498	0.154583922	0.003830373	0.008143136
TSS1500	DEPDC7	11	33036664/33037397/33036574	3	cg00035630/cg07491796/cg07675337	0.430156071	0.342611739	0.272290683	-0.157865389	1.54E-10	3.32E-09
TSS1500	DGKG	3	186080868/186080245/186080359/186080243	4	cg00434119/cg08675193/cg23517298/cg25487529	0.229783728	0.268326922	0.384716933	0.154933205	1.62E-05	5.70E-05
TSS1500	DGUOK	2	74153317/74153195/74153086/74153213	4	cg01629435/cg02478828/cg08971637/cg19752861	0.561847103	0.455869054	0.360101085	-0.201746018	3.99E-10	6.74E-09

TSS1500	DHRS7C	17	9695137/9696016/9694993	3	cg00352872/cg15476474/cg17444582	0.581065413	0.513199864	0.429859223	-0.15120619	3.26E-09	3.55E-08
TSS1500	DKFZp566F0947	19	31640194/31640249/31640401	3	cg12507030/cg20985037/cg23018111	0.692995605	0.610901078	0.538937891	-0.154057714	1.88E-08	1.51E-07
TSS1500	DKFZP686I15217	6	2986848/2987708/2986843	3	cg00184279/cg01454349/cg12335476	0.665292253	0.507222892	0.410613164	-0.254679089	8.22E-12	4.90E-10
TSS1500	DKK2	4	107958572/107958152/107957941/107958491	4	cg01404615/cg03504865/cg14129775/cg25262044	0.209778004	0.293982674	0.422676613	0.212898609	1.05E-06	4.99E-06
TSS1500	DLX3	17	48073423/48072833	2	cg00594561/cg06864796	0.58151512	0.534061883	0.420075095	-0.161440025	2.60E-08	1.99E-07
TSS1500	DNAH11	7	21582131/21582502/21582444	3	cg00016360/cg06270661/cg09447578	0.350061853	0.346009398	0.53481313	0.184751277	2.94E-05	9.77E-05
TSS1500	DNAH6	2	84743142/84743263/84743279	3	cg08026651/cg09321363/cg10929210	0.481577883	0.384332985	0.285897377	-0.195680506	2.80E-08	2.12E-07
TSS1500	DNAH8	6	38690157/38689670	2	cg04581728/cg04897208	0.532745265	0.403037156	0.360058005	-0.17268726	1.33E-07	8.20E-07
TSS1500	DNM1	9	130964374/130964918	2	cg00407062/cg14532894	0.561360988	0.438879403	0.37003195	-0.191329038	7.27E-14	3.94E-11
TSS1500	DNMT3L	21	45683302/45683528/45683146/45683513	4	cg09247319/cg27076046/cg27187798/cg27237537	0.756631602	0.694458174	0.588792827	-0.167838774	3.19E-07	1.76E-06
TSS1500	DOK6	18	67067721/67067893/67067261/67067867	4	cg05588496/cg12799689/cg24479776/cg27347269	0.346447457	0.428701092	0.563326977	0.21687952	3.62E-07	1.97E-06
TSS1500	DOPEY2	21	37536083/37535372/37536137	3	cg17946714/cg19413435/cg26080567	0.654718378	0.559995444	0.502873067	-0.151845312	8.79E-12	5.03E-10
TSS1500	DPM2	9	130701076	1	cg00921973	0.57796396	0.486989906	0.384054164	-0.193909796	8.12E-10	1.16E-08
TSS1500	DPP10	2	115919006/115919366/115918745/115199477/115919009	5	cg03753331/cg04617755/cg12666279/cg16146353/cg21678377	0.291421825	0.302930935	0.468908183	0.177486358	0.000401394	0.001041724
TSS1500	DPP4	2	162931397/162932048/162931638	3	cg11460364/cg20539283/cg23246095	0.54862809	0.410740283	0.315133877	-0.233494213	5.33E-11	1.60E-09
TSS1500	DPP6	7	153583318/153749206/153748818/154001891/154002064/153583867/153583563/153748370	8	cg00017489/cg00446413/cg04083575/cg06002683/cg10552126/cg18252309/cg19161124/cg25502399	0.427256819	0.508095605	0.620565627	0.193308808	2.98E-09	3.31E-08

TSS1500	DPY19L2	12	64062724/64062564/64063071	3	cg00845942/cg04455164/cg16971885	0.21846244	0.313160749	0.504428995	0.285966556	1.11E-08	9.69E-08
TSS1500	DRGX	10	50600265/50600744	2	cg10192713/cg17494438	0.63884408	0.546119314	0.468446602	-0.170397478	1.32E-09	1.71E-08
TSS1500	DSCR6	21	38378539/38377938/38378406/38378634	4	cg04965987/cg12564962/cg16345647/cg16886987	0.451410338	0.523641657	0.652791998	0.20138166	8.43E-10	1.19E-08
TSS1500	DSPP	4	88528262/88528331	2	cg03459617/cg21592690	0.62235408	0.492350011	0.405686	-0.21666808	5.07E-09	5.10E-08
TSS1500	DTX1	12	113495386/113494422/113494429/113495037	4	cg04562491/cg10670310/cg11939071/cg22685245	0.289817244	0.444244252	0.548626283	0.25880904	3.97E-09	4.18E-08
TSS1500	DUSP26	8	33458593/33457822	2	cg00441301/cg01869632	0.67953711	0.585778539	0.528670941	-0.150866169	2.60E-06	1.11E-05
TSS1500	DYDC1	10	82117089/82117119/82117257	3	cg03701427/cg06411551/cg17703212	0.341397575	0.543918117	0.676124495	0.33472692	4.04E-08	2.90E-07
TSS1500	DYM	18	46987532/46987786	2	cg12875426/cg23865597	0.73457248	0.706864103	0.575342257	-0.159230223	0.000122798	0.000354828
TSS1500	DZIP1	13	96297197/96297338/96297224/96297281	4	cg04101379/cg05074631/cg17922851/cg21627412	0.303140255	0.349764267	0.460832659	0.157692404	0.000359912	0.000943359
TSS1500	EBF1	5	158527817/158527707/158527702/158527982/158527469/158528040/158527573/158527463/158527458	9	cg00251610/cg01135780/cg05056120/cg05774756/cg07607126/cg15963563/cg16126280/cg16317459/cg26664797	0.140235014	0.223235776	0.402209818	0.261974803	4.35E-07	2.30E-06
TSS1500	EBPL	13	50265850/50265991/50266227	3	cg03658251/cg04663916/cg20399252	0.465668185	0.358645097	0.275350452	-0.190317733	3.55E-11	1.23E-09
TSS1500	ECH1	19	39323158/39323060/39322968	3	cg00204262/cg07859880/cg13659813	0.435535168	0.326156108	0.274216921	-0.161318247	1.29E-08	1.10E-07

TSS1500	EDNRB	13	78493182/78493657/78493561/78550939/78493704/78494064/78493878/78494330/78493651/78494010/78493313/78494272/78550373/78494442/78550384/78493294/78493349/78493590/78493920/78493305/78551078/78493854/78493229/78493712/78494171/78493297/78493365/78494067/78493203/78493874/78493564/78550069/78493232/78493666/78493671/78493215/78493205/78493282/78493958/78494462/78493631/78493583	42	cg01910869/cg02147695/cg06057566/cg06106063/cg07035515/cg07974719/cg08321129/cg08634041/cg09152886/cg09786383/cg10016380/cg10764762/cg10792120/cg11074192/cg12321193/cg12602112/cg12847373/cg12935136/cg12983394/cg13434989/cg13937208/cg16739796/cg18032190/cg18568990/cg19111971/cg19650157/cg19742055/cg19759502/cg19916212/cg21364111/cg21675115/cg22154447/cg22310279/cg22541679/cg23494140/cg23702615/cg23766591/cg24236409/cg24745738/cg24942919/cg25717994/cg26022015	0.381633288	0.455473798	0.542449532	0.160816244	8.83E-09	7.96E-08
TSS1500	EFHA2	8	16884225/16884197/16884364	3	cg03919781/cg21653826/cg23218760	0.179144757	0.265004541	0.489821532	0.310676775	4.01E-07	2.15E-06
TSS1500	EGFL7	9	139556554	1	cg05037688	0.54490394	0.462024394	0.392028123	-0.152875817	2.26E-06	9.79E-06
TSS1500	EGOT	3	4794061/4794082/4794020	3	cg02799411/cg05795849/cg11600734	0.435363745	0.320693406	0.247763118	-0.187600627	4.33E-11	1.40E-09
TSS1500	EHD3	2	31456217/31455890/31455443	3	cg00648955/cg12045528/cg13149833	0.713891333	0.637924717	0.541761407	-0.172129926	1.33E-08	1.12E-07
TSS1500	EHF	11	34641925/34642463/34642303	3	cg05503887/cg13084525/cg14022090	0.445937695	0.336296294	0.271712023	-0.174225672	8.21E-12	4.90E-10
TSS1500	EID3	12	104697193/104697220/104697224	3	cg01857475/cg09884423/cg10572274	0.247947366	0.421289865	0.607087922	0.359140557	7.30E-10	1.07E-08

TSS1500	EIF3G	19	10231566/10231711/10231701	3	cg05328461/cg19766988/cg26826183	0.558072095	0.435691902	0.32501452	-0.233057575	3.49E-13	7.96E-11
TSS1500	EIF3K	19	39109076/39109456/39108723/39108529/39108609/39108672	6	cg00233307/cg02306730/cg04292549/cg15679651/cg19457477/cg24337786	0.452079576	0.551410713	0.614407209	0.162327633	5.37E-08	3.70E-07
TSS1500	EIF5AL1	10	81271195/81271276/81271491	3	cg13429435/cg16836040/cg22529822	0.786189702	0.717451826	0.631736645	-0.154453056	4.22E-10	7.00E-09
TSS1500	ELF1	13	41556827/41594904/41594509	3	cg11793332/cg12691679/cg25878131	0.615990328	0.539245564	0.462670591	-0.153319737	1.11E-09	1.50E-08
TSS1500	ELTD1	1	79473783/79472917	2	cg07692939/cg17750919	0.76251754	0.653689556	0.589304036	-0.173213504	3.15E-05	0.000103915
TSS1500	EMCN	4	101440278/101440020/101439954	3	cg10512376/cg12532667/cg24807169	0.514521912	0.408220706	0.301690146	-0.212831766	5.10E-10	8.10E-09
TSS1500	EMR1	19	6886962	1	cg22889448	0.47911394	0.398218067	0.300828236	-0.178285704	5.96E-10	9.11E-09
TSS1500	EMR4P	19	6991872	1	cg22840583	0.83419752	0.780525883	0.682766168	-0.151431352	8.48E-05	0.000254395
TSS1500	ENTHD1	22	40290976/40290752	2	cg00044729/cg10658197	0.749997188	0.689050206	0.595833443	-0.154163744	4.72E-06	1.88E-05
TSS1500	EOMES	3	27764473/27764660/27765232/27765283/27764810/27764816	6	cg01714811/cg02694810/cg06014401/cg15540820/cg16971668/cg22383888	0.27810847	0.367711204	0.52080839	0.24269992	1.60E-07	9.68E-07
TSS1500	EPHA3	3	89156118/89156071	2	cg01360628/cg16797972	0.3478503	0.254859844	0.19639241	-0.15145789	6.72E-08	4.50E-07
TSS1500	EPHA7	6	94129611/94129627/94129582/94129636/94129697	5	cg02168857/cg06740629/cg08001895/cg21552709/cg22263131	0.186532958	0.25859245	0.4403509	0.253817942	3.71E-05	0.000120385
TSS1500	EPHX3	19	15345307/15343786/15344364/15344186/15344239/15344046	6	cg05249937/cg08457898/cg18366919/cg21595709/cg24745495/cg26010734	0.373039106	0.439630137	0.564532494	0.191493388	6.99E-08	4.64E-07
TSS1500	EPPK1	8	144948315/144948764/144948781	3	cg04904458/cg06009267/cg06668829	0.590361175	0.483683683	0.416666252	-0.173694923	6.37E-10	9.62E-09
TSS1500	EREG	4	75229506/75230615/75230391	3	cg08649765/cg11646192/cg19308222	0.56094542	0.687410693	0.720377383	0.159431963	6.88E-09	6.46E-08
TSS1500	ESPNL	2	239007828/239007919/239008679	3	cg04003327/cg06574296/cg21395812	0.650867595	0.556416356	0.489510782	-0.161356813	6.45E-08	4.34E-07

TSS1500	ETNK1	12	22777790/22777465/22777820/22777409/22777430	5	cg02550949/cg02723291/cg11204099/cg17839399/cg21477262	0.525872365	0.41147782	0.335001012	-0.190871353	2.86E-07	1.60E-06
TSS1500	ETV3L	1	157071076/157070675/157070745/157070730	4	cg10956811/cg13981120/cg22023197/cg24681898	0.799147855	0.740744667	0.642373186	-0.156774669	1.21E-06	5.62E-06
TSS1500	EVC	4	5712581/5712676/5712592	3	cg03881544/cg15305511/cg26875219	0.215831775	0.277033162	0.449048542	0.233216767	3.50E-05	0.000114069
TSS1500	EVC2	4	5710767/5712581/5711301/5712676/5710761/5712592	6	cg03278643/cg03881544/cg09558315/cg15305511/cg17242664/cg26875219	0.215831775	0.277033162	0.449048542	0.233216767	3.50E-05	0.000114069
TSS1500	EVI2B	17	29641333	1	cg05109049	0.367865207	0.149288223	0.121072471	-0.246792736	3.31E-07	1.82E-06
TSS1500	EVI5	1	93258319/93258288/93258908/93258316	4	cg00519208/cg04982834/cg10989326/cg24699699	0.636497564	0.544050161	0.472102786	-0.164394777	8.36E-10	1.19E-08
TSS1500	EXD3	9	140318662/140318649/140318854	3	cg03383434/cg04837071/cg14435671	0.503200503	0.395513056	0.313456621	-0.189743882	6.08E-11	1.71E-09
TSS1500	EXOC1	4	56718320/56718365/56719469	3	cg18463607/cg20618651/cg26329992	0.667689465	0.543687509	0.472212618	-0.195476847	1.15E-11	5.74E-10
TSS1500	EXPH5	11	108410088/108464931/108409825/108464736/108409365/108423292/108423325	7	cg03062551/cg10022155/cg11340537/cg20577535/cg20929568/cg23322223/cg23755074	0.563015386	0.463764704	0.394688456	-0.16832693	1.67E-13	6.23E-11

TSS1500	EYA4	6	133562275/133561716/133562101/133562269/133562035/133562196/133561630/133561765/133561368/133561898/133562246/133561886/133561649/133562193/133562056/133561614/133561224/133561647/133562267/133561932/133561866/133562258/133561660/133561677/133561756/133561386/133561719/133562087/133561814	29	cg01162672/cg01957732/cg02446647/cg05062333/cg05493394/cg06181518/cg06525347/cg07327468/cg08885224/cg10675276/cg11518846/cg12152919/cg13912545/cg14017655/cg14343214/cg15729404/cg17348951/cg17985029/cg20286200/cg20330472/cg20611276/cg20980055/cg21296676/cg21390624/cg22462983/cg24535176/cg24842760/cg26449787/cg26501369	0.298127576	0.337609117	0.452043916	0.15391634	0.000802498	0.001954384
TSS1500	F10	13	113776252/113776873/113776126	3	cg21367957/cg21714557/cg26608199	0.74022244	0.634144169	0.564456584	-0.175765856	2.51E-09	2.87E-08
TSS1500	F11R	1	160992358/160992352/160991549	3	cg01423643/cg05754905/cg20012024	0.646706065	0.567873411	0.485685563	-0.161020502	4.99E-09	5.04E-08
TSS1500	F13B	1	197037504/197037732	2	cg01488306/cg07839513	0.6148797	0.559666358	0.460119668	-0.154760032	9.51E-08	6.08E-07
TSS1500	FAAH	1	46859671/46858748/46859680	3	cg06911238/cg15198148/cg19921581	0.59004246	0.468175033	0.365547773	-0.224494687	9.20E-12	5.15E-10
TSS1500	FABP7	6	123099534/123100189	2	cg18555555/cg21020855	0.61922	0.51319515	0.4618227	-0.1573973	3.19E-08	2.36E-07
TSS1500	FADS2	11	61595494/61594997/61594965/61595485	4	cg05698098/cg06781209/cg21709803/cg23760165	0.159061191	0.182416376	0.386897281	0.22783609	0.0001129	0.000328855
TSS1500	FAM129A	1	184944785/184944153/184944109/184943911/184944288	5	cg12141056/cg18166655/cg19255053/cg24325991/cg25182523	0.566982912	0.501678851	0.407351065	-0.159631847	1.03E-09	1.42E-08
TSS1500	FAM150A	8	53478624/53478454/53478706	3	cg05965392/cg17162024/cg26021627	0.23671131	0.229776212	0.400791592	0.164080282	0.002115172	0.004734373

TSS1500	FAM155A	13	108520039/108520481/108519752/108520391/108520189/108520252/108520827/108520945/108520566/108519880	10	cg03141482/cg05905988/cg06446668/cg06833978/cg07034928/cg13582072/cg18588768/cg22302985/cg24567424/cg25985659	0.25719381	0.285549024	0.451570455	0.194376645	3.32E-05	0.000108905
TSS1500	FAM159B	5	63985377/63985489/63985282/63985374	4	cg00811565/cg17035996/cg18909711/cg27637940	0.719465798	0.624303389	0.483779925	-0.235685873	1.23E-10	2.83E-09
TSS1500	FAM179A	2	29202812/29202836/29203230/29203014	4	cg02182081/cg04026326/cg16944977/cg25553279	0.549097928	0.439609308	0.376706627	-0.1723913	3.39E-11	1.19E-09
TSS1500	FAM184B	4	17783936/17783356/17783502	3	cg00306311/cg02816816/cg07696699	0.391798968	0.4544131	0.596469302	0.204670335	2.83E-07	1.58E-06
TSS1500	FAM188B	7	30810183/30810504	2	cg02543636/cg27070397	0.738768665	0.613962272	0.476933505	-0.26183516	5.15E-10	8.14E-09
TSS1500	FAM18A	16	10913854/10913704/10913597	3	cg04482884/cg26636054/cg27584980	0.803846268	0.724877311	0.6450552	-0.158791068	1.59E-09	1.98E-08
TSS1500	FAM19A4	3	68982098/68981956/68982064	3	cg03427298/cg05893614/cg08038353	0.428240753	0.465134535	0.607088792	0.178848039	1.33E-07	8.19E-07
TSS1500	FAM19A5	22	48970993/48970871/48884463/48884960/48884945/48884885/48884089/48971051	8	cg02895639/cg02905830/cg03196739/cg04304705/cg14894848/cg23936463/cg24343361/cg25975712	0.330864584	0.381474192	0.494202275	0.163337691	1.01E-07	6.40E-07
TSS1500	FAM38B	18	11149841/11149435/11149470/11149763/11149068/11150043	6	cg02664349/cg03117976/cg03686593/cg06510438/cg10567810/cg12673429	0.341067111	0.492337197	0.61288286	0.271815749	2.66E-08	2.03E-07
TSS1500	FAM3D	3	58653593/58652897/58653196	3	cg02658330/cg21770617/cg26334888	0.48546966	0.400412975	0.327898852	-0.157570808	1.40E-10	3.09E-09
TSS1500	FAM55B	11	114548759/114548872	2	cg07340922/cg19113081	0.645004755	0.482875078	0.366004345	-0.27900041	2.73E-12	2.67E-10
TSS1500	FAM55D	11	114467115/114466865	2	cg22223402/cg27230999	0.515671985	0.416182611	0.35739035	-0.158281635	2.19E-08	1.72E-07
TSS1500	FAM5B	1	177140145/177140203/177140322/177140126/177140305/177139876	6	cg04209911/cg06758591/cg13685951/cg13843613/cg20846447/cg22371227	0.200418514	0.275819626	0.410171494	0.20975298	5.59E-07	2.87E-06
TSS1500	FAM71C	12	100041071	1	cg14893473	0.696000595	0.610819911	0.501094759	-0.194905836	5.41E-06	2.12E-05

TSS1500	FAM71E2	19	55874851/55876060/55875605	3	cg01532284/cg16737991/cg26367719	0.73459156	0.642271446	0.572653405	-0.161938155	3.42E-15	1.30E-11
TSS1500	FAM71F1	7	128354967/128354965/128354862	3	cg10157098/cg12560447/cg21351241	0.596234335	0.517858906	0.412902732	-0.183331603	2.73E-08	2.08E-07
TSS1500	FAM72A	1	206137774/206137767/206137759/206138116/206138190	5	cg05122861/cg09315918/cg10439914/cg20259129/cg23089764	0.099334954	0.198036057	0.316157556	0.216822601	1.36E-08	1.15E-07
TSS1500	FAM72B	1	120838644/120837955/120838320/120838718/120838323/120838387/120838445	7	cg00183340/cg02905065/cg07784526/cg08728856/cg18948743/cg22493809/cg25103766	0.118222883	0.211454706	0.350380295	0.232157412	1.47E-09	1.87E-08
TSS1500	FAM72D	1	143913847/143913418/143913409/143913569/143913552	5	cg04968835/cg07344025/cg16903605/cg19895380/cg22710840	0.128562783	0.201122905	0.316391145	0.187828363	1.74E-09	2.14E-08
TSS1500	FAM74A3	9	40714141	1	cg21009400	0.61727257	0.519415711	0.416773918	-0.200498652	1.21E-09	1.60E-08
TSS1500	FAM75A6	9	43631161	1	cg10034990	0.530349525	0.371002656	0.276844323	-0.253505202	1.41E-13	5.41E-11
TSS1500	FAM81B	5	94726091/94726815/94726774	3	cg03304472/cg07721901/cg23360388	0.684618213	0.599003541	0.530422456	-0.154195757	9.35E-09	8.38E-08
TSS1500	FAM83E	19	49118114/49118165	2	cg00846121/cg04177684	0.425278483	0.344719828	0.274001845	-0.151276637	7.01E-11	1.88E-09
TSS1500	FAM92A3	4	183958214/183957914/183957644	3	cg01615271/cg19843540/cg25072945	0.701164178	0.642677854	0.547825844	-0.153338334	9.36E-07	4.51E-06
TSS1500	FAP	2	163101493/163100512	2	cg03591467/cg20838429	0.656710718	0.555828358	0.489021659	-0.167689058	1.19E-09	1.58E-08
TSS1500	FASLG	1	172627039	1	cg01880648	0.81637039	0.767162278	0.662785045	-0.153585345	5.78E-05	0.000179007
TSS1500	FAT2	5	150949152/150948901/150950000	3	cg09801065/cg11356004/cg26090075	0.547446783	0.438098881	0.378423395	-0.169023387	1.10E-08	9.61E-08
TSS1500	FBN2	5	127874478/127874587/127873980/127874463/127875163/127874466/127874825	7	cg01939274/cg05686497/cg06993118/cg11965976/cg20708842/cg25532099/cg27223047	0.233092783	0.267891083	0.458669824	0.225577041	4.52E-05	0.000144079
TSS1500	FBXO40	3	121311075/121310923	2	cg09824889/cg14974147	0.82601353	0.757520125	0.643227477	-0.182786053	3.94E-07	2.12E-06
TSS1500	FCGBP	19	40441647/40441469/40440963/40441611	4	cg03747345/cg17842966/cg19103704/cg22509205	0.727675924	0.653342744	0.56820904	-0.159466884	1.14E-08	9.91E-08
TSS1500	FCGR3B	1	161602335/161601721/161602002	3	cg19234439/cg22504013/cg26561570	0.646229915	0.506448889	0.371795414	-0.274434501	3.17E-12	2.85E-10

TSS1500	FCN2	9	137771506	1	cg26164184	0.75138032	0.619819144	0.498994923	-0.252385397	3.40E-08	2.50E-07
TSS1500	FCRL1	1	157790499/157790434	2	cg22515937/cg24344394	0.82022193	0.743530061	0.638941868	-0.181280062	2.42E-07	1.39E-06
TSS1500	FCRL3	1	157671386/157671759/157670869/157672074	4	cg05384663/cg15202552/cg19602479/cg24881910	0.730430815	0.608402315	0.521511495	-0.20891932	1.39E-08	1.17E-07
TSS1500	FCRLB	1	161691911/161691663/161691014/161692247	4	cg00160981/cg17036419/cg22678092/cg26015133	0.707141973	0.596650057	0.519515608	-0.187626366	7.20E-10	1.07E-08
TSS1500	FERD3L	7	19185260/19185407/19185267	3	cg07270153/cg10043037/cg23054426	0.235090854	0.267454665	0.413280798	0.178189944	4.90E-06	1.94E-05
TSS1500	FEV	2	219851181	1	cg18664514	0.310611665	0.235495761	0.150289331	-0.160322334	6.50E-10	9.75E-09
TSS1500	FEZ2	2	36825736/36825888/36825704	3	cg01206211/cg07416916/cg09467607	0.631230878	0.55758738	0.458600571	-0.172630307	1.87E-07	1.11E-06
TSS1500	FEZF2	3	62360464/62359420/62360240/62360674/62359773/62359924/62359677/62360417/62359390/62359392/62359456/62360162	12	cg04927889/cg06423920/cg06707343/cg06819546/cg08252579/cg08681924/cg11374425/cg14261840/cg15812348/cg19227130/cg24705960/cg27564792	0.229878165	0.309906044	0.437997543	0.208119379	1.02E-06	4.86E-06
TSS1500	FFAR3	19	35849024/35849176/35848801	3	cg01047748/cg15391601/cg21624359	0.692702305	0.561318139	0.463317859	-0.229384446	1.22E-08	1.05E-07
TSS1500	FGF10	5	44389769/44389406/44389062/44389848/44390215/44389282	6	cg06850283/cg08976810/cg09792008/cg13996155/cg16536718/cg20387341	0.355421997	0.4201884	0.535410554	0.179988557	3.14E-08	2.34E-07
TSS1500	FGF12	3	192127991/192445594/192127457/192446883/192127327/192127330/192127356	7	cg01255414/cg03401266/cg08002883/cg09725157/cg10511904/cg16376000/cg17393267	0.330327825	0.472380506	0.604079386	0.27375156	3.08E-11	1.11E-09
TSS1500	FGF14	13	103055487/103054743/102569538/103054846/102570476/102569894/102570482/103054752/103054860/102569876/102569815	11	cg00273543/cg04046599/cg05210258/cg06960698/cg11897909/cg15972331/cg17810588/cg21448145/cg21513836/cg23809442/cg27134816	0.231418779	0.293870246	0.409459493	0.178040714	7.48E-09	6.94E-08

TSS1500	FGF2	4	123747634/123747558/123747542/123747533/123747447	5	cg03941587/cg11327857/cg17214107/cg21550024/cg24683623	0.56496432	0.67285939	0.732034869	0.167070549	1.82E-11	7.71E-10
TSS1500	FGF5	4	81186795/81187125/81187011/81187198/81186901	5	cg05894124/cg10218799/cg12087412/cg18638496/cg21477508	0.166089672	0.211075347	0.431028501	0.26493883	1.97E-07	1.16E-06
TSS1500	FGF7	15	49713977	1	cg27039983	0.79558028	0.689933628	0.569927141	-0.225653139	4.47E-07	2.36E-06
TSS1500	FGF8	10	103536348/103536063/103536342	3	cg06169131/cg16248277/cg25209842	0.311807978	0.442923196	0.550929223	0.239121244	1.34E-08	1.13E-07
TSS1500	FGFBP3	10	93669852	1	cg02631917	0.785741315	0.695652361	0.616623991	-0.169117324	9.84E-06	3.63E-05
TSS1500	FHAD1	1	15573457/15573566/15573050/15573525	4	cg00524963/cg02394978/cg13234882/cg17750038	0.666546743	0.584749344	0.510991039	-0.155555704	1.65E-08	1.35E-07
TSS1500	FHL5	6	97009594/97010071	2	cg15802639/cg20719439	0.68488086	0.600364925	0.462790959	-0.222089901	1.30E-10	2.93E-09
TSS1500	FKBPL	6	32099450/32099564/32098344/32098346	4	cg03514937/cg07790778/cg08577517/cg16437896	0.692362945	0.529541622	0.446631627	-0.245731318	1.40E-09	1.79E-08
TSS1500	FKSG83	6	27291585/27291363/27292364	3	cg04988917/cg07968979/cg09240001	0.68586384	0.5926793	0.523708627	-0.162155213	0.000290136	0.000773833
TSS1500	FLG	1	152299041/152298884/152299078	3	cg10500702/cg13447818/cg26390526	0.74868366	0.658341672	0.5224244	-0.22625926	9.07E-09	8.15E-08
TSS1500	FLG2	1	152333922/152333423/152333596	3	cg03439811/cg03957898/cg23812489	0.48509233	0.334225467	0.227919445	-0.257172885	7.77E-10	1.12E-08
TSS1500	FLJ20184	4	106472990/106473040/106473233	3	cg08970682/cg14020824/cg25370441	0.442739302	0.365685204	0.28381527	-0.158924032	1.37E-09	1.77E-08
TSS1500	FLJ25758	19	7007910/7007104	2	cg01993907/cg27270214	0.654019365	0.523425708	0.398617102	-0.255402263	1.92E-11	8.02E-10
TSS1500	FLJ33360	5	6338418/6338017/6338529/6338794	4	cg02202411/cg02328035/cg17491560/cg22778952	0.7507706	0.694758528	0.477771682	-0.272998918	1.85E-08	1.49E-07
TSS1500	FLJ37307	13	52419714/52419689	2	cg16096766/cg18997137	0.490066513	0.389937956	0.307269466	-0.182797047	1.05E-12	1.52E-10
TSS1500	FLJ41562	4	69084723	1	cg23645302	0.49640466	0.398810878	0.319981855	-0.176422805	7.88E-09	7.25E-08
TSS1500	FLJ45079	17	75880542/75881093/75881232	3	cg04837616/cg09889477/cg11598872	0.595361763	0.499064048	0.413521735	-0.181840028	4.13E-09	4.32E-08
TSS1500	FLJ46361	10	124515334/124514711	2	cg10038490/cg24310126	0.69962434	0.630215064	0.51887257	-0.18075177	5.23E-07	2.71E-06

TSS1500	FLRT2	14	85996202/85995726/85996260/85995859/85996274/85995655/85996101	7	cg02305242/cg10406690/cg12080751/cg12155356/cg18845692/cg23934295/cg26651233	0.218268422	0.301255612	0.465920785	0.247652364	9.41E-08	6.03E-07
TSS1500	FMO9P	1	166571691/166572716	2	cg24117017/cg24272559	0.61617929	0.515784328	0.452963773	-0.163215517	0.000766794	0.001874648
TSS1500	FN3KRP	17	80673184/80674221/80673958/80674189/80673675	5	cg11026808/cg11954715/cg14688272/cg21624854/cg23522895	0.733421112	0.640418881	0.551613855	-0.181807257	1.05E-10	2.53E-09
TSS1500	FNDC1	6	159589874/159589483/159590059/159590155	4	cg08397344/cg08631819/cg09107912/cg09375620	0.263319685	0.299042562	0.425179148	0.161859463	0.000100507	0.000296766
TSS1500	FOXC2	16	86599976/86599468/86600499/86600309/86600326/86599745/86599714/86600389	8	cg01296593/cg02187214/cg02943578/cg07249459/cg09360770/cg10448808/cg23096689/cg24063470	0.277226397	0.325394744	0.474970348	0.197743951	3.08E-06	1.29E-05
TSS1500	FOXE1	9	100615244/100614879/100615084/100615201	4	cg02157015/cg13564742/cg13827859/cg15802898	0.214906266	0.291284665	0.413989536	0.199083271	4.26E-07	2.27E-06
TSS1500	FOXG1	14	29236008/29236017/29234981/29235973/29235448/29235092/29236013/29235376/29235907/29235196/29235928/29235984/29235904/29235469/29235123/29234978/29235894/29235193/29234890/29234904/29236034	21	cg02681442/cg02991338/cg03523785/cg04525757/cg07150445/cg07974473/cg08120263/cg09231862/cg10912240/cg16038120/cg18299578/cg19714132/cg19786627/cg21923619/cg23191956/cg23571812/cg24981833/cg25078444/cg26194477/cg26206396/cg27006650	0.311992748	0.354651158	0.500559358	0.18856661	3.77E-05	0.000121974
TSS1500	FOXI1	5	169532702/169532686/169531595	3	cg01106114/cg08560074/cg21503476	0.613300032	0.511036241	0.460781186	-0.152518845	2.08E-10	4.15E-09

TSS1500	FOXI2	10	129534182/129534613/129535138/129534411/129534217/129534722/129534107/129535186	8	cg00405688/cg02523640/cg04617948/cg06213468/cg18047662/cg24826696/cg25043050/cg25539045	0.376623567	0.445151151	0.551313015	0.174689448	4.68E-06	1.87E-05
TSS1500	FPR3	19	52297968/52297519	2	cg06784466/cg23886141	0.50555781	0.473946097	0.336397845	-0.169159965	2.33E-09	2.69E-08
TSS1500	FRMPD2	10	49483356/49483700	2	cg05859842/cg14979482	0.66701264	0.589427406	0.508465536	-0.158547104	6.37E-06	2.46E-05
TSS1500	FSHB	11	30251197/30251556/30251473	3	cg15159060/cg17685019/cg27420123	0.415140167	0.319891044	0.2424729	-0.172667267	5.88E-14	3.40E-11
TSS1500	FUT2	19	49197853/49198900/49198516	3	cg15197218/cg19025034/cg22654949	0.657818793	0.538283989	0.462275253	-0.19554354	1.97E-10	3.98E-09
TSS1500	FUT3	19	5852498/5851762	2	cg02284900/cg24314434	0.47289072	0.368456239	0.2974476	-0.17544312	4.83E-11	1.51E-09
TSS1500	FUT4	11	94276072/94275751	2	cg18834652/cg24256772	0.704500418	0.617967569	0.523169295	-0.181331122	4.17E-11	1.37E-09
TSS1500	FUT9	6	96463185/96463246/96462814	3	cg01837719/cg22782271/cg23997263	0.38322732	0.452863714	0.671393257	0.288165937	2.47E-10	4.71E-09
TSS1500	FZD10	12	130645645/130646461/130646022/130646129/130646256/130646370/130646497/130645566	8	cg02214745/cg02291403/cg09048530/cg17537177/cg18209212/cg20058043/cg21386611/cg22813950	0.29197381	0.332261222	0.541514312	0.249540502	9.37E-08	6.01E-07
TSS1500	GABRA6	5	161111187/161112334/161111441	3	cg16404898/cg19537070/cg22691257	0.587408405	0.470572333	0.369624839	-0.217783566	3.52E-13	7.96E-11
TSS1500	GABRG3	15	27215962/27216008/27215997/27215757/27216012/27215448/27215457/27215971/27216088/27215898/27215622/27215952/27215681/27216083	14	cg02069715/cg02281208/cg03024760/cg04646186/cg08182446/cg08460080/cg08920810/cg09257796/cg12919520/cg16384136/cg20362634/cg21711132/cg26508619/cg27553667	0.583282203	0.65619416	0.738250959	0.154968757	3.98E-10	6.73E-09
TSS1500	GABRR3	3	97754720	1	cg11726229	0.680513285	0.527521344	0.427380682	-0.253132603	3.53E-09	3.78E-08
TSS1500	GAD2	10	26504580/26504950/26504013/26504969/26503897/26504259	6	cg11500467/cg12527175/cg24097797/cg24820783/cg25835733/cg27372732	0.268145307	0.349401824	0.494764204	0.226618897	8.94E-08	5.77E-07

TSS1500	GAL3ST2	2	242715019/242715022/242715549/242715041	4	cg04144151/cg13345353/cg15072976/cg20302497	0.590108107	0.499956254	0.426985498	-0.163122608	9.73E-09	8.66E-08
TSS1500	GALNT13	2	154728210/154727485/154727907/154728133/154728062/154727925/154728202	7	cg07547000/cg08042220/cg12398124/cg12424785/cg14635767/cg21998983/cg26983710	0.398731636	0.398701913	0.550407905	0.151676268	4.84E-07	2.53E-06
TSS1500	GALNTL2	3	16215248/16215315/16215032	3	cg05287437/cg06853602/cg18772158	0.651893463	0.615331067	0.48970773	-0.162185733	7.31E-07	3.63E-06
TSS1500	GALNTL6	4	172734276/172734347/172734031/172733566/172733760/172734266	6	cg03304610/cg05295006/cg05678100/cg09892710/cg17896776/cg26814276	0.318865286	0.409996313	0.609992463	0.291127177	9.12E-11	2.29E-09
TSS1500	GALP	19	56686707/56686406/56686339	3	cg01454134/cg07682659/cg13688765	0.706592948	0.611872036	0.540568214	-0.166024734	8.84E-08	5.70E-07
TSS1500	GAP43	3	115340791/115341135/115341429	3	cg05773207/cg07863831/cg27608911	0.802694753	0.715902007	0.622085358	-0.180609396	1.30E-08	1.10E-07
TSS1500	GAPDHS	19	36023054/36023909	2	cg07868917/cg14946950	0.71102107	0.611907956	0.496276559	-0.214744511	1.09E-08	9.55E-08
TSS1500	GATA5	20	61051685/61051548/61051802/61051915/61051795/61051432/61051585/61051348/61051777/61051341/61051438/61051751/61052060/61051561/61051317/61051423/61051762/61051667	18	cg00677866/cg02146001/cg03777459/cg04742780/cg07914822/cg08568720/cg09339194/cg11982072/cg14388488/cg16714055/cg18278265/cg18872321/cg20525917/cg23770904/cg24320612/cg24500900/cg25667841/cg25988317	0.220801963	0.29125495	0.405140576	0.184338613	9.14E-05	0.000272471
TSS1500	GC	4	72651102/72650992/72650088	3	cg02087383/cg07376029/cg09816180	0.655018298	0.554332611	0.456003933	-0.199014364	3.12E-08	2.33E-07
TSS1500	GCM2	6	10882927/10883105/10882336/10883376/10882974/10883054/10882326	7	cg08510658/cg10074727/cg14000361/cg15244049/cg17991695/cg19951298/cg24329557	0.326755807	0.395915067	0.543143625	0.216387818	6.69E-09	6.34E-08

TSS1500	GCOM1	15	57883511/57883427/57883722/57883392	4	cg07084709/cg20043937/cg22184210/cg22708112	0.634315612	0.516472202	0.425577895	-0.208737716	3.67E-11	1.26E-09
TSS1500	GDE1	16	19533815/19534852	2	cg06784594/cg27259633	0.70235553	0.550445267	0.462975068	-0.239380462	1.39E-12	1.75E-10
TSS1500	GDF6	8	97173526/97173722/97173466/97174382/97173335	5	cg03734437/cg05447833/cg05552220/cg09730795/cg19939997	0.263622577	0.324730486	0.443267773	0.179645196	3.95E-05	0.000127246
TSS1500	GFRA1	10	118032626/118032355/118032033/118032892/118033581/118033312/118033762/118033370/118032872/118033115/118032081/118032879/118032330/118033902/118034031/118032948/118034357/118032987/118033273/118033290/118032905	21	cg03503087/cg04983142/cg06039355/cg08194313/cg09146197/cg11390957/cg11842610/cg12087643/cg13346013/cg13890706/cg18044663/cg18672939/cg19236679/cg20095233/cg23524195/cg23898073/cg24012811/cg25022687/cg25351565/cg25617725/cg27341926	0.221108251	0.304943749	0.445879434	0.224771184	1.17E-05	4.26E-05
TSS1500	GFRA2	8	21647596/21646662/21647026/21646704	4	cg03330485/cg03453638/cg05021638/cg14072903	0.238076203	0.245361925	0.397112514	0.159036311	0.000235296	0.000639156
TSS1500	GFRAL	6	55190909/55192049	2	cg11823729/cg23978358	0.584753985	0.453854192	0.318581841	-0.266172144	4.48E-13	8.76E-11
TSS1500	GGT6	17	4464379/4464294/4464400	3	cg01819502/cg21805880/cg22628873	0.633037058	0.514170641	0.458532962	-0.174504096	2.10E-08	1.66E-07
TSS1500	GHITM	10	85898700/85898649	2	cg12706330/cg25783892	0.508170923	0.353381897	0.258773808	-0.249397115	1.84E-11	7.74E-10
TSS1500	GHSR	3	172167445/172167644/172166987/172166920/172166517/172167527/172167030/172167610	8	cg04851268/cg06737494/cg07848409/cg11812218/cg14768785/cg22070855/cg22459146/cg23500122	0.359082754	0.42709868	0.551867745	0.192784991	2.28E-08	1.78E-07
TSS1500	GIP	17	47046292	1	cg20551517	0.83768907	0.659426294	0.564808618	-0.272880452	9.22E-08	5.93E-07
TSS1500	GIPC1	19	14607318/14607384/14607713/14607597/14608092	5	cg08312600/cg13825475/cg14528319/cg22760304/cg27651355	0.477127523	0.403001694	0.316530952	-0.160596572	3.56E-10	6.22E-09

TSS1500	GIPC2	1	78511360/78511344/78511235/78511140	4	cg09826056/cg19766489/cg20785560/cg26263401	0.421805445	0.32802308	0.266652909	-0.155152536	1.46E-10	3.19E-09
TSS1500	GIPC3	19	3584954	1	cg01895124	0.6403884	0.547575233	0.426509755	-0.213878645	4.28E-11	1.39E-09
TSS1500	GJC2	1	228336474/228337311/228336130/228337181/228336717	5	cg00393263/cg01789576/cg22844623/cg24052475/cg25136124	0.65882737	0.575216417	0.474556886	-0.184270484	9.29E-10	1.30E-08
TSS1500	GJD2	15	35047023/35047189/35047203/35047411/35047394/35047942/35047401	7	cg08095196/cg10386483/cg16729415/cg18415687/cg19999645/cg22562674/cg27532847	0.296011892	0.399459499	0.522280373	0.226268481	3.81E-08	2.74E-07
TSS1500	GLB1L3	11	134145979/134145889/134145490/134145940	4	cg13491690/cg23170850/cg23808004/cg25999867	0.137599071	0.192298363	0.297967898	0.160368828	0.000977703	0.002340221
TSS1500	GLDC	9	6646386	1	cg14538238	0.245986995	0.330779944	0.432050665	0.18606367	0.004265028	0.00898464
TSS1500	GLI2	2	121554307/121554418	2	cg10520924/cg27120020	0.7774622	0.697636383	0.577560223	-0.199901977	2.83E-09	3.17E-08
TSS1500	GLRA3	4	175750666/175751001/175750792/175750802	4	cg15104299/cg16592586/cg19968631/cg22045888	0.188807151	0.24187582	0.373663576	0.184856425	5.60E-09	5.50E-08
TSS1500	GLRB	4	157997173/157997189/157997167/157997086/157996959/157997079/157997064/157997178	8	cg04006722/cg05263495/cg06436905/cg07084358/cg10120856/cg18344745/cg24568468/cg25911468	0.375425395	0.400772235	0.531676319	0.156250924	6.85E-06	2.62E-05
TSS1500	GLRX	5	95159639/95159614/95159423/95159365	4	cg03852144/cg10949007/cg16677191/cg22689909	0.288228939	0.162668511	0.117161139	-0.1710678	1.30E-09	1.69E-08
TSS1500	GNAO1	16	56224782/56224504/56224901/56224627/56224793	5	cg00866976/cg04156464/cg06728579/cg08613597/cg10273340	0.121886658	0.203293909	0.404969255	0.283082597	1.18E-07	7.36E-07
TSS1500	GNAT1	3	50227735/50228220/50228212	3	cg00373148/cg01042877/cg22241014	0.64737922	0.561689794	0.430273139	-0.217106081	3.17E-10	5.70E-09
TSS1500	GNAT2	1	110156682/110156820/110156110	3	cg02492799/cg12234947/cg22941294	0.783376293	0.694172715	0.625757667	-0.157618627	2.54E-08	1.95E-07
TSS1500	GNPNAT1	14	53258644/53258590/53258684/53258601	4	cg02247430/cg11045303/cg16764848/cg20395072	0.444977933	0.367989403	0.293520493	-0.151457439	1.26E-09	1.65E-08

TSS1500	GNRHR	4	68622534/68622520/68622191	3	cg08916385/cg18003698/cg27219973	0.60504517	0.456784639	0.380771895	-0.224273275	2.81E-12	2.70E-10
TSS1500	GNS	12	65153982/65153459/65153561/65153476	4	cg00626466/cg02083989/cg07951810/cg15956273	0.63106359	0.4841896	0.360686759	-0.270376831	7.29E-10	1.07E-08
TSS1500	GOLGA8B	15	34876295/34876347/34876471/34876396/34876283	5	cg01471153/cg02700714/cg02966722/cg09670701/cg09684485	0.110715298	0.201695694	0.274303233	0.163587935	0.00030929	0.000820881
TSS1500	GON4L	1	155827556/155828357/155828174	3	cg00504075/cg12738981/cg17930201	0.503600785	0.410822348	0.342831706	-0.160769079	1.31E-10	2.95E-09
TSS1500	GP1BB	22	19710091/19709755/19710163/19709696	4	cg03970899/cg05726109/cg12899065/cg20193802	0.582109428	0.498699031	0.425653265	-0.156456163	3.84E-10	6.56E-09
TSS1500	GPA33	1	167060565/167060788	2	cg01607187/cg24987706	0.794774235	0.726788717	0.638605507	-0.156168728	2.74E-10	5.10E-09
TSS1500	GPHB5	14	63785334/63785523/63785287	3	cg02527030/cg02938807/cg04236382	0.56578788	0.463877017	0.367342977	-0.198444903	5.99E-10	9.14E-09
TSS1500	GPHN	14	66972956/66973361	2	cg15824062/cg23677229	0.86137562	0.803309378	0.675601732	-0.185773888	1.77E-05	6.19E-05
TSS1500	GPM6A	4	176924985/176735244	2	cg05224524/cg17304111	0.693465735	0.553623328	0.433092857	-0.260372878	2.65E-09	3.01E-08
TSS1500	GPR133	12	131437035/131437802/131438016	3	cg02075142/cg08943696/cg24033224	0.80877667	0.733452628	0.625779864	-0.182996806	5.31E-05	0.000166237
TSS1500	GPR141	7	37779122	1	cg24946941	0.804884015	0.7372793	0.643373055	-0.16151096	4.68E-05	0.000148757
TSS1500	GPR162	12	6929671/6929591/6930624/6930084	4	cg03795245/cg08349804/cg17805404/cg20093940	0.648837393	0.564521161	0.480883961	-0.167953433	2.76E-08	2.09E-07
TSS1500	GPR17	2	128402294/128403084/128403133/128402415	4	cg11067412/cg19724910/cg23528975/cg24131343	0.65779554	0.553877983	0.464734895	-0.193060645	1.07E-09	1.46E-08
TSS1500	GPR26	10	125425583/125425262/125425625	3	cg02721665/cg07036530/cg16783279	0.37946213	0.451469135	0.587918635	0.208456505	4.45E-08	3.15E-07
TSS1500	GPR27	3	71802533/71802608/71802420/71802594	4	cg03604840/cg04408595/cg22631938/cg22823146	0.20313981	0.221876609	0.396107026	0.192967216	6.53E-06	2.51E-05
TSS1500	GPR44	11	60624162/60623782/60623918	3	cg13109410/cg19254163/cg25087851	0.608081928	0.547852891	0.455812508	-0.152269421	1.36E-11	6.44E-10
TSS1500	GPR62	3	51988425	1	cg19238325	0.63293058	0.540711567	0.479703623	-0.153226957	1.24E-05	4.48E-05

TSS1500	GPR81	12	123215553/123215471/123215524/123215684	4	cg13702536/cg19328828/cg23505823/cg26643476	0.625405677	0.558253356	0.4302671	-0.195138577	2.39E-06	1.03E-05
TSS1500	GPR83	11	94135006/94134827/94135029	3	cg12226717/cg19851909/cg26738987	0.473334752	0.557997452	0.673637398	0.200302647	7.37E-07	3.66E-06
TSS1500	GPT	8	145728388/145729149/145728507/145728587/145728490/145728138/145728526/145728209/145729126/145729106/145728438/145728203/145729135/145728630/145729196/145728285/145728543/145728501	18	cg00280345/cg01919204/cg05241828/cg06110286/cg07658280/cg09265054/cg09957864/cg14476479/cg15685971/cg15983520/cg16582889/cg16587265/cg18555289/cg19352605/cg20545776/cg23793500/cg25600446/cg26572973	0.53467719	0.449175899	0.378689126	-0.155988064	6.88E-11	1.86E-09
TSS1500	GPX5	6	28493500/28493019/28493113/28493284/28493312	5	cg11562153/cg13679778/cg26313247/cg26398793/cg27123522	0.627405838	0.553652199	0.471714878	-0.15569096	1.67E-10	3.50E-09
TSS1500	GRAMD1B	11	123396039/123396299/123396133	3	cg03627771/cg26393964/cg26617787	0.539845423	0.445901998	0.377796439	-0.162048984	4.54E-11	1.44E-09
TSS1500	GREB1	2	11679605/11673928/11679872/11679845/11682017/11681405/11672761/11681908/11673360	9	cg01384111/cg10612997/cg13808071/cg15707428/cg18584561/cg19749001/cg25649765/cg25785303/cg25793243	0.593966826	0.477820935	0.409346799	-0.184620027	1.13E-11	5.66E-10
TSS1500	GRIA1	5	152869009/152869431/152869882/152869193	4	cg07633435/cg16281600/cg17987968/cg21146273	0.418881148	0.340422306	0.255120393	-0.163760755	5.50E-12	3.91E-10
TSS1500	GRIA2	4	158141570/158141076/158141542/158141526/158141492/158141449	6	cg00699993/cg02757172/cg10410142/cg13914083/cg15425280/cg23734973	0.409073504	0.484409099	0.627229025	0.218155521	3.89E-11	1.31E-09
TSS1500	GRID1	10	88126853/88127572/88127188/88127363	4	cg01229798/cg05349542/cg09168604/cg19014941	0.216951013	0.277379755	0.41945845	0.202507436	3.47E-05	0.000113272

TSS1500	GRIK1	21	31312905/31312643/31312535	3	cg07028661/cg08685096/cg24868359	0.275396941	0.346484311	0.429112839	0.153715898	4.39E-06	1.76E-05
TSS1500	GRIK3	1	37500790/37500195/37500369/37500358/37500441/37500508/37501164	7	cg06165395/cg10126715/cg16379885/cg16599703/cg19206040/cg19727439/cg24673742	0.316014279	0.370265047	0.484528205	0.168513925	1.23E-07	7.62E-07
TSS1500	GRIK5	19	42570406/42571402/42571339	3	cg00553487/cg03100024/cg13510813	0.521882803	0.408216576	0.296266229	-0.225616575	4.06E-11	1.34E-09
TSS1500	GRIN2A	16	10277505/10276375/10276984/10277317/10276383/10276674/10277254/10276580/10276799/10276600/10277017	11	cg00036299/cg00287829/cg00518386/cg01722994/cg06758670/cg07642043/cg08621611/cg09239744/cg16368442/cg24764979/cg25047001	0.187488334	0.294552973	0.470548664	0.28306033	4.85E-09	4.93E-08
TSS1500	GRIN2C	17	72857095/72857354/72856452/72856825/72857076/72857053/72857430	7	cg05410012/cg05848509/cg09169283/cg13715127/cg19700341/cg21264131/cg23513814	0.163162292	0.2084697	0.336178929	0.173016638	6.34E-06	2.45E-05
TSS1500	GRP	18	56886915/56887194/56887002/56887030	4	cg01625242/cg15633664/cg16899265/cg25260683	0.270584434	0.335783928	0.439009534	0.1684251	1.53E-06	6.90E-06
TSS1500	GRWD1	19	48947560/48947631/48947572/48947578/48948005	5	cg04988514/cg14594362/cg16875863/cg22242216/cg26362197	0.337209907	0.448778694	0.62741754	0.290207633	3.16E-09	3.46E-08
TSS1500	GRXCR1	4	42894337/42894981	2	cg05279772/cg20682222	0.75030223	0.683377903	0.587615532	-0.162686698	1.73E-07	1.04E-06
TSS1500	GSDMC	8	130799851	1	cg24243265	0.78446785	0.719909561	0.619964818	-0.164503032	1.25E-06	5.79E-06
TSS1500	GSX1	13	28365587/28365853/28365638/28366427/28365723/28366498	6	cg11444278/cg11508413/cg11571585/cg14973588/cg17352540/cg26270746	0.275504668	0.336323827	0.439153811	0.163649143	2.33E-07	1.34E-06
TSS1500	GTF2B	1	89357988/89357908/89357911/89358043	4	cg10430205/cg13868361/cg14010405/cg17811845	0.551929088	0.432387982	0.321511108	-0.23041798	4.67E-11	1.48E-09
TSS1500	GUCA1A	6	42121902/42121811	2	cg11971121/cg24216990	0.462109265	0.38630545	0.307492391	-0.154616874	1.15E-09	1.54E-08

TSS1500	GUCY1A2	11	106889557/106890150/106890247/106890084/106889745	5	cg04948524/cg10249705/cg13042926/cg21958798/cg23573913	0.186021068	0.215698291	0.338517068	0.152496	9.77E-06	3.60E-05
TSS1500	GYPA	4	145062810/145062551	2	cg16422931/cg25932299	0.614799865	0.558460942	0.458109759	-0.156690106	5.64E-07	2.89E-06
TSS1500	GYPB	4	144941588/144941609	2	cg05188963/cg16177314	0.741587975	0.664210031	0.561034986	-0.180552989	6.07E-10	9.23E-09
TSS1500	H2AFJ	12	14926909/14926986/14926572/14926317/14926744	5	cg07112131/cg09163005/cg12806797/cg23368416/cg26127836	0.456277333	0.517601821	0.616651501	0.160374169	0.000153301	0.000433427
TSS1500	HAVCR1	5	156486186/156486422/156486840	3	cg01485975/cg01551315/cg26432088	0.551772	0.444726806	0.35687703	-0.19489497	6.80E-09	6.41E-08
TSS1500	HBB	11	5249528/5248566	2	cg06233985/cg14544583	0.61646197	0.503877603	0.403433477	-0.213028493	2.48E-12	2.46E-10
TSS1500	HBBP1	11	5265215/5265155/5265992	3	cg01832325/cg20785459/cg26078436	0.629606845	0.519527303	0.451990174	-0.177616671	1.44E-08	1.20E-07
TSS1500	HCK	20	30639816/30639705/30639305	3	cg02311725/cg17326769/cg17508991	0.25352192	0.377866302	0.46868729	0.21516537	3.55E-05	0.000115405
TSS1500	HDGFL1	6	22568625/22568278	2	cg20209681/cg21898884	0.593182415	0.4328928	0.344404064	-0.248778351	4.04E-10	6.77E-09
TSS1500	HEPN1	11	124788414	1	cg23530543	0.777910525	0.658016239	0.490776441	-0.287134084	8.42E-08	5.46E-07
TSS1500	HERC6	4	89299173/89299314	2	cg08684066/cg24804970	0.828776775	0.723952339	0.637933505	-0.19084327	4.30E-08	3.05E-07
TSS1500	HHATL	3	42743794/42744889/42743921/42743744/42745757/42744301/42745362	7	cg00407150/cg08060987/cg08776619/cg18817801/cg19606364/cg19827650/cg24962576	0.617358187	0.528969819	0.434289509	-0.183068678	9.54E-11	2.36E-09
TSS1500	HHLA2	3	108021106/108020451/108020727	3	cg02059214/cg02124498/cg08817540	0.660093073	0.545509414	0.430773614	-0.229319459	3.98E-07	2.14E-06
TSS1500	HIBADH	7	27704065/27703436/27702803	3	cg01662334/cg11724147/cg22168087	0.724416758	0.582106253	0.520662452	-0.203754305	1.90E-07	1.12E-06
TSS1500	HIGD1B	17	42924794/42924231/42923814	3	cg15460047/cg20127596/cg24417621	0.78544294	0.683533878	0.603909509	-0.181533431	7.10E-06	2.70E-05
TSS1500	HIST1H3J	6	27859811/27859160	2	cg12629515/cg23504411	0.440070303	0.362063121	0.262609053	-0.17746125	7.24E-07	3.60E-06

TSS1500	HIST2H2BF	1	149784851/149784212/149785211/149784571/149399930/149399454/149400292/149399911/149400082/149784776/149400352	11	cg00428476/cg04888113/cg08636083/cg12769994/cg13082351/cg14497172/cg15157455/cg16519842/cg17313031/cg23782591/cg24437737	0.267308122	0.335265354	0.441835125	0.174527003	5.53E-10	8.61E-09
TSS1500	HIST4H4	12	14924953/14924930	2	cg08746853/cg13064062	0.548641208	0.483775031	0.35650017	-0.192141037	2.85E-07	1.60E-06
TSS1500	HK3	5	176326619/176327783/176326924	3	cg17809377/cg19791262/cg23912072	0.492673845	0.429004306	0.3098316	-0.182842245	1.22E-11	5.98E-10
TSS1500	HKDC1	10	70979827/70979811/70979777	3	cg05532178/cg11639651/cg23341612	0.811678557	0.715256174	0.537345724	-0.274332832	1.10E-06	5.16E-06
TSS1500	HLA-DOA	6	32978548/32978233/32978281/32978593/32978610/32978129/32978516/32978271/32978775/32978379/32977674/32978196/32978140/32978053/32977983/32978570/32977708/32978718	18	cg00988577/cg01715800/cg01720176/cg02172533/cg03546401/cg04615290/cg05043389/cg05408403/cg05683739/cg06504039/cg08147094/cg11076970/cg13132775/cg16914277/cg17313945/cg18278290/cg20024110/cg24476363	0.73392532	0.657341578	0.559822115	-0.174103204	5.40E-14	3.40E-11
TSS1500	HLA-DQB2	6	32732685/32732371/32732585/32732806/32732764/32732247	6	cg06499030/cg16315262/cg16899306/cg17043222/cg21566383/cg23743845	0.526920723	0.451237178	0.322530036	-0.204390687	1.08E-08	9.47E-08
TSS1500	HMHB1	5	143190384/143191194/143191226	3	cg12672779/cg14461752/cg15823954	0.872872	0.77037915	0.612053091	-0.260818909	6.82E-09	6.42E-08
TSS1500	HMP19	5	173472066/173472097	2	cg19414040/cg25494605	0.620565258	0.529324583	0.467093693	-0.153471564	2.06E-06	9.04E-06
TSS1500	HOOK2	19	12887524/12886986/12886980/12886982	4	cg00072407/cg07484849/cg10810394/cg15683940	0.63322083	0.543957313	0.480309717	-0.152911113	2.23E-11	8.79E-10
TSS1500	HORMAD2	22	30476206/30476098/30476089	3	cg04046669/cg16686158/cg21890667	0.61742774	0.710920494	0.775279118	0.157851378	3.53E-10	6.18E-09

TSS1500	HOXC10	12	54378500/54378736/54377550/54377457	4	cg03833604/cg04710764/cg16898193/cg20403938	0.266000433	0.330528956	0.429185785	0.163185352	0.000640479	0.001592719
TSS1500	HOXC13	12	54332222/54331892/54332026/54332209/54332181	5	cg01577751/cg04727865/cg17085352/cg20587394/cg22830707	0.422407746	0.480509992	0.582601793	0.160194047	1.37E-10	3.05E-09
TSS1500	HPVC1	7	54271282/54271152	2	cg21336434/cg23860325	0.609088925	0.465808281	0.326292093	-0.282796832	1.12E-09	1.51E-08
TSS1500	HS3ST2	16	22824489/22824877/22825100/22825046/22825158/22825282/22825621	7	cg03033688/cg03132773/cg03713642/cg09191731/cg10370599/cg20023231/cg27403265	0.289570293	0.322859115	0.446038792	0.156468499	3.54E-05	0.000115308
TSS1500	HS3ST3A1	17	13506186/13505751/13505558/13506230/13505590/13506284/13505799	7	cg00203913/cg09463656/cg12503102/cg15214448/cg18236079/cg19298225/cg24375938	0.328428358	0.422663599	0.548477802	0.220049445	4.64E-07	2.44E-06
TSS1500	HS3ST5	6	114385144/114384871/114384629	3	cg06694119/cg18192491/cg23968650	0.557370025	0.396287689	0.334053877	-0.223316148	5.49E-10	8.55E-09
TSS1500	HSD17B14	19	49340489/49340574/49340765/49340593	4	cg04777726/cg16594139/cg19024700/cg26267310	0.376007511	0.484255928	0.568531901	0.19252439	1.14E-06	5.34E-06
TSS1500	HTR1A	5	63257885/63257813/63257847/63257867/63257941/63257873/63257753	7	cg07839533/cg08259925/cg09698471/cg11615755/cg13666507/cg15092168/cg16280141	0.338136579	0.413936406	0.581700744	0.243564165	4.67E-10	7.57E-09
TSS1500	HTR1E	6	87646738/87646462/87646740/87646804	4	cg02508664/cg15888097/cg20991421/cg22442841	0.321823975	0.230113461	0.171712014	-0.150111961	5.23E-12	3.76E-10
TSS1500	HTR3D	3	183749212/183749414/183750284/183749227	4	cg00576550/cg04200192/cg10685228/cg14483391	0.543917035	0.470620147	0.380217845	-0.16369919	1.21E-09	1.60E-08
TSS1500	HYAL4	7	123484985/123484534/123484898	3	cg00422716/cg03085932/cg15560337	0.562269267	0.474617861	0.40113375	-0.161135517	3.52E-11	1.22E-09
TSS1500	IBSP	4	88720194	1	cg08422456	0.81885805	0.748870656	0.657580914	-0.161277136	2.09E-05	7.16E-05
TSS1500	IDO2	8	39792086/39791224/39791430	3	cg11363097/cg16957569/cg17533575	0.663849933	0.549809731	0.443291468	-0.220558465	3.24E-10	5.78E-09
TSS1500	IER3IP1	18	44703596	1	cg12128262	0.831279815	0.776058533	0.650141227	-0.181138588	2.87E-05	9.56E-05

TSS1500	IFT52	20	42218758/42219223	2	cg03539340/cg22031417	0.44370789	0.327934328	0.256021827	-0.187686063	1.02E-10	2.49E-09
TSS1500	IGFBP3	7	45961473/45961455/45961524/45961102/45961849/45961311/45962236/45961346/45961289/45961778/45961126/45962052/45961469/45961074/45961943/45961865/45961794/45961874/45962185/45961089/45961401/45962090/45961960/45961078/45961508/45961419/45961537/45962054/45961772/45961569	30	cg02120774/cg03776080/cg04690927/cg04796162/cg05083496/cg07910986/cg08541297/cg08831744/cg09619271/cg10094651/cg10677697/cg11753867/cg12244052/cg14228300/cg15208757/cg16447589/cg16460681/cg19536059/cg20850023/cg21268650/cg22083798/cg22403266/cg22800581/cg23193639/cg23455440/cg24381682/cg24772240/cg24901098/cg24942272/cg26434048	0.258169452	0.353855144	0.458315967	0.200146515	1.42E-07	8.68E-07
TSS1500	IGFL4	19	46544662/46545605/46544711/46544802	4	cg13095627/cg15458254/cg17720013/cg25574111	0.463024185	0.35958294	0.27607766	-0.186946525	2.22E-11	8.76E-10
TSS1500	IGJ	4	71533675	1	cg17075352	0.548423785	0.494963422	0.3914145	-0.157009285	1.31E-05	4.72E-05
TSS1500	IGLON5	19	51814765/51814782	2	cg22489583/cg25728174	0.066697245	0.071265356	0.282444154	0.21574691	0.000556643	0.001403369
TSS1500	IKZF1	7	50343298/50343883/50343131/50343361/50343869	5	cg01139861/cg07589773/cg14216940/cg16697214/cg18607529	0.247046594	0.35052237	0.447245417	0.200198823	3.09E-08	2.31E-07
TSS1500	IL17A	6	52050133/52050597	2	cg09582880/cg11924517	0.67080109	0.520380394	0.348856723	-0.321944367	5.51E-11	1.62E-09
TSS1500	IL18	11	112035679/112035945/112035175	3	cg04100971/cg05687149/cg09122223	0.511182303	0.39276607	0.322028429	-0.189153875	5.64E-10	8.74E-09
TSS1500	IL1RL1	2	102953421/102927488/102927397/102927278/102952285/102952419	6	cg01254707/cg11916609/cg16386158/cg17738684/cg19795292/cg25869196	0.762629115	0.686094569	0.610272636	-0.152356479	7.01E-07	3.51E-06
TSS1500	IL22	12	68647498/68647590	2	cg13195526/cg27518047	0.66151086	0.570971714	0.507683802	-0.153827058	7.53E-09	6.97E-08

TSS1500	IL22RA1	1	24470205/24470647/24470527/24470228	4	cg02072322/cg08880327/cg11576349/cg26405802	0.630749995	0.5591269	0.479367123	-0.151382872	1.58E-09	1.98E-08
TSS1500	IL25	14	23841754/23840837/23841104	3	cg14366598/cg16025120/cg23619217	0.51775648	0.386178833	0.242359564	-0.275396916	3.98E-11	1.32E-09
TSS1500	IL26	12	68620926/68621047/68620370	3	cg08338368/cg24700219/cg26871735	0.681791252	0.60146402	0.502473458	-0.179317794	4.25E-09	4.41E-08
TSS1500	IL28B	19	39736699/39736956/39736690	3	cg03346025/cg11270633/cg11781854	0.692607653	0.59424287	0.468001588	-0.224606065	1.26E-09	1.65E-08
TSS1500	INA	10	105036611/105036701/105036645	3	cg18673954/cg21384402/cg23642747	0.276782288	0.405315217	0.614194856	0.337412569	2.11E-07	1.23E-06
TSS1500	INADL	1	62207325/62207459/62207888	3	cg03605365/cg04914562/cg12128740	0.481722762	0.366462233	0.296634845	-0.185087916	5.48E-11	1.62E-09
TSS1500	INHBA	7	41742993/41743001/41743606	3	cg14527389/cg16415646/cg19535073	0.587903653	0.509307967	0.420310994	-0.167592659	3.85E-10	6.57E-09
TSS1500	INS	11	2182644/2183434/2183864/2182795/2182783/2183505/2183832/2183708/2182672/2183420/2183824	11	cg03366382/cg09864961/cg10732560/cg11208229/cg11322849/cg16711450/cg18098639/cg23390871/cg24338752/cg25601886/cg25969082	0.689767896	0.612410613	0.513122089	-0.176645807	7.49E-11	1.97E-09
TSS1500	INS-IGF2	11	2182644/2183434/2183864/2182795/2182783/2183505/2183832/2183708/2182672/2183420/2183824	11	cg03366382/cg09864961/cg10732560/cg11208229/cg11322849/cg16711450/cg18098639/cg23390871/cg24338752/cg25601886/cg25969082	0.689767896	0.612410613	0.513122089	-0.176645807	7.49E-11	1.97E-09
TSS1500	IPW	15	25360934/25360436	2	cg11811341/cg16217553	0.670196175	0.596967167	0.481156432	-0.189039743	6.05E-07	3.08E-06
TSS1500	IQCJ	3	158786187/158786193/158786242	3	cg02325869/cg15736726/cg26659665	0.784742753	0.739937919	0.631615188	-0.153127565	3.84E-06	1.57E-05
TSS1500	IRF4	6	391189/391208/391114/391441	4	cg01108118/cg10379890/cg25140370/cg26433102	0.159531561	0.300096832	0.450682769	0.291151208	3.32E-08	2.45E-07

TSS1500	IRX1	5	3594679/3595728/3595907/3595106	4	cg01790920/cg05534710/cg06689918/cg07903370	0.257273828	0.338832611	0.4921754	0.234901573	3.31E-11	1.17E-09
TSS1500	IRX4	5	1883413/1883514/1884212/1884365/1883940/1883954/1883214	7	cg00243313/cg00264591/cg05941108/cg14823763/cg18009496/cg23121993/cg24876960	0.254729214	0.364092219	0.533084286	0.278355072	1.46E-06	6.66E-06
TSS1500	IRX6	16	55357253/55357776/55357842/55358072	4	cg02859129/cg04622777/cg08295543/cg09659661	0.237643127	0.322108204	0.43116555	0.193522423	1.84E-06	8.15E-06
TSS1500	ISLR2	15	74422275/74421535/74420796/74421935/74420890/74421635/74422330/74421523/74422528/74421857/74421739/74421584/74420511/74421848/74420307/74421887/74420657/74422282/74421918/74421531/74421909	21	cg00550503/cg00781110/cg01568668/cg02709205/cg04138492/cg04920649/cg05505745/cg05547902/cg05923854/cg06041677/cg07216112/cg07917269/cg08036502/cg08703540/cg11977693/cg12017911/cg21322819/cg23152885/cg25705558/cg25828462/cg27200833	0.225178705	0.26188545	0.386005899	0.160827194	2.28E-06	9.88E-06
TSS1500	ITGBL1	13	102104573/102104707/102103930	3	cg02119955/cg05138203/cg16852483	0.682733358	0.596603048	0.476781662	-0.205951696	8.23E-12	4.90E-10
TSS1500	ITIH4	3	52865917/52865592/52865637	3	cg06204229/cg22534898/cg27125504	0.55623455	0.4652603	0.371328418	-0.184906132	4.47E-12	3.50E-10
TSS1500	IVL	1	152879586/152880324/152880108	3	cg02889647/cg15059851/cg25082710	0.557640102	0.435763476	0.330629729	-0.227010373	1.04E-10	2.50E-09
TSS1500	IZUMO1	19	49250439/49250452/49250561	3	cg03609666/cg08536841/cg15146515	0.301826057	0.327932451	0.466086427	0.164260371	0.000189135	0.000524794
TSS1500	KAT2A	17	40274811/40274670/40273815/40274740/40274728/40274703/40274524/40274722	8	cg00582337/cg01501896/cg04278905/cg07168142/cg08880849/cg16208084/cg26916966/cg26934993	0.586550897	0.501542763	0.433139376	-0.153411521	1.76E-09	2.14E-08

TSS1500	KCNA1	12	5018338/5018513/5018715/5018815/5018229/5018805/5018798	7	cg00838874/cg04891086/cg06652199/cg11388325/cg15930596/cg19651132/cg20392607	0.183212817	0.279456873	0.503915957	0.32070314	6.22E-11	1.73E-09
TSS1500	KCNA4	11	30038788/30039113/30039004/30038797/30039031/30038692	6	cg03506489/cg11735997/cg12122597/cg13161658/cg14161359/cg22685409	0.382359359	0.468829547	0.610019638	0.227660279	1.86E-08	1.49E-07
TSS1500	KCNA7	19	49577206/49577162/49577303/49576955	4	cg03564727/cg07992308/cg16693612/cg27435138	0.5416406	0.442799737	0.382365655	-0.159274945	1.84E-07	1.09E-06
TSS1500	KCNH7	2	163696187/163696000/163695776/163695793/163695965/163695882	6	cg07150045/cg09454925/cg12302070/cg19965810/cg21302951/cg25668368	0.367267259	0.432803777	0.538715473	0.171448214	1.10E-09	1.48E-08
TSS1500	KCNIP1	5	169930841/169930535/169780480/169929560/169780299/169780320	6	cg11956545/cg12833011/cg16335868/cg23479949/cg24615189/cg26947766	0.386437808	0.464275517	0.543464659	0.157026852	2.33E-10	4.51E-09
TSS1500	KCNK12	2	47797953/47798679/47798477/47797963/47798396	5	cg01826863/cg04934807/cg04981611/cg13913015/cg27320127	0.092764989	0.153461384	0.33881546	0.24605047	1.97E-05	6.80E-05
TSS1500	KCNK16	6	39290711/39290877/39290715	3	cg01429075/cg01669948/cg24018148	0.59993263	0.484186694	0.381925432	-0.218007198	1.35E-11	6.42E-10
TSS1500	KCNMB1	5	169817797/169817247/169816960	3	cg07802909/cg17425484/cg24990238	0.478795875	0.348029622	0.264546905	-0.21424897	1.19E-11	5.86E-10
TSS1500	KCNQ5	6	73330358/73330297/73330208/73331191/73330112/73331114/73331290/73331238/73330106	9	cg03020545/cg03922126/cg04290119/cg04377145/cg05350268/cg05447008/cg09303936/cg11008243/cg15120732	0.155452546	0.241257703	0.333359766	0.177907219	1.13E-07	7.07E-07
TSS1500	KDELR2	7	6524998/6525025	2	cg08622675/cg27221338	0.756145083	0.619762497	0.491512	-0.264633083	1.00E-10	2.45E-09
TSS1500	KHDC1L	6	73935584/73935676	2	cg00732807/cg10190334	0.710557635	0.553520339	0.403592364	-0.306965271	2.54E-14	2.33E-11
TSS1500	KHDRBS2	6	62996702/62996697/62996627/62996664	4	cg00578220/cg02217159/cg04398581/cg23926793	0.212554239	0.312072944	0.510115303	0.297561064	9.37E-07	4.51E-06

TSS1500	KIAA0408	6	127781063/127781873/127781298	3	cg05798147/cg06003184/cg13662246	0.737810268	0.639857235	0.555318829	-0.18249144	6.14E-10	9.33E-09
TSS1500	KIAA1024	15	79724044/79723997/79724519/79724517/79723655	5	cg05390694/cg05419425/cg05804863/cg11821817/cg12284802	0.154960982	0.2105245	0.305212052	0.15025107	0.000226655	0.000617364
TSS1500	KIAA1199	15	81070337/81070851/81071273	3	cg08083041/cg20828084/cg24159335	0.450242945	0.367014461	0.289188219	-0.161054726	1.10E-11	5.59E-10
TSS1500	KIAA1211	4	57035957/57035948	2	cg24814707/cg25769043	0.590043015	0.46921345	0.398481877	-0.191561138	1.18E-09	1.57E-08
TSS1500	KIAA1644	22	44709224/44709891/44709614	3	cg03392837/cg18649854/cg22576171	0.640219883	0.543210783	0.422231193	-0.217988689	4.68E-12	3.53E-10
TSS1500	KIAA1671	22	25422889/25423700/25423531	3	cg04692156/cg14966613/cg17889356	0.683305805	0.57995265	0.525666317	-0.157639488	2.61E-11	9.87E-10
TSS1500	KIF16B	20	16555226/16554531/16555549/16555519/16555475	5	cg04189665/cg06390653/cg14406419/cg17003395/cg26272575	0.572599097	0.468765763	0.403130248	-0.169468848	1.69E-07	1.01E-06
TSS1500	KIF4B	5	154392274	1	cg11365072	0.37462481	0.281270878	0.20956445	-0.16506036	3.47E-11	1.21E-09
TSS1500	KIR2DL4	19	55314050/55314542	2	cg01306806/cg13444135	0.558463075	0.48903265	0.389622305	-0.16884077	0.000113757	0.000331098
TSS1500	KIR3DX1	19	55042650/55042837/55042806	3	cg03108250/cg10731960/cg23037932	0.56462195	0.505934433	0.402681918	-0.161940032	1.30E-06	5.97E-06
TSS1500	KLC3	19	45843694/45842723	2	cg06864587/cg16855440	0.73479337	0.631872261	0.540724059	-0.194069311	4.05E-08	2.90E-07
TSS1500	KLK9	19	51514146/51513611/51513252/51513296/51513883	5	cg01144251/cg17031773/cg18504937/cg20670939/cg27068996	0.636909479	0.572596844	0.474862456	-0.162047023	4.25E-09	4.41E-08
TSS1500	KLRG1	12	9141740/9141775	2	cg00443307/cg26806779	0.721784318	0.576945203	0.381956675	-0.339827643	4.18E-11	1.37E-09
TSS1500	KRBA1	7	149411377/149411652/149410923/149411256/149411498	5	cg01269670/cg08131100/cg09089451/cg14671222/cg23778596	0.408030093	0.479871996	0.596456009	0.188425916	1.10E-08	9.57E-08
TSS1500	KRT15	17	39676096/39676704	2	cg08469845/cg15801433	0.597117215	0.521795806	0.438599891	-0.158517324	7.46E-12	4.72E-10
TSS1500	KRT26	17	38929184/38929662	2	cg06456531/cg11079711	0.477870633	0.344278794	0.26043897	-0.217431663	4.86E-12	3.60E-10
TSS1500	KRT27	17	38939796/38940118/38939673/38940132	4	cg09563851/cg10173723/cg14504826/cg16488522	0.688018896	0.583417704	0.455518333	-0.232500563	2.76E-10	5.13E-09
TSS1500	KRT28	17	38956837/38957507	2	cg16048803/cg17900015	0.580754185	0.506782667	0.407487061	-0.173267124	2.32E-08	1.80E-07

TSS1500	KRT38	17	39598940/39598430/39597980/39597909/39597813	5	cg07933967/cg14680360/cg18425570/cg18848394/cg22139563	0.714402239	0.610894572	0.496418522	-0.217983717	2.25E-09	2.63E-08
TSS1500	KRT5	12	52915064/52915036/52915646	3	cg00368577/cg17565113/cg22566740	0.46932701	0.362008222	0.291836832	-0.177490178	1.79E-12	2.02E-10
TSS1500	KRT8	12	53299310/53299145/53300293	3	cg01835489/cg20324165/cg26357344	0.516273617	0.411677035	0.320502232	-0.195771385	6.28E-11	1.74E-09
TSS1500	KRTAP10-5	21	46001928/46001680	2	cg14363932/cg25937451	0.692162755	0.616624286	0.540662	-0.151500755	4.60E-06	1.84E-05
TSS1500	KRTAP12-3	21	46077454/46077582/46077562	3	cg04257752/cg15021280/cg17839758	0.46506563	0.351853017	0.299782016	-0.165283614	1.44E-05	5.12E-05
TSS1500	KRTAP13-3	21	31799248/31799091	2	cg07621949/cg24500711	0.656129755	0.590849072	0.478530098	-0.177599657	2.46E-07	1.41E-06
TSS1500	KRTAP13-4	21	31802169	1	cg24082983	0.558472755	0.452200783	0.301083264	-0.257389491	3.49E-10	6.12E-09
TSS1500	KRTAP19-8	21	32412191	1	cg06790305	0.648607205	0.483502822	0.346723436	-0.301883769	1.02E-08	8.97E-08
TSS1500	KRTAP20-3	21	32014696/32013856/32014658	3	cg06002471/cg06100608/cg19708992	0.79047898	0.744003217	0.639977961	-0.150501019	4.39E-06	1.77E-05
TSS1500	KRTAP21-1	21	32128696/32129060/32128800/32128708	4	cg11354857/cg22373097/cg22405653/cg22961727	0.757560785	0.6887691	0.593213933	-0.164346852	3.12E-09	3.42E-08
TSS1500	KRTAP22-1	21	31972533/31972672/31972923/31972913	4	cg05572621/cg07945323/cg26294716/cg27456885	0.703983528	0.603841164	0.52957761	-0.174405917	5.41E-09	5.35E-08
TSS1500	KRTAP23-1	21	31722001	1	cg24786671	0.54144102	0.471419328	0.358596168	-0.182844852	1.14E-07	7.14E-07
TSS1500	KRTAP24-1	21	31655654/31655822/31655982	3	cg09892262/cg16692258/cg24184737	0.73508976	0.631146252	0.542897868	-0.192191892	1.57E-06	7.06E-06
TSS1500	KRTAP25-1	21	31663266	1	cg11475420	0.791629345	0.749222833	0.609950545	-0.1816788	0.001132803	0.002675688
TSS1500	KRTAP3-1	17	39165675/39165697	2	cg04566037/cg14957731	0.401147948	0.314112767	0.245759118	-0.155388829	1.45E-09	1.84E-08
TSS1500	KRTAP4-7	17	39239008/39239918	2	cg16629452/cg25341923	0.553094613	0.429659439	0.321880909	-0.231213703	1.91E-12	2.06E-10
TSS1500	KRTAP4-9	17	39261100	1	cg15015397	0.573342895	0.438338633	0.339173591	-0.234169304	3.34E-13	7.87E-11
TSS1500	KRTAP6-2	21	31972533/31972672	2	cg05572621/cg07945323	0.622442423	0.470917492	0.362859402	-0.25958302	5.23E-12	3.76E-10
TSS1500	KRTAP7-1	21	32202284	1	cg02642585	0.765460345	0.707974806	0.601410059	-0.164050286	2.55E-05	8.58E-05
TSS1500	L1TD1	1	62660188/62660191	2	cg06190807/cg14850026	0.616042125	0.497507064	0.454561398	-0.161480727	4.86E-10	7.80E-09
TSS1500	LACRT	12	55029147/55028956/55029660	3	cg07643942/cg07855572/cg19880901	0.530885175	0.4219179	0.313562711	-0.217322464	1.88E-09	2.26E-08

TSS1500	LAMA1	18	7118077/7118122/7118092	3	cg03159124/cg14950072/cg19909349	0.32049846	0.412715654	0.601877356	0.281378896	3.13E-08	2.33E-07
TSS1500	LAMA2	6	129203075/129203783/129203887	3	cg01881054/cg15801019/cg20237610	0.531843185	0.444779656	0.37181315	-0.160030035	5.07E-08	3.53E-07
TSS1500	LAMA4	6	112576119/112576393/112576342	3	cg07628084/cg08844365/cg18080303	0.608370817	0.553389943	0.445629029	-0.162741788	8.77E-09	7.93E-08
TSS1500	LAMB3	1	209825856/209826863/209825747/209825943/209825849/209825678/209825672/209825731	8	cg01580568/cg03967293/cg03977657/cg04873061/cg19709585/cg22502856/cg26107033/cg26533764	0.55592853	0.448079312	0.365375495	-0.190553035	5.34E-11	1.60E-09
TSS1500	LAMC2	1	183154778/183153944	2	cg00208830/cg10519299	0.421969505	0.344875911	0.270515936	-0.151453569	3.00E-09	3.32E-08
TSS1500	LARP4	12	50793981/50793961/50794308/50794088	4	cg01928411/cg11063877/cg12582030/cg21902817	0.385205922	0.313389002	0.234615597	-0.150590325	8.35E-12	4.91E-10
TSS1500	LASS3	15	101085341/101085710/101085210	3	cg08428878/cg12131707/cg15580989	0.715416375	0.606574572	0.553232118	-0.162184257	1.84E-05	6.40E-05
TSS1500	LBH	2	30453395/30454146/30453758	3	cg03801871/cg04092762/cg04253876	0.234521091	0.306540372	0.388444163	0.153923072	1.21E-05	4.39E-05
TSS1500	LCE1A	1	152798912/152799138	2	cg01975957/cg06221609	0.732903533	0.688560611	0.57326007	-0.159643462	7.26E-08	4.80E-07
TSS1500	LCE1F	1	152747562/152747472/152747501	3	cg13493250/cg23413307/cg26302157	0.524542505	0.434719876	0.333897429	-0.190645076	1.20E-11	5.93E-10
TSS1500	LCE2A	1	152670598/152669985	2	cg13974761/cg22616525	0.445367135	0.343731917	0.231782325	-0.21358481	1.54E-15	8.67E-12
TSS1500	LCE6A	1	152814273/152814831/152813850/152815068	4	cg09417355/cg16265542/cg22124859/cg26531371	0.685775702	0.568900296	0.446591103	-0.239184599	2.31E-09	2.68E-08
TSS1500	LCN1	9	138413012	1	cg23815000	0.587504685	0.509539744	0.40991645	-0.177588235	9.42E-09	8.43E-08
TSS1500	LCN9	9	138554641/138554704	2	cg05025211/cg13555662	0.435718723	0.378925769	0.283764598	-0.151954125	1.42E-09	1.81E-08
TSS1500	LEFTY1	1	226077628/226078237/226077303/226078135/226077287	5	cg00103855/cg12319004/cg14264773/cg15604953/cg25552768	0.54437355	0.459823659	0.387227207	-0.157146343	6.98E-09	6.53E-08
TSS1500	LELP1	1	153175248/153174654/153175262/153174759	4	cg02303399/cg06804411/cg23219336/cg24091195	0.715165969	0.646089385	0.526680835	-0.188485134	9.77E-09	8.69E-08
TSS1500	LHFPL4	3	9596482/9596849	2	cg00242951/cg23223755	0.653730158	0.565931014	0.459743066	-0.193987092	1.13E-07	7.07E-07

TSS1500	LHX8	1	75593476/75592950/75593804/75593018/75593865/75593596	6	cg00419321/cg01005080/cg01971160/cg09766820/cg11196437/cg21009965	0.367582565	0.444971906	0.536440255	0.16885769	1.58E-07	9.56E-07
TSS1500	LIM2	19	51891526/51892428/51892299	3	cg12491710/cg23389776/cg24724596	0.390809215	0.290902511	0.210774955	-0.18003426	2.23E-13	7.11E-11
TSS1500	LIPH	3	185271312/185271385/185271383	3	cg01390445/cg04513882/cg16255307	0.861541818	0.827888689	0.665705432	-0.195836386	7.48E-05	0.000226759
TSS1500	LOC100127888	20	61298677/61299106/61299190/61299126	4	cg10486610/cg12502929/cg14966074/cg18645150	0.537722223	0.449535475	0.380737498	-0.156984725	1.71E-09	2.10E-08
TSS1500	LOC100129794	14	37640247/37640455/37639757	3	cg13017854/cg20597409/cg26342676	0.763945803	0.696803739	0.560492405	-0.203453398	1.45E-06	6.61E-06
TSS1500	LOC100130933	17	73641809/73642080/73642404	3	cg07251887/cg11619216/cg19468528	0.49860129	0.393641959	0.320715924	-0.177885366	1.01E-11	5.38E-10
TSS1500	LOC100131193	9	139704416/139704146	2	cg08055330/cg13728106	0.705329465	0.589939867	0.469584555	-0.23574491	5.25E-07	2.72E-06
TSS1500	LOC100131496	20	45947025/45946429/45946271	3	cg03082779/cg04504205/cg20645040	0.363758615	0.261173481	0.195131955	-0.16862666	1.09E-10	2.59E-09
TSS1500	LOC100133469	14	106937310/106938234/106937869	3	cg13275724/cg16023283/cg20900869	0.723368845	0.641521967	0.490657027	-0.232711818	1.84E-10	3.79E-09
TSS1500	LOC100133893	12	27925367	1	cg04279139	0.451170915	0.299006994	0.241501863	-0.209669052	5.98E-12	4.10E-10
TSS1500	LOC100190940	12	130527106/130527104/130527212	3	cg02681173/cg10233825/cg15848986	0.33267563	0.438905169	0.628995864	0.296320234	4.86E-08	3.40E-07
TSS1500	LOC100240726	20	45094318/45094548/45095158	3	cg00351202/cg15815117/cg21164545	0.717545525	0.647700422	0.553335232	-0.164210293	1.58E-05	5.58E-05
TSS1500	LOC100240734	12	54494573/54494546	2	cg23295636/cg24134056	0.64105307	0.549873431	0.46494085	-0.17611222	1.32E-09	1.71E-08
TSS1500	LOC100302640	3	106960066/106959716/106959864/106959817	4	cg09545443/cg12550012/cg13694746/cg25233816	0.094515851	0.184385669	0.255761091	0.16124524	5.48E-05	0.000170967
TSS1500	LOC149134	1	246952508/246952362/246952118/246952495/246952215/246952048	6	cg03340119/cg03596016/cg05314024/cg08535928/cg15176829/cg26898024	0.441644319	0.593780953	0.640010458	0.198366139	2.44E-07	1.39E-06
TSS1500	LOC149620	1	111822494/111822347/111822068/111822043	4	cg02815538/cg09138965/cg09333723/cg21527708	0.61799501	0.471464439	0.348441073	-0.269553937	4.65E-13	8.92E-11

TSS1500	LOC154822	7	158800547/158799748/158799715/158799777/158800132	5	cg00600916/cg10874111/cg11772799/cg14302471/cg18194535	0.618417935	0.5266688	0.444839188	-0.173578747	7.04E-12	4.53E-10
TSS1500	LOC157627	8	9761905/9761454/9761141/9761171/9762300/9762100/9762057	7	cg06292304/cg12616174/cg14278808/cg15248835/cg15537082/cg18246262/cg24185864	0.21117185	0.309867936	0.474917205	0.263745355	2.25E-07	1.30E-06
TSS1500	LOC220115	13	53061810/53061702	2	cg24527452/cg24577699	0.737200465	0.608814525	0.487636289	-0.249564176	9.91E-13	1.45E-10
TSS1500	LOC254312	10	10994495/10995060	2	cg17261125/cg23610154	0.648268078	0.556177344	0.47912463	-0.169143448	3.24E-07	1.78E-06
TSS1500	LOC283392	12	72667526/72667493/72667512/72667707	4	cg04640250/cg11054631/cg19188182/cg19516457	0.337556387	0.429673511	0.577333436	0.239777049	1.32E-08	1.12E-07
TSS1500	LOC283731	15	74422275/74422750/74421935/74422330/74422528/74421857/74421848/74422632/74422935/74421887/74422572/74422761/74422282/74422629/74422550/74422933/74422545/74421918/74423077/74421909	20	cg00550503/cg02378847/cg02709205/cg05505745/cg05923854/cg06041677/cg08703540/cg10070260/cg11315754/cg12017911/cg18887483/cg21063758/cg23152885/cg23392501/cg24123518/cg24374861/cg24653100/cg25705558/cg26091142/cg27200833	0.218381254	0.238989787	0.370833067	0.152451813	3.63E-05	0.000118026
TSS1500	LOC283761	15	90068229/90068044/90068496	3	cg00502017/cg07518380/cg14584565	0.58161704	0.496992733	0.404183895	-0.177433145	2.66E-07	1.50E-06
TSS1500	LOC283867	16	65611542/65611369/65610659	3	cg02944238/cg03000116/cg07779653	0.66190025	0.580839717	0.477156227	-0.184744023	2.85E-06	1.20E-05
TSS1500	LOC283999	17	76226563/76226735/76226122	3	cg08930147/cg09445803/cg22108646	0.497796893	0.394618636	0.332521795	-0.165275097	6.72E-11	1.83E-09
TSS1500	LOC284688	1	170254397/170253944/170253617/170254369	4	cg01057196/cg04909854/cg13065624/cg23038594	0.701184026	0.572779628	0.541802827	-0.159381199	1.40E-05	5.00E-05
TSS1500	LOC285205	3	107601033/107601227/107601098	3	cg22488719/cg22949077/cg25703122	0.6312059	0.548032717	0.46758275	-0.16362315	3.94E-09	4.17E-08
TSS1500	LOC285375	3	13691504/13691967	2	cg01325116/cg20187719	0.58385671	0.50451725	0.417564986	-0.166291724	2.50E-07	1.43E-06

TSS1500	LOC285419	4	124572578/124694661/124694178/124694137	4	cg15826810/cg19297164/cg19922435/cg22872906	0.458488961	0.371239064	0.302701918	-0.155787044	9.33E-11	2.33E-09
TSS1500	LOC285593	5	173005254/173005657/173006447/173005622/173005325	5	cg14904688/cg16026902/cg23987381/cg27033712/cg27128739	0.808501848	0.740257783	0.642807986	-0.165693861	1.57E-05	5.57E-05
TSS1500	LOC285627	5	158894390/158894440/158893488	3	cg07829289/cg16338178/cg19017803	0.750007188	0.520880192	0.426324782	-0.323682406	4.84E-11	1.51E-09
TSS1500	LOC285796	6	163746113/163746145/163746175	3	cg14529406/cg17684207/cg21229979	0.244707735	0.347835903	0.420380989	0.175673254	0.00025674	0.000692386
TSS1500	LOC286094	8	136245114/136245226/136245867	3	cg00937286/cg25053164/cg25188398	0.739040135	0.665511231	0.524096264	-0.214943871	1.16E-07	7.22E-07
TSS1500	LOC388428	17	79138873/79139087/79139096	3	cg05569220/cg06136185/cg11689732	0.638980265	0.562542474	0.471873509	-0.167106756	1.09E-11	5.55E-10
TSS1500	LOC400804	1	221510889/221510043/221510354	3	cg19865770/cg20482462/cg27518240	0.70046702	0.630086897	0.494556416	-0.205910604	1.04E-07	6.60E-07
TSS1500	LOC400931	22	46481099/46480891/46481248/46481603	4	cg03412618/cg04329382/cg10352237/cg21094022	0.689967818	0.562328522	0.452296267	-0.237671552	5.40E-11	1.60E-09
TSS1500	LOC402644	7	28319676/28319594/28319589	3	cg00863007/cg00924052/cg00927666	0.60195079	0.483976194	0.380082259	-0.221868531	3.76E-09	3.99E-08
TSS1500	LOC402778	11	1770066/1770595/1770181/1769645/1770394	5	cg07523553/cg15536116/cg16328251/cg16934127/cg27226043	0.288526535	0.341644683	0.466506763	0.177980228	2.83E-05	9.45E-05
TSS1500	LOC407835	7	128765150/128765077/128765024	3	cg02625727/cg06785701/cg16588297	0.831062313	0.684978942	0.567967386	-0.263094926	3.09E-11	1.11E-09
TSS1500	LOC441666	10	42864633/42864912	2	cg08967936/cg23853165	0.756067858	0.617767356	0.499858898	-0.25620896	1.92E-08	1.54E-07
TSS1500	LOC642587	1	209600926/209601066/209601422/209600938	4	cg04295258/cg21823119/cg22166446/cg25733599	0.703638349	0.631787419	0.52822314	-0.175415209	7.38E-10	1.08E-08
TSS1500	LOC642852	21	46706579/46706820/46706759/46707012	4	cg05091734/cg08538258/cg14522034/cg17353354	0.585240355	0.672704184	0.755061431	0.169821076	2.02E-10	4.07E-09
TSS1500	LOC643387	2	239139518/239139911/239140032/239140024	4	cg02536451/cg06102419/cg11904906/cg13957874	0.475030078	0.540146742	0.658296873	0.183266795	7.98E-10	1.14E-08

TSS1500	LOC644145	4	56685845/56685972	2	cg13985817/cg16606320	0.518198053	0.612309308	0.698672482	0.180474429	5.87E-11	1.67E-09
TSS1500	LOC723809	7	104568180/104567372/104567387	3	cg02384419/cg05595832/cg16314851	0.703838912	0.606622094	0.524737771	-0.17910114	9.75E-08	6.21E-07
TSS1500	LOC729384	11	89656189/89654946/89654367/89655871	4	cg03068446/cg13738382/cg22405122/cg23481862	0.62846035	0.488757044	0.367714518	-0.260745832	4.94E-11	1.53E-09
TSS1500	LOC732275	16	86380594/86379872	2	cg04475868/cg05825085	0.67583703	0.604839006	0.512462468	-0.163374562	0.000216844	0.000593023
TSS1500	LOC90246	3	128225824/128226071/128225898	3	cg01886524/cg19234171/cg27319445	0.58364543	0.486038464	0.37908442	-0.20456101	7.34E-12	4.66E-10
TSS1500	LONP2	16	48277879/48277559	2	cg03354717/cg09539739	0.594269068	0.443638378	0.358753823	-0.235515245	1.49E-11	6.82E-10
TSS1500	LPPR5	1	99471295/99470895/99470709/99470967/99470801	5	cg03593336/cg05657427/cg06124224/cg14241810/cg26266842	0.234989543	0.288484527	0.41691794	0.181928397	7.78E-08	5.10E-07
TSS1500	LRAT	4	155664242/155664954/155664941/155664150/155664311/155664223/155663689/155663836/155663990	9	cg00714184/cg05763228/cg07207220/cg10203610/cg16566627/cg22888418/cg23587449/cg23676439/cg25806701	0.260433553	0.312097273	0.448931213	0.18849766	4.24E-06	1.71E-05
TSS1500	LRFN1	19	39806765/39806522/39807167	3	cg00471142/cg18592745/cg26768966	0.779306737	0.679745354	0.552807456	-0.226499281	1.41E-08	1.18E-07
TSS1500	LRFN5	14	42075865/42075554/42075760/42075975/42075810/42075406/42075664/42076492	8	cg02150867/cg02454806/cg04171065/cg05943996/cg10160975/cg10327400/cg21526847/cg23955431	0.219774383	0.317605529	0.485380155	0.265605772	5.17E-08	3.58E-07
TSS1500	LRGUK	7	133811808/133811837/133811828	3	cg14455998/cg17676607/cg19967800	0.106828097	0.17019928	0.297776353	0.190948256	0.000560825	0.001412971
TSS1500	LRIT2	10	85986738/85986565/85985779	3	cg01614169/cg02790260/cg19717640	0.68959963	0.640053965	0.537046526	-0.152553104	2.94E-07	1.64E-06
TSS1500	LRP1B	2	142890490/142890700	2	cg04133673/cg21340845	0.501603585	0.430920775	0.303982293	-0.197621292	2.61E-09	2.97E-08
TSS1500	LRRC15	3	194091192/194091167/194090730	3	cg02121427/cg07048348/cg13321259	0.436879946	0.33448417	0.242659559	-0.194220387	5.56E-11	1.63E-09
TSS1500	LRRC18	10	50123030/50123344/50123203	3	cg01357233/cg11915309/cg17858662	0.783549975	0.664677335	0.533254877	-0.250295098	2.81E-09	3.16E-08

TSS1500	LRRC30	18	7230489/7229726/7230245	3	cg03759556/cg06616178/cg13543667	0.733707528	0.649004335	0.566750261	-0.166957268	9.41E-11	2.34E-09
TSS1500	LRRC31	3	169588236/169588423	2	cg04548519/cg05924537	0.531880333	0.433872622	0.345394005	-0.186486328	2.15E-11	8.62E-10
TSS1500	LRRC3B	3	26663593/26663805/26663626/26663740/26663885/26663551	6	cg04954680/cg10332616/cg10768206/cg13689261/cg13964105/cg18404374	0.394196023	0.481931106	0.616193942	0.22199792	2.15E-09	2.54E-08
TSS1500	LRRC4	7	127672235/127672473/127672458/127672169/127672152	5	cg04153784/cg09087503/cg12628196/cg15087147/cg27573591	0.088532415	0.264454207	0.408407066	0.319874652	4.94E-11	1.53E-09
TSS1500	LRRC52	1	165512398/165513253/165512980	3	cg03454607/cg16509443/cg17495466	0.642337243	0.551586514	0.456601775	-0.185735468	9.92E-09	8.81E-08
TSS1500	LRRC55	11	56948018/56947754/56948015	3	cg03717588/cg11751645/cg24458609	0.235242513	0.401743172	0.461201923	0.22595941	4.25E-05	0.000136187
TSS1500	LRRC8E	19	7953071/7953054/7953081	3	cg17494927/cg17731776/cg26149920	0.576554692	0.437104054	0.360019982	-0.21653471	1.96E-12	2.06E-10
TSS1500	LRRK2	12	40618560/40618356/40618575/40618284	4	cg05667817/cg11684647/cg18050543/cg23699274	0.086289493	0.133620735	0.246389655	0.160100162	0.000944048	0.002266826
TSS1500	LRTM1	3	54962954/54962380/54963186	3	cg02810692/cg11383981/cg16418228	0.620259175	0.530860483	0.438679721	-0.181579454	3.77E-09	4.00E-08
TSS1500	LUZP1	1	23496648/23495577/23495997/23496304	4	cg01435109/cg25610515/cg26051750/cg26530485	0.450025862	0.363369439	0.285846568	-0.164179293	1.83E-05	6.38E-05
TSS1500	LUZP2	11	24518281/24518225/24518149	3	cg10345326/cg13376598/cg24473393	0.24979755	0.299755389	0.410934256	0.161136706	0.000345447	0.000908204
TSS1500	LVRN	5	115297723/115297690/115297853/115297488	4	cg06644553/cg10199250/cg15489294/cg23915769	0.390596855	0.434036431	0.606987731	0.216390876	1.39E-08	1.17E-07
TSS1500	LXN	3	158390769/158390821/158390778	3	cg08263647/cg17481912/cg17977250	0.362378473	0.263283233	0.2025658	-0.159812673	3.32E-11	1.17E-09
TSS1500	LY86	6	6588693/6587445/6588089	3	cg03349397/cg13125224/cg20162076	0.73167312	0.691454739	0.570090132	-0.161582988	2.10E-05	7.21E-05
TSS1500	LYSMD4	15	100274128/100274248/100273828/100274195	4	cg02329205/cg02724404/cg05414235/cg12967560	0.368548174	0.261224847	0.201254954	-0.167293219	3.33E-13	7.87E-11

TSS1500	MAEL	1	166958221/166958308/166958273/166958233	4	cg18894878/cg23968893/cg24453820/cg27053975	0.786243959	0.712467403	0.631748922	-0.154495037	2.33E-09	2.69E-08
TSS1500	MAL	2	95691255/95690821/95690169/95691228/95691051	5	cg04804539/cg05142617/cg05314420/cg09983051/cg21245652	0.407244651	0.472105504	0.585547321	0.17830267	2.87E-08	2.17E-07
TSS1500	MAPRE1	20	31407338/31407348	2	cg11800794/cg14303674	0.647181228	0.565159331	0.488071902	-0.159109325	2.39E-06	1.03E-05
TSS1500	9-Mar	12	58147425	1	cg03249863	0.7284345	0.668873322	0.576746177	-0.151688323	1.49E-06	6.75E-06
TSS1500	MAT1A	10	82050793/82050672/82049858	3	cg00977842/cg07959747/cg19765377	0.48812237	0.385289378	0.298287173	-0.189835197	7.77E-11	2.02E-09
TSS1500	MBL1P	10	81679675/81678829	2	cg07726667/cg27320518	0.523452085	0.443618861	0.354106232	-0.169345853	9.06E-07	4.39E-06
TSS1500	MBLAC1	7	99723162/99724112/99723100	3	cg09417640/cg24028459/cg26650651	0.7201235	0.613934419	0.499565611	-0.220557889	2.63E-09	2.98E-08
TSS1500	MCAM	11	119189289/119188145	2	cg08861493/cg21096399	0.67546607	0.560402567	0.458232082	-0.217233988	6.29E-12	4.16E-10
TSS1500	MCHR2	6	100442553/100442489	2	cg03809898/cg08430325	0.668931825	0.609099583	0.48698425	-0.181947575	1.28E-06	5.91E-06
TSS1500	MDGA2	14	48145108/48144280/48145000/48144369/48144765	5	cg08217024/cg09155219/cg18856581/cg19672997/cg23254031	0.372531368	0.458571993	0.601586988	0.22905562	1.56E-10	3.34E-09
TSS1500	MELK	9	36572645/36572411/36572421/36572685	4	cg01288067/cg14279528/cg14431547/cg14552260	0.492432652	0.387820779	0.325611903	-0.166820749	2.97E-10	5.41E-09
TSS1500	MEOX2	7	15727441/15726821/15727598/15727525/15727127/15726649	6	cg00839579/cg02908900/cg02969141/cg03985727/cg07395354/cg13047892	0.333475658	0.396422489	0.494892993	0.161417335	1.83E-05	6.36E-05
TSS1500	MEPE	4	88753434/88753179/88752660	3	cg07651189/cg10702457/cg13255629	0.65143326	0.571670817	0.487158295	-0.164274965	1.12E-07	7.04E-07

TSS1500	MESTIT1	7	130131633/130132265/130132422/130131484/130131403/130131691/130132161/130132259/130131826/130131916/130131931/130131887/130132413/130131829/130131676/130131905/130131258/130132453/130131869/130131480/130132199/130131566/130132298/130131709/130131923/130132319/130131921/130131359/130132419/130131797/130131367/130132305/130131268/130132360/130131730/130131885/130132286	37	cg00286878/cg01784351/cg03588221/cg04344875/cg04678950/cg04786207/cg05556276/cg05862114/cg06212135/cg07315018/cg07870293/cg09080913/cg09462536/cg10249538/cg12347392/cg13104298/cg13917504/cg13986840/cg16823958/cg17079325/cg17580798/cg18934293/cg19344806/cg19386484/cg20050761/cg21200654/cg21629528/cg21667116/cg22592140/cg22705386/cg23156962/cg23714917/cg25407198/cg25519926/cg26708559/cg27338480/cg27589003	0.6173555	0.683000804	0.769635892	0.152280392	6.46E-13	1.11E-10
TSS1500	METTL8	2	172291680	1	cg06772099	0.558694	0.451223483	0.365377459	-0.193316541	1.50E-11	6.86E-10
TSS1500	MFRP	11	119218027/119217884/119218238	3	cg10904867/cg16996281/cg19560971	0.499881308	0.387882361	0.274490673	-0.225390635	6.58E-13	1.11E-10
TSS1500	MGC27382	1	78694398/78693899	2	cg06975633/cg22559013	0.517239735	0.615099331	0.678263693	0.161023958	4.99E-10	7.96E-09
TSS1500	MGC42105	5	43191993/43191975	2	cg21098557/cg23502772	0.137492416	0.21099606	0.323634028	0.186141612	0.001056628	0.002509194
TSS1500	MGC45800	4	183066237/183066224/183066337	3	cg03839661/cg04143736/cg10603183	0.139401321	0.197654173	0.346387791	0.20698647	0.000683807	0.001690055
TSS1500	MGP	12	15039432/15039560/15039397	3	cg13302154/cg16617257/cg22221831	0.41907688	0.317507364	0.244825323	-0.174251557	5.86E-10	8.99E-09
TSS1500	MGST1	12	16505000/16499190/16505650/16499963/16500185/16500403/16500077	7	cg00609333/cg06610614/cg10449466/cg10548102/cg11203041/cg18233746/cg27117982	0.50644357	0.411752113	0.355370401	-0.151073169	3.11E-12	2.81E-10

TSS1500	MINPP1	10	89263900/89263946/89263949	3	cg02614016/cg06090298/cg16132151	0.759484993	0.648474591	0.575135247	-0.184349746	1.32E-08	1.12E-07
TSS1500	MIOX	22	50923890/50924745/50923963	3	cg13332571/cg19621271/cg23513727	0.744776612	0.648096317	0.562427082	-0.18234953	2.48E-10	4.72E-09
TSS1500	MIR100	11	122023504	1	cg24655701	0.533257555	0.429848106	0.302563009	-0.230694546	1.04E-12	1.51E-10
TSS1500	MIR10A	17	46657555/46658170/46657656	3	cg01572694/cg04514255/cg14884929	0.409854038	0.318621502	0.259074667	-0.150779372	4.87E-10	7.82E-09
TSS1500	MIR1179	15	89149849/89150842	2	cg05013243/cg08867933	0.59760518	0.532840106	0.401139295	-0.196465885	5.42E-07	2.80E-06
TSS1500	MIR1185-2	14	101509249/101509391/101510016/101509281/101509144	5	cg01205338/cg01709493/cg02975060/cg03547809/cg13098800	0.787765985	0.706664617	0.616338652	-0.171427333	2.91E-08	2.20E-07
TSS1500	MIR124-1	8	9761905/9761454/9762300/9762100/9762057	5	cg06292304/cg12616174/cg15537082/cg18246262/cg24185864	0.220295895	0.314818128	0.457661601	0.237365706	7.73E-07	3.82E-06
TSS1500	MIR124-2	8	65291287/65291341/65290484/65290320/65290848	5	cg02226645/cg04104463/cg07792478/cg10698928/cg14590098	0.376358443	0.479843157	0.585473476	0.209115034	1.68E-08	1.37E-07
TSS1500	MIR124-3	20	61809035/61808888/61809348/61808691/61809557	5	cg02065637/cg02650317/cg04927004/cg08737296/cg15699267	0.187117414	0.288151058	0.443465535	0.256348122	1.86E-07	1.11E-06
TSS1500	MIR1251	12	97885353/97884975/97885270	3	cg00030523/cg17967602/cg27533635	0.597145763	0.51609387	0.43601167	-0.161134094	9.51E-12	5.15E-10
TSS1500	MIR125B1	11	121971219/121970768/121971954/121971232/121971092/121971247	6	cg06749053/cg07281370/cg07685357/cg20475322/cg24150623/cg24603444	0.409153656	0.465950062	0.577771757	0.168618102	4.86E-09	4.94E-08
TSS1500	MIR1283-2	19	54260014/54260952/54260627	3	cg02991888/cg16908824/cg26242772	0.643736055	0.508172161	0.389747636	-0.253988419	2.05E-09	2.44E-08
TSS1500	MIR129-1	7	127846739/127847603/127847045	3	cg04840800/cg11364290/cg24044186	0.685890575	0.579163158	0.429474025	-0.25641655	2.63E-11	9.91E-10
TSS1500	MIR129-2	11	43602179/43602436/43602000/43601969/43602420/43602017	6	cg01514668/cg04355791/cg06372015/cg11074814/cg11638181/cg14901205	0.239342681	0.324975682	0.451839116	0.212496435	2.43E-06	1.04E-05
TSS1500	MIR133A2	20	61160996/61161073/61161232	3	cg04990378/cg07915635/cg17267710	0.736885295	0.627608472	0.53484778	-0.202037515	1.63E-08	1.33E-07

TSS1500	MIR135B	1	205418278/205418410	2	cg02520707/cg13061767	0.725982255	0.550687044	0.453863557	-0.272118698	2.65E-10	4.98E-09
TSS1500	MIR138-2	16	56891173/56891111	2	cg02961808/cg26475094	0.785997763	0.683107044	0.533238943	-0.252758819	2.37E-10	4.58E-09
TSS1500	MIR141	12	7072696/7072891/7072765/7071814/7072727	5	cg00366413/cg18959988/cg22413603/cg24702147/cg27534624	0.515926418	0.409691613	0.324465546	-0.191460872	5.99E-11	1.69E-09
TSS1500	MIR147	9	123007911	1	cg09399225	0.55051652	0.421065983	0.333741941	-0.216774579	3.31E-10	5.88E-09
TSS1500	MIR147B	15	45724277/45724580	2	cg20636382/cg23505252	0.621534738	0.484264992	0.39664795	-0.224886788	1.06E-11	5.47E-10
TSS1500	MIR154	14	101525784/101525465/101525106/101524635	4	cg06265809/cg11069071/cg19477205/cg23029159	0.607642039	0.523455899	0.411739017	-0.195903022	1.17E-09	1.56E-08
TSS1500	MIR184	15	79500886/79501491/79501052	3	cg01207916/cg23121785/cg23721598	0.708715365	0.630393444	0.555096323	-0.153619042	2.97E-09	3.30E-08
TSS1500	MIR187	18	33485300/33485594	2	cg03599197/cg16673522	0.705142875	0.602534189	0.514274645	-0.19086823	2.92E-05	9.70E-05
TSS1500	MIR192	11	64659065/64659060/64658940/64659387/64660076/64658936/64658946/64659044	8	cg00376448/cg00400165/cg00589493/cg02494703/cg06104877/cg08432452/cg13092487/cg24154336	0.549972634	0.458912892	0.384133263	-0.165839371	3.28E-11	1.16E-09
TSS1500	MIR194-1	1	220292508/220292590	2	cg23617067/cg24658737	0.595965473	0.450043589	0.349890777	-0.246074695	2.15E-11	8.62E-10
TSS1500	MIR194-2	11	64659387/64660076/64660386	3	cg02494703/cg06104877/cg23266943	0.447213755	0.362904281	0.293650795	-0.15356296	6.63E-11	1.82E-09
TSS1500	MIR1973	4	117220363/117220469	2	cg23666362/cg23740016	0.74332074	0.678466014	0.587393198	-0.155927542	2.35E-08	1.82E-07
TSS1500	MIR205	1	209604583/209604397/209605223/209605104/209604378	5	cg01334432/cg05922993/cg12386297/cg17860090/cg27261597	0.695530373	0.606375639	0.507846248	-0.187684125	5.24E-09	5.23E-08
TSS1500	MIR2053	8	113655436	1	cg15943335	0.59865631	0.5075743	0.416271955	-0.182384355	3.28E-05	0.000107778
TSS1500	MIR2113	6	98471368	1	cg25199591	0.462028045	0.334985306	0.247081214	-0.214946831	6.38E-10	9.62E-09
TSS1500	MIR215	1	220292508/220292590	2	cg23617067/cg24658737	0.595965473	0.450043589	0.349890777	-0.246074695	2.15E-11	8.62E-10
TSS1500	MIR216B	2	56229060	1	cg02071553	0.738162795	0.683389278	0.5301385	-0.208024295	4.11E-06	1.67E-05
TSS1500	MIR218-2	5	168196464/168195460/168196172	3	cg00534479/cg09887953/cg24141911	0.66623901	0.527391889	0.415007068	-0.251231942	2.77E-11	1.03E-09

TSS1500	MIR296	20	57394000/57393844/57394097/57393380/57393277	5	cg03030267/cg11954355/cg15428904/cg21983491/cg25188071	0.736996827	0.655026802	0.576379371	-0.160617455	1.76E-07	1.05E-06
TSS1500	MIR298	20	57394000/57393844/57394097	3	cg03030267/cg11954355/cg15428904	0.74324864	0.658549428	0.58082605	-0.16242259	2.55E-05	8.58E-05
TSS1500	MIR30A	6	72114693	1	cg16506910	0.387141005	0.297486239	0.225188323	-0.161952682	9.95E-13	1.45E-10
TSS1500	MIR30C2	6	72088126	1	cg15319027	0.73638431	0.646403472	0.585904486	-0.150479824	9.70E-06	3.58E-05
TSS1500	MIR34C	11	111383668/111382915/111383603/111383288/111383576/111383083/111383515/111383624/111383889/111383653	10	cg01192900/cg03242880/cg13767940/cg18515591/cg21881253/cg22806002/cg22879515/cg23211240/cg24041078/cg26561785	0.304172077	0.372950868	0.546756216	0.242584139	1.12E-06	5.26E-06
TSS1500	MIR376C	14	101505523/101505130/101505598	3	cg01200264/cg02047319/cg10268144	0.785509913	0.690672498	0.620266924	-0.165242989	6.66E-11	1.82E-09
TSS1500	MIR377	14	101527688/101528123/101528156/101527046/101527144/101527072/101526893	7	cg03663955/cg05138957/cg11721554/cg11869269/cg18588811/cg20474788/cg23468699	0.735225586	0.633640854	0.572917813	-0.162307773	8.99E-10	1.26E-08
TSS1500	MIR489	7	93114192	1	cg04650977	0.67619819	0.595550022	0.517460436	-0.158737754	0.000281273	0.000752307
TSS1500	MIR494	14	101495047/101495604/101495758	3	cg01131100/cg07429629/cg10400174	0.817443933	0.760106092	0.645709443	-0.171734489	9.20E-07	4.44E-06
TSS1500	MIR518A2	19	54242243/54242124/54241421	3	cg02336334/cg09430586/cg22468497	0.729264248	0.645512458	0.575922323	-0.153341925	9.01E-09	8.11E-08
TSS1500	MIR541	14	101530580/101530343/101529855/101530537	4	cg03050096/cg04235416/cg04638655/cg07126399	0.632969792	0.525123398	0.44402917	-0.188940622	4.13E-09	4.32E-08
TSS1500	MIR548A1	6	18571548	1	cg03000711	0.719775305	0.63767465	0.515342773	-0.204432532	1.95E-05	6.73E-05
TSS1500	MIR548D2	1	58333307/58332862	2	cg00944580/cg18642361	0.48692311	0.372359772	0.301802393	-0.185120717	4.87E-12	3.60E-10
TSS1500	MIR548F2	2	213292541	1	cg20953052	0.42382712	0.354604533	0.232070873	-0.191756247	2.21E-09	2.58E-08
TSS1500	MIR548I4	7	147256123/147256263/147256367	3	cg02515354/cg07299645/cg16341266	0.74525388	0.650377206	0.574960491	-0.170293389	2.17E-13	7.10E-11
TSS1500	MIR551A	1	3478216/3477690/3478767/3477695	4	cg01432902/cg07830126/cg12427039/cg24536161	0.696001913	0.582556053	0.492431661	-0.203570251	4.48E-11	1.44E-09

TSS1500	MIR551B	3	168268757/168268421	2	cg07747690/cg21490751	0.730831	0.636815628	0.535881484	-0.194949516	3.78E-07	2.04E-06
TSS1500	MIR620	12	116586898/116586872/116587387	3	cg09230679/cg18087803/cg19685229	0.655619202	0.556600363	0.505465965	-0.150153237	1.27E-12	1.65E-10
TSS1500	MIR663	20	26189510/26190328/26190354/26189665/26189240/26190347	6	cg01860778/cg04150495/cg10715092/cg11220245/cg14345012/cg20395967	0.452555565	0.509105472	0.611950982	0.159395417	4.83E-11	1.51E-09
TSS1500	MIR708	11	79114457/79114296/79114133	3	cg04726985/cg17233829/cg25837979	0.777650382	0.674587989	0.598956709	-0.178693673	1.01E-08	8.92E-08
TSS1500	MIR920	12	24365132	1	cg07739398	0.854453365	0.779061706	0.698192582	-0.156260783	0.000203914	0.000561549
TSS1500	MIRLET7A2	11	122018517/122018662	2	cg06811361/cg18590130	0.634759205	0.531353511	0.417370495	-0.21738871	2.78E-10	5.16E-09
TSS1500	MKX	10	28035631/28035655/28035208/28035864/28035894/28035731/28035157/28036151/28035719/28035822	10	cg02161900/cg02249292/cg05381746/cg05485137/cg07964848/cg08815081/cg09713234/cg14947429/cg17918002/cg27303223	0.295523692	0.367885474	0.494327492	0.198803801	1.62E-07	9.76E-07
TSS1500	MMADHC	2	150444762/150444834/150444569	3	cg00486525/cg07483086/cg11713727	0.565267658	0.511713652	0.394917624	-0.170350034	1.25E-07	7.74E-07
TSS1500	MMD2	7	5000111/5000219	2	cg02276823/cg25523580	0.674385015	0.575368811	0.514176527	-0.160208488	2.33E-05	7.93E-05
TSS1500	MME	3	154797349/154797867/154797508/154797083/154797399/154797592/154797589/154797563/154797677/154797779/154796861/154796701	12	cg00992239/cg02108850/cg03648334/cg07862554/cg08085954/cg14750844/cg16580737/cg17344755/cg21496658/cg22471230/cg23273897/cg24033859	0.177400593	0.220188435	0.340930097	0.163529505	0.000893848	0.002157744
TSS1500	MMP16	8	89340750/89340069/89341103/89340205/89340139/89340331/89340480	7	cg01673994/cg05033271/cg07609388/cg08397978/cg12818557/cg14644752/cg24456365	0.327120406	0.40430779	0.575818649	0.248698243	3.14E-07	1.74E-06
TSS1500	MMP2	16	55512870/55514470/55514269/55512806/55514378/55512822/55512868	7	cg01821058/cg02458945/cg07582157/cg08318842/cg09350341/cg09530163/cg12317456	0.231178489	0.283300107	0.390696867	0.159518378	1.50E-05	5.32E-05

TSS1500	MNDA	1	158800024/158799935	2	cg05304729/cg25119415	0.52524288	0.405687272	0.305605705	-0.219637175	4.17E-12	3.36E-10
TSS1500	MOXD2	7	141947168/141947898/141947347	3	cg02814416/cg03129645/cg06058073	0.685689607	0.604163709	0.518270926	-0.167418681	1.01E-07	6.41E-07
TSS1500	MRGPRD	11	68748907/68749124	2	cg11903239/cg20791323	0.62277544	0.443668222	0.358181995	-0.264593445	3.35E-09	3.62E-08
TSS1500	MRGPRX2	11	19083542/19082514	2	cg10422455/cg13062935	0.531498233	0.433999244	0.327454177	-0.204044055	6.16E-13	1.07E-10
TSS1500	MRGPRX4	11	18193422/18193674/18193360	3	cg02608124/cg09691574/cg16446783	0.638002827	0.548582467	0.458618794	-0.179384033	1.26E-10	2.89E-09
TSS1500	MRPL3	3	131222041/131222106	2	cg12405775/cg20894410	0.523065455	0.378796017	0.291372573	-0.231692882	2.33E-13	7.11E-11
TSS1500	MRPL34	19	17414977/17415626/17415155	3	cg01445659/cg08702612/cg17328880	0.544542253	0.449392267	0.385518386	-0.159023866	8.16E-12	4.90E-10
TSS1500	MRPL4	19	10362061/10361286	2	cg01409448/cg12507788	0.592785645	0.472625761	0.374090207	-0.218695438	1.65E-11	7.34E-10
TSS1500	MS4A4A	11	60049447/60049475/60049097/60047483/60046908	5	cg01229998/cg12604169/cg16954525/cg23754934/cg27008678	0.657691098	0.566088192	0.478658109	-0.179032988	4.45E-09	4.58E-08
TSS1500	MS4A5	11	60196790/60195729/60196778	3	cg00534700/cg04382783/cg17630127	0.73209927	0.604259689	0.541099527	-0.190999743	1.84E-05	6.41E-05
TSS1500	MSGN1	2	17997138/17996994/17997117	3	cg03377767/cg22762189/cg26632171	0.496319117	0.403741398	0.345607421	-0.150711695	3.46E-09	3.71E-08
TSS1500	MSX1	4	4860026/4860431/4860468/4860655/4860551/4859975/4859985/4861122/4861101/4860082/4860617/4860190/4859996/4860061/4861183/4860698/4859935/4859960/4860439/4860547/4860590/4860157	22	cg00486340/cg03335246/cg03672274/cg04100843/cg04660422/cg07035659/cg07462756/cg08121252/cg09918082/cg10266211/cg11566977/cg13207326/cg13882284/cg15092343/cg15696627/cg17938612/cg18849102/cg19846622/cg20588069/cg21689228/cg22371591/cg24469464	0.356029471	0.422432098	0.549218078	0.193188607	1.83E-09	2.22E-08
TSS1500	MTIF2	2	55496552/55497012	2	cg05986680/cg27478579	0.627054505	0.4972025	0.404546427	-0.222508078	4.77E-12	3.58E-10
TSS1500	MTMR6	13	25862332/25862639/25862438	3	cg13555116/cg15071391/cg24801023	0.68594255	0.639700797	0.503333325	-0.182609225	1.51E-08	1.25E-07

TSS1500	MTMR9	8	11141680/11141415/11141424/11141365	4	cg03034634/cg08623223/cg21337580/cg24378421	0.346501575	0.405252514	0.498643881	0.152142307	5.46E-05	0.000170176
TSS1500	MTUS2	13	30002334/30002475/29597447/30002471/29597851	5	cg03989020/cg06472554/cg07790085/cg16315060/cg23509128	0.486457913	0.397255731	0.286765231	-0.199692681	3.25E-08	2.40E-07
TSS1500	MTVR2	17	54962555/54962812	2	cg14039347/cg25273584	0.63381135	0.523852786	0.436492609	-0.197318741	1.48E-08	1.23E-07
TSS1500	MUC13	3	124654885	1	cg14497879	0.62254475	0.46971605	0.371612186	-0.250932564	1.43E-10	3.13E-09
TSS1500	MUC16	19	9093098/9093198	2	cg05222216/cg15811371	0.60867806	0.529651506	0.458486075	-0.150191985	3.19E-07	1.76E-06
TSS1500	MUSK	9	113430005	1	cg01737465	0.782561555	0.74197165	0.588620055	-0.1939415	3.28E-05	0.000107802
TSS1500	MYCT1	6	153018721	1	cg02830467	0.61936345	0.468785022	0.323156964	-0.296206486	1.12E-09	1.51E-08
TSS1500	MYEOV	11	69061344/69060542/69061244	3	cg01638792/cg13213814/cg26548122	0.755805578	0.664958744	0.594016741	-0.161788837	6.23E-08	4.20E-07
TSS1500	MYF6	12	81100403/81101022/81100305	3	cg08352786/cg15166296/cg25178519	0.73796728	0.631542031	0.566700609	-0.171266671	2.17E-10	4.29E-09
TSS1500	MYH14	19	50705432/50706387/50706483/50706536/50706577	5	cg00919509/cg03344031/cg06182329/cg11558731/cg25311666	0.438303566	0.309824556	0.287559616	-0.15074395	1.51E-09	1.91E-08
TSS1500	MYH15	3	108249446	1	cg03110703	0.696281105	0.566756444	0.432034318	-0.264246787	1.58E-09	1.97E-08
TSS1500	MYL1	2	211168547/211180386/211169638	3	cg13027933/cg22904096/cg27305342	0.653161028	0.569141898	0.484481033	-0.168679995	1.21E-06	5.62E-06
TSS1500	MYOD1	11	17740887/17740430	2	cg04499648/cg24322623	0.214798545	0.300851656	0.403722645	0.1889241	6.94E-06	2.65E-05
TSS1500	NALCN	13	102069132/102069251/102069556/102069296/102069169/102069257	6	cg01105058/cg04117435/cg11147919/cg11621911/cg18115507/cg18686498	0.247045602	0.313659702	0.445308149	0.198262547	3.62E-05	0.000117555
TSS1500	NAPEPLD	7	102790168/102790119/102789948/102790406/102790112	5	cg08159594/cg08904363/cg08905662/cg11692070/cg19273773	0.54446077	0.418115428	0.306560248	-0.237900522	3.94E-13	8.13E-11
TSS1500	NAT2	8	18248453	1	cg14494313	0.549401215	0.413995506	0.327277118	-0.222124097	4.54E-12	3.51E-10
TSS1500	NBLA00301	4	174451398/174451251/174450722/174450353/174450408/174451394/174451141	7	cg00443971/cg02774439/cg10541864/cg15707093/cg19178853/cg21530280/cg25102370	0.237734421	0.310234254	0.429972749	0.192238328	4.57E-06	1.83E-05

TSS1500	NCAM2	21	22370347/22369485/22370237/22369908/22369802/22370046/22369310	7	cg12426234/cg13062913/cg13297960/cg18128969/cg18492126/cg19556343/cg21160372	0.398778226	0.47196108	0.55456889	0.155790665	2.73E-07	1.54E-06
TSS1500	NCR3	6	31561515/31561732	2	cg03686228/cg15311651	0.862665245	0.79043275	0.7108108	-0.151854445	1.93E-07	1.14E-06
TSS1500	NCRNA00052	15	88119834/88119539	2	cg04579183/cg13382840	0.785534115	0.722269833	0.589440864	-0.196093251	4.38E-05	0.000139819
TSS1500	NCRNA00093	10	101686538/101686326	2	cg10727259/cg27434467	0.45689218	0.351860589	0.274728473	-0.182163707	1.39E-10	3.08E-09
TSS1500	NCRNA00157	21	19258310	1	cg26060835	0.500227875	0.346046228	0.238001077	-0.262226798	4.12E-15	1.30E-11
TSS1500	NCRNA00161	21	29910762/29910411	2	cg24617741/cg25912938	0.785438628	0.714075372	0.618457255	-0.166981373	3.20E-08	2.37E-07
TSS1500	NCRNA00162	21	46424976/46424880/46424860	3	cg07073072/cg13136556/cg17843722	0.465455965	0.376709767	0.308664117	-0.156791848	1.05E-11	5.47E-10
TSS1500	NDRG4	16	58497236/58498151/58497395/58497815/58497230/58498190/58497239/58498456/58498534/58497801/58497714/58497767/58496542	13	cg00687686/cg00984694/cg01466678/cg02040433/cg04190807/cg04797985/cg04942472/cg05469759/cg08384171/cg08791131/cg11306587/cg13031432/cg27147718	0.145856594	0.232042654	0.31887565	0.173019056	5.98E-05	0.000184782
TSS1500	NEFH	22	29875592/29875578/29875947/29875784	4	cg01907106/cg09828714/cg16042149/cg22766080	0.502167125	0.540143189	0.658142485	0.15597536	1.35E-06	6.17E-06
TSS1500	NEFM	8	24772137/24771645/24771259/24771326/24771114/24771125/24771273/24770381/24771466/24770342	10	cg02106941/cg02761376/cg07502389/cg07552803/cg09234518/cg16459364/cg18267374/cg18898125/cg23290344/cg26330518	0.404633198	0.469540744	0.599201504	0.194568306	5.20E-09	5.20E-08
TSS1500	NEK3	13	52734432/52734018/52734154/52734032/52734071/52733860/52735075/52734134	8	cg00595898/cg01345727/cg06906869/cg12277225/cg12861503/cg14572307/cg19524009/cg27065979	0.660014018	0.54014406	0.461945924	-0.198068094	1.82E-11	7.71E-10

TSS1500	NELL1	11	20690807/20690682/20690223/20690720/20690628	5	cg02510267/cg06873316/cg10964385/cg14749465/cg22769941	0.311200314	0.395912428	0.53861594	0.227415626	3.48E-08	2.55E-07
TSS1500	NENF	1	212605266/212605929/212604918/212605702	4	cg02783662/cg02901139/cg10487521/cg11836829	0.45540438	0.370738194	0.295411301	-0.159993079	1.81E-10	3.75E-09
TSS1500	NETO1	18	70534316/70535336/70535925/70534110/70534298/70535406	6	cg03524083/cg12921750/cg21922574/cg23505303/cg23883696/cg24863335	0.392586818	0.480920291	0.592840855	0.200254037	3.85E-11	1.30E-09
TSS1500	NF1P1	15	21135690/21135675	2	cg03606214/cg18714358	0.543297948	0.442067992	0.329205445	-0.214092502	2.42E-12	2.44E-10
TSS1500	NHLRC1	6	18123224/18123164/18123232	3	cg06646708/cg18068140/cg18232313	0.66611549	0.53489405	0.475232498	-0.190882992	4.02E-06	1.63E-05
TSS1500	NHP2	5	177581512/177581506/177581603/177581310/177581713/177581771	6	cg00273124/cg05654329/cg07869651/cg08525345/cg12634840/cg24546426	0.624976474	0.526433708	0.422183344	-0.202793129	5.55E-12	3.92E-10
TSS1500	NINJ2	12	772966/772982/773616	3	cg15884713/cg25089903/cg26549831	0.751122928	0.646556548	0.571294258	-0.179828671	7.18E-11	1.92E-09
TSS1500	NIPAL2	8	99306953/99306870	2	cg00330789/cg09965939	0.39312997	0.309941272	0.241287105	-0.151842865	3.10E-12	2.81E-10
TSS1500	NIPSNAP1	22	29977949/29977677/29978158/29977742	4	cg00266865/cg00939432/cg08492625/cg13797031	0.692148885	0.533068886	0.442208673	-0.249940212	4.64E-12	3.51E-10
TSS1500	NKAIN2	6	124124620/124124275/124123931/124123898/124123960	5	cg04856292/cg05343073/cg12851429/cg18457076/cg22375674	0.109640991	0.171199606	0.279320977	0.169679986	0.000370132	0.000967074
TSS1500	NKAIN4	20	61886262/61886142/61886587/61886313	4	cg07675169/cg07986239/cg13657510/cg14877226	0.273636822	0.401481094	0.550131777	0.276494955	5.59E-07	2.87E-06
TSS1500	NKAPL	6	28226203/28226885/28226094	3	cg00482232/cg21251018/cg26881102	0.787481103	0.701836978	0.584997023	-0.20248408	2.96E-09	3.29E-08
TSS1500	NKX2-6	8	23565098/23564156/23564591/23564294/23564717/23564193	6	cg03495753/cg03694713/cg03749016/cg10603004/cg16544169/cg22747746	0.412060697	0.527145658	0.654465984	0.242405287	9.66E-11	2.39E-09
TSS1500	NLRP11	19	56349119/56348742/56348889	3	cg02345758/cg02861926/cg22747516	0.425095376	0.329939112	0.264604368	-0.160491008	1.51E-08	1.25E-07

TSS1500	NMBR	6	142410216/142410235/142410221	3	cg13202845/cg18425786/cg21460020	0.342598663	0.382262107	0.546260842	0.203662179	1.03E-06	4.91E-06
TSS1500	NOL12	22	38081176/38082108/38081493	3	cg14370507/cg14398464/cg22123728	0.57919944	0.473608478	0.386252334	-0.192947106	1.03E-10	2.49E-09
TSS1500	NOTCH4	6	32192510/32193107/32193235/32192350	4	cg02662596/cg03250947/cg07010337/cg07822788	0.70264848	0.609518224	0.506467755	-0.196180725	1.42E-08	1.19E-07
TSS1500	NOV	8	120428057/120427810	2	cg05020510/cg15718811	0.42107289	0.345437442	0.268170941	-0.152901949	1.38E-10	3.07E-09
TSS1500	NOVA1	14	27067707/27067895/27068354/27068093/27067638/27067372/27068135	7	cg02014003/cg07543626/cg11146971/cg16791424/cg18468511/cg20478129/cg23502778	0.274856558	0.292430327	0.457206281	0.182349723	3.85E-05	0.000124268
TSS1500	NPAS4	11	66188019/66188203/66187533	3	cg02055483/cg09835239/cg24435401	0.218762555	0.328106943	0.536197375	0.317434821	4.56E-09	4.68E-08
TSS1500	NPBWR1	8	53852101/53851613/53852184/53852030/53851707/53851156	6	cg02115041/cg07629017/cg15531403/cg16330450/cg24857620/cg26205771	0.253709521	0.413337796	0.606035915	0.352326394	1.62E-10	3.43E-09
TSS1500	NPHP1	2	110963774	1	cg25350097	0.760918895	0.700804217	0.554123123	-0.206795772	4.80E-06	1.91E-05
TSS1500	NPHS1	19	36343530/36344293/36343521	3	cg14327228/cg19519626/cg21459969	0.6956954	0.612915528	0.524694682	-0.171000718	0.000342403	0.00090075
TSS1500	NPS	10	129346680/129346391	2	cg02304033/cg09640621	0.57664421	0.469273547	0.363227489	-0.213416721	1.41E-10	3.10E-09
TSS1500	NPTX1	17	78451573/78450855/78451349/78451563	4	cg13391810/cg17775235/cg19177795/cg25924217	0.137769768	0.176656147	0.325586605	0.187816837	0.00014822	0.000419812
TSS1500	NPTX2	7	98245830/98246006/98245716/98246001	4	cg00548268/cg08315202/cg09596818/cg13314145	0.416650292	0.435665248	0.592178547	0.175528255	0.000281576	0.000753013
TSS1500	NPY	7	24323128/24323559/24323261	3	cg01050885/cg05158615/cg18106668	0.349575665	0.509746457	0.621323027	0.271747362	1.18E-10	2.76E-09
TSS1500	NPY2R	4	156128904/156129574/156129169/156129081/156129424	5	cg00954382/cg06812991/cg07121547/cg07707546/cg18930994	0.230257255	0.30646618	0.417696499	0.187439244	5.36E-06	2.10E-05
TSS1500	NR2E1	6	108486517/108486387/108486450/108486773/108486123/108485837/108486007	7	cg03950614/cg03958979/cg08640634/cg12463600/cg18710462/cg20652607/cg21239311	0.325656174	0.404558707	0.563828238	0.238172064	1.26E-08	1.08E-07

TSS1500	NR2E3	15	72101672/72102213/72101891	3	cg04664328/cg07931844/cg16586105	0.510809095	0.406143444	0.324469854	-0.186339241	1.05E-13	4.63E-11
TSS1500	NRBF2	10	64892616/64892673/64892741	3	cg05393736/cg13216112/cg23221090	0.747390067	0.6172161	0.504231647	-0.24315842	1.19E-11	5.86E-10
TSS1500	NRG3	10	83634485/83636782/83636325/83636676/83636127/83633980/83634276/83636072/83636249/83633679	10	cg01151966/cg01164344/cg02100674/cg03480666/cg05087983/cg07023791/cg10401879/cg18988560/cg22928016/cg23255963	0.193393267	0.234592236	0.357821913	0.164428646	5.51E-08	3.78E-07
TSS1500	NRXN1	2	51260399/50575324/50575326/50575098/51260003/51260739	6	cg00345522/cg02495518/cg04707706/cg07529754/cg13999106/cg23273041	0.445478344	0.512897301	0.597293583	0.151815239	1.49E-06	6.76E-06
TSS1500	NSMCE4A	10	123735128	1	cg09536375	0.52175561	0.452216628	0.327232327	-0.194523283	3.36E-09	3.63E-08
TSS1500	NTRK3	15	88799973/88800510/88800567/88799999/88801004/88800624	6	cg04627496/cg04920689/cg05901579/cg07252731/cg11108676/cg14384532	0.170978061	0.318451373	0.507354069	0.336376008	2.28E-08	1.78E-07
TSS1500	NTSR1	20	61339804/61339871/61339875	3	cg02658437/cg03567830/cg11877251	0.172778239	0.226102439	0.338334882	0.165556643	0.000374719	0.00097825
TSS1500	NUB1	7	151037549/151038446/151038594/151037527	4	cg02217041/cg03480605/cg19553910/cg21639188	0.508299146	0.445958303	0.348054188	-0.160244959	4.67E-09	4.78E-08
TSS1500	OAS1	12	113343409/113343413/113343603	3	cg09151598/cg17445535/cg25668626	0.605758568	0.486313428	0.39123885	-0.214519718	1.15E-11	5.74E-10
TSS1500	OC90	8	133072665/133072043/133072873/133072269	4	cg02759725/cg21594839/cg23399286/cg25626375	0.493540953	0.339717638	0.227361109	-0.266179843	3.41E-16	3.24E-12
TSS1500	OCEL1	19	17336525/17336467/17336377	3	cg00003818/cg19461907/cg25689649	0.532605983	0.424502522	0.341566345	-0.191039637	2.68E-12	2.65E-10
TSS1500	OCLN	5	68787760/68787696/68787747	3	cg17772163/cg17818912/cg21126626	0.490919378	0.410403022	0.318510425	-0.172408953	6.94E-09	6.50E-08
TSS1500	OCM2	7	97620915/97620123/97620777	3	cg12844913/cg14260458/cg26262988	0.470654058	0.380728544	0.282407012	-0.188247046	2.96E-12	2.76E-10
TSS1500	ODF3	11	195572/195323/195390	3	cg10335112/cg13470155/cg26190363	0.586654625	0.475777517	0.377634736	-0.209019889	1.36E-10	3.03E-09

TSS1500	ODZ2	5	166710846/166711594/166710712	3	cg01227558/cg09171112/cg12659981	0.582959705	0.508615989	0.389516866	-0.193442839	4.08E-08	2.92E-07
TSS1500	ODZ4	11	79152969/79152112/79152102/79152821/79151966	5	cg00908927/cg02409108/cg12246510/cg22782986/cg25965355	0.256022576	0.344062704	0.483050142	0.227027566	3.78E-07	2.04E-06
TSS1500	OLFM1	9	137966584/137966498	2	cg08268099/cg21222830	0.54100499	0.456826472	0.384505495	-0.156499495	6.08E-08	4.12E-07
TSS1500	OLFM4	13	53602266/53602385/53601944	3	cg01098981/cg08119452/cg10246520	0.593189043	0.44778	0.39081028	-0.202378763	1.93E-09	2.32E-08
TSS1500	OLIG2	21	34396986/34396944/34397784/34397135/34397654	5	cg07601542/cg08729810/cg10217445/cg16713743/cg22593533	0.293543595	0.340770936	0.448347443	0.154803848	3.07E-06	1.28E-05
TSS1500	OLIG3	6	137816623/137817008/137816620/137816475/137816901	5	cg01889237/cg02799448/cg15324651/cg20336981/cg26672688	0.213087572	0.254771144	0.373065908	0.159978336	1.51E-07	9.18E-07
TSS1500	ONECUT1	15	53083238/53082477/53083587/53083532/53082902/53083518/53083135	7	cg01196531/cg02061705/cg06657050/cg13877670/cg15628498/cg20056542/cg26158897	0.294836457	0.431906515	0.589879428	0.295042971	8.67E-11	2.20E-09
TSS1500	OPRK1	8	54164757/54164442/54165371	3	cg01281904/cg09816500/cg20336122	0.275814	0.379214372	0.510390523	0.234576523	9.02E-06	3.36E-05
TSS1500	OR10A4	11	6897491/6896603	2	cg03898365/cg15375826	0.38460844	0.280502089	0.2197564	-0.16485204	1.00E-07	6.35E-07
TSS1500	OR10A7	12	55613318	1	cg26442740	0.702508535	0.621660633	0.458238027	-0.244270508	1.99E-06	8.76E-06
TSS1500	OR10AG1	11	55736968/55737045/55737072	3	cg05343512/cg18099058/cg22724854	0.610757173	0.492530581	0.388895602	-0.221861572	2.31E-12	2.35E-10
TSS1500	OR10J1	1	159409207/159408079	2	cg15700197/cg23689219	0.660124183	0.513700247	0.359709559	-0.300414623	3.60E-13	7.96E-11
TSS1500	OR10J5	1	159507078/159506621/159506033/159506559/159507162	5	cg06251978/cg07037019/cg12068949/cg15015426/cg26043322	0.563549202	0.486898869	0.386419456	-0.177129746	3.08E-09	3.39E-08
TSS1500	OR10K2	1	158391119	1	cg01733284	0.809446245	0.676295411	0.484221795	-0.32522445	2.01E-07	1.18E-06
TSS1500	OR10P1	12	56030437/56030407/56030242	3	cg03072740/cg20228385/cg26148774	0.73461338	0.664023774	0.567318432	-0.167294948	1.52E-05	5.40E-05
TSS1500	OR10Z1	1	158575773/158575177	2	cg04436634/cg25940557	0.551617035	0.422707394	0.367883907	-0.183733128	1.19E-10	2.78E-09
TSS1500	OR12D2	6	29364174	1	cg10916646	0.8041645	0.762870067	0.652826555	-0.151337945	0.002946118	0.006411979

TSS1500	OR13A1	10	45811730/45811882	2	cg08244656/cg16348164	0.850205443	0.789640889	0.68926543	-0.160940013	6.22E-07	3.16E-06
TSS1500	OR13C3	9	107299308	1	cg06565452	0.6851264	0.616573333	0.498076218	-0.187050182	1.75E-05	6.14E-05
TSS1500	OR1A2	17	3100171/3099847	2	cg03271520/cg27429194	0.601605063	0.492386736	0.380554532	-0.221050531	3.18E-10	5.71E-09
TSS1500	OR1E2	17	3337719/3337590	2	cg10231746/cg26048334	0.513915868	0.413014469	0.289777491	-0.224138377	1.13E-11	5.66E-10
TSS1500	OR1G1	17	3031874/3031618/3031354/3031450	4	cg00085430/cg02499925/cg06882926/cg26313699	0.623778385	0.531348825	0.427514652	-0.196263733	1.80E-09	2.18E-08
TSS1500	OR2A5	7	143746979/143746594/143746413	3	cg07119136/cg18845598/cg19437001	0.661934	0.516021297	0.397315886	-0.264618114	1.55E-10	3.33E-09
TSS1500	OR2AG1	11	6805810/6805701/6804983	3	cg02634507/cg04895001/cg12186981	0.524300233	0.434514633	0.355036635	-0.169263598	5.72E-10	8.85E-09
TSS1500	OR2B11	1	247616523/247615658/247616686/247615683	4	cg00112952/cg06852298/cg14830002/cg21302594	0.638484377	0.559944263	0.480157585	-0.158326792	1.07E-10	2.56E-09
TSS1500	OR2B3	6	29055885	1	cg16908601	0.569006465	0.419974078	0.288868686	-0.280137779	1.85E-14	2.04E-11
TSS1500	OR2C3	1	247697875/247697502/247698341/247698186	4	cg02795546/cg20320823/cg22699727/cg23016600	0.656873617	0.576313594	0.492141241	-0.164732376	9.28E-14	4.52E-11
TSS1500	OR2F1	7	143655754/143655651/143656153	3	cg07839906/cg13050390/cg21275920	0.666483783	0.568414586	0.471344682	-0.195139101	1.96E-08	1.56E-07
TSS1500	OR2G3	1	247767976	1	cg09766710	0.63676276	0.581245239	0.448602795	-0.188159965	4.20E-05	0.00013476
TSS1500	OR2G6	1	248683682/248684628	2	cg03623288/cg07395806	0.78161718	0.719056036	0.625789348	-0.155827832	1.00E-09	1.38E-08
TSS1500	OR2J2	6	29140768/29140622/29140126	3	cg13169479/cg13299436/cg27191906	0.734226688	0.641388881	0.568359573	-0.165867115	6.37E-09	6.10E-08
TSS1500	OR2L8	1	248111283/248110661	2	cg10580513/cg18857759	0.613446275	0.493381778	0.319458732	-0.293987543	1.23E-14	1.87E-11
TSS1500	OR2M2	1	248342467/248342534	2	cg10515630/cg22490229	0.409496655	0.2953628	0.206906645	-0.20259001	8.31E-13	1.29E-10
TSS1500	OR2M3	1	248365229	1	cg23028721	0.60097878	0.4924264	0.418715382	-0.182263398	4.82E-11	1.51E-09
TSS1500	OR2T10	1	248758128	1	cg22673542	0.81877778	0.724369522	0.614701241	-0.204076539	2.29E-05	7.79E-05
TSS1500	OR2T33	1	248438569	1	cg06400301	0.35495367	0.269525494	0.198565408	-0.156388262	1.95E-12	2.06E-10
TSS1500	OR2T34	1	248738984/248738754	2	cg04102905/cg13441213	0.39234342	0.292704539	0.201744141	-0.190599279	2.96E-14	2.34E-11
TSS1500	OR2T6	1	248549941/248549671	2	cg02077084/cg06846214	0.646097265	0.518750161	0.390261425	-0.25583584	2.03E-11	8.33E-10
TSS1500	OR2W1	6	29014046/29013336	2	cg05779068/cg25133685	0.457924378	0.369909933	0.250572923	-0.207351455	5.60E-12	3.93E-10
TSS1500	OR4A47	11	48509778/48509488	2	cg13319761/cg17017814	0.826385585	0.787807428	0.66361185	-0.162773735	2.15E-05	7.34E-05

TSS1500	OR4D11	11	59269966/59270409/59270264	3	cg17165818/cg20181079/cg21943004	0.76043115	0.7200874	0.588896523	-0.171534627	0.000172515	0.000483359
TSS1500	OR4D2	17	56245848/56246650/56246435	3	cg02874396/cg10233463/cg14819820	0.766537472	0.696998965	0.603597921	-0.16293955	3.15E-09	3.45E-08
TSS1500	OR4D6	11	59224044/59223996	2	cg17257444/cg23713772	0.709235645	0.614718789	0.543353427	-0.165882218	1.99E-05	6.86E-05
TSS1500	OR4K5	14	20387292	1	cg06749213	0.76080899	0.660395411	0.558850545	-0.201958445	4.70E-06	1.87E-05
TSS1500	OR51B2	11	5346249/5346163	2	cg14269191/cg21057473	0.6091635	0.480761589	0.343335405	-0.265828095	1.14E-12	1.54E-10
TSS1500	OR51B4	11	5324199	1	cg26800915	0.508702785	0.362163839	0.244985814	-0.263716971	1.16E-13	4.78E-11
TSS1500	OR51B5	11	5365377/5364972	2	cg07568203/cg20490724	0.65111691	0.554630022	0.465040523	-0.186076387	6.47E-07	3.27E-06
TSS1500	OR51B6	11	5372503	1	cg12930930	0.37852885	0.272639311	0.193925141	-0.184603709	1.23E-11	5.99E-10
TSS1500	OR51I2	11	5474001	1	cg12687069	0.76563654	0.726511311	0.595507877	-0.170128663	9.06E-06	3.37E-05
TSS1500	OR51S1	11	4871411/4871813	2	cg15781504/cg20867746	0.58657604	0.515343631	0.374817345	-0.211758695	7.64E-08	5.02E-07
TSS1500	OR52A1	11	5174413	1	cg25310592	0.61949272	0.520463283	0.385828859	-0.233663861	4.62E-06	1.85E-05
TSS1500	OR52A4	11	5143555/5144101	2	cg12593634/cg26365623	0.585631025	0.498621708	0.417428286	-0.168202739	1.24E-08	1.06E-07
TSS1500	OR52B2	11	6192419/6192943/6192512	3	cg17163363/cg20709280/cg21966764	0.325565596	0.215716622	0.15523722	-0.170328376	3.24E-13	7.87E-11
TSS1500	OR52K2	11	4469604/4469073/4469894	3	cg05117324/cg12250761/cg23562479	0.577747605	0.486708467	0.397521538	-0.180226068	1.60E-06	7.20E-06
TSS1500	OR56A1	11	6049729	1	cg18512262	0.57736578	0.416929178	0.255862718	-0.321503062	2.82E-14	2.33E-11
TSS1500	OR56A3	11	5967520	1	cg25451456	0.610336965	0.454748644	0.318152514	-0.292184451	1.19E-10	2.78E-09
TSS1500	OR5AU1	14	21624639/21625063/21625173	3	cg07555731/cg11725400/cg13816428	0.470629048	0.382931557	0.274508099	-0.196120949	7.74E-11	2.02E-09
TSS1500	OR5D14	11	55561545	1	cg21052687	0.415296465	0.317666189	0.231353923	-0.183942542	2.25E-10	4.39E-09
TSS1500	OR5D16	11	55605161	1	cg25384000	0.47912568	0.368980472	0.259364377	-0.219761303	2.68E-11	1.01E-09
TSS1500	OR5H15	3	97886467/97886603	2	cg01495831/cg03507018	0.5079947	0.339622783	0.22202145	-0.28597325	2.34E-13	7.11E-11
TSS1500	OR5I1	11	55704601/55704364/55705284	3	cg05421392/cg15717907/cg17120792	0.69979639	0.584795139	0.505317734	-0.194478656	2.72E-08	2.07E-07
TSS1500	OR5K2	3	98216207	1	cg04490079	0.78112885	0.6731842	0.527136227	-0.253992623	3.47E-08	2.54E-07
TSS1500	OR5L1	11	55577775	1	cg05788138	0.43409608	0.315650428	0.232912597	-0.201183483	1.09E-13	4.63E-11
TSS1500	OR5M1	11	56381677	1	cg26600826	0.78159673	0.690647444	0.596866755	-0.184729975	3.17E-06	1.32E-05
TSS1500	OR5M3	11	56239108	1	cg21570611	0.73608132	0.654032872	0.512018095	-0.224063225	3.84E-06	1.57E-05
TSS1500	OR5P2	11	7819874/7819960/7819812	3	cg05957546/cg06921176/cg21732056	0.660994498	0.583950753	0.497356861	-0.163637636	2.07E-08	1.63E-07

TSS1500	OR5P3	11	7847876/7848885/7848142	3	cg16226642/cg17086533/cg25541968	0.468963508	0.342519611	0.236386068	-0.232577439	2.35E-16	3.24E-12
TSS1500	OR5T3	11	56018532	1	cg14284630	0.551362685	0.359413183	0.227832333	-0.323530352	6.82E-14	3.81E-11
TSS1500	OR5V1	6	29324414/29324300/29325117/29325133	4	cg07150255/cg18949521/cg20141359/cg24076830	0.676405032	0.5926789	0.495186223	-0.181218809	3.91E-07	2.11E-06
TSS1500	OR6B3	2	240986651/240986437/240986376	3	cg05740739/cg18794622/cg19821536	0.675063407	0.594787409	0.490746942	-0.184316464	1.77E-10	3.68E-09
TSS1500	OR6C74	12	55639928/55640113	2	cg09358376/cg12548176	0.539952325	0.4403373	0.308674359	-0.231277966	1.82E-10	3.75E-09
TSS1500	OR6K2	1	158671873	1	cg04237529	0.786127795	0.720068622	0.574184814	-0.211942981	7.75E-06	2.92E-05
TSS1500	OR6K3	1	158689263/158688926/158688775/158689141/158688804	5	cg00920395/cg05369553/cg08918576/cg09318068/cg12811284	0.656165129	0.569626347	0.476177247	-0.179987882	8.64E-12	5.01E-10
TSS1500	OR6P1	1	158534143/158534679	2	cg04386839/cg20778958	0.53787173	0.458221153	0.377921573	-0.159950157	1.66E-10	3.49E-09
TSS1500	OR7A5	19	14940319	1	cg06639544	0.641888165	0.572123094	0.456431364	-0.185456801	8.39E-06	3.14E-05
TSS1500	OR7G3	19	9238958	1	cg03494634	0.49909479	0.375233094	0.270939959	-0.228154831	6.34E-09	6.08E-08
TSS1500	OR8A1	11	124438770/124439146	2	cg24693160/cg27049594	0.441613575	0.31723085	0.244323223	-0.197290352	7.01E-11	1.88E-09
TSS1500	OR8B12	11	124414205	1	cg24360034	0.568804685	0.433410094	0.285303991	-0.283500694	5.46E-11	1.62E-09
TSS1500	OR8B2	11	124254360/124253666	2	cg13012115/cg22089701	0.691964098	0.551748556	0.431076286	-0.260887811	9.21E-10	1.28E-08
TSS1500	OR8D2	11	124191389/124190997	2	cg04629433/cg15952370	0.544132685	0.465419733	0.256743609	-0.287389076	3.75E-10	6.46E-09
TSS1500	OR8H1	11	56059643	1	cg22629065	0.400954475	0.230849657	0.166024259	-0.234930216	1.73E-12	2.01E-10
TSS1500	OSTN	3	190929946	1	cg06976994	0.849629745	0.7469692	0.641975355	-0.20765439	9.45E-07	4.54E-06
TSS1500	OTOA	16	21694834/21715960/21694626/21715860	4	cg04651601/cg07084143/cg08964929/cg27514538	0.739613886	0.655108318	0.5742688	-0.165345086	1.86E-12	2.06E-10
TSS1500	OTOP1	4	4229061/4229810/4229883	3	cg03160466/cg06658468/cg20849299	0.347108885	0.414799181	0.581701818	0.234592933	7.14E-07	3.56E-06
TSS1500	OTOS	2	241080394/241080464/241081288	3	cg09186122/cg18790143/cg19795150	0.600215705	0.514112394	0.377325005	-0.2228907	3.21E-10	5.73E-09
TSS1500	OXT	20	3051954	1	cg04731988	0.565110025	0.466638878	0.399150264	-0.165959761	5.13E-09	5.15E-08
TSS1500	P2RX2	12	133195094/133193959/133195196/133195061	4	cg00058329/cg02315111/cg22368664/cg26620450	0.209241909	0.234090244	0.44044457	0.231202662	2.64E-06	1.12E-05
TSS1500	P2RX7	12	121569924/121570081/121569969	3	cg03248483/cg08688169/cg23801057	0.66978377	0.571548214	0.47603027	-0.1937535	4.64E-08	3.27E-07

TSS1500	PA2G4P4	3	156530506/156530658	2	cg08651937/cg25738116	0.64870593	0.536705056	0.459252914	-0.189453016	1.90E-09	2.29E-08
TSS1500	PABPC1P2	2	147344418/147344230	2	cg07167132/cg15973369	0.8605219	0.791493717	0.703893659	-0.156628241	0.000296246	0.000788465
TSS1500	PACSIN2	22	43343608/43343959/43343992	3	cg02940070/cg14859088/cg20522029	0.828972365	0.686343339	0.559136014	-0.269836351	4.19E-09	4.37E-08
TSS1500	PAEP	9	138452950/138452724	2	cg01055695/cg13831723	0.61616945	0.4870127	0.412020073	-0.204149377	3.15E-11	1.13E-09
TSS1500	PALM2	9	112402580/112402515	2	cg00752376/cg14102267	0.148940632	0.183175232	0.313359897	0.164419265	6.60E-06	2.53E-05
TSS1500	PALM3	19	14170785/14170956/14171165/14171331	4	cg05555515/cg06633013/cg11437328/cg21648202	0.465385665	0.385986	0.304704646	-0.160681019	1.12E-10	2.66E-09
TSS1500	PAMR1	11	35547548/35547903/35547514/35547519	4	cg14346208/cg14642338/cg20027133/cg27635267	0.368073558	0.424060173	0.518652397	0.150578839	4.30E-05	0.000137809
TSS1500	PANX3	11	124480990/124481193/124481218	3	cg05616355/cg08317412/cg11720054	0.577884335	0.527518525	0.410355043	-0.167529292	8.25E-08	5.37E-07
TSS1500	PAPPA2	1	176430983	1	cg11736104	0.488475425	0.365831606	0.266814614	-0.221660811	5.92E-11	1.68E-09
TSS1500	PAQR9	3	142683176/142682652/142683329/142683069/142682378/142682682	6	cg00970325/cg04912999/cg08113027/cg08784462/cg14081924/cg17627617	0.223809501	0.252425027	0.378689552	0.154880051	0.000318029	0.000841837
TSS1500	PARD6B	20	49347371/49346745/49346624/49346996	4	cg03077621/cg03812837/cg07803218/cg09072870	0.471656451	0.385844349	0.298160328	-0.173496123	1.22E-10	2.81E-09
TSS1500	PATE1	11	125615317/125615388	2	cg03455964/cg19345602	0.627274818	0.544619586	0.474143473	-0.153131345	6.73E-08	4.50E-07
TSS1500	PATE2	11	125649030/125649398/125649040	3	cg02827046/cg07747336/cg15229773	0.38344405	0.2918925	0.229487009	-0.153957041	9.61E-12	5.17E-10
TSS1500	PATE3	11	125657662/125656966/125657430	3	cg09427644/cg24859617/cg27129320	0.648229465	0.556081847	0.436058495	-0.21217097	1.38E-07	8.48E-07
TSS1500	PAX2	10	102504226/102504893/102504649/102504236/102505266/102504022/102504297/102504039	8	cg01275523/cg05044739/cg09222673/cg14096353/cg15416233/cg16443970/cg17945419/cg25523509	0.154330824	0.217752711	0.376641182	0.222310358	4.73E-07	2.48E-06
TSS1500	PAX7	1	18956762/18957020/18957232/18957023/18956947	5	cg04129469/cg05052324/cg07536847/cg08235970/cg25020286	0.288184994	0.35674689	0.480504976	0.192319982	2.69E-05	9.01E-05

TSS1500	PCBP3	21	47269627/47268670/47269180	3	cg07685034/cg11121969/cg12492289	0.644069435	0.563205506	0.478153968	-0.165915467	3.87E-07	2.09E-06
TSS1500	PCDH10	4	134069409/134070235/134069304/134069670/134070039/134069388/134069593/134069236	8	cg00945238/cg02043159/cg02114924/cg04035209/cg05401965/cg07665387/cg10196720/cg14146100	0.321056674	0.419374559	0.61695395	0.295897277	1.53E-10	3.29E-09
TSS1500	PCDH17	13	58205001/58204379/58205045/58204365/58205111	5	cg07430472/cg12606396/cg13806135/cg15112032/cg25891355	0.293968366	0.406563267	0.570533475	0.276565109	6.24E-11	1.74E-09
TSS1500	PCDH8	13	53423078/53423940/53424032/53424127/53423262/53424049/53423132/53423037	8	cg01382137/cg06114087/cg10323552/cg10484958/cg12545687/cg12568898/cg19456622/cg24560494	0.287963129	0.346247767	0.453926438	0.165963309	2.59E-05	8.73E-05
TSS1500	PCDHA11	5	140246511/140246626	2	cg08974687/cg19976449	0.625021505	0.553701083	0.450787459	-0.174234046	0.000354254	0.000929427
TSS1500	PCDHB13	5	140592867	1	cg14640659	0.53019649	0.422112661	0.332099091	-0.198097399	6.88E-10	1.02E-08
TSS1500	PCDHB15	5	140624869/140624874/140624184/140624888	4	cg03572772/cg17023770/cg17300966/cg22732749	0.52387506	0.59467537	0.682976882	0.159101822	1.37E-07	8.43E-07
TSS1500	PCDHB19P	5	140619446	1	cg19727801	0.42712357	0.509465556	0.595608027	0.168484457	0.000346368	0.000910121
TSS1500	PCDHB3	5	140479983/140479979/140479025	3	cg02125910/cg18782991/cg23918315	0.3560458	0.425231208	0.558751841	0.202706041	6.59E-06	2.53E-05
TSS1500	PCDHB6	5	140529580/140529627/140529499/140528785	4	cg09178190/cg15038110/cg24811352/cg26866168	0.410451715	0.477121828	0.574078646	0.163626931	2.04E-05	7.01E-05
TSS1500	PCDHGA5	5	140743575	1	cg07520649	0.476589265	0.527419161	0.635315509	0.158726244	0.002278614	0.005066138
TSS1500	PCDHGB3	5	140749735/140749745/140748559	3	cg02575448/cg04242499/cg17227466	0.372638778	0.490735672	0.636786955	0.264148177	6.04E-09	5.84E-08
TSS1500	PCDHGB4	5	140767197/140767242/140766911/140767200	4	cg02741229/cg22041640/cg23347399/cg26282384	0.366960643	0.434457358	0.585020359	0.218059717	2.02E-06	8.85E-06
TSS1500	PCDHGB5	5	140777473/140777344	2	cg11686427/cg25426815	0.374205635	0.391285904	0.576918885	0.20271325	1.86E-06	8.22E-06
TSS1500	PCDHGC4	5	140864276/140864474/140864020	3	cg06428620/cg12648074/cg26890354	0.26878578	0.380269639	0.540655073	0.271869293	1.71E-08	1.39E-07

TSS1500	PCK1	20	56135029/56134648/56135615	3	cg10278454/cg13655303/cg27661711	0.513722698	0.410655397	0.317222318	-0.196500379	2.25E-11	8.82E-10
TSS1500	PCSK2	20	17206999/17206946/17207337/17207246/17206529/17206720/17206245	7	cg00621913/cg01815529/cg02979923/cg06441668/cg15060366/cg19864758/cg20737505	0.263348347	0.293499158	0.429381111	0.166032765	2.84E-07	1.59E-06
TSS1500	PDE6C	10	95372040/95372069	2	cg02444484/cg16316382	0.70831645	0.581169372	0.465490445	-0.242826005	9.22E-11	2.31E-09
TSS1500	PDGFD	11	104035557/104035942/104035761	3	cg04228039/cg12686407/cg16322276	0.763762493	0.684322381	0.556202448	-0.207560045	6.25E-10	9.48E-09
TSS1500	PDPN	1	13909623/13910667/13910698/13910700/13909522/13910555/13910569/13909743/13909161/13910796/13909821	11	cg00831909/cg05140069/cg05281894/cg09804380/cg16590190/cg18877506/cg21819468/cg22798977/cg23954416/cg24671344/cg25286482	0.403051092	0.493909931	0.578509288	0.175458196	6.65E-07	3.35E-06
TSS1500	PENK	8	57360226/57359200/57359956/57359970/57359258/57359414/57360727/57360711/57360586/57360613/57359399/57360738/57359992/57358928/57359908	15	cg03650233/cg06066137/cg07080864/cg09015433/cg10397440/cg11060276/cg11610346/cg16072688/cg16219603/cg16419235/cg19149681/cg20787219/cg23350294/cg24645221/cg27531336	0.4137276	0.48479181	0.567386419	0.153658819	1.02E-07	6.45E-07
TSS1500	PEX5L	3	179755285/179755086/179755235/179754761	4	cg05131623/cg18780412/cg21176048/cg21564242	0.251006861	0.344533715	0.455294833	0.204287973	2.60E-08	1.99E-07
TSS1500	PFKFB2	1	207225647/207225128/207225424/207226112/207226369	5	cg01380361/cg02710090/cg06119874/cg17808646/cg20291513	0.552623797	0.424167892	0.329906596	-0.222717201	2.07E-12	2.14E-10
TSS1500	PGLYRP4	1	153322488/153321421/153322011/153321907	4	cg01093088/cg02956542/cg06954535/cg21691717	0.733646965	0.670511981	0.574906157	-0.158740808	1.86E-06	8.23E-06
TSS1500	PHF14	7	11012873/11013274/11012967	3	cg07230486/cg23083824/cg27578754	0.44488043	0.355290359	0.282614076	-0.162266354	4.28E-12	3.41E-10

TSS1500	PHGR1	15	40641810/40642998/40641778	3	cg01376665/cg07047589/cg21067750	0.447002958	0.341258356	0.271650589	-0.175352369	4.82E-13	9.15E-11
TSS1500	PHOX2A	11	71956145/71955547/71955439/71955599	4	cg01527777/cg08876932/cg16922279/cg24530250	0.301572288	0.360743145	0.521985263	0.220412975	7.42E-06	2.81E-05
TSS1500	PI3	20	43802992/43803224	2	cg02733351/cg19931348	0.547077243	0.396620872	0.291040902	-0.25603634	2.01E-13	6.78E-11
TSS1500	PI4KAP1	22	20399601/20399974/20400015	3	cg11814655/cg20969061/cg24289553	0.657877928	0.517192664	0.418995705	-0.238882223	1.21E-13	4.87E-11
TSS1500	PICK1	22	38452315/38452866	2	cg14332918/cg16760049	0.742848465	0.614979067	0.563908509	-0.178939956	2.46E-06	1.05E-05
TSS1500	PIGB	15	55610624	1	cg19991916	0.88449073	0.625994622	0.479371295	-0.405119435	1.04E-07	6.60E-07
TSS1500	PKD1L2	16	81254719/81255310/81254209	3	cg00163702/cg04175109/cg07091758	0.563397308	0.504784178	0.407313	-0.156084308	1.21E-10	2.81E-09
TSS1500	PKIA	8	79427993/79428117	2	cg04689061/cg17686936	0.133843346	0.214565327	0.341871826	0.20802848	1.92E-05	6.66E-05
TSS1500	PKP3	11	393606/393570/393938/393392/393038/392903/393648/393029/393328	9	cg03547062/cg03638937/cg05214427/cg09950681/cg10517281/cg15002541/cg18308516/cg20268054/cg20992002	0.663288687	0.588267227	0.511135906	-0.152152781	1.20E-09	1.59E-08
TSS1500	PLA2G12B	10	74714935/74714745	2	cg02044879/cg10842126	0.52159846	0.417312822	0.336302905	-0.185295555	1.30E-09	1.69E-08
TSS1500	PLA2G4B	15	42130662/42130650/42130735	3	cg07178968/cg13038025/cg18827756	0.532365117	0.422651831	0.342931191	-0.189433926	2.33E-11	8.97E-10
TSS1500	PLCB1	20	8112972/8112956/8112754	3	cg17510056/cg21844450/cg23657409	0.155928824	0.213287391	0.358267503	0.202338679	0.000574701	0.001444479
TSS1500	PLD5	1	242688320/242688226/242688681/242688591/242688523	5	cg04205107/cg08659667/cg12613383/cg18789663/cg22679728	0.257086537	0.276485099	0.425248052	0.168161515	0.000189003	0.000524521
TSS1500	PLEK2	14	67879385/67879101/67879126/67879166	4	cg14437634/cg18306861/cg24995657/cg26225496	0.404374269	0.312495453	0.252504709	-0.15186956	1.62E-09	2.01E-08
TSS1500	PLIN1	15	90223301/90224098/90223712	3	cg01348757/cg08749443/cg11526413	0.67200997	0.514776276	0.43185497	-0.240155	5.88E-12	4.07E-10
TSS1500	PNLIP	10	118304610/118304534	2	cg23728588/cg24571875	0.544443475	0.428520861	0.306191127	-0.238252348	2.22E-11	8.76E-10
TSS1500	PNLIPRP1	10	118349587/118349430	2	cg20730333/cg27600794	0.475173458	0.380116472	0.313393393	-0.161780064	3.69E-11	1.26E-09
TSS1500	PNLIPRP2	10	118380177/118380175	2	cg07093456/cg11310496	0.653975088	0.541016308	0.437239598	-0.21673549	3.14E-10	5.65E-09

TSS1500	PNLIPRP3	10	118187088/118187002/118186901	3	cg07037454/cg14539856/cg17197596	0.637080098	0.529848331	0.428697098	-0.208383	7.77E-10	1.12E-08
TSS1500	PNOC	8	28173887/28173732/28174192/28174350	4	cg05988605/cg19391527/cg22632756/cg27336360	0.536138543	0.441124608	0.378128732	-0.158009811	5.98E-10	9.13E-09
TSS1500	POLD3	11	74302893/74303294/74303012	3	cg01173485/cg08923614/cg26885816	0.678336867	0.512326711	0.399130685	-0.279206182	4.46E-12	3.50E-10
TSS1500	POM121L12	7	53102694/53102721	2	cg12333737/cg20170989	0.42947934	0.279301172	0.187995841	-0.241483499	8.06E-13	1.26E-10
TSS1500	PON3	7	95026095/95026106/95027158/95026248/95026097/95026181/95026073/95026937/95025924/95025905/95026502/95026538/95026211/95026066/95025955/95026672/95026121/95026193/95026090	19	cg03301582/cg04080282/cg04350520/cg08461772/cg08898155/cg10329418/cg11435506/cg11700490/cg15500865/cg15927196/cg18241308/cg20985067/cg24750391/cg25161512/cg25572105/cg25879118/cg26457160/cg27166921/cg27413563	0.410517907	0.386368056	0.248245585	-0.162272322	5.90E-09	5.74E-08
TSS1500	POU4F2	4	147559648/147559579/147559423/147558544	4	cg12935979/cg13262687/cg19597382/cg25970230	0.33730075	0.459647814	0.634065168	0.296764418	1.22E-09	1.60E-08
TSS1500	POU4F3	5	145717597/145717661/145717858/145717283/145717140/145717145/145718098	7	cg01386275/cg08531042/cg10627091/cg14099595/cg16101493/cg26932154/cg27309276	0.280609568	0.352104207	0.469329703	0.188720135	4.48E-06	1.80E-05
TSS1500	PPFIA4	1	203019520/203019107/203018871	3	cg03511671/cg04914198/cg19824396	0.631080523	0.533745854	0.438609358	-0.192471166	2.71E-10	5.07E-09
TSS1500	PPIAL4A	1	147956744	1	cg23350716	0.590929435	0.499748721	0.350470012	-0.240459423	0.000139014	0.000396402
TSS1500	PPIAL4B	1	147956744	1	cg23350716	0.590929435	0.499748721	0.350470012	-0.240459423	0.000139014	0.000396402
TSS1500	PPIAL4D	1	148203924	1	cg18072778	0.70183991	0.588887167	0.473528791	-0.228311119	3.80E-12	3.20E-10
TSS1500	PPIAL4F	1	148203924	1	cg18072778	0.70183991	0.588887167	0.473528791	-0.228311119	3.80E-12	3.20E-10
TSS1500	PPIL1	6	36843333/36843324	2	cg01910741/cg13939462	0.53291697	0.452263067	0.372321852	-0.160595118	4.21E-08	3.00E-07
TSS1500	PPP1R15A	19	49374447	1	cg19366381	0.764133405	0.659515422	0.595463086	-0.168670319	4.36E-09	4.51E-08
TSS1500	PPP1R2P1	6	32843055/32844032	2	cg13617603/cg22744098	0.57436797	0.467743344	0.305982527	-0.268385443	4.15E-14	3.03E-11

TSS1500	PPP3R2	9	104357967/104358101	2	cg14285012/cg15765694	0.7871305	0.684650894	0.609841055	-0.177289445	1.42E-06	6.49E-06
TSS1500	PPP4R2	3	73045556/73045440/73045686	3	cg03847932/cg06051312/cg20540428	0.798157715	0.585135433	0.506348109	-0.291809606	2.54E-06	1.08E-05
TSS1500	PRAMEF12	1	12833744/12834742	2	cg11346333/cg11737871	0.73273357	0.653420256	0.555544648	-0.177188922	5.27E-07	2.73E-06
TSS1500	PRB1	12	11509583	1	cg27345534	0.627720395	0.482526639	0.475575409	-0.152144986	4.08E-05	0.000131228
TSS1500	PRB2	12	11548867/11549192/11549555	3	cg20480899/cg23372297/cg24891312	0.516973713	0.334886022	0.339073968	-0.177899744	1.92E-05	6.65E-05
TSS1500	PREX2	8	68864318/68864247/68864012/68864179/68864324	5	cg05293738/cg11549615/cg13652336/cg16009633/cg17747005	0.212036291	0.282992822	0.431062793	0.219026502	4.54E-05	0.000144809
TSS1500	PRG2	11	57158970/57158361/57159174	3	cg05471009/cg15357945/cg15971518	0.580150103	0.4524716	0.354212243	-0.225937859	8.71E-10	1.23E-08
TSS1500	PRG3	11	57149840/57148918	2	cg04620833/cg23995769	0.70829775	0.630752489	0.496240977	-0.212056773	2.00E-07	1.17E-06
TSS1500	PRKG2	4	82127274/82126891	2	cg10060562/cg12977791	0.61671862	0.471055278	0.410353882	-0.206364738	8.47E-08	5.49E-07
TSS1500	PROCA1	17	27039523/27039120/27039132	3	cg04578090/cg07585069/cg18448746	0.375061977	0.511718469	0.604883885	0.229821908	6.77E-08	4.52E-07
TSS1500	PRODH2	19	36305584/36305049	2	cg17122301/cg27039606	0.7312012	0.623197111	0.556722855	-0.174478345	1.75E-05	6.11E-05
TSS1500	PROX2	14	75331272/75330792/75331970/75330790/75330798	5	cg02110830/cg10165162/cg10197640/cg20382622/cg21112954	0.705497983	0.621966173	0.541448418	-0.164049565	1.62E-11	7.24E-10
TSS1500	PRR18	6	166722248/166722165/166722773	3	cg01243879/cg04329390/cg05089968	0.55142445	0.424501033	0.334371664	-0.217052786	1.49E-11	6.82E-10
TSS1500	PRSS1	7	142456124	1	cg12878228	0.61023168	0.443697222	0.330479082	-0.279752598	1.26E-12	1.65E-10
TSS1500	PRSSL1	19	696859/696946/696832	3	cg07898360/cg10746622/cg18754695	0.63056396	0.510106139	0.414960555	-0.215603405	6.43E-12	4.20E-10
TSS1500	PRTG	15	56036455/56035454/56035459/56035463/56035476/56035616	6	cg04124457/cg06877762/cg13333185/cg13778723/cg18132228/cg20951555	0.286004209	0.301078656	0.438573546	0.152569337	0.00026932	0.000723192
TSS1500	PRTN3	19	839689/840737/840447/839678	4	cg00615241/cg09930046/cg17483523/cg24347562	0.494939495	0.412101494	0.319676868	-0.175262627	3.99E-11	1.32E-09
TSS1500	PSMB11	14	23510934/23511078/23511170	3	cg09857247/cg13487992/cg14291751	0.670911618	0.594378436	0.5102768	-0.160634818	1.53E-09	1.93E-08

TSS1500	PSMB4	1	151370645/151371309/151371285	3	cg04396915/cg23106844/cg25306093	0.709466485	0.574615767	0.4856838	-0.223782685	9.84E-11	2.42E-09
TSS1500	PTCRA	6	42882406/42883504	2	cg00047532/cg11898695	0.670440075	0.606883469	0.518140575	-0.1522995	3.51E-12	3.08E-10
TSS1500	PTF1A	10	23480190/23480237/23480002/23479993/23480730/23480018/23480316/23481176/23480803	9	cg02836919/cg04829946/cg06550984/cg09795588/cg17389519/cg19161112/cg20487710/cg22746058/cg25684999	0.338391577	0.381627901	0.499160176	0.160768599	6.63E-06	2.54E-05
TSS1500	PTH1R	3	46918266/46918356	2	cg03391568/cg06042608	0.58139015	0.485863922	0.404405191	-0.176984959	1.27E-09	1.66E-08
TSS1500	PTPN5	11	18814078/18814418/18813979	3	cg07217924/cg17233601/cg17250701	0.305738046	0.384040091	0.466929189	0.161191143	7.45E-08	4.91E-07
TSS1500	PTPRH	19	55721638/55721792/55721180/55721290	4	cg07097098/cg11613450/cg19673376/cg21559943	0.527590698	0.468796079	0.364999041	-0.162591657	1.68E-08	1.37E-07
TSS1500	PTPRQ	12	80836629	1	cg20238308	0.47658491	0.339737956	0.225000109	-0.251584801	1.09E-11	5.56E-10
TSS1500	PTPRT	20	41819125/41818788/41818770/41818905	4	cg04541293/cg07167168/cg17859110/cg21672843	0.184511835	0.266124741	0.432638811	0.248126976	1.95E-08	1.55E-07
TSS1500	PUM2	2	20527635/20527677/20527833	3	cg01888166/cg08659707/cg22528172	0.823101043	0.667000056	0.617046847	-0.206054196	3.26E-09	3.54E-08
TSS1500	PUS10	2	61246248	1	cg00326648	0.65441283	0.547016283	0.433104527	-0.221308303	7.17E-10	1.06E-08
TSS1500	PUS3	11	125774092/125774090/125774447/125774406/125774311/125774082	6	cg01736784/cg04609163/cg11413039/cg19792599/cg22097425/cg23727983	0.459958998	0.547179494	0.654676633	0.194717635	8.88E-11	2.24E-09
TSS1500	QKI	6	163834924/163834452/163834872/163834903/163835251/163835395/163834176/163835117	8	cg00852768/cg07618175/cg08169901/cg09854653/cg13695877/cg20930060/cg23954057/cg25157874	0.087814187	0.144904504	0.300689284	0.212875097	3.27E-05	0.00010736
TSS1500	QPCT	2	37570604/37571401/37571178	3	cg00881168/cg08240529/cg09386458	0.829521905	0.758092957	0.675023062	-0.154498843	8.14E-08	5.31E-07
TSS1500	RAB20	13	111215148/111214457/111214541	3	cg12386015/cg12736438/cg17935813	0.615138495	0.517934717	0.409811273	-0.205327222	5.68E-08	3.89E-07
TSS1500	RAB31	18	9708009/9707896/9707753/9707031	4	cg13462028/cg17982102/cg18456459/cg27298007	0.370245541	0.461836787	0.549050938	0.178805398	8.77E-09	7.93E-08

TSS1500	RAB36	22	23486678/23487048/23487027/23487104	4	cg05366982/cg14497203/cg15450768/cg20721738	0.427684495	0.318629989	0.246558759	-0.181125736	1.28E-12	1.65E-10
TSS1500	RABEPK	9	127962544/127961717/127962481	3	cg01211109/cg14222630/cg26753512	0.577314585	0.481293378	0.352772273	-0.224542312	1.87E-08	1.50E-07
TSS1500	RAD18	3	9005400/9006242/9005405/9005479	4	cg01884537/cg03287469/cg21902095/cg22371859	0.478882823	0.344610822	0.25333239	-0.225550432	1.73E-12	2.01E-10
TSS1500	RAD21L1	20	1205357/1206255/1205378	3	cg00435238/cg15575222/cg27346545	0.588269413	0.475656622	0.39385462	-0.194414792	1.62E-08	1.33E-07
TSS1500	RALYL	8	85096037/85095275/85094904/85096835/85094923/85094842/85094742/85094437/85096015/85096868/85095654/85095697	12	cg00034076/cg00266009/cg01134282/cg01538821/cg06955749/cg08327106/cg09757712/cg11089595/cg22403811/cg23458558/cg23777958/cg26669044	0.27086144	0.310675616	0.502710953	0.231849513	2.98E-07	1.66E-06
TSS1500	RAMP3	7	45196849/45196501/45196262	3	cg02792877/cg09314155/cg27027655	0.592349285	0.513128228	0.417991629	-0.174357656	5.48E-10	8.55E-09
TSS1500	RANBP17	5	170288757/170288766/170288255/170288742	4	cg01579024/cg02091185/cg05742691/cg08358803	0.631094554	0.527023968	0.472358027	-0.158736526	1.71E-08	1.39E-07
TSS1500	RASAL1	12	113574276/113574368/113575128	3	cg08865522/cg16890739/cg24109790	0.556637685	0.394471098	0.306957808	-0.249679877	2.58E-13	7.42E-11
TSS1500	RAX	18	56941706/56941473/56942066/56941246	4	cg00814752/cg03671802/cg06620569/cg27189087	0.284265084	0.355136394	0.461853657	0.177588573	1.13E-06	5.29E-06
TSS1500	REG1P	2	79366964/79366059/79365892	3	cg00689213/cg02657641/cg15359390	0.37879362	0.265920872	0.213292559	-0.165501061	3.59E-12	3.11E-10
TSS1500	REG4	1	120355361/120354655/120354772/120354493	4	cg06832956/cg11958962/cg15138570/cg19736110	0.811452353	0.725142541	0.594490276	-0.216962078	5.42E-07	2.80E-06
TSS1500	REP15	12	27848523	1	cg14128259	0.4861896	0.380428489	0.29947025	-0.18671935	1.19E-08	1.03E-07
TSS1500	RESP18	2	220199185/220199208/220199310	3	cg00793981/cg19020434/cg23985420	0.719000508	0.621389361	0.522786091	-0.196214417	1.15E-06	5.37E-06
TSS1500	RETN	19	7733401/7733174	2	cg08132525/cg20692181	0.76756135	0.681919586	0.611314436	-0.156246914	7.26E-11	1.93E-09
TSS1500	RETNLB	3	108476878/108476540	2	cg00003965/cg05163071	0.452974965	0.345343956	0.277469041	-0.175505924	2.18E-10	4.30E-09

TSS1500	RFTN2	2	198541655/198541772	2	cg00826638/cg22387019	0.729523938	0.550981583	0.404589005	-0.324934933	2.97E-12	2.76E-10
TSS1500	RGS21	1	192285333	1	cg00158654	0.79529358	0.666336306	0.512647895	-0.282645685	1.22E-09	1.61E-08
TSS1500	RGS5	1	163173262	1	cg02579477	0.720273725	0.600141856	0.493346264	-0.226927461	2.45E-06	1.05E-05
TSS1500	RGS6	14	72398920/72399553/72398856/72398839	4	cg14702655/cg15457745/cg16396954/cg23576118	0.132310638	0.143585694	0.298340158	0.16602952	9.34E-05	0.000278028
TSS1500	RGS7BP	5	63801834/63801893/63802184/63802106/63801847	5	cg03827337/cg05455931/cg08709073/cg12158471/cg14864853	0.289337943	0.367243305	0.480519267	0.191181324	7.12E-06	2.71E-05
TSS1500	RGS8	1	182643158/182643312/182642748	3	cg06894198/cg16349160/cg17124700	0.778956495	0.695825594	0.626056682	-0.152899813	6.88E-09	6.46E-08
TSS1500	RIC3	11	8190837/8190834/8190822	3	cg01560972/cg06327746/cg15524336	0.424478423	0.495988111	0.601982407	0.177503984	3.77E-10	6.47E-09
TSS1500	RIMS2	8	104512423/104512168/104512317/104511776	4	cg03350814/cg16648062/cg20129213/cg20426946	0.150552628	0.175882172	0.315339034	0.164786406	0.000544913	0.001376721
TSS1500	RNASE1	14	21270924/21270850/21271148/21271313/21271355/21271018/21272532	7	cg05958352/cg08122234/cg12940993/cg13718960/cg18832407/cg18836485/cg21908129	0.596407348	0.514317372	0.437191112	-0.159216236	3.35E-11	1.18E-09
TSS1500	RNASE11	14	21059287/21058901	2	cg06760771/cg19937577	0.724848255	0.615646178	0.483483082	-0.241365173	2.42E-08	1.87E-07
TSS1500	RNASE12	14	21060022/21060292/21059287/21060349	4	cg02647684/cg04957177/cg06760771/cg14149503	0.753956926	0.649277086	0.51817454	-0.235782386	2.56E-11	9.78E-10
TSS1500	RND1	12	49261012/49259997	2	cg04640834/cg22514963	0.611850365	0.476849194	0.376277832	-0.235572533	3.06E-11	1.11E-09
TSS1500	ROBO3	11	124735089/124734412	2	cg10680051/cg12398330	0.291793068	0.32671232	0.449011244	0.157218177	7.68E-07	3.80E-06
TSS1500	RPL13AP17	7	77975971/77975491	2	cg09196534/cg11407989	0.487634405	0.40765795	0.326397282	-0.161237123	2.63E-10	4.94E-09
TSS1500	RPL19P12	7	102783873/102784027/102784228	3	cg01946097/cg10767164/cg20256707	0.682760178	0.580220717	0.495319661	-0.187440516	6.30E-11	1.75E-09
TSS1500	RPL31P11	1	161655895	1	cg22445346	0.748590945	0.614487478	0.448657486	-0.299933459	4.03E-10	6.77E-09
TSS1500	RPL39L	3	186857475/186857593/186857537	3	cg07441272/cg07681728/cg13432744	0.118771249	0.177451184	0.313922096	0.195150847	0.00043417	0.001118069
TSS1500	RPTN	1	152132853/152132450/152132592	3	cg05765254/cg11535506/cg25304681	0.707785727	0.570031028	0.490223189	-0.217562537	4.87E-09	4.94E-08
TSS1500	RRN3P2	16	29084744/29085130	2	cg00525931/cg00821510	0.600590285	0.488272594	0.4472372	-0.153353085	4.76E-07	2.50E-06

TSS1500	RRP7A	22	42917251/42916332	2	cg04222463/cg19987256	0.508316435	0.388638333	0.335987332	-0.172329103	3.36E-09	3.63E-08
TSS1500	RSPO1	1	38100826/38100837/38100811/38101652/38100795/38101266	6	cg03654735/cg10573018/cg12391690/cg17161617/cg22063989/cg26519919	0.242804174	0.331854258	0.425898634	0.18309446	2.44E-05	8.25E-05
TSS1500	RSPO4	20	983130/983354	2	cg17870408/cg26515460	0.11937369	0.133190122	0.277627904	0.158254214	0.000682277	0.001687111
TSS1500	RTKN	2	74668477/74668072/74669380/74668672/74669349/74668976/74668286/74669375/74669516/74669387/74669048	11	cg00326131/cg00610692/cg00658082/cg00689340/cg03295928/cg03719070/cg09692695/cg14481208/cg19515035/cg26090072/cg26676034	0.460440434	0.384269086	0.307391244	-0.15304919	4.62E-10	7.51E-09
TSS1500	RXFP1	4	159442782/159441687	2	cg03875996/cg24035112	0.460707315	0.379733394	0.296914514	-0.163792801	6.68E-09	6.33E-08
TSS1500	RXFP3	5	33936262/33936253/33935027/33936171/33936232	5	cg00981837/cg07832473/cg08709576/cg10795738/cg18689958	0.184337113	0.239851174	0.341022532	0.156685419	0.000643651	0.001599139
TSS1500	RXFP4	1	155910958/155911238/155910526/155910967/155910523	5	cg04233230/cg08403419/cg21284779/cg22863889/cg25214914	0.496143702	0.41149893	0.337757391	-0.158386311	2.52E-10	4.80E-09
TSS1500	RYR2	1	237205295/237205174/237205409/237205098	4	cg03422911/cg07790615/cg18375860/cg19764418	0.452385778	0.41005682	0.648201086	0.195815309	1.95E-06	8.57E-06
TSS1500	RYR3	15	33602817/33602824/33602821/33602758	4	cg02186782/cg11789740/cg12860391/cg21413325	0.37828397	0.469135644	0.598814657	0.220530687	2.19E-10	4.32E-09
TSS1500	S100P	4	6694923/6695203/6695319	3	cg02883621/cg14323984/cg27027375	0.725823043	0.578028219	0.436999474	-0.288823569	5.87E-11	1.67E-09
TSS1500	S1PR4	19	3178413/3178512/3177310	3	cg02716776/cg02996471/cg20656868	0.450700283	0.372951108	0.29506796	-0.155632323	9.48E-06	3.51E-05
TSS1500	SAAL1	11	18128177/18127958/18128126	3	cg00345366/cg05093315/cg12377368	0.564864755	0.476098867	0.385892909	-0.178971846	5.34E-12	3.81E-10

TSS1500	SALL1	16	51185039/51186266/51185346/51185147/51185672/51184786/51185407/51186211/51185132/51185236/51185082/51185459/51184886/51185461/51185060/51186260/51185001/51185110/51185772/51185394/51186463	21	cg00124695/cg00177451/cg00310215/cg01146232/cg01500945/cg05151154/cg05213609/cg05404010/cg06232807/cg06274671/cg06653699/cg06724588/cg07498275/cg07502439/cg08439930/cg08776356/cg08806408/cg09016242/cg13755795/cg22674717/cg27423760	0.33784132	0.431377988	0.58118652	0.2433452	3.03E-09	3.35E-08
TSS1500	SALL3	18	76739409/76739226/76739559/76738999	4	cg05080154/cg13634602/cg16580499/cg21779611	0.227578384	0.312004714	0.426567904	0.198989519	1.33E-10	2.98E-09
TSS1500	SCARF2	22	20792476/20792535	2	cg08748948/cg14785479	0.279022718	0.351433703	0.511275693	0.232252976	2.37E-07	1.36E-06
TSS1500	SCEL	13	78109017/78108374/78108474	3	cg11215283/cg19734822/cg26389381	0.682622005	0.571589861	0.474082948	-0.208539057	4.94E-12	3.63E-10
TSS1500	SCGB1D1	11	61956390/61956523/61956860	3	cg04011620/cg14980165/cg26907473	0.792025305	0.700007933	0.624008441	-0.168016864	4.69E-05	0.000148801
TSS1500	SCGB1D2	11	62009065/62008949	2	cg10848367/cg19015654	0.418597895	0.333634672	0.244493755	-0.17410414	2.99E-13	7.87E-11
TSS1500	SCN3A	2	166061599/166061891	2	cg03180819/cg17754914	0.64390047	0.524578964	0.404113411	-0.239787059	4.95E-13	9.21E-11
TSS1500	SCN4A	17	62051709/62051773/62051767	3	cg05818824/cg14258078/cg25507121	0.500798332	0.406223619	0.316439573	-0.184358759	1.35E-11	6.42E-10
TSS1500	SCN7A	2	167343774/167344555	2	cg05331214/cg06746492	0.803588825	0.729972667	0.646842091	-0.156746734	0.000715033	0.001758756
TSS1500	SCNN1B	16	23313148/23313295/23313293/23313240	4	cg00925717/cg03102879/cg23113963/cg26590082	0.40002322	0.47441955	0.600356732	0.200333512	9.67E-07	4.64E-06
TSS1500	SCRN2	17	45919287/45919004/45919419	3	cg01390728/cg10924080/cg11242907	0.735759298	0.645223169	0.577818033	-0.157941265	1.26E-08	1.08E-07
TSS1500	SDR9C7	12	57329353/57328406/57328644	3	cg12524061/cg13446622/cg20596724	0.624260958	0.487108706	0.37423312	-0.250027837	1.19E-12	1.60E-10
TSS1500	SEC14L4	22	30902615/30902642	2	cg14324805/cg20436912	0.70924878	0.5795005	0.464872736	-0.244376044	3.18E-10	5.71E-09
TSS1500	SEC16B	1	177939546/177939251/177939484	3	cg02289040/cg25135333/cg27451587	0.475520343	0.364833392	0.286121564	-0.189398779	6.68E-11	1.82E-09

TSS1500	SEL1L2	20	13971811/13972022	2	cg10640284/cg18690833	0.663615808	0.592615739	0.493940532	-0.169675276	4.88E-09	4.95E-08
TSS1500	SELENBP1	1	151345771/151346048/151346122/151346400	4	cg00978415/cg07916011/cg16911672/cg17759475	0.583955216	0.482675893	0.401884319	-0.182070897	7.24E-11	1.93E-09
TSS1500	SELP	1	169600704/169599754	2	cg05343289/cg17167832	0.672707098	0.601581661	0.498938002	-0.173769095	1.78E-11	7.71E-10
TSS1500	SEMA3C	7	80549660/80549171	2	cg08799727/cg09225287	0.354210555	0.250496138	0.18889342	-0.165317135	1.52E-12	1.86E-10
TSS1500	SEMA6B	19	4558718/4559799/4558740	3	cg06746744/cg12941931/cg23957285	0.481234883	0.407950294	0.309337705	-0.171897179	4.08E-07	2.18E-06
TSS1500	SERPINB12	18	61222687/61222064	2	cg11435943/cg12412154	0.63696564	0.552976661	0.437255214	-0.199710426	5.02E-08	3.50E-07
TSS1500	SERPINB13	18	61253151/61253847	2	cg01056135/cg06538870	0.69047051	0.579388286	0.489712689	-0.200757821	2.32E-09	2.69E-08
TSS1500	SERPINB4	18	61312477/61312367	2	cg03294557/cg25485091	0.436330985	0.359068656	0.280836127	-0.155494858	3.23E-12	2.88E-10
TSS1500	SERPINI2	3	167193257	1	cg11375831	0.705032655	0.641912456	0.5387208	-0.166311855	1.16E-05	4.23E-05
TSS1500	SFMBT2	10	7454561/7453871/7454759/7454063	4	cg02353184/cg03031609/cg03237648/cg06352406	0.228107157	0.354522398	0.491777277	0.26367012	8.57E-08	5.54E-07
TSS1500	SFN	1	27189270/27188505/27189298/27188587/27189349	5	cg11348165/cg13235717/cg14333454/cg16549596/cg21950166	0.460494623	0.367894009	0.303945274	-0.156549349	1.46E-11	6.74E-10

TSS1500	SFRP2	4	154710796/154711183/154711457/154711347/154711675/154710750/154711563/154711519/154710523/154710961/154711081/154711629/154711597/154710429/154711171/154711512/154710475/154711070/154711280/154710826/154711304/154711470/154710499/154710535/154711115/154710873/154710900/154711099/154711620/154710598	30	cg00082664/cg00705808/cg01298731/cg01821854/cg04959480/cg04965141/cg05050042/cg05241277/cg05961809/cg07999845/cg09788843/cg10663078/cg10790791/cg10942078/cg13357229/cg13732865/cg14289246/cg14435644/cg20727217/cg20881942/cg21630608/cg21657059/cg22178613/cg23207990/cg23502475/cg23714408/cg23910835/cg24241928/cg24968721/cg25645268	0.38689186	0.434917837	0.539214983	0.152323124	5.37E-06	2.11E-05
TSS1500	SFT2D3	2	128458281/128457218/128458240/128458248	4	cg03339817/cg15127867/cg20154206/cg24925163	0.555468707	0.472033541	0.371775474	-0.183693232	5.81E-08	3.96E-07
TSS1500	SFTPA1	10	81370414/81369426/81370291	3	cg01134967/cg17410079/cg27452691	0.609497265	0.535546654	0.444298668	-0.165198597	4.81E-09	4.90E-08
TSS1500	SGCD	5	155752506	1	cg24795745	0.67582789	0.547530106	0.402935255	-0.272892635	5.81E-11	1.67E-09
TSS1500	SGCZ	8	15096879/15096774	2	cg09766556/cg20596629	0.49802097	0.428804461	0.315799741	-0.182221229	5.08E-11	1.56E-09
TSS1500	SGOL1	3	20228976/20227940/20227925	3	cg00235661/cg02114449/cg22604940	0.42658549	0.327688083	0.261436877	-0.165148613	1.75E-08	1.42E-07
TSS1500	SH2D2A	1	156786653/156786643/156786601	3	cg12039611/cg12499211/cg20839149	0.457687128	0.351473181	0.267618898	-0.19006823	2.11E-08	1.66E-07
TSS1500	SH2D4A	8	19170250/19170432/19170080/19170890	4	cg03138955/cg09140851/cg14936266/cg22708914	0.4777416	0.390207311	0.314751147	-0.162990453	7.02E-12	4.53E-10
TSS1500	SH3TC2	5	148443744/148443366	2	cg09548638/cg13018402	0.56922699	0.4492207	0.381051241	-0.188175749	7.03E-11	1.89E-09
TSS1500	SHE	1	154475139/154475068/154475173/154475269/154475688/154474800	6	cg02457680/cg05637536/cg18081940/cg19360104/cg24889998/cg25460143	0.434378221	0.481532957	0.597734064	0.163355843	3.04E-09	3.36E-08

TSS1500	SHISA2	13	26626092/26626663/26625766/26626134	4	cg02126117/cg07984168/cg16572375/cg20193324	0.304890892	0.349902814	0.460093257	0.155202365	2.64E-05	8.86E-05
TSS1500	SHISA7	19	55954973/55955292/55954621	3	cg01641455/cg02852989/cg10638599	0.180670474	0.250233824	0.38234927	0.201678796	2.12E-07	1.23E-06
TSS1500	SHISA9	16	12994049/12995011/12995078/12994998	4	cg02748479/cg09229683/cg21432375/cg26752526	0.225686592	0.278124102	0.430683681	0.204997089	1.02E-05	3.76E-05
TSS1500	SIGLEC1	20	3687992/3689084/3688314	3	cg02288165/cg03478070/cg15271461	0.783227098	0.700512417	0.614492255	-0.168734843	4.59E-07	2.42E-06
TSS1500	SIGLEC11	19	50464713/50464781/50464887	3	cg03283694/cg22187733/cg24311282	0.68402912	0.611325391	0.50186635	-0.18216277	1.12E-11	5.66E-10
TSS1500	SIGLEC8	19	51961937/51962338	2	cg02427831/cg27040525	0.589162065	0.44700045	0.371387741	-0.217774324	2.06E-11	8.38E-10
TSS1500	SIGLECP3	19	51670251/51670315/51669595	3	cg02018326/cg08861404/cg22658316	0.433519667	0.355606472	0.263784859	-0.169734808	6.32E-12	4.16E-10
TSS1500	SIM1	6	100911949/100912072/100913030/100912946/100911964/100912822/100912906/100912168/100912940	9	cg01258751/cg11540692/cg12233363/cg17380661/cg17942121/cg18528367/cg21684012/cg26966124/cg27252696	0.290137441	0.33962136	0.480856492	0.190719051	9.16E-06	3.40E-05
TSS1500	SIRPG	20	1639325/1639816/1639250	3	cg11061975/cg13408605/cg23295754	0.702641013	0.50884555	0.339862545	-0.362778467	1.83E-15	8.67E-12
TSS1500	SLC10A5	8	82608057/82607505/82608298	3	cg00161955/cg02573176/cg26164767	0.641460465	0.571431067	0.488673926	-0.152786539	1.80E-11	7.71E-10
TSS1500	SLC10A6	4	87770782/87770744	2	cg13119182/cg15881238	0.828922718	0.733751794	0.566891625	-0.262031093	7.69E-08	5.05E-07
TSS1500	SLC13A5	17	6617580/6617192/6617470/6617030/6617124	5	cg01974875/cg13545212/cg21872037/cg22040627/cg26782108	0.516337783	0.588659689	0.6925767	0.176238918	9.81E-08	6.25E-07
TSS1500	SLC17A2	6	25931205/25931659/25931557	3	cg01936555/cg20765441/cg24163360	0.6328762	0.465428419	0.373585605	-0.259290595	2.80E-14	2.33E-11
TSS1500	SLC18A3	10	50817104/50817213/50817804/50816963/50816917/50817306/50817810/50817651/50817096	9	cg05997350/cg06108782/cg08996579/cg12052765/cg17284779/cg18592174/cg23987444/cg26813646/cg27500798	0.298896711	0.373556264	0.526404253	0.227507542	1.30E-07	8.01E-07

TSS1500	SLC22A14	3	38346525	1	cg27520549	0.73062987	0.687163794	0.572297355	-0.158332515	8.51E-06	3.18E-05
TSS1500	SLC22A18AS	11	2926601/2925768/2925473/2925470/2926361/2926344/2925570/2925969/2925869/2926422/2925594/2925951/2925847/2926143/2925996/2925623/2926337/2925439/2925634	19	cg02025860/cg02390725/cg06048910/cg06495763/cg06669405/cg06981073/cg08472797/cg08827700/cg08999895/cg09731124/cg14101500/cg16530128/cg20716202/cg21991825/cg22272492/cg22833478/cg23912877/cg24724917/cg26137286	0.624482738	0.534072063	0.470561551	-0.153921187	1.49E-12	1.84E-10
TSS1500	SLC22A24	11	62912823	1	cg04426607	0.67878832	0.516373306	0.407864932	-0.270923388	1.22E-08	1.05E-07
TSS1500	SLC22A6	11	62753561/62753944/62753072	3	cg03539808/cg04458776/cg12428447	0.628114222	0.523771187	0.408429053	-0.219685169	3.84E-13	8.04E-11
TSS1500	SLC24A2	9	19788317/19788257	2	cg14439761/cg14442421	0.344403925	0.468337139	0.609366514	0.264962589	9.20E-09	8.26E-08
TSS1500	SLC24A5	15	48412241/48411862	2	cg09456905/cg24125468	0.80405332	0.686609572	0.592618041	-0.211435279	3.63E-07	1.97E-06
TSS1500	SLC25A18	22	18042722/18042550/18042646	3	cg00748432/cg04139184/cg04460557	0.479019123	0.407684367	0.316667421	-0.162351702	5.61E-12	3.93E-10
TSS1500	SLC27A6	5	128300967/128300696/128300708	3	cg00076307/cg20103758/cg20417527	0.242221315	0.30317555	0.492429005	0.25020769	5.63E-06	2.20E-05
TSS1500	SLC28A1	15	85427348/85426911	2	cg08271842/cg12302621	0.511796785	0.4267913	0.352837459	-0.158959326	3.69E-11	1.26E-09
TSS1500	SLC2A8	9	130158144	1	cg00114012	0.660497665	0.519128167	0.445666732	-0.214830933	4.74E-11	1.50E-09
TSS1500	SLC30A8	8	118146418	1	cg23338195	0.482035695	0.366651428	0.244319205	-0.23771649	3.36E-13	7.87E-11
TSS1500	SLC32A1	20	37352792/37352612/37352085/37351945	4	cg03556497/cg08535373/cg15490840/cg19675084	0.267058138	0.353513704	0.476395385	0.209337247	1.80E-09	2.18E-08
TSS1500	SLC35B3	6	8436296/8436218/8436263/8436466	4	cg02152351/cg09548084/cg12178445/cg15983580	0.569324571	0.486138297	0.399614758	-0.169709813	8.74E-09	7.91E-08
TSS1500	SLC35B4	7	134003124/134002145/134002209	3	cg02659138/cg02820759/cg27581047	0.71033233	0.543116639	0.410342268	-0.299990062	4.49E-11	1.44E-09
TSS1500	SLC35D2	9	99146400	1	cg13771161	0.41161746	0.310505806	0.260671868	-0.150945592	7.53E-09	6.97E-08
TSS1500	SLC35F4	14	58064926/58064661/58064775	3	cg01702210/cg20038225/cg21168134	0.676928443	0.601172564	0.487351205	-0.189577238	3.30E-07	1.81E-06

TSS1500	SLC36A2	5	150727812/150727459/150728626/150728369	4	cg10345936/cg22505977/cg23502298/cg24780796	0.419857938	0.324556026	0.260306089	-0.159551849	1.86E-09	2.25E-08
TSS1500	SLC38A3	3	50241300/50242325	2	cg00390253/cg15335139	0.568906938	0.505776369	0.418390309	-0.150516628	2.79E-07	1.57E-06
TSS1500	SLC39A4	8	145643535/145642295/145642456/145642243/145642291/145642421/145643083/145643626	8	cg03453890/cg04803798/cg05574324/cg06700234/cg11800672/cg12503742/cg12676500/cg20259557	0.534995386	0.428679607	0.339464564	-0.195530823	8.97E-12	5.08E-10
TSS1500	SLC44A5	1	76078176/76077832/76078230	3	cg12659128/cg17755518/cg27336379	0.740184425	0.66982192	0.563821165	-0.17636326	7.46E-06	2.82E-05
TSS1500	SLC4A1	17	42346894/42346923	2	cg04433322/cg16464328	0.484415493	0.383630533	0.301240068	-0.183175424	1.49E-10	3.23E-09
TSS1500	SLC5A4	22	32651693/32651562/32651776	3	cg01337931/cg09244200/cg22027471	0.622292395	0.539065925	0.46364093	-0.158651465	1.85E-05	6.43E-05
TSS1500	SLC6A1	3	11033866/11033246/11034013/11033977	4	cg00375819/cg01078332/cg07646731/cg08072251	0.39186199	0.262314478	0.188607636	-0.203254354	2.68E-14	2.33E-11
TSS1500	SLC6A11	3	10857688/10857703/10857456/10857699	4	cg00605982/cg07744841/cg09395732/cg18290624	0.467192398	0.543815768	0.647508145	0.180315748	2.98E-11	1.09E-09
TSS1500	SLC6A15	12	85306916/85307131/85307152/85306854/85307424/85306828/85307080	7	cg03064067/cg07811110/cg08143343/cg08254307/cg09892273/cg18023283/cg19686001	0.434885071	0.518090029	0.629540763	0.194655692	1.21E-08	1.04E-07
TSS1500	SLC6A18	5	1225224/1224256/1225074	3	cg19421752/cg21133868/cg26427908	0.785790838	0.737902733	0.629150873	-0.156639965	2.02E-05	6.95E-05
TSS1500	SLC6A5	11	20620287/20620181/20619598/20619733/20620089/20620002/20620680	7	cg03573068/cg04592728/cg09696301/cg11288764/cg11784785/cg24420041/cg27036111	0.292496997	0.377680645	0.456664748	0.16416775	1.65E-06	7.41E-06
TSS1500	SLC7A14	3	170304889/170304760/170305197	3	cg07475940/cg11624728/cg24977541	0.672917303	0.618695489	0.507449468	-0.165467835	2.34E-09	2.70E-08
TSS1500	SLC7A9	19	33361552/33361032/33361317/33361127	4	cg05337042/cg05467458/cg08836353/cg16861209	0.703402151	0.613326908	0.519409316	-0.183992835	4.69E-10	7.59E-09
TSS1500	SLCO1A2	12	21488180	1	cg19659215	0.492274655	0.439451306	0.325578005	-0.16669665	9.49E-06	3.51E-05

TSS1500	SLFN11	17	33701776/33701350/33701353/33701529/33701321	5	cg00080081/cg03668718/cg11309496/cg13341380/cg18108623	0.253235105	0.40114549	0.526908069	0.273672965	8.19E-09	7.49E-08
TSS1500	SLFN12	17	33760527/33759986/33760293/33760230/33759929/33760419/33759965/33760249/33759957	9	cg03237218/cg05174890/cg08330690/cg10431713/cg11346248/cg18587557/cg19566405/cg20354552/cg21697381	0.436417532	0.531965852	0.596674342	0.160256809	7.07E-06	2.69E-05
TSS1500	SLFN13	17	33777219/33776554/33776683/33776116/33776345	5	cg00364956/cg01565438/cg02380585/cg17653886/cg18067859	0.552219067	0.64444791	0.704980193	0.152761126	5.90E-10	9.04E-09
TSS1500	SLITRK1	13	84457340	1	cg08697689	0.629818235	0.690742539	0.783754355	0.15393612	5.61E-11	1.65E-09
TSS1500	SLITRK5	13	88324193/88324597/88324432/88324570/88323396/88323992/88323940/88324169/88323607/88323452	10	cg08492619/cg09083627/cg15461335/cg15778745/cg16328172/cg19391029/cg21194448/cg25081106/cg26110710/cg27151812	0.199629367	0.201339328	0.355461468	0.155832101	1.08E-05	3.96E-05
TSS1500	SMOX	20	4128004/4128750/4128752/4129121	4	cg02494904/cg02813644/cg03408497/cg21038957	0.439515799	0.348324875	0.280320116	-0.159195683	9.73E-12	5.21E-10
TSS1500	SNAP25	20	10198044/10198732/10198391/10198659/10199210	5	cg11355215/cg14851482/cg19239848/cg22537474/cg25307902	0.259553636	0.353771505	0.496165355	0.236611719	7.08E-08	4.70E-07
TSS1500	SNAP91	6	84419352/84419329/84419360	3	cg03815358/cg05384102/cg24638849	0.314998225	0.370889691	0.513772638	0.198774413	3.23E-05	0.000106121
TSS1500	SNORA42	1	155891244/155890816	2	cg01769968/cg06894549	0.83111467	0.721922933	0.643285927	-0.187828743	1.03E-08	9.06E-08
TSS1500	SNORA5B	7	45145961/45146807/45147131	3	cg02468649/cg07992143/cg26720010	0.68895493	0.599089781	0.52229922	-0.16665571	4.58E-09	4.69E-08
TSS1500	SNORA80	21	33750733/33749986	2	cg09199848/cg09516696	0.790117165	0.638312894	0.608907745	-0.18120942	3.84E-07	2.07E-06
TSS1500	SNORD109A	15	25285748/25522171/25285663	3	cg09358388/cg18499731/cg23225193	0.716071598	0.65879935	0.540442882	-0.175628716	1.90E-08	1.52E-07
TSS1500	SNORD109B	15	25285748/25522171/25285663	3	cg09358388/cg18499731/cg23225193	0.716071598	0.65879935	0.540442882	-0.175628716	1.90E-08	1.52E-07

TSS1500	SNORD113-2	14	101393226/101393397	2	cg02675047/cg04997124	0.69371903	0.56477565	0.422582695	-0.271136335	5.22E-09	5.22E-08
TSS1500	SNORD113-4	14	101402192/101402494	2	cg03629141/cg05218696	0.690925805	0.640672289	0.459901064	-0.231024741	2.74E-06	1.16E-05
TSS1500	SNORD113-9	14	101410882/101411642	2	cg02990507/cg21053224	0.793084445	0.759374817	0.630533627	-0.162550818	0.000153993	0.000434996
TSS1500	SNORD114-10	14	101432887/101432414/101432120	3	cg07472806/cg16689211/cg26435481	0.735467073	0.638246278	0.534806884	-0.200660188	1.34E-08	1.13E-07
TSS1500	SNORD114-12	14	101434601	1	cg03401464	0.404682125	0.265217628	0.178182428	-0.226499697	9.74E-14	4.59E-11
TSS1500	SNORD114-16	14	101438461/101439569/101438663/101438491	4	cg08023458/cg12725520/cg14144771/cg26277720	0.798786395	0.722975064	0.640851034	-0.157935361	4.17E-07	2.22E-06
TSS1500	SNORD114-17	14	101440409	1	cg23811289	0.48256067	0.3469528	0.233578145	-0.248982525	5.51E-15	1.31E-11
TSS1500	SNORD114-20	14	101445987/101447095	2	cg04966586/cg10000148	0.651522265	0.560284575	0.475949361	-0.175572904	6.98E-11	1.88E-09
TSS1500	SNORD114-21	14	101447389/101447985/101447095/101448093	4	cg02351406/cg09848749/cg10000148/cg14219236	0.592687767	0.444999248	0.346827865	-0.245859902	1.96E-14	2.04E-11
TSS1500	SNORD114-22	14	101448585/101447985/101448093	3	cg00554539/cg09848749/cg14219236	0.728325218	0.629576253	0.557602525	-0.170722693	1.82E-09	2.21E-08
TSS1500	SNORD114-23	14	101449839/101449311	2	cg01013868/cg07947615	0.614066498	0.492349583	0.411275643	-0.202790854	5.06E-13	9.33E-11
TSS1500	SNORD114-24	14	101449839/101450261/101450218/101450066	4	cg01013868/cg06963672/cg20326359/cg24947073	0.746300454	0.663042039	0.593891252	-0.152409201	4.62E-12	3.51E-10
TSS1500	SNORD114-9	14	101432120	1	cg26435481	0.732402195	0.610818572	0.536843668	-0.195558527	1.76E-09	2.14E-08
TSS1500	SNORD115-25	15	25459305/25459423	2	cg05055833/cg26800551	0.73794274	0.649745578	0.549649118	-0.188293622	5.91E-06	2.30E-05
TSS1500	SNORD115-30	15	25469014/25469866/25469586	3	cg12227411/cg12310189/cg13615402	0.649238397	0.564579043	0.496141679	-0.153096718	6.89E-09	6.47E-08
TSS1500	SNORD115-34	15	25476386/25476056	2	cg14154381/cg18972584	0.64555767	0.5585294	0.4241033	-0.22145437	1.29E-10	2.92E-09
TSS1500	SNORD115-40	15	25488114/25487893/25487823	3	cg00078513/cg09457527/cg26344776	0.64255571	0.560220389	0.468575614	-0.173980096	4.57E-10	7.44E-09
TSS1500	SNORD115-6	15	25425099/25425436/25425326	3	cg03959681/cg12604036/cg27455606	0.611900315	0.508426122	0.444602636	-0.167297679	1.36E-07	8.34E-07
TSS1500	SNORD116-17	15	25331391/25330536/25327489/25328405/25327919/25330341/25330370/25330242/25327713	9	cg00476675/cg01871408/cg05172655/cg05736011/cg07897695/cg08051964/cg13075322/cg16978004/cg17198397	0.684438451	0.624119408	0.533239238	-0.151199213	1.89E-07	1.12E-06

TSS1500	SNORD116-19	15	25331391/25330536/25327489/25328405/25327919/25330341/25330370/25330242/25327713	9	cg00476675/cg01871408/cg05172655/cg05736011/cg07897695/cg08051964/cg13075322/cg16978004/cg17198397	0.684438451	0.624119408	0.533239238	-0.151199213	1.89E-07	1.12E-06
TSS1500	SNORD116-24	15	25338790/25338219	2	cg09455126/cg15590133	0.62163681	0.544631489	0.459451116	-0.162185694	1.13E-06	5.29E-06
TSS1500	SNORD116-25	15	25341985/25342118	2	cg10153019/cg17720150	0.780028055	0.734443411	0.596121691	-0.183906364	1.71E-05	6.00E-05
TSS1500	SNORD116-28	15	25349082	1	cg00465883	0.832748675	0.78076155	0.646365073	-0.186383602	4.13E-05	0.000132663
TSS1500	SNORD116-29	15	25350925	1	cg09275980	0.739011805	0.668713922	0.541917427	-0.197094378	4.96E-05	0.000156263
TSS1500	SNORD19	3	52721831/52722445/52722452	3	cg00150837/cg13917433/cg22692725	0.60200432	0.4625509	0.365865543	-0.236138777	3.68E-10	6.37E-09
TSS1500	SNORD32B	6	29548535/29548691/29549180/29549352/29549724/29549147/29549759	7	cg01465527/cg06308109/cg08196667/cg10139151/cg12322235/cg13311832/cg20838323	0.638616929	0.561200806	0.459959703	-0.178657226	4.78E-09	4.88E-08
TSS1500	SNORD87	8	67835853/67835351	2	cg00640314/cg20217649	0.65718307	0.501438994	0.424463568	-0.232719502	1.98E-06	8.72E-06
TSS1500	SNRPC	6	34723390/34723499	2	cg04690793/cg15265092	0.586039375	0.495273744	0.393376809	-0.192662566	1.48E-08	1.23E-07
TSS1500	SNRPE	1	203829775/203830200/203829890/203829481	4	cg06642677/cg11405475/cg15481791/cg23035341	0.502464901	0.406815731	0.32702841	-0.175436491	3.02E-10	5.48E-09
TSS1500	SNTG1	8	50824244/50823541/50823173/50823468/50823377/50823252/50823124/50824358	8	cg06248179/cg07739205/cg07854547/cg08896053/cg08954417/cg22095914/cg25086136/cg26461695	0.249223634	0.339200155	0.493951034	0.2447274	1.71E-08	1.39E-07
TSS1500	SNTG2	2	945298/946010/945423/946127/945697	5	cg06431025/cg14256587/cg16137949/cg20225749/cg20390515	0.173346682	0.178217636	0.324336245	0.150989563	0.003327538	0.007162417
TSS1500	SNX32	11	65600846/65600739/65600888/65600773/65601167/65601202	6	cg05758094/cg06678084/cg13467162/cg21971621/cg22959820/cg25616216	0.266128677	0.351482356	0.483737663	0.217608986	1.90E-06	8.38E-06

TSS1500	SORCS3	10	106400259/106400114/106399513/106400565/106399957/106400454/106399877	7	cg02320740/cg02392179/cg07126167/cg08768569/cg09551147/cg14752336/cg23797411	0.259994593	0.362678822	0.524607345	0.264612752	1.80E-09	2.18E-08
TSS1500	SOX11	2	5831722/5831394/5832430	3	cg08432727/cg08526991/cg12312988	0.39844467	0.480870659	0.601111159	0.202666489	1.12E-09	1.50E-08
TSS1500	SOX17	8	55370283/55370192	2	cg03329976/cg09626193	0.350871955	0.395748067	0.506181368	0.155309413	0.000250366	0.000676895
TSS1500	SOX7	8	10588486/10589359/10588386/10589093	4	cg11603463/cg16799111/cg22008625/cg24690731	0.194024899	0.257929076	0.388488334	0.194463435	9.86E-05	0.000292043
TSS1500	SOX8	16	1031458/1031205/1030388/1030383/1030561/1030619/1030444/1030586/1030568/1030401/1031428	11	cg02049499/cg02925162/cg04493430/cg04809453/cg05031521/cg05520409/cg06777194/cg08965276/cg09854626/cg21696393/cg22904679	0.242730752	0.288053858	0.435825115	0.193094363	2.76E-07	1.55E-06
TSS1500	SPAG11B	8	7311130	1	cg14600420	0.61582796	0.442693267	0.288175909	-0.327652051	9.33E-12	5.15E-10
TSS1500	SPARCL1	4	88451223/88451496/88451346	3	cg05350879/cg08003102/cg14334389	0.466098305	0.380375717	0.254148514	-0.211949791	9.02E-09	8.12E-08
TSS1500	SPDYE4	17	8662302/8663295/8662770	3	cg08036512/cg25304136/cg26665785	0.69900518	0.584019178	0.451488177	-0.247517003	2.04E-09	2.42E-08
TSS1500	SPEF1	20	3762520/3762422/3762653	3	cg02347859/cg08708079/cg17185202	0.72432255	0.619286833	0.555667832	-0.168654718	2.63E-07	1.49E-06
TSS1500	SPEG	2	220299116/220298547/220299484	3	cg14074251/cg22126505/cg25925023	0.66372998	0.599546931	0.498857559	-0.164872421	6.45E-09	6.17E-08
TSS1500	SPINK4	9	33239641	1	cg00079056	0.847814665	0.771030078	0.683332064	-0.164482601	2.18E-05	7.45E-05
TSS1500	SPOCK2	10	73849626/73849124/73849106/73849167	4	cg01861509/cg02909446/cg12752647/cg13665981	0.496127355	0.368386306	0.294616839	-0.201510516	7.82E-11	2.03E-09
TSS1500	SPP1	4	88895691/88896208	2	cg00583003/cg02549628	0.60551281	0.4814535	0.373312357	-0.232200453	1.27E-10	2.90E-09
TSS1500	SPRR1A	1	152955080/152956186/152955543	3	cg00922841/cg04505023/cg06101324	0.64891722	0.590983239	0.492823048	-0.156094172	8.48E-09	7.71E-08
TSS1500	SPRR2C	1	153114690/153114766/153115333	3	cg07804289/cg10119001/cg23606417	0.60033274	0.528740198	0.444398412	-0.155934328	4.07E-08	2.91E-07
TSS1500	SPRR2G	1	153124825	1	cg20141817	0.770604855	0.654291583	0.556063064	-0.214541791	4.31E-07	2.29E-06

TSS1500	SPRYD5	11	55649706/55650046	2	cg07200877/cg18268863	0.74857808	0.692483661	0.5673093	-0.18126878	4.03E-09	4.23E-08
TSS1500	SPTA1	1	158656718	1	cg22730004	0.37384369	0.224023283	0.148832282	-0.225011408	1.86E-11	7.80E-10
TSS1500	SPTBN5	15	42187649/42186830/42187036/42186945/42187179	5	cg00165394/cg13586457/cg18188010/cg19691260/cg24203709	0.460790181	0.369827779	0.30171024	-0.159079941	1.30E-10	2.94E-09
TSS1500	SRPK1	6	35890197/35889177/35889893	3	cg08603125/cg13866752/cg18198550	0.429342355	0.343766878	0.276669678	-0.152672677	1.63E-10	3.45E-09
TSS1500	SSC5D	19	55998490/55998732/55998931	3	cg03645984/cg17379828/cg19820609	0.509887665	0.4367637	0.359526945	-0.15036072	1.45E-09	1.84E-08
TSS1500	SSPO	7	149472049/149471884/149472134	3	cg01776278/cg16902863/cg19351398	0.593245355	0.517769731	0.425362711	-0.167882644	3.93E-10	6.66E-09
TSS1500	ST6GALNAC1	17	74641275/74641294/74641167	3	cg00898480/cg03096803/cg13049471	0.627073715	0.4775208	0.373668823	-0.253404892	2.18E-11	8.71E-10
TSS1500	ST6GALNAC5	1	77331940	1	cg20417424	0.641121125	0.541041667	0.391830709	-0.249290416	2.94E-09	3.28E-08
TSS1500	ST8SIA4	5	100239919/100240048/100239319/100240059/100240223/100239529	6	cg00405699/cg02318139/cg19088984/cg22965432/cg26872028/cg27367469	0.268651367	0.330234897	0.46248451	0.193833144	4.04E-07	2.16E-06
TSS1500	ST8SIA5	18	44338099/44337910/44337664/44337922/44338147/44337853	6	cg01059449/cg15049968/cg15966229/cg23129478/cg23302884/cg26155939	0.285734185	0.334694973	0.535361973	0.249627787	1.02E-06	4.88E-06
TSS1500	STAB2	12	103979670/103980767/103980801/103980017	4	cg00550529/cg11010909/cg23131355/cg23227824	0.725736221	0.655772115	0.561423039	-0.164313183	6.64E-07	3.34E-06
TSS1500	STK31	7	23749740/23749667/23749722/23749533/23749607/23749639	6	cg05000488/cg05407490/cg13514718/cg13802883/cg14898779/cg25612145	0.808057841	0.715949206	0.578179044	-0.229878797	7.31E-09	6.80E-08
TSS1500	STK32B	4	5052464/5052379/5052393/5053085/5052795/5053152/5052618/5052526	8	cg00225765/cg08961294/cg10182321/cg11682071/cg11768886/cg19764048/cg22551741/cg25688113	0.153532076	0.21550239	0.323113689	0.169581612	1.28E-05	4.60E-05
TSS1500	STK38	6	36515676/36515856	2	cg02964385/cg23670203	0.598610195	0.443515925	0.373382268	-0.225227927	1.99E-14	2.04E-11
TSS1500	SUB1	5	32585396/32585221/32584912	3	cg04225902/cg12600337/cg18721397	0.754064323	0.669797307	0.528998767	-0.225065557	6.40E-06	2.47E-05

TSS1500	SULT2A1	19	48390114	1	cg26574395	0.741835755	0.669565939	0.531003336	-0.210832419	0.000122619	0.000354608
TSS1500	SVOP	12	109460299/109459622	2	cg00461311/cg25374714	0.61544229	0.488755606	0.389417661	-0.226024629	1.69E-10	3.55E-09
TSS1500	SYN2	3	12045449/12045459/12045461	3	cg02700894/cg15873301/cg22839075	0.304309728	0.327207939	0.49542752	0.191117792	0.002016991	0.004529041
TSS1500	SYNPO2	4	119808935/119808885	2	cg10320997/cg26325949	0.59746355	0.527540356	0.415245266	-0.182218284	2.36E-08	1.83E-07
TSS1500	SYT14	1	210111057/210110998/210111028/210111011/210111162	5	cg02795029/cg07581146/cg15389528/cg25371503/cg26158959	0.254276755	0.356179969	0.427411854	0.173135099	6.98E-06	2.66E-05
TSS1500	SYT6	1	114697582/114696698/114697523/114696822/114697352/114697113/114696946/114697095/114697032/114696798/114697707	11	cg00764109/cg03514545/cg06620092/cg08883066/cg12097117/cg12804010/cg15976388/cg19874857/cg19901801/cg22646710/cg26426632	0.241304647	0.277428165	0.427951805	0.186647158	2.43E-05	8.22E-05
TSS1500	SYT9	11	7272804/7272653/7272867/7272876	4	cg01806928/cg08913010/cg14243481/cg26945996	0.281621628	0.351672528	0.545256369	0.263634741	5.83E-06	2.27E-05
TSS1500	T-SP1	8	10382165/10381792	2	cg01072821/cg21997510	0.53560619	0.424380069	0.341125661	-0.194480529	1.87E-10	3.84E-09
TSS1500	TAAR9	6	132858438/132858546/132858597	3	cg07882863/cg14047911/cg15698945	0.775874275	0.636632196	0.499383806	-0.276490469	2.60E-10	4.91E-09
TSS1500	TACR3	4	104641319/104641548/104641250	3	cg05389335/cg07824172/cg17160751	0.360435123	0.430035367	0.540108238	0.179673115	8.45E-07	4.13E-06
TSS1500	TAF4B	18	23805713/23806189	2	cg02833108/cg23398091	0.81811406	0.69689905	0.573304609	-0.244809451	6.28E-09	6.02E-08
TSS1500	TAS2R1	5	9631216/9631117	2	cg09532664/cg23248452	0.6423812	0.597948228	0.4551479	-0.1872333	6.84E-06	2.62E-05
TSS1500	TAS2R16	7	122636106/122636635	2	cg19660744/cg23719342	0.649059773	0.548548883	0.426384368	-0.222675404	1.73E-10	3.61E-09
TSS1500	TAS2R39	7	142879929/142879966	2	cg06489609/cg20896431	0.725847275	0.631062525	0.535081236	-0.190766039	2.15E-06	9.38E-06
TSS1500	TAT	16	71611519/71612168	2	cg10251217/cg27189824	0.595823588	0.506140222	0.41498112	-0.180842467	1.76E-12	2.01E-10
TSS1500	TBX15	1	119532850/119532773/119532655/119532542/119532736/119532925	6	cg00466334/cg02362103/cg03942051/cg08942939/cg22820316/cg23371746	0.396286828	0.521989196	0.651061339	0.254774511	2.25E-11	8.82E-10

TSS1500	TBX18	6	85474585/85474595/85474424/85474209/85474313/85474188	6	cg01114937/cg05674784/cg09259037/cg09749813/cg09817427/cg16210497	0.2202741	0.297825676	0.419324686	0.199050586	4.22E-06	1.70E-05
TSS1500	TBX20	7	35294640/35295198/35294949/35294430/35295120/35294658	6	cg02524946/cg09349530/cg11117878/cg12467749/cg18011916/cg25377206	0.377328403	0.461968742	0.610150885	0.232822483	5.40E-09	5.35E-08
TSS1500	TBX5	12	114842700/114846968/114847641/114847043/114847508/114847164/114844265/114847578/114843188/114845180/114846503/114846849/114845223/114841980/114847438/114842031	16	cg00756451/cg03843000/cg05555207/cg05769349/cg05929882/cg06911121/cg08318726/cg12670347/cg14214262/cg14264795/cg16559598/cg16605327/cg16805360/cg17462200/cg19290410/cg20099830	0.295115396	0.352978794	0.48168945	0.186574054	8.42E-07	4.12E-06
TSS1500	TCERG1L	10	133110349/133110646/133110731/133111331/133110244	5	cg03109827/cg03943081/cg18138206/cg20239333/cg23632875	0.327061502	0.421557845	0.566537581	0.239476079	2.80E-09	3.15E-08
TSS1500	TCF15	20	592164/591151/591400/591221	4	cg00983437/cg02760031/cg06143901/cg11076994	0.353209088	0.413009124	0.520730805	0.167521716	6.66E-07	3.35E-06
TSS1500	TCHHL1	1	152062567	1	cg01925883	0.73411146	0.6784471	0.570087314	-0.164024146	5.61E-05	0.000174362
TSS1500	TCL6	14	96116261/96117286/96116699	3	cg03050975/cg16209310/cg18114811	0.587117548	0.498578483	0.37726267	-0.209854877	2.95E-09	3.29E-08
TSS1500	TCN1	11	59634276	1	cg03928812	0.50406189	0.338640556	0.238165301	-0.265896589	2.50E-09	2.86E-08
TSS1500	TCP10L	21	33958437/33958060/33958055/33958411	4	cg01194119/cg09247736/cg12549554/cg16417831	0.789257317	0.673304213	0.5452121	-0.244045217	1.62E-10	3.43E-09
TSS1500	TCP11	6	35109548/35109435/35109485/35109722/35109398	5	cg09027493/cg12090052/cg12835524/cg16083558/cg24623244	0.611134185	0.509901217	0.460077355	-0.15105683	3.82E-08	2.75E-07
TSS1500	TCTEX1D1	1	67217826/67217769/67217886/67217673/67217853	5	cg08857729/cg09769134/cg14207728/cg17819635/cg24110050	0.28786256	0.335832583	0.470465905	0.182603345	8.98E-05	0.000268088

TSS1500	TEKT1	17	6735316/6735539/6735272	3	cg03549162/cg12685753/cg15615160	0.201185964	0.266171536	0.444536309	0.243350345	2.35E-06	1.01E-05
TSS1500	TEX12	11	112037427/112037134/112037102	3	cg03304763/cg07267984/cg13591377	0.734878423	0.627130404	0.568257998	-0.166620425	1.42E-10	3.13E-09
TSS1500	TEX15	8	30707156/30707701	2	cg00831770/cg20939319	0.458801065	0.340908156	0.243172445	-0.21562862	3.61E-13	7.96E-11
TSS1500	TF	3	133464047/133464651/133464463/133464713	4	cg02621528/cg10772086/cg16267236/cg18286850	0.470224748	0.387035917	0.298962354	-0.171262394	1.97E-10	3.98E-09
TSS1500	TFF1	21	43787747/43786997	2	cg01442565/cg01886855	0.567226565	0.465155125	0.374617761	-0.192608804	1.20E-10	2.79E-09
TSS1500	TFF3	21	43737065/43736736/43736926/43737014	4	cg08198280/cg19452276/cg20488657/cg24230696	0.54827054	0.433578464	0.341220225	-0.207050315	2.82E-12	2.70E-10
TSS1500	TFPI2	7	93520346/93520323/93521153/93520445/93520275/93520527/93520269/93521093/93520566/93520452/93520288/93521196	12	cg03333330/cg07380959/cg07603382/cg09558850/cg10539069/cg13328713/cg15649801/cg18302726/cg19103770/cg19854521/cg24084681/cg27496965	0.425195168	0.499148146	0.59194832	0.166753152	3.40E-08	2.50E-07
TSS1500	TFR2	7	100239764/100240094/100240341	3	cg01919768/cg16628205/cg26369382	0.481687452	0.41517958	0.331301329	-0.150386123	5.04E-11	1.55E-09
TSS1500	TH	11	2193742/2193284/2193293	3	cg04204669/cg05141787/cg21644468	0.528481238	0.4062004	0.30862163	-0.219859608	1.85E-13	6.62E-11
TSS1500	THRSP	11	77773924/77774337	2	cg09721595/cg16505204	0.62045304	0.551424858	0.454123527	-0.166329513	5.76E-11	1.66E-09
TSS1500	THSD7B	2	137747719/137747982	2	cg08084967/cg25714147	0.763253023	0.662500381	0.552185261	-0.211067761	3.04E-09	3.35E-08
TSS1500	TIGIT	3	114011433/114012316	2	cg19440299/cg22577252	0.677761113	0.536701422	0.44276013	-0.235000983	9.61E-12	5.17E-10
TSS1500	TINAG	6	54172997/54172183/54172378	3	cg00012148/cg12397274/cg27090087	0.433067683	0.322588361	0.256430597	-0.176637086	3.08E-12	2.81E-10
TSS1500	TINAGL1	1	32041732/32041052/32041605/32041507	4	cg03314569/cg09070415/cg10530055/cg16103203	0.45305019	0.35656175	0.285581742	-0.167468448	7.45E-11	1.96E-09
TSS1500	TIPIN	15	66649558/66649861	2	cg11164506/cg12541836	0.686503283	0.551740306	0.431130809	-0.255372473	8.59E-13	1.33E-10
TSS1500	TK1	17	76183602/76183641/76184081/76183551	4	cg00739217/cg02441982/cg21940220/cg22061523	0.502505785	0.413918914	0.32701407	-0.175491715	9.40E-10	1.31E-08

TSS1500	TLR6	4	38859706/38859339/38859770/38859728	4	cg02221520/cg04840108/cg14578677/cg14665413	0.376540585	0.306625811	0.225306259	-0.151234326	1.67E-07	1.00E-06
TSS1500	TLX3	5	170735186/170735264/170736021/170734856/170735754/170735347	6	cg05787556/cg18336674/cg18468394/cg22160448/cg24881420/cg26517171	0.386740033	0.468786828	0.621915544	0.235175511	5.73E-09	5.60E-08
TSS1500	TM2D3	15	102193344/102193228/102192853/102193416	4	cg01537571/cg13202221/cg17115258/cg25863289	0.453025078	0.383101185	0.273167243	-0.179857834	2.16E-11	8.64E-10
TSS1500	TM4SF19	3	196065569/196065506/196065485/196065688	4	cg05445326/cg13314965/cg15044270/cg23250593	0.719088245	0.569534231	0.433443041	-0.285645204	9.77E-12	5.21E-10
TSS1500	TM6SF1	15	83775850/83775931/83776023	3	cg04120171/cg17018527/cg22719913	0.224306397	0.294544478	0.410487654	0.186181257	4.57E-06	1.83E-05
TSS1500	TM9SF4	20	30696827/30697017/30696538	3	cg20672708/cg25953453/cg26450387	0.658819115	0.5663431	0.488864793	-0.169954322	1.51E-11	6.86E-10
TSS1500	TMCO5A	15	38226316/38226454	2	cg11854043/cg23254045	0.547123465	0.464455933	0.353271732	-0.193851733	8.47E-07	4.14E-06
TSS1500	TMEM130	7	98468038/98468167/98468047	3	cg02132470/cg06124528/cg19974428	0.128754732	0.207886171	0.355085309	0.226330578	2.86E-05	9.54E-05
TSS1500	TMEM132B	12	125810767/125810688	2	cg01012836/cg10520543	0.479971125	0.360555911	0.256327255	-0.22364387	5.65E-12	3.94E-10
TSS1500	TMEM132C	12	128750971/128751460/128751582/128751042/128751048	5	cg00698908/cg03530754/cg06164660/cg10406124/cg18628094	0.398911408	0.448370586	0.572123577	0.17321217	5.71E-05	0.000177281
TSS1500	TMEM132E	17	32907132/32906622/32906456/32907388/32907002/32906454/32906394/32906991	8	cg00196787/cg04790084/cg04906989/cg06459443/cg06557358/cg08967802/cg09628838/cg23791505	0.15414405	0.183092805	0.328770194	0.174626143	5.82E-05	0.000180153
TSS1500	TMEM139	7	142981776/142981689/142981779	3	cg15652212/cg21852589/cg22531018	0.641732465	0.502494211	0.370342645	-0.27138982	1.41E-11	6.59E-10
TSS1500	TMEM14E	3	152059421	1	cg15012282	0.622756345	0.477845667	0.380257859	-0.242498486	2.16E-12	2.21E-10
TSS1500	TMEM156	4	39034637/39034284	2	cg02131853/cg25246082	0.401111045	0.3130807	0.242156216	-0.158954829	1.67E-09	2.06E-08
TSS1500	TMEM163	2	135476907/135476909/135477056/135476880/135477425/135476893	6	cg08343042/cg10025300/cg15297650/cg17319374/cg22242207/cg25476312	0.296316872	0.333074003	0.481755746	0.185438874	4.76E-05	0.000150823

TSS1500	TMEM177	2	120435949/120436534/120436430/120436039	4	cg12108912/cg20302082/cg23456409/cg24441810	0.414599078	0.31309656	0.251530321	-0.163068758	1.28E-10	2.91E-09
TSS1500	TMEM196	7	19813299/19813297/19813525/19813149/19812850/19813079/19812760/19813244/19813493/19813001	10	cg04111071/cg06896909/cg10182869/cg10184740/cg11124364/cg15377585/cg19037304/cg19803052/cg21760040/cg25184481	0.332884224	0.398183188	0.493455612	0.160571388	9.97E-07	4.77E-06
TSS1500	TMEM200A	6	130757762	1	cg26931050	0.74042799	0.583572439	0.493609582	-0.246818408	2.27E-08	1.77E-07
TSS1500	TMPRSS11F	4	68995995	1	cg02936740	0.771209585	0.68159355	0.568941105	-0.20226848	2.13E-05	7.29E-05
TSS1500	TMPRSS6	22	37500588/37500694/37500722	3	cg00556408/cg25786785/cg26679348	0.71978451	0.5996113	0.50827153	-0.21151298	1.03E-08	9.08E-08
TSS1500	TMX3	18	66383305/66383213/66382559	3	cg05188150/cg12487792/cg12522039	0.63030602	0.532984164	0.43167377	-0.19863225	4.17E-09	4.35E-08
TSS1500	TNFRSF10A	8	23082961/23083578/23082951/23083551/23083353	5	cg22843797/cg23303108/cg23882019/cg24278165/cg26530341	0.419538741	0.31454578	0.249660829	-0.169877911	4.95E-10	7.92E-09
TSS1500	TNFSF13	17	7461272/7461260/7460690/7460485	4	cg00816039/cg05872923/cg13829089/cg17267493	0.457469138	0.374500224	0.296581927	-0.160887211	3.09E-10	5.60E-09
TSS1500	TNN	1	175035502/175036687/175036403	3	cg02138218/cg05643339/cg11741115	0.6357775	0.532945261	0.470377027	-0.165400473	3.57E-07	1.95E-06
TSS1500	TNNI1	1	201391627/201391747/201392102	3	cg07236127/cg13172153/cg22449085	0.58347014	0.504390994	0.424812243	-0.158657897	3.40E-07	1.86E-06
TSS1500	TNNT1	19	55661330/55661872	2	cg00844197/cg19393762	0.613030585	0.532714206	0.455586245	-0.15744434	1.70E-06	7.60E-06
TSS1500	TNNT2	1	201347917/201348231/201347483/201347479/201347550	5	cg07596645/cg09274810/cg18692273/cg23594208/cg24249973	0.587884016	0.474955813	0.390601241	-0.197282775	2.29E-13	7.11E-11
TSS1500	TOMM20L	14	58862246/58862417/58862146	3	cg04749433/cg04752257/cg18549946	0.478697182	0.38250328	0.300235805	-0.178461377	1.88E-11	7.87E-10
TSS1500	TP53RK	20	45319455/45318890/45319350	3	cg07291836/cg07987148/cg15305633	0.584501578	0.510682097	0.408316168	-0.176185409	6.25E-10	9.48E-09

TSS1500	TP63	3	189506187/189348936/189349003/189506825/189347820	5	cg02122981/cg05129081/cg12188416/cg13518031/cg16764781	0.46543456	0.385155256	0.292724629	-0.172709932	5.79E-07	2.96E-06
TSS1500	TPRG1	3	188888497/188888486/188888584	3	cg14768087/cg19682367/cg23259542	0.471147038	0.377139217	0.305462548	-0.16568449	1.75E-10	3.65E-09
TSS1500	TPSD1	16	1305420/1305718/1305579	3	cg01375871/cg03466598/cg09956782	0.660017477	0.590258054	0.468394686	-0.19162279	2.59E-09	2.95E-08
TSS1500	TPSG1	16	1276278/1275895/1275475/1275785	4	cg02194908/cg03497872/cg05026881/cg13997068	0.55756113	0.465591611	0.372483227	-0.185077903	2.04E-10	4.10E-09
TSS1500	TRABD	22	50623687/50623165/50623692/50624030	4	cg05297854/cg06372718/cg11213574/cg18205517	0.386087803	0.495962518	0.569740425	0.183652623	6.01E-08	4.08E-07
TSS1500	TRAM1L1	4	118007225/118007206/118007342	3	cg03345976/cg22155281/cg25548984	0.712247112	0.623745276	0.479285747	-0.232961365	5.51E-11	1.62E-09
TSS1500	TRDN	6	123959184	1	cg14462830	0.72351412	0.616815044	0.432497877	-0.291016243	3.59E-08	2.61E-07
TSS1500	TREH	11	118550644/118551771/118551168	3	cg08498647/cg17695682/cg25740070	0.581995718	0.505011267	0.412755589	-0.169240129	3.17E-09	3.46E-08
TSS1500	TREM2	6	41131863/41132055/41131213	3	cg00718409/cg02539671/cg25748868	0.730799315	0.611025004	0.511847806	-0.218951509	5.00E-11	1.55E-09
TSS1500	TREML1	6	41123396/41123086/41123110	3	cg04710115/cg10792307/cg15153887	0.475444743	0.403416358	0.314912505	-0.160532238	6.12E-11	1.71E-09
TSS1500	TREML4	6	41195195/41195092/41194812	3	cg01552173/cg17295842/cg25535435	0.760755897	0.697347587	0.592525214	-0.168230683	6.10E-06	2.37E-05
TSS1500	TRHDE	12	72665510/72666264/72665880/72666281/72665376/72665282/72665771	7	cg01817029/cg02511156/cg04775889/cg06622999/cg17771605/cg18440199/cg23142394	0.235902135	0.300701726	0.453103565	0.21720143	5.41E-06	2.12E-05
TSS1500	TRIM31	6	30081408	1	cg15238224	0.44635788	0.339683289	0.264747577	-0.181610303	2.28E-11	8.89E-10
TSS1500	TRIM42	3	140395771/140395898	2	cg06339924/cg18814069	0.521648123	0.413629797	0.31878273	-0.202865393	1.65E-08	1.35E-07
TSS1500	TRIM61	4	165900224/165899396/165900285	3	cg00231192/cg20233582/cg21550098	0.86365995	0.821945225	0.704392261	-0.159267689	1.68E-06	7.51E-06
TSS1500	TRIM63	1	26394593/26394435	2	cg06161375/cg13998293	0.552648855	0.426710944	0.334190902	-0.218457953	1.56E-10	3.35E-09
TSS1500	TRIM71	3	32858541/32859099/32858280	3	cg08145617/cg14657525/cg18310412	0.108400209	0.163988728	0.307136231	0.198736022	5.53E-05	0.000172326
TSS1500	TRIM75	4	165979202	1	cg23735682	0.714337205	0.609817106	0.509495205	-0.204842	1.01E-06	4.83E-06

TSS1500	TRIM77	11	89442829	1	cg27391117	0.74484231	0.645401172	0.572006695	-0.172835615	6.66E-06	2.56E-05
TSS1500	TRIP6	7	100463985/100463759/100464145/100463508/100464553/100463583	6	cg01008256/cg04742719/cg12967723/cg19279257/cg21406967/cg26305174	0.515007872	0.432765747	0.355556169	-0.159451703	8.28E-12	4.90E-10
TSS1500	TRPM8	2	234824777/234824933	2	cg12488871/cg15746445	0.681576873	0.625594922	0.512412098	-0.169164775	9.60E-08	6.13E-07
TSS1500	TRUB1	10	116697604/116697070	2	cg03934681/cg08898349	0.430034612	0.311587452	0.245818672	-0.18421594	3.05E-09	3.36E-08
TSS1500	TSGA13	7	130372419/130372807/130372167	3	cg02332073/cg10421595/cg11058932	0.643008985	0.572044283	0.490231005	-0.15277798	1.34E-06	6.16E-06
TSS1500	TSPAN12	7	120498729/120499546	2	cg22032706/cg23627038	0.618166025	0.460064511	0.394421307	-0.223744718	1.55E-09	1.95E-08
TSS1500	TSPAN8	12	71552457/71552336/71552188/71552569	4	cg04950931/cg12965512/cg15684563/cg19871235	0.421708438	0.335905124	0.267752997	-0.153955441	1.62E-11	7.24E-10
TSS1500	TTLL1	22	43485660/43486524/43486472	3	cg18982625/cg22637865/cg25347941	0.582596383	0.507237454	0.418939579	-0.163656805	5.40E-08	3.72E-07
TSS1500	TTN	2	179672691/179672637	2	cg11111214/cg15133917	0.713177923	0.568056803	0.455002148	-0.258175775	9.18E-10	1.28E-08
TSS1500	TUBGCP2	10	135122875/135123764/135123006/135123239/135124069/135123337/135123067/135123421	8	cg02441467/cg07219844/cg07908874/cg12275410/cg12642705/cg16284674/cg19495428/cg24424217	0.546987877	0.45691581	0.385182962	-0.161804914	1.08E-10	2.58E-09
TSS1500	TWIST1	7	19157650/19157938/19158692/19157839/19157813/19158134/19158664/19158747/19157739/19158721/19158123/19158647/19158041/19157634/19158566/19158210/19158001/19158666/19157710/19158349/19158030/19158378	22	cg01202666/cg02400740/cg04904385/cg04917226/cg06243400/cg09674215/cg10126205/cg10624122/cg11147786/cg12307484/cg12514789/cg14391419/cg14515453/cg14782672/cg16168668/cg19064523/cg20121142/cg21424940/cg22498251/cg26279021/cg27013696/cg27334919	0.334220304	0.405283853	0.529831818	0.195611514	1.32E-08	1.12E-07
TSS1500	UBQLN3	11	5531524	1	cg15553770	0.770926495	0.646663706	0.521146309	-0.249780186	5.68E-08	3.89E-07
TSS1500	UCMA	10	13277824/13276892	2	cg03816954/cg27243297	0.63790748	0.559738011	0.450752618	-0.187154862	1.90E-06	8.40E-06

TSS1500	UCP1	4	141490377/141490428/141491214/141490351/141490354	5	cg07100771/cg09181644/cg14078662/cg16512615/cg21325154	0.741431015	0.670080028	0.541320591	-0.200110424	6.89E-07	3.45E-06
TSS1500	UGT1A6	2	234601254/234599026/234599988/234600149/234600147/234600423	6	cg04449108/cg07512295/cg09950076/cg12385643/cg14121772/cg23338993	0.5552985	0.439026182	0.393610685	-0.161687815	8.21E-10	1.17E-08
TSS1500	UGT1A8	2	234525618/234526077	2	cg10596609/cg18313132	0.458042645	0.352433189	0.266454164	-0.191588481	4.20E-11	1.37E-09
TSS1500	UGT2B7	4	69960869	1	cg04558553	0.486010185	0.411888578	0.324395491	-0.161614694	7.05E-09	6.58E-08
TSS1500	UGT8	4	115519006/115518992	2	cg11950778/cg22609233	0.57674276	0.463072719	0.389045302	-0.187697458	2.41E-10	4.64E-09
TSS1500	UMOD	16	20364284	1	cg17812431	0.728370775	0.603621294	0.477368095	-0.25100268	2.53E-08	1.95E-07
TSS1500	UNC45B	17	33474376/33473881	2	cg07264238/cg10174170	0.61279604	0.510610611	0.410609023	-0.202187017	1.35E-07	8.31E-07
TSS1500	UNC5C	4	96470626/96471105/96470993/96470584/96471143/96470887	6	cg01194057/cg12584684/cg12802900/cg13265789/cg15701178/cg21782409	0.219830513	0.338336784	0.487739705	0.267909192	5.65E-07	2.90E-06
TSS1500	UNC5CL	6	41007485/41007692/41007450/41007522	4	cg15849648/cg17826424/cg19175193/cg22346765	0.729970745	0.566412708	0.438502102	-0.291468643	8.63E-14	4.43E-11
TSS1500	UNK	17	73780373/73780396/73780246/73780035	4	cg08301181/cg13182713/cg25355213/cg26332052	0.430155931	0.32378419	0.236664827	-0.193491104	2.94E-09	3.27E-08
TSS1500	USP44	12	95942964/95943114/95942907/95945767/95942847	5	cg07783282/cg09883286/cg13879483/cg16192751/cg17368254	0.196333114	0.242103689	0.431633	0.235299886	3.59E-05	0.000116746
TSS1500	UTF1	10	135043492/135043508/135043506/135042885	4	cg02767771/cg03755123/cg08708684/cg12465710	0.37683011	0.490416753	0.612698456	0.235868346	3.50E-08	2.56E-07
TSS1500	VASH1	14	77227574/77227860/77227570/77227545/77227645	5	cg02735446/cg07559696/cg12825804/cg14124894/cg23948080	0.176556459	0.181655931	0.337989367	0.161432909	0.00103921	0.002472472
TSS1500	VAT1L	16	77822083/77822135	2	cg06165506/cg06989330	0.090584311	0.131680684	0.253444695	0.162860384	0.001060526	0.00251719
TSS1500	VAV3	1	108508207/108508548/108232421/108508067/108508185/108508113/108507766	7	cg05937496/cg06543087/cg09896211/cg12619536/cg17099397/cg19918758/cg25848557	0.248664977	0.383928911	0.484115169	0.235450192	3.55E-07	1.93E-06

TSS1500	VCAM1	1	101184332/101185070/101184304	3	cg04295372/cg04743650/cg25763716	0.49824432	0.442099406	0.333153561	-0.165090759	1.28E-08	1.09E-07
TSS1500	VEGFC	4	177714373/177714350/177714456/177715040	4	cg01590753/cg06679347/cg17177660/cg24194285	0.384570977	0.409092681	0.559936502	0.175365525	1.32E-05	4.73E-05
TSS1500	VHLL	1	156270260/156270253/156270918/156270281	4	cg05795077/cg08656082/cg12664795/cg16558208	0.716687372	0.64059177	0.522653977	-0.194033394	1.21E-08	1.04E-07
TSS1500	VILL	3	38033770/38033934/38034235	3	cg03203287/cg05673892/cg06641593	0.63210314	0.536947737	0.442891745	-0.189211395	5.03E-09	5.06E-08
TSS1500	VNN1	6	133036573/133036545/133036651	3	cg01052291/cg22498396/cg22618219	0.655706088	0.517355744	0.412301364	-0.243404724	5.88E-13	1.04E-10
TSS1500	VPREB1	22	22597869/22597965/22598937	3	cg01891260/cg04776489/cg14844130	0.353633734	0.258033336	0.178746913	-0.174886821	2.54E-13	7.42E-11
TSS1500	VSTM2B	19	30016147/30016478/30016511/30016136	4	cg01464835/cg02012703/cg05395302/cg18802754	0.384355415	0.491734456	0.61733325	0.232977835	2.36E-07	1.36E-06
TSS1500	VSX1	20	25064127/25063052/25063817	3	cg17650274/cg19592637/cg23097006	0.477176173	0.564642289	0.667121195	0.189945022	6.62E-09	6.28E-08
TSS1500	WBP11P1	18	30091235/30090866	2	cg13340705/cg16566595	0.71459601	0.636055753	0.552891389	-0.161704621	1.81E-07	1.07E-06
TSS1500	WBSCR17	7	70596377/70597091/70597058/70597065/70597458/70596308	6	cg01159623/cg01366419/cg02300154/cg03044249/cg03893271/cg10239388	0.394991448	0.47322018	0.639233247	0.244241799	9.68E-11	2.39E-09
TSS1500	WDR12	2	203777863/203778143	2	cg15057621/cg17745291	0.66161852	0.567587578	0.453666141	-0.207952379	5.32E-09	5.29E-08
TSS1500	WDR36	5	110427072/110427346/110427112	3	cg00601387/cg11585022/cg18807895	0.580139953	0.463570969	0.320444241	-0.259695712	3.58E-12	3.11E-10
TSS1500	WDR59	16	75020429/75019382/75019367	3	cg00519320/cg05828191/cg16379316	0.601968697	0.524578172	0.450119017	-0.15184968	8.12E-07	4.00E-06

TSS1500	WDR8	1	3567870/3567303/3568004/3566992/3567646/3567719/3568154/3566963/3567852/3568124/3567412/3567119/3567214/3567930/3567408/3567109/3567163/3566950/3567986/3567732/3567738/3567550	22	cg01434649/cg04021697/cg04391111/cg04865841/cg07382920/cg10038618/cg10143426/cg11256802/cg12475507/cg14781922/cg16823083/cg18021902/cg19135761/cg20611911/cg21000072/cg21012455/cg22614891/cg22822803/cg24073122/cg24678611/cg25731359/cg26128092	0.364920319	0.467848881	0.620655629	0.25573531	6.34E-12	4.16E-10
TSS1500	WDR86	7	151107807/151108340/151107637/151107924/151107400	5	cg00878038/cg03693105/cg16482474/cg19831077/cg21532325	0.367021215	0.434092614	0.527694646	0.160673431	2.25E-05	7.65E-05
TSS1500	WIPF3	7	29873076/29873786/29872894	3	cg18581663/cg24384632/cg25037274	0.702222167	0.646635256	0.526807121	-0.175415045	4.42E-08	3.13E-07
TSS1500	WNT3	17	44897512/44896923/44896748/44896424/44896877/44897024/44897431	7	cg00817598/cg02017282/cg03186851/cg12379948/cg13998093/cg15237742/cg24114556	0.212191774	0.261458875	0.368208683	0.156016909	1.15E-05	4.19E-05
TSS1500	WSB1	17	25620677/25619743/25619946/25620596/25620638	5	cg02789515/cg05922422/cg07564598/cg15326755/cg23536675	0.789047844	0.654636015	0.565146146	-0.223901698	4.03E-10	6.77E-09
TSS1500	WT1	11	32457878/32457727/32457386	3	cg12600018/cg22975913/cg26232818	0.182944365	0.213337707	0.375527668	0.192583303	0.000668507	0.001655908
TSS1500	XDH	2	31638511/31637849	2	cg09239591/cg09842053	0.55308488	0.435740339	0.3470439	-0.20604098	1.77E-11	7.71E-10
TSS1500	XKR4	8	56013556/56013800/56014406/56014433	4	cg08417620/cg09388605/cg09524907/cg20948997	0.293191333	0.29172405	0.443875378	0.150684045	0.000153748	0.000434563
TSS1500	YIPF7	4	44654206	1	cg03815116	0.65073397	0.494116667	0.3368489	-0.31388507	1.87E-12	2.06E-10
TSS1500	YOD1	1	207225647/207225128/207224740/207225424/207224982	5	cg01380361/cg02710090/cg05398095/cg06119874/cg24040043	0.631585616	0.486708478	0.375190038	-0.256395579	6.27E-12	4.16E-10

TSS1500	ZBTB11	3	101396658/101396855/101396897	3	cg02395779/cg05405742/cg05890243	0.660308475	0.557454997	0.454581139	-0.205727336	2.20E-08	1.73E-07
TSS1500	ZBTB32	19	36203512/36202390/36203089	3	cg03068068/cg09231418/cg12497692	0.546518783	0.456254374	0.37350443	-0.173014353	1.31E-08	1.11E-07
TSS1500	ZC3H8	2	113013154/113012921	2	cg00535466/cg09759808	0.589286333	0.5261378	0.437825757	-0.151460576	4.87E-09	4.94E-08
TSS1500	ZFP28	19	57049530/57049695/57049857/57049777	4	cg03305181/cg12973930/cg23850212/cg25963041	0.290740397	0.323551596	0.441215127	0.150474731	0.004281556	0.009014451
TSS1500	ZG16	16	29789103	1	cg09229061	0.70386272	0.569923422	0.460841123	-0.243021597	2.05E-10	4.12E-09
TSS1500	ZIC1	3	147125955/147126753/147126961/147125926/147125758/147125962/147126638/147126244/147125765/147126121/147125924/147126444/147126206/147126403/147126703/147125714/147125712/147125942/147126425/147125782/147126763/147126119	22	cg00475509/cg02519751/cg05700079/cg07291439/cg08070771/cg08855449/cg09241332/cg10107050/cg11142389/cg12419052/cg12965599/cg15002294/cg16181396/cg16636671/cg17280346/cg17546247/cg23189410/cg23449696/cg23468878/cg24686610/cg26014538/cg26400885	0.514521073	0.604640659	0.721842117	0.207321045	1.96E-10	3.97E-09

TSS1500	ZIC4	3	147111280/147110595/147123457/147124827/147124363/147111135/147111660/147124543/147122664/147124523/147125758/147125765/147110499/147125114/147125287/147110667/147123429/147111308/147125714/147123475/147124417/147123123/147125712/147123199/147125782/147122315/147124687/147111120	28	cg00154357/cg01581018/cg02387803/cg02820514/cg03355998/cg03881775/cg03900143/cg05548555/cg06166523/cg06369327/cg08070771/cg11142389/cg12388007/cg13015925/cg13278496/cg13340636/cg16790847/cg17003736/cg17546247/cg19516404/cg21127068/cg21639713/cg23189410/cg24620761/cg24686610/cg26224785/cg26791399/cg27606499	0.358553099	0.425560582	0.530670317	0.172117218	7.51E-09	6.96E-08
TSS1500	ZIK1	19	58095424/58095155/58094517/58095011	4	cg02653557/cg06707978/cg17798563/cg21911021	0.512088898	0.607626404	0.699078256	0.186989358	7.72E-12	4.85E-10
TSS1500	ZKSCAN1	7	99612634/99612773/99612715	3	cg02891218/cg07125725/cg21540035	0.58125791	0.441799652	0.384122103	-0.197135807	1.10E-13	4.63E-11
TSS1500	ZNF132	19	58951814/58952361/58951885/58952108/58952249	5	cg00868383/cg03043130/cg07878486/cg11618529/cg16929496	0.258725671	0.382328816	0.467619038	0.208893367	4.04E-06	1.64E-05
TSS1500	ZNF154	19	58220818/58220837	2	cg01268824/cg27324426	0.498936993	0.640335011	0.713483861	0.214546869	1.19E-09	1.58E-08
TSS1500	ZNF169	9	97020667	1	cg14220634	0.557733235	0.457490872	0.355628891	-0.202104344	9.54E-12	5.16E-10
TSS1500	ZNF177	19	9472210/9473058/9473240	3	cg09492640/cg14323854/cg19275200	0.5429892	0.422993233	0.317120995	-0.225868205	7.82E-13	1.26E-10
TSS1500	ZNF229	19	44953982/44953377	2	cg19772066/cg26535330	0.733923575	0.659407664	0.580021082	-0.153902493	7.26E-08	4.80E-07
TSS1500	ZNF256	19	58459358/58459283	2	cg02959669/cg12048031	0.091643475	0.136415784	0.291683919	0.200040444	6.03E-05	0.000186288
TSS1500	ZNF283	19	44331009/44331239/44330665/44330910	4	cg04895616/cg06574716/cg18514705/cg25309775	0.566995222	0.627872415	0.718635538	0.151640316	2.03E-08	1.61E-07
TSS1500	ZNF300	5	150284600/150284796	2	cg18237551/cg21228005	0.367075915	0.360543939	0.578431705	0.21135579	5.11E-05	0.000160396

TSS1500	ZNF304	19	57862442/57862242/57862410	3	cg10157975/cg22376081/cg23250910	0.065461025	0.200111538	0.321294501	0.255833476	1.10E-06	5.18E-06
TSS1500	ZNF347	19	53662594/53662620/53662499/53662680/53662958/53662723	6	cg03434128/cg04254066/cg15167155/cg15584946/cg19722781/cg24708545	0.272944047	0.360317284	0.490563301	0.217619254	5.91E-05	0.000182758
TSS1500	ZNF354C	5	178487123/178487382/178487384/178487310	4	cg08522107/cg10864596/cg12623648/cg19761115	0.162476908	0.239685872	0.401820943	0.239344035	0.000108386	0.000317461
TSS1500	ZNF382	19	37095681/37096010/37095466/37095757	4	cg09430118/cg19406736/cg24871714/cg27658967	0.271240398	0.404384565	0.540972873	0.269732475	6.94E-09	6.50E-08
TSS1500	ZNF454	5	178367423/178367621/178367827	3	cg02031359/cg17741986/cg24843380	0.470541208	0.532250313	0.64055532	0.170014111	6.72E-09	6.35E-08
TSS1500	ZNF469	16	88492755/88492868/88493613	3	cg03892041/cg04675221/cg27245950	0.73823703	0.678512944	0.578940005	-0.159297025	2.67E-07	1.51E-06
TSS1500	ZNF471	19	57018614/57019005/57018933/57018848	4	cg00711090/cg14042851/cg14277392/cg14289985	0.272664298	0.304327608	0.448504634	0.175840336	2.63E-06	1.12E-05
TSS1500	ZNF479	7	57208277/57207952	2	cg10652558/cg20306025	0.646167473	0.597221094	0.468992086	-0.177175386	3.92E-08	2.82E-07
TSS1500	ZNF484	9	95640659	1	cg14646548	0.676359665	0.519359111	0.425553664	-0.250806001	4.63E-12	3.51E-10
TSS1500	ZNF512B	20	62601662/62601556/62601568/62601743	4	cg03065311/cg16746938/cg23931672/cg26760212	0.221317378	0.292906869	0.392666678	0.1713493	0.000246521	0.000667355
TSS1500	ZNF543	19	57831632/57831544/57831549/57831662	4	cg00846300/cg14383655/cg14603375/cg19533530	0.096920063	0.268037434	0.374851989	0.277931926	0.000275525	0.000738703
TSS1500	ZNF544	19	58739805/58739803/58739778/58739734	4	cg11279918/cg21020710/cg21994576/cg26263766	0.091791085	0.169904413	0.272457184	0.180666099	0.000418687	0.001080986
TSS1500	ZNF568	19	37406349/37406932/37406949	3	cg09560297/cg15746891/cg21292152	0.280337563	0.371768881	0.455913321	0.175575758	5.49E-05	0.000171157
TSS1500	ZNF582	19	56905152/56905383/56905094	3	cg07135042/cg07778983/cg25267765	0.499889563	0.593394981	0.693164416	0.193274853	5.38E-08	3.70E-07
TSS1500	ZNF586	19	58280728/58279724/58280767/58280801	4	cg08140458/cg09859795/cg13382661/cg27046034	0.574240878	0.663384878	0.749990068	0.175749191	1.29E-10	2.92E-09
TSS1500	ZNF619	3	40518138/40518091	2	cg12176605/cg21942438	0.648849048	0.537052625	0.4616731	-0.187175948	5.69E-09	5.57E-08

TSS1500	ZNF620	3	40547217/40547232	2	cg07448319/cg13461273	0.445257293	0.3670533	0.290535611	-0.154721681	2.98E-09	3.31E-08
TSS1500	ZNF625	19	12267796/12267776/12267734	3	cg02842629/cg17456704/cg24115221	0.094828471	0.218627344	0.383485125	0.288656654	4.46E-05	0.000142206
TSS1500	ZNF648	1	182031601	1	cg13498800	0.67062413	0.582208533	0.478677968	-0.191946162	3.85E-08	2.77E-07
TSS1500	ZNF667	19	56989102/56989543/56989812/56989740	4	cg03289872/cg08063125/cg21287936/cg24411929	0.414287348	0.566804689	0.724532957	0.310245609	2.10E-11	8.50E-10
TSS1500	ZNF671	19	58239222/58239424	2	cg21305471/cg22001557	0.434555714	0.516558613	0.659281339	0.224725624	1.08E-08	9.47E-08
TSS1500	ZNF677	19	53758521/53758609	2	cg04154138/cg16708981	0.35791966	0.495035972	0.645107336	0.287187676	2.36E-11	9.09E-10
TSS1500	ZNF688	16	30584858/30584674	2	cg03945387/cg07300610	0.701700785	0.572555378	0.499054925	-0.20264586	1.35E-10	3.01E-09
TSS1500	ZNF705A	12	8324628/8324153	2	cg12193649/cg20325280	0.531736225	0.398408633	0.289799809	-0.241936416	1.50E-12	1.85E-10
TSS1500	ZNF75A	16	3354207/3355079	2	cg02825709/cg07949597	0.603154285	0.733300611	0.818337745	0.21518346	2.97E-09	3.30E-08
TSS1500	ZNF790	19	37329498/37329859/37329760/37329500/37329666/37329517	6	cg02343318/cg04803153/cg07808761/cg16646909/cg17656260/cg25772438	0.390937583	0.471806543	0.597862544	0.206924961	1.03E-09	1.41E-08
TSS1500	ZNF793	19	37997294/37997471	2	cg11623861/cg17332326	0.095571162	0.228164667	0.381753861	0.286182699	1.72E-05	6.01E-05
TSS1500	ZNF80	3	113957903/113957908	2	cg03109316/cg11380748	0.83477924	0.715704675	0.602813348	-0.231965892	1.62E-06	7.26E-06
TSS1500	ZNF804A	2	185462269/185462493/185462408/185462397/185462655/185462847/185462338	7	cg01715143/cg01968493/cg11811513/cg13121699/cg15577595/cg21739754/cg24166893	0.191639624	0.294429471	0.415138829	0.223499205	1.17E-07	7.32E-07
TSS1500	ZNF804B	7	88388244/88387861	2	cg09591286/cg12745764	0.168159466	0.234363019	0.337595722	0.169436257	3.04E-05	0.000100612
TSS1500	ZNF805	19	57751816/57751691/57751591	3	cg01607934/cg02446106/cg03252986	0.27841615	0.410302136	0.541371161	0.262955012	6.60E-10	9.85E-09
TSS1500	ZNF813	19	53970618/53970585/53970754	3	cg05503527/cg09435109/cg13948710	0.146824803	0.20720027	0.363508075	0.216683272	1.86E-06	8.21E-06
TSS1500	ZNF829	19	37407462/37407486/37407889/37407413	4	cg03443751/cg20154403/cg21117780/cg26612849	0.138315112	0.255437701	0.384277621	0.245962509	4.27E-05	0.000136861
TSS1500	ZNF880	19	52872916	1	cg22080027	0.339262505	0.44441555	0.544427895	0.20516539	6.09E-05	0.000187773
TSS1500	ZNHIT6	1	86174739/86175136/86174929/86174607/86174642	5	cg17807903/cg18878378/cg22676901/cg23216685/cg27462398	0.636343953	0.522373814	0.417289657	-0.219054296	3.09E-10	5.60E-09

TSS1500	ZP4	1	238055429/238054569	2	cg03673470/cg26810908	0.663329823	0.502237561	0.392643039	-0.270686784	2.06E-11	8.38E-10
TSS1500	ZPBP	7	50133212/50133131/50133100	3	cg00303745/cg09921682/cg10349880	0.84136324	0.669956002	0.48186017	-0.35950307	3.20E-12	2.87E-10
TSS1500	ZSCAN1	19	58545122/58545001/58545149/58545160/58545182	5	cg11312896/cg14886059/cg21331821/cg24368848/cg25537993	0.544930566	0.643858004	0.739010143	0.194079577	2.06E-09	2.45E-08
TSS1500	ZSCAN12	6	28367883/28367749/28367898	3	cg07660236/cg20275132/cg21488066	0.41985598	0.502801883	0.612002164	0.192146184	1.23E-05	4.45E-05
TSS1500	ZSCAN23	6	28412636/28412139/28412459	3	cg00249576/cg01837113/cg23847843	0.810580558	0.720399025	0.60841772	-0.202162837	2.47E-10	4.71E-09
TSS200	DEC1	9	117903983	1	cg16682686	0.761866575	0.663234317	0.544463618	-0.217402957	9.09E-08	1.01E-06
TSS200	MARCH1	4	165304473/165304496/165304531/165304488/165304540/165304443	6	cg00032805/cg10127275/cg10943359/cg21169914/cg21300373/cg22299454	0.345778885	0.422284654	0.547985671	0.202206786	7.86E-08	8.90E-07
TSS200	MARCH11	5	16180048/16180055/16180072/16180062/16180068/16180076/16180033	7	cg00339556/cg01791874/cg16150752/cg17030173/cg17712694/cg21901718/cg25092681	0.288388658	0.381014661	0.566613481	0.278224823	2.14E-06	1.68E-05
TSS200	A1BG	19	58864876/58864922/58864929	3	cg02957155/cg04269689/cg17498272	0.738497243	0.639695161	0.528866241	-0.209631002	2.32E-10	7.10E-09
TSS200	ABCA8	17	66951572	1	cg27464144	0.51720667	0.416295917	0.309227664	-0.207979006	1.56E-12	2.73E-10
TSS200	ABCC11	16	48266305/48269105	2	cg07383076/cg25274975	0.5685022	0.461615661	0.343803182	-0.224699018	4.87E-12	5.26E-10
TSS200	ABCC2	10	101542449	1	cg09448875	0.574886295	0.452145	0.352785186	-0.222101109	2.64E-12	4.00E-10
TSS200	ACAN	15	89346629	1	cg16968596	0.207001321	0.401839487	0.631561205	0.424559884	6.38E-07	5.67E-06
TSS200	ACER1	19	6333761/6333701	2	cg17667294/cg19440035	0.508393625	0.408996622	0.312122845	-0.19627078	2.59E-13	1.20E-10
TSS200	ACSS2	20	33462656/33462683	2	cg09801828/cg22989379	0.618966915	0.502109067	0.406813632	-0.212153283	2.94E-10	8.62E-09
TSS200	ACSS3	12	81471678/81471649/81471754/81471757	4	cg11659501/cg21754400/cg23614791/cg25358039	0.286447766	0.497189308	0.572053364	0.285605598	2.17E-08	2.94E-07
TSS200	ACTA1	1	229569894/229569902/229569892/229569877/229569965/229569969	6	cg02767613/cg04030615/cg05347845/cg14852384/cg25792990/cg26163852	0.245709466	0.291858642	0.437069386	0.19135992	1.05E-06	8.94E-06
TSS200	ACTBL2	5	56778651	1	cg20772593	0.770452585	0.662244533	0.518878727	-0.251573858	3.09E-10	8.96E-09
TSS200	ACTN2	1	236849653	1	cg14932016	0.27037604	0.281198133	0.474309027	0.203932987	2.54E-05	0.000151296

TSS200	ADAM28	8	24151514/24151472	2	cg17969683/cg18757155	0.346509395	0.216299969	0.182859208	-0.163650188	6.98E-07	6.15E-06
TSS200	ADAM3A	8	39380592	1	cg07927909	0.771113865	0.664860067	0.610387159	-0.160726706	1.55E-05	9.79E-05
TSS200	ADAM5P	8	39172120/39172022/39172111/39172020/39172097/39172099	6	cg07387286/cg10327428/cg11199639/cg11583762/cg14742937/cg19659741	0.538381784	0.635769374	0.713173556	0.174791772	5.81E-08	6.86E-07
TSS200	ADAM6	14	106438465/106438519	2	cg03899708/cg12797492	0.635713118	0.577081303	0.476884327	-0.15882879	3.94E-12	4.83E-10
TSS200	ADAMTS16	5	5140406	1	cg01717727	0.375045035	0.4556967	0.623165405	0.24812037	2.18E-10	6.83E-09
TSS200	ADAMTS18	16	77469167	1	cg02186542	0.273228825	0.282129683	0.439599427	0.166370602	5.54E-05	0.00030285
TSS200	ADAMTS2	5	178772372/178772385/178772390	3	cg13909534/cg18331515/cg26429655	0.182990746	0.2626889	0.337992382	0.155001635	2.05E-05	0.000125361
TSS200	ADAMTS5	21	28339442/28339487/28339477	3	cg08190291/cg13601496/cg19523085	0.220681917	0.294013407	0.471714923	0.251033006	2.01E-06	1.59E-05
TSS200	ADARB2	10	1779835/1779904/1779819/1779730/1779760	5	cg02899206/cg03410436/cg06730723/cg14590817/cg24184687	0.120993329	0.238864613	0.388903645	0.267910317	2.21E-06	1.73E-05
TSS200	ADCY4	14	24804022/24803903/24803917/24803873/24803925	5	cg12265829/cg13631572/cg16215203/cg23179456/cg25556905	0.163532662	0.21288422	0.371171979	0.207639316	0.004127831	0.014884922
TSS200	ADCY5	3	123167522/123167507	2	cg04138502/cg15993383	0.263099946	0.310493049	0.50221849	0.239118544	1.60E-06	1.30E-05
TSS200	ADCY8	8	132052870/132052934/132052942/132052843/132052887	5	cg07557260/cg16696270/cg22060073/cg22762091/cg26332560	0.33809967	0.427895521	0.561584072	0.223484402	7.77E-11	3.26E-09
TSS200	ADCYAP1	18	905156/905180/905252/905127/904899/904851/905101/904920/904885/905177/904878	11	cg00128702/cg11773720/cg14200170/cg15194943/cg15458338/cg16457786/cg17059658/cg18319029/cg21438101/cg22388954/cg24287438	0.276022909	0.405755226	0.544920581	0.268897672	2.45E-07	2.41E-06
TSS200	ADD2	2	70995440/70995522/70995351/70995459/70995444/70995349/70995527	7	cg03384579/cg05659187/cg08857144/cg15170605/cg23427269/cg24347663/cg25631352	0.121104374	0.227999381	0.42689553	0.305791156	1.37E-05	8.75E-05
TSS200	ADH6	4	100140474	1	cg10099209	0.58879384	0.465720711	0.338628314	-0.250165526	1.80E-11	1.22E-09

TSS200	ADHFE1	8	67344553/67344640/67344556/67344588/67344642/67344665	6	cg01588438/cg08090772/cg09383816/cg18065361/cg19283840/cg20295442	0.237875042	0.386282476	0.615113493	0.377238451	6.18E-10	1.54E-08
TSS200	ADORA2A	22	24823389/24823519/24823455/24823514/24823509	5	cg01373166/cg02237342/cg08025954/cg20660269/cg23763137	0.587043485	0.485027076	0.390485036	-0.196558449	1.45E-10	5.04E-09
TSS200	ADRA1A	8	26722965/26722933	2	cg20303399/cg23008606	0.388836703	0.4551341	0.605570534	0.216733832	3.76E-07	3.52E-06
TSS200	ADRA1B	5	159343549	1	cg07011172	0.110053362	0.153461401	0.276518966	0.166465604	0.00139416	0.005606863
TSS200	AEBP1	7	44143774	1	cg25289803	0.126515985	0.170135441	0.327045437	0.200529453	0.000517164	0.002278613
TSS200	AGPAT3	21	45345152	1	cg01331461	0.49130884	0.400000961	0.326541441	-0.164767399	3.77E-11	1.96E-09
TSS200	AGR2	7	16844852	1	cg24426405	0.405596495	0.303695883	0.218098841	-0.187497654	1.08E-12	2.27E-10
TSS200	AGR3	7	16921699	1	cg11039314	0.582580955	0.449586267	0.334640868	-0.247940087	1.10E-11	9.04E-10
TSS200	AGXT2	5	35048098	1	cg09414557	0.57072187	0.470930911	0.391149277	-0.179572593	2.38E-12	3.81E-10
TSS200	AHSP	16	31539169	1	cg05772125	0.338102345	0.220724994	0.172116405	-0.16598594	1.59E-10	5.39E-09
TSS200	AK5	1	77748190/77748136/77748221/77748129	4	cg02099814/cg17014953/cg17445007/cg26466094	0.207252802	0.290472878	0.405999654	0.198746852	3.91E-05	0.000222571
TSS200	AKR1B1	7	134144055/134143971/134144062/134143988/134143919/134143991/134143906/134144036	8	cg02215070/cg06864853/cg09957386/cg10795359/cg13801416/cg14629509/cg16132520/cg21079345	0.120978536	0.255759676	0.346781128	0.225802593	0.000542382	0.002372487
TSS200	ALB	4	74269908	1	cg02951062	0.58224563	0.543654539	0.396507564	-0.185738066	5.53E-09	9.23E-08
TSS200	ALDH3A1	17	19648972/19648925/19648855/19648853/19649051/19649004/19648970/19648846/19651881/19651940	10	cg01471036/cg05447343/cg06390079/cg11475454/cg12787571/cg15849060/cg18957070/cg23098051/cg25145360/cg27329371	0.500738424	0.407594416	0.33568294	-0.165055484	4.20E-10	1.14E-08
TSS200	ALK	2	30144623/30144547/30144579/30144566	4	cg12740757/cg13552710/cg14163665/cg25999722	0.104995013	0.186948581	0.333230032	0.228235019	0.000326756	0.001505349
TSS200	ALOX12B	17	7991103/7991169	2	cg06835483/cg22299191	0.48868159	0.387138611	0.328515698	-0.160165892	9.23E-13	2.12E-10
TSS200	ALPI	2	233320796/233320760	2	cg04519870/cg25752514	0.46623903	0.384597222	0.309468395	-0.156770635	4.58E-11	2.23E-09
TSS200	ALPP	2	233243204	1	cg09760618	0.779325595	0.664133383	0.580322668	-0.199002927	9.47E-09	1.45E-07

TSS200	ALX4	11	44331905	1	cg23089549	0.18546659	0.238488311	0.405366405	0.219899815	0.000200742	0.0009731
TSS200	AMBN	4	71457838/71457880	2	cg13523386/cg14322040	0.676795818	0.527071361	0.402634502	-0.274161315	1.90E-11	1.27E-09
TSS200	AMICA1	11	118084192	1	cg12789173	0.584538275	0.4417041	0.365294977	-0.219243298	1.26E-11	9.91E-10
TSS200	AMN	14	103388797/103388859/103388958	3	cg01759155/cg07378067/cg19659004	0.541512168	0.459527272	0.390072893	-0.151439274	1.55E-10	5.32E-09
TSS200	AMPD1	1	115238213	1	cg05499890	0.432405895	0.362587672	0.279111518	-0.153294377	4.57E-10	1.21E-08
TSS200	ANGPTL1	1	178840298	1	cg05630800	0.52292468	0.413086789	0.3627692	-0.16015548	4.67E-09	8.09E-08
TSS200	ANGPTL2	9	129885080	1	cg08076018	0.53873826	0.613055878	0.695600336	0.156862076	9.18E-11	3.64E-09
TSS200	ANKMY1	2	241497599/241497583/241497412/241497417/241497554/241497415	6	cg13030331/cg13198503/cg13604246/cg15766075/cg20002504/cg23964682	0.204200586	0.28514408	0.435984256	0.231783669	7.27E-05	0.000386794
TSS200	ANKRD20B	2	95522902/95522896/95522876	3	cg08721908/cg23363202/cg24524352	0.401647363	0.509299233	0.619958921	0.218311558	1.93E-06	1.53E-05
TSS200	ANKRD22	10	90611855/90611911/90611782	3	cg01561719/cg02538681/cg03249630	0.495251923	0.368030807	0.29051432	-0.204737604	2.57E-11	1.54E-09
TSS200	ANKRD24	19	4183268	1	cg19489188	0.568420715	0.472839067	0.380032332	-0.188388383	2.52E-10	7.58E-09
TSS200	ANKRD34B	5	79866431/79866319/79866368/79866379	4	cg03478969/cg14478163/cg21800232/cg25316339	0.213590115	0.2542383	0.3824274	0.168837285	0.000504962	0.002231085
TSS200	ANKRD34C	15	79575048/79574993/79574964	3	cg09103619/cg17337118/cg22101924	0.15143175	0.225983466	0.357870463	0.206438713	7.01E-08	8.09E-07
TSS200	ANKS4B	16	21244898/21244915	2	cg08030625/cg09752011	0.529291903	0.396084606	0.303389507	-0.225902396	4.51E-11	2.21E-09
TSS200	ANO2	12	6055430	1	cg00282706	0.428246835	0.295469	0.178942335	-0.2493045	3.37E-11	1.83E-09
TSS200	ANO5	11	22214554/22214565/22214683	3	cg09968630/cg13205384/cg20634738	0.110190664	0.156672659	0.299666489	0.189475825	0.006729151	0.022958023
TSS200	APBB1IP	10	26727173/26727261/26727069/26727246/26727195	5	cg06157318/cg06535993/cg14022711/cg26241806/cg27303981	0.237127988	0.399557535	0.505817762	0.268689774	4.45E-08	5.44E-07
TSS200	APBB2	4	41216800/40859280/40859259/40859249/40859293/41216812/40859251/40859344	8	cg01954188/cg02379560/cg04917391/cg05894734/cg06485940/cg09616640/cg16364495/cg26244738	0.280931146	0.416313494	0.497835854	0.216904708	3.54E-09	6.43E-08

TSS200	APCDD1L	20	57090015/57090090/57090104/57089961/57090013/57090122/57089953	7	cg07402669/cg10354244/cg13552201/cg13778709/cg20600210/cg23487201/cg24115032	0.42089102	0.487043289	0.629301859	0.208410839	1.74E-09	3.51E-08
TSS200	APOBEC1	12	7818635/7818654/7818585	3	cg11304383/cg17187764/cg20597842	0.513610838	0.401134222	0.313431189	-0.200179649	6.31E-11	2.82E-09
TSS200	APOBEC4	1	183622470	1	cg19264170	0.434361075	0.331664567	0.245767905	-0.18859317	7.94E-12	7.10E-10
TSS200	ARHGAP20	11	110583649/110583549/110583543/110583590/110583599	5	cg03532274/cg04974587/cg13643376/cg14652031/cg26135012	0.122905577	0.209620451	0.32270661	0.199801033	4.08E-05	0.00023138
TSS200	ARHGEF10L	1	17907024	1	cg09854011	0.509748005	0.385387567	0.317621118	-0.192126887	1.88E-11	1.27E-09
TSS200	ARMC3	10	23216847/23216843/23216841	3	cg06560760/cg08839858/cg19523082	0.156176652	0.256517689	0.475421363	0.319244711	3.92E-07	3.65E-06
TSS200	ARMS2	10	124214151/124214120	2	cg13265583/cg24296920	0.70374238	0.600907469	0.474264136	-0.229478244	7.13E-10	1.72E-08
TSS200	ARRDC5	19	4902899/4902950	2	cg04946561/cg25539255	0.666320735	0.566748817	0.475369577	-0.190951158	5.52E-08	6.55E-07
TSS200	ART1	11	3666271/3666278	2	cg03245218/cg11173076	0.530438475	0.416615628	0.328015677	-0.202422798	8.31E-10	1.95E-08
TSS200	ASB10	7	150884979/150885084/150885027/150884641/150885103/150884486	6	cg04671739/cg05181846/cg10980293/cg13594957/cg13645954/cg25786651	0.589422239	0.493514747	0.390472201	-0.198950038	4.53E-12	5.18E-10
TSS200	ASB4	7	95115232/95115246/95115194/95115163	4	cg19100779/cg20040691/cg21787063/cg25692621	0.514547569	0.418078465	0.326832825	-0.187714744	3.67E-10	1.02E-08
TSS200	ASGR1	17	7083064/7083015	2	cg05331340/cg14018648	0.606701045	0.49076275	0.369823423	-0.236877622	7.37E-12	6.96E-10
TSS200	ATCAY	19	3880535/3880606/3880544	3	cg12443227/cg17818344/cg22796572	0.203428111	0.25685613	0.354777461	0.15134935	6.30E-06	4.36E-05
TSS200	ATG9B	7	150721650	1	cg12229082	0.4947485	0.406135444	0.319899555	-0.174848945	1.30E-09	2.75E-08
TSS200	ATP10A	15	26108391/26108399/26108401/26108410/26108412	5	cg03419058/cg16389285/cg20124450/cg22113930/cg26230285	0.184105866	0.276954253	0.400864824	0.216758958	1.43E-07	1.49E-06
TSS200	ATP4B	13	114312577/114312686/114312617/114312504/114312624	5	cg01064131/cg01233487/cg01957900/cg09556939/cg12795643	0.726451575	0.649913061	0.569038273	-0.157413302	2.24E-05	0.000135346
TSS200	ATP6V1G3	1	198510253	1	cg13100753	0.665884585	0.526101967	0.395414614	-0.270469971	6.64E-12	6.35E-10

TSS200	AZU1	19	827715/827776/827739/827821	4	cg02147126/cg10752406/cg14663914/cg15610437	0.416693988	0.330244007	0.26463662	-0.152057367	1.71E-11	1.20E-09
TSS200	B3GALNT1	3	160823318/160823255/160823231/160823315/160822728/160822711/160822720	7	cg00160619/cg12716838/cg13072057/cg13279566/cg16496687/cg25469234/cg25635352	0.183049295	0.279005064	0.358239667	0.175190372	0.001648859	0.00653124
TSS200	BAALC	8	104152750/104152754	2	cg10163483/cg14284952	0.24627411	0.358346559	0.546722218	0.300448108	1.02E-06	8.68E-06
TSS200	BAIAP2L2	22	38506737/38506781/38506771/38506712	4	cg08196512/cg09247692/cg11744966/cg20207973	0.553262019	0.446624538	0.352571103	-0.200690915	3.88E-10	1.07E-08
TSS200	BARHL2	1	91182989/91182856	2	cg00010742/cg17241310	0.319597535	0.354711339	0.507436764	0.187839229	4.28E-06	3.09E-05
TSS200	BASE	20	31781277/31781232/31781393/31781333	4	cg01923514/cg11274778/cg20539474/cg24866792	0.556454206	0.480108329	0.384046014	-0.172408193	1.13E-10	4.23E-09
TSS200	BBOX1	11	27062416	1	cg20910746	0.64548082	0.501748578	0.399057405	-0.246423415	9.78E-10	2.21E-08
TSS200	BCAS1	20	52687394/52687365	2	cg08927738/cg23682609	0.47305303	0.364033217	0.285960409	-0.187092621	8.95E-12	7.77E-10
TSS200	BCL2L14	12	12224246/12223841	2	cg01175610/cg18223430	0.33042476	0.211356972	0.15726861	-0.17315615	7.85E-09	1.24E-07
TSS200	BCL2L15	1	114430280	1	cg13991834	0.387349145	0.248221828	0.196603304	-0.190745841	1.96E-10	6.34E-09
TSS200	BEND4	4	42154927/42154977/42154934/42154983/42154912/42154949	6	cg03167389/cg07033093/cg18144906/cg19009280/cg23951961/cg27450499	0.182139365	0.251943134	0.385098053	0.202958689	1.98E-07	1.98E-06
TSS200	BEST2	19	12863295	1	cg16554020	0.76392972	0.63914735	0.497493332	-0.266436388	1.96E-09	3.86E-08
TSS200	BEST3	12	70083213/70083096/70093350/70083193/70083105/70083090	6	cg03196364/cg05493841/cg07618900/cg07911961/cg14834675/cg22610980	0.342816921	0.261395022	0.174915573	-0.167901349	4.41E-09	7.74E-08
TSS200	BET3L	6	116866784	1	cg12262781	0.524387955	0.435040828	0.317179432	-0.207208523	5.97E-08	7.01E-07
TSS200	BHLHE23	20	61638488/61638420/61638518/61638433/61638501/61638574	6	cg01699217/cg06550340/cg14060496/cg14373923/cg23806621/cg26492446	0.280085534	0.394567118	0.514069551	0.233984017	1.29E-09	2.74E-08
TSS200	BOLL	2	198650208/198650985/198651076/198650123/198650987/198650112	6	cg03433510/cg05783139/cg07495363/cg10547527/cg13356896/cg21300403	0.603959952	0.659385432	0.773824135	0.169864183	5.09E-09	8.64E-08
TSS200	BPIL1	20	31595346/31595360	2	cg10968815/cg13696012	0.74723877	0.610260339	0.45784017	-0.2893986	1.50E-12	2.65E-10

TSS200	BPIL3	20	31619420	1	cg25345102	0.68629173	0.526548167	0.469432586	-0.216859144	7.17E-08	8.25E-07
TSS200	BSX	11	122852454/122852523/122852429/122852412/122852417/122852461	6	cg02078690/cg02159381/cg02179652/cg04092231/cg11141380/cg27314569	0.353419475	0.420348966	0.586804642	0.233385167	1.22E-09	2.64E-08
TSS200	BTF3L1	13	77502558	1	cg18990874	0.636846275	0.487598311	0.387678309	-0.249167966	4.93E-11	2.38E-09
TSS200	BTNL2	6	32374926/32374956	2	cg09748960/cg10254167	0.509486675	0.392747239	0.30567807	-0.203808605	2.53E-11	1.54E-09
TSS200	BTNL8	5	180325954/180325969/180336487	3	cg05624226/cg10906849/cg17826428	0.515427543	0.402136906	0.316388893	-0.199038649	7.18E-11	3.12E-09
TSS200	BZRAP1	17	56406294	1	cg12117658	0.3768359	0.250601428	0.146685415	-0.230150485	1.15E-10	4.24E-09
TSS200	C10orf72	10	50323706/50323708	2	cg06365057/cg24139737	0.300202745	0.372589422	0.477602068	0.177399323	6.27E-10	1.56E-08
TSS200	C10orf81	10	115511135/115511153	2	cg10627429/cg11204562	0.534802678	0.430468611	0.345354982	-0.189447696	5.76E-11	2.67E-09
TSS200	C10orf88	10	124714041/124714047/124714098/124714072/124713989	5	cg02463329/cg05596319/cg18565342/cg25222014/cg26956371	0.134768884	0.197947641	0.307683149	0.172914265	8.20E-05	0.00043299
TSS200	C10orf90	10	128210076/128210036	2	cg09621603/cg10005270	0.60503916	0.431178194	0.292599823	-0.312439337	7.21E-15	1.49E-11
TSS200	C11orf52	11	111789493/111789452	2	cg08775230/cg23921031	0.478575773	0.382350964	0.293517739	-0.185058034	5.24E-10	1.36E-08
TSS200	C11orf87	11	109292803/109292809/109292789	3	cg11473876/cg16359550/cg27471124	0.343952475	0.433410228	0.584383979	0.240431504	1.75E-08	2.41E-07
TSS200	C11orf88	11	111385357/111385496/111385338/111385450/111385461	5	cg03629499/cg05216730/cg08533783/cg10606490/cg25782847	0.145538933	0.176178693	0.316088397	0.170549464	2.69E-05	0.000159144
TSS200	C11orf94	11	45928848/45928937/45928988/45928854/45928841/45928898	6	cg01972688/cg02164088/cg03135577/cg05223459/cg12019650/cg25518365	0.619191618	0.488567313	0.413108785	-0.206082833	7.66E-13	1.98E-10
TSS200	C12orf42	12	103889798/103889903/103889784/103889789/103889928/103889772	6	cg03882242/cg06664258/cg11342468/cg13702005/cg14380586/cg26381364	0.238222217	0.411233297	0.54528253	0.307060312	1.92E-10	6.19E-09
TSS200	C12orf56	12	64784498/64784483	2	cg13867865/cg15731056	0.208991981	0.256064203	0.47905462	0.27006264	1.60E-06	1.29E-05
TSS200	C12orf74	12	93096780/93096819/93096811	3	cg12755471/cg26383838/cg27576271	0.473736345	0.391258385	0.304769186	-0.168967159	1.57E-10	5.36E-09

TSS200	C13orf16	13	111972854/111972919/111972997/111972898/111972954/111972886	6	cg04752989/cg15511286/cg16384137/cg21308062/cg22102391/cg26299239	0.769141245	0.698106115	0.568651548	-0.200489697	3.84E-09	6.86E-08
TSS200	C13orf30	13	43355514	1	cg24818699	0.725343805	0.683871289	0.530825736	-0.194518069	2.91E-05	0.000170524
TSS200	C13orf38	13	36872166/36872048/36872155/36872135/36872148/36872086	6	cg05927742/cg11678027/cg19437258/cg19801062/cg23003720/cg23710498	0.205579832	0.286229888	0.37322158	0.167641748	2.18E-05	0.000132579
TSS200	C14orf177	14	99177777	1	cg06906435	0.632554745	0.557396089	0.363526641	-0.269028104	1.02E-07	1.12E-06
TSS200	C14orf39	14	60952945/60952933/60952781	3	cg12189551/cg26132298/cg26248878	0.279060523	0.386990292	0.543512184	0.264451662	2.27E-06	1.77E-05
TSS200	C14orf48	14	94463488	1	cg16506185	0.69540914	0.529832594	0.4340211	-0.26138804	6.47E-09	1.05E-07
TSS200	C15orf63	15	44092488/44092606	2	cg02394812/cg24125648	0.4792219	0.410348928	0.307880623	-0.171341277	2.37E-08	3.17E-07
TSS200	C16orf73	16	1922370/1922149/1922155/1922216/1922207	5	cg03467725/cg05041351/cg07775371/cg08959396/cg27308021	0.155952418	0.191239859	0.348454698	0.19250228	5.85E-06	4.08E-05
TSS200	C16orf78	16	49407794	1	cg14933439	0.603215615	0.497054906	0.408461659	-0.194753956	4.56E-09	7.96E-08
TSS200	C16orf82	16	27078053	1	cg17306401	0.458099185	0.350210861	0.270635577	-0.187463608	8.14E-13	2.04E-10
TSS200	C17orf102	17	32906456/32906454/32906394	3	cg04906989/cg08967802/cg09628838	0.102800783	0.123376076	0.260820184	0.158019402	0.000819832	0.003459442
TSS200	C17orf104	17	42733698/42733724/42733729/42733592/42733662/42733600	6	cg02932314/cg12127472/cg12781700/cg12892303/cg15000379/cg17269633	0.327629685	0.414478568	0.574156066	0.246526381	6.40E-08	7.48E-07
TSS200	C17orf46	17	43339512/43339497/43339515/43339589/43339594	5	cg01627847/cg03048083/cg08124910/cg24542751/cg26742995	0.102473477	0.223282182	0.347194491	0.244721014	0.000126967	0.000644812
TSS200	C17orf54	17	71824806	1	cg19786648	0.688294275	0.571451172	0.477503564	-0.210790711	2.49E-09	4.71E-08
TSS200	C17orf66	17	34195902/34196023	2	cg16030510/cg24092442	0.763559448	0.675188539	0.553623868	-0.209935579	1.13E-07	1.23E-06
TSS200	C17orf73	17	48845064/48845052/48845009	3	cg07443932/cg12235260/cg25518968	0.729788185	0.563210294	0.471729965	-0.25805822	1.78E-11	1.22E-09
TSS200	C17orf74	17	7328814/7328921/7328734	3	cg02173388/cg13155863/cg25627098	0.616287973	0.52635305	0.43421705	-0.182070923	2.16E-08	2.93E-07
TSS200	C18orf34	18	31020754/31020806	2	cg00625334/cg05994094	0.264556073	0.34116821	0.511657836	0.247101764	4.57E-07	4.19E-06
TSS200	C19orf21	19	751098/751124	2	cg03018001/cg03230858	0.491678995	0.409519864	0.3357804	-0.155898595	2.72E-10	8.08E-09

TSS200	C1orf110	1	162838743/162838649	2	cg01450736/cg20511534	0.7294112	0.590675372	0.451808127	-0.277603073	5.54E-13	1.75E-10
TSS200	C1orf114	1	169396706/169396858/169396712/169396834/169396785/169396868	6	cg00002719/cg08047907/cg08104202/cg16998150/cg23818870/cg24808280	0.207405182	0.32520246	0.443909426	0.236504245	8.03E-06	5.41E-05
TSS200	C1orf129	1	170904596	1	cg08663653	0.72147691	0.675346506	0.558396341	-0.163080569	1.63E-05	0.000102144
TSS200	C1orf14	1	182922705/182922593/182922582	3	cg02721693/cg02925590/cg22237670	0.43660276	0.284083983	0.213868618	-0.222734142	2.51E-11	1.54E-09
TSS200	C1orf150	1	247712377/247712383	2	cg12769118/cg17518550	0.461933712	0.336924981	0.23501434	-0.226919371	3.55E-07	3.35E-06
TSS200	C1orf173	1	75139583/75139490/75139482/75139470/75139581	5	cg13341453/cg15386773/cg19695945/cg22427475/cg27268184	0.321432568	0.428771639	0.554361991	0.232929423	3.34E-08	4.24E-07
TSS200	C1orf180	1	85100819	1	cg00062020	0.72743088	0.514032939	0.472090786	-0.255340094	8.75E-10	2.03E-08
TSS200	C1orf210	1	43751370/43751270/43751469/43751363/43751376	5	cg00500789/cg04289036/cg09462269/cg12752420/cg23800435	0.528542293	0.450714807	0.366218498	-0.162323795	2.40E-08	3.19E-07
TSS200	C1orf226	1	162351367	1	cg19678111	0.412865015	0.301160994	0.238033195	-0.17483182	1.52E-11	1.12E-09
TSS200	C1orf64	1	16330625	1	cg16510654	0.631408815	0.509595917	0.39826415	-0.233144665	5.54E-12	5.68E-10
TSS200	C1orf70	1	1475742	1	cg15487867	0.078401843	0.225160943	0.516900801	0.438498958	6.79E-10	1.65E-08
TSS200	C1QL2	2	119916510/119916571/119916662/119916581/119916486	5	cg07212778/cg15931721/cg16849609/cg17319142/cg21858380	0.283311327	0.397913469	0.549646376	0.266335049	1.52E-08	2.15E-07
TSS200	C1QTNF8	16	1146421/1146277	2	cg02076607/cg14016390	0.693316705	0.615796867	0.5425053	-0.150811405	3.36E-12	4.48E-10
TSS200	C1S	12	7167781/7167900	2	cg15626285/cg24535475	0.512977363	0.395059083	0.297517975	-0.215459388	5.21E-12	5.51E-10
TSS200	C20orf114	20	31870884/31870935/31870888/31870775	4	cg03033543/cg11035100/cg16762308/cg19378216	0.466077545	0.370986143	0.291087513	-0.174990032	3.80E-12	4.74E-10
TSS200	C20orf118	20	35504511/35504403/35504371/35504553	4	cg00414077/cg09227119/cg09241885/cg18062064	0.481399744	0.381123068	0.317883556	-0.163516188	6.16E-12	6.03E-10
TSS200	C20orf123	20	45179230/45179226/45179354/45179345	4	cg01700462/cg11173131/cg17775490/cg17801864	0.512827775	0.456476589	0.348015814	-0.164811961	6.97E-10	1.68E-08

TSS200	C20orf151	20	61002595/61002657/61002739/61002711	4	cg02537838/cg06154311/cg13290149/cg17565967	0.509861393	0.415810674	0.339791998	-0.170069395	3.46E-12	4.51E-10
TSS200	C20orf79	20	18794246/18794303	2	cg11901715/cg27132814	0.547870935	0.397907622	0.291088214	-0.256782721	2.81E-15	1.23E-11
TSS200	C21orf129	21	43135973/43135949	2	cg01688776/cg26048478	0.49878362	0.405223106	0.322974966	-0.175808654	1.91E-10	6.18E-09
TSS200	C21orf131	21	22175522	1	cg26977737	0.62175238	0.501565889	0.392280795	-0.229471585	4.71E-08	5.70E-07
TSS200	C21orf34	21	17442663/17442803	2	cg12166520/cg19422019	0.680448375	0.556397075	0.460319666	-0.220128709	2.28E-09	4.37E-08
TSS200	C2orf40	2	106681983/106681998/106681937/106681945	4	cg06499647/cg07510423/cg14535980/cg21838979	0.366548016	0.394762189	0.533100355	0.166552338	1.40E-06	1.15E-05
TSS200	C2orf74	2	61372066/61372117/61372138/61372165/61372058/61372226	6	cg01648237/cg05037927/cg16328106/cg19790321/cg23405039/cg24757310	0.38050331	0.560060123	0.670541623	0.290038313	8.57E-11	3.50E-09
TSS200	C3orf32	3	8693756/8693859/8693749/8693781	4	cg01104466/cg02100848/cg06610172/cg15770125	0.461624165	0.372541911	0.307005748	-0.154618417	8.94E-13	2.12E-10
TSS200	C3orf79	3	153202275	1	cg00731062	0.53057651	0.390812894	0.256475114	-0.274101396	6.91E-15	1.49E-11
TSS200	C4BPA	1	207277547/207277466/207277581/207277450	4	cg14856606/cg22491058/cg26430305/cg26514552	0.40844033	0.313015825	0.240104755	-0.168335575	4.49E-11	2.21E-09
TSS200	C4BPB	1	207262190/207262428/207262553	3	cg05659947/cg14740771/cg21571160	0.490815382	0.389026433	0.299401971	-0.19141341	5.95E-11	2.71E-09
TSS200	C4orf26	4	76481169	1	cg22960952	0.47216265	0.342518861	0.27130791	-0.20085474	1.37E-09	2.86E-08
TSS200	C4orf47	4	186350505	1	cg18740810	0.627730495	0.531216306	0.377883173	-0.249847322	1.51E-10	5.20E-09
TSS200	C6orf155	6	72130615/72130647/72130553/72130547/72130539	5	cg11574469/cg15832847/cg22300282/cg23281154/cg26162616	0.43985552	0.532175863	0.633696194	0.193840674	3.41E-06	2.53E-05
TSS200	C6orf186	6	110679566	1	cg20737185	0.166152772	0.251012479	0.434007986	0.267855214	0.00019529	0.000949301
TSS200	C6orf222	6	36304794/36304683	2	cg10550074/cg14673936	0.416463845	0.302862578	0.246320325	-0.17014352	7.28E-11	3.14E-09
TSS200	C8orf12	8	11225875/11225832/11225793	3	cg05445291/cg23282442/cg24901707	0.631553115	0.574388472	0.455623005	-0.17593011	7.24E-09	1.16E-07
TSS200	C8orf85	8	117950454/117950335/117950348/117950395	4	cg05591716/cg07064711/cg07868721/cg08466968	0.329963244	0.408788685	0.50094221	0.170978966	4.93E-08	5.94E-07

TSS200	C8ORFK29	8	145578603/145578687/145578601	3	cg01836096/cg16808444/cg26137121	0.697626427	0.607197796	0.524854617	-0.17277181	3.73E-10	1.04E-08
TSS200	C9orf50	9	132383095	1	cg10640119	0.180273475	0.386498772	0.440187277	0.259913802	2.37E-10	7.19E-09
TSS200	CA10	17	50237205/50237401/50237403/50237576/50237267/50237315	6	cg07398767/cg08605326/cg13125157/cg20552747/cg22855255/cg25592977	0.454836172	0.523712522	0.662433459	0.207597287	4.83E-09	8.31E-08
TSS200	CA3	8	86351018/86350913/86350919/86350889/86350927	5	cg08582356/cg09435929/cg10184889/cg17480760/cg21172458	0.378530393	0.507014574	0.618871927	0.240341534	1.10E-11	9.04E-10
TSS200	CA6	1	9005734/9005910	2	cg02981703/cg06962067	0.617088085	0.509553539	0.437271609	-0.179816476	2.48E-08	3.28E-07
TSS200	CABP5	19	48547362/48547314/48547448	3	cg05308117/cg10149529/cg18534730	0.627265225	0.580844817	0.474680541	-0.152584684	0.000143114	0.000718714
TSS200	CACNA1A	19	13617366/13617382	2	cg18011401/cg26554567	0.142978877	0.199515259	0.312378377	0.1693995	0.000553221	0.00241254
TSS200	CACNA1E	1	181452707/181452618	2	cg04902729/cg21378238	0.313242515	0.407576581	0.493559109	0.180316594	1.41E-08	2.02E-07
TSS200	CACNA1I	22	39966585/39966653/39966711	3	cg03390717/cg11312108/cg18001869	0.694599428	0.613027817	0.51746318	-0.177136248	2.79E-07	2.70E-06
TSS200	CACNG8	19	54466187	1	cg23752563	0.33931252	0.383725244	0.608583723	0.269271203	3.54E-10	9.96E-09
TSS200	CALML4	15	68498589	1	cg08642285	0.37774085	0.264938189	0.217778586	-0.159962264	3.64E-12	4.62E-10
TSS200	CAPG	2	85637834/85637851/85637739	3	cg07215695/cg26476820/cg27139457	0.4685858	0.380579343	0.307348302	-0.161237498	1.63E-11	1.17E-09
TSS200	CAPN14	2	31440451	1	cg15741855	0.83436935	0.748225589	0.620555414	-0.213813936	1.62E-06	1.31E-05
TSS200	CAPS	19	5914009/5914158	2	cg03908904/cg06713671	0.521864175	0.420033217	0.34569362	-0.176170555	9.33E-11	3.69E-09
TSS200	CAPZA3	12	18890872	1	cg06233731	0.659154945	0.580735178	0.465161123	-0.193993822	2.34E-07	2.31E-06
TSS200	CARD14	17	78161482/78152232/78152169/78161572/78161479/78161495/78152219/78161467	8	cg03130276/cg03290213/cg06209035/cg11447922/cg15910686/cg20571913/cg20623503/cg24206431	0.540594582	0.432789598	0.345467708	-0.195126874	2.52E-11	1.54E-09
TSS200	CARD6	5	40841298/40841315	2	cg01784614/cg05305046	0.55928784	0.493785244	0.384101791	-0.175186049	4.62E-05	0.000258164
TSS200	CARTPT	5	71014792/71014820/71014900/71014918/71014857	5	cg02762440/cg08351336/cg20798152/cg22517388/cg26371061	0.422181271	0.494360633	0.590158493	0.167977222	1.28E-10	4.60E-09
TSS200	CASP14	19	15162971/15162821	2	cg15930041/cg23207876	0.710652855	0.603860717	0.538632355	-0.1720205	8.03E-07	6.98E-06

TSS200	CBLN2	18	70211774/70211916/70211835	3	cg05928649/cg26966630/cg27376271	0.117289561	0.189560737	0.292354303	0.175064743	1.10E-05	7.13E-05
TSS200	CBLN4	20	54580133/54580175/54580070/54580196	4	cg00480389/cg06672560/cg20779964/cg25993718	0.44991325	0.490613225	0.63081295	0.1808997	5.55E-10	1.42E-08
TSS200	CCBP2	3	42850848/42850941	2	cg13742532/cg21474351	0.732403503	0.630272103	0.566398802	-0.1660047	2.90E-11	1.66E-09
TSS200	CCDC105	19	15121531/15121516/15121509/15121385	4	cg02489552/cg05923226/cg09470638/cg18739061	0.571636803	0.632686991	0.751769962	0.180133159	3.98E-10	1.09E-08
TSS200	CCDC140	2	223162692/223162797/223162800/223162666	4	cg01790894/cg10416206/cg13767755/cg14093610	0.266659694	0.281424995	0.436827723	0.170168029	3.81E-05	0.000217567
TSS200	CCDC141	2	179749875	1	cg24782949	0.84185333	0.711108883	0.648219109	-0.193634221	8.97E-06	5.98E-05
TSS200	CCDC162	6	109615419/109615455	2	cg02774282/cg15411840	0.509370615	0.404036675	0.311233316	-0.198137299	8.96E-12	7.77E-10
TSS200	CCDC37	3	126113663/126113658/126113682/126113707/126113695/126113743/126113640	7	cg01815671/cg09686443/cg11229862/cg20312228/cg20952286/cg21109744/cg23816347	0.296193755	0.326978721	0.5068705	0.210676745	3.09E-06	2.32E-05
TSS200	CCDC42	17	8648180/8648193/8648175	3	cg15394558/cg19093680/cg27513572	0.706042672	0.554612809	0.448698632	-0.25734404	3.84E-10	1.06E-08
TSS200	CCDC70	13	52436047	1	cg11631011	0.71729355	0.560230817	0.398430955	-0.318862595	1.43E-10	4.99E-09
TSS200	CCDC8	19	46917061/46917018	2	cg03576469/cg18653451	0.416366145	0.515087372	0.651822661	0.235456516	3.61E-07	3.40E-06
TSS200	CCDC81	11	86085715/86085623/86085757/86085694	4	cg10395685/cg16404106/cg18121003/cg18282849	0.327380417	0.444136939	0.489811833	0.162431417	1.96E-05	0.000120121
TSS200	CCK	3	42306524/42306467/42306580/42306493	4	cg03724463/cg05471845/cg07871202/cg21821833	0.391473403	0.434901556	0.57534962	0.183876218	9.49E-06	6.27E-05
TSS200	CCL1	17	32690255/32690367/32690412	3	cg01387036/cg22186223/cg26101277	0.405112055	0.289678217	0.220513923	-0.184598132	5.08E-13	1.67E-10
TSS200	CCL11	17	32612684/32612675	2	cg05999628/cg24870391	0.595159858	0.529180808	0.419075273	-0.176084585	4.69E-07	4.28E-06
TSS200	CCL17	16	57438524	1	cg07634706	0.8068675	0.748396939	0.635426027	-0.171441473	1.72E-06	1.39E-05
TSS200	CCL20	2	228678500	1	cg08575688	0.35453925	0.263878794	0.202312391	-0.152226859	1.58E-09	3.24E-08
TSS200	CCL8	17	32646006	1	cg06083483	0.386547545	0.274091633	0.19501377	-0.191533775	1.30E-10	4.61E-09
TSS200	CCT8L2	22	17073884	1	cg01474260	0.621185775	0.538517511	0.462010945	-0.15917483	3.94E-11	2.01E-09
TSS200	CD1B	1	158301451	1	cg02478603	0.808618955	0.706624789	0.561706105	-0.24691285	1.78E-07	1.81E-06

TSS200	CD300C	17	72542430	1	cg05892213	0.68572244	0.565528433	0.514696636	-0.171025804	8.75E-07	7.54E-06
TSS200	CD40	20	44746823/44746751/44746825/44746902/44746767	5	cg06571407/cg11841529/cg22232207/cg24575067/cg25239996	0.184764494	0.271162715	0.375573884	0.19080939	2.25E-07	2.23E-06
TSS200	CD53	1	111413643	1	cg16896205	0.455283065	0.375745128	0.300335959	-0.154947106	6.12E-12	6.03E-10
TSS200	CD5L	1	157811724/157811790	2	cg03761113/cg11139878	0.69022407	0.550894139	0.444928248	-0.245295822	6.42E-09	1.04E-07
TSS200	CDH10	5	24644988/24645093/24645065/24645024	4	cg02617312/cg05683049/cg17030055/cg26161643	0.200284973	0.257347642	0.414638753	0.21435378	4.72E-09	8.14E-08
TSS200	CDH13	16	82660450/82660464/82660434/82660490/82660505	5	cg01396387/cg07301944/cg08497530/cg08977371/cg09825093	0.279708551	0.336435058	0.447371988	0.167663437	0.000241333	0.001150069
TSS200	CDH18	5	19988368/19988366	2	cg16503924/cg18710278	0.371138315	0.453583656	0.615012223	0.243873908	2.79E-07	2.70E-06
TSS200	CDH19	18	64271341	1	cg16489926	0.75453834	0.579833833	0.40824685	-0.34629149	8.75E-15	1.53E-11
TSS200	CDH2	18	25757555	1	cg06090660	0.197414205	0.281984406	0.417557636	0.220143431	9.04E-07	7.77E-06
TSS200	CDH26	20	58533421/58533443	2	cg14115220/cg20895028	0.568432953	0.466094806	0.394623234	-0.173809718	2.78E-10	8.21E-09
TSS200	CDH8	16	62070184/62070086/62070234/62070219/62070164/62070072	6	cg01718742/cg06831576/cg07681938/cg10241484/cg27120816/cg27444994	0.470606927	0.550407352	0.671704271	0.201097345	6.13E-11	2.77E-09
TSS200	CDO1	5	115152420/115152492/115152494/115152431/115152413/115152485	6	cg02792792/cg08516516/cg11036833/cg14470895/cg16707405/cg23180938	0.272915407	0.463583631	0.59145335	0.318537943	1.28E-09	2.73E-08
TSS200	CEACAM20	19	45033565/45033572/45033577	3	cg10375597/cg13566596/cg20248549	0.78058014	0.667718153	0.539153582	-0.241426558	3.79E-08	4.76E-07
TSS200	CES3	16	66994947	1	cg26538442	0.434121805	0.347465911	0.283320995	-0.15080081	5.04E-09	8.58E-08
TSS200	CES7	16	55909393	1	cg07105499	0.58377174	0.446478756	0.325313445	-0.258458295	1.69E-13	8.86E-11
TSS200	CGA	6	87804962	1	cg12078738	0.473137805	0.3783482	0.279368259	-0.193769546	5.84E-11	2.69E-09
TSS200	CHADL	22	41636941/41637080	2	cg19833679/cg22970173	0.689963155	0.594263394	0.527227418	-0.162735737	3.76E-10	1.04E-08
TSS200	CHL1	3	238615/238611/238618/238496	4	cg07746943/cg08421685/cg12065366/cg25482786	0.32114382	0.329874344	0.537976477	0.216832657	5.80E-10	1.47E-08
TSS200	CHODL	21	19617021/19617145/19617138	3	cg03886449/cg12743978/cg18349138	0.372128911	0.424850881	0.526353994	0.154225083	7.52E-09	1.20E-07

TSS200	CHRM2	7	136553243/136553327/136553882/136553855/136553289/136553778/136553263/136553682/136553822/136553868/136553884/136553728	12	cg04748704/cg05680531/cg07664198/cg08323651/cg09546907/cg10454514/cg17405012/cg22471517/cg23300659/cg24228819/cg24575234/cg25632105	0.313548296	0.418235346	0.566806688	0.253258392	1.45E-11	1.10E-09
TSS200	CHRNA1	2	175629219/175629267/175629224	3	cg15580167/cg18003659/cg21552300	0.70420214	0.591301586	0.46163307	-0.24256907	1.56E-08	2.19E-07
TSS200	CHRNA6	8	42623786/42623730/42623718/42623771/42623686	5	cg06523556/cg07157107/cg20539142/cg27051318/cg27395654	0.412791175	0.268098944	0.183260991	-0.229530184	3.77E-09	6.76E-08
TSS200	CHST10	2	101034279/101034257/101034270	3	cg03853987/cg16882226/cg19283506	0.110492482	0.247492011	0.52275403	0.412261548	5.11E-07	4.64E-06
TSS200	CHST11	12	104850716/104850718/104850759/104850745/104850695/104850767	6	cg01583034/cg03313212/cg04578997/cg05119480/cg12411093/cg21548032	0.077040666	0.127219009	0.235361951	0.158321285	0.00090975	0.003803248
TSS200	CIDEA	18	12254175/12254148/12254566/12254551/12254173/12254469/12254556/12254473/12254153/12254452/12254241/12254120	12	cg00851770/cg03245632/cg05700339/cg07204280/cg12072560/cg12395205/cg16727201/cg17991823/cg18309817/cg21114773/cg21376774/cg26974111	0.335076963	0.456629373	0.535275714	0.200198752	2.69E-06	2.05E-05
TSS200	CLCA1	1	86934365/86934325	2	cg09785991/cg22181664	0.6135676	0.489503294	0.366309359	-0.247258241	2.02E-10	6.49E-09
TSS200	CLDN10	13	96204870/96204856/96204917/96204873/96204854/96204860	6	cg04603730/cg06428163/cg09469554/cg10305311/cg16232183/cg16275739	0.169117983	0.278977054	0.520358225	0.351240242	1.79E-10	5.82E-09
TSS200	CLDN22	4	184242105/184242058	2	cg02882785/cg20141229	0.59442529	0.499899233	0.39533327	-0.19909202	4.28E-08	5.26E-07
TSS200	CLIP4	2	29338258/29338109/29338121/29338113	4	cg03135351/cg13257636/cg23255835/cg23428985	0.354324741	0.520574639	0.639799002	0.285474262	1.32E-09	2.79E-08
TSS200	CLRN1	3	150690810	1	cg06822943	0.41950601	0.305310744	0.227068418	-0.192437592	2.58E-10	7.73E-09

TSS200	CLVS2	6	123317411/123317569/123317487/123317473/123317408	5	cg05009934/cg08194879/cg10182317/cg15749870/cg18366209	0.337506568	0.437158306	0.571718995	0.234212428	3.67E-09	6.61E-08
TSS200	CMTM2	16	66613334/66613220/66613278/66613206/66613273/66613266	6	cg00049664/cg05546863/cg06666025/cg07153665/cg09854734/cg16626067	0.326302001	0.389529445	0.592895287	0.266593286	2.79E-08	3.62E-07
TSS200	CMTM3	16	66638407/66638396/66638438/66638412/66638433/66638210	6	cg06445928/cg08601917/cg08682544/cg09233013/cg26560414/cg26658728	0.109438043	0.271262986	0.43647724	0.327039197	2.55E-07	2.51E-06
TSS200	CMYA5	5	78985489/78985588/78985495/78985484/78985562/78985592	6	cg03546977/cg04154027/cg09655403/cg10257870/cg15197065/cg23279355	0.688829998	0.768865107	0.846506731	0.157676733	9.26E-10	2.12E-08
TSS200	CNBD1	8	87878605	1	cg00374101	0.69649682	0.572691322	0.398347268	-0.298149552	5.85E-11	2.69E-09
TSS200	CNKSR1	1	26503847/26503858/26503862/26503979/26503933	5	cg00300879/cg09890400/cg10096100/cg23602478/cg26412358	0.537256148	0.438973347	0.356895845	-0.180360302	3.02E-10	8.77E-09
TSS200	CNTN1	12	41086274/41086308/41086227/41086282/41086198/41086277	6	cg02622052/cg14436862/cg15087347/cg20574502/cg22560190/cg23500059	0.178425071	0.255709375	0.405423064	0.226997993	8.87E-07	7.64E-06
TSS200	CNTNAP2	7	145813413/145813439/145813417/145813437/145813421/145813432	6	cg05749717/cg07028533/cg11592503/cg16910830/cg21126583/cg23708361	0.267646063	0.322436781	0.459613186	0.191967122	4.81E-06	3.43E-05
TSS200	CNTNAP5	2	124782817/124782831/124782713	3	cg04168670/cg08790440/cg12919006	0.445512275	0.494088333	0.675895377	0.230383102	1.23E-08	1.81E-07
TSS200	COL14A1	8	121137288	1	cg04242021	0.225259055	0.263707654	0.38538923	0.160130175	0.00269389	0.010131158
TSS200	COL15A1	9	101706041/101705939	2	cg13952031/cg18115656	0.188495416	0.24934604	0.373713715	0.185218299	5.44E-05	0.000298407
TSS200	COL17A1	10	105845720	1	cg08509991	0.449985545	0.355990467	0.274267736	-0.175717809	5.49E-10	1.41E-08
TSS200	COL23A1	5	178017669/178017571/178017578/178017667	4	cg08496276/cg23437733/cg25437410/cg26667699	0.058939886	0.151928206	0.27882534	0.219885454	0.001074817	0.004427582
TSS200	COL25A1	4	110223830/110223969/110223980/110223967/110223959	5	cg04188273/cg05791136/cg07095995/cg11979589/cg22266749	0.212437717	0.290883166	0.44103929	0.228601573	0.000180435	0.000884829

TSS200	COL8A1	3	99357437	1	cg19968403	0.15295945	0.204669234	0.326836297	0.173876847	0.000103197	0.000534599
TSS200	COLEC12	18	500817	1	cg16943083	0.118796845	0.134338515	0.334893467	0.216096622	9.29E-05	0.000486486
TSS200	CPA1	7	130020130/130020096	2	cg02303016/cg25718383	0.529344893	0.470278289	0.367697393	-0.161647499	2.22E-09	4.28E-08
TSS200	CPA2	7	129906627	1	cg22213042	0.54288492	0.411284294	0.312412886	-0.230472034	3.37E-12	4.48E-10
TSS200	CPB2	13	46679242	1	cg18634760	0.664349625	0.532603528	0.409007359	-0.255342266	3.61E-11	1.90E-09
TSS200	CPO	2	207804081	1	cg03731898	0.724231925	0.6369456	0.506386923	-0.217845002	8.95E-06	5.97E-05
TSS200	CPXM2	10	125651526/125651549/125651621/125651697/125651588	5	cg03630088/cg05801246/cg08615567/cg08627010/cg13702196	0.291234521	0.35415227	0.444101616	0.152867094	2.04E-06	1.61E-05
TSS200	CR1L	1	207818434/207818395/207818423/207818449	4	cg00115980/cg09557387/cg11752769/cg26750487	0.205639833	0.286118137	0.388702509	0.183062676	9.19E-06	6.10E-05
TSS200	CRHR2	7	30722327/30722163/30722177/30722320	4	cg01718447/cg04863452/cg07658503/cg21773872	0.184872435	0.304892999	0.468855352	0.283982917	1.79E-06	1.43E-05
TSS200	CRISP1	6	49834296	1	cg23370360	0.781384375	0.700545567	0.573292968	-0.208091407	6.80E-06	4.67E-05
TSS200	CRISPLD1	8	75896836/75896798/75896801/75896720/75896645	5	cg01937650/cg04410024/cg14283968/cg15953602/cg21844856	0.248088089	0.316216044	0.455118015	0.207029926	3.87E-09	6.90E-08
TSS200	CRTAC1	10	99790596/99790761/99790593	3	cg11302943/cg14233354/cg23335460	0.22937053	0.276184788	0.412515619	0.183145089	1.70E-05	0.000105905
TSS200	CRYGA	2	209028478/209028399	2	cg11729430/cg24360225	0.607936485	0.515281142	0.429922868	-0.178013617	2.21E-08	2.97E-07
TSS200	CRYGD	2	208989425/208989469/208989382/208989324	4	cg00018606/cg12073779/cg23405212/cg25429719	0.43459905	0.504436481	0.641548767	0.206949717	7.20E-08	8.28E-07
TSS200	CSF3	17	38171530	1	cg23207054	0.43406295	0.356452567	0.280213882	-0.153849068	3.56E-09	6.46E-08
TSS200	CSRNP3	2	166428802/166428866	2	cg24993990/cg26853855	0.422139738	0.334958972	0.265341907	-0.156797831	6.23E-11	2.81E-09
TSS200	CST9L	20	23549522/23549467/23549495/23549525/23549455	5	cg04982166/cg08648286/cg12184157/cg12633918/cg27655855	0.692268265	0.638631	0.523703145	-0.16856512	7.48E-09	1.19E-07
TSS200	CTSE	1	206317403	1	cg21478437	0.578389865	0.471967594	0.400056514	-0.178333351	3.22E-09	5.91E-08
TSS200	CTXN3	5	126984564/126988647	2	cg02743342/cg19804027	0.678026338	0.6133783	0.521053257	-0.156973081	8.00E-08	9.04E-07
TSS200	CXCL14	5	134915085/134915088/134915042	3	cg07516956/cg10179196/cg25011337	0.27851219	0.365116611	0.449525473	0.171013283	7.20E-06	4.91E-05

TSS200	CYP17A1	10	104597307/104597435/104597359	3	cg02721701/cg04471890/cg05885577	0.660912105	0.565726278	0.488894895	-0.17201721	1.37E-06	1.13E-05
TSS200	CYP1A2	15	75041150	1	cg11473616	0.81962889	0.702935961	0.541688595	-0.277940295	1.06E-08	1.60E-07
TSS200	CYP26C1	10	94820892/94820954/94820923/94820945/94820927/94820988	6	cg01416891/cg04564481/cg05219493/cg11234637/cg17743250/cg25083618	0.623799233	0.725643172	0.805741153	0.18194192	1.71E-10	5.66E-09
TSS200	CYP2C9	10	96698366	1	cg09550024	0.493582465	0.394407794	0.327890914	-0.165691551	2.80E-10	8.24E-09
TSS200	CYP2E1	10	135340740/135340721/135340785/135340850	4	cg00436603/cg01465364/cg14250048/cg19571004	0.666363424	0.584704046	0.473894984	-0.19246844	7.59E-09	1.20E-07
TSS200	CYP4F8	19	15725989/15725874	2	cg07512206/cg18680587	0.57535598	0.496007906	0.395980332	-0.179375648	7.77E-08	8.81E-07
TSS200	CYP7B1	8	65711391/65711396/65711369/65711522/65711416	5	cg03535659/cg09975850/cg15160198/cg19424531/cg22287624	0.227793819	0.299743626	0.391811323	0.164017504	0.001131323	0.004633698
TSS200	CYTL1	4	5021311/5021328/5021203/5021236/5021268	5	cg04887807/cg05985589/cg06007271/cg13255282/cg17563034	0.298601852	0.400899528	0.465318106	0.166716254	0.000184837	0.000901825
TSS200	CYYR1	21	27945708	1	cg21039874	0.210667947	0.284675544	0.377040555	0.166372608	1.49E-05	9.43E-05
TSS200	DAB1	1	58716377/58716306/58716257	3	cg06710648/cg11256607/cg26345888	0.371692692	0.441107141	0.559489904	0.187797213	1.54E-05	9.71E-05
TSS200	DAO	12	109273766/109273835/109273780	3	cg01694331/cg18037826/cg25362648	0.591403012	0.510217652	0.415892145	-0.175510866	1.15E-10	4.24E-09
TSS200	DBC1	9	122131895	1	cg12821278	0.38583843	0.46795035	0.665142118	0.279303688	1.60E-06	1.30E-05
TSS200	DBH	9	136501420	1	cg06708731	0.617715115	0.515037606	0.423435255	-0.19427986	7.31E-09	1.17E-07
TSS200	DBX1	11	20181911/20181887/20181879/20182021/20182063/20181877	6	cg02878244/cg03983645/cg06989930/cg11831238/cg16938805/cg18292664	0.37737959	0.465553361	0.594145718	0.216766128	2.69E-08	3.51E-07
TSS200	DBX2	12	45445057/45444902/45444895/45445061/45445023	5	cg12915695/cg13031210/cg13186327/cg16080781/cg26274596	0.205081183	0.259889082	0.434139299	0.229058116	5.20E-05	0.000286754
TSS200	DCC	18	49866412/49866531/49866407/49866371/49866361	5	cg00027400/cg07897248/cg07935012/cg19042459/cg19613722	0.267360484	0.321942894	0.458893908	0.191533424	3.62E-06	2.66E-05
TSS200	DCD	12	55042293	1	cg25372195	0.53370068	0.415094111	0.301568245	-0.232132435	8.05E-11	3.33E-09

TSS200	DCLK1	13	36705622/36705606/36705475/36705608	4	cg24239329/cg25012919/cg25724283/cg25943503	0.308429298	0.336695052	0.48766075	0.179231453	0.001575491	0.006264125
TSS200	DDX25	11	125774092/125774090/125774082	3	cg01736784/cg04609163/cg23727983	0.494427625	0.631675983	0.748766741	0.254339116	3.99E-11	2.02E-09
TSS200	DEFA6	8	6783670	1	cg08312330	0.76306387	0.602948422	0.492708895	-0.270354975	1.01E-12	2.17E-10
TSS200	DEFB121	20	29994173/29994089	2	cg04476891/cg23204384	0.5179359	0.4071666	0.2997649	-0.218171	1.73E-11	1.20E-09
TSS200	DEFB124	20	30060884/30060822/30060868/30061002	4	cg02612927/cg03900072/cg13405782/cg17418055	0.558587691	0.472635844	0.349981488	-0.208606204	2.59E-12	3.96E-10
TSS200	DEFB125	20	68170	1	cg08088390	0.547577015	0.441089389	0.388875386	-0.158701629	3.62E-06	2.66E-05
TSS200	DEFB129	20	207858/207803	2	cg02046532/cg26281051	0.77596844	0.667373461	0.546815114	-0.229153326	1.28E-07	1.36E-06
TSS200	DEFB134	8	11853920/11853878	2	cg01468193/cg02101571	0.619004203	0.478109281	0.363018307	-0.255985896	3.23E-10	9.22E-09
TSS200	DGKG	3	186080180/186080043/186080208/186080023	4	cg02567082/cg14601136/cg15738605/cg25808906	0.100521938	0.145630393	0.326807706	0.226285768	2.75E-06	2.09E-05
TSS200	DGKI	7	137531612/137531801/137531791/137531697	4	cg04425632/cg18324707/cg22428147/cg23299133	0.326016445	0.368118769	0.508073432	0.182056987	2.52E-06	1.94E-05
TSS200	DKFZP434H168	16	56228498/56228511/56228505/56228577/56228467/56228495/56228442	7	cg00246932/cg01157070/cg02667667/cg06780839/cg07700514/cg07829842/cg19047292	0.113126065	0.229547982	0.505039465	0.391913399	6.59E-08	7.66E-07
TSS200	DKK3	11	12031099/12030268/12031097/12030639/12030278/12031093/12031113/12030643/12030375/12030187/12030272/12030648/12030289/12031046	14	cg01286655/cg04100696/cg05106294/cg06188670/cg06943667/cg08662017/cg12668155/cg13216057/cg13259205/cg16915821/cg17039391/cg18768414/cg20973347/cg24543696	0.204293821	0.235952564	0.379257316	0.174963495	2.50E-05	0.000148941
TSS200	DLGAP1	18	3845348	1	cg27602828	0.753566085	0.705258728	0.592490614	-0.161075471	9.29E-05	0.000486613
TSS200	DNAH11	7	21582758/21582749/21582745	3	cg01122187/cg08570341/cg16474297	0.158832395	0.15329108	0.341847322	0.183014927	0.000637313	0.002744346
TSS200	DNAH8	6	38690530	1	cg24883664	0.56260724	0.428523194	0.342720082	-0.219887158	4.08E-12	4.87E-10
TSS200	DNAJC15	13	43597297/43597331	2	cg05035143/cg12504148	0.69693372	0.556005967	0.52909105	-0.16784267	0.00408135	0.014730787

TSS200	DOCK2	5	169064071	1	cg21771173	0.213913895	0.308646972	0.386503018	0.172589123	3.24E-05	0.00018734
TSS200	DOK5	20	53092233/53092259	2	cg00741836/cg27341128	0.209063085	0.261758188	0.426475869	0.217412784	0.000110314	0.000568106
TSS200	DPCR1	6	30908739/30908706/30908620/30908579	4	cg04572540/cg13978352/cg15480919/cg24836242	0.570527761	0.480700028	0.413546302	-0.156981459	6.99E-08	8.07E-07
TSS200	DPP6	7	153749759/154002270/154002312/153749738/153749756/153749736/153584416	7	cg02485566/cg05640128/cg07590961/cg09124223/cg15549927/cg18940047/cg22620221	0.387334567	0.460988191	0.583931457	0.19659689	3.61E-10	1.01E-08
TSS200	DPPA2	3	109035405/109035469/109035383/109035381	4	cg01895333/cg11759378/cg18098079/cg18992201	0.848832195	0.775281698	0.639093261	-0.209738934	9.16E-07	7.87E-06
TSS200	DPPA3	12	7864076/7864058/7864013/7864047	4	cg06872381/cg15873123/cg17723761/cg22205501	0.797260523	0.736395638	0.627559497	-0.169701026	1.64E-06	1.32E-05
TSS200	DPPA5	6	74063982/74064149/74064047/74064140/74064064	5	cg01652244/cg09071762/cg15414745/cg18052665/cg23414861	0.589432718	0.513170675	0.419295386	-0.170137331	2.10E-05	0.000128069
TSS200	DPY19L2	12	64062505/64062544/64062542/64062526/64062384/64062498	6	cg02660347/cg06995920/cg10967101/cg17477445/cg21346925/cg23148701	0.29425179	0.373861847	0.518522093	0.224270303	1.17E-08	1.73E-07
TSS200	DRD4	11	637173/637170/637175/637162	4	cg00556112/cg06825142/cg12928379/cg20931042	0.132138934	0.124251499	0.296712788	0.164573854	0.002212486	0.008507361
TSS200	DSCAM	21	42219193/42219139/42219202/42219211/42219221	5	cg07962392/cg12135856/cg12201779/cg12409525/cg19776080	0.387601025	0.444155772	0.58755047	0.199949445	1.54E-07	1.60E-06
TSS200	DSG3	18	29027701/29027658	2	cg03238162/cg06559576	0.48270069	0.38610165	0.293976427	-0.188724263	2.70E-09	5.07E-08
TSS200	DSPP	4	88529516/88529584	2	cg04503912/cg05292310	0.612353905	0.446710172	0.312553486	-0.299800419	1.93E-13	9.53E-11
TSS200	DUSP13	10	76859383/76859441/76859295/76859248/76859323/76859350	6	cg04369919/cg10522115/cg11709896/cg12256803/cg19532742/cg24634577	0.632840253	0.542655666	0.440210864	-0.192629389	1.73E-11	1.20E-09
TSS200	DYDC1	10	82116571/82116596	2	cg01527459/cg12640394	0.2575856	0.335183755	0.495825223	0.238239623	0.000142361	0.000715391

TSS200	DYDC2	10	82116382/82116365/82116446/82116405/82116392/82116456	6	cg11036831/cg11854806/cg12091732/cg13087076/cg16771783/cg25535587	0.191602304	0.26811273	0.413836301	0.222233996	0.00010477	0.000541858
TSS200	DZIP1	13	96296983/96296997/96297051/96297053/96297058/96296979	6	cg02788400/cg05505803/cg15792252/cg18206952/cg24107021/cg26886381	0.175503546	0.211596428	0.332465496	0.156961951	0.001616396	0.006413926
TSS200	EDNRB	13	78493100/78493012/78493066/78493126/78493128	5	cg04390523/cg06179060/cg06971129/cg13818654/cg15836660	0.245177538	0.395607701	0.575186885	0.330009348	2.32E-11	1.46E-09
TSS200	EFEMP1	2	56150942/56150478/56150475/56151071/56150549	5	cg05168033/cg05385513/cg16100120/cg16118212/cg25046074	0.310631115	0.377432707	0.500137535	0.18950642	1.55E-06	1.27E-05
TSS200	EFHA2	8	16884576/16884555/16884549	3	cg00720475/cg05956498/cg18577280	0.119189488	0.235860501	0.466411998	0.34722251	1.88E-08	2.58E-07
TSS200	EFHB	3	19975738	1	cg08219773	0.594174605	0.495843889	0.419835668	-0.174338937	9.13E-09	1.41E-07
TSS200	EFS	14	23834995/23834860/23834909/23834891/23834977/23834985	6	cg02213260/cg11840125/cg13011388/cg14370314/cg18844382/cg21013866	0.272455322	0.338436705	0.487286281	0.214830959	1.61E-06	1.30E-05
TSS200	EHD3	2	31456761/31456789/31456747/31456775/31456741/31456804	6	cg00981472/cg05882522/cg06773122/cg18444347/cg25202298/cg27230038	0.065592762	0.163218186	0.232593569	0.167000807	0.001246305	0.005071739
TSS200	EID3	12	104697389/104697545/104697387/104697419/104697526/104697532/104697514	7	cg03817911/cg09477407/cg18633684/cg20923245/cg21234561/cg26614816/cg27205904	0.245901051	0.400084094	0.594826503	0.348925453	1.98E-10	6.39E-09
TSS200	ELANE	19	852284/852114	2	cg03633458/cg06100973	0.583008765	0.450556036	0.389131357	-0.193877408	1.70E-12	2.88E-10
TSS200	ELK4	1	205602036/205602113	2	cg16894746/cg23201841	0.543342913	0.442797003	0.379610564	-0.163732349	3.35E-10	9.53E-09
TSS200	ELMO3	16	67232921	1	cg26178184	0.596437915	0.492490056	0.4066583	-0.189779615	2.47E-10	7.45E-09
TSS200	EMID2	7	101006063/101006019/101006089/101006035/101006052/101006058	6	cg14874750/cg17361163/cg18762727/cg23424003/cg27179533/cg27528510	0.313617047	0.41950658	0.576084525	0.262467479	1.72E-07	1.76E-06

TSS200	EMILIN3	20	39995652/39995649/39995546/39995654/39995539/39995542	6	cg07609788/cg08463758/cg13919438/cg18587340/cg23226129/cg25481073	0.187842254	0.212291102	0.352683859	0.164841605	7.61E-06	5.16E-05
TSS200	EMR4P	19	6990919	1	cg11579116	0.467739415	0.366626939	0.292965636	-0.174773779	8.09E-10	1.90E-08
TSS200	EPHA10	1	38230887/38230827/38230834/38230845	4	cg06163371/cg07447922/cg18537001/cg21306240	0.373490368	0.466220863	0.59515204	0.221661672	2.39E-06	1.85E-05
TSS200	EPHA5	4	66535732/66535655	2	cg01101647/cg24620673	0.19284365	0.2527522	0.35487468	0.16203103	6.84E-05	0.000366248
TSS200	EPHA7	6	94129431/94129481/94129412	3	cg03696441/cg08734918/cg19504032	0.175495591	0.174092491	0.326565406	0.151069815	0.004098518	0.014789364
TSS200	EPO	7	100318319/100318223/100318321/100318239	4	cg01692340/cg07841815/cg13207797/cg26071806	0.119480817	0.14771532	0.292341055	0.172860238	0.001415413	0.005685084
TSS200	EPS8L3	1	110306591/110306642	2	cg00491404/cg03956353	0.517413713	0.423706358	0.347689391	-0.169724322	4.82E-11	2.34E-09
TSS200	ESPNL	2	239008929/239008943	2	cg02413938/cg19391697	0.65977344	0.568363578	0.494395164	-0.165378276	6.64E-08	7.71E-07
TSS200	EVC	4	5712884/5712840/5712842	3	cg01333350/cg06918887/cg25213928	0.127460136	0.230972145	0.452348692	0.324888556	3.37E-07	3.20E-06
TSS200	EVC2	4	5710411/5710407/5710403/5711301/5710319/5710372/5710337	7	cg01834022/cg05542338/cg06426416/cg09558315/cg14654886/cg17255450/cg27434509	0.422789143	0.5009266	0.635948132	0.213158989	1.04E-07	1.14E-06
TSS200	EVPL	17	74023630/74023625/74023633	3	cg18657389/cg19927565/cg25255429	0.534442692	0.460397057	0.381546762	-0.15289593	1.45E-10	5.04E-09
TSS200	EYA4	6	133562332/133562461/133562466/133562463/133562479/133562470/133562485/133562336/133562334/133562475/133562492	11	cg01328892/cg05085230/cg06132028/cg08712932/cg11664500/cg11942956/cg14287112/cg16347317/cg17838029/cg20787173/cg22871668	0.202485412	0.319657004	0.467307546	0.264822134	1.29E-06	1.07E-05
TSS200	FABP4	8	82395569	1	cg19422565	0.687758385	0.65046045	0.498557782	-0.189200603	1.08E-09	2.37E-08
TSS200	FAIM2	12	50297793/50297777	2	cg06947913/cg18486102	0.294380946	0.37885231	0.542116516	0.24773557	4.02E-05	0.000228258
TSS200	FAM102A	9	130713088	1	cg13692655	0.679282115	0.563830344	0.513330286	-0.165951829	2.19E-10	6.83E-09

TSS200	FAM123A	13	25745946/25745986/25746007/25746012	4	cg03369269/cg18034232/cg22141111/cg24296626	0.267819258	0.346982947	0.456655305	0.188836047	1.58E-07	1.63E-06
TSS200	FAM151A	1	55089365/55089372	2	cg04919581/cg13458781	0.412900655	0.315883683	0.258974023	-0.153926632	2.27E-09	4.36E-08
TSS200	FAM155A	13	108519554/108519547	2	cg10118210/cg10245910	0.363702403	0.376628327	0.540404452	0.17670205	0.001723238	0.006800236
TSS200	FAM162B	6	117087083/117087010/117087056/117086931/117086941/117086996	6	cg03137522/cg08849150/cg09251292/cg13631916/cg24805875/cg27550270	0.213215596	0.254507884	0.398737478	0.185521882	7.69E-07	6.70E-06
TSS200	FAM170A	5	118965233/118965093/118965199/118965202	4	cg01229506/cg06903010/cg23314948/cg24537512	0.792483791	0.723170746	0.609695519	-0.182788272	3.15E-09	5.79E-08
TSS200	FAM170B	10	50342081/50342114/50342145	3	cg12771178/cg14924620/cg16512990	0.575756508	0.464462764	0.358137361	-0.217619146	3.51E-11	1.88E-09
TSS200	FAM183A	1	43613567/43613565/43613440/43613433/43613494	5	cg04900427/cg05125578/cg15961211/cg15997906/cg22608128	0.195339208	0.243309023	0.421580725	0.226241517	0.000140221	0.000705535
TSS200	FAM183B	7	38726758	1	cg03517998	0.536463415	0.393821633	0.296173718	-0.240289697	8.13E-13	2.04E-10
TSS200	FAM184B	4	17783205/17783294/17783261/17783240/17783331/17783322	6	cg01886556/cg02108033/cg05624214/cg15917116/cg18790597/cg27207796	0.215687576	0.234713841	0.420879943	0.205192367	0.000235566	0.001124966
TSS200	FAM19A1	3	68053273/68053371	2	cg09533390/cg22706186	0.676065713	0.569016886	0.460938423	-0.21512729	1.69E-08	2.34E-07
TSS200	FAM19A4	3	68981866/68981854/68981877/68981852/68981890/68981895	6	cg09868336/cg12219082/cg12412079/cg12417685/cg13921352/cg20375342	0.25409692	0.303376729	0.531583568	0.277486648	1.70E-07	1.74E-06
TSS200	FAM19A5	22	48971959	1	cg04198308	0.131805311	0.240858172	0.383621199	0.251815888	5.07E-10	1.32E-08
TSS200	FAM20A	17	66554600/66554677	2	cg10374534/cg24909667	0.771803725	0.673444878	0.582990091	-0.188813634	1.57E-08	2.20E-07
TSS200	FAM24A	10	124670078	1	cg18822598	0.724795685	0.642023011	0.492706173	-0.232089512	3.67E-06	2.69E-05
TSS200	FAM25A	10	88779914/88779935/88779941	3	cg04807739/cg15263682/cg23424357	0.702269747	0.610583219	0.520554547	-0.1817152	9.92E-11	3.89E-09
TSS200	FAM26D	6	116850057	1	cg03299329	0.685941595	0.567968856	0.500291	-0.185650595	5.65E-09	9.41E-08
TSS200	FAM26E	6	116832794/116832611	2	cg14547726/cg21741010	0.476882028	0.388619914	0.297254395	-0.179627632	6.30E-10	1.56E-08

TSS200	FAM38B	18	11148914/11148783/11148932/11148776/11148769/11148832	6	cg03602280/cg10023530/cg12951849/cg23556443/cg24362812/cg25995825	0.424474602	0.542645269	0.655660971	0.23118637	7.52E-11	3.20E-09
TSS200	FAM43B	1	20878928/20878760/20878811/20878787	4	cg09511914/cg15142222/cg19507093/cg26557658	0.181229409	0.261726838	0.368824624	0.187595214	7.24E-06	4.94E-05
TSS200	FAM58B	1	200182508	1	cg17956775	0.707585145	0.576531939	0.454063423	-0.253521722	5.21E-09	8.78E-08
TSS200	FAM5B	1	177140510/177140609	2	cg19993589/cg21523908	0.308914248	0.453156744	0.561300273	0.252386025	4.00E-07	3.72E-06
TSS200	FAM83C	20	33880266/33880337/33880353/33880370	4	cg20555922/cg24855818/cg26826207/cg27629776	0.737102603	0.654619175	0.568809005	-0.168293598	4.88E-08	5.89E-07
TSS200	FAM83E	19	49116839/49116824/49116870	3	cg10772322/cg20082196/cg27530053	0.54354827	0.452141297	0.352447482	-0.191100788	1.72E-10	5.66E-09
TSS200	FAM99A	11	1686642	1	cg04353359	0.72733742	0.658173006	0.528193814	-0.199143606	1.08E-08	1.61E-07
TSS200	FAT4	4	126237464/126237371/126237421/126237373/126237513	5	cg03527919/cg05118638/cg08644023/cg10731073/cg15795630	0.191660035	0.241493606	0.408754979	0.217094944	3.24E-06	2.42E-05
TSS200	FBLIM1	1	16085148	1	cg04897742	0.070202859	0.292923521	0.476116717	0.405913859	1.29E-09	2.74E-08
TSS200	FBXL21	5	135265931/135265849/135265937	3	cg13557668/cg20924470/cg26664161	0.258532948	0.330707628	0.44757673	0.189043782	0.000113538	0.000582808
TSS200	FBXO39	17	6679495/6679482/6679499/6679484/6679476/6679541	6	cg02093112/cg06091013/cg07540103/cg08843809/cg11871421/cg17097923	0.414734219	0.514230606	0.680763773	0.266029554	8.70E-11	3.54E-09
TSS200	FCGR3A	1	161519989	1	cg18298815	0.60392521	0.507207417	0.405779795	-0.198145415	5.88E-10	1.48E-08
TSS200	FCRL3	1	157670710/157670825	2	cg08786003/cg15602298	0.71051115	0.669765689	0.479001236	-0.231509914	2.15E-05	0.000131005
TSS200	FER1L4	20	34195484	1	cg06575425	0.44958139	0.359398572	0.273202859	-0.176378531	3.63E-11	1.90E-09
TSS200	FERD3L	7	19185139/19185165	2	cg05328547/cg11241627	0.31792262	0.423956408	0.554786209	0.236863589	2.44E-09	4.63E-08
TSS200	FERMT1	20	6104261/6104215/6104267/6104274	4	cg07718903/cg09539538/cg15692360/cg26758396	0.416649188	0.312095402	0.243597807	-0.173051381	2.98E-12	4.35E-10
TSS200	FFAR1	19	35842431	1	cg14978687	0.79397802	0.681085172	0.5487251	-0.24525292	2.76E-10	8.14E-09
TSS200	FFAR2	19	35940461/35940483/35940593/35940425	4	cg02361306/cg03062717/cg18755094/cg20199836	0.473830825	0.379270826	0.280947162	-0.192883663	1.10E-10	4.18E-09
TSS200	FGA	4	155511987	1	cg12827188	0.40421215	0.309626172	0.249576477	-0.154635673	8.47E-11	3.49E-09

TSS200	FGD4	12	32655034/32654929/32654992	3	cg05681757/cg11809958/cg22431093	0.435127108	0.361625136	0.28116905	-0.153958058	3.08E-09	5.67E-08
TSS200	FGF10	5	44388799/44388972/44388907/44388949/44388958	5	cg00167491/cg00272200/cg02443428/cg16384588/cg25103492	0.268241866	0.356122283	0.459234148	0.190992282	2.23E-08	3.00E-07
TSS200	FGF12	3	192445514/192126996/192445500/192445533/192445538/192126935/192445388/192126849/192445582/192126960	10	cg00902847/cg01257828/cg02403395/cg06374165/cg06804312/cg15048660/cg15104158/cg16366473/cg22069098/cg23207710	0.187608949	0.27125367	0.409443492	0.221834543	8.29E-06	5.57E-05
TSS200	FGF2	4	123747750/123747672	2	cg04332818/cg15128226	0.47547618	0.600769	0.715658059	0.240181879	1.79E-09	3.57E-08
TSS200	FGF3	11	69634240/69634296/69634334/69634363/69634384/69634372	6	cg00043379/cg04495670/cg06329574/cg15119027/cg15554555/cg24359323	0.316839583	0.374040917	0.500367588	0.183528005	1.84E-05	0.000113673
TSS200	FGF4	11	69590287/69590332/69590309/69590282/69590328	5	cg00022558/cg02882381/cg03631755/cg18947739/cg24199112	0.196365536	0.227998034	0.347300778	0.150935242	0.000580637	0.002522334
TSS200	FGF5	4	81187601/81187605/81187610	3	cg10031614/cg12528713/cg20528583	0.140989149	0.219427081	0.391214603	0.250225454	3.59E-06	2.64E-05
TSS200	FIGN	2	164592568/164592561/164592571	3	cg06564900/cg15259986/cg19156483	0.330015127	0.401965244	0.537242421	0.207227295	0.000109712	0.000565559
TSS200	FLG	1	152297873	1	cg19855573	0.48787534	0.363211183	0.25841335	-0.22946199	9.86E-14	6.22E-11
TSS200	FLG2	1	152332651	1	cg17542852	0.43763764	0.315574994	0.253628518	-0.184009122	1.44E-12	2.62E-10
TSS200	FLI1	11	128563716/128563634/128563714/128562353/128563618/128562297/128562245/128563685	8	cg02526522/cg08590601/cg13755070/cg14570838/cg18142615/cg19296671/cg19482025/cg19897940	0.17102074	0.25094951	0.387007941	0.215987201	3.47E-05	0.000199374
TSS200	FLJ16779	20	61885236/61885285/61885270/61885249/61885291/61885262	6	cg06763829/cg07785314/cg09196068/cg15571277/cg18412834/cg21258057	0.069564278	0.171933272	0.37376137	0.304197093	6.05E-07	5.41E-06
TSS200	FLJ23834	7	105603622	1	cg25747783	0.69548239	0.574931128	0.46851185	-0.22697054	4.74E-07	4.32E-06

TSS200	FLJ33360	5	6337408/6337520/6337430	3	cg05940966/cg23328308/cg26874163	0.713688583	0.630555587	0.562330502	-0.151358082	2.00E-06	1.58E-05
TSS200	FLJ40330	2	89065379/89065330/89065319/89065350	4	cg02646394/cg17108748/cg20584997/cg23042796	0.253752197	0.309471928	0.436620545	0.182868348	9.89E-07	8.45E-06
TSS200	FLJ42393	3	187896222/187896277	2	cg21909391/cg24454374	0.53177815	0.407245044	0.314350191	-0.217427959	7.62E-11	3.23E-09
TSS200	FLJ42875	1	2984470/2984445	2	cg07363855/cg11229543	0.363139135	0.435250697	0.557406816	0.194267681	2.29E-05	0.000138149
TSS200	FLJ43390	14	62584024/62583968/62584005/62584037/62583919/62583962	6	cg01613638/cg01866038/cg05173737/cg10227327/cg14926717/cg15897970	0.139913616	0.27133286	0.422528908	0.282615292	2.50E-08	3.29E-07
TSS200	FLJ43860	8	142517474/142517440/142517341/142517494	4	cg00328935/cg04744713/cg25697881/cg26654519	0.569931713	0.463472271	0.418773811	-0.151157901	1.02E-08	1.55E-07
TSS200	FLRT2	14	85996352/85996393/85996364/85996315	4	cg01537995/cg04975505/cg16800165/cg23400512	0.355363578	0.460736528	0.564026858	0.208663279	1.40E-07	1.47E-06
TSS200	FMO1	1	171217592/171217555	2	cg04413226/cg15514848	0.76698708	0.706803978	0.58903115	-0.17795593	1.20E-06	1.01E-05
TSS200	FNDC1	6	159590231	1	cg00157796	0.201576911	0.360219017	0.580746665	0.379169755	2.22E-09	4.28E-08
TSS200	FNDC7	1	109255376/109255473/109255480	3	cg01826444/cg12483833/cg23041045	0.650664255	0.567170378	0.466373036	-0.184291219	9.32E-09	1.43E-07
TSS200	FOLR1	11	71902997/71903119/71900411	3	cg06400204/cg14818960/cg15418509	0.64784956	0.560432781	0.497206102	-0.150643458	7.61E-08	8.65E-07
TSS200	FOXG1	14	29236221	1	cg17525102	0.369317025	0.4401068	0.57813315	0.208816125	2.57E-06	1.98E-05
TSS200	FOXI1	5	169532775/169532758/169532721/169532752	4	cg03817107/cg08295639/cg11552829/cg19233472	0.495492788	0.400371043	0.344017725	-0.151475063	2.56E-09	4.85E-08
TSS200	FOXI2	10	129535348/129535509/129535378/129535458	4	cg08829841/cg09614415/cg16642284/cg25576011	0.528354993	0.543296683	0.683065261	0.154710267	4.32E-05	0.000243158
TSS200	FPR2	19	52264411/52266482/52264413/52264429	4	cg16052198/cg16726204/cg24964368/cg25653204	0.606611637	0.677628937	0.758661003	0.152049366	4.08E-09	7.22E-08
TSS200	FREM3	4	144621938/144621952/144621898/144621971/144621945	5	cg08334984/cg09556043/cg10056356/cg15042811/cg17495719	0.276775177	0.444996611	0.597750659	0.320975482	6.48E-09	1.05E-07

TSS200	FRK	6	116381966/116381976/116381957	3	cg05304507/cg15226275/cg18764771	0.471421303	0.38271912	0.310020133	-0.16140117	1.64E-10	5.51E-09
TSS200	FRMD6	14	52118390/51955716	2	cg09410986/cg18894781	0.676334455	0.565851583	0.438723055	-0.2376114	2.51E-11	1.54E-09
TSS200	FTCD	21	47575498/47575504/47575547	3	cg09436823/cg18024037/cg25322086	0.858618705	0.786953669	0.692557889	-0.166060816	5.02E-06	3.55E-05
TSS200	FURIN	15	91411838	1	cg02864248	0.607789675	0.482099811	0.383847264	-0.223942411	3.46E-11	1.87E-09
TSS200	FUT2	19	49199172/49199217/49199029	3	cg01656853/cg08873673/cg20441902	0.456567493	0.363231854	0.27694748	-0.179620013	1.23E-11	9.73E-10
TSS200	FUT9	6	96463710	1	cg01489686	0.325684825	0.383907833	0.511040618	0.185355793	2.44E-05	0.000146295
TSS200	FXYD4	10	43867064/43867041/43867024/43866966/43867071	5	cg00827318/cg03687765/cg22992966/cg24294544/cg25081201	0.493938703	0.413418148	0.335468974	-0.158469729	1.16E-10	4.28E-09
TSS200	FZD10	12	130647004	1	cg21885046	0.255520285	0.31653685	0.479572268	0.224051983	1.65E-10	5.51E-09
TSS200	G6PC2	2	169757618/169757561	2	cg00689835/cg01111179	0.50789981	0.410616317	0.302426995	-0.205472815	2.24E-10	6.93E-09
TSS200	GABBR2	9	101471553	1	cg18507707	0.10555472	0.163371657	0.275268034	0.169713314	0.00027156	0.001275994
TSS200	GABRA5	15	27112197/27111774/27111699/27111749/27111687/27112119	6	cg05044185/cg05614333/cg12555380/cg14417760/cg15035033/cg15665646	0.391612329	0.484264828	0.571898394	0.180286065	5.16E-08	6.16E-07
TSS200	GABRA6	5	161112530	1	cg02703002	0.649196995	0.480224794	0.373673241	-0.275523754	9.96E-10	2.23E-08
TSS200	GAD2	10	26505127/26505069/26505156	3	cg00733780/cg04838147/cg23050442	0.234790059	0.393877345	0.584842092	0.350052034	2.85E-09	5.29E-08
TSS200	GAL3ST2	2	242716203/242716183	2	cg03898010/cg04140903	0.53114712	0.453668944	0.372693909	-0.158453211	1.11E-09	2.42E-08
TSS200	GAL3ST3	11	65816809/65816819/65816655	3	cg04337534/cg09700701/cg26763523	0.420913183	0.491998972	0.598047577	0.177134394	6.72E-10	1.64E-08
TSS200	GALNT13	2	154728308	1	cg02593932	0.38300914	0.394353661	0.590584686	0.207575546	7.49E-08	8.56E-07
TSS200	GALNT14	2	31361687/31361593/31361701/31361760/31361692/31361757	6	cg04050000/cg10258271/cg14514751/cg20599798/cg22395019/cg26988215	0.127646205	0.155388395	0.307139157	0.179492953	2.25E-05	0.000135892
TSS200	GALNT9	12	132690692/132690651/132906010/132690752/132690643	5	cg08957127/cg09879122/cg12075445/cg14591001/cg27483007	0.72790701	0.682352328	0.55231575	-0.17559126	2.89E-05	0.000169176
TSS200	GALNTL5	7	151653454	1	cg11091262	0.51972099	0.397371144	0.304276432	-0.215444558	1.18E-11	9.37E-10

TSS200	GALNTL6	4	172734514/172734507/172734431	3	cg09772954/cg14307443/cg22153181	0.275216999	0.353055264	0.547891504	0.272674505	1.00E-08	1.52E-07
TSS200	GALR1	18	74961809/74961966/74962000/74961968	4	cg03032214/cg03659519/cg17911318/cg20872937	0.361228119	0.356662058	0.524020253	0.162792135	0.000752631	0.003198124
TSS200	GALR2	17	74070698	1	cg07274618	0.38491863	0.425699411	0.566461882	0.181543252	3.90E-09	6.94E-08
TSS200	GAPT	5	57787167	1	cg26453169	0.417140745	0.297783261	0.214805513	-0.202335232	2.77E-06	2.11E-05
TSS200	GAST	17	39868610/39868441	2	cg08928038/cg23010077	0.687027015	0.5970171	0.494716818	-0.192310197	4.25E-08	5.23E-07
TSS200	GATA4	8	11561593/11561531/11561584/11561620	4	cg00397986/cg02726121/cg09951349/cg14900471	0.206063138	0.286203333	0.487388156	0.281325018	3.94E-08	4.90E-07
TSS200	GATA5	20	61051036/61051029/61051039/61051032	4	cg02484469/cg13845982/cg14980983/cg20265733	0.239226922	0.330063522	0.460847161	0.221620239	0.000207261	0.001001012
TSS200	GATM	15	45671028/45671018/45671155/45671148/45671016/45671001	6	cg04025675/cg05896902/cg18435832/cg20381115/cg24328539/cg26796135	0.486115712	0.586891059	0.673496633	0.187380921	1.83E-09	3.65E-08
TSS200	GBGT1	9	136039474	1	cg09513259	0.223261225	0.278216422	0.382270164	0.159008939	6.93E-05	0.000370486
TSS200	GCET2	3	111852227/111852312/111852156	3	cg06510261/cg12663553/cg25462303	0.459048548	0.547589572	0.6277133	0.168664753	1.11E-08	1.66E-07
TSS200	GCM1	6	53013647	1	cg20967220	0.57719989	0.438029878	0.343543877	-0.233656013	3.59E-11	1.90E-09
TSS200	GCM2	6	10882238/10882240/10882250	3	cg09829319/cg14250833/cg20229534	0.197304442	0.244470093	0.457785477	0.260481035	2.79E-05	0.000164077
TSS200	GCNT3	15	59903968	1	cg00437411	0.48122003	0.396078944	0.315942755	-0.165277275	4.37E-10	1.17E-08
TSS200	GDEP	4	80748552	1	cg07124719	0.6897729	0.531455989	0.462399959	-0.227372941	1.69E-11	1.19E-09
TSS200	GDF6	8	97173034	1	cg21859781	0.236787125	0.334630139	0.483549077	0.246761953	9.68E-08	1.07E-06
TSS200	GFAP	17	42992917/42992924/42993050/42992976	4	cg01769243/cg03383006/cg08156531/cg21601845	0.780574764	0.701050817	0.61741326	-0.163161504	1.79E-10	5.82E-09
TSS200	GFRA1	10	118031864/118032892/118033312/118032872/118031950/118032879/118031870/118032948/118032987/118033273/118033290/118032905	12	cg06298519/cg08194313/cg11390957/cg13346013/cg17256532/cg18672939/cg19485539/cg23898073/cg25022687/cg25351565/cg25617725/cg27341926	0.201009229	0.301030061	0.451227324	0.250218095	2.32E-05	0.000139397

TSS200	GGT6	17	4464022/4464011/4463911/4464075/4464024	5	cg00145141/cg00382185/cg01984798/cg02986643/cg03044452	0.534290296	0.441044603	0.353287253	-0.181003043	1.99E-10	6.40E-09
TSS200	GGTLC2	22	22988617	1	cg01285756	0.69294069	0.590919378	0.526311986	-0.166628704	1.29E-11	1.00E-09
TSS200	GHRH	20	35885301	1	cg11375072	0.655161085	0.595725111	0.494697123	-0.160463962	0.000110266	0.000568046
TSS200	GHSR	3	172166234/172166258/172166242	3	cg15987088/cg17152757/cg24599434	0.287919458	0.372009895	0.533990108	0.246070649	1.70E-09	3.44E-08
TSS200	GIF	11	59613120	1	cg16550010	0.48282656	0.370489117	0.287877491	-0.194949069	3.10E-11	1.72E-09
TSS200	GJC2	1	228337503/228337366/228337379	3	cg03065165/cg07103618/cg10950111	0.639068372	0.548993856	0.435880727	-0.203187644	2.68E-08	3.50E-07
TSS200	GJD2	15	35046765/35046760	2	cg11525643/cg21053529	0.413471005	0.549616089	0.711265257	0.297794252	1.63E-11	1.17E-09
TSS200	GLB1L3	11	134146266/134146132/134146253/134146153/134146075	5	cg03108229/cg03323292/cg05621343/cg09395318/cg21692846	0.119275277	0.193457919	0.321228146	0.201952869	0.000463443	0.002063251
TSS200	GLIS2	16	4382072/4382081/4382085/4382027	4	cg01262566/cg04719112/cg08909416/cg22616007	0.772746479	0.672128056	0.540527945	-0.232218533	4.49E-10	1.20E-08
TSS200	GLRA3	4	175750583/175750562/175750527/175750637/175750510/175750507	6	cg02521102/cg05990549/cg09841488/cg21365094/cg23697278/cg26226802	0.232201128	0.307315318	0.494499608	0.26229848	1.27E-07	1.35E-06
TSS200	GLRB	4	157997173/157997189/157997167/157997086/157997526/157997363/157997367/157997079/157997360/157997178	10	cg04006722/cg05263495/cg06436905/cg07084358/cg08712866/cg09688546/cg16269431/cg18344745/cg22065614/cg25911468	0.324132312	0.357718207	0.505912604	0.181780292	2.24E-06	1.75E-05
TSS200	GLT1D1	12	129337897/129337910	2	cg04054313/cg06419750	0.437228165	0.582049456	0.717655577	0.280427412	1.59E-12	2.76E-10
TSS200	GLT8D2	12	104444006/104443953/104443925/104444017/104444011/104444060	6	cg00987461/cg01283482/cg08101375/cg14849798/cg16721177/cg21988041	0.112445104	0.170939527	0.280732598	0.168287495	0.00054679	0.00238912
TSS200	GNE	9	36258600	1	cg21210985	0.090427894	0.144191242	0.288399809	0.197971915	0.000898887	0.003764821
TSS200	GNGT1	7	93535731	1	cg20829211	0.745195415	0.6841593	0.54698355	-0.198211865	1.38E-05	8.77E-05
TSS200	GOLGA6L6	15	20747248/20747200	2	cg15030631/cg23673919	0.65519362	0.552391811	0.481065005	-0.174128615	6.52E-13	1.88E-10

TSS200	GPC6	13	93879022/93878969/93879042/93879008	4	cg00191477/cg07529657/cg23246978/cg25772299	0.162927445	0.19970389	0.350968335	0.188040891	4.93E-05	0.000273635
TSS200	GPR110	6	47010091/47010177	2	cg06228599/cg09556286	0.45448143	0.320804539	0.243978345	-0.210503085	6.38E-12	6.17E-10
TSS200	GPR111	6	47624276/47624247	2	cg16361253/cg18184910	0.502977895	0.389388033	0.322885564	-0.180092331	5.14E-09	8.70E-08
TSS200	GPR115	6	47666248	1	cg26451345	0.51234432	0.381327294	0.29107665	-0.22126767	3.90E-11	2.01E-09
TSS200	GPR123	10	134901279/134901294/134901297/134901301/134901305/134901208	6	cg07068327/cg09639725/cg15825786/cg18029761/cg23380558/cg23633543	0.218956693	0.310640385	0.446477687	0.227520994	2.92E-05	0.000170682
TSS200	GPR139	16	20085214	1	cg20086208	0.20680717	0.263741031	0.387992806	0.181185636	0.003721845	0.013563617
TSS200	GPR15	3	98250723	1	cg19614811	0.638956855	0.549638306	0.483918055	-0.1550388	3.13E-05	0.000181617
TSS200	GPR151	5	145895766	1	cg19700872	0.38276473	0.323722572	0.218283155	-0.164481575	3.96E-08	4.92E-07
TSS200	GPR152	11	67220223/67220263/67220318	3	cg00587613/cg12145564/cg27265307	0.833516395	0.717543544	0.680730945	-0.15278545	2.83E-06	2.14E-05
TSS200	GPR26	10	125425690/125425688/125425692	3	cg04549162/cg11893763/cg26047950	0.338052003	0.407872328	0.530995215	0.192943212	3.02E-08	3.88E-07
TSS200	GPR6	6	110300233/110300218/110300101/110300146/110300099	5	cg01261007/cg01769232/cg05756933/cg12800028/cg15381304	0.391339138	0.407526868	0.563711615	0.172372477	5.17E-06	3.65E-05
TSS200	GPR75	2	54087200/54087173/54087178/54087209/54087189/54087184	6	cg00701946/cg05414908/cg08611810/cg12451530/cg13356253/cg19878627	0.592243067	0.708420354	0.827794662	0.235551595	6.99E-14	5.51E-11
TSS200	GPR83	11	94134666/94134732/94134739/94134756/94134646	5	cg05374654/cg09891129/cg22638597/cg23739994/cg27634151	0.259935851	0.342202788	0.491914985	0.231979134	1.45E-07	1.51E-06
TSS200	GPR85	7	112726659/112726653/112726547/112727914/112726538/112726549/112726541/112726396	8	cg01491795/cg03119639/cg06931815/cg09506675/cg13251269/cg20019019/cg20513548/cg24310431	0.189850148	0.254072383	0.344976102	0.155125954	0.000702846	0.003003571
TSS200	GPR87	3	151034669	1	cg09432154	0.47921643	0.334064389	0.234356582	-0.244859848	6.69E-13	1.88E-10
TSS200	GPR88	1	101003688/101003634	2	cg06223162/cg09408571	0.567033713	0.495271725	0.383171809	-0.183861903	6.48E-08	7.54E-07
TSS200	GPSM1	9	139247374	1	cg14040131	0.64215983	0.381567561	0.278549441	-0.363610389	2.58E-09	4.87E-08
TSS200	GPX7	1	53067965/53067894	2	cg11953272/cg22129364	0.146971405	0.191489454	0.313742583	0.166771178	0.000167401	0.00082647

TSS200	GREB1	2	11674057/11680057/11680020/11679879	4	cg02584498/cg03877767/cg11534680/cg14653284	0.483677323	0.381441526	0.311466058	-0.172211266	1.29E-11	1.00E-09
TSS200	GRIA2	4	158141570/158141542	2	cg00699993/cg10410142	0.399915025	0.481114561	0.637241957	0.237326932	3.95E-12	4.83E-10
TSS200	GRIA4	11	105481306/105481317/105481319/105481322/105480771/105480788/105480790/105481283/105481406/105481292/105480792	11	cg00343633/cg03225817/cg04747226/cg07972135/cg09147777/cg11308643/cg12754421/cg15603568/cg21217024/cg23559689/cg23676682	0.303668483	0.408511226	0.583959483	0.280291001	8.01E-08	9.05E-07
TSS200	GRIK1	21	31312333/31312328	2	cg07950000/cg21816539	0.293747293	0.373807092	0.490491752	0.19674446	1.14E-07	1.23E-06
TSS200	GRIK2	6	101846805/101846791/101846797/101846707/101846779/101846767	6	cg05942459/cg06247406/cg13080565/cg16009558/cg22541254/cg24753760	0.431177119	0.544488494	0.638849685	0.207672566	1.67E-08	2.32E-07
TSS200	GRIN2A	16	10276375/10276092/10275934/10276383/10276674/10276081/10276799/10276119	8	cg00287829/cg02585344/cg05406943/cg06758670/cg07642043/cg09461286/cg16368442/cg16904585	0.151818985	0.213582708	0.351725017	0.199906032	9.21E-07	7.91E-06
TSS200	GRM6	5	178422128/178422260/178422244/178422140	4	cg00582971/cg01281157/cg14859460/cg19806642	0.487445315	0.579523385	0.705797465	0.21835215	7.25E-11	3.14E-09
TSS200	GRM7	3	6902689	1	cg24213507	0.21921605	0.296931333	0.473498077	0.254282027	6.76E-06	4.65E-05
TSS200	GRP	18	56887279/56887320/56887351/56887310/56887271	5	cg03563308/cg12927990/cg15108347/cg24131595/cg26460471	0.273055189	0.364582398	0.473771942	0.200716753	7.56E-09	1.20E-07
TSS200	GRXCR1	4	42895240	1	cg10469791	0.37041938	0.2781758	0.200581791	-0.169837589	1.30E-13	7.29E-11
TSS200	GSC2	22	19137968/19137874	2	cg06695663/cg23774356	0.33885151	0.429404128	0.559446507	0.220594997	4.94E-10	1.29E-08
TSS200	GSTM2	1	110210641/110210444	2	cg02337836/cg20676696	0.188026501	0.32030378	0.457824632	0.269798132	8.29E-08	9.32E-07
TSS200	GSX1	13	28366645/28366600/28366759/28366598/28366606/28366702	6	cg03388293/cg05344430/cg10071824/cg11354629/cg14168923/cg17369527	0.279743824	0.309918543	0.464428891	0.184685067	0.000183473	0.000896833
TSS200	GUCA1C	3	108672872	1	cg19812613	0.80017399	0.653651272	0.582884532	-0.217289458	1.37E-07	1.44E-06

TSS200	GUCA2A	1	42630526/42630436/42630567	3	cg09212118/cg13452215/cg17739643	0.45055423	0.364540715	0.297038814	-0.153515416	2.17E-10	6.81E-09
TSS200	GUCY1A2	11	106889320/106889329/106889318/106889364/106889334	5	cg02989669/cg12243375/cg15847198/cg20905516/cg25918303	0.078953429	0.122064987	0.326497579	0.24754415	0.000268944	0.001264454
TSS200	GUCY1B3	4	156680007/156680050/156680029	3	cg04040095/cg08358983/cg18129755	0.442044198	0.527887006	0.656646489	0.214602291	5.94E-09	9.79E-08
TSS200	GYPC	2	127413530/127413505	2	cg00014112/cg04453971	0.29059013	0.379654733	0.489831673	0.199241543	1.76E-07	1.79E-06
TSS200	HAO1	20	7921280	1	cg19201723	0.544532915	0.42124845	0.334135936	-0.210396979	7.66E-12	7.03E-10
TSS200	HAS1	19	52227357/52227348/52227309	3	cg06785213/cg10807260/cg17657179	0.31371506	0.374843465	0.471854998	0.158139938	3.58E-05	0.000205091
TSS200	HBBP1	11	5265008	1	cg05133853	0.369525795	0.2611101	0.178725934	-0.190799861	1.23E-08	1.81E-07
TSS200	HBM	16	215845/215960/215791/215781/215864	5	cg01558909/cg06319822/cg08393070/cg09812623/cg26540367	0.162495193	0.220721911	0.344256486	0.181761293	0.000652031	0.002803127
TSS200	HECW1	7	43152125/43152021	2	cg03004999/cg13298841	0.254652345	0.296185131	0.465199643	0.210547298	4.68E-06	3.35E-05
TSS200	HES3	1	6304199/6304220/6304217	3	cg00765655/cg19743103/cg20157525	0.277954863	0.37617282	0.44550905	0.167554187	0.000390838	0.001763914
TSS200	HHLA2	3	108021214/108021293	2	cg10431989/cg22926869	0.72711398	0.648615889	0.570291477	-0.156822503	0.000483954	0.002146082
TSS200	HIC1	17	1958206/1959596/1958237	3	cg02756676/cg20664636/cg25053531	0.119704759	0.207886952	0.294555484	0.174850725	5.35E-07	4.83E-06
TSS200	HIST1H4F	6	26240505/26240579/26240519/26240528	4	cg00436476/cg05159188/cg12260798/cg22723502	0.382213723	0.497467678	0.589769416	0.207555693	3.61E-08	4.56E-07
TSS200	HKR1	19	37825388/37825446/37825406	3	cg05280698/cg12948621/cg24834889	0.416202347	0.530739537	0.568308579	0.152106232	0.002551275	0.009682565
TSS200	HMX2	10	124907600/124907540/124907553/124907457	4	cg07229938/cg17498296/cg22603247/cg23437459	0.107673106	0.17093666	0.261057942	0.153384836	0.000412741	0.001855336
TSS200	HNF1A	12	121416512/121416547/121416390/121416510/121416501	5	cg01341572/cg01394199/cg05934698/cg06444781/cg14996810	0.562732005	0.487819969	0.408070226	-0.154661779	3.82E-09	6.83E-08

TSS200	HNF4A	20	43029895/42984320/42984324/43029732/42984394/42984338/42984347/42984276/43029777	9	cg03862380/cg06126829/cg06640637/cg07006673/cg16221969/cg20848979/cg22958104/cg24084358/cg27420224	0.533742603	0.440728555	0.354330681	-0.179411922	7.82E-11	3.27E-09
TSS200	HOXA2	7	27142547/27142527/27142535/27142427/27142437	5	cg02979457/cg05921905/cg06769202/cg23979631/cg24058604	0.268984518	0.406471907	0.543246351	0.274261833	2.19E-05	0.000132812
TSS200	HOXA6	7	27187502/27187372/27187374/27187560/27187556/27187553	6	cg03529432/cg04265576/cg12810523/cg14044640/cg17994139/cg22469274	0.156703575	0.246488623	0.317372508	0.160668933	0.004413667	0.015774959
TSS200	HOXB6	17	46682398/46682390/46682394/46682413	4	cg02574073/cg10906729/cg18684142/cg19828220	0.480171541	0.362185738	0.262243568	-0.217927974	1.46E-10	5.07E-09
TSS200	HOXC10	12	54378789/54378815/54378896/54378821/54378787/54378826	6	cg03556393/cg08737755/cg08900043/cg16253259/cg21623748/cg25057269	0.160594537	0.213917573	0.311043306	0.150448769	0.000404926	0.001822283
TSS200	HOXC11	12	54366760/54366875/54366816/54366859	4	cg03132556/cg06630413/cg08962452/cg26386624	0.424190768	0.538414197	0.640461675	0.216270908	2.20E-08	2.96E-07
TSS200	HOXC13	12	54332463/54332501/54332530/54332385/54332457	5	cg01839657/cg09106624/cg10961753/cg11466369/cg26283893	0.233238621	0.298792299	0.404352779	0.171114158	4.55E-06	3.26E-05
TSS200	HOXC9	12	54393687/54393709/54393725	3	cg03215137/cg13233407/cg21272774	0.314765248	0.420804026	0.596110945	0.281345697	1.38E-09	2.88E-08
TSS200	HPYR1	8	133573809/133573739	2	cg07696099/cg24627900	0.463879428	0.333557311	0.242673395	-0.221206032	5.80E-14	5.04E-11
TSS200	HS3ST3A1	17	13505433	1	cg16861955	0.222824465	0.300127556	0.427290614	0.204466149	0.000445662	0.001988278
TSS200	HS3ST4	16	25703160/25703153	2	cg24862668/cg26933063	0.116458718	0.118546268	0.275044375	0.158585657	0.002450276	0.009332947
TSS200	HSD17B2	16	82068803/82068702	2	cg20373326/cg26479028	0.47452185	0.376815939	0.286402014	-0.188119836	1.10E-10	4.16E-09
TSS200	HSD17B3	9	99064450	1	cg14356530	0.62764841	0.593422244	0.470116273	-0.157532137	5.27E-06	3.70E-05
TSS200	HSF4	16	67197180/67197186/67197150/67197129	4	cg05436064/cg07816687/cg09030672/cg18705279	0.243967256	0.330710823	0.434190789	0.190223534	1.10E-09	2.41E-08
TSS200	HSPB3	5	53751272/53751411	2	cg17839166/cg20169062	0.615868975	0.425499872	0.292604077	-0.323264898	1.47E-14	1.93E-11

TSS200	HSPB9	17	40274670/40274740/40274728/40274703/40274722	5	cg01501896/cg07168142/cg08880849/cg16208084/cg26934993	0.499179343	0.39830171	0.31683343	-0.182345913	1.58E-08	2.22E-07
TSS200	HTA	16	73126120	1	cg00293191	0.77247929	0.635101672	0.532098973	-0.240380317	1.17E-11	9.37E-10
TSS200	HTR1A	5	63257710/63257554	2	cg02266732/cg04694812	0.37726663	0.462507822	0.601886391	0.224619761	1.78E-09	3.57E-08
TSS200	HTR1E	6	87646972/87646930/87646876/87646986/87646922	5	cg00454577/cg03472798/cg13748928/cg21286526/cg22141389	0.195128451	0.265592081	0.414021672	0.21889322	1.94E-06	1.54E-05
TSS200	HTR3C	3	183770677/183770650	2	cg19233761/cg19815720	0.678129205	0.550280939	0.400421809	-0.277707396	3.34E-12	4.48E-10
TSS200	HTR3D	3	183749212/183749414/183749227/183750429	4	cg00576550/cg04200192/cg14483391/cg17645664	0.543917035	0.470620147	0.380217845	-0.16369919	1.21E-09	2.60E-08
TSS200	HTR5A	7	154862441/154862524	2	cg00903099/cg20967585	0.40199582	0.516462503	0.665576832	0.263581012	1.12E-09	2.44E-08
TSS200	HYAL1	3	50341183/50341074/50341009/50349868	4	cg06360465/cg11607701/cg14943722/cg16240550	0.608920143	0.466573292	0.379649527	-0.229270615	2.78E-09	5.20E-08
TSS200	HYAL4	7	123485052/123485043	2	cg08158805/cg13882046	0.67382397	0.53954895	0.438621732	-0.235202238	2.19E-10	6.83E-09
TSS200	HYDIN	16	71264678/71264658/71264579	3	cg02293437/cg09794131/cg09918510	0.240958013	0.305490331	0.449993583	0.20903557	5.96E-05	0.000323826
TSS200	IFNA7	9	21202213	1	cg18403613	0.50355786	0.4263086	0.318881373	-0.184676487	8.76E-11	3.55E-09
TSS200	IGF2AS	11	2161586	1	cg25163476	0.230925235	0.272385617	0.438010264	0.207085029	1.85E-05	0.000114554
TSS200	IGFL1	19	46732864	1	cg25246031	0.476276465	0.367012422	0.294366755	-0.18190971	1.32E-14	1.93E-11
TSS200	IKZF1	7	50344242/50344331/50344294	3	cg18810347/cg20078466/cg27633530	0.072719946	0.176396283	0.335746248	0.263026302	2.68E-05	0.000158986
TSS200	IL12B	5	158757659	1	cg06111286	0.357650125	0.251628711	0.195009495	-0.16264063	7.45E-10	1.78E-08
TSS200	IL16	15	81489160/81489075	2	cg08255105/cg23936860	0.545684615	0.474092156	0.378542282	-0.167142333	2.65E-08	3.47E-07
TSS200	IL17RE	3	9944183	1	cg18738581	0.349872465	0.239464678	0.185066232	-0.164806233	4.95E-13	1.67E-10
TSS200	IL19	1	206972130/206972113	2	cg11582579/cg20998511	0.586337805	0.519033217	0.384996775	-0.20134103	3.77E-07	3.53E-06
TSS200	IL1F9	2	113735451/113735503	2	cg13298167/cg27067035	0.6037464	0.468935467	0.411721918	-0.192024482	1.08E-09	2.37E-08
TSS200	IL1RL2	2	102803290/102803407	2	cg15077070/cg22797169	0.475451963	0.433785828	0.324164011	-0.151287951	2.33E-05	0.000140179
TSS200	IL20RA	6	137366432/137366346/137366322/137366336/137366369/137366492	6	cg13714540/cg15992935/cg22487322/cg24405068/cg26864905/cg27656754	0.606338933	0.437797385	0.326750488	-0.279588445	6.27E-11	2.81E-09

TSS200	IL23R	1	67632028	1	cg07842386	0.56604364	0.651066817	0.721488827	0.155445187	1.34E-09	2.82E-08
TSS200	IMP5	17	43922210/43922240/43922244/43922122	4	cg03882071/cg08568298/cg23907852/cg24515861	0.467624366	0.385905172	0.316818241	-0.150806125	1.42E-10	4.98E-09
TSS200	INA	10	105036811/105036863/105036747/105036727	4	cg08050235/cg17567560/cg23003534/cg24680586	0.143560112	0.243447131	0.447113657	0.303553545	1.62E-06	1.31E-05
TSS200	INHBA	7	41742795	1	cg18413237	0.77036114	0.618036522	0.489446836	-0.280914304	1.73E-10	5.70E-09
TSS200	INS	11	2182540/2182457/2182573/2182618	4	cg02749887/cg20254598/cg21574853/cg25336198	0.603619085	0.525042196	0.43354588	-0.170073205	5.20E-09	8.78E-08
TSS200	INS-IGF2	11	2182540/2182457/2182573/2182618	4	cg02749887/cg20254598/cg21574853/cg25336198	0.603619085	0.525042196	0.43354588	-0.170073205	5.20E-09	8.78E-08
TSS200	INSC	11	15133828/15136456/15136475/15136345/15136380/15136376	6	cg01223673/cg02434443/cg13997645/cg22792646/cg23463269/cg23930945	0.264872593	0.326389709	0.419956715	0.155084122	0.002654428	0.010013797
TSS200	INSL5	1	67267129	1	cg02120470	0.60222788	0.478791589	0.369967527	-0.232260353	2.30E-11	1.46E-09
TSS200	IRAK3	12	66582780/66582805	2	cg20395892/cg26279550	0.330652861	0.392081044	0.569845831	0.23919297	3.30E-07	3.14E-06
TSS200	IRF4	6	391680/391743/391683/391664	4	cg03410961/cg05766140/cg16046028/cg16643840	0.082199335	0.178686749	0.337683454	0.255484119	3.31E-06	2.46E-05
TSS200	IRX4	5	1883011/1882952/1883005/1882885/1882900/1882902	6	cg02641288/cg07368937/cg07882671/cg14507560/cg23620282/cg24937747	0.397676793	0.493791531	0.598395711	0.200718919	2.17E-05	0.000131991
TSS200	IRX6	16	55358461/55358458	2	cg04853843/cg10250663	0.30543853	0.370463272	0.465869659	0.160431129	1.01E-05	6.60E-05
TSS200	ISLR2	15	74421535/74421935/74421635/74421523/74422528/74421857/74421584/74421848/74422632/74421887/74422572/74422629/74422550/74422545/74421918/74421531/74421909	17	cg00781110/cg02709205/cg04920649/cg05547902/cg05923854/cg06041677/cg07917269/cg08703540/cg10070260/cg12017911/cg18887483/cg23392501/cg24123518/cg24653100/cg25705558/cg25828462/cg27200833	0.243653318	0.282673849	0.425638663	0.181985345	2.75E-06	2.09E-05

TSS200	ITGA4	2	182321496/182321489	2	cg10965575/cg17265419	0.338721798	0.454148258	0.526380743	0.187658946	1.50E-06	1.22E-05
TSS200	ITGA8	10	15761869/15761881/15761854/15761876	4	cg07073391/cg16422098/cg19944763/cg25825488	0.261845831	0.379988305	0.523987721	0.262141891	4.16E-06	3.01E-05
TSS200	ITGB6	2	161056600/161056639	2	cg07896068/cg18437633	0.627600435	0.488388908	0.385050082	-0.242550353	1.12E-11	9.12E-10
TSS200	ITIH2	10	7745191	1	cg06440087	0.62501054	0.528964628	0.417236995	-0.207773545	1.50E-08	2.12E-07
TSS200	ITIH4	3	52864907/52864812/52864816	3	cg05721858/cg07095346/cg17890764	0.49948116	0.385902778	0.3157466	-0.18373456	3.89E-10	1.07E-08
TSS200	IYD	6	150689918	1	cg22804839	0.517396385	0.382214878	0.28541745	-0.231978935	1.46E-11	1.10E-09
TSS200	IZUMO1	19	49250252/49250180	2	cg15085603/cg16732175	0.124496334	0.157671631	0.313989755	0.189493422	1.07E-05	6.97E-05
TSS200	JAKMIP1	4	6202381/6202384/6202379	3	cg08073882/cg10748160/cg13168683	0.054793405	0.126586193	0.228282325	0.17348892	0.000834965	0.003516711
TSS200	JAM2	21	27011462/27011480/27011482/27011436	4	cg05504541/cg06716684/cg16532755/cg21042970	0.063294654	0.167859389	0.226439507	0.163144853	0.000323032	0.001489443
TSS200	JAM3	11	133938788/133938676/133938757/133938810	4	cg03637878/cg19055936/cg24140030/cg24899571	0.146781501	0.214326459	0.413833505	0.267052004	7.01E-08	8.09E-07
TSS200	KATNAL2	18	44526756/44526707/44526700/44526605/44526743	5	cg05498007/cg06198398/cg12171183/cg19558029/cg20469799	0.087198262	0.169967627	0.289067493	0.201869231	0.000244602	0.001162485
TSS200	KCNA1	12	5019017/5019004/5019066/5018984/5019024	5	cg03929977/cg10569606/cg10767336/cg12080391/cg15543616	0.181326338	0.291514145	0.499834589	0.318508251	6.31E-10	1.56E-08
TSS200	KCNA3	1	111217691/111217712/111217715/111217785	4	cg06750832/cg07808555/cg09410234/cg26071016	0.25023847	0.437710297	0.609975493	0.359737024	1.26E-12	2.41E-10
TSS200	KCNA4	11	30038685/30038675/30038615/30038672/30038677/30038619	6	cg05756220/cg08490115/cg10387551/cg15044957/cg15310492/cg17714025	0.424733574	0.488604048	0.597385488	0.172651914	1.74E-08	2.41E-07
TSS200	KCNA5	12	5153054	1	cg17653824	0.276973885	0.3924915	0.480052273	0.203078388	4.48E-08	5.48E-07
TSS200	KCNC1	11	17757415/17757342	2	cg00413617/cg19947104	0.477201608	0.544435308	0.676659805	0.199458197	2.63E-11	1.54E-09
TSS200	KCNE2	21	35736252	1	cg14127954	0.47691791	0.378690722	0.305793614	-0.171124296	8.56E-11	3.50E-09

TSS200	KCNIP1	5	169930938/169930870/169780747/169930919/169930879/169930905/169780772	7	cg04248157/cg08360721/cg10608615/cg11782635/cg15124711/cg15713707/cg22464105	0.411386769	0.46655677	0.567726992	0.156340223	4.95E-06	3.51E-05
TSS200	KCNJ1	11	128712399/128737467/128737463/128737429/128737415/128737319/128712462/128712417/128737300	9	cg07713705/cg10280034/cg12408998/cg13267718/cg13675753/cg14481339/cg20962532/cg25194720/cg27056194	0.762149996	0.710318675	0.608141639	-0.154008358	9.57E-07	8.20E-06
TSS200	KCNK12	2	47797488/47797569/47797590	3	cg03603951/cg04431946/cg08074851	0.245250457	0.372603034	0.554110771	0.308860313	1.09E-10	4.14E-09
TSS200	KCNK16	6	39290478/39290342/39290526/39290415	4	cg05897803/cg06543101/cg07970874/cg18754335	0.655187386	0.547124025	0.41851878	-0.236668607	8.05E-12	7.13E-10
TSS200	KCNMB1	5	169816792	1	cg22646937	0.469126495	0.390604461	0.312520409	-0.156606086	4.58E-11	2.23E-09
TSS200	KCNQ5	6	73331405	1	cg00852573	0.236188001	0.456947117	0.638643491	0.40245549	4.26E-12	5.02E-10
TSS200	KDR	4	55991860/55991782/55991850/55991943/55991824/55991818	6	cg00989765/cg07893544/cg11505338/cg14323109/cg18177533/cg21161891	0.298562403	0.408836202	0.557840105	0.259277702	8.90E-07	7.66E-06
TSS200	KHDC1L	6	73935200/73935270	2	cg19565171/cg24543552	0.6973452	0.536029308	0.394430645	-0.302914555	3.99E-12	4.85E-10
TSS200	KHDRBS2	6	62996132/62996299/62996183/62996214/62996130/62996119	6	cg03656899/cg11315153/cg18134312/cg18884037/cg22014661/cg26715952	0.292970528	0.420608942	0.557564465	0.264593937	8.12E-08	9.16E-07
TSS200	KIAA1217	10	23983538/23983534/24497713/23983498/23983496	5	cg08539841/cg08704681/cg11502597/cg13882278/cg24487076	0.146130786	0.19490086	0.31299218	0.166861394	5.58E-07	5.03E-06
TSS200	KIAA1644	22	44708843/44708761/44708861/44708794/44708908	5	cg02687592/cg19189176/cg22866759/cg23509574/cg25466154	0.79957062	0.721569567	0.648987805	-0.150582815	4.28E-09	7.52E-08
TSS200	KIAA1683	19	18385502/18385409	2	cg01087239/cg24782497	0.408123373	0.307092547	0.255588084	-0.152535288	1.24E-09	2.66E-08
TSS200	KLB	4	39408418/39408416	2	cg22953731/cg25064910	0.59930657	0.485325792	0.380033184	-0.219273386	1.03E-10	3.98E-09

TSS200	KLF17	1	44584446/44584350/44584429/44584518/44584417	5	cg04877567/cg08679985/cg16903122/cg20243285/cg21864563	0.94874659	0.825052944	0.675926584	-0.272820006	4.49E-07	4.12E-06
TSS200	KRT1	12	53074328/53074313/53074257	3	cg10538533/cg10926148/cg17405586	0.546406978	0.480530731	0.382627048	-0.16377993	4.71E-09	8.13E-08
TSS200	KRT16	17	39769175/39769213	2	cg13179549/cg26336935	0.5766012	0.468799861	0.385048595	-0.191552605	4.52E-09	7.93E-08
TSS200	KRT20	17	39041602	1	cg00091693	0.654840195	0.478480711	0.349085609	-0.305754586	2.09E-11	1.37E-09
TSS200	KRT24	17	38860042/38860097	2	cg04219544/cg23737768	0.50982608	0.392596294	0.299409282	-0.210416798	1.13E-11	9.19E-10
TSS200	KRT28	17	38956362	1	cg07008343	0.659471165	0.557694322	0.428230009	-0.231241156	2.33E-06	1.81E-05
TSS200	KRT31	17	39553960	1	cg11473240	0.526746535	0.409767844	0.335885514	-0.190861021	5.84E-07	5.24E-06
TSS200	KRT37	17	39580880	1	cg15059743	0.565611215	0.451885111	0.385385923	-0.180225292	6.82E-09	1.10E-07
TSS200	KRT6A	12	52887263	1	cg03462053	0.803110855	0.653543944	0.517739759	-0.285371096	4.62E-09	8.03E-08
TSS200	KRT6B	12	52845930/52846073/52845938/52846088	4	cg02068361/cg11601375/cg12795208/cg13221107	0.60088502	0.399430506	0.348907464	-0.251977556	1.19E-08	1.75E-07
TSS200	KRT7	12	52626831/52626814/52626818/52626889/52626904	5	cg07007400/cg07967679/cg08975803/cg14482313/cg14537533	0.505103519	0.599367097	0.707058041	0.201954522	8.84E-10	2.04E-08
TSS200	KRT71	12	52947004	1	cg05358758	0.601289135	0.413676656	0.312331736	-0.288957399	9.54E-10	2.17E-08
TSS200	KRT8	12	53298911/53298888	2	cg03327403/cg21340733	0.480000408	0.384279567	0.303229127	-0.17677128	1.10E-09	2.42E-08
TSS200	KRT80	12	52585844/52585786/52585849/52585892	4	cg00280812/cg04472592/cg20225745/cg24174145	0.425115458	0.329014435	0.259179361	-0.165936098	7.59E-11	3.23E-09
TSS200	KRTAP1-1	17	39197721	1	cg02022375	0.650459885	0.513534644	0.430641918	-0.219817967	1.72E-09	3.46E-08
TSS200	KRTAP11-1	21	32253964	1	cg22643217	0.67525976	0.623237167	0.507881595	-0.167378165	1.16E-05	7.53E-05
TSS200	KRTAP12-1	21	46102242	1	cg06683078	0.395013455	0.304620194	0.231999136	-0.163014319	7.09E-10	1.71E-08
TSS200	KRTAP12-2	21	46087026/46086855	2	cg08152198/cg22347126	0.43836179	0.344599533	0.260349341	-0.178012449	1.11E-10	4.21E-09
TSS200	KRTAP12-4	21	46074616	1	cg07018760	0.61169551	0.5075375	0.410091464	-0.201604046	6.53E-07	5.78E-06
TSS200	KRTAP13-1	21	31768311/31768319	2	cg02764897/cg26740230	0.502679203	0.405289803	0.283117902	-0.2195613	4.79E-13	1.67E-10
TSS200	KRTAP13-3	21	31798236	1	cg06468780	0.74166367	0.704265667	0.581097995	-0.160565675	1.62E-05	0.00010147
TSS200	KRTAP19-1	21	31852793	1	cg13139843	0.48232829	0.364093794	0.285575118	-0.196753172	1.13E-12	2.34E-10
TSS200	KRTAP19-5	21	31874502	1	cg14054990	0.53858839	0.410367172	0.260509864	-0.278078526	3.49E-15	1.23E-11
TSS200	KRTAP25-1	21	31661998	1	cg24145481	0.55644044	0.397667394	0.302504641	-0.253935799	1.12E-15	8.86E-12
TSS200	KRTAP3-1	17	39165425	1	cg24833464	0.693517695	0.625774083	0.5305573	-0.162960395	2.54E-07	2.49E-06

TSS200	KRTAP4-7	17	39240343	1	cg16090541	0.595406955	0.489856633	0.416410586	-0.178996369	2.11E-09	4.10E-08
TSS200	KRTAP4-8	17	39254427/39254439	2	cg12981595/cg20663846	0.55585559	0.403320633	0.297085225	-0.258770365	1.76E-12	2.96E-10
TSS200	KRTAP9-4	17	39405817	1	cg00550617	0.43807172	0.345016444	0.280069459	-0.158002261	5.49E-12	5.68E-10
TSS200	KRTDAP	19	35981371/35981517/35981531/35981549	4	cg00083616/cg01585096/cg22802461/cg25717032	0.685287755	0.603508197	0.522446325	-0.16284143	4.88E-07	4.44E-06
TSS200	KY	3	134369974/134369919/134369987/134370015/134369939/134370046	6	cg00060320/cg00329270/cg12307306/cg12573071/cg22889573/cg24370755	0.191735639	0.235910166	0.342388241	0.150652603	0.000778042	0.003297219
TSS200	L1TD1	1	62660421/62660281	2	cg14254748/cg27300647	0.605336813	0.443143486	0.360132848	-0.245203965	2.95E-12	4.35E-10
TSS200	LACRT	12	55028756	1	cg26218982	0.773553815	0.658598289	0.547215936	-0.226337879	3.39E-08	4.30E-07
TSS200	LAMB3	1	209825856/209825747/209825943/209825849/209824825/209825678/209825731	7	cg01580568/cg03977657/cg04873061/cg19709585/cg20416769/cg22502856/cg26533764	0.588906464	0.475318851	0.39392389	-0.194982574	1.06E-10	4.07E-09
TSS200	LAYN	11	111411071/111411199/111411176/111411216/111411076	5	cg04226536/cg06459000/cg12506005/cg19820372/cg21703322	0.162957659	0.248335517	0.33857091	0.175613251	8.20E-05	0.000432874
TSS200	LCE1F	1	152748838	1	cg01915081	0.657349355	0.581917528	0.429483991	-0.227865364	6.97E-10	1.68E-08
TSS200	LCE2A	1	152670764	1	cg13705774	0.726300065	0.651140572	0.528898373	-0.197401692	1.14E-07	1.23E-06
TSS200	LCN2	9	130911577	1	cg14615559	0.486324855	0.365967717	0.272160432	-0.214164423	6.60E-11	2.90E-09
TSS200	LDHD	16	75150819/75150833/75150799/75150784/75150744	5	cg03085549/cg03743982/cg09899215/cg16540789/cg24429836	0.442058635	0.348694067	0.274865191	-0.167193444	1.75E-08	2.41E-07
TSS200	LEAP2	5	132209201	1	cg11397957	0.543176875	0.42993275	0.329382123	-0.213794752	2.20E-11	1.42E-09
TSS200	LHCGR	2	48982957/48982950	2	cg12351433/cg19403014	0.385139748	0.376765842	0.550063805	0.164924057	6.61E-05	0.000354875
TSS200	LHX1	17	35294472/35294476/35294481/35294491	4	cg04993975/cg05527869/cg10043865/cg10356613	0.139836631	0.200465452	0.3048517	0.16501507	0.012437651	0.039446767
TSS200	LHX5	12	113909934/113909993/113910012/113909932/113909948/113910034	6	cg02328332/cg06198190/cg08745498/cg09432902/cg14868703/cg23497704	0.228372586	0.284207649	0.37891423	0.150541644	0.000200821	0.000973187
TSS200	LHX8	1	75594016/75594053/75594066	3	cg01185626/cg09222749/cg26688527	0.288953108	0.396863354	0.514879792	0.225926684	2.14E-07	2.13E-06

TSS200	LIN28	1	26737116/26737247/26737262/26737199	4	cg07095205/cg14011486/cg18634211/cg27391649	0.23520769	0.310829081	0.419153482	0.183945792	5.18E-05	0.000285827
TSS200	LIPH	3	185270558	1	cg23620049	0.449205525	0.33774755	0.259690332	-0.189515193	1.60E-10	5.42E-09
TSS200	LOC100128288	17	8263941	1	cg23574443	0.85852091	0.743088261	0.631132682	-0.227388228	3.54E-07	3.35E-06
TSS200	LOC100130933	17	73642628/73642503/73642547/73642607/73642486	5	cg13420383/cg14691671/cg15335334/cg23702727/cg24869815	0.405777271	0.313562163	0.251435492	-0.154341779	9.37E-12	7.90E-10
TSS200	LOC100131726	8	124215033/124215152/124215129	3	cg00831735/cg00944227/cg08256536	0.546131143	0.439160239	0.394253152	-0.151877992	8.58E-11	3.50E-09
TSS200	LOC100132111	1	151810904/151810893/151810899/151810887	4	cg04296699/cg05021743/cg10781408/cg15015892	0.176477322	0.284262843	0.40436531	0.227887988	1.47E-09	3.05E-08
TSS200	LOC100133545	11	2011341/2011312/2011282/2011216/2011346/2011238	6	cg04859117/cg07397493/cg08273750/cg09248429/cg18716210/cg27196158	0.537713545	0.451979667	0.386905146	-0.150808399	1.20E-10	4.39E-09
TSS200	LOC100133991	17	43339512/43339497/43339515/43339358/43325167/43339476	6	cg01627847/cg03048083/cg08124910/cg08247376/cg10842515/cg17222164	0.089378461	0.190352702	0.29351037	0.204131909	0.00015904	0.00079014
TSS200	LOC100188949	13	30948114/30948103/30948122	3	cg06524165/cg08859309/cg12286415	0.51701402	0.62381902	0.709096855	0.192082835	3.55E-11	1.89E-09
TSS200	LOC100190940	12	130526916/130527038/130526930/130527046/130527030	5	cg04745161/cg13576883/cg13822303/cg15584445/cg24642065	0.332160548	0.403325354	0.52784197	0.195681421	4.72E-07	4.31E-06
TSS200	LOC100192379	4	122685570/122685651/122685675/122685641	4	cg10620484/cg13640145/cg16790291/cg18149485	0.451123006	0.526547567	0.640531033	0.189408027	7.76E-07	6.75E-06
TSS200	LOC100286948	5	156351055/156351090/156351010	3	cg05482306/cg07755752/cg12932211	0.71944209	0.623704798	0.533731644	-0.185710446	7.28E-08	8.35E-07
TSS200	LOC100302652	2	54087200/54087173/54087178/54087209/54087189/54087343/54087184	7	cg00701946/cg05414908/cg08611810/cg12451530/cg13356253/cg19664945/cg19878627	0.572473798	0.691265792	0.817638091	0.245164293	9.62E-14	6.22E-11

TSS200	LOC134466	5	150326174/150326312/150326193/150326213/150326190	5	cg05016408/cg07499553/cg19585597/cg26212163/cg27079680	0.464399059	0.548733801	0.625114989	0.16071593	5.18E-09	8.76E-08
TSS200	LOC145837	15	69854024	1	cg02711212	0.5383661	0.43331305	0.355184927	-0.183181173	3.93E-11	2.01E-09
TSS200	LOC148145	19	29455913/29455873	2	cg09364980/cg10578996	0.5570755	0.424992344	0.3306826	-0.2263929	1.74E-10	5.70E-09
TSS200	LOC148824	1	247694271/247694275	2	cg12754571/cg12758973	0.671489918	0.750275328	0.837403355	0.165913437	4.80E-09	8.27E-08
TSS200	LOC151174	2	239140340/239140318/239140369/239140485/239140473	5	cg04166618/cg06732439/cg12608247/cg24079051/cg24534135	0.386820893	0.431539149	0.574672066	0.187851173	5.56E-08	6.59E-07
TSS200	LOC157627	8	9760957/9760869/9760880/9760855/9760877	5	cg00571033/cg10866298/cg12877251/cg16424920/cg21535000	0.277610124	0.377193257	0.554742041	0.277131917	8.71E-08	9.74E-07
TSS200	LOC158381	9	35406590	1	cg27416261	0.535522295	0.6236495	0.706005795	0.1704835	3.14E-09	5.78E-08
TSS200	LOC169834	9	115774505	1	cg08476595	0.41280071	0.487354261	0.577460477	0.164659767	0.0005009	0.002215003
TSS200	LOC200726	2	207506975/207506991/207507001/207507096	4	cg08057136/cg12110911/cg17165841/cg21541833	0.328318215	0.440644479	0.59412389	0.265805675	1.67E-09	3.39E-08
TSS200	LOC283392	12	72667386/72667326/72667398	3	cg00580978/cg09972192/cg13099890	0.354012765	0.410007472	0.539793214	0.185780449	5.71E-07	5.13E-06
TSS200	LOC283731	15	74421635/74421739	2	cg04920649/cg07216112	0.260177435	0.329811139	0.443036491	0.182859056	5.85E-05	0.000318986
TSS200	LOC284441	19	20368371	1	cg22155405	0.608043385	0.476266761	0.347606018	-0.260437367	4.59E-12	5.20E-10
TSS200	LOC284688	1	170253405	1	cg25100722	0.757265525	0.583277372	0.524935673	-0.232329852	1.65E-07	1.70E-06
TSS200	LOC285501	4	178649743/178649783	2	cg05769106/cg09364582	0.735367258	0.591805431	0.451730898	-0.28363636	4.75E-12	5.24E-10
TSS200	LOC285627	5	158893318/158893353/158893397	3	cg06042124/cg18015622/cg18079534	0.860837883	0.783446644	0.65057715	-0.210260733	4.87E-09	8.35E-08
TSS200	LOC285629	5	160365753	1	cg04390328	0.544169265	0.390341617	0.310438068	-0.233731197	1.29E-10	4.60E-09
TSS200	LOC285780	6	6623143/6623243	2	cg06961205/cg23123545	0.58611883	0.453344733	0.342556241	-0.243562589	1.46E-12	2.62E-10
TSS200	LOC285796	6	163745629	1	cg24288715	0.51485234	0.431478417	0.3509485	-0.16390384	1.05E-09	2.33E-08
TSS200	LOC285954	7	41733356/41733448	2	cg00285343/cg07650391	0.53933112	0.422982725	0.332149609	-0.207181511	1.70E-09	3.44E-08
TSS200	LOC340357	8	12669060/12668973	2	cg02249228/cg06066180	0.608897505	0.536558667	0.423890941	-0.185006564	5.12E-05	0.000282914
TSS200	LOC392196	8	11986876/11986933/11986864/11986813	4	cg13475862/cg15581360/cg21157301/cg26167010	0.814727778	0.71277585	0.64816752	-0.166560257	1.77E-09	3.55E-08

TSS200	LOC400940	2	6122107/6121915/6121939/6121909	4	cg13217590/cg14686555/cg22202692/cg22314787	0.235264575	0.294333858	0.403572459	0.168307884	1.76E-07	1.79E-06
TSS200	LOC401463	8	65489939/65489906/65489863/65489937/65489881	5	cg00023185/cg12874181/cg14588078/cg20702056/cg22585595	0.183547905	0.262283186	0.383378378	0.199830473	2.30E-05	0.000138384
TSS200	LOC440335	16	4845285/4845348	2	cg07773582/cg08271443	0.519411825	0.403470475	0.317811925	-0.2015999	1.03E-10	3.98E-09
TSS200	LOC440461	17	66194621/66194706/66194695/66194721/66194797	5	cg01797488/cg05247809/cg20689647/cg22083325/cg24011405	0.305786418	0.375654606	0.463101671	0.157315253	3.15E-06	2.36E-05
TSS200	LOC441666	10	42863594/42863508/42863539/42863550	4	cg01485177/cg24527560/cg24598948/cg24714864	0.314473069	0.432830554	0.551810431	0.237337362	5.11E-08	6.10E-07
TSS200	LOC441897	1	111932556	1	cg24550933	0.658127425	0.608548	0.503346895	-0.15478053	3.55E-05	0.000203487
TSS200	LOC643387	2	239140318/239140169/239140190/239140296/239140182	5	cg06732439/cg07323141/cg13530938/cg21272465/cg23497383	0.443331268	0.496596531	0.651977202	0.208645934	8.95E-08	9.98E-07
TSS200	LOC643719	19	35068788/35068710/35068628	3	cg02921624/cg04714041/cg07599133	0.294482413	0.365617248	0.507746775	0.213264363	4.51E-08	5.51E-07
TSS200	LOC643923	11	107462437/107462445/107462430	3	cg02989521/cg19266910/cg21068911	0.056736119	0.134151078	0.290224436	0.233488316	6.47E-05	0.000348079
TSS200	LOC644649	16	59789219	1	cg13027458	0.406717145	0.300971356	0.238677409	-0.168039736	7.52E-14	5.65E-11
TSS200	LOC644936	5	79596319	1	cg09387179	0.761945425	0.676012	0.603630391	-0.158315034	1.70E-07	1.74E-06
TSS200	LOC646214	15	21940895	1	cg20344956	0.553579835	0.482734394	0.369699491	-0.183880344	3.62E-08	4.57E-07
TSS200	LOC653486	11	193044/193021/192996/192897/193062/193016	6	cg02378673/cg03960562/cg17345964/cg18793661/cg20297976/cg22280333	0.602697462	0.490036682	0.398992062	-0.2037054	1.21E-12	2.38E-10
TSS200	LOC653566	1	28423139	1	cg07844563	0.80585265	0.711424778	0.584357341	-0.221495309	5.73E-09	9.52E-08
TSS200	LOC653786	16	22556947	1	cg15592863	0.74911871	0.659778694	0.580490086	-0.168628624	6.04E-05	0.000327854
TSS200	LOC728606	18	24283669/24283684/24283696	3	cg07108927/cg13758913/cg24667758	0.539963518	0.440310294	0.385225429	-0.15473809	2.17E-08	2.94E-07
TSS200	LOC729668	10	30663455/30663426/30663547	3	cg12819931/cg16018729/cg23746239	0.77425692	0.710564272	0.577785636	-0.196471284	1.04E-08	1.57E-07
TSS200	LOC84931	2	121223947/121224009/121223964	3	cg11455444/cg18434354/cg23689428	0.484106305	0.347058294	0.271185868	-0.212920437	4.26E-12	5.02E-10

TSS200	LOC90246	3	128226649/128226499/128226565	3	cg05272921/cg06620972/cg18453551	0.55796157	0.458854219	0.364326683	-0.193634887	2.49E-12	3.89E-10
TSS200	LOC92973	9	35860158	1	cg22924796	0.658685235	0.606817306	0.508653782	-0.150031453	0.000166485	0.000822737
TSS200	LONRF2	2	100939284	1	cg08017756	0.25777184	0.416011089	0.529280941	0.271509101	9.26E-07	7.94E-06
TSS200	LPA	6	161087439/161087573/161087506	3	cg11975608/cg16960593/cg17189167	0.510142453	0.413812769	0.359483107	-0.150659346	4.65E-09	8.07E-08
TSS200	LPIN2	18	3012017/3011980/3011965	3	cg10904070/cg12052497/cg16857548	0.653245712	0.5580302	0.486006368	-0.167239343	6.45E-07	5.72E-06
TSS200	LPPR4	1	99729804/99729807/99729757/99729790/99729769/99729780	6	cg03580297/cg03646889/cg05596294/cg06060191/cg18947801/cg26164310	0.251086583	0.325342127	0.476043734	0.224957151	1.20E-06	1.01E-05
TSS200	LPPR5	1	99470550/99470560/99470618	3	cg03120289/cg10159215/cg19365673	0.311524188	0.365179886	0.470671907	0.159147719	9.12E-08	1.01E-06
TSS200	LRFN5	14	42076759/42076710/42076757	3	cg03566719/cg13526007/cg14021345	0.34677136	0.464009417	0.635554882	0.288783522	1.32E-09	2.79E-08
TSS200	LRRC3B	3	26664181/26664115/26664285	3	cg05583398/cg13787438/cg24216220	0.259333422	0.366976277	0.51808965	0.258756228	6.93E-10	1.68E-08
TSS200	LRRC4B	19	51071307/51071328/51071316/51071309	4	cg07522981/cg14110073/cg14684296/cg22231329	0.235906646	0.283215518	0.412806969	0.176900323	0.002095886	0.008106458
TSS200	LRRC67	8	67940929/67940913/67940823/67940950	4	cg03024587/cg03780545/cg15744359/cg21189727	0.108376956	0.129075504	0.283722116	0.17534516	0.000944857	0.003932291
TSS200	LRRN4	20	6034694/6034888	2	cg20918682/cg26175343	0.464005903	0.399916233	0.29600672	-0.167999182	2.50E-11	1.54E-09
TSS200	LST1	6	31554848/31554946/31554829	3	cg14324675/cg19271190/cg27616007	0.535650105	0.473558652	0.384672186	-0.150977919	1.04E-07	1.14E-06
TSS200	LTBP1	2	33359529/33359550	2	cg03906115/cg08357990	0.345918218	0.230651672	0.17554961	-0.170368608	1.01E-10	3.93E-09
TSS200	LTF	3	46506554/46506483/46506481/46506404/46506552/46506519	6	cg00716083/cg03933131/cg12295918/cg17527798/cg26307117/cg27314002	0.433721122	0.504568967	0.600405533	0.166684412	2.41E-09	4.60E-08
TSS200	LUZP2	11	24518448/24518367/24518414/24518374/24518381/24518379	6	cg01557027/cg08068240/cg13083436/cg13922669/cg25469414/cg26242583	0.263513052	0.291130256	0.434018725	0.170505673	0.00029372	0.001368128
TSS200	LVRN	5	115297989/115298079	2	cg03890037/cg16620526	0.4494843	0.504920733	0.654695045	0.205210745	7.87E-12	7.10E-10

TSS200	LY6D	8	143868062/143868013/143868023/143868049/143868136/143868110/143868158	7	cg04811942/cg07572435/cg08137669/cg08209691/cg09964873/cg10140583/cg14585892	0.581887103	0.505047627	0.431736409	-0.150150694	4.20E-11	2.13E-09
TSS200	LY6H	8	144242106/144241809	2	cg24971209/cg26663686	0.172028078	0.212918355	0.34102369	0.168995612	0.000562317	0.002448818
TSS200	MADCAM1	19	496425/496419/496428/496296	4	cg08403345/cg13777292/cg14985989/cg26522278	0.319433613	0.403379662	0.535114655	0.215681042	3.57E-08	4.51E-07
TSS200	MAP3K13	3	185080905/185080816/185080951	3	cg11743827/cg19418951/cg21574349	0.369315628	0.489911959	0.65445397	0.285138341	2.43E-11	1.51E-09
TSS200	MAP4K1	19	39108723/39108672	2	cg04292549/cg24337786	0.46620693	0.594419856	0.66790725	0.20170032	1.88E-07	1.90E-06
TSS200	MBOAT4	8	30002223	1	cg21058822	0.39984712	0.298299461	0.223907241	-0.175939879	1.28E-10	4.60E-09
TSS200	MCHR2	6	100442105/100442118/100442151/100442120	4	cg06610484/cg09393254/cg22473637/cg26466587	0.492219206	0.588150617	0.743534964	0.251315757	2.16E-11	1.40E-09
TSS200	MDFI	6	41605994	1	cg08625380	0.113236383	0.194036997	0.356328224	0.243091842	4.39E-05	0.000246463
TSS200	MDS2	1	23953778	1	cg18418928	0.43154022	0.324073006	0.259054545	-0.172485675	5.65E-10	1.44E-08
TSS200	MEI1	22	42095368/42095430/42095347/42095441/42095443	5	cg10296410/cg10384919/cg20522370/cg22476848/cg22804236	0.362991954	0.454106674	0.551851857	0.188859903	2.83E-06	2.15E-05
TSS200	MEP1A	6	46761071/46761090/46761035	3	cg02449202/cg16019620/cg20980592	0.554129822	0.444787735	0.35605627	-0.198073552	4.86E-11	2.35E-09
TSS200	MEP1B	18	29769853	1	cg12602653	0.64568932	0.529973822	0.415921186	-0.229768134	1.01E-10	3.94E-09
TSS200	MEST	7	130126024/130131146/130131136/130131826/130131916/130131931/130130995/130126018/130131887/130131829/130125932/130131905/130131085/130131869/130131923/130131138/130131921/130131797/130131885	19	cg01793704/cg05369791/cg06100421/cg06212135/cg07315018/cg07870293/cg09003373/cg09059945/cg09080913/cg10249538/cg11865119/cg13104298/cg14088957/cg16823958/cg20050761/cg20297423/cg21629528/cg22705386/cg27338480	0.526886242	0.592524174	0.678105422	0.15121918	6.95E-13	1.88E-10

TSS200	MESTIT1	7	130131146/130131136/130131189/130131085/130131138	5	cg05369791/cg06100421/cg08734637/cg14088957/cg20297423	0.562154725	0.650380607	0.767327435	0.20517271	4.31E-13	1.62E-10
TSS200	MGAM	7	141695533/141695623	2	cg10681483/cg26125550	0.521825835	0.446723831	0.364010068	-0.157815767	4.20E-07	3.88E-06
TSS200	MGAT3	22	39883192/39883190	2	cg00101350/cg00550928	0.790418525	0.660400725	0.610290702	-0.180127823	1.99E-08	2.71E-07
TSS200	MGAT5B	17	74868670/74868561/74868604/74868725	4	cg05514299/cg16671079/cg23838005/cg27149973	0.398209123	0.304454233	0.208404534	-0.189804589	1.27E-12	2.41E-10
TSS200	MGC23270	14	105287408/105287427	2	cg08111922/cg14152268	0.517514685	0.442565211	0.345114364	-0.172400321	3.49E-09	6.36E-08
TSS200	MGC26647	7	88425077/88425085/88425139/88425122/88425148	5	cg00729133/cg05079191/cg13354414/cg15325154/cg22946147	0.80310907	0.694699378	0.599071029	-0.204038041	2.64E-07	2.58E-06
TSS200	MGC2889	3	192959372	1	cg13810562	0.25417425	0.296998489	0.503987091	0.249812841	1.01E-05	6.64E-05
TSS200	MIA2	14	39702965	1	cg23292259	0.656321615	0.552622678	0.491461964	-0.164859651	1.60E-08	2.23E-07
TSS200	MIR10A	17	46657504/46657346	2	cg15649236/cg26916621	0.493043483	0.389800267	0.310432995	-0.182610487	6.89E-11	3.01E-09
TSS200	MIR1204	8	128808063/128808017	2	cg11201447/cg25247520	0.436898588	0.262711901	0.179305958	-0.257592629	1.07E-10	4.10E-09
TSS200	MIR124-1	8	9761141/9761171	2	cg14278808/cg15248835	0.17467567	0.290067164	0.54393962	0.36926395	2.38E-07	2.35E-06
TSS200	MIR124-2	8	65291703/65291523/65291562/65291682/65291513/65291542	6	cg05455720/cg05474726/cg16189671/cg20653075/cg25900085/cg27313642	0.345955406	0.466054145	0.593240028	0.247284622	1.11E-09	2.42E-08
TSS200	MIR124-3	20	61809830/61809827/61809841/61809834/61809724/61809716	6	cg01052879/cg03387135/cg15028514/cg18627360/cg19267861/cg20277905	0.280579503	0.414029426	0.563995831	0.283416329	1.45E-07	1.52E-06
TSS200	MIR1260	14	77732494/77732468	2	cg10134156/cg25952096	0.792346298	0.717037181	0.613726984	-0.178619313	1.04E-07	1.13E-06
TSS200	MIR1272	15	65054892/65054827	2	cg10537328/cg12002820	0.682885625	0.609756883	0.498837091	-0.184048534	6.86E-07	6.05E-06
TSS200	MIR1283-1	19	54191610/54191706	2	cg09318158/cg26377653	0.682152285	0.5586917	0.470267086	-0.211885199	2.76E-10	8.14E-09
TSS200	MIR129-1	7	127847835	1	cg04524088	0.4984081	0.400670611	0.307622123	-0.190785977	2.01E-07	2.01E-06
TSS200	MIR129-2	11	43602879/43602920/43602847/43602857/43602845/43602914	6	cg01939477/cg05376374/cg14416371/cg14944647/cg15556502/cg16407471	0.279857002	0.39352092	0.603149488	0.323292486	8.90E-09	1.38E-07
TSS200	MIR134	14	101520989	1	cg13753460	0.55769707	0.460452167	0.394394582	-0.163302488	9.93E-11	3.89E-09

TSS200	MIR137	1	98511908/98511792/98511789	3	cg05423529/cg14783814/cg22333214	0.279652767	0.369001891	0.565508567	0.2858558	1.18E-08	1.73E-07
TSS200	MIR138-1	3	44155650	1	cg03918530	0.635006725	0.549483161	0.440437595	-0.19456913	2.41E-08	3.21E-07
TSS200	MIR141	12	7073247/7073242/7073240/7073179	4	cg12161331/cg18185189/cg19794481/cg23067082	0.569519493	0.46804974	0.389014711	-0.180504781	5.35E-11	2.53E-09
TSS200	MIR1910	16	85775359	1	cg25575961	0.659314945	0.503709539	0.3686335	-0.290681445	1.83E-11	1.24E-09
TSS200	MIR192	11	64658765/64658819/64658903/64658726	4	cg09349409/cg18262830/cg24803202/cg27083891	0.569253283	0.46756105	0.384682885	-0.184570398	1.64E-11	1.17E-09
TSS200	MIR194-2	11	64659065/64659060/64658940/64658936/64658946/64659044	6	cg00376448/cg00400165/cg00589493/cg08432452/cg13092487/cg24154336	0.575365246	0.480658808	0.403189081	-0.172176165	2.83E-11	1.63E-09
TSS200	MIR196A1	17	46710001	1	cg02329038	0.428088995	0.301733672	0.236522977	-0.191566018	4.08E-09	7.22E-08
TSS200	MIR1974	5	93905358/93905427/93905343/93905257/93905270	5	cg00964137/cg03964851/cg16695999/cg25368943/cg26338386	0.72605759	0.637869939	0.550460209	-0.175597381	6.37E-10	1.57E-08
TSS200	MIR200C	12	7072696/7072765/7072727	3	cg00366413/cg22413603/cg27534624	0.505175742	0.396195065	0.309023638	-0.196152104	2.54E-11	1.54E-09
TSS200	MIR205	1	209605401/209605304	2	cg03794445/cg17848546	0.68037358	0.599237278	0.511087373	-0.169286207	5.64E-11	2.62E-09
TSS200	MIR216A	2	56216320/56216234/56216195	3	cg16403360/cg24343835/cg25249448	0.629184945	0.557722164	0.46861227	-0.160572675	8.04E-07	6.98E-06
TSS200	MIR2276	13	24736455/24736518	2	cg00861695/cg07152812	0.63067521	0.506310128	0.377013732	-0.253661478	9.65E-13	2.12E-10
TSS200	MIR298	20	57393380	1	cg21983491	0.625575105	0.533881883	0.398177314	-0.227397791	3.46E-09	6.32E-08
TSS200	MIR300	14	101507570/101507590	2	cg02174748/cg10137253	0.582182385	0.511466611	0.403604759	-0.178577626	3.25E-08	4.15E-07
TSS200	MIR30E	1	41220011	1	cg14092259	0.834400415	0.701360878	0.623178191	-0.211222224	9.56E-06	6.31E-05
TSS200	MIR331	12	95702136/95702071	2	cg09995736/cg11690824	0.766605178	0.617743514	0.552339775	-0.214265403	2.46E-10	7.42E-09
TSS200	MIR338	17	79099785/79099882/79099777/79099819/79099942	5	cg06807993/cg06869212/cg18637486/cg21473782/cg23295826	0.772468942	0.59259095	0.577218555	-0.195250387	9.90E-06	6.51E-05
TSS200	MIR346	10	88024553/88024557/88024598/88024572/88024673/88024569	6	cg14368220/cg15282281/cg16570507/cg16692757/cg23322812/cg25972714	0.592430308	0.480534302	0.384381735	-0.208048573	1.53E-10	5.26E-09

TSS200	MIR34B	11	111383603/111383576/111383515/111383624/111383653	5	cg13767940/cg21881253/cg22879515/cg23211240/cg26561785	0.161390003	0.272821181	0.522451961	0.361061958	1.91E-06	1.52E-05
TSS200	MIR365-1	16	14403022/14403040/14403004/14403138	4	cg00928596/cg02533339/cg06916001/cg22388260	0.499707091	0.613790888	0.706811303	0.207104212	5.03E-10	1.31E-08
TSS200	MIR376A2	14	101506323/101506379/101506246	3	cg12881150/cg14144314/cg19757573	0.742856413	0.636958117	0.575756877	-0.167099536	4.72E-10	1.25E-08
TSS200	MIR383	8	14711027	1	cg19004110	0.615369055	0.479261789	0.416784764	-0.198584291	3.75E-08	4.71E-07
TSS200	MIR409	14	101531617/101531510/101531593	3	cg14285142/cg20547131/cg23270924	0.665522942	0.553785044	0.48645887	-0.179064072	6.09E-12	6.03E-10
TSS200	MIR455	9	116971621/116971578	2	cg14361119/cg18717689	0.542717455	0.416557061	0.364778045	-0.17793941	6.95E-08	8.05E-07
TSS200	MIR487A	14	101518615/101518766	2	cg00395657/cg23399577	0.740178605	0.662458381	0.58874707	-0.151431535	1.11E-09	2.42E-08
TSS200	MIR493	14	101335381/101335283/101335228/101335386	4	cg04802696/cg11346901/cg12785573/cg16762843	0.691728341	0.603911956	0.524062593	-0.167665748	7.55E-07	6.60E-06
TSS200	MIR520B	19	54204449/54204403	2	cg17857791/cg19606290	0.685668638	0.628304847	0.503216936	-0.182451701	5.64E-07	5.08E-06
TSS200	MIR520D	19	54223189	1	cg22457503	0.61583299	0.4944214	0.398564273	-0.217268717	6.08E-09	9.97E-08
TSS200	MIR525	19	54200652	1	cg09514545	0.38079224	0.282318928	0.229140314	-0.151651926	1.57E-08	2.20E-07
TSS200	MIR526A2	19	54230170	1	cg15937784	0.494793435	0.410986617	0.291583436	-0.203209999	5.09E-13	1.67E-10
TSS200	MIR548B	6	119390418	1	cg12396622	0.879808835	0.786142539	0.643347727	-0.236461108	9.03E-08	1.01E-06
TSS200	MIR549	15	81134479/81134497	2	cg16530970/cg22929280	0.75898514	0.642816275	0.503819807	-0.255165333	1.15E-08	1.70E-07
TSS200	MIR563	3	15915216	1	cg23023604	0.66340685	0.508635728	0.382680332	-0.280726518	5.60E-13	1.75E-10
TSS200	MIR628	15	55665236	1	cg09672452	0.532736895	0.465960717	0.375190145	-0.15754675	2.64E-05	0.000156657
TSS200	MIR654	14	101506532/101506379	2	cg04585937/cg14144314	0.692308923	0.597723656	0.506532516	-0.185776407	1.38E-08	2.00E-07
TSS200	MIR662	16	820050/820122/820155	3	cg06502071/cg16108835/cg26945867	0.692884463	0.61703585	0.526998143	-0.165886319	3.92E-09	6.97E-08
TSS200	MIR670	11	43581175/43581118/43581108	3	cg07616094/cg14294096/cg26024682	0.571509677	0.480157613	0.371256305	-0.200253372	5.57E-10	1.42E-08
TSS200	MIR708	11	79113178	1	cg05473648	0.75209949	0.680079061	0.566415323	-0.185684167	4.50E-06	3.23E-05
TSS200	MIR885	3	10436406/10436304/10436249/10436325/10436278	5	cg05365685/cg05571558/cg20745248/cg23230910/cg24605341	0.74391386	0.672547542	0.566578798	-0.177335062	3.23E-10	9.22E-09
TSS200	MIR889	14	101514051	1	cg03290602	0.77199904	0.711557767	0.545312605	-0.226686435	1.35E-06	1.12E-05

TSS200	MIR9-3	15	89911148	1	cg12530503	0.54023667	0.58918945	0.704973841	0.164737171	3.19E-07	3.05E-06
TSS200	MME	3	154797349/154797867/154797508/154797399/154797592/154797589/154797563/154797958/154797677/154797779/154797947/154797917/154797892	13	cg00992239/cg02108850/cg03648334/cg08085954/cg14750844/cg16580737/cg17344755/cg20216802/cg21496658/cg22471230/cg22955973/cg23209255/cg26872138	0.11955087	0.164302741	0.288402509	0.168851638	0.002667606	0.010056294
TSS200	MMP1	11	102669090	1	cg14543953	0.720343735	0.589016539	0.404577436	-0.315766299	1.27E-08	1.85E-07
TSS200	MMP12	11	102745934/102745854	2	cg09002726/cg23979520	0.651482835	0.527188967	0.453365866	-0.198116969	2.09E-11	1.37E-09
TSS200	MMP13	11	102826570/102826565/102826469	3	cg14995062/cg19620758/cg22658979	0.752634965	0.557625358	0.396195655	-0.35643931	3.31E-13	1.34E-10
TSS200	MMP16	8	89339819	1	cg04684553	0.136884403	0.189701904	0.422589257	0.285704854	7.63E-05	0.000404791
TSS200	MMP20	11	102496104/102496115/102496159	3	cg12020179/cg22322158/cg26757793	0.568922442	0.48167883	0.379348689	-0.189573752	1.61E-11	1.17E-09
TSS200	MMP26	11	5009311	1	cg12493906	0.7180576	0.542074328	0.422492664	-0.295564936	3.59E-13	1.38E-10
TSS200	MOXD1	6	132722810/132722664/132722778/132722770/132722822	5	cg01019028/cg08661899/cg13640626/cg21294812/cg26178272	0.127174648	0.13160774	0.286870367	0.159695718	0.001967537	0.007651318
TSS200	MOXD2	7	141946928	1	cg16697850	0.40665397	0.291804311	0.226183195	-0.180470775	1.37E-11	1.06E-09
TSS200	MPP7	10	28571186	1	cg17749384	0.65685662	0.5836249	0.498176509	-0.158680111	9.49E-07	8.13E-06
TSS200	MPPED1	22	43807949/43807980	2	cg01148441/cg22347322	0.247144288	0.289267228	0.42319412	0.176049833	1.54E-05	9.71E-05
TSS200	MPZ	1	161279762	1	cg00259886	0.48032496	0.387680144	0.274989318	-0.205335642	7.84E-12	7.10E-10
TSS200	MRGPRD	11	68748461/68748528/68748506/68748584	4	cg02023548/cg10791422/cg16659728/cg18790382	0.595963315	0.490875254	0.394591273	-0.201372042	4.50E-11	2.21E-09
TSS200	MRGPRG	11	3240201/3240068/3240122	3	cg02681040/cg24702253/cg26462595	0.667035373	0.580236394	0.499476932	-0.167558441	3.48E-10	9.86E-09
TSS200	MRGPRX1	11	18956637	1	cg09346831	0.66649953	0.583576022	0.4947381	-0.17176143	4.86E-09	8.34E-08
TSS200	MRPS21	1	150266242/150266158/150266227/150266194	4	cg01566242/cg04724277/cg23650482/cg25373063	0.130523937	0.188370463	0.288049793	0.157525856	6.74E-05	0.000361026
TSS200	MSC	8	72756882/72756863/72756812/72756901/72756839	5	cg03347018/cg14546394/cg20675505/cg24258699/cg25827490	0.243803204	0.377820598	0.542911809	0.299108604	4.29E-08	5.27E-07

TSS200	MT4	16	56598820	1	cg13373996	0.676624915	0.616311461	0.521333891	-0.155291024	3.42E-07	3.24E-06
TSS200	MTMR7	8	17271105/17271093/17271067/17271119	4	cg04792712/cg08231493/cg12296772/cg15600488	0.428963725	0.454124111	0.608298614	0.179334889	0.004579839	0.016283988
TSS200	MUC13	3	124653632/124653659/124653718	3	cg06196689/cg22907034/cg26225893	0.487771923	0.383388117	0.292570168	-0.195201755	2.34E-10	7.12E-09
TSS200	MUC2	11	1074869/1074682/1074873/1074749/1074715/1074757	6	cg02332740/cg03502236/cg04657268/cg11024522/cg11247695/cg13911594	0.546999983	0.434163449	0.349758981	-0.197241002	1.76E-11	1.21E-09
TSS200	MUC20	3	195447691	1	cg05012239	0.48568542	0.381295906	0.295891841	-0.189793579	2.32E-10	7.10E-09
TSS200	MUC21	6	30951394/30951377	2	cg07538160/cg15442792	0.497656298	0.369530936	0.280403155	-0.217253143	5.92E-12	5.98E-10
TSS200	MUM1	19	1356269/1356153/1356150/1356304/1356315/1356278	6	cg02413187/cg04604656/cg08643131/cg13646005/cg23057220/cg23674169	0.485383692	0.399680735	0.310210267	-0.175173425	8.95E-09	1.38E-07
TSS200	MYF5	12	81110706	1	cg14067873	0.39254629	0.462450628	0.580411205	0.187864915	9.00E-06	6.00E-05
TSS200	MYH4	17	10372972	1	cg08014964	0.501631285	0.437755567	0.341907541	-0.159723744	0.000174779	0.000860741
TSS200	MYL5	4	671550/671543	2	cg09959931/cg21254883	0.47465933	0.389817233	0.302585759	-0.172073571	4.01E-10	1.09E-08
TSS200	MYLK4	6	2751204	1	cg25513776	0.44642793	0.363208144	0.279663864	-0.166764066	2.29E-08	3.07E-07
TSS200	MYO15B	17	73583945/73584108/73584111/73584068/73584029/73584070	6	cg00204465/cg03744440/cg04738888/cg06390536/cg12184886/cg22287064	0.441245633	0.51855737	0.63098182	0.189736187	1.48E-05	9.37E-05
TSS200	MYOD1	11	17740977/17741070/17740967	3	cg07271264/cg22653976/cg26410450	0.2867668	0.3895931	0.629171445	0.342404645	6.47E-10	1.59E-08
TSS200	MYOG	1	203055422/203055479/203055507	3	cg10901806/cg19251719/cg23098069	0.599581802	0.494840748	0.423962858	-0.175618944	1.12E-10	4.21E-09
TSS200	MYOT	5	137203424/137203358	2	cg13660086/cg21589375	0.676106598	0.591997544	0.515949066	-0.160157532	2.69E-06	2.05E-05
TSS200	MYT1	20	62795688/62795807/62795658	3	cg12828073/cg16772207/cg21367256	0.676374315	0.59385585	0.461912716	-0.214461599	1.48E-11	1.11E-09
TSS200	NAALADL1	11	64826135	1	cg27607805	0.50257657	0.393199961	0.313416773	-0.189159797	2.11E-11	1.38E-09
TSS200	NALCN	13	102068989/102068996/102069010	3	cg10774877/cg15202954/cg23056157	0.393140547	0.52235077	0.672534109	0.279393562	7.63E-10	1.81E-08
TSS200	NANOG	12	7941959	1	cg25540142	0.690967845	0.612211528	0.539083059	-0.151884786	0.000354867	0.001622469
TSS200	NCAM2	21	22370444	1	cg02842227	0.258064435	0.334532006	0.424384645	0.16632021	8.23E-10	1.93E-08

TSS200	NCAN	19	19322676/19322778/19322640/19322678	4	cg08181476/cg13724788/cg14316898/cg22090773	0.251505604	0.331823175	0.498386361	0.246880758	4.32E-07	3.98E-06
TSS200	NCR3	6	31560871/31560776	2	cg08610982/cg23484380	0.384610105	0.311848406	0.229959132	-0.154650973	1.96E-06	1.55E-05
TSS200	NCRNA00157	21	19258040	1	cg14288673	0.49441642	0.420160894	0.339186423	-0.155229997	1.25E-09	2.69E-08
TSS200	NCRNA00162	21	46424834/46424753/46424715	3	cg04070987/cg22495068/cg22942384	0.666334835	0.582441909	0.51051608	-0.155818755	1.61E-11	1.17E-09
TSS200	NDRG4	16	58498574/58497395/58497815/58498636/58498585/58497801/58498710/58498754	8	cg00262031/cg01466678/cg02040433/cg04005075/cg06650115/cg08791131/cg09324514/cg16812519	0.10765913	0.17862198	0.310097551	0.202438421	2.28E-06	1.77E-05
TSS200	NDST1	5	149887486/149887497	2	cg06677890/cg16674484	0.4413471	0.326739653	0.282865268	-0.158481832	1.17E-12	2.37E-10
TSS200	NDST4	4	116035217/116035082	2	cg14204430/cg26869131	0.582727335	0.539693156	0.424504486	-0.158222849	2.60E-06	1.99E-05
TSS200	NECAB1	8	91803805/91803792	2	cg01026458/cg20040743	0.351661485	0.41792425	0.545203416	0.193541931	5.48E-08	6.51E-07
TSS200	NEFL	8	24814137	1	cg01614020	0.31224608	0.329022772	0.493136339	0.180890259	6.22E-05	0.000335838
TSS200	NEFM	8	24772435/24772350/24771259/24771114/24771125/24772344/24772270/24772333/24772309	9	cg03169018/cg04118306/cg07502389/cg09234518/cg16459364/cg17078116/cg19677607/cg20585869/cg22562942	0.411648573	0.464888808	0.577686086	0.166037513	1.41E-10	4.95E-09
TSS200	NELL1	11	20690934/20690930/20690957	3	cg10824063/cg12071328/cg17371081	0.478953862	0.577332452	0.686328424	0.207374563	1.40E-08	2.01E-07
TSS200	NETO1	18	70533078/70535005	2	cg13861749/cg18651870	0.6870667	0.5101433	0.36370005	-0.32336665	1.42E-14	1.93E-11
TSS200	NGEF	2	233877969/233878015/233878004/233878150	4	cg04363282/cg06143658/cg09312809/cg21998869	0.518786182	0.422450967	0.342445038	-0.176341144	1.60E-10	5.42E-09
TSS200	NGF	1	115880865/115881044/115880935	3	cg09008705/cg13613280/cg13804058	0.223457763	0.326913945	0.504004302	0.28054654	9.98E-07	8.52E-06
TSS200	NHLH1	1	160336699/160336712	2	cg00006397/cg00881010	0.50540425	0.410566239	0.332137055	-0.173267195	8.64E-09	1.35E-07
TSS200	NKAIN4	20	61886082/61886079/61885926	3	cg01911708/cg08111446/cg23942897	0.236576717	0.36331312	0.519381209	0.282804493	3.66E-09	6.60E-08

TSS200	NKAPL	6	28226905/28227091/28227093/28227085/28227068/28227079	6	cg00765128/cg01031101/cg09523275/cg10253847/cg17384889/cg18694169	0.472472486	0.579508745	0.649611709	0.177139223	3.90E-10	1.07E-08
TSS200	NKX2-2	20	21494712/21494689/21494748/21494762/21494679/21494831/21494716	7	cg06178563/cg06624291/cg07070391/cg11651285/cg16524928/cg21409881/cg22265644	0.330671879	0.408267321	0.548217548	0.217545669	1.56E-06	1.27E-05
TSS200	NKX2-4	20	21378220/21378211/21378072/21378227/21378200/21378074	6	cg05227131/cg21200408/cg21200656/cg22204479/cg26156448/cg27147819	0.227931307	0.268171764	0.413709508	0.185778202	7.06E-07	6.22E-06
TSS200	NKX2-6	8	23564008/23564031/23563925/23563970/23564025	5	cg05477514/cg13324546/cg14428146/cg15854847/cg20892260	0.419070284	0.516927756	0.641974525	0.222904241	9.35E-12	7.90E-10
TSS200	NLRP6	11	278538/278394/278462/278421/278403/278450	6	cg03009397/cg09205751/cg15451020/cg18016138/cg22331032/cg23437420	0.597769605	0.511934688	0.433281844	-0.164487761	9.01E-12	7.77E-10
TSS200	NMBR	6	142410100/142409939/142410098/142409993/142410061	5	cg17256157/cg17345081/cg19233753/cg24924958/cg26365690	0.353814663	0.463131303	0.595425561	0.241610899	2.22E-11	1.42E-09
TSS200	NPAS4	11	66188441/66188433	2	cg13215078/cg23484234	0.236016241	0.321604036	0.514617661	0.278601421	5.85E-08	6.89E-07
TSS200	NPBWR1	8	53852274/53852443/53852422/53852446	4	cg01559663/cg02237470/cg07770968/cg18755296	0.356122273	0.446057847	0.591269406	0.235147133	6.86E-10	1.67E-08
TSS200	NPR3	5	32711517	1	cg18261223	0.083820478	0.107360912	0.28028821	0.196467732	7.90E-05	0.000418196
TSS200	NPY	7	24323767/24323792/24323799/24323675/24323764	5	cg00355281/cg15929698/cg16964348/cg24242823/cg24885417	0.22485096	0.459305334	0.603738122	0.378887161	6.39E-11	2.84E-09
TSS200	NPY2R	4	156129735/156129762/156129655/156129725	4	cg03202738/cg14811608/cg26384430/cg27504805	0.128974155	0.194864671	0.299241116	0.170266962	0.000452748	0.002017345
TSS200	NR1H4	12	100867509/100867529	2	cg15381313/cg18874332	0.437591085	0.358142175	0.284710657	-0.152880428	1.26E-08	1.85E-07

TSS200	NR2E1	6	108487100/108487072/108487183/108487160/108487078/108487063	6	cg07411432/cg08616702/cg18093751/cg18918349/cg24104938/cg24225517	0.250054981	0.263668597	0.45908247	0.209027489	2.88E-05	0.000168797
TSS200	NRXN1	2	51259697/50574912/51259693/50575030/51259807/50575093/50575069/50575037/51259703/51259786	10	cg03168108/cg03295083/cg05495351/cg10099415/cg13594075/cg13906811/cg17677030/cg18201351/cg22324022/cg25717438	0.253003499	0.31461233	0.405949359	0.15294586	6.55E-07	5.80E-06
TSS200	NTRK3	15	88799820/88799707	2	cg13773705/cg21956337	0.24426909	0.412779906	0.575837173	0.331568083	6.11E-07	5.45E-06
TSS200	NTSR1	20	61340116/61340107/61340109	3	cg01539036/cg05295038/cg09893588	0.097805414	0.142690528	0.295746321	0.197940907	0.000317768	0.001469037
TSS200	NUAK1	12	106533903/106533840/106533867/106533898/106533863	5	cg02518216/cg17393917/cg18525352/cg20060685/cg23555120	0.496374778	0.653919296	0.726852429	0.230477651	1.71E-10	5.66E-09
TSS200	NWD1	19	16830613/16830749/16830746/16830739	4	cg15845821/cg19344626/cg19784428/cg20249566	0.429900116	0.353897649	0.271156008	-0.158744108	1.55E-09	3.19E-08
TSS200	NXPH2	2	139537845/139537826/139537824	3	cg00804628/cg14923640/cg18150439	0.318403323	0.395305481	0.514745968	0.196342645	2.97E-11	1.68E-09
TSS200	OBP2A	9	138437868	1	cg08028341	0.55521711	0.46230235	0.397909868	-0.157307242	9.37E-08	1.04E-06
TSS200	OBP2B	9	136084818	1	cg15538820	0.516362855	0.412994322	0.331981636	-0.184381219	6.08E-11	2.75E-09
TSS200	ODZ4	11	79151719/79151814/79151791/79151811	4	cg04983516/cg05099909/cg05218311/cg09673208	0.120875758	0.168718308	0.297606181	0.176730423	0.00091667	0.003828117
TSS200	OLIG2	21	34398114/34398199/34398131/34398222/34398085/34398226	6	cg03696345/cg08870743/cg15299832/cg23253569/cg27254482/cg27357571	0.254698848	0.380666611	0.584316247	0.329617399	6.39E-10	1.57E-08
TSS200	OLIG3	6	137815699/137815725/137815569/137815539/137815648/137815572	6	cg02550277/cg03093838/cg05262436/cg06918467/cg10632395/cg14726488	0.324246414	0.401423876	0.517361073	0.19311466	3.79E-07	3.54E-06
TSS200	OPCML	11	132813235/133402545/133402497/133402500/133402589	5	cg08769451/cg15964611/cg16639665/cg23236270/cg24250669	0.331918792	0.382152184	0.497281194	0.165362402	3.55E-06	2.62E-05

TSS200	OPRK1	8	54164310/54164296/54164391/54164374	4	cg09999109/cg13405783/cg16491617/cg25614094	0.334068585	0.414222804	0.575456953	0.241388368	3.47E-06	2.57E-05
TSS200	OPRM1	6	154360483/154360351/154360344	3	cg06649410/cg14262937/cg22370006	0.335197423	0.401278389	0.521347702	0.18615028	4.46E-06	3.21E-05
TSS200	OR10A2	11	6890860	1	cg09577324	0.467693435	0.370606628	0.265803114	-0.201890321	5.64E-10	1.44E-08
TSS200	OR10A6	11	7950225	1	cg00444390	0.602448615	0.459103683	0.296502964	-0.305945651	1.38E-12	2.58E-10
TSS200	OR11A1	6	29395657	1	cg16487621	0.716882105	0.675083656	0.537499155	-0.17938295	6.52E-07	5.78E-06
TSS200	OR1J4	9	125281227	1	cg24137660	0.44187848	0.3713173	0.252136536	-0.189741944	1.27E-06	1.06E-05
TSS200	OR2A5	7	143747474/143747422	2	cg07055879/cg08425482	0.593725525	0.494588533	0.425970782	-0.167754743	1.03E-05	6.74E-05
TSS200	OR2G6	1	248684766	1	cg25819275	0.735832695	0.613297533	0.421585591	-0.314247104	1.96E-09	3.86E-08
TSS200	OR2L3	1	248223974	1	cg21207403	0.536489	0.408806294	0.320260268	-0.216228732	2.07E-09	4.03E-08
TSS200	OR2M3	1	248366332	1	cg06393354	0.652677565	0.498601872	0.411762314	-0.240915251	6.05E-09	9.93E-08
TSS200	OR2T3	1	248636455	1	cg12085619	0.745767715	0.665975322	0.590819186	-0.154948529	1.53E-07	1.58E-06
TSS200	OR4C12	11	50004210	1	cg15690511	0.425303555	0.317127417	0.24445805	-0.180845505	1.96E-12	3.21E-10
TSS200	OR4C46	11	51515083	1	cg18369913	0.52651766	0.397022644	0.279748995	-0.246768665	1.15E-13	6.70E-11
TSS200	OR4D1	17	56232499/56232357	2	cg12463722/cg22466653	0.62969987	0.522533925	0.401109232	-0.228590638	5.02E-12	5.39E-10
TSS200	OR51E2	11	4719138	1	cg25322008	0.40619577	0.323781111	0.251573682	-0.154622088	1.52E-11	1.12E-09
TSS200	OR51M1	11	5410452	1	cg27093637	0.659121375	0.532627261	0.382448045	-0.27667333	9.75E-09	1.48E-07
TSS200	OR52A1	11	5173605	1	cg22812280	0.70131895	0.619109628	0.49463495	-0.206684	2.26E-06	1.77E-05
TSS200	OR52B2	11	6191635	1	cg20213166	0.523759635	0.397554928	0.297644791	-0.226114844	2.39E-12	3.81E-10
TSS200	OR52L1	11	6008246	1	cg27480913	0.440982445	0.322647794	0.208424695	-0.23255775	3.89E-15	1.23E-11
TSS200	OR52N2	11	5841482	1	cg03895540	0.597053695	0.476309817	0.315040418	-0.282013277	1.07E-13	6.48E-11
TSS200	OR5A1	11	59210634	1	cg10919344	0.518778455	0.366911578	0.2463723	-0.272406155	8.65E-14	5.93E-11
TSS200	OR5D16	11	55606216	1	cg22937354	0.53126326	0.466551078	0.300326945	-0.230936315	2.70E-07	2.63E-06
TSS200	OR5T1	11	56043040	1	cg01464104	0.62377062	0.491010067	0.419395941	-0.204374679	5.72E-06	3.99E-05
TSS200	OR5W2	11	55682099	1	cg26349048	0.58245256	0.516382378	0.341970668	-0.240481892	1.51E-11	1.12E-09
TSS200	OR6K2	1	158670617	1	cg02245566	0.751338125	0.663984689	0.559207132	-0.192130993	1.82E-05	0.000112937
TSS200	OR7A5	19	14939300	1	cg25851651	0.728034585	0.677322967	0.573283773	-0.154750812	0.000178057	0.000875242
TSS200	OR8B12	11	124413574/124413728	2	cg03168749/cg25362709	0.720342223	0.607519542	0.452668577	-0.267673645	2.23E-09	4.29E-08
TSS200	OR8B4	11	124294778	1	cg24653728	0.6793003	0.600804211	0.507089673	-0.172210627	9.48E-09	1.45E-07
TSS200	OR8J3	11	55905246	1	cg11323650	0.578564595	0.408161344	0.2954314	-0.283133195	3.06E-11	1.71E-09
TSS200	OSMR	5	38846034/38845762	2	cg02390103/cg19609242	0.330263328	0.417235351	0.549658657	0.219395329	2.12E-10	6.69E-09

TSS200	OTP	5	76934693/76934634/76934696	3	cg13378628/cg16691593/cg21269843	0.281914829	0.337635057	0.48559508	0.203680251	9.69E-06	6.39E-05
TSS200	OTX2	14	57277227/57277366	2	cg15607672/cg26998537	0.388683973	0.487170308	0.585204866	0.196520893	7.09E-07	6.24E-06
TSS200	PABPC4L	4	135122993	1	cg11815708	0.307129375	0.367251017	0.543101264	0.235971889	1.45E-06	1.19E-05
TSS200	PABPN1L	16	88933182/88933070/88933068	3	cg04587581/cg06076690/cg08999742	0.5992099	0.496629044	0.401524527	-0.197685373	9.64E-10	2.18E-08
TSS200	PALM2	9	112402882	1	cg02442436	0.23853382	0.350415644	0.511096645	0.272562825	2.03E-08	2.77E-07
TSS200	PAPPA2	1	176432162	1	cg02051981	0.49337363	0.386976028	0.308672577	-0.184701053	7.39E-11	3.17E-09
TSS200	PAQR9	3	142682288/142682291	2	cg15852572/cg27406373	0.081945404	0.091917814	0.253962734	0.17201733	0.000216837	0.001043105
TSS200	PARP12	7	139763542/139763574/139763659	3	cg21015022/cg22165171/cg25279100	0.601529968	0.506477167	0.436651298	-0.16487867	2.75E-10	8.14E-09
TSS200	PART1	5	59783629/59783668	2	cg09712066/cg18704047	0.422541028	0.342170589	0.270053657	-0.152487371	1.05E-09	2.33E-08
TSS200	PATE1	11	125616118/125616160/125616168	3	cg02685436/cg05391338/cg11283289	0.645320025	0.545441806	0.451285217	-0.194034808	6.01E-09	9.88E-08
TSS200	PATE3	11	125657958	1	cg20737028	0.40751477	0.297983333	0.223018836	-0.184495934	1.11E-08	1.66E-07
TSS200	PCDH10	4	134070437/134070369/134070433/134070389/134070441/134070416	6	cg01408654/cg02562431/cg10524033/cg12746059/cg14400886/cg27600205	0.277331374	0.356228214	0.532015105	0.254683731	1.35E-07	1.43E-06
TSS200	PCDH17	13	58205678	1	cg03865667	0.45875174	0.534007094	0.637959727	0.179207987	1.10E-09	2.42E-08
TSS200	PCDH24	5	175976342/175976214/175976188	3	cg02615833/cg09734011/cg10462998	0.54325131	0.459487703	0.382872145	-0.160379165	1.43E-10	4.99E-09
TSS200	PCDH8	13	53422847/53422860/53422871/53422808/53422777/53422780	6	cg03315215/cg03850426/cg16027761/cg17535595/cg19712603/cg22763718	0.221548474	0.259730501	0.414794607	0.193246133	0.000648057	0.002788323
TSS200	PCDHA1	5	140165687	1	cg12150457	0.36141468	0.449483122	0.582886055	0.221471375	4.20E-06	3.03E-05
TSS200	PCDHA13	5	140261749/140261697/140261663/140261653/140261759/140261810	6	cg00275741/cg01202150/cg02051771/cg19876814/cg25967418/cg26556719	0.513958965	0.594796847	0.692800887	0.178841922	2.73E-10	8.10E-09
TSS200	PCDHA3	5	140180637/140180691	2	cg01089834/cg19777540	0.405721173	0.513173864	0.625355866	0.219634693	1.31E-08	1.91E-07
TSS200	PCDHA7	5	140213795/140213838/140213924/140213783/140213825	5	cg06827234/cg07745166/cg12962191/cg18117367/cg26618441	0.473146746	0.556270029	0.65707222	0.183925474	1.27E-07	1.35E-06

TSS200	PCDHB1	5	140430921/140430962/140430875	3	cg06827143/cg06899976/cg12664940	0.299211843	0.355705656	0.449979266	0.150767423	4.69E-05	0.000261262
TSS200	PCDHB15	5	140625059/140625004/140625130/140625068	4	cg11368643/cg18664869/cg23941075/cg24951286	0.505167158	0.592685225	0.668388075	0.163220918	1.16E-06	9.78E-06
TSS200	PCDHB18	5	140613869/140613740/140613859/140613884/140613820	5	cg04511041/cg06302533/cg11286063/cg18661205/cg27086874	0.358742672	0.469277813	0.591563247	0.232820575	9.37E-10	2.14E-08
TSS200	PCDHB19P	5	140619567/140619586	2	cg10553103/cg25010500	0.532207835	0.611180508	0.704408741	0.172200906	6.12E-07	5.46E-06
TSS200	PCDHGA11	5	140800495/140800474/140800424/140800398	4	cg03892308/cg17857974/cg18118262/cg26647197	0.420062418	0.588938167	0.720942086	0.300879669	2.99E-10	8.72E-09
TSS200	PCDHGA12	5	140810109/140810137/140810123/140810106/140810051	5	cg02452944/cg07730329/cg12304520/cg21627409/cg23082339	0.468492233	0.600537814	0.700518228	0.232025995	5.90E-10	1.48E-08
TSS200	PCDHGA2	5	140718339/140718283/140718317	3	cg04830357/cg11011625/cg18878616	0.172768198	0.240883724	0.352288352	0.179520155	0.000380198	0.001724288
TSS200	PCDHGA3	5	140723455/140723568/140723509/140723583/140723549	5	cg06742719/cg12608145/cg14468658/cg17588578/cg18781988	0.481615587	0.554700815	0.650656171	0.169040585	1.24E-08	1.82E-07
TSS200	PCDHGA7	5	140762315/140762305	2	cg12459904/cg21908557	0.369187088	0.449634844	0.564998014	0.195810926	2.91E-06	2.20E-05
TSS200	PCDHGB1	5	140729719/140729813/140729653	3	cg12375722/cg21073350/cg21161927	0.384624802	0.46478868	0.587375938	0.202751136	2.07E-07	2.07E-06
TSS200	PCDHGB3	5	140749834/140749783	2	cg23348764/cg26337998	0.269500098	0.335242484	0.422175191	0.152675093	0.000237891	0.001134353
TSS200	PCDHGB4	5	140767255/140767364/140767412/140767318	4	cg00911351/cg07125905/cg19734465/cg22648135	0.407194805	0.483649346	0.589756299	0.182561494	3.78E-08	4.75E-07
TSS200	PCDHGB5	5	140777607/140777501/140777503/140777611	4	cg03608712/cg04553690/cg08491188/cg24954590	0.424718165	0.474536371	0.611287741	0.186569576	6.81E-08	7.88E-07
TSS200	PCDHGB6	5	140787623	1	cg09465698	0.5019589	0.657085994	0.708289209	0.206330309	2.52E-06	1.94E-05
TSS200	PCDHGB7	5	140797280/140797172/140797234	3	cg10435816/cg13933262/cg14011639	0.411212445	0.552137115	0.66463272	0.253420275	3.73E-07	3.50E-06

TSS200	PCDHGC4	5	140864578/140864593/140864701/140864549/140864733	5	cg02022733/cg03640756/cg15361590/cg15949044/cg23445461	0.296129086	0.459798741	0.667165305	0.371036219	4.48E-14	4.41E-11
TSS200	PCP4L1	1	161228455/161228496	2	cg10775792/cg27223727	0.101200112	0.089806782	0.263402179	0.162202067	0.000528043	0.002320063
TSS200	PDE1B	12	54943102/54943009/54954941	3	cg00639886/cg13565157/cg16643603	0.118706076	0.218631614	0.362128276	0.2434222	4.01E-05	0.000227604
TSS200	PDE4D	5	59189791/59189788/59189724/59189649	4	cg11258089/cg16261871/cg22706610/cg26870744	0.322156955	0.360674003	0.494226345	0.17206939	2.42E-05	0.000145054
TSS200	PDGFD	11	104035151/104035204/104035136/104035087	4	cg03145963/cg08596000/cg12027636/cg26756506	0.108127433	0.206495605	0.362922495	0.254795063	2.53E-06	1.95E-05
TSS200	PDPN	1	13910224/13910206/13910138/13910164/13910209/13910156	6	cg02933679/cg10743104/cg11791751/cg15563963/cg16277479/cg22436123	0.257968218	0.342812772	0.504722091	0.246753873	4.08E-07	3.78E-06
TSS200	PDZK1	1	145727720	1	cg13019092	0.549227525	0.440546956	0.3495518	-0.199675725	4.71E-12	5.24E-10
TSS200	PEG10	7	94285501/94285520	2	cg04366249/cg14388858	0.168816405	0.201851454	0.330592637	0.161776232	0.001810142	0.007093472
TSS200	PENK	8	57358651/57358713/57359414/57358625/57359399	5	cg03483150/cg04612444/cg11060276/cg18742346/cg19149681	0.401505867	0.50844703	0.65580363	0.254297764	4.42E-12	5.13E-10
TSS200	PER4	7	9673790	1	cg16465407	0.731968155	0.654732922	0.576260605	-0.15570755	8.64E-05	0.000453967
TSS200	PEX5L	3	179754529/179754603/179754615/179754613/179754533/179754521	6	cg02009585/cg02119363/cg04894619/cg13473356/cg23346462/cg23756251	0.273908536	0.36076177	0.479007376	0.20509884	3.91E-08	4.88E-07
TSS200	PHACTR1	6	12717790/12717776	2	cg07279321/cg14931071	0.63859504	0.503514406	0.413683873	-0.224911167	4.56E-09	7.96E-08
TSS200	PHF21B	22	45405827/45405904/45405627/45405919/45405624/45405768/45405880/45405899/45405621	9	cg01995480/cg10080155/cg15679813/cg16551454/cg20495645/cg20525183/cg23080354/cg25561581/cg25976257	0.078780276	0.106175468	0.245312103	0.166531827	3.75E-05	0.000214365
TSS200	PHOX2A	11	71955322/71955299/71955332/71955401/71955337	5	cg01670677/cg02910180/cg04543008/cg05093169/cg11334771	0.317748893	0.371521035	0.510596207	0.192847314	2.76E-06	2.10E-05

TSS200	PIGR	1	207119987	1	cg12751565	0.491153695	0.397246378	0.320769264	-0.170384431	4.23E-10	1.15E-08
TSS200	PIK3R5	17	8869105/8815843/8869099/8869136/8815876/8869213/8815841/8869155	8	cg01653005/cg08003321/cg09359907/cg12863545/cg16672810/cg16918989/cg24251850/cg26998044	0.337925005	0.451489235	0.507608275	0.169683271	4.75E-05	0.00026433
TSS200	PKD1L2	16	81254010/81254169/81254051	3	cg00908271/cg03406106/cg06048169	0.679193025	0.587003689	0.46340112	-0.215791905	5.90E-11	2.71E-09
TSS200	PKD2L2	5	137224967	1	cg24330204	0.464248035	0.607403861	0.648410059	0.184162024	6.00E-06	4.17E-05
TSS200	PKIA	8	79428313/79428325	2	cg12850252/cg21612207	0.059183456	0.124409844	0.294882193	0.235698737	0.000918545	0.003834931
TSS200	PLA2G2A	1	20307002	1	cg11037787	0.47784928	0.393858028	0.327697923	-0.150151357	7.44E-10	1.78E-08
TSS200	PLA2G4F	15	42449015	1	cg26350373	0.545449265	0.425320756	0.330076345	-0.21537292	9.06E-11	3.62E-09
TSS200	PLCB1	20	8113191	1	cg27591117	0.198205965	0.268068172	0.419899173	0.221693208	2.90E-06	2.19E-05
TSS200	PLCH2	1	2407652/2407718	2	cg11229415/cg21285056	0.600133603	0.530291011	0.441910884	-0.158222718	7.17E-06	4.90E-05
TSS200	PNLIPRP2	10	118380396/118380345/118380370	3	cg01047476/cg15988569/cg25120325	0.524042465	0.3830562	0.368410718	-0.155631747	4.12E-06	2.98E-05
TSS200	PNMAL1	19	46974886/46974908/46974972	3	cg10788735/cg17787159/cg22824998	0.214271869	0.310690015	0.389562009	0.175290141	2.47E-05	0.000147476
TSS200	PNOC	8	28174455	1	cg13077562	0.68826165	0.540080617	0.412117905	-0.276143745	9.88E-10	2.22E-08
TSS200	PNPLA1	6	36238095/36238178/36238230/36238043/36238213/36238123	6	cg00980581/cg02683846/cg17767484/cg18927901/cg23059700/cg26681975	0.569272611	0.49960711	0.413286681	-0.15598593	1.19E-09	2.58E-08
TSS200	PNPLA5	22	44287876/44287958/44288021/44287930/44287905	5	cg04839422/cg11187025/cg11250773/cg20377114/cg24534477	0.26159551	0.325538919	0.443201775	0.181606265	4.61E-05	0.000257839
TSS200	POU4F3	5	145718401/145718405/145718392/145718491	4	cg01593834/cg05666607/cg18369866/cg19974854	0.326611139	0.396140022	0.518172249	0.19156111	3.24E-05	0.000187586
TSS200	PPFIA4	1	203020141	1	cg09122593	0.53092531	0.461835983	0.371604295	-0.159321015	1.84E-07	1.87E-06
TSS200	PPM1E	17	56833096/56833060/56833076/56833043/56833195/56833099/56833197/56833201	8	cg02071825/cg05264587/cg08592707/cg11019211/cg14207210/cg15386368/cg19401340/cg24526702	0.104678628	0.148781092	0.29684172	0.192163092	0.000242784	0.001156634

TSS200	PPP1R16B	20	37434231/37434167/37434229/37434260/37434158/37434262	6	cg06207052/cg10305789/cg11172693/cg12510981/cg15821319/cg21656726	0.211792482	0.350361329	0.428409348	0.216616867	1.23E-05	7.93E-05
TSS200	PPP1R1B	17	37783011/37784711/37784699/37784694	4	cg00112517/cg06068801/cg14697334/cg24520381	0.422967765	0.325790749	0.254840764	-0.168127001	3.57E-12	4.62E-10
TSS200	PPP1R3A	7	113559086	1	cg01214063	0.633804605	0.488613383	0.368097382	-0.265707223	4.21E-08	5.20E-07
TSS200	PPP2R2B	5	146461045/146258461/146258354/146258189/146258181/146258195/146258237/146258512/146258615/146258159/146258522/146258546/146258427/146258463	14	cg01987925/cg03659005/cg03851984/cg04583285/cg08774231/cg09528265/cg11826826/cg13971892/cg13983063/cg14693112/cg15927927/cg25021259/cg25149751/cg25363885	0.217695908	0.273562204	0.409593783	0.191897875	9.71E-06	6.40E-05
TSS200	PPP2R2C	4	6383645	1	cg01208224	0.638608455	0.569046378	0.463482045	-0.17512641	1.18E-05	7.65E-05
TSS200	PPYR1	10	47083426/47083355/47083392/47083342/47083398/47083504	6	cg02394955/cg04127044/cg11861387/cg17771050/cg25344503/cg25761626	0.439079737	0.525658706	0.618115232	0.179035495	4.77E-12	5.24E-10
TSS200	PRDM14	8	70983567/70983600/70983760/70983741	4	cg00384539/cg13267264/cg21255438/cg27029821	0.365703203	0.439224286	0.567735724	0.202032521	0.000302036	0.001400827
TSS200	PREX2	8	68864549/68864546/68864523/68864529	4	cg15745900/cg17147045/cg17459204/cg27467929	0.268288854	0.364995097	0.471921505	0.203632651	5.51E-06	3.86E-05
TSS200	PRKAR1B	7	767474/753007/767490/752743/752292/752715/752180/752632/752286/752624/752238/752979	12	cg02943676/cg03982150/cg06461306/cg12816748/cg13895235/cg14835981/cg16305865/cg18091301/cg18601167/cg19363669/cg20381963/cg22084339	0.385914153	0.486478513	0.615181921	0.229267768	7.57E-12	6.98E-10
TSS200	PRMT8	12	3600317/3600273/3600229	3	cg06029846/cg07912789/cg19656577	0.22945001	0.254280452	0.390998177	0.161548168	2.04E-05	0.000124676

TSS200	PRND	20	4702381/4702538/4702464/4702516	4	cg04636876/cg05837890/cg12587930/cg24427947	0.745093789	0.581627543	0.455482394	-0.289611394	4.00E-10	1.09E-08
TSS200	PROC	2	128175891/128175905	2	cg10021288/cg22856114	0.821833368	0.741553419	0.639526277	-0.18230709	6.59E-07	5.83E-06
TSS200	PROCA1	17	27039032/27039058/27038886	3	cg03049691/cg10495754/cg14676825	0.268143283	0.411542398	0.485133381	0.216990099	1.50E-07	1.56E-06
TSS200	PROKR2	20	5295137	1	cg04354077	0.642575555	0.559678556	0.449269686	-0.193305869	5.87E-09	9.70E-08
TSS200	PROM2	2	95940031/95940111	2	cg05989861/cg09664492	0.509402383	0.411794242	0.3444405	-0.164961883	4.36E-10	1.17E-08
TSS200	PRSS1	7	142457299/142457284	2	cg02329916/cg14795939	0.652315815	0.469829786	0.349246105	-0.30306971	3.62E-12	4.62E-10
TSS200	PRSS45	3	46786387	1	cg21587375	0.5802546	0.449848272	0.313391259	-0.266863341	1.77E-11	1.22E-09
TSS200	PRTN3	19	840873/840795/840950	3	cg12230709/cg17004025/cg23386659	0.38018479	0.28710825	0.218288357	-0.161896433	1.16E-11	9.36E-10
TSS200	PSCA	8	143761811/143761680/143761697	3	cg00014754/cg02616368/cg13392302	0.60395514	0.534282789	0.441146534	-0.162808606	9.84E-12	8.25E-10
TSS200	PSG8	19	43269877/43269832	2	cg11387248/cg12109087	0.533947545	0.441007428	0.355234168	-0.178713377	4.47E-12	5.15E-10
TSS200	PSORS1C2	6	31107186/31107321/31107240	3	cg07414487/cg13617087/cg26965877	0.88972946	0.789996339	0.725275318	-0.164454142	2.88E-05	0.000168679
TSS200	PTF1A	10	23481421/23481389/23481385	3	cg17076890/cg18920423/cg20014049	0.270220597	0.266804783	0.422044574	0.151823976	0.000473334	0.002103562
TSS200	PTGFR	1	78956570	1	cg24022301	0.332471	0.385515761	0.520876136	0.188405136	2.21E-06	1.73E-05
TSS200	PTH2	19	49926866/49926878/49926748	3	cg12986327/cg23996617/cg27103130	0.24650308	0.313716275	0.411745373	0.165242293	0.000347415	0.001592093
TSS200	PTH2R	2	209271383/209271497/209271505	3	cg01288372/cg23524735/cg25057743	0.302449738	0.337687743	0.481392933	0.178943195	4.02E-05	0.000228252
TSS200	PTPLAD2	9	21031822	1	cg14498666	0.382645655	0.535438133	0.649741386	0.267095731	2.57E-08	3.37E-07
TSS200	PTPN5	11	18813477/18813466/18813544/18813556/18813484	5	cg06671842/cg07717429/cg13062406/cg16426537/cg18678353	0.395132593	0.490124489	0.591420466	0.196287873	0.000157886	0.000785397
TSS200	PTPRN	2	220174251/220174232/220174207/220174201	4	cg01382864/cg03970036/cg10140638/cg16166796	0.453454794	0.549340568	0.676919491	0.223464697	1.49E-08	2.11E-07
TSS200	PTPRT	20	41818756/41818574	2	cg08413157/cg27625055	0.126841351	0.20140185	0.363025578	0.236184227	8.69E-08	9.73E-07
TSS200	PVRL4	1	161059535/161059458/161059429	3	cg03166324/cg14158583/cg16557111	0.534196657	0.444325406	0.354403842	-0.179792814	2.31E-10	7.10E-09

TSS200	PWRN1	15	24803245/24803181	2	cg03402443/cg22884857	0.56207729	0.491806878	0.407357932	-0.154719358	1.44E-09	2.98E-08
TSS200	PYGO1	15	55881162/55881246/55881083/55881075/55881089/55881081	6	cg02715546/cg06364757/cg09275667/cg11480800/cg17841803/cg23030278	0.283902338	0.400563924	0.508187398	0.22428506	2.70E-06	2.06E-05
TSS200	PZP	12	9360986	1	cg08729600	0.46703459	0.3780515	0.299266245	-0.167768345	1.58E-10	5.38E-09
TSS200	QRFPR	4	122302276/122302268/122302226/122302331/122302232/122302265	6	cg03356900/cg05604079/cg13643914/cg15105182/cg16999370/cg24727399	0.318385988	0.350584718	0.498228426	0.179842438	0.000246957	0.001170504
TSS200	R3HDM2	12	57704322	1	cg16346236	0.826596475	0.766385589	0.668009077	-0.158587398	3.41E-08	4.33E-07
TSS200	RAB25	1	156030844/156030809	2	cg09243900/cg15896939	0.545682275	0.454175328	0.3636734	-0.182008875	3.66E-10	1.02E-08
TSS200	RAB34	17	27045048/27045302/27045043	3	cg08839210/cg19982230/cg21237418	0.468963962	0.569896974	0.648155276	0.179191314	1.39E-09	2.89E-08
TSS200	RADIL	7	4923383	1	cg27546065	0.060555804	0.091632795	0.224144239	0.163588435	0.001739177	0.006847712
TSS200	RALYL	8	85095275/85097056/85095498/85095394/85097031/85097049	6	cg00266009/cg04842146/cg05716166/cg16020747/cg21671806/cg23649708	0.300238937	0.341182228	0.527346692	0.227107755	3.69E-08	4.65E-07
TSS200	RARRES2	7	150038898	1	cg27494647	0.588244105	0.641652089	0.757733695	0.16948959	1.19E-05	7.70E-05
TSS200	RASGRF1	15	79383413/79383385	2	cg15156078/cg19431448	0.196443749	0.311090539	0.479263118	0.282819369	1.12E-08	1.67E-07
TSS200	RAX	18	56940767/56940755/56940693	3	cg03074888/cg08724563/cg27019126	0.190562846	0.254722714	0.372007909	0.181445063	2.18E-08	2.94E-07
TSS200	REG1A	2	79347532/79347474	2	cg16791528/cg23111544	0.593622985	0.47183715	0.376801032	-0.216821953	2.62E-11	1.54E-09
TSS200	REG3G	2	79252794/79252673	2	cg01357846/cg18145810	0.45832843	0.361260508	0.294910659	-0.163417771	3.69E-10	1.03E-08
TSS200	REG4	1	120354250	1	cg07193534	0.631496085	0.488209211	0.378247941	-0.253248144	4.47E-11	2.21E-09
TSS200	RETNLB	3	108476278/108476163	2	cg01287218/cg20441894	0.351536945	0.2460125	0.184027688	-0.167509257	3.10E-12	4.37E-10
TSS200	RFPL1	22	29834550/29834420/29834388	3	cg03012854/cg24246245/cg26288779	0.75423756	0.662904756	0.590332515	-0.163905045	1.39E-08	2.00E-07
TSS200	RFPL3S	22	32767128/32767094/32767162/32767005	4	cg05484394/cg05544007/cg16655295/cg22217678	0.735468285	0.628548556	0.525246669	-0.210221616	9.66E-13	2.12E-10
TSS200	RFPL4B	6	112668522/112668401/112668454	3	cg00088428/cg13585985/cg21295811	0.744031847	0.639042052	0.553107327	-0.190924519	3.09E-08	3.97E-07

TSS200	RFTN1	3	16555401/16555347/16555412/16555311/16555379/16555288	6	cg09375878/cg17377625/cg20660627/cg22044848/cg23718026/cg23955684	0.086252716	0.116371045	0.242106302	0.155853587	0.001440909	0.005775723
TSS200	RGS13	1	192605253	1	cg05023691	0.610716955	0.517053761	0.459578459	-0.151138496	5.94E-05	0.00032291
TSS200	RGS22	8	101118512/101118377/101118525	3	cg20051292/cg23208513/cg24848035	0.27818651	0.360213028	0.499529882	0.221343372	7.30E-09	1.17E-07
TSS200	RGS6	14	72399591	1	cg01767732	0.21390877	0.249187733	0.369543673	0.155634903	0.000300595	0.001394965
TSS200	RGS7BP	5	63802280/63802412/63802272	3	cg11359210/cg15426956/cg17318297	0.201072582	0.295581207	0.449753811	0.248681229	1.05E-05	6.87E-05
TSS200	RICS	11	129062277	1	cg06466839	0.430118655	0.356806583	0.269111209	-0.161007446	2.98E-11	1.68E-09
TSS200	RILP	17	1553585/1553415/1553453/1553591/1553502/1553484	6	cg03044281/cg05548425/cg08625564/cg19502936/cg23488607/cg24096415	0.503475452	0.416796007	0.339952787	-0.163522665	6.69E-10	1.64E-08
TSS200	RIPPLY2	6	84562908/84562888/84562788/84562930/84562892/84562928	6	cg00270497/cg00575005/cg01965173/cg08154348/cg16528511/cg23215729	0.250136248	0.263950537	0.41576834	0.165632092	0.001667121	0.006596948
TSS200	RLBP1	15	89765061/89765045/89764999	3	cg00376910/cg08746496/cg20989926	0.622213552	0.453167837	0.37248542	-0.249728132	7.58E-15	1.49E-11
TSS200	RNASE1	14	21270575/21271148	2	cg05153345/cg12940993	0.77605042	0.640709322	0.522556386	-0.253494034	3.10E-08	3.98E-07
TSS200	RNASE10	14	20978612/20978481	2	cg01826623/cg03795378	0.836169018	0.752462458	0.678460773	-0.157708245	1.38E-08	2.00E-07
TSS200	RNF126P1	17	55122828/55122747	2	cg02486253/cg12636325	0.594525843	0.654717533	0.7485369	0.154011058	1.48E-08	2.10E-07
TSS200	RNF165	18	43914047	1	cg09730015	0.19832376	0.236244739	0.403254159	0.204930399	4.04E-05	0.000229336
TSS200	RNF222	17	8301192/8301166/8301183	3	cg00011924/cg03163302/cg10218000	0.574430085	0.4375991	0.360629968	-0.213800117	1.58E-09	3.24E-08
TSS200	RPGRIP1	14	21756040	1	cg18996099	0.766999555	0.680127089	0.584770718	-0.182228837	8.25E-07	7.15E-06
TSS200	RPH3A	12	113229534/113229392/113229362/113229374/113229465	5	cg09384610/cg16403344/cg18629535/cg19712198/cg23596620	0.292809903	0.359079111	0.489106429	0.196296525	9.37E-06	6.21E-05
TSS200	RPL31P11	1	161655056/161655124	2	cg11174769/cg20491963	0.8127583	0.753215047	0.65513652	-0.15762178	1.88E-06	1.50E-05
TSS200	RPL39L	3	186857284/186857318/186857397/186857389	4	cg05882938/cg06190677/cg14370628/cg24524308	0.146080201	0.219501346	0.340588902	0.194508701	0.000520674	0.002288963

TSS200	RPS6KL1	14	75389222/75389280/75389200	3	cg02858826/cg03394422/cg18664866	0.766297618	0.672074637	0.574574129	-0.19172349	3.79E-09	6.79E-08
TSS200	RSPO2	8	109095975/109095951/109096064/109096007/109095991/109095918	6	cg04549460/cg13177747/cg13700897/cg16845394/cg22600043/cg24807106	0.262442026	0.310940995	0.41345383	0.151011805	4.11E-07	3.80E-06
TSS200	RSPO3	6	127440000	1	cg19365062	0.081379366	0.202619072	0.399630693	0.318251327	2.08E-05	0.000126871
TSS200	RXFP3	5	33936402/33936307/33936362/33936322/33936462/33936292	6	cg12238343/cg13349651/cg13912115/cg15408073/cg26390889/cg26986911	0.236808687	0.332395803	0.460501079	0.223692393	6.16E-05	0.000333522
TSS200	RXRG	1	165414583/165414611/165414579/165414514/165414491/165414544	6	cg01913568/cg08623787/cg11717564/cg13901501/cg17885806/cg22675486	0.212607257	0.287671093	0.400540364	0.187933106	1.28E-05	8.18E-05
TSS200	RYR1	19	38924182/38924282/38924185/38924319/38924330	5	cg02026160/cg05304622/cg11288278/cg11297107/cg27049517	0.134705266	0.165874668	0.287582128	0.152876862	0.000293771	0.001368128
TSS200	RYR3	15	33603026/33603070	2	cg24632756/cg25405123	0.182847468	0.237028529	0.349497715	0.166650247	8.11E-07	7.04E-06
TSS200	S100A14	1	153588927	1	cg13098855	0.48401158	0.394778033	0.323388595	-0.160622985	8.97E-10	2.07E-08
TSS200	S100P	4	6695475/6695380/6695377/6695464	4	cg07210669/cg14140379/cg14900031/cg25083732	0.668117435	0.4932814	0.366300711	-0.301816724	1.17E-12	2.37E-10
TSS200	S1PR4	19	3178759/3178742	2	cg20583073/cg27121758	0.58912087	0.487021958	0.40517907	-0.1839418	2.94E-08	3.79E-07
TSS200	SALL1	16	51185346/51184583/51185236/51184562	4	cg00310215/cg02864757/cg06274671/cg08526074	0.277403258	0.371629721	0.502743952	0.225340694	1.21E-07	1.30E-06
TSS200	SCEL	13	78109801	1	cg21063899	0.529078975	0.419898372	0.302566886	-0.226512089	1.66E-12	2.84E-10
TSS200	SCGB1C1	11	193044/193021/192996/192897/193062/193016	6	cg02378673/cg03960562/cg17345964/cg18793661/cg20297976/cg22280333	0.602697462	0.490036682	0.398992062	-0.2037054	1.21E-12	2.38E-10
TSS200	SCGB1D1	11	61957695	1	cg01772980	0.57206801	0.45797545	0.318077836	-0.253990174	8.94E-13	2.12E-10
TSS200	SCNN1D	1	1217511	1	cg00825734	0.446074995	0.360330078	0.294948259	-0.151126736	5.99E-11	2.72E-09
TSS200	SDC2	8	97505843/97505764/97505788/97505818/97505868	5	cg08979737/cg13096260/cg18719750/cg24732574/cg25070637	0.138730784	0.195537039	0.319846831	0.181116047	0.000297231	0.001382259

TSS200	SDCBP2	20	1310000/1294311/1294361/1294376/1294313	5	cg08443357/cg15645491/cg21737976/cg21871261/cg23100411	0.399876965	0.30721735	0.232282209	-0.167594756	6.43E-11	2.84E-09
TSS200	SDR16C5	8	57233391/57233373/57233314/57233269	4	cg09038599/cg10700380/cg16683508/cg21488289	0.568808697	0.448989006	0.348119482	-0.220689215	1.20E-10	4.38E-09
TSS200	SEC16B	1	177939225	1	cg24974611	0.506462615	0.418321811	0.352042559	-0.154420056	3.15E-10	9.11E-09
TSS200	SEC31B	10	102279703/102279697/102279758/102279690/102279694/102279791	6	cg00730561/cg04611437/cg04835284/cg23725321/cg26458072/cg26705561	0.481888908	0.58023177	0.68200556	0.200116652	8.08E-10	1.90E-08
TSS200	SELENBP1	1	151345248/151345181/151345173	3	cg07680533/cg24486037/cg26065909	0.528834983	0.443050653	0.356088245	-0.172746737	4.66E-08	5.66E-07
TSS200	SEMA4A	1	156123352	1	cg21858764	0.538403395	0.428945944	0.365717314	-0.172686081	4.01E-10	1.09E-08
TSS200	12-Sep	16	4838559	1	cg11353547	0.434620945	0.343855678	0.262169559	-0.172451386	1.43E-10	4.99E-09
TSS200	SERINC4	15	44092345/44092314/44092398	3	cg08278937/cg13793048/cg26552774	0.771587202	0.705422983	0.594831842	-0.176755359	5.23E-07	4.73E-06
TSS200	SERPINA10	14	94759767/94759646/94759743	3	cg19937039/cg20599748/cg21246665	0.42591855	0.321990706	0.254552289	-0.171366261	5.28E-12	5.55E-10
TSS200	SERPINA11	14	94919295	1	cg04461867	0.554117995	0.422035333	0.290875923	-0.263242072	1.48E-11	1.11E-09
TSS200	SERPINA4	14	95027692	1	cg05186455	0.526946425	0.421184128	0.343107968	-0.183838457	1.64E-09	3.34E-08
TSS200	SERPINA6	14	94789853/94789777	2	cg09147827/cg09318122	0.398345665	0.3064748	0.243027064	-0.155318601	3.47E-09	6.34E-08
TSS200	SERPINB12	18	61223377	1	cg00005215	0.689158025	0.493514389	0.371855277	-0.317302748	5.33E-11	2.53E-09
TSS200	SERPINB3	18	61329252	1	cg03404572	0.501890355	0.411714367	0.315621232	-0.186269123	3.81E-08	4.77E-07
TSS200	SERPINB5	18	61143961/61143945/61143993	3	cg11862144/cg19022006/cg26753302	0.582697615	0.421249333	0.317292064	-0.265405551	2.04E-09	3.98E-08
TSS200	SFMBT2	10	7453455/7453507/7453471/7453594/7453511/7453452	6	cg02866454/cg07423571/cg16818993/cg23056729/cg25433188/cg26978668	0.167705009	0.31664858	0.443756978	0.276051969	1.10E-06	9.28E-06
TSS200	SFRP1	8	41167087/41167113/41167109/41167107/41166990	5	cg01495122/cg10406295/cg17816908/cg21517947/cg24319902	0.402568131	0.483173198	0.584995039	0.182426908	2.39E-08	3.19E-07

TSS200	SFRP2	4	154710353/154710418/154710371/154710399/154710421/154710425/154710373	7	cg03202804/cg05164933/cg11354906/cg14330641/cg23121156/cg23292160/cg25775322	0.278165405	0.374927197	0.516227677	0.238062272	5.34E-06	3.74E-05
TSS200	SFRS13B	6	89827915/89827912	2	cg00673557/cg23356850	0.153346168	0.240254519	0.380549118	0.22720295	5.44E-05	0.000298278
TSS200	SFTA3	14	36983146/36983171/36983129	3	cg10385303/cg16478719/cg23542968	0.240015405	0.3144001	0.406191697	0.166176292	3.63E-05	0.000207686
TSS200	SFTPA1	10	81370678	1	cg19183317	0.476576135	0.409494678	0.296817977	-0.179758158	2.63E-08	3.44E-07
TSS200	SFTPD	10	81708991	1	cg03600318	0.47173821	0.382253356	0.303419691	-0.168318519	3.16E-10	9.11E-09
TSS200	SGCZ	8	15095914	1	cg26247737	0.244888445	0.368719333	0.477364418	0.232475973	1.98E-06	1.57E-05
TSS200	SH3GL3	15	84116018/84115895/84115932/84115897/84115969	5	cg01667646/cg04779631/cg11797092/cg26215967/cg27021357	0.186091531	0.245437704	0.396256235	0.210164704	0.0001259	0.000640014
TSS200	SHC3	9	91793721	1	cg26493353	0.139038882	0.243951039	0.390867054	0.251828172	0.000103698	0.000536661
TSS200	SHISA2	13	26625273/26625307/26625396/26625317/26625325/26625359	6	cg00099976/cg00109356/cg03099431/cg08386692/cg09668400/cg17830985	0.355392107	0.458862792	0.58670131	0.231309203	1.06E-08	1.60E-07
TSS200	SHISA9	16	12995292/12995300/12995296	3	cg02860732/cg03057083/cg04342955	0.259598656	0.369636269	0.595409569	0.335810913	3.26E-06	2.43E-05
TSS200	SIAH3	13	46425999/46425866/46425929/46425904/46426026/46425874	6	cg02017534/cg08018585/cg12979844/cg14269973/cg25658983/cg26667946	0.431872835	0.507638368	0.61389035	0.182017515	5.38E-05	0.000295599
TSS200	SIM1	6	100911701/100911746/100911687/100911727/100911709/100911744	6	cg04927931/cg08133486/cg11471772/cg11891393/cg21063722/cg22478310	0.31500923	0.3846802	0.592480535	0.277471305	3.30E-08	4.20E-07
TSS200	SIRPB1	20	1600705	1	cg09577651	0.402374255	0.300649072	0.251513979	-0.150860276	1.26E-06	1.05E-05
TSS200	SIRPD	20	1538366/1538353	2	cg01247127/cg11295724	0.671586475	0.492569622	0.370846718	-0.300739757	2.34E-14	2.83E-11
TSS200	SIX6	14	60975811/60975837/60975846/60975841/60975912	5	cg08132837/cg14611174/cg18639233/cg20576153/cg20585530	0.270983193	0.353799089	0.500806855	0.229823662	3.34E-08	4.24E-07
TSS200	SLAMF7	1	160708987/160708990	2	cg04244970/cg07837085	0.579450968	0.44451915	0.339808134	-0.239642833	2.68E-08	3.50E-07
TSS200	SLC13A5	17	6616799/6616883/6616867	3	cg08049640/cg12146546/cg13781853	0.141488693	0.245602827	0.417813991	0.276325299	0.000448817	0.002001528

TSS200	SLC16A4	1	110933767	1	cg00961640	0.6624791	0.489536761	0.375002668	-0.287476432	9.29E-13	2.12E-10
TSS200	SLC18A2	10	119000638	1	cg15173134	0.378897845	0.376248644	0.554818941	0.175921096	0.000199022	0.000966251
TSS200	SLC18A3	10	50818153/50818299	2	cg02728050/cg11389172	0.289713635	0.359840561	0.472400116	0.182686481	3.02E-05	0.000176143
TSS200	SLC22A17	14	23821596/23821570/23822265	3	cg15476528/cg23411440/cg23464698	0.17715308	0.178381296	0.343550598	0.166397518	0.000367977	0.001675608
TSS200	SLC22A8	11	62783482/62783394/62783361/62783386	4	cg08288703/cg10068408/cg18305416/cg22907415	0.548348963	0.487710039	0.395285835	-0.153063127	6.10E-10	1.52E-08
TSS200	SLC22A9	11	63137125/63137152	2	cg07751764/cg23683201	0.571637175	0.474130622	0.370182582	-0.201454593	5.55E-10	1.42E-08
TSS200	SLC23A3	2	220034947	1	cg27264181	0.6316455	0.514063856	0.407961059	-0.223684441	3.80E-12	4.74E-10
TSS200	SLC24A4	14	92790097/92788914/92790072	3	cg01802258/cg14512346/cg24766334	0.349080048	0.436036108	0.505370111	0.156290064	1.24E-05	7.95E-05
TSS200	SLC25A26	3	66119234/66119094/66119154	3	cg02784780/cg07847658/cg15364450	0.763605135	0.637503167	0.509974241	-0.253630894	7.40E-09	1.18E-07
TSS200	SLC25A41	19	6433825/6433858	2	cg13742513/cg26851500	0.597148808	0.465674269	0.365551309	-0.231597498	3.00E-10	8.76E-09
TSS200	SLC26A1	4	987391/987303	2	cg15138543/cg21616051	0.564887935	0.427673722	0.334574214	-0.230313721	3.69E-11	1.92E-09
TSS200	SLC26A9	1	205897545/205912646	2	cg02117416/cg09852221	0.747889415	0.690168417	0.584144264	-0.163745151	1.73E-06	1.39E-05
TSS200	SLC27A5	19	59023484/59023537	2	cg16278661/cg18495710	0.498341008	0.403756028	0.332026557	-0.166314451	8.90E-11	3.57E-09
TSS200	SLC27A6	5	128301136/128301156/128301185/128301083/128301146	5	cg07421806/cg09538995/cg13985726/cg14441976/cg15899948	0.229054425	0.288089102	0.458344828	0.229290403	2.95E-05	0.000172482
TSS200	SLC30A8	8	118147200/118147163/118147191	3	cg07061355/cg07459489/cg09439192	0.753967547	0.688790974	0.591915073	-0.162052474	5.28E-08	6.29E-07
TSS200	SLC32A1	20	37353032/37353096/37353037/37353035	4	cg07033372/cg10615414/cg12180703/cg25307168	0.332205109	0.399512449	0.502594101	0.170388992	1.39E-10	4.91E-09
TSS200	SLC34A1	5	176811257/176811305/176811239	3	cg06501790/cg14819088/cg18126247	0.712696443	0.659663114	0.528137007	-0.184559436	1.07E-08	1.60E-07
TSS200	SLC35F4	14	58063811/58063727	2	cg04913974/cg13998988	0.554431965	0.460712758	0.343836684	-0.210595281	8.85E-11	3.57E-09
TSS200	SLC36A3	5	150683530	1	cg01678321	0.799630055	0.744428944	0.595900532	-0.203729523	4.84E-06	3.44E-05

TSS200	SLC39A4	8	145641991/145642295/145642456/145641996/145642114/145642024/145642291/145642421/145642018	9	cg00065570/cg04803798/cg05574324/cg07933378/cg08969922/cg09155025/cg11800672/cg12503742/cg23697467	0.541852177	0.45510863	0.375549739	-0.166302438	7.94E-11	3.31E-09
TSS200	SLC43A3	11	57195187/57194562	2	cg00354381/cg07326438	0.074301857	0.106917992	0.286199424	0.211897568	0.000713689	0.003047432
TSS200	SLC44A4	6	31846970/31846956/31846996/31846899/31846924/31847009	6	cg03045620/cg04567302/cg07363637/cg21236501/cg24529722/cg24707219	0.504383783	0.4257982	0.35223017	-0.152153612	1.58E-11	1.16E-09
TSS200	SLC45A4	8	142238752/142238770/142238691	3	cg01081737/cg07769015/cg15586392	0.457864923	0.357061217	0.275025271	-0.182839652	2.57E-11	1.54E-09
TSS200	SLC5A11	16	24857497	1	cg00339695	0.489326095	0.384489094	0.322641955	-0.16668414	9.21E-10	2.11E-08
TSS200	SLC5A4	22	32651345	1	cg21578906	0.51292286	0.4029516	0.323956227	-0.188966633	9.21E-13	2.12E-10
TSS200	SLC5A7	2	108602906/108602860/108602931/108602937/108602979	5	cg05311412/cg11355135/cg14613271/cg18771173/cg26001902	0.412205918	0.439223517	0.638806236	0.226600319	8.15E-06	5.48E-05
TSS200	SLC6A1	3	11034279/11034311/11034345/11034281	4	cg11021744/cg15445554/cg16164276/cg23405575	0.262292329	0.307416941	0.515257988	0.252965659	1.40E-05	8.89E-05
TSS200	SLC6A11	3	10857798/10857795/10857719/10857717/10857800	5	cg09022422/cg10527010/cg21123160/cg22331862/cg26836233	0.441025074	0.509574136	0.611106668	0.170081594	1.03E-12	2.19E-10
TSS200	SLC6A15	12	85306797/85306741/85306648	3	cg14510812/cg17222500/cg21702506	0.26073564	0.350206439	0.474285445	0.213549805	8.44E-06	5.66E-05
TSS200	SLC6A2	16	55690378/55690443/55690381	3	cg03226000/cg09321400/cg09774787	0.439240143	0.578017011	0.686536679	0.247296535	4.01E-08	4.98E-07
TSS200	SLC6A3	5	1445561/1445593/1445549/1445567	4	cg04210284/cg05030481/cg12882697/cg27037018	0.250250824	0.253240823	0.419007345	0.168756522	0.00056613	0.002464745
TSS200	SLC6A5	11	20620830/20620854/20620824/20620914/20620920	5	cg04642741/cg06804921/cg08530317/cg16635352/cg20551181	0.303545038	0.408895462	0.503909556	0.200364519	9.40E-08	1.04E-06
TSS200	SLC7A13	8	87242630	1	cg14588399	0.563304845	0.42473725	0.384666373	-0.178638472	5.32E-09	8.93E-08

TSS200	SLC8A3	14	70655871/70655862/70655920/70655805/70655845/70655803	6	cg08265644/cg14174099/cg14358879/cg21512124/cg23609571/cg23702412	0.065894933	0.154513577	0.27919208	0.213297148	9.38E-05	0.000490236
TSS200	SLCO4C1	5	101632341/101632286/101632321/101632372/101632327/101632310/101632314	7	cg04621020/cg04633600/cg06480736/cg11009817/cg11267955/cg19788741/cg22149516	0.094948704	0.147271233	0.314547799	0.219599095	0.000267481	0.001258324
TSS200	SLFN11	17	33700757/33700801/33700817/33700747/33700759/33700745	6	cg01348733/cg05224998/cg10911913/cg14380270/cg18608369/cg26573518	0.127703475	0.233569838	0.330782992	0.203079518	2.25E-06	1.76E-05
TSS200	SLFN13	17	33775963/33775933/33775917/33775860/33775961/33775952	6	cg04354393/cg04862556/cg08151705/cg12788037/cg13443575/cg16563370	0.341152953	0.391982763	0.494026305	0.152873353	9.88E-07	8.44E-06
TSS200	SLFNL1	1	41487437/41487408	2	cg22005565/cg22435441	0.77349496	0.682462194	0.590677959	-0.182817001	7.69E-08	8.73E-07
TSS200	SLIT2	4	20255082/20255061/20255187	3	cg13078140/cg13281139/cg15469350	0.337305058	0.451698381	0.642086455	0.304781397	4.29E-10	1.16E-08
TSS200	SLIT3	5	168728193/168728213/168728270/168728149/168728236	5	cg02403292/cg14658804/cg15637465/cg15927720/cg26492514	0.339987309	0.422433059	0.582153003	0.242165694	2.47E-06	1.91E-05
TSS200	SLITRK1	13	84456722/84456547/84456681	3	cg13988851/cg14246335/cg25938646	0.342205445	0.423133528	0.528081884	0.185876439	4.63E-08	5.63E-07
TSS200	SLITRK5	13	88324711	1	cg17967577	0.27742316	0.270804299	0.542421245	0.264998085	2.61E-06	2.00E-05
TSS200	SLN	11	107582818/107582876/107582804/107582884	4	cg11117131/cg16476427/cg17971003/cg24307368	0.472451895	0.382161665	0.29869123	-0.173760665	4.81E-12	5.24E-10
TSS200	SLURP1	8	143823917/143824001/143823898	3	cg06565975/cg07441143/cg13411229	0.536085418	0.446243831	0.361320815	-0.174764603	9.25E-12	7.88E-10
TSS200	SMTNL1	11	57308952/57308888	2	cg17039830/cg26445999	0.822588333	0.739956269	0.649863727	-0.172724605	4.24E-07	3.91E-06
TSS200	SNAP91	6	84419244/84419202/84419189/84419234/84419242/84419237	6	cg09227138/cg17767285/cg18213472/cg19671533/cg20631014/cg24842733	0.291554167	0.335079723	0.497929821	0.206375655	1.79E-06	1.43E-05
TSS200	SNORA14A	7	75573071	1	cg10902139	0.850573725	0.784973744	0.687199564	-0.163374161	0.000107963	0.000557819
TSS200	SNORA8	11	93465688	1	cg15924285	0.45951232	0.357257767	0.288638668	-0.170873652	5.11E-10	1.33E-08
TSS200	SNORA80B	2	10587052	1	cg15075241	0.66628874	0.490802811	0.352196073	-0.314092667	7.97E-14	5.71E-11

TSS200	SNORD113-1	14	101390989	1	cg07010373	0.521743515	0.41395475	0.333578095	-0.18816542	7.84E-10	1.86E-08
TSS200	SNORD113-4	14	101402822	1	cg04784410	0.323328705	0.211694711	0.149351969	-0.173976736	1.93E-13	9.53E-11
TSS200	SNORD113-6	14	101405693/101405755/101405842	3	cg04262465/cg09293488/cg25045972	0.725350198	0.627180839	0.534762498	-0.1905877	6.76E-08	7.84E-07
TSS200	SNORD114-1	14	101416128	1	cg20052865	0.4111624	0.274981028	0.194244682	-0.216917718	5.65E-13	1.75E-10
TSS200	SNORD114-10	14	101433314	1	cg10931901	0.734299255	0.61662955	0.476671109	-0.257628146	1.88E-08	2.58E-07
TSS200	SNORD114-25	14	101452368	1	cg10472263	0.581028335	0.443832711	0.374705109	-0.206323226	1.97E-12	3.21E-10
TSS200	SNORD115-37	15	25483088	1	cg17208360	0.649078055	0.557369589	0.416258845	-0.23281921	8.27E-08	9.31E-07
TSS200	SNORD115-40	15	25488753	1	cg00361898	0.72299292	0.607727211	0.523623586	-0.199369334	1.34E-07	1.41E-06
TSS200	SNORD116-11	15	25320886	1	cg04524239	0.749921535	0.72353905	0.594504673	-0.155416862	9.00E-06	6.00E-05
TSS200	SNORD116-15	15	25326263	1	cg20385284	0.743657135	0.674473917	0.563627091	-0.180030044	2.13E-06	1.68E-05
TSS200	SNORD116-16	15	25327713	1	cg17198397	0.63367499	0.559752922	0.464799609	-0.168875381	3.45E-06	2.55E-05
TSS200	SNORD116-23	15	25336770	1	cg17727579	0.66721669	0.580290561	0.464517673	-0.202699017	3.61E-07	3.40E-06
TSS200	SNORD116-4	15	25304609/25304625	2	cg06502456/cg11063170	0.70877131	0.634176611	0.535065177	-0.173706133	1.39E-06	1.14E-05
TSS200	SNORD16	15	66795345	1	cg00090787	0.54083598	0.439707511	0.363335564	-0.177500416	3.44E-07	3.26E-06
TSS200	SNX32	11	65601332/65601267/65601265/65601301/65601385/65601328	6	cg11198128/cg13708218/cg15890274/cg16624692/cg17318632/cg19018097	0.079105997	0.103562392	0.2491938	0.170087803	0.001369406	0.005512933
TSS200	SORCS1	10	108924366/108924398	2	cg24403845/cg26554592	0.190141066	0.282526276	0.41691187	0.226770805	0.000128659	0.000651726
TSS200	SORCS3	10	106400686/106400824/106400702/106400667	4	cg03958798/cg08495770/cg10601616/cg20482521	0.309774398	0.482957725	0.657551995	0.347777597	1.78E-09	3.57E-08
TSS200	SOST	17	41836261	1	cg01898628	0.64740774	0.538135711	0.451258136	-0.196149604	6.65E-09	1.07E-07
TSS200	SOX14	3	137483503/137483555/137483506/137483479/137483543	5	cg04374393/cg08073312/cg10343742/cg16428251/cg27505273	0.317285466	0.352387063	0.473015898	0.155730432	0.000310141	0.001435881
TSS200	SOX15	17	7493572	1	cg12615880	0.551596445	0.4232491	0.343736141	-0.207860304	2.54E-11	1.54E-09
TSS200	SOX17	8	55370423/55370429/55370434/55370334/55370407/55370336	6	cg04672706/cg11214140/cg15186181/cg15710198/cg24891539/cg24928391	0.26956427	0.335158679	0.467490402	0.197926132	1.63E-05	0.000102144
TSS200	SOX21	13	95364514/95364569/95364586/95364510	4	cg01212887/cg02736420/cg16201674/cg23111830	0.413740165	0.420654	0.611093993	0.197353828	4.46E-05	0.00025005

TSS200	SOX5	12	24715484/24715564/24715491/24715508/24715478/24102830/24715538/24102807/24102832	9	cg03625010/cg04389422/cg04925841/cg06867829/cg08972130/cg15990409/cg17853504/cg24237081/cg25925006	0.153892613	0.199314207	0.312246283	0.15835367	0.00201002	0.007799213
TSS200	SOX7	8	10588040	1	cg07233097	0.086721234	0.122383937	0.247570358	0.160849124	0.005580181	0.019394726
TSS200	SP7	12	53729629/53729659	2	cg07937786/cg22285914	0.553234633	0.492553206	0.402101323	-0.15113331	1.33E-06	1.10E-05
TSS200	SPARCL1	4	88450824	1	cg15552249	0.490304185	0.440458989	0.314260523	-0.176043662	1.69E-05	0.000105556
TSS200	SPATA19	11	133715511	1	cg26959392	0.657002815	0.581037083	0.475344023	-0.181658792	8.09E-06	5.44E-05
TSS200	SPATS1	6	44310324/44310350/44310345/44310331/44310228/44310257	6	cg03400828/cg13077262/cg18959554/cg22674613/cg22970435/cg26145959	0.214740013	0.256383987	0.384852793	0.170112779	3.08E-05	0.000179221
TSS200	SPDEF	6	34524290/34524097/34524278/34524095	4	cg05724257/cg10159596/cg17240454/cg26578149	0.528432705	0.423306575	0.353072734	-0.175359971	4.39E-11	2.18E-09
TSS200	SPDYE4	17	8661887/8661927/8661909/8662011/8662002	5	cg11927584/cg19963471/cg21610999/cg22664250/cg25245118	0.739558195	0.680354786	0.588808109	-0.150750086	3.09E-12	4.37E-10
TSS200	SPEG	2	220299584/220299653/220299643/220299604/220299659/220299672	6	cg07820064/cg12213062/cg15634877/cg18984724/cg23065715/cg24207529	0.469303025	0.54779978	0.627414462	0.158111437	1.70E-06	1.37E-05
TSS200	SPERT	13	46276342	1	cg05014103	0.54399399	0.393818894	0.3123524	-0.23164159	1.63E-13	8.86E-11
TSS200	SPHKAP	2	229046406/229046515	2	cg06092815/cg10959353	0.426582138	0.506556308	0.66497103	0.238388892	1.10E-10	4.16E-09
TSS200	SPINK7	5	147691898	1	cg27488807	0.44029912	0.340496206	0.263366845	-0.176932275	2.98E-10	8.72E-09
TSS200	SPOCK3	4	168155888/168155853	2	cg05158197/cg22255664	0.134234852	0.149327418	0.311082549	0.176847697	2.99E-05	0.000174442
TSS200	SPON1	11	13983893/13983793/13983784/13983818/13983851/13983790	6	cg10153349/cg11028624/cg12085698/cg22805485/cg24258347/cg25486824	0.609389123	0.536292551	0.444381265	-0.165007858	1.06E-09	2.35E-08
TSS200	SPRR2C	1	153114002/153114115	2	cg00535683/cg25212571	0.68623381	0.537395661	0.421915818	-0.264317992	9.32E-09	1.43E-07
TSS200	SPRR2G	1	153123593	1	cg11267802	0.47503494	0.346106867	0.21350869	-0.26152625	2.69E-13	1.21E-10
TSS200	SPSB4	3	140770588/140770608/140770617/140770549/140770683/140770599	6	cg04353095/cg05258261/cg15562912/cg19443257/cg20979852/cg27515369	0.144978153	0.212109015	0.376636693	0.23165854	0.000113819	0.000584061

TSS200	SPTBN5	15	42186377/42186473	2	cg04717847/cg10715147	0.87845406	0.728555511	0.703730018	-0.174724042	6.17E-06	4.28E-05
TSS200	SRC	20	35972924/35972957/35973064/35973042	4	cg13052876/cg18858144/cg19757422/cg24055525	0.612199035	0.469545556	0.344896282	-0.267302753	3.17E-12	4.39E-10
TSS200	SRD5A2	2	31806234/31806042/31806183	3	cg08894761/cg18529845/cg26638505	0.497335412	0.607666044	0.712515223	0.215179811	1.89E-10	6.13E-09
TSS200	SST	3	187388281/187388225	2	cg07120369/cg13206017	0.257519023	0.362481681	0.444945477	0.187426455	4.60E-06	3.29E-05
TSS200	SSTR2	17	71161157	1	cg22277994	0.282628835	0.36149265	0.522970159	0.240341324	3.55E-07	3.35E-06
TSS200	SSTR4	20	23015932/23015899/23015906/23015908/23015936	5	cg07676859/cg14631053/cg17586860/cg18197392/cg22534145	0.491664125	0.570694337	0.663223045	0.17155892	2.32E-10	7.10E-09
TSS200	ST6GALNAC1	17	74639894/74640078/74639928	3	cg00440980/cg19829001/cg26550194	0.409592447	0.294223787	0.23215168	-0.177440766	4.71E-12	5.24E-10
TSS200	ST6GALNAC5	1	77332991/77333159/77333138/77332993/77333122/77333074	6	cg09511846/cg13463054/cg13823136/cg15100100/cg16966815/cg27519691	0.192329344	0.327340075	0.47973563	0.287406286	7.55E-08	8.59E-07
TSS200	ST8SIA3	18	55019621/55019669/55019708/55019711	4	cg09036612/cg16902385/cg22070406/cg24811864	0.331821554	0.419644847	0.527504034	0.19568248	2.04E-08	2.77E-07
TSS200	ST8SIA4	5	100239017/100238977/100238983/100239050/100239071/100239022	6	cg01922936/cg03975797/cg04380513/cg12665504/cg16673106/cg26337020	0.091095768	0.164102127	0.294174757	0.203078989	2.63E-05	0.000156345
TSS200	STAC	3	36422059/36422093/36422061	3	cg04273811/cg24202123/cg26843074	0.370310257	0.450839685	0.587633052	0.217322795	1.43E-08	2.04E-07
TSS200	STAG3	7	99775422/99775508/99775521/99775443/99775532/99775425	6	cg00048759/cg04778012/cg10084644/cg13210467/cg15140703/cg18691434	0.588213756	0.663371457	0.74003122	0.151817464	1.07E-09	2.37E-08
TSS200	STATH	4	70861449	1	cg00436282	0.82924721	0.755521022	0.633677132	-0.195570078	2.30E-05	0.000138384
TSS200	STK32B	4	5053504/5053341/5053496	3	cg08323075/cg10351287/cg23300732	0.364420718	0.451671887	0.586809124	0.222388406	1.57E-07	1.63E-06
TSS200	STK33	11	8615694/8615685/8615575/8615506/8615579/8615675	6	cg00393798/cg00450824/cg02541031/cg08788717/cg12675870/cg20771178	0.066160605	0.18140213	0.263018018	0.196857413	0.001309253	0.005299183
TSS200	STMN4	8	27115956	1	cg11688949	0.409378225	0.305232394	0.230441918	-0.178936307	6.06E-12	6.03E-10

TSS200	STON1-GTF2A1L	2	48796151/48795994	2	cg06092244/cg14965639	0.640535798	0.529495028	0.442253284	-0.198282513	2.16E-07	2.14E-06
TSS200	STOX2	4	184826314/184826423/184826443/184826421/184826324/184826479	6	cg07089892/cg12843518/cg20026798/cg23649435/cg25669309/cg25882830	0.195849138	0.203230864	0.354199004	0.158349866	0.003750651	0.013650264
TSS200	STRA6	15	74495354/74495603/74495657/74501549/74495637/74495384	6	cg00075967/cg13888509/cg18640660/cg21631918/cg22672067/cg26774156	0.571048283	0.466345474	0.368150524	-0.202897759	2.92E-13	1.25E-10
TSS200	STX19	3	93747528	1	cg19847038	0.57732419	0.473473	0.378321095	-0.199003095	6.44E-11	2.84E-09
TSS200	STX1B	16	31021933/31021905	2	cg02043600/cg03793804	0.104529743	0.202669005	0.32675116	0.222221418	0.000298404	0.001386836
TSS200	SULT4A1	22	44258572/44258574	2	cg15471953/cg21925310	0.3316428	0.379083708	0.511421812	0.179779012	0.000908978	0.003801028
TSS200	SUNC1	7	48068758/48068728	2	cg03609427/cg07298482	0.699570785	0.555518042	0.383530455	-0.31604033	3.60E-13	1.38E-10
TSS200	SUSD5	3	33260743/33260732/33260752	3	cg02239862/cg04545136/cg13601427	0.232784649	0.387156452	0.541407395	0.308622747	2.44E-09	4.63E-08
TSS200	SVEP1	9	113342326	1	cg01503348	0.175970525	0.200032697	0.358150679	0.182180154	0.001707494	0.006744858
TSS200	SYNPR	3	63263828/63428587/63263832/63263824/63263752	5	cg04785972/cg05401575/cg06314761/cg09462808/cg22189386	0.361922459	0.4180359	0.54385938	0.181936921	5.07E-06	3.59E-05
TSS200	SYT14	1	210111403	1	cg04932544	0.187407365	0.28068998	0.374528645	0.187121281	2.37E-07	2.34E-06
TSS200	SYT6	1	114696671/114696641	2	cg01567634/cg21920775	0.229697972	0.279092855	0.456830873	0.227132901	3.10E-07	2.97E-06
TSS200	SYT8	11	1855606/1855561/1855502/1855660/1855665	5	cg02294791/cg09575189/cg16657886/cg21544658/cg24331162	0.570932575	0.459229006	0.382027417	-0.188905158	2.12E-11	1.38E-09
TSS200	SYT9	11	7273154/7273049/7273046/7273148	4	cg02269161/cg03226737/cg16437728/cg18560328	0.265101867	0.336651784	0.4938824	0.228780533	0.000112325	0.000577331
TSS200	SYTL3	6	159070959/159070994/159071008	3	cg02829601/cg04205041/cg07265541	0.399519952	0.262627531	0.218657572	-0.18086238	2.66E-07	2.59E-06
TSS200	T	6	166582159/166582188/166582310/166582201/166582197/166582206	6	cg02149708/cg05655837/cg06073449/cg14638883/cg17188046/cg19675288	0.180627035	0.24209568	0.342000668	0.161373633	0.013206939	0.041593812
TSS200	T-SP1	8	10383071	1	cg00014260	0.5466238	0.424706289	0.296280595	-0.250343205	3.17E-12	4.39E-10

TSS200	TAC1	7	97361241/97361148/97361201/97361114/97361244/97361252	6	cg01287975/cg02539083/cg07550362/cg09236284/cg11873482/cg17437939	0.312087028	0.366139039	0.500784109	0.188697082	2.83E-05	0.000166307
TSS200	TACR3	4	104641146/104641033/104641124/104641072	4	cg00875511/cg04535008/cg19163049/cg24090629	0.330819935	0.381400272	0.516370473	0.185550538	3.94E-06	2.86E-05
TSS200	TAS1R2	1	19186245/19186260	2	cg07947459/cg20678988	0.757506495	0.608475239	0.437779341	-0.319727154	7.48E-13	1.97E-10
TSS200	TAS2R40	7	142919080/142919141	2	cg02341670/cg22691319	0.77244144	0.626515364	0.49837982	-0.27406162	1.60E-11	1.17E-09
TSS200	TBC1D3C	17	34808203/34592075	2	cg16780784/cg23350580	0.57225207	0.476445956	0.411471289	-0.160780781	2.05E-13	9.79E-11
TSS200	TBC1D3G	17	34808203	1	cg16780784	0.58803006	0.473529456	0.396497141	-0.191532919	3.81E-12	4.74E-10
TSS200	TBX15	1	119532189/119532352/119532195/119532320	4	cg05940231/cg12664119/cg25340966/cg26272623	0.366163911	0.455126192	0.618629788	0.252465876	2.27E-10	7.00E-09
TSS200	TBX18	6	85474093/85474028/85474090/85474087	4	cg02106850/cg04515996/cg07028914/cg14068328	0.38047449	0.474290124	0.573827582	0.193353092	1.03E-06	8.77E-06
TSS200	TCL1A	14	96180653/96180648/96180576/96180643/96180598	5	cg07445246/cg13282837/cg19992633/cg24996565/cg27089768	0.409429202	0.510696565	0.602653592	0.19322439	1.35E-08	1.96E-07
TSS200	TCL6	14	96117415	1	cg07734106	0.572103825	0.465468094	0.400013436	-0.172090389	4.39E-08	5.37E-07
TSS200	TCN1	11	59634218/59634191	2	cg00187686/cg23741006	0.476723923	0.324451908	0.230647861	-0.246076061	4.37E-11	2.18E-09
TSS200	TCP10L	21	33957957/33957846	2	cg08949101/cg15713719	0.669835563	0.550494103	0.381136684	-0.288698878	4.64E-13	1.66E-10
TSS200	TCTEX1D1	1	67218090/67218080/67217950/67218135/67217984	5	cg09318375/cg11824639/cg16996144/cg17284804/cg19808205	0.224831671	0.295018495	0.415352769	0.190521098	4.36E-06	3.14E-05
TSS200	TDRG1	6	40346035/40346114/40346044	3	cg02995731/cg08415973/cg26748297	0.47634473	0.371496411	0.312484768	-0.163859962	1.62E-10	5.45E-09
TSS200	TEKT1	17	6735063/6735195/6735065/6735152/6735146/6735116	6	cg00414171/cg01990878/cg05094548/cg07989568/cg15727185/cg20354430	0.158426185	0.217603589	0.342974001	0.184547816	3.20E-06	2.39E-05
TSS200	TFEB	6	41704003	1	cg09490007	0.646303465	0.543771017	0.487805014	-0.158498451	2.13E-06	1.68E-05
TSS200	TFF3	21	43735844	1	cg21970261	0.50459979	0.394474317	0.304437227	-0.200162563	9.42E-11	3.71E-09

TSS200	TFPI2	7	93520183/93520074/93520175/93520172	4	cg14377593/cg16934178/cg22441533/cg26739865	0.108107687	0.218527103	0.371475189	0.263367502	2.62E-06	2.01E-05
TSS200	TG	8	133879048/133879162	2	cg14340089/cg18235635	0.616033933	0.538055742	0.43028305	-0.185750883	2.34E-09	4.48E-08
TSS200	TGM3	20	2276435/2276526/2276571/2276503	4	cg09561663/cg11195933/cg12761282/cg24138234	0.667619143	0.589707739	0.509047856	-0.158571287	8.56E-09	1.33E-07
TSS200	TGM4	3	44915918/44915985/44916012	3	cg09111917/cg18293158/cg21616161	0.712523233	0.590160375	0.460092709	-0.252430523	1.19E-10	4.38E-09
TSS200	TGM5	15	43559170	1	cg27496506	0.60236013	0.494114083	0.40193165	-0.20042848	3.78E-10	1.05E-08
TSS200	THBD	20	23030434/23030446/23030343/23030442	4	cg09083579/cg22152407/cg24136114/cg25397597	0.195704543	0.208160946	0.360467495	0.164762953	0.001810921	0.007094762
TSS200	THBS4	5	79330929/79330799/79330943/79330959	4	cg00795341/cg05527185/cg10784386/cg18110483	0.368587944	0.455905008	0.571257951	0.202670007	3.46E-05	0.000198859
TSS200	THEM5	1	151826205	1	cg26292918	0.46167944	0.378515772	0.298265282	-0.163414158	5.58E-12	5.68E-10
TSS200	THSD7B	2	137748455/137748366/137748460/137748382	4	cg06848148/cg07412043/cg08231096/cg15750500	0.750765971	0.672058824	0.557499951	-0.19326602	1.55E-08	2.19E-07
TSS200	TK2	16	66584358	1	cg09238666	0.4455406	0.328500167	0.29134115	-0.15419945	7.58E-09	1.20E-07
TSS200	TKTL2	4	164395093/164395149	2	cg12267786/cg16413535	0.70964706	0.626301142	0.550838093	-0.158808967	4.33E-08	5.30E-07
TSS200	TLX1	10	102891045/102890984/102890974/102890886/102890986/102891019	6	cg02567119/cg03020810/cg08908184/cg11196237/cg18964775/cg25741023	0.180028495	0.168964721	0.331625611	0.151597116	0.001485164	0.005939526
TSS200	TLX1NB	10	102891076/102891045/102890984/102890974/102890986/102891019/102891080	7	cg02096397/cg02567119/cg03020810/cg08908184/cg18964775/cg25741023/cg27272677	0.17461684	0.155415108	0.326264762	0.151647922	0.00194113	0.007559812
TSS200	TLX3	5	170736180/170736132/170736201/170736251/170736245/170736277	6	cg02676375/cg05190315/cg17833476/cg25942450/cg26517714/cg26844246	0.376615909	0.390529986	0.531840525	0.155224617	0.000209849	0.001012581
TSS200	TM4SF4	3	149192304/149192377	2	cg13235059/cg13688966	0.3812444	0.288978125	0.227290243	-0.153954157	1.03E-10	3.98E-09
TSS200	TM6SF1	15	83776271/83776269	2	cg08452658/cg11167100	0.151241291	0.376860035	0.565246468	0.414005177	6.07E-14	5.04E-11
TSS200	TMCO2	1	40713471	1	cg18124737	0.543647395	0.428685711	0.343484505	-0.20016289	8.79E-10	2.03E-08

TSS200	TMCO5A	15	38227285	1	cg03998348	0.67664391	0.564434928	0.434818514	-0.241825396	1.90E-07	1.92E-06
TSS200	TMED6	16	69385761/69385827	2	cg08627125/cg18368125	0.57885771	0.468375772	0.382623114	-0.196234596	3.09E-11	1.72E-09
TSS200	TMEFF2	2	193059717/193059645	2	cg01808545/cg06008912	0.226116285	0.341391714	0.472140502	0.246024217	3.76E-06	2.74E-05
TSS200	TMEM105	17	79304594/79304600/79304498/79304628/79304613/79304649	6	cg03841376/cg09695261/cg11758345/cg21591452/cg21735384/cg25555068	0.642696368	0.572918681	0.481116411	-0.161579957	7.08E-09	1.14E-07
TSS200	TMEM108	3	132757106/132756991/132757087/132756986/132757090/132757102	6	cg02745211/cg05075118/cg06288251/cg17385936/cg18115215/cg24198558	0.335006086	0.438743076	0.612661876	0.27765579	4.49E-10	1.20E-08
TSS200	TMEM132D	12	130388216/130388234	2	cg12122146/cg23891360	0.432046123	0.547361186	0.713278861	0.281232739	1.24E-12	2.41E-10
TSS200	TMEM132E	17	32907639/32907705/32907636/32907731	4	cg03442425/cg10451253/cg21745612/cg27213352	0.330240289	0.433951689	0.587126273	0.256885984	2.61E-07	2.55E-06
TSS200	TMEM155	4	122686432/122686453/122686493/122686456	4	cg03227184/cg04638468/cg07978472/cg08553437	0.299777159	0.451787739	0.640223931	0.340446772	2.61E-11	1.54E-09
TSS200	TMEM163	2	135476601/135476701/135476679	3	cg03556069/cg19589919/cg23057992	0.068451525	0.086850362	0.236914525	0.168463	0.000363374	0.001657518
TSS200	TMEM184A	7	1596260/1596217/1596103/1596118/1596136	5	cg00335591/cg06194808/cg09990600/cg12503394/cg21368161	0.309717886	0.21227702	0.158921911	-0.150795975	2.38E-11	1.49E-09
TSS200	TMEM196	7	19812479/19812570/19812578/19812548/19812592	5	cg11876022/cg18505401/cg21657955/cg23181844/cg27341472	0.227853645	0.284924911	0.43160987	0.203756225	4.65E-06	3.32E-05
TSS200	TMEM215	9	32783463	1	cg14614901	0.312512325	0.411833711	0.542524859	0.230012534	8.00E-09	1.26E-07
TSS200	TMEM26	10	63213391/63213385/63213396/63213339	4	cg00360414/cg06067842/cg12160258/cg18286501	0.281732012	0.289902709	0.452608389	0.170876378	0.000120966	0.000616919
TSS200	TMEM40	3	12800836/12800816	2	cg07908984/cg19384379	0.648811635	0.546956033	0.456400284	-0.192411351	3.01E-12	4.35E-10
TSS200	TMEM90A	14	74892986	1	cg06204735	0.359359645	0.4851486	0.670560677	0.311201032	4.78E-08	5.79E-07
TSS200	TMEM90B	20	24449734/24449668/24449760/24449704/24449804	5	cg02997755/cg05347927/cg22452236/cg22479299/cg25300584	0.265945449	0.362779114	0.456381273	0.190435824	3.20E-07	3.06E-06
TSS200	TMIGD1	17	28661120	1	cg22547480	0.586335145	0.474878183	0.353830964	-0.232504181	9.03E-11	3.61E-09

TSS200	TMPRSS11B	4	69111580	1	cg19510180	0.714751535	0.542103178	0.4340898	-0.280661735	5.39E-12	5.62E-10
TSS200	TMPRSS11F	4	68995759	1	cg20695587	0.56302791	0.444433178	0.349888768	-0.213139142	3.25E-12	4.45E-10
TSS200	TMPRSS4	11	117947611/117947555/117947657/117947628	4	cg03634928/cg05775918/cg25116503/cg27300950	0.47516815	0.390537706	0.311708605	-0.163459545	1.21E-10	4.41E-09
TSS200	TMPRSS5	11	113577071/113577118/113577120	3	cg15844374/cg18165914/cg25460340	0.781289865	0.614981269	0.563340545	-0.21794932	1.08E-09	2.37E-08
TSS200	TMPRSS6	22	37499741/37499859	2	cg05754122/cg16971128	0.547677188	0.451717842	0.383273891	-0.164403297	1.47E-06	1.20E-05
TSS200	TMPRSS8	16	2892766/2892769/2892912/2892809/2892782	5	cg01006802/cg03625141/cg10186456/cg10448227/cg27137258	0.511066378	0.571128017	0.66412063	0.153054252	6.37E-08	7.45E-07
TSS200	TNFRSF25	1	6526319/6526325/6526309/6526398	4	cg02064442/cg03893150/cg10539898/cg23114616	0.470808438	0.574527461	0.638723134	0.167914697	6.99E-06	4.78E-05
TSS200	TNFRSF8	1	12123313/12123339/12123308/12123262/12185845	5	cg00653960/cg01966612/cg14834850/cg19896198/cg22123711	0.268694715	0.332373553	0.452692939	0.183998225	4.83E-06	3.43E-05
TSS200	TNIP3	4	122137696	1	cg05559643	0.771613395	0.681577756	0.545177509	-0.226435886	1.21E-06	1.01E-05
TSS200	TNNT2	1	201346876	1	cg19233001	0.55244492	0.455045644	0.399744355	-0.152700565	1.51E-06	1.23E-05
TSS200	TNP1	2	217724794/217724937/217724866	3	cg19384546/cg21808287/cg24545125	0.580238463	0.456472925	0.353513675	-0.226724788	2.49E-12	3.89E-10
TSS200	TNS4	17	38657949/38657915/38657934/38657875	4	cg08074477/cg09303236/cg20468081/cg25936381	0.498679745	0.402321397	0.322580061	-0.176099684	4.35E-11	2.18E-09
TSS200	TP63	3	189349129/189507396/189349021	3	cg04489243/cg06520450/cg06720722	0.719073787	0.641785876	0.566298702	-0.152775085	5.29E-06	3.72E-05
TSS200	TREH	11	118550485/118550461/118550443	3	cg09144398/cg15474711/cg21424090	0.506665405	0.425926917	0.356635091	-0.150030314	6.35E-11	2.83E-09
TSS200	TREML3	6	41185715	1	cg05143424	0.806213305	0.763482017	0.578187677	-0.228025628	5.08E-06	3.59E-05
TSS200	TRIM17	1	228604595	1	cg18875460	0.109818668	0.174334918	0.276380892	0.166562224	0.001847863	0.007221549
TSS200	TRIM53	11	89735737	1	cg13218423	0.39197496	0.291447678	0.211083082	-0.180891878	6.62E-12	6.35E-10
TSS200	TRIM67	1	231298610/231298651	2	cg21178978/cg27504802	0.36290941	0.421774669	0.531577916	0.168668506	0.000184633	0.000901107
TSS200	TRIM71	3	32859445/32859407/32859377/32859417/32859438	5	cg17029062/cg19127283/cg21124497/cg21484228/cg23528400	0.177171096	0.312241233	0.489104515	0.311933419	4.30E-08	5.27E-07

TSS200	TRIM72	16	31225234	1	cg24918715	0.827228695	0.730094806	0.598446886	-0.228781809	3.99E-07	3.72E-06
TSS200	TRIM9	14	51562550/51562526/51562486/51562591	4	cg04234680/cg05021896/cg15353061/cg25077778	0.179649223	0.258085222	0.342889668	0.163240445	0.000118414	0.000605075
TSS200	TRMT12	8	125462982/125463024/125463007	3	cg09825146/cg11829072/cg26141173	0.025965686	0.044628105	0.184920773	0.158955087	0.003930133	0.01423387
TSS200	TRPC4	13	38444087/38444052/38444134/38443950/38444012	5	cg01764020/cg13549719/cg15696906/cg19275632/cg20535787	0.235588367	0.307854211	0.42716698	0.191578613	1.47E-05	9.29E-05
TSS200	TRPC6	11	101454733/101454683/101454765/101454823/101454676/101454680	6	cg04553838/cg07013734/cg14186937/cg23644960/cg23650423/cg27296293	0.334916158	0.415615892	0.60046499	0.265548833	2.96E-09	5.48E-08
TSS200	TRPC7	5	135701240/135701184	2	cg22770135/cg23409403	0.408309693	0.504725928	0.661992732	0.253683039	3.50E-11	1.88E-09
TSS200	TRPM5	11	2444462	1	cg24894353	0.522255405	0.436107117	0.36481245	-0.157442955	1.48E-09	3.05E-08
TSS200	TRPM8	2	234825859	1	cg23688111	0.34673836	0.280116867	0.190185001	-0.156553359	1.05E-09	2.34E-08
TSS200	TSPAN11	12	31079694	1	cg21701379	0.280128765	0.375699956	0.461281841	0.181153076	1.91E-05	0.00011744
TSS200	TSPAN8	12	71551926	1	cg02965411	0.4956817	0.3893717	0.312714341	-0.182967359	5.86E-13	1.78E-10
TSS200	TSPYL5	8	98290372/98290359/98290229/98290361/98290310	5	cg00032205/cg00249621/cg04917181/cg08858210/cg22319311	0.479892745	0.560587386	0.65569305	0.175800305	4.35E-10	1.17E-08
TSS200	TTBK1	6	43211213/43211208	2	cg16620382/cg27363327	0.211685645	0.279681931	0.453220344	0.241534699	0.000598988	0.002595613
TSS200	TTYH1	19	54926514/54926617/54926437	3	cg15241920/cg15723536/cg21883754	0.192593199	0.35211354	0.553464397	0.360871198	1.62E-07	1.67E-06
TSS200	TUSC3	8	15397637	1	cg16243756	0.34866517	0.475554461	0.627883064	0.279217894	1.30E-08	1.89E-07
TSS200	TWIST1	7	19157296/19157304/19157420/19157339	4	cg00240432/cg09799658/cg10420952/cg12926104	0.124634029	0.213812571	0.414031915	0.289397886	5.84E-07	5.24E-06
TSS200	TYRP1	9	12693326	1	cg25989745	0.604146725	0.508024117	0.384553745	-0.21959298	5.84E-07	5.24E-06
TSS200	UBD	6	29527870/29527885	2	cg05087623/cg05157433	0.62102572	0.5307049	0.44991987	-0.17110585	5.68E-07	5.10E-06
TSS200	UBQLN3	11	5531235/5531199/5531283	3	cg13372231/cg15541062/cg24229750	0.667960305	0.574863861	0.44839652	-0.219563785	3.03E-08	3.89E-07
TSS200	UBQLNL	11	5538030	1	cg09232851	0.76521776	0.69953415	0.536760745	-0.228457015	3.58E-06	2.64E-05
TSS200	UCHL1	4	41258790/41258794	2	cg04178266/cg24715245	0.355670548	0.443175861	0.5294623	0.173791753	0.000307202	0.001423527
TSS200	UGT1A10	2	234544995/234545110	2	cg19975800/cg26238727	0.517837795	0.412424044	0.324400152	-0.193437643	7.47E-11	3.19E-09

TSS200	UNC13A	19	17799037	1	cg12075720	0.2873246	0.340007889	0.476782937	0.189458337	0.000309318	0.001432494
TSS200	UNC5C	4	96470237/96470286/96470349/96470293/96470250	5	cg07234102/cg11723848/cg15984718/cg17334018/cg22634891	0.26381941	0.359211378	0.47920597	0.21538656	2.95E-07	2.84E-06
TSS200	UNC5D	8	35092876/35092823/35092870	3	cg06010588/cg08000065/cg26872137	0.431068495	0.468972635	0.612479635	0.18141114	1.09E-07	1.18E-06
TSS200	UNC80	2	210636560/210636533/210636539	3	cg04100532/cg09438147/cg24938830	0.38753957	0.459278506	0.628306471	0.240766901	3.65E-06	2.68E-05
TSS200	UNCX	7	1272545/1272512/1272559/1272515/1272526/1272486	6	cg05157140/cg08507422/cg17294725/cg20870512/cg21158633/cg21543987	0.340667745	0.361306234	0.521764314	0.181096569	2.33E-05	0.000140333
TSS200	USH2A	1	216596808	1	cg27566805	0.51911659	0.426679828	0.321774473	-0.197342117	4.82E-12	5.24E-10
TSS200	USP17L2	8	11996358/11996327	2	cg01055129/cg04900893	0.67856897	0.547141683	0.462403295	-0.216165675	2.76E-11	1.59E-09
TSS200	USP6	17	5031675	1	cg23416909	0.651823145	0.6215993	0.490925905	-0.16089724	3.23E-05	0.000186821
TSS200	UTF1	10	135043586	1	cg11234328	0.42125665	0.533051528	0.667157064	0.245900414	1.41E-12	2.62E-10
TSS200	VAMP5	2	85811471/85811421/85811432	3	cg11108890/cg16719560/cg19513321	0.385464956	0.505288313	0.57924543	0.193780475	5.92E-05	0.00032249
TSS200	VAT1L	16	77822438/77822350/77822299/77822433/77822419/77822441	6	cg00497967/cg02214096/cg03074283/cg04658772/cg07821427/cg07857243	0.083508518	0.175044789	0.306165967	0.222657449	0.000609431	0.002637967
TSS200	VAV3	1	108231160	1	cg07243548	0.462081	0.338020622	0.255977832	-0.206103168	4.30E-12	5.02E-10
TSS200	VCAN	5	82767394/82767297/82767450	3	cg02551029/cg19540689/cg21810188	0.130012098	0.174571956	0.370658713	0.240646615	2.87E-05	0.000168594
TSS200	VIL1	2	219283823	1	cg18970338	0.41553916	0.332385483	0.258843809	-0.156695351	1.34E-09	2.82E-08
TSS200	VNN1	6	133035235/133035379/133035266	3	cg02184413/cg24964103/cg25654653	0.38331087	0.279078594	0.22145863	-0.16185224	1.85E-12	3.07E-10
TSS200	VPREB1	22	22599128/22599189	2	cg03763796/cg25330366	0.371563065	0.215505522	0.142456698	-0.229106367	6.70E-10	1.64E-08
TSS200	VSIG10L	19	51845446	1	cg07056626	0.442133385	0.33737305	0.2721618	-0.169971585	8.13E-11	3.36E-09
TSS200	VSTM2A	7	54609953/54609836/54609968/54609819	4	cg03817667/cg08532834/cg22131234/cg25022866	0.378145683	0.450642006	0.543144761	0.164999077	6.49E-05	0.000349025
TSS200	VSX1	20	25062827/25062771/25062777/25062860/25062823/25062819	6	cg01715455/cg01941895/cg02631468/cg03111498/cg15272362/cg16185834	0.327231481	0.389951827	0.576211427	0.248979946	6.69E-10	1.64E-08

TSS200	VSX2	14	74706113/74706031/74706016/74706062/74706074	5	cg09816693/cg12827555/cg15416329/cg21057587/cg23118730	0.330851714	0.373553735	0.534947252	0.204095539	7.32E-08	8.38E-07
TSS200	VTCN1	1	117753741/117753602/117753591/117753616	4	cg04718492/cg15597855/cg16408593/cg24006253	0.47737598	0.381396872	0.303938705	-0.173437275	5.35E-11	2.53E-09
TSS200	VWC2	7	49813088/49813102/49813111/49813065	4	cg01893212/cg02467990/cg09493505/cg14045872	0.186555477	0.208329984	0.45091293	0.264357453	0.000294349	0.001370413
TSS200	WBSCR17	7	70597774/70597778/70597713/70597687/70597599	5	cg02172150/cg08251037/cg16005494/cg21135135/cg23839136	0.453998509	0.535313332	0.673429656	0.219431147	2.75E-11	1.59E-09
TSS200	WBSCR26	7	73149356	1	cg17240725	0.345454875	0.253107767	0.190880347	-0.154574528	9.70E-11	3.82E-09
TSS200	WDR17	4	176986922/176986856/176986950/176986787/176986845	5	cg07760910/cg16974909/cg18443378/cg22520644/cg24141863	0.100029361	0.214771414	0.303190996	0.203161634	8.39E-06	5.63E-05
TSS200	WDR86	7	151107285/151107257/151107261	3	cg10770742/cg17362861/cg24068372	0.258717153	0.351823141	0.541493242	0.28277609	6.20E-07	5.52E-06
TSS200	WFDC13	20	44330621	1	cg17890298	0.655958435	0.513161717	0.424119641	-0.231838794	3.26E-13	1.34E-10
TSS200	WIF1	12	65515290/65515276	2	cg24166864/cg26397188	0.41899307	0.498607872	0.575173375	0.156180305	0.000281361	0.00131577
TSS200	WIPF3	7	29874333	1	cg27182527	0.68072004	0.651343228	0.514045509	-0.166674531	1.62E-05	0.00010147
TSS200	WISP3	6	112375302/112375333/112375200/112375186/112375324/112375206	6	cg07709205/cg10525372/cg17694848/cg19804071/cg21773633/cg26528298	0.544106227	0.454339027	0.370440439	-0.173665788	2.61E-11	1.54E-09
TSS200	WNT2	7	116963492/116963502/116963500	3	cg01830294/cg06987468/cg17339147	0.366105237	0.418289035	0.541441255	0.175336018	6.34E-06	4.38E-05
TSS200	XDH	2	31637622	1	cg16862361	0.514609625	0.388478056	0.281634355	-0.23297527	5.19E-12	5.51E-10
TSS200	XIRP1	3	39234137	1	cg14694744	0.528002785	0.381264489	0.258521405	-0.26948138	5.29E-16	8.34E-12
TSS200	XKR7	20	30555644	1	cg16649052	0.2137369	0.264967606	0.386384568	0.172647668	0.001525389	0.006084962
TSS200	ZBTB38	3	141042988	1	cg17495555	0.3893721	0.279197067	0.216185891	-0.173186209	7.53E-12	6.98E-10
TSS200	ZBTB46	20	62436995/62437035/62437033	3	cg02630914/cg21291985/cg23831891	0.708620888	0.663210894	0.539145227	-0.16947566	2.20E-08	2.96E-07
TSS200	ZFR2	19	3869086/3869172/3869174	3	cg13786089/cg15823845/cg24876786	0.229491011	0.334829491	0.581684005	0.352192993	1.28E-08	1.87E-07
TSS200	ZG16	16	29789503	1	cg06289826	0.50826192	0.415210722	0.345703609	-0.162558311	4.38E-11	2.18E-09

TSS200	ZIC1	3	147127012/147127143/147127010/147127137/147127097	5	cg01227537/cg02647941/cg14456683/cg14741939/cg18424634	0.375980816	0.452008149	0.587370597	0.211389782	5.74E-09	9.52E-08
TSS200	ZIC4	3	147110322/147123457/147124543/147124523/147110378/147110367/147123429/147123475/147124417/147110295/147110229	11	cg00235367/cg02387803/cg05548555/cg06369327/cg07850418/cg08812189/cg16790847/cg19516404/cg21127068/cg22796509/cg27416372	0.323738729	0.38568618	0.520684173	0.196945444	1.08E-08	1.61E-07
TSS200	ZIK1	19	58095518/58095588/58095468/58095581/58095445/58095595	6	cg00800512/cg01046104/cg04342092/cg12060744/cg18435449/cg18579862	0.370981891	0.542182535	0.688697526	0.317715635	5.18E-11	2.47E-09
TSS200	ZNF132	19	58951756/58951602/58951684/58951599/58951672/58951778	6	cg00547077/cg03735888/cg05249988/cg12042659/cg13877915/cg24366702	0.176354115	0.30949705	0.432205603	0.255851487	1.37E-05	8.71E-05
TSS200	ZNF134	19	58125672/58125678/58125659	3	cg02835214/cg07697981/cg17655978	0.065203934	0.130905307	0.235507727	0.170303793	0.001996692	0.007753222
TSS200	ZNF135	19	58570454/58570427/58570419/58570466/58570468/58570491	6	cg02473540/cg06454760/cg08701621/cg09907936/cg16638540/cg18430128	0.515488848	0.613870122	0.747959922	0.232471074	2.61E-11	1.54E-09
TSS200	ZNF154	19	58220657/58220662/58220718/58220773	4	cg03234186/cg08668790/cg12506930/cg26465391	0.535933813	0.654501499	0.726430635	0.190496823	6.20E-09	1.01E-07
TSS200	ZNF167	3	44596538/44596512/44596684/44596665/44596536	5	cg03646916/cg03795847/cg08846783/cg19235095/cg21867345	0.344665024	0.451960516	0.524335032	0.179670007	4.58E-05	0.000255995
TSS200	ZNF177	19	9473565/9473598/9473691/9473684/9473688/9473674	6	cg05250458/cg05928342/cg07788092/cg08065231/cg09578475/cg13703871	0.499611157	0.613720865	0.713492797	0.21388164	1.26E-10	4.56E-09
TSS200	ZNF229	19	44952678/44952808/44952766/44952665/44952730/44952725	6	cg09793279/cg09896900/cg10166283/cg14056357/cg19915738/cg22720041	0.294778567	0.383179016	0.492680462	0.197901896	2.68E-05	0.000158914
TSS200	ZNF254	19	24269890/24269968	2	cg09777776/cg17268801	0.223341051	0.255914403	0.389547305	0.166206254	0.00062037	0.002679433

TSS200	ZNF256	19	58459218/58459142/58459139/58459136/58459097/58459248	6	cg05119514/cg12555306/cg16246961/cg22731359/cg23484087/cg27388983	0.101489456	0.147795324	0.335643988	0.234154532	8.55E-06	5.72E-05
TSS200	ZNF264	19	57702809/57702790/57702772/57702697/57702803/57702793	6	cg12096988/cg15257259/cg18576158/cg20715764/cg25800082/cg26970847	0.045250513	0.0991774	0.299380467	0.254129954	0.000223985	0.001073909
TSS200	ZNF280B	22	22863655/22863663	2	cg01223204/cg10719664	0.630906455	0.482624208	0.339280491	-0.291625964	2.94E-12	4.35E-10
TSS200	ZNF304	19	57862484/57862612/57862554/57862627/57862480/57862638	6	cg00397851/cg13788592/cg14015503/cg16848116/cg17849956/cg21627760	0.092965816	0.246682449	0.401504245	0.30853843	5.16E-07	4.68E-06
TSS200	ZNF331	19	54041329/54024110/54058440/54058516/54041303/54023999/54041308/54024023/54041163/54058500/54024076/54041251	12	cg04522821/cg07952047/cg08561667/cg08887146/cg10023249/cg11332363/cg15607708/cg21525020/cg22475353/cg25407227/cg26705425/cg27296330	0.385072945	0.451300946	0.556284818	0.171211873	7.90E-07	6.88E-06
TSS200	ZNF334	20	45142244/45142258/45142214/45142206/45142336	5	cg07139762/cg10140114/cg25828181/cg26238800/cg27385032	0.222102354	0.384134366	0.487326399	0.265224045	9.65E-08	1.06E-06
TSS200	ZNF347	19	53662467/53662439/53662353	3	cg11081729/cg23052615/cg27209110	0.035441866	0.061775464	0.257478493	0.222036627	0.000596006	0.002583404
TSS200	ZNF354C	5	178487465/178487576/178487485/178487500/178487481/178487410	6	cg07470489/cg07515940/cg08278489/cg10802132/cg11538128/cg25942031	0.228625668	0.322063171	0.46824192	0.239616253	3.86E-05	0.000219815
TSS200	ZNF415	19	53636226/53636309/53636229/53636347	4	cg02886522/cg24892510/cg26087117/cg27076144	0.222448654	0.324462103	0.383564948	0.161116294	0.002322917	0.008877868
TSS200	ZNF418	19	58446770/58446758/58446745/58446783/58446898/58446787	6	cg11788523/cg12961842/cg13668618/cg15060012/cg18673377/cg21444693	0.496433315	0.633033017	0.718227547	0.221794232	9.84E-10	2.22E-08
TSS200	ZNF43	19	22019044/22019094/22019059	3	cg13858139/cg18875716/cg19063564	0.222900055	0.288537061	0.438528713	0.215628658	6.31E-07	5.60E-06

TSS200	ZNF454	5	178368071/178368123/178368160/178368185/178368205/178368183	6	cg02165355/cg03234732/cg10575261/cg16536329/cg20778451/cg23037403	0.412298729	0.466807437	0.627704703	0.215405974	5.12E-09	8.67E-08
TSS200	ZNF461	19	37157879/37157922/37157899/37157861/37157847/37157776	6	cg00498155/cg02306127/cg08066844/cg11419456/cg15131414/cg26613140	0.063699029	0.077798669	0.220632815	0.156933786	0.003388021	0.012462258
TSS200	ZNF471	19	57019069/57019016/57019022	3	cg00674365/cg11539780/cg19811761	0.426889097	0.439897093	0.604074329	0.177185232	0.000749437	0.003186268
TSS200	ZNF492	19	22816980/22817096/22817017/22817039/22816950	5	cg05241461/cg06748831/cg11785166/cg23217946/cg24889512	0.29730671	0.401194691	0.561396058	0.264089348	1.98E-09	3.89E-08
TSS200	ZNF528	19	52900980/52900970/52900927/52900988/52900974	5	cg09267217/cg12950007/cg20725941/cg22935432/cg27258025	0.142143558	0.254904129	0.339809338	0.197665781	0.001144182	0.004677887
TSS200	ZNF529	19	37096321/37064303/37096329/37064219/37064289/37064254/37064380/37096323/37064375	9	cg02587316/cg03222310/cg05020604/cg07624650/cg07904448/cg09500430/cg17313483/cg18630667/cg27335720	0.263995848	0.338022123	0.441277201	0.177281353	1.56E-09	3.20E-08
TSS200	ZNF540	19	38042154/38042109/38042123	3	cg03320255/cg17431391/cg27389185	0.335901932	0.419555614	0.523873674	0.187971743	1.03E-07	1.13E-06
TSS200	ZNF542	19	56879662/56879432/56879369/56879554/56879559/56879418/56879571/56879613/56879645	9	cg03146949/cg06942685/cg10638415/cg15708153/cg18787401/cg21493505/cg26309134/cg27062795/cg27477373	0.311414553	0.421541164	0.586709571	0.275295018	2.21E-09	4.27E-08
TSS200	ZNF543	19	57831834/57831819/57831705/57831816/57831849/57831684/57831678	7	cg01044608/cg01325460/cg01923740/cg14786398/cg17826375/cg20655405/cg23986470	0.06741207	0.189605625	0.285694766	0.218282696	0.000319525	0.001475431
TSS200	ZNF544	19	58739944/58739986/58740004	3	cg00683895/cg01859739/cg16187092	0.042368174	0.090580143	0.222478302	0.180110128	0.002711621	0.010188122

TSS200	ZNF549	19	58038689/58038573/58038585/58038633/58038588/58038621	6	cg01601746/cg06458239/cg10729426/cg16697731/cg19060970/cg20607331	0.285128359	0.456519629	0.650572955	0.365444596	3.64E-09	6.58E-08
TSS200	ZNF560	19	9609397/9609395/9609335/9609422/9609405/9609342	6	cg05221167/cg18568589/cg18712973/cg19254119/cg19544372/cg24818573	0.396684993	0.482161754	0.569145295	0.172460302	3.90E-10	1.07E-08
TSS200	ZNF568	19	37407060/37407041/37407214/37407152/37407216/37407123	6	cg02370417/cg03584288/cg05661809/cg15322783/cg20680720/cg24052101	0.148157455	0.291012401	0.402926108	0.254768653	4.65E-05	0.000259182
TSS200	ZNF570	19	37959949/37959940/37959855/37959864/37959961/37959853	6	cg04524120/cg07746323/cg11237751/cg11737710/cg19655456/cg21850254	0.068099201	0.114861064	0.227201987	0.159102787	0.000613586	0.00265304
TSS200	ZNF578	19	52956674/52956657/52956771/52956683/52956729/52956821	6	cg11909748/cg12665460/cg13461241/cg14582763/cg15449323/cg21553182	0.43395605	0.516626312	0.657462475	0.223506425	1.30E-11	1.01E-09
TSS200	ZNF582	19	56904945/56904977/56904901/56904997/56905032/56904965/56905013	7	cg02763101/cg08464824/cg09568464/cg13916740/cg20984085/cg22647407/cg24039631	0.192596768	0.301554531	0.450527057	0.257930289	3.19E-05	0.000185005
TSS200	ZNF586	19	58280832/58280891/58280927/58281016/58281019/58280994	6	cg03584535/cg07685728/cg13636880/cg18575209/cg20751795/cg24298255	0.138086232	0.14550535	0.337383294	0.199297062	0.0029918	0.011123312
TSS200	ZNF625	19	12267676/12267685/12267592	3	cg02914087/cg06429617/cg17287725	0.101405888	0.261550961	0.374411985	0.273006097	1.75E-05	0.00010938
TSS200	ZNF626	19	20844422	1	cg01808171	0.27876178	0.446592467	0.591171732	0.312409952	5.57E-09	9.29E-08
TSS200	ZNF660	3	44626453/44626269/44626304/44626400	4	cg03340649/cg11542789/cg15372508/cg21771463	0.128708754	0.209880812	0.33976017	0.211051416	6.55E-06	4.52E-05
TSS200	ZNF667	19	56988813/56988956/56988929/56988935/56988778/56988806	6	cg00502665/cg07967091/cg08687005/cg15805104/cg24464397/cg26612735	0.335945727	0.442726872	0.572549724	0.236603998	4.29E-10	1.16E-08
TSS200	ZNF671	19	58239012/58239135	2	cg08048222/cg24016939	0.220574027	0.315750092	0.473614271	0.253040244	3.04E-06	2.29E-05

TSS200	ZNF677	19	53758289/53758297/53758233/53758309	4	cg06506966/cg09379485/cg24114314/cg25404339	0.141947758	0.240661004	0.43732722	0.295379463	8.83E-06	5.90E-05
TSS200	ZNF683	1	26699448	1	cg06114363	0.611410575	0.528032094	0.436944536	-0.174466039	1.40E-05	8.88E-05
TSS200	ZNF75A	16	3355361/3355411/3355295	3	cg03004280/cg07306531/cg16480692	0.240135958	0.370008164	0.553979262	0.313843304	9.75E-09	1.48E-07
TSS200	ZNF772	19	57989134/57989117/57989099/57988964/57989027/57988958	6	cg02298956/cg03526224/cg03553544/cg23235772/cg24911359/cg25419914	0.115992377	0.203781421	0.334551282	0.218558904	6.50E-06	4.49E-05
TSS200	ZNF773	19	58011241/58011244/58011257	3	cg03612039/cg21117673/cg22411784	0.088530294	0.154564377	0.266428029	0.177897735	0.000683576	0.002925978
TSS200	ZNF781	19	38183253/38183360/38183262/38183259	4	cg03611452/cg10701051/cg14587524/cg25324105	0.182495559	0.231975475	0.360267866	0.177772307	0.000298925	0.001388561
TSS200	ZNF788	19	12203066/12203029	2	cg09489445/cg20773651	0.156508032	0.269657983	0.497029508	0.340521476	2.40E-07	2.37E-06
TSS200	ZNF790	19	37329448/37329366/37329403/37329330/37329475/37329381/37329431	7	cg06466031/cg07825094/cg09567473/cg10400937/cg14195122/cg18876786/cg24775172	0.09227895	0.14907985	0.280611718	0.188332768	0.000183121	0.00089567
TSS200	ZNF793	19	37997798/37997730/37997675/37997831/37997817/37997703/37997791/37997682	8	cg02711801/cg07482795/cg13181745/cg14732998/cg23296010/cg24078985/cg24588375/cg25361907	0.087173797	0.202610546	0.329949659	0.242775862	2.34E-05	0.000140651
TSS200	ZNF814	19	58400494/58400574/58400559/58400504	4	cg15746696/cg18860954/cg25400396/cg27112264	0.34868974	0.438296465	0.601844211	0.253154471	1.26E-07	1.34E-06
TSS200	ZNF826	19	20607771	1	cg25404914	0.43316604	0.563562906	0.649471594	0.216305554	5.29E-06	3.72E-05
TSS200	ZNF829	19	37407284/37407374/37407214/37407216/37407257	5	cg02459604/cg03060201/cg05661809/cg20680720/cg21761844	0.150904284	0.296808366	0.422480145	0.271575861	9.65E-05	0.000502624
TSS200	ZNF85	19	21106043/21106016/21106027/21106036/21106053/21106002	6	cg05310990/cg15690721/cg19381810/cg21448033/cg23216292/cg25464210	0.283905038	0.392508137	0.572298856	0.288393818	1.88E-09	3.73E-08

TSS200	ZNF876P	4	206267/206282/206232/206339	4	cg13535626/cg14186187/cg18005867/cg23063647	0.42917707	0.514725788	0.59120688	0.16202981	8.52E-08	9.56E-07
TSS200	ZNF879	5	178450758/178450646/178450763/178450595	4	cg00601350/cg05244234/cg17861295/cg25396971	0.190092853	0.201390715	0.34142491	0.151332057	0.011926274	0.03798534
TSS200	ZNF880	19	52873085/52873125/52873106/52873101/52873019	5	cg02627531/cg09313053/cg13393110/cg14224937/cg17650272	0.167456923	0.248452032	0.394450676	0.226993753	0.000245164	0.001164105
TSS200	ZNF98	19	22605186/22605188/22605234/22605151	4	cg01436128/cg09768178/cg10601582/cg13371839	0.402760787	0.491516887	0.611237127	0.208476341	2.34E-10	7.12E-09
TSS200	ZSCAN12	6	28367644/28367571/28367544	3	cg18815343/cg25060829/cg27577527	0.115882911	0.156114557	0.285765645	0.169882734	0.005423791	0.018922069
TSS200	ZSCAN18	19	58629828/58609764/58609793/58629837/58629901/58629967/58609744/58629975/58609806/58609730/58609770	11	cg03145999/cg07657064/cg07924874/cg08873805/cg14231297/cg17408527/cg18428688/cg18693673/cg18888520/cg22031998/cg23229395	0.362318709	0.429500804	0.550129553	0.187810844	1.84E-05	0.000114047
TSS200	ZSCAN23	6	28411279/28411423/28411287/28411285/28411378/28411309	6	cg00651523/cg07836142/cg09682330/cg14547644/cg21486944/cg24703168	0.17786891	0.239535867	0.410736147	0.232867237	6.10E-07	5.44E-06
TSS200	ZSWIM2	2	187713986/187713964/187713902/187714068/187714055/187714003	6	cg05907046/cg07548607/cg10396546/cg14762973/cg16516691/cg24617723	0.238870902	0.309867499	0.478734976	0.239864075	3.47E-08	4.40E-07
UTR5	A2ML1	12	8975196/8975198	2	cg03490200/cg13863204	0.798196353	0.682005733	0.605282773	-0.19291358	5.85E-07	4.38E-06
UTR5	A4GNT	3	137851111	1	cg17687282	0.555490305	0.439574633	0.352429409	-0.203060896	4.65E-08	4.91E-07
UTR5	ABCA8	17	66942706/66950545	2	cg03850529/cg21660392	0.63174626	0.588156994	0.461315914	-0.170430346	8.89E-07	6.31E-06
UTR5	ABCB5	7	20656503/20687223/20689757	3	cg21202529/cg22066521/cg22426329	0.781326097	0.685453402	0.590205333	-0.191120763	1.04E-10	3.83E-09
UTR5	ABCC11	16	48266305	1	cg07383076	0.5685022	0.461615661	0.343803182	-0.224699018	4.87E-12	6.36E-10
UTR5	ABCC2	10	101542587	1	cg14947634	0.80488305	0.72094985	0.644778177	-0.160104873	0.00026172	0.001009608
UTR5	ACCSL	11	44069581	1	cg23980760	0.65016958	0.530142694	0.413524532	-0.236645048	3.77E-11	2.11E-09

UTR5	ACER1	19	6333587/6333593	2	cg12999109/cg19598519	0.732704423	0.602333214	0.524733277	-0.207971145	1.37E-08	1.70E-07
UTR5	ACOT7	1	6420733/6453807/6420713/6445872/6445804	5	cg00821310/cg02568911/cg10219223/cg20414935/cg23095988	0.483786784	0.399748578	0.326385994	-0.15740079	3.49E-11	2.03E-09
UTR5	ACSM5	16	20421627	1	cg16736889	0.420887785	0.3644144	0.265794305	-0.15509348	8.75E-07	6.23E-06
UTR5	ACSS3	12	81471882/81471867/81471884	3	cg10789281/cg13273396/cg26272220	0.33108129	0.531606769	0.618354419	0.287273129	3.71E-08	4.05E-07
UTR5	ACTN2	1	236849942/236849966	2	cg11215976/cg18770350	0.46544647	0.527563422	0.624981395	0.159534925	3.63E-07	2.93E-06
UTR5	ADAM28	8	24151625	1	cg22915945	0.315348114	0.155805396	0.11973464	-0.195613474	1.37E-07	1.25E-06
UTR5	ADAMTS1	21	28217676	1	cg00472814	0.255875925	0.265509433	0.409879018	0.154003093	0.004952119	0.014068237
UTR5	ADAMTS18	16	77468893/77468850	2	cg03238797/cg07568194	0.242036425	0.243960726	0.416279982	0.174243557	0.000215896	0.000852723
UTR5	ADAMTS5	21	28338836/28339334/28339262	3	cg03202077/cg15237494/cg23986671	0.33495262	0.433389163	0.603467985	0.268515365	1.37E-08	1.70E-07
UTR5	ADAMTSL3	15	84323770/84323154/84322946/84323963	4	cg01152302/cg11611600/cg14230666/cg21377071	0.436340716	0.522116803	0.615921415	0.179580699	2.76E-10	7.96E-09
UTR5	ADARB2	10	1779432/1779667	2	cg00467420/cg05307923	0.152456797	0.280145489	0.436033709	0.283576912	4.02E-07	3.18E-06
UTR5	ADCY8	8	132052779/132052702	2	cg03120091/cg05229355	0.291294298	0.390955817	0.553517995	0.262223698	1.72E-09	3.10E-08
UTR5	ADCYAP1	18	905156/905382/905180/904963/905252/905127/905101/905177	8	cg00128702/cg07211875/cg11773720/cg11850773/cg14200170/cg15194943/cg17059658/cg22388954	0.344375033	0.469901083	0.581655572	0.237280539	1.40E-08	1.74E-07
UTR5	ADH6	4	100140325/100140396	2	cg06518271/cg08988543	0.581643105	0.443150483	0.352798286	-0.228844819	6.58E-11	3.00E-09
UTR5	ADHFE1	8	67344720	1	cg20912169	0.256459935	0.411193623	0.660422359	0.403962425	7.83E-11	3.23E-09
UTR5	ADIG	20	37209890	1	cg17349406	0.62893823	0.49931435	0.415873145	-0.213065085	4.12E-12	5.61E-10
UTR5	ADRA1A	8	26722635/26722861/26722496	3	cg07645844/cg17963840/cg21230493	0.442435768	0.519901074	0.629701238	0.18726547	1.06E-07	1.01E-06
UTR5	AEBP1	7	44143998/44143993	2	cg02126753/cg14249876	0.1369801	0.257681438	0.504188142	0.367208042	4.84E-07	3.72E-06
UTR5	AGR2	7	16841504/16844606	2	cg02066184/cg21201572	0.587817313	0.470783503	0.342052432	-0.245764881	1.52E-11	1.31E-09
UTR5	AGR3	7	16920271	1	cg16702815	0.544244365	0.419571967	0.322374955	-0.22186941	2.58E-11	1.74E-09
UTR5	AKNA	9	117150495	1	cg14080475	0.2317446	0.393075678	0.524686191	0.292941591	4.74E-14	4.22E-11
UTR5	AKR1B1	7	134143823	1	cg18416881	0.146743665	0.255080443	0.329499074	0.18275541	0.00348242	0.010288856

UTR5	AKR1B10	7	134212463	1	cg25171118	0.49394053	0.383134544	0.2866655	-0.20727503	1.87E-10	5.82E-09
UTR5	ALDH3A1	17	19651653/19648972/19648925/19648855/19648853/19650069/19649051/19649293/19648718/19651190/19649004/19648970/19648846/19649191	14	cg00516966/cg01471036/cg05447343/cg06390079/cg11475454/cg12258471/cg12787571/cg13295878/cg15046965/cg15796819/cg15849060/cg18957070/cg23098051/cg23392390	0.529564653	0.440579233	0.376167973	-0.15339668	1.34E-10	4.59E-09
UTR5	ALG1L	3	125655381/125655407	2	cg05084668/cg07473553	0.68058229	0.463321817	0.311121282	-0.369461008	2.07E-12	3.76E-10
UTR5	ALK	2	30144376/30143585/30143739/30144152	4	cg06905692/cg07443217/cg12976501/cg18277754	0.264042581	0.363556944	0.467856574	0.203813992	6.02E-10	1.38E-08
UTR5	ALOX12B	17	7990985	1	cg05952715	0.777149395	0.686436261	0.583084073	-0.194065322	1.15E-10	4.10E-09
UTR5	ALPI	2	233320854	1	cg08210297	0.528081415	0.441415483	0.3559656	-0.172115815	8.65E-10	1.84E-08
UTR5	ALPP	2	233243398	1	cg19654061	0.600517355	0.462496261	0.365646864	-0.234870491	8.19E-11	3.31E-09
UTR5	ALX4	11	44331629/44331623	2	cg02504416/cg22931182	0.231549365	0.276250108	0.424848216	0.193298851	4.13E-05	0.000192354
UTR5	AMPH	7	38670957/38670987/38670985/38671001	4	cg02383130/cg07926691/cg10293925/cg26122980	0.220771274	0.30209	0.49313757	0.272366296	4.26E-06	2.50E-05
UTR5	AMTN	4	71384359	1	cg05329893	0.581913015	0.491348928	0.376376132	-0.205536883	1.07E-06	7.43E-06
UTR5	ANK1	8	41754172/41522721/41754181/41655078/41754060	5	cg05166490/cg12439423/cg17331296/cg26326633/cg26530758	0.358238866	0.433221078	0.510682627	0.152443761	1.19E-07	1.11E-06
UTR5	ANKLE1	19	17392656	1	cg00433770	0.083000247	0.137272889	0.251535186	0.168534939	0.001854386	0.005824123
UTR5	ANKRD30B	18	14748285/14748298/14748250	3	cg03014326/cg21281009/cg24061208	0.535134225	0.60648387	0.721314165	0.18617994	5.14E-06	2.96E-05
UTR5	ANKRD33B	5	10564448/10564514	2	cg07682578/cg09877593	0.031648307	0.067566136	0.200698759	0.169050452	0.00116464	0.00384167
UTR5	ANKRD55	5	55529124/55412572	2	cg11264935/cg22709202	0.610293455	0.5384717	0.450503718	-0.159789737	1.15E-07	1.08E-06
UTR5	ANO5	11	22214934/22214726	2	cg19639184/cg20115218	0.162117194	0.239535159	0.344009078	0.181891884	0.001526342	0.004886658

UTR5	ANXA2	15	60689285/60688010/60689670/60683059/60687284/60690026/60688948/60689852/60688622	9	cg02072495/cg06738887/cg09533293/cg11681321/cg13313836/cg13634501/cg14397690/cg22293140/cg22365276	0.420798919	0.317853054	0.229039977	-0.191758941	1.55E-12	3.31E-10
UTR5	ANXA9	1	150954826/150954554	2	cg13320146/cg20437604	0.51022676	0.410074872	0.3194991	-0.19072766	7.53E-11	3.22E-09
UTR5	AOAH	7	36764019/36764140/36764082	3	cg14451276/cg18652683/cg25733272	0.549081075	0.630627097	0.711896268	0.162815193	1.18E-09	2.32E-08
UTR5	AOX1	2	201450731/201450743	2	cg12627583/cg13875120	0.148069937	0.191967324	0.30264263	0.154572693	0.004463237	0.012799565
UTR5	APCS	1	159557632	1	cg03856044	0.480257805	0.425551878	0.307269341	-0.172988464	7.47E-11	3.22E-09
UTR5	APOBEC3A	22	39353650/39353550	2	cg21345826/cg27504369	0.587978545	0.466772394	0.394652645	-0.1933259	1.24E-09	2.41E-08
UTR5	AQP5	12	50355307	1	cg15336765	0.174818933	0.236789049	0.489375127	0.314556195	1.46E-10	4.86E-09
UTR5	ARHGAP20	11	110583036/110583377	2	cg03813168/cg26578621	0.096245742	0.161655252	0.26764626	0.171400518	0.000212975	0.00084312
UTR5	ARHGAP28	18	6835382/6834653	2	cg00402547/cg27314249	0.69040329	0.60615055	0.53742815	-0.15297514	7.13E-09	9.90E-08
UTR5	ARL14	3	160395073	1	cg11896170	0.58850083	0.444467894	0.350764123	-0.237736707	1.67E-12	3.47E-10
UTR5	ARMC3	10	23217173/23217017	2	cg11710969/cg15193782	0.220265833	0.254205498	0.38360255	0.163336717	0.000177634	0.000714106
UTR5	ASB10	7	150884848/150884429/150884914	3	cg16537044/cg16564894/cg26159905	0.60730881	0.506939487	0.397815974	-0.209492836	8.73E-11	3.43E-09
UTR5	ASB14	3	57326068	1	cg21455927	0.645924565	0.524519633	0.434560355	-0.21136421	1.21E-12	2.89E-10
UTR5	ASB15	7	123249130/123252579	2	cg02300083/cg26067096	0.61231217	0.532766017	0.371533668	-0.240778502	4.45E-07	3.46E-06
UTR5	ASCL3	11	8964544/8964505	2	cg02127238/cg18920846	0.818647735	0.756566697	0.657828745	-0.16081899	2.99E-06	1.85E-05
UTR5	ASCL4	12	108168562/108168987/108168824	3	cg01952953/cg04290586/cg13721169	0.503941785	0.630292217	0.746275273	0.242333488	2.84E-11	1.85E-09
UTR5	ASTN1	1	177133946/177134019	2	cg06650419/cg14659404	0.207876626	0.272115327	0.411315161	0.203438536	4.18E-07	3.29E-06
UTR5	ATP10A	15	26108263	1	cg20174066	0.210780559	0.283416574	0.366172759	0.155392201	8.55E-06	4.69E-05
UTR5	ATP1B2	17	7554827/7554666/7554357/7554513/7554681	5	cg00053373/cg02705837/cg03554817/cg06200013/cg22448889	0.120678939	0.205364455	0.328424325	0.207745385	2.84E-06	1.76E-05
UTR5	B3GNT6	11	76750184/76747513/76750413	3	cg01974921/cg06085657/cg09467902	0.678626115	0.531252989	0.432564527	-0.246061588	7.50E-11	3.22E-09
UTR5	BAIAP2L2	22	38506623/38506589	2	cg17838773/cg27505627	0.585663558	0.461452011	0.364674816	-0.220988742	5.72E-11	2.74E-09
UTR5	BARHL2	1	91182777	1	cg18322569	0.486139255	0.506292575	0.753573723	0.267434468	6.83E-05	0.00030275

UTR5	BCAT1	12	25102274/25102072	2	cg04011247/cg10764357	0.202790783	0.308302367	0.516316832	0.313526049	1.76E-09	3.17E-08
UTR5	BCL2L15	1	114430031	1	cg24550026	0.46598524	0.326013478	0.250770314	-0.215214926	1.16E-11	1.11E-09
UTR5	BDKRB1	14	96729656/96727955	2	cg05158854/cg23347654	0.43763738	0.290761967	0.20292176	-0.23471562	3.63E-13	1.45E-10
UTR5	BEND4	4	42154385/42154840	2	cg05674475/cg10172669	0.214397236	0.342908991	0.455064775	0.240667539	1.39E-07	1.26E-06
UTR5	BEND5	1	49242513/49242519	2	cg11666087/cg16573178	0.065825382	0.246131481	0.318706693	0.25288131	0.000244154	0.000950415
UTR5	BEST3	12	70082885/70093008/70092840	3	cg12596182/cg17277939/cg23591609	0.585985325	0.471389889	0.406789225	-0.1791961	2.92E-10	8.28E-09
UTR5	BHLHE23	20	61638149	1	cg21161253	0.108113161	0.261707707	0.349949555	0.241836394	7.75E-07	5.61E-06
UTR5	BIRC7	20	61867349	1	cg00895528	0.476063275	0.387165683	0.313177091	-0.162886184	1.43E-08	1.77E-07
UTR5	BNIPL	1	151009588	1	cg11584936	0.86080939	0.759247922	0.646079755	-0.214729635	1.82E-06	1.18E-05
UTR5	BRUNOL4	18	35145983/35145650	2	cg11154070/cg19492446	0.24945564	0.282276786	0.408337759	0.158882119	0.000224815	0.000883731
UTR5	BTNL3	5	180415938	1	cg02720155	0.51763518	0.42233935	0.341113614	-0.176521566	1.48E-07	1.33E-06
UTR5	BVES	6	105584149/105584216	2	cg20624391/cg25280433	0.19679352	0.254422961	0.366656853	0.169863333	0.000714712	0.002471216
UTR5	C10orf81	10	115514781	1	cg18203466	0.55010488	0.414215044	0.325414677	-0.224690203	1.98E-11	1.58E-09
UTR5	C10orf90	10	128209980	1	cg24143287	0.67340542	0.465538717	0.322228568	-0.351176852	1.91E-12	3.63E-10
UTR5	C10orf99	10	85933586	1	cg10737718	0.41991726	0.323038672	0.257776177	-0.162141083	4.24E-11	2.29E-09
UTR5	C11orf41	11	33563946	1	cg22937632	0.678477485	0.593174794	0.471460355	-0.20701713	1.13E-08	1.46E-07
UTR5	C11orf53	11	111126790/111148753	2	cg19964761/cg27649396	0.807520505	0.7183644	0.614021768	-0.193498737	1.20E-05	6.32E-05
UTR5	C11orf87	11	109293614/109293070/109292894/109293216/109294168/109294233/109293945/109294044/109293068/109294190/109294069	11	cg00830817/cg04861929/cg06719900/cg07142201/cg07180307/cg07428323/cg10454766/cg13952556/cg15948088/cg21852408/cg26014266	0.293567021	0.379175347	0.557336049	0.263769028	1.85E-09	3.29E-08
UTR5	C11orf94	11	45928805	1	cg04310460	0.54808115	0.430428489	0.356957232	-0.191123918	5.65E-11	2.74E-09
UTR5	C12orf59	12	10331614/10331666	2	cg25494227/cg26718420	0.6664606	0.575493056	0.485267255	-0.181193345	4.23E-07	3.31E-06
UTR5	C13orf38	13	36871878/36871646/36871962/36871754/36871948/36871943/36867876	7	cg04902286/cg07525003/cg13258563/cg13455704/cg15167956/cg26439710/cg26822330	0.312398906	0.380508123	0.469342215	0.156943309	5.49E-06	3.14E-05

UTR5	C14orf180	14	105051261/105048339/105048531/105046247/105052560	5	cg01952537/cg04141770/cg13276428/cg15421520/cg18313013	0.556174145	0.442103461	0.37211446	-0.184059685	1.19E-14	3.57E-11
UTR5	C14orf39	14	60952097/60952599/60952196/60952405/60952738	5	cg05255275/cg18240143/cg18921771/cg23989821/cg27398547	0.28767244	0.40725815	0.622664336	0.334991896	6.54E-11	2.99E-09
UTR5	C15orf62	15	41062273/41062224	2	cg05638787/cg07306253	0.452227933	0.362755947	0.286699936	-0.165527996	2.87E-11	1.86E-09
UTR5	C16orf11	16	612306/611985/611384/613142/610895/610499/611177/613006	8	cg01994216/cg05325887/cg07576033/cg08864283/cg08950915/cg23008708/cg24160381/cg27558697	0.522122171	0.441372156	0.369168685	-0.152953486	1.35E-12	3.05E-10
UTR5	C17orf104	17	42733976	1	cg22190438	0.175055483	0.221011376	0.410953534	0.235898052	1.34E-06	9.08E-06
UTR5	C17orf46	17	43339476	1	cg17222164	0.110370772	0.218942219	0.334238974	0.223868202	0.000188332	0.000754179
UTR5	C17orf47	17	56621578	1	cg26956135	0.53089084	0.426637511	0.297128173	-0.233762667	1.23E-13	7.31E-11
UTR5	C19orf21	19	751241/751159/752860/755197	4	cg04245402/cg14052044/cg19546012/cg26813907	0.557156342	0.448712193	0.352329909	-0.204826433	9.75E-11	3.70E-09
UTR5	C19orf33	19	38794845/38794814	2	cg00412772/cg11272232	0.434242788	0.347337644	0.281336405	-0.152906383	8.75E-10	1.85E-08
UTR5	C19orf75	19	51762753/51761116	2	cg05619872/cg21930712	0.50822113	0.406950344	0.293409632	-0.214811498	2.13E-11	1.64E-09
UTR5	C1orf106	1	200864307/200864223	2	cg00971747/cg16257559	0.53500073	0.427568308	0.344432727	-0.190568003	2.74E-11	1.81E-09
UTR5	C1orf173	1	75139347/75139364	2	cg24155871/cg27393126	0.295957565	0.367192178	0.573761716	0.277804151	1.35E-07	1.24E-06
UTR5	C1orf226	1	162351664	1	cg25212701	0.509942485	0.389087383	0.300863691	-0.209078794	3.86E-12	5.51E-10
UTR5	C1orf70	1	1475737	1	cg16601494	0.18564747	0.361693367	0.665361532	0.479714062	1.67E-10	5.40E-09
UTR5	C1QC	1	22970339/22970132	2	cg00136477/cg17104151	0.497507993	0.381689306	0.256685968	-0.240822024	3.12E-13	1.29E-10
UTR5	C1QL2	2	119916017/119916431	2	cg00690148/cg26530498	0.298093565	0.394724053	0.512992241	0.214898676	4.77E-09	7.20E-08
UTR5	C1QTNF8	16	1145964/1146194	2	cg06208229/cg07412281	0.54548979	0.466190267	0.382244541	-0.163245249	1.29E-10	4.43E-09
UTR5	C20orf103	20	9495495/9495326/9495404	3	cg12650655/cg20191310/cg26582643	0.155063925	0.172190491	0.307051689	0.151987763	0.005606782	0.015687355
UTR5	C20orf71	20	31805291/31805182	2	cg09742177/cg15131258	0.67492102	0.584522178	0.477997677	-0.196923343	2.99E-05	0.000143808
UTR5	C4BPB	1	207262428/207262553/207262706	3	cg14740771/cg21571160/cg26407558	0.428842915	0.337610065	0.25503923	-0.173803685	7.93E-12	8.70E-10
UTR5	C4orf26	4	76481269	1	cg16870215	0.51492345	0.433237433	0.347102814	-0.167820636	4.75E-09	7.18E-08

UTR5	C4orf49	4	140201433	1	cg18065318	0.24092675	0.284793844	0.398248473	0.157321723	0.000128356	0.000533641
UTR5	C6orf105	6	11779039	1	cg26761744	0.56337731	0.424441856	0.353218586	-0.210158724	4.48E-10	1.11E-08
UTR5	C6orf222	6	36303570/36304456	2	cg11562147/cg13319417	0.54654998	0.457730333	0.395577541	-0.150972439	7.36E-11	3.21E-09
UTR5	C8orf34	8	69351585/69350927	2	cg00280504/cg22199118	0.331943675	0.235495517	0.18020561	-0.151738065	3.21E-11	1.98E-09
UTR5	C8orf46	8	67405742	1	cg23704362	0.666288825	0.594698256	0.515657332	-0.150631493	1.50E-05	7.74E-05
UTR5	C8orf85	8	117950473	1	cg05996052	0.367959095	0.481860539	0.645422682	0.277463587	1.08E-12	2.76E-10
UTR5	C9orf152	9	112970404	1	cg09596614	0.476353965	0.383152094	0.307880405	-0.16847356	3.32E-10	9.10E-09
UTR5	C9orf47	9	91605893	1	cg12972233	0.4305414	0.565103506	0.613298873	0.182757473	6.36E-07	4.70E-06
UTR5	C9orf50	9	132383003	1	cg09731694	0.31731967	0.549861672	0.697157327	0.379837657	6.72E-16	4.47E-12
UTR5	CA10	17	50236572/50237205/50236087/50235328/50236465/50235737/50235393/50236765/50235965/50237267/50235279/50236344/50237315	13	cg04881814/cg07398767/cg08034077/cg14054928/cg14056110/cg14073722/cg20405017/cg21328033/cg22702328/cg22855255/cg24341611/cg25039722/cg25592977	0.409826752	0.479281985	0.619400012	0.20957326	6.25E-10	1.42E-08
UTR5	CACNA1A	19	13617094/13617091	2	cg17509967/cg22491927	0.24286006	0.353768224	0.512181286	0.269321226	3.76E-07	3.01E-06
UTR5	CACNG3	16	24267226/24267399/24267559/24267526/24266905	5	cg02692912/cg04441477/cg04543289/cg07514158/cg17234150	0.338346394	0.442153518	0.596171655	0.257825261	2.71E-09	4.47E-08
UTR5	CACNG8	19	54466297	1	cg08653692	0.27789309	0.375280813	0.674200927	0.396307837	1.20E-09	2.35E-08
UTR5	CADM2	3	85008678/85008587/85008156	3	cg05152589/cg13331200/cg26113636	0.306559718	0.336317308	0.462555287	0.155995569	1.36E-06	9.21E-06
UTR5	CALHM3	10	105238873/105238964	2	cg04804772/cg11957248	0.55451158	0.463332944	0.39100485	-0.16350673	5.45E-11	2.67E-09
UTR5	CALML4	15	68498251/68497992	2	cg05338167/cg11530995	0.45808637	0.373735086	0.304724143	-0.153362227	6.76E-11	3.04E-09
UTR5	CAPN14	2	31440349/31439687	2	cg04132353/cg15941014	0.500511375	0.314608206	0.241193082	-0.259318293	3.17E-10	8.78E-09
UTR5	CASS4	20	54987330/54987270	2	cg10463299/cg14303122	0.52536621	0.617127156	0.681160934	0.155794724	8.83E-07	6.28E-06
UTR5	CAV3	3	8775516	1	cg06072564	0.72348022	0.657715439	0.519373645	-0.204106575	7.73E-08	7.69E-07

UTR5	CBLN2	18	70210483/70210433/70209432/70209551/70210025/70210955/70210494/70210819/70211515	9	cg01908010/cg03561455/cg13478228/cg15733692/cg17952513/cg18596362/cg19202058/cg21529405/cg21902544	0.239248293	0.344807051	0.495925716	0.256677423	1.24E-09	2.41E-08
UTR5	CBLN4	20	54579355/54579814	2	cg02501779/cg14184078	0.51584526	0.547781811	0.713582132	0.197736872	2.71E-09	4.47E-08
UTR5	CBS	21	44493901/44494722/44496012/44494997/44495495/44494906/44495595/44495288/44495223/44495141/44495796	11	cg01071153/cg01193064/cg03559229/cg10457056/cg14364356/cg15108590/cg19950455/cg24437122/cg24496978/cg26141228/cg27024932	0.212937344	0.236612672	0.366228591	0.153291247	0.00020507	0.00081594
UTR5	CCDC105	19	15121591/15121596	2	cg03928546/cg06522681	0.439871208	0.515696853	0.689640884	0.249769677	1.44E-09	2.69E-08
UTR5	CCDC114	19	48823178	1	cg25533551	0.57711474	0.477702278	0.392349423	-0.184765317	9.75E-11	3.70E-09
UTR5	CCDC30	1	43001072	1	cg24501381	0.781376155	0.676996133	0.606270155	-0.175106	1.72E-09	3.10E-08
UTR5	CCDC37	3	126113784/126113918/126114064	3	cg00891278/cg19628148/cg20707970	0.357535978	0.414504994	0.58240912	0.224873143	2.09E-07	1.80E-06
UTR5	CCDC42	17	8647944/8648035	2	cg06468695/cg10057436	0.7384243	0.602032211	0.476622014	-0.261802286	5.68E-12	6.95E-10
UTR5	CCDC8	19	46916741/46916520/46916588	3	cg06747432/cg15984661/cg25987744	0.134449806	0.228445801	0.356166245	0.221716439	1.33E-06	9.00E-06
UTR5	CCNA1	13	37006063/37006127/37006265/37006748/37006611/37006734/37006340/37006107/37006116/37006133	10	cg02478448/cg05137358/cg10158541/cg12571423/cg13060997/cg16422907/cg17495912/cg18348647/cg18948722/cg26984343	0.442595546	0.55143001	0.676126255	0.233530709	1.24E-12	2.91E-10
UTR5	CCT8L2	22	17073594	1	cg07443748	0.56714542	0.458567933	0.362340368	-0.204805052	3.14E-11	1.96E-09
UTR5	CD300LB	17	72527607	1	cg14075731	0.48006532	0.375505917	0.309218268	-0.170847052	4.79E-08	5.03E-07
UTR5	CD34	1	208084456/208084640/208084436	3	cg04292718/cg19591881/cg21455438	0.243883901	0.37397278	0.541480049	0.297596148	3.75E-09	5.88E-08
UTR5	CD38	4	15779999/15780011	2	cg02183671/cg24819835	0.21345019	0.439392783	0.552727605	0.339277415	6.81E-09	9.55E-08
UTR5	CDH10	5	24644878/24622791	2	cg00068408/cg12559474	0.188328965	0.251849002	0.367313545	0.17898458	0.000262701	0.001013078

UTR5	CDH2	18	25757211/25757438/25757202	3	cg09313439/cg12208258/cg13464915	0.291554003	0.338988742	0.495758347	0.204204344	1.28E-05	6.72E-05
UTR5	CDK3	17	73997417/73997287	2	cg10324640/cg15259920	0.61953952	0.50405365	0.439915459	-0.179624061	2.77E-08	3.12E-07
UTR5	CDO1	5	115152386/115152326	2	cg12880658/cg16265906	0.259336185	0.425709006	0.578118532	0.318782347	1.43E-08	1.77E-07
UTR5	CHAT	10	50822650/50822175/50820278/50821093/50818153/50824106/50817213/50822752/50821518/50820604/50822606/50817804/50818299/50821161/50818911/50818652/50821652/50821031/50817306/50820803/50822158/50819500/50817810/50818707/50821078/50821989/50821597/50822391/50817651/50821053	30	cg00378730/cg00586644/cg01295399/cg02336542/cg02728050/cg02949574/cg06108782/cg06300254/cg06737561/cg07637213/cg08327532/cg08996579/cg11389172/cg11811216/cg14008883/cg16494192/cg17994569/cg18199666/cg18592174/cg19535607/cg20405893/cg20457796/cg23987444/cg24109980/cg25155846/cg25207127/cg25609507/cg26473651/cg26813646/cg27591375	0.305213688	0.36475114	0.483683885	0.178470197	1.93E-07	1.69E-06
UTR5	CHODL	21	19617439	1	cg21350575	0.49047568	0.565352933	0.691209159	0.200733479	2.36E-11	1.69E-09
UTR5	CHST11	12	104851172	1	cg11425280	0.139467763	0.258243201	0.399273716	0.259805953	3.09E-06	1.90E-05
UTR5	CHST2	3	142838847/142839578/142838938/142839022/142838682	5	cg00995327/cg08774368/cg08858437/cg09638407/cg17815252	0.091566806	0.197840252	0.359917922	0.268351115	1.05E-06	7.33E-06
UTR5	CIDEA	18	12254690/12254365/12254377	3	cg00590620/cg15063116/cg20950011	0.29549468	0.381982467	0.449232986	0.153738306	6.59E-05	0.000292852
UTR5	CIDEC	3	9920572/9920615/9920535/9920698	4	cg05684195/cg07222243/cg16856722/cg21562208	0.511532463	0.413116494	0.330206432	-0.181326032	1.95E-11	1.56E-09
UTR5	CLDN10	13	96085969/96086023/96204978	3	cg08418978/cg22122715/cg25032595	0.53449756	0.578368461	0.717565459	0.183067899	3.35E-06	2.04E-05
UTR5	CLEC4G	19	7797031/7797052	2	cg16626670/cg19001261	0.557977435	0.466949472	0.397688936	-0.160288499	1.98E-10	6.12E-09

UTR5	CLEC4M	19	7828144	1	cg21372914	0.669008555	0.59473485	0.472102182	-0.196906373	7.13E-07	5.20E-06
UTR5	CLIP4	2	29338636/29339091/29338432/29344035/29343915/29339076/29339307	7	cg02774855/cg05038216/cg08808128/cg15377152/cg19955105/cg21972382/cg26052635	0.428963233	0.487489432	0.582906782	0.153943549	2.39E-08	2.74E-07
UTR5	CLLU1	12	92818240	1	cg13003571	0.857301425	0.694630783	0.60484675	-0.252454675	1.50E-09	2.79E-08
UTR5	CLPS	6	35765093	1	cg01670608	0.617996745	0.516676956	0.4087381	-0.209258645	3.20E-10	8.86E-09
UTR5	CLRN1	3	150690585/150661892	2	cg17808195/cg26062204	0.44536716	0.323139761	0.221471445	-0.223895715	7.69E-14	5.97E-11
UTR5	CLVS2	6	123318023/123317714/123317875	3	cg08737743/cg18210226/cg24728018	0.21959894	0.268317521	0.382576382	0.162977442	0.000169222	0.000684056
UTR5	CMTM3	16	66638320/66638599/66638702/66638407/66638396/66638438/66638412/66638293/66638433/66638210	10	cg00355909/cg03860859/cg06314872/cg06445928/cg08601917/cg08682544/cg09233013/cg23297477/cg26560414/cg26658728	0.110735955	0.28202328	0.429371915	0.31863596	1.64E-08	1.98E-07
UTR5	CNGA3	2	98963582/98966779/98963134/98964691/98962900/98964186/98962974/98963585/98963408/98964436/98986375/98963062/98963768	13	cg02076785/cg08327518/cg10583000/cg16404530/cg18404308/cg19370054/cg19671120/cg20581321/cg20727142/cg21488538/cg22241124/cg24778248/cg25045746	0.391719553	0.474355693	0.617267388	0.225547835	3.43E-10	9.23E-09
UTR5	CNRIP1	2	68546899/68546688/68547088/68547141/68547052/68546579	6	cg00020052/cg04020079/cg07594636/cg08157672/cg15313459/cg24171907	0.228849671	0.442461689	0.576738373	0.347888702	1.68E-09	3.06E-08
UTR5	CNTNAP2	7	145813915/145813946/145813494	3	cg07612562/cg16521917/cg24887139	0.255507874	0.340686325	0.489679141	0.234171267	1.27E-09	2.45E-08
UTR5	CNTNAP4	16	76311266	1	cg26577454	0.127225389	0.143523389	0.282300908	0.15507552	0.007209683	0.019692498
UTR5	COL20A1	20	61924802	1	cg15278386	0.77746356	0.678887878	0.611441955	-0.166021605	3.36E-06	2.04E-05
UTR5	COL23A1	5	178017376/178017260/178017300	3	cg20546002/cg22960186/cg25552435	0.102119519	0.195361626	0.290433439	0.18831392	0.000642465	0.002248355
UTR5	COL24A1	1	86622113	1	cg12644264	0.141704516	0.22757463	0.38242285	0.240718334	4.56E-05	0.000210214

UTR5	COL25A1	4	110223646/110223713/110223700/110223795/110223429/110223310/110223598/110223252	8	cg02441747/cg06468908/cg10074544/cg11935021/cg19039028/cg23501177/cg25088758/cg26005761	0.235074504	0.326068794	0.47518805	0.240113546	2.41E-08	2.76E-07
UTR5	COL4A2	13	110959668/110960198/110959650/110959647/110960186/110960177	6	cg01956420/cg15871127/cg17541528/cg20327444/cg22757824/cg24089600	0.340165647	0.420348213	0.536637432	0.196471785	4.77E-05	0.000218421
UTR5	COL5A1	9	137533851/137533949	2	cg14252519/cg26024843	0.33844217	0.423000736	0.548963739	0.210521569	8.50E-08	8.33E-07
UTR5	CPA4	7	129933014	1	cg01796223	0.58549644	0.538058156	0.374964577	-0.210531863	1.29E-06	8.76E-06
UTR5	CPEB1	15	83316640/83240550	2	cg04184836/cg06263843	0.148901132	0.251138156	0.324252168	0.175351036	7.43E-07	5.40E-06
UTR5	CPLX1	4	819151/819927/819227	3	cg11219917/cg11286023/cg12598635	0.181021881	0.240983096	0.343469158	0.162447276	1.33E-06	9.00E-06
UTR5	CPN1	10	101841416/101841526	2	cg06024295/cg16409039	0.478950655	0.402232547	0.324959941	-0.153990714	1.15E-08	1.47E-07
UTR5	CPNE8	12	39299364	1	cg01963134	0.257536033	0.263024829	0.455733981	0.198197949	0.003417397	0.010114626
UTR5	CPXM1	20	2781262/2781241	2	cg07113642/cg22304612	0.308282015	0.313874517	0.484651884	0.176369869	0.001250049	0.004091849
UTR5	CR1	1	207669576/207669544	2	cg05825950/cg18147366	0.083524104	0.161724098	0.2775816	0.194057496	2.55E-06	1.60E-05
UTR5	CR1L	1	207818493	1	cg24557058	0.25046316	0.380392383	0.543904982	0.293441822	1.09E-07	1.03E-06
UTR5	CRH	8	67089831/67090581/67090250/67089887	4	cg00603617/cg17305181/cg20329958/cg21878188	0.249373672	0.346949164	0.421601668	0.172227996	4.10E-07	3.24E-06
UTR5	CRISP1	6	49833706/49833873/49826783	3	cg04201526/cg10632722/cg16023304	0.711031133	0.617335211	0.50707143	-0.203959703	4.12E-10	1.05E-08
UTR5	CRISP3	6	49706534/49705127/49705602	3	cg09375907/cg24747763/cg27136847	0.679797375	0.601978243	0.527467833	-0.152329542	1.27E-08	1.60E-07
UTR5	CRISPLD1	8	75896975/75897115/75897310/75897297	4	cg01410472/cg02942845/cg05624932/cg13534450	0.29672969	0.373407267	0.516262164	0.219532474	5.66E-09	8.26E-08
UTR5	CRMP1	4	5890289	1	cg06256858	0.22577877	0.292910678	0.384815409	0.159036639	0.000164362	0.000666149
UTR5	CRYGD	2	208989209/208989248	2	cg17916432/cg22399133	0.566959963	0.630215325	0.747540161	0.180580199	1.86E-08	2.20E-07
UTR5	CSMD1	8	4852036	1	cg12258042	0.56875419	0.654968889	0.762469864	0.193715674	1.56E-09	2.88E-08
UTR5	CTNNA3	10	69424581/69419453/69425930/69455804/69426778	5	cg05668351/cg05766921/cg12768447/cg19996026/cg26297493	0.682086848	0.606561544	0.511104958	-0.17098189	3.80E-12	5.48E-10

UTR5	CUX2	12	111471963	1	cg09229912	0.293054925	0.402388711	0.519260705	0.22620578	2.99E-08	3.34E-07
UTR5	CXCR1	2	219030006/219030123/219030898/219030752/219030830/219031519/219031640	7	cg00832199/cg13048967/cg13519373/cg15768138/cg15908708/cg18467756/cg20025658	0.597961272	0.494546808	0.412102536	-0.185858736	4.95E-11	2.52E-09
UTR5	CYP1B1	2	38303138/38302587/38302674/38303145/38302892/38303219	6	cg07078841/cg11751707/cg16439198/cg18451022/cg23151000/cg25856383	0.199766348	0.279891665	0.419449856	0.219683507	9.82E-07	6.91E-06
UTR5	CYP2C18	10	96443383	1	cg14196507	0.65630379	0.492147161	0.376205891	-0.280097899	5.45E-11	2.67E-09
UTR5	CYYR1	21	27945413	1	cg19678828	0.535953395	0.613151833	0.695989627	0.160036232	1.28E-10	4.43E-09
UTR5	DAO	12	109273999/109274241	2	cg12592321/cg22801690	0.561895295	0.470840789	0.365507909	-0.196387386	4.90E-10	1.18E-08
UTR5	DCC	18	49867117/49866548/49867126	3	cg06261937/cg21669679/cg25266232	0.332915208	0.41640653	0.524336224	0.191421016	5.94E-07	4.44E-06
UTR5	DCT	13	95131809	1	cg05807291	0.63958114	0.60577005	0.481196077	-0.158385063	3.11E-05	0.000148962
UTR5	DDIT4L	4	101111261/101111314/101111147/101111427	4	cg12829717/cg18603154/cg18714412/cg27062369	0.150814596	0.191819361	0.359491989	0.208677393	0.000239496	0.000934337
UTR5	DDX25	11	125774406/125774311	2	cg19792599/cg22097425	0.42813761	0.496372142	0.590836425	0.162698815	6.87E-10	1.52E-08
UTR5	DGKG	3	186078568/186080015/186075879/186078847/186079142/186078640/186079367	7	cg00404400/cg05287817/cg07138366/cg10730421/cg15055817/cg15548101/cg16657397	0.351788577	0.421841382	0.522981659	0.171193082	2.57E-08	2.93E-07
UTR5	DKK2	4	107957265/107957362/107956955/107957430/107957072	5	cg01471384/cg01962428/cg08341316/cg13139972/cg26955835	0.182295618	0.342343733	0.467727194	0.285431576	2.41E-08	2.76E-07
UTR5	DLEU7	13	51417846	1	cg20170533	0.44724189	0.564400672	0.69818495	0.25094306	5.19E-11	2.59E-09
UTR5	DLK1	14	101193397	1	cg02874376	0.4214206	0.485681622	0.585657955	0.164237355	1.88E-06	1.22E-05
UTR5	DNAH11	7	21582834	1	cg05285902	0.182962681	0.209432983	0.381891586	0.198928906	0.000344154	0.00128718
UTR5	DNAH9	17	11786412/11788016	2	cg12626882/cg14135174	0.500866753	0.436152728	0.339406835	-0.161459918	2.09E-09	3.63E-08
UTR5	DNAJA4	15	78556512/78556534/78557094/78556502	4	cg08594606/cg09214398/cg13747794/cg23366752	0.6657829	0.575533097	0.497727018	-0.168055882	2.43E-10	7.23E-09
UTR5	DNAJC5B	8	66955031/66946188	2	cg09312407/cg17373345	0.416816328	0.325444219	0.262663945	-0.154152382	1.28E-10	4.43E-09
UTR5	DNASE2B	1	84876625	1	cg24868292	0.522377195	0.38675595	0.301916732	-0.220460463	8.45E-13	2.38E-10

UTR5	DNM3	1	171810778	1	cg06211893	0.092526396	0.164498276	0.24640649	0.153880094	0.011113431	0.028801593
UTR5	DNMT3L	21	45681834	1	cg15635600	0.53082813	0.4463379	0.377105464	-0.153722666	1.90E-09	3.33E-08
UTR5	DOK5	20	53092474/53092334	2	cg00055557/cg20107395	0.245197855	0.316964298	0.471235526	0.226037671	6.40E-06	3.61E-05
UTR5	DOK6	18	67068457	1	cg15916399	0.281403135	0.369422233	0.461386509	0.179983374	3.06E-12	4.87E-10
UTR5	DPP6	7	153584582/154002372/153584597/153584748/153584609	5	cg06495961/cg13544851/cg14523847/cg24011260/cg27032232	0.464160719	0.551574422	0.688293491	0.224132772	3.32E-10	9.10E-09
UTR5	DQX1	2	74753358/74753281/74753334/74753312	4	cg01410453/cg02034222/cg06151718/cg24544105	0.643364703	0.502117763	0.42691287	-0.216451834	1.56E-09	2.88E-08
UTR5	DSPP	4	88530371	1	cg09590733	0.63317119	0.475644672	0.391535282	-0.241635908	2.66E-08	3.03E-07
UTR5	DTX3	12	57998762/57998786/57998656	3	cg11654179/cg17730484/cg19217692	0.092657477	0.100876562	0.279461681	0.186804204	0.001536626	0.00491451
UTR5	DYDC2	10	82119370/82116571/82117089/82119561/82117119/82116596/82117257	7	cg00649199/cg01527459/cg03701427/cg05905523/cg06411551/cg12640394/cg17703212	0.45025132	0.565637193	0.67035718	0.22010586	5.01E-07	3.83E-06
UTR5	EBF1	5	158526693/158526614/158526642	3	cg04217450/cg05530568/cg17009297	0.283888828	0.337726565	0.504129801	0.220240973	3.94E-06	2.34E-05
UTR5	ECEL1	2	233351921/233352148/233351564/233351609/233351525/233351735	6	cg01228134/cg04593426/cg07503630/cg09736968/cg19047707/cg27023555	0.340520554	0.387458512	0.499138452	0.158617898	3.14E-05	0.000150396
UTR5	EDNRA	4	148402748/148402408/148402899/148402128/148402399/148405579/148402574	7	cg03580568/cg04045079/cg05102394/cg12009023/cg20557687/cg23865342/cg24789447	0.415082923	0.487928112	0.590800429	0.175717506	1.23E-06	8.43E-06

UTR5	EDNRB	13	78493182/78493657/78493100/78493561/78493012/78493066/78493704/78494064/78493878/78494330/78549582/78493651/78494010/78493313/78494272/78494442/78493294/78493349/78493590/78493920/78493305/78493126/78493128/78495961/78492922/78493854/78549657/78493229/78493712/78494171/78493297/78493365/78494067/78493203/78493874/78493564/78493232/78493666/78492916/78493671/78493215/78493205/78493282/78493958/78494462/78493631/78493583/78492730	48	cg01910869/cg02147695/cg04390523/cg06057566/cg06179060/cg06971129/cg07035515/cg07974719/cg08321129/cg08634041/cg08983760/cg09152886/cg09786383/cg10016380/cg10764762/cg11074192/cg12602112/cg12847373/cg12935136/cg12983394/cg13434989/cg13818654/cg15836660/cg16203262/cg16571983/cg16739796/cg18003135/cg18032190/cg18568990/cg19111971/cg19650157/cg19742055/cg19759502/cg19916212/cg21364111/cg21675115/cg22310279/cg22541679/cg23316360/cg23494140/cg23702615/cg23766591/cg24236409/cg24745738/cg24942919/cg25717994/cg26022015/cg26622320	0.366351187	0.458914464	0.565985089	0.199633903	4.80E-10	1.17E-08
UTR5	EFCAB1	8	49647734/49647809/49647717/49647833	4	cg08258526/cg11469098/cg22836229/cg23562261	0.371245304	0.41917724	0.54160763	0.170362326	2.10E-05	0.000104676
UTR5	EFEMP1	2	56150341/56150478/56150925/56150475/56150255/56150675/56150549/56149825	8	cg03122624/cg05385513/cg08130988/cg16100120/cg20786074/cg24719005/cg25046074/cg25711779	0.317840707	0.370426108	0.494984797	0.17714409	5.95E-07	4.44E-06

UTR5	EHD3	2	31457287/31457061/31456966/31457058/31456964/31457043	6	cg01163837/cg13795465/cg15355118/cg24743639/cg25428398/cg25840208	0.113760179	0.296217255	0.397088917	0.283328738	1.09E-06	7.60E-06
UTR5	EHF	11	34642885/34654233	2	cg18414381/cg18560551	0.45383171	0.351679986	0.27473027	-0.17910144	9.82E-12	9.95E-10
UTR5	ELF3	1	201979938/201979846/201979764	3	cg02076020/cg07897871/cg26328757	0.58622302	0.48765833	0.401774527	-0.184448493	1.19E-10	4.18E-09
UTR5	ELOVL2	6	11044558/11044541	2	cg05446010/cg22143569	0.028742374	0.092247237	0.198802878	0.170060504	7.53E-05	0.000329781
UTR5	EMB	5	49737178/49737182/49736988	3	cg03179043/cg08722065/cg18110165	0.230660551	0.447511756	0.520510282	0.289849731	2.05E-12	3.76E-10
UTR5	ENPP6	4	185139078	1	cg08493463	0.55805469	0.491997322	0.384458145	-0.173596545	1.94E-11	1.56E-09
UTR5	EPHA10	1	38230769/38230779	2	cg12128017/cg20510207	0.302970382	0.384643194	0.540645805	0.237675423	1.60E-06	1.06E-05
UTR5	EPHA5	4	66535575	1	cg02463418	0.40428126	0.519426406	0.618419409	0.214138149	5.02E-05	0.000228442
UTR5	EPS8L3	1	110306507/110305591	2	cg00515905/cg23957643	0.531275918	0.421435061	0.3361768	-0.195099118	1.09E-11	1.07E-09
UTR5	EPYC	12	91396741	1	cg13548607	0.853448815	0.784587617	0.691189509	-0.162259306	0.000546175	0.001942058
UTR5	EVC	4	5713100/5712937/5713005	3	cg16418810/cg17460447/cg21210758	0.246805393	0.344737494	0.514194583	0.26738919	1.46E-09	2.72E-08
UTR5	EVC2	4	5708474/5710411/5710767/5710407/5709858/5710403/5706354/5710319/5710761/5710372/5710016/5710098/5710337	13	cg01290904/cg01834022/cg03278643/cg05542338/cg05571820/cg06426416/cg07421005/cg14654886/cg17242664/cg17255450/cg22290744/cg23665381/cg27434509	0.446532783	0.50251781	0.602783279	0.156250495	3.06E-06	1.88E-05
UTR5	EYA4	6	133563342/133563764/133564066/133564466/133564546/133562494/133563868/133584189/133563382/133564578/133576214/133563532/133562776/133563779/133562774	15	cg01401376/cg01805282/cg06393563/cg06764333/cg08917489/cg14270292/cg14817541/cg15246805/cg17722675/cg18714224/cg20422212/cg21607030/cg24176563/cg24675448/cg26656135	0.27335734	0.319411567	0.427561952	0.154204613	7.20E-06	4.01E-05
UTR5	FADS2	11	61595807	1	cg25303599	0.0880115	0.159008576	0.40492206	0.31691056	1.27E-05	6.67E-05
UTR5	FAIM2	12	50297588/50297581	2	cg16115588/cg27188703	0.355989765	0.464132031	0.593607943	0.237618178	2.37E-07	2.01E-06

UTR5	FAM102A	9	130712990	1	cg14080888	0.56706459	0.456293178	0.4012331	-0.16583149	6.72E-10	1.50E-08
UTR5	FAM115A	7	143579698/143580147/143582146/143579951/143579665/143582308/143582630/143584683/143582499/143598702/143596849/143599078	12	cg01030534/cg02245020/cg02266399/cg03225210/cg03514404/cg09671837/cg09846875/cg11067338/cg16807089/cg19548738/cg22280402/cg26309303	0.366802654	0.4564707	0.546040056	0.179237401	6.39E-11	2.95E-09
UTR5	FAM123A	13	25745768/25745784	2	cg14880499/cg22029275	0.23601127	0.382200712	0.570263075	0.334251805	4.43E-10	1.10E-08
UTR5	FAM124B	2	225266656/225266580	2	cg00698413/cg18890556	0.50715081	0.594005306	0.689245982	0.182095172	2.90E-09	4.71E-08
UTR5	FAM153A	5	177196865	1	cg06965744	0.734184735	0.624720967	0.507362886	-0.226821849	1.15E-09	2.28E-08
UTR5	FAM155A	13	108519213/108518955/108519318	3	cg04607400/cg05383490/cg19839798	0.331693743	0.384843347	0.596236664	0.264542921	5.17E-08	5.37E-07
UTR5	FAM159B	5	63986392	1	cg10981909	0.354039175	0.485957006	0.671355627	0.317316452	8.47E-10	1.81E-08
UTR5	FAM162B	6	117086850	1	cg07641160	0.204753153	0.284185165	0.499210776	0.294457623	1.50E-06	1.00E-05
UTR5	FAM183A	1	43613606	1	cg20991612	0.26131836	0.304062056	0.435364445	0.174046085	1.45E-06	9.69E-06
UTR5	FAM196B	5	169407472/169404742/169331882/169350021/169333109/169407439/169398708/169322052/169373347/169390597/169361351	11	cg00357551/cg00547068/cg03850213/cg09011473/cg09143964/cg12688234/cg14781262/cg15102430/cg22809017/cg23230904/cg27068707	0.695606819	0.605029072	0.520006523	-0.175600295	1.58E-09	2.90E-08
UTR5	FAM26F	6	116783000	1	cg15172529	0.299070495	0.388476994	0.495076323	0.196005828	8.96E-05	0.00038672
UTR5	FAM43B	1	20879029/20879286/20879296	3	cg03754311/cg05771261/cg20404387	0.292133723	0.381013617	0.551905216	0.259771493	4.42E-08	4.70E-07
UTR5	FAM55D	11	114453402/114466093	2	cg18384712/cg21833776	0.483290695	0.364575761	0.295816255	-0.18747444	4.74E-10	1.16E-08
UTR5	FAM71C	12	100041844/100041683	2	cg10635722/cg23170057	0.669029785	0.575467478	0.502464909	-0.166564876	1.22E-07	1.13E-06
UTR5	FAM72B	1	120839380/120839385/120839390	3	cg00198436/cg07236150/cg09169215	0.066481005	0.145489607	0.294410058	0.227929053	5.58E-07	4.20E-06
UTR5	FBN1	15	48937856/48937213/48937319/48937958	4	cg03535663/cg04125371/cg12975862/cg19908534	0.144832838	0.280812161	0.438539708	0.293706871	5.15E-07	3.92E-06
UTR5	FBN2	5	127873397/127873552/127873711	3	cg12940822/cg17958315/cg25084878	0.205810473	0.311272031	0.51417982	0.308369348	5.35E-07	4.05E-06

UTR5	FBXL7	5	15500714	1	cg06577205	0.344421575	0.375808717	0.528633736	0.184212161	0.000116015	0.000488555
UTR5	FBXO39	17	6679564/6679597/6679781	3	cg02374745/cg20723355/cg21985295	0.475388303	0.55881715	0.706998377	0.231610075	4.87E-10	1.18E-08
UTR5	FCRL3	1	157670328	1	cg17134153	0.557148105	0.464769817	0.338287986	-0.218860119	1.30E-07	1.20E-06
UTR5	FEZF2	3	62358825/62358610/62358980	3	cg14651453/cg18902742/cg19629292	0.233843444	0.299242695	0.412969885	0.179126441	3.96E-05	0.00018541
UTR5	FGD5	3	14860482	1	cg04943612	0.79655157	0.702215333	0.617282536	-0.179269034	8.04E-08	7.95E-07
UTR5	FGF12	3	192126244/192445380/192126165/192126584/192444985/192445040/192445202/192126825/192126023/192445307/192126742	11	cg00386551/cg02992632/cg04003850/cg04350913/cg04988476/cg07127762/cg15543551/cg16209795/cg18780257/cg19507527/cg21197774	0.226401814	0.310071935	0.455091224	0.22868941	3.10E-08	3.44E-07
UTR5	FGF3	11	69633716/69633959	2	cg15020425/cg17277529	0.226332898	0.26778208	0.377243639	0.150910741	5.81E-05	0.000260856
UTR5	FGF4	11	69590090/69590113	2	cg19831575/cg23964057	0.151178155	0.239285329	0.42840766	0.277229506	7.18E-07	5.24E-06
UTR5	FGF5	4	81187798/81187906	2	cg02002231/cg14197923	0.106380913	0.196157855	0.391203448	0.284822535	2.36E-05	0.000115707
UTR5	FGFBP1	4	15939889/15939862	2	cg09565002/cg13929970	0.54022117	0.435373733	0.351539527	-0.188681643	1.67E-10	5.40E-09
UTR5	FHL5	6	97010903/97010439	2	cg03043157/cg23978322	0.533942893	0.4290563	0.364579459	-0.169363433	7.48E-09	1.02E-07
UTR5	FIBIN	11	27015991/27015872/27015656/27015808/27015813	5	cg01649773/cg04622802/cg07042914/cg07777008/cg15621260	0.483024114	0.554100422	0.667452933	0.184428819	4.00E-09	6.21E-08
UTR5	FLG2	1	152331529	1	cg20694572	0.7220716	0.610989517	0.441182641	-0.280888959	1.21E-08	1.54E-07
UTR5	FLJ20184	4	106473806	1	cg27126442	0.54336524	0.439075839	0.345794486	-0.197570754	2.09E-09	3.63E-08
UTR5	FLJ23834	7	105603743	1	cg02661623	0.676365055	0.588094428	0.513351982	-0.163013073	3.30E-05	0.000156784
UTR5	FNDC1	6	159590578	1	cg14971597	0.174748926	0.286958762	0.397212366	0.222463441	1.04E-06	7.28E-06
UTR5	FOLH1	11	49230168/49230036	2	cg14775560/cg25346915	0.291977923	0.319281246	0.501149359	0.209171437	1.49E-05	7.72E-05
UTR5	FOLH1B	11	89392829	1	cg02915544	0.59441058	0.412695106	0.265445155	-0.328965425	2.35E-14	3.93E-11
UTR5	FOXD4L1	2	114256781/114256673	2	cg10602757/cg16708281	0.30246558	0.351444594	0.485350636	0.182885056	7.30E-07	5.31E-06
UTR5	FOXE1	9	100615796	1	cg19292008	0.15018847	0.258647846	0.361871411	0.211682941	1.63E-05	8.34E-05
UTR5	FOXG1	14	29236323/29236476	2	cg10300684/cg10828337	0.355856943	0.45415355	0.627157636	0.271300694	1.32E-09	2.51E-08
UTR5	FOXI1	5	169532943	1	cg16137007	0.638671515	0.549498461	0.479292482	-0.159379033	4.34E-10	1.09E-08
UTR5	FOXL1	16	86612312	1	cg00584450	0.517955895	0.582548389	0.693978082	0.176022187	3.16E-11	1.96E-09

UTR5	FOXN4	12	109747000	1	cg22326903	0.068558682	0.145944608	0.267626035	0.199067353	0.000908907	0.003078323
UTR5	FPR1	19	52254222/52253373	2	cg15811427/cg22152605	0.613606575	0.508523403	0.405170659	-0.208435916	2.71E-08	3.07E-07
UTR5	FPR2	19	52265748/52268814/52266295/52266642/52266482	5	cg03195665/cg04554195/cg12306057/cg14203721/cg16726204	0.592783775	0.494880604	0.417787033	-0.174996742	1.57E-09	2.90E-08
UTR5	FRK	6	116381609/116381904	2	cg16176600/cg26893134	0.429479963	0.340561322	0.272251695	-0.157228267	8.52E-12	9.11E-10
UTR5	FSCN3	7	127233742	1	cg16725721	0.80322332	0.700517317	0.613380168	-0.189843152	4.00E-07	3.17E-06
UTR5	FSTL1	3	120169783/120169821	2	cg03424342/cg15109851	0.275980793	0.326929212	0.451583101	0.175602309	0.000543829	0.001934274
UTR5	FUT3	19	5846599/5851255/5846552/5846586/5851465	5	cg14529758/cg17471102/cg19263069/cg19442149/cg26899156	0.552526154	0.425135961	0.364135888	-0.188390266	2.45E-07	2.07E-06
UTR5	FXYD3	19	35606877/35606732/35607180/35607221	4	cg00480115/cg02633817/cg02704949/cg21304163	0.51582892	0.421460122	0.329591473	-0.186237447	2.35E-11	1.69E-09
UTR5	FZD10	12	130647213/130647289	2	cg15928093/cg27232145	0.358142533	0.401554761	0.524662725	0.166520193	3.32E-09	5.28E-08
UTR5	GABRA5	15	27113913/27111911/27111949/27112902/27112197/27111940/27112430/27113511/27113322/27112119/27113459/27111993/27111884/27112287/27112305/27112049	16	cg00329728/cg01378667/cg03325535/cg03462380/cg05044185/cg10318222/cg10652393/cg11786657/cg14508508/cg15665646/cg17481117/cg18255240/cg20005743/cg21276413/cg21402921/cg22523852	0.378295317	0.453643683	0.563795061	0.185499744	3.59E-11	2.05E-09
UTR5	GABRA6	5	161112787	1	cg22672790	0.391572255	0.294535011	0.191274473	-0.200297782	4.56E-11	2.39E-09
UTR5	GABRB3	15	27018874	1	cg01690182	0.349999235	0.444493578	0.569842055	0.21984282	1.05E-08	1.37E-07
UTR5	GAD2	10	26505442/26505503/26505440/26505245	4	cg04599297/cg09056181/cg11328303/cg15850954	0.277074423	0.44180043	0.613517622	0.336443198	2.92E-10	8.28E-09
UTR5	GAL3ST3	11	65816027/65813790/65816521/65816646/65816642/65816463	6	cg02839195/cg11287219/cg14780632/cg15916004/cg21238818/cg24960947	0.536715678	0.627212439	0.711567258	0.17485158	5.05E-10	1.20E-08

UTR5	GALNT13	2	154729200/154728735/154728468/154729059/154729917/154729995/154729622/154730326/154730157/154729189/154729763	11	cg01011748/cg10033761/cg12079322/cg13101087/cg14630357/cg17130964/cg18118685/cg23762263/cg24412079/cg25703346/cg26660002	0.32878657	0.345853486	0.537751066	0.208964496	3.16E-08	3.50E-07
UTR5	GALNT14	2	31361104/31361371	2	cg05714219/cg24597158	0.05724639	0.146009878	0.298807256	0.241560866	8.02E-05	0.000349575
UTR5	GALNTL5	7	151664041/151653523/151664190/151664071/151653890	5	cg01760103/cg11727304/cg13835670/cg21565299/cg25310567	0.547648193	0.425118525	0.322293601	-0.225354591	1.43E-13	7.73E-11
UTR5	GALNTL6	4	172734776/172734843/172734718	3	cg03413465/cg15533075/cg26588943	0.276865617	0.363287785	0.517858048	0.240992432	8.58E-10	1.83E-08
UTR5	GALR1	18	74962216/74962133/74962369	3	cg01178451/cg03502002/cg04534765	0.49479416	0.546031383	0.688295793	0.193501633	4.55E-07	3.52E-06
UTR5	GATA3	10	8097183/8097354/8096818/8097331/8096669/8096991	6	cg10008757/cg13409449/cg14098681/cg14327531/cg15330117/cg18599069	0.227427828	0.243942861	0.389058727	0.161630899	0.000898001	0.003046376
UTR5	GATA4	8	11562367/11562685/11565530/11565277/11561724/11561728/11562155/11565725/11563023/11565351	10	cg00090147/cg03007522/cg06704518/cg14487356/cg14666113/cg18283386/cg21073927/cg24646414/cg24738627/cg25216696	0.304089279	0.399121798	0.562679571	0.258590292	3.77E-10	9.85E-09
UTR5	GATA5	20	61051021/61050885/61050914	3	cg12664464/cg14831174/cg16919517	0.269452062	0.297427782	0.426344664	0.156892603	0.00203124	0.006314098
UTR5	GCM1	6	53013589/53011657	2	cg26023389/cg27550998	0.55671574	0.423751589	0.338603055	-0.218112685	4.02E-12	5.61E-10
UTR5	GCNT3	15	59904009/59908160/59908622/59909035/59910436/59909007/59910408/59908999/59908652/59907427	10	cg02073465/cg05289698/cg05641048/cg06817269/cg09407660/cg11020468/cg19285777/cg22112110/cg23877385/cg25311764	0.626550447	0.541618708	0.461402301	-0.165148146	8.20E-09	1.11E-07
UTR5	GDF6	8	97172961	1	cg00421139	0.25141105	0.384316417	0.55991715	0.3085061	3.78E-08	4.10E-07

UTR5	GFRA1	10	118032626/118032355/118032033/118031864/118032892/118032872/118033115/118031950/118032081/118032879/118032330/118031870/118032948/118031632/118032987/118031654/118032905	17	cg03503087/cg04983142/cg06039355/cg06298519/cg08194313/cg13346013/cg13890706/cg17256532/cg18044663/cg18672939/cg19236679/cg19485539/cg23898073/cg24792682/cg25022687/cg27034576/cg27341926	0.212450643	0.305961437	0.49126561	0.278814968	7.29E-06	4.06E-05
UTR5	GFRA2	8	21645807/21646174/21645923/21645870	4	cg00902636/cg06067208/cg09459291/cg14633783	0.166316193	0.209752566	0.325174522	0.158858329	0.002345091	0.007179961
UTR5	GIF	11	59612937	1	cg01282204	0.759301325	0.533209667	0.396963041	-0.362338284	3.62E-14	3.95E-11
UTR5	GK2	4	80329368	1	cg01807825	0.51540091	0.413789967	0.324463386	-0.190937524	1.54E-10	5.04E-09
UTR5	GLB1L3	11	134146324	1	cg18375494	0.065705825	0.098374453	0.246427984	0.180722159	0.003831347	0.011186559
UTR5	GLDC	9	6645686	1	cg17009433	0.119558025	0.22920865	0.331112304	0.211554279	0.000997096	0.003334385
UTR5	GLIPR1L1	12	75728469/75728471	2	cg21087137/cg24234651	0.374702302	0.486920539	0.57722738	0.202525077	2.41E-08	2.76E-07
UTR5	GLRA3	4	175750373/175750109/175750280	3	cg05424071/cg21094669/cg27517702	0.22069827	0.294578594	0.467080559	0.24638229	8.08E-08	7.98E-07
UTR5	GLRB	4	157997526/157997363/157998201/157997750/157997554/157997880/157997367/157997360	8	cg08712866/cg09688546/cg10146199/cg10999932/cg11163620/cg13828794/cg16269431/cg22065614	0.223893995	0.308681188	0.499797991	0.275903996	3.38E-07	2.75E-06
UTR5	GLTPD2	17	4692287	1	cg03704673	0.617921565	0.51379225	0.435410741	-0.182510824	1.98E-09	3.46E-08
UTR5	GNAL	18	11689284/11752215/11751566/11689206/11689218/11752089	6	cg09331011/cg10068300/cg14612428/cg15616946/cg15653282/cg25958283	0.257031918	0.333332644	0.428672075	0.171640157	1.43E-11	1.27E-09
UTR5	GPC6	13	93879544/93879361/93879670/93879303	4	cg12989574/cg16792800/cg18058689/cg19824907	0.36752897	0.43732878	0.647716442	0.280187472	5.95E-09	8.62E-08
UTR5	GPR115	6	47666329/47668044	2	cg14105536/cg20457051	0.641221538	0.496847425	0.380031241	-0.261190297	4.04E-12	5.61E-10

UTR5	GPR133	12	131438788/131438737/131438527/131438626/131438523/131438540	6	cg02717046/cg19642421/cg20712263/cg21502466/cg22882539/cg23379751	0.256900721	0.378264591	0.463866477	0.206965756	1.67E-06	1.10E-05
UTR5	GPR139	16	20084944/20085047	2	cg00938050/cg01126567	0.314935875	0.394364972	0.519040991	0.204105116	1.19E-08	1.51E-07
UTR5	GPR183	13	99959606	1	cg16131748	0.503339165	0.602018767	0.683022432	0.179683267	4.96E-11	2.52E-09
UTR5	GPR6	6	110300308	1	cg00613752	0.57212571	0.640209217	0.740149914	0.168024204	3.00E-09	4.86E-08
UTR5	GPR75	2	54085310/54087008/54087028/54086854/54084393/54087066	6	cg00933224/cg01876338/cg14832904/cg19592277/cg19911257/cg19961545	0.681594619	0.769199809	0.854285538	0.172690919	1.54E-13	7.95E-11
UTR5	GPR83	11	94134446/94134580	2	cg03345037/cg07586272	0.23907202	0.323759189	0.475437886	0.236365866	3.15E-07	2.59E-06
UTR5	GPR85	7	112726089/112726659/112726653/112726547/112726774/112726802/112725899/112726538/112726549/112726541/112726869/112726396/112727423/112727506	14	cg00851377/cg01491795/cg03119639/cg06931815/cg08220649/cg10129816/cg10336790/cg13251269/cg20019019/cg20513548/cg21852992/cg24310431/cg24786658/cg25263801	0.24230561	0.315573642	0.397332842	0.155027232	0.000176976	0.000711689
UTR5	GPR88	1	101004474/101004472	2	cg10298815/cg14855972	0.32309361	0.389216217	0.523344336	0.200250726	0.000317051	0.001196997
UTR5	GPX2	14	65409452	1	cg09643186	0.557926885	0.447100383	0.363442959	-0.194483926	5.04E-11	2.54E-09
UTR5	GREB1L	18	18822817/18822994/18822637/18822579/18823249/18822927/18823107/18823509	8	cg01562349/cg01655831/cg03735496/cg05109245/cg07653946/cg12473406/cg14034197/cg26518100	0.229694722	0.270573745	0.380273234	0.150578513	0.003667696	0.010768534
UTR5	GRIA1	5	152870258	1	cg17020834	0.227224285	0.232838994	0.383978405	0.15675412	9.80E-05	0.000417762
UTR5	GRIA4	11	105481306/105481317/105480979/105481319/105481322/105481283/105481509/105481406/105481292	9	cg00343633/cg03225817/cg03243226/cg04747226/cg07972135/cg15603568/cg19343464/cg21217024/cg23559689	0.280322231	0.413955547	0.601997234	0.321675003	1.68E-08	2.02E-07
UTR5	GRIK1	21	31312091/31311842	2	cg09542111/cg23475371	0.298044425	0.394232864	0.496377848	0.198333423	1.11E-07	1.04E-06

UTR5	GRIK2	6	101846916/101846905/101847058/101846872/101846967	5	cg10591607/cg18193094/cg21635870/cg24301620/cg26316946	0.430119524	0.531147041	0.650800781	0.220681257	1.78E-10	5.64E-09
UTR5	GRIN2A	16	10276375/10276092/10274847/10275410/10274317/10274315/10275934/10275767/10276383/10274632/10276580/10276081/10274415/10276119/10274963/10276600	16	cg00287829/cg02585344/cg03762483/cg03764381/cg04498349/cg04761722/cg05406943/cg06193383/cg06758670/cg06922606/cg09239744/cg09461286/cg15134033/cg16904585/cg18391758/cg24764979	0.195926575	0.243702152	0.378993939	0.183067364	2.18E-06	1.39E-05
UTR5	GRM1	6	146348913/146350434/146350618/146350230/146350131/146350585/146350041/146350346/146349488/146349527/146348901/146348890/146349312/146349638	14	cg03478199/cg06015218/cg07218663/cg08076125/cg08958294/cg09558195/cg13371951/cg13722123/cg14526297/cg17199007/cg19512268/cg21725954/cg23696752/cg26256158	0.323994011	0.399514664	0.511344383	0.187350372	2.77E-09	4.55E-08
UTR5	GRM7	3	6902824/6902845/6903019	3	cg17892178/cg18863595/cg27199820	0.435273323	0.5295267	0.687263277	0.251989955	7.76E-13	2.31E-10
UTR5	GRP	18	56887410/56887404	2	cg17093795/cg27338487	0.37645826	0.466691264	0.559288411	0.182830151	4.00E-07	3.17E-06
UTR5	GSDMB	17	38074149/38074070/38074753	3	cg10057218/cg12360886/cg16822095	0.702448817	0.569832328	0.463117958	-0.239330859	9.63E-09	1.27E-07
UTR5	GSDMC	8	130799007/130799093	2	cg05316065/cg26073844	0.558587478	0.434942542	0.3371831	-0.221404378	1.51E-10	4.97E-09
UTR5	GSTA3	6	52773711/52774462	2	cg02075593/cg12445586	0.717886065	0.618936706	0.5224458	-0.195440265	5.95E-09	8.62E-08
UTR5	GSX1	13	28366814	1	cg26609631	0.432714115	0.458144839	0.595039364	0.162325249	6.08E-07	4.52E-06
UTR5	GUCA2B	1	42619118	1	cg15976404	0.652472495	0.540815244	0.454725536	-0.197746959	1.61E-09	2.94E-08
UTR5	GUCY1B3	4	156680256/156680207	2	cg03584891/cg16700392	0.418848618	0.499077622	0.617188584	0.198339967	1.79E-09	3.19E-08
UTR5	GUCY2D	17	7906042/7906098	2	cg10020356/cg25465406	0.24352308	0.337549628	0.4396389	0.19611582	0.000274942	0.001055036
UTR5	GYPC	2	127413831	1	cg15975865	0.30371499	0.489046279	0.648228845	0.344513855	2.22E-08	2.57E-07
UTR5	GYS2	12	21757759	1	cg04184278	0.744283985	0.595737211	0.455469341	-0.288814644	4.46E-10	1.11E-08

UTR5	HAND2	4	174451251/174450722/174451141	3	cg02774439/cg10541864/cg25102370	0.327494068	0.395729665	0.526202071	0.198708003	1.46E-05	7.56E-05
UTR5	HAPLN4	19	19373566/19373575	2	cg01297721/cg22694191	0.33803368	0.375740819	0.525827877	0.187794197	0.000405526	0.001488007
UTR5	HBE1	11	5291298/5291214	2	cg03306956/cg16279158	0.5609776	0.500748483	0.367565418	-0.193412182	9.25E-09	1.23E-07
UTR5	HBQ1	16	230343/230341	2	cg02401454/cg07703401	0.32724683	0.338138094	0.520321184	0.193074354	4.73E-05	0.000216952
UTR5	HCRTR2	6	55039232/55039259/55039382/55039295	4	cg10725720/cg13948987/cg17240815/cg27596495	0.282031535	0.328409048	0.488993624	0.206962089	1.05E-07	1.00E-06
UTR5	HECW2	2	197454821/197456953/197457277/197456534/197399559/197328556	6	cg12949542/cg13308279/cg13821072/cg16616765/cg20197814/cg23490246	0.5250839	0.403734391	0.307906789	-0.217177111	6.49E-13	2.12E-10
UTR5	HEPHL1	11	93754390	1	cg18109485	0.454738565	0.361792817	0.26383015	-0.190908415	1.92E-12	3.63E-10
UTR5	HGD	3	120401058/120401095	2	cg04546097/cg14527029	0.534031683	0.436645867	0.35923247	-0.174799212	2.45E-10	7.26E-09
UTR5	HK3	5	176325836/176323419	2	cg14709481/cg26455624	0.6726854	0.599768147	0.521455927	-0.151229473	4.05E-09	6.27E-08
UTR5	HMGCS2	1	120311542	1	cg13976683	0.542833315	0.417908067	0.341307155	-0.20152616	4.79E-11	2.47E-09
UTR5	HMX2	10	124907708	1	cg19909787	0.159048506	0.238984549	0.40401015	0.244961644	4.69E-06	2.73E-05
UTR5	HNF4A	20	43029994/43029992/43029997	3	cg07064544/cg11542165/cg21058973	0.61706196	0.507987878	0.420796634	-0.196265326	5.76E-10	1.33E-08
UTR5	HOXA2	7	27142204/27142275/27142200	3	cg06055873/cg20747380/cg26069745	0.427260785	0.507594139	0.626423341	0.199162556	1.51E-05	7.80E-05
UTR5	HOXB8	17	46692248/46692168	2	cg15539420/cg22913933	0.481826075	0.346464283	0.284562341	-0.197263734	9.15E-05	0.000394069
UTR5	HOXC9	12	54393966/54393971	2	cg05069426/cg12071536	0.28800951	0.364417672	0.572085011	0.284075501	5.59E-07	4.21E-06
UTR5	HRNR	1	152196597/152196415/152196106	3	cg07252308/cg11344574/cg26020982	0.661658033	0.553834609	0.482814003	-0.17884403	8.79E-14	6.42E-11
UTR5	HS3ST3A1	17	13504687/13505174/13504472	3	cg03436461/cg07480567/cg22583981	0.316983335	0.388993817	0.490615809	0.173632474	6.13E-05	0.000274353
UTR5	HS3ST4	16	25703528	1	cg27014135	0.138987107	0.142758383	0.304199981	0.165212874	0.011901243	0.030556047
UTR5	HSPB2	11	111783499	1	cg12947833	0.51617949	0.628325778	0.707200559	0.191021069	7.60E-11	3.22E-09
UTR5	HSPB3	5	53751493	1	cg01246617	0.697327045	0.493130772	0.357341864	-0.339985181	1.38E-14	3.57E-11
UTR5	HTN3	4	70896083	1	cg14948804	0.57341634	0.450426156	0.325037164	-0.248379176	6.94E-09	9.69E-08
UTR5	HTR5A	7	154862548	1	cg11298960	0.458647245	0.538161761	0.638002945	0.1793557	6.67E-08	6.73E-07
UTR5	HTR7	10	92617429/92617562	2	cg15364725/cg26135506	0.201898507	0.225477729	0.384857195	0.182958688	0.000187765	0.000752637
UTR5	HTRA3	4	8271507	1	cg09495977	0.246006178	0.249152199	0.40332575	0.157319573	0.002922907	0.008765188

UTR5	HYAL1	3	50341183/50340564/50341074/50341293/50341009/50341694	6	cg06360465/cg10580282/cg11607701/cg12930727/cg14943722/cg20724227	0.677079064	0.550714635	0.473902068	-0.203176996	6.44E-09	9.18E-08
UTR5	IBSP	4	88722338	1	cg16909685	0.68263131	0.55958395	0.432273336	-0.250357974	1.83E-08	2.17E-07
UTR5	IFI27	14	94577218/94577101/94577514	3	cg08036899/cg10778971/cg20161089	0.46710237	0.384939517	0.315388636	-0.151713734	2.70E-09	4.47E-08
UTR5	IFLTD1	12	25705953	1	cg15461781	0.743101665	0.645694244	0.503263105	-0.23983856	2.64E-05	0.000128366
UTR5	IGFL2	19	46651227/46651087/46651553	3	cg10833393/cg20459022/cg21084674	0.684816412	0.582644956	0.532426605	-0.152389807	1.18E-11	1.11E-09
UTR5	IGFL4	19	46544240	1	cg27048142	0.61426804	0.466338333	0.368022105	-0.246245935	1.02E-09	2.06E-08
UTR5	IL12B	5	158754684/158757456	2	cg15216232/cg18307303	0.651192595	0.548948333	0.458741455	-0.19245114	6.27E-08	6.36E-07
UTR5	IL18RAP	2	103039542/103035835/103038171	3	cg13897122/cg21282997/cg27361520	0.649130895	0.567147328	0.493154689	-0.155976206	4.65E-08	4.91E-07
UTR5	IL22	12	68647280	1	cg11520493	0.83779575	0.797853883	0.657353691	-0.180442059	3.79E-06	2.27E-05
UTR5	IL25	14	23842125	1	cg07258507	0.42593412	0.331148294	0.238360264	-0.187573856	2.46E-11	1.71E-09
UTR5	IL4	5	132009630	1	cg12377972	0.79358193	0.722593817	0.610814186	-0.182767744	1.10E-06	7.65E-06
UTR5	INHBA	7	41741428/41742630/41741671	3	cg09138133/cg11079619/cg15291905	0.69404725	0.619499531	0.528663752	-0.165383498	1.82E-08	2.17E-07
UTR5	IRAK3	12	66583029/66583048	2	cg01263292/cg26415547	0.088190073	0.150723497	0.330097085	0.241907013	2.15E-05	0.000106558
UTR5	IRF4	6	391936/392555/393110/392131/391764	5	cg06392169/cg12612118/cg12684209/cg12741420/cg17228900	0.237617969	0.435077595	0.610867689	0.37324972	1.36E-09	2.57E-08
UTR5	IRX4	5	1882775/1882871	2	cg03679565/cg03963198	0.296017495	0.365641589	0.537900323	0.241882828	0.000114971	0.000485145
UTR5	ITGA4	2	182322268/182322058/182321855/182322279/182321786	5	cg06952671/cg10526659/cg20415809/cg21995919/cg25652029	0.264610925	0.464160694	0.63316878	0.368557855	3.16E-10	8.78E-09
UTR5	ITIH4	3	52864659/52864694	2	cg10929387/cg11645453	0.718289485	0.606850944	0.554575986	-0.163713499	1.61E-06	1.07E-05
UTR5	ITIH5	10	7661611/7708869	2	cg00677195/cg04711998	0.50065485	0.566033614	0.659438561	0.158783711	6.94E-09	9.69E-08
UTR5	IVL	1	152881119/152881815/152881930	3	cg05440289/cg15486123/cg21419383	0.762569447	0.6899959	0.60233433	-0.160235116	2.25E-09	3.85E-08
UTR5	IYD	6	150690125/150690143	2	cg02307277/cg20416179	0.531765885	0.431119939	0.344286614	-0.187479271	4.21E-11	2.28E-09
UTR5	IZUMO1	19	49249123/49249932/49250080	3	cg08610426/cg10234985/cg18537730	0.557122048	0.64113363	0.751633662	0.194511614	2.14E-08	2.50E-07

UTR5	JAM2	21	27011788/27011963/27011793/27012066	4	cg02485200/cg13769223/cg14553600/cg22627390	0.114209011	0.203837442	0.343226049	0.229017038	1.60E-05	8.20E-05
UTR5	KAZALD1	10	102821427/102821670/102822249/102821684/102822002/102821848/102822201/102821552/102821565	9	cg07638935/cg08227353/cg10342899/cg12060422/cg13269555/cg15302379/cg17333042/cg23534216/cg26605164	0.422940507	0.313637413	0.238274637	-0.18466587	5.25E-13	1.80E-10
UTR5	KCNA1	12	5019933/5019311/5019482/5019091/5020169/5020202	6	cg06204922/cg09487611/cg10363246/cg12908522/cg16907029/cg26590537	0.213254822	0.335016138	0.536760808	0.323505987	1.83E-08	2.17E-07
UTR5	KCNA3	1	111217575/111217497	2	cg01423964/cg11595545	0.225400212	0.447031806	0.578786818	0.353386607	1.86E-11	1.53E-09
UTR5	KCNA5	12	5153174/5153288	2	cg16897114/cg20792062	0.322068494	0.458806277	0.587434582	0.265366088	7.63E-11	3.22E-09
UTR5	KCNA6	12	4919001/4919138/4919081/4918391/4918848	5	cg01175812/cg11224582/cg19870512/cg26162582/cg26858704	0.232089593	0.336341236	0.440985895	0.208896302	0.000989008	0.00331343
UTR5	KCNB1	20	48099146	1	cg26709285	0.531989195	0.600984622	0.722353068	0.190363873	4.13E-10	1.05E-08
UTR5	KCNC2	12	75601824/75603343/75601928/75603401/75603184/75601803/75603068/75603480	8	cg10148473/cg11308277/cg16370491/cg18573383/cg21500166/cg25820699/cg26074603/cg27154343	0.37640507	0.495046153	0.635358596	0.258953526	1.54E-10	5.03E-09
UTR5	KCNH4	17	40333009	1	cg17812788	0.175100328	0.203774286	0.3549292	0.179828873	0.000911142	0.003084209
UTR5	KCNH7	2	163695190/163695111	2	cg10132588/cg14781189	0.41570884	0.508834197	0.626071409	0.210362569	1.41E-10	4.77E-09
UTR5	KCNIP1	5	169931363/169931092/169931241	3	cg01585372/cg08422599/cg11284411	0.389190825	0.48636765	0.599274682	0.210083857	1.08E-08	1.40E-07
UTR5	KCNJ1	11	128729652/128737201/128736844/128712697/128712280/128712399/128730011/128712645/128732986/128729883/128712462/128729960/128712417/128711233/128712932	15	cg04191300/cg04367216/cg05193832/cg06995869/cg07168810/cg07713705/cg12055220/cg12204555/cg14631438/cg15127791/cg20962532/cg23418180/cg25194720/cg25751961/cg25837340	0.706942754	0.645965869	0.55372432	-0.153218433	1.54E-08	1.88E-07

UTR5	KDR	4	55991683/55991701	2	cg10740902/cg25722465	0.523004225	0.591848633	0.682941223	0.159936998	3.79E-10	9.88E-09
UTR5	KERA	12	91451819	1	cg20332354	0.574654435	0.43442825	0.333692345	-0.24096209	4.87E-11	2.50E-09
UTR5	KHDRBS2	6	62995876/62996022/62995963	3	cg00472801/cg16587616/cg18239753	0.395316767	0.479496617	0.604159612	0.208842845	8.58E-08	8.39E-07
UTR5	KIAA0495	1	3663339/3663832/3663435/3663531/3663164/3663705	6	cg01793449/cg05491852/cg10634619/cg15472784/cg23666378/cg25467973	0.449539479	0.568619208	0.703423341	0.253883862	8.91E-10	1.88E-08
UTR5	KIAA0802	18	8717926/8717905	2	cg07955887/cg19522644	0.68367303	0.471543494	0.395284905	-0.288388125	9.83E-13	2.71E-10
UTR5	KIRREL2	19	36347907/36347919	2	cg00519185/cg09479015	0.162045664	0.267688109	0.350421579	0.188375915	0.000146957	0.000602097
UTR5	KLHL1	13	70682098/70682019/70682324/70682604/70682127/70682525	6	cg01661350/cg12917718/cg20523861/cg23267558/cg24014143/cg24401870	0.368882528	0.435305449	0.519576602	0.150694074	7.49E-07	5.44E-06
UTR5	KLK12	19	51538134	1	cg24368865	0.46914602	0.380552894	0.280538386	-0.188607634	2.98E-10	8.41E-09
UTR5	KLKB1	4	187148688	1	cg12536028	0.557554245	0.435691661	0.345760305	-0.21179394	1.79E-10	5.65E-09
UTR5	KRT38	17	39597402	1	cg25306838	0.4231071	0.347799894	0.264167418	-0.158939682	4.41E-10	1.10E-08
UTR5	KRT7	12	52627047	1	cg09670128	0.37070131	0.531059411	0.734100745	0.363399435	6.18E-11	2.88E-09
UTR5	KRT72	12	52995250/52995295	2	cg18161890/cg20050826	0.537895373	0.634271478	0.723837475	0.185942103	1.89E-09	3.32E-08
UTR5	KRT80	12	52585695	1	cg16432350	0.55077068	0.459402311	0.381649923	-0.169120757	1.30E-09	2.50E-08
UTR5	KRTAP24-1	21	31655261	1	cg07172334	0.56064531	0.332883772	0.239309864	-0.321335446	3.78E-12	5.48E-10
UTR5	KY	3	134369828	1	cg23201032	0.232375405	0.286864915	0.3872008	0.154825395	0.000320049	0.001207214
UTR5	L1TD1	1	62660861/62664360/62660741/62660556/62660624/62662166	6	cg03731268/cg11146821/cg12640000/cg19594218/cg23049458/cg27424906	0.471648051	0.338231044	0.296492537	-0.175155514	1.88E-09	3.31E-08
UTR5	LAMA1	18	7117790	1	cg18764577	0.33890274	0.440777189	0.599022482	0.260119742	1.03E-11	1.03E-09
UTR5	LAMB3	1	209825747/209824825/209825678/209825672/209825731	5	cg03977657/cg20416769/cg22502856/cg26107033/cg26533764	0.565542667	0.455648091	0.373599841	-0.191942826	7.97E-11	3.27E-09
UTR5	LAYN	11	111411289/111411381	2	cg02532096/cg03864000	0.291250943	0.420109953	0.501159723	0.20990878	7.65E-06	4.23E-05
UTR5	LCE1B	1	152784914	1	cg07090653	0.729669125	0.6528083	0.479028127	-0.250640998	1.89E-06	1.23E-05
UTR5	LCE1D	1	152769246/152769896	2	cg11583211/cg11835200	0.513716928	0.378414683	0.274873037	-0.23884389	5.17E-13	1.80E-10
UTR5	LCE1E	1	152759772/152758755	2	cg16987735/cg21065959	0.50352849	0.384179033	0.302360068	-0.201168422	2.16E-10	6.60E-09
UTR5	LCE2D	1	152635878/152636074	2	cg16361876/cg21312148	0.680842535	0.5766596	0.4701227	-0.210719835	1.34E-07	1.23E-06

UTR5	LCE3D	1	152552494	1	cg20676475	0.71794003	0.551679256	0.380826986	-0.337113044	2.19E-11	1.65E-09
UTR5	LCE3E	1	152538888/152538857	2	cg23110514/cg24000437	0.577017838	0.439957958	0.318514782	-0.258503056	1.33E-13	7.47E-11
UTR5	LCE6A	1	152815459/152815843	2	cg00998782/cg13366738	0.582232658	0.480953378	0.40564598	-0.176586678	5.28E-08	5.48E-07
UTR5	LCN2	9	130911792	1	cg22438810	0.47500126	0.371037206	0.284364823	-0.190636437	4.28E-12	5.77E-10
UTR5	LELP1	1	153176083/153177032	2	cg02886578/cg21936464	0.572080785	0.457009633	0.37048255	-0.201598235	4.33E-06	2.54E-05
UTR5	LFNG	7	2557715	1	cg20572537	0.539025405	0.371185094	0.294986845	-0.24403856	2.44E-11	1.71E-09
UTR5	LGALS4	19	39303698	1	cg19419519	0.51058788	0.380183917	0.294491536	-0.216096344	1.30E-10	4.46E-09
UTR5	LHFPL4	3	9594385/9595269/9594382/9595395	4	cg04061117/cg06838365/cg19099050/cg19822251	0.399782828	0.469894613	0.597688253	0.197905426	2.04E-10	6.30E-09
UTR5	LILRA6	19	54746613	1	cg19835973	0.72572726	0.584950672	0.542445532	-0.183281728	6.83E-05	0.000302509
UTR5	LILRB3	19	54726870	1	cg15816012	0.64083175	0.532339556	0.488177741	-0.152654009	1.77E-06	1.16E-05
UTR5	LIN28	1	26737318/26737272	2	cg02388865/cg19706817	0.316462558	0.4353874	0.497957273	0.181494715	7.56E-07	5.49E-06
UTR5	LIN28B	6	105405068	1	cg04560456	0.330428215	0.378783794	0.543564627	0.213136412	2.34E-06	1.49E-05
UTR5	LMO1	11	8285086/8284997/8285031	3	cg05245226/cg07283152/cg21460402	0.147183635	0.161614728	0.299076456	0.151892821	0.001208018	0.003972084
UTR5	LOC100302652	2	54087066	1	cg19961545	0.564480035	0.679589256	0.815680955	0.25120092	2.04E-13	9.39E-11
UTR5	LOC169834	9	115773738/115771599	2	cg00019877/cg14056849	0.69703368	0.614172244	0.5114797	-0.18555398	9.82E-08	9.40E-07
UTR5	LOC200726	2	207507528/207507163/207507421	3	cg12848065/cg22944499/cg25146557	0.439611425	0.500116722	0.624982723	0.185371298	1.21E-07	1.13E-06
UTR5	LOC728392	17	5404508/5404330/5404337	3	cg06462347/cg22298430/cg27230784	0.06618335	0.104406035	0.269477279	0.203293929	0.000446186	0.001620089
UTR5	LONRF2	2	100939045/100938903/100938813/100938799	4	cg07304692/cg14675211/cg14997226/cg23977631	0.084088822	0.258085331	0.391470726	0.307381903	4.06E-08	4.36E-07
UTR5	LOXHD1	18	44098826/44099028/44139159	3	cg07931960/cg12934788/cg20454888	0.602301588	0.502350575	0.388695468	-0.213606119	6.10E-11	2.86E-09
UTR5	LPA	6	161087339/161086715	2	cg10836120/cg17028067	0.66854474	0.563267111	0.491352743	-0.177191997	1.46E-07	1.32E-06
UTR5	LRFN4	11	66625106/66625164	2	cg11862081/cg15320059	0.553181175	0.449167631	0.38776835	-0.165412825	4.02E-13	1.56E-10
UTR5	LTF	3	46506374	1	cg15261665	0.51536741	0.585800356	0.667935541	0.152568131	6.48E-09	9.21E-08
UTR5	LUZP2	11	24518737/24518776	2	cg06931933/cg09717526	0.277360133	0.309575603	0.460539434	0.183179302	2.11E-05	0.000104776

UTR5	LY6H	8	144241434/144242029/144241120/144241104/144241407/144241809	6	cg02539855/cg03699469/cg09528449/cg10821845/cg23484951/cg26663686	0.242604549	0.322622021	0.429467291	0.186862742	5.08E-05	0.000230732
UTR5	LYZL4	3	42451096/42451876/42451986	3	cg00212520/cg21044104/cg25867632	0.5998932	0.495703911	0.433341662	-0.166551538	3.06E-08	3.41E-07
UTR5	LZTS1	8	20112763	1	cg01664925	0.648981185	0.574257428	0.496948045	-0.15203314	5.23E-06	3.01E-05
UTR5	MAL	2	95691500	1	cg22403344	0.132396917	0.293686192	0.438046159	0.305649243	1.19E-08	1.52E-07
UTR5	MAP4K1	19	39108609	1	cg19457477	0.526199	0.639454106	0.724490768	0.198291768	8.73E-08	8.50E-07
UTR5	MAP9	4	156297854/156297942/156298050/156297858/156297616/156298002	6	cg03405515/cg03616357/cg08015755/cg15397448/cg17780956/cg20334243	0.167640528	0.307872058	0.421026315	0.253385787	3.36E-05	0.000159643
UTR5	1-Mar	4	165119501/165118829/165118368/165118930/165118938/165118868/165304394	7	cg09248897/cg16244944/cg19400238/cg23679141/cg24132580/cg25932496/cg26320830	0.713920753	0.617985136	0.563648559	-0.150272193	2.41E-11	1.70E-09
UTR5	MARVELD2	5	68711277/68711640/68711519	3	cg02505827/cg04091702/cg16419724	0.41348682	0.316281989	0.256951732	-0.156535088	3.68E-10	9.68E-09
UTR5	MC3R	20	54823878	1	cg24298684	0.450022515	0.331019572	0.246476718	-0.203545797	1.68E-12	3.47E-10
UTR5	MDFI	6	41606317/41606439	2	cg01350077/cg27200446	0.134761777	0.262817074	0.459091135	0.324329358	1.36E-06	9.17E-06
UTR5	MEI1	22	42095536	1	cg11260046	0.40945288	0.503480361	0.618011595	0.208558715	5.50E-06	3.14E-05
UTR5	MFRP	11	119217331	1	cg09186818	0.48301678	0.414818267	0.315081205	-0.167935575	1.64E-09	2.99E-08
UTR5	MGAM	7	141695711/141695759	2	cg01551699/cg18971054	0.521092418	0.434172056	0.3607364	-0.160356018	8.10E-11	3.29E-09
UTR5	MGAT5B	17	74865068	1	cg00654814	0.078900668	0.159799848	0.252306276	0.173405608	1.20E-05	6.31E-05
UTR5	MGC26647	7	88424985	1	cg19682786	0.787220455	0.720656683	0.610087027	-0.177133428	4.98E-06	2.88E-05
UTR5	MIA2	14	39703302	1	cg24603941	0.64484315	0.5140377	0.41682725	-0.2280159	8.05E-09	1.10E-07
UTR5	MMD2	7	4998714/4998649	2	cg26468007/cg27603283	0.263741423	0.340103578	0.49909633	0.235354907	3.27E-07	2.68E-06
UTR5	MME	3	154797867/154797508/154798079/154797592/154797589/154797563/154797958/154797677/154797779/154797947/154797917/154797892	12	cg02108850/cg03648334/cg05962239/cg14750844/cg16580737/cg17344755/cg20216802/cg21496658/cg22471230/cg22955973/cg23209255/cg26872138	0.104540567	0.14668136	0.259106174	0.154565607	0.004505674	0.012915294
UTR5	MMP12	11	102745682	1	cg20487452	0.61555095	0.455733928	0.3300052	-0.28554575	3.77E-14	3.95E-11

UTR5	MMP7	11	102401438	1	cg25511807	0.37490179	0.281074117	0.208489595	-0.166412195	9.84E-11	3.70E-09
UTR5	MN1	22	28196834/28197197/28197091	3	cg00278028/cg26099061/cg27269921	0.19237339	0.270592073	0.392676425	0.200303036	0.00011587	0.000488278
UTR5	MRGPRX3	11	18157898/18157405	2	cg07656579/cg10378819	0.802569685	0.719069706	0.548679564	-0.253890121	3.78E-07	3.03E-06
UTR5	MRPS21	1	150266604/150266477/150266643/150266645/150266311/150266488/150266313	7	cg02460371/cg08293086/cg09808343/cg15934674/cg18031008/cg23819679/cg26535484	0.197437178	0.26105832	0.399404685	0.201967507	7.03E-05	0.000310574
UTR5	MS4A2	11	59856225	1	cg27533472	0.77252679	0.670307089	0.591417223	-0.181109567	2.14E-09	3.70E-08
UTR5	MSC	8	72756656	1	cg23710218	0.321671095	0.438835856	0.545340823	0.223669728	1.26E-07	1.16E-06
UTR5	MST1	3	49726151	1	cg17781925	0.560604335	0.465950656	0.407951073	-0.152653262	3.60E-10	9.55E-09
UTR5	MUC15	11	26593556/26593658	2	cg03087937/cg06056884	0.568207505	0.502954472	0.397117164	-0.171090341	2.25E-07	1.93E-06
UTR5	MYF6	12	81101474	1	cg20171297	0.473562055	0.4037161	0.306037386	-0.167524669	9.87E-10	2.01E-08
UTR5	MYH1	17	10421635/10421365	2	cg00421089/cg00933692	0.815634563	0.738517422	0.642775495	-0.172859067	3.67E-08	4.01E-07
UTR5	MYH13	17	10276260	1	cg21376148	0.52202167	0.422401772	0.343106009	-0.178915661	6.73E-09	9.47E-08
UTR5	MYH6	14	23876824/23877130	2	cg10250660/cg14541311	0.5903452	0.497532986	0.422626382	-0.167718818	1.13E-10	4.05E-09
UTR5	MYL5	4	671791	1	cg17073306	0.415675175	0.343768378	0.262411736	-0.153263439	7.29E-10	1.60E-08
UTR5	MYO1A	12	57443622/57443357	2	cg09541248/cg22614532	0.513802985	0.407921597	0.323402307	-0.190400678	3.66E-11	2.07E-09
UTR5	MYOD1	11	17741243	1	cg16519321	0.279759915	0.339630044	0.473221386	0.193461471	6.49E-06	3.65E-05
UTR5	NAALAD2	11	89867819/89867911	2	cg05500015/cg14304817	0.282223865	0.356518817	0.438218755	0.15599489	0.001114024	0.003684506
UTR5	NCAM2	21	22370733/22370864/22370758	3	cg15681537/cg17475987/cg25818292	0.158762917	0.227909182	0.326792251	0.168029334	1.17E-05	6.18E-05
UTR5	NCAN	19	19323996/19322818/19327542/19323330/19322902/19325920	6	cg02918253/cg08249988/cg13118849/cg15976079/cg17767542/cg26856607	0.38890778	0.459038992	0.555911934	0.167004154	2.22E-08	2.58E-07
UTR5	NEFL	8	24814126/24814035	2	cg22978087/cg24032666	0.368008958	0.387229822	0.542913533	0.174904575	2.80E-05	0.000135081
UTR5	NEFM	8	24773148/24772878/24773060/24771273/24772513	5	cg01583969/cg02002551/cg12026749/cg18267374/cg26980244	0.38603517	0.488248139	0.589292386	0.203257216	2.45E-07	2.07E-06
UTR5	NELL1	11	20691161/20691180/20691126	3	cg14689623/cg20113732/cg23861668	0.302093187	0.3874827	0.537339132	0.235245945	3.54E-07	2.87E-06
UTR5	NETO1	18	70534767/70534534	2	cg18448581/cg26186727	0.328851298	0.397823596	0.482569505	0.153718207	3.56E-10	9.49E-09

UTR5	NEUROD6	7	31379820/31380390	2	cg25597535/cg26312920	0.380424165	0.292083483	0.224579068	-0.155845097	5.07E-10	1.20E-08
UTR5	NEUROG3	10	71333037/71332804	2	cg03425110/cg17583413	0.225495702	0.331307686	0.573642898	0.348147196	6.76E-10	1.50E-08
UTR5	NFAM1	22	42828386	1	cg03365751	0.54159879	0.634140517	0.700656695	0.159057905	2.06E-09	3.59E-08
UTR5	NGB	14	77737621/77737495	2	cg02181639/cg02881570	0.375804095	0.441153767	0.623488245	0.24768415	3.50E-07	2.84E-06
UTR5	NKX2-2	20	21494547	1	cg17029168	0.2650935	0.329316172	0.497733132	0.232639632	1.83E-05	9.27E-05
UTR5	NKX2-3	10	101292766	1	cg08287265	0.209799967	0.241813265	0.370159051	0.160359085	0.001187002	0.003909199
UTR5	NMBR	6	142409831	1	cg08109815	0.430450735	0.510800806	0.609909305	0.17945857	1.22E-11	1.12E-09
UTR5	NOS2	17	26127480/26127522/26127542/26127537/26127438	5	cg02196147/cg06301529/cg07150830/cg07704981/cg26853415	0.531012951	0.422848119	0.331358986	-0.199653965	1.43E-10	4.82E-09
UTR5	NOVA1	14	27066771	1	cg15602241	0.21546241	0.203697178	0.385430986	0.169968576	0.000721205	0.00249158
UTR5	NPAS4	11	66188522	1	cg14699728	0.61101075	0.731231661	0.824678177	0.213667427	1.72E-11	1.44E-09
UTR5	NPHS1	19	36342859	1	cg04941018	0.64290901	0.533100622	0.438364864	-0.204544146	1.45E-09	2.71E-08
UTR5	NPR3	5	32711881	1	cg01252672	0.087079092	0.174021886	0.459977908	0.372898816	1.57E-06	1.04E-05
UTR5	NPY	7	24324570/24324435/24323939/24323840	4	cg11475550/cg12614105/cg21097881/cg25884711	0.288807862	0.444132025	0.564067368	0.275259506	3.62E-09	5.67E-08
UTR5	NR1I3	1	161206502/161207966	2	cg17028814/cg25214346	0.60810911	0.493879067	0.394479575	-0.213629535	1.11E-10	4.03E-09
UTR5	NR2E1	6	108487388/108487506/108487903/108487820	4	cg02062480/cg03383382/cg06559274/cg19697981	0.240879177	0.25947047	0.396833913	0.155954736	2.00E-05	0.000100253
UTR5	NRG2	5	139422822/139422829	2	cg02128325/cg02725413	0.080611928	0.140070739	0.251405918	0.17079399	0.000972439	0.003261443
UTR5	NTF4	19	49566731	1	cg25937395	0.488421705	0.397439061	0.335996991	-0.152424714	2.65E-11	1.77E-09
UTR5	NTNG1	1	107684059/107682963/107683725/107684339/107683187/107683775/107684425/107684255/107682798/107688142/107683715/107682674/107684751/107683532/107683677/107682818	16	cg01559617/cg02361557/cg02636497/cg06368118/cg07005523/cg07155336/cg07470367/cg09508770/cg09822538/cg10887385/cg11396157/cg15128200/cg15697822/cg16136098/cg18200760/cg20170831	0.16137007	0.212047113	0.318257479	0.156887409	0.000432347	0.001575751
UTR5	NTRK3	15	88799523/88799526	2	cg11525479/cg27034819	0.074820707	0.151314393	0.25776619	0.182945484	2.58E-07	2.17E-06

UTR5	NTSR1	20	61340542/61340340	2	cg00254133/cg08678514	0.26435482	0.305493228	0.441554541	0.177199721	1.79E-05	9.08E-05
UTR5	NWD1	19	16844603/16830859	2	cg10589577/cg21035374	0.46745219	0.38020425	0.290009155	-0.177443035	6.02E-10	1.38E-08
UTR5	NXPH1	7	8475198/8474544/8473990/8474830	4	cg02124383/cg03099208/cg06444755/cg19469357	0.352218618	0.427701069	0.534196289	0.181977671	1.92E-07	1.68E-06
UTR5	ODF3	11	196824/197224	2	cg04874875/cg16225091	0.759704613	0.695349486	0.586404105	-0.173300508	9.80E-07	6.91E-06
UTR5	OLIG2	21	34398532/34398263/34398849/34398265	4	cg05724110/cg09793121/cg14843922/cg22869726	0.317999256	0.411034344	0.58256032	0.264561064	2.43E-09	4.12E-08
UTR5	OLIG3	6	137815333	1	cg03889226	0.18031487	0.24152574	0.411535868	0.231220998	5.07E-06	2.93E-05
UTR5	OPCML	11	133402230/132812991/133402246	3	cg03923934/cg09314708/cg19349861	0.329729523	0.404297156	0.49141755	0.161688028	6.17E-06	3.48E-05
UTR5	OPRK1	8	54163693/54164161/54163622/54164051/54164081	5	cg06808751/cg07303244/cg10095242/cg11701471/cg25990647	0.381651307	0.471933456	0.620641206	0.238989899	1.88E-09	3.32E-08
UTR5	OR2B3	6	29055042	1	cg27458549	0.723920105	0.577724044	0.444687195	-0.27923291	4.11E-09	6.34E-08
UTR5	OR5V1	6	29323978	1	cg19323832	0.791713435	0.679382694	0.539983595	-0.25172984	6.64E-06	3.72E-05
UTR5	OTP	5	76934461/76934501	2	cg21097283/cg24231716	0.199478577	0.271493168	0.380937562	0.181458985	6.10E-07	4.53E-06
UTR5	PABPC3	13	25670319/25670327	2	cg18474108/cg21341928	0.531042993	0.58932225	0.686503539	0.155460546	5.70E-09	8.31E-08
UTR5	PATE3	11	125658038	1	cg09752271	0.421375365	0.327739161	0.242760424	-0.178614941	6.08E-07	4.52E-06
UTR5	PAX4	7	127255636/127255591	2	cg20082779/cg20672477	0.489752483	0.415560414	0.330597914	-0.159154569	2.61E-09	4.35E-08
UTR5	PAX7	1	18957632/18958084	2	cg11428724/cg13969001	0.182770352	0.194844565	0.339914824	0.157144472	0.003890899	0.011339086
UTR5	PCDH10	4	134070598/134070819	2	cg14410319/cg14795750	0.2297729	0.321316182	0.433004927	0.203232027	4.65E-07	3.58E-06
UTR5	PCDH17	13	58206058/58206036	2	cg00208153/cg14893163	0.31001712	0.411174189	0.570123352	0.260106232	6.67E-10	1.49E-08
UTR5	PCDH8	13	53422691	1	cg27360326	0.40974839	0.4804092	0.591171164	0.181422774	7.41E-05	0.000325595
UTR5	PCDHA9	5	140227765	1	cg11445191	0.54467526	0.461306417	0.347419155	-0.197256105	3.44E-05	0.000163204
UTR5	PCDHB5	5	140514944/140514811/140514824	3	cg18021368/cg19568544/cg24583624	0.462452523	0.553871594	0.665145345	0.202692822	2.85E-09	4.65E-08
UTR5	PCDHGA11	5	140800586/140800629/140800761	3	cg05870739/cg10679597/cg24954895	0.41512593	0.572358914	0.715389425	0.300263495	1.72E-11	1.44E-09
UTR5	PCDHGA12	5	140810260/140810161	2	cg07802710/cg11647681	0.345940625	0.459376522	0.5954184	0.249477775	2.10E-08	2.46E-07
UTR5	PCDHGA2	5	140718405	1	cg03617826	0.35435397	0.444526017	0.568896195	0.214542225	1.56E-07	1.40E-06

UTR5	PCDHGA8	5	140772256/140772286/140772182	3	cg14458068/cg21117330/cg26811372	0.564974788	0.664611019	0.729108303	0.164133515	1.12E-07	1.06E-06
UTR5	PCDHGB7	5	140797286	1	cg23563234	0.48308974	0.586047222	0.681570527	0.198480787	6.83E-07	5.01E-06
UTR5	PCDHGC3	5	140855582	1	cg10858746	0.104250289	0.151458377	0.272282888	0.168032599	0.005111005	0.01447321
UTR5	PCK1	20	56136167	1	cg20605413	0.399125295	0.314853322	0.242899114	-0.156226181	2.25E-10	6.80E-09
UTR5	PCOLCE	7	100200009/100199982/100199952/100200003	4	cg02797569/cg06402330/cg26777475/cg27069566	0.633549886	0.575186529	0.459504847	-0.17404504	1.90E-08	2.24E-07
UTR5	PCP4L1	1	161228674	1	cg11912330	0.16781842	0.160256398	0.392305085	0.224486665	0.000207649	0.00082435
UTR5	PCSK2	20	17207697	1	cg27343616	0.308719235	0.355116233	0.475753955	0.16703472	5.03E-08	5.25E-07
UTR5	PDCD1LG2	9	5515324	1	cg14351952	0.812666195	0.682149372	0.589016491	-0.223649704	7.47E-09	1.02E-07
UTR5	PDE1B	12	54955143/54943545/54943484	3	cg01961252/cg15927682/cg24020814	0.248393013	0.331568553	0.422807558	0.174414545	8.91E-06	4.88E-05
UTR5	PDE4A	19	10531434	1	cg23103993	0.095087647	0.097623676	0.362455849	0.267368202	0.00012013	0.000503831
UTR5	PDGFD	11	104034754/104034982	2	cg02120582/cg18403606	0.049574196	0.177355176	0.363817949	0.314243754	8.35E-07	5.98E-06
UTR5	PENK	8	57358651/57358713/57359200/57359258/57358625/57358590/57358928	7	cg03483150/cg04612444/cg06066137/cg10397440/cg18742346/cg21694941/cg24645221	0.405802708	0.492353038	0.610716113	0.204913405	1.93E-12	3.63E-10
UTR5	PEX5L	3	179754483	1	cg03175305	0.176515565	0.282426469	0.423390118	0.246874553	1.27E-07	1.17E-06
UTR5	PGLYRP1	19	46526321	1	cg14001023	0.593738825	0.692920044	0.795988614	0.202249789	2.86E-10	8.16E-09
UTR5	PHF21B	22	45405627/45405624/45405768/45405621	4	cg15679813/cg20495645/cg20525183/cg25976257	0.094925203	0.148587111	0.297336512	0.202411309	9.48E-06	5.14E-05
UTR5	PHGR1	15	40644728/40644696	2	cg00114694/cg16058105	0.508438575	0.392952178	0.299063073	-0.209375502	2.30E-11	1.69E-09
UTR5	PHOX2A	11	71955113/71955164	2	cg13103303/cg19651694	0.364023335	0.428198539	0.596162923	0.232139588	3.48E-10	9.33E-09
UTR5	PI15	8	75737456	1	cg00128482	0.451085095	0.333594339	0.249696736	-0.201388359	8.82E-09	1.19E-07
UTR5	PIK3R6	17	8762014/8770954/8770908	3	cg00409104/cg09209669/cg24092340	0.414249083	0.299857572	0.223271247	-0.190977836	9.26E-12	9.57E-10
UTR5	PLA1A	3	119316759	1	cg15362455	0.425528655	0.340435272	0.265920373	-0.159608282	7.83E-11	3.23E-09
UTR5	PLA2G4F	15	42448786/42448760	2	cg10917153/cg16920001	0.482703	0.393574192	0.321426993	-0.161276007	7.69E-11	3.22E-09
UTR5	PLCZ1	12	18890872/18890579	2	cg06233731/cg25573386	0.56617049	0.462223856	0.365700895	-0.200469595	1.10E-09	2.19E-08
UTR5	PLEKHA4	19	49371285/49371079	2	cg06339706/cg06705122	0.613221388	0.533954208	0.430637975	-0.182583413	8.84E-09	1.19E-07
UTR5	PLSCR5	3	146323078	1	cg22594071	0.469473455	0.390588256	0.302888591	-0.166584864	9.13E-10	1.90E-08

UTR5	PNLIPRP1	10	118350608	1	cg23148094	0.59423929	0.479172828	0.389032045	-0.205207245	8.24E-11	3.31E-09
UTR5	POM121L2	6	27280010	1	cg07085962	0.085615451	0.126727028	0.278740917	0.193125467	0.000584613	0.002068657
UTR5	POU4F2	4	147560282/147560286/147560126	3	cg02610222/cg14631165/cg24199834	0.322328865	0.391729356	0.513645348	0.191316483	1.32E-10	4.53E-09
UTR5	POU4F3	5	145718669/145718597	2	cg17871749/cg23303408	0.331677935	0.393681303	0.499733048	0.168055113	1.26E-06	8.62E-06
UTR5	PPFIA4	1	203020566/203020617/203020660	3	cg00594917/cg10952234/cg24000087	0.729849245	0.657511617	0.57815983	-0.151689415	1.56E-06	1.04E-05
UTR5	PPP1R16B	20	37462474/37464179/37435477/37435204/37435716/37438823/37434409/37434552/37435119/37434950	10	cg02811473/cg03288217/cg03563630/cg06733470/cg07906520/cg18674961/cg22128431/cg24834740/cg25433624/cg25932164	0.274278028	0.353648219	0.458385561	0.184107533	2.14E-06	1.37E-05
UTR5	PPP1R1B	17	37783488/37785426/37784895	3	cg10498434/cg18575532/cg21320242	0.626764635	0.5270592	0.408900736	-0.217863899	9.83E-11	3.70E-09
UTR5	PPP2R2C	4	6473964	1	cg18013519	0.07588347	0.151003771	0.353179632	0.277296162	4.14E-06	2.45E-05
UTR5	PRDM11	11	45115584/45115819	2	cg03470671/cg20227165	0.7306028	0.644063231	0.521855536	-0.208747264	2.90E-08	3.26E-07
UTR5	PRDM14	8	70982285/70983348/70983079/70983532/70982255/70983530/70982935/70982867	8	cg02919936/cg07719492/cg10457247/cg14928902/cg17450425/cg19363499/cg21135533/cg23758305	0.226713718	0.269793244	0.399402039	0.172688321	0.000121324	0.000507981
UTR5	PRG2	11	57157508/57157607/57157632	3	cg04549076/cg08773180/cg12819873	0.648617448	0.560375756	0.48030543	-0.168312018	5.23E-11	2.59E-09
UTR5	PRG4	1	186265765/186265423	2	cg12626411/cg24669741	0.49889375	0.420284861	0.317732418	-0.181161332	2.71E-10	7.85E-09
UTR5	PRIMA1	14	94254351/94254726/94254586/94254131	4	cg06133145/cg10002178/cg20895877/cg23889730	0.349452212	0.421909473	0.624263842	0.27481163	1.32E-09	2.51E-08
UTR5	PRMT8	12	3600764/3600666	2	cg11596580/cg23739862	0.27294165	0.299425	0.423234441	0.150292791	4.65E-05	0.000213552
UTR5	PRND	20	4702581/4704964	2	cg09906458/cg15795984	0.57554116	0.446774367	0.330079114	-0.245462046	1.90E-13	9.07E-11
UTR5	PROCA1	17	27038861/27038686	2	cg02685896/cg24841318	0.159798654	0.293014926	0.409614889	0.249816236	7.27E-07	5.30E-06
UTR5	PRSS27	16	2770211/2770429/2770336/2770298	4	cg01195196/cg05144884/cg12391048/cg16444062	0.74622224	0.619735257	0.552289117	-0.193933123	2.05E-07	1.78E-06

UTR5	PRSS35	6	84227227/84232241/84222360/84232599/84222308	5	cg06032735/cg09115026/cg15269863/cg16162611/cg26156382	0.510103054	0.422760453	0.356038143	-0.154064911	3.47E-11	2.03E-09
UTR5	PTGFR	1	78957253/78957515/78956905/78956845/78957682/78957203/78956847	7	cg00701741/cg02859837/cg03495868/cg03949391/cg19403909/cg24924936/cg27046936	0.406667808	0.473427984	0.594946235	0.188278427	2.11E-10	6.49E-09
UTR5	PVRL4	1	161059267/161059327	2	cg10841756/cg22585988	0.561919435	0.461592733	0.371334232	-0.190585203	8.87E-11	3.46E-09
UTR5	QRFPR	4	122302007/122301996/122301816	3	cg00015770/cg14012124/cg16326674	0.311049757	0.38931335	0.538028636	0.226978879	3.89E-07	3.10E-06
UTR5	RAB11FIP3	16	475948/524857	2	cg05098233/cg07740599	0.558404785	0.414761883	0.311925982	-0.246478803	2.49E-11	1.71E-09
UTR5	RAB17	2	238499464	1	cg01157280	0.594524595	0.492280311	0.398829073	-0.195695522	3.57E-10	9.49E-09
UTR5	RAB25	1	156031182	1	cg19580810	0.566289525	0.4548067	0.357817941	-0.208471584	2.48E-10	7.30E-09
UTR5	RAB34	17	27044745/27044322/27044685/27044629	4	cg02530437/cg12873610/cg18686527/cg21816330	0.40829401	0.526384144	0.650034676	0.241740666	5.93E-11	2.79E-09
UTR5	RAB37	17	72667394/72732964/72667378/72667709	4	cg03932361/cg10817887/cg12448933/cg16347279	0.332886797	0.389007527	0.490841823	0.157955025	5.76E-10	1.33E-08
UTR5	RAB6C	2	130737494/130737664	2	cg06288154/cg09676860	0.28602157	0.337789244	0.468909518	0.182887948	4.89E-10	1.18E-08
UTR5	RALYL	8	85096037/85267094/85096835/85097056/85095498/85097429/85178030/85097246/85129152/85097157/85165845/85097031/85096015/85096868/85097049/85095654/85097195/85095697	18	cg00034076/cg00431317/cg01538821/cg04842146/cg05716166/cg14153637/cg16769791/cg17215863/cg17822502/cg18016194/cg21163701/cg21671806/cg22403811/cg23458558/cg23649708/cg23777958/cg25757598/cg26669044	0.426563578	0.440956065	0.57732122	0.150757642	6.18E-07	4.58E-06
UTR5	RAPSN	11	47470633/47470674	2	cg01947936/cg15679098	0.77149761	0.72687405	0.617255845	-0.154241765	1.52E-05	7.83E-05
UTR5	RAX	18	56940463	1	cg05945059	0.46560232	0.527555411	0.639636873	0.174034553	1.37E-09	2.58E-08
UTR5	RBP1	3	139258658	1	cg06208339	0.123450014	0.144507533	0.277254184	0.15380417	0.002334998	0.007150824
UTR5	RCSD1	1	167599527/167599631	2	cg00450304/cg02079933	0.026154343	0.066027011	0.212225592	0.186071249	0.00069075	0.002397047

UTR5	RDH5	12	56114155/56114269	2	cg02192520/cg17243643	0.52343967	0.430334986	0.336311564	-0.187128106	9.38E-10	1.95E-08
UTR5	REP15	12	27849452	1	cg07809176	0.483809695	0.390924644	0.323047568	-0.160762127	3.58E-08	3.92E-07
UTR5	RERG	12	15374175/15371727/15373987/15374303	4	cg08830758/cg12642725/cg19205533/cg24979348	0.301404652	0.3551473	0.462855066	0.161450414	0.000119835	0.000502765
UTR5	RIC3	11	8190565/8190572	2	cg08383315/cg25778535	0.300068005	0.452253319	0.576551684	0.276483679	7.12E-11	3.17E-09
UTR5	RICS	11	128893940/128893851	2	cg03231860/cg20892287	0.463703378	0.359730039	0.284669923	-0.179033455	3.32E-11	2.01E-09
UTR5	RLBP1	15	89764836/89764895	2	cg10173075/cg15808008	0.640042538	0.455113978	0.3678254	-0.272217138	2.52E-13	1.08E-10
UTR5	RNASE1	14	21270575/21270924/21270850/21270407/21271018	5	cg05153345/cg05958352/cg08122234/cg13787135/cg18836485	0.552248462	0.451036064	0.35464128	-0.197607182	2.73E-10	7.88E-09
UTR5	RNF150	4	142054417/142054329/142054254/142054446/142053970	5	cg03563667/cg06340552/cg18120376/cg20599022/cg24053721	0.144494352	0.226072682	0.346786786	0.202292435	0.000279212	0.001070758
UTR5	RNF175	4	154681066/154681197/154681323/154681128	4	cg01440841/cg02647878/cg08660876/cg18355902	0.295499914	0.337412067	0.46049854	0.164998626	0.000599655	0.002112961
UTR5	RNF186	1	20141662	1	cg04994456	0.51651407	0.408298611	0.333090264	-0.183423806	4.56E-10	1.12E-08
UTR5	RNF220	1	44872463/44871429/44870976/44873692/44871606/44873064/44873592/44874524/44871634/44873229/44872075/44873057/44874649/44877749/44872502/44871099/44874576/44871297/44871156/44872657	20	cg00023154/cg00825951/cg01267315/cg01422881/cg03946896/cg04023150/cg04541474/cg05597624/cg06550214/cg10224098/cg13675568/cg14403130/cg15590950/cg15830864/cg16822216/cg17243637/cg19719902/cg24515341/cg26035323/cg26341102	0.204029068	0.268822602	0.362602666	0.158573598	3.57E-09	5.61E-08
UTR5	RNF43	17	56493260/56494554	2	cg14398214/cg24835159	0.481662105	0.379250986	0.29474697	-0.186915135	8.94E-11	3.48E-09
UTR5	RSPO2	8	109095568/109095572/109094880/109095264/109095782/109095388	6	cg00910695/cg04050867/cg07390122/cg14070647/cg14733048/cg20061155	0.274606971	0.358213946	0.512446105	0.237839134	2.13E-08	2.49E-07

UTR5	RSPO3	6	127440104	1	cg09979256	0.094536832	0.195482422	0.362228321	0.26769149	2.01E-05	0.0001005
UTR5	RSPO4	20	982831	1	cg01188592	0.303446555	0.340147172	0.454667214	0.151220659	0.000408172	0.001495715
UTR5	RXFP3	5	33936752/33936520/33936597	3	cg02341815/cg02640041/cg03909500	0.351970218	0.438276889	0.550538432	0.198568213	1.41E-07	1.29E-06
UTR5	RXRG	1	165414189/165414332/165414379	3	cg00349776/cg08905496/cg10364513	0.201971838	0.243573256	0.353013446	0.151041608	0.001049961	0.003490304
UTR5	RYR1	19	38924347/38924349/38924436	3	cg04571321/cg05471297/cg06740600	0.19628607	0.287594851	0.464376086	0.268090016	1.11E-05	5.91E-05
UTR5	S100A14	1	153588782	1	cg25433648	0.517648555	0.412206472	0.3245839	-0.193064655	9.46E-11	3.66E-09
UTR5	S100A16	1	153581750/153581494/153581535/153581793/153581655/153584920	6	cg04990202/cg07910075/cg11820824/cg18859033/cg19255608/cg23499956	0.430958912	0.349746561	0.274321637	-0.156637274	1.74E-09	3.13E-08
UTR5	S100A7	1	153432128/153432054	2	cg02892624/cg17421062	0.527180345	0.421562317	0.323614475	-0.20356587	3.42E-11	2.03E-09
UTR5	S100A7A	1	153389957/153389629	2	cg11790580/cg24255159	0.558100615	0.449242547	0.323893911	-0.234206704	2.71E-12	4.68E-10
UTR5	S100P	4	6695614	1	cg26233331	0.376429735	0.286374472	0.222196445	-0.15423329	7.80E-11	3.23E-09
UTR5	S1PR1	1	101702519/101702744/101704473/101704504/101703628/101703749/101702602/101704441/101702782/101702689	10	cg04156369/cg05053688/cg07400091/cg10020333/cg10210739/cg17859448/cg18323466/cg18757941/cg20166532/cg21385047	0.119261569	0.172882457	0.29512414	0.175862572	1.46E-05	7.56E-05
UTR5	SAA4	11	18258153/18258055/18258323	3	cg04334121/cg22288982/cg22587758	0.612559677	0.52097017	0.442492719	-0.170066957	2.74E-14	3.93E-11
UTR5	SALL1	16	51184392/51183988/51183533/51180521/51184355/51184379/51184205/51183473/51183363/51184152	10	cg00582524/cg02288754/cg02417084/cg03961481/cg04550052/cg04698114/cg04844564/cg08199953/cg08878368/cg10297473	0.339892097	0.439168399	0.539588305	0.199696208	1.39E-07	1.27E-06
UTR5	SAMD3	6	130537970/130543838	2	cg09345868/cg11249120	0.85004046	0.761382511	0.63501365	-0.21502681	2.17E-06	1.39E-05
UTR5	SAMD4A	14	55222988/55226755	2	cg13994599/cg21554616	0.603148065	0.55176655	0.4454153	-0.157732765	5.82E-07	4.36E-06
UTR5	SCEL	13	78125149	1	cg13446874	0.7497091	0.664911083	0.591769614	-0.157939486	6.88E-07	5.04E-06
UTR5	SCGB1D1	11	61957732	1	cg07532354	0.46097693	0.372608122	0.283897536	-0.177079394	6.07E-10	1.39E-08
UTR5	SCGB2A1	11	61976195/61976174	2	cg06334737/cg16986846	0.56752183	0.431454211	0.326036145	-0.241485685	5.46E-11	2.67E-09

UTR5	SCGB2A2	11	62037659	1	cg22862656	0.81272038	0.609554289	0.447701014	-0.365019366	1.48E-11	1.29E-09
UTR5	SCN2B	11	118047203	1	cg10513118	0.491962675	0.402305178	0.320984305	-0.17097837	4.66E-08	4.92E-07
UTR5	SCTR	2	120281999/120281813	2	cg07176264/cg20505223	0.287517405	0.314268917	0.480911932	0.193394527	4.00E-05	0.000186988
UTR5	SDC2	8	97506180/97506251	2	cg14538332/cg16935295	0.12978423	0.237232389	0.44108355	0.31129932	3.81E-06	2.28E-05
UTR5	SEC31B	10	102279330/102278918/102279373/102279455	4	cg01072942/cg02593403/cg20831708/cg26381313	0.41692605	0.507569263	0.603449915	0.186523865	1.09E-09	2.17E-08
UTR5	SELENBP1	1	151345123	1	cg24480379	0.490887215	0.421913322	0.335055195	-0.15583202	1.12E-08	1.45E-07
UTR5	SERINC4	15	44092166	1	cg02556940	0.86925858	0.809470183	0.6736888	-0.19556978	1.03E-05	5.54E-05
UTR5	SERPINB11	18	61375622	1	cg17192315	0.679125675	0.548245278	0.4215508	-0.257574875	2.71E-08	3.07E-07
UTR5	SERPINB2	18	61557735/61557687	2	cg20875821/cg23582644	0.56843992	0.429821339	0.336689211	-0.231750709	2.78E-12	4.70E-10
UTR5	SERPINB5	18	61144250/61144339/61144177/61144150	4	cg08411049/cg15422147/cg20837735/cg21807034	0.514257392	0.4132338	0.328815711	-0.185441681	1.06E-09	2.12E-08
UTR5	SFMBT2	10	7452391/7446012/7451314/7451838/7452950/7450456/7451376/7453329/7452815/7450355/7449665/7448857/7451822/7452242/7452598	15	cg01056653/cg02980650/cg09060610/cg12328063/cg12767281/cg15248577/cg20299663/cg20506184/cg20506550/cg22862480/cg22881174/cg24020806/cg25291138/cg26076412/cg26878816	0.267518479	0.38926313	0.529735242	0.262216762	3.69E-07	2.96E-06
UTR5	SFN	1	27189679/27189648	2	cg13466284/cg17330303	0.55060472	0.469117461	0.397089241	-0.153515479	1.54E-09	2.85E-08
UTR5	SFTA3	14	36982649/36982447	2	cg01611862/cg04926361	0.306812683	0.387726467	0.485149523	0.17833684	1.33E-07	1.22E-06
UTR5	SGCE	7	94285501/94285520	2	cg04366249/cg14388858	0.168816405	0.201851454	0.330592637	0.161776232	0.001810142	0.005708343
UTR5	SGCZ	8	15095163	1	cg16266667	0.35641897	0.505215806	0.654992595	0.298573625	4.45E-09	6.80E-08
UTR5	SH2D4B	10	82297793/82300600/82297920/82300588	4	cg08772206/cg10374402/cg10859151/cg22633936	0.667622097	0.592609963	0.515918545	-0.151703551	1.91E-11	1.55E-09
UTR5	SH3GL3	15	84116151/84116115/84116107	3	cg09201151/cg21145136/cg22946150	0.231795257	0.242610657	0.400988149	0.169192892	0.002187862	0.006745183
UTR5	SHC3	9	91793648	1	cg13351583	0.24135894	0.329513467	0.48266265	0.24130371	3.52E-05	0.000166488
UTR5	SHISA9	16	12995568/12995603	2	cg02482218/cg04106647	0.223821843	0.279243776	0.446874112	0.223052269	1.00E-05	5.38E-05
UTR5	SIGLEC12	19	52004992	1	cg27086014	0.48186367	0.415249422	0.329431645	-0.152432025	3.37E-06	2.05E-05

UTR5	SIM1	6	100911526/100911550	2	cg12865837/cg24592906	0.328473495	0.406196656	0.5159396	0.187466105	2.70E-07	2.26E-06
UTR5	SIRPG	20	1638393	1	cg15722679	0.39737777	0.292677606	0.216841866	-0.180535904	4.52E-07	3.50E-06
UTR5	SIX6	14	60975964/60976115	2	cg06785999/cg14186066	0.393968125	0.517370867	0.66005525	0.266087125	1.07E-10	3.93E-09
UTR5	SLC11A1	2	219246985/219247055	2	cg05007997/cg18854666	0.626903165	0.502097061	0.37951783	-0.247385335	9.88E-11	3.70E-09
UTR5	SLC12A5	20	44657838/44657948/44650380	3	cg10439765/cg22752533/cg23720732	0.339649725	0.428707452	0.522097081	0.182447356	6.88E-08	6.93E-07
UTR5	SLC13A5	17	6616653	1	cg16652063	0.406413895	0.545217111	0.646828482	0.240414587	1.23E-08	1.56E-07
UTR5	SLC16A4	1	110933248/110933345	2	cg09494546/cg25320780	0.696382015	0.540663081	0.445331232	-0.251050783	1.45E-10	4.84E-09
UTR5	SLC18A2	10	119000991/119000927/119001066	3	cg00512279/cg08521987/cg19721867	0.234134234	0.306309958	0.424603072	0.190468838	7.65E-05	0.000334991
UTR5	SLC18A3	10	50818652/50818707	2	cg16494192/cg24109980	0.36460248	0.436509039	0.568479809	0.203877329	8.45E-07	6.04E-06
UTR5	SLC22A10	11	63057478/63057592	2	cg01306183/cg16351441	0.60468788	0.479163578	0.355024259	-0.249663621	3.71E-10	9.72E-09
UTR5	SLC22A16	6	110797797	1	cg05415020	0.42231626	0.479833089	0.615592823	0.193276563	9.92E-09	1.30E-07
UTR5	SLC22A24	11	62911616	1	cg16950247	0.52666063	0.380126061	0.307141736	-0.219518894	1.83E-13	9.06E-11
UTR5	SLC22A8	11	62783123/62783272/62782446	3	cg06917325/cg22306408/cg24011351	0.640748493	0.567121428	0.446527155	-0.194221338	5.93E-08	6.08E-07
UTR5	SLC23A3	2	220034739	1	cg15797218	0.56123755	0.462417461	0.394757336	-0.166480214	3.45E-11	2.03E-09
UTR5	SLC24A4	14	92788960/92790097/92790580/92789193/92790364/92790798/92790711/92790285/92789092/92789500/92790072	11	cg01064918/cg01802258/cg04556542/cg04880732/cg07285995/cg10113212/cg11294692/cg18229521/cg18587837/cg22138430/cg24766334	0.23921772	0.317027636	0.398084526	0.158866806	5.60E-07	4.21E-06
UTR5	SLC25A34	1	16062915/16062894	2	cg03970032/cg15616400	0.583427555	0.495090472	0.428497568	-0.154929987	7.76E-11	3.23E-09
UTR5	SLC26A9	1	205897159/205906409/205909935/205897165	4	cg06437052/cg11335179/cg16003790/cg22863880	0.667467823	0.586239322	0.508433944	-0.159033878	3.81E-09	5.93E-08
UTR5	SLC27A6	5	128301328/128301232/128301230/128301488	4	cg02841155/cg07103493/cg17391928/cg25555059	0.405231333	0.465439279	0.578193858	0.172962525	6.52E-07	4.80E-06
UTR5	SLC32A1	20	37353117/37353158/37353126	3	cg08313939/cg10216615/cg24454144	0.250015918	0.34512885	0.489483206	0.239467288	2.72E-11	1.81E-09
UTR5	SLC35F1	6	118228869/118228871	2	cg11430157/cg26532358	0.100903596	0.172972118	0.269015835	0.168112239	1.81E-05	9.19E-05

UTR5	SLC35F3	1	234040765/234040781	2	cg05051043/cg09424759	0.30379271	0.357964728	0.510501209	0.206708499	1.30E-06	8.84E-06
UTR5	SLC36A2	5	150727045	1	cg19562321	0.47641976	0.321748678	0.196324459	-0.280095301	1.44E-14	3.57E-11
UTR5	SLC36A3	5	150683039/150683178	2	cg00109131/cg22445920	0.750848095	0.711970936	0.588135614	-0.162712481	1.18E-07	1.10E-06
UTR5	SLC39A4	8	145642243/145641882	2	cg06700234/cg22059438	0.54565889	0.434823242	0.340892114	-0.204766776	2.90E-11	1.87E-09
UTR5	SLC39A5	12	56624552/56623826/56623942	3	cg00668685/cg08214142/cg12745325	0.482296458	0.380748719	0.313085075	-0.169211383	5.42E-12	6.79E-10
UTR5	SLC44A5	1	76076088/76053565	2	cg15977773/cg17868936	0.582668055	0.55741365	0.432408841	-0.150259214	5.63E-07	4.23E-06
UTR5	SLC4A1	17	42344234/42345488/42345478	3	cg02998129/cg03580247/cg16063416	0.768574982	0.663660937	0.539253386	-0.229321595	1.87E-10	5.82E-09
UTR5	SLC5A1	22	32439052/32439254/32439259	3	cg03604322/cg03962522/cg09547224	0.427942595	0.368783344	0.251296545	-0.17664605	1.57E-06	1.04E-05
UTR5	SLC5A11	16	24864984/24857601/24865363/24857579/24862006	5	cg04022912/cg04756594/cg06028605/cg23280807/cg26870567	0.516950245	0.421185069	0.346774797	-0.170175448	2.15E-10	6.59E-09
UTR5	SLC6A17	1	110693985/110697298/110694747/110693757/110693237/110693612/110694263	7	cg01791587/cg05348973/cg05783233/cg05949020/cg11885396/cg13048512/cg15374686	0.304254397	0.390263773	0.532612144	0.228357747	2.61E-09	4.35E-08
UTR5	SLC6A2	16	55690564	1	cg04490714	0.321060065	0.374514678	0.499764455	0.17870439	1.23E-05	6.49E-05
UTR5	SLC8A3	14	70653919/70654290/70654639/70655686/70653719/70654824/70654597/70536311/70546695/70654247/70655056/70655382/70652710/70653964	14	cg00890485/cg01646639/cg04140862/cg05203877/cg10848272/cg12858895/cg13652513/cg15971826/cg16799926/cg19545232/cg20267521/cg20409590/cg22823236/cg23241781	0.288400506	0.360642314	0.460250721	0.171850215	1.21E-06	8.32E-06
UTR5	SLC9A11	1	173571308/173571345/173572181	3	cg02890044/cg15975283/cg25096142	0.790686825	0.674756647	0.601773645	-0.18891318	5.56E-09	8.15E-08
UTR5	SLFN11	17	33698288/33700513/33700230/33700295/33700255/33700180	6	cg01723139/cg05504685/cg08764758/cg12581244/cg18411103/cg25054416	0.365546976	0.434115784	0.515931222	0.150384246	4.61E-07	3.56E-06
UTR5	SLIT1	10	98945465/98945664	2	cg02741216/cg13196826	0.077506079	0.132029509	0.305303885	0.227797806	9.23E-05	0.000396439

UTR5	SLIT2	4	20255264/20255343	2	cg03742003/cg08428452	0.257782725	0.366029311	0.529762009	0.271979284	1.72E-07	1.52E-06
UTR5	SLIT3	5	168727752/168728081/168727762/168728076	4	cg01663016/cg07136998/cg09073398/cg11732619	0.318936618	0.409686742	0.554919341	0.235982722	1.46E-07	1.32E-06
UTR5	SLITRK1	13	84456486/84456127/84455742/84456410/84456171/84456308/84455651/84456258	8	cg01729717/cg07104706/cg10236414/cg18215217/cg19696317/cg20312205/cg22057720/cg26998274	0.356682661	0.463665988	0.606855882	0.250173222	2.80E-12	4.70E-10
UTR5	SLITRK5	13	88326918/88324879/88325384/88326752/88326244/88325263/88326635	7	cg04479219/cg06682197/cg07710481/cg12271981/cg16821446/cg19109538/cg19577779	0.225055161	0.301587606	0.457285961	0.2322308	7.74E-08	7.69E-07
UTR5	SLMAP	3	57743377	1	cg15172734	0.39085969	0.310144111	0.230296577	-0.160563113	2.30E-07	1.97E-06
UTR5	SLN	11	107582734/107578665/107579390/107578793	4	cg12237269/cg14434149/cg21832801/cg27262398	0.479840683	0.406178433	0.322702748	-0.157137935	3.41E-09	5.42E-08
UTR5	SMAGP	12	51663310/51663484	2	cg07663278/cg21513385	0.418494155	0.319970672	0.2576271	-0.160867055	5.94E-11	2.79E-09
UTR5	SMOC1	14	70346221/70346151	2	cg15996043/cg18493027	0.080132343	0.181771166	0.361537276	0.281404933	5.10E-06	2.94E-05
UTR5	SNAP91	6	84417933/84418659/84418789/84418724/84418433	5	cg07335294/cg15950068/cg16334314/cg21688264/cg24190603	0.314300099	0.403981004	0.583119889	0.26881979	1.59E-10	5.16E-09
UTR5	SNCG	10	88718317/88718393	2	cg03677069/cg21012874	0.497614665	0.392017356	0.296029682	-0.201584983	9.97E-11	3.73E-09
UTR5	SNTG2	2	946648	1	cg09426834	0.305865165	0.362097389	0.540930227	0.235065062	1.41E-06	9.50E-06
UTR5	SORCS3	10	106400880	1	cg16787600	0.341068585	0.5001111	0.680446518	0.339377933	1.49E-10	4.94E-09
UTR5	SOX1	13	112721950	1	cg16705627	0.63466706	0.682100433	0.805633495	0.170966435	4.07E-09	6.30E-08
UTR5	SOX14	3	137483617	1	cg06613095	0.181116544	0.230885608	0.385953961	0.204837417	0.003543619	0.010449761
UTR5	SOX17	8	55370544/55370579	2	cg02919422/cg26059468	0.485218765	0.550892078	0.669415714	0.184196949	1.00E-08	1.31E-07
UTR5	SOX7	8	10588013	1	cg08056146	0.078167564	0.105904248	0.230397847	0.152230283	0.005176467	0.014615213
UTR5	SPAG4L	20	31592147	1	cg02510802	0.74023952	0.573631311	0.371162491	-0.369077029	4.41E-12	5.82E-10
UTR5	SPAG6	10	22634432/22634439	2	cg05099508/cg24031355	0.14319122	0.251403974	0.375843679	0.23265246	1.01E-05	5.41E-05
UTR5	SPATA16	3	172859022/172858917/172858390/172841689/172856620/172858969	6	cg01216369/cg06577725/cg06728232/cg16760223/cg19391006/cg19933965	0.789834203	0.669323656	0.528735991	-0.261098212	9.73E-10	2.00E-08

UTR5	SPATS1	6	44310640/44310445	2	cg05010967/cg09662584	0.316295765	0.389153327	0.488022368	0.171726603	7.81E-07	5.64E-06
UTR5	SPG20	13	36920965/36919960/36919819/36919738/36920813/36924539/36920644/36920987/36919344/36921107/36944294/36919409/36916913/36920685/36920771/36921096/36920332/36919905/36927379/36919674/36920975/36920662/36921003/36918614/36920660/36921174	26	cg00576301/cg00947032/cg01404317/cg03966514/cg04117229/cg04610375/cg04939555/cg05123138/cg09072216/cg09184842/cg09190748/cg10558887/cg11583802/cg12363807/cg13996405/cg15390960/cg16741041/cg18755783/cg19712291/cg20691205/cg22170732/cg22609576/cg22831315/cg25179758/cg25468863/cg25921358	0.199111736	0.263337087	0.374902111	0.175790375	1.17E-05	6.21E-05
UTR5	SPHKAP	2	229046325	1	cg04072323	0.285360715	0.389797139	0.577976023	0.292615308	6.01E-09	8.67E-08
UTR5	SPO11	20	55904856/55904875	2	cg19911058/cg26645834	0.728778565	0.583588719	0.48247803	-0.246300535	1.48E-12	3.26E-10
UTR5	SPOCK1	5	136834877/136834294/136834863/136834492/136834453/136834392/136834464	7	cg05143123/cg09054633/cg12832649/cg14650610/cg18263365/cg18603028/cg24847829	0.263187511	0.34068157	0.506172196	0.242984685	1.88E-07	1.65E-06
UTR5	SPOCK3	4	168155510/168155695/168155687	3	cg00901765/cg09722742/cg13913012	0.134281269	0.199346221	0.333962825	0.199681555	3.23E-06	1.98E-05
UTR5	SPRR1A	1	152957379/152957018	2	cg07909128/cg27569034	0.68510742	0.56492285	0.513602959	-0.171504461	9.18E-06	5.00E-05
UTR5	SRD5A2	2	31805970	1	cg18948125	0.494859255	0.564542572	0.671387095	0.17652784	1.70E-11	1.44E-09
UTR5	SST	3	187388148/187388128	2	cg02164046/cg16927040	0.42472524	0.517541022	0.644218814	0.219493574	2.32E-11	1.69E-09
UTR5	SSTR2	17	71162349/71161502/71161322/71161926/71162393/71161178/71161796/71162159/71161160/71161415/71161258	11	cg00902823/cg03693811/cg06685177/cg09563244/cg09584801/cg13344169/cg14232289/cg18248022/cg21547708/cg26729197/cg27066284	0.12660554	0.194900051	0.292620743	0.166015203	0.000143893	0.000591301

UTR5	ST6GALNAC1	17	74639849/74639793/74639731	3	cg11469587/cg13015534/cg15503752	0.385521762	0.281389767	0.225724409	-0.159797353	2.00E-11	1.58E-09
UTR5	ST6GALNAC5	1	77333229/77333198	2	cg04077662/cg06201642	0.26808874	0.377675175	0.53033383	0.26224509	1.48E-07	1.33E-06
UTR5	ST8SIA1	12	22487437/22487459/22487219	3	cg09354309/cg10333808/cg24723331	0.135148496	0.178398841	0.346324339	0.211175843	0.00031756	0.001198556
UTR5	ST8SIA3	18	55019843	1	cg13096208	0.440201565	0.552931606	0.745647236	0.305445671	1.71E-12	3.47E-10
UTR5	ST8SIA4	5	100238977/100238983/100238819	3	cg03975797/cg04380513/cg18428516	0.123182906	0.209479751	0.350628455	0.227445549	7.85E-06	4.34E-05
UTR5	STAB2	12	103981113	1	cg10622586	0.53435505	0.432629811	0.376232255	-0.158122795	1.01E-09	2.05E-08
UTR5	STAP1	4	68424495	1	cg12879425	0.515848295	0.456623606	0.33362903	-0.182219265	0.000125038	0.000521071
UTR5	STMN2	8	80523413	1	cg09071889	0.37668888	0.499617317	0.643886368	0.267197488	1.24E-09	2.42E-08
UTR5	STOX2	4	184826953/184827086/184827754/184827491/184826704	5	cg01758805/cg02071076/cg02500300/cg06367693/cg11354594	0.222166173	0.24050692	0.39943448	0.177268306	0.00033048	0.00124203
UTR5	STRA6	15	74495354/74496040/74494909/74494861/74501317/74495109/74495603/74494781/74495657/74496576/74495637/74494854/74494900/74495384	14	cg00075967/cg00316939/cg00597876/cg01332054/cg11680605/cg11787522/cg13888509/cg17516330/cg18640660/cg21068293/cg22672067/cg23887948/cg26451735/cg26774156	0.598030574	0.521504559	0.435804479	-0.162226096	3.65E-12	5.48E-10
UTR5	STX19	3	93740824/93747410	2	cg16645976/cg27200466	0.58232939	0.443080756	0.341259436	-0.241069954	2.25E-11	1.67E-09
UTR5	SUNC1	7	48068665	1	cg23509098	0.70715429	0.561490989	0.376269341	-0.330884949	9.92E-14	6.84E-11
UTR5	SUSD5	3	33260366	1	cg09463233	0.175253587	0.312971031	0.436781936	0.261528349	9.08E-06	4.96E-05
UTR5	SV2A	1	149889364/149889361/149889339/149889330	4	cg01705587/cg04927502/cg11085602/cg24629122	0.090721684	0.157913011	0.265194618	0.174472933	6.84E-05	0.000303113
UTR5	SYCP1	1	115397857/115397597/115398073/115397616	4	cg10440578/cg18087477/cg18396987/cg27332337	0.495611519	0.594240294	0.681232969	0.185621451	6.64E-10	1.49E-08
UTR5	SYCP2L	6	10887185	1	cg26237681	0.159986403	0.224231299	0.459437943	0.29945154	3.42E-06	2.07E-05
UTR5	SYT8	11	1855746	1	cg03861143	0.55054825	0.440729306	0.354293586	-0.196254664	1.30E-11	1.18E-09
UTR5	SYT9	11	7273378	1	cg22723056	0.207953585	0.270369861	0.429017443	0.221063858	7.66E-05	0.000335215

UTR5	SYTL3	6	159071106/159084032/159075773/159071076	4	cg00370106/cg10296205/cg11879354/cg17768957	0.454297626	0.352622036	0.269022336	-0.18527529	1.75E-08	2.10E-07
UTR5	T	6	166581272/166581538/166581519/166581929	4	cg00070318/cg05478631/cg07991951/cg23688510	0.201115466	0.265016835	0.374334827	0.173219361	0.000614666	0.002160212
UTR5	TAC1	7	97361759/97361408	2	cg14221171/cg16288089	0.38903177	0.447987311	0.610351032	0.221319262	1.29E-05	6.77E-05
UTR5	TACR3	4	104640833	1	cg16461251	0.38470884	0.475705944	0.620553073	0.235844233	6.65E-09	9.39E-08
UTR5	TBX15	1	119530144/119529930/119527008/119531718/119529360/119530600/119528018/119526783/119529219/119522637/119528424/119528638/119532093/119529266/119524323/119527884/119532116/119521928/119527156/119522425/119522386/119527638/119531122/119532044/119528688/119520934/119526060/119502537/119522855/119526437/119528164/119531625/119530677/119527111/119522188/119530048/119526882/119530932/119526255/119531857/119532056/119531093/119527377/119519115/119530702/119528848	46	cg00173504/cg02177231/cg02829688/cg03760839/cg03989260/cg04550737/cg04707332/cg05172122/cg05363616/cg06158650/cg06204711/cg07820868/cg07892597/cg09260773/cg09789768/cg10145246/cg10703826/cg11391335/cg13080379/cg13245593/cg13655674/cg14175690/cg14415885/cg14565725/cg15513743/cg15553612/cg16990168/cg17868079/cg18944010/cg19730691/cg21170796/cg21295088/cg21392341/cg21647227/cg22378919/cg23588217/cg24144440/cg24475782/cg24720355/cg24842086/cg24884142/cg25755283/cg26104752/cg26164798/cg27262412/cg27517681	0.360006966	0.408948693	0.531217371	0.171210405	2.12E-07	1.83E-06

UTR5	TBX20	7	35293537/35293309/35293279/35293245	4	cg02008154/cg08959216/cg10778441/cg18249173	0.31528686	0.383309981	0.471653007	0.156366147	0.000679395	0.002362931
UTR5	TCF21	6	134210273/134210279/134210307	3	cg12468478/cg17470942/cg24157171	0.345662453	0.370698205	0.496025864	0.150363411	3.84E-06	2.29E-05
UTR5	TCP10	6	167797877	1	cg19930296	0.7115431	0.607950922	0.492313495	-0.219229605	3.59E-08	3.93E-07
UTR5	TCP10L	21	33956668/33957290	2	cg04986276/cg20857253	0.525256333	0.431422028	0.353399936	-0.171856396	1.34E-12	3.05E-10
UTR5	TCP10L2	6	167584900/167584201	2	cg11121709/cg16214245	0.718269	0.609363256	0.498562314	-0.219706686	6.80E-08	6.85E-07
UTR5	TCTEX1D1	1	67218505/67218275/67218165	3	cg19159365/cg24161501/cg25339566	0.339405563	0.419645643	0.538412515	0.199006952	1.56E-05	8.04E-05
UTR5	TDO2	4	156824883	1	cg04795662	0.510825065	0.410470811	0.313949386	-0.196875679	2.15E-07	1.85E-06
UTR5	TEKT1	17	6734991/6734940	2	cg13337865/cg20663042	0.189928838	0.21198984	0.389665511	0.199736674	4.53E-06	2.65E-05
UTR5	TEKT2	1	36549719/36549726	2	cg19644018/cg24813212	0.123482982	0.178071389	0.275627008	0.152144026	0.000826083	0.002827919
UTR5	TEKT5	16	10788751	1	cg01573635	0.47936451	0.369222778	0.292530468	-0.186834042	1.50E-12	3.26E-10
UTR5	TET1	10	70321580/70320213/70320532/70321383/70320734/70321668/70321959/70321574/70321243/70321874/70322874/70322442/70320470/70321770/70321554/70321889	16	cg02774862/cg03651138/cg03756448/cg06767766/cg09183181/cg09978996/cg10403849/cg12630147/cg13848707/cg15254238/cg17817532/cg19127638/cg19439331/cg20326704/cg23350336/cg25926515	0.376264301	0.463444731	0.534331812	0.158067511	5.85E-09	8.50E-08
UTR5	TF	3	133465180	1	cg24879335	0.359086565	0.403408922	0.534619114	0.175532549	7.70E-05	0.000336616
UTR5	TFEC	7	115670795	1	cg15339605	0.260855737	0.142948516	0.083422935	-0.177432802	2.39E-08	2.74E-07
UTR5	TFF1	21	43786627	1	cg06099014	0.495460795	0.409595083	0.328818418	-0.166642377	2.91E-11	1.87E-09
UTR5	TFPI2	7	93520036/93520024/93520012	3	cg14775114/cg17338208/cg24531255	0.139304337	0.329598872	0.545375324	0.406070988	2.55E-11	1.74E-09
UTR5	TGM3	20	2276663	1	cg24731241	0.624083145	0.540739672	0.429558477	-0.194524668	2.28E-10	6.88E-09
UTR5	TGM4	3	44916102	1	cg26264008	0.49521531	0.4171876	0.333413573	-0.161801737	1.62E-08	1.96E-07
UTR5	THBS4	5	79331047/79331135	2	cg03336086/cg23244289	0.21131581	0.27488459	0.410746151	0.199430341	2.79E-05	0.000134558
UTR5	THEM5	1	151826167/151826123	2	cg11344950/cg18081104	0.50145591	0.420248339	0.32742578	-0.17403013	1.44E-10	4.82E-09

UTR5	THY1	11	119293385/119291765/119293601/119292885/119292737/119293861/119293780/119293869/119293863/119292770	10	cg02455397/cg02632800/cg02955631/cg04976484/cg05916744/cg10601287/cg12508624/cg16566400/cg17236709/cg20884984	0.216442944	0.280876213	0.40587568	0.189432736	6.44E-07	4.75E-06
UTR5	TLL1	4	166794471/166794895/166794971/166794786	4	cg08570521/cg09669853/cg19898128/cg24521633	0.14149001	0.220108282	0.378027405	0.236537396	9.05E-06	4.95E-05
UTR5	TLX1	10	102891076/102891280/102891080	3	cg02096397/cg05329692/cg27272677	0.223234961	0.216491831	0.393620272	0.170385311	0.000543386	0.001933252
UTR5	TLX3	5	170736335	1	cg02743222	0.338009175	0.343386228	0.514287045	0.17627787	0.001138108	0.003760152
UTR5	TM4SF19	3	196065189/196065106	2	cg02322879/cg05556202	0.732734068	0.6506297	0.554201373	-0.178532695	6.22E-08	6.32E-07
UTR5	TM4SF4	3	149192609	1	cg05483388	0.533137955	0.438724928	0.357236132	-0.175901823	3.76E-12	5.48E-10
UTR5	TM6SF1	15	83776422/83776420	2	cg03063639/cg26460092	0.288529638	0.477104186	0.674643993	0.386114356	3.82E-14	3.95E-11
UTR5	TMCO5A	15	38228220	1	cg03832697	0.73689691	0.667293122	0.542543155	-0.194353755	2.33E-06	1.48E-05
UTR5	TMEFF2	2	193059405	1	cg06856528	0.23311806	0.307321056	0.414922759	0.181804699	4.02E-05	0.000187886
UTR5	TMEM126A	11	85359218/85359560	2	cg06698742/cg26796873	0.426457665	0.344058511	0.248411477	-0.178046188	3.74E-10	9.79E-09
UTR5	TMEM130	7	98467571	1	cg01307939	0.072572016	0.210948573	0.306677468	0.234105452	2.16E-11	1.65E-09
UTR5	TMEM132D	12	130388198	1	cg23266743	0.351685445	0.429984967	0.534556495	0.18287105	2.16E-06	1.38E-05
UTR5	TMEM139	7	142982463/142982262	2	cg08261841/cg26511386	0.576420968	0.44608855	0.335559352	-0.240861615	4.41E-12	5.82E-10
UTR5	TMEM155	4	122685352/122686269/122685570/122686038/122685651/122685145/122685675/122685641/122684903/122685423/122686319	11	cg03077077/cg05396987/cg10620484/cg10863741/cg13640145/cg14654731/cg16790291/cg18149485/cg19122004/cg19737970/cg21077559	0.465590966	0.536994112	0.62922631	0.163635344	4.87E-08	5.09E-07
UTR5	TMEM196	7	19812327	1	cg08991339	0.276333955	0.324308372	0.432952155	0.1566182	1.58E-05	8.11E-05
UTR5	TMEM215	9	32783516	1	cg11308840	0.262321	0.324663561	0.42953105	0.16721005	4.92E-05	0.000224677
UTR5	TMEM220	17	10633470	1	cg01275588	0.219031841	0.251888634	0.387590759	0.168558918	0.010184019	0.026609717
UTR5	TMEM71	8	133772657/133772889/133772742	3	cg03141298/cg20955688/cg27159719	0.321192697	0.169322804	0.169164931	-0.152027765	9.77E-06	5.28E-05
UTR5	TMEM79	1	156254149/156254757	2	cg00249974/cg05954120	0.63132282	0.508628472	0.461014418	-0.170308402	4.96E-09	7.40E-08

UTR5	TMEM90B	20	24455463/24450353/24452036/24451084/24449865/24449916/24451518/24452131/24451769/24450698/24450361/24452007/24451890/24451428/24452091	15	cg06271023/cg09623400/cg12831866/cg15343461/cg16098981/cg16120788/cg16793866/cg16847695/cg17523124/cg17739841/cg19654195/cg21338660/cg23849311/cg24504569/cg24598087	0.248426224	0.327700825	0.422902594	0.17447637	1.80E-05	9.13E-05
UTR5	TMPRSS11B	4	69111391	1	cg06399881	0.723383995	0.599150383	0.495239614	-0.228144381	1.62E-09	2.96E-08
UTR5	TMPRSS11E	4	69313197	1	cg24792113	0.396462015	0.294216533	0.240996355	-0.15546566	1.26E-09	2.44E-08
UTR5	TMPRSS4	11	117947997/117947877	2	cg05416223/cg22957898	0.420708625	0.338771175	0.268289677	-0.152418948	8.31E-11	3.33E-09
UTR5	TMTC1	12	29925201/29936075/29937343/29935873/29936839/29936174/29936407/29937188/29932047/29936486/29935411/29936972/29936125/29936845/29937585	15	cg01401465/cg04822518/cg06630204/cg08275602/cg08729318/cg10512875/cg12718339/cg13239483/cg13318543/cg13434308/cg14258853/cg15720995/cg19792268/cg20140110/cg20784775	0.327751996	0.374996381	0.4922112	0.164459204	9.40E-07	6.64E-06
UTR5	TNFAIP2	14	103592734	1	cg04264002	0.54440615	0.451647061	0.385773686	-0.158632464	1.62E-06	1.07E-05
UTR5	TNS4	17	38657310/38655492/38655903	3	cg08696192/cg15379400/cg27016929	0.46670449	0.350242465	0.277668714	-0.189035776	2.25E-13	9.98E-11
UTR5	TP63	3	189349278	1	cg04483101	0.789884795	0.654894444	0.517826005	-0.27205879	2.99E-07	2.47E-06
UTR5	TPD52	8	80992750/81083829	2	cg16773115/cg22273042	0.61247576	0.492659661	0.383975359	-0.228500401	2.19E-11	1.65E-09
UTR5	TPD52L3	9	6328386/6328493	2	cg03485840/cg14286682	0.624802443	0.523139206	0.448331048	-0.176471395	7.60E-11	3.22E-09
UTR5	TPM4	19	16178426/16187364	2	cg22270364/cg25593560	0.784131385	0.736797406	0.588106991	-0.196024394	0.000303998	0.001156514
UTR5	TPTE2	13	20110871	1	cg19950767	0.73145477	0.68496045	0.573175373	-0.158279397	2.86E-06	1.78E-05
UTR5	TREM2	6	41130917	1	cg01980222	0.5824335	0.493762728	0.41245735	-0.16997615	2.57E-06	1.62E-05

UTR5	TRH	3	129693613/129693586/129694057/129693791/129694487/129693385/129694156/129693370/129693489/129694392	10	cg01009664/cg02700891/cg05013728/cg05835982/cg08195943/cg11940285/cg14595003/cg18862481/cg22512438/cg22931604	0.303001046	0.394063468	0.582192628	0.279191582	2.75E-09	4.52E-08
UTR5	TRIM31	6	30080782/30080642	2	cg00679556/cg03339321	0.54873495	0.415189644	0.298069295	-0.250665655	1.89E-12	3.63E-10
UTR5	TRIM42	3	140396963	1	cg06090362	0.55280799	0.451339978	0.374193964	-0.178614026	5.27E-08	5.46E-07
UTR5	TRIM43B	2	96149768	1	cg00135121	0.803248445	0.751737411	0.639437432	-0.163811013	1.30E-06	8.83E-06
UTR5	TRIM61	4	165898666/165898707	2	cg16691177/cg25147158	0.521835355	0.616170856	0.697822795	0.17598744	8.25E-09	1.12E-07
UTR5	TRIM71	3	32859558	1	cg23243038	0.213643365	0.280635194	0.42770525	0.214061885	3.59E-05	0.000169486
UTR5	TRPC6	11	101454317/101454626	2	cg03411507/cg11016563	0.33482509	0.403225172	0.58180788	0.24698279	2.97E-11	1.89E-09
UTR5	TRPC7	5	135701094/135701050	2	cg06448705/cg23288973	0.416880065	0.493009661	0.615086455	0.19820639	1.17E-08	1.50E-07
UTR5	TRPM6	9	77502963	1	cg16509045	0.516212135	0.397709394	0.298444241	-0.217767894	2.33E-11	1.69E-09
UTR5	TSPAN16	19	11406877	1	cg25382751	0.71516107	0.638278567	0.526796245	-0.188364825	5.46E-06	3.13E-05
UTR5	TSPYL5	8	98290148	1	cg18233405	0.201159378	0.305637734	0.42233355	0.221174172	2.20E-09	3.78E-08
UTR5	TSSK6	19	19626428/19626251	2	cg03854071/cg23069923	0.632560395	0.530680122	0.459436059	-0.173124336	3.76E-09	5.88E-08
UTR5	TTN	2	179672121/179672126	2	cg05185019/cg10859358	0.776356565	0.58309665	0.495723477	-0.280633088	8.73E-09	1.17E-07
UTR5	TUSC3	8	15397899/15397731/15397729	3	cg03032098/cg03127174/cg18145877	0.356067475	0.480679052	0.611933545	0.25586607	3.46E-09	5.46E-08
UTR5	TWIST1	7	19157193/19157263	2	cg17839237/cg24446548	0.083262939	0.206188919	0.519511387	0.436248448	2.31E-07	1.97E-06
UTR5	TXLNB	6	139613145	1	cg18953104	0.409258315	0.539306706	0.625136177	0.215877862	1.34E-09	2.54E-08
UTR5	UBE2QL1	5	6449001	1	cg26692294	0.08178838	0.158280672	0.276657516	0.194869137	3.83E-05	0.000179623
UTR5	UCHL1	4	41258910/41258935	2	cg07068756/cg16142306	0.180193261	0.216928306	0.377133643	0.196940382	0.001489093	0.004780974
UTR5	UGT1A6	2	234601254/234600423/234601443/234601954	4	cg04449108/cg23338993/cg23510527/cg23874600	0.583247518	0.479041402	0.415079109	-0.168168409	9.67E-11	3.69E-09
UTR5	UGT3A1	5	35991382	1	cg19897071	0.50859441	0.588819472	0.686613518	0.178019108	2.84E-08	3.20E-07
UTR5	UGT3A2	5	36066972/36066944	2	cg10140794/cg27638727	0.24182068	0.320017039	0.399293809	0.157473129	2.25E-06	1.43E-05
UTR5	UMOD	16	20363009/20363711	2	cg04231019/cg07456201	0.656458963	0.488502464	0.322445711	-0.334013251	6.88E-13	2.19E-10
UTR5	UNC45B	17	33475031/33474914	2	cg25013053/cg26751372	0.82058443	0.772294197	0.670343723	-0.150240707	3.65E-06	2.19E-05
UTR5	UNC5C	4	96470053/96470094	2	cg00329039/cg10520887	0.247111658	0.332582539	0.448170536	0.201058879	3.76E-06	2.25E-05

UTR5	UNC5CL	6	41006345	1	cg09489757	0.76133877	0.690613439	0.568967068	-0.192371702	4.74E-07	3.65E-06
UTR5	UNC80	2	210636748/210636783	2	cg13347071/cg24915947	0.29672162	0.357229878	0.484749443	0.188027823	1.03E-06	7.25E-06
UTR5	UPK1B	3	118903257/118895485	2	cg04857792/cg15717250	0.638241475	0.519996278	0.429178418	-0.209063057	3.31E-08	3.65E-07
UTR5	USH2A	1	216596639	1	cg15590780	0.428820805	0.352626139	0.254043968	-0.174776837	5.18E-12	6.55E-10
UTR5	USP44	12	95941920/95941571/95942287/95945120/95942964/95943114/95945082/95942907/95942847/95941988/95942761/95941869/95939442	13	cg00927554/cg03162823/cg03308628/cg03453449/cg07783282/cg09883286/cg11069430/cg13879483/cg17368254/cg22538054/cg22802813/cg23982858/cg26561986	0.329253982	0.384570931	0.534045518	0.204791536	4.01E-06	2.38E-05
UTR5	VAT1L	16	77822500	1	cg01256089	0.105097593	0.179936416	0.274560032	0.169462439	0.001965622	0.006134719
UTR5	VCAM1	1	101185311	1	cg25762679	0.47501733	0.571155178	0.627295764	0.152278434	2.94E-06	1.82E-05
UTR5	VEGFC	4	177713813	1	cg13378388	0.29362454	0.326766511	0.49613375	0.20250921	0.000434442	0.001581944
UTR5	VIPR2	7	158937610/158937494	2	cg03976877/cg13794530	0.344609673	0.441673113	0.646611789	0.302002116	8.30E-13	2.38E-10
UTR5	VSTM2A	7	54610022/54610192/54610139/54610320	4	cg02910208/cg03775422/cg04024095/cg14304469	0.27133063	0.342809737	0.472008952	0.200678322	2.41E-05	0.000118037
UTR5	VSTM2L	20	36531652	1	cg26366107	0.115763061	0.169990506	0.278770598	0.163007538	0.000469668	0.001696767
UTR5	VSX1	20	25062754	1	cg00043788	0.33244861	0.353225556	0.551050359	0.218601749	3.99E-07	3.17E-06
UTR5	VSX2	14	74706196	1	cg07573727	0.28435026	0.385045844	0.5782105	0.29386024	9.35E-08	9.03E-07
UTR5	VWC2	7	49813763/49814781/49814435/49814597/49813486	5	cg00333226/cg05470523/cg07039180/cg07379434/cg18206027	0.204754923	0.225809094	0.388402172	0.183647249	4.99E-05	0.000227525
UTR5	WDR17	4	176987577/176987020/176987313/176987359/176987009/176987453/176987174	7	cg03909602/cg08095852/cg08684639/cg11674404/cg11923920/cg25276412/cg27486637	0.135154711	0.249051956	0.361266876	0.226112166	3.29E-07	2.69E-06
UTR5	WDR86	7	151106990	1	cg12433277	0.31276473	0.418221856	0.539497191	0.226732461	7.03E-07	5.13E-06
UTR5	WISP3	6	112375302/112375333/112375324	3	cg07709205/cg10525372/cg21773633	0.52809172	0.437901892	0.354784148	-0.173307572	2.37E-11	1.69E-09
UTR5	WNT2	7	116963259/116963193	2	cg07697895/cg27331524	0.269514918	0.365072882	0.541466436	0.271951519	9.01E-10	1.89E-08
UTR5	XKR3	22	17290861	1	cg07193389	0.405922355	0.307553306	0.238541805	-0.16738055	7.81E-12	8.70E-10
UTR5	XKR7	20	30555908	1	cg18405539	0.09956515	0.173101257	0.264530641	0.164965491	0.002093947	0.006487889

UTR5	ZEB2	2	145277807/145277646/145277852/145277659/145277790/145274975/145275363/145277734/145275322/145275197/145277741/145275441/145275868/145275199/145277596/145277843	16	cg00323965/cg03424727/cg04836221/cg07246225/cg09670616/cg11540007/cg13887004/cg14421309/cg15120754/cg17751438/cg19123296/cg19190728/cg20177310/cg21701531/cg23639412/cg26522240	0.111480049	0.145501947	0.293038442	0.181558394	3.11E-05	0.000148962
UTR5	ZFP28	19	57050370/57050367/57050359	3	cg21970554/cg22054362/cg24152605	0.279058381	0.324144566	0.470186472	0.191128091	0.000719048	0.002484821
UTR5	ZFP82	19	36909326/36906819/36909413/36909418	4	cg07900968/cg19750174/cg24724633/cg25886284	0.340750433	0.49869339	0.634785768	0.294035336	4.39E-08	4.67E-07
UTR5	ZIC1	3	147127579/147127662/147127193/147127898	4	cg01610632/cg04738965/cg05073035/cg26375057	0.444019475	0.510451474	0.642729435	0.19870996	8.96E-10	1.88E-08
UTR5	ZIK1	19	58095659	1	cg26246807	0.375614655	0.597706644	0.758164927	0.382550272	1.78E-10	5.64E-09
UTR5	ZNF132	19	58951434	1	cg19776201	0.121933814	0.220579396	0.34146989	0.219536076	8.65E-05	0.000374994
UTR5	ZNF134	19	58125953/58129233/58125867/58126053	4	cg08849574/cg13715319/cg18113994/cg23950001	0.196277209	0.338983166	0.474386418	0.278109209	3.00E-07	2.48E-06
UTR5	ZNF135	19	58570995/58570790	2	cg22457238/cg22748799	0.36675218	0.4747417	0.624085245	0.257333065	1.52E-09	2.83E-08
UTR5	ZNF154	19	58220370/58220494/58220516	3	cg05661282/cg21790626/cg27049766	0.477791493	0.60086215	0.697451393	0.219659901	1.32E-09	2.51E-08
UTR5	ZNF169	9	97021919/97022269/97025960/97040460/97021966	5	cg09923314/cg13980266/cg14032959/cg15264273/cg16093876	0.444990219	0.331727033	0.25659538	-0.188394839	5.36E-13	1.80E-10
UTR5	ZNF177	19	9476402/9473696/9473715/9474128/9473880/9473781	6	cg02672003/cg09643544/cg12089570/cg14737994/cg17283453/cg24189904	0.483899472	0.57424297	0.674350926	0.190451454	1.06E-10	3.90E-09
UTR5	ZNF208	19	22193724	1	cg26938561	0.51009076	0.573753211	0.687665023	0.177574263	2.48E-11	1.71E-09
UTR5	ZNF238	1	244214593/244214679	2	cg01105418/cg23829949	0.551873955	0.439334472	0.352255439	-0.199618516	3.77E-12	5.48E-10
UTR5	ZNF256	19	58458917/58458979	2	cg14556070/cg20176532	0.177109025	0.217259773	0.435286696	0.258177672	3.65E-05	0.000171958

UTR5	ZNF257	19	22235281/22235272/22235409	3	cg02912127/cg24072202/cg25514554	0.40164807	0.510587817	0.599723843	0.198075773	4.12E-10	1.05E-08
UTR5	ZNF264	19	57702917/57702927/57703144/57702994	4	cg08504049/cg22307908/cg23257840/cg24150232	0.064751927	0.119210851	0.293953594	0.229201667	0.000249647	0.000969062
UTR5	ZNF280A	22	22874447/22874590	2	cg08088989/cg20403031	0.670706765	0.544298861	0.4968019	-0.173904865	2.72E-05	0.000131645
UTR5	ZNF285A	19	44905343	1	cg09030119	0.315716085	0.3535753	0.481396682	0.165680597	9.52E-05	0.000407002
UTR5	ZNF304	19	57862713	1	cg07494047	0.084922142	0.236218978	0.429114705	0.344192563	2.47E-07	2.08E-06
UTR5	ZNF382	19	37096321/37096487/37096329/37099702/37096323/37096816/37096588	7	cg02587316/cg04332534/cg05020604/cg15463135/cg18630667/cg20440041/cg22517995	0.480212297	0.564451695	0.653332141	0.173119844	4.51E-10	1.11E-08
UTR5	ZNF385D	3	21792684/21792434/21792543	3	cg00485194/cg18267381/cg18503679	0.322803982	0.428720288	0.56774422	0.244940238	1.32E-09	2.51E-08
UTR5	ZNF389	6	28129556/28129616	2	cg08968635/cg26335602	0.29862421	0.420266606	0.550124559	0.251500349	8.40E-07	6.02E-06
UTR5	ZNF418	19	58446600/58446436/58446669/58446312	4	cg07474494/cg08648727/cg11998703/cg26671652	0.385154163	0.514900719	0.658765421	0.273611258	3.60E-10	9.55E-09
UTR5	ZNF492	19	22817371/22817176/22817496/22817348/22817774	5	cg01485075/cg03230154/cg18365865/cg18599790/cg21466963	0.321620655	0.403231496	0.556110682	0.234490027	6.48E-09	9.21E-08
UTR5	ZNF543	19	57831909	1	cg04696193	0.090497869	0.197819797	0.282008214	0.191510345	0.000735246	0.002535852
UTR5	ZNF544	19	58740365/58740455/58740549/58740861/58740253/58740255/58740747	7	cg04352026/cg08397273/cg10051935/cg14386312/cg21155461/cg21747958/cg24167652	0.134519428	0.196379974	0.323874238	0.18935481	0.001708222	0.005418614
UTR5	ZNF547	19	57875238/57874988/57875014/57878074/57875295	5	cg03007201/cg10066151/cg12230795/cg17313042/cg21068576	0.108855118	0.166959063	0.285722302	0.176867184	4.08E-05	0.000190216
UTR5	ZNF549	19	58038856/58038742	2	cg07054095/cg13644629	0.160202411	0.300530641	0.464759873	0.304557462	5.76E-09	8.37E-08
UTR5	ZNF560	19	9609204/9605778/9609152/9608947/9608637	5	cg04062391/cg11396499/cg15222651/cg16646743/cg18452695	0.473421404	0.548576922	0.629188636	0.155767232	5.13E-10	1.21E-08

UTR5	ZNF568	19	37407284/37407374/37407462/37409826/37407486/37407889/37407257/37407413	8	cg02459604/cg03060201/cg03443751/cg03530260/cg20154403/cg21117780/cg21761844/cg26612849	0.231325747	0.337568668	0.433281885	0.201956138	5.44E-05	0.000246066
UTR5	ZNF586	19	58281117	1	cg22956410	0.104819689	0.117110548	0.328906385	0.224086697	0.001471101	0.004729329
UTR5	ZNF606	19	58513648/58514119/58513526/58514022/58513203/58514694/58514497	7	cg00664634/cg01214989/cg06368093/cg08397818/cg11740087/cg12259537/cg25480379	0.307946371	0.380300445	0.485988408	0.178042036	5.74E-06	3.27E-05
UTR5	ZNF625	19	12267308/12267464/12266489	3	cg00875960/cg17892556/cg22606224	0.285091691	0.489904084	0.591177046	0.306085355	1.47E-07	1.33E-06
UTR5	ZNF660	3	44626538/44626860/44626697/44626492/44629283	5	cg01139508/cg15688619/cg17536166/cg22598028/cg27607520	0.277848656	0.340552112	0.450679912	0.172831256	3.02E-06	1.86E-05
UTR5	ZNF671	19	58238987/58238928	2	cg11977686/cg19246110	0.266571018	0.393356944	0.579648984	0.313077966	5.62E-08	5.79E-07
UTR5	ZNF677	19	53758055/53757910/53757677/53758031	4	cg03217253/cg18335068/cg21907914/cg24685755	0.335146615	0.442322344	0.568818125	0.23367151	7.11E-08	7.13E-07
UTR5	ZNF772	19	57988860	1	cg26929012	0.085492994	0.170699922	0.28206878	0.196575786	1.47E-05	7.64E-05
UTR5	ZNF773	19	58011393/58011309/58011345	3	cg00757182/cg09555914/cg22806837	0.039144991	0.141414265	0.224253849	0.185108858	0.000296824	0.001130958
UTR5	ZNF781	19	38183130/38182773/38182950/38180021/38183210/38183055	6	cg04784475/cg06940614/cg09449030/cg17823234/cg22521696/cg25875213	0.127790987	0.181818997	0.328970448	0.20117946	0.000338656	0.001268912
UTR5	ZNF793	19	37998171/37999281/37997867/37997991	4	cg04818919/cg12283916/cg15139588/cg18154014	0.290045332	0.378452602	0.504859287	0.214813955	1.59E-06	1.05E-05
UTR5	ZNF804A	2	185463132/185463209/185463583/185463588/185463218	5	cg00554682/cg13107760/cg13536757/cg16573328/cg21245277	0.093647484	0.124077633	0.269781843	0.176134359	0.000321392	0.001211175
UTR5	ZNF813	19	53971035/53973920/53971464	3	cg12142445/cg22866218/cg27264706	0.11934244	0.204465153	0.378819685	0.259477245	4.20E-05	0.000195457
UTR5	ZNF814	19	58400325/58400275	2	cg15759937/cg24124798	0.218604415	0.327995989	0.5074221	0.288817685	5.60E-07	4.21E-06

UTR5	ZNF829	19	37407060/37407041/37406349/37407152/37406932/37406949/37407123	7	cg02370417/cg03584288/cg09560297/cg15322783/cg15746891/cg21292152/cg24052101	0.204806073	0.325622321	0.425634913	0.220828841	4.02E-05	0.000187652
UTR5	ZNF880	19	52873173	1	cg03005603	0.264131695	0.347941017	0.448765031	0.184633336	0.00027543	0.001056581
UTR5	ZSCAN18	19	58609269/58609473/58609361/58607035/58609618/58609602	6	cg00370414/cg06243556/cg21653184/cg21890309/cg22721334/cg25784220	0.447807464	0.506429461	0.601717486	0.153910022	0.000176876	0.00071152
UTR5	ZSCAN23	6	28411271/28411030/28411240	3	cg06818710/cg07838603/cg13525197	0.204968139	0.274659697	0.464516997	0.259548858	5.29E-07	4.02E-06
EXON1	A1CF	10	52645399/52645408	2	cg03817621/cg24411946	0.63791302	0.522432422	0.415836795	-0.222076225	2.68E-11	2.08E-09
EXON1	A2ML1	12	8975196/8975198	2	cg03490200/cg13863204	0.798196353	0.682005733	0.605282773	-0.19291358	5.85E-07	5.52E-06
EXON1	A4GNT	3	137851111	1	cg17687282	0.555490305	0.439574633	0.352429409	-0.203060896	4.65E-08	6.18E-07
EXON1	ABCB5	7	20687223	1	cg22066521	0.840938375	0.707326378	0.538969	-0.301969375	6.19E-12	7.73E-10
EXON1	ABCC11	16	48265760	1	cg09228051	0.527173005	0.33331525	0.231701973	-0.295471032	8.37E-12	9.97E-10
EXON1	ABCC2	10	101542587	1	cg14947634	0.80488305	0.72094985	0.644778177	-0.160104873	2.62E-04	0.001249793
EXON1	ABCC9	12	22089560	1	cg20025970	0.69504631	0.639950522	0.503078109	-0.191968201	0.000783363	0.003331118
EXON1	ACAN	15	89346989/89346795	2	cg03184141/cg20510724	0.3016413	0.409444617	0.493055441	0.191414141	2.26E-07	2.43E-06
EXON1	ACCSL	11	44069581	1	cg23980760	0.65016958	0.530142694	0.413524532	-0.236645048	3.77E-11	2.64E-09
EXON1	ACER1	19	6333587/6333593/6333547	3	cg12999109/cg19598519/cg25221254	0.7483543	0.623571344	0.550758623	-0.197595677	4.60E-09	9.04E-08
EXON1	ACSS3	12	81472177/81471938/81471882/81471867/81472118/81471884	6	cg01283289/cg05143887/cg10789281/cg13273396/cg24711649/cg26272220	0.333184308	0.50958241	0.580906063	0.247721755	1.52E-07	1.73E-06
EXON1	ACTN2	1	236849942/236849966/236849994	3	cg11215976/cg18770350/cg20482698	0.455674445	0.51672723	0.614542092	0.158867647	5.04E-07	4.83E-06
EXON1	ADAM28	8	24151625	1	cg22915945	0.315348114	0.155805396	0.11973464	-0.195613474	1.37E-07	1.58E-06
EXON1	ADAMTS16	5	5140646	1	cg22784954	0.491887775	0.589761867	0.707872386	0.215984611	5.30E-08	6.91E-07
EXON1	ADAMTS18	16	77468893/77468850	2	cg03238797/cg07568194	0.242036425	0.243960726	0.416279982	0.174243557	0.000215896	0.001055074
EXON1	ADAMTS2	5	178772203	1	cg05214690	0.1248214	0.207791128	0.320605475	0.195784076	1.09E-06	9.64E-06
EXON1	ADAMTS20	12	43945680	1	cg11751806	0.336769935	0.441441006	0.572781686	0.236011751	9.48E-08	1.15E-06

EXON1	ADAMTS5	21	28338836/28337857/28337869/28339334/28339262	5	cg03202077/cg08979041/cg12078031/cg15237494/cg23986671	0.375383208	0.462682385	0.618631833	0.243248625	1.98E-08	3.03E-07
EXON1	ADARB2	10	1779432/1779667	2	cg00467420/cg05307923	0.152456797	0.280145489	0.436033709	0.283576912	4.02E-07	3.98E-06
EXON1	ADCY4	14	24803807	1	cg16761581	0.166709615	0.198068025	0.32355559	0.156845975	6.77E-03	2.29E-02
EXON1	ADCY8	8	132052779/132052702/132052044	3	cg03120091/cg05229355/cg12446629	0.339781073	0.426269307	0.570765788	0.230984715	9.65E-10	2.53E-08
EXON1	ADCYAP1	18	905382/905450/904963	3	cg07211875/cg07376535/cg11850773	0.377236108	0.476772894	0.589408932	0.212172823	4.26E-09	8.48E-08
EXON1	ADH6	4	100140325/100140396	2	cg06518271/cg08988543	0.581643105	0.443150483	0.352798286	-0.228844819	6.58E-11	3.84E-09
EXON1	ADHFE1	8	67344720	1	cg20912169	0.256459935	0.411193623	0.660422359	0.403962425	7.83E-11	4.17E-09
EXON1	ADIG	20	37209999/37209890	2	cg14271690/cg17349406	0.718053708	0.592508044	0.524476436	-0.193577271	2.90E-09	6.04E-08
EXON1	ADRA1A	8	26721870/26722635/26721736/26722861/26722496	5	cg02409177/cg07645844/cg09557462/cg17963840/cg21230493	0.437989944	0.51766866	0.635304282	0.197314338	3.47E-08	4.89E-07
EXON1	ADRA2C	4	3769558/3769468/3768458/3768638/3769754/3768555/3769810	7	cg02086094/cg04167239/cg04888241/cg06149034/cg10231049/cg10235817/cg13244559	0.370857996	0.418502919	0.532037665	0.161179669	8.11E-10	2.24E-08
EXON1	AEBP1	7	44143998/44144434/44144360/44143993/44144279	5	cg02126753/cg06128448/cg08739576/cg14249876/cg27493928	0.14101586	0.261169783	0.450443613	0.309427753	1.39E-07	1.60E-06
EXON1	AGR2	7	16844606	1	cg21201572	0.58272229	0.446446089	0.331783418	-0.250938872	8.14E-12	9.97E-10
EXON1	AKR1B1	7	134143823	1	cg18416881	0.146743665	0.255080443	0.329499074	0.18275541	3.48E-03	1.27E-02
EXON1	AKR1B10	7	134212463	1	cg25171118	0.49394053	0.383134544	0.2866655	-0.20727503	1.87E-10	7.41E-09
EXON1	AKR1C4	10	5238881	1	cg09272256	0.60339281	0.527832572	0.434293668	-0.169099142	1.01E-07	1.21E-06
EXON1	ALDH3B2	11	67442075/67442067	2	cg07891457/cg20420868	0.60026377	0.482674306	0.378574191	-0.221689579	1.70E-10	7.02E-09
EXON1	ALK	2	30142990/30144376/30143585/30143739/30144152	5	cg02355885/cg06905692/cg07443217/cg12976501/cg18277754	0.264042581	0.363556944	0.467856574	0.203813992	6.02E-10	1.74E-08
EXON1	ALOX12B	17	7990705/7990985	2	cg03742272/cg05952715	0.754120235	0.663542361	0.559306527	-0.194813708	1.12E-09	2.82E-08
EXON1	ALPI	2	233320854	1	cg08210297	0.528081415	0.441415483	0.3559656	-0.172115815	8.65E-10	2.34E-08
EXON1	ALPL	1	21835945	1	cg05364759	0.215893632	0.240307554	0.424732568	0.208838937	3.99E-06	3.01E-05

EXON1	ALPP	2	233243398	1	cg19654061	0.600517355	0.462496261	0.365646864	-0.234870491	8.19E-11	4.31E-09
EXON1	ALX3	1	110613072	1	cg05622465	0.163861709	0.208511138	0.372320473	0.208458764	1.65E-04	0.000833103
EXON1	ALX4	11	44331629/44331577/44331362/44331623	4	cg02504416/cg04988423/cg15817236/cg22931182	0.194598571	0.243938988	0.385977323	0.191378752	1.72E-05	0.000110777
EXON1	AMOTL1	11	94501636/94501718	2	cg09671810/cg11117364	0.232078258	0.281349986	0.4418333	0.209755043	6.48E-04	0.002813595
EXON1	AMPH	7	38670957/38670987/38670985/38671001	4	cg02383130/cg07926691/cg10293925/cg26122980	0.220771274	0.30209	0.49313757	0.272366296	4.26E-06	3.18E-05
EXON1	AMTN	4	71384359	1	cg05329893	0.581913015	0.491348928	0.376376132	-0.205536883	1.07E-06	9.45E-06
EXON1	ANGPT2	8	6420726/6420242/6420770/6420506	4	cg02402368/cg02548132/cg03631656/cg24670715	0.420858167	0.479337611	0.591663792	0.170805626	1.73E-06	1.44E-05
EXON1	ANGPTL2	9	129884948	1	cg13662634	0.438163435	0.511000944	0.598008373	0.159844938	3.47E-10	1.15E-08
EXON1	ANK1	8	41754172/41522721/41754181/41655078/41754060	5	cg05166490/cg12439423/cg17331296/cg26326633/cg26530758	0.358238866	0.433221078	0.510682627	0.152443761	1.19E-07	1.39E-06
EXON1	ANKLE1	19	17392656/17392770	2	cg00433770/cg27101125	0.088966223	0.134141224	0.257005048	0.168038825	1.21E-03	0.004910921
EXON1	ANKRD30B	18	14748439/14748285/14748298/14748250	4	cg02992546/cg03014326/cg21281009/cg24061208	0.538124739	0.616041	0.721766368	0.183641629	1.35E-06	1.17E-05
EXON1	ANKRD33B	5	10564690/10564448/10564514	3	cg01259029/cg07682578/cg09877593	0.060307918	0.132939988	0.281293543	0.220985625	2.46E-05	0.000151172
EXON1	ANKRD34C	15	79575485/79575334	2	cg06015523/cg25456849	0.516340225	0.635969644	0.737739055	0.22139883	2.06E-11	1.81E-09
EXON1	ANKRD55	5	55529124/55412572	2	cg11264935/cg22709202	0.610293455	0.5384717	0.450503718	-0.159789737	1.15E-07	1.36E-06
EXON1	ANKS4B	16	21245151	1	cg26937434	0.567352275	0.405970672	0.279589323	-0.287762952	6.89E-11	3.86E-09
EXON1	ANO5	11	22214934/22214726	2	cg19639184/cg20115218	0.162117194	0.239535159	0.344009078	0.181891884	1.53E-03	0.00607095
EXON1	ANP32C	4	165118829/165118368	2	cg16244944/cg19400238	0.713920753	0.617985136	0.563648559	-0.150272193	2.41E-11	1.97E-09
EXON1	ANXA13	8	124749564	1	cg00283535	0.51764548	0.418693367	0.325426782	-0.192218698	5.39E-10	1.61E-08
EXON1	ANXA9	1	150954826/150954554	2	cg13320146/cg20437604	0.51022676	0.410074872	0.3194991	-0.19072766	7.53E-11	4.10E-09
EXON1	AOAH	7	36764019/36764140/36764082	3	cg14451276/cg18652683/cg25733272	0.549081075	0.630627097	0.711896268	0.162815193	1.18E-09	2.95E-08
EXON1	AOX1	2	201450731/201450743	2	cg12627583/cg13875120	0.148069937	0.191967324	0.30264263	0.154572693	4.46E-03	0.015799883
EXON1	APBB1IP	10	26727318	1	cg05475934	0.381048095	0.5482303	0.644688227	0.263640132	1.54E-09	3.65E-08

EXON1	APCS	1	159557632	1	cg03856044	0.480257805	0.425551878	0.307269341	-0.172988464	7.47E-11	4.10E-09
EXON1	APOBEC3A	22	39353650/39353550	2	cg21345826/cg27504369	0.587978545	0.466772394	0.394652645	-0.1933259	1.24E-09	3.06E-08
EXON1	AQP10	1	154293651	1	cg01359534	0.53928651	0.470388844	0.383903682	-0.155382828	1.41E-08	2.30E-07
EXON1	AQP5	12	50355995/50355307/50355821	3	cg04450003/cg15336765/cg23855989	0.482038021	0.539928161	0.664679767	0.182641746	6.85E-11	3.86E-09
EXON1	ARHGAP20	11	110583377	1	cg26578621	0.14361743	0.229177771	0.37131297	0.22769554	4.07E-05	0.000236852
EXON1	ARHGDIB	12	15114494	1	cg10087172	0.274435595	0.179145101	0.097067151	-0.177368444	9.82E-08	1.18E-06
EXON1	ARL14	3	160395073/160395719/160395420	3	cg11896170/cg20725880/cg24147596	0.510067087	0.394944848	0.3051834	-0.204883687	1.32E-12	3.13E-10
EXON1	ARMC3	10	23217017	1	cg15193782	0.125462671	0.184802217	0.35818351	0.232720839	3.57E-05	0.000210463
EXON1	ASB10	7	150884003/150884641/150884848/150884429/150884914	5	cg13289321/cg13594957/cg16537044/cg16564894/cg26159905	0.676565363	0.59461537	0.510523753	-0.16604161	8.31E-11	4.31E-09
EXON1	ASB15	7	123249130	1	cg02300083	0.61231217	0.532766017	0.371533668	-0.240778502	4.45E-07	4.34E-06
EXON1	ASB17	1	76397847	1	cg17041296	0.71839635	0.680643083	0.568250468	-0.150145882	3.08E-04	0.001442789
EXON1	ASB4	7	95115354/95115289	2	cg11554605/cg26847490	0.62529292	0.502638811	0.408500291	-0.216792629	2.51E-10	9.14E-09
EXON1	ASCL1	12	103352235/103352326/103352000/103351987/103352694/103352294/103352454/103352267/103351855	9	cg02246645/cg03700449/cg16712637/cg16921310/cg17015844/cg20718350/cg22356339/cg27420520/cg27569040	0.10422316	0.15695333	0.269524773	0.165301613	2.32E-05	0.000144054
EXON1	ASCL4	12	108169569/108168562/108169928/108168987/108168824/108169020/108170183/108169333	8	cg01295392/cg01952953/cg01985833/cg04290586/cg13721169/cg20443254/cg23699926/cg24856726	0.505613222	0.609480159	0.716396462	0.21078324	1.36E-12	3.13E-10
EXON1	ASTN1	1	177133737/177133946/177133536/177133734/177134019/177133566	6	cg00319545/cg06650419/cg12268575/cg12907702/cg14659404/cg17105834	0.232585843	0.300357935	0.409380638	0.176794795	1.37E-07	1.58E-06
EXON1	ATP10A	15	26108084/26108263	2	cg17793621/cg20174066	0.274127209	0.360125318	0.474313777	0.200186568	5.59E-08	7.23E-07
EXON1	ATP13A4	3	193272561/193272655	2	cg05820087/cg09216823	0.459120505	0.357587378	0.275725277	-0.183395228	3.53E-10	1.17E-08

EXON1	ATP1B2	17	7554827/7554930/7554666/7554357/7554513/7554681	6	cg00053373/cg00996262/cg02705837/cg03554817/cg06200013/cg22448889	0.120678939	0.205364455	0.328424325	0.207745385	2.84E-06	2.23E-05
EXON1	AUTS2	7	69064884/69064868/69063971/69064093/69064801	5	cg00585733/cg08676249/cg13234848/cg17027195/cg21393713	0.191128351	0.340246041	0.468972373	0.277844022	1.46E-06	1.24E-05
EXON1	B4GALT2	1	44446201	1	cg02416986	0.578692005	0.510638594	0.420132314	-0.158559691	2.96E-07	3.07E-06
EXON1	BAI1	8	143545949/143545940/143545478/143545789/143545574/143545390	6	cg00713567/cg02478907/cg03924566/cg09968723/cg14695492/cg21210789	0.33967415	0.404488839	0.493859428	0.154185278	2.33E-06	1.87E-05
EXON1	BAIAP2L2	22	38506623/38506589	2	cg17838773/cg27505627	0.585663558	0.461452011	0.364674816	-0.220988742	5.72E-11	3.47E-09
EXON1	BARHL1	9	135458173/135458625	2	cg12622986/cg14375461	0.290552243	0.358053772	0.48756932	0.197017078	1.39E-08	2.28E-07
EXON1	BARHL2	1	91182534/91182708/91182731/91182288/91182215/91182128/91182777/91182305	8	cg01921432/cg06384463/cg06959142/cg13486820/cg13975503/cg15979173/cg18322569/cg26332310	0.349082339	0.386844012	0.539468922	0.190386583	9.16E-06	6.28E-05
EXON1	BCAT1	12	25102274/25102072	2	cg04011247/cg10764357	0.202790783	0.308302367	0.516316832	0.313526049	1.76E-09	4.04E-08
EXON1	BCL2L15	1	114429965/114430031	2	cg13801402/cg24550026	0.494552815	0.351283019	0.268456568	-0.226096247	3.11E-12	5.21E-10
EXON1	BEND4	4	42154840	1	cg10172669	0.29211493	0.440483528	0.56288045	0.27076552	3.95E-08	5.44E-07
EXON1	BEND5	1	49242359/49242513/49242519	3	cg06744574/cg11666087/cg16573178	0.07530915	0.259731009	0.33691097	0.26160182	5.14E-05	0.000293061
EXON1	BEST2	19	12863557	1	cg19743791	0.656048925	0.50185895	0.384092309	-0.271956616	1.15E-11	1.24E-09
EXON1	BFSP2	3	133118839/133118940	2	cg12354382/cg25249068	0.63191017	0.508993228	0.392938859	-0.238971311	4.83E-12	6.55E-10
EXON1	BIRC7	20	61867349/61867533	2	cg00895528/cg17237881	0.476063275	0.387165683	0.313177091	-0.162886184	1.43E-08	2.32E-07
EXON1	BLK	8	11351571/11351846	2	cg03860768/cg19882315	0.64872187	0.556635978	0.453380141	-0.195341729	1.52E-08	2.43E-07
EXON1	BMP10	2	69098569/69098272	2	cg10976975/cg11308639	0.75313066	0.702807206	0.579605368	-0.173525292	3.46E-05	0.000204814
EXON1	BMP6	6	7727270	1	cg16392727	0.126534887	0.229251004	0.328189528	0.201654641	0.002856053	0.010628053
EXON1	BPIL1	20	31595432/31595509	2	cg08918845/cg08925606	0.5670372	0.468596339	0.350106768	-0.216930432	1.39E-12	3.13E-10
EXON1	BRDT	1	92415048/92414985/92415145	3	cg03136151/cg08172037/cg09250199	0.88405712	0.780984089	0.702533286	-0.181523834	1.60E-05	0.0001042

EXON1	BRUNOL4	18	35145535/35145353/35145983/35145473/35145650	5	cg06761921/cg07300558/cg11154070/cg18397864/cg19492446	0.214005816	0.26576577	0.401787196	0.18778138	2.62E-05	0.000160048
EXON1	BSX	11	122852284	1	cg16167240	0.40348509	0.487954133	0.636420345	0.232935255	4.60E-10	1.41E-08
EXON1	BTNL3	5	180415938	1	cg02720155	0.51763518	0.42233935	0.341113614	-0.176521566	1.48E-07	1.69E-06
EXON1	BTNL8	5	180326186/180326142	2	cg03723845/cg24024214	0.531435345	0.419498567	0.334261309	-0.197174036	2.30E-11	1.92E-09
EXON1	BVES	6	105584149/105584216	2	cg20624391/cg25280433	0.19679352	0.254422961	0.366656853	0.169863333	0.000714712	0.003062822
EXON1	C10orf90	10	128209980	1	cg24143287	0.67340542	0.465538717	0.322228568	-0.351176852	1.91E-12	3.84E-10
EXON1	C10orf93	10	134755955	1	cg23962250	0.26105547	0.337518594	0.460069805	0.199014335	0.000211436	0.001036725
EXON1	C10orf96	10	118084109	1	cg24407204	0.66575674	0.546362117	0.49450745	-0.17124929	1.43E-06	1.22E-05
EXON1	C10orf99	10	85933586	1	cg10737718	0.41991726	0.323038672	0.257776177	-0.162141083	4.24E-11	2.84E-09
EXON1	C11orf41	11	33563946	1	cg22937632	0.678477485	0.593174794	0.471460355	-0.20701713	1.13E-08	1.90E-07
EXON1	C11orf53	11	111126790	1	cg19964761	0.807520505	0.7183644	0.614021768	-0.193498737	1.20E-05	8.00E-05
EXON1	C11orf87	11	109292894	1	cg06719900	0.405233015	0.474809478	0.586746568	0.181513553	2.75E-08	4.04E-07
EXON1	C11orf94	11	45928805	1	cg04310460	0.54808115	0.430428489	0.356957232	-0.191123918	5.65E-11	3.45E-09
EXON1	C12orf56	12	64784177/64784173/64784252	3	cg04162059/cg13446772/cg19998368	0.313611043	0.367380625	0.513623067	0.200012023	2.80E-08	4.10E-07
EXON1	C12orf59	12	10331614/10331666	2	cg25494227/cg26718420	0.6664606	0.575493056	0.485267255	-0.181193345	4.23E-07	4.16E-06
EXON1	C12orf74	12	93096844	1	cg25382573	0.444039435	0.343317206	0.267986741	-0.176052694	9.39E-10	2.49E-08
EXON1	C13orf38	13	36871878/36871962/36871948/36871943	4	cg04902286/cg13258563/cg15167956/cg26439710	0.186418729	0.267530791	0.371579939	0.18516121	2.38E-05	0.000147161
EXON1	C16orf73	16	1922149/1922155	2	cg05041351/cg07775371	0.155952418	0.191239859	0.348454698	0.19250228	5.85E-06	4.21E-05
EXON1	C17orf104	17	42733994/42733976	2	cg21095561/cg22190438	0.268131321	0.326896583	0.53463631	0.266504989	2.84E-07	2.96E-06
EXON1	C17orf105	17	41857926	1	cg18515343	0.733378725	0.646996922	0.527324627	-0.206054098	7.69E-09	1.39E-07
EXON1	C17orf46	17	43339476	1	cg17222164	0.110370772	0.218942219	0.334238974	0.223868202	0.000188332	0.000934525
EXON1	C17orf47	17	56621448/56621578	2	cg11890622/cg26956135	0.637106035	0.543989381	0.434952645	-0.20215339	8.65E-10	2.34E-08
EXON1	C19orf21	19	751159	1	cg14052044	0.585568995	0.485448128	0.386443682	-0.199125313	4.48E-10	1.38E-08
EXON1	C19orf33	19	38794845/38794814	2	cg00412772/cg11272232	0.434242788	0.347337644	0.281336405	-0.152906383	8.75E-10	2.37E-08
EXON1	C19orf75	19	51761116	1	cg21930712	0.50822113	0.406950344	0.293409632	-0.214811498	2.13E-11	1.85E-09
EXON1	C1orf114	1	169396635/169396637	2	cg00100121/cg13958426	0.12173728	0.250425353	0.371139035	0.249401754	2.96E-05	0.000178824
EXON1	C1orf173	1	75139347/75139364	2	cg24155871/cg27393126	0.295957565	0.367192178	0.573761716	0.277804151	1.35E-07	1.56E-06
EXON1	C1orf190	1	46669221	1	cg15696662	0.304778516	0.346362061	0.460521201	0.155742685	0.004869277	0.017072798

EXON1	C1orf70	1	1475675/1475737	2	cg16306898/cg16601494	0.134715278	0.32452273	0.606946738	0.472231459	6.77E-11	3.86E-09
EXON1	C1orf94	1	34642862/34642899/34632658/34642745/34642609/34643106/34642605	7	cg04025150/cg04323427/cg09270225/cg13329248/cg16348470/cg18328755/cg22697574	0.375496506	0.460387739	0.595330099	0.219833593	2.03E-10	7.89E-09
EXON1	C1QC	1	22970132	1	cg17104151	0.46445252	0.340469811	0.226817273	-0.237635247	5.28E-13	1.76E-10
EXON1	C1QL1	17	43045368/43045014/43045372/43045386/43045068	5	cg03385864/cg04819499/cg13818573/cg15891422/cg19226007	0.184384351	0.250416889	0.353737757	0.169353406	2.05E-05	0.000129091
EXON1	C1QL2	2	119916017/119915729/119915368/119915783/119916431	5	cg00690148/cg10996058/cg15089219/cg20470857/cg26530498	0.339145462	0.416204746	0.524014235	0.184868773	3.99E-10	1.28E-08
EXON1	C1QL3	10	16563821/16562917/16563586/16562626/16562998	5	cg10982433/cg11880855/cg15345154/cg24037897/cg25104555	0.106647108	0.180994227	0.363638429	0.256991321	6.55E-06	4.66E-05
EXON1	C1QTNF5	11	119217331	1	cg09186818	0.48301678	0.414818267	0.315081205	-0.167935575	1.64E-09	3.80E-08
EXON1	C20orf103	20	9495495/9495326/9495404	3	cg12650655/cg20191310/cg26582643	0.155063925	0.172190491	0.307051689	0.151987763	0.005606782	0.019401547
EXON1	C20orf186	20	31669392	1	cg04087207	0.476719275	0.355235783	0.264749995	-0.21196928	1.47E-11	1.45E-09
EXON1	C20orf200	20	61148684	1	cg00499838	0.182461058	0.260689047	0.378726132	0.196265074	1.15E-05	7.72E-05
EXON1	C20orf71	20	31805291/31805182	2	cg09742177/cg15131258	0.67492102	0.584522178	0.477997677	-0.196923343	2.99E-05	0.000180139
EXON1	C2orf74	2	61372316/61372256	2	cg10580144/cg18158151	0.310297213	0.508793317	0.667898914	0.357601701	4.61E-12	6.53E-10
EXON1	C2orf90	2	903676	1	cg09613130	0.83853972	0.775605406	0.631398655	-0.207141065	1.39E-05	9.14E-05
EXON1	C3AR1	12	8218922	1	cg09238677	0.39392092	0.260403385	0.177022935	-0.216897985	4.71E-08	6.24E-07
EXON1	C3orf22	3	126277693	1	cg24389347	0.61844124	0.53159915	0.418475436	-0.199965804	9.89E-09	1.71E-07
EXON1	C4BPB	1	207262706	1	cg26407558	0.34993362	0.263663656	0.197965268	-0.151968352	1.57E-13	7.71E-11
EXON1	C4orf39	4	165878742/165878219/165878136/165878317	4	cg03508095/cg08992305/cg11630554/cg13684141	0.110093365	0.199651663	0.266455738	0.156362372	0.001948892	0.007573998
EXON1	C4orf49	4	140201433/140201262	2	cg18065318/cg20710842	0.1592298	0.219469219	0.337969581	0.178739781	3.57E-05	0.00021047
EXON1	C6orf105	6	11778902/11779039	2	cg14178895/cg26761744	0.471314278	0.342208347	0.284761011	-0.186553266	2.10E-10	8.12E-09
EXON1	C7orf52	7	100823377/100823400	2	cg14562712/cg19828791	0.218592698	0.287711292	0.3996654	0.181072703	0.000297605	0.001400077
EXON1	C7orf72	7	50136102	1	cg09857577	0.506747495	0.374728667	0.237272214	-0.269475281	1.51E-12	3.13E-10

EXON1	C8orf34	8	69351585/69350927	2	cg00280504/cg22199118	0.331943675	0.235495517	0.18020561	-0.151738065	3.21E-11	2.36E-09
EXON1	C8orf46	8	67405742	1	cg23704362	0.666288825	0.594698256	0.515657332	-0.150631493	1.50E-05	9.80E-05
EXON1	C8orf85	8	117950504/117950473	2	cg04271218/cg05996052	0.378524738	0.489477106	0.632894352	0.254369615	7.95E-13	2.34E-10
EXON1	C9orf129	9	96108467	1	cg10065823	0.36883572	0.460165744	0.576210927	0.207375207	7.11E-07	6.58E-06
EXON1	C9orf144B	9	34729282/34729355	2	cg09187598/cg14598211	0.819016255	0.76346715	0.643566423	-0.175449832	2.19E-06	1.77E-05
EXON1	C9orf152	9	112970404	1	cg09596614	0.476353965	0.383152094	0.307880405	-0.16847356	3.32E-10	1.12E-08
EXON1	C9orf47	9	91605893/91605999	2	cg12972233/cg20083676	0.437742505	0.562415053	0.589325675	0.15158317	4.62E-06	3.43E-05
EXON1	C9orf50	9	132383003/132382812/132382433	3	cg09731694/cg13405887/cg14015706	0.231429344	0.52166887	0.667683026	0.436253682	4.86E-16	6.43E-12
EXON1	CA10	17	50236087/50235328/50235737/50235393/50236765/50235965/50235279/50237315	8	cg08034077/cg14054928/cg14073722/cg20405017/cg21328033/cg22702328/cg24341611/cg25592977	0.427952226	0.501691851	0.638853658	0.210901432	5.56E-10	1.65E-08
EXON1	CA9	9	35674000	1	cg20610181	0.560768595	0.46196675	0.385375305	-0.17539329	1.71E-07	1.91E-06
EXON1	CACNA1A	19	13617012/13617094/13616871/13617091	4	cg17509220/cg17509967/cg22187630/cg22491927	0.31954001	0.413770184	0.546276039	0.226736029	6.72E-07	6.25E-06
EXON1	CACNG3	16	24267226/24267399/24267559/24267526/24266905	5	cg02692912/cg04441477/cg04543289/cg07514158/cg17234150	0.338346394	0.442153518	0.596171655	0.257825261	2.71E-09	5.69E-08
EXON1	CACNG8	19	54466297/54466538/54466441	3	cg08653692/cg22049569/cg24415208	0.344097342	0.443230114	0.651466188	0.307368846	3.14E-10	1.08E-08
EXON1	CADM2	3	85008678/85008587/85008786/85008156	4	cg05152589/cg13331200/cg22380921/cg26113636	0.306559718	0.336317308	0.462555287	0.155995569	1.36E-06	1.18E-05
EXON1	CALCB	11	15095178	1	cg23558842	0.303237325	0.309508378	0.475860182	0.172622857	0.003155125	0.011636146
EXON1	CALML4	15	68498251/68497992	2	cg05338167/cg11530995	0.45808637	0.373735086	0.304724143	-0.153362227	6.76E-11	3.86E-09
EXON1	CALN1	7	71801793/71877113/71802184/71877155/71877237	5	cg05223720/cg10928348/cg17239236/cg17932934/cg21003606	0.31873279	0.456246006	0.562331473	0.243598683	2.04E-09	4.54E-08
EXON1	CAPN8	1	223853145	1	cg19249749	0.867833045	0.762871883	0.662754586	-0.205078459	5.48E-05	0.000310349
EXON1	CARD11	7	3083541/3083333/3083338	3	cg14549249/cg16842053/cg26937500	0.125202067	0.265370706	0.413698738	0.288496671	4.40E-06	3.28E-05

EXON1	CARD6	5	40841590/40841579/40841493	3	cg05981038/cg16250754/cg26818786	0.485350275	0.404721856	0.264265357	-0.221084918	1.37E-06	1.18E-05
EXON1	CASS4	20	54987330	1	cg10463299	0.537502275	0.622229189	0.705192095	0.16768982	2.64E-07	2.76E-06
EXON1	CBLN2	18	70211515	1	cg21902544	0.273541708	0.40733855	0.624527209	0.350985502	2.53E-09	5.39E-08
EXON1	CBLN4	20	54579084/54579355/54579814	3	cg01729827/cg02501779/cg14184078	0.484643977	0.508003546	0.681354433	0.196710457	9.32E-10	2.48E-08
EXON1	CCDC105	19	15122142/15121591/15122135/15121596/15122181/15121913	6	cg02241330/cg03928546/cg04768697/cg06522681/cg14884776/cg18721420	0.373889938	0.45019532	0.586352816	0.212462878	4.27E-10	1.34E-08
EXON1	CCDC114	19	48823178	1	cg25533551	0.57711474	0.477702278	0.392349423	-0.184765317	9.75E-11	4.75E-09
EXON1	CCDC140	2	223162881/223163033/223162875	3	cg06916239/cg23546474/cg25596297	0.422570955	0.382108772	0.580172291	0.157601336	0.006122858	0.020962548
EXON1	CCDC36	3	49236946	1	cg03540175	0.61096671	0.660020428	0.798652814	0.187686104	9.92E-10	2.58E-08
EXON1	CCDC37	3	126113784	1	cg00891278	0.319879045	0.361500828	0.544311159	0.224432114	4.67E-06	3.46E-05
EXON1	CCDC42	17	8647944/8648035	2	cg06468695/cg10057436	0.7384243	0.602032211	0.476622014	-0.261802286	5.68E-12	7.30E-10
EXON1	CCDC54	3	107097413	1	cg15507901	0.576766305	0.438565783	0.374125105	-0.2026412	1.27E-07	1.47E-06
EXON1	CCKBR	11	6281180/6280988/6281197/6281235/6281104	5	cg01487187/cg06646346/cg20209956/cg21957616/cg26686277	0.111458803	0.146587496	0.286460796	0.175001993	0.000290053	0.001369908
EXON1	CCL1	17	32690127	1	cg20556988	0.7418272	0.594454389	0.491574041	-0.250253159	3.51E-08	4.94E-07
EXON1	CCL20	2	228678693	1	cg21643045	0.589008875	0.466751633	0.372675441	-0.216333434	1.07E-11	1.20E-09
EXON1	CCNA1	13	37006063/37006127/37006265/37006748/37006611/37006734/37006107/37006116/37006133	9	cg02478448/cg05137358/cg10158541/cg12571423/cg13060997/cg16422907/cg18348647/cg18948722/cg26984343	0.423211149	0.540206756	0.67690676	0.253695611	9.77E-13	2.68E-10
EXON1	CCT8L2	22	17073594	1	cg07443748	0.56714542	0.458567933	0.362340368	-0.204805052	3.14E-11	2.33E-09
EXON1	CD226	18	67624071	1	cg13164537	0.54714104	0.614850244	0.710015336	0.162874296	3.58E-10	1.17E-08
EXON1	CD34	1	208084456/208084415/208084640/208084436	4	cg04292718/cg16115720/cg19591881/cg21455438	0.238943501	0.37336436	0.543432812	0.304489311	2.42E-09	5.20E-08
EXON1	CD38	4	15779999/15780011/15780238	3	cg02183671/cg24819835/cg26043257	0.21345019	0.439392783	0.552727605	0.339277415	6.81E-09	1.26E-07
EXON1	CD40	20	44746944/44747006	2	cg01943874/cg21601405	0.20207869	0.279558925	0.410242973	0.208164283	2.28E-06	1.83E-05

EXON1	CDH10	5	24644878	1	cg00068408	0.188328965	0.251849002	0.367313545	0.17898458	0.000262701	0.001253706
EXON1	CDH12	5	22853730/22853703/22853464	3	cg10303842/cg18394953/cg26227411	0.289133825	0.333335681	0.461759328	0.172625503	0.000135084	0.000692108
EXON1	CDH13	16	82660630/82660727/82660596/82660670	4	cg01301138/cg05374412/cg05949171/cg08747377	0.213631258	0.243126457	0.375924352	0.162293094	0.001413874	0.005667923
EXON1	CDH18	5	19988200/19988288	2	cg06076122/cg06781712	0.272073128	0.394306767	0.572143656	0.300070528	1.14E-07	1.35E-06
EXON1	CDH2	18	25757211/25757438/25757202	3	cg09313439/cg12208258/cg13464915	0.291554003	0.338988742	0.495758347	0.204204344	1.28E-05	8.50E-05
EXON1	CDH8	16	62070005	1	cg09515947	0.33996726	0.47995985	0.651866577	0.311899317	4.34E-11	2.88E-09
EXON1	CDO1	5	115152019/115152386/115152326	3	cg07405021/cg12880658/cg16265906	0.245180578	0.397157344	0.52278282	0.277602243	6.74E-08	8.51E-07
EXON1	CHAT	10	50822175/50817213/50821161/50822158/50822391	5	cg00586644/cg06108782/cg11811216/cg20405893/cg26473651	0.22912098	0.286928603	0.436374917	0.207253937	3.90E-07	3.88E-06
EXON1	CHODL	21	19617439	1	cg21350575	0.49047568	0.565352933	0.691209159	0.200733479	2.36E-11	1.95E-09
EXON1	CHRM1	11	62688751/62688748	2	cg00987015/cg03360907	0.522923135	0.447229633	0.363043625	-0.15987951	6.11E-10	1.76E-08
EXON1	CHRM2	7	136553595/136553882/136553855/136554160/136553682/136553868/136553884/136553728	8	cg00973677/cg07664198/cg08323651/cg20847733/cg22471517/cg24228819/cg24575234/cg25632105	0.330892161	0.431609421	0.583658591	0.25276643	2.42E-12	4.39E-10
EXON1	CHST2	3	142838847/142838938/142839022/142838682	4	cg00995327/cg08858437/cg09638407/cg17815252	0.088903637	0.191432253	0.350789196	0.261885559	2.35E-06	1.89E-05
EXON1	CIDEA	18	12254690/12254365/12254377	3	cg00590620/cg15063116/cg20950011	0.29549468	0.381982467	0.449232986	0.153738306	6.59E-05	0.000365133
EXON1	CLDN5	22	19511707/19512712/19512084/19511987/19512320/19510877/19511967/19512228/19510977	9	cg00811132/cg04463638/cg06315607/cg09092054/cg09446908/cg17411190/cg17577122/cg20486569/cg21872764	0.223979252	0.274254651	0.411113158	0.187133906	1.15E-07	1.36E-06

EXON1	CLEC14A	14	38725536/38724675/38724255/38725312/38723500/38724648/38724945	7	cg04681600/cg05057720/cg06284231/cg08139247/cg16125874/cg16404157/cg23288059	0.496551778	0.5404342	0.649950598	0.15339882	1.56E-07	1.76E-06
EXON1	CLEC2L	7	139208852	1	cg08832906	0.360356285	0.359281556	0.511563559	0.151207274	0.002696784	0.010106448
EXON1	CLEC4G	19	7797031/7797052	2	cg16626670/cg19001261	0.557977435	0.466949472	0.397688936	-0.160288499	1.98E-10	7.76E-09
EXON1	CLEC4M	19	7828144	1	cg21372914	0.669008555	0.59473485	0.472102182	-0.196906373	7.13E-07	6.59E-06
EXON1	CLEC7A	12	10282621	1	cg26066361	0.587201725	0.487356139	0.373359232	-0.213842493	4.60E-06	3.42E-05
EXON1	CLIC6	21	36042752/36042344/36042224/36041699/36042464/36042170	6	cg02488385/cg04125350/cg04497512/cg10722799/cg12742338/cg17935217	0.476268398	0.52168884	0.654676344	0.178407947	0.000176199	0.000880597
EXON1	CLIP4	2	29338432	1	cg08808128	0.152998709	0.321004766	0.562527791	0.409529082	1.97E-11	1.77E-09
EXON1	CLLU1	12	92818240	1	cg13003571	0.857301425	0.694630783	0.60484675	-0.252454675	1.50E-09	3.56E-08
EXON1	CLPS	6	35765093	1	cg01670608	0.617996745	0.516676956	0.4087381	-0.209258645	3.20E-10	1.09E-08
EXON1	CLRN3	10	129690850	1	cg20424781	0.589042825	0.441748633	0.349449841	-0.239592984	3.54E-11	2.52E-09
EXON1	CLVS2	6	123317714/123317875	2	cg18210226/cg24728018	0.21959894	0.268317521	0.382576382	0.162977442	0.000169222	0.000849816
EXON1	CMTM3	16	66638320/66638599/66638702/66638407/66638396/66638438/66638412/66638293/66638433/66638210	10	cg00355909/cg03860859/cg06314872/cg06445928/cg08601917/cg08682544/cg09233013/cg23297477/cg26560414/cg26658728	0.110735955	0.28202328	0.429371915	0.31863596	1.64E-08	2.57E-07
EXON1	CNGA3	2	98962900/98962974	2	cg18404308/cg19671120	0.331170344	0.394459683	0.577932857	0.246762513	5.73E-09	1.09E-07
EXON1	CNGB3	8	87755844	1	cg21221571	0.71771295	0.659968667	0.5530391	-0.16467385	7.15E-05	0.00039234
EXON1	CNRIP1	2	68546899/68546688/68546507/68547088/68547141/68546467/68547052/68546579	8	cg00020052/cg04020079/cg07080358/cg07594636/cg08157672/cg11573679/cg15313459/cg24171907	0.217559235	0.445870725	0.58209091	0.364531675	8.42E-10	2.32E-08
EXON1	CNTN1	12	41086365	1	cg08990926	0.200349787	0.302127793	0.453710018	0.253360231	3.30E-06	2.55E-05
EXON1	CNTNAP2	7	145814003/145813915/145813946/145813494	4	cg05422683/cg07612562/cg16521917/cg24887139	0.255183548	0.334700622	0.482120427	0.22693688	1.90E-09	4.29E-08
EXON1	CNTNAP4	16	76311266	1	cg26577454	0.127225389	0.143523389	0.282300908	0.15507552	0.007209683	0.024281287

EXON1	CNTNAP5	2	124783243/124782885/124783254	3	cg01308258/cg11344566/cg16198087	0.349953968	0.431409758	0.560647352	0.210693385	1.25E-08	2.07E-07
EXON1	COL22A1	8	139926188	1	cg12304113	0.17062913	0.248627404	0.343433726	0.172804596	8.91E-06	6.15E-05
EXON1	COL23A1	5	178017376/178017260/178017300	3	cg20546002/cg22960186/cg25552435	0.102119519	0.195361626	0.290433439	0.18831392	0.000642465	0.002790271
EXON1	COL24A1	1	86622113	1	cg12644264	0.141704516	0.22757463	0.38242285	0.240718334	4.56E-05	0.000262689
EXON1	COL25A1	4	110223646/110223713/110223700/110223795/110223429/110223598	6	cg02441747/cg06468908/cg10074544/cg11935021/cg19039028/cg25088758	0.256102573	0.356471273	0.53354126	0.277438687	3.02E-08	4.35E-07
EXON1	COL5A1	9	137533851/137533949	2	cg14252519/cg26024843	0.33844217	0.423000736	0.548963739	0.210521569	8.50E-08	1.05E-06
EXON1	COL8A2	1	36565739/36565772	2	cg15818357/cg18931815	0.555285925	0.438568967	0.331806073	-0.223479852	1.40E-12	3.13E-10
EXON1	CPA4	7	129933014	1	cg01796223	0.58549644	0.538058156	0.374964577	-0.210531863	1.29E-06	1.12E-05
EXON1	CPB2	13	46679081	1	cg14662172	0.886822105	0.825670706	0.627236732	-0.259585373	2.19E-06	1.77E-05
EXON1	CPNE8	12	39299364/39299326	2	cg01963134/cg13603508	0.245409402	0.243125381	0.406029529	0.160620127	0.00743476	0.024944063
EXON1	CPXM1	20	2781262/2781122/2781241	3	cg07113642/cg15547669/cg22304612	0.261711148	0.28907327	0.448230117	0.186518969	0.000704961	0.003024954
EXON1	CR1	1	207669716/207669576/207669544	3	cg02588107/cg05825950/cg18147366	0.083524104	0.161724098	0.2775816	0.194057496	2.55E-06	2.02E-05
EXON1	CR1L	1	207818493	1	cg24557058	0.25046316	0.380392383	0.543904982	0.293441822	1.09E-07	1.30E-06
EXON1	CREB5	7	28338985/28452289/28475265	3	cg02611282/cg16534307/cg20306842	0.534178985	0.427034094	0.331157832	-0.203021153	2.73E-10	9.80E-09
EXON1	CRH	8	67090581	1	cg17305181	0.42448583	0.523840244	0.664674323	0.240188493	1.15E-10	5.33E-09
EXON1	CRHR2	7	30721888/30721797/30722114	3	cg04922810/cg04923928/cg24610236	0.197184223	0.25295113	0.364745407	0.167561184	7.36E-06	5.17E-05
EXON1	CRISPLD1	8	75896975/75897115	2	cg01410472/cg02942845	0.29672969	0.373407267	0.516262164	0.219532474	5.66E-09	1.08E-07
EXON1	CRYGD	2	208989209/208989248	2	cg17916432/cg22399133	0.566959963	0.630215325	0.747540161	0.180580199	1.86E-08	2.86E-07
EXON1	CSF2	5	131409637/131409634	2	cg02196805/cg08686879	0.686377968	0.581226558	0.467545507	-0.218832461	2.59E-09	5.50E-08
EXON1	CSGALNACT1	8	19459672/19459996/19539991	3	cg15232539/cg17470251/cg23328404	0.373529295	0.285281789	0.213618641	-0.159910654	2.35E-07	2.51E-06
EXON1	CSMD1	8	4852036	1	cg12258042	0.56875419	0.654968889	0.762469864	0.193715674	1.56E-09	3.67E-08
EXON1	CSN2	4	70826695	1	cg21945989	0.64838953	0.593371167	0.49804175	-0.15034778	0.001902626	0.00740943
EXON1	CST5	20	23860242	1	cg01454519	0.74384544	0.624202256	0.553199659	-0.190645781	1.95E-06	1.60E-05
EXON1	CTNNA3	10	69455804	1	cg19996026	0.509914225	0.4564797	0.297704323	-0.212209902	4.91E-07	4.72E-06

EXON1	CUX2	12	111471963	1	cg09229912	0.293054925	0.402388711	0.519260705	0.22620578	2.99E-08	4.32E-07
EXON1	CXCR1	2	219031640	1	cg20025658	0.438184495	0.294536117	0.207657482	-0.230527013	3.43E-12	5.46E-10
EXON1	CYP26C1	10	94821085	1	cg20322977	0.505071995	0.608325311	0.722379009	0.217307014	9.80E-11	4.75E-09
EXON1	CYP2B6	19	41497222	1	cg19756068	0.47676136	0.393884778	0.323333391	-0.153427969	9.62E-11	4.71E-09
EXON1	CYP2C18	10	96443383	1	cg14196507	0.65630379	0.492147161	0.376205891	-0.280097899	5.45E-11	3.35E-09
EXON1	CYP3A5	7	99277496	1	cg15257783	0.496916545	0.41772885	0.330803141	-0.166113404	1.72E-10	7.06E-09
EXON1	CYYR1	21	27945413	1	cg19678828	0.535953395	0.613151833	0.695989627	0.160036232	1.28E-10	5.73E-09
EXON1	D4S234E	4	4388505	1	cg13432708	0.152787557	0.195513763	0.351984998	0.199197441	0.00167928	0.006613632
EXON1	DAB1	1	58716018/58716033	2	cg00848728/cg24351410	0.21101627	0.301038222	0.424539043	0.213522773	1.02E-06	9.12E-06
EXON1	DAO	12	109273999	1	cg12592321	0.561895295	0.470840789	0.365507909	-0.196387386	4.90E-10	1.49E-08
EXON1	DBC1	9	122131497	1	cg06602847	0.41118854	0.459553056	0.602685645	0.191497105	4.05E-05	0.000235707
EXON1	DBX1	11	20181725	1	cg13445796	0.474141205	0.593457472	0.754344464	0.280203259	7.58E-10	2.12E-08
EXON1	DBX2	12	45444420/45444767/45444623	3	cg02332982/cg07469402/cg19788831	0.371399733	0.478532342	0.592899759	0.221500027	1.07E-08	1.83E-07
EXON1	DCC	18	49867117/49866548/49867126	3	cg06261937/cg21669679/cg25266232	0.332915208	0.41640653	0.524336224	0.191421016	5.94E-07	5.60E-06
EXON1	DCLK1	13	36705446	1	cg20219457	0.321214625	0.385892277	0.57984025	0.258625625	5.24E-06	3.83E-05
EXON1	DCT	13	95131809	1	cg05807291	0.63958114	0.60577005	0.481196077	-0.158385063	3.11E-05	0.000186313
EXON1	DDX25	11	125774447/125774406/125774311	3	cg11413039/cg19792599/cg22097425	0.448469455	0.519013998	0.623313264	0.174843809	3.77E-10	1.21E-08
EXON1	DEFB125	20	68396	1	cg01958189	0.495010375	0.382493333	0.299602441	-0.195407934	2.40E-06	1.92E-05
EXON1	DEFB132	20	238452	1	cg00545462	0.47173859	0.374398744	0.261537495	-0.210201095	4.03E-13	1.48E-10
EXON1	DEFB135	8	11839844	1	cg22743884	0.656798795	0.489811556	0.313000655	-0.34379814	7.45E-14	5.19E-11
EXON1	DFNA5	7	24797363/24797486	2	cg04770504/cg24805239	0.13050853	0.192500801	0.322753753	0.192245224	0.000123079	0.000637264
EXON1	DISP1	1	223101848/223101782	2	cg10942056/cg13952483	0.58366515	0.478180539	0.432368886	-0.151296264	5.76E-06	4.15E-05
EXON1	DKK2	4	107956556/107957265/107957362/107956955/107957430/107957072	6	cg00594011/cg01471384/cg01962428/cg08341316/cg13139972/cg26955835	0.202380163	0.362494168	0.489249718	0.286869556	6.91E-09	1.27E-07
EXON1	DLEU7	13	51417686/51417469/51417846	3	cg06679720/cg17241776/cg20170533	0.42299608	0.534649796	0.6745798	0.25158372	1.18E-11	1.25E-09
EXON1	DLK1	14	101193397/101193432	2	cg02874376/cg21575295	0.365188708	0.426066808	0.524482223	0.159293515	5.18E-07	4.94E-06
EXON1	DMRT1	9	841850	1	cg03168582	0.093905036	0.264365498	0.371218771	0.277313735	0.000187585	0.000932219
EXON1	DNAH11	7	21582834	1	cg05285902	0.182962681	0.209432983	0.381891586	0.198928906	0.000344154	0.00159524

EXON1	DNAJA4	15	78556512/78556534/78557094/78556502	4	cg08594606/cg09214398/cg13747794/cg23366752	0.6657829	0.575533097	0.497727018	-0.168055882	2.43E-10	8.98E-09
EXON1	DNM3	1	171810910/171810778	2	cg05377226/cg06211893	0.092526396	0.164498276	0.24640649	0.153880094	0.011113431	0.035537727
EXON1	DNMT3L	21	45681834	1	cg15635600	0.53082813	0.4463379	0.377105464	-0.153722666	1.90E-09	4.29E-08
EXON1	DOK5	20	53092474/53092334	2	cg00055557/cg20107395	0.245197855	0.316964298	0.471235526	0.226037671	6.40E-06	4.56E-05
EXON1	DOK6	18	67068457	1	cg15916399	0.281403135	0.369422233	0.461386509	0.179983374	3.06E-12	5.19E-10
EXON1	DPP6	7	153749964/153584582/154002372/153584597/154002618/153584748/153584609	7	cg02855576/cg06495961/cg13544851/cg14523847/cg21389309/cg24011260/cg27032232	0.469985229	0.554915753	0.688593164	0.218607935	3.04E-10	1.06E-08
EXON1	DPPA2	3	109035305	1	cg15286037	0.631205185	0.5388412	0.435731673	-0.195473512	1.06E-08	1.82E-07
EXON1	DPYS	8	105479248/105479058/105479215	3	cg05736768/cg10303487/cg17087640	0.35026123	0.397745774	0.506450508	0.156189278	1.89E-05	0.000120632
EXON1	DQX1	2	74753358/74753281/74753334/74753312	4	cg01410453/cg02034222/cg06151718/cg24544105	0.643364703	0.502117763	0.42691287	-0.216451834	1.56E-09	3.67E-08
EXON1	DRD4	11	637491	1	cg09607276	0.33616394	0.423128867	0.532067159	0.195903219	2.30E-06	1.85E-05
EXON1	DSCAM	21	42218932/42218551/42218964	3	cg09880551/cg10644072/cg21173447	0.275889612	0.313908063	0.431539523	0.155649911	0.00019725	0.000976214
EXON1	DYDC2	10	82116571	1	cg01527459	0.2575856	0.335183755	0.495825223	0.238239623	0.000142361	0.000725425
EXON1	DZIP1	13	96296844/96296708	2	cg00756058/cg08386091	0.092726217	0.112865545	0.25564515	0.162918934	0.003179271	0.011718745
EXON1	EBF1	5	158526693/158526614/158526642	3	cg04217450/cg05530568/cg17009297	0.283888828	0.337726565	0.504129801	0.220240973	3.94E-06	2.98E-05
EXON1	EBF2	8	25902284	1	cg22841810	0.22877555	0.283158901	0.411130284	0.182354734	0.002885229	0.010724554
EXON1	EDNRA	4	148402408/148402128/148402399	3	cg04045079/cg12009023/cg20557687	0.32039387	0.400135897	0.534961491	0.214567621	1.85E-06	1.53E-05
EXON1	EFCAB1	8	49647734/49647809/49647703/49647717/49647833	5	cg08258526/cg11469098/cg15397374/cg22836229/cg23562261	0.369355676	0.425960703	0.55363708	0.184281404	6.40E-06	4.56E-05
EXON1	EFCAB3	17	60447682	1	cg17876595	0.77645862	0.605235417	0.423534982	-0.352923638	1.13E-11	1.24E-09
EXON1	EFCAB9	5	171621251	1	cg14069601	0.740556155	0.6604426	0.560342682	-0.180213473	4.01E-07	3.97E-06
EXON1	EFEMP1	2	56150341/56150925/56150255	3	cg03122624/cg08130988/cg20786074	0.30868343	0.372410063	0.482420894	0.173737464	7.84E-06	5.47E-05
EXON1	EFHA2	8	16885000	1	cg12097222	0.533506075	0.625467383	0.735539968	0.202033893	3.36E-13	1.31E-10

EXON1	EGFL7	9	139557460	1	cg17443080	0.562301065	0.448419544	0.348661809	-0.213639256	6.72E-13	2.12E-10
EXON1	EHD3	2	31457287/31457608/31457061/31456966/31457058/31456964/31457043	7	cg01163837/cg08251399/cg13795465/cg15355118/cg24743639/cg25428398/cg25840208	0.301393544	0.441993062	0.52556991	0.224176366	1.34E-07	1.55E-06
EXON1	EID3	12	104697620/104697983/104697631	3	cg00588621/cg01848457/cg05057777	0.58006345	0.645740822	0.735975395	0.155911945	5.02E-11	3.15E-09
EXON1	ELF3	1	201979938/201979846/201979764	3	cg02076020/cg07897871/cg26328757	0.58622302	0.48765833	0.401774527	-0.184448493	1.19E-10	5.44E-09
EXON1	ELOVL2	6	11044558/11044541	2	cg05446010/cg22143569	0.028742374	0.092247237	0.198802878	0.170060504	7.53E-05	0.000410365
EXON1	ELOVL4	6	80656973/80657193	2	cg02169734/cg21992350	0.0539617	0.107211908	0.257500826	0.203539126	0.000204501	0.001006148
EXON1	EMB	5	49737178/49737182/49736988	3	cg03179043/cg08722065/cg18110165	0.230660551	0.447511756	0.520510282	0.289849731	2.05E-12	4.05E-10
EXON1	ENPP3	6	131958550	1	cg25461865	0.515706525	0.403366617	0.334653768	-0.181052757	5.68E-10	1.67E-08
EXON1	ENPP6	4	185139078	1	cg08493463	0.55805469	0.491997322	0.384458145	-0.173596545	1.94E-11	1.75E-09
EXON1	EPHA10	1	38230769/38230779	2	cg12128017/cg20510207	0.302970382	0.384643194	0.540645805	0.237675423	1.60E-06	1.35E-05
EXON1	EPHA5	4	66535575/66535383/66535403/66535412/66535351	5	cg02463418/cg09009536/cg12682684/cg14810013/cg25798792	0.38301656	0.469309788	0.564483564	0.181467004	1.01E-06	8.98E-06
EXON1	EPHA6	3	96533699/96533520/96533511/96533530/96533824	5	cg05827732/cg09301294/cg11410023/cg15405439/cg26364205	0.30481357	0.351154689	0.518230145	0.213416575	2.50E-05	0.000153618
EXON1	EPS8L3	1	110306507	1	cg00515905	0.58956258	0.476793306	0.375044255	-0.214518325	4.24E-11	2.84E-09
EXON1	ERBB4	2	213403212/213403321	2	cg07015629/cg19079194	0.22029798	0.223880067	0.386143727	0.165845747	5.79E-05	0.000324929
EXON1	ESR1	6	152129388/152011656/152128535/152129036/152128515/152128634/152128865/152126938/152011666/152128471/152129400/152126895/152128483/152128584/152128588/152128537/152128528	17	cg00920970/cg04211581/cg05171584/cg11251858/cg11813455/cg13612689/cg15980539/cg17264271/cg18007957/cg19411146/cg20253551/cg21157690/cg21950534/cg22839866/cg23009221/cg23467008/cg24900983	0.310897523	0.382499544	0.479582414	0.16868489	2.04E-07	2.22E-06

EXON1	EVC	4	5713100/5712937/5713005	3	cg16418810/cg17460447/cg21210758	0.246805393	0.344737494	0.514194583	0.26738919	1.46E-09	3.49E-08
EXON1	EVC2	4	5710767/5710761/5710016/5710098	4	cg03278643/cg17242664/cg22290744/cg23665381	0.300297383	0.386255564	0.527761918	0.227464536	1.95E-06	1.60E-05
EXON1	EYA4	6	133562494/133562776/133562774	3	cg14270292/cg24176563/cg26656135	0.247029795	0.351895392	0.483049186	0.236019391	4.25E-06	3.18E-05
EXON1	FADS2	11	61595956/61596068/61595807	3	cg07005513/cg16576620/cg25303599	0.104630764	0.177917187	0.324015462	0.219384698	5.63E-05	0.00031733
EXON1	FADS6	17	72889549	1	cg00266322	0.164498418	0.244591401	0.396734987	0.23223657	4.85E-05	0.000277888
EXON1	FAIM2	12	50297588/50297581	2	cg16115588/cg27188703	0.355989765	0.464132031	0.593607943	0.237618178	2.37E-07	2.52E-06
EXON1	FAM110B	8	58907242	1	cg17151990	0.248215041	0.292197493	0.424324382	0.176109341	0.000763723	0.003252829
EXON1	FAM123A	13	25745406/25743608/25743999/25745768/25744676/25745784	6	cg07920503/cg11030811/cg13055385/cg14880499/cg18815647/cg22029275	0.519146995	0.603244805	0.707382414	0.188235419	1.57E-09	3.69E-08
EXON1	FAM132A	1	1181956	1	cg04719903	0.244664875	0.288058028	0.459094609	0.214429734	2.78E-05	0.000168355
EXON1	FAM155A	13	108518237/108519213/108518955/108518179/108518152/108518445/108518621/108518419/108519318	9	cg03333286/cg04607400/cg05383490/cg07223990/cg13398192/cg14119999/cg15669183/cg17673237/cg19839798	0.354260508	0.408212486	0.565954616	0.211694108	8.64E-08	1.06E-06
EXON1	FAM159A	1	53099248	1	cg18146843	0.36751812	0.476092667	0.610046791	0.242528671	1.08E-08	1.84E-07
EXON1	FAM159B	5	63986392	1	cg10981909	0.354039175	0.485957006	0.671355627	0.317316452	8.47E-10	2.32E-08
EXON1	FAM162B	6	117086850/117086601	2	cg07641160/cg23367608	0.232302324	0.278231633	0.45443854	0.222136216	1.08E-06	9.58E-06
EXON1	FAM183A	1	43613627/43613606	2	cg12059147/cg20991612	0.26131836	0.304062056	0.435364445	0.174046085	1.45E-06	1.23E-05
EXON1	FAM19A2	12	62585204/62585579/62585877/62586425/62585467/62586267/62586003	7	cg00089798/cg03001945/cg12243597/cg15736338/cg20010506/cg20041366/cg26953435	0.348404564	0.437283461	0.569949066	0.221544502	1.21E-10	5.44E-09
EXON1	FAM19A4	3	68981503	1	cg23967169	0.309262405	0.36991745	0.578523827	0.269261422	3.01E-08	4.34E-07
EXON1	FAM19A5	22	48972349/48885506/48885446/48972331	4	cg06273376/cg19030607/cg22643811/cg22989103	0.277995578	0.356453258	0.479829676	0.201834097	7.58E-09	1.37E-07
EXON1	FAM26E	6	116832940	1	cg23741330	0.508259095	0.4046786	0.306573218	-0.201685877	9.92E-13	2.68E-10

EXON1	FAM3D	3	58652346/58652503	2	cg02194211/cg24121168	0.547127148	0.456620672	0.382733141	-0.164394007	1.20E-10	5.44E-09
EXON1	FAM43B	1	20880663/20879813/20879029/20880461/20879286/20879644/20879547/20880378/20879296/20880591	10	cg00527174/cg02582387/cg03754311/cg04850423/cg05771261/cg06393286/cg12768681/cg17543296/cg20404387/cg23993425	0.266384089	0.336156505	0.456769948	0.190385859	7.44E-09	1.35E-07
EXON1	FAM72B	1	120839380/120839385/120839390	3	cg00198436/cg07236150/cg09169215	0.066481005	0.145489607	0.294410058	0.227929053	5.58E-07	5.29E-06
EXON1	FBN1	15	48937856/48937958	2	cg03535663/cg19908534	0.11212821	0.219897732	0.34313974	0.231011531	4.17E-05	0.00024232
EXON1	FBN2	5	127873283/127873397/127873106/127873552/127873711	5	cg05209584/cg12940822/cg17564775/cg17958315/cg25084878	0.26655335	0.371927499	0.558581465	0.292028115	1.74E-07	1.93E-06
EXON1	FBXL21	5	135266135	1	cg01283246	0.340430175	0.415659217	0.519995168	0.179564993	9.29E-05	0.000494763
EXON1	FBXL7	5	15500714	1	cg06577205	0.344421575	0.375808717	0.528633736	0.184212161	0.000116015	0.000603524
EXON1	FBXO39	17	6679564	1	cg02374745	0.51562654	0.604659667	0.748948491	0.233321951	2.26E-10	8.48E-09
EXON1	FERD3L	7	19184427/19184950/19184555/19184961	4	cg02503874/cg10477621/cg23536473/cg25691167	0.403259229	0.521929385	0.647663228	0.244404	1.13E-11	1.24E-09
EXON1	FEZF2	3	62358980	1	cg19629292	0.211013726	0.295144095	0.404459859	0.193446134	0.000369415	0.001701599
EXON1	FGB	4	155484272	1	cg21082050	0.60566691	0.555381978	0.422779214	-0.182887696	4.55E-07	4.42E-06
EXON1	FGF10	5	44388688/44388596	2	cg14654926/cg22574802	0.24725103	0.306616033	0.40693698	0.15968595	1.16E-05	7.75E-05
EXON1	FGF12	3	192126244/192445380/192126165/192126584/192126825/192126023/192445307/192126742	8	cg00386551/cg02992632/cg04003850/cg04350913/cg16209795/cg18780257/cg19507527/cg21197774	0.1959348	0.283200399	0.43707825	0.241143451	3.80E-08	5.26E-07
EXON1	FGF4	11	69590090/69590113	2	cg19831575/cg23964057	0.151178155	0.239285329	0.42840766	0.277229506	7.18E-07	6.63E-06
EXON1	FGF5	4	81187798/81187906	2	cg02002231/cg14197923	0.106380913	0.196157855	0.391203448	0.284822535	2.36E-05	0.000145811
EXON1	FHL5	6	97010439	1	cg23978322	0.786763655	0.627829161	0.527864168	-0.258899487	2.96E-08	4.29E-07
EXON1	FIGLA	2	71017557/71017637/71017541	3	cg00469856/cg16907558/cg22650617	0.34936377	0.449135156	0.614173406	0.264809636	4.35E-10	1.36E-08
EXON1	FLI1	11	128564106/128563903/128562408	3	cg06072021/cg06172475/cg17476271	0.115084187	0.270050914	0.480289485	0.365205298	9.37E-06	6.41E-05

EXON1	FLJ20184	4	106473806	1	cg27126442	0.54336524	0.439075839	0.345794486	-0.197570754	2.09E-09	4.62E-08
EXON1	FLJ23834	7	105603743	1	cg02661623	0.676365055	0.588094428	0.513351982	-0.163013073	3.30E-05	0.000196301
EXON1	FLRT2	14	85996499/85996751/85996495/85996873	4	cg01711160/cg05855039/cg17410236/cg25120290	0.387769243	0.505374665	0.658286638	0.270517395	1.76E-10	7.11E-09
EXON1	FMN2	1	240255377/240256014/240256644/240256603/240255691/240255627/240256019/240255486	8	cg02574509/cg05658491/cg06400704/cg15031661/cg17012160/cg22931725/cg24643282/cg25208017	0.195649768	0.235530623	0.356289674	0.160639906	0.00079231	0.003363494
EXON1	FNDC1	6	159590578	1	cg14971597	0.174748926	0.286958762	0.397212366	0.222463441	1.04E-06	9.25E-06
EXON1	FOLH1	11	49230168/49230036	2	cg14775560/cg25346915	0.291977923	0.319281246	0.501149359	0.209171437	1.49E-05	9.78E-05
EXON1	FOLH1B	11	89392829	1	cg02915544	0.59441058	0.412695106	0.265445155	-0.328965425	2.35E-14	3.78E-11
EXON1	FOXC2	16	86602533/86602339/86601024/86601520/86601361/86601133/86602083/86602471	8	cg03190266/cg03613339/cg04081201/cg04510512/cg07844659/cg08384637/cg09195198/cg26948907	0.264726497	0.320529613	0.448292781	0.183566284	8.88E-07	8.01E-06
EXON1	FOXE1	9	100618041/100617710/100616607/100617370/100615796/100617999/100616469	7	cg04007303/cg13696609/cg13791254/cg17702692/cg19292008/cg21174841/cg24393316	0.33428108	0.428452115	0.533851838	0.199570757	8.32E-09	1.48E-07
EXON1	FOXF1	16	86544346/86544308/86544787/86544339/86544658	5	cg00314966/cg08157228/cg10514097/cg25971347/cg27122536	0.21386133	0.244452614	0.425902228	0.212040898	1.20E-07	1.40E-06
EXON1	FOXF2	6	1390998/1390361/1390622/1391265/1390838/1391207	6	cg02386403/cg02547269/cg12221475/cg17305266/cg24151352/cg26987597	0.250818699	0.285224878	0.423519598	0.172700899	0.000222651	0.00108488
EXON1	FOXG1	14	29236535/29236898/29236323/29236476/29238731/29237202/29237480	7	cg05872306/cg07489048/cg10300684/cg10828337/cg12150366/cg16582779/cg16804284	0.45243924	0.523635318	0.654720264	0.202281024	8.33E-11	4.31E-09
EXON1	FOXI2	10	129535786/129535669/129535898/129535968/129535893	5	cg02595832/cg07918545/cg13929328/cg19884262/cg26115633	0.477692066	0.541990444	0.67681477	0.199122704	1.12E-07	1.34E-06

EXON1	FOXN4	12	109747000	1	cg22326903	0.068558682	0.145944608	0.267626035	0.199067353	0.000908907	0.003803836
EXON1	FPR2	19	52266642	1	cg14203721	0.69256589	0.55737735	0.455171773	-0.237394117	3.72E-06	2.84E-05
EXON1	FREM3	4	144621089/144621385/144620341/144618119	4	cg00816770/cg04514249/cg04993441/cg19172665	0.536107185	0.621068931	0.699362274	0.163255089	4.27E-09	8.48E-08
EXON1	FRK	6	116381609/116381904	2	cg16176600/cg26893134	0.429479963	0.340561322	0.272251695	-0.157228267	8.52E-12	9.97E-10
EXON1	FSCN3	7	127233742	1	cg16725721	0.80322332	0.700517317	0.613380168	-0.189843152	4.00E-07	3.97E-06
EXON1	FSTL1	3	120169783/120169821	2	cg03424342/cg15109851	0.275980793	0.326929212	0.451583101	0.175602309	0.000543829	0.002403715
EXON1	FUT3	19	5851465	1	cg26899156	0.40437472	0.318910683	0.251205532	-0.153169188	9.96E-10	2.58E-08
EXON1	FUT9	6	96463902/96463959	2	cg01758512/cg25114913	0.474523435	0.551099139	0.648341002	0.173817567	3.02E-10	1.06E-08
EXON1	FZD10	12	130647890/130648987/130649720/130647213/130647874/130648519/130647580/130647289	8	cg01568919/cg07742404/cg13859208/cg15928093/cg16498194/cg22347212/cg23054883/cg27232145	0.409506219	0.47294812	0.583741882	0.174235663	1.10E-09	2.80E-08
EXON1	G6PC	17	41053112	1	cg10596768	0.47740397	0.409225667	0.261498982	-0.215904988	4.51E-08	6.03E-07
EXON1	GABBR1	6	29595349/29595335/29595653/29595299/29595315/29595506/29595661/29600642/29595491/29595347	10	cg00594408/cg02014853/cg06078334/cg08093277/cg08862148/cg15385139/cg18344922/cg19456996/cg20997792/cg25729445	0.295175135	0.368277819	0.446131639	0.150956504	7.23E-07	6.66E-06
EXON1	GABRA5	15	27111911/27111949/27112197/27111940/27112430/27112119/27111993/27111884/27112287/27112305/27112049	11	cg01378667/cg03325535/cg05044185/cg10318222/cg10652393/cg15665646/cg18255240/cg20005743/cg21276413/cg21402921/cg22523852	0.318817158	0.398405872	0.515148873	0.196331715	6.96E-11	3.89E-09
EXON1	GABRA6	5	161112787	1	cg22672790	0.391572255	0.294535011	0.191274473	-0.200297782	4.56E-11	2.99E-09
EXON1	GABRB3	15	27018874/27018037	2	cg01690182/cg18026588	0.345069133	0.4230742	0.546525002	0.20145587	4.64E-09	9.07E-08
EXON1	GABRR3	3	97753734	1	cg02058357	0.70333369	0.615681872	0.507811586	-0.195522104	7.47E-06	5.24E-05
EXON1	GAD2	10	26505442/26505503/26505440/26505245	4	cg04599297/cg09056181/cg11328303/cg15850954	0.277074423	0.44180043	0.613517622	0.336443198	2.92E-10	1.03E-08

EXON1	GAL3ST3	11	65816521/65816646/65816642	3	cg14780632/cg15916004/cg21238818	0.443559352	0.545256459	0.644654	0.201094648	9.52E-11	4.69E-09
EXON1	GALNT13	2	154728735/154728468	2	cg10033761/cg12079322	0.429723645	0.429760575	0.596179464	0.166455819	3.83E-08	5.30E-07
EXON1	GALNT14	2	31361104/31361371	2	cg05714219/cg24597158	0.05724639	0.146009878	0.298807256	0.241560866	8.02E-05	0.000434104
EXON1	GALNT9	12	132690570/132905819/132905735/132905611/132690536	5	cg01658960/cg02228111/cg04606861/cg05143633/cg15128141	0.38866032	0.454216559	0.576499965	0.187839645	7.64E-08	9.52E-07
EXON1	GALNTL6	4	172734776/172734843/172734718	3	cg03413465/cg15533075/cg26588943	0.276865617	0.363287785	0.517858048	0.240992432	8.58E-10	2.34E-08
EXON1	GALR1	18	74962216/74962133/74962369/74962672/74962794	5	cg01178451/cg03502002/cg04534765/cg06360427/cg10390058	0.4910424	0.546092374	0.680061158	0.189018758	5.01E-08	6.60E-07
EXON1	GATA4	8	11561724/11561728	2	cg14666113/cg18283386	0.206845058	0.371160593	0.619421941	0.412576883	1.47E-11	1.45E-09
EXON1	GATA5	20	61051021	1	cg12664464	0.24066349	0.317183878	0.472858841	0.232195351	8.96E-05	0.000479981
EXON1	GCM1	6	53013589	1	cg26023389	0.55671574	0.423751589	0.338603055	-0.218112685	4.02E-12	6.05E-10
EXON1	GCM2	6	10882043/10881949/10882050	3	cg02844545/cg22851295/cg25861699	0.29229044	0.380182044	0.518191273	0.225900833	1.99E-07	2.17E-06
EXON1	GCNT3	15	59904009	1	cg02073465	0.46463514	0.361757133	0.278809759	-0.185825381	3.66E-10	1.19E-08
EXON1	GDF6	8	97172961/97172825	2	cg00421139/cg05701791	0.27426345	0.383657553	0.526804948	0.252541498	1.35E-08	2.21E-07
EXON1	GFRA1	10	118032892/118032872/118033115/118032879/118032948/118031632/118032987/118031654/118032905	9	cg08194313/cg13346013/cg13890706/cg18672939/cg23898073/cg24792682/cg25022687/cg27034576/cg27341926	0.191021015	0.299478243	0.461559397	0.270538381	2.51E-05	0.00015391
EXON1	GFRA2	8	21645807/21646174/21645923/21645870	4	cg00902636/cg06067208/cg09459291/cg14633783	0.166316193	0.209752566	0.325174522	0.158858329	0.002345091	0.0088896
EXON1	GHSR	3	172165696/172166182/172165884/172165517	4	cg03587978/cg07852825/cg10109500/cg18875012	0.359880243	0.427030631	0.542640266	0.182760023	8.32E-11	4.31E-09
EXON1	GIF	11	59612937	1	cg01282204	0.759301325	0.533209667	0.396963041	-0.362338284	3.62E-14	3.78E-11
EXON1	GIPC3	19	3585609	1	cg26797585	0.090448185	0.131938538	0.24506883	0.154620645	0.000893599	0.003745883
EXON1	GJC2	1	228337626	1	cg08228672	0.699027695	0.637135711	0.506537068	-0.192490627	5.95E-07	5.60E-06
EXON1	GJD2	15	35046650	1	cg14576896	0.336838605	0.433453756	0.548715186	0.211876581	3.73E-08	5.17E-07

EXON1	GLB1L3	11	134146324	1	cg18375494	0.065705825	0.098374453	0.246427984	0.180722159	0.003831347	0.013833147
EXON1	GLDC	9	6645468/6645686	2	cg11747771/cg17009433	0.118373012	0.231472501	0.38422785	0.265854839	2.71E-05	0.000164629
EXON1	GLIPR1L1	12	75728469/75728471	2	cg21087137/cg24234651	0.374702302	0.486920539	0.57722738	0.202525077	2.41E-08	3.61E-07
EXON1	GLRA3	4	175750373/175750109/175750280	3	cg05424071/cg21094669/cg27517702	0.22069827	0.294578594	0.467080559	0.24638229	8.08E-08	1.00E-06
EXON1	GLRB	4	157997363/157997554/157997367/157997360	4	cg09688546/cg11163620/cg16269431/cg22065614	0.223893995	0.308681188	0.499797991	0.275903996	3.38E-07	3.43E-06
EXON1	GNAL	18	11752538/11689284/11752215/11689613/11751566/11689206/11689218/11752089	8	cg06522054/cg09331011/cg10068300/cg12585806/cg14612428/cg15616946/cg15653282/cg25958283	0.233843885	0.31017893	0.396972271	0.163128386	1.08E-10	5.09E-09
EXON1	GPC6	13	93879769/93879544/93879361/93879670/93879303	5	cg07576142/cg12989574/cg16792800/cg18058689/cg19824907	0.360206601	0.431576618	0.653171036	0.292964435	2.57E-08	3.81E-07
EXON1	GPNMB	7	23286463/23286319/23286539	3	cg08455073/cg14444376/cg17274742	0.680779238	0.591910219	0.485355214	-0.195424024	8.49E-11	4.37E-09
EXON1	GPR115	6	47666329	1	cg14105536	0.624055265	0.479605228	0.351843545	-0.27221172	4.64E-13	1.66E-10
EXON1	GPR128	3	100328745/100328713	2	cg04071964/cg23855093	0.52189019	0.364772678	0.299061832	-0.222828358	3.32E-15	2.19E-11
EXON1	GPR133	12	131438788/131438737/131438527/131438626/131438523/131438540	6	cg02717046/cg19642421/cg20712263/cg21502466/cg22882539/cg23379751	0.256900721	0.378264591	0.463866477	0.206965756	1.67E-06	1.39E-05
EXON1	GPR139	16	20084944/20085047/20084851	3	cg00938050/cg01126567/cg01944044	0.292818248	0.375403906	0.500397327	0.20757908	9.72E-08	1.17E-06
EXON1	GPR149	3	154146816/154146871/154146536/154146704	4	cg02589901/cg04919274/cg08447525/cg26856316	0.324658003	0.404312196	0.523140693	0.198482691	2.57E-10	9.32E-09
EXON1	GPR183	13	99959606	1	cg16131748	0.503339165	0.602018767	0.683022432	0.179683267	4.96E-11	3.15E-09
EXON1	GPR31	6	167571172	1	cg22182975	0.823243145	0.702426	0.629086391	-0.194156754	2.45E-08	3.66E-07
EXON1	GPR75	2	54087008/54087028/54087066	3	cg01876338/cg14832904/cg19961545	0.543281248	0.671198724	0.80921843	0.265937182	6.06E-14	4.72E-11
EXON1	GPR83	11	94134446/94134580/94134235	3	cg03345037/cg07586272/cg22325703	0.24938343	0.344388867	0.465161561	0.215778131	7.01E-08	8.80E-07

EXON1	GPR85	7	112726089/112726538/112726541/112726396/112727506	5	cg00851377/cg13251269/cg20513548/cg24310431/cg25263801	0.284175822	0.365147168	0.439890258	0.155714437	0.000160892	0.000811455
EXON1	GPT2	16	46919112/46919194	2	cg03533472/cg23684449	0.68016188	0.507733967	0.4307959	-0.24936598	2.41E-07	2.56E-06
EXON1	GPX2	14	65409452/65409354	2	cg09643186/cg19502457	0.551342598	0.449911528	0.374119852	-0.177222745	1.05E-10	4.95E-09
EXON1	GPX7	1	53068197/53068181	2	cg16557944/cg20950465	0.104817322	0.149943626	0.313647448	0.208830126	0.000146528	0.000744399
EXON1	GRASP	12	52400907	1	cg04034767	0.093413792	0.201368981	0.460650285	0.367236493	1.13E-06	9.93E-06
EXON1	GRIA1	5	152870490/152870258	2	cg08578734/cg17020834	0.255845245	0.277135519	0.417668555	0.16182331	0.00013114	0.000675303
EXON1	GRIA2	4	158141936	1	cg25148589	0.41564434	0.496915111	0.609750136	0.194105796	2.22E-07	2.38E-06
EXON1	GRIA4	11	105480979/105481802/105481509	3	cg03243226/cg09980522/cg19343464	0.278805595	0.397171828	0.567878198	0.289072603	6.78E-09	1.25E-07
EXON1	GRID1	10	88126089/88126104	2	cg03356747/cg03370738	0.110334487	0.1974978	0.303397618	0.193063131	0.000132632	0.000681658
EXON1	GRIK1	21	31311797/31312091/31311842	3	cg09199698/cg09542111/cg23475371	0.305930122	0.396502452	0.500280458	0.194350336	5.42E-08	7.04E-07
EXON1	GRIK2	6	101846916/101846905/101847058/101846872/101846967	5	cg10591607/cg18193094/cg21635870/cg24301620/cg26316946	0.430119524	0.531147041	0.650800781	0.220681257	1.78E-10	7.14E-09
EXON1	GRIK3	1	37499649	1	cg20168230	0.414037925	0.498189444	0.588076255	0.17403833	3.89E-09	7.79E-08
EXON1	GRIN2A	16	10276092/10275934/10275767/10276580/10276081/10276119/10276600	7	cg02585344/cg05406943/cg06193383/cg09239744/cg09461286/cg16904585/cg24764979	0.142133897	0.16958455	0.314497083	0.172363186	3.31E-05	0.000196844
EXON1	GRM1	6	146348913/146348901/146348890	3	cg03478199/cg19512268/cg21725954	0.342325205	0.398700128	0.542430305	0.2001051	7.56E-07	6.94E-06
EXON1	GRM7	3	6903153/6902824/6902845/6903327/6903019	5	cg02332525/cg17892178/cg18863595/cg21187352/cg27199820	0.444673461	0.536179492	0.688703268	0.244029807	1.07E-12	2.80E-10
EXON1	GRP	18	56887410/56887404	2	cg17093795/cg27338487	0.37645826	0.466691264	0.559288411	0.182830151	4.00E-07	3.97E-06
EXON1	GRXCR1	4	42895607	1	cg14184077	0.715493885	0.63083845	0.511156164	-0.204337721	1.23E-05	8.21E-05
EXON1	GSC	14	95236123	1	cg10042799	0.11632951	0.085090548	0.282819955	0.166490446	0.0060141	0.020648982
EXON1	GSDMB	17	38073560	1	cg21300187	0.86010892	0.746410522	0.623917	-0.23619192	2.27E-06	1.83E-05
EXON1	GSDMC	8	130799007/130799093	2	cg05316065/cg26073844	0.558587478	0.434942542	0.3371831	-0.221404378	1.51E-10	6.51E-09

EXON1	GSTM2	1	110210684/110210770/110210699	3	cg03070194/cg07219542/cg08142344	0.115605779	0.204242237	0.294182994	0.178577215	4.04E-07	4.00E-06
EXON1	GSX1	13	28366814	1	cg26609631	0.432714115	0.458144839	0.595039364	0.162325249	6.08E-07	5.69E-06
EXON1	GTSF1L	20	42355625/42354942/42354839	3	cg11398517/cg23727943/cg24118151	0.713177073	0.624946717	0.555956691	-0.157220382	1.69E-11	1.61E-09
EXON1	GUCA2A	1	42630335	1	cg05398883	0.584170085	0.446874589	0.356594955	-0.22757513	5.78E-11	3.47E-09
EXON1	GUCA2B	1	42619118/42619163	2	cg15976404/cg19728577	0.652472495	0.540815244	0.454725536	-0.197746959	1.61E-09	3.75E-08
EXON1	GUCY1B3	4	156680256/156680207	2	cg03584891/cg16700392	0.418848618	0.499077622	0.617188584	0.198339967	1.79E-09	4.08E-08
EXON1	GYPC	2	127413831	1	cg15975865	0.30371499	0.489046279	0.648228845	0.344513855	2.22E-08	3.35E-07
EXON1	GYS2	12	21757759/21757466	2	cg04184278/cg06141025	0.64837717	0.5401587	0.437659693	-0.210717477	1.17E-09	2.92E-08
EXON1	HAND2	4	174450016/174451251/174450722/174450353/174450408/174451141	6	cg01580681/cg02774439/cg10541864/cg15707093/cg19178853/cg25102370	0.300950688	0.391175132	0.534724169	0.233773481	8.90E-07	8.02E-06
EXON1	HAPLN4	19	19373566/19373575	2	cg01297721/cg22694191	0.33803368	0.375740819	0.525827877	0.187794197	0.000405526	0.001847349
EXON1	HBE1	11	5291298/5291214	2	cg03306956/cg16279158	0.5609776	0.500748483	0.367565418	-0.193412182	9.25E-09	1.62E-07
EXON1	HBQ1	16	230343/230341	2	cg02401454/cg07703401	0.32724683	0.338138094	0.520321184	0.193074354	4.73E-05	0.000271373
EXON1	HCK	20	30640164/30640256/30640121	3	cg08623947/cg13042543/cg24606935	0.094874883	0.177125793	0.29829087	0.203415987	0.000378298	0.00173647
EXON1	HCRTR2	6	55039232/55039259/55039382/55039295	4	cg10725720/cg13948987/cg17240815/cg27596495	0.282031535	0.328409048	0.488993624	0.206962089	1.05E-07	1.26E-06
EXON1	HECW1	7	43152254/43152468	2	cg08039116/cg17438849	0.341357783	0.396508517	0.493948634	0.152590852	2.01E-06	1.64E-05
EXON1	HELT	4	185940173	1	cg17186107	0.251086025	0.284845711	0.490349859	0.239263834	7.71E-07	7.05E-06
EXON1	HEPACAM2	7	92848714/92855729	2	cg04992673/cg11058951	0.704928635	0.603657517	0.540253266	-0.164675369	1.18E-10	5.40E-09
EXON1	HEPHL1	11	93754390	1	cg18109485	0.454738565	0.361792817	0.26383015	-0.190908415	1.92E-12	3.84E-10
EXON1	HEPN1	11	124789499/124790365	2	cg20325560/cg22871233	0.86396429	0.800697256	0.701462732	-0.162501558	1.43E-05	9.42E-05
EXON1	HES3	1	6304272	1	cg22699074	0.35536053	0.423482011	0.517931182	0.162570652	0.000234729	0.001134124
EXON1	HFE2	1	145413337/145413193	2	cg00987513/cg06589885	0.715596228	0.667351964	0.561544945	-0.154051282	5.60E-08	7.24E-07
EXON1	HGD	3	120401058/120401095	2	cg04546097/cg14527029	0.534031683	0.436645867	0.35923247	-0.174799212	2.45E-10	9.01E-09
EXON1	HHLA1	8	133117493	1	cg18187729	0.47645342	0.331186889	0.240830777	-0.235622643	2.35E-13	1.07E-10
EXON1	HIST1H4F	6	26240920/26240782/26240939	3	cg08260959/cg10723962/cg21425842	0.396915558	0.543511659	0.643314298	0.246398741	7.04E-09	1.29E-07

EXON1	HMGCS2	1	120311542/120311439	2	cg13976683/cg22718139	0.542833315	0.417908067	0.341307155	-0.20152616	4.79E-11	3.07E-09
EXON1	HMX2	10	124907942/124908083/124907708	3	cg00474465/cg05189242/cg19909787	0.179368496	0.209047892	0.348239122	0.168870626	6.78E-05	0.000374947
EXON1	HMX3	10	124895886/124895638/124895697/124895693	4	cg10491556/cg11628754/cg19564098/cg20403498	0.153732523	0.206982268	0.329484849	0.175752327	0.000155028	0.000784569
EXON1	HNF4A	20	43029994/43029992/43029997/42984453	4	cg07064544/cg11542165/cg21058973/cg23792485	0.643394972	0.525393524	0.420941898	-0.222453073	4.99E-11	3.15E-09
EXON1	HOXA2	7	27142100/27142204/27141774/27142275/27142200	5	cg04481096/cg06055873/cg17353412/cg20747380/cg26069745	0.408681513	0.517218417	0.664497248	0.255815735	5.11E-07	4.89E-06
EXON1	HOXB8	17	46692021/46692008/46691820/46692248/46692168	5	cg04453140/cg06760710/cg08151857/cg15539420/cg22913933	0.440359205	0.310490867	0.262706035	-0.17765317	2.86E-05	0.00017313
EXON1	HOXC10	12	54379609/54379056	2	cg00699029/cg26153631	0.432911375	0.499609328	0.628421255	0.19550988	3.61E-08	5.03E-07
EXON1	HOXC11	12	54367460/54367250/54367008/54366909	4	cg03536474/cg07123069/cg15830509/cg22709192	0.366256267	0.424192346	0.532338264	0.166081997	4.14E-08	5.63E-07
EXON1	HOXC13	12	54333179/54333323/54333224/54332992/54332695/54332624	6	cg03972398/cg07892422/cg11255394/cg16856286/cg19977966/cg25671500	0.406879896	0.47553009	0.578163491	0.171283595	2.70E-09	5.69E-08
EXON1	HOXC9	12	54393966/54393971/54394194/54394212/54394116	5	cg05069426/cg12071536/cg12699286/cg24500832/cg25456468	0.295738199	0.348657289	0.573071898	0.2773337	9.21E-08	1.12E-06
EXON1	HS3ST2	16	22826113/22826243/22826117	3	cg03757784/cg08214995/cg19064258	0.366387783	0.449275592	0.592313416	0.225925633	2.05E-07	2.23E-06
EXON1	HS3ST3A1	17	13504687/13504008/13505174/13504399/13504472	5	cg03436461/cg04211115/cg07480567/cg18255166/cg22583981	0.371618757	0.451906654	0.555304765	0.183686008	1.02E-07	1.22E-06
EXON1	HSPB2	11	111783563/111783499	2	cg11694519/cg12947833	0.51541418	0.643971275	0.732348393	0.216934213	1.24E-10	5.58E-09
EXON1	HSPB3	5	53751493/53751919/53752133	3	cg01246617/cg11391732/cg21168101	0.760983625	0.678681707	0.60616392	-0.154819705	2.01E-09	4.51E-08

EXON1	HTR1A	5	63257499/63256926/63256619/63256285/63257092	5	cg04427003/cg04799838/cg10588470/cg23448729/cg27615388	0.423006308	0.497169644	0.577117131	0.154110823	4.52E-09	8.89E-08
EXON1	HTR1B	6	78172691/78172337/78172192/78172950	4	cg00181439/cg15368905/cg21232620/cg25763788	0.398094659	0.482755352	0.627270459	0.2291758	3.17E-10	1.09E-08
EXON1	HTR1E	6	87647154/87647399/87647147/87647376/87647129/87647527/87647370	7	cg02928916/cg04278702/cg07833420/cg09297468/cg09863441/cg10135483/cg14774364	0.273100513	0.369062302	0.56478146	0.291680947	5.87E-09	1.11E-07
EXON1	HTR3E	3	183817976/183818368	2	cg01953456/cg26153642	0.632772755	0.5249243	0.437669218	-0.195103537	9.38E-09	1.64E-07
EXON1	HTR7	10	92617162/92617429/92617562	3	cg06291867/cg15364725/cg26135506	0.283561111	0.327760229	0.454404764	0.170843653	3.53E-05	0.000208598
EXON1	HTRA3	4	8271507/8271792	2	cg09495977/cg23365637	0.246006178	0.249152199	0.40332575	0.157319573	0.002922907	0.010849365
EXON1	IFNA8	9	21409505	1	cg15669228	0.68945982	0.614549933	0.486504291	-0.202955529	5.16E-08	6.76E-07
EXON1	IGFL3	19	46627896	1	cg15507486	0.5851034	0.4689182	0.341397627	-0.243705773	6.11E-12	7.73E-10
EXON1	IGFL4	19	46544240	1	cg27048142	0.61426804	0.466338333	0.368022105	-0.246245935	1.02E-09	2.62E-08
EXON1	IL12B	5	158757456	1	cg18307303	0.651192595	0.548948333	0.458741455	-0.19245114	6.27E-08	7.99E-07
EXON1	IL1F9	2	113735612	1	cg15657888	0.43023582	0.325292839	0.273220991	-0.157014829	4.65E-12	6.53E-10
EXON1	IL1R2	2	102615524/102608349	2	cg12910851/cg20340242	0.41936118	0.312422933	0.241203418	-0.178157762	7.27E-11	4.02E-09
EXON1	IL22	12	68647280	1	cg11520493	0.83779575	0.797853883	0.657353691	-0.180442059	3.79E-06	2.89E-05
EXON1	IL25	14	23842125	1	cg07258507	0.42593412	0.331148294	0.238360264	-0.187573856	2.46E-11	1.98E-09
EXON1	IL4	5	132009630	1	cg12377972	0.79358193	0.722593817	0.610814186	-0.182767744	1.10E-06	9.70E-06
EXON1	INHBA	7	41742630	1	cg11079619	0.67982674	0.570753806	0.474324818	-0.205501922	3.82E-08	5.28E-07
EXON1	INHBB	2	121104029/121104187	2	cg02173749/cg03699182	0.185696665	0.210141327	0.36664773	0.180951065	8.47E-05	0.000455244
EXON1	IQCF2	3	51895692	1	cg14940420	0.690985415	0.567873522	0.437973032	-0.253012383	1.92E-07	2.11E-06
EXON1	IRAK3	12	66583029/66583203/66583048	3	cg01263292/cg10389229/cg26415547	0.092280667	0.152925613	0.311538727	0.21925806	1.53E-05	9.98E-05
EXON1	IRF4	6	391764	1	cg17228900	0.100866439	0.314322966	0.585962585	0.485096146	1.58E-08	2.49E-07
EXON1	IRX4	5	1882775/1882871	2	cg03679565/cg03963198	0.296017495	0.365641589	0.537900323	0.241882828	0.000114971	0.000599033

EXON1	ISLR2	15	74422275/74422750/74421935/74421857/74421739/74421848/74422935/74421887/74422761/74422282/74422933/74421918/74421909	13	cg00550503/cg02378847/cg02709205/cg06041677/cg07216112/cg08703540/cg11315754/cg12017911/cg21063758/cg23152885/cg24374861/cg25705558/cg27200833	0.211121754	0.244540606	0.386707196	0.175585442	2.07E-05	0.000130371
EXON1	ITGA4	2	182322268/182322058/182321855/182322279/182322501/182321786	6	cg06952671/cg10526659/cg20415809/cg21995919/cg25024074/cg25652029	0.260763156	0.458986749	0.611804352	0.351041196	4.37E-10	1.36E-08
EXON1	ITGA8	10	15761657	1	cg01078989	0.197064855	0.313386939	0.426789773	0.229724918	4.06E-06	3.06E-05
EXON1	ITGB6	2	161056520	1	cg21105318	0.499303555	0.393083061	0.310163259	-0.189140296	2.60E-11	2.03E-09
EXON1	ITIH2	10	7745454	1	cg06224510	0.52290188	0.361924694	0.230658309	-0.292243571	9.34E-14	5.47E-11
EXON1	ITIH4	3	52864659/52864694	2	cg10929387/cg11645453	0.718289485	0.606850944	0.554575986	-0.163713499	1.61E-06	1.35E-05
EXON1	ITIH5	10	7661611/7708869/7708805	3	cg00677195/cg04711998/cg22444507	0.393885451	0.476519057	0.592943632	0.199058181	4.97E-09	9.60E-08
EXON1	IYD	6	150690125/150690143	2	cg02307277/cg20416179	0.531765885	0.431119939	0.344286614	-0.187479271	4.21E-11	2.84E-09
EXON1	IZUMO1	19	49249932/49250080	2	cg10234985/cg18537730	0.491661105	0.579073742	0.712085841	0.220424736	2.33E-09	5.04E-08
EXON1	JAKMIP1	4	6202091	1	cg01519742	0.131983322	0.208737012	0.290841264	0.158857942	1.47E-05	9.66E-05
EXON1	JAM2	21	27011788/27012176/27011963/27011793/27012066	5	cg02485200/cg03382304/cg13769223/cg14553600/cg22627390	0.119049045	0.211693495	0.363780131	0.244731086	3.83E-06	2.91E-05
EXON1	JAM3	11	133938941	1	cg02174225	0.125843112	0.265508881	0.442115258	0.316272146	9.84E-08	1.18E-06
EXON1	JPH3	16	87636539/87636782/87636594	3	cg08145698/cg09552213/cg10453425	0.133119763	0.238026907	0.346069867	0.212950105	1.40E-06	1.20E-05
EXON1	KANK1	9	707166	1	cg14399863	0.515628	0.623913972	0.715907782	0.200279782	4.18E-09	8.34E-08
EXON1	KATNAL2	18	44526867	1	cg05529506	0.053363597	0.135794353	0.245377774	0.192014177	0.00010515	0.000552214
EXON1	KAZALD1	10	102821427/102821670/102821684/102821848/102821552/102821565	6	cg07638935/cg08227353/cg12060422/cg15302379/cg23534216/cg26605164	0.448643923	0.345533509	0.266995382	-0.181648541	2.94E-12	5.13E-10
EXON1	KCNA1	12	5019311/5019482/5019091	3	cg09487611/cg10363246/cg12908522	0.126730906	0.241964179	0.476888771	0.350157865	1.56E-07	1.76E-06

EXON1	KCNA3	1	111217575/111217497/111216387/111216000/111216839/111214851/111216332/111217194/111216661/111217406	10	cg01423964/cg11595545/cg13925011/cg14200574/cg15726426/cg17033047/cg19942256/cg20302133/cg21286921/cg26013553	0.385808761	0.49588748	0.584506598	0.198697838	2.82E-12	4.99E-10
EXON1	KCNA4	11	30038310/30038286	2	cg15183083/cg27319123	0.342299205	0.441663047	0.574568002	0.232268797	1.70E-07	1.90E-06
EXON1	KCNC2	12	75603343/75603401/75603184/75603068/75603480	5	cg11308277/cg18573383/cg21500166/cg26074603/cg27154343	0.382166804	0.505617997	0.63906464	0.256897836	3.01E-10	1.06E-08
EXON1	KCNH5	14	63511872	1	cg11755405	0.38196622	0.495121994	0.6589143	0.27694808	1.88E-10	7.44E-09
EXON1	KCNH7	2	163695190/163695111	2	cg10132588/cg14781189	0.41570884	0.508834197	0.626071409	0.210362569	1.41E-10	6.16E-09
EXON1	KCNK12	2	47797415/47797133	2	cg02318629/cg13223402	0.116632551	0.206696124	0.337202411	0.22056986	3.42E-08	4.84E-07
EXON1	KCNQ2	20	62103627/62103541	2	cg08003150/cg26495865	0.049402304	0.116375314	0.215174603	0.165772299	0.000104027	0.00054697
EXON1	KCNQ5	6	73331680/73331652/73332073	3	cg03786924/cg15717808/cg24687051	0.241818556	0.390541533	0.588875241	0.347056685	1.78E-11	1.66E-09
EXON1	KCTD8	4	44450234/44450358/44449738	3	cg10059660/cg12300353/cg20646280	0.354856387	0.402319196	0.602880356	0.248023969	4.67E-11	3.01E-09
EXON1	KDR	4	55991418/55991683/55991701	3	cg07098391/cg10740902/cg25722465	0.523004225	0.591848633	0.682941223	0.159936998	3.79E-10	1.22E-08
EXON1	KERA	12	91451819	1	cg20332354	0.574654435	0.43442825	0.333692345	-0.24096209	4.87E-11	3.11E-09
EXON1	KHDRBS2	6	62995876/62996022/62995963	3	cg00472801/cg16587616/cg18239753	0.395316767	0.479496617	0.604159612	0.208842845	8.58E-08	1.05E-06
EXON1	KIAA1383	1	232941775/232940990/232941055/232944625/232941530/232941706/232941253/232941468/232941176	9	cg00951395/cg02191044/cg02903907/cg03756929/cg05460318/cg06935979/cg15542798/cg16430166/cg18196782	0.368493227	0.417297662	0.527028638	0.158535411	7.16E-06	5.04E-05
EXON1	KIAA1683	19	18385244	1	cg15084902	0.505192385	0.391143	0.343202486	-0.161989899	2.18E-07	2.35E-06
EXON1	KIF5A	12	57944059/57943921/57944044	3	cg04270799/cg19323374/cg24405617	0.140581884	0.171334904	0.294062288	0.153480405	1.11E-05	7.47E-05
EXON1	KIF5C	2	149632960/149633226/149633112	3	cg11650013/cg12035092/cg24461964	0.071515292	0.156074336	0.259239909	0.187724617	0.000207364	0.001018271

EXON1	KIRREL2	19	36347907/36348034/36347919	3	cg00519185/cg06945936/cg09479015	0.162045664	0.267688109	0.350421579	0.188375915	0.000146957	0.000745722
EXON1	KLB	4	39408665	1	cg21880903	0.538633785	0.439864594	0.34328745	-0.195346335	5.16E-10	1.56E-08
EXON1	KLHL1	13	70682098/70682019/70682324/70682604/70682127/70682525/70681804	7	cg01661350/cg12917718/cg20523861/cg23267558/cg24014143/cg24401870/cg25514273	0.389431144	0.460954766	0.551210339	0.161779195	9.40E-08	1.14E-06
EXON1	KLK11	19	51530751/51529846	2	cg24762501/cg26310000	0.7145356	0.625658072	0.544115091	-0.170420509	1.43E-08	2.32E-07
EXON1	KLK12	19	51538134	1	cg24368865	0.46914602	0.380552894	0.280538386	-0.188607634	2.98E-10	1.05E-08
EXON1	KLK8	19	51504823	1	cg15153394	0.5790325	0.513655728	0.415149877	-0.163882623	2.05E-06	1.67E-05
EXON1	KLKB1	4	187148688	1	cg12536028	0.557554245	0.435691661	0.345760305	-0.21179394	1.79E-10	7.16E-09
EXON1	KPNA7	7	98805084	1	cg22498453	0.764286435	0.594902611	0.590208441	-0.174077994	3.57E-10	1.17E-08
EXON1	KRBA1	7	149412290	1	cg03788131	0.138089557	0.242209075	0.483524739	0.345435182	1.87E-09	4.24E-08
EXON1	KRT20	17	39041266/39041199/39041119/39041110	4	cg19114576/cg19449377/cg23901700/cg25124433	0.783862687	0.553260496	0.399192683	-0.384670003	1.24E-12	3.03E-10
EXON1	KRT31	17	39553736	1	cg20125516	0.537462945	0.431333867	0.3549294	-0.182533545	2.82E-11	2.17E-09
EXON1	KRT33A	17	39506964/39506938	2	cg00629088/cg15983005	0.637122283	0.539671822	0.449566309	-0.187555973	1.22E-11	1.28E-09
EXON1	KRT38	17	39597402	1	cg25306838	0.4231071	0.347799894	0.264167418	-0.158939682	4.41E-10	1.36E-08
EXON1	KRT7	12	52627342/52627047/52627272	3	cg09522147/cg09670128/cg25313172	0.421868168	0.536565783	0.689640498	0.26777233	2.68E-11	2.08E-09
EXON1	KRT72	12	52995070/52995250/52995295/52994933	4	cg10292376/cg18161890/cg20050826/cg23337631	0.555931681	0.639695472	0.712559533	0.156627852	1.45E-09	3.47E-08
EXON1	KRT80	12	52585695	1	cg16432350	0.55077068	0.459402311	0.381649923	-0.169120757	1.30E-09	3.17E-08
EXON1	KRTAP10-1	21	45959142	1	cg17903229	0.46414623	0.348451517	0.299173286	-0.164972944	2.59E-07	2.72E-06
EXON1	KRTAP10-11	21	46067411/46066942	2	cg00947859/cg04218030	0.46744202	0.381149633	0.308647309	-0.158794711	1.42E-08	2.32E-07
EXON1	KRTAP10-8	21	46032026/46032086	2	cg17778867/cg26386968	0.658003125	0.556273314	0.492793645	-0.16520948	7.89E-07	7.20E-06
EXON1	KRTAP11-1	21	32253206/32253760/32253584	3	cg02930687/cg07014174/cg12111295	0.633474755	0.555026842	0.457464657	-0.176010098	7.48E-10	2.10E-08
EXON1	KRTAP12-2	21	46086578	1	cg17970078	0.73003605	0.690192767	0.556822914	-0.173213136	6.36E-06	4.54E-05
EXON1	KRTAP15-1	21	31813075	1	cg16812893	0.59362799	0.440570944	0.334187759	-0.259440231	3.58E-13	1.35E-10
EXON1	KRTAP19-1	21	31852504	1	cg06692927	0.7788402	0.700502217	0.623202464	-0.155637736	1.98E-05	0.000125078
EXON1	KRTAP21-2	21	32119309	1	cg23581186	0.679008385	0.542251406	0.39001255	-0.288995835	2.13E-10	8.20E-09
EXON1	KRTAP23-1	21	31720835	1	cg18594551	0.65171676	0.562210428	0.464774514	-0.186942246	4.01E-10	1.28E-08

EXON1	KRTAP24-1	21	31654844/31654005/31655261	3	cg02131862/cg03721387/cg07172334	0.66400019	0.542804124	0.469869259	-0.194130931	2.29E-12	4.33E-10
EXON1	KRTAP4-12	17	39279735	1	cg10686758	0.445949885	0.330617539	0.281250095	-0.16469979	1.68E-09	3.88E-08
EXON1	KRTAP7-1	21	32202010	1	cg05533037	0.61733834	0.439819778	0.331629845	-0.285708495	4.13E-11	2.84E-09
EXON1	KRTAP8-1	21	32185331/32185365	2	cg12346967/cg24423088	0.722758035	0.599290661	0.512131177	-0.210626858	8.78E-08	1.07E-06
EXON1	KY	3	134369828	1	cg23201032	0.232375405	0.286864915	0.3872008	0.154825395	0.000320049	0.001494503
EXON1	L1TD1	1	62660556	1	cg19594218	0.36242214	0.2872157	0.210043786	-0.152378354	2.29E-10	8.57E-09
EXON1	LAMA1	18	7117680/7117790	2	cg07846220/cg18764577	0.361940153	0.458143292	0.61602922	0.254089068	7.13E-12	8.81E-10
EXON1	LAMB3	1	209825747/209825672/209825731	3	cg03977657/cg26107033/cg26533764	0.561902485	0.458811914	0.382028341	-0.179874144	1.09E-10	5.10E-09
EXON1	LAYN	11	111411289/111411381	2	cg02532096/cg03864000	0.291250943	0.420109953	0.501159723	0.20990878	7.65E-06	5.35E-05
EXON1	LBXCOR1	15	68118006	1	cg17082347	0.135106277	0.183650134	0.36080405	0.225697774	0.000144605	0.000735193
EXON1	LCE1B	1	152784914/152785473	2	cg07090653/cg12449852	0.729669125	0.6528083	0.479028127	-0.250640998	1.89E-06	1.56E-05
EXON1	LCE1D	1	152769246	1	cg11583211	0.5111435	0.4167869	0.316977677	-0.194165823	1.19E-09	2.96E-08
EXON1	LCN2	9	130911792	1	cg22438810	0.47500126	0.371037206	0.284364823	-0.190636437	4.28E-12	6.29E-10
EXON1	LGALS4	19	39303506/39303698	2	cg16731016/cg19419519	0.565538958	0.443910339	0.351229698	-0.21430926	2.49E-10	9.09E-09
EXON1	LHCGR	2	48982683/48982754/48982832	3	cg00209038/cg13423075/cg20156659	0.419088745	0.4553261	0.617299805	0.19821106	2.31E-09	5.02E-08
EXON1	LHFPL4	3	9595395	1	cg19822251	0.3022505	0.373086122	0.478690786	0.176440286	4.01E-08	5.48E-07
EXON1	LIFR	5	38595383/38556435	2	cg05912079/cg12587766	0.446497587	0.507735082	0.604393019	0.157895432	9.21E-06	6.32E-05
EXON1	LILRA6	19	54746613	1	cg19835973	0.72572726	0.584950672	0.542445532	-0.183281728	6.83E-05	0.000376701
EXON1	LILRB3	19	54726870	1	cg15816012	0.64083175	0.532339556	0.488177741	-0.152654009	1.77E-06	1.47E-05
EXON1	LIN28	1	26737318/26737272	2	cg02388865/cg19706817	0.316462558	0.4353874	0.497957273	0.181494715	7.56E-07	6.94E-06
EXON1	LIN28B	6	105405068	1	cg04560456	0.330428215	0.378783794	0.543564627	0.213136412	2.34E-06	1.88E-05
EXON1	LMO1	11	8285086/8284997/8285031	3	cg05245226/cg07283152/cg21460402	0.147183635	0.161614728	0.299076456	0.151892821	0.001208018	0.004915678
EXON1	LMX1A	1	165324810/165324990	2	cg14662355/cg24650501	0.200555817	0.223314299	0.353967346	0.153411529	0.007483472	0.025075698
EXON1	LOC100302652	2	54087008/54087028/54087066	3	cg01876338/cg14832904/cg19961545	0.543281248	0.671198724	0.80921843	0.265937182	6.06E-14	4.72E-11
EXON1	LOC401387	7	91792839	1	cg04644523	0.80108622	0.736191411	0.616002973	-0.185083247	7.44E-06	5.22E-05

EXON1	LOC728392	17	5403516/5403879/5404508/5403907/5404090/5403805/5404330/5404337	8	cg00343839/cg01433610/cg06462347/cg08554115/cg12848098/cg16558432/cg22298430/cg27230784	0.11312254	0.152819626	0.296134267	0.183011727	0.000246752	0.001182711
EXON1	LONRF2	2	100937944/100939045/100938444/100938903/100938813/100938799	6	cg03559235/cg07304692/cg14348439/cg14675211/cg14997226/cg23977631	0.084088822	0.258085331	0.391470726	0.307381903	4.06E-08	5.54E-07
EXON1	LOXHD1	18	44236918/44099028/44139159	3	cg06520279/cg12934788/cg20454888	0.666217658	0.598453056	0.515179192	-0.151038466	3.15E-10	1.08E-08
EXON1	LPPR5	1	99470129/99470207	2	cg14991984/cg18623672	0.270830868	0.405834217	0.581562782	0.310731914	1.66E-10	6.94E-09
EXON1	LRFN5	14	42077674/42077327/42077061	3	cg04784672/cg21547690/cg26967167	0.262014391	0.338581776	0.508601712	0.246587321	1.87E-08	2.89E-07
EXON1	LRG1	19	4540003	1	cg22375763	0.454345995	0.358875856	0.297928741	-0.156417254	4.31E-07	4.22E-06
EXON1	LRRC30	18	7231534	1	cg16326062	0.731889485	0.653939206	0.565861873	-0.166027612	4.71E-08	6.24E-07
EXON1	LRRC31	3	169587454	1	cg12183875	0.565481385	0.430653789	0.324949809	-0.240531576	4.74E-12	6.53E-10
EXON1	LRRC3B	3	26664404/26664308/26664621	3	cg03496122/cg18679410/cg26035366	0.342295433	0.425779706	0.567975393	0.225679961	3.23E-12	5.34E-10
EXON1	LRRC4B	19	51071294	1	cg00908733	0.222528575	0.263188078	0.381361082	0.158832507	0.003323802	0.01219711
EXON1	LRRC4C	11	40314618/40315404/40315392/40314978/40314771/40315337	6	cg09147985/cg17579667/cg17949440/cg19849428/cg22645719/cg23850899	0.428304199	0.444728341	0.643843741	0.215539542	1.47E-08	2.36E-07
EXON1	LRRC66	4	52883367	1	cg23789604	0.52948459	0.395611372	0.314139909	-0.215344681	6.74E-11	3.86E-09
EXON1	LRRIQ4	3	169539775/169540297/169540504/169540079	4	cg04748593/cg13793580/cg18155853/cg23081534	0.810342383	0.576680539	0.389583758	-0.420758625	1.79E-14	3.38E-11
EXON1	LTBP2	14	75078611/75078834/75078283/75078842	4	cg03802461/cg12921961/cg15926671/cg20718816	0.263384902	0.312184188	0.440212553	0.176827651	1.50E-06	1.27E-05
EXON1	LTF	3	46506374	1	cg15261665	0.51536741	0.585800356	0.667935541	0.152568131	6.48E-09	1.21E-07
EXON1	LUM	12	91505197	1	cg10401088	0.41827388	0.367109339	0.254836577	-0.163437303	2.41E-05	0.000148649
EXON1	LUZP2	11	24518737/24518776	2	cg06931933/cg09717526	0.277360133	0.309575603	0.460539434	0.183179302	2.11E-05	0.000132291
EXON1	LVRN	5	115298920/115298511/115298704/115298231/115298720	5	cg06410191/cg07224726/cg22010052/cg22489785/cg25044651	0.381499909	0.4665959	0.60040156	0.218901651	2.16E-10	8.27E-09

EXON1	LY6H	8	144241434/144242029/144241407	3	cg02539855/cg03699469/cg23484951	0.160616104	0.247647482	0.369943235	0.209327132	5.93E-05	0.000332183
EXON1	LY86	6	6589075	1	cg02212836	0.51580359	0.6107202	0.673016159	0.157212569	1.30E-10	5.79E-09
EXON1	LY96	8	74903761	1	cg13213009	0.51478751	0.629726933	0.686249568	0.171462058	2.96E-08	4.29E-07
EXON1	LYPD5	19	44324747/44306565	2	cg11898486/cg12072164	0.386054995	0.4309358	0.601844391	0.215789396	4.64E-06	3.44E-05
EXON1	LYZ	12	69742232	1	cg16097772	0.50166135	0.387022656	0.296439909	-0.205221441	1.47E-12	3.13E-10
EXON1	LYZL4	3	42451986	1	cg25867632	0.484291835	0.381461239	0.304597891	-0.179693944	1.94E-08	2.98E-07
EXON1	MADCAM1	19	496528	1	cg02783799	0.27667364	0.31780925	0.436273664	0.159600024	0.000112976	0.000589105
EXON1	MAL	2	95691500	1	cg22403344	0.132396917	0.293686192	0.438046159	0.305649243	1.19E-08	1.99E-07
EXON1	MAMSTR	19	49220102/49220142	2	cg15475323/cg18046677	0.479300838	0.396879964	0.310396886	-0.168903951	7.75E-09	1.39E-07
EXON1	MAP3K13	3	185080997	1	cg25264265	0.378890525	0.470843783	0.607942355	0.22905183	1.77E-09	4.05E-08
EXON1	MAP4K1	19	39108529/39108609	2	cg15679651/cg19457477	0.508028538	0.601004919	0.668260009	0.160231472	8.43E-08	1.04E-06
EXON1	MAP9	4	156297942/156298050/156298002	3	cg03616357/cg08015755/cg20334243	0.207608486	0.306153983	0.365018895	0.15741041	0.000411197	0.001869322
EXON1	MAS1	6	160328002	1	cg08784110	0.802870265	0.757641183	0.636234505	-0.16663576	7.30E-06	5.13E-05
EXON1	MATK	19	3801548/3801727/3786246	3	cg01388796/cg09416313/cg17429382	0.19456021	0.267437483	0.454817496	0.260257286	3.00E-07	3.10E-06
EXON1	MATN4	20	43936853	1	cg16667508	0.452680055	0.37490005	0.297240064	-0.155439991	2.59E-09	5.50E-08
EXON1	MCHR2	6	100442105/100442002	2	cg06610484/cg26981309	0.34170225	0.40551125	0.537922023	0.196219773	3.88E-09	7.79E-08
EXON1	MCM3AP	21	47704251/47705150	2	cg14211055/cg25737836	0.56452808	0.483792667	0.408676113	-0.155851967	3.24E-11	2.37E-09
EXON1	MDFI	6	41606317	1	cg01350077	0.134761777	0.262817074	0.459091135	0.324329358	1.36E-06	1.17E-05
EXON1	MEI1	22	42095536	1	cg11260046	0.40945288	0.503480361	0.618011595	0.208558715	5.50E-06	3.99E-05
EXON1	MEP1B	18	29770081	1	cg01941619	0.42988382	0.343256161	0.278936173	-0.150947647	1.27E-09	3.11E-08
EXON1	MFRP	11	119217331	1	cg09186818	0.48301678	0.414818267	0.315081205	-0.167935575	1.64E-09	3.80E-08
EXON1	MGC26647	7	88424985	1	cg19682786	0.787220455	0.720656683	0.610087027	-0.177133428	4.98E-06	3.66E-05
EXON1	MGST1	12	16500077	1	cg27117982	0.44279536	0.335651328	0.265778864	-0.177016496	2.16E-11	1.85E-09
EXON1	MIA2	14	39703302	1	cg24603941	0.64484315	0.5140377	0.41682725	-0.2280159	8.05E-09	1.44E-07
EXON1	MLNR	13	49794681/49794635	2	cg02620013/cg10795666	0.345723668	0.416838925	0.499742018	0.154018351	0.000137809	0.000705074
EXON1	MMD2	7	4998714/4998649	2	cg26468007/cg27603283	0.263741423	0.340103578	0.49909633	0.235354907	3.27E-07	3.34E-06
EXON1	MMP12	11	102745682	1	cg20487452	0.61555095	0.455733928	0.3300052	-0.28554575	3.77E-14	3.78E-11
EXON1	MMP16	8	89339609/89339404/89339473/89339622	4	cg02889488/cg11536457/cg16852892/cg26756083	0.08758281	0.157189095	0.309519002	0.221936192	0.000488053	0.002181973
EXON1	MMP7	11	102401438	1	cg25511807	0.37490179	0.281074117	0.208489595	-0.166412195	9.84E-11	4.75E-09

EXON1	MORN4	10	99393217/99393886	2	cg13196796/cg15711987	0.50371698	0.393463672	0.305211082	-0.198505898	1.67E-10	6.94E-09
EXON1	MOS	8	57025881/57025662/57026502/57026301	4	cg10102762/cg15836635/cg22130262/cg22411207	0.268521659	0.375837887	0.489576789	0.221055129	6.58E-08	8.32E-07
EXON1	MOXD1	6	132722421/132722315	2	cg07570142/cg16478774	0.101546219	0.17423656	0.388545952	0.286999733	1.10E-05	7.37E-05
EXON1	MRPS21	1	150266604/150266477/150266643/150266645/150266311/150266488/150266313	7	cg02460371/cg08293086/cg09808343/cg15934674/cg18031008/cg23819679/cg26535484	0.197437178	0.26105832	0.399404685	0.201967507	7.03E-05	0.000386672
EXON1	MS4A10	11	60552841	1	cg07221454	0.608028155	0.520192817	0.453788941	-0.154239214	3.14E-09	6.51E-08
EXON1	MS4A2	11	59856225	1	cg27533472	0.77252679	0.670307089	0.591417223	-0.181109567	2.14E-09	4.73E-08
EXON1	MSC	8	72756155/72756341/72756656/72756058	4	cg09734791/cg14409559/cg23710218/cg25832771	0.208250749	0.380812405	0.516248264	0.307997515	2.36E-07	2.52E-06
EXON1	MST1	3	49726151	1	cg17781925	0.560604335	0.465950656	0.407951073	-0.152653262	3.60E-10	1.18E-08
EXON1	MST1R	3	49941151/49940853/49940126/49940919/49940245/49940353	6	cg03332271/cg08687163/cg11001085/cg11839681/cg18706476/cg23437479	0.735407847	0.547416054	0.432983918	-0.302423929	4.29E-14	3.78E-11
EXON1	MUC7	4	71337873/71337726	2	cg06792975/cg10189763	0.414972255	0.302408433	0.235466747	-0.179505508	2.21E-09	4.86E-08
EXON1	MYH10	17	8534013	1	cg06011292	0.035441597	0.044081865	0.240930607	0.20548901	0.0007458	0.00318264
EXON1	MYL5	4	671791	1	cg17073306	0.415675175	0.343768378	0.262411736	-0.153263439	7.29E-10	2.06E-08
EXON1	MYLK	3	123339334/123339417	2	cg20050484/cg20669834	0.602496535	0.555005994	0.436432773	-0.166063762	8.63E-10	2.34E-08
EXON1	MYOD1	11	17741925/17741243/17741687	3	cg06425919/cg16519321/cg18555440	0.333780485	0.387984559	0.492567606	0.158787121	2.66E-08	3.93E-07
EXON1	MYOT	5	137203640	1	cg04956382	0.507573345	0.394165694	0.307983523	-0.199589822	4.60E-11	2.99E-09
EXON1	NAALAD2	11	89867819/89867911/89867976	3	cg05500015/cg14304817/cg21500966	0.22276217	0.293999184	0.387023888	0.164261718	0.00029068	0.001371888
EXON1	NCAM2	21	22370733/22370864/22370758	3	cg15681537/cg17475987/cg25818292	0.158762917	0.227909182	0.326792251	0.168029334	1.17E-05	7.79E-05
EXON1	NCAN	19	19322818	1	cg08249988	0.38897671	0.513108794	0.684720918	0.295744208	2.42E-10	8.95E-09
EXON1	NDRG4	16	58498151/58498190/58498818	3	cg00984694/cg04797985/cg26824423	0.114577602	0.178434689	0.265996681	0.15141908	0.000111015	0.000580024
EXON1	NELL1	11	20691161/20691180/20691126	3	cg14689623/cg20113732/cg23861668	0.302093187	0.3874827	0.537339132	0.235245945	3.54E-07	3.56E-06

EXON1	NETO1	18	70534767/70534534	2	cg18448581/cg26186727	0.328851298	0.397823596	0.482569505	0.153718207	3.56E-10	1.17E-08
EXON1	NEUROD1	2	182545209	1	cg01897496	0.306702385	0.362163333	0.486254718	0.179552333	7.63E-05	0.000415852
EXON1	NEUROD2	17	37764153/37764158/37764118	3	cg03679521/cg11634198/cg18482164	0.26187175	0.307161628	0.419439552	0.157567802	0.00017892	0.00089352
EXON1	NEUROD4	12	55413878/55413872	2	cg09014354/cg22047387	0.389255158	0.468533778	0.59492828	0.205673122	3.69E-08	5.13E-07
EXON1	NEUROD6	7	31380390	1	cg26312920	0.380424165	0.292083483	0.224579068	-0.155845097	5.07E-10	1.53E-08
EXON1	NEUROG3	10	71333037	1	cg03425110	0.200846189	0.313128656	0.547800477	0.346954289	9.95E-10	2.58E-08
EXON1	NFAM1	22	42828386	1	cg03365751	0.54159879	0.634140517	0.700656695	0.159057905	2.06E-09	4.56E-08
EXON1	NGB	14	77737621/77737495	2	cg02181639/cg02881570	0.375804095	0.441153767	0.623488245	0.24768415	3.50E-07	3.53E-06
EXON1	NIN	14	51290136/51290169	2	cg08189198/cg16449219	0.586387413	0.706010744	0.768622389	0.182234976	5.24E-12	6.87E-10
EXON1	NKAPL	6	28227482/28227392/28227220/28227127/28228206	5	cg00448814/cg02578944/cg10055566/cg18675097/cg18900535	0.584734375	0.669008272	0.738908041	0.154173666	7.71E-10	2.15E-08
EXON1	NKX1-2	10	126138415	1	cg13959280	0.240577635	0.255832856	0.40051425	0.159936615	0.000330041	0.001534124
EXON1	NKX2-2	20	21494084/21494547	2	cg16390060/cg17029168	0.29859384	0.403195008	0.56332042	0.26472658	5.55E-07	5.26E-06
EXON1	NLRP10	11	7984973	1	cg18484189	0.561835355	0.489049094	0.394013105	-0.16782225	5.84E-10	1.70E-08
EXON1	NOS1	12	117799370/117799447	2	cg03538436/cg21273407	0.17058017	0.235782614	0.323966108	0.153385938	2.59E-05	0.000158103
EXON1	NOS2	17	26127480/26127522/26127542/26127537/26127438	5	cg02196147/cg06301529/cg07150830/cg07704981/cg26853415	0.531012951	0.422848119	0.331358986	-0.199653965	1.43E-10	6.26E-09
EXON1	NPAS4	11	66188522/66188745	2	cg14699728/cg22134325	0.61101075	0.731231661	0.824678177	0.213667427	1.72E-11	1.62E-09
EXON1	NPBWR1	8	53853444/53852661/53853112	3	cg02640612/cg06528306/cg21001487	0.391862003	0.454914864	0.620630359	0.228768357	9.58E-10	2.52E-08
EXON1	NPFFR2	4	72897565/72897782/72897868/72897698/72897613	5	cg02192673/cg04008252/cg20289913/cg21266330/cg26656113	0.431398243	0.491396208	0.591193389	0.159795146	3.83E-06	2.91E-05
EXON1	NPHS1	19	36342859/36342725	2	cg04941018/cg19498228	0.620830105	0.515835708	0.425516268	-0.195313837	2.46E-11	1.98E-09
EXON1	NPR3	5	32711881/32712237/32712623	3	cg01252672/cg05839235/cg17630392	0.316180277	0.398280525	0.576090841	0.259910563	1.04E-08	1.78E-07
EXON1	NPY2R	4	156130206/156129862/156130016	3	cg14586939/cg20271517/cg21512644	0.28446994	0.395941781	0.500783826	0.216313885	3.69E-06	2.82E-05
EXON1	NR1I3	1	161207966	1	cg25214346	0.591692035	0.452438228	0.339290932	-0.252401103	3.65E-14	3.78E-11

EXON1	NR2E1	6	108487388/108487506/108487903/108487820	4	cg02062480/cg03383382/cg06559274/cg19697981	0.240879177	0.25947047	0.396833913	0.155954736	2.00E-05	0.000126448
EXON1	NR2E3	15	72103133	1	cg05175964	0.617447225	0.492032528	0.386912323	-0.230534902	4.00E-12	6.05E-10
EXON1	NRG3	10	83635375/83635300	2	cg02690638/cg04529370	0.346941915	0.431284142	0.572608784	0.225666869	1.21E-10	5.44E-09
EXON1	NRXN1	2	50574624/51259493/50574196/50574708/50574516/51259552	6	cg05015185/cg08008931/cg14875171/cg17526573/cg22001533/cg27112565	0.290331083	0.367558091	0.473173299	0.182842216	2.53E-09	5.39E-08
EXON1	NT5C1A	1	40137636	1	cg23045719	0.073181116	0.129791245	0.234315919	0.161134803	0.001024251	0.004232996
EXON1	NTRK3	15	88799523/88799526	2	cg11525479/cg27034819	0.074820707	0.151314393	0.25776619	0.182945484	2.58E-07	2.72E-06
EXON1	NWD1	19	16830859	1	cg21035374	0.46745219	0.38020425	0.290009155	-0.177443035	6.02E-10	1.74E-08
EXON1	NXPH1	7	8473990	1	cg06444755	0.347468845	0.403766956	0.508039186	0.160570341	7.06E-05	0.000388326
EXON1	ODF3	11	196824	1	cg04874875	0.80516377	0.767069428	0.603839582	-0.201324188	1.35E-05	8.87E-05
EXON1	ODZ4	11	79151611	1	cg06892009	0.079381814	0.154169337	0.306221042	0.226839228	0.000206265	0.001013624
EXON1	OLFM4	13	53603157	1	cg24932628	0.615176795	0.434478633	0.355098023	-0.260078772	6.20E-10	1.78E-08
EXON1	OLIG2	21	34398263/34398265	2	cg09793121/cg22869726	0.46519302	0.585147628	0.71594535	0.25075233	1.01E-10	4.78E-09
EXON1	OLIG3	6	137814728/137814078/137815333/137813878/137814356/137814960/137813584/137813648/137814971	9	cg01972751/cg03602029/cg03889226/cg04508701/cg10071643/cg12744820/cg13560452/cg15980797/cg17295225	0.369407105	0.427597218	0.556984729	0.187577624	1.02E-06	9.08E-06
EXON1	ONECUT1	15	53081526/53081248	2	cg14217069/cg17500265	0.36876441	0.425367464	0.599747198	0.230982788	5.58E-08	7.23E-07
EXON1	OPCML	11	133402230/132812991/133402184/133402246	4	cg03923934/cg09314708/cg19151121/cg19349861	0.329729523	0.404297156	0.49141755	0.161688028	6.17E-06	4.43E-05
EXON1	OPRK1	8	54164161/54164051/54164081	3	cg07303244/cg11701471/cg25990647	0.33181452	0.425166318	0.593733085	0.261918565	2.12E-08	3.22E-07
EXON1	OR10A3	11	7960899	1	cg09140665	0.49569433	0.408675017	0.333927714	-0.161766616	1.28E-06	1.12E-05
EXON1	OR10A4	11	6898245	1	cg04456892	0.686775465	0.612365856	0.51522325	-0.171552215	2.23E-05	0.000139127
EXON1	OR10J3	1	159284298/159283536/159284082	3	cg13507224/cg19378892/cg23774016	0.671290432	0.554635069	0.448159027	-0.223131404	1.35E-11	1.39E-09
EXON1	OR10P1	12	56031142	1	cg18770739	0.601520555	0.4993668	0.407141841	-0.194378714	6.81E-10	1.94E-08
EXON1	OR10T2	1	158369112/158368889	2	cg15593510/cg15852352	0.678088725	0.501589039	0.350021123	-0.328067602	4.23E-14	3.78E-11
EXON1	OR10Z1	1	158576809	1	cg20985269	0.64585868	0.498914672	0.3964669	-0.24939178	1.14E-12	2.88E-10

EXON1	OR11H6	14	20692622	1	cg03008515	0.520105455	0.42227725	0.31043085	-0.209674605	3.63E-07	3.63E-06
EXON1	OR13A1	10	45811038	1	cg05877590	0.74817376	0.714776239	0.584376964	-0.163796796	5.40E-06	3.93E-05
EXON1	OR1A2	17	3100972	1	cg16678925	0.622805125	0.538005189	0.426941236	-0.195863889	8.88E-09	1.56E-07
EXON1	OR1G1	17	3030185	1	cg27622610	0.53168163	0.454564772	0.36863775	-0.16304388	5.89E-11	3.49E-09
EXON1	OR1J2	9	125273193	1	cg02644195	0.58050807	0.504360617	0.412877982	-0.167630088	5.04E-11	3.15E-09
EXON1	OR1L3	9	125437568/125438219	2	cg01693284/cg13652536	0.702679435	0.604558789	0.546706259	-0.155973176	0.000182877	0.00091156
EXON1	OR2AK2	1	248129192	1	cg11977100	0.593765145	0.440189311	0.286650286	-0.307114859	2.78E-13	1.14E-10
EXON1	OR2B6	6	27925166	1	cg03340878	0.684246695	0.597933917	0.519217023	-0.165029672	0.000300251	0.001411021
EXON1	OR2D2	11	6913644	1	cg00243661	0.75473994	0.635957867	0.559619082	-0.195120858	5.02E-06	3.68E-05
EXON1	OR2G3	1	247769804/247769251	2	cg03618016/cg15231462	0.74263449	0.700470306	0.58910078	-0.15353371	1.95E-05	0.000123903
EXON1	OR2H1	6	29426320	1	cg13680362	0.66452185	0.557526567	0.472516695	-0.192005155	1.67E-05	0.000107941
EXON1	OR2M2	1	248343651/248343317	2	cg00073384/cg20812354	0.72903652	0.647914311	0.5447131	-0.18432342	1.51E-09	3.58E-08
EXON1	OR2M3	1	248366399/248366883	2	cg05353872/cg07598464	0.687784385	0.588039617	0.455329084	-0.232455301	1.56E-07	1.76E-06
EXON1	OR2M5	1	248308934/248308813	2	cg02332123/cg04283307	0.652761025	0.561371367	0.460385391	-0.192375634	3.19E-09	6.57E-08
EXON1	OR2T33	1	248436202	1	cg16126058	0.366782645	0.285497222	0.210676073	-0.156106572	1.27E-11	1.32E-09
EXON1	OR2T4	1	248525007	1	cg07483650	0.366023425	0.278007917	0.196313532	-0.169709893	9.88E-14	5.47E-11
EXON1	OR2T6	1	248551173/248551409	2	cg00883837/cg20465661	0.63138572	0.5134356	0.385281855	-0.246103865	1.87E-09	4.24E-08
EXON1	OR2W1	6	29012859/29012352/29012588/29012269	4	cg01947943/cg02023215/cg07053841/cg12081389	0.747435905	0.669465578	0.556029711	-0.191406194	5.17E-07	4.93E-06
EXON1	OR4C12	11	50003963	1	cg15905666	0.698601595	0.596263256	0.465559855	-0.23304174	1.48E-05	9.71E-05
EXON1	OR4K17	14	20585753/20586367	2	cg04588710/cg10836431	0.539907743	0.457262717	0.375232505	-0.164675238	1.03E-07	1.23E-06
EXON1	OR51B4	11	5322976	1	cg06353345	0.64987143	0.514448006	0.319087886	-0.330783544	8.21E-14	5.43E-11
EXON1	OR51B6	11	5372944	1	cg00423453	0.5330523	0.391589678	0.268986232	-0.264066068	1.52E-14	3.38E-11
EXON1	OR51I1	11	5462540	1	cg04874562	0.553066405	0.445523361	0.34538165	-0.207684755	1.36E-13	7.19E-11
EXON1	OR51L1	11	5020582	1	cg06866232	0.44972989	0.390325183	0.295179773	-0.154550117	3.54E-08	4.97E-07
EXON1	OR52A4	11	5142602	1	cg02211735	0.45781345	0.343789278	0.226432155	-0.231381295	3.32E-12	5.38E-10
EXON1	OR52B2	11	6191109/6191393	2	cg05005432/cg16309319	0.726049495	0.662184994	0.564208095	-0.1618414	9.90E-05	0.000523442
EXON1	OR52B6	11	5602123	1	cg00294684	0.77197956	0.691746872	0.553328591	-0.218650969	6.37E-06	4.54E-05
EXON1	OR52D1	11	5510396/5510732	2	cg04956485/cg12860254	0.84537203	0.797214739	0.663536245	-0.181835785	5.97E-05	0.000334292
EXON1	OR52J3	11	5067927	1	cg17379345	0.74748444	0.630067617	0.547998545	-0.199485895	2.91E-07	3.03E-06
EXON1	OR52N1	11	5809911	1	cg10440696	0.63821711	0.563754844	0.425155482	-0.213061628	4.10E-06	3.08E-05
EXON1	OR52N2	11	5841688	1	cg05703009	0.611918655	0.5406584	0.422179595	-0.18973906	1.20E-08	2.00E-07

EXON1	OR56A1	11	6048685/6047917	2	cg01242426/cg12415917	0.706553545	0.62847565	0.521433182	-0.185120363	7.66E-11	4.12E-09
EXON1	OR56B4	11	6129548/6129408	2	cg10092198/cg25678587	0.547243855	0.436509525	0.312700455	-0.2345434	2.12E-10	8.18E-09
EXON1	OR5AR1	11	56431525	1	cg14029856	0.635247195	0.581912356	0.466349745	-0.16889745	1.64E-05	0.000106077
EXON1	OR5AS1	11	55798206	1	cg14569837	0.724193485	0.661185767	0.553617986	-0.170575499	1.61E-05	0.000104769
EXON1	OR5H14	3	97868656	1	cg15154635	0.638656275	0.574328578	0.477130614	-0.161525661	2.18E-07	2.35E-06
EXON1	OR5H6	3	97983540/97983253	2	cg05232914/cg20367065	0.777116725	0.671791272	0.590553909	-0.186562816	5.01E-06	3.68E-05
EXON1	OR5L2	11	55594854	1	cg02459415	0.67929876	0.598909694	0.455660786	-0.223637974	3.58E-07	3.59E-06
EXON1	OR5T1	11	56043514	1	cg27149471	0.704785315	0.61706415	0.498464873	-0.206320442	3.91E-06	2.96E-05
EXON1	OR6C65	12	55794783	1	cg25236050	0.674340085	0.570522283	0.42028155	-0.254058535	1.44E-07	1.65E-06
EXON1	OR6K3	1	158687431	1	cg20268857	0.71700447	0.638179611	0.530855236	-0.186149234	1.84E-05	0.000117861
EXON1	OR6N1	1	158736112/158736312	2	cg04972930/cg24381587	0.712903723	0.629850064	0.553657575	-0.159246148	5.00E-13	1.74E-10
EXON1	OR7A17	19	14991752/14992047	2	cg12506013/cg20792256	0.30965707	0.2240131	0.151004445	-0.158652625	1.43E-13	7.27E-11
EXON1	OR8K3	11	56086656	1	cg27314580	0.701372765	0.610796761	0.501831082	-0.199541683	8.01E-07	7.29E-06
EXON1	OR8K5	11	55927697	1	cg11371231	0.636005695	0.531065117	0.396211641	-0.239794054	4.95E-09	9.58E-08
EXON1	OR8S1	12	48919444/48919471	2	cg01881778/cg27104671	0.70300426	0.651432325	0.54836763	-0.15463663	2.53E-05	0.00015525
EXON1	OR9K2	12	55524042	1	cg13860283	0.445972335	0.366307611	0.292927618	-0.153044717	2.97E-08	4.30E-07
EXON1	OR9Q1	11	57791564	1	cg03034198	0.750499935	0.57641685	0.519473877	-0.231026058	5.81E-10	1.70E-08
EXON1	OSMR	5	38846034/38846475/38846100	3	cg02390103/cg05485663/cg17528648	0.291284015	0.350370437	0.449132152	0.157848137	4.27E-07	4.19E-06
EXON1	OTOL1	3	161214690/161214813	2	cg01015123/cg13212525	0.556621783	0.409018661	0.325907804	-0.230713979	7.41E-10	2.09E-08
EXON1	OTOS	2	241080057	1	cg19697911	0.463261025	0.392938306	0.308255473	-0.155005552	1.75E-10	7.11E-09
EXON1	OTP	5	76934461/76934501	2	cg21097283/cg24231716	0.199478577	0.271493168	0.380937562	0.181458985	6.10E-07	5.71E-06
EXON1	P2RY12	3	151058527	1	cg03379681	0.5499868	0.474373656	0.373792782	-0.176194018	8.55E-06	5.93E-05
EXON1	PAK7	20	9819467	1	cg18034428	0.34452928	0.323443256	0.515732564	0.171203284	3.84E-05	0.000224733
EXON1	PANX3	11	124481614	1	cg08191915	0.683063245	0.60932475	0.476780723	-0.206282522	3.72E-09	7.53E-08
EXON1	PAPPA2	1	176432498	1	cg10994126	0.47482309	0.359825356	0.271975341	-0.202847749	9.33E-12	1.07E-09
EXON1	PAQR5	15	69591355/69591357/69606770	3	cg00225576/cg13117105/cg25963511	0.541098615	0.4359517	0.36547975	-0.175618865	3.66E-09	7.41E-08
EXON1	PARVB	22	44420316	1	cg20991347	0.128513228	0.199922744	0.374301399	0.245788171	8.35E-05	0.000450291
EXON1	PATE3	11	125658038	1	cg09752271	0.421375365	0.327739161	0.242760424	-0.178614941	6.08E-07	5.69E-06
EXON1	PAX7	1	18957632/18958084	2	cg11428724/cg13969001	0.182770352	0.194844565	0.339914824	0.157144472	0.003890899	0.014009989

EXON1	PCDH10	4	134073627/134071663/134072931/134072237/134070598/134070819/134072723/134074369	8	cg06667761/cg09221867/cg09693004/cg09960109/cg14410319/cg14795750/cg17504999/cg23713176	0.325112394	0.421639558	0.558368096	0.233255702	4.12E-10	1.30E-08
EXON1	PCDH17	13	58206058/58208929/58208350/58207013/58209133/58207058/58206036/58207650/58207859	9	cg00208153/cg04413320/cg07921625/cg11312408/cg12432236/cg14427009/cg14893163/cg22840780/cg26444528	0.443761173	0.551015106	0.674702436	0.230941263	1.82E-10	7.25E-09
EXON1	PCDHGA11	5	140802831/140803130/140802804/140800586/140800929/140802135/140800983/140800629/140801286/140801354/140802669/140801482/140800761/140802432	14	cg01729977/cg02715006/cg04845915/cg05870739/cg06572465/cg07179872/cg08395122/cg10679597/cg13598256/cg13718539/cg21928406/cg22175873/cg24954895/cg26485937	0.50997514	0.610009547	0.70583027	0.195855129	4.39E-10	1.36E-08
EXON1	PCDHGA12	5	140811520/140810404/140811253/140811642/140811312/140810260/140810920/140812464/140810161/140810726/140812558/140810433/140810805/140811102/140810562/140812236	16	cg01224715/cg02267483/cg04080041/cg07489502/cg07539798/cg07802710/cg09639151/cg10805220/cg11647681/cg14253517/cg19294953/cg21185686/cg22151446/cg23244790/cg24576050/cg26375473	0.52913674	0.625404835	0.713070255	0.183933515	1.02E-09	2.62E-08
EXON1	PCDHGB7	5	140798095/140798758/140799811/140798188/140798161/140797509/140799290/140799572/140797521/140798971/140797286/140799305	12	cg02331883/cg02707176/cg03456338/cg04503600/cg08935238/cg08938584/cg13951490/cg15874877/cg17011276/cg18297751/cg23563234/cg27630202	0.590398071	0.660112676	0.744720048	0.154321977	1.58E-09	3.70E-08

EXON1	PCDHGC3	5	140857995/140856330/140856469/140855857/140857813/140857474/140855582/140857192/140857941/140857889/140858046/140856971/140857647/140858107	14	cg00589850/cg04453180/cg07445963/cg07448606/cg08155325/cg09581226/cg10858746/cg12877039/cg14672084/cg18858331/cg24024424/cg24942747/cg25600472/cg27639030	0.39986379	0.488760097	0.62326545	0.223401661	1.10E-10	5.12E-09
EXON1	PCDHGC4	5	140865433/140864834	2	cg11830096/cg12145907	0.590769333	0.696767328	0.800635389	0.209866056	2.79E-13	1.14E-10
EXON1	PCK1	20	56136167	1	cg20605413	0.399125295	0.314853322	0.242899114	-0.156226181	2.25E-10	8.47E-09
EXON1	PCOLCE	7	100200009/100199982/100199952/100200003	4	cg02797569/cg06402330/cg26777475/cg27069566	0.633549886	0.575186529	0.459504847	-0.17404504	1.90E-08	2.92E-07
EXON1	PCP4L1	1	161228674	1	cg11912330	0.16781842	0.160256398	0.392305085	0.224486665	0.000207649	0.001019291
EXON1	PDE1B	12	54955143/54943545/54943484	3	cg01961252/cg15927682/cg24020814	0.248393013	0.331568553	0.422807558	0.174414545	8.91E-06	6.15E-05
EXON1	PDE8B	5	76506759/76507050	2	cg08311610/cg18089852	0.204729229	0.324509538	0.441491449	0.23676222	5.09E-07	4.88E-06
EXON1	PDGFD	11	104034754/104034619/104034982	3	cg02120582/cg07748540/cg18403606	0.067299898	0.215763276	0.412432378	0.34513248	2.09E-07	2.26E-06
EXON1	PDPN	1	13912116/13910555/13910569	3	cg17826518/cg18877506/cg21819468	0.398049335	0.512292985	0.623260467	0.225211132	9.24E-08	1.12E-06
EXON1	PDZRN4	12	41831558/41582362/41582349	3	cg15417244/cg16896079/cg21926708	0.448558728	0.532102548	0.641976517	0.193417788	3.43E-14	3.78E-11
EXON1	PENK	8	57358505/57359200/57359258/57358590	4	cg04598121/cg06066137/cg10397440/cg21694941	0.409083774	0.481465306	0.587195616	0.178111842	1.54E-11	1.50E-09
EXON1	PEX5L	3	179754483	1	cg03175305	0.176515565	0.282426469	0.423390118	0.246874553	1.27E-07	1.47E-06
EXON1	PHOX2A	11	71955113/71954982/71955164	3	cg13103303/cg18722841/cg19651694	0.354161368	0.423400856	0.581726418	0.227565051	8.43E-10	2.32E-08
EXON1	PIK3R6	17	8770954/8770908	2	cg09209669/cg24092340	0.394042978	0.273141422	0.181220932	-0.212822046	8.93E-11	4.49E-09
EXON1	PIWIL3	22	25170371	1	cg07971797	0.833815295	0.796256428	0.667576014	-0.166239281	0.000207121	0.001017456
EXON1	PKD2L2	5	137225191	1	cg21051519	0.33712707	0.471419183	0.552794859	0.215667789	8.53E-07	7.70E-06
EXON1	PKIA	8	79428444/79428569/79428434	3	cg09043127/cg12330330/cg27140220	0.062668109	0.138695383	0.293404026	0.230735917	4.75E-05	0.00027216
EXON1	PLA2G4F	15	42448786/42448760	2	cg10917153/cg16920001	0.482703	0.393574192	0.321426993	-0.161276007	7.69E-11	4.12E-09

EXON1	PLCZ1	12	18890872	1	cg06233731	0.659154945	0.580735178	0.465161123	-0.193993822	2.34E-07	2.50E-06
EXON1	PLSCR5	3	146323078	1	cg22594071	0.469473455	0.390588256	0.302888591	-0.166584864	9.13E-10	2.45E-08
EXON1	POSTN	13	38172803/38172876	2	cg04922971/cg24719107	0.499432845	0.395833544	0.289274882	-0.210157963	1.49E-08	2.39E-07
EXON1	POU3F1	1	38512238/38509934/38510933/38512250/38510074/38511455/38511677/38512230/38511105/38510522/38510383/38510182	12	cg05336094/cg05785481/cg08599259/cg12714180/cg13149116/cg13193455/cg18451114/cg18755829/cg19769760/cg22821908/cg25488697/cg25609528	0.173647221	0.263071241	0.398562127	0.224914906	3.56E-09	7.24E-08
EXON1	POU4F2	4	147560282/147560286/147560126	3	cg02610222/cg14631165/cg24199834	0.322328865	0.391729356	0.513645348	0.191316483	1.32E-10	5.85E-09
EXON1	POU4F3	5	145718669/145718597	2	cg17871749/cg23303408	0.331677935	0.393681303	0.499733048	0.168055113	1.26E-06	1.10E-05
EXON1	PPAPDC1A	10	122216848	1	cg17087331	0.225069555	0.180014741	0.390885319	0.165815764	0.002345204	0.0088896
EXON1	PPFIA4	1	203020566/203020617/203020660	3	cg00594917/cg10952234/cg24000087	0.729849245	0.657511617	0.57815983	-0.151689415	1.56E-06	1.31E-05
EXON1	PPP1R14D	15	41120711	1	cg04968426	0.49174356	0.384359917	0.314411773	-0.177331787	7.07E-11	3.93E-09
EXON1	PPP1R16B	20	37434409	1	cg22128431	0.071897924	0.171597465	0.302441241	0.230543317	0.000224216	0.0010913
EXON1	PPP1R3A	7	113558794	1	cg00967316	0.771687275	0.687066378	0.567661268	-0.204026007	8.43E-07	7.63E-06
EXON1	PPP2R2B	5	146460987/146257653/146258034/146258354/146258189/146258181/146258195/146258237/146258159/146435656/146257862/146258427	12	cg01112778/cg03225002/cg03693911/cg03851984/cg04583285/cg08774231/cg09528265/cg11826826/cg14693112/cg16617774/cg21938261/cg25149751	0.220000274	0.269256711	0.382457051	0.162456777	4.17E-05	0.000242344
EXON1	PPP2R2C	4	6473964	1	cg18013519	0.07588347	0.151003771	0.353179632	0.277296162	4.14E-06	3.10E-05
EXON1	PPYR1	10	47083632	1	cg06939941	0.568657925	0.670370039	0.744070564	0.175412639	2.16E-12	4.14E-10
EXON1	PRAMEF12	1	12835245	1	cg13394110	0.711959785	0.618704606	0.525610973	-0.186348812	1.98E-05	0.000125078
EXON1	PRAMEF21	1	13522252	1	cg11999044	0.514005275	0.420667506	0.3360326	-0.177972675	1.85E-11	1.71E-09
EXON1	PRDM11	11	45115584	1	cg03470671	0.812032445	0.748265267	0.606485836	-0.205546609	3.24E-06	2.51E-05
EXON1	PRDM12	9	133540108	1	cg09191327	0.17442775	0.232800424	0.383041491	0.208613741	0.000100592	0.000531018
EXON1	PRELP	1	203444945	1	cg07947930	0.451737085	0.389516283	0.297888855	-0.15384823	3.47E-08	4.90E-07
EXON1	PREX1	20	47444241	1	cg03361585	0.100361965	0.269619792	0.393830719	0.293468755	6.94E-08	8.72E-07

EXON1	PREX2	8	68864769	1	cg06617456	0.31326061	0.437296561	0.573209405	0.259948795	1.38E-10	6.07E-09
EXON1	PRG4	1	186265423	1	cg24669741	0.49889375	0.420284861	0.317732418	-0.181161332	2.71E-10	9.76E-09
EXON1	PRLHR	10	120355139	1	cg16196812	0.49566566	0.589193594	0.70521705	0.20955139	2.07E-11	1.82E-09
EXON1	PRMT8	12	3600764/3600666	2	cg11596580/cg23739862	0.27294165	0.299425	0.423234441	0.150292791	4.65E-05	0.000267107
EXON1	PRND	20	4702581	1	cg09906458	0.66278959	0.504456711	0.349004655	-0.313784935	1.68E-14	3.38E-11
EXON1	PROCA1	17	27038861/27038664/27038686	3	cg02685896/cg19462352/cg24841318	0.13259097	0.230131542	0.326429853	0.193838883	1.43E-06	1.22E-05
EXON1	PRODH2	19	36304079	1	cg18640606	0.73577225	0.673229444	0.501053945	-0.234718305	1.72E-07	1.91E-06
EXON1	PROM2	2	95940475	1	cg25241038	0.64004414	0.558917589	0.486534114	-0.153510026	1.76E-08	2.73E-07
EXON1	PRR4	12	11324142/11002071/11324180	3	cg21216543/cg23272369/cg26983578	0.813557555	0.652031544	0.578963895	-0.23459366	2.60E-08	3.84E-07
EXON1	PRSS27	16	2770211/2770429/2770336/2770298	4	cg01195196/cg05144884/cg12391048/cg16444062	0.74622224	0.619735257	0.552289117	-0.193933123	2.05E-07	2.23E-06
EXON1	PTGFR	1	78956905/78956845/78956847	3	cg03495868/cg03949391/cg27046936	0.393904195	0.462070296	0.596562179	0.202657984	2.50E-11	1.98E-09
EXON1	PTH2R	2	209271661/209271785	2	cg06391199/cg24769821	0.27094058	0.304544699	0.461541468	0.190600888	0.000465446	0.002092078
EXON1	PTPN5	11	18813191	1	cg11334818	0.316661295	0.431670789	0.507937245	0.19127595	0.000132911	0.000682691
EXON1	PTPRT	20	41818358/41818356	2	cg03403065/cg13168820	0.191056606	0.262741759	0.392869729	0.201813123	3.16E-09	6.53E-08
EXON1	PVRL4	1	161059267/161059327	2	cg10841756/cg22585988	0.561919435	0.461592733	0.371334232	-0.190585203	8.87E-11	4.48E-09
EXON1	PXDN	2	1748112/1748132	2	cg12164282/cg25181651	0.332990048	0.383183358	0.483251011	0.150260964	0.000612782	0.002671881
EXON1	PYDC1	16	31228299/31228202/31228263	3	cg08346922/cg10038009/cg13352836	0.350772645	0.447509511	0.65483555	0.304062905	2.23E-10	8.45E-09
EXON1	PYDC2	3	191179074	1	cg16739580	0.702488185	0.609279044	0.5201291	-0.182359085	3.11E-05	0.000186192
EXON1	PYGO1	15	55881044	1	cg25902187	0.173664853	0.357517393	0.522236799	0.348571946	5.07E-06	3.72E-05
EXON1	PZP	12	9360854/9360933	2	cg01714932/cg10479063	0.698024228	0.623041672	0.517973932	-0.180050296	2.63E-09	5.58E-08
EXON1	QKI	6	163836348/163836245	2	cg06844545/cg09339301	0.174056859	0.207228581	0.360244865	0.186188007	0.000154318	0.000781275
EXON1	QRFPR	4	122302007/122301724/122301573/122301996/122301816/122301740	6	cg00015770/cg06991300/cg10114327/cg14012124/cg16326674/cg19971716	0.307967968	0.396433607	0.5574158	0.249447832	2.25E-08	3.40E-07
EXON1	RAB11FIP4	17	29719023	1	cg12419135	0.41374687	0.474749556	0.604057318	0.190310448	0.00017538	0.000877164
EXON1	RAB17	2	238499464	1	cg01157280	0.594524595	0.492280311	0.398829073	-0.195695522	3.57E-10	1.17E-08
EXON1	RAB25	1	156031182	1	cg19580810	0.566289525	0.4548067	0.357817941	-0.208471584	2.48E-10	9.08E-09

EXON1	RAB34	17	27044745/27045176/27044322/27044685/27044629/27045164	6	cg02530437/cg08032476/cg12873610/cg18686527/cg21816330/cg22803868	0.489640619	0.592465949	0.690550013	0.200909394	2.26E-11	1.91E-09
EXON1	RALYL	8	85096037/85095498/85097246/85097157/85096015/85095654/85097195/85095697	8	cg00034076/cg05716166/cg17215863/cg18016194/cg22403811/cg23777958/cg25757598/cg26669044	0.368062837	0.414865296	0.604808588	0.236745751	1.61E-08	2.54E-07
EXON1	RAMP3	7	45197441	1	cg14999001	0.24034283	0.418481828	0.540330959	0.299988129	4.36E-08	5.86E-07
EXON1	RAPSN	11	47470633/47470674	2	cg01947936/cg15679098	0.77149761	0.72687405	0.617255845	-0.154241765	1.52E-05	9.93E-05
EXON1	RASGRF1	15	79382995/79382708/79382767/79382998/79382807/79383167/79297210/79297159	8	cg14510404/cg14984684/cg16154416/cg17507573/cg20574415/cg20973720/cg21840434/cg24739098	0.287670839	0.384281087	0.493672307	0.206001468	5.23E-08	6.82E-07
EXON1	RAX	18	56940418/56940463/56940333	3	cg00397673/cg05945059/cg06676637	0.473504282	0.529920683	0.633184589	0.159680308	3.04E-10	1.06E-08
EXON1	RCN3	19	50031246/50031273/50031017	3	cg19403104/cg19808978/cg21941030	0.566667185	0.676641842	0.742609445	0.17594226	4.17E-10	1.32E-08
EXON1	RCSD1	1	167599527/167599631	2	cg00450304/cg02079933	0.026154343	0.066027011	0.212225592	0.186071249	0.00069075	0.00297129
EXON1	RDH5	12	56114155/56114269	2	cg02192520/cg17243643	0.52343967	0.430334986	0.336311564	-0.187128106	9.38E-10	2.49E-08
EXON1	REG4	1	120354159	1	cg00808492	0.575735325	0.437262439	0.328892459	-0.246842866	3.19E-11	2.36E-09
EXON1	RELN	7	103629618	1	cg17923358	0.267672155	0.337602217	0.448984718	0.181312563	0.000404414	0.001843556
EXON1	REM1	20	30063129	1	cg12153542	0.22427104	0.335859228	0.380584059	0.156313019	0.000516776	0.002294872
EXON1	REP15	12	27849452	1	cg07809176	0.483809695	0.390924644	0.323047568	-0.160762127	3.58E-08	5.02E-07
EXON1	RERG	12	15374175/15374303	2	cg08830758/cg24979348	0.067929305	0.09755375	0.263818665	0.19588936	0.000792502	0.003363494
EXON1	RGS12	4	3371928/3371876/3315925	3	cg09921821/cg19974227/cg24466927	0.477248238	0.549716931	0.656136598	0.17888836	2.03E-09	4.54E-08
EXON1	RGS7BP	5	63802593/63802491	2	cg00750523/cg18388380	0.187152259	0.331437807	0.507750723	0.320598464	4.37E-06	3.26E-05
EXON1	RHAG	6	49604442	1	cg23172892	0.525934335	0.397075233	0.287293968	-0.238640367	3.58E-11	2.52E-09
EXON1	RIC3	11	8190448/8190565/8190572	3	cg03707776/cg08383315/cg25778535	0.292124702	0.424474943	0.539278052	0.24715335	8.79E-11	4.46E-09
EXON1	RICS	11	128893940/128893851	2	cg03231860/cg20892287	0.463703378	0.359730039	0.284669923	-0.179033455	3.32E-11	2.38E-09
EXON1	RLBP1	15	89764836/89764895	2	cg10173075/cg15808008	0.640042538	0.455113978	0.3678254	-0.272217138	2.52E-13	1.11E-10

EXON1	RNF150	4	142053720/142054417/142053623/142054329/142054254/142054446/142053970	7	cg03112087/cg03563667/cg05212802/cg06340552/cg18120376/cg20599022/cg24053721	0.180049123	0.266463777	0.399462806	0.219413683	1.66E-05	0.000107451
EXON1	RNF175	4	154681066/154681197/154681323/154681128	4	cg01440841/cg02647878/cg08660876/cg18355902	0.295499914	0.337412067	0.46049854	0.164998626	0.000599655	0.002618099
EXON1	RNF186	1	20141662/20140828	2	cg04994456/cg25096740	0.54771735	0.454155589	0.395728416	-0.151988934	1.23E-09	3.05E-08
EXON1	RNF222	17	8301118	1	cg12920094	0.67082034	0.573779922	0.461031095	-0.209789245	1.45E-12	3.13E-10
EXON1	RNF43	17	56494554	1	cg24835159	0.492136535	0.405239378	0.324541164	-0.167595371	2.54E-09	5.41E-08
EXON1	RORB	9	77112326/77112896	2	cg07536920/cg14170313	0.128788008	0.182273709	0.344570812	0.215782804	2.67E-05	0.00016268
EXON1	RSPO2	8	109095568/109095572/109095782	3	cg00910695/cg04050867/cg14733048	0.274932993	0.351034819	0.527101124	0.252168131	1.80E-09	4.10E-08
EXON1	RSPO3	6	127440413/127440104	2	cg07279933/cg09979256	0.117421047	0.22518447	0.402175854	0.284754807	6.38E-06	4.55E-05
EXON1	RSPO4	20	982831/982768	2	cg01188592/cg22709307	0.303446555	0.340147172	0.454667214	0.151220659	0.000408172	0.001857484
EXON1	RXFP3	5	33937182/33936752/33936520/33936597/33938213/33938225/33937673/33937876	8	cg02305687/cg02341815/cg02640041/cg03909500/cg05574272/cg07917609/cg10350492/cg18294646	0.471641434	0.542159039	0.633953488	0.162312054	1.07E-08	1.83E-07
EXON1	RXFP4	1	155912569	1	cg27187375	0.610697645	0.504840306	0.414932432	-0.195765213	2.16E-10	8.26E-09
EXON1	RXRG	1	165414189/165414332/165414379	3	cg00349776/cg08905496/cg10364513	0.201971838	0.243573256	0.353013446	0.151041608	0.001049961	0.004324387
EXON1	RYR1	19	38924347/38924349/38924436	3	cg04571321/cg05471297/cg06740600	0.19628607	0.287594851	0.464376086	0.268090016	1.11E-05	7.46E-05
EXON1	S100A14	1	153588782	1	cg25433648	0.517648555	0.412206472	0.3245839	-0.193064655	9.46E-11	4.69E-09
EXON1	S100P	4	6695698/6695614	2	cg22266967/cg26233331	0.51050012	0.402098875	0.304532536	-0.205967584	1.48E-12	3.13E-10
EXON1	S1PR1	1	101702519/101702602	2	cg04156369/cg18323466	0.103536975	0.214787156	0.40288088	0.299343905	7.74E-05	0.000420982
EXON1	SAA4	11	18258323	1	cg22587758	0.639549715	0.497548717	0.374011736	-0.265537979	8.87E-10	2.39E-08
EXON1	SALL1	16	51184392/51185147/51184355/51184379/51185132/51185082/51185110	7	cg00582524/cg01146232/cg04550052/cg04698114/cg06232807/cg06653699/cg09016242	0.465916713	0.597657404	0.716352588	0.250435875	1.43E-09	3.43E-08

EXON1	SARM1	17	26699187/26699169/26699485	3	cg05854644/cg15760257/cg20686234	0.13072045	0.295548424	0.409477434	0.278756984	2.12E-06	1.72E-05
EXON1	SCGB1D1	11	61957732/61957788	2	cg07532354/cg13916742	0.503236883	0.418792739	0.343602141	-0.159634742	1.12E-09	2.82E-08
EXON1	SCGB2A1	11	61976195/61976174	2	cg06334737/cg16986846	0.56752183	0.431454211	0.326036145	-0.241485685	5.46E-11	3.35E-09
EXON1	SCGB2A2	11	62037659	1	cg22862656	0.81272038	0.609554289	0.447701014	-0.365019366	1.48E-11	1.45E-09
EXON1	SCN2B	11	118047203/118047121	2	cg10513118/cg13348944	0.491962675	0.402305178	0.320984305	-0.17097837	4.66E-08	6.20E-07
EXON1	SCNN1A	12	6483772/6484729/6483708/6483739/6484219/6484713	6	cg00773370/cg11785537/cg16048383/cg18738906/cg23245485/cg25098281	0.540871468	0.444526764	0.371125275	-0.169746193	1.01E-10	4.78E-09
EXON1	SCTR	2	120281999/120281813	2	cg07176264/cg20505223	0.287517405	0.314268917	0.480911932	0.193394527	4.00E-05	0.000233461
EXON1	SDC2	8	97506180/97506251	2	cg14538332/cg16935295	0.12978423	0.237232389	0.44108355	0.31129932	3.81E-06	2.90E-05
EXON1	SDR16C6	8	57307649	1	cg05553829	0.481092895	0.391468678	0.318698386	-0.162394509	1.47E-09	3.51E-08
EXON1	SELENBP1	1	151345123	1	cg24480379	0.490887215	0.421913322	0.335055195	-0.15583202	1.12E-08	1.89E-07
EXON1	SELV	19	40006451/40005836/40005812/40006292/40006192/40006625/40005758	7	cg00452257/cg04736260/cg11396791/cg12225910/cg12562967/cg21342437/cg22961275	0.175127779	0.239213195	0.379485826	0.204358047	3.39E-06	2.62E-05
EXON1	SERINC4	15	44092166	1	cg02556940	0.86925858	0.809470183	0.6736888	-0.19556978	1.03E-05	6.98E-05
EXON1	SERPINA1	14	94855034/94856984/94855088/94855099	4	cg02126235/cg02181506/cg05346611/cg22828990	0.728270755	0.525884633	0.3817073	-0.346563455	5.33E-15	2.35E-11
EXON1	SERPINB5	18	61144250/61144177/61144150	3	cg08411049/cg20837735/cg21807034	0.514257392	0.4132338	0.328815711	-0.185441681	1.06E-09	2.70E-08
EXON1	SERPINB7	18	61420334	1	cg26646554	0.621149885	0.534320311	0.470877209	-0.150272676	1.83E-08	2.83E-07
EXON1	SFMBT2	10	7453329	1	cg20506184	0.156079529	0.405638556	0.595730313	0.439650784	2.86E-08	4.18E-07
EXON1	SFRP1	8	41166169/41166530/41166738	3	cg13398291/cg15839448/cg22418909	0.372899918	0.444137375	0.558119745	0.185219828	1.71E-07	1.91E-06
EXON1	SFRP2	4	154709756/154709828/154710224/154709878	4	cg05774801/cg05874561/cg06549216/cg14063488	0.170399716	0.233315581	0.360160385	0.18976067	0.000943009	0.003937835
EXON1	SFTA3	14	36982649/36982447	2	cg01611862/cg04926361	0.306812683	0.387726467	0.485149523	0.17833684	1.33E-07	1.54E-06
EXON1	SGCZ	8	15095163	1	cg16266667	0.35641897	0.505215806	0.654992595	0.298573625	4.45E-09	8.81E-08

EXON1	SH2D4B	10	82297793/82300600/82297920/82300588	4	cg08772206/cg10374402/cg10859151/cg22633936	0.667622097	0.592609963	0.515918545	-0.151703551	1.91E-11	1.75E-09
EXON1	SH3GL3	15	84116151/84116115/84116107	3	cg09201151/cg21145136/cg22946150	0.231795257	0.242610657	0.400988149	0.169192892	0.002187862	0.008369932
EXON1	SHC3	9	91793648/91793365/91793192	3	cg13351583/cg14048780/cg17806889	0.193664948	0.247666081	0.35787482	0.164209872	0.000556702	0.002453235
EXON1	SHISA9	16	12996338/12996234/12995568/12995603/12996046/12996419/12996057	7	cg01341170/cg01705052/cg02482218/cg04106647/cg04557544/cg04613834/cg18578405	0.180185421	0.229827183	0.364016007	0.183830586	9.78E-06	6.65E-05
EXON1	SIAH3	13	46425712/46425837	2	cg00613224/cg02674384	0.255779335	0.338534439	0.470898677	0.215119342	6.54E-07	6.10E-06
EXON1	SIGLEC6	19	52034861/52034970	2	cg12008991/cg16293105	0.318704997	0.3756759	0.471545377	0.15284038	3.05E-05	0.000183526
EXON1	SIRPG	20	1638393	1	cg15722679	0.39737777	0.292677606	0.216841866	-0.180535904	4.52E-07	4.39E-06
EXON1	SIX6	14	60975964/60976115/60976285	3	cg06785999/cg14186066/cg19456540	0.39678214	0.533290539	0.68257002	0.28578788	1.63E-10	6.85E-09
EXON1	SLC11A1	2	219246985/219247055	2	cg05007997/cg18854666	0.626903165	0.502097061	0.37951783	-0.247385335	9.88E-11	4.75E-09
EXON1	SLC12A5	20	44657838/44650449/44657948/44650380	4	cg10439765/cg14921743/cg22752533/cg23720732	0.271350453	0.347029448	0.435984983	0.16463453	1.47E-06	1.25E-05
EXON1	SLC13A5	17	6616653/6616644	2	cg16652063/cg24970327	0.397946898	0.528784761	0.626882582	0.228935684	6.45E-08	8.18E-07
EXON1	SLC14A2	18	43195041	1	cg22207479	0.59877399	0.498156444	0.4311451	-0.16762889	9.03E-12	1.05E-09
EXON1	SLC18A3	10	50820278/50820604/50818911/50818652/50819500/50818707	6	cg01295399/cg07637213/cg14008883/cg16494192/cg20457796/cg24109980	0.459676352	0.523295926	0.622336302	0.162659951	3.93E-08	5.41E-07
EXON1	SLC22A10	11	63057478/63057592	2	cg01306183/cg16351441	0.60468788	0.479163578	0.355024259	-0.249663621	3.71E-10	1.20E-08
EXON1	SLC22A16	6	110797797	1	cg05415020	0.42231626	0.479833089	0.615592823	0.193276563	9.92E-09	1.72E-07
EXON1	SLC22A24	11	62911616	1	cg16950247	0.52666063	0.380126061	0.307141736	-0.219518894	1.83E-13	8.62E-11
EXON1	SLC23A1	5	138718914	1	cg21631086	0.51746788	0.419325183	0.351424255	-0.166043625	2.49E-11	1.98E-09
EXON1	SLC23A3	2	220034739	1	cg15797218	0.56123755	0.462417461	0.394757336	-0.166480214	3.45E-11	2.47E-09
EXON1	SLC24A4	14	92788960/92790285	2	cg01064918/cg18229521	0.370237775	0.463126767	0.521846393	0.151608618	1.66E-05	0.000107126
EXON1	SLC26A1	4	987108	1	cg08393516	0.704847275	0.581294967	0.527146518	-0.177700757	3.88E-09	7.79E-08
EXON1	SLC26A9	1	205897159/205897165	2	cg06437052/cg22863880	0.686240295	0.603381958	0.502892759	-0.183347536	1.65E-07	1.85E-06

EXON1	SLC30A10	1	220101785/220101371/220101698/220101945/220101725/220101728/220101962/220101368	8	cg10325600/cg14848594/cg17721710/cg23815582/cg24396691/cg24933645/cg25317664/cg26685195	0.1694661	0.217278925	0.331099629	0.16163353	1.02E-05	6.92E-05
EXON1	SLC32A1	20	37353117/37353158/37353700/37353126	4	cg08313939/cg10216615/cg15230781/cg24454144	0.262829423	0.364909067	0.511360519	0.248531097	3.31E-11	2.38E-09
EXON1	SLC35F1	6	118228869/118228871	2	cg11430157/cg26532358	0.100903596	0.172972118	0.269015835	0.168112239	1.81E-05	0.000116593
EXON1	SLC35F3	1	234040765/234040781/234040833	3	cg05051043/cg09424759/cg25437385	0.28101686	0.333445822	0.492317723	0.211300863	2.28E-06	1.83E-05
EXON1	SLC36A2	5	150727045	1	cg19562321	0.47641976	0.321748678	0.196324459	-0.280095301	1.44E-14	3.38E-11
EXON1	SLC36A3	5	150683039/150683178	2	cg00109131/cg22445920	0.750848095	0.711970936	0.588135614	-0.162712481	1.18E-07	1.39E-06
EXON1	SLC38A4	12	47219705/47219737	2	cg04053798/cg11146691	0.385961155	0.2896573	0.218037345	-0.16792381	5.47E-06	3.97E-05
EXON1	SLC39A5	12	56624552/56623826	2	cg00668685/cg08214142	0.51483157	0.417882722	0.343827905	-0.171003665	6.59E-11	3.84E-09
EXON1	SLC3A1	2	44502740/44502977	2	cg02192965/cg03217954	0.59685885	0.421190456	0.334986914	-0.261871936	3.07E-10	1.06E-08
EXON1	SLC46A2	9	115653026/115652824	2	cg04881348/cg07758904	0.329247395	0.467430022	0.5527728	0.223525405	1.13E-05	7.59E-05
EXON1	SLC4A1	17	42345488/42345478	2	cg03580247/cg16063416	0.81532451	0.733635875	0.601379539	-0.213944971	2.91E-08	4.23E-07
EXON1	SLC5A11	16	24857601/24857579	2	cg04756594/cg23280807	0.53221779	0.430310758	0.353194905	-0.179022885	1.21E-09	2.99E-08
EXON1	SLC5A7	2	108603005/108603193	2	cg16232126/cg26333822	0.336260815	0.343567149	0.498572323	0.162311508	2.17E-05	0.000135661
EXON1	SLC6A11	3	10858003	1	cg16435571	0.25899077	0.344020061	0.457519318	0.198528548	1.82E-10	7.25E-09
EXON1	SLC6A15	12	85306548/85306474/85306323/85306574	4	cg02492791/cg14449051/cg19952303/cg24418420	0.229127629	0.269826865	0.424654816	0.195527187	0.000143945	0.000732813
EXON1	SLC6A2	16	55690564/55690873	2	cg04490714/cg04874129	0.354770675	0.431056711	0.546153043	0.191382368	8.78E-08	1.07E-06
EXON1	SLC7A14	3	170303830/170303721	2	cg18774195/cg21884231	0.328577135	0.405935067	0.585724873	0.257147738	5.20E-08	6.80E-07
EXON1	SLC8A3	14	70655686/70546695/70655382	3	cg05203877/cg16799926/cg20409590	0.306404613	0.379666643	0.512636003	0.20623139	9.78E-06	6.65E-05
EXON1	SLC9A11	1	173572181	1	cg25096142	0.833196155	0.726588017	0.636620873	-0.196575282	7.94E-06	5.53E-05
EXON1	SLC9A9	3	143567031	1	cg23538718	0.161553006	0.338766835	0.377645814	0.216092808	2.42E-05	0.000148957
EXON1	SLFN11	17	33700513	1	cg05504685	0.362066225	0.451851989	0.559316886	0.197250661	2.97E-08	4.30E-07
EXON1	SLFN14	17	33885049	1	cg00397422	0.745824695	0.608531922	0.483036455	-0.26278824	3.63E-07	3.63E-06

EXON1	SLIT1	10	98945465/98945300/98945664	3	cg02741216/cg07143898/cg13196826	0.080148571	0.131306492	0.281122222	0.200973651	0.000132371	0.000680847
EXON1	SLIT2	4	20255264/20255343	2	cg03742003/cg08428452	0.257782725	0.366029311	0.529762009	0.271979284	1.72E-07	1.91E-06
EXON1	SLIT3	5	168727752/168728081/168727762/168728076/168727686	5	cg01663016/cg07136998/cg09073398/cg11732619/cg17386093	0.301162019	0.384144305	0.518711883	0.217549864	2.86E-08	4.18E-07
EXON1	SLITRK1	13	84456486/84455525/84454495/84453280/84456127/84455742/84453665/84456410/84452994/84454283/84456171/84456308/84455126/84453836/84455651/84455028/84455576/84456258	18	cg01729717/cg02365360/cg02772430/cg04095724/cg07104706/cg10236414/cg16727923/cg18215217/cg18789918/cg18937395/cg19696317/cg20312205/cg20826348/cg21448057/cg22057720/cg25188032/cg26799881/cg26998274	0.343236057	0.431788205	0.55327161	0.210035553	1.10E-11	1.22E-09
EXON1	SLITRK5	13	88324879	1	cg06682197	0.173845211	0.201134676	0.395404609	0.221559398	6.03E-07	5.67E-06
EXON1	SLMAP	3	57743543/57743377	2	cg00002426/cg15172734	0.47129935	0.38730215	0.30803932	-0.16326003	1.44E-08	2.33E-07
EXON1	SLPI	20	43883172	1	cg14609407	0.373817255	0.2850587	0.214713523	-0.159103732	2.35E-12	4.38E-10
EXON1	SMOC1	14	70346413/70346477/70346221/70346151/70346417	5	cg15239123/cg15724184/cg15996043/cg18493027/cg19504005	0.124645166	0.219719925	0.38550761	0.260862444	8.81E-06	6.09E-05
EXON1	SNAP25	20	10199536	1	cg16564824	0.264246075	0.33519145	0.472985409	0.208739334	1.30E-06	1.13E-05
EXON1	SNCG	10	88718317/88718393	2	cg03677069/cg21012874	0.497614665	0.392017356	0.296029682	-0.201584983	9.97E-11	4.78E-09
EXON1	SNTG2	2	946648	1	cg09426834	0.305865165	0.362097389	0.540930227	0.235065062	1.41E-06	1.20E-05
EXON1	SORCS1	10	108924132/108923781	2	cg13563903/cg16415058	0.275082459	0.335265179	0.458621235	0.183538776	4.24E-08	5.74E-07
EXON1	SORCS3	10	106401517/106401319/106400880/106401479	4	cg01874697/cg10778841/cg16787600/cg18326021	0.313590103	0.407276963	0.531378789	0.217788686	4.07E-12	6.05E-10

EXON1	SOX1	13	112724583/112723581/112723477/112723421/112725903/112725605/112722233/112723226/112722333/112723034/112721950/112722719/112724270/112724245/112724454/112724221	16	cg00073003/cg01236132/cg04047221/cg04865691/cg06488256/cg10245273/cg11750165/cg14842777/cg15466862/cg15653173/cg16705627/cg19802138/cg21385666/cg23668285/cg25476766/cg26151467	0.345366265	0.381839098	0.496207146	0.150840882	1.41E-06	1.20E-05
EXON1	SOX11	2	5837429/5837929/5836366/5835853/5838258/5833017/5837348/5836256/5836111/5837057/5834638/5832882/5833195/5836231/5840827/5833169/5834062/5837002/5836713/5833774/5836181	21	cg00078318/cg03594550/cg04043795/cg06173889/cg06830064/cg08165221/cg11716272/cg14776201/cg15034345/cg15989068/cg16330247/cg18449120/cg18863333/cg18897632/cg19958021/cg20008332/cg20401252/cg20927661/cg23668184/cg24837370/cg26659079	0.447223431	0.526076141	0.649590502	0.202367071	4.35E-11	2.88E-09
EXON1	SOX14	3	137483696/137483992/137483617/137484364/137484220	5	cg03811478/cg04945331/cg06613095/cg11630154/cg22885558	0.238239099	0.29130647	0.421321141	0.183082042	0.000158636	0.000801298
EXON1	SOX17	8	55370951/55370544/55370579	3	cg00123055/cg02919422/cg26059468	0.485218765	0.550892078	0.669415714	0.184196949	1.00E-08	1.73E-07
EXON1	SOX7	8	10588013/10587769	2	cg08056146/cg26077100	0.076560048	0.141384326	0.268622334	0.192062286	0.002228053	0.008499111
EXON1	SPAG4L	20	31592147	1	cg02510802	0.74023952	0.573631311	0.371162491	-0.369077029	4.41E-12	6.34E-10
EXON1	SPAG6	10	22634432/22634439	2	cg05099508/cg24031355	0.14319122	0.251403974	0.375843679	0.23265246	1.01E-05	6.83E-05
EXON1	SPARC	5	151066460/151066486/151066484	3	cg08331313/cg08879559/cg25587069	0.40899071	0.459904543	0.567749924	0.158759214	4.21E-06	3.15E-05
EXON1	SPATA16	3	172859022/172858917/172858969	3	cg01216369/cg06577725/cg19933965	0.841335905	0.708925717	0.607703923	-0.233631982	1.04E-08	1.78E-07
EXON1	SPATA19	11	133715264	1	cg16327839	0.738463435	0.701864861	0.583711655	-0.15475178	9.82E-06	6.67E-05

EXON1	SPATA21	1	16763671	1	cg26845278	0.769193215	0.715965794	0.614876923	-0.154316292	2.95E-05	0.000177969
EXON1	SPATS1	6	44310445	1	cg09662584	0.157505635	0.218517676	0.333426755	0.17592112	1.04E-05	7.01E-05
EXON1	SPDEF	6	34523925	1	cg07705908	0.440337315	0.355744344	0.276909786	-0.163427529	1.63E-11	1.57E-09
EXON1	SPEG	2	220299740/220299900	2	cg07921384/cg20174636	0.213680036	0.331056603	0.515142732	0.301462696	4.32E-07	4.23E-06
EXON1	SPHKAP	2	229046325	1	cg04072323	0.285360715	0.389797139	0.577976023	0.292615308	6.01E-09	1.13E-07
EXON1	SPO11	20	55904856/55904875	2	cg19911058/cg26645834	0.728778565	0.583588719	0.48247803	-0.246300535	1.48E-12	3.13E-10
EXON1	SPOCK1	5	136834877	1	cg05143123	0.139690965	0.215181332	0.399490303	0.259799338	8.55E-05	0.000459608
EXON1	SPOCK3	4	168155695/168155687	2	cg09722742/cg13913012	0.145011377	0.209011643	0.353015779	0.208004402	4.98E-06	3.66E-05
EXON1	SPSB4	3	140770751	1	cg13874817	0.223711903	0.288352178	0.423245082	0.199533179	5.66E-05	0.000318568
EXON1	SPZ1	5	79616145	1	cg07184465	0.57792236	0.473602033	0.359226345	-0.218696015	2.01E-07	2.19E-06
EXON1	SRD5A2	2	31805970/31805915	2	cg18948125/cg19502744	0.494859255	0.564542572	0.671387095	0.17652784	1.70E-11	1.61E-09
EXON1	SST	3	187388148/187387999/187388128	3	cg02164046/cg15347189/cg16927040	0.423200967	0.51935202	0.650999459	0.227798492	8.20E-11	4.31E-09
EXON1	SSTR2	17	71161322/71161178/71161160/71161415/71161258	5	cg06685177/cg13344169/cg21547708/cg26729197/cg27066284	0.14472133	0.222564019	0.340479593	0.195758263	3.36E-05	0.00019965
EXON1	ST6GALNAC1	17	74639849/74639793/74639731	3	cg11469587/cg13015534/cg15503752	0.385521762	0.281389767	0.225724409	-0.159797353	2.00E-11	1.78E-09
EXON1	ST6GALNAC5	1	77333229/77333198	2	cg04077662/cg06201642	0.26808874	0.377675175	0.53033383	0.26224509	1.48E-07	1.69E-06
EXON1	ST8SIA1	12	22487437/22487459/22487219	3	cg09354309/cg10333808/cg24723331	0.135148496	0.178398841	0.346324339	0.211175843	0.00031756	0.001484979
EXON1	ST8SIA3	18	55020099/55019843	2	cg04017533/cg13096208	0.41537209	0.499723978	0.684239282	0.268867192	2.50E-10	9.09E-09
EXON1	ST8SIA4	5	100238977/100238983/100238655/100238819	4	cg03975797/cg04380513/cg13320626/cg18428516	0.130378203	0.211127316	0.324879061	0.194500859	1.60E-05	0.000103895
EXON1	ST8SIA5	18	44336399	1	cg19751300	0.246936895	0.371629106	0.542666518	0.295729623	1.23E-08	2.04E-07
EXON1	STAB2	12	103981113	1	cg10622586	0.53435505	0.432629811	0.376232255	-0.158122795	1.01E-09	2.60E-08
EXON1	STAC	3	36422198/36422209	2	cg15321293/cg26615813	0.288692615	0.406833989	0.543879364	0.255186749	1.93E-11	1.75E-09
EXON1	STAG3	7	99775558	1	cg00553149	0.51321916	0.611819217	0.685586805	0.172367645	1.50E-08	2.41E-07
EXON1	STAP1	4	68424495	1	cg12879425	0.515848295	0.456623606	0.33362903	-0.182219265	0.000125038	0.000647157
EXON1	STK33	11	8615456/8615356	2	cg21045608/cg26668837	0.141130164	0.273080832	0.449408659	0.308278495	1.12E-06	9.87E-06
EXON1	STMN2	8	80523413	1	cg09071889	0.37668888	0.499617317	0.643886368	0.267197488	1.24E-09	3.07E-08

EXON1	STOX2	4	184826953/184827086/184827754/184827491/184826704	5	cg01758805/cg02071076/cg02500300/cg06367693/cg11354594	0.222166173	0.24050692	0.39943448	0.177268306	0.00033048	0.001535625
EXON1	STRA6	15	74495354/74501317/74495109/74495384	4	cg00075967/cg11680605/cg11787522/cg26774156	0.54655957	0.447397081	0.348826711	-0.197732859	2.83E-13	1.14E-10
EXON1	STX19	3	93747410	1	cg27200466	0.58232939	0.443080756	0.341259436	-0.241069954	2.25E-11	1.91E-09
EXON1	SUNC1	7	48068665	1	cg23509098	0.70715429	0.561490989	0.376269341	-0.330884949	9.92E-14	5.47E-11
EXON1	SUSD5	3	33260366	1	cg09463233	0.175253587	0.312971031	0.436781936	0.261528349	9.08E-06	6.25E-05
EXON1	SV2A	1	149889364/149889361/149889339/149889330	4	cg01705587/cg04927502/cg11085602/cg24629122	0.090721684	0.157913011	0.265194618	0.174472933	6.84E-05	0.00037754
EXON1	SYCN	19	39694617/39694831/39694709	3	cg00880452/cg02863073/cg22290648	0.364387483	0.456311865	0.593968955	0.229581471	1.89E-05	0.000120902
EXON1	SYCP2L	6	10887367/10887185	2	cg03320827/cg26237681	0.251650399	0.311186155	0.50790396	0.256253561	8.40E-07	7.61E-06
EXON1	SYT16	14	62462754	1	cg14479704	0.537808285	0.489511656	0.366810405	-0.17099788	6.53E-08	8.27E-07
EXON1	SYT6	1	114696465/114696462/114696350	3	cg05368341/cg09177131/cg19304150	0.224724152	0.298136266	0.469972501	0.245248349	3.20E-07	3.29E-06
EXON1	SYT8	11	1855746	1	cg03861143	0.55054825	0.440729306	0.354293586	-0.196254664	1.30E-11	1.34E-09
EXON1	SYT9	11	7273498/7273378	2	cg08185661/cg22723056	0.271750638	0.313409167	0.454316533	0.182565895	0.000126964	0.000656096
EXON1	SYTL3	6	159071106/159071076	2	cg00370106/cg17768957	0.327156584	0.194945248	0.15898317	-0.168173414	4.06E-08	5.54E-07
EXON1	T	6	166581929	1	cg23688510	0.214341215	0.322834378	0.444819487	0.230478272	0.000973211	0.004049891
EXON1	T-SP1	8	10383156	1	cg23996846	0.74477846	0.684692189	0.577011155	-0.167767305	2.71E-06	2.14E-05
EXON1	TAAR5	6	132910681	1	cg17829936	0.580202615	0.508016006	0.359380223	-0.220822392	4.15E-08	5.63E-07
EXON1	TAC1	7	97361408	1	cg16288089	0.38903177	0.447987311	0.610351032	0.221319262	1.29E-05	8.55E-05
EXON1	TACR3	4	104640489/104640772/104640833/104640662/104640560	5	cg04263186/cg14664621/cg16461251/cg18538958/cg20128928	0.423942778	0.4944614	0.608301675	0.184358897	4.55E-10	1.40E-08
EXON1	TAS2R16	7	122635026/122635522	2	cg03640110/cg12150401	0.6599219	0.617255522	0.500084118	-0.159837782	0.001662246	0.006554354
EXON1	TAS2R8	12	10958919	1	cg21156620	0.639956295	0.561372061	0.399305305	-0.24065099	5.33E-13	1.76E-10
EXON1	TAS2R9	12	10962437	1	cg20090497	0.68535001	0.525841789	0.424362132	-0.260987878	6.57E-08	8.31E-07
EXON1	TBX15	1	119532093/119532116/119532044/119532056	4	cg07892597/cg10703826/cg14565725/cg24884142	0.444946935	0.553125828	0.682131064	0.237184129	1.12E-08	1.88E-07
EXON1	TBX18	6	85473773	1	cg01461410	0.269517745	0.379933733	0.499295677	0.229777932	3.68E-08	5.12E-07

EXON1	TBXAS1	7	139478207/139529130	2	cg01014432/cg12104698	0.50486484	0.410135933	0.337697018	-0.167167822	6.15E-11	3.63E-09
EXON1	TCERG1L	10	133109867	1	cg06304097	0.25418814	0.348217361	0.449764073	0.195575933	3.60E-08	5.03E-07
EXON1	TCF21	6	134210667/134210946/134210812/134210273/134210279/134210307/134210598	7	cg03087607/cg04467618/cg04692403/cg12468478/cg17470942/cg24157171/cg24215443	0.370400736	0.415368787	0.53662237	0.166221634	8.07E-08	1.00E-06
EXON1	TCP10	6	167797877	1	cg19930296	0.7115431	0.607950922	0.492313495	-0.219229605	3.59E-08	5.03E-07
EXON1	TCP10L2	6	167584201	1	cg16214245	0.718269	0.609363256	0.498562314	-0.219706686	6.80E-08	8.58E-07
EXON1	TCTEX1D1	1	67218165	1	cg25339566	0.366130975	0.456969483	0.614285277	0.248154302	8.62E-06	5.97E-05
EXON1	TDO2	4	156824883	1	cg04795662	0.510825065	0.410470811	0.313949386	-0.196875679	2.15E-07	2.32E-06
EXON1	TDRD10	1	154475139/154475068/154475173/154475269/154474800	5	cg02457680/cg05637536/cg18081940/cg19360104/cg25460143	0.359023792	0.42043062	0.549326857	0.190303065	2.01E-09	4.51E-08
EXON1	TEKT1	17	6734991	1	cg13337865	0.17634453	0.218720239	0.343863945	0.167519415	4.18E-05	0.000242753
EXON1	TEKT2	1	36549719/36549726	2	cg19644018/cg24813212	0.123482982	0.178071389	0.275627008	0.152144026	0.000826083	0.003491452
EXON1	TEX15	8	30705886	1	cg19418958	0.4878085	0.373841783	0.287975295	-0.199833205	4.86E-12	6.55E-10
EXON1	TF	3	133465180	1	cg24879335	0.359086565	0.403408922	0.534619114	0.175532549	7.70E-05	0.000418721
EXON1	TFEC	7	115670795	1	cg15339605	0.260855737	0.142948516	0.083422935	-0.177432802	2.39E-08	3.58E-07
EXON1	TFF1	21	43786627	1	cg06099014	0.495460795	0.409595083	0.328818418	-0.166642377	2.91E-11	2.21E-09
EXON1	TFPI2	7	93520036/93520024/93519924/93520012	4	cg14775114/cg17338208/cg23686014/cg24531255	0.142450764	0.321753576	0.519702184	0.377251421	1.00E-11	1.15E-09
EXON1	TGM3	20	2276663	1	cg24731241	0.624083145	0.540739672	0.429558477	-0.194524668	2.28E-10	8.55E-09
EXON1	TGM4	3	44916102	1	cg26264008	0.49521531	0.4171876	0.333413573	-0.161801737	1.62E-08	2.55E-07
EXON1	THBD	20	23028977/23029640/23030250/23028890/23030102/23029298/23029287/23028304/23029806	9	cg07255664/cg08354093/cg09279949/cg10505785/cg11769163/cg16339238/cg17368760/cg20096897/cg24562819	0.389573577	0.478798267	0.575408563	0.185834986	3.13E-08	4.49E-07
EXON1	THBS4	5	79331047	1	cg03336086	0.23092927	0.293593278	0.419941482	0.189012212	0.000416201	0.001888826
EXON1	THEM5	1	151825974/151826167/151826123	3	cg01062247/cg11344950/cg18081104	0.50145591	0.420248339	0.32742578	-0.17403013	1.44E-10	6.27E-09
EXON1	THEMIS	6	128222053/128239611	2	cg11610626/cg17113883	0.623721595	0.534845003	0.44263518	-0.181086415	9.78E-09	1.70E-07

EXON1	THY1	11	119293861/119293869/119293863	3	cg10601287/cg16566400/cg17236709	0.307441023	0.412989475	0.529199909	0.221758887	1.44E-05	9.46E-05
EXON1	TIAM2	6	155538155	1	cg16333846	0.616862365	0.544267617	0.437494659	-0.179367706	6.37E-09	1.19E-07
EXON1	TLL1	4	166794471/166794895/166794971/166794786	4	cg08570521/cg09669853/cg19898128/cg24521633	0.14149001	0.220108282	0.378027405	0.236537396	9.05E-06	6.24E-05
EXON1	TLX1	10	102891076/102891568/102891280/102891080	4	cg02096397/cg02450004/cg05329692/cg27272677	0.206961461	0.194047207	0.357389394	0.150427934	0.000995969	0.004132895
EXON1	TLX1NB	10	102890886	1	cg11196237	0.213237165	0.223588122	0.368317932	0.155080767	0.000303516	0.00142535
EXON1	TM4SF19	3	196065189	1	cg02322879	0.796740025	0.720172167	0.631556873	-0.165183152	1.17E-05	7.79E-05
EXON1	TM4SF20	2	228243972	1	cg20683151	0.61808082	0.523253783	0.456970095	-0.161110725	1.16E-07	1.37E-06
EXON1	TM4SF4	3	149192609	1	cg05483388	0.533137955	0.438724928	0.357236132	-0.175901823	3.76E-12	5.86E-10
EXON1	TM6SF1	15	83776422/83776446/83776420	3	cg03063639/cg14696396/cg26460092	0.288529638	0.477104186	0.674643993	0.386114356	3.82E-14	3.78E-11
EXON1	TMEFF2	2	193059405/193059230	2	cg06856528/cg18221862	0.227860498	0.331233553	0.445708082	0.217847584	1.40E-06	1.20E-05
EXON1	TMEM105	17	79304420/79304188	2	cg15913671/cg23514211	0.403521475	0.322171772	0.251286836	-0.152234639	4.18E-11	2.84E-09
EXON1	TMEM108	3	132757267	1	cg09090724	0.31674414	0.423175194	0.552739118	0.235994978	9.13E-10	2.45E-08
EXON1	TMEM130	7	98467571	1	cg01307939	0.072572016	0.210948573	0.306677468	0.234105452	2.16E-11	1.85E-09
EXON1	TMEM132D	12	130387861/130387821/130388198	3	cg03469054/cg11160362/cg23266743	0.420658665	0.495962311	0.607788034	0.187129369	1.57E-09	3.69E-08
EXON1	TMEM139	7	142982463/142982262	2	cg08261841/cg26511386	0.576420968	0.44608855	0.335559352	-0.240861615	4.41E-12	6.34E-10
EXON1	TMEM155	4	122686269/122686038/122686319	3	cg05396987/cg10863741/cg21077559	0.25409643	0.370161301	0.524858427	0.270761997	2.02E-10	7.89E-09
EXON1	TMEM179	14	105070998/105070864	2	cg00107187/cg10281977	0.279454658	0.306052686	0.436962132	0.157507474	0.000189462	0.000939777
EXON1	TMEM196	7	19812327	1	cg08991339	0.276333955	0.324308372	0.432952155	0.1566182	1.58E-05	0.000102975
EXON1	TMEM212	3	171561201	1	cg13581779	0.62128399	0.7349586	0.78306295	0.16177896	3.66E-12	5.77E-10
EXON1	TMEM215	9	32783516	1	cg11308840	0.262321	0.324663561	0.42953105	0.16721005	4.92E-05	0.00028155
EXON1	TMEM220	17	10633470	1	cg01275588	0.219031841	0.251888634	0.387590759	0.168558918	0.010184019	0.032843585
EXON1	TMEM26	10	63213192/63212862/63213104/63212815	4	cg02853012/cg09135551/cg18192919/cg26785954	0.177527685	0.231648921	0.34482665	0.167298965	8.97E-07	8.08E-06
EXON1	TMEM40	3	12800802	1	cg24290948	0.75814872	0.6240729	0.501171659	-0.256977061	8.40E-12	9.97E-10
EXON1	TMEM71	8	133772889/133772742	2	cg20955688/cg27159719	0.321192697	0.169322804	0.169164931	-0.152027765	9.77E-06	6.65E-05

EXON1	TMEM90B	20	24450353/24449865/24449916/24450361	4	cg09623400/cg16098981/cg16120788/cg19654195	0.277829595	0.376966383	0.457964334	0.180134739	8.15E-06	5.67E-05
EXON1	TMPRSS11A	4	68829101	1	cg23734503	0.8005691	0.724733106	0.645190427	-0.155378673	0.001023074	0.004230775
EXON1	TMPRSS11B	4	69111391	1	cg06399881	0.723383995	0.599150383	0.495239614	-0.228144381	1.62E-09	3.76E-08
EXON1	TMPRSS11E	4	69313197	1	cg24792113	0.396462015	0.294216533	0.240996355	-0.15546566	1.26E-09	3.10E-08
EXON1	TMPRSS4	11	117947997/117947877	2	cg05416223/cg22957898	0.420708625	0.338771175	0.268289677	-0.152418948	8.31E-11	4.31E-09
EXON1	TNFAIP2	14	103592734	1	cg04264002	0.54440615	0.451647061	0.385773686	-0.158632464	1.62E-06	1.35E-05
EXON1	TNFRSF25	1	6526129/6526182	2	cg10059687/cg12814070	0.49407118	0.586896289	0.646923132	0.152851952	1.05E-05	7.07E-05
EXON1	TNFRSF8	1	12123554/12186079/12123437	3	cg05121790/cg10058766/cg17897879	0.374147229	0.453641684	0.546550893	0.172403665	4.59E-08	6.13E-07
EXON1	TNR	1	175712552/175712382	2	cg05996213/cg07107171	0.22574769	0.271962389	0.393704888	0.167957198	0.00163262	0.006450993
EXON1	TOB1	17	48939676/48941039	2	cg13119853/cg14494812	0.57467162	0.464720517	0.376260136	-0.198411484	1.36E-09	3.29E-08
EXON1	TP53BP1	15	43802612	1	cg04678936	0.384831545	0.476032261	0.53960045	0.154768905	3.62E-07	3.62E-06
EXON1	TP63	3	189349278/189349323	2	cg04483101/cg21723486	0.731350968	0.580754875	0.449463195	-0.281887772	6.78E-09	1.25E-07
EXON1	TPD52	8	80992750/81083829	2	cg16773115/cg22273042	0.61247576	0.492659661	0.383975359	-0.228500401	2.19E-11	1.87E-09
EXON1	TPD52L3	9	6328386/6328493	2	cg03485840/cg14286682	0.624802443	0.523139206	0.448331048	-0.176471395	7.60E-11	4.12E-09
EXON1	TPM4	19	16178514/16178426/16187364	3	cg09926488/cg22270364/cg25593560	0.7752139	0.741008956	0.6135898	-0.1616241	0.000402334	0.001835335
EXON1	TPTE2	13	20110871	1	cg19950767	0.73145477	0.68496045	0.573175373	-0.158279397	2.86E-06	2.25E-05
EXON1	TREM2	6	41130917	1	cg01980222	0.5824335	0.493762728	0.41245735	-0.16997615	2.57E-06	2.04E-05
EXON1	TRH	3	129693613/129693586/129693385/129693370/129693489	5	cg01009664/cg02700891/cg11940285/cg18862481/cg22512438	0.30922142	0.419885811	0.616723861	0.307502441	1.16E-08	1.93E-07
EXON1	TRHDE	12	72667236/72667326/72666976/72667150	4	cg01030121/cg09972192/cg13663218/cg27579953	0.312842455	0.397417921	0.574754972	0.261912517	7.01E-10	1.99E-08
EXON1	TRIB2	2	12857398/12858655/12858604/12857067/12858298/12858555/12857572/12857811/12857652/12858483	10	cg01322165/cg05353478/cg11385684/cg13769605/cg13776499/cg13921956/cg15033181/cg18155032/cg24543400/cg25623934	0.087995671	0.146418624	0.244196487	0.156200817	0.000290954	0.001372693

EXON1	TRIM15	6	30131821/30131219/30131283/30131840/30131570/30131526/30131806/30131755/30131361/30131001/30131634/30131719/30131458/30131613/30131586/30131214/30131691/30131189/30131749/30131467	20	cg00628477/cg00720829/cg00879790/cg01905142/cg02012483/cg03609216/cg05235884/cg05664039/cg05701418/cg06051311/cg07421969/cg09769113/cg12232118/cg14815183/cg20488756/cg21856784/cg21988950/cg22425467/cg23778358/cg27583815	0.51885103	0.43111449	0.35348023	-0.1653708	1.42E-11	1.44E-09
EXON1	TRIM42	3	140396963/140397089	2	cg06090362/cg12242338	0.604944873	0.495699642	0.41210398	-0.192840893	3.19E-08	4.56E-07
EXON1	TRIM61	4	165898707	1	cg25147158	0.521835355	0.616170856	0.697822795	0.17598744	8.25E-09	1.47E-07
EXON1	TRIM64B	11	89609024	1	cg24005701	0.519617555	0.447735233	0.368904495	-0.15071306	3.43E-08	4.85E-07
EXON1	TRIM71	3	32860178/32860004/32859587/32859558	4	cg12338417/cg18405727/cg19741945/cg23243038	0.212840747	0.273316637	0.402333762	0.189493016	3.43E-06	2.64E-05
EXON1	TRIM72	16	31225357/31225350	2	cg07620544/cg27124512	0.55506601	0.466283875	0.388951707	-0.166114303	2.46E-08	3.68E-07
EXON1	TRIM75	4	165980885	1	cg03505125	0.62360929	0.557372533	0.456281373	-0.167327917	1.05E-05	7.09E-05
EXON1	TRPC4	13	38443783/38443809	2	cg02057391/cg21432954	0.319926323	0.397168133	0.53258843	0.212662107	4.47E-05	0.000257725
EXON1	TRPC6	11	101454317/101454626	2	cg03411507/cg11016563	0.33482509	0.403225172	0.58180788	0.24698279	2.97E-11	2.24E-09
EXON1	TRPC7	5	135701094/135701050	2	cg06448705/cg23288973	0.416880065	0.493009661	0.615086455	0.19820639	1.17E-08	1.95E-07
EXON1	TRPM6	9	77502747/77502963	2	cg14095101/cg16509045	0.41669142	0.324089622	0.245631121	-0.171060299	1.68E-10	6.97E-09
EXON1	TSPAN16	19	11406993/11406877	2	cg17496921/cg25382751	0.606808085	0.511094744	0.415950014	-0.190858071	1.25E-08	2.07E-07
EXON1	TSPAN18	11	44927976	1	cg06401614	0.48198153	0.3867593	0.316124595	-0.165856935	1.09E-10	5.10E-09
EXON1	TTN	2	179672121/179672126	2	cg05185019/cg10859358	0.776356565	0.58309665	0.495723477	-0.280633088	8.73E-09	1.54E-07
EXON1	TTYH1	19	54926805/54926734	2	cg09474331/cg23695687	0.184290943	0.320623184	0.49677406	0.312483117	1.70E-07	1.90E-06
EXON1	TUSC3	8	15397899/15397731/15397729/15398053	4	cg03032098/cg03127174/cg18145877/cg26374823	0.317675669	0.429652578	0.539913742	0.222238073	4.75E-09	9.22E-08
EXON1	TWIST1	7	19157193/19156902/19157263/19156550/19156621	5	cg17839237/cg20052718/cg24446548/cg26312150/cg26818735	0.110370943	0.198173023	0.364804639	0.254433696	1.25E-08	2.08E-07

EXON1	TXLNB	6	139613145	1	cg18953104	0.409258315	0.539306706	0.625136177	0.215877862	1.34E-09	3.24E-08
EXON1	TXNDC3	7	37888257	1	cg11932205	0.60284149	0.518042039	0.436088327	-0.166753163	3.03E-10	1.06E-08
EXON1	UBE2QL1	5	6449090/6449153/6449001	3	cg06997381/cg13955436/cg26692294	0.090937498	0.163753928	0.286507531	0.195570033	3.46E-05	0.000204814
EXON1	UCHL1	4	41258910/41258935	2	cg07068756/cg16142306	0.180193261	0.216928306	0.377133643	0.196940382	0.001489093	0.005942445
EXON1	UGT2A2	4	70504750	1	cg02219607	0.854529735	0.804295483	0.638369209	-0.216160526	9.11E-06	6.26E-05
EXON1	UGT2B15	4	69536319	1	cg22161115	0.42884956	0.323746544	0.245807791	-0.183041769	1.59E-11	1.53E-09
EXON1	UGT3A1	5	35991382	1	cg19897071	0.50859441	0.588819472	0.686613518	0.178019108	2.84E-08	4.17E-07
EXON1	UGT3A2	5	36066972/36066944	2	cg10140794/cg27638727	0.24182068	0.320017039	0.399293809	0.157473129	2.25E-06	1.81E-05
EXON1	UNC5C	4	96470053/96470094	2	cg00329039/cg10520887	0.247111658	0.332582539	0.448170536	0.201058879	3.76E-06	2.87E-05
EXON1	UNC80	2	210636844/210636748/210636783	3	cg01082843/cg13347071/cg24915947	0.299552768	0.363986007	0.503958223	0.204405454	5.58E-08	7.23E-07
EXON1	UNCX	7	1272710	1	cg03092191	0.266196025	0.287000883	0.467925414	0.201729389	6.69E-05	0.000370432
EXON1	USH2A	1	216596639	1	cg15590780	0.428820805	0.352626139	0.254043968	-0.174776837	5.18E-12	6.85E-10
EXON1	USP54	10	75335352	1	cg12303623	0.56002716	0.400204194	0.346340677	-0.213686483	1.34E-08	2.21E-07
EXON1	UTF1	10	135044114/135044040	2	cg09053680/cg09714379	0.20498208	0.253523268	0.380217198	0.175235118	7.32E-05	0.00040047
EXON1	UTS2R	17	80332278/80333257/80333040	3	cg12598178/cg19954017/cg24192805	0.480132765	0.556091644	0.678547882	0.198415117	5.39E-09	1.03E-07
EXON1	VAT1L	16	77822500/77822771	2	cg01256089/cg02761480	0.105097593	0.179936416	0.274560032	0.169462439	0.001965622	0.007632291
EXON1	VAV3	1	108507340/108507468	2	cg16120828/cg24662718	0.147082161	0.261738394	0.415378062	0.268295901	1.37E-06	1.18E-05
EXON1	VAX2	2	71127779/71127892	2	cg15579817/cg21637670	0.197576615	0.19675699	0.417413989	0.219837374	0.000948719	0.00395918
EXON1	VCAM1	1	101185311	1	cg25762679	0.47501733	0.571155178	0.627295764	0.152278434	2.94E-06	2.30E-05
EXON1	VCAN	5	82767526/82767617	2	cg04525496/cg05176349	0.132559309	0.18446299	0.343477828	0.210918519	2.62E-06	2.07E-05
EXON1	VEGFC	4	177713813/177713423	2	cg13378388/cg26301788	0.270426783	0.300360647	0.449001314	0.178574531	9.27E-05	0.000494152
EXON1	VIPR2	7	158937610/158937494	2	cg03976877/cg13794530	0.344609673	0.441673113	0.646611789	0.302002116	8.30E-13	2.39E-10
EXON1	VMO1	17	4689655/4689713/4689640	3	cg08341874/cg21898424/cg27453857	0.352679173	0.399235657	0.510220052	0.157540878	8.10E-05	0.000438547
EXON1	VSTM2A	7	54610022/54610192/54610139/54610320	4	cg02910208/cg03775422/cg04024095/cg14304469	0.27133063	0.342809737	0.472008952	0.200678322	2.41E-05	0.000148742
EXON1	VSTM2B	19	30017511	1	cg04711162	0.427465445	0.502654067	0.623896918	0.196431473	7.04E-10	1.99E-08
EXON1	VSTM2L	20	36531652	1	cg26366107	0.115763061	0.169990506	0.278770598	0.163007538	0.000469668	0.002107611

EXON1	VSX1	20	25062754/25062447	2	cg00043788/cg14763548	0.309602228	0.34355494	0.553490257	0.24388803	3.61E-08	5.04E-07
EXON1	VSX2	14	74706196/74706336	2	cg07573727/cg11525285	0.291580825	0.398279478	0.534100418	0.242519593	5.94E-08	7.61E-07
EXON1	VWC2	7	49813486	1	cg18206027	0.182120415	0.220693698	0.416763476	0.234643062	3.27E-06	2.54E-05
EXON1	WDR17	4	176987020/176987009/176987174	3	cg08095852/cg11923920/cg27486637	0.094271628	0.242014108	0.383819349	0.289547722	1.67E-05	0.000107968
EXON1	WDR86	7	151106990	1	cg12433277	0.31276473	0.418221856	0.539497191	0.226732461	7.03E-07	6.51E-06
EXON1	WIPF3	7	29874354	1	cg14354418	0.71834715	0.688226222	0.554054345	-0.164292805	6.18E-06	4.43E-05
EXON1	WISP3	6	112375302/112375333/112375324	3	cg07709205/cg10525372/cg21773633	0.52809172	0.437901892	0.354784148	-0.173307572	2.37E-11	1.95E-09
EXON1	WNT2	7	116963259/116963193	2	cg07697895/cg27331524	0.269514918	0.365072882	0.541466436	0.271951519	9.01E-10	2.43E-08
EXON1	WTIP	19	34973341/34973191	2	cg15409931/cg24916013	0.129627403	0.212062824	0.32917737	0.199549968	1.03E-06	9.19E-06
EXON1	XKR4	8	56015399/56015576/56015785/56015066/56015750	5	cg02509825/cg03751030/cg06868946/cg12751432/cg16939364	0.35407659	0.367238825	0.507349858	0.153273268	6.37E-08	8.10E-07
EXON1	XKR6	8	11058710/11058145/11058770/11058690/11058395	5	cg10947146/cg11051055/cg15301489/cg24664689/cg27487839	0.107823541	0.165539226	0.307550126	0.199726585	0.000200296	0.000989071
EXON1	ZFHX4	8	77593623	1	cg07585876	0.209150756	0.269712738	0.490332389	0.281181633	7.69E-05	0.000418614
EXON1	ZFP28	19	57050370/57050367/57050359	3	cg21970554/cg22054362/cg24152605	0.279058381	0.324144566	0.470186472	0.191128091	0.000719048	0.003080403
EXON1	ZFP57	6	29644756	1	cg12463578	0.413040475	0.281781067	0.22202345	-0.191017025	1.97E-09	4.43E-08
EXON1	ZFP82	19	36909413/36909418	2	cg24724633/cg25886284	0.162126202	0.374580514	0.543410133	0.381283931	2.69E-08	3.97E-07
EXON1	ZG16B	16	2880326	1	cg02558476	0.351762275	0.267562544	0.199170775	-0.1525915	5.03E-11	3.15E-09
EXON1	ZIC1	3	147128679/147127579/147127662/147127193/147128157/147128123/147127898	7	cg01458605/cg01610632/cg04738965/cg05073035/cg05371578/cg12595013/cg26375057	0.432710412	0.491087474	0.625479803	0.19276939	6.87E-10	1.95E-08
EXON1	ZIK1	19	58095659	1	cg26246807	0.375614655	0.597706644	0.758164927	0.382550272	1.78E-10	7.14E-09
EXON1	ZNF132	19	58951434	1	cg19776201	0.121933814	0.220579396	0.34146989	0.219536076	8.65E-05	0.00046446
EXON1	ZNF134	19	58125867	1	cg18113994	0.069991208	0.193658918	0.288664563	0.218673356	0.000268342	0.001276942
EXON1	ZNF154	19	58220370/58220494/58220516	3	cg05661282/cg21790626/cg27049766	0.477791493	0.60086215	0.697451393	0.219659901	1.32E-09	3.20E-08
EXON1	ZNF167	3	44596770	1	cg25805709	0.093279883	0.206520921	0.270121228	0.176841345	0.00706351	0.02384982

EXON1	ZNF177	19	9473696/9473715	2	cg09643544/cg12089570	0.44713019	0.535981542	0.648741052	0.201610862	1.56E-10	6.60E-09
EXON1	ZNF208	19	22193724	1	cg26938561	0.51009076	0.573753211	0.687665023	0.177574263	2.48E-11	1.98E-09
EXON1	ZNF229	19	44952618/44952572/44952604	3	cg13498858/cg17904997/cg19375479	0.209783132	0.319397329	0.421844632	0.2120615	8.90E-06	6.15E-05
EXON1	ZNF256	19	58458917/58458979	2	cg14556070/cg20176532	0.177109025	0.217259773	0.435286696	0.258177672	3.65E-05	0.000215014
EXON1	ZNF257	19	22235281/22235272/22235409	3	cg02912127/cg24072202/cg25514554	0.40164807	0.510587817	0.599723843	0.198075773	4.12E-10	1.30E-08
EXON1	ZNF264	19	57702917/57703301/57702927/57703144/57702994	5	cg08504049/cg11140785/cg22307908/cg23257840/cg24150232	0.058423139	0.122947295	0.293788536	0.235365398	0.000252406	0.001208062
EXON1	ZNF280A	22	22874447/22874590	2	cg08088989/cg20403031	0.670706765	0.544298861	0.4968019	-0.173904865	2.72E-05	0.000165227
EXON1	ZNF304	19	57863057/57862713	2	cg04685743/cg07494047	0.084922142	0.236218978	0.429114705	0.344192563	2.47E-07	2.62E-06
EXON1	ZNF311	6	28972891/28972937/28972975/28972876/28972963	5	cg12064504/cg12244205/cg14102966/cg15810199/cg18305433	0.639864042	0.531427548	0.457415673	-0.182448369	2.37E-07	2.52E-06
EXON1	ZNF331	19	54041398/54024806/54024649/54024834/54024744/54024323/54024182/54024435/54058767/54024430	10	cg05338009/cg06551490/cg09918296/cg10359230/cg10851698/cg15205507/cg16969623/cg17352157/cg19006302/cg25538376	0.319344545	0.402036101	0.517971536	0.19862699	3.73E-10	1.21E-08
EXON1	ZNF334	20	45142065/45141911	2	cg01471196/cg10705541	0.4193698	0.539774567	0.641194845	0.221825045	8.54E-09	1.52E-07
EXON1	ZNF347	19	53662261	1	cg15038664	0.083548618	0.1557514	0.332093959	0.248545341	0.000345753	0.001601531
EXON1	ZNF385D	3	21792684/21792434/21792543	3	cg00485194/cg18267381/cg18503679	0.322803982	0.428720288	0.56774422	0.244940238	1.32E-09	3.20E-08
EXON1	ZNF415	19	53636077/53636108	2	cg10332700/cg15202738	0.119376933	0.222854366	0.322519575	0.203142642	0.001037438	0.004282143
EXON1	ZNF418	19	58446600/58446669	2	cg07474494/cg11998703	0.36820772	0.488271678	0.607748914	0.239541194	1.43E-08	2.32E-07
EXON1	ZNF454	5	178368253	1	cg17840719	0.645646885	0.7032895	0.838915886	0.193269001	5.82E-11	3.47E-09
EXON1	ZNF460	19	57792200/57791959/57791887	3	cg17412580/cg23255774/cg25549230	0.102977625	0.197412435	0.256368278	0.153390654	1.20E-06	1.05E-05
EXON1	ZNF471	19	57019279	1	cg19358877	0.320278515	0.361112144	0.575129291	0.254850776	0.000274435	0.001303592
EXON1	ZNF492	19	22817176	1	cg03230154	0.249439945	0.350137439	0.5459557	0.296515755	4.46E-09	8.81E-08

EXON1	ZNF540	19	38042472/38042418	2	cg03975694/cg15415136	0.452827885	0.531260714	0.635684148	0.182856263	2.57E-08	3.81E-07
EXON1	ZNF543	19	57832235/57831909	2	cg02798548/cg04696193	0.090497869	0.197819797	0.282008214	0.191510345	0.000735246	0.003141656
EXON1	ZNF544	19	58740253/58740255	2	cg21155461/cg21747958	0.025687876	0.087893725	0.236233864	0.210545988	0.002518441	0.009478368
EXON1	ZNF549	19	58038856/58038742	2	cg07054095/cg13644629	0.160202411	0.300530641	0.464759873	0.304557462	5.76E-09	1.09E-07
EXON1	ZNF568	19	37407284/37407374/37407462/37407486/37407257/37407413	6	cg02459604/cg03060201/cg03443751/cg20154403/cg21761844/cg26612849	0.133586251	0.276222588	0.414501024	0.280914773	5.42E-05	0.000307366
EXON1	ZNF569	19	37957997/37957995	2	cg03884783/cg14142713	0.057391128	0.079105674	0.227363062	0.169971934	0.001800009	0.007055503
EXON1	ZNF578	19	52956832	1	cg25763393	0.48121531	0.540438578	0.650774995	0.169559685	3.28E-10	1.11E-08
EXON1	ZNF586	19	58281117	1	cg22956410	0.104819689	0.117110548	0.328906385	0.224086697	0.001471101	0.00587774
EXON1	ZNF606	19	58514694/58514497	2	cg12259537/cg25480379	0.4771288	0.5724297	0.663777841	0.186649041	3.34E-09	6.85E-08
EXON1	ZNF625	19	12267464	1	cg17892556	0.184477883	0.425167174	0.539363618	0.354885736	1.95E-06	1.60E-05
EXON1	ZNF660	3	44626492	1	cg22598028	0.205992665	0.316678834	0.524859862	0.318867197	8.15E-07	7.41E-06
EXON1	ZNF667	19	56988702	1	cg24732137	0.349056805	0.418095594	0.523384273	0.174327468	7.18E-09	1.31E-07
EXON1	ZNF671	19	58238987/58238850/58238928	3	cg11977686/cg12074025/cg19246110	0.284639445	0.427167596	0.614744311	0.330104865	1.96E-08	2.99E-07
EXON1	ZNF75A	16	3355553	1	cg05540369	0.223151031	0.339592739	0.496508255	0.273357224	1.39E-08	2.27E-07
EXON1	ZNF772	19	57988860	1	cg26929012	0.085492994	0.170699922	0.28206878	0.196575786	1.47E-05	9.66E-05
EXON1	ZNF773	19	58011393/58011309/58011345	3	cg00757182/cg09555914/cg22806837	0.039144991	0.141414265	0.224253849	0.185108858	0.000296824	0.001397397
EXON1	ZNF781	19	38183130/38183210/38183055	3	cg04784475/cg22521696/cg25875213	0.094544436	0.145204896	0.307763135	0.213218699	0.000261787	0.001249793
EXON1	ZNF793	19	37997867	1	cg15139588	0.098841377	0.255724284	0.475644413	0.376803036	7.56E-07	6.94E-06
EXON1	ZNF804A	2	185463132/185463209/185463583/185463588/185463218	5	cg00554682/cg13107760/cg13536757/cg16573328/cg21245277	0.093647484	0.124077633	0.269781843	0.176134359	0.000321392	0.001499716
EXON1	ZNF813	19	53971035	1	cg12142445	0.11934244	0.204465153	0.378819685	0.259477245	4.20E-05	0.00024349
EXON1	ZNF814	19	58400325/58400275	2	cg15759937/cg24124798	0.218604415	0.327995989	0.5074221	0.288817685	5.60E-07	5.30E-06
EXON1	ZNF829	19	37407060/37407041/37407152/37406932/37406949/37407123	6	cg02370417/cg03584288/cg15322783/cg15746891/cg21292152/cg24052101	0.134895251	0.273031172	0.385681899	0.250786648	5.63E-05	0.000317351
EXON1	ZNF880	19	52873173	1	cg03005603	0.264131695	0.347941017	0.448765031	0.184633336	0.00027543	0.001307847

EXON1	ZP4	1	238053828	1	cg03872376	0.62503481	0.542928461	0.428492859	-0.196541951	8.32E-08	1.03E-06
EXON1	ZSCAN18	19	58609473/58629719/58609361/58609618/58609602	5	cg06243556/cg10659886/cg21653184/cg22721334/cg25784220	0.387147531	0.451577266	0.552968708	0.165821178	2.33E-05	0.000144412
EXON1	ZSCAN23	6	28411271/28411240	2	cg06818710/cg13525197	0.218626205	0.290955486	0.471140323	0.252514118	4.50E-07	4.37E-06
EXON1	ZSCAN5B	19	56704341	1	cg18591235	0.747912715	0.6750543	0.580162764	-0.167749951	1.37E-05	9.05E-05
EXON1	ZYG11A	1	53308597	1	cg16015712	0.2988786	0.42913595	0.527121636	0.228243036	4.16E-05	0.000241805
GENEBODY	SEPT14	7	55902220/55874624	2	cg08489623/cg17007389	0.701087255	0.602111194	0.539044618	-0.162042637	1.05E-07	6.54E-07
GENEBODY	A2M	12	9231867/9227195/9265989/9227173/9265053/9225334/9265030/9227320/9227237/9227209	10	cg00141925/cg08012045/cg08300930/cg11139127/cg12187098/cg12417807/cg15688197/cg16314263/cg26665904/cg27166707	0.724781899	0.635538197	0.564690158	-0.16009174	2.34E-09	2.73E-08
GENEBODY	A2ML1	12	8995660/8994648/8994672/9019044/8995557/8995808/8986988/8995965/8995591/8975309	10	cg03314100/cg04884118/cg05589691/cg11641391/cg13209739/cg15384867/cg21416544/cg24309769/cg27629124/cg27653134	0.70627356	0.605035131	0.520051112	-0.186222448	3.99E-11	1.32E-09
GENEBODY	ABCA8	17	66925642/66926513/66905630	3	cg17334372/cg17461670/cg20561863	0.372540965	0.264106356	0.175743455	-0.19679751	1.79E-10	3.73E-09
GENEBODY	ABCC13	21	15647368/15646187/15646635/15646312	4	cg01335663/cg05202042/cg11429658/cg12162424	0.59325333	0.516750706	0.441786923	-0.151466407	3.97E-08	2.83E-07
GENEBODY	ABCD2	12	39981136	1	cg13406085	0.717780775	0.565277444	0.388307936	-0.329472839	2.09E-12	2.35E-10
GENEBODY	ABHD1	2	27346938/27349220	2	cg03315230/cg17345276	0.531101125	0.411027406	0.331153189	-0.199947936	8.02E-12	4.83E-10
GENEBODY	ACSM5	16	20429506	1	cg27514874	0.76220994	0.691020928	0.610951877	-0.151258063	3.02E-05	9.91E-05
GENEBODY	ACTA1	1	229568077/229567560/229568252/229567914/229568360/229568467/229567626	7	cg00034468/cg02970384/cg04568492/cg05353133/cg11668749/cg12341314/cg24104241	0.417363278	0.493218573	0.628459703	0.211096426	3.66E-10	6.27E-09
GENEBODY	ACY3	11	67414373	1	cg23879118	0.339937625	0.226226498	0.17070377	-0.169233855	2.21E-08	1.72E-07

GENEBODY	ADAD1	4	123301310/123304626/123326231/123301256	4	cg16264330/cg17139861/cg20603162/cg22162821	0.576242355	0.491466206	0.385902736	-0.190339619	1.33E-11	6.59E-10
GENEBODY	ADAMTS12	5	33890324/33737936/33842297/33659084/33852528/33835713/33788976/33794720/33889461/33649717/33772916/33758285/33822930/33807278/33832949/33855241/33727007/33764247/33563927/33809376/33698653	21	cg01990593/cg03592903/cg04578894/cg06448603/cg06893139/cg07043044/cg07700393/cg07784793/cg08768395/cg10594543/cg10627511/cg12917072/cg14827469/cg15833353/cg19641747/cg21569398/cg21874902/cg23359363/cg24777564/cg26077446/ch.5.731560F	0.593019341	0.505387449	0.436439844	-0.156579497	8.34E-11	2.13E-09
GENEBODY	ADCYAP1	18	908130/908745/907092/905450/906224/909086/908505/908396/909154/907605/905611/906020/908290/906726	14	cg00067606/cg04105966/cg06372303/cg07376535/cg07788286/cg10384245/cg11402363/cg11771234/cg11859607/cg13940693/cg14489474/cg17439660/cg21331088/cg22374233	0.319629228	0.409173016	0.550701367	0.23107214	1.63E-09	2.01E-08
GENEBODY	ADD2	2	70932722/70933326/70933416/70903803/70933502/70931257/70915210	7	cg02674324/cg06752779/cg09364770/cg13284981/cg13998281/cg17075459/cg18067420	0.685027895	0.609449547	0.519611965	-0.16541593	2.32E-10	4.53E-09
GENEBODY	ADORA2A	22	24829716/24829462	2	cg19855777/cg21584430	0.68218459	0.5944658	0.523996377	-0.158188213	4.04E-11	1.33E-09
GENEBODY	ADRA1A	8	26717875/26720076/26688088/26627454/26681894/26614263/26720681/26687962	8	cg02501086/cg02776627/cg07572251/cg10241718/cg14825340/cg16895486/cg23289779/cg23328194	0.614074213	0.544911369	0.454862399	-0.159211814	1.79E-10	3.73E-09
GENEBODY	AGR2	7	16841311	1	cg25343204	0.59904114	0.490179217	0.366087627	-0.232953513	3.57E-09	3.81E-08
GENEBODY	AKR7L	1	19596472/19599516/19600141	3	cg03985478/cg17431922/cg23625458	0.701048328	0.59951272	0.541531723	-0.159516606	5.17E-06	2.03E-05

GENEBODY	AKT3	1	243723585/243877024/244006288/244006397/244004889/243670224/243754323/243904118/243663227/243877447/243902978/243712954/243736307/243967381/243869070	15	cg02288831/cg04221461/cg11314684/cg11496569/cg11973514/cg13396060/cg15604398/cg15962538/cg16404569/cg17133045/cg20686125/cg22629515/cg24455383/cg24739596/cg27438690	0.682528058	0.600768571	0.5141943	-0.168333758	3.71E-13	8.58E-11
GENEBODY	ALDOA	16	30080799/30079121	2	cg01667953/cg26519342	0.737584385	0.646764039	0.550730859	-0.186853526	1.28E-06	5.96E-06
GENEBODY	ANKDD1A	15	65204391/65214165/65239308/65214075/65204428/65223141/65204370/65214588/65207109	9	cg00958451/cg01497527/cg02539274/cg03775123/cg05527113/cg09291474/cg10097464/cg12119988/cg21153004	0.611957014	0.527110344	0.444063916	-0.167893098	8.25E-11	2.11E-09
GENEBODY	ANXA2	15	60689285/60654693/60688010/60689670/60644157/60683059/60687284/60690026/60688948/60662488/60664570/60689852/60688622/60666575/60673898	15	cg02072495/cg03957109/cg06738887/cg09533293/cg09785377/cg11681321/cg13313836/cg13634501/cg14397690/cg17893775/cg21711008/cg22293140/cg22365276/cg22581200/cg25515997	0.553756335	0.469198306	0.39381976	-0.159936574	6.17E-12	4.14E-10
GENEBODY	AOC2	17	41001944/41001849	2	cg16048817/cg22530519	0.5593076	0.45642565	0.385593986	-0.173713614	1.03E-10	2.48E-09
GENEBODY	APOBEC3H	22	39496387	1	cg17972162	0.544766345	0.466363289	0.372024159	-0.172742186	2.77E-10	5.12E-09
GENEBODY	APOC1P1	19	45430113	1	cg24084606	0.640969445	0.579160056	0.486872786	-0.154096659	4.93E-07	2.57E-06
GENEBODY	APOH	17	64225346	1	cg17095279	0.621719185	0.494591128	0.393896214	-0.227822971	3.60E-11	1.24E-09
GENEBODY	APOL5	22	36123779	1	cg25036527	0.61314906	0.455815106	0.348639441	-0.264509619	5.92E-11	1.69E-09

GENEBODY	APP	21	27540106/27542749/27372396/27541894/27542843/27538021/27372461/27540728/27540220/27372446/27372387/27354743/27297434	13	cg01286133/cg10129201/cg11321156/cg12144476/cg13169373/cg14414154/cg18274664/cg18597421/cg19788250/cg23269692/cg24168308/cg25314245/ch.21.228978R	0.539598548	0.447073257	0.385659041	-0.153939506	9.85E-13	1.45E-10
GENEBODY	ARHGAP15	2	144223682/144246478/144462886/144359756/144282743/144500981/144503739/144501227/144303521/144448686/143927903/144271431/144477916/144053418/144234430/144515038/144105257/144486573/143987421/143999025/144099197/144498168/144519856	23	cg00016324/cg00859755/cg05307752/cg07211155/cg07409629/cg07539108/cg07621645/cg08820401/cg11310863/cg12058385/cg14851140/cg17056048/cg17592268/cg17600630/cg19867914/cg21291880/cg21338523/cg21339799/cg22922100/cg23498755/cg25026949/cg27455061/cg27492839	0.732609214	0.645983516	0.565320411	-0.167288803	3.99E-10	6.72E-09
GENEBODY	ARL5C	17	37318180/37321490/37321890/37321631/37321201	5	cg11090211/cg15790037/cg16202734/cg20555674/cg26465214	0.329569929	0.415841338	0.486067192	0.156497263	9.70E-08	6.13E-07
GENEBODY	ARRDC5	19	4891238/4891420/4894976/4902576/4891192/4891471/4891305	7	cg07755245/cg08364410/cg08873568/cg10031203/cg16117653/cg18859211/cg20230308	0.808635148	0.716852569	0.597540458	-0.211094691	2.68E-10	5.00E-09
GENEBODY	ASB17	1	76396165/76397847	2	cg15990658/cg17041296	0.736653898	0.680856442	0.547862298	-0.1887916	2.95E-07	1.63E-06
GENEBODY	ASCC2	22	30195299	1	cg14742445	0.623584125	0.427142461	0.297950882	-0.325633243	5.71E-14	3.80E-11
GENEBODY	ASCL3	11	8959438	1	cg14711859	0.801660675	0.6463918	0.528616877	-0.273043798	1.08E-09	1.43E-08
GENEBODY	ATP13A5	3	193078051/193066097/193096009	3	cg20118157/cg24671330/cg25013044	0.80682305	0.735072519	0.652345489	-0.154477561	3.95E-09	4.14E-08

GENEBODY	ATP1B2	17	7555322/7555132/7559144/7555411/7558889/7559041/7555298	7	cg05070518/cg08581937/cg16991298/cg17379325/cg19178081/cg20682573/cg22237200	0.353785818	0.398109056	0.51723176	0.163445942	1.54E-05	5.37E-05
GENEBODY	ATP6AP1L	5	81608485	1	cg07807695	0.621896035	0.474271061	0.403411523	-0.218484512	7.68E-11	2.00E-09
GENEBODY	ATP6V1G3	1	198509669	1	cg12958813	0.820138695	0.625598444	0.495645964	-0.324492731	2.74E-09	3.08E-08
GENEBODY	AXIN2	17	63537711/63532630/63530270/63534625/63553581/63530161/63535223/63554468/63532898/63550766/63534470/63533778/63535567/63554536/63533152/63534340/63549322/63549924/63545705/63527924/63543769/63534688/63533303	23	cg01434611/cg01982533/cg02161011/cg04239967/cg04293307/cg06136432/cg06405341/cg06875131/cg08005093/cg09119854/cg09231741/cg10965669/cg11096905/cg11122009/cg12255061/cg18956355/cg19949591/cg22809682/cg23011419/cg23475474/cg25661571/cg26921093/cg27527595	0.660607046	0.52527306	0.445998522	-0.214608523	6.73E-14	4.17E-11
GENEBODY	AZU1	19	828170/831456	2	cg17823175/cg24165638	0.596399873	0.504322528	0.428856182	-0.167543691	4.43E-13	9.38E-11
GENEBODY	B3GNT5	3	182987901	1	cg16262284	0.67505214	0.527117333	0.415287586	-0.259764554	2.23E-13	6.76E-11
GENEBODY	BBOX1	11	27145585/27097023/27105814	3	cg03058733/cg06847828/cg24585203	0.628308817	0.541562856	0.476988902	-0.151319915	2.14E-06	9.29E-06
GENEBODY	BCAR4	16	11922606	1	cg05694761	0.871228205	0.798105094	0.682879855	-0.18834835	1.41E-04	3.99E-04
GENEBODY	BCL2L15	1	114429815	1	cg12486521	0.62545038	0.505361367	0.404165823	-0.221284557	8.98E-11	2.24E-09
GENEBODY	BIK	22	43523016/43523322	2	cg02601893/cg03015585	0.540223563	0.434473208	0.357581439	-0.182642124	6.28E-12	4.17E-10
GENEBODY	BNC1	15	83951913/83952345/83952774/83952875/83953068/83952019/83951663/83952722/83951732/83952508/83949772/83952808/83952420	13	cg00222341/cg02318926/cg04090392/cg07639720/cg08939095/cg10398682/cg12250049/cg14431006/cg15736165/cg17051321/cg17151123/cg19988449/cg23989963	0.336788053	0.436541103	0.568156758	0.231368705	5.14E-11	1.55E-09
GENEBODY	BNIPL	1	151019134/151009588	2	cg04244851/cg11584936	0.86080939	0.759247922	0.646079755	-0.214729635	1.82E-06	8.04E-06

GENEBODY	BPI	20	36933189	1	cg08254089	0.48949337	0.339998589	0.210791691	-0.278701679	5.96E-13	1.10E-10
GENEBODY	BPIL3	20	31619647	1	cg18223379	0.690191385	0.52916155	0.452184077	-0.238007308	6.80E-08	4.50E-07
GENEBODY	BSX	11	122851122/122848983/122849037/122850545/122850972/122848518/122850522/122850140/122850741/122850227/122850490	11	cg00408348/cg02851062/cg03812107/cg06191454/cg11718501/cg12114834/cg15503722/cg16808373/cg17121178/cg22289581/cg24719321	0.371464051	0.477866572	0.617852922	0.24638887	3.54E-10	6.15E-09
GENEBODY	BTF3L1	13	77502805	1	cg01665118	0.446121545	0.319807511	0.243037727	-0.203083818	1.56E-11	7.32E-10
GENEBODY	BTG3	21	18980308	1	cg08875503	0.56439063	0.466887072	0.366370541	-0.198020089	3.26E-10	5.77E-09
GENEBODY	BTG4	11	111364291	1	cg23756005	0.600384555	0.465027928	0.357941832	-0.242442723	9.96E-12	5.39E-10
GENEBODY	C10orf50	10	26883013/26883079/26883165/26881860	4	cg22822446/cg23318538/cg23378142/cg25037043	0.512032235	0.433760739	0.344742057	-0.167290178	2.88E-10	5.24E-09
GENEBODY	C11orf36	11	3240365/3241424/3240201/3239953/3243069/3243891/3243281/3240399/3240068/3240122	10	cg01738984/cg02082210/cg02681040/cg04656692/cg08093323/cg17957235/cg18169940/cg24038255/cg24702253/cg26462595	0.578796143	0.50031766	0.416960611	-0.161835532	2.94E-12	2.73E-10
GENEBODY	C11orf52	11	111796862/111794346	2	cg12247500/cg15318568	0.579033225	0.484909567	0.405061727	-0.173971498	1.86E-08	1.50E-07
GENEBODY	C11orf53	11	111154742/111155021/111155888/111155014/111154375/111154934/111154826/111154754	8	cg03078767/cg03554573/cg06246444/cg06978117/cg14675241/cg18246298/cg21127079/cg23091777	0.557567098	0.472839572	0.390458011	-0.167109087	2.37E-10	4.59E-09
GENEBODY	C12orf39	12	21680848/21679634/21680409/21680669/21680426/21681006/21680508/21680981/21680220	9	cg02125614/cg02393721/cg05161082/cg06241792/cg08347047/cg08828403/cg10437806/cg15308062/cg19499361	0.084036882	0.137582897	0.260407689	0.176370807	0.000148248	0.000417128
GENEBODY	C12orf53	12	6807082	1	cg12051956	0.476141025	0.337174722	0.252782268	-0.223358757	5.72E-12	3.96E-10
GENEBODY	C13orf29	13	111521981/111522314/111522651/111522222	4	cg03757805/cg04513422/cg13698168/cg16032841	0.564229618	0.5081458	0.358088846	-0.206140771	1.23E-09	1.60E-08

GENEBODY	C13orf39	13	103345722/103340215	2	cg03972040/cg07951201	0.674567425	0.59340735	0.480139823	-0.194427602	2.27E-10	4.46E-09
GENEBODY	C14orf105	14	57952160	1	cg20469764	0.74590461	0.661443183	0.580634459	-0.165270151	5.24E-05	0.000162929
GENEBODY	C14orf139	14	95875792	1	cg10145926	0.512898735	0.398599789	0.321036291	-0.191862444	1.57E-10	3.39E-09
GENEBODY	C14orf23	14	29254853/29247148/29242823/29244153/29247336/29243234/29243762/29255119/29250912/29243173/29254530/29254961/29247605/29254680/29254942/29247396/29258014/29256427/29243504/29244185/29247458/29244278/29243622/29243404/29242040/29253725/29243690/29247705	28	cg01302656/cg02008651/cg02273846/cg03505995/cg03796729/cg04184232/cg04991036/cg05119057/cg05374809/cg06779110/cg09241022/cg11226913/cg12967137/cg13046832/cg14006181/cg15482122/cg15506863/cg16733558/cg17007640/cg18875371/cg20043105/cg21195256/cg21542248/cg22337128/cg22400166/cg23059304/cg23302649/cg24154325	0.339200707	0.419109893	0.541993803	0.202793095	3.11E-09	3.43E-08
GENEBODY	C14orf37	14	58567637/58528263/58543900/58599910/58599692/58499562	6	cg01533082/cg02128489/cg05939089/cg15817440/cg16499677/cg23642334	0.715069617	0.610660754	0.552156811	-0.162912806	1.05E-12	1.51E-10
GENEBODY	C14orf38	14	60001474/60043289/60040732/60032921/60032188/60032511	6	cg13059113/cg14428252/cg21074911/cg23984976/cg25066134/cg27143664	0.506751613	0.419323883	0.34797005	-0.158781563	6.59E-12	4.32E-10
GENEBODY	C15orf48	15	45724277/45724580	2	cg20636382/cg23505252	0.621534738	0.484264992	0.39664795	-0.224886788	1.06E-11	5.65E-10
GENEBODY	C15orf59	15	74037743/74040580/74033803	3	cg16900255/cg17284334/cg22843146	0.477220277	0.40070898	0.318695352	-0.158524925	3.48E-09	3.74E-08
GENEBODY	C16orf11	16	615073/613632/614986/614645/613343/613526/614852	7	cg00131705/cg01435178/cg05389958/cg06951750/cg09476347/cg16452248/cg27409401	0.834766665	0.719300422	0.637916636	-0.196850029	1.77E-11	7.93E-10
GENEBODY	C16orf92	16	30034801	1	cg05034471	0.71218241	0.639790444	0.519420723	-0.192761687	2.09E-07	1.20E-06

GENEBODY	C17orf106	17	73996245/73993843/73996331	3	cg07471052/cg11061343/cg14040633	0.576958515	0.441725786	0.371846536	-0.205111979	5.75E-11	1.65E-09
GENEBODY	C17orf46	17	43339328/43335632/43339358/43339040/43339223	5	cg04992638/cg05892673/cg08247376/cg11933779/cg13001868	0.342498056	0.440215596	0.53911391	0.196615854	1.12E-05	4.03E-05
GENEBODY	C17orf73	17	48844124/48841566	2	cg03016571/cg12881765	0.589294773	0.468265725	0.3843354	-0.204959373	1.40E-11	6.85E-10
GENEBODY	C18orf20	18	61751615	1	cg06069685	0.601641725	0.505239633	0.424268005	-0.17737372	3.47E-10	6.04E-09
GENEBODY	C18orf26	18	52262555	1	cg01174441	0.704881995	0.533918367	0.387608936	-0.317273059	1.88E-12	2.17E-10
GENEBODY	C19orf26	19	1230988/1235093/1231165/1231292/1233232/1235003	6	cg01297744/cg03284113/cg12062088/cg19058262/cg23357435/cg27594157	0.664054918	0.560682328	0.506711187	-0.157343731	1.64E-09	2.03E-08
GENEBODY	C19orf35	19	2278451/2276371/2278708/2278773/2275741/2280748/2278847/2276322/2276471/2278618	10	cg01985343/cg07352691/cg07595134/cg08399733/cg10627703/cg14744050/cg17437218/cg21449569/cg26472036/cg27341866	0.600029579	0.507751294	0.437974555	-0.162055024	1.35E-10	3.05E-09
GENEBODY	C19orf38	19	10978833/10980000	2	cg11354682/cg27589572	0.81887869	0.745483894	0.657669527	-0.161209163	8.53E-06	3.18E-05
GENEBODY	C1orf129	1	170964477	1	cg03458344	0.441931635	0.347910817	0.225445495	-0.21648614	2.13E-10	4.25E-09
GENEBODY	C1orf158	1	12806667	1	cg24338843	0.782470565	0.695402917	0.498194609	-0.284275956	8.21E-08	5.29E-07
GENEBODY	C1orf168	1	57263497/57265449	2	cg09684245/cg20704530	0.53133186	0.450454467	0.340683705	-0.190648155	1.29E-10	2.93E-09
GENEBODY	C1orf180	1	85100313/85098598	2	cg11387751/cg25388882	0.62865284	0.476172272	0.39536335	-0.23328949	1.47E-09	1.84E-08
GENEBODY	C1orf186	1	206240661	1	cg23738708	0.74506173	0.682724617	0.57851695	-0.16654478	4.93E-05	0.000154212
GENEBODY	C1orf226	1	162352023/162351057/162351367/162351976/162351664	5	cg11342789/cg15492834/cg19678111/cg21547763/cg25212701	0.49387937	0.378519667	0.298744819	-0.195134551	4.87E-12	3.69E-10
GENEBODY	C1orf49	1	178488431	1	cg07961456	0.557506865	0.463849467	0.392401091	-0.165105774	5.05E-11	1.54E-09
GENEBODY	C1QL2	2	119914744/119914553/119915139	3	cg00820718/cg07090701/cg11873147	0.277001978	0.364477144	0.50214335	0.225141373	6.54E-08	4.35E-07
GENEBODY	C1QTNF4	11	47612070/47611780	2	cg05537653/cg18356785	0.258025811	0.411925617	0.491033436	0.233007626	2.43E-10	4.64E-09
GENEBODY	C20orf123	20	45170254/45174465/45170494	3	cg12288076/cg22094045/cg24550212	0.70718541	0.572817228	0.535750782	-0.171434628	0.000123932	0.000354308
GENEBODY	C20orf196	20	5829461	1	cg10547761	0.530999825	0.393510911	0.294486191	-0.236513634	8.06E-14	4.51E-11

GENEBODY	C20orf197	20	58645949	1	cg06347896	0.63527504	0.528443756	0.434222573	-0.201052467	1.67E-06	7.49E-06
GENEBODY	C20orf94	20	10492319/10508668/10492394/10500546	4	cg02861178/cg07941953/cg23558337/ch.20.230983F	0.243657374	0.127434121	0.091783265	-0.151874109	1.59E-08	1.32E-07
GENEBODY	C21orf131	21	22175001	1	cg09614626	0.750141095	0.645449372	0.468220914	-0.281920181	2.19E-08	1.71E-07
GENEBODY	C21orf84	21	44898090	1	cg23732182	0.49169906	0.4092034	0.3327424	-0.15895666	5.44E-11	1.60E-09
GENEBODY	C21orf90	21	45937406/45937201	2	cg04289880/cg06399419	0.52138309	0.403219428	0.312947382	-0.208435708	1.25E-09	1.62E-08
GENEBODY	C22orf15	22	24105692	1	cg10756887	0.455853575	0.558876878	0.609118645	0.15326507	2.60E-09	2.97E-08
GENEBODY	C22orf25	22	20050298/20044371	2	cg03175417/cg04419754	0.852329493	0.756382203	0.7000014	-0.152328093	1.60E-11	7.40E-10
GENEBODY	C2CD4D	1	151810904/151810893/151810899/151811364/151810887/151810586/151810589/151810485	8	cg04296699/cg05021743/cg10781408/cg12749132/cg15015892/cg19255477/cg22604218/cg26426745	0.285316502	0.391937868	0.504602615	0.219286113	8.21E-10	1.16E-08
GENEBODY	C4BPB	1	207271346	1	cg19707677	0.49020137	0.351117572	0.257840991	-0.232360379	1.68E-13	6.13E-11
GENEBODY	C4orf17	4	100434274	1	cg15411303	0.54089219	0.414874839	0.327330295	-0.213561895	1.01E-08	8.97E-08
GENEBODY	C4orf37	4	99064102/98690072/99061101/99008595/99031533	5	cg03676636/cg08746785/cg09681278/cg11579222/cg11861579	0.660260377	0.57946456	0.490608621	-0.169651756	3.29E-10	5.82E-09
GENEBODY	C4orf47	4	186356202	1	cg13054212	0.854377895	0.731720367	0.568434236	-0.285943659	2.71E-11	1.06E-09
GENEBODY	C4orf49	4	140200520/140201100/140200723/140201065/140200658	5	cg00208504/cg01733638/cg05924540/cg07776847/cg13272465	0.150081319	0.244701395	0.399377023	0.249295704	1.60E-08	1.33E-07
GENEBODY	C5orf38	5	2753746/2753375/2754274/2754016/2753928/2753876/2752601/2753852/2754074/2754148/2754753/2755004/2754894/2752882/2754240/2754322	16	cg02509058/cg03570636/cg04509074/cg10958362/cg12824782/cg12860686/cg13702053/cg13944468/cg18652721/cg18693345/cg21629500/cg21999939/cg23164183/cg26504021/cg26932441/cg27144115	0.243845646	0.330042624	0.442586406	0.19874076	2.41E-07	1.37E-06
GENEBODY	C5orf4	5	154214618	1	cg02379533	0.42599846	0.343909439	0.275215377	-0.150783083	9.22E-11	2.27E-09
GENEBODY	C5orf58	5	169663955	1	cg17038633	0.812719335	0.70161615	0.531221877	-0.281497458	3.90E-09	4.09E-08

GENEBODY	C6	5	41160373/41152767	2	cg02598319/cg16582255	0.664062255	0.508529911	0.424378073	-0.239684182	1.43E-10	3.17E-09
GENEBODY	C6orf138	6	48030011/47890537/48033856	3	cg04421392/cg09219575/cg16000300	0.62871339	0.547888969	0.471015939	-0.157697451	1.68E-05	5.82E-05
GENEBODY	C6orf58	6	127911362	1	cg03346692	0.328331685	0.231389494	0.173702295	-0.15462939	1.81E-08	1.46E-07
GENEBODY	C7orf16	7	31745454	1	cg23110407	0.7770301	0.720457139	0.58055675	-0.19647335	1.98E-05	6.77E-05
GENEBODY	C7orf33	7	148303238/148297082	2	cg04464504/cg09843869	0.754527008	0.640737833	0.53003033	-0.224496678	2.22E-08	1.73E-07
GENEBODY	C7orf42	7	66417818	1	cg18921475	0.509465275	0.397975594	0.294769914	-0.214695361	8.09E-12	4.83E-10
GENEBODY	C7orf44	7	43686889	1	cg20741105	0.566863025	0.682208033	0.753825405	0.18696238	7.74E-12	4.73E-10
GENEBODY	C7orf45	7	129850238/129849144	2	cg05423713/cg23739525	0.613656165	0.527664822	0.422489877	-0.191166288	4.41E-11	1.40E-09
GENEBODY	C7orf53	7	112124979	1	cg19543867	0.616641305	0.495213811	0.351521768	-0.265119537	3.48E-10	6.05E-09
GENEBODY	C7orf66	7	108524200	1	cg21462681	0.755043265	0.636153822	0.435511264	-0.319532001	3.11E-09	3.43E-08
GENEBODY	C8A	1	57320685/57368084/57376284	3	cg16648841/cg19926245/cg22877129	0.650853318	0.606787242	0.490298814	-0.160554504	2.22E-07	1.27E-06
GENEBODY	C8B	1	57420485	1	cg13047196	0.58937025	0.4685176	0.36754115	-0.2218291	3.23E-09	3.52E-08
GENEBODY	C8orf45	8	67813257/67806112	2	cg15903955/cg19407435	0.522605355	0.4381076	0.319413614	-0.203191741	4.06E-09	4.24E-08
GENEBODY	C8orf56	8	104152279/104153148/104145585/104152750/104153173/104152588/104152754/104152244/104149619	9	cg02389252/cg06399148/cg06450952/cg10163483/cg10918202/cg11318251/cg14284952/cg14884291/cg24633771	0.187019399	0.260934758	0.407430775	0.220411376	7.62E-06	2.88E-05
GENEBODY	C9orf153	9	88844429	1	cg14325674	0.61069315	0.509280833	0.412591941	-0.198101209	3.71E-09	3.93E-08
GENEBODY	C9orf173	9	140147188	1	cg21174903	0.51807392	0.402703261	0.339554973	-0.178518947	9.95E-09	8.86E-08
GENEBODY	C9orf70	9	3899055	1	cg14269813	0.515813755	0.299383833	0.253730073	-0.262083682	6.28E-11	1.76E-09
GENEBODY	C9orf75	9	140092222	1	cg13851744	0.56522085	0.458802578	0.364773245	-0.200447605	1.50E-10	3.28E-09
GENEBODY	C9orf79	9	90498981/90498223	2	cg14331104/cg27495908	0.703789208	0.652843361	0.546248795	-0.157540412	2.82E-06	1.18E-05
GENEBODY	CA11	19	49141420/49143404	2	cg15172844/cg15967501	0.71194344	0.631728272	0.538537041	-0.173406399	6.64E-06	2.55E-05
GENEBODY	CABYR	18	21737584/21725731	2	cg05926667/cg19966472	0.75140954	0.683161289	0.584597436	-0.166812104	3.98E-05	0.000126958

GENEBODY	CACNA2D1	7	82019862/81996559/81809719/82004187/81746161/81977646/81638451/81752694/82072223/81638368/81792131/82071752/82072141/81637024/81666278/81935073/81601173/81964885/82057703/81949245/82072332/81679070/81860423/81805241/82066996	25	cg00145985/cg00704369/cg00712593/cg01454305/cg02857074/cg03771436/cg06559389/cg07535316/cg08992818/cg09270031/cg10030980/cg10369665/cg11581706/cg12536527/cg13420207/cg13558509/cg14363242/cg14607894/cg16867817/cg17294136/cg20288892/cg24426822/cg25585043/cg26613778/cg27470827	0.561184771	0.472513066	0.381804078	-0.179380693	3.54E-13	8.43E-11
GENEBODY	CALCOCO2	17	46919180	1	cg02753187	0.407011215	0.524217861	0.601138023	0.194126808	5.60E-09	5.52E-08
GENEBODY	CAMK1G	1	209780966	1	cg20994118	0.395489065	0.312407689	0.21479675	-0.180692315	8.17E-13	1.29E-10
GENEBODY	CANT1	17	76993394/76989832/76989680/76993272/76991208/76993506/76993374/76989965/76993698/76991378/76991253/76993618	12	cg00902147/cg10976778/cg16337457/cg17129554/cg17838182/cg18186771/cg22687077/cg23225637/cg23507761/cg23911097/cg25052374/cg26055488	0.782328371	0.690642685	0.600940522	-0.181387849	5.39E-08	3.67E-07
GENEBODY	CAP2	6	17492721/17470162/17472892/17519387/17505703	5	cg06573088/cg16709403/cg22753611/cg23036213/cg25416270	0.656450298	0.576869611	0.485568008	-0.17088229	2.90E-08	2.17E-07
GENEBODY	CAPN11	6	44134066/44144029	2	cg06432342/cg22798503	0.856237165	0.779400136	0.689293332	-0.166943833	7.52E-08	4.92E-07
GENEBODY	CARTPT	5	71016343/71015523/71015432	3	cg14999396/cg18250028/cg23300372	0.590938803	0.671535867	0.742013114	0.151074311	8.91E-08	5.70E-07
GENEBODY	CBLN2	18	70206471/70206925/70208357/70208064	4	cg03521982/cg19181692/cg24956253/cg27373591	0.60105974	0.530936726	0.413174638	-0.187885102	2.61E-09	2.97E-08
GENEBODY	CBWD6	9	69260795	1	cg01119453	0.815019895	0.723716361	0.651987968	-0.163031927	8.96E-06	3.32E-05
GENEBODY	CCDC162	6	109615579	1	cg00509249	0.789235055	0.642713406	0.527462918	-0.261772137	1.98E-10	4.00E-09

GENEBODY	CCDC28B	1	32669150/32669293/32670695/32670486	4	cg01235983/cg12462377/cg19721787/cg23885562	0.623867668	0.519440442	0.464291014	-0.159576654	5.93E-10	9.10E-09
GENEBODY	CCDC52	3	113213220/113217290	2	cg10572188/cg19892944	0.609257035	0.495454989	0.442251141	-0.167005894	1.06E-07	6.62E-07
GENEBODY	CCDC67	11	93082066/93143810/93067324	3	cg09919809/cg21115391/cg21567022	0.619897248	0.538317674	0.451682661	-0.168214588	2.08E-08	1.64E-07
GENEBODY	CCDC79	16	66789395	1	cg07722333	0.67643985	0.604216611	0.481075127	-0.195364723	9.09E-10	1.25E-08
GENEBODY	CCDC83	11	85601106/85596390	2	cg07092880/cg24166175	0.821923575	0.752734567	0.669314845	-0.15260873	1.22E-06	5.72E-06
GENEBODY	CCK	3	42304966/42301921/42304070/42305063	4	cg06085877/cg13427828/cg15778433/cg21327694	0.186010025	0.240556373	0.392592868	0.206582843	0.000113774	0.000327454
GENEBODY	CCRL2	3	46449636/46449313	2	cg14749678/cg16021018	0.437872943	0.360796239	0.284994182	-0.152878761	4.62E-11	1.44E-09
GENEBODY	CD1B	1	158301038/158300733	2	cg04574507/cg15952487	0.51078378	0.358514756	0.2767323	-0.23405148	2.06E-13	6.68E-11
GENEBODY	CD1E	1	158325254/158324331/158324471	3	cg09664215/cg12200412/cg26014409	0.614816155	0.483412517	0.376173212	-0.238642943	3.32E-12	2.99E-10
GENEBODY	CD200R1	3	112692897/112647810	2	cg10708271/cg17069590	0.665344945	0.536198383	0.430932968	-0.234411977	6.51E-10	9.74E-09
GENEBODY	CD300E	17	72619556	1	cg04995095	0.639208915	0.586559739	0.457235768	-0.181973147	7.31E-05	0.000219864
GENEBODY	CD34	1	208084255/208084030/208083913/208063733/208084071/208081541/208084099	7	cg01566217/cg03990033/cg04197452/cg09788693/cg16291276/cg21697512/cg27566858	0.434061102	0.511811157	0.605030287	0.170969185	2.90E-08	2.17E-07
GENEBODY	CD36	7	80291794/80291832	2	cg02325411/cg05345249	0.57524186	0.486998328	0.420398886	-0.154842974	1.07E-09	1.42E-08
GENEBODY	CD8A	2	87012810/87017419/87018382/87018104/87016436/87017591/87014979/87016720/87016129/87017689/87017953/87015813/87016259/87018054/87018585	15	cg00219921/cg00916536/cg02170525/cg05163496/cg07152196/cg09046939/cg11423206/cg12653796/cg13803976/cg13946520/cg17108819/cg24345747/cg26057751/cg27247697/cg27502457	0.279459625	0.344021236	0.444266894	0.164807269	3.51E-06	1.44E-05

GENEBODY	CDK14	7	90595609/90664816/90393740/90346445/90454617/90355905/90673305/90581938/90670577/90682867/90406191/90339132/90630110/90390136/90533507/90562815	16	cg05467568/cg06745145/cg07521988/cg08583610/cg09314495/cg09781028/cg14139051/cg19617217/cg20411384/cg20499825/cg21700774/cg21743182/cg21769057/cg25150238/cg25619459/cg27193735	0.706679665	0.62652391	0.534418827	-0.172260838	1.79E-12	2.11E-10
GENEBODY	CEACAM18	19	51984816	1	cg03110722	0.537041	0.449763111	0.386227418	-0.150813582	4.65E-08	3.23E-07
GENEBODY	CEACAM21	19	42083748	1	cg10389454	0.65132238	0.542951506	0.491541864	-0.159780516	2.27E-05	7.66E-05
GENEBODY	CEACAM3	19	42300812	1	cg23181133	0.51520107	0.3874973	0.322241255	-0.192959815	2.12E-11	8.96E-10
GENEBODY	CEACAM5	19	42221239/42213763/42213020/42219575/42221417/42219138	6	cg02768390/cg17794784/cg21505334/cg22808163/cg23351290/cg26257634	0.624930584	0.539440044	0.453361965	-0.171568619	1.23E-09	1.59E-08
GENEBODY	CEACAM6	19	42260485	1	cg26813458	0.66001207	0.575946272	0.500648891	-0.159363179	2.63E-07	1.47E-06
GENEBODY	CEND1	11	788180/788406	2	cg02554274/cg24193659	0.515985763	0.396734322	0.324084141	-0.191901622	4.67E-14	3.73E-11
GENEBODY	CES3	16	66995461	1	cg09407859	0.644195175	0.501121989	0.384668623	-0.259526552	5.16E-12	3.73E-10
GENEBODY	CFH	1	196702931/196624484	2	cg05922384/cg06377993	0.71928739	0.629771844	0.529944805	-0.189342585	4.67E-05	0.000146997
GENEBODY	CFHR1	1	196795943	1	cg07209298	0.44851971	0.333937817	0.247106627	-0.201413083	4.60E-11	1.44E-09
GENEBODY	CFHR2	1	196923877	1	cg09753006	0.48501923	0.369925206	0.264522118	-0.220497112	1.39E-13	5.63E-11
GENEBODY	CFHR5	1	196976319	1	cg26049187	0.55615472	0.426080089	0.383495009	-0.172659711	0.000192924	0.000529777
GENEBODY	CHIT1	1	203186092/203192720/203192517/203192642	4	cg15209419/cg17591915/cg23683497/cg25732687	0.69525625	0.571828991	0.502249609	-0.193006641	4.58E-09	4.66E-08
GENEBODY	CHODL	21	19617873/19618088	2	cg11433319/cg17040471	0.40303238	0.512811294	0.693392332	0.290359952	3.63E-11	1.24E-09
GENEBODY	CHRNA6	8	42611544/42619185/42608976	3	cg06292444/cg16417043/cg19352832	0.708599585	0.655779078	0.556684536	-0.151915049	0.000315481	0.000827191
GENEBODY	CHST10	2	101015494/101014098/101009789/101010769/101009928/101010037/101014002	7	cg05141465/cg12002119/cg12338127/cg18406852/cg18845236/cg20147389/cg21336456	0.842579298	0.731185044	0.618323625	-0.224255673	5.48E-10	8.56E-09

GENEBODY	CHST2	3	142840240/142839991/142839903/142840129/142839837/142840837	6	cg14473344/cg17373442/cg18507129/cg22536150/cg23749856/cg26399662	0.347364705	0.438624984	0.604279828	0.256915123	4.10E-11	1.33E-09
GENEBODY	CIB3	19	16272369	1	cg02870741	0.66539429	0.5682174	0.468801568	-0.196592722	5.97E-09	5.77E-08
GENEBODY	CISD3	17	36889069/36888147	2	cg07095330/cg24534731	0.57280588	0.459706594	0.379211368	-0.193594512	2.32E-10	4.53E-09
GENEBODY	CKM	19	45822962	1	cg00878747	0.680393845	0.544300933	0.447835418	-0.232558427	5.98E-10	9.14E-09
GENEBODY	CLC	19	40227949	1	cg18254848	0.77530625	0.701204794	0.573958777	-0.201347473	6.17E-07	3.13E-06
GENEBODY	CLDN1	3	190038679/190037695/190039141/190030195/190030381	5	cg00804587/cg03601836/cg25330387/cg27177296/cg27326372	0.712564182	0.628719213	0.557685008	-0.154879174	7.01E-10	1.03E-08
GENEBODY	CLDN12	7	90042240	1	cg25244356	0.788119685	0.567068389	0.470522159	-0.317597526	1.93E-11	8.46E-10
GENEBODY	CLDN5	22	19511707/19511987/19511967	3	cg00811132/cg09092054/cg17577122	0.170191199	0.277565413	0.4992414	0.329050201	8.48E-09	7.70E-08
GENEBODY	CLEC10A	17	6979505	1	cg13516928	0.739062255	0.667320994	0.584381095	-0.15468116	2.54E-06	1.08E-05
GENEBODY	CLEC1B	12	10150905	1	cg23325997	0.84795976	0.82003375	0.697038123	-0.150921637	0.000330497	0.000860194
GENEBODY	CLEC2B	12	10007156	1	cg08753403	0.87810528	0.81864865	0.677912227	-0.200193053	2.51E-05	8.37E-05
GENEBODY	CLEC3A	16	78056736	1	cg04365980	0.50303315	0.402729922	0.311195664	-0.191837486	1.18E-09	1.54E-08
GENEBODY	CLEC4G	19	7796382/7795192/7794952/7795428/7795619/7795988	6	cg04816348/cg05842480/cg09772661/cg12768640/cg13950603/cg23853484	0.383921066	0.436241719	0.545577043	0.161655977	7.74E-08	5.03E-07
GENEBODY	CLEC4M	19	7834128/7831896/7828144	3	cg11615959/cg18515510/cg21372914	0.658196345	0.597386833	0.487052403	-0.171143942	4.76E-09	4.81E-08
GENEBODY	CLEC7A	12	10280440	1	cg11650987	0.571332045	0.501499889	0.417748127	-0.153583918	3.29E-07	1.79E-06
GENEBODY	CLPS	6	35764958	1	cg25107791	0.55239714	0.443891833	0.359574932	-0.192822208	1.61E-10	3.47E-09
GENEBODY	CLRN1	3	150663357/150690183/150662697/150661892	4	cg10625737/cg21847114/cg24721964/cg26062204	0.594813654	0.487601472	0.40037854	-0.194435114	1.09E-12	1.52E-10
GENEBODY	CLUL1	18	635375	1	cg25420230	0.68358776	0.58515865	0.481325577	-0.202262183	3.34E-11	1.19E-09
GENEBODY	CMKLR1	12	108686134	1	cg08337166	0.47663929	0.38012545	0.3071157	-0.16952359	1.73E-13	6.13E-11
GENEBODY	CNDP2	18	72168601/72167213	2	cg15896356/cg25817165	0.483174658	0.383792042	0.317607791	-0.165566867	6.70E-09	6.32E-08
GENEBODY	CNFN	19	42891379/42893103/42891428/42891346	4	cg00912049/cg05206832/cg11673741/cg11692371	0.727915265	0.631831661	0.565484364	-0.162430901	3.68E-10	6.31E-09

GENEBODY	CNGB3	8	87632363/87684002/87721838/87684132	4	cg06690689/cg09567596/cg14224760/cg14283802	0.712180115	0.625792988	0.531086606	-0.181093509	3.35E-10	5.90E-09
GENEBODY	CNNM1	10	101118946/101091481/101118273/101094135	4	cg10064248/cg12079603/cg26459859/cg27604200	0.800192034	0.720270192	0.642046109	-0.158145925	1.28E-09	1.65E-08
GENEBODY	CNOT8	5	154253549/154245989	2	cg12938814/cg27633096	0.638388063	0.527326836	0.457329316	-0.181058747	3.35E-11	1.19E-09
GENEBODY	CNPY2	12	56708578	1	cg06617217	0.636306345	0.5696452	0.430539232	-0.205767113	3.71E-08	2.67E-07
GENEBODY	CNTNAP4	16	76342377/76349097/76521159/76343868/76342620/76342678/76343663/76349255	8	cg01151029/cg04556349/cg05630943/cg06793062/cg09094037/cg09966303/cg25383242/cg27420415	0.698162343	0.631361089	0.534820515	-0.163341827	1.32E-09	1.69E-08
GENEBODY	COL3A1	2	189859022/189839474/189850571	3	cg12923449/cg20770175/cg23235135	0.77037944	0.690482978	0.601203136	-0.169176304	4.28E-06	1.72E-05
GENEBODY	COL5A2	2	190028935/190019594/190037419/189957139/189986317/189981405/189914501/190035925	8	cg01247454/cg07466651/cg08247938/cg08466256/cg12329318/cg18445715/cg23165166/cg26137350	0.730615638	0.663417743	0.579299399	-0.151316239	1.26E-10	2.89E-09
GENEBODY	COX6A2	16	31439106/31439393/31439191	3	cg06507634/cg09117448/cg19927508	0.539587792	0.556801622	0.704187644	0.164599852	1.67E-07	9.83E-07
GENEBODY	CPA4	7	129945684/129933646/129945688	3	cg16535352/cg19690404/cg20845858	0.571739215	0.465804122	0.372698977	-0.199040238	1.82E-06	8.06E-06
GENEBODY	CPO	2	207814687	1	cg06539006	0.35561406	0.268571372	0.190536509	-0.165077551	1.82E-09	2.21E-08
GENEBODY	CPT1C	19	50202524/50204862/50205634/50202599/50196837/50204707/50207768	7	cg14067541/cg14318858/cg16206364/cg19325093/cg22793832/cg24201033/cg25326729	0.729272	0.640909332	0.5729241	-0.1563479	2.72E-10	5.04E-09
GENEBODY	CRB3	19	6464885	1	cg24798010	0.540825735	0.45650285	0.359844027	-0.180981708	3.52E-08	2.56E-07
GENEBODY	CRISP2	6	49670744	1	cg25734198	0.63112058	0.498214589	0.444177677	-0.186942903	7.24E-07	3.60E-06
GENEBODY	CRNN	1	152383842	1	cg22164207	0.56625306	0.470552183	0.340070045	-0.226183015	1.90E-10	3.89E-09
GENEBODY	CRYM	16	21272887/21289268/21287038	3	cg00687714/cg01930441/cg06816254	0.419521723	0.323017981	0.2499097	-0.169612023	3.52E-12	3.02E-10
GENEBODY	CSF2RB	22	37320126/37319042	2	cg20017381/cg21209356	0.50866471	0.399040333	0.320229382	-0.188435328	3.06E-11	1.13E-09
GENEBODY	CST1	20	23728814	1	cg26854096	0.62301204	0.502245517	0.38728465	-0.23572739	2.06E-11	8.77E-10

GENEBODY	CST5	20	23860078	1	cg02882813	0.691158085	0.588927756	0.47503695	-0.216121135	1.35E-07	8.15E-07
GENEBODY	CSTA	3	122058117	1	cg16896618	0.659543285	0.51898935	0.453166441	-0.206376844	9.54E-08	6.05E-07
GENEBODY	CTRB1	16	75255590/75252973	2	cg09686298/cg21686987	0.67584896	0.592074228	0.500159884	-0.175689076	3.30E-09	3.58E-08
GENEBODY	CTSB	8	11710911	1	cg03654169	0.647987555	0.464031372	0.3500838	-0.297903755	4.89E-14	3.73E-11
GENEBODY	CTSS	1	150722805	1	cg10954654	0.80894692	0.599731433	0.489601082	-0.319345838	7.56E-14	4.38E-11
GENEBODY	CXADRP2	15	22014725	1	cg08883696	0.58764544	0.508337244	0.404744291	-0.182901149	1.10E-08	9.57E-08
GENEBODY	CYLC2	9	105764606	1	cg14150186	0.56748472	0.431117722	0.313584627	-0.253900093	7.10E-12	4.47E-10
GENEBODY	CYP11B2	8	143995000	1	cg15847600	0.535918465	0.456219994	0.354607359	-0.181311106	3.63E-08	2.62E-07
GENEBODY	CYP26A1	10	94834557/94834219/94834582/94835255/94835624/94834763/94834201	7	cg02325540/cg09192377/cg10716835/cg12157438/cg16445268/cg17538572/cg22536554	0.268875982	0.36807192	0.464625937	0.195749955	2.12E-06	9.21E-06
GENEBODY	CYP27A1	2	219671206/219675921/219649224/219647360/219647167	5	cg02930667/cg12806497/cg21459645/cg26104932/cg26175971	0.500144093	0.416686251	0.348757854	-0.151386239	1.48E-11	7.16E-10
GENEBODY	CYP7A1	8	59405619	1	cg22550275	0.693364355	0.577079222	0.46551185	-0.227852505	5.71E-11	1.65E-09
GENEBODY	DBX1	11	20178496/20177852/20178979/20181456/20178138/20178040/20180666/20178627/20180336/20178677/20180700/20180437	12	cg00192136/cg04290346/cg05469285/cg08525845/cg08835103/cg09767822/cg13696942/cg14515571/cg14777561/cg21146503/cg21678461/cg24074033	0.264579145	0.280978452	0.417644494	0.15306535	3.24E-06	1.34E-05
GENEBODY	DCD	12	55041948/55041379	2	cg00868860/cg13396068	0.579831453	0.417986736	0.28471787	-0.295113582	4.54E-13	9.40E-11
GENEBODY	DCT	13	95116571	1	cg24157814	0.61398984	0.515280144	0.448377505	-0.165612335	4.79E-09	4.83E-08
GENEBODY	DDX4	5	55061610/55061936/55092708	3	cg02269094/cg20414753/cg24751730	0.742627565	0.663423378	0.574653539	-0.167974026	6.21E-10	9.37E-09
GENEBODY	DEFA10P	8	6826392	1	cg05787038	0.44866998	0.35651815	0.267873955	-0.180796025	6.11E-10	9.28E-09
GENEBODY	DEFB110	6	49988602/49986643	2	cg02585622/cg19765351	0.64758293	0.523558475	0.419462702	-0.228120228	1.01E-09	1.35E-08
GENEBODY	DEFB118	20	29956585	1	cg20312687	0.716861345	0.64576825	0.509959636	-0.206901709	8.98E-06	3.33E-05
GENEBODY	DEFB119	20	29978114/29965177/29978176	3	cg03262887/cg07594472/cg18462653	0.658123905	0.560924528	0.454900241	-0.203223664	1.47E-05	5.17E-05
GENEBODY	DEFB122	20	30012193	1	cg01349088	0.63772139	0.558780289	0.38016645	-0.25755494	1.28E-08	1.09E-07
GENEBODY	DEFB126	20	123613	1	cg20305726	0.538635045	0.414299239	0.354083155	-0.18455189	2.79E-09	3.12E-08

GENEBODY	DEFB133	6	49916931	1	cg00938547	0.768466425	0.662500878	0.572155855	-0.19631057	1.50E-05	5.26E-05
GENEBODY	DGCR10	22	19010419/19010468	2	cg00458681/cg07167594	0.38674499	0.271615647	0.224242523	-0.162502467	3.74E-12	3.16E-10
GENEBODY	DIRC3	2	218251446/218466469/218619744/218299024/218612384/218277531/218151983/218375971/218558933/218182275/218154430/218332980/218418852/218465569/218464166/218438884/218169223/218520767/218487226/218301065/218231805/218550825/218578423/218258333/218465695/218621258/218597777/218531045/218269472/218196152/218295405/218340518/218268053/218481168/218247632/218285297/218149237/218179272/218620242/218493108/218618094/218231392/218416812/218316451/218577902/218354150/218343265/218219798/218515567/218466307/218157055	51	cg00085256/cg00792015/cg00829041/cg01396774/cg01603549/cg01821113/cg02916472/cg03013118/cg03512250/cg03991534/cg04942260/cg05502445/cg05511924/cg06504205/cg06523618/cg07152837/cg07502050/cg07934818/cg08401872/cg09151613/cg09386620/cg09519273/cg09684856/cg10763594/cg11770920/cg12309703/cg12596243/cg12820310/cg13502823/cg13765685/cg14506194/cg14538944/cg15335768/cg15353469/cg15912082/cg16003672/cg17316747/cg18309255/cg20103054/cg20270231/cg21776524/cg21898226/cg22039960/cg22245446/cg22675745/cg23340935/cg24079981/cg24980995/cg25928629/cg26501046/cg26830902	0.624894609	0.548450348	0.469901852	-0.154992757	1.70E-11	7.74E-10
GENEBODY	DKFZP434H168	16	56228114/56228385/56228188/56227065/56227690	5	cg00348762/cg03399271/cg07636145/cg09659734/cg27271486	0.308138805	0.371024859	0.498040949	0.189902144	6.30E-08	4.21E-07
GENEBODY	DKFZp434J0226	19	46713976/46714834	2	cg00145150/cg24473594	0.587093088	0.489403286	0.421254805	-0.165838283	7.65E-10	1.11E-08
GENEBODY	DKFZp434L192	7	56564743	1	cg03660331	0.448396405	0.328675994	0.290292227	-0.158104178	9.82E-10	1.33E-08

GENEBODY	DKK2	4	107955266/107955541/107952897/107956287/107955762/107956405	6	cg02034675/cg03903991/cg04777988/cg17307558/cg17362052/cg24874180	0.239676985	0.309899092	0.411615536	0.171938551	2.06E-05	7.00E-05
GENEBODY	DKK4	8	42234243	1	cg09184899	0.77903867	0.742232667	0.5192362	-0.25980247	1.23E-05	4.42E-05
GENEBODY	DLX5	7	96650323/96650509/96652245/96653183/96652894/96652222/96652481/96650192/96652464/96650096/96651983/96652123/96652153/96650171/96651281/96650407/96651537/96651915/96651111/96650668/96652375/96651586/96652115	23	cg00400832/cg00503840/cg02101486/cg06537230/cg06911084/cg08101303/cg08832603/cg08835113/cg08878323/cg09359114/cg11367354/cg11500797/cg11891395/cg12041387/cg13462129/cg15339231/cg15732768/cg18873386/cg20080624/cg20377305/cg24115040/cg27016494/cg27032146	0.312312286	0.381586771	0.522114544	0.209802258	2.81E-08	2.11E-07
GENEBODY	DMRTA2	1	50886411/50885352/50886920/50885132/50886393/50884544/50884480/50886969/50886071/50886782/50885369/50886949	12	cg00945234/cg03989480/cg04455430/cg04931216/cg07362328/cg09792881/cg10512745/cg12756396/cg13909582/cg16732616/cg16809460/cg25191628	0.242798509	0.261597178	0.40754812	0.164749611	1.08E-05	3.90E-05
GENEBODY	DNAJB13	11	73668757/73669256/73669449/73668626/73669290/73676012	6	cg04044120/cg15670924/cg15851278/cg20737388/cg23327896/cg25592907	0.58036392	0.528503581	0.412315884	-0.168048036	1.03E-08	9.11E-08
GENEBODY	DSC1	18	28737288	1	cg27444744	0.685038485	0.640228611	0.523130864	-0.161907621	3.96E-05	0.000126628
GENEBODY	DSEL	18	65180255/65181755	2	cg25533423/cg25677027	0.738221205	0.631348842	0.540128409	-0.198092796	4.60E-11	1.44E-09
GENEBODY	DSTN	20	17554599/17552881/17552529/17551166/17582752	5	cg00401234/cg02245875/cg03340964/cg13462232/cg25694790	0.453332995	0.342078361	0.270128798	-0.183204197	1.01E-11	5.42E-10

GENEBODY	DUSP6	12	89744471/89744877/89744524/89744701/89744488/89743788/89744621/89743281/89744609/89745192/89744662/89744150	12	cg00772407/cg01242903/cg01814191/cg05769889/cg07239716/cg10077746/cg10233654/cg13691961/cg17740822/cg20521715/cg25124406/cg27365701	0.41191367	0.301923749	0.234842943	-0.177070727	4.12E-11	1.33E-09
GENEBODY	DYDC1	10	82103151/82103052	2	cg01248810/cg24687020	0.673570085	0.581009433	0.440630723	-0.232939362	4.47E-08	3.12E-07
GENEBODY	DYNC1I2	2	172581104/172546870	2	cg13460297/cg13504410	0.71809179	0.637776056	0.560508684	-0.157583106	2.76E-09	3.10E-08
GENEBODY	DYTN	2	207537139/207516641	2	cg04589523/cg18921607	0.42604688	0.338904667	0.249361591	-0.176685289	3.35E-10	5.90E-09
GENEBODY	DZIP1L	3	137787902/137816617/137787060/137808241	4	cg05265703/cg07665466/cg17394126/cg24631735	0.67428192	0.587823339	0.518054452	-0.156227468	5.94E-10	9.11E-09
GENEBODY	ECM1	1	150484766/150480856	2	cg11404129/cg16386293	0.67509026	0.616675828	0.522128741	-0.152961519	9.90E-06	3.64E-05
GENEBODY	EFCAB8	20	31482267	1	cg24384212	0.75876768	0.701050311	0.602331964	-0.156435716	0.0001542	0.000432402
GENEBODY	EFCAB9	5	171621698	1	cg22203867	0.526595235	0.422979683	0.31276675	-0.213828485	4.71E-12	3.62E-10
GENEBODY	EFNB3	17	7609416/7611315	2	cg22761431/cg27401891	0.76269603	0.662044322	0.582484023	-0.180212007	2.18E-07	1.25E-06
GENEBODY	EFS	14	23830974	1	cg18289581	0.498173575	0.391135828	0.284435145	-0.21373843	2.94E-11	1.10E-09
GENEBODY	EGR4	2	73519364/73520043/73519034/73518702/73519343	5	cg06079106/cg13481359/cg15769184/cg26049726/cg26647617	0.271071411	0.324044954	0.46778692	0.196715509	9.78E-07	4.71E-06
GENEBODY	EIF3IP1	7	109599644	1	cg14146669	0.715886195	0.555398583	0.426187	-0.289699195	8.88E-08	5.68E-07
GENEBODY	ELF5	11	34517751/34517541/34523679/34517699	4	cg02882375/cg10631356/cg21038795/cg24935217	0.59136958	0.482424185	0.405169194	-0.186200386	5.12E-09	5.13E-08
GENEBODY	ELTD1	1	79472282/79471984	2	cg14319235/cg17095278	0.5566883	0.614574883	0.718865995	0.162177695	1.78E-10	3.72E-09
GENEBODY	EME1	17	48454200	1	cg16622353	0.60114464	0.469594944	0.366771655	-0.234372985	6.30E-13	1.14E-10
GENEBODY	EPHA5	4	66321852/66533472/66535203/66534040/66535145/66534326/66534218/66534538	8	cg01694632/cg04322202/cg10334354/cg14647451/cg18420965/cg19410532/cg23193365/cg23842170	0.421110616	0.491374165	0.580203542	0.159092927	4.05E-07	2.15E-06

GENEBODY	EPHB6	7	142561918/142564916/142564463/142562041/142562153/142564897	6	cg03217572/cg04990850/cg08415172/cg14349531/cg16914425/cg21577626	0.685865485	0.600568664	0.522832005	-0.16303348	1.05E-11	5.65E-10
GENEBODY	EPS8	12	15822734/15790461/15815827/15830414	4	cg03954343/cg05343665/cg10231182/cg23200995	0.80322547	0.666487953	0.606446752	-0.196778718	2.95E-13	7.92E-11
GENEBODY	ERGIC2	12	29506350	1	cg07216656	0.75708351	0.686323039	0.605618645	-0.151464865	2.53E-07	1.42E-06
GENEBODY	ESD	13	47352461	1	cg22733207	0.63846893	0.489193439	0.432157541	-0.206311389	1.27E-11	6.43E-10
GENEBODY	ESM1	5	54275198	1	cg09452568	0.848279415	0.742931278	0.652884932	-0.195394483	3.24E-06	1.34E-05
GENEBODY	F13B	1	197030122	1	cg03138560	0.64337009	0.521757806	0.38473505	-0.25863504	2.68E-12	2.64E-10
GENEBODY	FAM12B	14	21238436	1	cg01724456	0.64670852	0.53674455	0.445334055	-0.201374465	2.60E-09	2.97E-08
GENEBODY	FAM153A	5	177165350	1	cg24668618	0.816211845	0.743645428	0.598093455	-0.21811839	7.99E-06	3.00E-05
GENEBODY	FAM153B	5	175526747/175531103/175526951	3	cg06853587/cg19956449/cg21983520	0.750455245	0.682799419	0.575548673	-0.174906572	1.14E-06	5.37E-06
GENEBODY	FAM166A	9	140138821	1	cg14010394	0.651466025	0.530640194	0.422880868	-0.228585157	5.92E-11	1.69E-09
GENEBODY	FAM180A	7	135429446	1	cg09935440	0.67682329	0.564978794	0.521712268	-0.155111022	2.89E-08	2.17E-07
GENEBODY	FAM26D	6	116879209	1	cg15314855	0.533883	0.456439	0.349367114	-0.184515886	8.40E-10	1.18E-08
GENEBODY	FAM49A	2	16767476	1	cg10090418	0.770699375	0.590443906	0.396925518	-0.373773857	2.52E-11	1.01E-09
GENEBODY	FAM71F1	7	128368940	1	cg13169491	0.73040038	0.65350435	0.521702445	-0.208697935	5.98E-09	5.77E-08
GENEBODY	FAM71F2	7	128312789	1	cg05780294	0.51044051	0.4027293	0.332807686	-0.177632824	5.67E-11	1.65E-09
GENEBODY	FAM72D	1	143907550	1	cg12386752	0.54440671	0.438273639	0.3464906	-0.19791611	2.82E-11	1.07E-09
GENEBODY	FAM92A3	4	183960120	1	cg08090384	0.535621085	0.410147389	0.285887109	-0.249733976	4.12E-11	1.33E-09
GENEBODY	FASTKD3	5	7866767/7866371	2	cg05495391/cg24575485	0.574449305	0.459530172	0.376068436	-0.198380869	1.53E-11	7.24E-10
GENEBODY	FBXL21	5	135273913	1	cg17796593	0.637710975	0.496861872	0.393550982	-0.244159993	2.63E-10	4.93E-09
GENEBODY	FBXO34	14	55818341	1	cg23234828	0.682846015	0.563222067	0.491955659	-0.190890356	7.52E-09	6.98E-08
GENEBODY	FCGR1B	1	120929152	1	cg20821544	0.611994795	0.501138567	0.391535082	-0.220459713	1.32E-09	1.69E-08
GENEBODY	FCGR3A	1	161519396/161516128	2	cg04384208/cg07111566	0.718840693	0.615740392	0.49458947	-0.224251222	2.94E-11	1.10E-09
GENEBODY	FCGR3B	1	161600769	1	cg04567009	0.673309755	0.510614594	0.379355841	-0.293953914	2.17E-11	9.11E-10
GENEBODY	FCN2	9	137772886/137775039	2	cg11884243/cg14033273	0.652238323	0.556451617	0.491710084	-0.160528238	4.54E-10	7.39E-09
GENEBODY	FCN3	1	27701171	1	cg02365086	0.640959805	0.507824483	0.390144855	-0.25081495	1.85E-09	2.24E-08
GENEBODY	FCRL1	1	157772525/157776690/157789662/157771971	4	cg00394221/cg08933467/cg09340639/cg11810135	0.532901654	0.420874889	0.331312738	-0.201588916	1.47E-13	5.80E-11

GENEBODY	FCRL3	1	157668115/157669111/157660319/157670220	4	cg05956521/cg07184316/cg11150520/cg25259754	0.728045736	0.631628311	0.562651805	-0.165393932	3.63E-08	2.62E-07
GENEBODY	FEM1C	5	114878738/114867569	2	cg02364826/cg25619717	0.42904047	0.351327544	0.274309552	-0.154730918	2.31E-11	9.43E-10
GENEBODY	FEZF2	3	62357769/62356042/62358234/62356223/62355884/62356774/62356323/62358241/62357176/62356962/62358379	11	cg02083234/cg03915638/cg03986829/cg10542584/cg12988424/cg15422685/cg16817826/cg18104012/cg18525486/cg20072171/cg26136365	0.310608993	0.383128865	0.529031671	0.218422678	6.79E-08	4.49E-07
GENEBODY	FGF10	5	44316088/44332757/44333003	3	cg04239786/cg16204420/cg25021530	0.566717853	0.461156296	0.393539517	-0.173178337	9.29E-14	4.89E-11
GENEBODY	FGF3	11	69632655/69625284/69631981/69625218/69626349/69625409/69628171/69632455/69632335/69633001/69632883/69632220/69632117/69633070/69633313	15	cg04602060/cg04691634/cg06727490/cg06889951/cg10361167/cg11410726/cg12141747/cg12319507/cg13703576/cg15380657/cg17038667/cg17540356/cg18222122/cg21283867/cg24773676	0.339347864	0.396072119	0.490115832	0.150767968	2.95E-05	9.69E-05
GENEBODY	FGF5	4	81189927/81188405/81188471/81189706/81189774	5	cg04401986/cg11580948/cg12341047/cg14349667/cg17080423	0.291678863	0.382729572	0.480519877	0.188841014	1.18E-07	7.23E-07
GENEBODY	FGFBP1	4	15938093	1	cg21039166	0.55243293	0.432582933	0.368246241	-0.184186689	5.66E-11	1.65E-09
GENEBODY	FHL5	6	97058943	1	cg22236418	0.48467161	0.443163689	0.329316986	-0.155354624	1.80E-05	6.21E-05
GENEBODY	FLJ16779	20	61886082/61885850/61885448/61886262/61886142/61886079/61886587/61886313/61890060/61885926	10	cg01911708/cg02165799/cg05326358/cg07675169/cg07986239/cg08111446/cg13657510/cg14877226/cg18822613/cg23942897	0.329838654	0.441325765	0.566237688	0.236399034	2.09E-08	1.65E-07
GENEBODY	FLJ20184	4	106477599/106515450	2	cg00796302/cg05626226	0.440447705	0.349669383	0.274798314	-0.165649391	4.85E-11	1.50E-09
GENEBODY	FLJ40330	2	89065442/89065605/89065509	3	cg01506298/cg09410752/cg12294208	0.377687627	0.45508888	0.555499145	0.177811519	8.19E-07	4.02E-06

GENEBODY	FLJ41856	19	45059935	1	cg04588257	0.59610764	0.508134228	0.394901732	-0.201205908	5.18E-08	3.56E-07
GENEBODY	FMO6P	1	171111193/171116322	2	cg05376227/cg07726215	0.5562736	0.6588193	0.706812109	0.150538509	5.63E-08	3.81E-07
GENEBODY	FOXI1	5	169534834	1	cg01288375	0.63690301	0.552765289	0.435594777	-0.201308233	9.95E-06	3.65E-05
GENEBODY	FRMD6	14	52164293/52171626/52182702/52191937/52174876/52160138	6	cg05858008/cg07507518/cg09987240/cg11777870/cg18899667/cg25584474	0.600488497	0.528044793	0.426526462	-0.173962035	7.22E-10	1.06E-08
GENEBODY	FUT7	9	139925395/139925856/139925750	3	cg02971262/cg03630596/cg14205519	0.771536373	0.66372385	0.578319041	-0.193217332	8.68E-11	2.19E-09
GENEBODY	GALNTL5	7	151716974/151664041/151653523/151664190/151664071/151692197/151653890	7	cg01662876/cg01760103/cg11727304/cg13835670/cg21565299/cg25213550/cg25310567	0.629539729	0.518002206	0.404858738	-0.224680991	8.28E-15	1.44E-11
GENEBODY	GALR1	18	74963364/74964030/74963807	3	cg00662556/cg05896682/cg26038190	0.36631598	0.452316078	0.616849118	0.250533138	1.26E-09	1.63E-08
GENEBODY	GAMT	19	1401118/1399338/1400851/1399166	4	cg00308133/cg09507386/cg23960750/cg24229707	0.522959114	0.411549359	0.335328745	-0.187630368	2.14E-13	6.75E-11
GENEBODY	GAPT	5	57790494	1	cg05904808	0.84336172	0.797591972	0.691763377	-0.151598343	0.000120833	0.000346162
GENEBODY	GATA2	3	128204813/128202493/128202731/128205496/128200769/128205143/128204927/128202797/128203414/128205274/128205418/128205200/128202176/128200723/128204047/128204413	16	cg01102073/cg01518889/cg02815312/cg04347582/cg04535236/cg06115614/cg06796779/cg07132710/cg07195926/cg07263393/cg08755743/cg09852607/cg10935762/cg15038286/cg22915582/cg25229470	0.416216061	0.468868378	0.578658028	0.162441968	3.36E-09	3.63E-08
GENEBODY	GDF1	19	18980443/18980744/18980103/18980110	4	cg01215442/cg19256094/cg19761304/cg21526172	0.47916622	0.560289072	0.643120468	0.163954248	2.32E-12	2.52E-10

GENEBODY	GDF6	8	97167170/97156859/97166036/97162431/97172012/97158015/97166291/97171332/97171668/97169621/97157878/97170097/97166862/97169767/97171901/97167470/97162506/97171827/97158052/97170666/97170364/97167768/97172219/97165770/97157856/97169920/97172480/97157453/97160999/97157756/97169764/97171126/97167878	33	cg00394180/cg00585714/cg01673674/cg02246426/cg03224572/cg05690644/cg07046818/cg07159961/cg07589612/cg08381274/cg09164580/cg10131194/cg10133465/cg10689528/cg11073558/cg11738701/cg12156944/cg13211683/cg15006881/cg15270654/cg15277906/cg16290866/cg16530959/cg16574871/cg17820365/cg17999376/cg18214379/cg18533201/cg18902541/cg19691778/cg20800022/cg26035118/cg26550710	0.328101586	0.389149618	0.512902275	0.184800689	9.12E-10	1.26E-08
GENEBODY	GFI1B	9	135865263/135866029/135863570/135863521	4	cg12658925/cg14020186/cg14475859/cg20143773	0.7395212	0.629373991	0.563725532	-0.175795668	3.17E-11	1.16E-09
GENEBODY	GJA1	6	121768597	1	cg23176309	0.75416899	0.695033606	0.581977623	-0.172191367	7.24E-06	2.75E-05
GENEBODY	GKN1	2	69206176	1	cg20874031	0.39956413	0.2527214	0.186330905	-0.213233225	1.03E-14	1.49E-11
GENEBODY	GNAT2	1	110153100	1	cg23985820	0.740908395	0.696802383	0.54897315	-0.191935245	2.44E-07	1.38E-06
GENEBODY	GNAT3	7	80096608	1	cg05869611	0.387229335	0.323123978	0.222492886	-0.164736449	2.68E-08	2.03E-07
GENEBODY	GNG13	16	848997	1	cg08515427	0.672862695	0.591957417	0.515389482	-0.157473213	1.02E-07	6.39E-07
GENEBODY	GOLGA6C	15	75551123	1	cg23516121	0.78995868	0.690753089	0.57137425	-0.21858443	1.26E-11	6.43E-10
GENEBODY	GOT1L1	8	37793789	1	cg20154938	0.693218705	0.626493511	0.520363668	-0.172855037	7.48E-07	3.71E-06
GENEBODY	GPAM	10	113941466/113939632	2	cg11888270/cg16794749	0.767883593	0.691112603	0.614746482	-0.153137111	1.98E-07	1.15E-06
GENEBODY	GPHB5	14	63781994	1	cg23301979	0.81597335	0.734470383	0.630290332	-0.185683018	6.97E-07	3.49E-06
GENEBODY	GPR120	10	95327305/95327654/95327884/95327292/95329303/95327325/95327683/95327107	8	cg05613447/cg08961547/cg09488203/cg10905401/cg13633964/cg15133448/cg19870668/cg22749310	0.329563668	0.227030612	0.166920966	-0.162642702	9.23E-11	2.27E-09

GENEBODY	GPR149	3	154146183/154146398/154145868/154145810	4	cg00046499/cg04151278/cg16619395/cg20498859	0.291585609	0.374960358	0.539452854	0.247867246	4.15E-09	4.31E-08
GENEBODY	GPR161	1	168058592/168060823	2	cg00699919/cg25097189	0.54511893	0.409240878	0.315100332	-0.230018598	2.16E-11	9.11E-10
GENEBODY	GPR171	3	150916446	1	cg15715853	0.763456085	0.561045239	0.4579293	-0.305526785	2.05E-14	2.37E-11
GENEBODY	GPR65	14	88477518	1	cg18185987	0.568922755	0.4569447	0.390321795	-0.17860096	1.53E-10	3.34E-09
GENEBODY	GPR84	12	54756590	1	cg10668189	0.731711015	0.648057883	0.510367568	-0.221343447	1.15E-08	9.96E-08
GENEBODY	GPR85	7	112724674	1	cg14511782	0.63689156	0.522556367	0.39373755	-0.24315401	1.49E-07	8.88E-07
GENEBODY	GPX7	1	53068587/53072410/53068834/53068579	4	cg02453146/cg03330022/cg09161043/cg23272399	0.362997962	0.433848498	0.524766123	0.161768161	4.63E-06	1.85E-05
GENEBODY	GRAP2	22	40355732	1	cg17988310	0.493742515	0.357406589	0.263253495	-0.23048902	5.62E-09	5.53E-08
GENEBODY	GREB1L	18	19024872	1	cg07490808	0.598833015	0.462903861	0.331397841	-0.267435174	1.52E-11	7.24E-10
GENEBODY	GRIA1	5	153160425/153038077/153039227/152896564/152949095/153008721	6	cg07601741/cg13583454/cg19752602/cg22798121/cg23165500/cg26343183	0.592354828	0.489614514	0.377359989	-0.214994839	1.06E-12	1.51E-10
GENEBODY	GSC	14	95235125/95235489/95235026/95235127/95235968/95234965/95235869/95235402	8	cg01163842/cg01533258/cg02948476/cg07785447/cg13252307/cg18687675/cg20804555/cg21063282	0.262750923	0.281539885	0.443667865	0.180916943	0.000815909	0.001959876
GENEBODY	GSC2	22	19137306/19137222/19136844/19136922/19137371	5	cg20209308/cg21384203/cg21854621/cg24926361/cg26599006	0.211347241	0.266278984	0.388174846	0.176827605	3.54E-06	1.45E-05
GENEBODY	GSS	20	33539306	1	cg13607138	0.721902275	0.595800028	0.451020814	-0.270881461	1.57E-07	9.29E-07
GENEBODY	GSTM1	1	110230545/110230633/110233940	3	cg06632762/cg10950028/cg23241950	0.405541355	0.529260925	0.595818757	0.190277402	1.52E-06	6.87E-06
GENEBODY	GSTM2	1	110210925/110211023/110210913/110214317	4	cg03942855/cg06615940/cg16670497/cg20944548	0.448848321	0.565330139	0.639534299	0.190685978	1.31E-09	1.67E-08
GENEBODY	GSTTP1	22	24345907	1	cg10678937	0.703289985	0.615674606	0.544561173	-0.158728812	1.86E-10	3.83E-09
GENEBODY	GSX1	13	28367245/28367741	2	cg05967403/cg14367229	0.487558255	0.572149678	0.69866592	0.211107665	8.55E-10	1.20E-08
GENEBODY	GTSF1L	20	42354942	1	cg23727943	0.70171297	0.580374411	0.485231582	-0.216481388	2.58E-12	2.60E-10

GENEBODY	HAND2	4	174449488/174448841/174448705/174449827/174448549	5	cg04771946/cg05155840/cg08297751/cg16162058/cg24974365	0.279087092	0.322111562	0.458464645	0.179377553	1.38E-05	4.89E-05
GENEBODY	HAPLN1	5	82949588	1	cg11215486	0.39758257	0.364209967	0.233645232	-0.163937338	3.50E-07	1.90E-06
GENEBODY	HAS2	8	122639115	1	cg20146177	0.547846495	0.640625172	0.697897782	0.150051287	1.56E-08	1.30E-07
GENEBODY	HAVCR2	5	156516798	1	cg19063654	0.65704751	0.554231283	0.450546559	-0.206500951	3.83E-11	1.30E-09
GENEBODY	HBBP1	11	5263432	1	cg04161236	0.6683465	0.518537633	0.364920186	-0.303426314	2.78E-11	1.07E-09
GENEBODY	HBG1	11	5270556	1	cg18768582	0.463221075	0.343144089	0.241405668	-0.221815407	9.11E-11	2.26E-09
GENEBODY	HBM	16	216626/216450/216244/216100	4	cg03309232/cg04141813/cg21664030/cg26976732	0.233676223	0.274872456	0.389071992	0.155395769	0.000164249	0.00045836
GENEBODY	HCG4	6	29760784/29760769/29760505/29760773/29760634/29760028/29760096/29759947/29759949/29760681/29760675/29759078/29760782/29760757/29760148/29760447/29760832/29760754/29760805/29760023/29760435/29760158/29760410/29760688/29760695/29759955/29759197/29760164/29760090/29759972/29760609/29760659/29760823/29759729/29759718/29760765/29760495/29759804/29760713	39	cg00030176/cg00142106/cg00501272/cg04214566/cg05216332/cg05907976/cg07457785/cg08560874/cg08696107/cg08867464/cg09112760/cg11036359/cg11232276/cg11278602/cg11753155/cg11901272/cg11973623/cg12154624/cg13300282/cg13321114/cg13497563/cg13688808/cg14443519/cg14652056/cg16640496/cg16787652/cg17068675/cg17394649/cg17512365/cg17670496/cg18299938/cg18457685/cg21039495/cg22053721/cg22499139/cg22903300/cg23849169/cg24621922/cg27012994	0.254701587	0.3421145	0.423188742	0.168487155	7.27E-09	6.79E-08
GENEBODY	HCLS1	3	121363104	1	cg18546037	0.797833285	0.715467333	0.58815315	-0.209680135	1.92E-07	1.12E-06
GENEBODY	HELB	12	66697621/66697281	2	cg06239064/cg22546775	0.528376855	0.431749483	0.375059736	-0.153317119	1.09E-08	9.50E-08

GENEBODY	HELT	4	185940595/185941265/185941304/185941625/185941601/185940969/185940549/185941088/185940638/185940923	10	cg00075634/cg00939735/cg04905049/cg06274396/cg10463150/cg11497952/cg13966883/cg17081772/cg18065811/cg22848896	0.229381335	0.271655583	0.434679543	0.205298208	2.47E-06	1.06E-05
GENEBODY	HGF	7	81391327/81381353/81372100	3	cg16033274/cg18944653/cg23269320	0.67146914	0.587581372	0.471089761	-0.200379379	5.48E-08	3.73E-07
GENEBODY	HLA-DQA2	6	32712093/32710583/32709470/32711008/32710209/32713018/32712979/32713188/32711632/32710516/32711617/32713607/32711102/32711889/32714066/32712103/32712045/32710506/32711028/32709511/32713827/32712930/32710933/32713044/32709909	25	cg00334532/cg01050736/cg01244342/cg02293354/cg03127506/cg04493778/cg05428452/cg06293782/cg06598146/cg10023855/cg10645648/cg14740554/cg15496763/cg17471365/cg17776207/cg18572898/cg18785140/cg19350679/cg22097941/cg22282941/cg22812614/cg23117647/cg23221363/cg23870181/cg26489369	0.725793465	0.626579453	0.537769412	-0.188024053	5.88E-11	1.68E-09
GENEBODY	HMGCS2	1	120310021	1	cg24482350	0.502739765	0.3791392	0.314991545	-0.18774822	8.02E-08	5.19E-07
GENEBODY	HMHB1	5	143192067/143192588	2	cg02228160/cg06471487	0.775363425	0.65827975	0.529884091	-0.245479334	1.80E-09	2.19E-08
GENEBODY	HNRNPA3P1	10	44285864	1	cg04625975	0.584867215	0.477091261	0.387502164	-0.197365051	4.71E-08	3.27E-07
GENEBODY	HNRNPH3	10	70098420	1	cg14517144	0.72739177	0.594846811	0.561025355	-0.166366415	7.31E-11	1.94E-09
GENEBODY	HORMAD2	22	30552801	1	cg10230314	0.448995555	0.33445175	0.286113841	-0.162881714	2.70E-09	3.05E-08
GENEBODY	HOXA2	7	27140797/27141067/27140942/27141388/27141088/27141139	6	cg00188704/cg02803819/cg03763508/cg06786372/cg13661519/cg21464565	0.564760608	0.65107556	0.823423477	0.258662868	5.60E-11	1.64E-09
GENEBODY	HOXA3	7	27150042/27147590/27150031/27149139/27148204/27148002	6	cg02439266/cg07153966/cg09591524/cg09865454/cg17800708/cg19048532	0.699223326	0.749983822	0.860057127	0.160833801	5.45E-10	8.53E-09

GENEBODY	HOXC11	12	54368639/54367822/54368332/54369102/54368272/54368203	6	cg02510471/cg04203883/cg07035173/cg12599390/cg13855243/cg17273416	0.313997705	0.385290205	0.46476603	0.150768326	4.40E-05	0.000139143
GENEBODY	HOXC13	12	54337929/54338824/54333587/54338937/54336246/54338743/54333565/54333823	8	cg00059246/cg08803500/cg15611413/cg20104341/cg20959780/cg23922739/cg24094550/cg25936147	0.276702051	0.334645964	0.444672973	0.167970922	8.45E-07	4.13E-06
GENEBODY	HOXC4	12	54447807/54448729/54448090/54448769/54448913/54448265/54447873	7	cg01524853/cg03146625/cg11746813/cg15648389/cg16370398/cg18473521/cg22747076	0.32349022	0.436043073	0.54035221	0.21686199	5.69E-09	5.57E-08
GENEBODY	HOXD1	2	177054573/177054306	2	cg02466815/cg02746725	0.444847885	0.519767193	0.65491423	0.210066345	5.06E-08	3.48E-07
GENEBODY	HOXD12	2	176965435	1	cg03371669	0.513124795	0.563995711	0.673270295	0.1601455	4.26E-07	2.25E-06
GENEBODY	HSD11B2	16	67466260/67467751/67465455/67465461	4	cg01443318/cg02734600/cg07724674/cg20981893	0.649359768	0.564178772	0.485042457	-0.164317311	3.13E-10	5.61E-09
GENEBODY	HTR2B	2	231977049	1	cg12793238	0.87486977	0.764924728	0.670620395	-0.204249375	2.59E-06	1.10E-05
GENEBODY	HTR3B	11	113778771/113775900/113779788/113778957/113804386/113779936	6	cg01614101/cg06961323/cg17637877/cg17647537/cg21960184/cg23851685	0.753256203	0.673635144	0.573436249	-0.179819954	1.74E-11	7.86E-10
GENEBODY	HYALP1	7	123459295	1	cg09630417	0.52883965	0.392865139	0.291143045	-0.237696605	2.63E-10	4.93E-09
GENEBODY	IBTK	6	82946781/82898963	2	cg14154456/ch.6.1678538R	0.621618835	0.515131694	0.459971168	-0.161647667	6.06E-06	2.35E-05
GENEBODY	ICAM2	17	62083407	1	cg07918509	0.53180796	0.450048789	0.342392455	-0.189415505	1.78E-08	1.45E-07
GENEBODY	ICOS	2	204822542	1	cg15247069	0.781445035	0.6497174	0.470782023	-0.310663012	1.31E-10	2.96E-09
GENEBODY	IFLTD1	12	25649090/25705861/25656194/25707564/25705953/25707667/25644389/25705290/25707569	9	cg06680863/cg10412312/cg11130955/cg13134916/cg15461781/cg15758700/cg21254753/cg23435916/cg24850296	0.584742941	0.495047946	0.42587064	-0.158872301	7.48E-11	1.96E-09
GENEBODY	IGFBP6	12	53491850/53495945/53492662	3	cg06408864/cg16748326/cg23841186	0.45006956	0.379769222	0.295921295	-0.154148265	1.39E-07	8.37E-07
GENEBODY	IGFL3	19	46624887	1	cg18249535	0.71493229	0.631810233	0.554645082	-0.160287208	4.83E-06	1.91E-05

GENEBODY	IGLON5	19	51827007/51815281/51830634/51830477/51831274/51815373/51818377/51830311/51815359/51829984/51815658/51830960	12	cg01592198/cg05630016/cg06667574/cg07382943/cg08874512/cg09928766/cg11381655/cg15985184/cg16980637/cg18363918/cg18568930/cg19725343	0.404835151	0.452983636	0.577810053	0.172974903	3.90E-07	2.08E-06
GENEBODY	IL1F10	2	113831203	1	cg24553058	0.714798375	0.657362644	0.532581105	-0.18221727	6.20E-06	2.39E-05
GENEBODY	IL22RA2	6	137477184	1	cg23507945	0.50142394	0.388849	0.346411709	-0.155012231	1.72E-05	5.97E-05
GENEBODY	IL2RB	22	37536156	1	cg22327543	0.6707666	0.570526994	0.402161141	-0.268605459	2.73E-09	3.07E-08
GENEBODY	IL5RA	3	3134102/3135232/3133700/3135242/3123308/3134187	6	cg08119928/cg09558975/cg15621546/cg19376823/cg23828301/cg25791578	0.789146337	0.710186732	0.628513976	-0.160632361	1.01E-08	8.97E-08
GENEBODY	IL7R	5	35860337	1	cg01027405	0.47155128	0.320009517	0.238115591	-0.233435689	1.02E-13	4.92E-11
GENEBODY	IMPG2	3	101010124/100990781/100970896	3	cg08660810/cg12249537/cg27318481	0.69985965	0.634552039	0.546051232	-0.153808418	1.86E-06	8.19E-06
GENEBODY	INTU	4	128630266	1	cg07752095	0.72256302	0.666802833	0.550397614	-0.172165406	5.15E-06	2.02E-05
GENEBODY	IPW	15	25362188	1	cg06121514	0.706685335	0.637791933	0.513800109	-0.192885226	2.35E-05	7.87E-05
GENEBODY	IQUB	7	123141765	1	cg14472291	0.4881498	0.377944178	0.24839861	-0.23975119	3.53E-09	3.78E-08
GENEBODY	IRF7	11	612680/612837/614761/613632/613792/613478/614787/612762	8	cg03755158/cg05309505/cg08926253/cg16486109/cg17114584/cg20989454/cg22016995/cg27271532	0.506447648	0.414499535	0.356433314	-0.150014334	3.84E-10	6.52E-09
GENEBODY	IRS2	13	110424686/110434016/110425835/110431612/110431691/110424497/110424782/110432220/110433636/110412178/110433784/110432935	12	cg01156012/cg01569664/cg08348990/cg11153485/cg12085119/cg12195446/cg13539803/cg20445402/cg21266798/cg21601493/cg24526103/cg25924746	0.748918938	0.632823224	0.57466497	-0.174253969	9.51E-08	6.03E-07

GENEBODY	IRX1	5	3599686/3599012/3600391/3596704/3597487/3597311/3598967/3599090/3597695/3598319/3596996/3600572/3599719/3597760	14	cg01294808/cg04302194/cg04365721/cg06060135/cg07881405/cg08492173/cg08746900/cg09731996/cg11605835/cg12468238/cg15505412/cg18100702/cg18635552/cg25410785	0.330609232	0.417503458	0.558162184	0.227552952	5.23E-10	8.28E-09
GENEBODY	IRX5	16	54966837/54965929/54965492/54965895/54967389/54967714/54967024/54966471	8	cg01901262/cg03665761/cg05266781/cg07254066/cg09492451/cg10824810/cg16376108/cg16642791	0.397374316	0.46151088	0.631797325	0.234423008	7.88E-10	1.13E-08
GENEBODY	ISLR2	15	74425552/74426925/74426334/74427234/74426086/74426273/74427239/74426957/74425757	9	cg01178680/cg03689324/cg03895392/cg13113525/cg13242000/cg20314548/cg25789916/cg25833989/cg27552679	0.313424293	0.342196129	0.468127702	0.154703409	5.16E-07	2.67E-06
GENEBODY	ITLN1	1	160850377	1	cg07177437	0.67076678	0.505370678	0.445825432	-0.224941348	4.89E-10	7.87E-09
GENEBODY	IVL	1	152882420	1	cg02855850	0.509808775	0.381492039	0.274810236	-0.234998539	5.78E-13	1.09E-10
GENEBODY	JAM2	21	27015734/27012878/27012313/27012615	4	cg01975706/cg04964944/cg06479755/cg26412722	0.322996433	0.396177302	0.519552811	0.196556377	1.77E-07	1.04E-06
GENEBODY	KCNA2	1	111147106	1	cg02076848	0.821111415	0.764080556	0.627981077	-0.193130338	2.02E-06	8.84E-06
GENEBODY	KCND2	7	120380099/120059583/120142732/119915979/120362441/119945494	6	cg01367751/cg12307557/cg18645002/cg22572258/cg22887467/cg25822376	0.765192823	0.69088387	0.607416806	-0.157776017	1.70E-10	3.60E-09
GENEBODY	KCNJ1	11	128711233/128710122	2	cg25751961/cg25893560	0.81504113	0.693855561	0.592575011	-0.222466119	5.99E-11	1.70E-09
GENEBODY	KCNK18	10	118957259	1	cg07637239	0.520687035	0.429026122	0.327734136	-0.192952899	2.50E-08	1.91E-07
GENEBODY	KDELR3	22	38864868/38866743	2	cg01640635/cg22024572	0.89051643	0.823748914	0.724423216	-0.166093214	1.02E-08	9.05E-08
GENEBODY	KHDC1	6	73951939	1	cg04303920	0.703446895	0.580690206	0.493696141	-0.209750754	7.43E-09	6.92E-08
GENEBODY	KIAA0125	14	106390641/106394764	2	cg02507952/cg15349304	0.657353515	0.578342497	0.505681634	-0.151671881	2.80E-10	5.16E-09
GENEBODY	KIAA0406	20	36621697	1	cg10626556	0.50913721	0.396869033	0.294662441	-0.214474769	3.46E-12	3.01E-10
GENEBODY	KIAA0495	1	3661862	1	cg14912788	0.81316239	0.718901144	0.526893782	-0.286268608	6.92E-11	1.86E-09

GENEBODY	KIAA0748	12	55362424/55368244	2	cg02710015/cg06352538	0.702532575	0.519941144	0.423248273	-0.279284302	8.45E-09	7.68E-08
GENEBODY	KIAA1045	9	34977523	1	cg14380939	0.33507383	0.240988478	0.175054416	-0.160019414	7.83E-12	4.75E-10
GENEBODY	KIF3B	20	30914444	1	cg11261691	0.54080805	0.425990672	0.323157082	-0.217650968	6.34E-11	1.77E-09
GENEBODY	KLHL24	3	183382726	1	cg02628016	0.60900404	0.477955339	0.382027473	-0.226976567	5.36E-10	8.41E-09
GENEBODY	KLK5	19	51446953	1	cg11855156	0.82022472	0.703309244	0.57214575	-0.24807897	6.44E-07	3.25E-06
GENEBODY	KLK9	19	51508927/51506888	2	cg07798605/cg20103311	0.48368356	0.377226342	0.281888639	-0.201794921	8.22E-11	2.10E-09
GENEBODY	KNG1	3	186458131/186460384	2	cg10773224/cg27376355	0.847853335	0.757162011	0.654905105	-0.19294823	5.08E-10	8.09E-09
GENEBODY	KRT14	17	39739598/39739541/39739912/39739860/39739524	5	cg03508348/cg05376477/cg06339505/cg12362508/cg15748407	0.828138185	0.673960283	0.631046352	-0.197091833	4.00E-10	6.72E-09
GENEBODY	KRT18	12	53343849/53343514/53344746/53343703/53343105/53344573	6	cg04799958/cg07652628/cg11698119/cg14212748/cg22221657/cg24969443	0.4911653	0.396120811	0.316656439	-0.174508861	6.68E-11	1.84E-09
GENEBODY	KRT4	12	53200975	1	cg21182263	0.71473191	0.686663217	0.534418023	-0.180313887	9.49E-06	3.50E-05
GENEBODY	KRT71	12	52938420	1	cg08186915	0.57539468	0.496847144	0.415035227	-0.160359453	2.14E-08	1.68E-07
GENEBODY	KRT78	12	53241452	1	cg19484680	0.83751809	0.747393283	0.672402768	-0.165115322	1.20E-07	7.36E-07
GENEBODY	KRTDAP	19	35981224	1	cg05241571	0.702843185	0.613275333	0.50759995	-0.195243235	4.85E-08	3.36E-07
GENEBODY	LACRT	12	55028545	1	cg10125195	0.679076895	0.582964378	0.495928995	-0.1831479	1.57E-08	1.30E-07
GENEBODY	LAIR2	19	55014357/55019619	2	cg01807649/cg24457158	0.59280262	0.511190433	0.405668055	-0.187134565	2.02E-06	8.84E-06
GENEBODY	LALBA	12	48963631/48962956	2	cg14338548/cg25953788	0.49071579	0.345169944	0.214784636	-0.275931154	1.03E-12	1.50E-10
GENEBODY	LASS4	19	8317932	1	cg13393785	0.41950647	0.326546922	0.246700127	-0.172806343	9.54E-11	2.34E-09
GENEBODY	LBX1	10	102987483/102987257/102988094/102987407/102987993	5	cg03053579/cg12646649/cg14374861/cg15268691/cg19547319	0.316967662	0.364041074	0.489750283	0.172782622	6.08E-06	2.35E-05
GENEBODY	LCN10	9	139634272/139635449/139634196/139636616/139636493/139634063	6	cg13731311/cg13754355/cg14052065/cg14494421/cg14640244/cg22588936	0.53322766	0.458821255	0.379749383	-0.153478277	4.22E-11	1.35E-09
GENEBODY	LCN6	9	139640325/139641402/139640661/139640053/139640884	5	cg02522367/cg13752460/cg13790576/cg14633329/cg23469878	0.695805427	0.603663529	0.529409373	-0.166396054	6.46E-12	4.27E-10

GENEBODY	LCP2	5	169695565/169695483/169706238/169713179/169693176/169720975	6	cg05265596/cg08748308/cg17752270/cg21748244/cg26104475/cg26518932	0.559571199	0.470114428	0.383199486	-0.176371712	3.77E-10	6.44E-09
GENEBODY	LETM2	8	38260675	1	cg26232451	0.582946025	0.496236939	0.424657932	-0.158288093	1.13E-09	1.49E-08
GENEBODY	LHX8	1	75602857/75599532/75596758/75597304/75596930/75600124/75599645/75600250/75602412/75602167/75600421/75598280/75598070/75599667/75598039/75602846/75596724/75596827/75600399/75602925/75597485/75602451/75606694/75600224/75601276/75600391/75597887/75601120/75600685/75600941	30	cg00145253/cg00681106/cg02861380/cg03326762/cg03445694/cg05352087/cg06615678/cg07404921/cg08146483/cg08272731/cg10095577/cg10237044/cg11750396/cg11842415/cg12435629/cg12764034/cg13478928/cg13492103/cg13691003/cg14035231/cg14613402/cg14832406/cg18876007/cg19411474/cg19764599/cg23166389/cg23845450/cg23952663/cg25368122/cg26309264	0.291892009	0.376153695	0.50515138	0.213259371	1.09E-08	9.52E-08
GENEBODY	LOC100128288	17	8263420	1	cg08906309	0.63322548	0.475523	0.337774082	-0.295451398	3.37E-11	1.19E-09
GENEBODY	LOC100169752	10	127263501	1	cg02173583	0.63245405	0.598162822	0.472293395	-0.160160655	0.000206737	0.000563872
GENEBODY	LOC100190940	12	130526797/130524707	2	cg07630723/cg08968978	0.4644509	0.539078339	0.621005714	0.156554814	1.68E-06	7.52E-06
GENEBODY	LOC100192379	4	122686432/122686453/122686269/122686493/122686456/122687552/122686038/122686602/122686319/122687481/122686564/122686667	12	cg03227184/cg04638468/cg05396987/cg07978472/cg08553437/cg10423757/cg10863741/cg16733855/cg21077559/cg21544585/cg24912438/cg27516568	0.375727689	0.474573992	0.604359631	0.228631942	2.41E-10	4.63E-09
GENEBODY	LOC100192426	18	8364304/8366955	2	cg14579769/cg23436819	0.66974253	0.576310194	0.473511291	-0.196231239	2.59E-08	1.97E-07

GENEBODY	LOC145663	15	45671279/45671028/45671347/45671018/45671708/45671246/45671195/45671155/45671148/45671016/45671001	11	cg00767496/cg04025675/cg04980849/cg05896902/cg06246941/cg11032707/cg15744692/cg18435832/cg20381115/cg24328539/cg26796135	0.566041589	0.649311005	0.716725618	0.150684029	3.20E-09	3.50E-08
GENEBODY	LOC145820	15	95985725	1	cg17048700	0.490771355	0.375637294	0.280933864	-0.209837491	9.81E-14	4.89E-11
GENEBODY	LOC200726	2	207511246	1	cg15579926	0.760571855	0.677117689	0.504789355	-0.2557825	4.27E-08	3.00E-07
GENEBODY	LOC201651	3	151499380	1	cg09683623	0.887488425	0.772692844	0.727886791	-0.159601634	4.43E-08	3.10E-07
GENEBODY	LOC282997	10	112628888	1	cg24662666	0.47664464	0.55340505	0.662365536	0.185720896	1.12E-07	6.94E-07
GENEBODY	LOC283392	12	72667236/72665510/72666264/72665880/72666281/72666976/72665376/72665282/72665771/72667150	10	cg01030121/cg01817029/cg02511156/cg04775889/cg06622999/cg13663218/cg17771605/cg18440199/cg23142394/cg27579953	0.2548672	0.328457629	0.493095163	0.238227963	2.07E-07	1.19E-06
GENEBODY	LOC283856	16	56224782/56150071/56224504/56224901/56224627/56224793/56221534	7	cg00866976/cg00962984/cg04156464/cg06728579/cg08613597/cg10273340/cg26832183	0.293019313	0.334235684	0.453282458	0.160263145	1.29E-06	5.98E-06
GENEBODY	LOC283914	16	34625446/34598029	2	cg03898993/cg26929163	0.58920205	0.527912472	0.395218659	-0.193983391	5.49E-08	3.73E-07
GENEBODY	LOC284749	20	46997755	1	cg22849543	0.721923115	0.583982667	0.454996091	-0.266927024	4.14E-09	4.30E-08
GENEBODY	LOC284805	20	31239171	1	cg26331247	0.46670876	0.331261439	0.237858136	-0.228850624	2.62E-11	1.03E-09
GENEBODY	LOC284837	21	45232232	1	cg00546897	0.513421185	0.387064144	0.314571205	-0.19884998	1.80E-11	7.99E-10
GENEBODY	LOC285370	3	10801997	1	cg07147846	0.489283015	0.353464739	0.231720641	-0.257562374	3.82E-12	3.21E-10
GENEBODY	LOC339568	20	37850248	1	cg04742334	0.57372006	0.391180028	0.263340682	-0.310379378	2.45E-12	2.58E-10
GENEBODY	LOC388499	19	7944881	1	cg20953510	0.82090899	0.697451539	0.554358145	-0.266550845	1.93E-09	2.33E-08
GENEBODY	LOC389705	9	14993466/14993584	2	cg13974505/cg14052511	0.271212788	0.323456475	0.43013973	0.158926942	5.15E-07	2.66E-06
GENEBODY	LOC401463	8	65489065/65489554/65488915/65488640/65488839/65488167/65488308/65489040/65489653/65488069	10	cg07283114/cg08188993/cg08334899/cg14930884/cg15740363/cg16510548/cg21917084/cg23554956/cg23778149/cg24255371	0.238468107	0.309763284	0.438828974	0.200360867	7.71E-06	2.91E-05

GENEBODY	LOC642587	1	209604583/209605401/209604397/209605223/209602955/209605304/209605104/209604378	8	cg01334432/cg03794445/cg05922993/cg12386297/cg14206882/cg17848546/cg17860090/cg27261597	0.694683624	0.606654352	0.502327369	-0.192356255	3.21E-11	1.16E-09
GENEBODY	LOC643406	20	5451900	1	cg04281261	0.74304902	0.662902644	0.521782745	-0.221266275	4.85E-07	2.53E-06
GENEBODY	LOC643719	19	35068555/35068221	2	cg23200020/cg25418852	0.438227565	0.589002589	0.745249427	0.307021862	4.15E-10	6.92E-09
GENEBODY	LOC643763	8	63895823	1	cg26022010	0.52041584	0.420473333	0.348848123	-0.171567717	1.14E-08	9.92E-08
GENEBODY	LOC645851	17	26937650	1	cg12560320	0.74944403	0.622230394	0.536892841	-0.212551189	2.15E-09	2.54E-08
GENEBODY	LOC647121	1	121261146/121261404/121261077/121261369/121261602/121305260	6	cg04374231/cg05827631/cg06970370/cg07794500/cg19165105/cg20769683	0.323912622	0.489471651	0.5845832	0.260670578	7.51E-12	4.64E-10
GENEBODY	LOC728989	1	146503900/146495202/146494928/146498485	4	cg13951007/cg19504089/cg24735611/cg27533767	0.75537632	0.645891483	0.566257505	-0.189118815	2.21E-09	2.60E-08
GENEBODY	LOC84931	2	121223534/121223909/121223847/121223740	4	cg07412254/cg17204557/cg21406402/cg23006519	0.533301478	0.396822489	0.314228018	-0.219073459	1.66E-12	1.99E-10
GENEBODY	LOR	1	153234037/153234342/153234268	3	cg08611411/cg11534293/cg21860629	0.090814989	0.186863206	0.375520182	0.284705193	7.78E-06	2.93E-05
GENEBODY	LPAR3	1	85313079/85288233/85313514/85331084	4	cg07110659/cg15937525/cg21422605/cg24574111	0.69009625	0.606518324	0.524164918	-0.165931332	1.07E-11	5.67E-10
GENEBODY	LRRC18	10	50120250	1	cg23211310	0.85665725	0.733184089	0.614679545	-0.241977705	3.66E-10	6.28E-09
GENEBODY	LRRC42	1	54423202	1	cg20460166	0.431117675	0.345345439	0.270230618	-0.160887057	1.02E-09	1.37E-08
GENEBODY	LRRC4C	11	40136810	1	cg00264754	0.612060085	0.479150444	0.376509877	-0.235550208	1.31E-13	5.53E-11
GENEBODY	LRRIQ3	1	74620779	1	cg14795163	0.6588679	0.5388706	0.372009832	-0.286858068	1.78E-09	2.18E-08
GENEBODY	LY6G6D	6	31683352/31685440/31685494/31685430/31685575/31685392/31685491/31685542	8	cg05218245/cg09409956/cg11165765/cg12951348/cg16271954/cg21952119/cg26015971/cg27248571	0.70397665	0.57882553	0.520170976	-0.183805674	4.75E-08	3.29E-07
GENEBODY	LY6H	8	144240945/144241434/144241120/144241104/144241407	5	cg00831710/cg02539855/cg09528449/cg10821845/cg23484951	0.332556566	0.417059477	0.510412279	0.177855714	1.24E-05	4.44E-05
GENEBODY	LY96	8	74903801	1	cg23732024	0.574359845	0.687735311	0.760874386	0.186514541	1.38E-09	1.75E-08

GENEBODY	LYG2	2	99867344	1	cg12213414	0.73208942	0.525633244	0.400153191	-0.331936229	2.73E-12	2.65E-10
GENEBODY	LYNX1	8	143856487/143851375/143857045/143852576/143856122/143851287/143852085/143852587/143851427/143851371	10	cg00801134/cg01652021/cg02228226/cg02415014/cg04350571/cg08530859/cg10210076/cg16120147/cg17770910/cg19877103	0.674260382	0.588288344	0.502342182	-0.1719182	1.22E-12	1.62E-10
GENEBODY	LYPD4	19	42342105	1	cg07959016	0.829059155	0.735874372	0.554447673	-0.274611482	4.53E-08	3.16E-07
GENEBODY	LYPLA2P1	6	33333563/33333945	2	cg06199753/cg25955341	0.764087635	0.677827439	0.589828523	-0.174259112	6.59E-13	1.15E-10
GENEBODY	MCHR2	6	100380550	1	cg07108581	0.649549445	0.485307878	0.381360482	-0.268188963	2.03E-11	8.69E-10
GENEBODY	MCOLN3	1	85510988	1	cg24535650	0.822618095	0.781970011	0.604710259	-0.217907836	4.66E-06	1.85E-05
GENEBODY	MEF2A	15	100249186	1	cg07382129	0.49037118	0.398517056	0.331268505	-0.159102675	1.19E-09	1.56E-08
GENEBODY	MEIS3P1	17	15690181/15690713/15692425	3	cg02539586/cg02628260/cg03956426	0.795992275	0.739504044	0.625972723	-0.170019552	1.86E-08	1.50E-07
GENEBODY	MGST1	12	16512895/16513009/16512994	3	cg00874480/cg04885072/cg15284911	0.662120378	0.594295	0.483937057	-0.178183321	1.74E-06	7.76E-06
GENEBODY	MIR124-1	8	9760957	1	cg00571033	0.33505103	0.403189689	0.497906564	0.162855534	2.17E-05	7.35E-05
GENEBODY	MIR124-2	8	65291730	1	cg04559779	0.448102125	0.571445239	0.678373882	0.230271757	2.01E-09	2.40E-08
GENEBODY	MIR124-3	20	61809911/61809932	2	cg06660530/cg18772588	0.130490972	0.244630445	0.388906181	0.258415209	5.08E-05	0.000158248
GENEBODY	MIR128-1	2	136422981	1	cg01243312	0.5233833	0.409603739	0.343384568	-0.179998732	3.23E-09	3.52E-08
GENEBODY	MIR129-2	11	43602965	1	cg03365311	0.2920819	0.386223178	0.586951064	0.294869164	4.39E-10	7.19E-09
GENEBODY	MIR130A	11	57408751	1	cg10512089	0.536843185	0.444654989	0.328181227	-0.208661958	2.01E-11	8.68E-10
GENEBODY	MIR137	1	98511629	1	cg04293733	0.353616195	0.387999328	0.558625427	0.205009232	1.16E-05	4.19E-05
GENEBODY	MIR141	12	7073264	1	cg02624246	0.527913925	0.427904056	0.3544678	-0.173446125	2.62E-10	4.91E-09
GENEBODY	MIR146B	10	104196339	1	cg13442016	0.727210405	0.607243967	0.501807336	-0.225403069	1.24E-09	1.61E-08
GENEBODY	MIR187	18	33484837	1	cg05827233	0.765774795	0.670894844	0.600741077	-0.165033718	1.81E-08	1.47E-07
GENEBODY	MIR192	11	64658622	1	cg02258444	0.538769055	0.423091144	0.338631518	-0.200137537	4.47E-11	1.41E-09
GENEBODY	MIR194-2	11	64658903	1	cg24803202	0.53691924	0.42221775	0.333940036	-0.202979204	4.98E-12	3.73E-10
GENEBODY	MIR200C	12	7072891	1	cg18959988	0.55218625	0.471094	0.384008641	-0.168177609	3.14E-11	1.15E-09
GENEBODY	MIR206	6	52009154	1	cg25284213	0.551188985	0.4380045	0.345680914	-0.205508071	8.20E-10	1.16E-08
GENEBODY	MIR216B	2	56227870	1	cg01160855	0.657757845	0.546920028	0.49197955	-0.165778295	6.07E-07	3.08E-06
GENEBODY	MIR2276	13	24736569	1	cg22412649	0.495311715	0.40770505	0.327803223	-0.167508492	3.49E-12	3.01E-10
GENEBODY	MIR299	14	101490186/101490145	2	cg00175487/cg10082525	0.59552164	0.481105761	0.390757355	-0.204764285	2.80E-12	2.65E-10
GENEBODY	MIR29A	7	130561511	1	cg11370011	0.644881505	0.553026489	0.487165309	-0.157716196	2.80E-11	1.07E-09

GENEBODY	MIR30A	6	72113322	1	cg19098437	0.62109517	0.601727172	0.454195595	-0.166899575	6.09E-06	2.36E-05
GENEBODY	MIR338	17	79099711	1	cg26766064	0.511717495	0.349082661	0.3546869	-0.157030595	9.15E-05	0.000268958
GENEBODY	MIR34B	11	111383668	1	cg01192900	0.19902581	0.250600589	0.418149077	0.219123267	1.96E-05	6.70E-05
GENEBODY	MIR365-1	16	14403144	1	cg02886263	0.46594534	0.568260911	0.653808695	0.187863355	4.99E-09	5.01E-08
GENEBODY	MIR376C	14	101506087	1	cg13995230	0.464168205	0.28744265	0.192639905	-0.2715283	6.68E-12	4.35E-10
GENEBODY	MIR382	14	101520662	1	cg26238975	0.87810463	0.795733122	0.714647409	-0.163457221	0.000883996	0.002105938
GENEBODY	MIR425	3	49057661	1	cg07109801	0.407468335	0.330439617	0.256427364	-0.151040971	6.07E-10	9.24E-09
GENEBODY	MIR449A	5	54466394	1	cg13650580	0.833229595	0.769999867	0.681101841	-0.152127754	3.10E-05	0.000101231
GENEBODY	MIR495	14	101500125	1	cg14910227	0.69446684	0.536242444	0.459293677	-0.235173163	1.75E-08	1.43E-07
GENEBODY	MIR519D	19	54216680	1	cg04573316	0.5399079	0.5916258	0.694703123	0.154795223	5.74E-05	0.000176731
GENEBODY	MIR548A3	3	103926553/103930387	2	cg17865718/cg21938179	0.73800807	0.621865211	0.509194032	-0.228814038	7.97E-06	3.00E-05
GENEBODY	MIR623	13	100008450	1	cg16193278	0.48301897	0.5792346	0.635709114	0.152690144	1.04E-06	4.96E-06
GENEBODY	MIR802	21	37093012	1	cg00871610	0.534480715	0.4201374	0.378997414	-0.155483301	1.51E-05	5.29E-05
GENEBODY	MIR885	3	10436197	1	cg08044634	0.857566015	0.760420911	0.678120305	-0.17944571	2.86E-05	9.43E-05
GENEBODY	MIR9-3	15	89911298/89911317	2	cg03082580/cg13888600	0.172127494	0.222306133	0.345540528	0.173413034	7.44E-06	2.82E-05
GENEBODY	MLN	6	33767951/33764239	2	cg01584000/cg10378792	0.743819225	0.683719567	0.576965218	-0.166854007	3.02E-07	1.67E-06
GENEBODY	MMP10	11	102651208	1	cg02061229	0.44091554	0.351989117	0.255131745	-0.185783795	1.84E-08	1.48E-07
GENEBODY	MMP7	11	102396717	1	cg25812146	0.590088195	0.490143678	0.414655773	-0.175432422	4.96E-06	1.96E-05
GENEBODY	MNDA	1	158817808	1	cg14216734	0.75194808	0.661976394	0.531712809	-0.220235271	5.22E-07	2.70E-06
GENEBODY	MOGAT2	11	75429059	1	cg12531542	0.60581302	0.484689983	0.391481118	-0.214331902	1.13E-10	2.67E-09
GENEBODY	MPDZ	9	13249504/13136318	2	cg03905144/cg14539466	0.60568189	0.467571292	0.349236009	-0.256445881	6.53E-15	1.35E-11
GENEBODY	MPPED2	11	30502936/30474351/30567189/30597981/30444984/30601783/30510489/30531731/30431965/30488071/30428652/30582012	12	cg01438090/cg02403522/cg05568454/cg05600421/cg05798059/cg06790862/cg17320698/cg19949932/cg20495540/cg20871721/cg23064082/cg25541653	0.588468317	0.517699758	0.42986966	-0.158598657	2.51E-09	2.89E-08
GENEBODY	MRAP2	6	84798113/84771783	2	cg08327884/cg23508908	0.623052445	0.513072711	0.461213064	-0.161839381	1.06E-06	5.07E-06
GENEBODY	MS4A7	11	60157161	1	cg08716584	0.49240782	0.630249239	0.651807773	0.159399953	2.37E-05	7.94E-05
GENEBODY	MSC	8	72755871/72755052/72755784/72755568/72754953/72755162	6	cg06269753/cg13620034/cg20079899/cg26280666/cg26799209/cg26876974	0.198986605	0.310751587	0.468738948	0.269752343	2.48E-08	1.90E-07

GENEBODY	MST1R	3	49938721/49938004/49939703	3	cg01917209/cg15211623/cg18292904	0.720263333	0.550355703	0.421140836	-0.299122496	5.34E-10	8.40E-09
GENEBODY	MSX1	4	4864833/4862770/4862184/4864625/4863356/4863874/4864532/4862240/4862910/4864293/4864110/4863282/4864711/4864488/4863016/4863678/4864430/4864302/4864320	19	cg01785568/cg03199651/cg03717979/cg03843978/cg06375949/cg09573795/cg09748975/cg14039306/cg14167596/cg15848031/cg19596468/cg20161179/cg20891301/cg21538208/cg22256027/cg22609784/cg24840099/cg25144207/cg27038439	0.386871456	0.458415534	0.552110839	0.165239383	1.24E-09	1.60E-08
GENEBODY	MSX2P1	17	56234826/56234476/56234897	3	cg09123473/cg21502786/cg21934311	0.238256188	0.323847989	0.466468558	0.22821237	3.98E-09	4.16E-08
GENEBODY	MURC	9	103342099	1	cg14429381	0.767664675	0.633701094	0.53592375	-0.231740925	8.24E-09	7.53E-08
GENEBODY	MVK	12	110025893/110026822/110026737/110026787/110013740/110026028	6	cg15342134/cg16260888/cg20424400/cg23364246/cg27263151/cg27604975	0.750296586	0.638155857	0.5424841	-0.207812486	1.72E-10	3.63E-09
GENEBODY	MYBL1	8	67521144	1	cg11785625	0.56504327	0.492096567	0.391296595	-0.173746675	7.45E-08	4.87E-07
GENEBODY	MYBPHL	1	109842096	1	cg17797848	0.731521935	0.590280228	0.492371995	-0.23914994	7.03E-12	4.45E-10
GENEBODY	MYCT1	6	153023279	1	cg15961007	0.35434064	0.253370928	0.170843986	-0.183496654	3.15E-13	7.92E-11
GENEBODY	MYF5	12	81111558	1	cg10868277	0.66851339	0.5278959	0.411802368	-0.256711022	5.25E-11	1.57E-09
GENEBODY	MYF6	12	81102123/81102486/81102562/81102035	4	cg00756032/cg05860723/cg05981335/cg22344727	0.366367685	0.474323297	0.569307997	0.202940312	1.43E-09	1.80E-08
GENEBODY	MYH1	17	10419499	1	cg00134787	0.77961829	0.688382022	0.587537686	-0.192080604	1.52E-05	5.32E-05
GENEBODY	MYH16	7	98885577/98872169/98885594/98871769/98889946	5	cg10213114/cg12492380/cg13373361/cg14606858/cg19428602	0.46999639	0.357906294	0.280486516	-0.189509874	9.29E-12	5.17E-10
GENEBODY	MYL1	2	211179560/211168547/211169638	3	cg11059341/cg13027933/cg27305342	0.796977923	0.707156278	0.599318057	-0.197659866	4.45E-06	1.78E-05
GENEBODY	MYL7	7	44180604	1	cg23370883	0.45903084	0.3427988	0.243010723	-0.216020117	2.27E-12	2.50E-10
GENEBODY	MYOD1	11	17742741/17742502/17742759/17742135	4	cg02728629/cg20289688/cg22040469/cg22926931	0.321351611	0.37751459	0.503118425	0.181766814	5.46E-06	2.13E-05

GENEBODY	MYOG	1	203053787	1	cg25145680	0.806581825	0.734224256	0.643965336	-0.162616489	2.32E-06	9.97E-06
GENEBODY	NCAM2	21	22373039/22372231	2	cg14355482/cg18345924	0.538012175	0.412442728	0.321368955	-0.21664322	4.43E-11	1.40E-09
GENEBODY	NCKAP1L	12	54935674/54920931	2	cg09039845/cg21072795	0.495273475	0.383999019	0.302932772	-0.192340703	1.33E-11	6.59E-10
GENEBODY	NCRNA00032	9	27282580/27248473/27282776	3	cg02388718/cg13913108/cg24670241	0.720612592	0.576927907	0.487126485	-0.233486107	3.14E-14	3.21E-11
GENEBODY	NCRNA00099	4	21849543	1	cg24817257	0.693328685	0.628690144	0.490776255	-0.20255243	5.40E-07	2.77E-06
GENEBODY	NCRNA00157	21	19231611	1	cg20316715	0.63082684	0.481931183	0.340326555	-0.290500285	6.41E-13	1.14E-10
GENEBODY	NCRNA00158	21	26803075	1	cg25864727	0.883825255	0.817263661	0.710849077	-0.172976178	5.56E-05	0.000171714
GENEBODY	NCRNA00207	22	44965381	1	cg06215908	0.497915075	0.4083154	0.294756077	-0.203158998	9.96E-09	8.86E-08
GENEBODY	NDST3	4	119093021/118975504/119095240	3	cg06837981/cg12311175/cg26126295	0.579595065	0.426202117	0.344429245	-0.23516582	1.04E-08	9.16E-08
GENEBODY	NEFH	22	29877285	1	cg20256245	0.35193242	0.450648994	0.581901541	0.229969121	9.50E-08	6.02E-07
GENEBODY	NEUROD1	2	182542864/182542901/182543233	3	cg01431993/cg11528849/cg16640855	0.379430115	0.458889536	0.660677221	0.281247107	5.18E-10	8.21E-09
GENEBODY	NEUROG3	10	71332776/71332744	2	cg12938159/cg26333837	0.199739422	0.271869736	0.472729803	0.272990381	1.19E-09	1.55E-08
GENEBODY	NF1P1	15	21131137	1	cg15400766	0.686234795	0.599878083	0.497365195	-0.1888696	4.12E-10	6.89E-09
GENEBODY	NHEDC1	4	103867628	1	cg15623444	0.85882682	0.7135649	0.550782377	-0.308044443	1.92E-08	1.54E-07
GENEBODY	NINL	20	25450839/25479848/25448861	3	cg09614389/cg13486641/cg26101502	0.683284493	0.593167531	0.433255398	-0.250029094	7.22E-07	3.60E-06
GENEBODY	NKAIN4	20	61884645/61878356/61885448/61885236/61877875/61885285/61876246/61885270/61883856/61877942/61878032/61885249/61885291/61876295/61874249/61885262	16	cg01883482/cg05133591/cg05326358/cg06763829/cg07711085/cg07785314/cg08878177/cg09196068/cg10436333/cg12091786/cg12649455/cg15571277/cg18412834/cg19610126/cg20738665/cg21258057	0.28987586	0.339207221	0.458193063	0.168317203	2.10E-06	9.12E-06
GENEBODY	NKX2-2	20	21493101/21492823/21492914	3	cg01397679/cg21977377/cg22474464	0.30181082	0.394914831	0.568672383	0.266861563	1.72E-06	7.66E-06
GENEBODY	NKX2-5	5	172661132/172660884/172660796/172661487/172659730/172660996/172660080	7	cg02051616/cg11330740/cg15929797/cg18017926/cg19923650/cg22730140/cg25916711	0.38725583	0.456078736	0.57162879	0.18437296	3.96E-07	2.11E-06

GENEBODY	NKX2-6	8	23563146/23563182/23563572/23562003/23560590/23560319/23562918/23560348/23559988	9	cg00425918/cg01336781/cg03330710/cg04434896/cg05310249/cg10345920/cg14451926/cg15446990/cg25499067	0.285703071	0.348424144	0.481923168	0.196220098	3.13E-09	3.44E-08
GENEBODY	NKX6-2	10	134598496/134598908/134598927	3	cg04436469/cg06082548/cg12816961	0.362629477	0.403539039	0.524397276	0.161767799	3.25E-08	2.39E-07
GENEBODY	NLGN1	3	173574358/173768723/173681262/173740927/173438075/173920226/173457176/173632551/173630182/173992754/173917837/173639566/173583617/173467741	14	cg00493429/cg01982637/cg02328758/cg02521771/cg02910194/cg06097077/cg07452098/cg14890220/cg15558616/cg17482360/cg19510409/cg21548131/cg21904441/cg22960621	0.662820778	0.554055089	0.472206206	-0.190614572	7.09E-12	4.47E-10
GENEBODY	NLRP10	11	7982706	1	cg06118609	0.637352005	0.553022311	0.443522486	-0.193829519	7.74E-07	3.82E-06
GENEBODY	NLRP7	19	55447414/55449439/55450872/55452080	4	cg01560554/cg14928510/cg18778097/cg26484916	0.696041305	0.566957344	0.442922027	-0.253119278	1.05E-08	9.19E-08
GENEBODY	NPFFR2	4	72900088/72950329/72903914/72925371/72903841/72898212/73012174/72996777	8	cg08414965/cg12986700/cg14010852/cg14992771/cg15264404/cg15867728/cg16084416/cg24923953	0.608458698	0.538041629	0.422329807	-0.186128891	8.81E-11	2.21E-09
GENEBODY	NPS	10	129349644	1	cg24631102	0.76417872	0.684049994	0.614130623	-0.150048097	0.001372952	0.003149298
GENEBODY	NPTXR	22	39238576/39236408	2	cg01503706/cg15251408	0.755539335	0.667006328	0.52920018	-0.226339155	8.02E-07	3.94E-06
GENEBODY	NR3C1	5	142729913/142779552/142757011/142780254/142740314/142692961/142757312/142776274	8	cg03857453/cg06613263/cg16586394/cg17342132/cg18484679/cg19457823/cg25535999/cg27107893	0.652768263	0.55125101	0.442838094	-0.209930169	3.63E-10	6.25E-09
GENEBODY	NRG4	15	76297890/76287483	2	cg21755367/cg22112083	0.666402755	0.568845317	0.470192141	-0.196210614	1.07E-09	1.42E-08
GENEBODY	OBP2A	9	138439003	1	cg14582108	0.7758893	0.697674594	0.5895234	-0.1863659	2.04E-06	8.88E-06
GENEBODY	OC90	8	133056586/133062974/133062851/133062214	4	cg00867889/cg03344782/cg07665115/cg20220737	0.710621694	0.58002026	0.441747478	-0.268874215	2.08E-13	6.68E-11

GENEBODY	OLAH	10	15110983	1	cg04762351	0.550208665	0.408004789	0.284452264	-0.265756401	3.22E-15	9.31E-12
GENEBODY	OLFM4	13	53618826/53603286	2	cg02570298/cg12582008	0.57908602	0.478155692	0.42762375	-0.15146227	1.08E-09	1.44E-08
GENEBODY	OLIG2	21	34399260/34400072/34399361	3	cg05238769/cg11950383/cg14293300	0.330209048	0.402073697	0.524951309	0.194742262	8.42E-09	7.66E-08
GENEBODY	OLR1	12	10322420	1	cg12203957	0.74355201	0.701605889	0.557129691	-0.186422319	4.02E-05	0.000128319
GENEBODY	OPTC	1	203465361	1	cg04830316	0.420095985	0.328130639	0.229627336	-0.190468649	2.78E-11	1.07E-09
GENEBODY	OR1F2P	16	3266276	1	cg08748105	0.64413183	0.556193817	0.4051304	-0.23900143	1.49E-07	8.91E-07
GENEBODY	OR2H1	6	29430096/29430435/29429909/29430158/29430334	5	cg00114160/cg01311802/cg15641340/cg16343924/cg21468949	0.559305293	0.478241569	0.40384861	-0.155456682	7.84E-11	2.02E-09
GENEBODY	OR2L13	1	248262824	1	cg16717532	0.534526385	0.435464461	0.331320441	-0.203205944	8.62E-13	1.35E-10
GENEBODY	OR7E91P	2	71251453/71251634	2	cg00208218/cg12073208	0.685742783	0.569623172	0.520958036	-0.164784746	1.13E-06	5.34E-06
GENEBODY	ORMDL2	12	56213916	1	cg27554856	0.880130045	0.724828161	0.691191491	-0.188938554	4.08E-07	2.16E-06
GENEBODY	OSR2	8	99961376/99963021/99961874/99961768/99961545/99963657/99962118/99962347/99962013	9	cg00388812/cg01148781/cg01581084/cg05754861/cg08202494/cg09607488/cg16746576/cg18744668/cg20823772	0.402276217	0.474009469	0.585376011	0.183099794	1.86E-05	6.39E-05
GENEBODY	OSTN	3	190931199/190956840	2	cg03192737/cg26764579	0.790382615	0.732383383	0.629806091	-0.160576524	8.03E-05	0.000239187
GENEBODY	OTX1	2	63281844/63282043/63280376/63281383/63282702/63282211/63283013/63282883/63280625/63281317/63283355/63280164/63281139/63281069	14	cg00445405/cg00463767/cg02606423/cg03655683/cg04654530/cg04808506/cg07974511/cg08473330/cg10640333/cg10859133/cg14604681/cg22732253/cg25622366/cg27364741	0.309800656	0.336931864	0.461400737	0.15160008	0.000252557	0.000676305
GENEBODY	PAFAH1B1	17	2551652	1	cg04957146	0.78390076	0.679253189	0.613538786	-0.170361974	7.14E-08	4.69E-07
GENEBODY	PAMR1	11	35475769/35499741/35546824/35520863/35542580	5	cg01664929/cg10832281/cg15406469/cg16273509/cg22785492	0.682185765	0.634645744	0.527421585	-0.15476418	4.32E-07	2.28E-06
GENEBODY	PATE3	11	125660444	1	cg24746315	0.747075705	0.580691672	0.517078341	-0.229997364	8.09E-06	3.04E-05
GENEBODY	PATE4	11	125707106	1	cg22329515	0.49080638	0.352153789	0.270334636	-0.220471744	1.62E-14	2.01E-11

GENEBODY	PAX6	11	31820572/31822341/31819678/31819162/31821064/31819148/31821590/31825214/31819464/31823191/31827775/31817824/31823141/31825605/31824327/31827379/31825166/31827593/31826574/31825833/31826421/31825756/31823282/31821452/31823238/31827899/31824973/31817881/31822243/31819219/31820441/31821388/31822302/31820209/31827757/31827084/31824262/31825792/31819444/31818474/31821764/31827920/31820658/31825969/31821298/31822526/31818791/31817810/31821313/31819380/31825226	51	cg01587682/cg02770983/cg02771142/cg03854796/cg03905867/cg04521333/cg04598774/cg04938549/cg06666008/cg06705930/cg07124117/cg07478918/cg08116462/cg08199056/cg08499046/cg08784129/cg09224689/cg09382096/cg09537620/cg11128216/cg11469061/cg11827910/cg12798259/cg13023210/cg13245152/cg13596833/cg13891702/cg14800351/cg15301794/cg16180353/cg16615954/cg16616521/cg16865446/cg16970357/cg18082638/cg18372607/cg18988498/cg20014398/cg21016855/cg21582373/cg22392038/cg22557091/cg22740492/cg22982368/cg23125492/cg23287710/cg24332783/cg25764105/cg26019112/cg26029734/cg27011060	0.315493726	0.366353366	0.498567282	0.183073555	1.92E-07	1.12E-06

GENEBODY	PAX9	14	37131329/37132490/37133285/37135435/37133116/37132291/37135898/37132774/37135384/37132665/37133714/37133168/37131998/37136192/37136391/37131601/37135671/37131555/37136283/37132764/37135586	21	cg01002381/cg01794426/cg01803925/cg04994761/cg05842348/cg06749715/cg06756919/cg07397956/cg09814975/cg10741025/cg10913846/cg11091113/cg13678371/cg17696160/cg18361098/cg19032066/cg20710709/cg22968936/cg23683588/cg24066316/cg26094789	0.352632712	0.418403506	0.527369436	0.174736724	7.91E-09	7.29E-08
GENEBODY	PCDH17	13	58240705/58299015/58227960/58246705/58256880/58299073	6	cg02994463/cg07629125/cg19637591/cg20594465/cg22103831/cg24586528	0.615687336	0.530037866	0.414609537	-0.201077799	2.84E-12	2.65E-10
GENEBODY	PCDH8	13	53420135/53419963/53419617/53419596/53419729	5	cg06460717/cg07847863/cg14950829/cg18002116/cg25600049	0.297761536	0.355381107	0.457716873	0.159955337	6.31E-06	2.43E-05

GENEBODY	PCDHGA10	5	140855562/140857995/140807787/140806126/140867386/140821244/140811520/140802831/140864578/140888468/140821687/140873765/140810404/140798095/140855547/140810109/140798758/140803130/140855463/140799811/140796240/140864593/140800495/140868130/140811253/140819300/140856330/140798188/140802804/140871156/140870164/140824037/140805732/140800586/140864276/140800929/140802135/140856469/140855857/140811642/140796892/140811312/140822776/140810137/140810260/140857813/140807666/140800983/140798161/140797509/140818522/140857474/140810920/140797280/140800629/140812464/140855582/140810161/140865433/140864834/140810123/140872334/140864474/140857192/140807490/	125	cg00281842/cg00589850/cg00808170/cg00888801/cg00943245/cg01000954/cg01224715/cg01729977/cg02022733/cg02056653/cg02161292/cg02249657/cg02267483/cg02331883/cg02430347/cg02452944/cg02707176/cg02715006/cg02780295/cg03456338/cg03486475/cg03640756/cg03892308/cg03898832/cg04080041/cg04117404/cg04453180/cg04503600/cg04845915/cg04964031/cg05149776/cg05197036/cg05670472/cg05870739/cg06428620/cg06572465/cg07179872/cg07445963/cg07448606/cg07489502/cg07524997/cg07539798/cg07608474/cg07730329/cg07802710/cg08155325/cg08200869/cg08395122/cg08935238/cg08938584/cg09484752/cg09581226/cg09639151/cg10435816/cg10679597/cg10805220/cg10858746/cg11647681/cg11830096/cg12145907/cg12304520/cg12487901/cg12648074/cg1287703	0.530000746	0.607268385	0.689949933	0.159949187	2.50E-10	4.74E-09

GENEBODY	PCDHGA11	5	140855562/140857995/140807787/140806126/140867386/140821244/140811520/140802831/140864578/140888468/140821687/140873765/140810404/140855547/140810109/140803130/140855463/140864593/140868130/140811253/140819300/140856330/140802804/140871156/140870164/140824037/140805732/140864276/140856469/140855857/140811642/140811312/140822776/140810137/140810260/140857813/140807666/140818522/140857474/140810920/140812464/140855582/140810161/140865433/140864834/140810123/140872334/140864474/140857192/140807490/140821425/140855482/140810726/140857941/140805681/140890718/140855469/140864701/140864549/140868422/140871464/140805841/140861242/140867724/140871590/	92	cg00281842/cg00589850/cg00808170/cg00888801/cg00943245/cg01000954/cg01224715/cg01729977/cg02022733/cg02056653/cg02161292/cg02249657/cg02267483/cg02430347/cg02452944/cg02715006/cg02780295/cg03640756/cg03898832/cg04080041/cg04117404/cg04453180/cg04845915/cg04964031/cg05149776/cg05197036/cg05670472/cg06428620/cg07445963/cg07448606/cg07489502/cg07539798/cg07608474/cg07730329/cg07802710/cg08155325/cg08200869/cg09484752/cg09581226/cg09639151/cg10805220/cg10858746/cg11647681/cg11830096/cg12145907/cg12304520/cg12487901/cg12648074/cg12877039/cg12972081/cg13023584/cg13993336/cg14253517/cg14672084/cg14681511/cg15004405/cg15124968/cg15361590/cg15949044/cg15988320/cg16032518/cg16316394/cg16548916/cg1710838	0.53032639	0.600464549	0.682415576	0.152089186	1.97E-10	3.99E-09

GENEBODY	PCDHGA8	5	140782367/140779112/140855562/140857995/140807787/140806126/140867386/140821244/140792918/140811520/140792558/140802831/140864578/140888468/140779461/140821687/140873765/140810404/140798095/140855547/140810109/140778546/140798758/140803130/140855463/140794962/140788613/140799811/140796240/140777607/140864593/140800495/140787013/140868130/140811253/140819300/140856330/140798188/140777501/140793307/140802804/140871156/140870164/140824037/140805732/140800586/140775631/140864276/140800929/140789450/140783647/140789247/140802135/140794359/140856469/140855857/140811642/140796892/140811312/140822776/140787430/140793470/140810137/140810260/140782755/	176	cg00048743/cg00118365/cg00281842/cg00589850/cg00808170/cg00888801/cg00943245/cg01000954/cg01169021/cg01224715/cg01311360/cg01729977/cg02022733/cg02056653/cg02074191/cg02161292/cg02249657/cg02267483/cg02331883/cg02430347/cg02452944/cg02563137/cg02707176/cg02715006/cg02780295/cg03400785/cg03415916/cg03456338/cg03486475/cg03608712/cg03640756/cg03892308/cg03898577/cg03898832/cg04080041/cg04117404/cg04453180/cg04503600/cg04553690/cg04726784/cg04845915/cg04964031/cg05149776/cg05197036/cg05670472/cg05870739/cg06065872/cg06428620/cg06572465/cg06757405/cg06776159/cg07017875/cg07179872/cg07231479/cg07445963/cg07448606/cg07489502/cg07524997/cg07539798/cg07608474/cg07613945/cg07620533/cg07730329/cg0780271	0.520675076	0.598193152	0.671267785	0.150592708	1.71E-10	3.60E-09

GENEBODY	PCDHGA9	5	140855562/140857995/140807787/140806126/140867386/140821244/140792918/140811520/140792558/140802831/140864578/140888468/140821687/140873765/140810404/140798095/140855547/140810109/140798758/140803130/140855463/140794962/140788613/140799811/140796240/140864593/140800495/140787013/140868130/140811253/140819300/140856330/140798188/140793307/140802804/140871156/140870164/140824037/140805732/140800586/140864276/140800929/140789450/140789247/140802135/140794359/140856469/140855857/140811642/140796892/140811312/140822776/140787430/140793470/140810137/140810260/140857813/140807666/140800983/140789708/140798161/140797509/140787623/140818522/140857474/	152	cg00281842/cg00589850/cg00808170/cg00888801/cg00943245/cg01000954/cg01169021/cg01224715/cg01311360/cg01729977/cg02022733/cg02056653/cg02161292/cg02249657/cg02267483/cg02331883/cg02430347/cg02452944/cg02707176/cg02715006/cg02780295/cg03400785/cg03415916/cg03456338/cg03486475/cg03640756/cg03892308/cg03898577/cg03898832/cg04080041/cg04117404/cg04453180/cg04503600/cg04726784/cg04845915/cg04964031/cg05149776/cg05197036/cg05670472/cg05870739/cg06428620/cg06572465/cg06757405/cg07017875/cg07179872/cg07231479/cg07445963/cg07448606/cg07489502/cg07524997/cg07539798/cg07608474/cg07613945/cg07620533/cg07730329/cg07802710/cg08155325/cg08200869/cg08395122/cg08854987/cg08935238/cg08938584/cg09465698/cg0948475	0.513139906	0.592403142	0.667432175	0.154292269	1.45E-10	3.21E-09

GENEBODY	PCDHGB5	5	140782367/140855562/140857995/140807787/140806126/140867386/140821244/140792918/140811520/140792558/140802831/140864578/140888468/140821687/140873765/140810404/140798095/140855547/140810109/140798758/140803130/140855463/140794962/140788613/140799811/140796240/140864593/140800495/140787013/140868130/140811253/140819300/140856330/140798188/140793307/140802804/140871156/140870164/140824037/140805732/140800586/140864276/140800929/140789450/140783647/140789247/140802135/140794359/140856469/140855857/140811642/140796892/140811312/140822776/140787430/140793470/140810137/140810260/140782755/140857813/140807666/140782510/140800983/140789708/140798161/	161	cg00048743/cg00281842/cg00589850/cg00808170/cg00888801/cg00943245/cg01000954/cg01169021/cg01224715/cg01311360/cg01729977/cg02022733/cg02056653/cg02161292/cg02249657/cg02267483/cg02331883/cg02430347/cg02452944/cg02707176/cg02715006/cg02780295/cg03400785/cg03415916/cg03456338/cg03486475/cg03640756/cg03892308/cg03898577/cg03898832/cg04080041/cg04117404/cg04453180/cg04503600/cg04726784/cg04845915/cg04964031/cg05149776/cg05197036/cg05670472/cg05870739/cg06428620/cg06572465/cg06757405/cg06776159/cg07017875/cg07179872/cg07231479/cg07445963/cg07448606/cg07489502/cg07524997/cg07539798/cg07608474/cg07613945/cg07620533/cg07730329/cg07802710/cg07816637/cg08155325/cg08200869/cg08292467/cg08395122/cg0885498	0.51864862	0.598171215	0.668942882	0.150294262	1.45E-10	3.21E-09

GENEBODY	PCDHGB6	5	140855562/140857995/140807787/140806126/140867386/140821244/140792918/140811520/140792558/140802831/140864578/140888468/140821687/140873765/140810404/140798095/140855547/140810109/140798758/140803130/140855463/140794962/140799811/140796240/140864593/140800495/140868130/140811253/140819300/140856330/140798188/140793307/140802804/140871156/140870164/140824037/140805732/140800586/140864276/140800929/140802135/140794359/140856469/140855857/140811642/140796892/140811312/140822776/140793470/140810137/140810260/140857813/140807666/140800983/140798161/140797509/140818522/140857474/140810920/140797280/140800629/140812464/140855582/140792684/140810161/	140	cg00281842/cg00589850/cg00808170/cg00888801/cg00943245/cg01000954/cg01169021/cg01224715/cg01311360/cg01729977/cg02022733/cg02056653/cg02161292/cg02249657/cg02267483/cg02331883/cg02430347/cg02452944/cg02707176/cg02715006/cg02780295/cg03400785/cg03456338/cg03486475/cg03640756/cg03892308/cg03898832/cg04080041/cg04117404/cg04453180/cg04503600/cg04726784/cg04845915/cg04964031/cg05149776/cg05197036/cg05670472/cg05870739/cg06428620/cg06572465/cg07179872/cg07231479/cg07445963/cg07448606/cg07489502/cg07524997/cg07539798/cg07608474/cg07620533/cg07730329/cg07802710/cg08155325/cg08200869/cg08395122/cg08935238/cg08938584/cg09484752/cg09581226/cg09639151/cg10435816/cg10679597/cg10805220/cg10858746/cg1091754	0.513457667	0.591000494	0.669084696	0.155627029	1.72E-10	3.63E-09

GENEBODY	PCDHGB7	5	140855562/140857995/140807787/140806126/140867386/140821244/140811520/140802831/140864578/140888468/140821687/140873765/140810404/140855547/140810109/140803130/140855463/140864593/140800495/140868130/140811253/140819300/140856330/140802804/140871156/140870164/140824037/140805732/140800586/140864276/140800929/140802135/140856469/140855857/140811642/140811312/140822776/140810137/140810260/140857813/140807666/140800983/140818522/140857474/140810920/140800629/140812464/140855582/140810161/140865433/140864834/140810123/140872334/140864474/140857192/140807490/140821425/140801286/140801354/140855482/140810726/140857941/140805681/140890718/140855469/	107	cg00281842/cg00589850/cg00808170/cg00888801/cg00943245/cg01000954/cg01224715/cg01729977/cg02022733/cg02056653/cg02161292/cg02249657/cg02267483/cg02430347/cg02452944/cg02715006/cg02780295/cg03640756/cg03892308/cg03898832/cg04080041/cg04117404/cg04453180/cg04845915/cg04964031/cg05149776/cg05197036/cg05670472/cg05870739/cg06428620/cg06572465/cg07179872/cg07445963/cg07448606/cg07489502/cg07539798/cg07608474/cg07730329/cg07802710/cg08155325/cg08200869/cg08395122/cg09484752/cg09581226/cg09639151/cg10679597/cg10805220/cg10858746/cg11647681/cg11830096/cg12145907/cg12304520/cg12487901/cg12648074/cg12877039/cg12972081/cg13023584/cg13598256/cg13718539/cg13993336/cg14253517/cg14672084/cg14681511/cg1500440	0.523604059	0.600720122	0.685587092	0.161983033	1.46E-10	3.22E-09

GENEBODY	PCDHGC3	5	140857995/140867386/140864578/140888468/140873765/140864593/140868130/140856330/140871156/140870164/140864276/140856469/140855857/140857813/140857474/140865433/140864834/140872334/140864474/140857192/140857941/140890718/140864701/140864549/140868422/140871464/140861242/140867724/140871590/140857889/140874572/140868686/140864733/140858046/140887302/140871385/140856971/140857647/140864020/140858107	40	cg00589850/cg00943245/cg02022733/cg02056653/cg02249657/cg03640756/cg03898832/cg04453180/cg04964031/cg05149776/cg06428620/cg07445963/cg07448606/cg08155325/cg09581226/cg11830096/cg12145907/cg12487901/cg12648074/cg12877039/cg14672084/cg15004405/cg15361590/cg15949044/cg15988320/cg16032518/cg16548916/cg17108383/cg17297238/cg18858331/cg21101631/cg22082176/cg23445461/cg24024424/cg24155123/cg24619378/cg24942747/cg25600472/cg26890354/cg27639030	0.502213763	0.585331792	0.687209331	0.184995567	4.46E-12	3.47E-10
GENEBODY	PCGEM1	2	193640765	1	cg21560241	0.60606139	0.506509228	0.433380836	-0.172680554	1.51E-07	9.01E-07
GENEBODY	PCGF1	2	74734085/74734609/74732471	3	cg03964696/cg22475904/cg24060542	0.39947488	0.316880961	0.240294223	-0.159180657	4.87E-10	7.84E-09
GENEBODY	PDCD1	2	242799312/242799562/242798512/242794981/242794854/242799489/242794941/242794928/242799553/242798478/242795082/242798443/242799460/242797846/242795282	15	cg01632474/cg03903296/cg04789125/cg06291111/cg07281781/cg09031938/cg09319815/cg10526431/cg10994870/cg15153118/cg16720890/cg20601192/cg21670983/cg24984297/cg25798782	0.753095851	0.644023415	0.53902606	-0.21406979	1.49E-14	1.99E-11
GENEBODY	PDS5B	13	33224942/33305966	2	cg18694313/cg23199907	0.63483244	0.56108645	0.464925668	-0.169906772	8.19E-09	7.50E-08

GENEBODY	PDX1	13	28497401/28497359/28495173/28495908/28495600/28496641/28494996/28498384/28496082/28496812/28498544/28498334/28496413/28498161/28497117/28495598/28498451/28495638/28498091	19	cg01622304/cg03860037/cg03881663/cg03907847/cg04109923/cg05395403/cg07860673/cg09975620/cg10782668/cg12238015/cg12815918/cg16302790/cg18064631/cg21900495/cg22268137/cg22578843/cg24601716/cg24983523/cg25299895	0.261283901	0.319019298	0.44655515	0.185271249	4.23E-09	4.38E-08
GENEBODY	PER4	7	9674902	1	cg00033909	0.313526365	0.208034044	0.153759197	-0.159767168	5.55E-12	3.89E-10
GENEBODY	PGAM2	7	44103276/44104584	2	cg02237368/cg21448875	0.51583931	0.463647508	0.358427059	-0.157412251	1.50E-11	7.17E-10
GENEBODY	PGLYRP3	1	153280070	1	cg12428378	0.789180925	0.6893515	0.5492556	-0.239925325	1.16E-06	5.47E-06
GENEBODY	PHOX2B	4	41748676/41750362/41749361/41749443/41747895/41749810/41748193/41748857	8	cg03742238/cg05323533/cg05722504/cg07907386/cg10192893/cg14755019/cg16318271/cg22300957	0.3750638	0.454136313	0.568124445	0.193060645	1.17E-08	1.01E-07
GENEBODY	PHYHD1	9	131684923	1	cg14299940	0.279666115	0.426487094	0.515844215	0.2361781	8.91E-06	3.31E-05
GENEBODY	PIF1	15	65116255/65115218/65113714/65116194	4	cg02018277/cg05869754/cg20714715/cg23845646	0.469714646	0.583327327	0.661337078	0.191622433	3.08E-09	3.40E-08
GENEBODY	PITX1	5	134369116/134367090/134366534/134366831/134369017/134366162/134364968/134365520/134367099/134368682/134364717/134366914/134365728/134366377/134367394	15	cg00089224/cg02100373/cg02213684/cg02495310/cg02948884/cg03347590/cg04101060/cg05171952/cg08373003/cg11512280/cg12673103/cg13715631/cg18606375/cg21614303/cg23064601	0.242394234	0.334946234	0.466915806	0.224521572	4.64E-09	4.71E-08
GENEBODY	PKD2L1	10	102050107	1	cg06298666	0.74549662	0.621716717	0.524351414	-0.221145206	1.37E-07	8.25E-07
GENEBODY	PKHD1L1	8	110497696/110470148/110520471/110456058	4	cg01995086/cg19153329/cg26971030/cg27519599	0.78696521	0.715398094	0.606944807	-0.180020403	3.18E-08	2.34E-07
GENEBODY	PLA2G2D	1	20441579/20445662	2	cg05048624/cg14321743	0.72265616	0.631564772	0.562409241	-0.160246919	0.000421383	0.001074034

GENEBODY	PLA2G4F	15	42448079/42448259/42447840/42447989/42448786/42448234/42448760/42434040	8	cg00150025/cg05397010/cg07720646/cg09927287/cg10917153/cg13065504/cg16920001/cg22490420	0.590224595	0.49206877	0.405089621	-0.185134974	8.79E-11	2.21E-09
GENEBODY	PLK5P	19	1526651/1526463/1524449/1525453/1525316/1528418/1524256/1525705/1526562/1529763	10	cg00851261/cg02351555/cg02789965/cg02872767/cg05308639/cg09760057/cg18300589/cg21964231/cg21991195/cg27277620	0.69735837	0.589673203	0.529644929	-0.167713441	3.69E-12	3.14E-10
GENEBODY	PLS1	3	142383107	1	cg05652551	0.60574264	0.470045383	0.354475555	-0.251267085	9.83E-11	2.38E-09
GENEBODY	PLUNC	20	31828749	1	cg06576557	0.45452721	0.317574856	0.199336355	-0.255190855	1.62E-17	1.41E-13
GENEBODY	PMCHL2	5	70671764	1	cg14277764	0.59191122	0.49622845	0.436196677	-0.155714543	7.32E-07	3.64E-06
GENEBODY	PNLIPRP3	10	118204535	1	cg12200270	0.51827738	0.371370589	0.233733395	-0.284543985	1.33E-13	5.53E-11
GENEBODY	POLR2L	11	840487/841335/841376/840407	4	cg01858337/cg03116740/cg11173246/cg13806461	0.727548019	0.644383175	0.559471714	-0.168076305	2.26E-08	1.76E-07
GENEBODY	POU4F2	4	147561669/147561203/147561775/147561339/147561900/147560770	6	cg00505045/cg06083330/cg16887264/cg19134568/cg19555331/cg25406138	0.443891576	0.549185858	0.675454296	0.23156272	8.70E-12	5.04E-10
GENEBODY	POU4F3	5	145719938/145719413/145718965	3	cg01819137/cg04701505/cg18482268	0.359385572	0.427875628	0.538171253	0.178785681	2.08E-07	1.20E-06
GENEBODY	PPFIA2	12	82123681/81763492/82121138/81694451	4	cg19888369/cg20484417/cg21665905/cg23926820	0.606702677	0.457746524	0.321623797	-0.28507888	1.48E-12	1.85E-10
GENEBODY	PPP1R14D	15	41118060	1	cg24886788	0.404137775	0.311828256	0.232683264	-0.171454511	2.73E-11	1.06E-09
GENEBODY	PPP1R1B	17	37789575/37784711/37784024/37783879/37784699/37785426/37787862/37784895/37784694	9	cg01894322/cg06068801/cg08411435/cg09762778/cg14697334/cg18575532/cg19526455/cg21320242/cg24520381	0.450607712	0.361362085	0.290201517	-0.160406195	1.54E-11	7.24E-10
GENEBODY	PRAME	22	22899164	1	cg17648213	0.639138815	0.528063828	0.4336709	-0.205467915	3.59E-11	1.24E-09
GENEBODY	PRAMEF20	1	13522252	1	cg11999044	0.514005275	0.420667506	0.3360326	-0.177972675	1.85E-11	8.18E-10
GENEBODY	PRKAG3	2	219695072	1	cg16792548	0.52770494	0.445790511	0.345442423	-0.182262517	5.75E-12	3.96E-10

GENEBODY	PRLHR	10	120354248/120354081/120354627	3	cg11705975/cg13654588/cg15748507	0.57032264	0.636302896	0.722855755	0.152533115	4.13E-11	1.33E-09
GENEBODY	PROL1	4	71270447	1	cg08712190	0.67536763	0.574553539	0.489292977	-0.186074653	8.03E-05	0.000239187
GENEBODY	PRR15L	17	46030312	1	cg18052778	0.501444175	0.407836833	0.336730273	-0.164713902	1.38E-10	3.09E-09
GENEBODY	PRSS45	3	46785957	1	cg01565013	0.621352525	0.520072494	0.394563973	-0.226788552	1.07E-07	6.67E-07
GENEBODY	PRSS8	16	31146682	1	cg04275947	0.51205637	0.441351806	0.360337309	-0.151719061	5.23E-09	5.21E-08
GENEBODY	PSG1	19	43383405	1	cg25477904	0.61481569	0.545823711	0.461511968	-0.153303722	1.23E-09	1.60E-08
GENEBODY	PSG3	19	43240516	1	cg24313473	0.44921919	0.344332911	0.270879386	-0.178339804	2.60E-11	1.03E-09
GENEBODY	PSG4	19	43709360	1	cg27257987	0.53977219	0.477842459	0.3736521	-0.16612009	0.003771619	0.007893927
GENEBODY	PSG8	19	43265778	1	cg10390696	0.6562031	0.5236886	0.437681218	-0.218521882	2.43E-10	4.64E-09
GENEBODY	PSG9	19	43769725	1	cg15401477	0.445327775	0.313800694	0.242209927	-0.203117848	1.35E-09	1.71E-08
GENEBODY	PSMG3	7	1607787/1607868/1607601/1607408/1607663	5	cg00375608/cg13042250/cg18958693/cg20151476/cg23903588	0.663483947	0.493261415	0.37804747	-0.285436477	1.68E-11	7.66E-10
GENEBODY	PTCH2	1	45288012/45308248/45308298/45297445/45304915/45292204	6	cg04145643/cg09941208/cg15329642/cg15684702/cg18646347/cg26297462	0.501116826	0.416925865	0.347265825	-0.153851001	4.88E-10	7.86E-09
GENEBODY	PTCHD3	10	27698606	1	cg07978901	0.696140455	0.605657967	0.504611064	-0.191529391	3.42E-07	1.86E-06
GENEBODY	PURG	8	30888801/30859349/30888925	3	cg02638909/cg03325522/cg18907656	0.764198185	0.657423417	0.591874309	-0.172323876	3.18E-09	3.48E-08
GENEBODY	PWRN1	15	24823713	1	cg15029747	0.51649206	0.401391294	0.297340064	-0.219151996	4.62E-10	7.49E-09
GENEBODY	PYY	17	42030842/42030479	2	cg01474257/cg25830696	0.304793746	0.368595931	0.465619647	0.160825901	0.000456745	0.001156691
GENEBODY	R3HDM1	2	136422981/136423459	2	cg01243312/cg05675362	0.5233833	0.409603739	0.343384568	-0.179998732	3.23E-09	3.52E-08
GENEBODY	R3HDML	20	42967118	1	cg08021653	0.60225943	0.513828372	0.43714995	-0.16510948	8.07E-10	1.15E-08
GENEBODY	RAB30	11	82708076	1	cg01250845	0.55726795	0.451751689	0.373305755	-0.183962195	6.29E-10	9.45E-09
GENEBODY	RARB	3	25614606/25493807/25616120/25483002/25635650/25634257/25482937/25616035/25623905/25513799/25472652/25583274	12	cg01697477/cg01794805/cg02746691/cg06705767/cg07405178/cg11151405/cg15011775/cg20981919/cg21646032/cg22231424/cg23518541/cg27574595	0.555555378	0.478943686	0.401504423	-0.154050955	6.69E-12	4.35E-10

GENEBODY	RASGRP3	2	33747506/33780366/33744326/33755653	4	cg16165651/cg18200741/cg26369614/cg27236007	0.64153703	0.5370365	0.430480862	-0.211056168	7.73E-10	1.11E-08
GENEBODY	RBM46	4	155720155	1	cg03174933	0.7254943	0.602266711	0.429098486	-0.296395814	1.77E-09	2.17E-08
GENEBODY	RDX	11	110108538/110108737/110120271/110122959	4	cg02243606/cg03141158/cg08451637/cg19336191	0.831112714	0.741504307	0.638808809	-0.192303905	2.89E-08	2.17E-07
GENEBODY	RFPL3S	22	32756957/32767005	2	cg04252889/cg22217678	0.648991785	0.538701578	0.412455345	-0.23653644	7.85E-10	1.13E-08
GENEBODY	RGS1	1	192548288	1	cg22360028	0.50273743	0.400195206	0.327457282	-0.175280148	4.32E-09	4.45E-08
GENEBODY	RIMS3	1	41094365	1	cg07920414	0.393852515	0.2902933	0.229636573	-0.164215942	1.02E-07	6.42E-07
GENEBODY	RNASE11	14	21052361	1	cg01620379	0.49821091	0.403106261	0.318064314	-0.180146596	5.63E-09	5.54E-08
GENEBODY	RNASE9	14	21024898	1	cg16701266	0.47897108	0.347028728	0.241015545	-0.237955535	1.80E-10	3.73E-09
GENEBODY	RNF43	17	56435455/56454528/56432612/56477476/56466866	5	cg04544475/cg04780984/cg11610460/cg18366480/cg21644778	0.669385706	0.55995674	0.483692099	-0.185693607	1.03E-14	1.49E-11
GENEBODY	RPL26	17	8282746	1	cg03734677	0.66792116	0.571355056	0.517033936	-0.150887224	1.73E-06	7.69E-06
GENEBODY	RSAD2	2	7029602	1	cg00186143	0.739571515	0.611205006	0.449552891	-0.290018624	3.40E-08	2.48E-07
GENEBODY	RSPH3	6	159420005/159416530	2	cg02895126/cg24249648	0.70916378	0.545194467	0.439121773	-0.270042007	3.04E-07	1.67E-06
GENEBODY	RSPO3	6	127475733/127441151/127440510/127484100/127441060/127440843/127441605/127440729/127441704/127442648/127441199/127441117/127441993/127513912	14	cg00042081/cg00752628/cg01043019/cg01694400/cg02690609/cg03778155/cg03983331/cg04974584/cg06437464/cg09526708/cg20172795/cg23232563/cg24470447/cg25671837	0.231508318	0.306698319	0.399169354	0.167661036	3.62E-06	1.48E-05
GENEBODY	RUFY4	2	218938006/218938889/218946721/218937982/218937635	5	cg01173650/cg01712671/cg06271623/cg20316538/cg22940954	0.543692453	0.445634296	0.334394413	-0.20929804	4.87E-11	1.50E-09
GENEBODY	S100A6	1	153507779/153507498	2	cg01910639/cg16291048	0.470612165	0.360896036	0.264822557	-0.205789608	9.48E-13	1.43E-10
GENEBODY	S100A7	1	153430296	1	cg00325910	0.427837735	0.324137928	0.236060509	-0.191777226	1.34E-13	5.53E-11
GENEBODY	SAA4	11	18254355	1	cg20357397	0.74639391	0.693612033	0.595278677	-0.151115233	3.16E-07	1.73E-06
GENEBODY	SAMD9	7	92731118	1	cg24669786	0.859169225	0.762183572	0.692919177	-0.166250048	1.16E-05	4.18E-05

GENEBODY	SAP30BP	17	73676600/73664606/73667384/73689641/73696509/73679983	6	cg02124060/cg03660451/cg04546917/cg06708315/cg18563987/cg20938708	0.704322185	0.605202156	0.540682807	-0.163639378	7.76E-13	1.26E-10
GENEBODY	SBSN	19	36017061	1	cg06924602	0.56526531	0.384811378	0.283984809	-0.281280501	1.22E-12	1.62E-10
GENEBODY	SCAMP5	15	75307476	1	cg16226627	0.62310084	0.504759028	0.391233559	-0.231867281	3.09E-10	5.55E-09
GENEBODY	SCG2	2	224462651	1	cg05986623	0.647967565	0.507988094	0.365699209	-0.282268356	3.37E-10	5.92E-09
GENEBODY	SCGB3A1	5	180017846/180017349/180017623/180017689/180018201/180018102	6	cg07317366/cg13004587/cg16585682/cg18652367/cg19897330/cg21919857	0.20540903	0.254657704	0.411046131	0.205637101	1.08E-05	3.90E-05
GENEBODY	SCN10A	3	38772426/38835179/38765009/38762483	4	cg00877619/cg01792749/cg11595270/cg19255722	0.600568547	0.492276489	0.360297566	-0.24027098	2.10E-11	8.89E-10
GENEBODY	SCN2A	2	166152734/166236760/166215248/166226560	4	cg00508771/cg15239914/cg18477797/cg20784295	0.708573813	0.632781296	0.537647944	-0.170925868	1.71E-09	2.10E-08
GENEBODY	SCN7A	2	167268207	1	cg19752565	0.643478245	0.573727228	0.472310959	-0.171167286	7.16E-07	3.57E-06
GENEBODY	SDR16C5	8	57228615	1	cg02141358	0.77667526	0.619863311	0.553771795	-0.222903465	7.03E-07	3.52E-06
GENEBODY	SECTM1	17	80280330/80282538	2	cg14728380/cg22156899	0.531056395	0.448040711	0.37119458	-0.159861815	9.15E-08	5.84E-07
GENEBODY	SELE	1	169696560	1	cg11132223	0.698568435	0.566948606	0.515794745	-0.18277369	7.40E-07	3.67E-06
GENEBODY	SELV	19	40007922	1	cg23201817	0.60075549	0.441228961	0.375011618	-0.225743872	3.20E-11	1.16E-09
GENEBODY	SEMA3D	7	84671333/84629717/84671582/84671308/84672041	5	cg00224028/cg00334933/cg02607810/cg10318039/cg26801812	0.621740985	0.502256144	0.43661905	-0.185121935	3.71E-10	6.35E-09
GENEBODY	SEMA3E	7	83087065/83081175/83146545/83057120/83270795/83097669/83240310/83055060/83043384	9	cg01932197/cg01983393/cg03542197/cg07449753/cg14411518/cg14519356/cg17616677/cg19907520/cg20587945	0.587243269	0.493482925	0.369837241	-0.217406028	1.50E-10	3.28E-09
GENEBODY	SERPINA11	14	94909938	1	cg23390762	0.64374094	0.58084855	0.456566041	-0.187174899	1.05E-06	5.00E-06
GENEBODY	SERPINB1	6	2840792	1	cg12586150	0.43386938	0.334061978	0.261824041	-0.172045339	2.66E-10	4.97E-09
GENEBODY	SERPINB10	18	61599595/61601962/61583699	3	cg06156844/cg22371956/cg23696618	0.759464128	0.630211744	0.52250787	-0.236956257	3.14E-10	5.63E-09
GENEBODY	SERPINB7	18	61455379	1	cg26877588	0.82685499	0.764884033	0.651473605	-0.175381385	9.57E-07	4.62E-06
GENEBODY	SERPINC1	1	173883567	1	cg02586268	0.497175275	0.391417694	0.328000791	-0.169174484	1.89E-11	8.32E-10
GENEBODY	SERPINE3	13	51917676	1	cg10474597	0.3605226	0.262788778	0.19934715	-0.16117545	3.62E-10	6.25E-09

GENEBODY	SERPING1	11	57365948	1	cg12783776	0.26612314	0.292039233	0.416576991	0.150453851	0.000559872	0.001390666
GENEBODY	SFRS3	6	36566732/36568488/36564708/36566714	4	cg00742040/cg11409081/cg16422343/cg26942169	0.698613524	0.606494385	0.542568906	-0.156044618	1.68E-08	1.38E-07
GENEBODY	SH2D5	1	21050526/21050467/21051448	3	cg11816566/cg13305458/cg24673385	0.741931422	0.62319477	0.528363774	-0.213567647	3.13E-11	1.15E-09
GENEBODY	SIGLEC11	19	50458233	1	cg11320438	0.550388875	0.486732694	0.379611105	-0.17077777	1.29E-08	1.10E-07
GENEBODY	SIGLEC12	19	52003566/52000825	2	cg02957799/cg04698472	0.440317075	0.337004078	0.268105745	-0.17221133	3.18E-13	7.92E-11
GENEBODY	SIGLEC7	19	51647083	1	cg14133643	0.729812865	0.662666811	0.562010818	-0.167802047	1.80E-05	6.19E-05
GENEBODY	SIGLEC8	19	51958448	1	cg22767164	0.55893205	0.487302389	0.381472795	-0.177459255	1.11E-07	6.87E-07
GENEBODY	SIGLEC9	19	51629205	1	cg17772789	0.69679793	0.624952517	0.531610955	-0.165186975	9.81E-08	6.18E-07
GENEBODY	SIRPB1	20	1578550	1	cg27365103	0.57808395	0.488472844	0.364299777	-0.213784173	4.42E-10	7.24E-09
GENEBODY	SIRPB2	20	1471884	1	cg00228984	0.552403565	0.466718389	0.3144807	-0.237922865	5.16E-09	5.16E-08
GENEBODY	SIRPD	20	1532543	1	cg03726785	0.7268426	0.642669639	0.434093927	-0.292748673	1.63E-10	3.50E-09
GENEBODY	SIX6	14	60977961/60977353/60977054/60977856/60976898/60977300	6	cg00157572/cg04666975/cg07862488/cg13019491/cg14511698/cg19981568	0.32836159	0.43885034	0.592218193	0.263856603	6.21E-10	9.37E-09
GENEBODY	SLA2	20	35258570	1	cg12504877	0.53039884	0.612679844	0.693819777	0.163420937	2.37E-09	2.76E-08
GENEBODY	SLC10A2	13	103702677	1	cg08450807	0.682875125	0.540926783	0.3800928	-0.302782325	2.81E-15	9.31E-12
GENEBODY	SLC12A1	15	48590100/48574462/48513336/48567756/48558703/48500240/48515109	7	cg10616694/cg13944105/cg14167964/cg14229516/cg14857792/cg14930674/cg23530596	0.634908433	0.593407993	0.479649495	-0.155258938	5.09E-08	3.50E-07
GENEBODY	SLC12A2	5	127423333/127420616/127420842/127420684	4	cg00308508/cg01159515/cg13676672/cg19767477	0.54067828	0.448886578	0.363778723	-0.176899557	2.20E-10	4.34E-09
GENEBODY	SLC16A9	10	61418311/61434211	2	cg08535112/cg24603972	0.62364834	0.514581322	0.413492886	-0.210155454	4.83E-09	4.87E-08
GENEBODY	SLC17A6	11	22362874/22365428/22364185/22364498/22363370/22364837/22370099/22362708/22364961/22364293/22363053	11	cg04583232/cg04954559/cg09150064/cg16079774/cg17298751/cg20809470/cg20998200/cg21518089/cg22371972/cg24151995/cg24454829	0.256200247	0.319089336	0.42193679	0.165736543	3.86E-08	2.76E-07
GENEBODY	SLC22A14	3	38358115	1	cg02646297	0.935948545	0.878315594	0.777767359	-0.158181186	3.05E-05	9.99E-05
GENEBODY	SLC22A25	11	62947565	1	cg21961694	0.58823213	0.397193411	0.254938509	-0.333293621	1.96E-13	6.68E-11

GENEBODY	SLC25A27	6	46621499/46623767	2	cg09906752/cg19101566	0.540622888	0.435873275	0.350258071	-0.190364817	2.45E-09	2.82E-08
GENEBODY	SLC25A34	1	16063471	1	cg21821674	0.510818975	0.408981939	0.334374264	-0.176444711	6.45E-11	1.79E-09
GENEBODY	SLC25A41	19	6429807/6427642	2	cg03722901/cg12096528	0.714492875	0.65418885	0.555333198	-0.159159677	9.09E-08	5.81E-07
GENEBODY	SLC30A10	1	220097971/220099770/220092812/220092843	4	cg07005353/cg07948194/cg16814688/cg27629095	0.592067168	0.456409069	0.359776375	-0.232290793	1.79E-12	2.11E-10
GENEBODY	SLC30A8	8	118158694	1	cg22342922	0.766687865	0.686521844	0.609364455	-0.15732341	1.16E-08	1.00E-07
GENEBODY	SLC32A1	20	37354889/37355188/37356844/37354117/37354790/37356466/37355965/37354254/37353907/37355280	10	cg01650904/cg02979819/cg05936441/cg06577241/cg10737118/cg12451099/cg13915754/cg16782807/cg26877715/cg27580318	0.261883662	0.352000615	0.470286055	0.208402393	1.15E-07	7.10E-07
GENEBODY	SLC35A3	1	100462710	1	cg22872634	0.670819465	0.573185989	0.498241723	-0.172577742	3.05E-09	3.37E-08
GENEBODY	SLC36A2	5	150701709/150706354/150715737	3	cg05672174/cg24131671/ch.5.2843133R	0.67218711	0.561881144	0.424160064	-0.248027046	3.60E-08	2.61E-07
GENEBODY	SLC38A11	2	165769913/165760725	2	cg01770810/cg16710042	0.50200045	0.393432094	0.339617795	-0.162382655	6.18E-08	4.14E-07
GENEBODY	SLC4A1	17	42336559	1	cg14381448	0.64852355	0.592750511	0.486012477	-0.162511073	7.82E-05	0.000233697
GENEBODY	SLC4A7	3	27472828/27450401	2	cg02344497/cg21518279	0.534249245	0.447411633	0.375017536	-0.159231709	7.61E-08	4.96E-07
GENEBODY	SLC5A12	11	26710632	1	cg13363904	0.5921416	0.44639285	0.378547118	-0.213594482	1.01E-08	8.95E-08
GENEBODY	SLC6A1	3	11059480/11059050/11059010/11058924/11058951/11062724	6	cg03047508/cg13418710/cg13889508/cg14673267/cg20241270/cg27082881	0.780468255	0.714899524	0.626942518	-0.153525737	3.22E-08	2.37E-07
GENEBODY	SLFN11	17	33679797	1	cg18124488	0.483705205	0.402775006	0.314314377	-0.169390828	7.79E-12	4.74E-10
GENEBODY	SMR3A	4	71230539	1	cg03300099	0.617099635	0.5218836	0.442332455	-0.17476718	6.70E-07	3.37E-06
GENEBODY	SNAP91	6	84417933/84415074/84418659/84418789/84417445/84418724/84289493/84418433	8	cg07335294/cg13886789/cg15950068/cg16334314/cg16457842/cg21688264/cg22048348/cg24190603	0.452216863	0.524178642	0.64912056	0.196903697	1.37E-10	3.07E-09
GENEBODY	SNN	16	11770053	1	cg04299808	0.69516685	0.557961833	0.506564973	-0.188601877	4.93E-12	3.70E-10
GENEBODY	SNORA8	11	93465580	1	cg23300851	0.43588728	0.345541061	0.283497809	-0.152389471	2.11E-10	4.21E-09
GENEBODY	SNORA80B	2	10586873	1	cg11668917	0.52827138	0.409054328	0.331031959	-0.197239421	1.57E-12	1.90E-10
GENEBODY	SNORD114-20	14	101447389	1	cg02351406	0.672360125	0.552280894	0.454482327	-0.217877798	6.87E-10	1.02E-08
GENEBODY	SNORD114-29	14	101456433	1	cg05899471	0.74318026	0.636617894	0.510825436	-0.232354824	1.13E-08	9.79E-08

GENEBODY	SNORD116-16	15	25327919	1	cg07897695	0.773205945	0.714791161	0.558580695	-0.21462525	7.82E-08	5.08E-07
GENEBODY	SNORD116-18	15	25330536	1	cg01871408	0.739671495	0.652255172	0.539758991	-0.199912504	6.93E-06	2.65E-05
GENEBODY	SNORD16	15	66795208	1	cg17352276	0.641547075	0.546695517	0.453846477	-0.187700598	1.95E-05	6.67E-05
GENEBODY	SNORD64	15	25230295	1	cg18488946	0.6237695	0.556032011	0.405183841	-0.218585659	4.65E-07	2.43E-06
GENEBODY	SOX17	8	55371829/55371013/55372392/55371369/55371392/55372111	6	cg02222728/cg08044097/cg12883523/cg15377283/cg16830930/cg24150172	0.485561365	0.577428062	0.690983374	0.205422009	3.59E-11	1.24E-09
GENEBODY	SPATA4	4	177116464/177107381	2	cg18237999/cg25209672	0.66166747	0.552719572	0.404006968	-0.257660502	8.62E-08	5.53E-07
GENEBODY	SPESP1	15	69223368/69224919/69223774/69223895	4	cg07775813/cg11147497/cg13757194/cg26820811	0.824374665	0.74509945	0.671473634	-0.152901031	8.88E-12	5.07E-10
GENEBODY	SPINK4	9	33240960/33245203	2	cg04103317/cg13823840	0.77713461	0.665101511	0.60163425	-0.17550036	9.76E-10	1.33E-08
GENEBODY	SPINK5L2	5	147552209	1	cg17496990	0.545994045	0.420752328	0.309993141	-0.236000904	1.77E-10	3.70E-09
GENEBODY	SPINK7	5	147693779/147692173	2	cg09574363/cg19404190	0.697437005	0.556692133	0.4302947	-0.267142305	2.78E-10	5.12E-09
GENEBODY	SPINT2	19	38756382/38757961/38755753	3	cg17344048/cg19562707/cg23090870	0.393932478	0.295429683	0.228395514	-0.165536964	4.20E-11	1.35E-09
GENEBODY	SPOCK3	4	167771886/168004573/168154775/168155219/167764264/167771769/168155222/168155124/167764172/168096506	10	cg05554543/cg05773958/cg09571713/cg09608712/cg16251342/cg18625258/cg20176349/cg21155380/cg21824455/cg22009329	0.453577405	0.512232437	0.632375524	0.178798119	3.51E-08	2.55E-07
GENEBODY	SPRR2A	1	153029005	1	cg14966325	0.761060365	0.6631467	0.55981105	-0.201249315	7.97E-05	0.000237636
GENEBODY	SPRY2	13	80911692/80911808/80911626	3	cg15374435/cg18607411/cg22369786	0.367941898	0.278514331	0.21106884	-0.156873057	1.28E-08	1.09E-07
GENEBODY	SQLE	8	126011954/126012434/126020660	3	cg00285394/cg14660676/cg23587955	0.515154165	0.438658322	0.347991936	-0.167162229	2.21E-06	9.55E-06
GENEBODY	SRGN	10	70849854/70863705/70863740/70863693/70863699	5	cg02851793/cg06041695/cg13645409/cg14898243/cg27141489	0.823076903	0.705401142	0.624587055	-0.198489848	6.91E-08	4.57E-07
GENEBODY	SSR2	1	155989796/155988060	2	cg21408061/cg26411367	0.522984405	0.406757811	0.327529268	-0.195455137	2.71E-10	5.03E-09
GENEBODY	ST3GAL1	8	134474257/134475410	2	cg12292213/cg14640789	0.76999565	0.535578333	0.411137514	-0.358858136	4.06E-10	6.81E-09

GENEBODY	ST8SIA3	18	55021277/55020324/55021542/55021669/55024674	5	cg00266920/cg03324087/cg03614193/cg06641153/cg21518865	0.36213622	0.460558244	0.551334091	0.189197871	1.89E-08	1.52E-07
GENEBODY	STAP2	19	4326524/4328759/4327106/4328745/4332056/4326975/4327350/4329581/4328818	9	cg00852565/cg06776703/cg10012059/cg14263858/cg15747839/cg16200879/cg19058865/cg24294041/cg25528916	0.478195827	0.578078872	0.644410325	0.166214498	3.10E-08	2.29E-07
GENEBODY	STAR	8	38006196	1	cg05580671	0.562135385	0.447851811	0.374502527	-0.187632858	1.37E-08	1.15E-07
GENEBODY	SUSD5	3	33259920/33245094/33259889	3	cg10906602/cg15469655/cg26298814	0.250510585	0.410026472	0.54748555	0.296974965	5.72E-06	2.23E-05
GENEBODY	SYNGR3	16	2041225/2043015/2040627/2041512/2040415/2041916/2042466/2041826/2042693/2040863/2042103/2040981	12	cg04514123/cg04519296/cg05239382/cg05915293/cg07519515/cg09229918/cg09286183/cg10022722/cg11220950/cg26486663/cg27028760/cg27616661	0.25555508	0.327057553	0.413159598	0.157604518	5.68E-09	5.57E-08
GENEBODY	SYT4	18	40853833	1	cg14462017	0.60654378	0.499964678	0.40323875	-0.20330503	6.54E-07	3.29E-06
GENEBODY	TAC3	12	57406900	1	cg10901383	0.44128207	0.342484589	0.251898105	-0.189383965	2.78E-11	1.07E-09
GENEBODY	TAF1A	1	222759637	1	cg05595429	0.72593794	0.652652933	0.555563195	-0.170374745	5.18E-07	2.68E-06
GENEBODY	TAF1D	11	93471889	1	cg03607573	0.3560487	0.256713278	0.205521087	-0.150527613	7.81E-10	1.12E-08
GENEBODY	TAS1R3	1	1268793/1267436/1267117/1268243/1267252/1267745/1267559/1268942/1267193/1269219/1268469/1268144/1267201/1269399	14	cg00002593/cg03402404/cg04910752/cg06395167/cg06802641/cg08621441/cg10065209/cg11808871/cg13246026/cg14911892/cg16174225/cg18386618/cg21396988/cg26814650	0.680429774	0.565768714	0.503014251	-0.177415523	1.17E-13	5.35E-11
GENEBODY	TBX18	6	85473195/85473238/85473344/85471973/85472307/85472949/85473171	7	cg03724990/cg06635392/cg06733419/cg07590529/cg09988118/cg22496809/cg26936429	0.312994691	0.401298208	0.513361989	0.200367299	4.03E-09	4.21E-08
GENEBODY	TCAP	17	37822045	1	cg01680823	0.55338361	0.468120661	0.395565745	-0.157817865	3.72E-16	2.15E-12

GENEBODY	TCTEX1D1	1	67220432	1	cg15026099	0.906453055	0.846221372	0.717773918	-0.188679137	2.77E-06	1.17E-05
GENEBODY	TCTN3	10	97450247/97453245	2	cg16489809/cg16514724	0.613010395	0.496197678	0.404123823	-0.208886572	5.04E-11	1.53E-09
GENEBODY	TDGF1	3	46621556/46621936/46622647/46621686	4	cg03679504/cg08058988/cg08962305/cg10031651	0.621145469	0.479744318	0.404091389	-0.21705408	2.26E-11	9.31E-10
GENEBODY	TEX15	8	30691170	1	cg26631437	0.622866005	0.453492078	0.33232755	-0.290538455	6.16E-12	4.14E-10
GENEBODY	TFAP2B	6	50810432/50787361/50808813/50788414/50791385/50810993/50787964/50808627/50808667/50787803/50808699/50810715/50803820/50803945/50794722/50792868/50810852/50804174/50803903/50803850/50794057/50791419/50810682/50787650/50804147/50791202	26	cg00908833/cg01610857/cg03159396/cg05928362/cg06117072/cg06564232/cg07103129/cg08178940/cg08857063/cg09247060/cg09363735/cg13223107/cg17083323/cg17343563/cg18442187/cg18886245/cg19620724/cg20432960/cg20894686/cg21317965/cg21650962/cg22248052/cg22282405/cg24366557/cg24641186/cg27260772	0.28033771	0.351626096	0.437550228	0.157212519	1.21E-07	7.39E-07
GENEBODY	TFAP2D	6	50691286/50690647/50682710/50692762/50683682/50682485/50683782/50692495/50682340/50683213/50690975/50692252/50692371/50691003/50685098/50690590/50697940/50692150/50683593/50695479/50692605/50685247	22	cg04573397/cg05139788/cg05255522/cg05650260/cg05798717/cg06804433/cg07625194/cg09086201/cg11038322/cg15412759/cg17191462/cg19384531/cg19509330/cg20180370/cg20229493/cg21760402/cg21871038/cg22235018/cg22286936/cg24094538/cg25049698/cg26680132	0.313749464	0.386309053	0.491498236	0.177748773	4.21E-09	4.36E-08
GENEBODY	TFB2M	1	246728677/246728727/246728663/246717609	4	cg12910806/cg14460735/cg17815049/cg24854957	0.59837027	0.452601656	0.39025005	-0.20812022	2.14E-10	4.25E-09

GENEBODY	TFEC	7	115608801/115608503/115586923/115609683/115583891	5	cg00927226/cg06493963/cg09281714/cg11479380/ch.7.2435427F	0.50113184	0.409877429	0.339399885	-0.161731955	5.17E-13	9.96E-11
GENEBODY	TFF3	21	43734404	1	cg18217706	0.47127797	0.385965178	0.308512286	-0.162765684	1.92E-10	3.91E-09
GENEBODY	TFPI2	7	93519473/93519220/93519401/93519855/93519621/93519892	6	cg12973591/cg18024479/cg19784477/cg20230721/cg22799321/cg23141855	0.200434836	0.348556788	0.571395334	0.370960498	1.50E-11	7.17E-10
GENEBODY	THPO	3	184090630	1	cg06522515	0.73609909	0.615043039	0.471935314	-0.264163776	1.17E-09	1.53E-08
GENEBODY	TIMP4	3	12199836/12197630	2	cg00314622/cg10110335	0.645457085	0.560144586	0.491313705	-0.15414338	9.16E-08	5.84E-07
GENEBODY	TLX1	10	102892528/102893925/102896475/102894227/102896546/102896640/102894441/102894342/102894639/102892131/102893980/102895708/102895043/102892787/102896102/102892391/102895133/102894120/102894148/102891879/102896376	21	cg00299972/cg00741609/cg01175020/cg04273871/cg04552206/cg06382559/cg07416656/cg07915434/cg08487063/cg10999413/cg12118269/cg14461582/cg14861089/cg15325875/cg20572816/cg20615879/cg23340017/cg24812837/cg25266629/cg25856018/cg27521476	0.281064455	0.315443011	0.463556207	0.182491751	4.27E-07	2.25E-06
GENEBODY	TLX2	2	74742362/74743009/74742593/74743243/74742786	5	cg05856387/cg07203423/cg07524679/cg16852704/cg19656282	0.325807415	0.400196506	0.525307159	0.199499744	5.56E-08	3.78E-07
GENEBODY	TM4SF4	3	149219783/149218938/149212391	3	cg08667024/cg12874219/cg19422253	0.647517357	0.594861565	0.478643503	-0.168873854	7.53E-10	1.09E-08
GENEBODY	TM7SF3	12	27156738/27164249/27166943	3	cg12659065/cg14606740/cg20673611	0.847389915	0.74886085	0.692225227	-0.155164688	1.04E-05	3.78E-05
GENEBODY	TM7SF4	8	105365603	1	cg08179564	0.51516027	0.449533489	0.351291141	-0.163869129	2.06E-08	1.63E-07
GENEBODY	TMCO5A	15	38233268	1	cg15286206	0.51195795	0.418156678	0.328500127	-0.183457823	1.62E-08	1.34E-07

GENEBODY	TMEM108	3	133048306/133002820/132977031/133103339/133098492/132990608/133094221/133112774/133097784/133020205/133097868	11	cg01058342/cg03187125/cg03593595/cg04411342/cg06030846/cg07594954/cg12668122/cg14668981/cg15425937/cg22264980/cg25488160	0.629623759	0.530265585	0.437245313	-0.192378446	2.18E-12	2.43E-10
GENEBODY	TMEM155	4	122682720	1	cg04476764	0.56074794	0.422988156	0.310239673	-0.250508267	6.44E-15	1.35E-11
GENEBODY	TMEM184B	22	38635773/38635620	2	cg02100011/cg09984479	0.585733335	0.496625039	0.415886823	-0.169846512	1.32E-05	4.68E-05
GENEBODY	TMEM19	12	72083379/72081013	2	cg12552402/cg13016773	0.604241375	0.511371964	0.43964327	-0.164598105	6.37E-05	0.000194153
GENEBODY	TMEM39A	3	119180915	1	cg08844169	0.85753726	0.742626456	0.589412418	-0.268124842	1.07E-08	9.34E-08
GENEBODY	TMIGD1	17	28648320	1	cg10781870	0.586152865	0.509687589	0.415103605	-0.17104926	3.66E-08	2.64E-07
GENEBODY	TMPRSS11B	4	69102304/69093675	2	cg03505857/cg09415518	0.52737457	0.435931333	0.355423232	-0.171951338	2.69E-08	2.03E-07
GENEBODY	TRAF5	1	211526705	1	cg13066703	0.33377811	0.192677654	0.147155244	-0.186622866	7.37E-10	1.08E-08
GENEBODY	TRAF6	11	36522143	1	cg01476222	0.45878218	0.365326367	0.279768232	-0.179013948	5.15E-07	2.66E-06
GENEBODY	TRAPPC2P1	19	57875238/57874988/57875014/57875295	4	cg03007201/cg10066151/cg12230795/cg21068576	0.108855118	0.166959063	0.285722302	0.176867184	4.08E-05	0.000129949
GENEBODY	TRAPPC5	19	7747220	1	cg24699296	0.639014495	0.751556344	0.833917505	0.19490301	8.67E-11	2.19E-09
GENEBODY	TRAT1	3	108564871	1	cg22211507	0.485813815	0.354774622	0.321024891	-0.164788924	6.21E-10	9.37E-09
GENEBODY	TRDN	6	123738929/123839720	2	cg10768734/cg19143211	0.607604075	0.551520067	0.448415727	-0.159188348	1.14E-05	4.10E-05
GENEBODY	TRIM16	17	15554603	1	cg02109507	0.43498378	0.357418006	0.283251186	-0.151732594	2.67E-10	4.99E-09
GENEBODY	TRIM49	11	89535662	1	cg23102197	0.60187731	0.490549672	0.376923514	-0.224953796	7.06E-11	1.89E-09
GENEBODY	TRIM55	8	67085823/67046248/67050040/67047851/67070006	5	cg04041976/cg21165595/cg25483191/cg26104239/cg26700215	0.570658019	0.470254794	0.407261335	-0.163396684	2.41E-10	4.63E-09
GENEBODY	TRIM77	11	89448414	1	cg15042801	0.58826783	0.453950444	0.300998227	-0.287269603	7.77E-13	1.26E-10
GENEBODY	TSGA10IP	11	65713893	1	cg21800989	0.693673805	0.633508528	0.47251895	-0.221154855	1.77E-07	1.04E-06
GENEBODY	TSPAN1	1	46649970/46649132	2	cg05371506/cg25369262	0.405575495	0.303571733	0.23783555	-0.167739945	3.91E-12	3.21E-10

GENEBODY	TSPAN4	11	850952/855971/859915/865673/856942/860134/864739/851048/862750/859532/858840/862820/865988/864819/859873/865575/857071/853761/857033/860157/865749/859670	22	cg00423364/cg03918313/cg05608543/cg08057037/cg09631059/cg13071185/cg13614606/cg13992402/cg14139652/cg14277925/cg15164881/cg15437031/cg16521773/cg17998346/cg18028767/cg20171011/cg20676047/cg22101701/cg22749642/cg23534766/cg23938637/cg24134897	0.557909795	0.459182072	0.383165935	-0.17474386	1.05E-12	1.51E-10
GENEBODY	TSPO	22	43559044/43556095	2	cg08391894/cg25672027	0.54712027	0.437453144	0.358051059	-0.189069211	2.41E-10	4.63E-09
GENEBODY	TST	22	37414044/37412173/37414747/37414442	4	cg01419582/cg01553653/cg08727202/cg17575915	0.740009713	0.568721289	0.472251452	-0.26775826	1.50E-10	3.28E-09
GENEBODY	TXNDC3	7	37923971/37899045/37924598	3	cg05088704/cg10576132/cg27518977	0.645847325	0.581371104	0.473627329	-0.172219996	8.84E-10	1.23E-08
GENEBODY	TYR	11	88953771	1	cg02618229	0.752075505	0.647699306	0.519959636	-0.232115869	2.95E-08	2.21E-07
GENEBODY	UGT2A1	4	70504750	1	cg02219607	0.854529735	0.804295483	0.638369209	-0.216160526	9.11E-06	3.37E-05
GENEBODY	ULBP1	6	150286838/150286157/150286646/150285333/150286508/150289488/150286212/150285634/150285815/150285475	10	cg00126498/cg01763051/cg01981252/cg04697775/cg11660826/cg12489846/cg12936335/cg14022137/cg16635480/cg25115993	0.244821174	0.314165703	0.414899613	0.170078439	7.84E-08	5.09E-07
GENEBODY	UNC13C	15	54638342/54582445/54897418	3	cg00517270/cg22825220/cg27553971	0.662577968	0.60218997	0.500774273	-0.161803696	2.17E-07	1.24E-06
GENEBODY	UNCX	7	1275483/1273835/1274752/1273200/1275630/1274166/1273394/1274142/1275258/1275252	10	cg01072217/cg02434035/cg03970319/cg06636427/cg09917979/cg11461298/cg14658067/cg16000637/cg23358612/cg23528705	0.379121901	0.430315663	0.555353414	0.176231513	7.70E-08	5.01E-07

GENEBODY	USP29	19	57641963/57642143/57641846	3	cg16594007/cg22338152/cg26587228	0.741866498	0.689605689	0.578578098	-0.1632884	1.82E-05	6.27E-05
GENEBODY	UTF1	10	135044856/135044789	2	cg00646347/cg11464895	0.462928785	0.512684333	0.630203091	0.167274306	2.73E-08	2.06E-07
GENEBODY	VCAM1	1	101188910/101186048	2	cg24593324/cg27544046	0.663223658	0.58657275	0.50240025	-0.160823408	1.00E-07	6.30E-07
GENEBODY	VMO1	17	4688683/4689284/4689165	3	cg06419850/cg09380695/cg26605335	0.471277785	0.5127324	0.656981086	0.185703301	4.27E-09	4.41E-08
GENEBODY	VNN2	6	133077819/133078831	2	cg19594626/cg25490477	0.618853865	0.5213141	0.443340423	-0.175513442	6.62E-08	4.39E-07
GENEBODY	VSTM2B	19	30020162/30019369/30018436/30018733/30020728/30020886/30021276/30024706/30019647/30017829/30019102/30019898/30022536/30019529	14	cg04756279/cg05984554/cg07955752/cg09064682/cg10743390/cg10836101/cg12776966/cg18375236/cg18716164/cg18777119/cg20695433/cg22143289/cg25690537/cg27058257	0.276356522	0.331611169	0.458490047	0.182133525	2.45E-08	1.88E-07
GENEBODY	VTRNA1-2	5	140098576	1	cg15697852	0.479840455	0.3511258	0.253039441	-0.226801014	1.45E-11	7.03E-10
GENEBODY	WDR49	3	167320581/167238645/167293056/167293354/167293814	5	cg03545548/cg04903759/cg14643415/cg17178922/cg18142906	0.622484424	0.535076164	0.455586806	-0.166897618	8.13E-13	1.29E-10
GENEBODY	WFDC10A	20	44259589	1	cg24337225	0.78751006	0.707709244	0.631765427	-0.155744633	5.82E-06	2.26E-05
GENEBODY	WIT1	11	32459954/32459428/32460980/32461240/32459626/32460375/32457878/32460656/32458769/32458656/32459057/32458714/32457727/32458966/32461212/32459760/32457386/32460799/32460587	19	cg03052301/cg04131898/cg05022105/cg06845853/cg07281879/cg09711746/cg12600018/cg12766736/cg14891410/cg16294566/cg16325378/cg19718882/cg22975913/cg24286165/cg25247290/cg25577842/cg26232818/cg27234067/cg27259320	0.283698073	0.356986224	0.47859278	0.194894707	5.24E-07	2.70E-06
GENEBODY	YPEL4	11	57414457/57414557/57414401	3	cg10366093/cg15363887/cg27368025	0.287362392	0.362775524	0.471969573	0.184607181	8.94E-06	3.32E-05
GENEBODY	ZFP28	19	57050834/57050620/57052834	3	cg04783231/cg05292954/cg21727463	0.310785263	0.434447739	0.55731685	0.246531588	1.67E-08	1.37E-07

GENEBODY	ZFP30	19	38129121	1	cg23355546	0.57848398	0.493870322	0.3960417	-0.18244228	3.25E-09	3.54E-08
GENEBODY	ZFP82	19	36891345	1	cg07673241	0.883843225	0.790912917	0.678376136	-0.205467089	3.67E-05	0.000118149
GENEBODY	ZG16	16	29790933	1	cg04125273	0.681148105	0.558352922	0.455213409	-0.225934696	1.92E-12	2.18E-10
GENEBODY	ZIC1	3	147129278/147129826/147129184/147131069/147128998/147131178/147130477/147131146/147129778/147131332/147130536/147129213/147129952	13	cg00842299/cg01376826/cg05095591/cg05716671/cg06306198/cg12204258/cg14750948/cg16209664/cg17482033/cg18297437/cg19029181/cg23448486/cg25590492	0.336063238	0.401763437	0.563720743	0.227657505	3.00E-08	2.23E-07

GENEBODY	ZIC4	3	147114406/147111280/147110322/147106010/147116807/147107349/147110595/147112438/147113092/147112081/147116135/147115506/147111135/147111660/147106635/147106561/147112605/147113700/147115930/147122664/147105190/147110378/147115043/147114986/147109426/147110367/147106489/147107109/147110499/147113918/147105899/147110667/147120248/147108916/147121229/147106890/147108843/147111308/147109490/147113726/147112541/147114352/147109629/147109784/147123123/147112316/147106208/147110295/147121892/147107900/147112143/147116420/147112096/147122315/147108512/147110229/147111120	57	cg00028935/cg00154357/cg00235367/cg00334063/cg00896370/cg01137401/cg01581018/cg02126051/cg02390329/cg02653559/cg03388789/cg03631131/cg03881775/cg03900143/cg04079301/cg04556126/cg04557018/cg05855917/cg06070263/cg06166523/cg07406191/cg07850418/cg08013557/cg08091192/cg08393041/cg08812189/cg08889797/cg11139044/cg12388007/cg12892506/cg12976081/cg13340636/cg13897134/cg15105326/cg15287443/cg16240162/cg16768018/cg17003736/cg17569743/cg18082337/cg18292434/cg18731327/cg18930354/cg20939084/cg21639713/cg22203776/cg22614239/cg22796509/cg23957311/cg23980740/cg25007283/cg25449440/cg26014036/cg26224785/cg26790247/cg27416372/cg27606499	0.358865792	0.411439705	0.510092814	0.151227022	7.56E-09	6.99E-08
GENEBODY	ZIK1	19	58096559/58096512/58099109	3	cg06412288/cg13617753/cg22836912	0.752243033	0.637577424	0.520320808	-0.231922226	2.47E-12	2.58E-10
GENEBODY	ZKSCAN2	16	25266553	1	cg09588492	0.61983746	0.523827922	0.465177723	-0.154659737	5.51E-09	5.45E-08

GENEBODY	ZMAT4	8	40432238/40602363/40618425/40552037/40543522/40627109/40478698/40613848/40616749/40392927/40625224/40443548	12	cg00935717/cg02458516/cg04364695/cg06454283/cg10260711/cg11491870/cg14822721/cg20071227/cg20630363/cg21093043/cg24882673/cg24887387	0.657354035	0.59878775	0.501365575	-0.155988461	6.73E-11	1.84E-09
GENEBODY	ZNF114	19	48785784	1	cg00843019	0.790551345	0.705236406	0.620201568	-0.170349777	5.28E-05	0.000163798
GENEBODY	ZNF154	19	58220080/58220295	2	cg03142586/cg11294513	0.483641065	0.614256869	0.703068255	0.21942719	9.86E-10	1.34E-08
GENEBODY	ZNF254	19	24270468	1	cg01000657	0.070355748	0.104261602	0.280390649	0.210034901	0.000911951	0.002165104
GENEBODY	ZNF257	19	22235850/22235454/22235551	3	cg06394874/cg06852575/cg11619707	0.382096038	0.498389683	0.63197652	0.249880483	6.72E-12	4.35E-10
GENEBODY	ZNF304	19	57863210/57867032	2	cg04546041/cg24788771	0.8722886	0.796608822	0.650300227	-0.221988373	6.61E-06	2.54E-05
GENEBODY	ZNF334	20	45138738	1	cg23845310	0.733046855	0.652532778	0.5730647	-0.159982155	1.01E-05	3.68E-05
GENEBODY	ZNF43	19	22014817/22018605/22018746/22018791/22018538	5	cg00749323/cg02612618/cg14252602/cg24324871/cg24886770	0.362986525	0.399688613	0.51600423	0.153017705	4.77E-05	0.00014966
GENEBODY	ZNF454	5	178371587/178381935	2	cg05461386/cg18358845	0.720645005	0.645089992	0.495048748	-0.225596257	2.10E-08	1.66E-07
GENEBODY	ZNF483	9	114290738	1	cg13813533	0.76082225	0.634520494	0.525915109	-0.234907141	9.79E-10	1.33E-08
GENEBODY	ZNF507	19	32865061	1	cg04771109	0.5156129	0.4185368	0.331010732	-0.184602168	4.06E-08	2.88E-07
GENEBODY	ZNF518B	4	10447440	1	cg11578055	0.86757463	0.808147272	0.613450536	-0.254124094	6.18E-05	0.000188834
GENEBODY	ZNF529	19	37095681/37061383/37096010/37046582/37095466/37096148/37095757	7	cg09430118/cg10172250/cg19406736/cg21486913/cg24871714/cg25397945/cg27658967	0.443580931	0.534415318	0.63017785	0.186596919	4.42E-09	4.52E-08
GENEBODY	ZNF530	19	58111705/58115732/58111647/58111767/58111769	5	cg03734784/cg09767616/cg12476763/cg13203804/cg24192482	0.230644361	0.317485943	0.415001635	0.184357274	4.25E-05	0.000134967
GENEBODY	ZNF542	19	56879662/56882355/56879933/56879554/56880388/56879559/56879994/56879571/56879613/56879645	10	cg03146949/cg07928808/cg08697092/cg15708153/cg18471660/cg18787401/cg20004406/cg26309134/cg27062795/cg27477373	0.351600762	0.463379549	0.613233508	0.261632746	5.73E-10	8.84E-09

GENEBODY	ZNF549	19	58039253/58043131/58039073	3	cg06616879/cg14592770/cg17684296	0.380423141	0.434851281	0.541282609	0.160859469	1.18E-07	7.28E-07
GENEBODY	ZNF563	19	12440923/12444273	2	cg13402083/cg25682920	0.752709545	0.572152017	0.47094505	-0.281764495	4.55E-13	9.40E-11
GENEBODY	ZNF586	19	58281450/58281797	2	cg14038484/cg16666206	0.474815677	0.513182135	0.629203648	0.154387971	0.001137177	0.002654076
GENEBODY	ZNF626	19	20844070	1	cg16510409	0.334466715	0.437191611	0.527248464	0.192781749	7.07E-10	1.04E-08
GENEBODY	ZNF671	19	58238600/58235167/58238435	3	cg03215105/cg07990873/cg26705688	0.490944233	0.549336074	0.642837827	0.151893595	5.36E-07	2.76E-06
GENEBODY	ZNF705A	12	8329330	1	cg17479811	0.692960605	0.636332856	0.535742814	-0.157217791	3.93E-08	2.81E-07
GENEBODY	ZNF732	4	265139	1	cg14446430	0.655922295	0.562328983	0.470016641	-0.185905654	4.66E-09	4.72E-08
GENEBODY	ZNF772	19	57988497	1	cg01017773	0.222995235	0.316335106	0.408892923	0.185897688	4.37E-05	0.000138392
GENEBODY	ZNF788	19	12203315/12203644/12203198/12203349/12207214	5	cg01172903/cg01404753/cg06384763/cg11767984/cg21289365	0.136134971	0.255735171	0.50351314	0.367378168	1.34E-07	8.10E-07
GENEBODY	ZNF793	19	38022450	1	cg16254093	0.85850379	0.79038605	0.676168068	-0.182335722	7.16E-06	2.72E-05
GENEBODY	ZNF804B	7	88425077/88583884/88423665/88425085/88594866/88858347/88425139/88425122/88425758/88424985/88425148/88426025	12	cg00729133/cg03108373/cg03183800/cg05079191/cg06820822/cg09795550/cg13354414/cg15325154/cg16781484/cg19682786/cg22946147/cg24975898	0.765890464	0.685058978	0.584061893	-0.181828571	3.66E-11	1.25E-09
GENEBODY	ZNF814	19	58400095/58399865/58397811/58399967	4	cg02280912/cg22299467/cg22481158/cg22510337	0.485496321	0.560678227	0.663601421	0.1781051	3.88E-07	2.07E-06
GENEBODY	ZNF85	19	21106370	1	cg11416076	0.152357116	0.290752656	0.445527541	0.293170425	1.32E-06	6.09E-06
GENEBODY	ZNF876P	4	207357/209682/206874/206877/206562/206442	6	cg07043305/cg10113414/cg11836119/cg12547166/cg17201760/cg20296343	0.476610028	0.566519825	0.665695598	0.18908557	1.81E-11	8.03E-10
GENEBODY	ZP4	1	238051451/238049504	2	cg17514199/cg27168737	0.66278006	0.595976408	0.512301293	-0.150478767	6.14E-08	4.12E-07
GENEBODY	ZPBP2	17	38028394	1	cg11212589	0.700927985	0.582755744	0.504632686	-0.196295299	1.10E-07	6.85E-07
GENEBODY	ZPLD1	3	102154087/102156072/102187934	3	cg00423655/cg04357717/cg08483570	0.694401172	0.580483544	0.473339185	-0.221061987	4.39E-08	3.07E-07
GENEBODY	ZSCAN23	6	28403086	1	cg20803211	0.499996225	0.430526039	0.297437109	-0.202559116	9.79E-09	8.74E-08
UTR3	A2ML1	12	9028945	1	cg11756734	0.638961415	0.601603228	0.486107759	-0.152853656	3.03E-07	2.28E-06

UTR3	ABCA12	2	215796436	1	cg04530015	0.57522704	0.427715561	0.311875464	-0.263351576	2.24E-13	5.46E-11
UTR3	ABCA4	1	94458645	1	cg18377941	0.687904555	0.576694828	0.489844095	-0.19806046	4.15E-05	0.000178105
UTR3	ABCG4	11	119032584	1	cg06622994	0.586185735	0.50533435	0.390541432	-0.195644303	3.15E-09	4.46E-08
UTR3	ABI3	17	47300216	1	cg14009013	0.761204525	0.676664889	0.581799882	-0.179404643	9.32E-07	6.05E-06
UTR3	ABLIM3	5	148638033	1	cg02306139	0.614524665	0.5010142	0.329975855	-0.28454881	8.83E-12	5.41E-10
UTR3	ACAN	15	89418456	1	cg08631783	0.67395309	0.566345628	0.483523068	-0.190430022	3.50E-07	2.58E-06
UTR3	ACER3	11	76733236/76731589	2	cg05476094/cg27121309	0.724103983	0.618855539	0.518345402	-0.20575858	3.58E-10	7.42E-09
UTR3	ADAM29	4	175899203	1	cg09975995	0.63443501	0.512790856	0.390626277	-0.243808733	4.09E-09	5.57E-08
UTR3	ADAMTS18	16	77317555	1	cg02084561	0.76230827	0.686389017	0.596039695	-0.166268575	1.10E-06	7.03E-06
UTR3	ADAMTS9	3	64504415	1	cg02810826	0.781008645	0.633513989	0.503249659	-0.277758986	6.61E-09	8.40E-08
UTR3	ADAMTSL3	15	84706833	1	cg08115297	0.672769115	0.53539575	0.326736905	-0.34603221	8.91E-13	1.17E-10
UTR3	ADD2	2	70903803/70889324	2	cg13284981/cg15016734	0.7276324	0.6447923	0.568877045	-0.158755355	6.99E-07	4.70E-06
UTR3	ADM2	22	50923890/50924745/50923963	3	cg13332571/cg19621271/cg23513727	0.744776612	0.648096317	0.562427082	-0.18234953	2.48E-10	5.48E-09
UTR3	ADORA2A	22	24838292	1	cg12727256	0.72455102	0.651873172	0.567412368	-0.157138652	2.52E-06	1.48E-05
UTR3	ADRA1B	5	159399506/159399681	2	cg03825010/cg14614811	0.166410423	0.231322961	0.346068013	0.179657589	0.000194931	0.000706806
UTR3	ADRB2	5	148208041	1	cg08370787	0.528259235	0.410485839	0.342388755	-0.18587048	1.06E-10	2.86E-09
UTR3	AFF4	5	132213462	1	cg06703856	0.51727074	0.439348106	0.348019764	-0.169250976	6.49E-10	1.18E-08
UTR3	AGBL3	7	134820312	1	cg18461626	0.82932953	0.727430106	0.644508309	-0.184821221	4.20E-05	0.000179852
UTR3	AGBL4	1	48998823	1	cg02057755	0.380710425	0.277773267	0.190820573	-0.189889852	3.76E-14	1.42E-11
UTR3	AGXT2	5	34998412	1	cg15664442	0.80849205	0.753906983	0.654287523	-0.154204527	3.02E-06	1.73E-05
UTR3	AGXT2L1	4	109663613	1	cg19486412	0.82405332	0.743231594	0.612550055	-0.211503265	1.03E-06	6.59E-06
UTR3	AHRR	5	435040/435388/435267/435613/434998/436816	6	cg14448919/cg14807090/cg16336872/cg21972741/cg22951524/cg26954197	0.623819285	0.550968883	0.471547414	-0.152271871	3.62E-06	2.02E-05
UTR3	AHSA1	14	77935699	1	cg14520941	0.613637915	0.511734744	0.428135505	-0.18550241	2.40E-11	1.05E-09
UTR3	AIFM3	22	21335632	1	cg25976804	0.62246171	0.506076589	0.445157382	-0.177304328	8.30E-08	7.39E-07
UTR3	AIPL1	17	6327601	1	cg17609294	0.763865555	0.646603222	0.514447655	-0.2494179	6.71E-08	6.13E-07
UTR3	AKAP11	13	42894507	1	cg03439687	0.612276475	0.5021941	0.414127118	-0.198149357	8.42E-13	1.13E-10
UTR3	AKAP5	14	64936525	1	cg05919314	0.750572145	0.637316439	0.555641536	-0.194930609	9.99E-08	8.66E-07
UTR3	AKAP6	14	33296030	1	cg15924738	0.59473448	0.531065528	0.413856027	-0.180878453	1.95E-09	2.98E-08
UTR3	AKAP7	6	131602870	1	cg00665374	0.51366372	0.609084361	0.693270741	0.179607021	1.18E-09	1.94E-08
UTR3	ALAD	9	116150138	1	cg14193097	0.5411247	0.424798272	0.340056877	-0.201067823	8.43E-11	2.47E-09
UTR3	ALB	4	74286665	1	cg08368094	0.778720585	0.666397389	0.518812423	-0.259908162	1.47E-08	1.66E-07

UTR3	ALDH1L2	12	105414154	1	cg14255981	0.672790595	0.528602033	0.444274377	-0.228516218	3.59E-09	4.97E-08
UTR3	ALG9	11	111653746	1	cg11259591	0.72719932	0.634226522	0.505274709	-0.221924611	1.07E-10	2.88E-09
UTR3	ALOXE3	17	7999745	1	cg05365385	0.55536219	0.453467494	0.403523736	-0.151838454	2.22E-09	3.31E-08
UTR3	ALPPL2	2	233274653/233274723	2	cg16700265/cg24331934	0.5487432	0.464692861	0.386979732	-0.161763468	4.05E-11	1.55E-09
UTR3	AMH	19	2252069/2252029	2	cg00299047/cg12534008	0.44810855	0.499917708	0.611727516	0.163618966	3.58E-05	0.00015613
UTR3	AMPH	7	38423649	1	cg15777769	0.6787354	0.559163772	0.423217873	-0.255517527	4.80E-11	1.76E-09
UTR3	ANK3	10	61802440	1	cg15627145	0.621806035	0.482421689	0.3703146	-0.251491435	4.16E-12	3.37E-10
UTR3	ANKMY2	7	16639517	1	cg10276016	0.50242528	0.410169906	0.298834709	-0.203590571	1.18E-11	6.50E-10
UTR3	ANKRD13A	12	110476821	1	cg13509421	0.448228205	0.359702839	0.274879641	-0.173348564	5.08E-10	9.59E-09
UTR3	ANKRD33B	5	10655518/10655569/10652296/10650351/10653304/10655725/10651897	7	cg01089425/cg04645676/cg05162281/cg06926314/cg10581071/cg11707556/cg23325245	0.750307347	0.64393118	0.543394964	-0.206912383	9.92E-10	1.68E-08
UTR3	ANKRD35	1	145568368	1	cg26537883	0.760453825	0.619142517	0.493559041	-0.266894784	4.32E-09	5.83E-08
UTR3	ANKRD46	8	101534515	1	cg11163330	0.831350875	0.712009756	0.643841623	-0.187509252	9.37E-11	2.65E-09
UTR3	ANKRD55	5	55395877	1	cg17577695	0.681304495	0.644753494	0.517329018	-0.163975477	8.39E-05	0.00033309
UTR3	ANO5	11	22301379	1	cg06703469	0.764614435	0.692608706	0.530419827	-0.234194608	4.98E-07	3.56E-06
UTR3	ANO7	2	242164067/242135579	2	cg01410615/cg17353895	0.531709145	0.407503228	0.340160364	-0.191548781	2.08E-11	9.77E-10
UTR3	APBB1	11	6416634	1	cg05668613	0.584274745	0.504451472	0.431933973	-0.152340772	2.75E-10	5.98E-09
UTR3	APBB1IP	10	26856664/26856454	2	cg18014277/cg19797516	0.634251078	0.567977744	0.447287009	-0.186964068	2.42E-07	1.88E-06
UTR3	APCDD1	18	10488229	1	cg18208602	0.757764415	0.591042433	0.443936245	-0.31382817	1.69E-11	8.39E-10
UTR3	APCS	1	159558512	1	cg21291134	0.689444045	0.605787644	0.427001636	-0.262442409	2.26E-07	1.77E-06
UTR3	APOA5	11	116660514	1	cg08598780	0.691337915	0.609925772	0.531392209	-0.159945706	7.65E-08	6.88E-07
UTR3	APOBEC3F	22	39440294/39449298	2	cg09582545/cg19333614	0.921456525	0.820357289	0.669946377	-0.251510148	2.40E-07	1.87E-06
UTR3	ARC	8	143692935/143694305/143694295/143694010	4	cg00487506/cg05415840/cg13172906/cg24450303	0.713940839	0.621943943	0.521987426	-0.191953413	3.29E-09	4.62E-08
UTR3	ARHGAP26	5	142602110	1	cg05185926	0.51960638	0.61873905	0.689532395	0.169926015	1.85E-09	2.85E-08
UTR3	ARHGDIA	17	79825681/79826316	2	cg10236857/cg25141766	0.614159513	0.506641975	0.424316248	-0.189843265	8.74E-12	5.41E-10
UTR3	ARHGEF15	17	8225715	1	cg03933062	0.645706645	0.546252844	0.415816968	-0.229889677	7.01E-10	1.25E-08
UTR3	ARL14	3	160395719	1	cg20725880	0.485930335	0.369562406	0.273610573	-0.212319762	6.70E-12	4.58E-10
UTR3	ARL4A	7	12729527/12728890/12729367	3	cg07302938/cg12785342/cg24092907	0.697944073	0.556255906	0.411261102	-0.28668297	4.39E-10	8.63E-09

UTR3	ARPP-21	3	35835606/35726493	2	cg16468417/cg21852346	0.6968133	0.644858322	0.534254105	-0.162559195	0.000533395	0.001752289
UTR3	ARRDC5	19	4890970	1	cg10130497	0.649060535	0.492724406	0.345601477	-0.303459058	9.55E-13	1.20E-10
UTR3	ARSJ	4	114823338	1	cg26728709	0.41836045	0.277633728	0.216224082	-0.202136368	2.35E-13	5.50E-11
UTR3	ASAM	11	122943141	1	cg18247124	0.593784845	0.472878706	0.431184855	-0.16259999	2.34E-07	1.83E-06
UTR3	ASB15	7	123277601	1	cg02621087	0.693562545	0.533291556	0.408837964	-0.284724581	2.32E-12	2.28E-10
UTR3	ASCL4	12	108169569/108169928/108170183	3	cg01295392/cg01985833/cg23699926	0.60857134	0.695006017	0.760260757	0.151689417	8.47E-12	5.34E-10
UTR3	ASPA	17	3402504	1	cg21185242	0.72593518	0.645842633	0.554620532	-0.171314648	2.54E-05	0.000115753
UTR3	ASTN1	1	176831160	1	cg08104310	0.499367355	0.375349833	0.28075905	-0.218608305	1.92E-10	4.58E-09
UTR3	ATG9B	7	150709877/150711138/150710730/150710447/150711449/150709463	6	cg00448659/cg02027878/cg09519327/cg15183092/cg25025646/cg25136644	0.47813196	0.595272763	0.673006548	0.194874588	9.42E-10	1.60E-08
UTR3	ATOH7	10	69990588	1	cg00001874	0.45284816	0.346283617	0.293820759	-0.159027401	2.70E-08	2.80E-07
UTR3	AVPR1A	12	63540400	1	cg10931900	0.73479733	0.681570672	0.535394582	-0.199402748	1.72E-05	8.06E-05
UTR3	BACH2	6	90639197	1	cg22758471	0.75454658	0.6842184	0.529889818	-0.224656762	8.48E-08	7.53E-07
UTR3	BAX	19	49465047	1	cg22237988	0.95223169	0.859445439	0.795971445	-0.156260245	5.60E-08	5.26E-07
UTR3	BBS12	4	123665848	1	cg12927785	0.70605296	0.587795556	0.477194623	-0.228858337	1.15E-10	3.04E-09
UTR3	BCL2L1	20	30252580	1	cg25291404	0.455911625	0.3690684	0.290757568	-0.165154057	4.65E-10	9.01E-09
UTR3	BFAR	16	14762377	1	cg06081917	0.7584357	0.619699583	0.574493441	-0.183942259	3.59E-09	4.97E-08
UTR3	BHLHE23	20	61637360	1	cg06228507	0.44108202	0.5173817	0.628947414	0.187865394	5.42E-08	5.12E-07
UTR3	BLOC1S1	12	56113434	1	cg08222053	0.67639435	0.585559583	0.502288741	-0.174105609	2.13E-09	3.22E-08
UTR3	BNC1	15	83926030	1	cg08633249	0.424365525	0.323622278	0.229137673	-0.195227852	2.52E-12	2.36E-10
UTR3	BNC2	9	16413736	1	cg14061067	0.549558195	0.451440711	0.359985318	-0.189572877	8.83E-11	2.52E-09
UTR3	BNIPL	1	151019560/151019727/151019274	3	cg11852073/cg13072446/cg23822289	0.853799177	0.731346567	0.640995742	-0.212803434	2.62E-07	2.02E-06
UTR3	BPIL2	22	32810284	1	cg09696535	0.591864745	0.450350756	0.388786845	-0.2030779	2.78E-07	2.13E-06
UTR3	BRD2	6	32948526	1	cg02590376	0.81883865	0.721197156	0.6679512	-0.15088745	1.28E-07	1.07E-06
UTR3	BTBD9	6	38141572/38141879/38141763	3	cg00382470/cg01504030/cg14151158	0.713871383	0.591239753	0.548570014	-0.165301369	9.39E-11	2.65E-09
UTR3	BTN3A2	6	26375990	1	cg21432745	0.826145275	0.694975528	0.6735399	-0.152605375	7.11E-10	1.27E-08
UTR3	C10orf128	10	50363896	1	cg25921502	0.56676901	0.409657111	0.286231195	-0.280537815	7.01E-15	7.46E-12
UTR3	C10orf53	10	50902733/50916667	2	cg13998871/cg16657724	0.652929945	0.559369928	0.432735875	-0.22019407	2.33E-10	5.25E-09
UTR3	C10orf71	10	50535073	1	cg06322518	0.60387193	0.481421506	0.4180215	-0.18585043	5.18E-10	9.72E-09
UTR3	C10orf72	10	50311040/50224628	2	cg00215019/cg11115673	0.653541793	0.546624864	0.420936489	-0.232605304	3.08E-10	6.55E-09

UTR3	C10orf96	10	118138962	1	cg26713755	0.736713595	0.656486389	0.561362468	-0.175351127	0.000331485	0.001147716
UTR3	C11orf53	11	111156908	1	cg25129781	0.535471085	0.388971633	0.317235595	-0.21823549	6.14E-11	2.01E-09
UTR3	C11orf9	11	61555930	1	cg14981432	0.52200928	0.424617683	0.367354064	-0.154655216	1.26E-05	6.15E-05
UTR3	C11orf93	11	111179338	1	cg05817996	0.670810695	0.576874311	0.520198595	-0.1506121	1.52E-09	2.41E-08
UTR3	C12orf53	12	6803244	1	cg23727813	0.553147925	0.432452039	0.358179905	-0.19496802	1.06E-12	1.30E-10
UTR3	C12orf77	12	25147218	1	cg21156602	0.85398101	0.789365994	0.701775209	-0.152205801	3.37E-05	0.000148011
UTR3	C13orf33	13	31499033	1	cg22591875	0.54903895	0.472093506	0.369233745	-0.179805205	3.62E-09	5.01E-08
UTR3	C13orf36	13	37271030	1	cg03603888	0.356577185	0.29698475	0.204972264	-0.151604921	2.77E-12	2.48E-10
UTR3	C14orf49	14	95884023	1	cg10506461	0.81608218	0.744470444	0.66099855	-0.15508363	6.77E-06	3.53E-05
UTR3	C15orf2	15	24924925	1	cg20809224	0.5228759	0.427715961	0.314691786	-0.208184114	3.80E-10	7.81E-09
UTR3	C15orf24	15	34376369	1	cg21292201	0.45959211	0.361517728	0.290818582	-0.168773528	2.30E-09	3.42E-08
UTR3	C15orf41	15	37100941	1	cg26451373	0.673976035	0.626238167	0.467211527	-0.206764508	1.15E-06	7.30E-06
UTR3	C15orf57	15	40846077	1	cg20602300	0.54771958	0.68910715	0.729808577	0.182088997	1.58E-08	1.76E-07
UTR3	C16orf62	16	19712046	1	cg03301165	0.572527425	0.463875044	0.371225995	-0.20130143	2.13E-10	4.92E-09
UTR3	C17orf102	17	32904255/32903717	2	cg12309348/cg26090020	0.678335128	0.564225969	0.39224943	-0.286085698	3.12E-14	1.22E-11
UTR3	C17orf106	17	73996423/73996656	2	cg14666354/cg25711239	0.66384261	0.526577311	0.457893418	-0.205949192	1.68E-08	1.86E-07
UTR3	C17orf81	17	7163136	1	cg21762727	0.596807885	0.450805822	0.363989409	-0.232818476	1.05E-11	5.93E-10
UTR3	C19orf57	19	13993318	1	cg07606495	0.448059815	0.302052767	0.255725632	-0.192334183	6.12E-13	9.33E-11
UTR3	C1orf64	1	16332953	1	cg06947191	0.828336895	0.730978	0.609793355	-0.21854354	7.04E-08	6.40E-07
UTR3	C1orf91	1	32681995	1	cg15726025	0.56673271	0.42936055	0.34954315	-0.21718956	1.76E-10	4.27E-09
UTR3	C1R	12	7187715	1	cg15837470	0.616887595	0.517457528	0.340778336	-0.276109259	2.61E-11	1.12E-09
UTR3	C1RL	12	7248724	1	cg11024687	0.508294735	0.375349833	0.283108391	-0.225186344	8.26E-15	7.46E-12
UTR3	C1S	12	7178171	1	cg24212392	0.72310005	0.566163411	0.452192391	-0.270907659	2.54E-10	5.59E-09
UTR3	C20orf103	20	9511102	1	cg09732036	0.46527833	0.358929122	0.304503323	-0.160775007	4.26E-09	5.78E-08
UTR3	C20orf186	20	31699304	1	cg26615188	0.63801296	0.534514744	0.40539595	-0.23261701	2.28E-11	1.02E-09
UTR3	C20orf197	20	58647209	1	cg17734137	0.783764305	0.707352856	0.612362223	-0.171402082	0.00015472	0.000574683
UTR3	C21orf7	21	30547239	1	cg26289665	0.56935713	0.471611994	0.416196705	-0.153160425	2.33E-06	1.37E-05
UTR3	C22orf32	22	42479360	1	cg05038367	0.799956925	0.744163067	0.618899545	-0.18105738	1.05E-07	9.08E-07
UTR3	C2orf71	2	29287725	1	cg23429306	0.67494109	0.570756717	0.484960073	-0.189981017	9.49E-09	1.14E-07
UTR3	C2orf88	2	191065479	1	cg05277425	0.51259744	0.453317144	0.360020968	-0.152576472	2.29E-08	2.44E-07
UTR3	C2orf90	2	903676	1	cg09613130	0.83853972	0.775605406	0.631398655	-0.207141065	1.39E-05	6.68E-05
UTR3	C3orf18	3	50596116	1	cg09562331	0.51806712	0.42759935	0.347452086	-0.170615034	2.27E-10	5.19E-09
UTR3	C3orf32	3	8661519	1	cg09248934	0.568627975	0.367923972	0.268243714	-0.300384261	1.28E-14	7.46E-12
UTR3	C4orf46	4	159589048	1	cg13698937	0.861857685	0.785895739	0.705059382	-0.156798303	1.60E-06	9.82E-06

UTR3	C5orf38	5	2755158/2755376	2	cg11337945/cg23248357	0.259368058	0.314174429	0.422762336	0.163394278	4.13E-05	0.000177621
UTR3	C5orf39	5	43039410	1	cg26942801	0.381407455	0.254058522	0.14865252	-0.232754935	6.67E-10	1.20E-08
UTR3	C5orf47	5	173430662	1	cg19486949	0.67620277	0.605296244	0.517407514	-0.158795256	3.58E-05	0.00015613
UTR3	C5orf49	5	7831537	1	cg04238199	0.527769625	0.395474722	0.336363391	-0.191406234	1.30E-08	1.51E-07
UTR3	C6	5	41142881	1	cg11590974	0.555414815	0.41633355	0.328930577	-0.226484238	1.08E-11	6.05E-10
UTR3	C6orf132	6	42071219	1	cg00173538	0.55992526	0.452869894	0.396360477	-0.163564783	2.23E-07	1.75E-06
UTR3	C6orf222	6	36283929	1	cg02959688	0.68958497	0.553119011	0.432943623	-0.256641347	4.95E-12	3.80E-10
UTR3	C7orf16	7	31747928	1	cg01227294	0.735600575	0.711654978	0.509239177	-0.226361398	9.26E-08	8.11E-07
UTR3	C7orf31	7	25174492	1	cg23302989	0.456370235	0.387594661	0.295347014	-0.161023221	2.21E-09	3.30E-08
UTR3	C7orf59	7	99751594	1	cg06739004	0.61314792	0.506904611	0.4465261	-0.16662182	6.24E-10	1.14E-08
UTR3	C8A	1	57383752	1	cg23844013	0.69419162	0.557325711	0.462433014	-0.231758606	8.10E-08	7.25E-07
UTR3	C9orf86	9	139735010/139735338	2	cg12986501/cg21175873	0.633834275	0.547546	0.483641805	-0.15019247	1.13E-09	1.86E-08
UTR3	CABIN1	22	24574487	1	cg22804358	0.801910295	0.729260528	0.631959032	-0.169951263	5.45E-08	5.14E-07
UTR3	CABYR	18	21741492/21737584	2	cg05060976/cg05926667	0.58443752	0.4744687	0.3797834	-0.20465412	2.46E-09	3.62E-08
UTR3	CACNA1H	16	1271741/1271153/1271505	3	cg03553819/cg16577083/cg27338302	0.702851358	0.585479443	0.464144995	-0.238706363	6.52E-13	9.65E-11
UTR3	CACNA1I	22	40084724/40082521	2	cg19287501/cg22807707	0.72517109	0.650355456	0.546275832	-0.178895258	5.65E-06	3.00E-05
UTR3	CACNA2D1	7	81579463	1	cg25161868	0.494502705	0.368516528	0.2991846	-0.195318105	1.31E-09	2.12E-08
UTR3	CACNB3	12	49222623	1	cg18046311	0.71615957	0.61241805	0.516350918	-0.199808652	2.70E-09	3.91E-08
UTR3	CACNG1	17	65052432	1	cg25551622	0.58069439	0.486268639	0.400511164	-0.180183226	3.18E-10	6.68E-09
UTR3	CACNG7	19	54445888	1	cg21477176	0.54189333	0.599618967	0.750982605	0.209089275	4.65E-09	6.20E-08
UTR3	CADM3	1	159171213/159172620	2	cg15722977/cg20944283	0.58348568	0.4691273	0.391362782	-0.192122898	8.61E-13	1.14E-10
UTR3	CALB2	16	71420541/71424252	2	cg04628802/cg06389098	0.501763715	0.410015206	0.323653652	-0.178110063	5.33E-11	1.88E-09
UTR3	CALCB	11	15099930	1	cg14023329	0.514916165	0.386493417	0.292157673	-0.222758492	3.90E-10	7.91E-09
UTR3	CALCRL	2	188210673	1	cg22374415	0.770731225	0.673276206	0.466355305	-0.30437592	6.33E-08	5.83E-07
UTR3	CALM2	2	47387494	1	cg21361646	0.72519012	0.612419233	0.545967055	-0.179223065	8.68E-11	2.49E-09
UTR3	CAMKV	3	49896651	1	cg08827110	0.61923488	0.493730678	0.386235873	-0.232999007	6.94E-10	1.24E-08
UTR3	CANX	5	179158569/179156985	2	cg04073204/cg12865675	0.79258621	0.711712756	0.631114159	-0.161472051	3.03E-06	1.74E-05
UTR3	CAV3	3	8787638	1	cg08872064	0.62623978	0.530147033	0.462441209	-0.163798571	5.92E-08	5.52E-07
UTR3	CBLN1	16	49312238/49312543/49313307/49312560	4	cg01687680/cg02696327/cg04438525/cg06919440	0.260911125	0.325161937	0.453050449	0.192139324	4.12E-08	4.02E-07
UTR3	CBLN2	18	70205221	1	cg21298444	0.548542305	0.445515383	0.3542196	-0.194322705	3.35E-08	3.36E-07

UTR3	CCDC102A	16	57546535	1	cg07241532	0.586131225	0.467632	0.405298682	-0.180832543	2.55E-11	1.10E-09
UTR3	CCDC105	19	15133948	1	cg25690897	0.37753688	0.451462378	0.548489895	0.170953015	9.24E-08	8.11E-07
UTR3	CCDC129	7	31692686	1	cg00397871	0.742470715	0.654237539	0.554428345	-0.18804237	1.01E-06	6.50E-06
UTR3	CCDC13	3	42750454	1	cg18816595	0.69771071	0.592404394	0.454997527	-0.242713183	5.50E-09	7.19E-08
UTR3	CCDC140	2	223169352/223169501/223169609/223169586	4	cg00699392/cg02224980/cg09486778/cg15825116	0.316794793	0.416841439	0.540333326	0.223538534	1.61E-08	1.79E-07
UTR3	CCDC21	1	26605141/26603970	2	cg12445424/cg21646082	0.524107395	0.417982561	0.349103759	-0.175003636	1.45E-09	2.30E-08
UTR3	CCDC33	15	74627589	1	cg08932277	0.723042435	0.662957506	0.562780114	-0.160262321	1.87E-05	8.78E-05
UTR3	CCDC40	17	78073854/78073693	2	cg04028464/cg06485101	0.62632461	0.529677075	0.436104464	-0.190220146	1.24E-11	6.78E-10
UTR3	CCDC46	17	63632069	1	cg11989257	0.866972145	0.705373456	0.653869918	-0.213102227	3.24E-08	3.27E-07
UTR3	CCDC56	17	40949954/40949811	2	cg22239334/cg24994625	0.599132055	0.471085933	0.364984909	-0.234147146	2.75E-11	1.15E-09
UTR3	CCDC74B	2	130897120	1	cg15638768	0.67402883	0.511845961	0.451492682	-0.222536148	4.94E-09	6.52E-08
UTR3	CCDC8	19	46913677	1	cg26232899	0.756164505	0.661324128	0.514846864	-0.241317641	6.88E-08	6.27E-07
UTR3	CCDC80	3	112323619	1	cg23451678	0.3660656	0.281015489	0.191044916	-0.175020684	2.52E-06	1.48E-05
UTR3	CCDC83	11	85630744	1	cg20891655	0.750618375	0.639808128	0.5349994	-0.215618975	9.50E-09	1.14E-07
UTR3	CCKBR	11	6293288/6292896	2	cg04488853/cg25740457	0.702458888	0.630611583	0.53709797	-0.165360917	4.14E-07	3.00E-06
UTR3	CCL19	9	34689585	1	cg13665853	0.52616351	0.422219206	0.341981305	-0.184182205	1.52E-10	3.74E-09
UTR3	CCNA1	13	37016876/37016859	2	cg01774835/cg07345354	0.708717358	0.635044906	0.502361166	-0.206356192	2.25E-05	0.000103823
UTR3	CCR3	3	46307918	1	cg03050629	0.80339943	0.67791555	0.569119505	-0.234279925	8.37E-07	5.50E-06
UTR3	CD1D	1	158155757	1	cg18234111	0.57826903	0.392698378	0.269737286	-0.308531744	1.04E-10	2.84E-09
UTR3	CD200R1	3	112642487/112647810	2	cg00246486/cg17069590	0.69815276	0.554718053	0.430179209	-0.267973551	9.94E-10	1.69E-08
UTR3	CD207	2	71057572	1	cg03976366	0.62993436	0.537241128	0.416844268	-0.213090092	3.57E-08	3.54E-07
UTR3	CD300A	17	72480644	1	cg14018141	0.35063887	0.265813022	0.19434425	-0.15629462	8.20E-10	1.42E-08
UTR3	CD300LF	17	72690539	1	cg08808817	0.71191849	0.547566756	0.448119541	-0.263798949	4.05E-09	5.52E-08
UTR3	CD4	12	6929671/6929591	2	cg03795245/cg08349804	0.63156232	0.535919747	0.445486777	-0.186075543	6.62E-09	8.40E-08
UTR3	CD69	12	9905753	1	cg05179921	0.649408575	0.522604261	0.408584964	-0.240823611	1.20E-10	3.13E-09
UTR3	CD96	3	111370016	1	cg25785241	0.65074642	0.555912633	0.449104745	-0.201641675	1.87E-09	2.88E-08
UTR3	CD97	19	14519121	1	cg16738984	0.49682087	0.380979556	0.304083459	-0.192737411	7.64E-11	2.32E-09
UTR3	CDC42EP1	22	37965042	1	cg26347632	0.44897573	0.364659733	0.282027723	-0.166948007	6.01E-10	1.11E-08
UTR3	CDCA5	11	64845676	1	cg09955152	0.63171157	0.557927928	0.449717609	-0.181993961	7.25E-08	6.57E-07
UTR3	CDH13	16	83829911	1	cg05847519	0.672865515	0.479692039	0.321072532	-0.351792983	1.61E-14	8.62E-12
UTR3	CDH18	5	19473185/19478817	2	cg06700166/cg08814749	0.682518545	0.563838433	0.441274355	-0.24124419	8.78E-07	5.73E-06

UTR3	CDH22	20	44802548	1	cg10329468	0.556791245	0.4375503	0.346644341	-0.210146904	1.36E-10	3.42E-09
UTR3	CDK6	7	92242243/92243191/92242370/92238207/92238086/92237896/92238248/92237376/92238364/92238156	10	cg03330377/cg05517400/cg05977021/cg06688763/cg07413467/cg14100946/cg15261712/cg15732164/cg23628117/cg25284397	0.79496262	0.679943925	0.594902049	-0.200060571	7.73E-05	0.000310424
UTR3	CEACAM21	19	42093086	1	cg08478046	0.53352205	0.417277772	0.306848605	-0.226673445	3.62E-13	6.37E-11
UTR3	CEACAM5	19	42234062	1	cg24790544	0.575697765	0.448352528	0.349409836	-0.226287929	4.18E-10	8.27E-09
UTR3	CEBPE	14	23586582	1	cg02246876	0.784518155	0.6987541	0.601300527	-0.183217628	7.25E-06	3.74E-05
UTR3	CENPQ	6	49460031	1	cg14479014	0.809034365	0.619657528	0.547317405	-0.26171696	7.29E-11	2.25E-09
UTR3	CERK	22	47081634/47082260/47082044/47081903/47081751	5	cg05602642/cg06766034/cg12509665/cg18614510/cg24157349	0.460573208	0.360941497	0.286367183	-0.174206025	1.77E-10	4.27E-09
UTR3	CES8	16	67043406	1	cg06815910	0.63243878	0.561410456	0.448128127	-0.184310653	1.40E-07	1.16E-06
UTR3	CHCHD1	10	75543235	1	cg27048208	0.45720657	0.363842811	0.271766059	-0.185440511	8.53E-12	5.34E-10
UTR3	CHGB	20	5905708	1	cg07140797	0.769578445	0.692347428	0.548339173	-0.221239272	1.09E-05	5.39E-05
UTR3	CHL1	3	447414	1	cg06088575	0.80915255	0.755713944	0.646481805	-0.162670745	5.27E-05	0.000220398
UTR3	CHRD	3	184107534	1	cg05308645	0.609541755	0.533995211	0.447928145	-0.16161361	2.69E-08	2.79E-07
UTR3	CHRNA2	8	27318934/27317394	2	cg00254556/cg16291069	0.561193683	0.452708514	0.370846175	-0.190347508	7.41E-12	4.98E-10
UTR3	CHST10	2	101008576	1	cg01660407	0.87220465	0.676626267	0.578576291	-0.293628359	2.20E-08	2.35E-07
UTR3	CHST4	16	71572050	1	cg02517497	0.457763735	0.359047444	0.300687573	-0.157076162	2.26E-11	1.02E-09
UTR3	CIB1	15	90773570	1	cg01438056	0.4691391	0.366088739	0.302516014	-0.166623086	1.33E-08	1.54E-07
UTR3	CIB3	19	16272242	1	cg01541846	0.66506362	0.510210433	0.394911218	-0.270152402	2.85E-11	1.18E-09
UTR3	CISD1	10	60047571	1	cg09500807	0.51932251	0.450967761	0.353116159	-0.166206351	1.64E-11	8.16E-10
UTR3	CLDN18	3	137750103	1	cg09236311	0.64847136	0.482089061	0.334410223	-0.314061137	8.37E-11	2.46E-09
UTR3	CLDN22	4	184240083	1	cg26806835	0.53614318	0.4404506	0.324162777	-0.211980403	6.63E-10	1.20E-08
UTR3	CLDN4	7	73246406	1	cg02874145	0.39144086	0.310749389	0.221575818	-0.169865042	2.12E-09	3.20E-08
UTR3	CLDN7	17	7163599	1	cg13724311	0.623190215	0.48396235	0.391549991	-0.231640224	7.74E-12	5.03E-10
UTR3	CLEC10A	17	6977901	1	cg21215820	0.747247565	0.634488444	0.596580677	-0.150666888	0.000108224	0.000419061
UTR3	CLEC2A	12	10065986	1	cg24640735	0.677238055	0.589198289	0.385397318	-0.291840737	2.61E-10	5.73E-09
UTR3	CLEC4D	12	8674684	1	cg05582397	0.660027525	0.503199733	0.378058127	-0.281969398	7.08E-11	2.20E-09
UTR3	CLEC4E	12	8686707	1	cg16009120	0.755298605	0.652566894	0.543987982	-0.211310623	7.43E-06	3.82E-05
UTR3	CLSTN2	3	140286910	1	cg05092583	0.774808055	0.647497739	0.514253723	-0.260554332	3.97E-07	2.89E-06
UTR3	CLTB	5	175819705	1	cg22584580	0.588831405	0.503498322	0.399519323	-0.189312082	5.67E-11	1.93E-09

UTR3	CLVS2	6	123385000	1	cg03469655	0.57915359	0.454929944	0.339208118	-0.239945472	9.53E-07	6.16E-06
UTR3	CMTM1	16	66612955	1	cg09849405	0.204523596	0.377786624	0.654003182	0.449479587	1.19E-09	1.96E-08
UTR3	CNDP1	18	72252164	1	cg21700214	0.68480289	0.509472078	0.431826264	-0.252976626	1.36E-09	2.17E-08
UTR3	CNPY4	7	99723100	1	cg26650651	0.73284279	0.631564522	0.522634659	-0.210208131	5.78E-08	5.41E-07
UTR3	CNTLN	9	17503269	1	cg14418582	0.765941525	0.699792756	0.5799673	-0.185974225	8.41E-06	4.28E-05
UTR3	CNTN5	11	100227279	1	cg03587117	0.686754415	0.589920333	0.417679709	-0.269074706	3.44E-08	3.43E-07
UTR3	COG5	7	106842512/106842632	2	cg10840361/cg24556660	0.504900923	0.399028481	0.33026438	-0.174636543	4.83E-11	1.76E-09
UTR3	COL29A1	3	130203349	1	cg24315710	0.472933315	0.351654789	0.235141541	-0.237791774	3.11E-13	5.79E-11
UTR3	COL9A1	6	70926272	1	cg20682720	0.72759427	0.624673161	0.5228355	-0.20475877	1.64E-07	1.34E-06
UTR3	COPS6	7	99689591	1	cg10192641	0.70685842	0.610558761	0.546908077	-0.159950343	6.12E-07	4.25E-06
UTR3	COQ7	16	19089709	1	cg16660394	0.63601539	0.753120594	0.787432045	0.151416655	4.06E-08	3.97E-07
UTR3	CORO2A	9	100883593	1	cg13836518	0.347780695	0.219338494	0.175570846	-0.172209849	5.46E-11	1.89E-09
UTR3	CPA3	3	148614668	1	cg18516150	0.371184205	0.282227461	0.217191218	-0.153992987	2.29E-11	1.02E-09
UTR3	CPLX1	4	779173/778827/778924/779230/779691/779480/779880/779568	8	cg05448404/cg06672737/cg06673130/cg06821199/cg07434284/cg17128947/cg17397631/cg18638914	0.373247237	0.5248243	0.645681274	0.272434036	2.70E-09	3.91E-08
UTR3	CPLX2	5	175309346	1	cg10663557	0.62226162	0.532455511	0.406938686	-0.215322934	1.24E-06	7.78E-06
UTR3	CRABP1	15	78640536/78640434	2	cg07199257/cg26270695	0.605740995	0.471064258	0.450081973	-0.155659022	5.65E-09	7.36E-08
UTR3	CRADD	12	94244496	1	cg05059922	0.693583035	0.546979089	0.489816114	-0.203766921	3.36E-09	4.70E-08
UTR3	CRISPLD1	8	75946479	1	cg12133103	0.792506815	0.670728394	0.492556241	-0.299950574	4.19E-09	5.69E-08
UTR3	CRP	1	159682497	1	cg04861230	0.58299582	0.429962206	0.335184786	-0.247811034	1.77E-13	4.62E-11
UTR3	CSMD2	1	33982683/33985111	2	cg04159098/cg08861449	0.730240505	0.637207475	0.557029289	-0.173211216	1.39E-08	1.60E-07
UTR3	CSPG5	3	47603786	1	cg23009042	0.40799713	0.294935517	0.2450812	-0.16291593	1.05E-09	1.75E-08
UTR3	CST4	20	23666505	1	cg13974790	0.48614463	0.385764172	0.27210395	-0.21404068	1.72E-13	4.61E-11
UTR3	CTSF	11	66330936	1	cg19255818	0.7097867	0.629014694	0.495598236	-0.214188464	1.70E-07	1.38E-06
UTR3	CX3CR1	3	39306129	1	cg04498110	0.63816995	0.511105922	0.357256145	-0.280913805	1.98E-10	4.66E-09
UTR3	CXCL2	4	74963112	1	cg01470535	0.588330015	0.436522583	0.34538545	-0.242944565	3.13E-13	5.79E-11
UTR3	CXCL3	4	74902951	1	cg13468041	0.41702008	0.307877383	0.230839191	-0.186180889	7.41E-13	1.04E-10
UTR3	CXCR1	2	219028181	1	cg09905973	0.57586265	0.516595767	0.418598023	-0.157264627	4.06E-07	2.95E-06
UTR3	CYB5D2	17	4060650	1	cg15741124	0.56388696	0.441718928	0.371963914	-0.191923046	4.63E-11	1.72E-09
UTR3	DAB1	1	57470853	1	cg01421140	0.501780055	0.351612833	0.254225914	-0.247554141	9.25E-13	1.18E-10
UTR3	DAPP1	4	100790361	1	cg12001078	0.792394045	0.717374578	0.6416928	-0.150701245	9.65E-06	4.83E-05

UTR3	DCAF4L2	8	88884156/88884985	2	cg13008331/cg15707869	0.539520485	0.451251525	0.377598298	-0.161922187	4.88E-09	6.47E-08
UTR3	DCDC2B	1	32681593	1	cg06909646	0.527690135	0.429460994	0.362153609	-0.165536526	1.33E-09	2.14E-08
UTR3	DCHS2	4	155244316	1	cg03842933	0.58072848	0.458519239	0.343800218	-0.236928262	1.07E-08	1.27E-07
UTR3	DCP2	5	112356289	1	cg10837404	0.83586094	0.694385061	0.548579195	-0.287281745	1.37E-08	1.58E-07
UTR3	DDI1	11	103908787	1	cg25859489	0.856011715	0.774900711	0.635912295	-0.22009942	6.22E-08	5.76E-07
UTR3	DDX51	12	132622514	1	cg21131424	0.801661825	0.687279917	0.612083855	-0.18957797	5.19E-08	4.93E-07
UTR3	DEFB110	6	49986643	1	cg19765351	0.765906165	0.668897244	0.574276618	-0.191629547	3.91E-05	0.0001691
UTR3	DEFB119	20	29965177	1	cg07594472	0.658123905	0.560924528	0.454900241	-0.203223664	1.47E-05	7.04E-05
UTR3	DENND5A	11	9160869	1	cg19450302	0.57565416	0.458060761	0.363100355	-0.212553805	3.98E-12	3.25E-10
UTR3	DENND5B	12	31538503	1	cg00182053	0.852821865	0.746444794	0.583098873	-0.269722992	6.16E-10	1.13E-08
UTR3	DFNA5	7	24738572	1	cg17790129	0.8416488	0.744626211	0.658500932	-0.183147868	1.43E-06	8.85E-06
UTR3	DGKH	13	42803600	1	cg19307180	0.64371907	0.542490233	0.467362936	-0.176356134	1.15E-12	1.36E-10
UTR3	DGKI	7	137075954	1	cg21146652	0.70149302	0.601263183	0.53968905	-0.16180397	1.43E-07	1.19E-06
UTR3	DIO2	14	80668655	1	cg14293892	0.601002125	0.509155211	0.411929086	-0.189073039	1.56E-06	9.56E-06
UTR3	DIRC1	2	189599296	1	cg26387064	0.51815818	0.382614578	0.262043336	-0.256114844	9.33E-12	5.60E-10
UTR3	DKK3	11	11984899	1	cg26235273	0.494295965	0.39256235	0.331494509	-0.162801456	9.82E-11	2.76E-09
UTR3	DKKL1	19	49878296	1	cg04309404	0.794934795	0.699702622	0.585434591	-0.209500204	1.53E-08	1.71E-07
UTR3	DLG2	11	83167314/83169193	2	cg23791672/cg26449294	0.68391667	0.591936442	0.513037457	-0.170879213	5.13E-12	3.88E-10
UTR3	DMRTA2	1	50884242/50883329/50883394	3	cg21774338/cg22711792/cg23097402	0.28725513	0.36524962	0.578860173	0.291605043	7.57E-07	5.06E-06
UTR3	DMRTB1	1	53932577	1	cg15962336	0.731929295	0.671436244	0.567648523	-0.164280772	9.56E-05	0.000374856
UTR3	DNAJC15	13	43682146	1	cg22318792	0.85943771	0.762210511	0.692338782	-0.167098928	1.48E-08	1.67E-07
UTR3	DNAJC5B	8	67012327	1	cg14928319	0.44775702	0.335352706	0.267571659	-0.180185361	5.83E-12	4.19E-10
UTR3	DNAJC7	17	40128637	1	cg03593057	0.324213295	0.438598261	0.496021845	0.17180855	2.11E-06	1.25E-05
UTR3	DNTT	10	98098271	1	cg13444670	0.713757315	0.649293511	0.483406882	-0.230350433	3.35E-07	2.49E-06
UTR3	DOC2A	16	30017470/30016874	2	cg08078586/cg09245872	0.611879845	0.525898778	0.445276293	-0.166603552	4.37E-09	5.89E-08
UTR3	DOCK2	5	169510081	1	cg09175734	0.637470355	0.46170475	0.335555305	-0.30191505	2.42E-13	5.50E-11
UTR3	DOLPP1	9	131851856	1	cg14126328	0.83625589	0.7636646	0.686154932	-0.150100958	2.07E-07	1.64E-06
UTR3	DPCR1	6	30920956/30921592/30921757/30921007/30920907/30921630	6	cg02818451/cg11485823/cg11554728/cg13871708/cg18796319/cg18964706	0.506285125	0.425013753	0.347263682	-0.159021443	1.83E-08	2.00E-07
UTR3	DPPA5	6	74063014	1	cg07464621	0.758456805	0.669273656	0.551647977	-0.206808828	8.73E-07	5.70E-06

UTR3	DUSP13	10	76859383/76857530/76859441/76859206/76854201/76859295/76859248/76860346/76860251/76859323/76860035/76859350	12	cg04369919/cg10353925/cg10522115/cg10963664/cg11612555/cg11709896/cg12256803/cg16221975/cg17292610/cg19532742/cg19591573/cg24634577	0.605509274	0.53316533	0.453687999	-0.151821276	3.76E-12	3.12E-10
UTR3	DYNLL1	12	120936243	1	cg23974249	0.515574245	0.4245887	0.339232186	-0.176342059	1.25E-10	3.23E-09
UTR3	EBF1	5	158125970	1	cg16394305	0.65559294	0.535946711	0.382240991	-0.273351949	5.10E-11	1.83E-09
UTR3	ECH1	19	39306244	1	cg12028674	0.537122755	0.407828261	0.296433405	-0.24068935	7.83E-10	1.36E-08
UTR3	ECM1	1	150486261	1	cg00175150	0.651042425	0.545232422	0.451020564	-0.200021861	1.29E-08	1.50E-07
UTR3	EDNRA	4	148465429	1	cg03573792	0.664956465	0.514164483	0.458468332	-0.206488133	4.22E-08	4.10E-07
UTR3	EEF1A2	20	62119492	1	cg26186239	0.38874984	0.487190211	0.579219764	0.190469924	1.13E-06	7.18E-06
UTR3	EFHD1	2	233547150	1	cg11593908	0.775196615	0.72410185	0.594341027	-0.180855588	8.34E-05	0.000331464
UTR3	EHD2	19	48246266	1	cg01559302	0.517371465	0.433027206	0.352763427	-0.164608038	1.85E-11	8.94E-10
UTR3	EIF4EBP2	10	72185663	1	cg05523877	0.693589435	0.560137044	0.481461823	-0.212127612	2.70E-11	1.14E-09
UTR3	ELF3	1	201985958/201984631	2	cg09262245/cg11802442	0.47771739	0.367612086	0.289984595	-0.187732795	3.61E-11	1.43E-09
UTR3	ELFN1	7	1787194/1786735/1787310/1787371	4	cg01310330/cg02691362/cg15703970/cg22104371	0.769912815	0.730319413	0.617637764	-0.152275051	6.29E-10	1.15E-08
UTR3	ELFN2	22	37765272/37768915	2	cg14378259/cg27107094	0.74699482	0.642178028	0.570214123	-0.176780697	4.39E-09	5.91E-08
UTR3	ELK4	1	205585639	1	cg04105597	0.68138763	0.518637028	0.415228641	-0.266158989	6.63E-11	2.11E-09
UTR3	ELOVL2	6	10981184	1	cg20462795	0.66241619	0.529885233	0.423678795	-0.238737395	6.62E-07	4.49E-06
UTR3	EMX1	2	73161954	1	cg13656408	0.578524215	0.484855806	0.375815318	-0.202708897	7.82E-10	1.36E-08
UTR3	EMX2	10	119307769/119307931/119307692	3	cg13630043/cg17320707/cg19925204	0.20821151	0.251114512	0.409820419	0.201608908	1.41E-06	8.75E-06
UTR3	EN1	2	119600348/119600002/119599819/119599748/119600408	5	cg15888290/cg16794506/cg21215767/cg21811143/cg22970357	0.23262711	0.297321828	0.42795329	0.19532618	1.34E-05	6.50E-05
UTR3	ENPP1	6	132216033	1	cg18796704	0.563352985	0.434267872	0.341923232	-0.221429753	1.55E-11	7.97E-10
UTR3	ENPP6	4	185012068	1	cg08334726	0.678058885	0.603845467	0.46766505	-0.210393835	4.01E-06	2.22E-05
UTR3	ENTHD1	22	40139626	1	cg11757326	0.792336605	0.769785883	0.641580127	-0.150756478	0.001365912	0.004065869
UTR3	EPHA8	1	22929080	1	cg10948827	0.500337725	0.412532017	0.321810932	-0.178526793	5.68E-11	1.93E-09
UTR3	EPHB4	7	100400274	1	cg22493616	0.578426675	0.486434267	0.387014936	-0.191411739	8.22E-11	2.43E-09

UTR3	EPS8L1	19	55599029	1	cg10016788	0.384508685	0.510922939	0.666173845	0.28166516	7.66E-08	6.89E-07
UTR3	EPYC	12	91357909	1	cg19537969	0.643523905	0.575862267	0.483298805	-0.1602251	3.04E-06	1.74E-05
UTR3	ESM1	5	54275155	1	cg00992687	0.718398305	0.596335983	0.464562891	-0.253835414	1.49E-11	7.78E-10
UTR3	ESRRB	14	76967670	1	cg11763914	0.567960305	0.457641139	0.354765068	-0.213195237	1.30E-07	1.09E-06
UTR3	EWSR1	22	29686496	1	cg11942221	0.79697509	0.713640467	0.638429677	-0.158545413	5.63E-06	2.99E-05
UTR3	EYA4	6	133851427/133851460/133851542	3	cg07817705/cg09649901/cg10782703	0.76600718	0.667307546	0.607034074	-0.158973106	8.11E-11	2.42E-09
UTR3	F2R	5	76029522	1	cg01005365	0.642821665	0.508404467	0.389723736	-0.253097929	2.37E-11	1.05E-09
UTR3	FAIM3	1	207077339	1	cg22671342	0.80168361	0.731530422	0.557446668	-0.244236942	1.34E-06	8.36E-06
UTR3	FAM104A	17	71204820	1	cg21700339	0.817183085	0.750012333	0.632379182	-0.184803903	7.73E-09	9.55E-08
UTR3	FAM113B	12	47630263	1	cg06547285	0.68285749	0.604956894	0.519730373	-0.163127117	4.87E-06	2.63E-05
UTR3	FAM126A	7	22982162	1	cg27229764	0.83985201	0.644167483	0.495510705	-0.344341305	1.07E-10	2.87E-09
UTR3	FAM12A	14	21216325	1	cg19906534	0.711316055	0.583779889	0.524257905	-0.18705815	2.18E-08	2.33E-07
UTR3	FAM13C	10	61007196	1	cg07625992	0.770272225	0.789121933	0.617386782	-0.152885443	0.001374368	0.004082704
UTR3	FAM150A	8	53450729	1	cg07055311	0.702819595	0.619731756	0.529788509	-0.173031086	7.99E-06	4.08E-05
UTR3	FAM153B	5	175541958	1	cg08252384	0.66048473	0.504976317	0.3864653	-0.27401943	1.32E-14	7.46E-12
UTR3	FAM158A	14	24608199	1	cg20933586	0.818297005	0.725956528	0.654738491	-0.163558514	6.06E-08	5.62E-07
UTR3	FAM169B	15	98982463	1	cg18576057	0.571798675	0.476288578	0.33510155	-0.236697125	1.93E-11	9.25E-10
UTR3	FAM177B	1	222923875	1	cg24666355	0.693060795	0.573116061	0.522239505	-0.17082129	2.82E-10	6.08E-09
UTR3	FAM181A	14	94395516	1	cg18857951	0.731475115	0.559816233	0.464809332	-0.266665783	9.54E-12	5.69E-10
UTR3	FAM19A2	12	62102663	1	cg10081664	0.804826995	0.744657072	0.6483451	-0.156481895	7.91E-06	4.04E-05
UTR3	FAM19A5	22	49146166/49147023/49146376/49146584	4	cg00373397/cg05532594/cg14432153/cg14596108	0.718646195	0.618306983	0.565055377	-0.153590818	2.96E-13	5.74E-11
UTR3	FAM26D	6	116879492	1	cg25351588	0.729955935	0.512860772	0.382538236	-0.347417699	6.93E-15	7.46E-12
UTR3	FAM26E	6	116839146	1	cg04312119	0.6660065	0.562379411	0.406034514	-0.259971986	6.58E-11	2.10E-09
UTR3	FAM43A	3	194408901/194408845/194408965/194409431	4	cg00352417/cg09652652/cg10623840/cg11740416	0.26215094	0.408936706	0.452273838	0.190122898	4.83E-05	0.000203738
UTR3	FAM59B	2	26411705	1	cg07902785	0.674212345	0.564079767	0.472085691	-0.202126654	3.41E-12	2.95E-10
UTR3	FAM5B	1	177251261	1	cg18766795	0.57424316	0.476150889	0.390396818	-0.183846342	4.64E-09	6.20E-08
UTR3	FAM71C	12	100043668	1	cg03657350	0.56038941	0.443812117	0.390348264	-0.170041146	5.01E-07	3.58E-06
UTR3	FAM71E1	19	50970203	1	cg00748226	0.39930922	0.3096889	0.245594759	-0.153714461	3.75E-11	1.47E-09
UTR3	FAM78A	9	134135008/134135934	2	cg00524964/cg14424770	0.794867283	0.693502653	0.602830959	-0.192036323	6.73E-10	1.21E-08
UTR3	FAM78B	1	166039444	1	cg21908886	0.60072842	0.4828818	0.368567059	-0.232161361	1.29E-10	3.32E-09
UTR3	FAM92B	16	85132373	1	cg02853355	0.748393785	0.662338694	0.586325368	-0.162068417	5.05E-07	3.60E-06

UTR3	FANCL	2	58386677	1	cg00869533	0.72031767	0.625852844	0.554663109	-0.165654561	4.67E-10	9.02E-09
UTR3	FBLN1	22	45996347/45959202/45961452	3	cg02163215/cg08414809/cg10289926	0.655140405	0.550175789	0.4379626	-0.217177805	1.63E-07	1.33E-06
UTR3	FBXL8	16	67198029	1	cg06277900	0.03345965	0.093408285	0.20158444	0.16812479	0.000124759	0.000473921
UTR3	FBXO17	19	39432669	1	cg08643128	0.593259145	0.526448839	0.443038727	-0.150220418	1.64E-05	7.73E-05
UTR3	FCN3	1	27695677	1	cg11529819	0.528560735	0.412520183	0.359926764	-0.168633971	1.74E-09	2.70E-08
UTR3	FCRLA	1	161684066	1	cg11238082	0.822824955	0.752089106	0.667346991	-0.155477964	1.70E-07	1.37E-06
UTR3	FCRLB	1	161697574	1	cg07385423	0.362544925	0.458632356	0.549546064	0.187001139	1.07E-06	6.88E-06
UTR3	FERD3L	7	19184427	1	cg02503874	0.218303745	0.3473513	0.473612477	0.255308732	2.15E-08	2.31E-07
UTR3	FEZF2	3	62355505/62355443/62355525	3	cg11178293/cg12378187/cg18175809	0.449229198	0.524748917	0.687487177	0.23825798	5.01E-11	1.81E-09
UTR3	FGF9	13	22277315/22275925	2	cg22356541/cg26585320	0.779463995	0.748968917	0.591004314	-0.188459681	0.000531471	0.001746531
UTR3	FIBIN	11	27017005	1	cg20119891	0.69863793	0.621167417	0.538131459	-0.160506471	0.000185568	0.000676946
UTR3	FLI1	11	128681282/128682689	2	cg07486669/cg11609631	0.835998915	0.730196511	0.63710583	-0.198893085	7.11E-08	6.45E-07
UTR3	FLJ10357	14	21557638	1	cg13093389	0.433563755	0.347984867	0.252233077	-0.181330678	7.60E-11	2.32E-09
UTR3	FLJ25006	17	26937650	1	cg12560320	0.74944403	0.622230394	0.536892841	-0.212551189	2.15E-09	3.23E-08
UTR3	FLJ37543	5	61001539	1	cg15930643	0.545289075	0.485036861	0.345844118	-0.199444957	1.26E-07	1.06E-06
UTR3	FLJ41603	5	149012102	1	cg01772439	0.52271914	0.37926915	0.299869986	-0.222849154	5.49E-13	8.49E-11
UTR3	FLJ43860	8	142444018	1	cg23647640	0.62587901	0.522229072	0.408431477	-0.217447533	1.20E-14	7.46E-12
UTR3	FMNL2	2	153504453	1	cg12226948	0.613968445	0.509929933	0.421226027	-0.192742418	6.83E-08	6.22E-07
UTR3	FMOD	1	203311314	1	cg26894354	0.367178645	0.243190667	0.198562577	-0.168616068	2.48E-11	1.08E-09
UTR3	FNDC1	6	159692484	1	cg13181026	0.726886705	0.618804239	0.507516609	-0.219370096	7.68E-09	9.50E-08
UTR3	FOXC2	16	86602533/86602471	2	cg03190266/cg26948907	0.362344055	0.445014956	0.575122759	0.212778704	8.95E-06	4.52E-05
UTR3	FOXD3	1	63790202	1	cg21836358	0.439180485	0.485118322	0.589245014	0.150064529	9.40E-05	0.000369662
UTR3	FOXF1	16	86547530/86547386/86547544/86547322/86547203/86546785/86546979/86546938	8	cg00551679/cg01243371/cg02783918/cg04787888/cg06834912/cg07056644/cg09338251/cg27453745	0.357489856	0.459747748	0.565244533	0.207754677	9.17E-07	5.97E-06
UTR3	FPR1	19	52249171	1	cg17724366	0.685523455	0.550598956	0.412818109	-0.272705346	3.64E-10	7.50E-09
UTR3	FSHB	11	30255943	1	cg13280803	0.569894375	0.459372722	0.363608491	-0.206285884	4.48E-08	4.33E-07
UTR3	FSTL4	5	132534569	1	cg27038382	0.587007545	0.512858583	0.393957323	-0.193050222	3.42E-08	3.42E-07
UTR3	FTCD	21	47556798/47556290	2	cg14268780/cg21230535	0.57140378	0.470037722	0.362505255	-0.208898525	2.42E-11	1.06E-09

UTR3	FUT4	11	94282491/94278912/94279068	3	cg02856190/cg10283505/cg13300301	0.522864862	0.418103174	0.334362005	-0.188502857	5.37E-11	1.88E-09
UTR3	FXC1	11	6505071/6504366/6504311/6503801	4	cg02796744/cg19461880/cg21750233/cg24744697	0.736319524	0.668920953	0.559092325	-0.177227199	2.16E-06	1.28E-05
UTR3	FZD5	2	208629716/208627796/208631684/208631081/208631259	5	cg01383128/cg01574741/cg15714846/cg19744498/cg19957162	0.499945375	0.393288659	0.32392232	-0.176023055	1.61E-11	8.11E-10
UTR3	FZD9	7	72850442	1	cg09804496	0.4738502	0.4056949	0.300895641	-0.172954559	8.89E-10	1.52E-08
UTR3	GAB4	22	17443619	1	cg04561937	0.692590555	0.561962828	0.457181718	-0.235408837	6.58E-10	1.19E-08
UTR3	GABRG3	15	27778081/27778361	2	cg09205438/cg09459714	0.657252493	0.567116417	0.494667473	-0.16258502	3.03E-09	4.34E-08
UTR3	GABRR1	6	89888334	1	cg06704469	0.6795119	0.609759544	0.467889105	-0.211622795	4.48E-07	3.23E-06
UTR3	GALNS	16	88880839/88880326	2	cg07774081/cg07947266	0.801448725	0.662117744	0.601679786	-0.199768939	2.54E-09	3.72E-08
UTR3	GALNT14	2	31133547	1	cg10582371	0.567094185	0.407295439	0.319374855	-0.24771933	1.14E-10	3.03E-09
UTR3	GALNTL5	7	151716974	1	cg01662876	0.66252006	0.586377156	0.456383764	-0.206136296	3.47E-09	4.83E-08
UTR3	GAMT	19	1397305	1	cg03076130	0.53456142	0.455096706	0.376107282	-0.158454138	1.26E-10	3.24E-09
UTR3	GAP43	3	115440301	1	cg12146703	0.81006068	0.706065717	0.64444565	-0.16561503	1.31E-06	8.16E-06
UTR3	GAS2L2	17	34071861	1	cg17190117	0.64903646	0.542702033	0.45122045	-0.19781601	1.56E-08	1.74E-07
UTR3	GAS7	17	9819556	1	cg01236299	0.677461515	0.546391144	0.417421977	-0.260039538	8.78E-12	5.41E-10
UTR3	GATA4	8	11617305/11616798/11617293	3	cg01644798/cg15644626/cg24387126	0.535576443	0.465358269	0.376187398	-0.159389045	4.11E-09	5.60E-08
UTR3	GBP6	1	89853364	1	cg24581183	0.790512085	0.738534294	0.636737391	-0.153774694	6.77E-05	0.000275013
UTR3	GC	4	72608149	1	cg04837494	0.56248382	0.441710256	0.325871568	-0.236612252	3.97E-10	7.93E-09
UTR3	GDF3	12	7842389	1	cg18607010	0.81561887	0.737630328	0.612860659	-0.202758211	4.98E-07	3.56E-06
UTR3	GEMIN7	19	45594670/45594493/45594410	3	cg03270332/cg08436089/cg18383668	0.517435248	0.444744219	0.35804318	-0.159392068	2.02E-08	2.18E-07
UTR3	GFPT2	5	179728553	1	cg26918954	0.687336895	0.585780728	0.423299977	-0.264036918	4.94E-09	6.52E-08
UTR3	GFRA4	20	3640545	1	cg09309950	0.805236475	0.685461756	0.618243764	-0.186992711	9.26E-07	6.02E-06
UTR3	GFRAL	6	55267138	1	cg15756928	0.77928246	0.6929783	0.565065773	-0.214216687	1.31E-07	1.10E-06
UTR3	GGA1	22	38029478/38013333	2	cg21268578/cg22511321	0.41633256	0.527307811	0.597284609	0.180952049	1.50E-07	1.23E-06
UTR3	GHR	5	42720521	1	cg18304305	0.629837335	0.449580439	0.387788564	-0.242048771	6.20E-07	4.25E-06
UTR3	GIMAP1	7	150418017/150418241	2	cg11844315/cg23713249	0.6269493	0.57887865	0.465591345	-0.161357955	4.31E-05	0.000183858
UTR3	GIP	17	47035958	1	cg16167524	0.45437836	0.365781261	0.283451841	-0.170926519	5.88E-11	1.98E-09
UTR3	GJA9	1	39339986	1	cg01562897	0.667237225	0.550578011	0.490785718	-0.176451507	2.81E-07	2.14E-06

UTR3	GJB6	13	20796216	1	cg03568673	0.620598725	0.480678711	0.357646886	-0.262951839	2.75E-12	2.48E-10
UTR3	GK2	4	80327523	1	cg17197065	0.520917465	0.397235117	0.302443518	-0.218473947	5.12E-09	6.72E-08
UTR3	GLI1	12	57865987	1	cg09071093	0.388413875	0.278451778	0.213209182	-0.175204693	2.14E-11	9.89E-10
UTR3	GLYATL2	11	58601627	1	cg21535655	0.807504265	0.695574106	0.609626059	-0.197878206	7.55E-08	6.81E-07
UTR3	GNG7	19	2514622/2513356/2513687/2512999/2513436/2513302/2511707/2511263/2511475	9	cg04158792/cg05456713/cg08626004/cg12211091/cg13701180/cg19293162/cg24299136/cg25556841/cg27181295	0.829295033	0.727728338	0.621307328	-0.207987704	2.84E-11	1.18E-09
UTR3	GOLGA6B	15	72958737	1	cg04899153	0.59450021	0.529191222	0.440586486	-0.153913724	8.62E-11	2.48E-09
UTR3	GOLGA6D	15	75587027	1	cg00218707	0.693449245	0.636085483	0.505548268	-0.187900977	2.42E-11	1.06E-09
UTR3	GP6	19	55526208	1	cg27154418	0.780080255	0.607387206	0.41956515	-0.360515105	1.46E-07	1.21E-06
UTR3	GPC5	13	93518730	1	cg16437048	0.70098535	0.565797717	0.482239086	-0.218746264	1.84E-06	1.12E-05
UTR3	GPC6	13	95055638	1	cg23445525	0.65325716	0.543046217	0.411580836	-0.241676324	4.18E-11	1.59E-09
UTR3	GPR108	19	6730061	1	cg21986821	0.68506961	0.554659461	0.449381755	-0.235687855	2.19E-11	9.99E-10
UTR3	GPR114	16	57610170	1	cg05686118	0.6629469	0.565690439	0.473101764	-0.189845136	1.12E-08	1.32E-07
UTR3	GPR26	10	125453638/125448352/125454278/125454530	4	cg09616721/cg13557752/cg14039479/cg23545828	0.630450795	0.542710531	0.43110465	-0.199346145	5.99E-07	4.18E-06
UTR3	GPR4	19	46094030	1	cg15173319	0.588234765	0.509642228	0.432966645	-0.15526812	4.48E-06	2.45E-05
UTR3	GPR45	2	105859806	1	cg03997393	0.768511135	0.69813085	0.602309723	-0.166201412	6.02E-07	4.20E-06
UTR3	GPR78	4	8589313	1	cg02847185	0.71327113	0.584407756	0.449716941	-0.263554189	2.19E-08	2.34E-07
UTR3	GPR85	7	112720816	1	cg03177735	0.769539105	0.683404133	0.591156045	-0.17838306	2.99E-06	1.72E-05
UTR3	GPR98	5	90459841	1	cg07706111	0.775706465	0.662210856	0.578939364	-0.196767101	1.12E-08	1.31E-07
UTR3	GPX2	14	65405931	1	cg14947787	0.52773459	0.396178722	0.306150059	-0.221584531	2.68E-11	1.13E-09
UTR3	GRIA1	5	153192843	1	cg20668321	0.688436055	0.594384461	0.4562353	-0.232200755	1.20E-05	5.88E-05
UTR3	GRID1	10	87360396	1	cg27317356	0.428314085	0.330057683	0.243350536	-0.184963549	1.30E-12	1.47E-10
UTR3	GRIN2D	19	48947560/48947631/48947572/48947578/48948005	5	cg04988514/cg14594362/cg16875863/cg22242216/cg26362197	0.337209907	0.448778694	0.62741754	0.290207633	3.16E-09	4.47E-08
UTR3	GRM2	3	51752143	1	cg11727252	0.77556587	0.611487583	0.5510914	-0.22447447	3.17E-10	6.68E-09
UTR3	GRM5	11	88241594/88238963/88240817	3	cg02647408/cg04367107/cg25520422	0.774452558	0.708243442	0.593620864	-0.180831694	4.80E-06	2.60E-05

UTR3	GRM7	3	7755472/7742036/7773492/7782210	4	cg00586732/cg13054613/cg17219740/cg20936920	0.627616701	0.530606288	0.444872451	-0.18274425	1.13E-08	1.33E-07
UTR3	GRPEL2	5	148733517	1	cg06560087	0.595196705	0.461151144	0.385033809	-0.210162896	5.62E-12	4.11E-10
UTR3	GSC	14	95234822/95234658/95234826	3	cg05142765/cg13073773/cg23695707	0.226465008	0.258448255	0.462748754	0.236283746	3.67E-05	0.000159859
UTR3	GSTK1	7	142965938	1	cg03879613	0.520789815	0.430808678	0.352281395	-0.16850842	5.73E-11	1.94E-09
UTR3	GSX1	13	28368087	1	cg21851351	0.380350495	0.489700006	0.650913655	0.27056316	1.02E-09	1.73E-08
UTR3	GTSF1	12	54851386	1	cg20676413	0.57207408	0.44665725	0.336129405	-0.235944675	1.01E-10	2.80E-09
UTR3	GUCA2A	1	42628449	1	cg04701869	0.68453852	0.605533389	0.524841345	-0.159697175	2.95E-09	4.23E-08
UTR3	GYPC	2	127453782	1	cg11611448	0.79602001	0.590340178	0.401720773	-0.394299237	1.38E-13	3.88E-11
UTR3	GZMA	5	54406016	1	cg05648002	0.67577133	0.510632344	0.356100214	-0.319671116	4.36E-15	7.46E-12
UTR3	HAO1	20	7863971	1	cg19862235	0.385423555	0.303144617	0.219343218	-0.166080337	2.03E-10	4.76E-09
UTR3	HAPLN4	19	19367084/19366511	2	cg11781421/cg11971944	0.659483618	0.569136542	0.452436057	-0.207047561	5.36E-11	1.88E-09
UTR3	HARS	5	140053663	1	cg15715507	0.631400235	0.535646289	0.458476173	-0.172924062	9.55E-11	2.69E-09
UTR3	HAVCR2	5	156513860	1	cg18374914	0.633590665	0.542993178	0.428177764	-0.205412901	5.02E-08	4.78E-07
UTR3	HBP1	7	106842512/106842632	2	cg10840361/cg24556660	0.504900923	0.399028481	0.33026438	-0.174636543	4.83E-11	1.76E-09
UTR3	HBZ	16	204474	1	cg02279719	0.205046045	0.199966012	0.363854455	0.15880841	0.001984347	0.005624612
UTR3	HCK	20	30689432	1	cg06614284	0.453769085	0.319431767	0.227233327	-0.226535758	9.89E-15	7.46E-12
UTR3	HCN4	15	73613841	1	cg10942561	0.7729768	0.665639744	0.565309127	-0.207667673	5.57E-09	7.26E-08
UTR3	HECW1	7	43601539	1	cg00802510	0.61021526	0.473671694	0.347497114	-0.262718146	3.43E-11	1.37E-09
UTR3	HEMGN	9	100689349	1	cg14566819	0.512526425	0.415129344	0.284867109	-0.227659316	6.44E-11	2.08E-09
UTR3	HEPN1	11	124790365	1	cg22871233	0.86396429	0.800697256	0.701462732	-0.162501558	1.43E-05	6.87E-05
UTR3	HFE2	1	145417038	1	cg18369654	0.6426173	0.565228011	0.474320168	-0.168297132	2.99E-08	3.06E-07
UTR3	HGF	7	81381353/81372100	2	cg18944653/cg23269320	0.6702799	0.609693986	0.497570584	-0.172709316	7.72E-07	5.13E-06
UTR3	HLA-DOA	6	32974517/32974479/32974122/32972902/32972093/32973717/32972970/32972157/32973306/32972492/32972989	11	cg06788504/cg07699648/cg09132634/cg10297701/cg11527772/cg14671277/cg18824596/cg22118369/cg25535873/cg26175846/cg26306056	0.708494918	0.610925098	0.513618819	-0.194876099	2.59E-12	2.40E-10
UTR3	HMCN1	1	186159350	1	cg09411231	0.65509952	0.531562683	0.418838273	-0.236261247	3.94E-07	2.87E-06
UTR3	HMP19	5	173535352	1	cg19431566	0.71502649	0.6073294	0.4851254	-0.22990109	2.02E-09	3.06E-08

UTR3	HOXA3	7	27147206/27146262/27146237/27146430/27145972/27147513/27146445/27147084	8	cg01175550/cg01964852/cg03308399/cg05109569/cg12538674/cg13240116/cg14216068/cg27539480	0.563694833	0.630829877	0.763252151	0.199557317	2.68E-09	3.90E-08
UTR3	HOXB9	17	46699073/46699155/46698881/46698973/46698598	5	cg04917446/cg12057127/cg15117739/cg23245942/cg25078137	0.578931189	0.449031508	0.341099134	-0.237832055	5.81E-07	4.08E-06
UTR3	HOXC11	12	54369638/54369336/54369987/54369514	4	cg02384661/cg06708937/cg08857479/cg23395715	0.293750088	0.433826801	0.453710035	0.159959948	5.48E-06	2.92E-05
UTR3	HOXC13	12	54339653/54339167/54339288	3	cg03239552/cg04819096/cg05771518	0.283762935	0.348560644	0.437201491	0.153438556	3.78E-07	2.77E-06
UTR3	HOXC5	12	54428592	1	cg05987823	0.56356161	0.663961572	0.755658718	0.192097108	5.87E-12	4.19E-10
UTR3	HOXC9	12	54396990	1	cg22679316	0.511116905	0.679663483	0.681937423	0.170820518	0.000191655	0.000696907
UTR3	HOXD10	2	176984634	1	cg06005169	0.511829205	0.349056389	0.349526773	-0.162302432	0.000122586	0.000466878
UTR3	HPCAL4	1	40146660	1	cg00036110	0.528117505	0.388537689	0.295399841	-0.232717664	6.42E-12	4.43E-10
UTR3	HRASLS5	11	63230511	1	cg04999435	0.57101505	0.430893911	0.309875468	-0.261139582	9.24E-13	1.18E-10
UTR3	HRH3	20	60791033	1	cg00418735	0.629686335	0.545992772	0.464287068	-0.165399267	6.72E-07	4.54E-06
UTR3	HS3ST3A1	17	13399070	1	cg27260984	0.81198985	0.756759083	0.600862695	-0.211127155	3.19E-05	0.000140943
UTR3	HSP90AB1	6	44221403	1	cg19091981	0.5186358	0.377760811	0.301414827	-0.217220973	5.44E-11	1.89E-09
UTR3	HSPB8	12	119632544	1	cg16425829	0.53882094	0.452123811	0.358405686	-0.180415254	3.40E-07	2.52E-06
UTR3	HTN1	4	70923371	1	cg16270018	0.674315945	0.621824361	0.460186018	-0.214129927	2.25E-06	1.33E-05
UTR3	HTR3A	11	113860607	1	cg20178075	0.762484975	0.668723378	0.54763885	-0.214846125	1.37E-06	8.50E-06
UTR3	IAPP	12	21531643	1	cg12127290	0.651986365	0.57416675	0.460210405	-0.19177596	2.33E-07	1.82E-06
UTR3	ICOS	2	204824513	1	cg17751550	0.75262835	0.713681606	0.581573223	-0.171055127	0.000266233	0.000939639
UTR3	IFFO1	12	6649349/6648770/6648862	3	cg02410245/cg11053466/cg17229929	0.508890283	0.4246969	0.357492974	-0.151397309	1.04E-10	2.84E-09
UTR3	IFLTD1	12	25649090/25656194/25644389	3	cg06680863/cg11130955/cg21254753	0.520388233	0.352889517	0.271136409	-0.249251823	8.34E-12	5.32E-10
UTR3	IGFBP1	7	45932989	1	cg06200449	0.789571655	0.733281061	0.634668373	-0.154903282	0.000376833	0.00128597
UTR3	IGLL1	22	23915440/23915573	2	cg00805441/cg25778076	0.70402175	0.602328289	0.452638964	-0.251382786	8.56E-11	2.48E-09
UTR3	IGSF11	3	118620082	1	cg09856271	0.73168655	0.6545739	0.541285882	-0.190400668	4.88E-05	0.000205494
UTR3	IL17A	6	52054186	1	cg05884768	0.81988249	0.733557161	0.603682105	-0.216200385	7.48E-06	3.84E-05
UTR3	IL18R1	2	103014454	1	cg20629735	0.825654225	0.68068905	0.566933268	-0.258720957	7.29E-10	1.29E-08

UTR3	IL1RAP	3	190374982/190366926	2	cg19375583/cg20962955	0.61749919	0.496025183	0.3456051	-0.27189409	6.54E-11	2.10E-09
UTR3	IL22	12	68642433	1	cg03694077	0.825826325	0.751050461	0.549177714	-0.276648611	1.06E-07	9.15E-07
UTR3	IL24	1	207077339	1	cg22671342	0.80168361	0.731530422	0.557446668	-0.244236942	1.34E-06	8.36E-06
UTR3	IL2RB	22	37523151	1	cg24509815	0.38590457	0.282691722	0.227374441	-0.158530129	2.94E-13	5.74E-11
UTR3	IL31	12	122656878	1	cg23253739	0.70508545	0.604561411	0.554529514	-0.150555936	0.00051009	0.001685494
UTR3	IL4R	16	27375732	1	cg05903710	0.574244555	0.426972522	0.318751195	-0.25549336	8.20E-13	1.12E-10
UTR3	INCA1	17	4891687/4891542	2	cg11644815/cg14928057	0.20554477	0.318148389	0.358292545	0.152747775	0.000200325	0.000725078
UTR3	IQCA1	2	237233067	1	cg14035807	0.641619045	0.554918011	0.435973645	-0.2056454	5.06E-07	3.60E-06
UTR3	IRF4	6	408730	1	cg16478536	0.673091255	0.510591667	0.359100409	-0.313990846	7.00E-11	2.19E-09
UTR3	IRF7	11	612588	1	cg18477816	0.656342095	0.565704439	0.490570923	-0.165771172	2.62E-08	2.74E-07
UTR3	IRX1	5	3601357	1	cg09813610	0.24163855	0.313139379	0.440883736	0.199245186	6.83E-08	6.22E-07
UTR3	IRX4	5	1877906	1	cg17838420	0.489190195	0.564378506	0.698162859	0.208972664	1.26E-08	1.47E-07
UTR3	IRX5	16	54967786	1	cg07452217	0.430253355	0.511959644	0.725156582	0.294903227	3.46E-08	3.44E-07
UTR3	ITGAL	16	30533458	1	cg02888243	0.49989375	0.41460555	0.339206645	-0.160687105	2.38E-11	1.05E-09
UTR3	ITIH3	3	52842806	1	cg14556868	0.48263843	0.365942844	0.281247182	-0.201391248	6.31E-11	2.05E-09
UTR3	KBTBD3	11	105923175/105922059	2	cg26356242/cg26800786	0.810361953	0.740652183	0.647372557	-0.162989396	3.94E-11	1.51E-09
UTR3	KCMF1	2	85281128	1	cg07002540	0.549829665	0.414224472	0.336815795	-0.21301387	1.95E-11	9.31E-10
UTR3	KCNA1	12	5024586	1	cg22669120	0.456833075	0.318736772	0.231787859	-0.225045216	3.85E-13	6.67E-11
UTR3	KCNH5	14	63174831/63179119	2	cg16093173/cg20326674	0.458429415	0.343654139	0.26352435	-0.194905065	7.67E-12	5.03E-10
UTR3	KCNJ1	11	128708512	1	cg22721851	0.830890615	0.759916289	0.607408118	-0.223482497	3.78E-06	2.10E-05
UTR3	KCNJ16	17	68131572	1	cg17746031	0.634809215	0.554466894	0.469564955	-0.16524426	1.96E-06	1.18E-05
UTR3	KCNJ3	2	155711998	1	cg04227743	0.89152566	0.7555509	0.710135705	-0.181389955	2.44E-05	0.00011172
UTR3	KCNK15	20	43379501	1	cg07046101	0.40507243	0.27917015	0.210942386	-0.194130044	1.04E-14	7.46E-12
UTR3	KCNK9	8	140629559/140627494/140630180	3	cg05988964/cg14321373/cg24020826	0.743244908	0.668425339	0.57042465	-0.172820258	2.12E-07	1.67E-06
UTR3	KCNMB1	5	169805583	1	cg16425489	0.65433255	0.457229906	0.333462355	-0.320870195	1.81E-14	8.81E-12
UTR3	KCNT1	9	138684093	1	cg06677048	0.719923815	0.650782983	0.567577627	-0.152346188	7.06E-06	3.65E-05
UTR3	KCNU1	8	36793457	1	cg05749559	0.64251751	0.504223344	0.427103168	-0.215414342	1.88E-08	2.05E-07
UTR3	KCP	7	128530129/128516960	2	cg00139092/cg17511168	0.63436876	0.513724378	0.431646093	-0.202722667	3.95E-12	3.25E-10
UTR3	KCTD7	7	66105984	1	cg07522403	0.587084105	0.434704094	0.3336382	-0.253445905	6.12E-11	2.01E-09
UTR3	KEL	7	142638303	1	cg14498745	0.474326075	0.330781556	0.252260614	-0.222065461	6.18E-12	4.35E-10
UTR3	KHDC1	6	73951145	1	cg07388171	0.730898335	0.589748067	0.508045177	-0.222853158	1.01E-10	2.81E-09
UTR3	KHDC1L	6	73933428	1	cg06485892	0.55026717	0.409860056	0.295795727	-0.254471443	8.31E-15	7.46E-12
UTR3	KIAA0182	16	85708039	1	cg04120520	0.482592025	0.373766944	0.317987786	-0.164604239	9.17E-09	1.11E-07

UTR3	KIAA0247	14	70180751/70178771	2	cg01570265/cg09869130	0.535664348	0.441863025	0.364101916	-0.171562432	8.52E-12	5.34E-10
UTR3	KIAA0495	1	3659656/3659644/3659702/3652931	4	cg12417704/cg19903298/cg23984080/cg24361048	0.784425733	0.709943736	0.614968466	-0.169457267	3.19E-11	1.28E-09
UTR3	KIAA1486	2	226518461	1	cg03731588	0.786968595	0.56651525	0.408501177	-0.378467418	1.44E-12	1.54E-10
UTR3	KIDINS220	2	8870227	1	cg26426395	0.81599487	0.672126689	0.497986095	-0.318008775	8.96E-07	5.85E-06
UTR3	KIF2B	17	51902426	1	cg15844441	0.82809001	0.739533328	0.614003827	-0.214086183	1.34E-06	8.35E-06
UTR3	KIR3DP1	19	55309528	1	cg19380156	0.5419285	0.431051678	0.284627395	-0.257301105	6.82E-14	2.24E-11
UTR3	KIRREL2	19	36357700	1	cg22467594	0.7143387	0.628661617	0.469341177	-0.244997523	5.32E-08	5.03E-07
UTR3	KIRREL3	11	126294215/126301004	2	cg08527161/cg09737499	0.532321035	0.422800211	0.327277059	-0.205043976	9.92E-11	2.78E-09
UTR3	KLHL35	11	75133593	1	cg05327192	0.55257321	0.427094311	0.328218332	-0.224354878	3.02E-10	6.44E-09
UTR3	KLK15	19	51328577	1	cg07577731	0.60091852	0.451617806	0.351085564	-0.249832956	4.25E-13	7.12E-11
UTR3	KNG1	3	186462194/186460384	2	cg05769975/cg27376355	0.78525471	0.68198585	0.584153902	-0.201100808	1.95E-10	4.61E-09
UTR3	KRBA1	7	149431265	1	cg21460729	0.64276954	0.513637917	0.377351205	-0.265418335	1.01E-11	5.82E-10
UTR3	KRBA2	17	8272249	1	cg21463907	0.856332625	0.716364489	0.682361341	-0.173971284	3.23E-08	3.26E-07
UTR3	KREMEN2	16	3018104/3018262	2	cg09660145/cg26229224	0.52625122	0.409738944	0.312099295	-0.214151925	3.57E-12	3.04E-10
UTR3	KRT37	17	39577117	1	cg23388392	0.688627715	0.5754188	0.440495823	-0.248131892	5.34E-07	3.78E-06
UTR3	KRT5	12	52908478	1	cg00028929	0.58837834	0.537542189	0.428127436	-0.160250904	3.47E-05	0.000152125
UTR3	KRT74	12	52960529	1	cg10637292	0.47247762	0.368572783	0.286011905	-0.186465715	1.01E-11	5.82E-10
UTR3	KRT76	12	53162093	1	cg10284006	0.57304022	0.440255006	0.307917577	-0.265122643	4.58E-12	3.57E-10
UTR3	KRTAP10-1	21	45959142	1	cg17903229	0.46414623	0.348451517	0.299173286	-0.164972944	2.59E-07	2.00E-06
UTR3	KRTAP15-1	21	31813075	1	cg16812893	0.59362799	0.440570944	0.334187759	-0.259440231	3.58E-13	6.37E-11
UTR3	KRTAP4-12	17	39279735	1	cg10686758	0.445949885	0.330617539	0.281250095	-0.16469979	1.68E-09	2.62E-08
UTR3	KRTAP5-10	11	71277473	1	cg12285580	0.736933565	0.6149521	0.570261923	-0.166671642	5.79E-07	4.07E-06
UTR3	LAIR1	19	54866514	1	cg22545168	0.42214049	0.317314706	0.236689845	-0.185450645	2.69E-12	2.46E-10
UTR3	LAMP3	3	182840603	1	cg04119852	0.37661814	0.279030739	0.213225627	-0.163392513	2.16E-10	4.97E-09
UTR3	LASS3	15	100941674	1	cg27358936	0.647137555	0.489550944	0.428181414	-0.218956141	1.34E-08	1.55E-07
UTR3	LASS4	19	8327065	1	cg02887374	0.818685195	0.709593661	0.588506577	-0.230178618	1.68E-09	2.62E-08
UTR3	LAYN	11	111431381	1	cg03222632	0.65106607	0.547388567	0.441677491	-0.209388579	1.38E-08	1.58E-07
UTR3	LCE1C	1	152777382	1	cg16011217	0.63745746	0.484337322	0.364683118	-0.272774342	2.82E-13	5.74E-11
UTR3	LCE2A	1	152671707	1	cg16354520	0.54498256	0.379409567	0.252350123	-0.292632437	2.88E-14	1.22E-11
UTR3	LCN10	9	139633680/139632839	2	cg10434996/cg13735759	0.67393744	0.549721972	0.399317668	-0.274619772	1.39E-12	1.54E-10
UTR3	LDHA	11	18429080	1	cg17607330	0.576029415	0.501892622	0.423978745	-0.15205067	4.74E-11	1.75E-09
UTR3	LELP1	1	153177577	1	cg18140889	0.373157445	0.269905067	0.2148949	-0.158262545	5.93E-12	4.20E-10

UTR3	LGI3	8	22005501/22005602/22005545/22004881	4	cg07772400/cg17316784/cg18440818/cg21609813	0.575764743	0.477986722	0.376635127	-0.199129616	5.26E-10	9.86E-09
UTR3	LHX5	12	113900719/113900751	2	cg00721315/cg11586330	0.33528102	0.364200703	0.51968583	0.18440481	4.16E-05	0.000178475
UTR3	LIFR	5	38479736	1	cg04479876	0.527305235	0.408852022	0.330475505	-0.19682973	1.36E-11	7.21E-10
UTR3	LILRB1	19	55148602	1	cg08386867	0.86199604	0.773936728	0.631132927	-0.230863113	4.30E-06	2.36E-05
UTR3	LIM2	19	51883242	1	cg22222644	0.62113576	0.529555994	0.449817582	-0.171318178	9.65E-08	8.42E-07
UTR3	LMO1	11	8245962	1	cg21650086	0.53703783	0.440329533	0.356649732	-0.180388098	1.56E-11	7.97E-10
UTR3	LMOD2	7	123304073	1	cg25055892	0.613362185	0.545868833	0.404198091	-0.209164094	2.50E-07	1.93E-06
UTR3	LOC150786	2	132119242	1	cg16657479	0.60938099	0.577807828	0.453020636	-0.156360354	3.95E-07	2.88E-06
UTR3	LOC643677	13	103381760	1	cg09714424	0.78100912	0.702736772	0.545224695	-0.235784425	8.73E-06	4.42E-05
UTR3	LPA	6	160952713	1	cg10234069	0.49841431	0.404357139	0.329412682	-0.169001628	3.58E-10	7.42E-09
UTR3	LPIN2	18	2917509	1	cg20404260	0.461370655	0.377178289	0.296529491	-0.164841164	1.24E-10	3.22E-09
UTR3	LRAT	4	155672265	1	cg03473809	0.740864195	0.639801678	0.541596491	-0.199267704	2.66E-06	1.55E-05
UTR3	LRRC29	16	67241345	1	cg05708497	0.566115475	0.465077361	0.379649341	-0.186466134	4.14E-14	1.46E-11
UTR3	LRRC33	3	196388687	1	cg07364240	0.49921071	0.430527778	0.308356614	-0.190854096	3.28E-10	6.86E-09
UTR3	LRRN2	1	204586547	1	cg11820840	0.61777169	0.484634922	0.331329018	-0.286442672	1.18E-11	6.50E-10
UTR3	LRTM2	12	1943889/1945300	2	cg02505216/cg08982864	0.59561029	0.503415908	0.44432668	-0.15128361	8.93E-09	1.08E-07
UTR3	LSAMP	3	115524190/115524031	2	cg17080138/cg23075139	0.613490253	0.524736856	0.433114432	-0.180375821	1.05E-07	9.09E-07
UTR3	LST1	6	31556482	1	cg25664715	0.77500226	0.702124844	0.543219168	-0.231783092	1.10E-07	9.41E-07
UTR3	LTB4R	14	24785933	1	cg10065809	0.767093265	0.664340967	0.610628241	-0.156465024	1.63E-06	9.97E-06
UTR3	LVRN	5	115363186	1	cg20546296	0.54527649	0.419731511	0.299930923	-0.245345567	9.41E-14	2.83E-11
UTR3	LYNX1	8	143856487/143856122/143845999	3	cg00801134/cg04350571/cg16737416	0.694591328	0.621861394	0.537503721	-0.157087607	5.22E-12	3.92E-10
UTR3	LYPD2	8	143831693	1	cg05904795	0.74817241	0.695047106	0.586163268	-0.162009142	2.95E-06	1.70E-05
UTR3	LYRM7	5	130540986	1	cg13386215	0.637160565	0.5536973	0.429474041	-0.207686524	7.11E-09	8.91E-08
UTR3	LZTS1	8	20106574/20104343	2	cg01815167/cg25306432	0.734748063	0.674227611	0.582203536	-0.152544526	3.87E-07	2.83E-06
UTR3	MAP1D	2	172945274/172945144/172945328/172945383	4	cg08402365/cg12608433/cg14996143/cg24724664	0.497804547	0.594814398	0.716287405	0.218482858	3.96E-10	7.93E-09
UTR3	MAPK12	22	50691611	1	cg26822572	0.628493555	0.520990167	0.45627885	-0.172214705	3.21E-12	2.80E-10
UTR3	MAPK3	16	30126595	1	cg05329317	0.46969132	0.386940122	0.315332677	-0.154358643	4.79E-11	1.76E-09
UTR3	MASP1	3	186953108/186965021/186937654	3	cg01187883/cg04846432/cg25818109	0.52071538	0.375958817	0.258824919	-0.261890461	1.93E-13	4.80E-11
UTR3	MATK	19	3778090	1	cg27083614	0.602399625	0.50942625	0.408988173	-0.193411452	3.70E-12	3.09E-10

UTR3	MEF2C	5	88017891	1	cg05654946	0.828766405	0.708024689	0.678073877	-0.150692528	9.06E-05	0.00035773
UTR3	MEIS3	19	47908061	1	cg15558904	0.633779625	0.539107806	0.467652614	-0.166127011	1.08E-05	5.34E-05
UTR3	MET	7	116438184	1	cg25557280	0.54399849	0.432251344	0.316785177	-0.227213313	7.35E-13	1.04E-10
UTR3	MFAP5	12	8799841	1	cg19543296	0.88506451	0.718067278	0.608226045	-0.276838465	3.66E-08	3.62E-07
UTR3	MFI2	3	196746085	1	cg01149239	0.488799475	0.391729639	0.300261245	-0.18853823	1.88E-10	4.50E-09
UTR3	MFSD6	2	191365138	1	cg13411286	0.542286085	0.439693072	0.377160023	-0.165126062	1.55E-05	7.38E-05
UTR3	MGAT5B	17	74945219/74944970/74944924	3	cg06127313/cg09150928/cg22347696	0.662366517	0.530799541	0.432844685	-0.229521832	2.90E-13	5.74E-11
UTR3	MIB2	1	1565931/1565856	2	cg04882394/cg24368383	0.258028163	0.376816479	0.490183548	0.232155386	2.03E-05	9.47E-05
UTR3	MKRN1	7	140153199/140156225	2	cg00123057/cg02976323	0.422508305	0.318246756	0.257069768	-0.165438537	7.19E-11	2.22E-09
UTR3	MMAB	12	109992779	1	cg16497413	0.696704405	0.569127339	0.476494177	-0.220210228	3.06E-14	1.22E-11
UTR3	MMP16	8	89053427	1	cg02526439	0.60355949	0.436553261	0.351015227	-0.252544263	8.12E-12	5.24E-10
UTR3	MMP8	11	102583226	1	cg08747557	0.56603512	0.442314639	0.326475773	-0.239559347	2.07E-07	1.64E-06
UTR3	MNX1	7	156797833/156797988	2	cg08309529/cg25090972	0.569414385	0.675814633	0.750543568	0.181129183	1.01E-10	2.81E-09
UTR3	MPZL2	11	118124297/118125492	2	cg19259082/cg25249241	0.651841265	0.532394122	0.445373523	-0.206467742	2.01E-09	3.06E-08
UTR3	MRAP2	6	84799458/84800491	2	cg02129814/cg11508013	0.5156959	0.619757428	0.681245495	0.165549595	2.97E-08	3.05E-07
UTR3	MRAS	3	138121287	1	cg22907952	0.631739155	0.513400206	0.386792786	-0.244946369	4.58E-10	8.91E-09
UTR3	MRM1	17	34965294	1	cg06419964	0.53498495	0.436132839	0.350797773	-0.184187177	5.53E-06	2.94E-05
UTR3	MSC	8	72754469/72754272/72753888	3	cg11382963/cg12809365/cg24142603	0.461455853	0.532262231	0.665183986	0.203728133	2.75E-08	2.85E-07
UTR3	MSL1	17	38290796	1	cg06833098	0.60631191	0.469128356	0.373427018	-0.232884892	8.83E-11	2.52E-09
UTR3	MUC13	3	124625655	1	cg00446763	0.34866579	0.250599922	0.195040055	-0.153625735	2.88E-11	1.18E-09
UTR3	MUC15	11	26581996	1	cg22946774	0.63064958	0.488837672	0.397135886	-0.233513694	4.93E-07	3.53E-06
UTR3	MYBPC3	11	47353259	1	cg23614000	0.46599205	0.383527061	0.298314068	-0.167677982	2.64E-11	1.12E-09
UTR3	MYH13	17	10204251	1	cg26086902	0.63082069	0.559814528	0.413222164	-0.217598526	5.55E-07	3.91E-06
UTR3	MYH6	14	23851239	1	cg01593969	0.493490985	0.402909956	0.330160368	-0.163330617	2.29E-10	5.20E-09
UTR3	MYL1	2	211155085	1	cg17362927	0.435472705	0.363302111	0.266745609	-0.168727096	8.43E-10	1.46E-08
UTR3	MYL4	17	45301019	1	cg23956680	0.50885743	0.429796411	0.355980077	-0.152877353	8.19E-08	7.32E-07
UTR3	MYLK2	20	30421774	1	cg19609923	0.537614895	0.447220422	0.368827009	-0.168787886	1.39E-11	7.34E-10
UTR3	MYO18B	22	26425557	1	cg09835278	0.66473375	0.570003494	0.491124841	-0.173608909	3.30E-07	2.46E-06
UTR3	MYO1H	12	109885015	1	cg24961970	0.614580855	0.493862956	0.431715032	-0.182865823	7.41E-06	3.82E-05
UTR3	MYO3A	10	26501031/26501065	2	cg02232704/cg03876032	0.54473274	0.648933586	0.74744327	0.20271053	3.10E-09	4.41E-08
UTR3	MYOC	1	171604882	1	cg06363798	0.700266475	0.667095028	0.542422627	-0.157843848	4.28E-06	2.35E-05

UTR3	MYOCD	17	12661608/12669028	2	cg03891384/cg10974219	0.573616243	0.480293708	0.394314221	-0.179302021	1.55E-07	1.27E-06
UTR3	MYOD1	11	17743080/17743282/17743610/17743667	4	cg05694840/cg17862113/cg22344611/cg23897913	0.388446704	0.450126965	0.570429447	0.181982743	1.23E-06	7.70E-06
UTR3	MYOM2	8	2092909/2093220/2093137	3	cg11955491/cg14696926/cg20149337	0.549047315	0.455994181	0.399022966	-0.150024349	2.27E-10	5.19E-09
UTR3	NAT14	19	55998490/55998732/55998931	3	cg03645984/cg17379828/cg19820609	0.509887665	0.4367637	0.359526945	-0.15036072	1.45E-09	2.30E-08
UTR3	NBEA	13	36246384	1	cg10564626	0.805383465	0.728326589	0.639357973	-0.166025492	3.50E-05	0.000153238
UTR3	NBEAL2	3	47050950	1	cg02534363	0.636312055	0.744510589	0.811088686	0.174776631	4.95E-07	3.54E-06
UTR3	NCAM1	11	113135432/113147175	2	cg02323699/cg20857767	0.596555053	0.493600614	0.412931148	-0.183623905	6.31E-12	4.38E-10
UTR3	NCKAP1L	12	54936840	1	cg26787915	0.870193355	0.717033178	0.51087105	-0.359322305	1.35E-08	1.56E-07
UTR3	NDNL2	15	29560915	1	cg25937230	0.52342311	0.596867411	0.696257459	0.172834349	5.70E-11	1.93E-09
UTR3	NDRG4	16	58545502/58545770	2	cg04194840/cg26514728	0.739003685	0.576222456	0.491452386	-0.247551299	4.51E-08	4.35E-07
UTR3	NDUFC2	11	77780019	1	cg07101980	0.612419155	0.538828356	0.454918164	-0.157500991	1.71E-10	4.16E-09
UTR3	NECAB2	16	84036179	1	cg07939567	0.650346875	0.527651583	0.416469259	-0.233877616	6.05E-08	5.62E-07
UTR3	NEDD4L	18	56067462	1	cg18687085	0.387013135	0.284105344	0.219590327	-0.167422808	1.35E-10	3.42E-09
UTR3	NEFM	8	24776195	1	cg13387869	0.753254105	0.691442006	0.594257755	-0.15899635	0.000289895	0.001015777
UTR3	NETO1	18	70502513	1	cg17327268	0.732852515	0.653492567	0.571180855	-0.16167166	0.000412012	0.001391138
UTR3	NEU4	2	242758725	1	cg05195017	0.44223969	0.2641673	0.193258385	-0.248981305	3.70E-09	5.10E-08
UTR3	NEURL4	17	7219261/7219403/7219025/7218959/7219021/7219319	6	cg12282267/cg12391328/cg13248200/cg15358396/cg16340178/cg26963545	0.645424193	0.543898394	0.488587789	-0.156836404	1.34E-09	2.16E-08
UTR3	NEUROD4	12	55423208	1	cg20207414	0.65093147	0.531818239	0.394863114	-0.256068356	6.30E-12	4.38E-10
UTR3	NHP2L1	22	42070666	1	cg08303253	0.617097185	0.546627661	0.465557418	-0.151539767	7.66E-10	1.34E-08
UTR3	NID1	1	236140577	1	cg07549090	0.901418005	0.816503083	0.740975345	-0.16044266	2.91E-07	2.20E-06
UTR3	NINL	20	25433878	1	cg06913219	0.53436316	0.457514033	0.349839682	-0.184523478	5.35E-11	1.88E-09
UTR3	NIPA2	15	23005487	1	cg17122577	0.689716695	0.639749083	0.537368495	-0.1523482	1.51E-08	1.69E-07
UTR3	NKX2-1	14	36986363/36985711/36985905	3	cg07345734/cg12103037/cg13980454	0.400352755	0.497149002	0.626119574	0.225766819	1.66E-06	1.01E-05
UTR3	NKX2-2	20	21492558/21491781	2	cg03133868/cg18464649	0.549591918	0.627321031	0.736630991	0.187039073	4.38E-08	4.24E-07
UTR3	NKX2-4	20	21376484/21376337	2	cg07247419/cg13880779	0.22287616	0.28210296	0.433882499	0.211006339	6.95E-05	0.000281579

UTR3	NKX2-5	5	172659512/172659241/172659730/172660080	4	cg03219908/cg07798980/cg19923650/cg25916711	0.349399772	0.389401576	0.501256997	0.151857225	0.000172277	0.000633668
UTR3	NKX2-8	14	37049893/37049565/37049299	3	cg18396984/cg23676577/cg23705938	0.365940128	0.4481516	0.543273797	0.177333669	2.73E-07	2.09E-06
UTR3	NKX6-2	10	134598352	1	cg11174855	0.57658977	0.650269983	0.743038327	0.166448557	3.05E-08	3.11E-07
UTR3	NLRP14	11	7092719	1	cg07103673	0.606905665	0.396998006	0.296656455	-0.31024921	1.28E-12	1.47E-10
UTR3	NLRP2	19	55512301	1	cg26489750	0.71910822	0.63593745	0.546334686	-0.172773534	1.07E-05	5.27E-05
UTR3	NLRP4	19	56393163	1	cg18740359	0.632099595	0.509583089	0.4214362	-0.210663395	2.33E-10	5.25E-09
UTR3	NLRP9	19	56220025	1	cg27637326	0.70927275	0.586231	0.504763755	-0.204508995	2.29E-07	1.79E-06
UTR3	NMUR2	5	151771606	1	cg07429087	0.503317865	0.430939256	0.345237274	-0.158080591	0.00014169	0.000530463
UTR3	NNAT	20	36151184/36151683/36151338	3	cg06198069/cg13668823/cg22582721	0.82321326	0.708262914	0.5991139	-0.22409936	6.03E-10	1.11E-08
UTR3	NOB1	16	69776039/69776100	2	cg05006942/cg16378003	0.606749335	0.5201219	0.448899973	-0.157849362	3.24E-10	6.79E-09
UTR3	NOTCH3	19	15271010	1	cg18875435	0.60321667	0.473108178	0.385741727	-0.217474943	7.22E-09	9.01E-08
UTR3	NOVA1	14	26916082	1	cg19534149	0.763085575	0.691129233	0.599598582	-0.163486993	9.59E-06	4.80E-05
UTR3	NPM1	5	170833685	1	cg13701509	0.587882345	0.480606983	0.400088368	-0.187793977	5.14E-10	9.66E-09
UTR3	NPPB	1	11917550	1	cg07585427	0.3497406	0.246548289	0.196773175	-0.152967425	2.56E-10	5.64E-09
UTR3	NPR3	5	32786671	1	cg25996586	0.608480995	0.487308006	0.314877286	-0.293603709	1.28E-09	2.08E-08
UTR3	NPS	10	129350922	1	cg19321696	0.57320293	0.429348739	0.273219223	-0.299983707	3.72E-16	1.90E-12
UTR3	NPY	7	24331369	1	cg11892240	0.6573079	0.535913494	0.401827741	-0.255480159	6.61E-09	8.40E-08
UTR3	NR5A1	9	127244764	1	cg13476313	0.442325205	0.337871522	0.292016977	-0.150308228	7.19E-07	4.82E-06
UTR3	NT5C	17	73126499	1	cg11409350	0.70601368	0.589535583	0.501447109	-0.204566571	2.54E-09	3.71E-08
UTR3	NTNG1	1	108023482	1	cg26015416	0.41246796	0.535561772	0.598977932	0.186509972	2.97E-05	0.000132259
UTR3	NUP153	6	17615976	1	cg20062978	0.864297075	0.739027794	0.623285886	-0.241011189	1.42E-08	1.62E-07
UTR3	NUP43	6	150045504	1	cg00424166	0.76030968	0.601710944	0.534083577	-0.226226103	1.55E-08	1.74E-07
UTR3	NXPH1	7	8791686	1	cg21001050	0.685860495	0.569091922	0.422650391	-0.263210104	1.55E-09	2.44E-08
UTR3	NXPH3	17	47656736/47661136	2	cg15797422/cg20925233	0.572202223	0.485269781	0.40845543	-0.163746793	1.05E-11	5.93E-10
UTR3	OBP2B	9	136081048	1	cg14372265	0.7211495	0.629608339	0.5188209	-0.2023286	1.41E-08	1.61E-07
UTR3	OCA2	15	28000367	1	cg26388707	0.654425955	0.512326694	0.419094805	-0.23533115	8.27E-09	1.01E-07
UTR3	ODAM	4	71069600	1	cg19903738	0.462427625	0.386892889	0.291205091	-0.171222534	6.86E-11	2.16E-09
UTR3	ODF3	11	200083	1	cg25718632	0.842562685	0.740593006	0.674275168	-0.168287517	4.07E-08	3.98E-07
UTR3	ODF4	17	8249349	1	cg09820557	0.78970371	0.703308028	0.5911714	-0.19853231	2.36E-06	1.39E-05
UTR3	ODZ2	5	167690914	1	cg18013921	0.56186244	0.472463728	0.331416923	-0.230445517	3.55E-09	4.93E-08

UTR3	OLIG3	6	137814078/137813878/137814356/137813584/137813648	5	cg03602029/cg04508701/cg10071643/cg13560452/cg15980797	0.327801704	0.382949581	0.491321403	0.163519698	3.92E-05	0.000169378
UTR3	OPA3	19	46052863/46056723/46031516	3	cg02288770/cg18489009/cg22706147	0.554000105	0.439895244	0.344507345	-0.20949276	8.48E-11	2.47E-09
UTR3	OPRL1	20	62730763	1	cg15824741	0.692466295	0.574920233	0.449933345	-0.24253295	1.64E-10	4.02E-09
UTR3	OR56A1	11	6047917	1	cg12415917	0.654851195	0.520480289	0.384424768	-0.270426427	2.62E-11	1.12E-09
UTR3	OR7D2	19	9299162	1	cg04521512	0.765251955	0.637935622	0.57060125	-0.194650705	3.46E-07	2.55E-06
UTR3	OSBPL6	2	179260782	1	cg21182526	0.747212875	0.665576506	0.515118623	-0.232094252	2.03E-06	1.22E-05
UTR3	OTUD7A	15	31775406	1	cg01763090	0.269188475	0.324889211	0.454107795	0.18491932	4.19E-06	2.30E-05
UTR3	OTX1	2	63283806/63283939/63284132/63283967/63284066	5	cg03299042/cg07938743/cg10122865/cg21472506/cg23229261	0.191192393	0.19920873	0.347481962	0.15628957	0.005596475	0.01397339
UTR3	P2RX5	17	3576856/3593588/3576600	3	cg04221683/cg10373741/cg15763823	0.641832925	0.527703169	0.452319212	-0.189513713	8.13E-11	2.42E-09
UTR3	P2RX6	22	21381565	1	cg25418528	0.67351304	0.598043928	0.511755986	-0.161757054	2.98E-08	3.05E-07
UTR3	P2RY1	3	152555206	1	cg06809364	0.56570539	0.432520194	0.384786859	-0.180918531	8.95E-10	1.53E-08
UTR3	PABPC4L	4	135121050	1	cg15867829	0.66543919	0.512347756	0.362865264	-0.302573926	5.79E-12	4.19E-10
UTR3	PAEP	9	138458521	1	cg14532484	0.59506906	0.519429894	0.435230982	-0.159838078	8.05E-07	5.33E-06
UTR3	PANX3	11	124490178	1	cg10118227	0.796440585	0.600716367	0.471743309	-0.324697276	2.98E-13	5.74E-11
UTR3	PATL2	15	44958131	1	cg24799947	0.703710905	0.607091956	0.453196259	-0.250514646	1.12E-09	1.86E-08
UTR3	PAX8	2	113975820/113976143	2	cg09234973/cg25758828	0.597235155	0.465870833	0.359228177	-0.238006978	1.01E-11	5.82E-10
UTR3	PBRM1	3	52580707	1	cg05923681	0.608143815	0.484950683	0.41407915	-0.194064665	1.05E-07	9.09E-07
UTR3	PCDH10	4	134074369	1	cg23713176	0.27967513	0.377428472	0.523473918	0.243798788	9.08E-06	4.57E-05
UTR3	PCDH12	5	141324726	1	cg26837730	0.628185675	0.490360344	0.429882545	-0.19830313	1.19E-16	1.21E-12
UTR3	PCDH9	13	66878738	1	cg15067350	0.79748509	0.704192917	0.620273123	-0.177211967	9.17E-08	8.07E-07
UTR3	PCDHA1	5	140389945	1	cg25487047	0.494478275	0.411816828	0.316270364	-0.178207911	3.06E-06	1.74E-05
UTR3	PCDHA10	5	140389945	1	cg25487047	0.494478275	0.411816828	0.316270364	-0.178207911	3.06E-06	1.74E-05
UTR3	PCDHA11	5	140389945	1	cg25487047	0.494478275	0.411816828	0.316270364	-0.178207911	3.06E-06	1.74E-05
UTR3	PCDHA12	5	140389945	1	cg25487047	0.494478275	0.411816828	0.316270364	-0.178207911	3.06E-06	1.74E-05
UTR3	PCDHA13	5	140389945	1	cg25487047	0.494478275	0.411816828	0.316270364	-0.178207911	3.06E-06	1.74E-05
UTR3	PCDHA3	5	140389945	1	cg25487047	0.494478275	0.411816828	0.316270364	-0.178207911	3.06E-06	1.74E-05
UTR3	PCDHA4	5	140389945	1	cg25487047	0.494478275	0.411816828	0.316270364	-0.178207911	3.06E-06	1.74E-05
UTR3	PCDHA5	5	140389945	1	cg25487047	0.494478275	0.411816828	0.316270364	-0.178207911	3.06E-06	1.74E-05

UTR3	PCDHA6	5	140389945	1	cg25487047	0.494478275	0.411816828	0.316270364	-0.178207911	3.06E-06	1.74E-05
UTR3	PCDHA7	5	140389945	1	cg25487047	0.494478275	0.411816828	0.316270364	-0.178207911	3.06E-06	1.74E-05
UTR3	PCDHA8	5	140389945	1	cg25487047	0.494478275	0.411816828	0.316270364	-0.178207911	3.06E-06	1.74E-05
UTR3	PCDHA9	5	140389945	1	cg25487047	0.494478275	0.411816828	0.316270364	-0.178207911	3.06E-06	1.74E-05
UTR3	PCDHAC2	5	140389945	1	cg25487047	0.494478275	0.411816828	0.316270364	-0.178207911	3.06E-06	1.74E-05
UTR3	PCDHGA1	5	140892370/140891767/140892308	3	cg00104845/cg00283283/cg05494467	0.409542857	0.466664586	0.566230655	0.156687798	6.23E-07	4.25E-06
UTR3	PCDHGA10	5	140892370/140891767/140892308	3	cg00104845/cg00283283/cg05494467	0.409542857	0.466664586	0.566230655	0.156687798	6.23E-07	4.25E-06
UTR3	PCDHGA11	5	140892370/140891767/140892308	3	cg00104845/cg00283283/cg05494467	0.409542857	0.466664586	0.566230655	0.156687798	6.23E-07	4.25E-06
UTR3	PCDHGA12	5	140892370/140891767/140892308	3	cg00104845/cg00283283/cg05494467	0.409542857	0.466664586	0.566230655	0.156687798	6.23E-07	4.25E-06
UTR3	PCDHGA2	5	140892370/140891767/140892308	3	cg00104845/cg00283283/cg05494467	0.409542857	0.466664586	0.566230655	0.156687798	6.23E-07	4.25E-06
UTR3	PCDHGA3	5	140892370/140891767/140892308	3	cg00104845/cg00283283/cg05494467	0.409542857	0.466664586	0.566230655	0.156687798	6.23E-07	4.25E-06
UTR3	PCDHGA4	5	140892370/140891767/140892308	3	cg00104845/cg00283283/cg05494467	0.409542857	0.466664586	0.566230655	0.156687798	6.23E-07	4.25E-06
UTR3	PCDHGA5	5	140892370/140891767/140892308	3	cg00104845/cg00283283/cg05494467	0.409542857	0.466664586	0.566230655	0.156687798	6.23E-07	4.25E-06
UTR3	PCDHGA6	5	140892370/140891767/140892308	3	cg00104845/cg00283283/cg05494467	0.409542857	0.466664586	0.566230655	0.156687798	6.23E-07	4.25E-06
UTR3	PCDHGA7	5	140892370/140891767/140892308	3	cg00104845/cg00283283/cg05494467	0.409542857	0.466664586	0.566230655	0.156687798	6.23E-07	4.25E-06
UTR3	PCDHGA8	5	140892370/140891767/140892308	3	cg00104845/cg00283283/cg05494467	0.409542857	0.466664586	0.566230655	0.156687798	6.23E-07	4.25E-06
UTR3	PCDHGA9	5	140892370/140891767/140892308	3	cg00104845/cg00283283/cg05494467	0.409542857	0.466664586	0.566230655	0.156687798	6.23E-07	4.25E-06
UTR3	PCDHGB1	5	140892370/140891767/140892308	3	cg00104845/cg00283283/cg05494467	0.409542857	0.466664586	0.566230655	0.156687798	6.23E-07	4.25E-06
UTR3	PCDHGB2	5	140892370/140891767/140892308	3	cg00104845/cg00283283/cg05494467	0.409542857	0.466664586	0.566230655	0.156687798	6.23E-07	4.25E-06
UTR3	PCDHGB3	5	140892370/140891767/140892308	3	cg00104845/cg00283283/cg05494467	0.409542857	0.466664586	0.566230655	0.156687798	6.23E-07	4.25E-06

UTR3	PCDHGB4	5	140892370/140891767/140892308	3	cg00104845/cg00283283/cg05494467	0.409542857	0.466664586	0.566230655	0.156687798	6.23E-07	4.25E-06
UTR3	PCDHGB5	5	140892370/140891767/140892308	3	cg00104845/cg00283283/cg05494467	0.409542857	0.466664586	0.566230655	0.156687798	6.23E-07	4.25E-06
UTR3	PCDHGB6	5	140892370/140891767/140892308	3	cg00104845/cg00283283/cg05494467	0.409542857	0.466664586	0.566230655	0.156687798	6.23E-07	4.25E-06
UTR3	PCDHGB7	5	140892370/140891767/140892308	3	cg00104845/cg00283283/cg05494467	0.409542857	0.466664586	0.566230655	0.156687798	6.23E-07	4.25E-06
UTR3	PCDHGC3	5	140892370/140891767/140892308	3	cg00104845/cg00283283/cg05494467	0.409542857	0.466664586	0.566230655	0.156687798	6.23E-07	4.25E-06
UTR3	PCDHGC4	5	140892370/140891767/140892308	3	cg00104845/cg00283283/cg05494467	0.409542857	0.466664586	0.566230655	0.156687798	6.23E-07	4.25E-06
UTR3	PCDHGC5	5	140892370/140891767/140892308	3	cg00104845/cg00283283/cg05494467	0.409542857	0.466664586	0.566230655	0.156687798	6.23E-07	4.25E-06
UTR3	PDE1B	12	54972872	1	cg15937109	0.651512635	0.598687561	0.475665073	-0.175847562	2.06E-06	1.23E-05
UTR3	PDE2A	11	72288270	1	cg24987622	0.464078155	0.359016933	0.272701995	-0.19137616	5.63E-12	4.11E-10
UTR3	PDGFC	4	157682848	1	cg15957807	0.72117245	0.583535839	0.400430395	-0.320742055	1.66E-10	4.04E-09
UTR3	PDX1	13	28499045/28498878/28498956	3	cg00184376/cg15961466/cg26299169	0.32888554	0.421182144	0.664261111	0.335375571	2.65E-09	3.85E-08
UTR3	PEA15	1	160184732	1	cg25312122	0.548236545	0.4424557	0.346786545	-0.20145	6.15E-11	2.01E-09
UTR3	PGAP3	17	37827454/37828296	2	cg05869169/cg19758448	0.468601195	0.345282383	0.279021277	-0.189579918	1.89E-10	4.53E-09
UTR3	PGK2	6	49753577	1	cg02482438	0.543149635	0.39180955	0.281179145	-0.26197049	4.92E-13	7.73E-11
UTR3	PHLDA3	1	201437101/201437298/201435275	3	cg04055835/cg08639762/cg26120226	0.62117279	0.591942722	0.438409741	-0.182763049	9.20E-09	1.11E-07
UTR3	PHOX2B	4	41746614/41747568/41746697/41747092/41746947	5	cg01470356/cg08062484/cg13123927/cg20438306/cg24693341	0.402785513	0.466400484	0.572572745	0.169787232	9.88E-09	1.18E-07
UTR3	PHYHIPL	10	61007196	1	cg07625992	0.770272225	0.789121933	0.617386782	-0.152885443	0.001374368	0.004082704
UTR3	PICALM	11	85668951	1	cg24200175	0.56308173	0.451235422	0.368202236	-0.194879494	2.28E-10	5.19E-09
UTR3	PIK3CG	7	106545895	1	cg24232378	0.75614835	0.641287256	0.486666368	-0.269481982	1.14E-06	7.22E-06
UTR3	PIK3R5	17	8783908	1	cg04907571	0.649269875	0.523826706	0.40164175	-0.247628125	3.17E-13	5.79E-11
UTR3	PITX1	5	134364387/134363562/134363877/134363973/134363637/134363823/134363516	7	cg00396667/cg02037307/cg04223420/cg07274716/cg08206318/cg22827250/cg26509691	0.441969368	0.559504247	0.696047968	0.2540786	2.70E-12	2.46E-10

UTR3	PKD2L1	10	102048137	1	cg25155149	0.68594925	0.588848139	0.471665482	-0.214283768	2.19E-07	1.72E-06
UTR3	PKHD1	6	51481908	1	cg01776172	0.54562239	0.440443122	0.284132482	-0.261489908	1.38E-11	7.27E-10
UTR3	PLA2G10	16	14766421	1	cg27058221	0.435201765	0.332823744	0.248110259	-0.187091506	3.83E-10	7.84E-09
UTR3	PLA2G4C	19	48551504	1	cg12986110	0.585142475	0.494844211	0.340336014	-0.244806461	5.10E-09	6.71E-08
UTR3	PLA2G6	22	38507857/38508132	2	cg15944459/cg21007971	0.577785435	0.4830396	0.399261364	-0.178524071	1.52E-11	7.90E-10
UTR3	PLAGL1	6	144262297/144262258	2	cg01659632/cg13607311	0.758548888	0.658284081	0.568083425	-0.190465463	1.30E-08	1.51E-07
UTR3	PLCL1	2	199013270	1	cg09349723	0.66798426	0.4970543	0.349158882	-0.318825378	7.68E-12	5.03E-10
UTR3	PLEKHA4	19	49340489/49340574/49340765/49340593	4	cg04777726/cg16594139/cg19024700/cg26267310	0.376007511	0.484255928	0.568531901	0.19252439	1.14E-06	7.22E-06
UTR3	PLG	6	161174486	1	cg16140548	0.621746305	0.578185828	0.440008064	-0.181738241	8.52E-06	4.33E-05
UTR3	PLIN5	19	4523345	1	cg02392228	0.365934375	0.291733839	0.215653977	-0.150280398	5.01E-10	9.48E-09
UTR3	PLN	6	118881378	1	cg05270007	0.77820197	0.654715783	0.61692585	-0.16127612	8.70E-10	1.50E-08
UTR3	PNLIPRP1	10	118368634	1	cg08580014	0.509272975	0.425569067	0.349114059	-0.160158916	3.68E-08	3.64E-07
UTR3	PNMAL1	19	46971373/46969919	2	cg11808658/cg25734089	0.634225603	0.550149044	0.467346595	-0.166879007	2.68E-10	5.85E-09
UTR3	PNPLA2	11	824970	1	cg27238887	0.63647616	0.543338828	0.474115291	-0.162360869	4.40E-10	8.63E-09
UTR3	PNPLA5	22	44276013	1	cg09142166	0.586836275	0.446737322	0.343632986	-0.243203289	6.56E-10	1.19E-08
UTR3	POLR1B	2	113333716	1	cg02614999	0.60449706	0.453095972	0.372716527	-0.231780533	1.30E-11	6.97E-10
UTR3	POMC	2	25383940/25383851	2	cg10045137/cg23809645	0.695748515	0.623282033	0.538582745	-0.15716577	2.64E-08	2.75E-07
UTR3	POMGNT1	1	46654770	1	cg04089800	0.57835433	0.467037922	0.372877323	-0.205477007	2.13E-10	4.92E-09
UTR3	POU3F1	1	38509934/38510933/38510074/38510522/38510383/38510182	6	cg05785481/cg08599259/cg13149116/cg22821908/cg25488697/cg25609528	0.200020794	0.318494176	0.469416162	0.269395368	1.19E-09	1.96E-08
UTR3	POU4F2	4	147562073	1	cg21200539	0.37320488	0.541917483	0.697049414	0.323844534	7.77E-11	2.34E-09
UTR3	PPEF2	4	76781503	1	cg12289476	0.59898212	0.528208294	0.446943636	-0.152038484	5.99E-08	5.58E-07
UTR3	PPFIA4	1	203046885/203047830	2	cg15493970/cg22126171	0.589916293	0.485700164	0.3596239	-0.230292393	1.35E-10	3.42E-09
UTR3	PPP1R14A	19	38741928	1	cg11854183	0.494427475	0.420065828	0.343928073	-0.150499402	1.03E-08	1.24E-07
UTR3	PPP1R3A	7	113517552	1	cg17844121	0.81121758	0.759289222	0.646646318	-0.164571262	0.000190472	0.000693596
UTR3	PRAMEF4	1	12939180	1	cg22596861	0.64920295	0.580771983	0.473105245	-0.176097705	2.78E-09	4.00E-08
UTR3	PRDM14	8	70964123	1	cg14642381	0.4759041	0.3550472	0.272281173	-0.203622927	7.95E-13	1.10E-10

UTR3	PRDM16	1	3352986/3352435/3351615/3352830/3354412/3350440/3351504/3352257/3351390/3352884/3354467/3354433/3350558/3351106/3352544	15	cg00063748/cg01062116/cg01773689/cg02333588/cg02587392/cg04285727/cg06313241/cg08865559/cg09867208/cg11848748/cg12694058/cg13479148/cg13692649/cg22608265/cg23022057	0.799503338	0.722370423	0.642214878	-0.15728846	7.69E-11	2.33E-09
UTR3	PRDM6	5	122523277	1	cg01546873	0.70618941	0.616933733	0.551063273	-0.155126137	1.02E-05	5.08E-05
UTR3	PRDM7	16	90124400	1	cg00974523	0.520645375	0.439151361	0.346898436	-0.173746939	5.50E-08	5.18E-07
UTR3	PRDX5	11	64089278	1	cg01708924	0.572890435	0.479857544	0.392391168	-0.180499267	5.36E-10	9.96E-09
UTR3	PRIMA1	14	94186558	1	cg16577789	0.398663235	0.2911601	0.217738141	-0.180925094	6.37E-13	9.56E-11
UTR3	PRKAA2	1	57176315	1	cg26052885	0.707487685	0.612257206	0.490384005	-0.21710368	9.94E-07	6.41E-06
UTR3	PRLHR	10	120353499	1	cg24611996	0.747502085	0.652230139	0.59015565	-0.157346435	2.13E-07	1.68E-06
UTR3	PROCR	20	33765072	1	cg18649632	0.7443944	0.612527667	0.538257232	-0.206137168	7.27E-10	1.29E-08
UTR3	PROK2	3	71820909	1	cg22799510	0.64998684	0.542825339	0.403688441	-0.246298399	2.92E-08	2.99E-07
UTR3	PRSS27	16	2762569	1	cg04880804	0.68324347	0.613835367	0.482393786	-0.200849684	1.40E-08	1.61E-07
UTR3	PRSS35	6	84234463	1	cg06771958	0.696766565	0.580780872	0.545259277	-0.151507288	2.22E-06	1.32E-05
UTR3	PRSS8	16	31142991	1	cg05106869	0.660538735	0.566047767	0.486953523	-0.173585212	1.55E-09	2.43E-08
UTR3	PRTG	15	55911779/55909920	2	cg05779057/cg10666761	0.71053494	0.566879656	0.450721023	-0.259813917	1.32E-10	3.39E-09
UTR3	PSD2	5	139223811	1	cg08682153	0.446859455	0.3336159	0.245687932	-0.201171523	1.09E-07	9.36E-07
UTR3	PSG4	19	43697480	1	cg21775122	0.53125808	0.443652517	0.308219264	-0.223038816	1.31E-09	2.12E-08
UTR3	PSMG3	7	1607247/1607146	2	cg00294534/cg26035201	0.51074825	0.402125539	0.316182009	-0.194566241	1.66E-09	2.59E-08
UTR3	PTAFR	1	28474511	1	cg24492886	0.5502658	0.45833065	0.348911136	-0.201354664	2.46E-12	2.35E-10
UTR3	PTGER3	1	71418388/71472738/71354374	3	cg12603671/cg16823292/cg17888090	0.704299847	0.60999955	0.502212895	-0.202086951	1.95E-09	2.98E-08
UTR3	PTHLH	12	28115804	1	cg14210985	0.69959215	0.602720411	0.392690036	-0.306902114	8.16E-14	2.58E-11
UTR3	PTPLA	10	17632231	1	cg05341848	0.657783465	0.602039561	0.45174495	-0.206038515	3.70E-07	2.71E-06
UTR3	PTPN1	20	49200925	1	cg05093113	0.617901985	0.517374794	0.43718335	-0.180718635	1.40E-10	3.49E-09
UTR3	PTPN5	11	18750217	1	cg22404856	0.750027185	0.677279456	0.578705609	-0.171321576	2.40E-06	1.41E-05
UTR3	PTPRM	18	8406757	1	cg12412030	0.65769117	0.568988494	0.477869623	-0.179821547	2.75E-07	2.10E-06
UTR3	PTPRN	2	220154779	1	cg03398156	0.648929025	0.545501933	0.461875532	-0.187053493	1.36E-10	3.42E-09

UTR3	PURG	8	30888801/30853564/30853662/30888925	4	cg02638909/cg04756577/cg14212788/cg18907656	0.667355732	0.556125078	0.424619926	-0.242735806	9.67E-12	5.71E-10
UTR3	PXDN	2	1637068	1	cg10888111	0.8753676	0.59430805	0.438202605	-0.437164995	4.79E-15	7.46E-12
UTR3	PYDC1	16	31228059/31227411/31227884/31227497/31227515	5	cg00133595/cg03255128/cg07275305/cg09921810/cg18682103	0.161289494	0.214000818	0.341226319	0.179936826	4.72E-05	0.000199739
UTR3	RAB24	5	176728620	1	cg18128495	0.604185895	0.4956448	0.414519105	-0.18966679	3.88E-11	1.50E-09
UTR3	RAB6B	3	133545640	1	cg26466524	0.728497465	0.640354644	0.484425677	-0.244071788	1.46E-10	3.63E-09
UTR3	RABL5	7	100958103	1	cg02892258	0.83085088	0.759864572	0.64520455	-0.18564633	9.58E-07	6.18E-06
UTR3	RAG1	11	36599110	1	cg08285589	0.45408164	0.376545589	0.285275132	-0.168806508	5.99E-11	2.00E-09
UTR3	RASGEF1C	5	179528386	1	cg25951075	0.53506495	0.429875817	0.298831155	-0.236233795	4.29E-15	7.46E-12
UTR3	RASSF8	12	26232255/26222178	2	cg05990101/cg24674577	0.770448655	0.651536033	0.559494055	-0.2109546	4.12E-07	2.99E-06
UTR3	RAX	18	56935937/56935446/56935013/56935199/56934834	5	cg07099161/cg07835051/cg12083217/cg12605662/cg26775191	0.238080271	0.320172294	0.453806529	0.215726258	1.12E-06	7.11E-06
UTR3	RBM12B	8	94744382	1	cg16659470	0.567832935	0.47602625	0.408443918	-0.159389017	2.07E-08	2.23E-07
UTR3	RBMXL2	11	7112356	1	cg06176750	0.652383075	0.45420675	0.274610925	-0.37777215	7.01E-13	1.02E-10
UTR3	RBP7	1	10075991	1	cg09162909	0.51951704	0.43841815	0.35221425	-0.16730279	1.70E-09	2.64E-08
UTR3	REEP6	19	1497483	1	cg26921566	0.615986405	0.488313083	0.406022827	-0.209963578	4.04E-10	8.03E-09
UTR3	REPIN1	7	150070692	1	cg10018233	0.58904	0.474253811	0.427973573	-0.161066427	2.15E-06	1.28E-05
UTR3	RFX2	19	5994823/5993794	2	cg08023527/cg08675117	0.75964104	0.654970739	0.593638441	-0.166002599	1.99E-09	3.04E-08
UTR3	RGMA	15	93587701/93587978/93588137	3	cg05869601/cg15288779/cg22111818	0.717091573	0.620070083	0.505279123	-0.21181245	2.03E-11	9.58E-10
UTR3	RGS11	16	318900/318694/318717	3	cg08735550/cg09192940/cg09937849	0.597262583	0.484749997	0.390714911	-0.206547671	8.95E-11	2.55E-09
UTR3	RGS22	8	100974617	1	cg01487694	0.659987835	0.569470694	0.442498118	-0.217489717	1.72E-09	2.66E-08
UTR3	RGS4	1	163044231/163046549	2	cg07835293/cg11754482	0.813911165	0.794015733	0.648526645	-0.16538452	0.000543752	0.001781727
UTR3	RGS5	1	163113390	1	cg10780638	0.675836345	0.576672406	0.415553482	-0.260282863	2.21E-09	3.30E-08
UTR3	RGS7BP	5	63907206	1	cg26655294	0.42050641	0.321961467	0.251556368	-0.168950042	4.56E-12	3.57E-10
UTR3	RHO	3	129252584	1	cg26283480	0.781149465	0.684512406	0.568303577	-0.212845888	2.52E-08	2.65E-07
UTR3	RHOB	2	20648194/20648580/20647987	3	cg16258854/cg25432336/cg25840926	0.58166411	0.462718739	0.3753929	-0.20627121	2.76E-10	5.98E-09
UTR3	RHOJ	14	63757917	1	cg24186774	0.68639383	0.515430683	0.367702455	-0.318691375	2.11E-11	9.86E-10

UTR3	RIC3	11	8128361	1	cg03054541	0.794465585	0.738012383	0.564055195	-0.23041039	9.79E-06	4.89E-05
UTR3	RIMKLB	12	8926941	1	cg05837753	0.83553775	0.776733867	0.6599501	-0.17558765	0.000524541	0.001725981
UTR3	RIMS2	8	105264286	1	cg07664999	0.5521154	0.373942011	0.249843391	-0.302272009	1.13E-12	1.36E-10
UTR3	RNASE1	14	21269744	1	cg10197238	0.398412265	0.2997561	0.221241768	-0.177170497	5.05E-12	3.85E-10
UTR3	RNASE9	14	21024443	1	cg24162270	0.63467593	0.547864767	0.472713227	-0.161962703	1.60E-05	7.57E-05
UTR3	RNF165	18	44038721	1	cg11877105	0.50449169	0.416397011	0.334985809	-0.169505881	1.34E-10	3.42E-09
UTR3	RNF19B	1	33402454	1	cg22163472	0.667426105	0.562308978	0.4733737	-0.194052405	1.45E-12	1.54E-10
UTR3	RNF207	1	6279843	1	cg26611832	0.69299201	0.582450494	0.522792141	-0.170199869	2.41E-13	5.50E-11
UTR3	RPH3A	12	113335032	1	cg07230786	0.629925275	0.545767956	0.467143755	-0.16278152	1.69E-05	7.96E-05
UTR3	RPRML	17	45055836/45056008	2	cg20129313/cg22674412	0.069754249	0.064663847	0.228397823	0.158643574	0.001783286	0.005114359
UTR3	RPS6KA5	14	91338327	1	cg05018835	0.655207325	0.541948989	0.42542635	-0.229780975	2.79E-12	2.48E-10
UTR3	RRBP1	20	17594530	1	cg15815375	0.611846605	0.511149283	0.453310959	-0.158535646	2.79E-11	1.16E-09
UTR3	RSPO1	1	38077461	1	cg12413421	0.61851534	0.50195305	0.36762805	-0.25088729	2.82E-10	6.08E-09
UTR3	RSPO3	6	127517608	1	cg10997634	0.747242355	0.633884561	0.500761991	-0.246480364	3.34E-08	3.36E-07
UTR3	RUFY4	2	218955163	1	cg11417360	0.62479257	0.572725617	0.442641555	-0.182151015	3.02E-06	1.73E-05
UTR3	RWDD2A	6	83906123	1	cg19570102	0.69648424	0.61691295	0.531959577	-0.164524663	5.75E-10	1.06E-08
UTR3	RXFP2	13	32376637	1	cg09573371	0.61305552	0.558240289	0.386196736	-0.226858784	7.22E-09	9.01E-08
UTR3	RYR2	1	237996893	1	cg19708201	0.68210385	0.549119272	0.416295409	-0.265808441	1.21E-09	1.98E-08
UTR3	S100A5	1	153509635	1	cg09152105	0.704648195	0.575162133	0.485428073	-0.219220122	2.61E-13	5.56E-11
UTR3	S1PR3	9	91618456	1	cg14448588	0.680314215	0.5393611	0.436338945	-0.24397527	1.86E-10	4.47E-09
UTR3	SACS	13	23904116	1	cg24140343	0.86852772	0.730204833	0.484653191	-0.383874529	1.92E-09	2.94E-08
UTR3	SALL2	14	21989279	1	cg23140396	0.368932605	0.284841117	0.185871023	-0.183061582	3.99E-14	1.46E-11
UTR3	SAMD13	1	84815929	1	cg23925111	0.443203	0.3598256	0.272155945	-0.171047055	1.50E-08	1.69E-07
UTR3	SCAI	9	127710572	1	cg13478668	0.591154655	0.474107167	0.4245602	-0.166594455	4.26E-10	8.42E-09
UTR3	SCFD2	4	53740030	1	cg23814002	0.797317905	0.671405217	0.579690564	-0.217627341	6.86E-07	4.63E-06
UTR3	SCGB3A1	5	180017227/180017195	2	cg00480161/cg26270345	0.221320517	0.279332522	0.414866936	0.193546419	2.90E-05	0.000129366
UTR3	SCN11A	3	38888170	1	cg03825810	0.505481805	0.389644972	0.316395118	-0.189086687	5.42E-12	4.04E-10
UTR3	SCN2B	11	118036424	1	cg04563671	0.79325773	0.732087461	0.617685795	-0.175571935	4.88E-05	0.000205494
UTR3	SCNN1G	16	23227394	1	cg00452252	0.67246135	0.637818306	0.480935632	-0.191525718	9.57E-05	0.000375213
UTR3	SEC14L4	22	30885012	1	cg23383138	0.577382695	0.499358072	0.386374682	-0.191008013	9.91E-12	5.81E-10
UTR3	SELE	1	169693534	1	cg18229820	0.669687885	0.515382478	0.47013515	-0.199552735	3.93E-09	5.38E-08
UTR3	SELV	19	40009835	1	cg07052524	0.61414098	0.452529678	0.299807617	-0.314333363	2.94E-11	1.20E-09
UTR3	SEMA7A	15	74702956	1	cg19160053	0.728831935	0.661563978	0.568706859	-0.160125076	1.30E-05	6.30E-05
UTR3	SENP2	3	185348303	1	cg25634041	0.453694935	0.35693205	0.27001695	-0.183677985	4.79E-10	9.17E-09

UTR3	SENP7	3	101044447	1	cg27104413	0.69892936	0.62622235	0.547107736	-0.151821624	4.79E-06	2.59E-05
UTR3	SERPINB7	18	61472145	1	cg21354912	0.748700435	0.671357806	0.569024427	-0.179676008	5.21E-05	0.000218574
UTR3	SEZ6	17	27282064	1	cg15189764	0.65169493	0.545556856	0.497256227	-0.154438703	3.06E-07	2.31E-06
UTR3	SFRS3	6	36570759	1	cg21205713	0.75566191	0.696384189	0.600782382	-0.154879528	1.33E-05	6.42E-05
UTR3	SGCG	13	23898864	1	cg04591230	0.43407435	0.347811572	0.249075491	-0.184998859	1.25E-14	7.46E-12
UTR3	SH2D4B	10	82404135	1	cg27421810	0.861564615	0.752647417	0.632606123	-0.228958492	8.24E-09	1.01E-07
UTR3	SH2D7	15	78396318	1	cg13415795	0.595601055	0.678444606	0.756488127	0.160887072	1.36E-08	1.57E-07
UTR3	SH3GL3	15	84287328	1	cg15564896	0.564724095	0.475488361	0.405425577	-0.159298518	4.52E-06	2.46E-05
UTR3	SHISA3	4	42403471	1	cg11065575	0.509154205	0.393758089	0.311977418	-0.197176787	5.56E-11	1.91E-09
UTR3	SIAH3	13	46355841	1	cg26020695	0.47884079	0.394052389	0.316308291	-0.162532499	1.62E-10	3.98E-09
UTR3	SIDT2	11	117068115	1	cg11029270	0.61011441	0.517128906	0.445052723	-0.165061687	7.35E-09	9.13E-08
UTR3	SIGLEC5	19	52114809	1	cg03541909	0.628039235	0.496249844	0.3628437	-0.265195535	1.14E-10	3.02E-09
UTR3	SIGLEC8	19	51955472	1	cg26980954	0.60280769	0.465458428	0.333433918	-0.269373772	1.89E-11	9.08E-10
UTR3	SIM1	6	100836916	1	cg24544512	0.570116665	0.520291622	0.357163086	-0.212953579	4.74E-11	1.75E-09
UTR3	SIRPB1	20	1546019/1578550	2	cg26616780/cg27365103	0.631637693	0.490402319	0.349350298	-0.282287395	1.16E-14	7.46E-12
UTR3	SIRPD	20	1514929	1	cg00472837	0.70686384	0.541445233	0.422869095	-0.283994745	3.46E-12	2.97E-10
UTR3	SIRT6	19	4174165	1	cg01983454	0.60129668	0.482842233	0.388618195	-0.212678485	2.89E-11	1.18E-09
UTR3	SIX3	2	45172201/45172169/45172265/45172972	4	cg03717442/cg08696165/cg16509777/cg23245620	0.319616795	0.473606983	0.553462845	0.23384605	3.36E-05	0.00014787
UTR3	SIX4	14	61176368	1	cg13065475	0.74852735	0.639871172	0.55052355	-0.1980038	1.98E-06	1.19E-05
UTR3	SIX6	14	60978094/60978275/60978161	3	cg00859478/cg11126767/cg14507337	0.288065885	0.357652443	0.481920539	0.193854654	0.000199984	0.000724099
UTR3	SLC10A7	4	147177156	1	cg11875744	0.77400594	0.662061472	0.606630064	-0.167375876	7.33E-09	9.13E-08
UTR3	SLC11A2	12	51381135	1	cg20872692	0.576334385	0.461350233	0.378714923	-0.197619462	2.29E-10	5.20E-09
UTR3	SLC12A5	20	44687454/44686493/44687092/44687730	4	cg01460436/cg04403809/cg07060233/cg20003638	0.478972619	0.565761801	0.678626355	0.199653736	1.97E-07	1.57E-06
UTR3	SLC14A2	18	43262611	1	cg15090262	0.728026735	0.643834861	0.466792805	-0.26123393	6.49E-09	8.30E-08
UTR3	SLC15A1	13	99336246	1	cg02319149	0.8117625	0.736890311	0.639021964	-0.172740536	1.36E-07	1.14E-06
UTR3	SLC16A14	2	230899944	1	cg10277956	0.788265805	0.700207411	0.575269618	-0.212996187	6.57E-07	4.46E-06
UTR3	SLC16A7	12	60174815	1	cg10812247	0.517918565	0.393064122	0.317389909	-0.200528656	2.22E-11	1.00E-09
UTR3	SLC18A3	10	50820604	1	cg07637213	0.48770187	0.543272439	0.639293005	0.151591135	3.51E-09	4.88E-08
UTR3	SLC1A3	5	36686601/36608769	2	cg17839232/cg20646491	0.756741325	0.652204122	0.534740682	-0.222000643	2.10E-06	1.25E-05
UTR3	SLC22A17	14	23815798	1	cg08243827	0.606713025	0.535553039	0.456229541	-0.150483484	3.23E-08	3.26E-07

UTR3	SLC26A4	7	107357932	1	cg06535968	0.727286615	0.557762561	0.438293591	-0.288993024	6.73E-12	4.58E-10
UTR3	SLC29A4	7	5343176/5342805/5342893/5343586/5343660/5342933	6	cg01185093/cg16645977/cg17230640/cg21432302/cg24340453/cg27637597	0.716108154	0.580232582	0.468833491	-0.247274663	1.41E-13	3.88E-11
UTR3	SLC30A3	2	27478146	1	cg24594459	0.39501735	0.305603539	0.238974445	-0.156042905	4.12E-10	8.16E-09
UTR3	SLC30A8	8	118186764	1	cg05548393	0.742550435	0.703538072	0.572445318	-0.170105117	0.000955994	0.00295066
UTR3	SLC38A10	17	79218991/79224770	2	cg19030682/cg27529668	0.61604824	0.506077194	0.407657068	-0.208391172	1.94E-10	4.61E-09
UTR3	SLC4A10	2	162838364	1	cg06581825	0.539182305	0.419763311	0.330044405	-0.2091379	4.48E-11	1.68E-09
UTR3	SLC6A13	12	329792	1	cg13880565	0.79351325	0.754650639	0.63118115	-0.1623321	9.38E-06	4.71E-05
UTR3	SLC8A3	14	70512583	1	cg11012557	0.542311495	0.436973083	0.3500929	-0.192218595	2.42E-12	2.34E-10
UTR3	SLCO2B1	11	74916485	1	cg08597735	0.576106395	0.450473217	0.369914945	-0.20619145	1.16E-11	6.45E-10
UTR3	SLCO4C1	5	101570551	1	cg00630480	0.55854585	0.483318517	0.393163405	-0.165382445	2.16E-10	4.97E-09
UTR3	SLFN13	17	33763575	1	cg15577706	0.766537865	0.695474433	0.566322718	-0.200215147	5.82E-07	4.08E-06
UTR3	SLITRK1	13	84453280/84452994	2	cg04095724/cg18789918	0.368923134	0.482578065	0.615613181	0.246690047	4.51E-09	6.05E-08
UTR3	SNAP25	20	10286857	1	cg03569068	0.791877555	0.749251044	0.58704295	-0.204834605	9.56E-05	0.000374923
UTR3	SNCB	5	176047485	1	cg20960405	0.22901774	0.248642821	0.392675547	0.163657807	0.00282111	0.007648093
UTR3	SOD3	4	24802340/24802387	2	cg13096007/cg16170614	0.530277665	0.630692189	0.7558146	0.225536935	6.70E-10	1.21E-08
UTR3	SOHLH1	9	138585730	1	cg13708759	0.51684011	0.409615883	0.329370641	-0.187469469	2.52E-12	2.36E-10
UTR3	SOS1	2	39209066	1	cg26354721	0.837476245	0.748319156	0.622192973	-0.215283272	4.03E-07	2.93E-06
UTR3	SOX1	13	112724583/112723581/112723477/112723421/112725903/112725605/112723226/112724270/112724245/112724454/112724221	11	cg00073003/cg01236132/cg04047221/cg04865691/cg06488256/cg10245273/cg14842777/cg21385666/cg23668285/cg25476766/cg26151467	0.337810676	0.382575293	0.511167791	0.173357115	8.95E-06	4.52E-05
UTR3	SOX11	2	5837429/5837929/5836366/5835853/5838258/5837348/5836256/5836111/5837057/5834638/5836231/5840827/5837002/5836713/5836181	15	cg00078318/cg03594550/cg04043795/cg06173889/cg06830064/cg11716272/cg14776201/cg15034345/cg15989068/cg16330247/cg18897632/cg19958021/cg20927661/cg23668184/cg26659079	0.467691548	0.550396922	0.676593494	0.208901946	1.31E-10	3.36E-09

UTR3	SOX14	3	137484364	1	cg11630154	0.140841972	0.192145647	0.298468921	0.15762695	0.001052853	0.003224264
UTR3	SOX17	8	55372569	1	cg25923240	0.35625779	0.414395072	0.558034441	0.201776651	1.93E-05	9.05E-05
UTR3	SOX18	20	62679255	1	cg01355392	0.39983349	0.285235394	0.230503318	-0.169330172	2.20E-12	2.18E-10
UTR3	SOX5	12	23686149	1	cg27480819	0.66852252	0.55698255	0.41917475	-0.24934777	6.32E-08	5.83E-07
UTR3	SP7	12	53721673	1	cg16438602	0.70590904	0.598383122	0.513132005	-0.192777035	7.56E-07	5.06E-06
UTR3	SPATA22	17	3343601	1	cg24156983	0.620195515	0.482483867	0.379743605	-0.24045191	1.81E-13	4.62E-11
UTR3	SPATC1	8	145101998/145101671/145101833	3	cg00423068/cg10145449/cg21624980	0.760303952	0.626422785	0.537273252	-0.2230307	7.59E-12	5.03E-10
UTR3	SPERT	13	46288513	1	cg02134046	0.67989703	0.54656715	0.496922505	-0.182974525	6.19E-10	1.14E-08
UTR3	SPHK2	19	49133598/49133647/49133421	3	cg02821871/cg17865555/cg21519787	0.545157895	0.449919575	0.343863241	-0.201294654	5.04E-11	1.81E-09
UTR3	SPINK8	3	48348370	1	cg20734569	0.352014678	0.253312878	0.162893441	-0.189121236	5.73E-09	7.44E-08
UTR3	SPINLW1	20	44170643	1	cg16471362	0.59868732	0.49599725	0.421673323	-0.177013997	1.40E-07	1.16E-06
UTR3	SPINT1	15	41149780	1	cg04519327	0.64893348	0.520424639	0.415018523	-0.233914957	1.35E-10	3.42E-09
UTR3	SPOCK1	5	136314223	1	cg22612642	0.445545335	0.296661761	0.209797259	-0.235748076	2.56E-13	5.56E-11
UTR3	SPRR1B	1	153005121	1	cg02919982	0.62953565	0.587374011	0.460579927	-0.168955723	2.34E-05	0.000107533
UTR3	SPRR3	1	152976275	1	cg20671415	0.649201945	0.525186383	0.439022373	-0.210179572	1.94E-09	2.97E-08
UTR3	SPRY2	13	80910763	1	cg00185066	0.58327754	0.492414972	0.37908555	-0.20419199	1.10E-09	1.82E-08
UTR3	SPTA1	1	158581040	1	cg07048608	0.759245825	0.651806039	0.53471755	-0.224528275	1.77E-07	1.43E-06
UTR3	SRL	16	4241254	1	cg26591162	0.60786137	0.45936075	0.367236064	-0.240625306	7.08E-12	4.78E-10
UTR3	SRRM3	7	75916480	1	cg22519356	0.64539845	0.577578344	0.492013514	-0.153384936	9.14E-08	8.05E-07
UTR3	SRRM4	12	119595495	1	cg00700239	0.57300562	0.428104583	0.306999132	-0.266006488	3.00E-14	1.22E-11
UTR3	SSH3	11	67079729	1	cg05295023	0.650486465	0.528351228	0.443207214	-0.207279251	3.79E-11	1.48E-09
UTR3	SSPO	7	149530984	1	cg22537597	0.504356695	0.408212011	0.315808609	-0.188548086	9.60E-12	5.69E-10
UTR3	SSTR3	22	37602542	1	cg14757173	0.62910349	0.518654156	0.445990623	-0.183112867	3.69E-10	7.59E-09
UTR3	ST6GALNAC5	1	77529073	1	cg19369529	0.639776235	0.564809994	0.459363145	-0.18041309	1.23E-05	6.02E-05
UTR3	ST8SIA2	15	93010191	1	cg09389826	0.784419935	0.693391206	0.6143188	-0.170101135	4.05E-06	2.23E-05
UTR3	STAG3	7	99811980	1	cg09293122	0.52275311	0.423486178	0.348278182	-0.174474928	1.49E-10	3.69E-09
UTR3	STAT5B	17	40352975	1	cg21821388	0.519414055	0.431267328	0.357459095	-0.16195496	4.71E-10	9.07E-09
UTR3	SUSD2	22	24584744	1	cg12473943	0.74540461	0.620133011	0.5533158	-0.19208881	0.000102329	0.00039835
UTR3	SUSD5	3	33194171	1	cg17216807	0.80492961	0.739345583	0.619362732	-0.185566878	1.03E-05	5.11E-05
UTR3	SV2A	1	149875698	1	cg25277573	0.760071305	0.699602906	0.608282505	-0.1517888	2.52E-07	1.95E-06
UTR3	SYCE1	10	135367706	1	cg09480264	0.57934362	0.467635794	0.333957527	-0.245386093	1.44E-09	2.30E-08
UTR3	SYPL2	1	110024289	1	cg23091122	0.549180325	0.415777306	0.300507227	-0.248673098	1.23E-12	1.45E-10
UTR3	SYT10	12	33528782	1	cg03354810	0.765560605	0.750931206	0.593719	-0.171841605	9.86E-05	0.000385728

UTR3	TBC1D21	15	74181456	1	cg24361079	0.64517505	0.551851917	0.423762436	-0.221412614	5.15E-07	3.66E-06
UTR3	TBPL1	6	134308414	1	cg18634443	0.40678245	0.304089117	0.240981486	-0.165800964	1.29E-11	6.96E-10
UTR3	TBX2	17	59485894	1	cg27027242	0.4185352	0.515738144	0.615647514	0.197112314	1.08E-09	1.80E-08
UTR3	TCEA2	20	62703578	1	cg03044302	0.769019855	0.650798256	0.501712395	-0.26730746	3.46E-08	3.45E-07
UTR3	TCF15	20	584773	1	cg02939767	0.5923177	0.34688905	0.227455723	-0.364861977	1.97E-14	9.15E-12
UTR3	TCFL5	20	61473305	1	cg19907964	0.50225213	0.451843783	0.329425664	-0.172826466	6.32E-08	5.83E-07
UTR3	TCHHL1	1	152056869	1	cg05388137	0.729877155	0.627862744	0.4650036	-0.264873555	5.02E-09	6.61E-08
UTR3	TCL1B	14	96158915	1	cg01018864	0.53212782	0.4344983	0.366392832	-0.165734988	5.51E-09	7.19E-08
UTR3	TCTEX1D1	1	67244062	1	cg10625686	0.60068317	0.54014705	0.365761418	-0.234921752	1.30E-08	1.51E-07
UTR3	TEX14	17	56634222	1	cg09327586	0.512008345	0.47315485	0.353673445	-0.1583349	8.82E-05	0.000348746
UTR3	TFAP2A	6	10398601/10398494	2	cg04389897/cg04406454	0.264736835	0.398388925	0.597863175	0.33312634	3.99E-08	3.91E-07
UTR3	TFEB	6	41652303	1	cg00216570	0.66345188	0.539049656	0.467721564	-0.195730316	7.05E-11	2.20E-09
UTR3	TFF3	21	43732208	1	cg24057615	0.58838744	0.512388339	0.396972695	-0.191414745	2.27E-10	5.19E-09
UTR3	TGFB1	19	41836950	1	cg04614483	0.5305956	0.442997583	0.375235268	-0.155360332	2.74E-09	3.95E-08
UTR3	TH	11	2185376	1	cg26966083	0.630299115	0.516691461	0.435687214	-0.194611901	1.59E-06	9.75E-06
UTR3	THSD7A	7	11412105	1	cg22383292	0.696334765	0.547514617	0.398341836	-0.297992929	4.88E-10	9.30E-09
UTR3	TIMP2	17	76850266/76850277/76849181/76850256/76850052/76850198/76849093	7	cg01502428/cg01761758/cg02969624/cg15010903/cg16347361/cg20761853/cg27377777	0.566670981	0.440428696	0.336183938	-0.230487043	2.43E-12	2.34E-10
UTR3	TLL1	4	167022415	1	cg20743881	0.549580585	0.443231167	0.371497918	-0.178082667	1.01E-06	6.52E-06
UTR3	TLL2	10	98126436	1	cg12801391	0.5492307	0.452564328	0.31710405	-0.23212665	7.62E-12	5.03E-10
UTR3	TLX2	2	74743664/74743839/74743437	3	cg03998104/cg14877896/cg21185289	0.505954352	0.599778024	0.702160758	0.196206406	2.91E-10	6.24E-09
UTR3	TM2D3	15	102182534	1	cg23049226	0.466942855	0.354317061	0.269929377	-0.197013478	3.04E-12	2.67E-10
UTR3	TM4SF1	3	149086884	1	cg00244111	0.665859265	0.57247115	0.494130677	-0.171728588	9.31E-10	1.59E-08
UTR3	TM4SF4	3	149220486	1	cg16954508	0.77612159	0.6388159	0.496524495	-0.279597095	3.29E-10	6.86E-09
UTR3	TMBIM1	2	219139048	1	cg23569941	0.60695414	0.4826953	0.398226127	-0.208728013	1.78E-11	8.63E-10
UTR3	TMC2	20	2622020	1	cg24848351	0.55852129	0.461947433	0.403025523	-0.155495767	1.31E-05	6.36E-05
UTR3	TMC8	17	76138514	1	cg03596178	0.54725217	0.439526756	0.372952455	-0.174299715	7.30E-09	9.09E-08
UTR3	TMCO5A	15	38243575	1	cg21629384	0.720481815	0.61869355	0.491287436	-0.229194379	3.22E-07	2.41E-06
UTR3	TMEM130	7	98444410	1	cg00833441	0.60206195	0.531283794	0.402695609	-0.199366341	1.94E-08	2.10E-07
UTR3	TMEM134	11	67231878	1	cg07303548	0.610202535	0.454796911	0.32130235	-0.288900185	7.37E-13	1.04E-10
UTR3	TMEM155	4	122680946	1	cg16298202	0.79069576	0.724691622	0.556162114	-0.234533646	1.91E-07	1.53E-06
UTR3	TMEM167B	1	109637162/109639152	2	cg05708073/cg07906745	0.592705195	0.506202158	0.418934834	-0.173770361	1.63E-11	8.16E-10

UTR3	TMEM169	2	216966036	1	cg07660494	0.64651965	0.498833772	0.392625705	-0.253893945	1.01E-12	1.25E-10
UTR3	TMEM188	16	50069454	1	cg02254879	0.569548405	0.442836317	0.343770914	-0.225777491	2.51E-11	1.09E-09
UTR3	TMEM198	2	220415201/220414726	2	cg03830936/cg06390613	0.667061355	0.585782472	0.485566095	-0.18149526	2.41E-09	3.57E-08
UTR3	TMEM215	9	32789031	1	cg14279416	0.73243203	0.587047889	0.425299618	-0.307132412	2.65E-08	2.76E-07
UTR3	TMEM26	10	63167726	1	cg18237739	0.69384678	0.580288889	0.424146123	-0.269700657	3.08E-10	6.56E-09
UTR3	TMEM30A	6	75965559	1	cg11887083	0.743614665	0.573787589	0.550888273	-0.192726392	1.68E-09	2.62E-08
UTR3	TMEM33	4	41961749	1	cg18811916	0.681134775	0.563145528	0.472287555	-0.20884722	1.61E-11	8.11E-10
UTR3	TMEM40	3	12776107	1	cg20504993	0.562520945	0.486443283	0.348545273	-0.213975672	5.34E-09	7.01E-08
UTR3	TMEM74	8	109796144	1	cg20189036	0.784316265	0.634820772	0.494780027	-0.289536238	8.09E-07	5.35E-06
UTR3	TMEM95	17	7260326	1	cg14288903	0.699415005	0.625491756	0.519510509	-0.179904496	6.03E-09	7.77E-08
UTR3	TMPRSS11A	4	68776652	1	cg26673722	0.59197598	0.424100256	0.300292691	-0.291683289	8.31E-12	5.32E-10
UTR3	TMX2	11	57508416	1	cg13305186	0.3831515	0.28445415	0.226755355	-0.156396145	4.93E-11	1.78E-09
UTR3	TNFRSF4	1	1146777/1146903	2	cg09503311/cg20544186	0.8360821	0.697237156	0.600139464	-0.235942636	3.56E-15	7.46E-12
UTR3	TNFRSF8	1	12204096/12203571/12203751	3	cg06853894/cg09247175/cg24492058	0.31410533	0.218858667	0.15418385	-0.15992148	6.85E-09	8.67E-08
UTR3	TNFSF12	17	7460690	1	cg13829089	0.54887827	0.442739328	0.355842995	-0.193035275	6.46E-09	8.27E-08
UTR3	TNIP3	4	122053520	1	cg20146506	0.72038511	0.602405844	0.503328627	-0.217056483	2.81E-08	2.90E-07
UTR3	TNNC1	3	52485216	1	cg12349422	0.66803184	0.543307239	0.40992175	-0.25811009	4.33E-12	3.48E-10
UTR3	TOB1	17	48939676	1	cg13119853	0.55585124	0.453988844	0.36159225	-0.19425899	3.86E-10	7.89E-09
UTR3	TPH2	12	72426134	1	cg19530293	0.74046647	0.6805497	0.549066959	-0.191399511	5.77E-06	3.06E-05
UTR3	TPRG1	3	189040394	1	cg21772826	0.597525175	0.487125761	0.396945659	-0.200579516	1.43E-07	1.18E-06
UTR3	TPRX1	19	48304772	1	cg15198344	0.735594995	0.64541555	0.577616291	-0.157978704	6.29E-06	3.30E-05
UTR3	TPSD1	16	1308385	1	cg09639264	0.540632595	0.449670111	0.376932359	-0.163700236	8.65E-11	2.49E-09
UTR3	TPSG1	16	1271741	1	cg03553819	0.68871236	0.539859583	0.398461127	-0.290251233	1.44E-12	1.54E-10
UTR3	TRERF1	6	42194691	1	cg05264377	0.526854895	0.4192109	0.336164036	-0.190690859	4.76E-10	9.14E-09
UTR3	TRH	3	129696735	1	cg15109144	0.771078315	0.706492244	0.6070087	-0.164069615	9.89E-05	0.000386649
UTR3	TRIM71	3	32933637	1	cg17980786	0.568945865	0.451060394	0.374609041	-0.194336824	1.10E-07	9.42E-07
UTR3	TRIM9	14	51463130/51443494	2	cg14837480/cg23574924	0.772739693	0.697459689	0.565641925	-0.207097768	2.40E-06	1.41E-05
UTR3	TRIML2	4	189012434	1	cg21529532	0.72318953	0.6361933	0.564212345	-0.158977185	2.03E-06	1.22E-05
UTR3	TRPC6	11	101323578	1	cg04554505	0.596598235	0.52333025	0.414612886	-0.181985349	3.86E-05	0.000167114
UTR3	TRPM1	15	31293713	1	cg21826258	0.754437845	0.6462158	0.562919814	-0.191518031	2.30E-08	2.45E-07
UTR3	TRPS1	8	116422845	1	cg18430465	0.635776765	0.479868744	0.343752909	-0.292023856	1.05E-14	7.46E-12
UTR3	TRPV4	12	110220970	1	cg01201138	0.569929145	0.485829911	0.415730073	-0.154199072	1.25E-08	1.46E-07
UTR3	TRPV6	7	142569053	1	cg08835464	0.811856095	0.661889144	0.468637341	-0.343218754	1.05E-10	2.85E-09

UTR3	TSC22D1	13	45008447/45008526	2	cg03097389/cg17600231	0.80057919	0.620083178	0.527408509	-0.273170681	7.36E-09	9.14E-08
UTR3	TSHZ2	20	52029943/52036204	2	cg07333305/cg16499956	0.57347573	0.496967528	0.388373289	-0.185102441	1.81E-07	1.46E-06
UTR3	TSPYL5	8	98288616/98286872/98288091	3	cg18458131/cg19653425/cg24512297	0.773494593	0.720154239	0.61222225	-0.161272343	1.08E-06	6.90E-06
UTR3	TTC1	5	159492309	1	cg14483162	0.471588795	0.370422428	0.290598891	-0.180989904	1.09E-09	1.82E-08
UTR3	TTC33	5	40712757	1	cg12455300	0.73764873	0.696041678	0.575416309	-0.162232421	3.26E-07	2.44E-06
UTR3	TTYH3	7	2701567/2701419/2701802/2702851/2701641/2701636/2704368	7	cg00035074/cg01370759/cg11411865/cg13676996/cg17337874/cg21452471/cg26282283	0.659954321	0.527734879	0.446803592	-0.213150729	1.02E-11	5.83E-10
UTR3	TULP1	6	35465845/35466014	2	cg04293930/cg26319579	0.44197854	0.496148411	0.594946123	0.152967583	2.30E-05	0.000105872
UTR3	TWIST1	7	19155806/19155785/19156086	3	cg04043591/cg13588800/cg20498685	0.194545309	0.287736599	0.410565572	0.216020263	0.000226444	0.000812037
UTR3	TXNL4A	18	77733105	1	cg26501139	0.598339155	0.456598328	0.394632645	-0.20370651	1.05E-10	2.85E-09
UTR3	UBASH3B	11	122684258	1	cg20249071	0.547086215	0.458773394	0.342684191	-0.204402024	2.10E-11	9.82E-10
UTR3	UBL4B	1	110656508	1	cg09826973	0.640216855	0.561730622	0.474523291	-0.165693564	3.40E-05	0.000149324
UTR3	UBQLN3	11	5528669	1	cg24832498	0.562355705	0.470788528	0.36332985	-0.199025855	1.10E-10	2.92E-09
UTR3	UCN	2	27530363	1	cg01454215	0.20537035	0.239092546	0.373542048	0.168171698	0.004350954	0.011206205
UTR3	UGT3A1	5	35954233	1	cg17210837	0.50550368	0.341676628	0.250504545	-0.254999135	1.21E-13	3.52E-11
UTR3	UNC119	17	26873903	1	cg12118708	0.706433345	0.628256311	0.541395768	-0.165037577	3.22E-07	2.41E-06
UTR3	UNC45B	17	33514885	1	cg00100703	0.51650594	0.406674367	0.308260473	-0.208245467	1.03E-10	2.83E-09
UTR3	UNC5A	5	176307766/176307743/176307836	3	cg00513735/cg19742888/cg25389791	0.78115073	0.648408567	0.518298045	-0.262852685	3.65E-11	1.44E-09
UTR3	UNC5C	4	96090330	1	cg03024478	0.629980515	0.467550089	0.362151282	-0.267829233	9.17E-12	5.57E-10
UTR3	UNC80	2	210863849	1	cg06379340	0.68075015	0.568068217	0.450864395	-0.229885755	2.21E-11	1.00E-09
UTR3	USH2A	1	216348212/215796556	2	cg00972761/cg14964274	0.671505375	0.496377683	0.362080127	-0.309425248	6.22E-10	1.14E-08
UTR3	USP48	1	22005650	1	cg15090198	0.62297305	0.498565689	0.422413009	-0.200560041	1.01E-09	1.72E-08
UTR3	VAV1	19	6857265	1	cg27388680	0.70747611	0.6149019	0.555391132	-0.152084978	0.000110957	0.000428063
UTR3	VCAM1	1	101204337	1	cg22454770	0.65708404	0.505680606	0.373783205	-0.283300835	2.48E-13	5.50E-11
UTR3	VDR	12	48237752/48235729	2	cg14854850/cg21843272	0.562221915	0.445455533	0.358124227	-0.204097688	2.46E-10	5.47E-09
UTR3	VGLL2	6	117594700	1	cg14664726	0.690373685	0.633957139	0.471073259	-0.219300426	1.18E-07	1.00E-06
UTR3	VIL1	2	219314121	1	cg18753162	0.754912775	0.612269017	0.521003364	-0.233909411	8.15E-11	2.42E-09
UTR3	VMO1	17	4688683/4688629/4689165	3	cg06419850/cg25913172/cg26605335	0.40557508	0.463299383	0.599079557	0.193504477	2.71E-09	3.91E-08

UTR3	VPS13B	8	100888033	1	cg14537031	0.601118815	0.4807892	0.432743027	-0.168375788	2.30E-08	2.44E-07
UTR3	VPS37D	7	73085754	1	cg15881597	0.58335113	0.448357089	0.393737009	-0.189614121	3.69E-12	3.09E-10
UTR3	VRK2	2	58386677	1	cg00869533	0.72031767	0.625852844	0.554663109	-0.165654561	4.67E-10	9.02E-09
UTR3	VSIG10	12	118502338	1	cg04910149	0.496299095	0.413441511	0.320461118	-0.175837977	3.94E-10	7.93E-09
UTR3	VSTM2L	20	36573019/36572741	2	cg08580545/cg17071589	0.71026816	0.62739535	0.549306468	-0.160961692	2.58E-06	1.51E-05
UTR3	VTCN1	1	117689356	1	cg27055365	0.560425635	0.498825528	0.402554636	-0.157870999	1.80E-07	1.45E-06
UTR3	VWCE	11	61025815	1	cg02566157	0.8282695	0.743458428	0.651661486	-0.176608014	3.53E-06	1.98E-05
UTR3	WDFY4	10	50190845	1	cg13623664	0.834008095	0.705666794	0.573100736	-0.260907359	9.33E-09	1.13E-07
UTR3	WDR64	1	241964727	1	cg26823144	0.801700945	0.701557506	0.651523436	-0.150177509	0.000342985	0.001183919
UTR3	WFDC10B	20	44313418	1	cg11783356	0.47643962	0.399614017	0.313590391	-0.162849229	1.96E-07	1.57E-06
UTR3	WNT7B	22	46318272	1	cg04197904	0.719541385	0.618853678	0.521607541	-0.197933844	7.51E-08	6.77E-07
UTR3	WNT9A	1	228109169	1	cg24312489	0.48887143	0.38302945	0.3364342	-0.15243723	8.31E-11	2.44E-09
UTR3	WSCD2	12	108643267	1	cg00736201	0.56974541	0.461176661	0.382201986	-0.187543424	3.52E-11	1.40E-09
UTR3	WWTR1	3	149238478	1	cg02013148	0.426941755	0.329094628	0.271385755	-0.155556	1.28E-11	6.96E-10
UTR3	ZAP70	2	98356101	1	cg14247635	0.595653965	0.483205139	0.388860077	-0.206793888	4.49E-13	7.38E-11
UTR3	ZAR1	4	48496404	1	cg22289785	0.576019555	0.424303894	0.342240323	-0.233779232	8.36E-14	2.58E-11
UTR3	ZC3H3	8	144519832	1	cg13169667	0.545866815	0.445731672	0.362743305	-0.18312351	1.55E-11	7.97E-10
UTR3	ZDHHC11	5	801195	1	cg12184012	0.812694455	0.725978833	0.651990073	-0.160704382	3.21E-06	1.82E-05
UTR3	ZDHHC22	14	77600014	1	cg09824615	0.53622097	0.449662428	0.373386786	-0.162834184	4.48E-09	6.01E-08
UTR3	ZEB2	2	145143754/145146928	2	cg11432960/cg15345847	0.685163138	0.601809033	0.518865961	-0.166297176	4.94E-10	9.39E-09
UTR3	ZFC3H1	12	72004098	1	cg14303478	0.68915096	0.575068444	0.529261055	-0.159889905	9.91E-06	4.94E-05
UTR3	ZFR2	19	3852027/3805860/3804563	3	cg00860425/cg10000139/cg24001611	0.700147518	0.607497522	0.488412027	-0.211735491	2.89E-10	6.22E-09
UTR3	ZHX2	8	123981935/123967055	2	cg23822407/ch.8.2442961R	0.48183225	0.388922167	0.301774691	-0.180057559	5.31E-10	9.92E-09
UTR3	ZIC4	3	147106010/147106635/147106561/147105190/147106489/147105899/147106208	7	cg00334063/cg04079301/cg04556126/cg07406191/cg08889797/cg12976081/cg22614239	0.297585039	0.351290028	0.463658664	0.166073626	3.34E-06	1.88E-05
UTR3	ZIC5	13	100616256/100617055	2	cg02909158/cg20382146	0.506346295	0.423631033	0.355191593	-0.151154702	2.28E-07	1.79E-06
UTR3	ZIK1	19	58102673/58102769	2	cg22981926/ch.19.1899367R	0.409624147	0.322419353	0.230786553	-0.178837594	1.70E-14	8.65E-12

UTR3	ZMIZ1	10	81075467/81073777/81075012/81075478/81075067/81075241/81075165/81075492	8	cg03707696/cg04256907/cg10313149/cg13676902/cg15733068/cg19666787/cg24752964/cg26300785	0.552831145	0.451407439	0.395318809	-0.157512336	8.11E-09	9.95E-08
UTR3	ZNF143	11	9549286	1	cg03668763	0.535881705	0.451317167	0.349655895	-0.18622581	1.12E-08	1.31E-07
UTR3	ZNF295	21	43406941	1	cg24950067	0.72708904	0.603511872	0.495914045	-0.231174995	1.38E-10	3.47E-09
UTR3	ZNF304	19	57870634	1	cg27366815	0.80288294	0.655238128	0.481440823	-0.321442117	2.22E-09	3.31E-08
UTR3	ZNF365	10	64160521/64239663/64431533	3	cg11685898/cg18632745/cg27076160	0.74041805	0.660595711	0.569022259	-0.171395791	8.50E-07	5.57E-06
UTR3	ZNF385B	2	180307728	1	cg17100158	0.765192235	0.710885594	0.552142264	-0.213049971	2.67E-06	1.55E-05
UTR3	ZNF395	8	28205705	1	cg21330896	0.52058207	0.627062844	0.688363945	0.167781875	5.27E-06	2.82E-05
UTR3	ZNF438	10	31133829	1	cg16124562	0.68614135	0.560721561	0.483246173	-0.202895177	2.13E-12	2.13E-10
UTR3	ZNF470	19	57091383	1	cg09287295	0.713085055	0.532754622	0.3893565	-0.323728555	2.82E-09	4.04E-08
UTR3	ZNF536	19	31048117	1	cg01094891	0.63516337	0.543370939	0.46431855	-0.17084482	7.07E-05	0.000286093
UTR3	ZNF546	19	40522655	1	cg15568892	0.852869015	0.8065091	0.588532277	-0.264336738	6.14E-08	5.70E-07
UTR3	ZNF549	19	58051563	1	cg21647626	0.854609165	0.706595533	0.489310455	-0.36529871	7.99E-11	2.40E-09
UTR3	ZNF589	3	48310544/48310897/48310616/48311815	4	cg01458495/cg05070273/cg11307417/cg22533419	0.708955989	0.602984474	0.548630463	-0.160325526	2.66E-11	1.13E-09
UTR3	ZNF599	19	35249217	1	cg00019275	0.628919475	0.501900294	0.400766259	-0.228153216	2.64E-08	2.75E-07
UTR3	ZNF648	1	182025232/182024689	2	cg02494593/cg06361127	0.741990248	0.66705845	0.572993318	-0.168996929	4.63E-10	8.98E-09
UTR3	ZSCAN5A	19	56732932	1	cg17767567	0.730605915	0.561204944	0.473755191	-0.256850724	7.03E-09	8.84E-08
UTR3	ZSCAN5B	19	56701088	1	cg08835915	0.60365558	0.512207739	0.406907455	-0.196748125	4.53E-12	3.57E-10
UTR3	ZSWIM1	20	44513768	1	cg13780718	0.41916749	0.281100889	0.237797105	-0.181370385	4.88E-08	4.67E-07
UTR3	ZSWIM7	17	15880379	1	cg19815271	0.383900145	0.259403756	0.210193373	-0.173706772	6.36E-11	2.06E-09

Table S4. Differentially methylated regions (DMRs) between high grade adenoma (HGA) and low grade adenoma (LGA)

Location	Gene	CHR	MAPINFO	Number of sites	Designed Probes	Normal	LGA	HGA	Different β	P value	Adjusted P value
TSS1500	ADCYAP1R1	7	31091245/3109182<br>5/31091813/31091<br>718	4	cg00256593/cg17617<br>930/cg20165074/cg2<br>7076139	0.121036297	0.162474221	0.386411279	0.223937058	2.62E-05	4.70E-03
TSS1500	AICDA	12	8766467/8766941	2	cg10465202/cg27562<br>311	0.756507785	0.647370642	0.488450232	-0.15892041	4.36E-05	4.88E-03
TSS1500	AQP5	12	50354962/5035499<br>8/50353951/50354<br>840	4	cg03020208/cg08266<br>366/cg19220825/cg2<br>6328335	0.300126293	0.318647856	0.47726052	0.158612664	3.17E-05	4.78E-03
TSS1500	BAT2L2	1	171454351/171453<br>943	2	cg00526163/cg10143<br>731	0.536697805	0.424156072	0.264537845	-0.159618227	6.35E-06	3.48E-03
TSS1500	BOLL	2	198651498/198650<br>880/198650985/19<br>8651076/19865060<br>3/198650752/1986<br>50987/198651576/<br>198651303/198651	10	cg00926400/cg03774<br>803/cg05783139/cg0<br>7495363/cg07734961<br>/cg10124201/cg1335<br>6896/cg16002818/cg<br>17560323/cg[illegible]	0.410338441	0.486456147	0.65089284	0.164436693	3.04E-04	7.65E-03
TSS1500	BTG4	11	111383808/111384<br>124/111384048/11<br>1384223/11138360<br>3/111383288/1113<br>83576/111383515/<br>111383624/111383<br>889/111383653	11	cg01192900/cg03281<br>603/cg08827001/cg0<br>9148270/cg13767940<br>/cg18515591/cg2188<br>1253/cg22879515/cg<br>23211240/cg2404107<br>8/cg26561785	0.197607313	0.243806879	0.403806738	0.159999859	9.05E-04	1.12E-02
TSS1500	C16orf73	16	1922425/1922370/<br>1922400/1922577	4	cg01422476/cg03467<br>725/cg08734647/cg2<br>7105890	0.315759298	0.350905211	0.511049441	0.16014423	5.75E-03	3.08E-02
TSS1500	C17orf102	17	32907132/3290763<br>9/32906622/32907<br>388/32907002/329<br>07705/32907636/3<br>2906991/32907731	9	cg00196787/cg03442<br>425/cg04790084/cg0<br>6459443/cg06557358<br>/cg10451253/cg2174<br>5612/cg23791505/cg	0.239433865	0.29955866	0.451134977	0.151576317	3.50E-04	8.00E-03
TSS1500	C20orf186	20	31668141/3166844<br>8	2	cg03442592/cg05890<br>956	0.749375975	0.709791322	0.547756936	-0.162034386	1.17E-03	1.27E-02
TSS1500	CCNA1	13	37005566/3700507<br>4/37004617/37006<br>063/37004721/370<br>05570/37005299/3<br>7006127/37005115<br>/37004812/370055<br>57/37004857/3700<br>5582/37004715/37<br>005501/37005489/<br>37005107/3700473<br>7/37004601/37005<br>035/37004853/370<br>06107/37006116/3<br>7004536/37004940<br>/37004747/370047<br>87/37004990/3700<br>5289/37005273/37	34	cg00282249/cg00370<br>179/cg01040523/cg0<br>2478448/cg03871460<br>/cg05089090/cg0512<br>0331/cg05137358/cg<br>06260815/cg0796212<br>8/cg08215918/cg082<br>96176/cg08676975/c<br>g11023392/cg115136<br>37/cg12417071/cg13<br>435392/cg13711394/<br>cg14089714/cg14499<br>563/cg17215831/cg1<br>8348647/cg18948722<br>/cg19866195/cg2158<br>7066/cg21754388/cg<br>23831735/cg2415405	0.336303202	0.466972428	0.62634131	0.159368882	8.40E-05	5.37E-03

TSS1500	CDH10	5	24645487/24645147	2	cg01058368/cg14730815	0.408848933	0.414785036	0.56782963	0.153044593	4.37E-05	4.88E-03
TSS1500	CDH19	18	64271503/64271469	2	cg13853450/cg15728909	0.747354578	0.626766872	0.47666073	-0.150106143	6.34E-06	3.48E-03
TSS1500	CDX2	13	28544814/28543520/28544592/28544760	4	cg01499197/cg02055963/cg07984133/cg16201038	0.443346519	0.280014939	0.431752602	0.151737663	1.08E-02	4.67E-02
TSS1500	CFHR4	1	196855851	1	cg11793499	0.78677561	0.6862508	0.510848623	-0.175402177	5.50E-06	3.48E-03
TSS1500	CHL1	3	238161/238392/238318/238048	4	cg00903242/cg01295518/cg04001014/cg11165881	0.319082383	0.331529224	0.563607323	0.232078099	1.40E-05	4.32E-03
TSS1500	CHST15	10	125806958/125807504/125807047	3	cg06034255/cg09595290/cg16449489	0.719218533	0.531967692	0.380170368	-0.151797323	2.01E-04	6.73E-03
TSS1500	CHST5	16	75569527/75569852/75569459	3	cg03517083/cg03858460/cg27232078	0.60959881	0.589231594	0.396092173	-0.193139422	3.14E-05	4.78E-03
TSS1500	CLIP4	2	29337946/29338077/29337984/29337988/29338100	5	cg01777397/cg06333058/cg07285673/cg09695033/cg25737323	0.288922638	0.419389181	0.595234157	0.175844976	3.57E-04	8.05E-03
TSS1500	CMTM2	16	66613053/66613096/66612955	3	cg01683883/cg08032924/cg09849405	0.270345705	0.400159912	0.603544868	0.203384956	6.82E-04	9.94E-03
TSS1500	CMTM3	16	66638320/66637927/66637919/66638293/66638210	5	cg00355909/cg01434608/cg16335762/cg23297477/cg26658728	0.173318059	0.470402982	0.643669665	0.173266684	6.63E-03	3.36E-02
TSS1500	CNRIP1	2	68548060/68547655	2	cg08174244/cg21216118	0.70666885	0.545216989	0.394946445	-0.150270543	4.68E-05	5.07E-03
TSS1500	DBC1	9	122132103/122132178/122132052/122132379/122132054/122132261	6	cg03625109/cg13685314/cg14445814/cg14470803/cg24818566/cg25935911	0.370155049	0.44613189	0.623822819	0.177690929	1.79E-04	6.42E-03
TSS1500	DNAH11	7	21582131/21582502/21582444	3	cg00016360/cg06270661/cg09447578	0.350061853	0.346009398	0.53481313	0.188803732	5.16E-05	5.15E-03
TSS1500	DPP10	2	115919006/115919366/115918745/115199477/11591900	5	cg03753331/cg04617755/cg12666279/cg16146353/cg21678377	0.291421825	0.302930935	0.468908183	0.165977248	3.72E-03	2.34E-02
TSS1500	DPY19L2	12	64062724/64062564/64063071	3	cg00845942/cg04455164/cg16971885	0.21846244	0.313160749	0.504428995	0.191268246	9.16E-05	0.005371466
TSS1500	EBF1	5	158527817/158527707/158527702/158527982/158527469/158528040/158527573/158527463/158527458	9	cg00251610/cg01135780/cg05056120/cg05774756/cg07607126/cg15963563/cg16126280/cg16317459/cg26664797	0.140235014	0.223235776	0.402209818	0.178974042	8.25E-04	1.07E-02
TSS1500	EFHA2	8	16884225/16884197/16884364	3	cg03919781/cg21653826/cg23218760	0.179144757	0.265004541	0.489821532	0.224816992	3.23E-04	7.85E-03
TSS1500	EID3	12	104697193/104697220/104697224	3	cg01857475/cg09884423/cg10572274	0.247947366	0.421289865	0.607087922	0.185798057	1.93E-03	1.61E-02
TSS1500	EOMES	3	27764473/27764660/27765232/27765283/27764810/27764816	6	cg01714811/cg02694810/cg06014401/cg15540820/cg16971668/cg22383888	0.27810847	0.367711204	0.52080839	0.153097186	4.66E-04	8.63E-03
TSS1500	EPHA7	6	94129611/94129627/94129582/94129636/94129697	5	cg02168857/cg06740629/cg08001895/cg21552709/cg22263131	0.186532958	0.25859245	0.4403509	0.18175845	5.41E-03	2.97E-02

TSS1500	EVC	4	5712581/5712676/5712592	3	cg03881544/cg15305511/cg26875219	0.215831775	0.277033162	0.449048542	0.17201538	3.14E-03	2.12E-02
TSS1500	EVC2	4	5710767/5712581/5711301/5712676/5710761/5712592	6	cg03278643/cg03881544/cg09558315/cg15305511/cg17242664/cg26875219	0.215831775	0.277033162	0.449048542	0.17201538	3.14E-03	2.12E-02
TSS1500	FADS2	11	61595494/61594997/61594965/61595485	4	cg05698098/cg06781209/cg21709803/cg23760165	0.159061191	0.182416376	0.386897281	0.204480905	2.97E-04	7.62E-03
TSS1500	FAM150A	8	53478624/53478454/53478706	3	cg05965392/cg17162024/cg26021627	0.23671131	0.229776212	0.400791592	0.17101538	2.64E-03	1.94E-02
TSS1500	FAM155A	13	108520039/108520481/108519752/108520391/108520189/108520252/108520827/108520945/108520566/108519	10	cg03141482/cg05905988/cg06446668/cg06833978/cg07034928/cg13582072/cg18588768/cg22302985/cg24567434/cg2508565	0.25719381	0.285549024	0.451570455	0.166021431	1.29E-03	1.33E-02
TSS1500	FBN2	5	127874478/127874587/127873980/127874463/127875163/127874466/1278	7	cg01939274/cg05686497/cg06993118/cg11965976/cg20708842/cg25532099/cg2722	0.233092783	0.267891083	0.458669824	0.190778741	1.01E-03	1.19E-02
TSS1500	FGF5	4	81186795/81187125/81187011/81187198/81186901	5	cg05894124/cg10218799/cg12087412/cg18638496/cg21477508	0.166089672	0.211075347	0.431028501	0.219953154	7.39E-06	3.51E-03
TSS1500	FLJ33360	5	6338418/6338017/6338529/6338794	4	cg02202411/cg02328035/cg17491560/cg22778952	0.7507706	0.694758528	0.477771682	-0.216986846	1.11E-06	3.48E-03
TSS1500	FLRT2	14	85996202/85995726/85996260/85995859/85996274/85995655/85996101	7	cg02305242/cg10406690/cg12080751/cg12155356/cg18845692/cg23934295/cg2665	0.218268422	0.301255612	0.465920785	0.164665173	3.39E-04	7.95E-03
TSS1500	FUT9	6	96463185/96463246/96462814	3	cg01837719/cg22782271/cg23997263	0.38322732	0.452863714	0.671393257	0.218529543	2.09E-05	4.47E-03
TSS1500	FZD10	12	130645645/130646461/130646022/130646129/130646256/130646370/130646497/130645566	8	cg02214745/cg02291403/cg09048530/cg17537177/cg18209212/cg20058043/cg21386611/cg22813950	0.29197381	0.332261222	0.541514312	0.209253089	3.31E-05	4.78E-03
TSS1500	GALNT13	2	154728210/154727485/154727907/154728133/154728062/154727925/1547	7	cg07547000/cg08042220/cg12398124/cg12424785/cg14635767/cg21998983/cg2698	0.398731636	0.398701913	0.550407905	0.151705991	5.39E-05	5.20E-03
TSS1500	GALNTL6	4	172734276/172734347/172734031/172733566/172733760/172734266	6	cg03304610/cg05295006/cg05678100/cg09892710/cg17896776/cg26814276	0.318865286	0.409996313	0.609992463	0.19999615	5.33E-05	5.20E-03
TSS1500	GFRA2	8	21647596/21646662/21647026/21646704	4	cg03330485/cg03453638/cg05021638/cg14072903	0.238076203	0.245361925	0.397112514	0.15175059	6.59E-04	9.84E-03
TSS1500	GNAO1	16	56224782/56224504/56224901/56224627/56224793	5	cg00866976/cg04156464/cg06728579/cg08613597/cg10273340	0.121886658	0.203293909	0.404969255	0.201675346	4.78E-04	8.72E-03

TSS1500	GPR27	3	71802533/71802608/71802420/71802594	4	cg03604840/cg04408595/cg22631938/cg22823146	0.20313981	0.221876609	0.396107026	0.174230416	5.03E-05	5.12E-03
TSS1500	GRIN2A	16	10277505/10276375/10276984/10277317/10276383/10276674/10277254/10276580/10276799/10276600/10277017	11	cg00036299/cg00287829/cg00518386/cg01722994/cg06758670/cg07642043/cg08621611/cg09239744/cg16368442/cg24764970/cg25047001	0.187488334	0.294552973	0.470548664	0.17599569	1.80E-04	6.42E-03
TSS1500	GRWD1	19	48947560/48947631/48947572/48947578/48948005	5	cg04988514/cg14594362/cg16875863/cg22242216/cg26362197	0.337209907	0.448778694	0.62741754	0.178638846	1.99E-04	6.68E-03
TSS1500	HEPN1	11	124788414	1	cg23530543	0.777910525	0.658016239	0.490776441	-0.167239798	4.36E-04	8.50E-03
TSS1500	HKDC1	10	70979827/70979811/70979777	3	cg05532178/cg11639651/cg23341612	0.811678557	0.715256174	0.537345724	-0.17791045	8.04E-04	1.06E-02
TSS1500	HMHB1	5	143190384/143191194/143191226	3	cg12672779/cg14461752/cg15823954	0.872872	0.77037915	0.612053091	-0.158326059	1.05E-04	5.67E-03
TSS1500	HTR1A	5	63257885/63257813/63257847/63257867/63257941/63257873/63257753	7	cg07839533/cg08259925/cg09698471/cg11615755/cg13666507/cg15092168/cg1628[illegible]	0.338136579	0.413936406	0.581700744	0.167764338	1.73E-05	4.46E-03
TSS1500	IGLON5	19	51814765/51814782	2	cg22489583/cg25728174	0.066697245	0.071265356	0.282444154	0.211178798	6.94E-04	1.01E-02
TSS1500	IL17A	6	52050133/52050597	2	cg09582880/cg11924517	0.67080109	0.520380394	0.348856723	-0.171523672	2.31E-04	7.11E-03
TSS1500	INA	10	105036611/105036701/105036645	3	cg18673954/cg21384402/cg23642747	0.276782288	0.405315217	0.614194856	0.208879639	1.41E-03	1.36E-02
TSS1500	IRF4	6	391189/391208/391114/391441	4	cg01108118/cg10379890/cg25140370/cg26433102	0.159531561	0.300096832	0.450682769	0.150585938	3.00E-03	2.07E-02
TSS1500	IRX1	5	3594679/3595728/3595907/3595106	4	cg01790920/cg05534710/cg06689918/cg07903370	0.257273828	0.338832611	0.4921754	0.153342789	8.77E-05	5.37E-03
TSS1500	IRX4	5	1883413/1883514/1884212/1884365/1883940/1883954/1883214	7	cg00243313/cg00264591/cg05941108/cg14823763/cg18009496/cg23121993/cg2487[illegible]	0.254729214	0.364092219	0.533084286	0.168992067	5.26E-03	2.92E-02
TSS1500	KCNA1	12	5018338/5018513/5018715/5018815/5018229/5018805/5018798	7	cg00838874/cg04891086/cg06652199/cg11388325/cg15930596/cg19651132/cg2039[illegible]	0.183212817	0.279456873	0.503915957	0.224459084	4.54E-06	3.48E-03
TSS1500	KCNK12	2	47797953/47798679/47798477/47797963/47798396	5	cg01826863/cg04934807/cg04981611/cg13913015/cg27320127	0.092764989	0.153461384	0.33881546	0.185354075	1.11E-03	1.24E-02
TSS1500	KHDRBS2	6	62996702/62996697/62996627/62996664	4	cg00578220/cg02217159/cg04398581/cg23926793	0.212554239	0.312072944	0.510115303	0.198042359	1.92E-03	1.61E-02
TSS1500	KLRG1	12	9141740/9141775	2	cg00443307/cg26806779	0.721784318	0.576945203	0.381956675	-0.194988528	1.97E-05	4.47E-03
TSS1500	KRTAP13-4	21	31802169	1	cg24082983	0.558472755	0.452200783	0.301083264	-0.15111752	9.73E-05	5.58E-03
TSS1500	LAMA1	18	7118077/7118122/7118092	3	cg03159124/cg14950072/cg19909349	0.32049846	0.412715654	0.601877356	0.189161702	5.32E-04	8.96E-03

TSS1500	LIPH	3	185271312/185271385/185271383	3	cg01390445/cg04513882/cg16255307	0.861541818	0.827888689	0.665705432	-0.162183257	6.87E-04	9.97E-03
TSS1500	LOC100133469	14	106937310/106938234/106937869	3	cg13275724/cg16023283/cg20900869	0.723368845	0.641521967	0.490657027	-0.150864939	5.27E-06	3.48E-03
TSS1500	LOC100190940	12	130527106/130527104/130527212	3	cg02681173/cg10233825/cg15848986	0.33267563	0.438905169	0.628995864	0.190090694	4.44E-04	8.50E-03
TSS1500	LOC157627	8	9761905/9761454/9761141/9761171/9762300/9762100/9762057	7	cg06292304/cg12616174/cg14278808/cg15248835/cg15537082/cg18246262/cg2418	0.21117185	0.309867936	0.474917205	0.165049269	7.98E-04	1.06E-02
TSS1500	LRFN5	14	42075865/42075554/42075760/42075975/42075810/42075406/42075664/42076492	8	cg02150867/cg02454806/cg04171065/cg05943996/cg10160975/cg10327400/cg21526847/cg23955431	0.219774383	0.317605529	0.485380155	0.167774626	6.16E-04	9.59E-03
TSS1500	LVRN	5	115297723/115297690/115297853/115297488	4	cg06644553/cg10199250/cg15489294/cg23915769	0.390596855	0.434036431	0.606987731	0.1729513	9.38E-06	3.60E-03
TSS1500	MIR124-3	20	61809035/61808888/61809348/61808691/61809557	5	cg02065637/cg02650317/cg04927004/cg08737296/cg15699267	0.187117414	0.288151058	0.443465535	0.155314477	1.19E-03	1.28E-02
TSS1500	MIR216B	2	56229060	1	cg02071553	0.738162795	0.683389278	0.5301385	-0.153250778	8.13E-04	1.06E-02
TSS1500	MIR34C	11	111383668/111382915/111383603/111383288/111383576/111383083/111383515/111383624/111383889/111383	10	cg01192900/cg03242880/cg13767940/cg18515591/cg21881253/cg22806002/cg22879515/cg23211240/cg24041978/cg2656178	0.304172077	0.372950868	0.546756216	0.173805348	6.29E-04	9.68E-03
TSS1500	MMP16	8	89340750/89340069/89341103/89340205/89340139/89340331/89340480	7	cg01673994/cg05033271/cg07609388/cg08397978/cg12818557/cg14644752/cg2445	0.327120406	0.40430779	0.575818649	0.171510859	4.89E-04	8.78E-03
TSS1500	MUSK	9	113430005	1	cg01737465	0.782561555	0.74197165	0.588620055	-0.153351595	6.63E-04	9.85E-03
TSS1500	NID2	14	52536175/52536436/52536147/52536721/52536893	5	cg04701034/cg16695483/cg22881914/cg25685519/cg26923084	0.528406575	0.523222246	0.673418497	0.150196251	1.47E-04	6.10E-03
TSS1500	NMBR	6	142410216/142410235/142410221	3	cg13202845/cg18425786/cg21460020	0.342598663	0.382262107	0.546260842	0.163998735	1.42E-04	5.98E-03
TSS1500	NOVA1	14	27067707/27067895/27068354/27068093/27067638/27067372/27068135	7	cg02014003/cg07543626/cg11146971/cg16791424/cg18468511/cg20478129/cg2350	0.274856558	0.292430327	0.457206281	0.164775954	4.00E-04	8.37E-03
TSS1500	NPAS4	11	66188019/66188203/66187533	3	cg02055483/cg09835239/cg24435401	0.218762555	0.328106943	0.536197375	0.208090432	9.13E-05	5.37E-03
TSS1500	NPBWR1	8	53852101/53851613/53852184/53852030/53851707/53851156	6	cg02115041/cg07629017/cg15531403/cg16330450/cg24857620/cg26205771	0.253709521	0.413337796	0.606035915	0.192698119	3.59E-04	8.05E-03
TSS1500	NPTX2	7	98245830/98246006/98245716/98246001	4	cg00548268/cg08315202/cg09596818/cg13314145	0.416650292	0.435665248	0.592178547	0.156513299	2.92E-03	2.04E-02

TSS1500	NR2E1	6	108486517/108486387/108486450/108486773/108486123/108485837/1084	7	cg03950614/cg03958979/cg08640634/cg12463600/cg18710462/cg20652607/cg2123	0.325656174	0.404558707	0.563828238	0.159269531	6.07E-05	5.29E-03
TSS1500	NTRK3	15	88799973/88800510/88800567/88799999/88801004/88800624	6	cg04627496/cg04920689/cg05901579/cg07252731/cg11108676/cg14384532	0.170978061	0.318451373	0.507354069	0.188902696	1.19E-03	1.28E-02
TSS1500	ONECUT1	15	53083238/53082477/53083587/53083532/53082902/53083518/53083135	7	cg01196531/cg02061705/cg06657050/cg13877670/cg15628498/cg20056542/cg2615	0.294836457	0.431906515	0.589879428	0.157972914	7.79E-05	5.34E-03
TSS1500	OR10A7	12	55613318	1	cg26442740	0.702508535	0.621660633	0.458238027	-0.163422606	7.68E-04	1.05E-02
TSS1500	OR10J1	1	159409207/159408079	2	cg15700197/cg23689219	0.660124183	0.513700247	0.359709559	-0.153990688	4.97E-05	5.10E-03
TSS1500	OR10K2	1	158391119	1	cg01733284	0.809446245	0.676295411	0.484221795	-0.192073616	5.24E-04	8.91E-03
TSS1500	OR2L8	1	248111283/248110661	2	cg10580513/cg18857759	0.613446275	0.493381778	0.319458732	-0.173923046	1.95E-06	3.48E-03
TSS1500	OR56A1	11	6049729	1	cg18512262	0.57736578	0.416929178	0.255862718	-0.16106646	4.72E-05	5.07E-03
TSS1500	OR8D2	11	124191389/124190997	2	cg04629433/cg15952370	0.544132685	0.465419733	0.256743609	-0.208676124	4.75E-06	3.48E-03
TSS1500	OTOP1	4	4229061/4229810/4229883	3	cg03160466/cg06658468/cg20849299	0.347108885	0.414799181	0.581701818	0.166902638	7.78E-04	1.05E-02
TSS1500	P2RX2	12	133195094/133193959/133195196/133195061	4	cg00058329/cg02315111/cg22368664/cg26620450	0.209241909	0.234090244	0.44044457	0.206354326	1.25E-04	5.92E-03
TSS1500	PAX2	10	102504226/102504893/102504649/102504236/102505266/102504022/102504297/102504039	8	cg01275523/cg05044739/cg09222673/cg14096353/cg15416233/cg16443970/cg17945419/cg25523509	0.154330824	0.217752711	0.376641182	0.158888471	0.000150033	0.006149797
TSS1500	PCDH10	4	134069409/134070235/134069304/134069670/134070039/134069388/134069593/134069236	8	cg00945238/cg02043159/cg02114924/cg04035209/cg05401965/cg07665387/cg10196720/cg14146100	0.321056674	0.419374559	0.61695395	0.197579391	4.56E-05	5.03E-03
TSS1500	PCDH17	13	58205001/58204379/58205045/58204365/58205111	5	cg07430472/cg12606396/cg13806135/cg15112032/cg25891355	0.293968366	0.406563267	0.570533475	0.163970208	8.61E-05	5.37E-03
TSS1500	PCDHGB4	5	140767197/140767242/140766911/140767200	4	cg02741229/cg22041640/cg23347399/cg26282384	0.366960643	0.434457358	0.585020359	0.150563001	4.73E-04	8.66E-03
TSS1500	PCDHGB5	5	140777473/140777344	2	cg11686427/cg25426815	0.374205635	0.391285904	0.576918885	0.185632981	2.13E-05	4.47E-03
TSS1500	PCDHGC4	5	140864276/140864474/140864020	3	cg06428620/cg12648074/cg26890354	0.26878578	0.380269639	0.540655073	0.160385434	4.46E-04	8.51E-03
TSS1500	PHOX2A	11	71956145/71955547/71955439/71955599	4	cg01527777/cg08876932/cg16922279/cg24530250	0.301572288	0.360743145	0.521985263	0.161242118	2.41E-03	1.84E-02
TSS1500	POU4F2	4	147559648/147559579/147559423/147558544	4	cg12935979/cg13262687/cg19597382/cg25970230	0.33730075	0.459647814	0.634065168	0.174417354	2.75E-04	7.48E-03
TSS1500	PPP1R2P1	6	32843055/32844032	2	cg13617603/cg22744098	0.57436797	0.467743344	0.305982527	-0.161760817	9.48E-06	3.60E-03

TSS1500	PTPRT	20	41819125/41818788/41818770/41818905	4	cg04541293/cg07167168/cg17859110/cg21672843	0.184511835	0.266124741	0.432638811	0.166514071	1.22E-04	5.88E-03
TSS1500	QKI	6	163834924/163834452/163834872/163834903/163835251/163835395/163834176/163835117,	8	cg00852768/cg07618175/cg08169901/cg09854653/cg13695877/cg20930060/cg23954057/cg25157874	0.087814187	0.144904504	0.300689284	0.15578478	4.26E-03	2.55E-02
TSS1500	RALYL	8	85096037/85095275/85094904/85096835/85094923/85094842/85094742/85094437/85096015/85096868/85095654/85095697	12	cg00034076/cg00266009/cg01134282/cg01538821/cg06955749/cg08327106/cg09757712/cg11089595/cg22403811/cg23458558/cg23777958/cg266	0.27086144	0.310675616	0.502710953	0.192035337	1.03E-04	5.66E-03
TSS1500	RGS21	1	192285333	1	cg00158654	0.79529358	0.666336306	0.512647895	-0.15368841	2.16E-04	6.89E-03
TSS1500	RGS6	14	72398920/72399553/72398856/72398839	4	cg14702655/cg15457745/cg16396954/cg23576118	0.132310638	0.143585694	0.298340158	0.154754464	2.54E-04	7.36E-03
TSS1500	RPL31P11	1	161655895	1	cg22445346	0.748590945	0.614487478	0.448657486	-0.165829991	7.08E-05	5.34E-03
TSS1500	RYR2	1	237205295/237205174/237205409/237205098	4	cg03422911/cg07790615/cg18375860/cg19764418	0.452385778	0.41005682	0.648201086	0.238144267	2.93E-06	3.48E-03
TSS1500	SCARF2	22	20792476/20792535	2	cg08748948/cg14785479	0.279022718	0.351433703	0.511275693	0.15984199	5.83E-04	9.39E-03
TSS1500	SHISA9	16	12994049/12995011/12995078/12994998	4	cg02748479/cg09229683/cg21432375/cg26752526	0.225686592	0.278124102	0.430683681	0.152559579	1.42E-03	1.37E-02
TSS1500	SIRPG	20	1639325/1639816/1639250	3	cg11061975/cg13408605/cg23295754	0.702641013	0.50884555	0.339862545	-0.168983005	5.63E-05	5.22E-03
TSS1500	SLC10A6	4	87770782/87770744	2	cg13119182/cg15881238	0.828922718	0.733751794	0.566891625	-0.166860169	1.46E-04	6.09E-03
TSS1500	SLC18A3	10	50817104/50817213/50817804/50816963/50816917/50817306/50817810/50817651/50817096	9	cg05997350/cg06108782/cg08996579/cg12052765/cg17284779/cg18592174/cg23987444/cg26813646/cg	0.298896711	0.373556264	0.526404253	0.152847989	5.10E-04	8.85E-03
TSS1500	SLC27A6	5	128300967/128300696/128300708	3	cg00076307/cg20103758/cg20417527	0.242221315	0.30317555	0.492429005	0.189253455	6.27E-04	9.68E-03
TSS1500	SLITRK5	13	88324193/88324597/88324432/88324570/88323396/88323992/88323940/88324169/88323607/88323452	10	cg08492619/cg09083627/cg15461335/cg15778745/cg16328172/cg19391029/cg21194448/cg25081106/cg26110710/cg2715181	0.199629367	0.201339328	0.355461468	0.15412214	1.43E-04	6.03E-03
TSS1500	SNORD113-4	14	101402192/101402494	2	cg03629141/cg05218696	0.690925805	0.640672289	0.459901064	-0.180771225	1.81E-04	6.43E-03
TSS1500	SNTG1	8	50824244/50823541/50823173/50823468/50823377/50823252/50823124/50824358	8	cg06248179/cg07739205/cg07854547/cg08896053/cg08954417/cg22095914/cg25086136/cg26461695	0.249223634	0.339200155	0.493951034	0.15475088	4.48E-04	8.51E-03

TSS1500	SORCS3	10	106400259/106400114/106399513/106400565/106399957/106400454/1063	7	cg02320740/cg02392179/cg07126167/cg08768569/cg09551147/cg14752336/cg2379	0.259994593	0.362678822	0.524607345	0.161928523	2.31E-04	7.09E-03
TSS1500	SPAG11B	8	7311130	1	cg14600420	0.61582796	0.442693267	0.288175909	-0.154517358	2.65E-04	7.43E-03
TSS1500	ST8SIA5	18	44338099/44337910/44337664/44337922/44338147/44337853	6	cg01059449/cg15049968/cg15966229/cg23129478/cg23302884/cg26155939	0.285734185	0.334694973	0.535361973	0.200667	4.19E-04	8.49E-03
TSS1500	SYT6	1	114697582/114696698/114697523/114696822/114697352/114697113/114696946/114697095/114697032/114696798/114697707	11	cg00764109/cg03514545/cg06620092/cg08883066/cg12097117/cg12804010/cg15976388/cg19874857/cg19901801/cg22646710/cg26426632	0.241304647	0.277428165	0.427951805	0.15052364	0.000663443	0.009850114
TSS1500	SYT9	11	7272804/7272653/7272867/7272876	4	cg01806928/cg08913010/cg14243481/cg26945996	0.281621628	0.351672528	0.545256369	0.193583841	1.56E-03	1.44E-02
TSS1500	TEKT1	17	6735316/6735539/6735272	3	cg03549162/cg12685753/cg15615160	0.201185964	0.266171536	0.444536309	0.178364773	4.90E-04	8.78E-03
TSS1500	TLX3	5	170735186/170735264/170736021/170734856/170735754/170735347	6	cg05787556/cg18336674/cg18468394/cg22160448/cg24881420/cg26517171	0.386740033	0.468786828	0.621915544	0.153128717	1.68E-04	6.40E-03
TSS1500	TRDN	6	123959184	1	cg14462830	0.72351412	0.616815044	0.432497877	-0.184317167	0.000164978	0.006393705
TSS1500	TRHDE	12	72665510/72666264/72665880/72666281/72665376/72665282/72665771	7	cg01817029/cg02511156/cg04775889/cg06622999/cg17771605/cg18440199/cg2314	0.235902135	0.300701726	0.453103565	0.152401839	2.05E-03	1.68E-02
TSS1500	USP44	12	95942964/95943114/95942907/95945767/95942847	5	cg07783282/cg09883286/cg13879483/cg16192751/cg17368254	0.196333114	0.242103689	0.431633	0.189529311	1.86E-03	1.59E-02
TSS1500	VASH1	14	77227574/77227860/77227570/77227545/77227645	5	cg02735446/cg07559696/cg12825804/cg14124894/cg23948080	0.176556459	0.181655931	0.337989367	0.156333436	2.49E-03	1.88E-02
TSS1500	VEGFC	4	177714373/177714350/177714456/177715040	4	cg01590753/cg06679347/cg17177660/cg24194285	0.384570977	0.409092681	0.559936502	0.150843821	5.99E-04	9.50E-03
TSS1500	WBSCR17	7	70596377/70597091/70597058/70597065/70597458/70596308	6	cg01159623/cg01366419/cg02300154/cg03044249/cg03893271/cg10239388	0.394991448	0.47322018	0.639233247	0.166013067	2.19E-04	6.95E-03

TSS1500	WDR8	1	3567870/3567303/3568004/3566992/3567646/3567719/3568154/3566963/3567852/3568124/3567412/3567119/3567214/3567930/3567408/3567109/3567163/3566950/3567986/3567732/3567738/3567550	22	cg01434649/cg04021697/cg04391111/cg04865841/cg07382920/cg10038618/cg10143426/cg11256802/cg12475507/cg14781922/cg16823083/cg18021902/cg19135761/cg20611911/cg21000072/cg21012455/cg22614891/cg22822803/cg24073122/cg24678	0.364920319	0.467848881	0.620655629	0.152806748	8.67E-05	5.37E-03
TSS1500	WT1	11	32457878/32457727/32457386	3	cg12600018/cg22975913/cg26232818	0.182944365	0.213337707	0.375527668	0.162189961	1.05E-02	4.61E-02
TSS1500	XKR4	8	56013556/56013800/56014406/56014433	4	cg08417620/cg09388605/cg09524907/cg20948997	0.293191333	0.29172405	0.443875378	0.152151329	1.30E-03	1.33E-02
TSS1500	YIPF7	4	44654206	1	cg03815116	0.65073397	0.494116667	0.3368489	-0.157267767	1.97E-04	6.67E-03
TSS1500	ZNF256	19	58459358/58459283	2	cg02959669/cg12048031	0.091643475	0.136415784	0.291683919	0.155268135	2.76E-03	1.98E-02
TSS1500	ZNF300	5	150284600/150284796	2	cg18237551/cg21228005	0.367075915	0.360543939	0.578431705	0.217887766	1.69E-04	6.41E-03
TSS1500	ZNF667	19	56989102/56989543/56989812/56989740	4	cg03289872/cg08063125/cg21287936/cg24411929	0.414287348	0.566804689	0.724532957	0.157728268	7.05E-04	1.01E-02
TSS1500	ZNF677	19	53758521/53758609	2	cg04154138/cg16708981	0.35791966	0.495035972	0.645107336	0.150071364	3.44E-04	7.97E-03
TSS1500	ZNF813	19	53970618/53970585/53970754	3	cg05503527/cg09435109/cg13948710	0.146824803	0.20720027	0.363508075	0.156307805	3.37E-04	7.95E-03
TSS1500	ZPBP	7	50133212/50133131/50133100	3	cg00303745/cg09921682/cg10349880	0.84136324	0.669956002	0.48186017	-0.188095832	3.64E-05	4.78E-03
TSS200	MARCH11	5	16180048/16180055/16180072/16180062/16180068/16180076/16180033	7	cg00339556/cg01791874/cg16150752/cg17030173/cg17712694/cg21901718/cg2509	0.288388658	0.381014661	0.566613481	0.18559882	3.01E-03	3.27E-02
TSS200	ACTN2	1	236849653	1	cg14932016	0.27037604	0.281198133	0.474309027	0.193110894	1.88E-04	1.06E-02
TSS200	ADAMTS16	5	5140406	1	cg01717727	0.375045035	0.4556967	0.623165405	0.167468705	5.19E-04	1.39E-02
TSS200	ADAMTS18	16	77469167	1	cg02186542	0.273228825	0.282129683	0.439599427	0.157469744	4.71E-04	1.35E-02
TSS200	ADAMTS5	21	28339442/28339487/28339477	3	cg08190291/cg13601496/cg19523085	0.220681917	0.294013407	0.471714923	0.177701515	8.79E-04	1.72E-02
TSS200	ADARB2	10	1779835/1779904/1779819/1779730/1779760	5	cg02899206/cg03410436/cg06730723/cg14590817/cg24184687	0.120993329	0.238864613	0.388903645	0.150039032	5.51E-03	4.76E-02
TSS200	ADCY5	3	123167522/123167507	2	cg04138502/cg15993383	0.263099946	0.310493049	0.50221849	0.191725442	2.28E-04	1.12E-02
TSS200	ADD2	2	70995440/70995522/70995351/70995459/70995444/70995349/70995527	7	cg03384579/cg05659187/cg08857144/cg15170605/cg23427269/cg24347663/cg2563	0.121104374	0.227999381	0.42689553	0.198896149	5.24E-03	4.61E-02
TSS200	ADHFE1	8	67344553/67344640/67344556/67344588/67344642/67344665	6	cg01588438/cg08090772/cg09383816/cg18065361/cg19283840/cg20295442	0.237875042	0.386282476	0.615113493	0.228831016	1.44E-04	1.06E-02

TSS200	ADRA1A	8	26722965/26722933	2	cg20303399/cg23008606	0.388836703	0.4551341	0.605570534	0.150436434	1.26E-04	9.92E-03
TSS200	AEBP1	7	44143774	1	cg25289803	0.126515985	0.170135441	0.327045437	0.156909997	5.36E-03	4.67E-02
TSS200	ARMC3	10	23216847/23216843/23216841	3	cg06560760/cg08839858/cg19523082	0.156176652	0.256517689	0.475421363	0.218903674	7.87E-04	1.63E-02
TSS200	BAALC	8	104152750/104152754	2	cg10163483/cg14284952	0.24627411	0.358346559	0.546722218	0.188375659	1.59E-03	2.32E-02
TSS200	BARHL2	1	91182989/91182856	2	cg00010742/cg17241310	0.319597535	0.354711339	0.507436764	0.152725425	9.03E-04	0.01746453
TSS200	BPIL1	20	31595346/31595360	2	cg10968815/cg13696012	0.74723877	0.610260339	0.45784017	-0.152420168	4.60E-05	7.87E-03
TSS200	BSX	11	122852454/122852523/122852429/122852412/122852417/122852461	6	cg02078690/cg02159381/cg02179652/cg04092231/cg11141380/cg27314569	0.353419475	0.420348966	0.586804642	0.166455676	2.32E-05	6.89E-03
TSS200	C11orf87	11	109292803/109292809/109292789	3	cg11473876/cg16359550/cg27471124	0.343952475	0.433410228	0.584383979	0.150973751	2.02E-04	1.06E-02
TSS200	C12orf56	12	64784498/64784483	2	cg13867865/cg15731056	0.208991981	0.256064203	0.47905462	0.222990417	2.99E-04	1.18E-02
TSS200	C13orf30	13	43355514	1	cg24818699	0.725343805	0.683871289	0.530825736	-0.153045553	7.18E-04	1.55E-02
TSS200	C14orf177	14	99177777	1	cg06906435	0.632554745	0.557396089	0.363526641	-0.193869448	1.21E-04	9.92E-03
TSS200	C14orf39	14	60952945/60952933/60952781	3	cg12189551/cg26132298/cg26248878	0.279060523	0.386990292	0.543512184	0.156521892	4.49E-03	0.041973444
TSS200	C16orf73	16	1922370/1922149/1922155/1922216/1922207	5	cg03467725/cg05041351/cg07775371/cg08959396/cg27308021	0.155952418	0.191239859	0.348454698	0.157214839	3.08E-04	1.20E-02
TSS200	C17orf104	17	42733698/42733724/42733729/42733592/42733662/42733600	6	cg02932314/cg12127472/cg12781700/cg12892303/cg15000379/cg17269633	0.327629685	0.414478568	0.574156066	0.159677498	1.24E-03	2.02E-02
TSS200	C18orf34	18	31020754/31020806	2	cg00625334/cg05994094	0.264556073	0.34116821	0.511657836	0.170489627	1.73E-03	0.024223818
TSS200	C1orf70	1	1475742	1	cg15487867	0.078401843	0.225160943	0.516900801	0.291739858	7.76E-05	9.06E-03
TSS200	C1QL2	2	119916510/119916571/119916662/119916581/11991648	5	cg07212778/cg15931721/cg16849609/cg17319142/cg21858380	0.283311327	0.397913469	0.549646376	0.151732907	2.81E-03	3.13E-02
TSS200	C4orf47	4	186350505	1	cg18740810	0.627730495	0.531216306	0.377883173	-0.153333133	6.44E-06	5.78E-03
TSS200	CACNG8	19	54466187	1	cg23752563	0.33931252	0.383725244	0.608583723	0.224858478	1.90E-06	4.51E-03
TSS200	CCDC140	2	223162692/223162797/223162800/223162666	4	cg01790894/cg10416206/cg13767755/cg14093610	0.266659694	0.281424995	0.436827723	0.155402728	1.72E-04	1.06E-02
TSS200	CCDC37	3	126113663/126113658/126113682/126113707/126113695/126113743/1261	7	cg01815671/cg09686443/cg11229862/cg20312228/cg20952286/cg21109744/cg2381	0.296193755	0.326978721	0.5068705	0.179891779	3.00E-04	1.18E-02
TSS200	CCDC70	13	52436047	1	cg11631011	0.71729355	0.560230817	0.398430955	-0.161799862	3.50E-04	1.24E-02
TSS200	CDH10	5	24644988/24645093/24645065/24645024	4	cg02617312/cg05683049/cg17030055/cg26161643	0.200284973	0.257347642	0.414638753	0.157291112	5.91E-05	8.40E-03
TSS200	CDH18	5	19988368/19988366	2	cg16503924/cg18710278	0.371138315	0.453583656	0.615012223	0.161428567	0.00097361	0.018233792
TSS200	CDH19	18	64271341	1	cg16489926	0.75453834	0.579833833	0.40824685	-0.171586983	8.62E-06	0.005906912

TSS200	CHL1	3	238615/238611/238618/238496	4	cg07746943/cg08421685/cg12065366/cg25482786	0.32114382	0.329874344	0.537976477	0.208102133	4.25E-05	7.61E-03
TSS200	CHST10	2	101034279/101034257/101034270	3	cg03853987/cg16882226/cg19283506	0.110492482	0.247492011	0.52275403	0.275262019	1.07E-03	1.89E-02
TSS200	CLDN10	13	96204870/96204856/96204917/96204873/96204854/96204860	6	cg04603730/cg06428163/cg09469554/cg10305311/cg16232183/cg16275739	0.169117983	0.278977054	0.520358225	0.241381171	1.13E-04	9.92E-03
TSS200	CMTM2	16	66613334/66613220/66613278/66613206/66613273/66613266	6	cg00049664/cg05546863/cg06666025/cg07153665/cg09854734/cg16626067	0.326302001	0.389529445	0.592895287	0.203365842	8.95E-05	9.57E-03
TSS200	CNBD1	8	87878605	1	cg00374101	0.69649682	0.572691322	0.398347268	-0.174344054	1.59E-05	6.89E-03
TSS200	CNTNAP5	2	124782817/124782831/124782713	3	cg04168670/cg08790440/cg12919006	0.445512275	0.494088333	0.675895377	0.181807044	2.82E-05	6.89E-03
TSS200	COLEC12	18	500817	1	cg16943083	0.118796845	0.134338515	0.334893467	0.200554952	3.42E-04	0.01228057
TSS200	CRHR2	7	30722327/30722163/30722177/30722320	4	cg01718447/cg04863452/cg07658503/cg21773872	0.184872435	0.304892999	0.468855352	0.163962352	5.12E-03	4.55E-02
TSS200	CYP1A2	15	75041150	1	cg11473616	0.81962889	0.702935961	0.541688595	-0.161247366	2.50E-04	1.12E-02
TSS200	DBC1	9	122131895	1	cg12821278	0.38583843	0.46795035	0.665142118	0.197191768	2.01E-03	0.026173398
TSS200	DBX2	12	45445057/45444902/45444895/45445061/45445023	5	cg12915695/cg13031210/cg13186327/cg16080781/cg26274596	0.205081183	0.259889082	0.434139299	0.174250217	2.17E-03	2.69E-02
TSS200	DGKG	3	186080180/186080043/186080208/186080023	4	cg02567082/cg14601136/cg15738605/cg25808906	0.100521938	0.145630393	0.326807706	0.181177314	9.27E-05	9.69E-03
TSS200	DKFZP434H168	16	56228498/56228511/56228505/56228577/56228467/56228495/56228442	7	cg00246932/cg01157070/cg02667667/cg06780839/cg07700514/cg07829842/cg1904	0.113126065	0.229547982	0.505039465	0.275491483	1.66E-04	1.06E-02
TSS200	DNAH11	7	21582758/21582749/21582745	3	cg01122187/cg08570341/cg16474297	0.158832395	0.15329108	0.341847322	0.188556242	1.13E-03	1.94E-02
TSS200	DRD4	11	637173/637170/637175/637162	4	cg00556112/cg06825142/cg12928379/cg20931042	0.132138934	0.124251499	0.296712788	0.172461289	1.90E-03	2.54E-02
TSS200	EDNRB	13	78493100/78493012/78493066/78493126/78493128	5	cg04390523/cg06179060/cg06971129/cg13818654/cg15836660	0.245177538	0.395607701	0.575186885	0.179579184	1.56E-04	1.06E-02
TSS200	EFHA2	8	16884576/16884555/16884549	3	cg00720475/cg05956498/cg18577280	0.119189488	0.235860501	0.466411998	0.230551496	1.23E-04	9.92E-03
TSS200	EID3	12	104697389/104697545/104697387/104697419/104697526/104697532/1046	7	cg03817911/cg09477407/cg18633684/cg20923245/cg21234561/cg26614816/cg2720	0.245901051	0.400084094	0.594826503	0.194742409	5.46E-04	1.42E-02
TSS200	EMID2	7	101006063/101006019/101006089/101006035/101006052/101006058	6	cg14874750/cg17361163/cg18762727/cg23424003/cg27179533/cg27528510	0.313617047	0.41950658	0.576084525	0.156577945	1.53E-03	2.28E-02
TSS200	EVC	4	5712884/5712840/5712842	3	cg01333350/cg06918887/cg25213928	0.127460136	0.230972145	0.452348692	0.221376547	3.53E-04	1.24E-02
TSS200	FABP4	8	82395569	1	cg19422565	0.687758385	0.65046045	0.498557782	-0.151902668	2.86E-06	0.004505573

TSS200	FAM184B	4	17783205/17783294/17783261/17783240/17783331/17783322	6	cg01886556/cg02108033/cg05624214/cg15917116/cg18790597/cg27207796	0.215687576	0.234713841	0.420879943	0.186166102	1.81E-03	2.47E-02
TSS200	FAM19A4	3	68981866/68981854/68981877/68981852/68981890/68981895	6	cg09868336/cg12219082/cg12412079/cg12417685/cg13921352/cg20375342	0.25409692	0.303376729	0.531583568	0.22820684	1.47E-04	1.06E-02
TSS200	FAT4	4	126237464/126237371/126237421/126237373/12623751	5	cg03527919/cg05118638/cg08644023/cg10731073/cg15795630	0.191660035	0.241493606	0.408754979	0.167261373	2.04E-04	1.06E-02
TSS200	FBLIM1	1	16085148	1	cg04897742	0.070202859	0.292923521	0.476116717	0.183193196	0.001446383	0.021994223
TSS200	FBXL7	5	15500249/15500270	2	cg24872782/cg26134895	0.15106963	0.139730535	0.296106291	0.156375756	2.45E-03	2.88E-02
TSS200	FBXO39	17	6679495/6679482/6679499/6679484/6679476/6679541	6	cg02093112/cg06091013/cg07540103/cg08843809/cg11871421/cg17097923	0.414734219	0.514230606	0.680763773	0.166533166	1.85E-04	1.06E-02
TSS200	FCRL3	1	157670710/157670825	2	cg08786003/cg15602298	0.71051115	0.669765689	0.479001236	-0.190764453	3.53E-04	1.24E-02
TSS200	FGF5	4	81187601/81187605/81187610	3	cg10031614/cg12528713/cg20528583	0.140989149	0.219427081	0.391214603	0.171787522	2.03E-03	2.62E-02
TSS200	FLJ16779	20	61885236/61885285/61885270/61885249/61885291/61885262	6	cg06763829/cg07785314/cg09196068/cg15571277/cg18412834/cg21258057	0.069564278	0.171933272	0.37376137	0.201828099	5.24E-04	1.40E-02
TSS200	FLJ43390	14	62584024/62583968/62584005/62584037/62583919/62583962	6	cg01613638/cg01866038/cg05173737/cg10227327/cg14926717/cg15897970	0.139913616	0.27133286	0.422528908	0.151196048	3.04E-03	3.30E-02
TSS200	FNDC1	6	159590231	1	cg00157796	0.201576911	0.360219017	0.580746665	0.220527648	1.78E-03	2.45E-02
TSS200	FREM3	4	144621938/144621952/144621898/144621971/14462194	5	cg08334984/cg09556043/cg10056356/cg15042811/cg17495719	0.276775177	0.444996611	0.597750659	0.152754048	2.11E-03	2.67E-02
TSS200	FZD10	12	130647004	1	cg21885046	0.255520285	0.31653685	0.479572268	0.163035418	8.25E-05	9.16E-03
TSS200	GAD2	10	26505127/26505069/26505156	3	cg00733780/cg04838147/cg23050442	0.234790059	0.393877345	0.584842092	0.190964748	1.07E-03	0.018927454
TSS200	GALNT13	2	154728308	1	cg02593932	0.38300914	0.394353661	0.590584686	0.196231025	2.28E-05	6.89E-03
TSS200	GALNT14	2	31361687/31361593/31361701/31361760/31361692/31361757	6	cg04050000/cg10258271/cg14514751/cg20599798/cg22395019/cg26988215	0.127646205	0.155388395	0.307139157	0.151750763	3.33E-04	1.22E-02
TSS200	GALNTL6	4	172734514/172734507/172734431	3	cg09772954/cg14307443/cg22153181	0.275216999	0.353055264	0.547891504	0.19483624	7.58E-05	9.06E-03
TSS200	GALR1	18	74961809/74961966/74962000/74961968	4	cg03032214/cg03659519/cg17911318/cg20872937	0.361228119	0.356662058	0.524020253	0.167358195	1.26E-03	2.04E-02
TSS200	GATA4	8	11561593/11561531/11561584/11561620	4	cg00397986/cg02726121/cg09951349/cg14900471	0.206063138	0.286203333	0.487388156	0.201184823	6.35E-05	8.63E-03
TSS200	GCM2	6	10882238/10882240/10882250	3	cg09829319/cg14250833/cg20229534	0.197304442	0.244470093	0.457785477	0.213315384	1.69E-03	2.40E-02
TSS200	GHSR	3	172166234/172166258/172166242	3	cg15987088/cg17152757/cg24599434	0.287919458	0.372009895	0.533990108	0.161980213	2.13E-04	1.09E-02

TSS200	GJD2	15	35046765/35046760	2	cg11525643/cg21053529	0.413471005	0.549616089	0.711265257	0.161649168	9.86E-05	9.77E-03
TSS200	GLRA3	4	175750583/175750562/175750527/175750637/175750510/175750507	6	cg02521102/cg05990549/cg09841488/cg21365094/cg23697278/cg26226802	0.232201128	0.307315318	0.494499608	0.18718429	5.15E-04	1.39E-02
TSS200	GPC6	13	93879022/93878969/93879042/93879008	4	cg00191477/cg07529657/cg23246978/cg25772299	0.162927445	0.19970389	0.350968335	0.151264445	4.76E-03	4.36E-02
TSS200	GPR6	6	110300233/110300218/110300101/110300146/11030009	5	cg01261007/cg01769232/cg05756933/cg12800028/cg15381304	0.391339138	0.407526868	0.563711615	0.156184747	1.81E-04	1.06E-02
TSS200	GRIA2	4	158141570/158141542	2	cg00699993/cg10410142	0.399915025	0.481114561	0.637241957	0.156127396	9.75E-05	9.77E-03
TSS200	GRIA4	11	105481306/105481317/105481319/105481322/105480771/105480788/105480790/105481283/105481406/105481292/105480792	11	cg00343633/cg03225817/cg04747226/cg07972135/cg09147777/cg11308643/cg12754421/cg15603568/cg21217024/cg23559689/cg23676682	0.303668483	0.408511226	0.583959483	0.175448258	3.41E-04	1.23E-02
TSS200	GRM7	3	6902689	1	cg24213507	0.21921605	0.296931333	0.473498077	0.176566744	2.62E-03	2.99E-02
TSS200	GSX1	13	28366645/28366600/28366759/28366598/28366606/28366702	6	cg03388293/cg05344430/cg10071824/cg11354629/cg14168923/cg17369527	0.279743824	0.309918543	0.464428891	0.154510348	0.005704538	0.04878246
TSS200	GUCY1A2	11	106889320/106889329/106889318/106889364/10688933	5	cg02989669/cg12243375/cg15847198/cg20905516/cg25918303	0.078953429	0.122064987	0.326497579	0.204432592	2.92E-03	3.21E-02
TSS200	HECW1	7	43152125/43152021	2	cg03004999/cg13298841	0.254652345	0.296185131	0.465199643	0.169014513	1.66E-04	1.06E-02
TSS200	HOXC9	12	54393687/54393709/54393725	3	cg03215137/cg13233407/cg21272774	0.314765248	0.420804026	0.596110945	0.17530692	2.73E-04	1.15E-02
TSS200	HOXD10	2	176981336/176981469/176981328/176981422	4	cg05979020/cg13217260/cg18115040/cg25371634	0.440592453	0.334801396	0.509576	0.174774604	3.78E-04	1.27E-02
TSS200	HS3ST4	16	25703160/25703153	2	cg24862668/cg26933063	0.116458718	0.118546268	0.275044375	0.156498107	4.48E-03	4.19E-02
TSS200	IGF2AS	11	2161586	1	cg25163476	0.230925235	0.272385617	0.438010264	0.165624647	1.08E-03	1.89E-02
TSS200	INA	10	105036811/105036863/105036747/105036727	4	cg08050235/cg17567560/cg23003534/cg24680586	0.143560112	0.243447131	0.447113657	0.203666526	1.91E-03	2.54E-02
TSS200	IRAK3	12	66582780/66582805	2	cg20395892/cg26279550	0.330652861	0.392081044	0.569845831	0.177764787	1.98E-04	1.06E-02
TSS200	IRF4	6	391680/391743/391683/391664	4	cg03410961/cg05766140/cg16046028/cg16643840	0.082199335	0.178686749	0.337683454	0.158996704	2.38E-03	2.84E-02
TSS200	IZUMO1	19	49250252/49250180	2	cg15085603/cg16732175	0.124496334	0.157671631	0.313989755	0.156318125	4.56E-04	1.34E-02
TSS200	JAM3	11	133938788/133938676/133938757/133938810	4	cg03637878/cg19055936/cg24140030/cg24899571	0.146781501	0.214326459	0.413833505	0.199507045	2.56E-05	6.89E-03

TSS200	KCNA1	12	5019017/5019004/5019066/5018984/5019024	5	cg03929977/cg10569606/cg10767336/cg12080391/cg15543616	0.181326338	0.291514145	0.499834589	0.208320445	1.58E-04	1.06E-02
TSS200	KCNA3	1	111217691/111217712/111217715/111217785	4	cg06750832/cg07808555/cg09410234/cg26071016	0.25023847	0.437710297	0.609975493	0.172265196	4.02E-04	1.30E-02
TSS200	KCNA7	19	49576200/49576241	2	cg01501819/cg26450866	0.257687215	0.216959519	0.37117688	0.15421736	3.02E-03	3.28E-02
TSS200	KCNK12	2	47797488/47797569/47797590	3	cg03603951/cg04431946/cg08074851	0.245250457	0.372603034	0.554110771	0.181507737	2.92E-04	1.17E-02
TSS200	KCNQ5	6	73331405	1	cg00852573	0.236188001	0.456947117	0.638643491	0.181696374	1.77E-03	2.45E-02
TSS200	LCE1F	1	152748838	1	cg01915081	0.657349355	0.581917528	0.429483991	-0.152433537	5.07E-06	5.00E-03
TSS200	LHCGR	2	48982957/48982950	2	cg12351433/cg19403014	0.385139748	0.376765842	0.550063805	0.173297963	6.57E-04	1.52E-02
TSS200	LOC157627	8	9760957/9760869/9760880/9760855/9760877	5	cg00571033/cg10866298/cg12877251/cg16424920/cg21535000	0.277610124	0.377193257	0.554742041	0.177548784	8.14E-04	1.65E-02
TSS200	LOC200726	2	207506975/207506991/207507001/207507096	4	cg08057136/cg12110911/cg17165841/cg21541833	0.328318215	0.440644479	0.59412389	0.153479411	1.58E-04	1.06E-02
TSS200	LOC643387	2	239140318/239140169/239140190/239140296/23914018	5	cg06732439/cg07323141/cg13530938/cg21272465/cg23497383	0.443331268	0.496596531	0.651977202	0.155380671	1.99E-05	6.89E-03
TSS200	LPPR4	1	99729804/99729807/99729757/99729790/99729769/99729780	6	cg03580297/cg03646889/cg05596294/cg06060191/cg18947801/cg26164310	0.251086583	0.325342127	0.476043734	0.150701607	2.25E-03	2.74E-02
TSS200	LRFN5	14	42076759/42076710/42076757	3	cg03566719/cg13526007/cg14021345	0.34677136	0.464009417	0.635554882	0.171545465	9.92E-04	1.83E-02
TSS200	LRRC3B	3	26664181/26664115/26664285	3	cg05583398/cg13787438/cg24216220	0.259333422	0.366976277	0.51808965	0.151113373	5.34E-04	0.014094129
TSS200	LRRC67	8	67940929/67940913/67940823/67940950	4	cg03024587/cg03780545/cg15744359/cg21189727	0.108376956	0.129075504	0.283722116	0.154646612	4.97E-03	4.46E-02
TSS200	MAP3K13	3	185080905/185080816/185080951	3	cg11743827/cg19418951/cg21574349	0.369315628	0.489911959	0.65445397	0.16454201	2.79E-04	1.16E-02
TSS200	MCHR2	6	100442105/100442118/100442151/100442120	4	cg06610484/cg09393254/cg22473637/cg26466587	0.492219206	0.588150617	0.743534964	0.155384347	1.09E-04	9.92E-03
TSS200	MGC2889	3	192959372	1	cg13810562	0.25417425	0.296998489	0.503987091	0.206988602	2.59E-04	0.011320864
TSS200	MIR124-1	8	9761141/9761171	2	cg14278808/cg15248835	0.17467567	0.290067164	0.54393962	0.253872457	3.72E-04	1.27E-02
TSS200	MIR129-2	11	43602879/43602920/43602847/43602857/43602845/43602914	6	cg01939477/cg05376374/cg14416371/cg14944647/cg15556502/cg16407471	0.279857002	0.39352092	0.603149488	0.209628568	6.02E-04	1.48E-02
TSS200	MIR137	1	98511908/98511792/98511789	3	cg05423529/cg14783814/cg22333214	0.279652767	0.369001891	0.565508567	0.196506676	3.18E-04	1.21E-02
TSS200	MIR34B	11	111383603/111383576/111383515/111383624/11138365	5	cg13767940/cg21881253/cg22879515/cg23211240/cg26561785	0.161390003	0.272821181	0.522451961	0.24963078	1.14E-03	1.95E-02
TSS200	MIR889	14	101514051	1	cg03290602	0.77199904	0.711557767	0.545312605	-0.166245162	2.04E-04	1.06E-02
TSS200	MMP1	11	102669090	1	cg14543953	0.720343735	0.589016539	0.404577436	-0.184439103	1.04E-04	0.00976695

TSS200	MMP13	11	102826570/102826565/102826469	3	cg14995062/cg19620758/cg22658979	0.752634965	0.557625358	0.396195655	-0.161429704	1.17E-04	0.009915092
TSS200	MMP16	8	89339819	1	cg04684553	0.136884403	0.189701904	0.422589257	0.232887353	1.73E-03	2.42E-02
TSS200	MOXD1	6	132722810/132722664/132722778/132722770/13272282	5	cg01019028/cg08661899/cg13640626/cg21294812/cg26178272	0.127174648	0.13160774	0.286870367	0.155262627	2.81E-03	3.13E-02
TSS200	MSC	8	72756882/72756863/72756812/72756901/72756839	5	cg03347018/cg14546394/cg20675505/cg24258699/cg25827490	0.243803204	0.377820598	0.542911809	0.165091211	3.42E-03	3.55E-02
TSS200	MYOD1	11	17740977/17741070/17740967	3	cg07271264/cg22653976/cg26410450	0.2867668	0.3895931	0.629171445	0.239578345	5.24E-05	8.03E-03
TSS200	NALCN	13	102068989/102068996/102069010	3	cg10774877/cg15202954/cg23056157	0.393140547	0.52235077	0.672534109	0.150183339	2.60E-03	2.99E-02
TSS200	NCAN	19	19322676/19322778/19322640/19322678	4	cg08181476/cg13724788/cg14316898/cg22090773	0.251505604	0.331823175	0.498386361	0.166563186	1.17E-03	1.97E-02
TSS200	NEFL	8	24814137	1	cg01614020	0.31224608	0.329022772	0.493136339	0.164113566	5.74E-03	4.90E-02
TSS200	NGF	1	115880865/115881044/115880935	3	cg09008705/cg13613280/cg13804058	0.223457763	0.326913945	0.504004302	0.177090357	0.001549176	0.022894986
TSS200	NKAIN4	20	61886082/61886079/61885926	3	cg01911708/cg08111446/cg23942897	0.236576717	0.36331312	0.519381209	0.15606809	1.86E-03	2.51E-02
TSS200	NPAS4	11	66188441/66188433	2	cg13215078/cg23484234	0.236016241	0.321604036	0.514617661	0.193013625	7.14E-05	0.00906104
TSS200	NPR3	5	32711517	1	cg18261223	0.083820478	0.107360912	0.28028821	0.172927298	6.09E-04	1.49E-02
TSS200	NR2E1	6	108487100/108487072/108487183/108487160/108487078/108487063	6	cg07411432/cg08616702/cg18093751/cg18918349/cg24104938/cg24225517	0.250054981	0.263668597	0.45908247	0.195413873	1.14E-04	9.92E-03
TSS200	OLIG2	21	34398114/34398199/34398131/34398222/34398085/34398226	6	cg03696345/cg08870743/cg15299832/cg23253569/cg27254482/cg27357571	0.254698848	0.380666611	0.584316247	0.203649636	2.42E-04	1.12E-02
TSS200	OR10A6	11	7950225	1	cg00444390	0.602448615	0.459103683	0.296502964	-0.16260072	6.02E-05	8.48E-03
TSS200	OR2G6	1	248684766	1	cg25819275	0.735832695	0.613297533	0.421585591	-0.191711942	4.84E-05	7.87E-03
TSS200	OR51M1	11	5410452	1	cg27093637	0.659121375	0.532627261	0.382448045	-0.150179216	7.22E-04	1.55E-02
TSS200	OR52N2	11	5841482	1	cg03895540	0.597053695	0.476309817	0.315040418	-0.161269398	3.64E-05	7.18E-03
TSS200	OR5D16	11	55606216	1	cg22937354	0.53126326	0.466551078	0.300326945	-0.166224132	5.77E-05	8.40E-03
TSS200	OR5W2	11	55682099	1	cg26349048	0.58245256	0.516382378	0.341970668	-0.17441171	1.49E-07	0.001173757
TSS200	OR8B12	11	124413574/124413728	2	cg03168749/cg25362709	0.720342223	0.607519542	0.452668577	-0.154850964	1.50E-04	1.06E-02
TSS200	PABPC4L	4	135122993	1	cg11815708	0.307129375	0.367251017	0.543101264	0.175850247	5.72E-04	1.44E-02
TSS200	PALM2	9	112402882	1	cg02442436	0.23853382	0.350415644	0.511096645	0.160681001	1.56E-04	1.06E-02
TSS200	PAQR9	3	142682288/142682291	2	cg15852572/cg27406373	0.081945404	0.091917814	0.253962734	0.16204492	4.99E-04	1.38E-02
TSS200	PCDH10	4	134070437/134070369/134070433/134070389/134070441/134070416	6	cg01408654/cg02562431/cg10524033/cg12746059/cg14400886/cg27600205	0.277331374	0.356228214	0.532015105	0.17578689	1.58E-03	2.32E-02
TSS200	PCDHGC4	5	140864578/140864593/140864701/140864549/14086473	5	cg02022733/cg03640756/cg15361590/cg15949044/cg23445461	0.296129086	0.459798741	0.667165305	0.207366564	2.31E-05	6.89E-03
TSS200	PCP4L1	1	161228455/161228496	2	cg10775792/cg27223727	0.101200112	0.089806782	0.263402179	0.173595397	1.15E-04	9.92E-03

TSS200	PDGFD	11	104035151/104035204/104035136/104035087	4	cg03145963/cg08596000/cg12027636/cg26756506	0.108127433	0.206495605	0.362922495	0.15642689	3.67E-03	3.71E-02
TSS200	PDPN	1	13910224/13910206/13910138/13910164/13910209/13910156	6	cg02933679/cg10743104/cg11791751/cg15563963/cg16277479/cg22436123	0.257968218	0.342812772	0.504722091	0.161909319	1.60E-03	2.34E-02
TSS200	PLCB1	20	8113191	1	cg27591117	0.198205965	0.268068172	0.419899173	0.151831001	2.17E-03	2.69E-02
TSS200	PTF1A	10	23481421/23481389/23481385	3	cg17076890/cg18920423/cg20014049	0.270220597	0.266804783	0.422044574	0.155239791	6.84E-04	1.54E-02
TSS200	PTPRT	20	41818756/41818574	2	cg08413157/cg27625055	0.126841351	0.20140185	0.363025578	0.161623728	4.04E-04	1.30E-02
TSS200	RALYL	8	85095275/85097056/85095498/85095394/85097031/85097049	6	cg00266009/cg04842146/cg05716166/cg16020747/cg21671806/cg23649708	0.300238937	0.341182228	0.527346692	0.186164464	1.77E-04	1.06E-02
TSS200	RASGRF1	15	79383413/79383385	2	cg15156078/cg19431448	0.196443749	0.311090539	0.479263118	0.168172579	2.51E-04	1.12E-02
TSS200	RNF165	18	43914047	1	cg09730015	0.19832376	0.236244739	0.403254159	0.16700942	6.89E-04	1.54E-02
TSS200	RSPO4	20	983104/983002/982991	3	cg05336115/cg19937938/cg23697417	0.219634654	0.173822182	0.329729948	0.155907766	5.21E-04	1.40E-02
TSS200	SHISA9	16	12995292/12995300/12995296	3	cg02860732/cg03057083/cg04342955	0.259598656	0.369636269	0.595409569	0.2257733	2.06E-03	2.63E-02
TSS200	SIM1	6	100911701/100911746/100911687/100911727/100911709/100911744	6	cg04927931/cg08133486/cg11471772/cg11891393/cg21063722/cg22478310	0.31500923	0.3846802	0.592480535	0.207800335	2.49E-05	6.89E-03
TSS200	SLC18A2	10	119000638	1	cg15173134	0.378897845	0.376248644	0.554818941	0.178570296	1.83E-03	2.49E-02
TSS200	SLC22A17	14	23821596/23821570/23822265	3	cg15476528/cg23411440/cg23464698	0.17715308	0.178381296	0.343550598	0.165169303	6.40E-04	1.51E-02
TSS200	SLC27A6	5	128301136/128301156/128301185/128301083/12830114	5	cg07421806/cg09538995/cg13985726/cg14441976/cg15899948	0.229054425	0.288089102	0.458344828	0.170255726	3.57E-03	3.66E-02
TSS200	SLC5A7	2	108602906/108602860/108602931/108602937/10860297	5	cg05311412/cg11355135/cg14613271/cg18771173/cg26001902	0.412205918	0.439223517	0.638806236	0.19958272	3.99E-04	1.29E-02
TSS200	SLC6A1	3	11034279/11034311/11034345/11034281	4	cg11021744/cg15445554/cg16164276/cg23405575	0.262292329	0.307416941	0.515257988	0.207841046	1.25E-03	2.02E-02
TSS200	SLC6A3	5	1445561/1445593/1445549/1445567	4	cg04210284/cg05030481/cg12882697/cg27037018	0.250250824	0.253240823	0.419007345	0.165766523	2.53E-03	2.94E-02
TSS200	SLCO4C1	5	101632341/101632286/101632321/101632372/101632327/101632310/1016	7	cg04621020/cg04633600/cg06480736/cg11009817/cg11267955/cg19788741/cg2214	0.094948704	0.147271233	0.314547799	0.167276567	5.32E-03	4.66E-02
TSS200	SLIT2	4	20255082/20255061/20255187	3	cg13078140/cg13281139/cg15469350	0.337305058	0.451698381	0.642086455	0.190388074	1.70E-04	1.06E-02
TSS200	SLIT3	5	168728193/168728213/168728270/168728149/16872823	5	cg02403292/cg14658804/cg15637465/cg15927720/cg26492514	0.339987309	0.422433059	0.582153003	0.159719943	2.66E-03	3.02E-02
TSS200	SLITRK5	13	88324711	1	cg17967577	0.27742316	0.270804299	0.542421245	0.271616946	7.81E-05	9.06E-03

TSS200	SNAP91	6	84419244/84419202/84419189/84419234/84419242/84419237	6	cg09227138/cg17767285/cg18213472/cg19671533/cg20631014/cg24842733	0.291554167	0.335079723	0.497929821	0.162850099	0.00132989	0.021027561
TSS200	SORCS3	10	106400686/106400824/106400702/106400667	4	cg03958798/cg08495770/cg10601616/cg20482521	0.309774398	0.482957725	0.657551995	0.17459427	1.97E-03	2.58E-02
TSS200	SOX21	13	95364514/95364569/95364586/95364510	4	cg01212887/cg02736420/cg16201674/cg23111830	0.413740165	0.420654	0.611093993	0.190439993	7.51E-04	1.59E-02
TSS200	SPHKAP	2	229046406/229046515	2	cg06092815/cg10959353	0.426582138	0.506556308	0.66497103	0.158414721	2.88E-05	6.89E-03
TSS200	SPOCK3	4	168155888/168155853	2	cg05158197/cg22255664	0.134234852	0.149327418	0.311082549	0.161755131	2.94E-04	1.17E-02
TSS200	SPSB4	3	140770588/140770608/140770617/140770549/140770683/140770599	6	cg04353095/cg05258261/cg15562912/cg19443257/cg20979852/cg27515369	0.144978153	0.212109015	0.376636693	0.164527678	5.68E-03	4.86E-02
TSS200	SSTR2	17	71161157	1	cg22277994	0.282628835	0.36149265	0.522970159	0.161477509	2.04E-03	2.63E-02
TSS200	SUNC1	7	48068758/48068728	2	cg03609427/cg07298482	0.699570785	0.555518042	0.383530455	-0.171987587	2.82E-06	4.51E-03
TSS200	SUSD5	3	33260743/33260732/33260752	3	cg02239862/cg04545136/cg13601427	0.232784649	0.387156452	0.541407395	0.154250944	2.00E-03	2.60E-02
TSS200	SYT6	1	114696671/114696641	2	cg01567634/cg21920775	0.229697972	0.279092855	0.456830873	0.177738017	0.000151467	0.010619412
TSS200	TAS1R2	1	19186245/19186260	2	cg07947459/cg20678988	0.757506495	0.608475239	0.437779341	-0.170695898	2.79E-05	0.006885757
TSS200	TBX15	1	119532189/119532352/119532195/119532320	4	cg05940231/cg12664119/cg25340966/cg26272623	0.366163911	0.455126192	0.618629788	0.163503595	2.26E-05	6.89E-03
TSS200	TCP10L	21	33957957/33957846	2	cg08949101/cg15713719	0.669835563	0.550494103	0.381136684	-0.169357419	6.03E-07	2.66E-03
TSS200	TFPI2	7	93520183/93520074/93520175/93520172	4	cg14377593/cg16934178/cg22441533/cg26739865	0.108107687	0.218527103	0.371475189	0.152948086	5.46E-03	4.73E-02
TSS200	TLX1	10	102891045/102890984/102890974/102890886/102890986/102891019	6	cg02567119/cg03020810/cg08908184/cg11196237/cg18964775/cg25741023	0.180028495	0.168964721	0.331625611	0.162660889	1.00E-03	1.83E-02
TSS200	TLX1NB	10	102891076/102891045/102890984/102890974/102890986/102891019/1028[illegible]	7	cg02096397/cg02567119/cg03020810/cg08908184/cg18964775/cg25741023/cg2727[illegible]	0.17461684	0.155415108	0.326264762	0.170849655	8.65E-04	1.71E-02
TSS200	TM6SF1	15	83776271/83776269	2	cg08452658/cg11167100	0.151241291	0.376860035	0.565246468	0.188386433	8.18E-04	1.65E-02
TSS200	TMEM108	3	132757106/132756991/132757087/132756986/132757090/132757102	6	cg02745211/cg05075118/cg06288251/cg17385936/cg18115215/cg24198558	0.335006086	0.438743076	0.612661876	0.173918801	3.18E-04	1.21E-02
TSS200	TMEM132D	12	130388216/130388234	2	cg12122146/cg23891360	0.432046123	0.547361186	0.713278861	0.165917675	1.33E-04	1.04E-02
TSS200	TMEM132E	17	32907639/32907705/32907636/32907731	4	cg03442425/cg10451253/cg21745612/cg27213352	0.330240289	0.433951689	0.587126273	0.153174584	1.86E-03	2.51E-02

TSS200	TMEM155	4	122686432/122686453/122686493/122686456	4	cg03227184/cg04638468/cg07978472/cg08553437	0.299777159	0.451787739	0.640223931	0.188436192	2.38E-04	0.011236785
TSS200	TMEM163	2	135476601/135476701/135476679	3	cg03556069/cg19589919/cg23057992	0.068451525	0.086850362	0.236914525	0.150064162	1.46E-03	2.21E-02
TSS200	TMEM26	10	63213391/63213385/63213396/63213339	4	cg00360414/cg06067842/cg12160258/cg18286501	0.281732012	0.289902709	0.452608389	0.16270568	2.37E-04	1.12E-02
TSS200	TMEM90A	14	74892986	1	cg06204735	0.359359645	0.4851486	0.670560677	0.185412077	1.06E-03	1.89E-02
TSS200	TREML3	6	41185715	1	cg05143424	0.806213305	0.763482017	0.578187677	-0.185294339	1.23E-04	9.92E-03
TSS200	TRIM71	3	32859445/32859407/32859377/32859417/32859438	5	cg17029062/cg19127283/cg21124497/cg21484228/cg23528400	0.177171096	0.312241233	0.489104515	0.176863282	2.38E-03	2.84E-02
TSS200	TRPC6	11	101454733/101454683/101454765/101454823/101454676/101454680	6	cg04553838/cg07013734/cg14186937/cg23644960/cg23650423/cg27296293	0.334916158	0.415615892	0.60046499	0.184849098	9.60E-05	9.77E-03
TSS200	TRPC7	5	135701240/135701184	2	cg22770135/cg23409403	0.408309693	0.504725928	0.661992732	0.157266804	1.24E-04	0.009915092
TSS200	TUSC3	8	15397637	1	cg16243756	0.34866517	0.475554461	0.627883064	0.152328603	1.87E-03	2.52E-02
TSS200	TWIST1	7	19157296/19157304/19157420/19157339	4	cg00240432/cg09799658/cg10420952/cg12926104	0.124634029	0.213812571	0.414031915	0.200219343	7.03E-04	1.54E-02
TSS200	UBQLNL	11	5538030	1	cg09232851	0.76521776	0.69953415	0.536760745	-0.162773405	6.19E-04	1.49E-02
TSS200	UNC80	2	210636560/210636533/210636539	3	cg04100532/cg09438147/cg24938830	0.38753957	0.459278506	0.628306471	0.169027966	2.09E-03	2.65E-02
TSS200	UNCX	7	1272545/1272512/1272559/1272515/1272526/1272486	6	cg05157140/cg08507422/cg17294725/cg20870512/cg21158633/cg21543987	0.340667745	0.361306234	0.521764314	0.160458079	4.83E-04	1.37E-02
TSS200	VCAN	5	82767394/82767297/82767450	3	cg02551029/cg19540689/cg21810188	0.130012098	0.174571956	0.370658713	0.196086757	7.20E-04	1.55E-02
TSS200	VSX1	20	25062827/25062771/25062777/25062860/25062823/25062819	6	cg01715455/cg01941895/cg02631468/cg03111498/cg15272362/cg16185834	0.327231481	0.389951827	0.576211427	0.1862596	4.65E-05	0.007874782
TSS200	VSX2	14	74706113/74706031/74706016/74706062/74706074	5	cg09816693/cg12827555/cg15416329/cg21057587/cg23118730	0.330851714	0.373553735	0.534947252	0.161393518	3.69E-05	7.18E-03
TSS200	VWC2	7	49813088/49813102/49813111/49813065	4	cg01893212/cg02467990/cg09493505/cg14045872	0.186555477	0.208329984	0.45091293	0.242582946	2.02E-03	2.62E-02
TSS200	WDR86	7	151107285/151107257/151107261	3	cg10770742/cg17362861/cg24068372	0.258717153	0.351823141	0.541493242	0.189670102	1.66E-03	2.37E-02
TSS200	ZFR2	19	3869086/3869172/3869174	3	cg13786089/cg15823845/cg24876786	0.229491011	0.334829491	0.581684005	0.246854514	1.74E-04	1.06E-02
TSS200	ZNF256	19	58459218/58459142/58459139/58459136/58459097/58459248	6	cg05119514/cg12555306/cg16246961/cg22731359/cg23484087/cg27388983	0.101489456	0.147795324	0.335643988	0.187848664	3.92E-04	1.29E-02
TSS200	ZNF264	19	57702809/57702790/57702772/57702697/57702803/57702793	6	cg12096988/cg15257259/cg18576158/cg20715764/cg25800082/cg26970847	0.045250513	0.0991774	0.299380467	0.200203067	4.25E-03	4.05E-02

TSS200	ZNF347	19	53662467/53662439/53662353	3	cg11081729/cg23052615/cg27209110	0.035441866	0.061775464	0.257478493	0.195703029	2.26E-03	2.74E-02
TSS200	ZNF454	5	178368071/178368123/178368160/178368185/178368205/178368183	6	cg02165355/cg03234732/cg10575261/cg16536329/cg20778451/cg23037403	0.412298729	0.466807437	0.627704703	0.160897266	1.79E-04	1.06E-02
TSS200	ZNF492	19	22816980/22817096/22817017/22817039/22816950	5	cg05241461/cg06748831/cg11785166/cg23217946/cg24889512	0.29730671	0.401194691	0.561396058	0.160201367	2.50E-04	1.12E-02
TSS200	ZNF542	19	56879662/56879432/56879369/56879554/56879559/56879418/56879571/56879613/56879645	9	cg03146949/cg06942685/cg10638415/cg15708153/cg18787401/cg21493505/cg26309134/cg27062795/cg	0.311414553	0.421541164	0.586709571	0.165168407	1.83E-04	1.06E-02
TSS200	ZNF549	19	58038689/58038573/58038585/58038633/58038588/58038621	6	cg01601746/cg06458239/cg10729426/cg16697731/cg19060970/cg20607331	0.285128359	0.456519629	0.650572955	0.194053326	2.47E-03	2.89E-02
TSS200	ZNF586	19	58280832/58280891/58280927/58281016/58281019/58280994	6	cg03584535/cg07685728/cg13636880/cg18575209/cg20751795/cg24298255	0.138086232	0.14550535	0.337383294	0.191877944	4.92E-03	4.44E-02
TSS200	ZNF671	19	58239012/58239135	2	cg08048222/cg24016939	0.220574027	0.315750092	0.473614271	0.15786418	2.37E-03	2.83E-02
TSS200	ZNF677	19	53758289/53758297/53758233/53758309	4	cg06506966/cg09379485/cg24114314/cg25404339	0.141947758	0.240661004	0.43732722	0.196666216	0.003849126	0.038304172
TSS200	ZNF75A	16	3355361/3355411/3355295	3	cg03004280/cg07306531/cg16480692	0.240135958	0.370008164	0.553979262	0.183971098	1.13E-03	1.94E-02
TSS200	ZNF788	19	12203066/12203029	2	cg09489445/cg20773651	0.156508032	0.269657983	0.497029508	0.227371525	1.00E-03	1.83E-02
TSS200	ZNF814	19	58400494/58400574/58400559/58400504	4	cg15746696/cg18860954/cg25400396/cg27112264	0.34868974	0.438296465	0.601844211	0.163547746	6.16E-04	1.49E-02
TSS200	ZNF85	19	21106043/21106016/21106027/21106036/21106053/21106002	6	cg05310990/cg15690721/cg19381810/cg21448033/cg23216292/cg25464210	0.283905038	0.392508137	0.572298856	0.179790719	5.85E-05	8.40E-03
TSS200	ZSCAN23	6	28411279/28411423/28411287/28411285/28411378/28411309	6	cg00651523/cg07836142/cg09682330/cg14547644/cg21486944/cg24703168	0.17786891	0.239535867	0.410736147	0.17120028	2.27E-04	1.12E-02
TSS200	ZSWIM2	2	187713986/187713964/187713902/187714068/187714055/187714003	6	cg05907046/cg07548607/cg10396546/cg14762973/cg16516691/cg24617723	0.238870902	0.309867499	0.478734976	0.168867478	6.28E-04	1.50E-02
UTR5	ADAMTS18	16	77468893/77468850	2	cg03238797/cg07568194	0.242036425	0.243960726	0.416279982	0.172319255	2.98E-03	0.029677392
UTR5	ADAMTS5	21	28338836/28339334/28339262	3	cg03202077/cg15237494/cg23986671	0.33495262	0.433389163	0.603467985	0.170078822	3.21E-04	0.012161575
UTR5	ADARB2	10	1779432/1779667	2	cg00467420/cg05307923	0.152456797	0.280145489	0.436033709	0.155888219	0.003017844	0.029761028
UTR5	ADCY8	8	132052779/132052702	2	cg03120091/cg05229355	0.291294298	0.390955817	0.553517995	0.162562179	2.16E-04	0.010992872

UTR5	ADHFE1	8	67344720	1	cg20912169	0.256459935	0.411193623	0.660422359	0.249228736	7.99E-05	9.15E-03
UTR5	AEBP1	7	44143998/44143993	2	cg02126753/cg14249876	0.1369801	0.257681438	0.504188142	0.246506704	0.000486398	0.013444565
UTR5	ALG1L	3	125655381/125655407	2	cg05084668/cg07473553	0.68058229	0.463321817	0.311121282	-0.152200535	0.00117478	0.01849533
UTR5	AMPH	7	38670957/38670987/38670985/38671001	4	cg02383130/cg07926691/cg10293925/cg26122980	0.220771274	0.30209	0.49313757	0.19104757	2.28E-03	0.025897156
UTR5	AQP5	12	50355307	1	cg15336765	0.174818933	0.236789049	0.489375127	0.252586078	1.67E-05	0.008208786
UTR5	ASB15	7	123249130/123252579	2	cg02300083/cg26067096	0.61231217	0.532766017	0.371533668	-0.161232348	4.67E-04	1.33E-02
UTR5	BARHL2	1	91182777	1	cg18322569	0.486139255	0.506292575	0.753573723	0.247281148	1.35E-03	0.019749219
UTR5	BCAT1	12	25102274/25102072	2	cg04011247/cg10764357	0.202790783	0.308302367	0.516316832	0.208014465	3.81E-05	0.008208786
UTR5	C11orf87	11	109293614/109293070/109292894/109293216/109294168/109294233/109293945/109294044/109293068/109294100/109294060	11	cg00830817/cg04861929/cg06719900/cg07142201/cg07180307/cg07428323/cg10454766/cg13952556/cg15948088/cg21852408/cg26014266	0.293567021	0.379175347	0.557336049	0.178160702	3.15E-05	0.008208786
UTR5	C14orf39	14	60952097/60952599/60952196/60952405/60952738	5	cg05255275/cg18240143/cg18921771/cg23989821/cg27398547	0.28767244	0.40725815	0.622664336	0.215406186	2.71E-05	0.008208786
UTR5	C17orf104	17	42733976	1	cg22190438	0.175055483	0.221011376	0.410953534	0.189942158	0.00017908	0.010630001
UTR5	C1orf173	1	75139347/75139364	2	cg24155871/cg27393126	0.295957565	0.367192178	0.573761716	0.206569538	8.54E-04	0.016216997
UTR5	C1orf70	1	1475737	1	cg16601494	0.18564747	0.361693367	0.665361532	0.303668165	1.33E-04	0.010271313
UTR5	C8orf85	8	117950473	1	cg05996052	0.367959095	0.481860539	0.645422682	0.163562143	4.22E-05	8.21E-03
UTR5	CACNA1A	19	13617094/13617091	2	cg17509967/cg22491927	0.24286006	0.353768224	0.512181286	0.158413062	4.24E-03	0.035960386
UTR5	CACNG3	16	24267226/24267399/24267559/24267526/24266905	5	cg02692912/cg04441477/cg04543289/cg07514158/cg17234150	0.338346394	0.442153518	0.596171655	0.154018137	5.15E-04	1.36E-02
UTR5	CACNG8	19	54466297	1	cg08653692	0.27789309	0.375280813	0.674200927	0.298920114	6.52E-05	0.008697427
UTR5	CBLN2	18	70210483/70210433/70209432/70209551/70210025/70210955/70210494/70210819/70211515	9	cg01908010/cg03561455/cg13478228/cg15733692/cg17952513/cg18596362/cg19202058/cg21529405/cg	0.239248293	0.344807051	0.495925716	0.151118665	6.16E-04	1.47E-02
UTR5	CBLN4	20	54579355/54579814	2	cg02501779/cg14184078	0.51584526	0.547781811	0.713582132	0.165800321	6.57E-06	0.008208786
UTR5	CCDC105	19	15121591/15121596	2	cg03928546/cg06522681	0.439871208	0.515696853	0.689640884	0.173944031	4.16E-05	0.008208786
UTR5	CCDC37	3	126113784/126113918/126114064	3	cg00891278/cg19628148/cg20707970	0.357535978	0.414504994	0.58240912	0.167904126	2.85E-04	1.17E-02
UTR5	CD34	1	208084456/208084640/208084436	3	cg04292718/cg19591881/cg21455438	0.243883901	0.37397278	0.541480049	0.16750727	0.002642359	0.02787509
UTR5	CDH2	18	25757211/25757438/25757202	3	cg09313439/cg12208258/cg13464915	0.291554003	0.338988742	0.495758347	0.156769605	2.49E-03	2.71E-02
UTR5	CDO1	5	115152386/115152326	2	cg12880658/cg16265906	0.259336185	0.425709006	0.578118532	0.152409526	0.002925677	0.029403885

UTR5	CHST2	3	142838847/142839578/142838938/142839022/14283868	5	cg00995327/cg08774368/cg08858437/cg09638407/cg17815252	0.091566806	0.197840252	0.359917922	0.162077669	0.002652779	0.027913807
UTR5	CPA4	7	129933014	1	cg01796223	0.58549644	0.538058156	0.374964577	-0.163093578	2.33E-04	0.010992872
UTR5	CR1L	1	207818493	1	cg24557058	0.25046316	0.380392383	0.543904982	0.163512598	4.13E-03	3.55E-02
UTR5	DNAH11	7	21582834	1	cg05285902	0.182962681	0.209432983	0.381891586	0.172458603	0.00300383	0.029749981
UTR5	DOK5	20	53092474/53092334	2	cg00055557/cg20107395	0.245197855	0.316964298	0.471235526	0.154271228	3.91E-03	3.43E-02
UTR5	DTX3	12	57998762/57998786/57998656	3	cg11654179/cg17730484/cg19217692	0.092657477	0.100876562	0.279461681	0.178585119	1.96E-03	0.023830755
UTR5	EBF1	5	158526693/158526614/158526642	3	cg04217450/cg05530568/cg17009297	0.283888828	0.337726565	0.504129801	0.166403236	2.24E-03	2.56E-02
UTR5	EPHA10	1	38230769/38230779	2	cg12128017/cg20510207	0.302970382	0.384643194	0.540645805	0.15600261	1.99E-03	0.023998112
UTR5	EVC	4	5713100/5712937/5713005	3	cg16418810/cg17460447/cg21210758	0.246805393	0.344737494	0.514194583	0.169457089	0.000376942	0.012730027
UTR5	FADS2	11	61595807	1	cg25303599	0.0880115	0.159008576	0.40492206	0.245913483	6.65E-04	0.014924788
UTR5	FAM123A	13	25745768/25745784	2	cg14880499/cg22029275	0.23601127	0.382200712	0.570263075	0.188062363	0.001209544	0.018724853
UTR5	FAM155A	13	108519213/108518955/108519318	3	cg04607400/cg05383490/cg19839798	0.331693743	0.384843347	0.596236664	0.211393316	1.10E-04	0.009896234
UTR5	FAM159B	5	63986392	1	cg10981909	0.354039175	0.485957006	0.671355627	0.185398622	0.001920078	0.023577238
UTR5	FAM162B	6	117086850	1	cg07641160	0.204753153	0.284185165	0.499210776	0.215025611	1.80E-03	2.28E-02
UTR5	FAM43B	1	20879029/20879286/20879296	3	cg03754311/cg05771261/cg20404387	0.292133723	0.381013617	0.551905216	0.170891599	6.79E-04	1.50E-02
UTR5	FBN2	5	127873397/127873552/127873711	3	cg12940822/cg17958315/cg25084878	0.205810473	0.311272031	0.51417982	0.202907789	1.75E-03	2.25E-02
UTR5	FBXL7	5	15500714	1	cg06577205	0.344421575	0.375808717	0.528633736	0.15282502	3.82E-03	0.033764732
UTR5	FGF4	11	69590090/69590113	2	cg19831575/cg23964057	0.151178155	0.239285329	0.42840766	0.189122331	0.001781228	0.022776365
UTR5	FGF5	4	81187798/81187906	2	cg02002231/cg14197923	0.106380913	0.196157855	0.391203448	0.195045593	5.27E-03	0.041238007
UTR5	FLG2	1	152331529	1	cg20694572	0.7220716	0.610989517	0.441182641	-0.169806876	0.000166714	0.010357405
UTR5	FOLH1	11	49230168/49230036	2	cg14775560/cg25346915	0.291977923	0.319281246	0.501149359	0.181868113	0.000494258	0.013447649
UTR5	FOXG1	14	29236323/29236476	2	cg10300684/cg10828337	0.355856943	0.45415355	0.627157636	0.173004086	1.06E-04	9.72E-03
UTR5	GAD2	10	26505442/26505503/26505440/26505245	4	cg04599297/cg09056181/cg11328303/cg15850954	0.277074423	0.44180043	0.613517622	0.171717191	6.95E-04	1.52E-02
UTR5	GALNT13	2	154729200/154728735/154728468/154729059/154729917/154729995/154729622/154730326/154730157/154729[illegible]	11	cg01011748/cg10033761/cg12079322/cg13101087/cg14630357/cg17130964/cg18118685/cg23762263/cg24412079/cg25703346[illegible]	0.32878657	0.345853486	0.537751066	0.19189758	2.23E-05	0.008208786
UTR5	GALNTL6	4	172734776/172734843/172734718	3	cg03413465/cg15533075/cg26588943	0.276865617	0.363287785	0.517858048	0.154570263	0.000294816	0.012031188

UTR5	GATA4	8	11562367/11562685/11565530/11565277/11561724/11561728/11562155/11565725/11563023/11565351	10	cg00090147/cg03007522/cg06704518/cg14487356/cg14666113/cg18283386/cg21073927/cg24646414/cg24738627/cg2521669	0.304089279	0.399121798	0.562679571	0.163557773	4.67E-05	0.008208786
UTR5	GDF6	8	97172961	1	cg00421139	0.25141105	0.384316417	0.55991715	0.175600733	0.003315762	0.031326458
UTR5	GFRA1	10	118032626/118032355/118032033/118031864/118032892/118032872/118033115/118031950/118032081/118032879/118032330/118031870/118032948/118031632/1180	17	cg03503087/cg04983142/cg06039355/cg06298519/cg08194313/cg13346013/cg13890706/cg17256532/cg18044663/cg18672939/cg19236679/cg19485539/cg23898073/cg24792682/cg250226	0.212450643	0.305961437	0.49126561	0.185304173	5.37E-03	0.041681605
UTR5	GLRA3	4	175750373/175750109/175750280	3	cg05424071/cg21094669/cg27517702	0.22069827	0.294578594	0.467080559	0.172501965	0.000228494	0.010992872
UTR5	GLRB	4	157997526/157997363/157998201/157997750/157997554/157997880/157997367/157997360	8	cg08712866/cg09688546/cg10146199/cg10999932/cg11163620/cg13828794/cg16269431/cg22065614	0.223893995	0.308681188	0.499797991	0.191116803	0.000954663	0.016992183
UTR5	GPC6	13	93879544/93879361/93879670/93879303	4	cg12989574/cg16792800/cg18058689/cg19824907	0.36752897	0.43732878	0.647716442	0.210387663	7.79E-05	0.009151315
UTR5	GPR83	11	94134446/94134580	2	cg03345037/cg07586272	0.23907202	0.323759189	0.475437886	0.151678697	0.002275376	0.025897156
UTR5	GRIA1	5	152870258	1	cg17020834	0.227224285	0.232838994	0.383978405	0.15113941	0.000363786	0.012730027
UTR5	GRIA4	11	105481306/105481317/105480979/105481319/105481322/105481283/105481509/105481406/105481292	9	cg00343633/cg03225817/cg03243226/cg04747226/cg07972135/cg15603568/cg19343464/cg21217024/cg23559689	0.280322231	0.413955547	0.601997234	0.188041687	0.000500896	0.01350896
UTR5	GRM7	3	6902824/6902845/6903019	3	cg17892178/cg18863595/cg27199820	0.435273323	0.5295267	0.687263277	0.157736577	6.38E-05	0.008692664
UTR5	HBQ1	16	230343/230341	2	cg02401454/cg07703401	0.32724683	0.338138094	0.520321184	0.18218309	0.000526728	0.013786045
UTR5	HCRTR2	6	55039232/55039259/55039382/55039295	4	cg10725720/cg13948987/cg17240815/cg27596495	0.282031535	0.328409048	0.488993624	0.160584575	0.000122578	0.010093278
UTR5	HMX2	10	124907708	1	cg19909787	0.159048506	0.238984549	0.40401015	0.165025601	0.003503653	0.032087739
UTR5	HOXC9	12	54393966/54393971	2	cg05069426/cg12071536	0.28800951	0.364417672	0.572085011	0.207667339	0.000550065	0.013926752
UTR5	HTR6	1	19992024/19992167	2	cg10741760/cg27549720	0.546786515	0.527339867	0.686340005	0.159000138	0.001085237	0.018115128
UTR5	HTR7	10	92617429/92617562	2	cg15364725/cg26135506	0.201898507	0.225477729	0.384857195	0.159379466	0.001960765	0.023830755
UTR5	IRAK3	12	66583029/66583048	2	cg01263292/cg26415547	0.088190073	0.150723497	0.330097085	0.179373588	0.002578868	0.027628182

UTR5	IRF4	6	391936/392555/393110/392131/391764	5	cg06392169/cg12612118/cg12684209/cg12741420/cg17228900	0.237617969	0.435077595	0.610867689	0.175790094	0.002040948	0.024252879
UTR5	ITGA4	2	182322268/182322058/182321855/182322279/18232178	5	cg06952671/cg10526659/cg20415809/cg21995919/cg25652029	0.264610925	0.464160694	0.63316878	0.169008085	0.001959557	0.023830755
UTR5	KCNA1	12	5019933/5019311/5019482/5019091/5020169/5020202	6	cg06204922/cg09487611/cg10363246/cg12908522/cg16907029/cg26590537	0.213254822	0.335016138	0.536760808	0.201744671	0.000530447	0.013802092
UTR5	KRT7	12	52627047	1	cg09670128	0.37070131	0.531059411	0.734100745	0.203041334	0.000562192	0.014030238
UTR5	LAMA1	18	7117790	1	cg18764577	0.33890274	0.440777189	0.599022482	0.158245293	4.05E-05	0.008208786
UTR5	LCE1B	1	152784914	1	cg07090653	0.729669125	0.6528083	0.479028127	-0.173780173	0.000359289	0.012730027
UTR5	LCE3D	1	152552494	1	cg20676475	0.71794003	0.551679256	0.380826986	-0.170852269	0.000131054	0.010271313
UTR5	LIN28B	6	105405068	1	cg04560456	0.330428215	0.378783794	0.543564627	0.164780833	6.63E-04	1.49E-02
UTR5	LOC728392	17	5404508/5404330/5404337	3	cg06462347/cg22298430/cg27230784	0.06618335	0.104406035	0.269477279	0.165071244	0.003555949	0.032357898
UTR5	LUZP2	11	24518737/24518776	2	cg06931933/cg09717526	0.277360133	0.309575603	0.460539434	0.150963831	1.11E-03	1.82E-02
UTR5	MDFI	6	41606317/41606439	2	cg01350077/cg27200446	0.134761777	0.262817074	0.459091135	0.196274061	4.17E-03	3.57E-02
UTR5	MMD2	7	4998714/4998649	2	cg26468007/cg27603283	0.263741423	0.340103578	0.49909633	0.158992752	9.47E-04	1.69E-02
UTR5	MRGPRX3	11	18157898/18157405	2	cg07656579/cg10378819	0.802569685	0.719069706	0.548679564	-0.170390142	1.95E-04	1.09E-02
UTR5	NEFL	8	24814126/24814035	2	cg22978087/cg24032666	0.368008958	0.387229822	0.542913533	0.15568371	3.59E-03	3.25E-02
UTR5	NEUROG3	10	71333037/71332804	2	cg03425110/cg17583413	0.225495702	0.331307686	0.573642898	0.242335212	1.53E-05	8.21E-03
UTR5	NGB	14	77737621/77737495	2	cg02181639/cg02881570	0.375804095	0.441153767	0.623488245	0.182334479	9.01E-04	1.66E-02
UTR5	NKX2-2	20	21494547	1	cg17029168	0.2650935	0.329316172	0.497733132	0.16841696	2.74E-03	2.83E-02
UTR5	NOVA1	14	27066771	1	cg15602241	0.21546241	0.203697178	0.385430986	0.181733809	4.06E-04	1.29E-02
UTR5	NPR3	5	32711881	1	cg01252672	0.087079092	0.174021886	0.459977908	0.285956023	1.49E-04	1.03E-02
UTR5	OLIG2	21	34398532/34398263/34398849/34398265	4	cg05724110/cg09793121/cg14843922/cg22869726	0.317999256	0.411034344	0.58256032	0.171525976	1.51E-04	1.03E-02
UTR5	OLIG3	6	137815333	1	cg03889226	0.18031487	0.24152574	0.411535868	0.170010128	1.03E-03	1.77E-02
UTR5	PCDH17	13	58206058/58206036	2	cg00208153/cg14893163	0.31001712	0.411174189	0.570123352	0.158949163	1.24E-04	1.01E-02
UTR5	PCP4L1	1	161228674	1	cg11912330	0.16781842	0.160256398	0.392305085	0.232048687	3.05E-04	1.22E-02
UTR5	PDE4A	19	10531434	1	cg23103993	0.095087647	0.097623676	0.362455849	0.264832173	0.000155999	0.010302252
UTR5	PDGFD	11	104034754/104034982	2	cg02120582/cg18403606	0.049574196	0.177355176	0.363817949	0.186462773	3.73E-03	3.32E-02
UTR5	PHOX2A	11	71955113/71955164	2	cg13103303/cg19651694	0.364023335	0.428198539	0.596162923	0.167964384	1.38E-04	1.03E-02
UTR5	POM121L2	6	27280010	1	cg07085962	0.085615451	0.126727028	0.278740917	0.152013889	7.17E-03	4.92E-02
UTR5	PPP2R2C	4	6473964	1	cg18013519	0.07588347	0.151003771	0.353179632	0.202175862	1.32E-03	1.96E-02
UTR5	PRIMA1	14	94254351/94254726/94254586/94254131	4	cg06133145/cg10002178/cg20895877/cg23889730	0.349452212	0.421909473	0.624263842	0.202354369	4.08E-06	8.21E-03

UTR5	RSPO2	8	109095568/109095572/109094880/109095264/109095782/109095388	6	cg00910695/cg04050867/cg07390122/cg14070647/cg14733048/cg20061155	0.274606971	0.358213946	0.512446105	0.154232158	9.80E-04	1.72E-02
UTR5	RYR1	19	38924347/38924349/38924436	3	cg04571321/cg05471297/cg06740600	0.19628607	0.287594851	0.464376086	0.176781235	5.19E-03	4.09E-02
UTR5	SCGB2A2	11	62037659	1	cg22862656	0.81272038	0.609554289	0.447701014	-0.161853275	4.52E-04	1.33E-02
UTR5	SCTR	2	120281999/120281813	2	cg07176264/cg20505223	0.287517405	0.314268917	0.480911932	0.166643015	2.82E-03	2.88E-02
UTR5	SDC2	8	97506180/97506251	2	cg14538332/cg16935295	0.12978423	0.237232389	0.44108355	0.203851161	2.42E-03	2.68E-02
UTR5	SHISA9	16	12995568/12995603	2	cg02482218/cg04106647	0.223821843	0.279243776	0.446874112	0.167630336	1.32E-03	1.96E-02
UTR5	SLC35F3	1	234040765/234040781	2	cg05051043/cg09424759	0.30379271	0.357964728	0.510501209	0.152536481	0.000626188	0.01471305
UTR5	SLIT1	10	98945465/98945664	2	cg02741216/cg13196826	0.077506079	0.132029509	0.305303885	0.173274376	3.08E-03	0.030217212
UTR5	SLIT2	4	20255264/20255343	2	cg03742003/cg08428452	0.257782725	0.366029311	0.529762009	0.163732698	2.64E-03	2.79E-02
UTR5	SLITRK5	13	88326918/88324879/88325384/88326752/88326244/88325263/88326635	7	cg04479219/cg06682197/cg07710481/cg12271981/cg16821446/cg19109538/cg1957	0.225055161	0.301587606	0.457285961	0.155698355	3.01E-04	1.21E-02
UTR5	SMOC1	14	70346221/70346151	2	cg15996043/cg18493027	0.080132343	0.181771166	0.361537276	0.17976611	0.002662869	0.027972523
UTR5	SNAP91	6	84417933/84418659/84418789/84418724/84418433	5	cg07335294/cg15950068/cg16334314/cg21688264/cg24190603	0.314300099	0.403981004	0.583119889	0.179138885	2.02E-04	1.10E-02
UTR5	SNTG2	2	946648	1	cg09426834	0.305865165	0.362097389	0.540930227	0.178832838	1.97E-03	2.38E-02
UTR5	SORCS3	10	106400880	1	cg16787600	0.341068585	0.5001111	0.680446518	0.180335418	7.49E-04	1.56E-02
UTR5	SPAG4L	20	31592147	1	cg02510802	0.74023952	0.573631311	0.371162491	-0.20246882	2.43E-05	8.21E-03
UTR5	SPHKAP	2	229046325	1	cg04072323	0.285360715	0.389797139	0.577976023	0.188178884	9.10E-05	9.41E-03
UTR5	SPOCK1	5	136834877/136834294/136834863/136834492/136834453/136834392/1368	7	cg05143123/cg09054633/cg12832649/cg14650610/cg18263365/cg18603028/cg2484	0.263187511	0.34068157	0.506172196	0.165490626	3.07E-04	1.22E-02
UTR5	ST6GALNAC5	1	77333229/77333198	2	cg04077662/cg06201642	0.26808874	0.377675175	0.53033383	0.152658655	3.03E-03	2.98E-02
UTR5	ST8SIA1	12	22487437/22487459/22487219	3	cg09354309/cg10333808/cg24723331	0.135148496	0.178398841	0.346324339	0.167925498	3.60E-03	3.26E-02
UTR5	ST8SIA3	18	55019843	1	cg13096208	0.440201565	0.552931606	0.745647236	0.192715631	4.96E-05	8.21E-03
UTR5	STOX2	4	184826953/184827086/184827754/184827491/18482670	5	cg01758805/cg02071076/cg02500300/cg06367693/cg11354594	0.222166173	0.24050692	0.39943448	0.15892756	2.62E-03	2.78E-02
UTR5	SUNC1	7	48068665	1	cg23509098	0.70715429	0.561490989	0.376269341	-0.185221648	1.07E-05	8.21E-03
UTR5	SYCP2L	6	10887185	1	cg26237681	0.159986403	0.224231299	0.459437943	0.235206644	1.60E-04	1.04E-02
UTR5	TAC1	7	97361759/97361408	2	cg14221171/cg16288089	0.38903177	0.447987311	0.610351032	0.162363721	3.64E-03	3.27E-02
UTR5	TEKT1	17	6734991/6734940	2	cg13337865/cg20663042	0.189928838	0.21198984	0.389665511	0.177675671	4.80E-05	8.21E-03
UTR5	TFPI2	7	93520036/93520024/93520012	3	cg14775114/cg17338208/cg24531255	0.139304337	0.329598872	0.545375324	0.215776452	4.49E-04	1.33E-02

UTR5	TLL1	4	166794471/166794895/166794971/166794786	4	cg08570521/cg09669853/cg19898128/cg24521633	0.14149001	0.220108282	0.378027405	0.157919123	2.96E-03	2.96E-02
UTR5	TLX1	10	102891076/102891280/102891080	3	cg02096397/cg05329692/cg27272677	0.223234961	0.216491831	0.393620272	0.177128441	5.04E-04	1.35E-02
UTR5	TLX3	5	170736335	1	cg02743222	0.338009175	0.343386228	0.514287045	0.170900818	5.10E-03	4.05E-02
UTR5	TM6SF1	15	83776422/83776420	2	cg03063639/cg26460092	0.288529638	0.477104186	0.674643993	0.197539807	1.96E-04	1.09E-02
UTR5	TRH	3	129693613/129693586/129694057/129693791/129694487/129693385/129694156/129693370/129693489/129694	10	cg01009664/cg02700891/cg05013728/cg05835982/cg08195943/cg11940285/cg14595003/cg18862481/cg22512438/cg2293160	0.303001046	0.394063468	0.582192628	0.188129161	6.07E-05	8.64E-03
UTR5	TRPC6	11	101454317/101454626	2	cg03411507/cg11016563	0.33482509	0.403225172	0.58180788	0.178582707	4.76E-06	0.008208786
UTR5	TWIST1	7	19157193/19157263	2	cg17839237/cg24446548	0.083262939	0.206188919	0.519511387	0.313322468	3.06E-04	1.22E-02
UTR5	UMOD	16	20363009/20363711	2	cg04231019/cg07456201	0.656458963	0.488502464	0.322445711	-0.166056753	1.01E-04	9.61E-03
UTR5	VEGFC	4	177713813	1	cg13378388	0.29362454	0.326766511	0.49613375	0.169367239	5.39E-03	4.17E-02
UTR5	VIPR2	7	158937610/158937494	2	cg03976877/cg13794530	0.344609673	0.441673113	0.646611789	0.204938676	3.56E-05	8.21E-03
UTR5	VSX1	20	25062754	1	cg00043788	0.33244861	0.353225556	0.551050359	0.197824804	1.61E-05	8.21E-03
UTR5	VSX2	14	74706196	1	cg07573727	0.28435026	0.385045844	0.5782105	0.193164656	1.20E-03	1.87E-02
UTR5	VWC2	7	49813763/49814781/49814435/49814597/49813486	5	cg00333226/cg05470523/cg07039180/cg07379434/cg18206027	0.204754923	0.225809094	0.388402172	0.162593078	7.72E-04	1.56E-02
UTR5	WNT2	7	116963259/116963193	2	cg07697895/cg27331524	0.269514918	0.365072882	0.541466436	0.176393554	4.88E-04	1.34E-02
UTR5	ZIK1	19	58095659	1	cg26246807	0.375614655	0.597706644	0.758164927	0.160458283	1.39E-03	2.01E-02
UTR5	ZNF256	19	58458917/58458979	2	cg14556070/cg20176532	0.177109025	0.217259773	0.435286696	0.218026924	1.08E-03	1.80E-02
UTR5	ZNF264	19	57702917/57702927/57703144/57702994	4	cg08504049/cg22307908/cg23257840/cg24150232	0.064751927	0.119210851	0.293953594	0.174742743	6.34E-03	4.60E-02
UTR5	ZNF304	19	57862713	1	cg07494047	0.084922142	0.236218978	0.429114705	0.192895727	0.001614243	0.021577232
UTR5	ZNF492	19	22817371/22817176/22817496/22817348/22817774	5	cg01485075/cg03230154/cg18365865/cg18599790/cg21466963	0.321620655	0.403231496	0.556110682	0.152879186	9.87E-05	9.61E-03
UTR5	ZNF549	19	58038856/58038742	2	cg07054095/cg13644629	0.160202411	0.300530641	0.464759873	0.164229233	3.46E-03	3.19E-02
UTR5	ZNF586	19	58281117	1	cg22956410	0.104819689	0.117110548	0.328906385	0.211795837	2.67E-03	2.80E-02
UTR5	ZNF671	19	58238987/58238928	2	cg11977686/cg19246110	0.266571018	0.393356944	0.579648984	0.18629204	3.53E-04	1.27E-02
UTR5	ZNF813	19	53971035/53973920/53971464	3	cg12142445/cg22866218/cg27264706	0.11934244	0.204465153	0.378819685	0.174354532	6.95E-03	4.85E-02
UTR5	ZNF814	19	58400325/58400275	2	cg15759937/cg24124798	0.218604415	0.327995989	0.5074221	0.179426111	1.10E-03	1.82E-02
UTR5	ZSCAN23	6	28411271/28411030/28411240	3	cg06818710/cg07838603/cg13525197	0.204968139	0.274659697	0.464516997	0.1898573	2.65E-04	1.13E-02
EXON1	ABCB5	7	20687223	1	cg22066521	0.840938375	0.707326378	0.538969	-0.168357378	5.14E-05	8.13E-03
EXON1	ADAMTS18	16	77468893/77468850	2	cg03238797/cg07568194	0.242036425	0.243960726	0.416279982	0.172319255	2.98E-03	3.34E-02

EXON1	ADAMTS5	21	28338836/28337857/28337869/28339334/28339262	5	cg03202077/cg08979041/cg12078031/cg15237494/cg23986671	0.375383208	0.462682385	0.618631833	0.155949448	4.21E-04	1.32E-02
EXON1	ADARB2	10	1779432/1779667	2	cg00467420/cg05307923	0.152456797	0.280145489	0.436033709	0.155888219	3.02E-03	3.36E-02
EXON1	ADHFE1	8	67344720	1	cg20912169	0.256459935	0.411193623	0.660422359	0.249228736	7.99E-05	8.98E-03
EXON1	AEBP1	7	44143998/44144434/44144360/44143993/44144279	5	cg02126753/cg06128448/cg08739576/cg14249876/cg27493928	0.14101586	0.261169783	0.450443613	0.189273831	8.43E-04	0.017407084
EXON1	ALPL	1	21835945	1	cg05364759	0.215893632	0.240307554	0.424732568	0.184425014	1.35E-04	9.68E-03
EXON1	ALX3	1	110613072	1	cg05622465	0.163861709	0.208511138	0.372320473	0.163809335	3.44E-03	3.64E-02
EXON1	AMPH	7	38670957/38670987/38670985/38671001	4	cg02383130/cg07926691/cg10293925/cg26122980	0.220771274	0.30209	0.49313757	0.19104757	2.28E-03	2.87E-02
EXON1	ARMC3	10	23217017	1	cg15193782	0.125462671	0.184802217	0.35818351	0.173381292	3.58E-03	3.74E-02
EXON1	ASB15	7	123249130	1	cg02300083	0.61231217	0.532766017	0.371533668	-0.161232348	4.67E-04	1.38E-02
EXON1	BARHL2	1	91182534/91182708/91182731/91182288/91182215/91182128/91182777/91182305	8	cg01921432/cg06384463/cg06959142/cg13486820/cg13975503/cg15979173/cg18322569/cg26332310	0.349082339	0.386844012	0.539468922	0.152624911	8.62E-04	1.75E-02
EXON1	BCAT1	12	25102274/25102072	2	cg04011247/cg10764357	0.202790783	0.308302367	0.516316832	0.208014465	3.81E-05	7.81E-03
EXON1	C16orf73	16	1922149/1922155	2	cg05041351/cg07775371	0.155952418	0.191239859	0.348454698	0.157214839	0.00030839	0.012062031
EXON1	C17orf104	17	42733994/42733976	2	cg21095561/cg22190438	0.268131321	0.326896583	0.53463631	0.207739728	0.000100187	0.009131312
EXON1	C1orf173	1	75139347/75139364	2	cg24155871/cg27393126	0.295957565	0.367192178	0.573761716	0.206569538	8.54E-04	1.75E-02
EXON1	C1orf70	1	1475675/1475737	2	cg16306898/cg16601494	0.134715278	0.32452273	0.606946738	0.282424008	1.36E-04	9.68E-03
EXON1	C1QL3	10	16563821/16562917/16563586/16562626/16562998	5	cg10982433/cg11880855/cg15345154/cg24037897/cg25104555	0.106647108	0.180994227	0.363638429	0.182644202	1.09E-03	1.98E-02
EXON1	C2orf74	2	61372316/61372256	2	cg10580144/cg18158151	0.310297213	0.508793317	0.667898914	0.159105597	9.44E-04	1.84E-02
EXON1	CACNG3	16	24267226/24267399/24267559/24267526/24266905	5	cg02692912/cg04441477/cg04543289/cg07514158/cg17234150	0.338346394	0.442153518	0.596171655	0.154018137	5.15E-04	1.43E-02
EXON1	CACNG8	19	54466297/54466538/54466441	3	cg08653692/cg22049569/cg24415208	0.344097342	0.443230114	0.651466188	0.208236074	1.05E-04	9.15E-03
EXON1	CBLN2	18	70211515	1	cg21902544	0.273541708	0.40733855	0.624527209	0.217188659	8.10E-04	1.73E-02
EXON1	CBLN4	20	54579084/54579355/54579814	3	cg01729827/cg02501779/cg14184078	0.484643977	0.508003546	0.681354433	0.173350887	5.46E-06	7.59E-03
EXON1	CCDC140	2	223162881/223163033/223162875	3	cg06916239/cg23546474/cg25596297	0.422570955	0.382108772	0.580172291	0.198063519	1.41E-03	2.22E-02
EXON1	CCDC37	3	126113784	1	cg00891278	0.319879045	0.361500828	0.544311159	0.182810331	3.12E-04	1.21E-02
EXON1	CD34	1	208084456/208084415/208084640/208084436	4	cg04292718/cg16115720/cg19591881/cg21455438	0.238943501	0.37336436	0.543432812	0.170068452	2.54E-03	3.05E-02
EXON1	CDH18	5	19988200/19988288	2	cg06076122/cg06781712	0.272073128	0.394306767	0.572143656	0.177836889	1.17E-03	2.02E-02
EXON1	CDH2	18	25757211/25757438/25757202	3	cg09313439/cg12208258/cg13464915	0.291554003	0.338988742	0.495758347	0.156769605	2.49E-03	3.03E-02

EXON1	CDH8	16	62070005	1	cg09515947	0.33996726	0.47995985	0.651866577	0.171906727	0.000804513	0.017249427
EXON1	CHRM2	7	136553595/136553882/136553855/136554160/136553682/136553868/136553884/136553728	8	cg00973677/cg07664198/cg08323651/cg20847733/cg22471517/cg24228819/cg24575234/cg25632105	0.330892161	0.431609421	0.583658591	0.15204917	3.90E-05	7.81E-03
EXON1	CHST2	3	142838847/142838938/142839022/142838682	4	cg00995327/cg08858437/cg09638407/cg17815252	0.088903637	0.191432253	0.350789196	0.159356942	3.23E-03	3.52E-02
EXON1	CLIP4	2	29338432	1	cg08808128	0.152998709	0.321004766	0.562527791	0.241523025	1.04E-04	9.13E-03
EXON1	CNGA3	2	98962900/98962974	2	cg18404308/cg19671120	0.331170344	0.394459683	0.577932857	0.183473174	6.27E-05	8.29E-03
EXON1	COL25A1	4	110223646/110223713/110223700/110223795/110223429/110223598	6	cg02441747/cg06468908/cg10074544/cg11935021/cg19039028/cg25088758	0.256102573	0.356471273	0.53354126	0.177069987	4.49E-04	1.35E-02
EXON1	CPA4	7	129933014	1	cg01796223	0.58549644	0.538058156	0.374964577	-0.163093578	2.33E-04	1.11E-02
EXON1	CPB2	13	46679081	1	cg14662172	0.886822105	0.825670706	0.627236732	-0.198433974	1.18E-04	9.37E-03
EXON1	CR1L	1	207818493	1	cg24557058	0.25046316	0.380392383	0.543904982	0.163512598	4.13E-03	4.10E-02
EXON1	CTNNA3	10	69455804	1	cg19996026	0.509914225	0.4564797	0.297704323	-0.158775377	1.57E-04	1.01E-02
EXON1	DBX1	11	20181725	1	cg13445796	0.474141205	0.593457472	0.754344464	0.160886991	1.92E-03	2.63E-02
EXON1	DCLK1	13	36705446	1	cg20219457	0.321214625	0.385892277	0.57984025	0.193947973	3.33E-03	3.58E-02
EXON1	DEFB135	8	11839844	1	cg22743884	0.656798795	0.489811556	0.313000655	-0.176810901	5.50E-05	0.008134271
EXON1	DNAH11	7	21582834	1	cg05285902	0.182962681	0.209432983	0.381891586	0.172458603	3.00E-03	3.35E-02
EXON1	DOK5	20	53092474/53092334	2	cg00055557/cg20107395	0.245197855	0.316964298	0.471235526	0.154271228	0.003911039	0.039465394
EXON1	EBF1	5	158526693/158526614/158526642	3	cg04217450/cg05530568/cg17009297	0.283888828	0.337726565	0.504129801	0.166403236	2.24E-03	2.85E-02
EXON1	EFCAB3	17	60447682	1	cg17876595	0.77645862	0.605235417	0.423534982	-0.181700435	0.000102023	0.009131312
EXON1	EPHA10	1	38230769/38230779	2	cg12128017/cg20510207	0.302970382	0.384643194	0.540645805	0.15600261	1.99E-03	2.67E-02
EXON1	EPHA6	3	96533699/96533520/96533511/96533530/96533824	5	cg05827732/cg09301294/cg11410023/cg15405439/cg26364205	0.30481357	0.351154689	0.518230145	0.167075456	2.19E-03	2.81E-02
EXON1	ERBB4	2	213403212/213403321	2	cg07015629/cg19079194	0.22029798	0.223880067	0.386143727	0.162263661	3.79E-04	1.28E-02
EXON1	EVC	4	5713100/5712937/5713005	3	cg16418810/cg17460447/cg21210758	0.246805393	0.344737494	0.514194583	0.169457089	3.77E-04	0.01283133
EXON1	FAM132A	1	1181956	1	cg04719903	0.244664875	0.288058028	0.459094609	0.171036581	1.46E-03	2.26E-02
EXON1	FAM155A	13	108518237/108519213/108518955/108518179/108518152/108518445/108518621/108518419/108519318	9	cg03333286/cg04607400/cg05383490/cg07223990/cg13398192/cg14119999/cg15669183/cg17673237/cg19839798	0.354260508	0.408212486	0.565954616	0.15774213	1.21E-04	9.52E-03
EXON1	FAM159B	5	63986392	1	cg10981909	0.354039175	0.485957006	0.671355627	0.185398622	1.92E-03	2.63E-02
EXON1	FAM162B	6	117086850/117086601	2	cg07641160/cg23367608	0.232302324	0.278231633	0.45443854	0.176206908	7.37E-04	0.016702275
EXON1	FAM19A4	3	68981503	1	cg23967169	0.309262405	0.36991745	0.578523827	0.208606377	4.36E-05	8.01E-03
EXON1	FBN2	5	127873283/127873397/127873106/127873552/12787371	5	cg05209584/cg12940822/cg17564775/cg17958315/cg25084878	0.26655335	0.371927499	0.558581465	0.186653967	1.35E-03	2.17E-02
EXON1	FBXL7	5	15500714	1	cg06577205	0.344421575	0.375808717	0.528633736	0.15282502	3.82E-03	3.89E-02

EXON1	FGF12	3	192126244/192445380/192126165/192126584/192126825/192126023/192445307/192126742	8	cg00386551/cg02992632/cg04003850/cg04350913/cg16209795/cg18780257/cg19507527/cg21197774	0.1959348	0.283200399	0.43707825	0.153877852	7.22E-04	1.65E-02
EXON1	FGF4	11	69590090/69590113	2	cg19831575/cg23964057	0.151178155	0.239285329	0.42840766	0.189122331	1.78E-03	2.50E-02
EXON1	FGF5	4	81187798/81187906	2	cg02002231/cg14197923	0.106380913	0.196157855	0.391203448	0.195045593	5.27E-03	4.76E-02
EXON1	FIGLA	2	71017557/71017637/71017541	3	cg00469856/cg16907558/cg22650617	0.34936377	0.449135156	0.614173406	0.165038251	2.62E-04	1.14E-02
EXON1	FLRT2	14	85996499/85996751/85996495/85996873	4	cg01711160/cg05855039/cg17410236/cg25120290	0.387769243	0.505374665	0.658286638	0.152911972	1.61E-04	1.02E-02
EXON1	FOLH1	11	49230168/49230036	2	cg14775560/cg25346915	0.291977923	0.319281246	0.501149359	0.181868113	4.94E-04	1.40E-02
EXON1	FOXF1	16	86544346/86544308/86544787/86544339/86544658	5	cg00314966/cg08157228/cg10514097/cg25971347/cg27122536	0.21386133	0.244452614	0.425902228	0.181449613	2.61E-05	7.81E-03
EXON1	GAD2	10	26505442/26505503/26505440/26505245	4	cg04599297/cg09056181/cg11328303/cg15850954	0.277074423	0.44180043	0.613517622	0.171717191	6.95E-04	1.63E-02
EXON1	GALNT13	2	154728735/154728468	2	cg10033761/cg12079322	0.429723645	0.429760575	0.596179464	0.166418889	5.41E-05	8.13E-03
EXON1	GALNTL6	4	172734776/172734843/172734718	3	cg03413465/cg15533075/cg26588943	0.276865617	0.363287785	0.517858048	0.154570263	2.95E-04	1.19E-02
EXON1	GATA4	8	11561724/11561728	2	cg14666113/cg18283386	0.206845058	0.371160593	0.619421941	0.248261348	5.14E-05	0.008134271
EXON1	GLRA3	4	175750373/175750109/175750280	3	cg05424071/cg21094669/cg27517702	0.22069827	0.294578594	0.467080559	0.172501965	2.28E-04	1.11E-02
EXON1	GLRB	4	157997363/157997554/157997367/157997360	4	cg09688546/cg11163620/cg16269431/cg22065614	0.223893995	0.308681188	0.499797991	0.191116803	9.55E-04	1.85E-02
EXON1	GPC6	13	93879769/93879544/93879361/93879670/93879303	5	cg07576142/cg12989574/cg16792800/cg18058689/cg19824907	0.360206601	0.431576618	0.653171036	0.221594418	1.08E-04	9.18E-03
EXON1	GRASP	12	52400907	1	cg04034767	0.093413792	0.201368981	0.460650285	0.259281303	6.43E-04	1.57E-02
EXON1	GRIA4	11	105480979/105481802/105481509	3	cg03243226/cg09980522/cg19343464	0.278805595	0.397171828	0.567878198	0.17070637	0.000486595	0.01389208
EXON1	GRM7	3	6903153/6902824/6902845/6903327/6903019	5	cg02332525/cg17892178/cg18863595/cg21187352/cg27199820	0.444673461	0.536179492	0.688703268	0.152523777	2.30E-05	7.81E-03
EXON1	GSC	14	95236123	1	cg10042799	0.11632951	0.085090548	0.282819955	0.197729407	1.60E-03	2.36E-02
EXON1	HBQ1	16	230343/230341	2	cg02401454/cg07703401	0.32724683	0.338138094	0.520321184	0.18218309	5.27E-04	1.44E-02
EXON1	HCRTR2	6	55039232/55039259/55039382/55039295	4	cg10725720/cg13948987/cg17240815/cg27596495	0.282031535	0.328409048	0.488993624	0.160584575	1.23E-04	9.52E-03
EXON1	HELT	4	185940173	1	cg17186107	0.251086025	0.284845711	0.490349859	0.205504148	2.90E-05	0.007809598
EXON1	HOXC9	12	54393966/54393971/54394194/54394212/54394116	5	cg05069426/cg12071536/cg12699286/cg24500832/cg25456468	0.295738199	0.348657289	0.573071898	0.22441461	9.56E-05	9.13E-03

EXON1	HOXD10	2	176982108/176981654/176982230/176981919	4	cg02249732/cg03918304/cg10125317/cg17489939	0.578035672	0.530000896	0.683258768	0.153257872	7.38E-05	8.87E-03
EXON1	HTR1E	6	87647154/87647399/87647147/87647376/87647129/87647527/87647370	7	cg02928916/cg04278702/cg07833420/cg09297468/cg09863441/cg10135483/cg1477	0.273100513	0.369062302	0.56478146	0.195719158	3.51E-04	1.26E-02
EXON1	INHBB	2	121104029/121104187	2	cg02173749/cg03699182	0.185696665	0.210141327	0.36664773	0.156506403	2.13E-03	2.76E-02
EXON1	IRAK3	12	66583029/66583203/66583048	3	cg01263292/cg10389229/cg26415547	0.092280667	0.152925613	0.311538727	0.158613114	2.71E-03	3.16E-02
EXON1	IRF4	6	391764	1	cg17228900	0.100866439	0.314322966	0.585962585	0.271639619	1.23E-03	2.07E-02
EXON1	ITGA4	2	182322268/182322058/182321855/182322279/182322501/182321786	6	cg06952671/cg10526659/cg20415809/cg21995919/cg25024074/cg25652029	0.260763156	0.458986749	0.611804352	0.152817603	2.48E-03	3.03E-02
EXON1	JAM3	11	133938941	1	cg02174225	0.125843112	0.265508881	0.442115258	0.176606377	4.02E-03	4.02E-02
EXON1	KCNA1	12	5019311/5019482/5019091	3	cg09487611/cg10363246/cg12908522	0.126730906	0.241964179	0.476888771	0.234924592	6.61E-04	1.59E-02
EXON1	KCNH5	14	63511872	1	cg11755405	0.38196622	0.495121994	0.6589143	0.163792306	2.20E-04	0.011032849
EXON1	KCNQ5	6	73331680/73331652/73332073	3	cg03786924/cg15717808/cg24687051	0.241818556	0.390541533	0.588875241	0.198333708	1.14E-04	9.26E-03
EXON1	KCTD8	4	44450234/44450358/44449738	3	cg10059660/cg12300353/cg20646280	0.354856387	0.402319196	0.602880356	0.20056116	2.14E-06	7.59E-03
EXON1	KRBA1	7	149412290	1	cg03788131	0.138089557	0.242209075	0.483524739	0.241315664	1.14E-05	7.59E-03
EXON1	KRT20	17	39041266/39041199/39041119/39041110	4	cg19114576/cg19449377/cg23901700/cg25124433	0.783862687	0.553260496	0.399192683	-0.154067813	7.87E-04	1.70E-02
EXON1	KRT7	12	52627342/52627047/52627272	3	cg09522147/cg09670128/cg25313172	0.421868168	0.536565783	0.689640498	0.153074714	2.27E-04	0.011056134
EXON1	KRTAP21-2	21	32119309	1	cg23581186	0.679008385	0.542251406	0.39001255	-0.152238856	0.000459846	0.013609116
EXON1	LAMA1	18	7117680/7117790	2	cg07846220/cg18764577	0.361940153	0.458143292	0.61602922	0.157885929	8.27E-05	8.98E-03
EXON1	LBXCOR1	15	68118006	1	cg17082347	0.135106277	0.183650134	0.36080405	0.177153917	4.77E-03	0.044984516
EXON1	LCE1B	1	152784914/152785473	2	cg07090653/cg12449852	0.729669125	0.6528083	0.479028127	-0.173780173	3.59E-04	0.012742728
EXON1	LHCGR	2	48982683/48982754/48982832	3	cg00209038/cg13423075/cg20156659	0.419088745	0.4553261	0.617299805	0.161973705	5.52E-05	8.13E-03
EXON1	LIN28B	6	105405068	1	cg04560456	0.330428215	0.378783794	0.543564627	0.164780833	6.63E-04	1.59E-02
EXON1	LPPR5	1	99470129/99470207	2	cg14991984/cg18623672	0.270830868	0.405834217	0.581562782	0.175728565	3.28E-04	1.23E-02
EXON1	LRFN5	14	42077674/42077327/42077061	3	cg04784672/cg21547690/cg26967167	0.262014391	0.338581776	0.508601712	0.170019935	0.000395735	0.01283133
EXON1	LRRC4C	11	40314618/40315404/40315392/40314978/40314771/40315337	6	cg09147985/cg17579667/cg17949440/cg19849428/cg22645719/cg23850899	0.428304199	0.444728341	0.643843741	0.1991154	1.25E-05	7.59E-03
EXON1	LRRIQ4	3	169539775/169540297/169540504/169540079	4	cg04748593/cg13793580/cg18155853/cg23081534	0.810342383	0.576680539	0.389583758	-0.187096781	5.74E-05	0.008134271
EXON1	LUZP2	11	24518737/24518776	2	cg06931933/cg09717526	0.277360133	0.309575603	0.460539434	0.150963831	1.11E-03	1.98E-02
EXON1	LYPD5	19	44324747/44306565	2	cg11898486/cg12072164	0.386054995	0.4309358	0.601844391	0.170908591	6.96E-04	1.63E-02

EXON1	MATK	19	3801548/3801727/3786246	3	cg01388796/cg09416313/cg17429382	0.19456021	0.267437483	0.454817496	0.187380014	5.28E-04	1.44E-02
EXON1	MDFI	6	41606317	1	cg01350077	0.134761777	0.262817074	0.459091135	0.196274061	4.17E-03	4.12E-02
EXON1	MMD2	7	4998714/4998649	2	cg26468007/cg27603283	0.263741423	0.340103578	0.49909633	0.158992752	9.47E-04	1.84E-02
EXON1	MOXD1	6	132722421/132722315	2	cg07570142/cg16478774	0.101546219	0.17423656	0.388545952	0.214309392	7.94E-04	1.71E-02
EXON1	MYH10	17	8534013	1	cg06011292	0.035441597	0.044081865	0.240930607	0.196848742	1.19E-03	2.04E-02
EXON1	NCAN	19	19322818	1	cg08249988	0.38897671	0.513108794	0.684720918	0.171612124	3.10E-04	1.21E-02
EXON1	NEUROG3	10	71333037	1	cg03425110	0.200846189	0.313128656	0.547800477	0.234671822	5.80E-05	8.13E-03
EXON1	NGB	14	77737621/77737495	2	cg02181639/cg02881570	0.375804095	0.441153767	0.623488245	0.182334479	9.01E-04	0.017925869
EXON1	NKX2-2	20	21494084/21494547	2	cg16390060/cg17029168	0.29859384	0.403195008	0.56332042	0.160125412	1.48E-03	2.28E-02
EXON1	NPBWR1	8	53853444/53852661/53853112	3	cg02640612/cg06528306/cg21001487	0.391862003	0.454914864	0.620630359	0.165715495	1.33E-04	9.68E-03
EXON1	NPR3	5	32711881/32712237/32712623	3	cg01252672/cg05839235/cg17630392	0.316180277	0.398280525	0.576090841	0.177810316	3.30E-05	7.81E-03
EXON1	ODF3	11	196824	1	cg04874875	0.80516377	0.767069428	0.603839582	-0.163229846	0.000275719	0.011606349
EXON1	ONECUT1	15	53081526/53081248	2	cg14217069/cg17500265	0.36876441	0.425367464	0.599747198	0.174379734	1.75E-04	1.03E-02
EXON1	OPRK1	8	54164161/54164051/54164081	3	cg07303244/cg11701471/cg25990647	0.33181452	0.425166318	0.593733085	0.168566767	1.63E-03	2.36E-02
EXON1	OR10T2	1	158369112/158368889	2	cg15593510/cg15852352	0.678088725	0.501589039	0.350021123	-0.151567916	3.64E-05	7.81E-03
EXON1	OR2AK2	1	248129192	1	cg11977100	0.593765145	0.440189311	0.286650286	-0.153539025	3.45E-05	7.81E-03
EXON1	OR51B4	11	5322976	1	cg06353345	0.64987143	0.514448006	0.319087886	-0.195360119	9.37E-07	0.007587652
EXON1	OR6C65	12	55794783	1	cg25236050	0.674340085	0.570522283	0.42028155	-0.150240733	1.02E-03	1.92E-02
EXON1	PAK7	20	9819467	1	cg18034428	0.34452928	0.323443256	0.515732564	0.192289308	0.000124667	0.009522661
EXON1	PARVB	22	44420316	1	cg20991347	0.128513228	0.199922744	0.374301399	0.174378655	4.54E-03	4.36E-02
EXON1	PCP4L1	1	161228674	1	cg11912330	0.16781842	0.160256398	0.392305085	0.232048687	3.05E-04	1.21E-02
EXON1	PDGFD	11	104034754/104034619/104034982	3	cg02120582/cg07748540/cg18403606	0.067299898	0.215763276	0.412432378	0.196669102	4.09E-03	4.07E-02
EXON1	PHOX2A	11	71955113/71954982/71955164	3	cg13103303/cg18722841/cg19651694	0.354161368	0.423400856	0.581726418	0.158325563	1.48E-04	0.010000173
EXON1	PKIA	8	79428444/79428569/79428434	3	cg09043127/cg12330330/cg27140220	0.062668109	0.138695383	0.293404026	0.154708643	4.13E-03	4.10E-02
EXON1	PPAPDC1A	10	122216848	1	cg17087331	0.225069555	0.180014741	0.390885319	0.210870578	2.18E-04	1.10E-02
EXON1	PPP2R2C	4	6473964	1	cg18013519	0.07588347	0.151003771	0.353179632	0.202175862	1.32E-03	2.15E-02
EXON1	PRND	20	4702581	1	cg09906458	0.66278959	0.504456711	0.349004655	-0.155452057	3.20E-05	0.007809598
EXON1	PRODH2	19	36304079	1	cg18640606	0.73577225	0.673229444	0.501053945	-0.172175499	2.46E-05	7.81E-03
EXON1	PTH2R	2	209271661/209271785	2	cg06391199/cg24769821	0.27094058	0.304544699	0.461541468	0.156996769	5.18E-03	4.72E-02
EXON1	PYDC1	16	31228299/31228202/31228263	3	cg08346922/cg10038009/cg13352836	0.350772645	0.447509511	0.65483555	0.207326039	7.67E-05	0.008983718
EXON1	QKI	6	163836348/163836245	2	cg06844545/cg09339301	0.174056859	0.207228581	0.360244865	0.153016285	3.89E-03	3.94E-02
EXON1	QRFPR	4	122302007/122301724/122301573/122301996/122301816/122301740	6	cg00015770/cg06991300/cg10114327/cg14012124/cg16326674/cg19971716	0.307967968	0.396433607	0.5574158	0.160982193	7.86E-04	1.70E-02

EXON1	RALYL	8	85096037/85095498/85097246/85097157/85096015/85095654/85097195/85095697	8	cg00034076/cg05716166/cg17215863/cg18016194/cg22403811/cg23777958/cg25757598/cg26669044	0.368062837	0.414865296	0.604808588	0.189943292	4.99E-05	8.13E-03
EXON1	RORB	9	77112326/77112896	2	cg07536920/cg14170313	0.128788008	0.182273709	0.344570812	0.162297103	2.08E-03	2.74E-02
EXON1	RSPO2	8	109095568/109095572/109095782	3	cg00910695/cg04050867/cg14733048	0.274932993	0.351034819	0.527101124	0.176066306	2.42E-04	1.12E-02
EXON1	RYR1	19	38924347/38924349/38924436	3	cg04571321/cg05471297/cg06740600	0.19628607	0.287594851	0.464376086	0.176781235	0.005193298	0.047217962
EXON1	SCGB2A2	11	62037659	1	cg22862656	0.81272038	0.609554289	0.447701014	-0.161853275	4.52E-04	0.013467227
EXON1	SCTR	2	120281999/120281813	2	cg07176264/cg20505223	0.287517405	0.314268917	0.480911932	0.166643015	2.82E-03	3.24E-02
EXON1	SDC2	8	97506180/97506251	2	cg14538332/cg16935295	0.12978423	0.237232389	0.44108355	0.203851161	0.002418525	0.029707217
EXON1	SLC35F3	1	234040765/234040781/234040833	3	cg05051043/cg09424759/cg25437385	0.28101686	0.333445822	0.492317723	0.158871901	6.27E-04	1.55E-02
EXON1	SLC5A7	2	108603005/108603193	2	cg16232126/cg26333822	0.336260815	0.343567149	0.498572323	0.155005173	1.73E-04	1.03E-02
EXON1	SLC6A15	12	85306548/85306474/85306323/85306574	4	cg02492791/cg14449051/cg19952303/cg24418420	0.229127629	0.269826865	0.424654816	0.154827951	3.64E-03	3.77E-02
EXON1	SLC7A14	3	170303830/170303721	2	cg18774195/cg21884231	0.328577135	0.405935067	0.585724873	0.179789806	2.29E-04	1.11E-02
EXON1	SLIT2	4	20255264/20255343	2	cg03742003/cg08428452	0.257782725	0.366029311	0.529762009	0.163732698	2.64E-03	3.11E-02
EXON1	SLITRK5	13	88324879	1	cg06682197	0.173845211	0.201134676	0.395404609	0.194269933	6.89E-05	8.60E-03
EXON1	SMOC1	14	70346413/70346477/70346221/70346151/70346417	5	cg15239123/cg15724184/cg15996043/cg18493027/cg19504005	0.124645166	0.219719925	0.38550761	0.165787685	3.42E-03	3.64E-02
EXON1	SNTG2	2	946648	1	cg09426834	0.305865165	0.362097389	0.540930227	0.178832838	1.97E-03	2.66E-02
EXON1	SPAG4L	20	31592147	1	cg02510802	0.74023952	0.573631311	0.371162491	-0.20246882	2.43E-05	7.81E-03
EXON1	SPHKAP	2	229046325	1	cg04072323	0.285360715	0.389797139	0.577976023	0.188178884	9.10E-05	9.13E-03
EXON1	SPOCK1	5	136834877	1	cg05143123	0.139690965	0.215181332	0.399490303	0.184308972	4.40E-03	4.27E-02
EXON1	ST6GALNAC5	1	77333229/77333198	2	cg04077662/cg06201642	0.26808874	0.377675175	0.53033383	0.152658655	3.03E-03	3.37E-02
EXON1	ST8SIA1	12	22487437/22487459/22487219	3	cg09354309/cg10333808/cg24723331	0.135148496	0.178398841	0.346324339	0.167925498	3.60E-03	3.75E-02
EXON1	ST8SIA3	18	55020099/55019843	2	cg04017533/cg13096208	0.41537209	0.499723978	0.684239282	0.184515304	1.21E-05	7.59E-03
EXON1	ST8SIA5	18	44336399	1	cg19751300	0.246936895	0.371629106	0.542666518	0.171037413	5.69E-04	1.47E-02
EXON1	STOX2	4	184826953/184827086/184827754/184827491/18482670	5	cg01758805/cg02071076/cg02500300/cg06367693/cg11354594	0.222166173	0.24050692	0.39943448	0.15892756	0.002615538	0.030976679
EXON1	SUNC1	7	48068665	1	cg23509098	0.70715429	0.561490989	0.376269341	-0.185221648	1.07E-05	7.59E-03
EXON1	SYCP2L	6	10887367/10887185	2	cg03320827/cg26237681	0.251650399	0.311186155	0.50790396	0.196717805	6.35E-05	8.32E-03
EXON1	SYT6	1	114696465/114696462/114696350	3	cg05368341/cg09177131/cg19304150	0.224724152	0.298136266	0.469972501	0.171836235	7.59E-04	0.016926036
EXON1	TAC1	7	97361408	1	cg16288089	0.38903177	0.447987311	0.610351032	0.162363721	3.64E-03	3.77E-02
EXON1	TAS2R8	12	10958919	1	cg21156620	0.639956295	0.561372061	0.399305305	-0.162066757	7.09E-06	7.59E-03

EXON1	TFPI2	7	93520036/93520024/93519924/93520012	4	cg14775114/cg17338208/cg23686014/cg24531255	0.142450764	0.321753576	0.519702184	0.197948608	4.38E-04	1.34E-02
EXON1	TLL1	4	166794471/166794895/166794971/166794786	4	cg08570521/cg09669853/cg19898128/cg24521633	0.14149001	0.220108282	0.378027405	0.157919123	2.96E-03	3.32E-02
EXON1	TLX1	10	102891076/102891568/102891280/102891080	4	cg02096397/cg02450004/cg05329692/cg27272677	0.206961461	0.194047207	0.357389394	0.163342187	5.40E-04	1.45E-02
EXON1	TM6SF1	15	83776422/83776446/83776420	3	cg03063639/cg14696396/cg26460092	0.288529638	0.477104186	0.674643993	0.197539807	1.96E-04	1.07E-02
EXON1	TMEM155	4	122686269/122686038/122686319	3	cg05396987/cg10863741/cg21077559	0.25409643	0.370161301	0.524858427	0.154697126	3.75E-04	1.28E-02
EXON1	TRH	3	129693613/129693586/129693385/129693370/12969348	5	cg01009664/cg02700891/cg11940285/cg18862481/cg22512438	0.30922142	0.419885811	0.616723861	0.196838051	0.00020499	0.011023645
EXON1	TRHDE	12	72667236/72667326/72666976/72667150	4	cg01030121/cg09972192/cg13663218/cg27579953	0.312842455	0.397417921	0.574754972	0.177337051	1.82E-04	1.03E-02
EXON1	TRPC6	11	101454317/101454626	2	cg03411507/cg11016563	0.33482509	0.403225172	0.58180788	0.178582707	4.76E-06	7.59E-03
EXON1	TTYH1	19	54926805/54926734	2	cg09474331/cg23695687	0.184290943	0.320623184	0.49677406	0.176150876	2.98E-03	3.34E-02
EXON1	TWIST1	7	19157193/19156902/19157263/19156550/19156621	5	cg17839237/cg20052718/cg24446548/cg26312150/cg26818735	0.110370943	0.198173023	0.364804639	0.166631616	1.72E-04	1.03E-02
EXON1	UGT2A2	4	70504750	1	cg02219607	0.854529735	0.804295483	0.638369209	-0.165926274	0.000313542	0.012062031
EXON1	UNCX	7	1272710	1	cg03092191	0.266196025	0.287000883	0.467925414	0.18092453	1.21E-03	2.05E-02
EXON1	VAX2	2	71127779/71127892	2	cg15579817/cg21637670	0.197576615	0.19675699	0.417413989	0.220656999	1.94E-03	2.64E-02
EXON1	VCAN	5	82767526/82767617	2	cg04525496/cg05176349	0.132559309	0.18446299	0.343477828	0.159014838	7.84E-04	1.70E-02
EXON1	VIPR2	7	158937610/158937494	2	cg03976877/cg13794530	0.344609673	0.441673113	0.646611789	0.204938676	3.56E-05	7.81E-03
EXON1	VSX1	20	25062754/25062447	2	cg00043788/cg14763548	0.309602228	0.34355494	0.553490257	0.209935317	7.18E-05	8.80E-03
EXON1	VWC2	7	49813486	1	cg18206027	0.182120415	0.220693698	0.416763476	0.196069778	3.49E-04	1.26E-02
EXON1	WNT2	7	116963259/116963193	2	cg07697895/cg27331524	0.269514918	0.365072882	0.541466436	0.176393554	4.88E-04	1.39E-02
EXON1	ZFHX4	8	77593623	1	cg07585876	0.209150756	0.269712738	0.490332389	0.220619651	4.33E-03	4.20E-02
EXON1	ZIK1	19	58095659	1	cg26246807	0.375614655	0.597706644	0.758164927	0.160458283	0.001386439	0.022127039
EXON1	ZNF256	19	58458917/58458979	2	cg14556070/cg20176532	0.177109025	0.217259773	0.435286696	0.218026924	0.001075868	0.019604206
EXON1	ZNF304	19	57863057/57862713	2	cg04685743/cg07494047	0.084922142	0.236218978	0.429114705	0.192895727	0.001614243	0.023593366
EXON1	ZNF492	19	22817176	1	cg03230154	0.249439945	0.350137439	0.5459557	0.195818261	7.81E-05	8.98E-03
EXON1	ZNF549	19	58038856/58038742	2	cg07054095/cg13644629	0.160202411	0.300530641	0.464759873	0.164229233	3.46E-03	3.66E-02
EXON1	ZNF586	19	58281117	1	cg22956410	0.104819689	0.117110548	0.328906385	0.211795837	2.67E-03	3.14E-02
EXON1	ZNF660	3	44626492	1	cg22598028	0.205992665	0.316678834	0.524859862	0.208181028	1.62E-03	2.36E-02
EXON1	ZNF671	19	58238987/58238850/58238928	3	cg11977686/cg12074025/cg19246110	0.284639445	0.427167596	0.614744311	0.187576714	4.39E-04	1.34E-02
EXON1	ZNF75A	16	3355553	1	cg05540369	0.223151031	0.339592739	0.496508255	0.156915516	1.52E-03	2.31E-02
EXON1	ZNF793	19	37997867	1	cg15139588	0.098841377	0.255724284	0.475644413	0.219920128	3.17E-03	3.47E-02

EXON1	ZNF814	19	58400325/58400275	2	cg15759937/cg24124798	0.218604415	0.327995989	0.5074221	0.179426111	1.10E-03	1.98E-02
EXON1	ZSCAN23	6	28411271/28411240	2	cg06818710/cg13525197	0.218626205	0.290955486	0.471140323	0.180184837	3.89E-04	1.28E-02
GENEBODY	ABCD2	12	39981136	1	cg13406085	0.717780775	0.565277444	0.388307936	-0.176969508	2.17E-05	4.67E-03
GENEBODY	C13orf29	13	111521981/111522314/111522651/111522222	4	cg03757805/cg04513422/cg13698168/cg16032841	0.564229618	0.5081458	0.358088846	-0.150056954	1.10E-05	4.67E-03
GENEBODY	C1orf158	1	12806667	1	cg24338843	0.782470565	0.695402917	0.498194609	-0.197208308	1.04E-04	5.69E-03
GENEBODY	C21orf131	21	22175001	1	cg09614626	0.750141095	0.645449372	0.468220914	-0.177228459	1.12E-04	5.85E-03
GENEBODY	C4orf47	4	186356202	1	cg13054212	0.854377895	0.731720367	0.568434236	-0.16328613	2.58E-05	4.67E-03
GENEBODY	C4orf49	4	140200520/140201100/140200723/140201065/14020065	5	cg00208504/cg01733638/cg05924540/cg07776847/cg13272465	0.150081319	0.244701395	0.399377023	0.154675628	7.31E-04	1.11E-02
GENEBODY	C5orf58	5	169663955	1	cg17038633	0.812719335	0.70161615	0.531221877	-0.170394273	4.03E-05	4.80E-03
GENEBODY	C7orf66	7	108524200	1	cg21462681	0.755043265	0.636153822	0.435511264	-0.200642559	2.60E-05	4.67E-03
GENEBODY	CCK	3	42304966/42301921/42304070/42305063	4	cg06085877/cg13427828/cg15778433/cg21327694	0.186010025	0.240556373	0.392592868	0.152036495	7.84E-03	3.95E-02
GENEBODY	CHODL	21	19617873/19618088	2	cg11433319/cg17040471	0.40303238	0.512811294	0.693392332	0.180581037	2.40E-04	7.38E-03
GENEBODY	CHST2	3	142840240/142839991/142839903/142840129/142839837/142840837	6	cg14473344/cg17373442/cg18507129/cg22536150/cg23749856/cg26399662	0.347364705	0.438624984	0.604279828	0.165654843	3.14E-05	4.67E-03
GENEBODY	CLDN5	22	19511707/19511987/19511967	3	cg00811132/cg09092054/cg17577122	0.170191199	0.277565413	0.4992414	0.221675987	7.68E-05	5.27E-03
GENEBODY	DEFB122	20	30012193	1	cg01349088	0.63772139	0.558780289	0.38016645	-0.178613839	3.96E-04	8.87E-03
GENEBODY	DKK4	8	42234243	1	cg09184899	0.77903867	0.742232667	0.5192362	-0.222996467	1.26E-04	6.19E-03
GENEBODY	FAM49A	2	16767476	1	cg10090418	0.770699375	0.590443906	0.396925518	-0.193518387	2.67E-04	7.73E-03
GENEBODY	GALR1	18	74963364/74964030/74963807	3	cg00662556/cg05896682/cg26038190	0.36631598	0.452316078	0.616849118	0.16453304	8.92E-05	5.45E-03
GENEBODY	GPR149	3	154146183/154146398/154145868/154145810	4	cg00046499/cg04151278/cg16619395/cg20498859	0.291585609	0.374960358	0.539452854	0.164492496	1.55E-04	6.28E-03
GENEBODY	GSC	14	95235125/95235489/95235026/95235127/95235968/95234965/95235869/95235402	8	cg01163842/cg01533258/cg02948476/cg07785447/cg13252307/cg18687675/cg20804555/cg21063282	0.262750923	0.281539885	0.443667865	0.16212798	4.00E-03	2.60E-02
GENEBODY	HBBP1	11	5263432	1	cg04161236	0.6683465	0.518537633	0.364920186	-0.153617447	4.39E-04	9.11E-03
GENEBODY	HELT	4	185940595/185941265/185941304/185941625/185941601/185940969/185940549/185941088/185940638/185940	10	cg00075634/cg00939735/cg04905049/cg06274396/cg10463150/cg11497952/cg13966883/cg17081772/cg18065811/cg2284889	0.229381335	0.271655583	0.434679543	0.16302396	3.47E-04	8.46E-03
GENEBODY	HOXA2	7	27140797/27141067/27140942/27141388/27141088/27141139	6	cg00188704/cg02803819/cg03763508/cg06786372/cg13661519/cg21464565	0.564760608	0.65107556	0.823423477	0.172347916	4.04E-04	8.92E-03
GENEBODY	HOXD13	2	176958962/176958552/176959229	3	cg09760956/cg18229422/cg24914355	0.315482725	0.239413681	0.412439295	0.173025614	2.86E-04	8.04E-03
GENEBODY	ICOS	2	204822542	1	cg15247069	0.781445035	0.6497174	0.470782023	-0.178935377	3.46E-05	4.67E-03

GENEBODY	IL2RB	22	37536156	1	cg22327543	0.6707666	0.570526994	0.402161141	-0.168365854	6.59E-05	4.98E-03
GENEBODY	IRX5	16	54966837/54965929/54965492/54965895/54967389/54967714/54967024/54966471	8	cg01901262/cg03665761/cg05266781/cg07254066/cg09492451/cg10824810/cg16376108/cg16642791	0.397374316	0.46151088	0.631797325	0.170286445	1.62E-05	4.67E-03
GENEBODY	KIAA0495	1	3661862	1	cg14912788	0.81316239	0.718901144	0.526893782	-0.192007363	1.79E-06	4.48E-03
GENEBODY	KRT4	12	53200975	1	cg21182263	0.71473191	0.686663217	0.534418023	-0.152245194	1.06E-04	5.72E-03
GENEBODY	LOC200726	2	207511246	1	cg15579926	0.760571855	0.677117689	0.504789355	-0.172328334	5.48E-05	4.80E-03
GENEBODY	LOC283392	12	72667236/72665510/72666264/72665880/72666281/72666976/72665376/72665282/72665771/72667150	10	cg01030121/cg01817029/cg02511156/cg04775889/cg06622999/cg13663218/cg17771605/cg18440199/cg23142394/cg2757995	0.2548672	0.328457629	0.493095163	0.164637534	5.96E-04	1.02E-02
GENEBODY	LOC643719	19	35068555/35068221	2	cg23200020/cg25418852	0.438227565	0.589002589	0.745249427	0.156246838	9.20E-04	1.21E-02
GENEBODY	LOR	1	153234037/153234342/153234268	3	cg08611411/cg11534293/cg21860629	0.090814989	0.186863206	0.375520182	0.188656975	2.15E-03	1.83E-02
GENEBODY	LRRIQ3	1	74620779	1	cg14795163	0.6588679	0.5388706	0.372009832	-0.166860768	8.03E-05	5.30E-03
GENEBODY	LYPD4	19	42342105	1	cg07959016	0.829059155	0.735874372	0.554447673	-0.181426699	1.03E-04	5.69E-03
GENEBODY	MCOLN3	1	85510988	1	cg24535650	0.822618095	0.781970011	0.604710259	-0.177259752	8.37E-05	5.41E-03
GENEBODY	MIR129-2	11	43602965	1	cg03365311	0.2920819	0.386223178	0.586951064	0.200727886	8.93E-05	5.45E-03
GENEBODY	MIR137	1	98511629	1	cg04293733	0.353616195	0.387999328	0.558625427	0.170626099	1.98E-03	1.76E-02
GENEBODY	MIR34B	11	111383668	1	cg01192900	0.19902581	0.250600589	0.418149077	0.167548488	1.79E-03	1.66E-02
GENEBODY	MSC	8	72755871/72755052/72755784/72755568/72754953/72755162	6	cg06269753/cg13620034/cg20079899/cg26280666/cg26799209/cg26876974	0.198986605	0.310751587	0.468738948	0.157987361	1.03E-03	1.27E-02
GENEBODY	NEUROD1	2	182542864/182542901/182543233	3	cg01431993/cg11528849/cg16640855	0.379430115	0.458889536	0.660677221	0.201787685	5.53E-05	4.80E-03
GENEBODY	NEUROG3	10	71332776/71332744	2	cg12938159/cg26333837	0.199739422	0.271869736	0.472729803	0.200860067	1.50E-05	4.67E-03
GENEBODY	NHEDC1	4	103867628	1	cg15623444	0.85882682	0.7135649	0.550782377	-0.162782523	4.08E-04	8.92E-03
GENEBODY	NINL	20	25450839/25479848/25448861	3	cg09614389/cg13486641/cg26101502	0.683284493	0.593167531	0.433255398	-0.159912132	1.38E-03	1.44E-02
GENEBODY	NKX2-2	20	21493101/21492823/21492914	3	cg01397679/cg21977377/cg22474464	0.30181082	0.394914831	0.568672383	0.173757552	4.01E-03	2.60E-02
GENEBODY	OR1F2P	16	3266276	1	cg08748105	0.64413183	0.556193817	0.4051304	-0.151063417	6.83E-04	1.07E-02
GENEBODY	RBM46	4	155720155	1	cg03174933	0.7254943	0.602266711	0.429098486	-0.173168225	1.02E-04	5.66E-03
GENEBODY	RSAD2	2	7029602	1	cg00186143	0.739571515	0.611205006	0.449552891	-0.161652115	1.08E-03	1.29E-02
GENEBODY	SCGB3A1	5	180017846/180017349/180017623/180017689/180018201/180018102	6	cg07317366/cg13004587/cg16585682/cg18652367/cg19897330/cg21919857	0.20540903	0.254657704	0.411046131	0.156388428	1.03E-03	1.27E-02
GENEBODY	SIRPB2	20	1471884	1	cg00228984	0.552403565	0.466718389	0.3144807	-0.152237689	9.09E-05	5.47E-03
GENEBODY	SIRPD	20	1532543	1	cg03726785	0.7268426	0.642669639	0.434093927	-0.208575712	8.57E-07	4.48E-03
GENEBODY	SIX6	14	60977961/60977353/60977054/60977856/60976898/60977300	6	cg00157572/cg04666975/cg07862488/cg13019491/cg14511698/cg19981568	0.32836159	0.43885034	0.592218193	0.153367853	3.04E-04	8.23E-03
GENEBODY	SLC10A2	13	103702677	1	cg08450807	0.682875125	0.540926783	0.3800928	-0.160833983	1.16E-05	4.67E-03
GENEBODY	SNORD116-	15	25327919	1	cg07897695	0.773205945	0.714791161	0.558580695	-0.156210466	2.23E-05	4.67E-03
GENEBODY	SNORD64	15	25230295	1	cg18488946	0.6237695	0.556032011	0.405183841	-0.15084817	1.40E-04	6.22E-03

GENEBODY	TFPI2	7	93519473/93519220/93519401/93519855/93519621/93519892	6	cg12973591/cg18024479/cg19784477/cg20230721/cg22799321/cg23141855	0.200434836	0.348556788	0.571395334	0.222838546	1.21E-05	4.67E-03
GENEBODY	TMEM39A	3	119180915	1	cg08844169	0.85753726	0.742626456	0.589412418	-0.153214037	2.11E-04	6.90E-03
GENEBODY	TRIM77	11	89448414	1	cg15042801	0.58826783	0.453950444	0.300998227	-0.152952217	7.22E-06	4.67E-03
GENEBODY	TSGA10IP	11	65713893	1	cg21800989	0.693673805	0.633508528	0.47251895	-0.160989578	1.38E-04	6.22E-03
GENEBODY	UGT2A1	4	70504750	1	cg02219607	0.854529735	0.804295483	0.638369209	-0.165926274	0.000313542	0.008229402
GENEBODY	ZIC1	3	147129278/147129826/147129184/147131069/147128998/147131178/147130477/147131146/147129778/147131332/147130536/14	13	cg00842299/cg01376826/cg05095591/cg05716671/cg06306198/cg12204258/cg14750948/cg16209664/cg17482033/cg18297437/cg19029181/cg234	0.336063238	0.401763437	0.563720743	0.161957306	1.58E-04	6.38E-03
GENEBODY	ZNF254	19	24270468	1	cg01000657	0.070355748	0.104261602	0.280390649	0.176129048	4.91E-03	2.93E-02
GENEBODY	ZNF454	5	178371587/178381935	2	cg05461386/cg18358845	0.720645005	0.645089992	0.495048748	-0.150041244	6.94E-05	5.06E-03
GENEBODY	ZNF518B	4	10447440	1	cg11578055	0.86757463	0.808147272	0.613450536	-0.194696736	1.29E-03	0.013995358
GENEBODY	ZNF788	19	12203315/12203644/12203198/12203349/12207214	5	cg01172903/cg01404753/cg06384763/cg11767984/cg21289365	0.136134971	0.255735171	0.50351314	0.247777969	3.22E-04	8.23E-03
GENEBODY	ZNF85	19	21106370	1	cg11416076	0.152357116	0.290752656	0.445527541	0.154774885	6.73E-03	3.58E-02
UTR3	ABLIM3	5	148638033	1	cg02306139	0.614524665	0.5010142	0.329975855	-0.171038345	2.21E-06	2.84E-03
UTR3	ADAMTSL3	15	84706833	1	cg08115297	0.672769115	0.53539575	0.326736905	-0.208658845	1.08E-06	2.63E-03
UTR3	ANO5	11	22301379	1	cg06703469	0.764614435	0.692608706	0.530419827	-0.162188878	1.88E-04	0.008869809
UTR3	APCS	1	159558512	1	cg21291134	0.689444045	0.605787644	0.427001636	-0.178786008	1.47E-04	8.38E-03
UTR3	APOBEC3F	22	39440294/39449298	2	cg09582545/cg19333614	0.921456525	0.820357289	0.669946377	-0.150410912	5.22E-04	1.28E-02
UTR3	BACH2	6	90639197	1	cg22758471	0.75454658	0.6842184	0.529889818	-0.154328582	1.10E-04	7.65E-03
UTR3	C15orf41	15	37100941	1	cg26451373	0.673976035	0.626238167	0.467211527	-0.159026639	8.43E-04	1.52E-02
UTR3	C17orf102	17	32904255/32903717	2	cg12309348/cg26090020	0.678335128	0.564225969	0.39224943	-0.17197654	4.81E-07	1.64E-03
UTR3	C1R	12	7187715	1	cg15837470	0.616887595	0.517457528	0.340778336	-0.176679191	4.11E-06	2.84E-03
UTR3	C7orf16	7	31747928	1	cg01227294	0.735600575	0.711654978	0.509239177	-0.202415801	1.29E-06	2.63E-03
UTR3	CACNG7	19	54445888	1	cg21477176	0.54189333	0.599618967	0.750982605	0.151363638	9.19E-05	7.33E-03
UTR3	CALCRL	2	188210673	1	cg22374415	0.770731225	0.673276206	0.466355305	-0.206920901	6.80E-05	6.42E-03
UTR3	CDH13	16	83829911	1	cg05847519	0.672865515	0.479692039	0.321072532	-0.158619507	5.37E-04	1.29E-02
UTR3	CLEC2A	12	10065986	1	cg24640735	0.677238055	0.589198289	0.385397318	-0.203800971	4.17E-06	2.84E-03
UTR3	CMTM1	16	66612955	1	cg09849405	0.204523596	0.377786624	0.654003182	0.276216558	3.78E-04	1.13E-02
UTR3	CNTN5	11	100227279	1	cg03587117	0.686754415	0.589920333	0.417679709	-0.172240624	1.42E-04	0.008282373
UTR3	CRISPLD1	8	75946479	1	cg12133103	0.792506815	0.670728394	0.492556241	-0.178172154	9.02E-05	7.25E-03
UTR3	CX3CR1	3	39306129	1	cg04498110	0.63816995	0.511105922	0.357256145	-0.153849777	5.58E-05	6.06E-03
UTR3	DENND5B	12	31538503	1	cg00182053	0.852821865	0.746444794	0.583098873	-0.163345922	2.91E-05	0.005482591
UTR3	DMRTA2	1	50884242/50883329/50883394	3	cg21774338/cg22711792/cg23097402	0.28725513	0.36524962	0.578860173	0.213610552	6.22E-04	1.35E-02
UTR3	DNTT	10	98098271	1	cg13444670	0.713757315	0.649293511	0.483406882	-0.165886629	6.19E-05	6.30E-03
UTR3	EBF1	5	158125970	1	cg16394305	0.65559294	0.535946711	0.382240991	-0.15370572	1.30E-04	8.12E-03
UTR3	EMX2	10	119307769/119307931/119307692	3	cg13630043/cg17320707/cg19925204	0.20821151	0.251114512	0.409820419	0.158705907	4.99E-04	1.26E-02
UTR3	EPS8L1	19	55599029	1	cg10016788	0.384508685	0.510922939	0.666173845	0.155250907	1.20E-03	1.82E-02
UTR3	FAIM3	1	207077339	1	cg22671342	0.80168361	0.731530422	0.557446668	-0.174083754	1.88E-04	8.87E-03
UTR3	FAM13C	10	61007196	1	cg07625992	0.770272225	0.789121933	0.617386782	-0.171735152	5.43E-04	1.29E-02

UTR3	FAM26E	6	116839146	1	cg04312119	0.6660065	0.562379411	0.406034514	-0.156344897	1.32E-04	8.19E-03
UTR3	FEZF2	3	62355505/62355443/62355525	3	cg11178293/cg12378187/cg18175809	0.449229198	0.524748917	0.687487177	0.162738261	9.01E-06	3.75E-03
UTR3	FGF9	13	22277315/22275925	2	cg22356541/cg26585320	0.779463995	0.748968917	0.591004314	-0.157964603	2.90E-03	2.87E-02
UTR3	GFPT2	5	179728553	1	cg26918954	0.687336895	0.585780728	0.423299977	-0.162480751	5.04E-05	5.83E-03
UTR3	GP6	19	55526208	1	cg27154418	0.780080255	0.607387206	0.41956515	-0.187822056	3.94E-03	3.37E-02
UTR3	GRIN2D	19	48947560/48947631/48947572/48947578/48948005	5	cg04988514/cg14594362/cg16875863/cg22242216/cg26362197	0.337209907	0.448778694	0.62741754	0.178638846	1.99E-04	8.88E-03
UTR3	GSC	14	95234822/95234658/95234826	3	cg05142765/cg13073773/cg23695707	0.226465008	0.258448255	0.462748754	0.204300499	5.22E-04	1.28E-02
UTR3	GSX1	13	28368087	1	cg21851351	0.380350495	0.489700006	0.650913655	0.161213649	6.64E-04	1.38E-02
UTR3	GYPC	2	127453782	1	cg11611448	0.79602001	0.590340178	0.401720773	-0.188619405	2.67E-04	9.71E-03
UTR3	GZMA	5	54406016	1	cg05648002	0.67577133	0.510632344	0.356100214	-0.154532131	6.00E-06	3.06E-03
UTR3	HBZ	16	204474	1	cg02279719	0.205046045	0.199966012	0.363854455	0.163888443	1.93E-03	0.022596059
UTR3	HOXD13	2	176960255	1	cg17495130	0.45263155	0.387686828	0.583684286	0.195997459	2.64E-03	2.72E-02
UTR3	HS3ST3A1	17	13399070	1	cg27260984	0.81198985	0.756759083	0.600862695	-0.155896388	8.78E-04	1.56E-02
UTR3	HTN1	4	70923371	1	cg16270018	0.674315945	0.621824361	0.460186018	-0.161638343	1.79E-04	8.78E-03
UTR3	IL1RAP	3	190374982/190366926	2	cg19375583/cg20962955	0.61749919	0.496025183	0.3456051	-0.150420083	1.22E-04	7.82E-03
UTR3	IL22	12	68642433	1	cg03694077	0.825826325	0.751050461	0.549177714	-0.201872747	4.55E-05	5.66E-03
UTR3	IL24	1	207077339	1	cg22671342	0.80168361	0.731530422	0.557446668	-0.174083754	1.88E-04	8.87E-03
UTR3	IRF4	6	408730	1	cg16478536	0.673091255	0.510591667	0.359100409	-0.151491258	9.65E-04	1.63E-02
UTR3	IRX5	16	54967786	1	cg07452217	0.430253355	0.511959644	0.725156582	0.213196937	1.51E-04	8.42E-03
UTR3	KCNJ1	11	128708512	1	cg22721851	0.830890615	0.759916289	0.607408118	-0.152508171	6.82E-04	1.40E-02
UTR3	KIAA1486	2	226518461	1	cg03731588	0.786968595	0.56651525	0.408501177	-0.158014073	3.94E-04	1.14E-02
UTR3	KIDINS220	2	8870227	1	cg26426395	0.81599487	0.672126689	0.497986095	-0.174140593	3.12E-03	2.98E-02
UTR3	KIRREL2	19	36357700	1	cg22467594	0.7143387	0.628661617	0.469341177	-0.159320439	1.68E-04	8.71E-03
UTR3	LCN10	9	139633680/139632839	2	cg10434996/cg13735759	0.67393744	0.549721972	0.399317668	-0.150404304	4.88E-06	2.93E-03
UTR3	LHX5	12	113900719/113900751	2	cg00721315/cg11586330	0.33528102	0.364200703	0.51968583	0.155485127	9.94E-04	1.66E-02
UTR3	LOC643677	13	103381760	1	cg09714424	0.78100912	0.702736772	0.545224695	-0.157512077	1.61E-03	2.06E-02
UTR3	LRRN2	1	204586547	1	cg11820840	0.61777169	0.484634922	0.331329018	-0.153305904	1.28E-05	3.96E-03
UTR3	LST1	6	31556482	1	cg25664715	0.77500226	0.702124844	0.543219168	-0.158905676	8.00E-05	6.98E-03
UTR3	NCKAP1L	12	54936840	1	cg26787915	0.870193355	0.717033178	0.51087105	-0.206162128	1.62E-04	8.63E-03
UTR3	NKX2-4	20	21376484/21376337	2	cg07247419/cg13880779	0.22287616	0.28210296	0.433882499	0.151779539	5.04E-03	3.96E-02
UTR3	NPR3	5	32786671	1	cg25996586	0.608480995	0.487308006	0.314877286	-0.172430719	8.59E-05	7.07E-03
UTR3	NPS	10	129350922	1	cg19321696	0.57320293	0.429348739	0.273219223	-0.156129516	3.19E-06	2.84E-03
UTR3	OSBPL6	2	179260782	1	cg21182526	0.747212875	0.665576506	0.515118623	-0.150457883	9.17E-04	1.59E-02
UTR3	PATL2	15	44958131	1	cg24799947	0.703710905	0.607091956	0.453196259	-0.153895696	3.85E-05	0.005660056
UTR3	PDGFC	4	157682848	1	cg15957807	0.72117245	0.583535839	0.400430395	-0.183105443	4.24E-05	5.66E-03
UTR3	PDX1	13	28499045/28498878/28498956	3	cg00184376/cg15961466/cg26299169	0.32888554	0.421182144	0.664261111	0.243078967	3.27E-05	5.54E-03
UTR3	PHLDA3	1	201437101/201437298/201435275	3	cg04055835/cg08639762/cg26120226	0.62117279	0.591942722	0.438409741	-0.153532981	3.77E-06	2.84E-03
UTR3	PHYHIPL	10	61007196	1	cg07625992	0.770272225	0.789121933	0.617386782	-0.171735152	5.43E-04	1.29E-02
UTR3	PIK3CG	7	106545895	1	cg24232378	0.75614835	0.641287256	0.486666368	-0.154620887	4.82E-03	3.85E-02
UTR3	PKHD1	6	51481908	1	cg01776172	0.54562239	0.440443122	0.284132482	-0.15631064	1.26E-05	3.96E-03
UTR3	PLA2G4C	19	48551504	1	cg12986110	0.585142475	0.494844211	0.340336014	-0.154508197	5.40E-04	1.29E-02

UTR3	POU3F1	1	38509934/38510933/38510074/38510522/38510383/38510182	6	cg05785481/cg08599259/cg13149116/cg22821908/cg25488697/cg25609528	0.200020794	0.318494176	0.469416162	0.150921985	5.73E-04	1.31E-02
UTR3	POU4F2	4	147562073	1	cg21200539	0.37320488	0.541917483	0.697049414	0.15513193	1.59E-03	2.05E-02
UTR3	PTHLH	12	28115804	1	cg14210985	0.69959215	0.602720411	0.392690036	-0.210030375	3.37E-08	3.44E-04
UTR3	PTPLA	10	17632231	1	cg05341848	0.657783465	0.602039561	0.45174495	-0.150294611	9.85E-05	7.45E-03
UTR3	PXDN	2	1637068	1	cg10888111	0.8753676	0.59430805	0.438202605	-0.156105445	1.65E-03	2.08E-02
UTR3	RAB6B	3	133545640	1	cg26466524	0.728497465	0.640354644	0.484425677	-0.155928967	4.13E-05	5.66E-03
UTR3	RASL10A	22	29709320/29709282	2	cg12552771/cg15383276	0.341383162	0.280065188	0.436352001	0.156286812	1.58E-03	2.05E-02
UTR3	RBMXL2	11	7112356	1	cg06176750	0.652383075	0.45420675	0.274610925	-0.179595825	3.29E-04	1.07E-02
UTR3	RGS5	1	163113390	1	cg10780638	0.675836345	0.576672406	0.415553482	-0.161118924	7.73E-05	6.98E-03
UTR3	RIC3	11	8128361	1	cg03054541	0.794465585	0.738012383	0.564055195	-0.173957188	5.31E-04	1.29E-02
UTR3	RPRML	17	45055836/45056008	2	cg20129313/cg22674412	0.069754249	0.064663847	0.228397823	0.163733976	1.38E-03	1.92E-02
UTR3	RXFP2	13	32376637	1	cg09573371	0.61305552	0.558240289	0.386196736	-0.172043553	1.73E-06	2.84E-03
UTR3	SACS	13	23904116	1	cg24140343	0.86852772	0.730204833	0.484653191	-0.245551642	1.20E-05	3.96E-03
UTR3	SCNN1G	16	23227394	1	cg00452252	0.67246135	0.637818306	0.480935632	-0.156882674	1.22E-03	1.82E-02
UTR3	SELV	19	40009835	1	cg07052524	0.61414098	0.452529678	0.299807617	-0.152722061	4.26E-04	1.19E-02
UTR3	SIM1	6	100836916	1	cg24544512	0.570116665	0.520291622	0.357163086	-0.163128536	9.56E-06	3.75E-03
UTR3	SLC14A2	18	43262611	1	cg15090262	0.728026735	0.643834861	0.466792805	-0.177042057	2.29E-05	5.47E-03
UTR3	SNAP25	20	10286857	1	cg03569068	0.791877555	0.749251044	0.58704295	-0.162208094	1.33E-03	1.89E-02
UTR3	SYT10	12	33528782	1	cg03354810	0.765560605	0.750931206	0.593719	-0.157212206	3.32E-04	1.07E-02
UTR3	TCHHL1	1	152056869	1	cg05388137	0.729877155	0.627862744	0.4650036	-0.162859144	1.77E-04	8.75E-03
UTR3	TCTEX1D1	1	67244062	1	cg10625686	0.60068317	0.54014705	0.365761418	-0.174385632	4.96E-05	5.82E-03
UTR3	TFAP2A	6	10398601/10398494	2	cg04389897/cg04406454	0.264736835	0.398388925	0.597863175	0.19947425	0.00139121	0.01921526
UTR3	TMEM155	4	122680946	1	cg16298202	0.79069576	0.724691622	0.556162114	-0.168529509	9.82E-05	7.45E-03
UTR3	TMEM215	9	32789031	1	cg14279416	0.73243203	0.587047889	0.425299618	-0.161748271	1.03E-03	1.68E-02
UTR3	TMEM26	10	63167726	1	cg18237739	0.69384678	0.580288889	0.424146123	-0.156142766	8.37E-05	7.07E-03
UTR3	TRPV6	7	142569053	1	cg08835464	0.811856095	0.661889144	0.468637341	-0.193251804	2.95E-05	5.48E-03
UTR3	VGLL2	6	117594700	1	cg14664726	0.690373685	0.633957139	0.471073259	-0.16288388	2.56E-05	0.005472627
UTR3	ZNF304	19	57870634	1	cg27366815	0.80288294	0.655238128	0.481440823	-0.173797305	3.64E-04	1.11E-02
UTR3	ZNF385B	2	180307728	1	cg17100158	0.765192235	0.710885594	0.552142264	-0.158743331	1.88E-04	8.87E-03
UTR3	ZNF546	19	40522655	1	cg15568892	0.852869015	0.8065091	0.588532277	-0.217976823	2.25E-06	2.84E-03
UTR3	ZNF549	19	58051563	1	cg21647626	0.854609165	0.706595533	0.489310455	-0.217285079	7.75E-06	3.44E-03

Table S5. Statistics for DNA samples loading in each section of the microarray

Sentrix_ID	Sentrix_Position	Status	Concentration (ng/ul)	Volume (ul)	260/280
3999442103	R01C01	LGA	>120	4	1.74
3999442103	R02C01	Normal	>120	4	1.9
3999442103	R03C01	HGA	>120	4	1.93
3999442103	R04C01	LGA	>120	4	1.93
3999442103	R05C01	Normal	108	4	1.9
3999442103	R06C01	HGA	>120	4	2.01
3999442103	R01C02	LGA	>120	4	1.93
3999442103	R02C02	Normal	>120	4	1.88
3999442103	R03C02	HGA	102	4	2.01
3999442103	R04C02	HGA	>120	4	1.9
3999442103	R05C02	Normal	>120	4	1.87
3999442103	R06C02	LGA	121	4	1.92
3999442051	R01C01	Normal	110	3.7	1.85
3999442051	R02C01	LGA	100	4	1.87
3999442051	R03C01	Normal	150	2.7	1.85
3999442051	R04C01	HGA	52.4	7.7	1.86
3999442051	R05C01	Normal	150	2.7	1.88
3999442051	R06C01	LGA	120	3.4	1.88
3999442051	R01C02	Normal	130	3.1	1.88
3999442051	R02C02	HGA	83.6	4.8	1.88
3999442051	R03C02	Normal	170	2.4	1.88
3999442051	R04C02	HGA	76.6	5.3	1.9
3999442051	R05C02	LGA	82.2	4.9	1.9
3999442051	R06C02	Normal	92.8	4.4	1.88
3999442112	R01C01	LGA	>120	4	1.87
3999442112	R02C01	HGA	>120	4	1.93
3999442112	R03C01	Normal	>120	4	1.89
3999442112	R04C01	HGA	>120	4	2
3999442112	R05C01	HGA	106	4	1.9
3999442112	R06C01	Normal	>120	4	1.88
3999442112	R01C02	HGA	>120	4	1.97
3999442112	R02C02	HGA	>120	4	1.94
3999442112	R03C02	Normal	83.4	5	1.88
3999442112	R04C02	HGA	>120	4	2.06
3999442112	R05C02	Normal	>120	4	1.89
3999442112	R06C02	LGA	>120	4	1.89
3999442050	R01C01	LGA	>120	4	1.9
3999442050	R02C01	HGA	>120	4	2.04
3999442050	R03C01	Normal	112	4	1.87
3999442050	R04C01	LGA	>120	4	1.88
3999442050	R05C01	LGA	>120	4	1.88
3999442050	R06C01	Normal	>120	4	1.88
3999442050	R01C02	LGA	99.6	4	1.91
3999442050	R02C02	Normal	>120	4	1.88

3999442050	R03C02	Normal	>120	4	1.88
3999442050	R04C02	LGA	>120	4	1.89
3999442050	R05C02	HGA	>120	4	1.93
3999442050	R06C02	LGA	>120	4	1.92
3999442119	R01C01	Normal	>120	4	1.9
3999442119	R02C01	Normal	>120	4	1.89
3999442119	R03C01	HGA	>120	4	1.89
3999442119	R04C01	LGA	>120	4	1.92
3999442119	R05C01	HGA	>120	4	1.97
3999442119	R06C01	HGA	>120	4	1.98
3999442119	R01C02	HGA	86.5	5	1.88
3999442119	R02C02	LGA	>120	4	1.88
3999442119	R03C02	LGA	>120	4	1.88
3999442119	R04C02	HGA	>120	4	1.91
3999442119	R05C02	HGA	>120	4	2.01
3999442119	R06C02	HGA	>120	4	1.85

LGA: Low-grade adenoma   HGA: High-grade adenoma. Sentrix_ID, the BeadChip ID provided by the manufacturer; Sentrix_Position, position of the sample on the BeadChip.

Table S6. Clinical and microarray information for each sample.

Gender	Age	Chemo- and radio-therapies	Status	Sentrix_ID	Sentrix_Position	Diameter(cm)
female	76	Treatment naive	LGA	3999442103	R01C01	0.9
			Normal	3999442103	R02C01	0.2
female	83	Treatment naive	HGA	3999442103	R03C01	0.2
female	64	Treatment naive	LGA	3999442103	R04C01	0.2
female	57	Treatment naive	Normal	3999442103	R05C01	1.5
male	78	Treatment naive	HGA	3999442103	R06C01	0.9
female	70	Treatment naive	LGA	3999442103	R01C02	0.9
			Normal	3999442103	R02C02	0.2
male	68	Treatment naive	HGA	3999442103	R03C02	0.9
			HGA	3999442119	R05C02	0.2
male	77	Treatment naive	LGA	3999442051	R05C02	0.2
			Normal	3999442051	R06C02	0.2
male	60	Treatment naive	LGA	3999442051	R02C01	0.9
			Normal	3999442051	R01C01	0.2
male	56	Treatment naive	LGA	3999442051	R06C01	0.2
			Normal	3999442051	R05C01	0.9
female	70	Treatment naive	HGA	3999442051	R04C01	1.3
			Normal	3999442051	R03C01	0.2
female	58	Treatment naive	LGA	3999442112	R01C01	0.8
			HGA	3999442112	R02C01	0.2
			Normal	3999442112	R03C01	0.9
male	59	Treatment naive	HGA	3999442112	R04C01	0.8
female	32	Treatment naive	HGA	3999442112	R05C01	0.8
			Normal	3999442112	R06C01	0.9
male	51	Treatment naive	HGA	3999442112	R01C02	NA

			HGA	3999442112	R02C02	0.8
			Normal	3999442112	R03C02	1.5
female	76	Treatment naive	HGA	3999442112	R04C02	0.2
			Normal	3999442112	R05C02	0.9
male	55	Treatment naive	LGA	3999442050	R01C01	1
			LGA	3999442112	R06C02	0.2
female	53	Treatment naive	HGA	3999442050	R02C01	5
			Normal	3999442050	R03C01	0.2
female	68	Treatment naive	LGA	3999442050	R04C01	0.2
male	51	Treatment naive	LGA	3999442050	R05C01	0.2
			Normal	3999442050	R06C01	0.2
male	48	Treatment naive	LGA	3999442050	R04C02	0.2
			HGA	3999442050	R05C02	2.5
			Normal	3999442050	R03C02	0.2
male	34	Treatment naive	LGA	3999442050	R06C02	0.9
			Normal	3999442119	R01C01	0.8
male	66	Treatment naive	HGA	3999442119	R01C02	1.5
			LGA	3999442119	R02C02	0.2
			LGA	3999442119	R03C02	NA
			HGA	3999442119	R04C02	1.5
female	57	Treatment naive	LGA	3999442103	R06C02	0.2
			LGA	3999442050	R01C02	0.7
			Normal	3999442050	R02C02	0.2
male	80	Treatment naive	HGA	3999442103	R04C02	NA
			HGA	3999442119	R06C02	0.2
			Normal	3999442103	R05C02	1.5

Sentrix_ID, the BeadChip ID provided by the manufacturer; Sentrix_Position, position of the sample on the BeadChip.

Table S7. Public datasets.

Dataset	Normal	Adenoma	Canner	Metastasis	White blood	Country/Source
GSE68060	36		82			Spain
GSE68838	38		255			TCGA
GSE77954	11	12	13	9		US
GSE77965	6		6			Austria
GSE81211	3					South Korea
GSE101764	149		112			Germany
GSE107352	21		30			Spain
E-MTAB-6450	8	39				Germany
GSE75546	6		6			China
GSE40279					656	US
Total	278	51	504	9	656	1498
